# Profile and Predictors of Hepatitis B Surface Antigen Seroreversion Following PEG-IFNα with or Without Therapeutic Vaccine BRII-179 or siRNA Elebsiran: A Post Hoc Pooled Analysis of Two Randomized Controlled Trials in Patients with Chronic Hepatitis B Virus Infection

**DOI:** 10.3390/vaccines14100868

**Published:** 2026-10-01

**Authors:** Zhiliang Gao, Bingliang Lin, Grace Lai-Hung Wong, Man-Fung Yuen, Yiwen Liu, Chong Zhu, Ke Cao, Alex Tian, Xiaofei Chen, Qing Zhu, David Margolis, Jidong Jia, Zhi Hong

**Affiliations:** 1 The Third Affiliated Hospital, Sun Yat-sen University, Guangzhou 510630, China; 2 Medical Data Analytics Centre (MDAC), Department of Medicine and Therapeutics, The Chinese University of Hong Kong, Hong Kong, China; 3 Department of Medicine, School of Clinical Medicine, The University of Hong Kong, Hong Kong, China; 4 Brii Biosciences (Beijing) Co., Limited, Beijing 100192, China; 5 Brii Biosciences, Inc., Durham, NC 27701, USA; 6 Liver Research Center, Beijing Friendship Hospital, Capital Medical University, Beijing 100050, China

**Keywords:** therapeutic vaccine, HBV, HBsAg loss, HBsAg seroreversion, pegylated interferon α, siRNA, combination therapy

## Abstract

**Background/Objectives:** The clinical implications of HBsAg seroreversion following HBV functional cure therapies remain unclear. In two randomized phase 2 trials, therapeutic vaccine BRII-179 or siRNA elebsiran in combination with PEG-IFNα was evaluated versus PEG-IFNα monotherapy. This pooled analysis characterized the profile of HBsAg seroreversion and explored potential predictors. **Methods:** Participants achieving HBsAg loss at or shortly after end of treatment (EOT) were included. Binary endpoints were summarized with two-sided 95% CIs using the Clopper–Pearson method. Time-to-event analyses used Kaplan–Meier methods and Cox regression. Longitudinal associations were evaluated using logistic mixed-effects models. **Results:** Among 55 participants included, 24 (43.6%) experienced HBsAg seroreversion with modest magnitude; all peak levels were <100 IU/mL and most were <10 IU/mL, infrequently accompanied by HBV DNA or ALT elevation after nucleos(t)ide analogue (NA) discontinuation. Anti-HBs > 100 IU/L at EOT was associated with a lower risk of HBsAg seroreversion (HR 0.30, 95% CI 0.10–0.92; *p* = 0.036). Sustained anti-HBs positivity during post-EOT follow-up was consistently associated with durable HBsAg loss in longitudinal analyses. Exploratory Kaplan–Meier analyses showed numerically higher HBsAg seroreversion rates among HBeAg-positive participants and those with PEG-IFNα treatment duration <12 weeks after HBsAg loss, although these findings were not statistically significant. **Conclusions:** HBsAg seroreversion after PEG-IFNα with or without BRII-179 or elebsiran was mild and rarely associated with clinically relevant relapse. Higher EOT anti-HBs levels and persistent anti-HBs positivity were associated with greater durability of HBsAg loss and warrant prospective evaluation as a candidate biomarker for risk-adapted off-treatment monitoring.

## 1. Introduction

Hepatitis B virus (HBV) infection remains a major global public health challenge, affecting more than 250 million people worldwide, causing over 1 million new infections annually, and contributing substantially to mortality, primarily through cirrhosis and hepatocellular carcinoma (HCC) [1]. “Functional cure”, defined as sustained HBsAg loss (HBsAg < 0.05 IU/mL) with undetectable HBV DNA for at least 6 months after discontinuation of all antiviral therapies, represents the ideal treatment goal for chronic HBV infection because of its durability, reduction in stigma and discrimination, and association with a lower risk of liver disease progression and HCC [2]. However, functional cure remains rare with current treatment options, including long-term nucleos(t)ide analogue (NA) therapy and finite PEG-IFNα therapy. PEG-IFNα add-on therapy to NA is currently recommended for selected patients [3], as accumulating evidence has demonstrated that it substantially increases HBsAg loss rate, particularly among patients with lower baseline HBsAg levels [4,5]. Nevertheless, the durability of HBsAg loss achieved with PEG-IFNα-based therapies has not been fully established in well-designed prospective clinical trials. Real-world data indicate that 15% to 41% of patients experienced HBsAg relapse after achieving HBsAg loss with PEG-IFNα monotherapies or PEG-IFNα combined with NA therapy. Moreover, the potential role of NA consolidation following HBsAg loss in preventing HBsAg seroreversion, defined as a change in HBsAg status from <0.05 IU/mL to ≥0.05 IU/mL following treatment cessation, remains insufficiently investigated [6,7,8,9,10].

Therapeutic strategies combining novel anti-HBV agents with PEG-IFNα are being actively explored to improve functional cure rates. However, the post-treatment kinetics of HBsAg following discontinuation of these combination regimens, as well as the factors associated with HBsAg seroreversion, remain poorly characterized. BRII-179 is a recombinant therapeutic vaccine comprising PreS1, PreS2 and S HBV surface antigens formulated with an aluminum phosphate adjuvant and is capable of inducing both humoral and cellular immune responses in patients with chronic HBV infection [11]. In the phase 2 BRII-179-002 study, the addition of BRII-179 to PEG-IFNα improved HBsAg loss rate at EOT, compared with PEG-IFNα monotherapy (15/57 [26.3%] vs. 11/57 [19.3%]), the numerical difference maintained through at least 24 weeks after NA discontinuation (11/57 [19.3%] vs. 7/57 [12.3%]). No participants with anti-HBs titer ≥ 100 IU/mL at EOT experienced HBsAg seroreversion through at least 24 weeks after NA discontinuation, suggesting that robust anti-HBs responses may be critical for sustained off-treatment HBsAg loss [12]. Elebsiran (BRII-835/VIR-2218) is a triantennary *N*-acetyl galactosamine-conjugated small interfering RNA targeting the *HBx* gene. In the phase 2 ENSURE study, elebsiran combined with PEG-IFNα improved HBsAg loss rate at EOT, compared with PEG-IFNα monotherapy (11/37 [29.7%] vs. 1/18 [5.6%]); the trend maintained through 24 weeks post-EOT [13]. Notwithstanding these encouraging results, HBsAg seroreversion was observed in a subset of participants in both trials after completion of PEG-IFNα-based therapy, occurring either during NA consolidation therapy or after NA discontinuation. A better understanding of the frequency, timing, and magnitude of post-treatment HBsAg seroreversion, as well as the factors associated with its development, is essential for optimizing strategies aimed at achieving durable functional cure. Therefore, we conducted a post hoc pooled analysis of the BRII-179-002 and ENSURE trials to evaluate the incidence and characteristics of post-EOT HBsAg seroreversion and to identify potential predictors of this event.

## 2. Materials and Methods

### 2.1. Participants

This post hoc analysis retrieved data from two phase 2 clinical trials: BRII-179-002 (CTR20213100; First Public Disclosure Date: 30 November 2021) and ENSURE (NCT05970289; First Posted on 1 August 2023). Both trials enrolled adults aged 18 to 60 years with chronic HBV infection, defined by positive serum HBsAg for at least 6 months before screening. Participants should be virologically suppressed by first-line NA therapy with HBV DNA below lower limit of quantification (LLOQ) for at least 6 months, without significant fibrosis or history of hepatic decompensation, and eligible for PEG-IFNα treatment. In addition to above criteria, participants enrolled in BRII-179-002 were required to have received 24 to 28 doses of PEG-IFNα and to have negative HBeAg, anti-HBs < 10 IU/L, and HBsAg ≥ 0.05 and <100 IU/mL at screening. Participants enrolled in ENSURE Part I could be either HBeAg-positive or HBeAg-negative and were required to have HBsAg > 100 and ≤3000 IU/mL at screening. ENSURE Part II enrolled rollover participants from the BRII-179-835-001 study (NCT04749368) [14] who had received elebsiran 100 mg every 4 weeks for 9 doses and BRII-179 40 µg every 4 weeks for 9 doses and completed treatment for at least 1 year. Detailed inclusion and exclusion criteria for both trials are provided in the Supplementary Methods.

Participants from either trial who achieved HBsAg < LLOQ (0.05 IU/mL) at EOT or the first follow-up visit after EOT were included in this post hoc analysis, irrespective of treatment assignment.

### 2.2. Trial Designs

In the multicenter, randomized, double-blind, phase 2 BRII-179-002 study, 114 participants were randomized 1:1 to receive BRII-179 40 µg or placebo every 3 weeks for 7 doses in combination with 24 weekly doses of PEG-IFNα. In the multicenter, open-label, phase 2 ENSURE study, 55 participants naive to both BRII-179 and elebsiran (Part I) were randomized 1:1:1 to receive 48 weekly doses of PEG-IFNα monotherapy or PEG-IFNα combined with elebsiran (200 mg or 100 mg every 4 weeks for 13 doses). An additional 31 participants (ENSURE Part II) were rolled over from a previous trial and had completed elebsiran plus BRII-179 treatment for at least one year; these participants received elebsiran 100 mg every 4 weeks for 13 doses together with 48 weekly doses of PEG-IFNα. Overall, in addition to background NA therapy, participants in both trials received approximately 48 weeks of PEG-IFNα, either alone or in combination with BRII-179 and/or elebsiran. After treatment completion, participants who met NA discontinuation criteria (HBsAg < 0.05 IU/mL for at least two consecutive visits, HBV DNA < LLOQ, ALT < 2 × upper limit of normal, and HBeAg negativity) discontinued NA therapy after a consolidation period, regardless of treatment assignment. In BRII-179-002, eligible participants discontinued NA at 12 to 24 weeks post-EOT and were followed for 48 weeks thereafter. In ENSURE, all eligible participants discontinued NA after 24 weeks of consolidation and were followed for 24 weeks after NA discontinuation. Detailed trial designs including NA discontinuation criteria for both trials are provided in the Supplementary Methods and illustrated in Figures S1 and S2.

Quantitative HBV DNA, HBsAg, and anti-HBs assessments were performed at the same central laboratory (Q^2^ Solutions/IQVIA Laboratories) using validated assays and standardized procedures across both trials. HBV DNA was quantified using the Cobas^®^ 6800 system, and HBsAg and anti-HBs were measured using the Elecsys HBsAg II quant assay and Elecsys Anti-HBs II assay, respectively (Roche Diagnostics (North Ryde, Australia)), according to manufacturer’s instructions. The LLOQs were 10 IU/mL for HBV DNA, 0.05 IU/mL for HBsAg, and 2.0 IU/L for anti-HBs.

**Figure 1:**
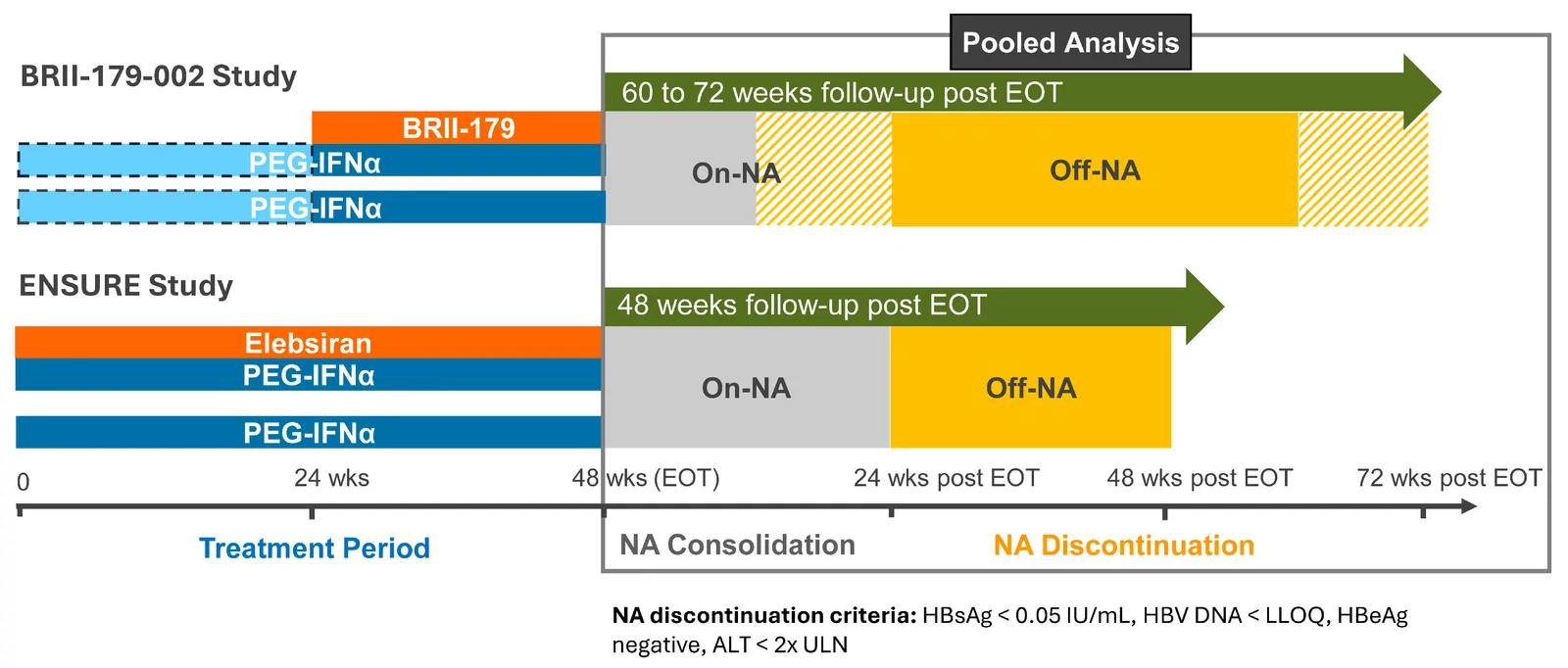
Strategy of pooled analysis. This analysis summarized post-EOT HBsAg seroreversion in pooled. BRII-179/elebsiran + PEG-IFNα cohorts and PEG-IFNα monotherapy cohorts in BRII-179-002 and ENSURE trials. Dashed box: participants had received 24 to 28 doses of PEG-IFNα at screening before BRII-179 add-on; diagonal hatching area: participants meeting the pre-defined criteria were to discontinue NA (Supplementary Methods) from 12 to 24 weeks post-EOT. No statistical testing was performed. ALT, alanine aminotransferase; EOT, end of treatment; LLOQ, lower limit of quantification; NA, nucleos(t)ide analogue; PEG-IFNα, pegylated interferon alpha; ULN, upper limit of normal; wks, weeks.

This post hoc pooled analysis evaluated the incidence, characteristics, clinical impact, and potential predictors of post-EOT HBsAg seroreversion among participants who achieved HBsAg loss following PEG-IFNα-based therapy in the pooled study population (Figure 1). This analysis was conducted and reported in accordance with the STROBE guideline.

### 2.3. Ethical Oversight

Both BRII-179-002 and ENSURE were conducted in accordance with the Declaration of Helsinki and the Declaration of Istanbul, Council for International Organizations of Medical Sciences International Ethical Guidelines for Biomedical Research Involving Human Subjects, Good Clinical Practice guidelines of the International Council for Harmonisation, and all applicable local regulations. Protocol and amendments were reviewed and approved by relevant institutional review boards or independent ethics committees at participating centers. Detailed ethics approval information is provided in the Supplementary Materials. Written informed consent was obtained from all participants before any trial procedures were performed in either trial. The analysis plan for the present pooled analysis was developed after completion of the parent trials. The sponsor, Brii Biosciences, was involved in the design, conduct, and data analysis of both trials and this post hoc study.

### 2.4. Statistical Analysis

Statistical analyses of this post hoc pooling study used harmonized participant-level data from the BRII-179-002 and ENSURE trials and were restricted to 55 participants achieving HBsAg loss (<LLOQ) at EOT or the first post-EOT follow-up visit. Common analysis definitions were applied across the two trials. EOT was defined as completion of all assigned study treatments (BRII-179/elebsiran + PEG-IFNα or PEG-IFNα monotherapy) other than background NA therapy. HBsAg seroreversion was defined as HBsAg ≥ LLOQ after a prior documented HBsAg loss. NA consolidation duration was derived using the same algorithm in both trials and was defined as the actual interval of NA consolidation treatment. For the overall time to HBsAg seroreversion analysis, the time origin was the first documented HBsAg measurement below the LLOQ at EOT or at the first post-EOT follow-up visit. Participants without a documented HBsAg seroreversion were censored at their last available HBsAg assessment. The two trials used different visit schedules after EOT and after NA discontinuation. To minimize potential bias arising from these differences, all time-to-event analyses were based on actual assessment dates rather than nominal visit windows, and follow-up time was calculated using observed visit dates. No covariate values were missing. Analyses of longitudinal laboratory measurements used all available observed data, without imputation for missing measurements or missed visits.

Binary outcomes, including HBsAg seroreversion rates, were summarized as proportions with two-sided 95% confidence intervals (CIs) using the Clopper–Pearson exact method.

Time-to-event endpoints were measured from the first documented HBsAg < LLOQ (at EOT or the first follow-up visit after EOT) to the first HBsAg seroreversion. To account for differences in follow-up duration, participants without documented HBsAg seroreversion were censored at their last available follow-up assessment. Kaplan–Meier methods were used to estimate cumulative HBsAg seroreversion-free survival probabilities, with between-group differences assessed descriptively using the log-rank test. Cox proportional hazards regression models were used to estimate hazard ratios (HRs) and 95% CIs for factors potentially associated with HBsAg seroreversion. The proportional hazards assumption was evaluated using standardized score processes based on cumulative martingale residuals. Given the limited number of HBsAg seroreversion events, a parsimonious model including anti-HBs level at EOT (>100 versus ≤100 IU/L) and PEG-IFNα-based treatment duration after HBsAg loss (≥12 versus <12 weeks) was fitted. These variables were selected because they represented the principal clinical hypotheses of interest and were considered biologically plausible. The model was stratified by parent trial, allowing separate baseline hazard functions for BRII-179-002 and ENSURE while estimating common covariate effects across trials. Owing to the limited number of HBsAg seroreversion events, results from Cox models were considered exploratory.

Longitudinal transitions between anti-HBs status and HBsAg seroreversion within 48 weeks after EOT were summarized using Sankey diagrams as a descriptive visualization of serological dynamics over follow-up. Longitudinal associations were assessed using generalized linear mixed-effects models with a logit link, with HBsAg seroreversion (yes/no) as the dependent variable, anti-HBs status (<10 vs. ≥10 IU/L) and follow-up time (weeks) as fixed effects, and a participant-specific random intercept to account for repeated measurements. The random effect was modeled using a variance-components covariance structure, and model parameters were estimated by maximum likelihood with the Laplace approximation. Model-based predicted probabilities were derived for graphical presentation.

In a separate analysis, Kaplan–Meier methods were used to estimate cumulative HBsAg seroreversion-free survival according to NA consolidation duration among the 41 participants who maintained HBsAg loss throughout NA consolidation and subsequently discontinued NA. Because differences in NA consolidation schedules and post-NA follow-up duration could specifically affect analyses of NA consolidation duration, a sensitivity analysis was conducted using a common 48-week observation window after EOT to minimize potential bias arising from unequal opportunities for HBsAg seroreversion ascertainment after EOT. Participants without documented HBsAg seroreversion were censored at 48 weeks after EOT or at their last available post-EOT HBsAg assessment if follow-up ended earlier.

All statistical tests were two-sided. Because the analyses were post hoc and exploratory and no confirmatory testing hierarchy was prespecified, no adjustment for multiplicity was applied. All *p* values were nominal and were interpreted descriptively in conjunction with effect estimates and confidence intervals. Statistical analyses were conducted using SAS Viya (version 2025.06; SAS Institute Inc., Cary, NC, USA) and R (version 4.5.0, R Foundation for Statistical Computing, Vienna, Austria).

Statistical analysis plans and study protocols of BRII-179-002 and ENSURE studies are submitted as Supplementary Materials.

## 3. Results

### 3.1. Study Population

**Figure 2:**
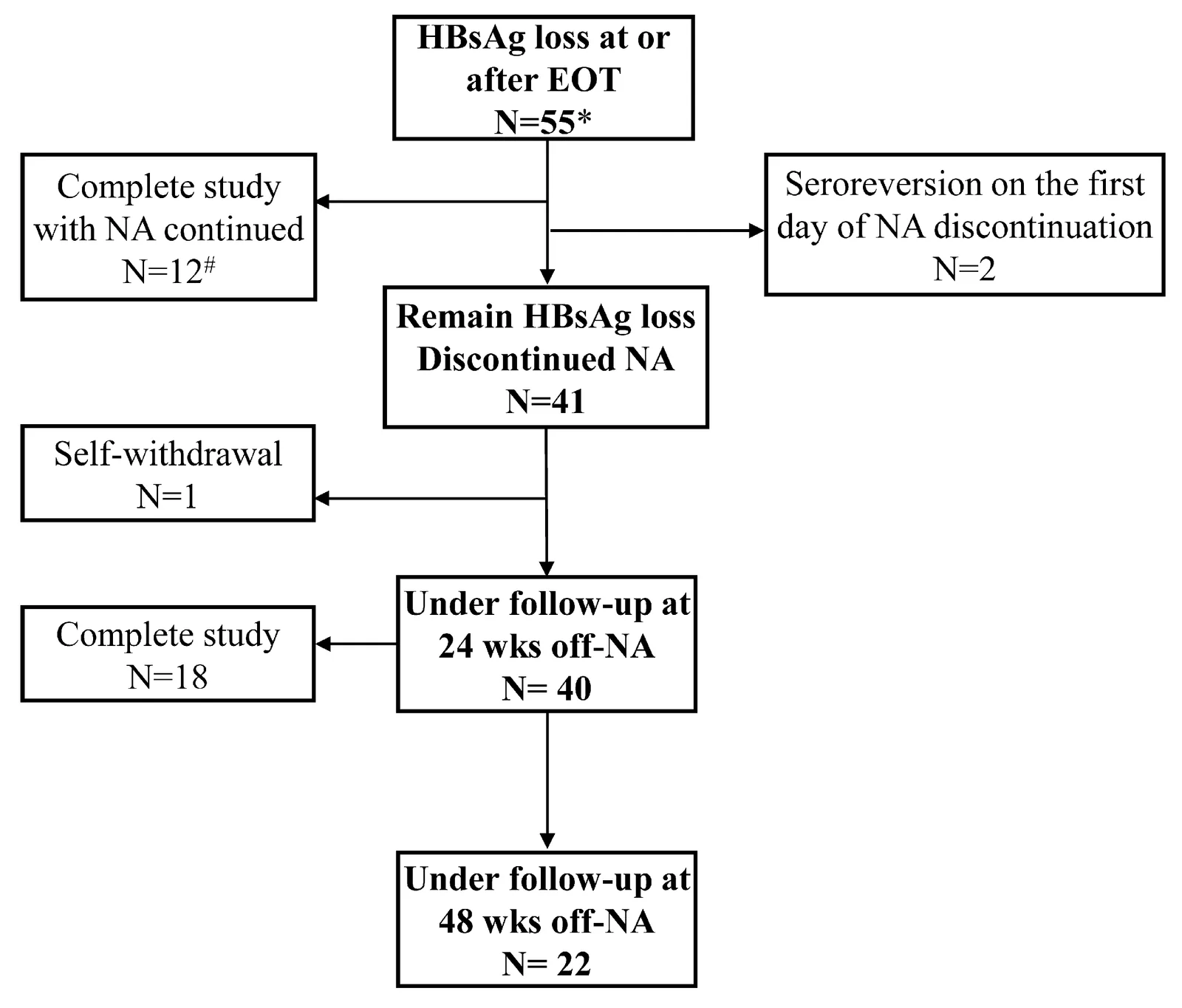
Participant disposition. *, 52 participants were HBsAg < LLOQ at EOT, while 3 participants achieved HBsAg loss at the next follow-up visit after EOT; #, 11 participants had HBsAg seroreversion and one remained HBeAg positive without HBsAg seroreversion; they completed study at 24-week post-EOT. EOT, end of treatment; N, number of participants; NA, nucleos(t)ide analogue; wks, weeks.

A total of 55 participants who achieved HBsAg loss with PEG-IFNα-based therapies were included in this pooled post hoc analysis (Figure 2), including 29 from BRII-179-002 and 26 from ENSURE. Participant disposition for each trial is presented in Figure S3.

Overall, included participants had a mean (SD) age of 41.93 (8.22) years. Most were male (37/55, 67.3%) and HBeAg-negative (51/55, 92.7%) at trial entry. Forty-two participants (76.4%) received PEG-IFNα-based combination therapy and 13 (23.6%) received PEG-IFNα monotherapy. The median duration of PEG-IFNα-based treatment from HBsAg loss to EOT was 17.9 weeks. At EOT, 26 participants (47.3%) had anti-HBs levels > 100 IU/L, whereas 21 (38.2%) had anti-HBs levels ≤ 10 IU/L. The median duration of NA consolidation, defined as the interval from EOT to NA discontinuation among participants who discontinued NA or to end of study date among those who remained on NA, was 23.9 weeks. Detailed baseline and treatment characteristics are presented in Table 1.

Among the 55 participants, 41 maintained HBsAg loss during NA consolidation and subsequently discontinued NA therapy. Forty participants were followed for at least 24 weeks after NA discontinuation, including 22 participants in BRII-179-002 with 48 weeks of off-NA follow-up. One participant prematurely withdrew from the trial at 12 weeks after NA discontinuation and remained HBsAg < LLOQ at the last available assessment.

### 3.2. Incidence and Timing of HBsAg Seroreversion

Overall, HBsAg seroreversion occurred in 24 of 55 participants (43.6%) during the trial period: 13 events during NA consolidation and 11 after NA discontinuation. Among participants who experienced HBsAg seroreversion after NA discontinuation, 8 events occurred within the first 24 weeks after NA discontinuation and 3 occurred between Week 24 and 48 off-NA (Figure 2). HBsAg seroreversion rates according to baseline characteristics and treatment-related factors are summarized in Table 2. While the limited sample sizes resulted in wide 95% CIs for most subgroup estimates, numerically higher HBsAg seroreversion rates were observed in participants with shorter PEG-IFNα-based treatment after HBsAg loss (<12 weeks versus ≥12 weeks) and in those with lower anti-HBs levels at EOT (≤100 IU/L versus >100 IU/L), supporting further time-to-event analyses of these factors.

### 3.3. Magnitude and Clinical Correlates of HBsAg Seroreversion

**Figure 3:**
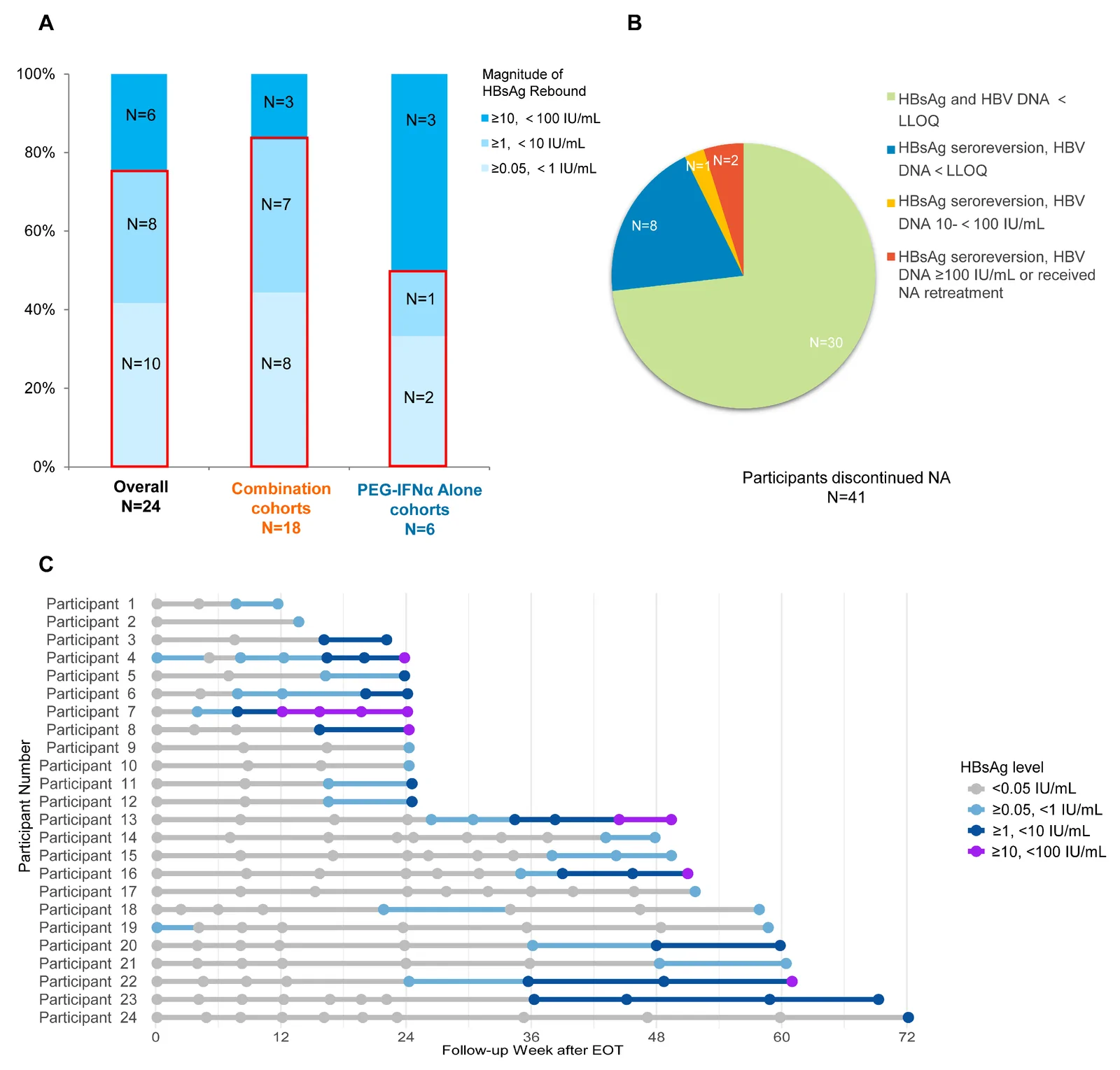
Magnitude of HBsAg seroreversion and virologic status at follow-up. (**A**) Maximum post-seroreversion HBsAg levels by category and treatment regimen. (**B**) HBsAg and HBV DNA status at the last follow-up visit in participants who discontinued NA. (**C**) Maximum HBsAg levels in individual participants with seroreversion; Participants 1 to 13 experienced HBsAg seroreversion during NA consolidation, Participants 14 to 24 experienced HBsAg seroreversion after NA discontinuation. EOT, end of treatment; NA, nucleos(t)ide analogue.

The magnitude of HBsAg seroreversion was modest across all participants experiencing seroreversion, irrespective of the timing of the event (Figure 3C). Although HBsAg levels gradually increased over time following seroreversion, the maximum HBsAg level remained <100 IU/mL in all participants, and most (18/24, 75.0%) had peak HBsAg levels < 10 IU/mL (Figure 3A).

Among the 41 participants who discontinued NA, 30 (73.2%) sustained HBsAg < 0.05 IU/mL at the last follow-up visit (Figure 3B). Of the 11 participants who experienced HBsAg seroreversion after NA discontinuation, 9 had HBsAg levels < 10 IU/mL at their last assessment, including 6 with HBsAg levels < 1 IU/mL; the remaining 2 participants had HBsAg levels of 11.7 IU/mL and 20.7 IU/mL, respectively.

Detectable HBV DNA and ALT elevation were infrequent. Among the 41 participants who discontinued NA therapy, 38 (92.7%) maintained HBV DNA levels < LLOQ (10 IU/mL) at the last visit (Figure 3B); 3 (7.3%) experienced HBsAg seroreversion accompanied by confirmed HBV DNA elevation, including 2 with HBV DNA levels ≥ 100 IU/mL. One participant (Figure S4A) with low-level HBsAg seroreversion had an increase in HBV DNA to 1210 IU/mL and developed a transient ALT elevation (maximum 93 U/L). NA retreatment was initiated at the investigator’s discretion, resulting in re-suppression of HBV DNA without clinically relevant adverse events. In the other participant with HBsAg seroreversion and HBV DNA levels ≥ 100 IU/mL, ALT levels remained within the normal range throughout follow-up, and no NA retreatment was administered (Figure S4B). No cases of hepatic decompensation or HCC were observed during follow-up.

### 3.4. Factors Associated with HBsAg Seroreversion

**Figure 4:**
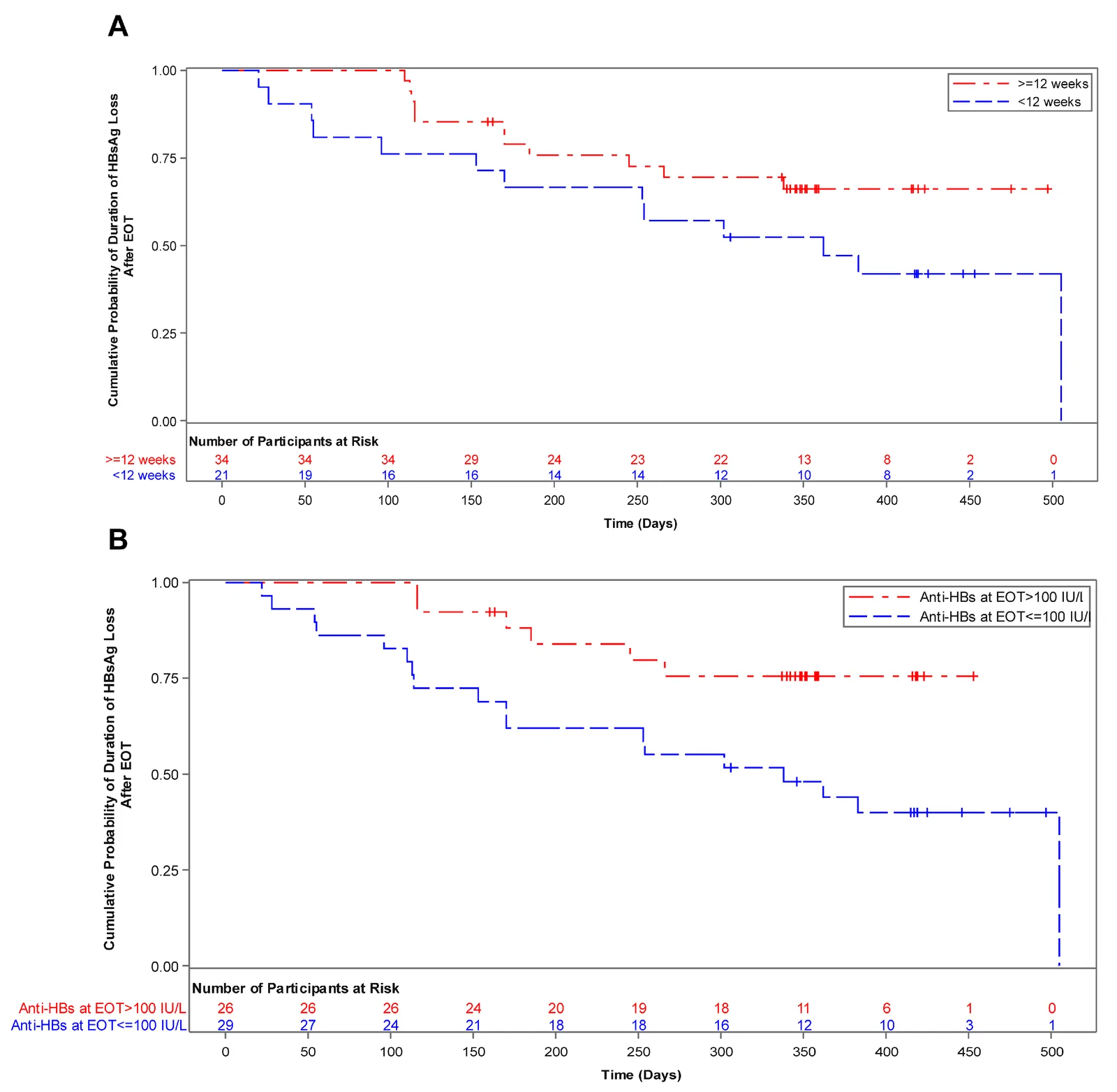
Kaplan–Meier plot of time to HBsAg seroreversion. (**A**) By PEG-IFNα-based study treatment duration after HBsAg loss. (**B**) By anti-HBs level at EOT (>100 IU/L or ≤100 IU/L). Log-rank *p* = 0.120 for panel A and *p* = 0.017 for panel (**B**). Anti-HBs, hepatitis B surface antibody; EOT, end of treatment.

Kaplan–Meier analyses (Figure 4 and Figure S5) and log-rank tests were performed to explore factors potentially associated with time to HBsAg seroreversion based on clinical relevance and biological plausibility. Time to HBsAg seroreversion was evaluated according to baseline HBeAg status; however, interpretation of findings in the HBeAg-positive subgroup was limited by the small sample size (n = 4). HBsAg seroreversion occurred in 3 of 4 (75.0%) HBeAg-positive participants compared with 21 of 51 (41.2%) HBeAg-negative participants. At Week 48 post-EOT, the estimated cumulative HBsAg seroreversion-free rate was 0.0% among HBeAg-positive participants and 66.3% (95% CI: 51.5–77.5) among HBeAg-negative participants, with a similar separation observed at Week 60 post-EOT. Consistent with these findings, the log-rank test indicated a numerical difference between groups that did not reach statistical significance (*p* = 0.072) (Table S3). Other baseline demographic characteristics, including age and sex, showed no apparent association with time to HBsAg seroreversion (Figure S5).

With respect to study treatment-related factors, neither treatment regimen (PEG-IFNα monotherapy versus PEG-IFNα combination therapy) nor therapeutic vaccine BRII-179 exposure was associated with HBsAg seroreversion, as indicated by overlapping Kaplan–Meier curves and non-significant log-rank test results (Tables S4 and S5, Figure S5). In contrast, a longer duration of PEG-IFNα-based study treatment after HBsAg loss showed a trend toward improved HBsAg seroreversion-free survival (Figure 4A). HBsAg seroreversion occurred in 32.4% (11/34) of participants who received ≥12 weeks of study treatment after HBsAg loss, compared with 61.9% (13/21) of those treated for <12 weeks. Kaplan–Meier analysis demonstrated separation of the seroreversion-free survival curves favoring longer treatment, although the difference was not statistically significant (log-rank *p* = 0.120; Table S6).

Given that HBsAg seroreversion frequently occurred before NA discontinuation, potentially confounding the assessment of post-discontinuation outcomes, analyses assessing the impact of NA consolidation duration were restricted to participants who maintained HBsAg loss throughout NA consolidation and subsequently discontinued NA therapy. Among the 41 participants who discontinued NA, 3/20 (15.0%) with NA consolidation durations < 24 weeks and 5/21 (23.8%) with NA consolidation durations ≥ 24 weeks experienced HBsAg seroreversion within 24 weeks after NA discontinuation (Table S1); the 3 participants in BRII-179-002 who developed HBsAg seroreversion between Weeks 24 and 48 off-NA had received 12, 12 and 23 weeks of NA consolidation, respectively. Kaplan–Meier curves comparing NA consolidation ≥ 24 weeks versus <24 weeks largely overlapped, and no significant difference in time to HBsAg seroreversion was identified by log-rank testing (*p* = 0.209; Table S7 and Figure S5). Consistently, the common-window (48-week observation window after EOT) sensitivity analysis (Table S8) also showed no statistically significant difference in HBsAg seroreversion-free survival between the NA consolidation-duration groups. The magnitude of HBsAg seroreversion likewise appeared to be unrelated to the duration of NA consolidation. By the last follow-up visit, maximum HBsAg levels remained <10 IU/mL in 4 of 5 participants with HBsAg seroreversion after <24 weeks of NA consolidation and in 5 of 6 participants with HBsAg seroreversion after ≥24 weeks of NA consolidation (Table S2).

Anti-HBs levels at EOT were significantly associated with subsequent HBsAg seroreversion. Kaplan–Meier analysis demonstrated early and sustained separation of seroreversion-free survival curves according to anti-HBs levels at EOT (Figure 4B). Participants with anti-HBs > 100 IU/L showed a consistently higher probability of maintaining HBsAg loss throughout follow-up than those with anti-HBs levels ≤ 100 IU/L, and the between-group difference was statistically significant by log-rank testing (*p* = 0.017; Table S9). To further explore factors associated with HBsAg seroreversion, a trial-stratified parsimonious Cox proportional hazards model including anti-HBs level at EOT and PEG-IFNα treatment duration after HBsAg loss was fitted (Table 3). No statistically significant evidence of violation of the proportional hazards assumption was observed for either covariate or the overall model, although the limited number of events reduced the power of these diagnostic assessments. An anti-HBs level >100 IU/L at EOT was associated with a lower observed hazard of HBsAg seroreversion (HR = 0.30, 95% CI: 0.10–0.92; *p* = 0.036), whereas PEG-IFNα-based treatment duration from HBsAg loss to EOT was not associated with the risk of HBsAg seroreversion.

**Figure 5:**
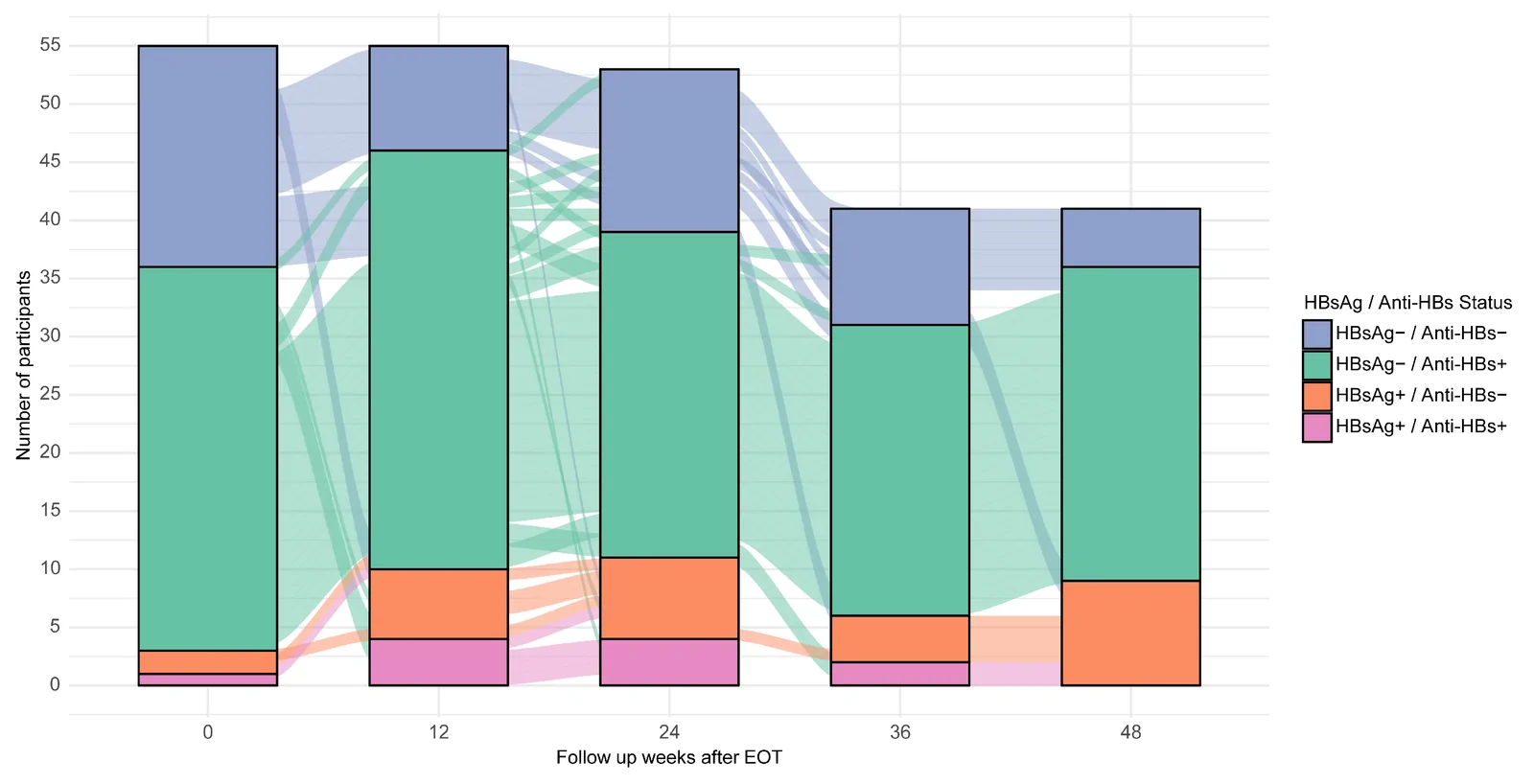
Sankey diagram for longitudinal transitions of HBsAg and anti-HBs serological status from EOT to 48 weeks post-EOT. Participants were categorized at each time point according to HBsAg level (<LLOQ versus ≥LLOQ) and anti-HBs status (<10 IU/L versus ≥10 IU/L), resulting in four serological states. The width of each colored block represents the number of participants at each visit, while the connecting flows indicate transitions in serological status over time. No statistical testing was performed. Anti-HBs, hepatitis B surface antibody; EOT, end of treatment; LLOQ, lower limit of quantification.

Among the 34 of the 55 participants (61.8%) with anti-HBs levels ≥ 10 IU/L at EOT, 24 maintained anti-HBs levels ≥ 10 IU/L through the last follow-up visit. In addition, 5 participants with anti-HBs levels < 10 IU/L at EOT subsequently achieved anti-HBs ≥ 10 IU/L during follow-up. To further characterize the temporal relationship between anti-HBs status and HBsAg seroreversion, longitudinal transitions between combined HBsAg (<LLOQ versus ≥LLOQ) and anti-HBs (<10 IU/L versus ≥10 IU/L) serological states from EOT to 48 weeks post-EOT were visualized using a Sankey diagram (Figure 5). Most participants with sustained HBsAg loss remained anti-HBs positive (≥10 IU/L) throughout follow-up, whereas HBsAg seroreversion occurred predominantly among those who were anti-HBs-negative at EOT or became anti-HBs-negative shortly thereafter. Although transient coexistence of HBsAg and anti-HBs was observed in a small number of participants, this state was generally unstable over time. Nevertheless, HBsAg seroreversion also occurred in a subset of participants despite persistent anti-HBs positivity. These visual patterns were corroborated by generalized linear mixed-effects modeling that accounted for repeated measurements and follow-up duration (Table S10). Anti-HBs level < 10 IU/L was associated with HBsAg seroreversion over time (*p* = 0.016), although the estimated odds ratio was large due to sparse data; the follow-up duration was also associated with an increased likelihood of HBsAg seroreversion (*p* = 0.020). Model-derived predicted probabilities highlighted a pronounced divergence by anti-HBs status. Individuals with anti-HBs < 10 IU/L showed a steep, time-dependent increase in the predicted probability of HBsAg seroreversion, whereas those maintaining anti-HBs ≥ 10 IU/L consistently exhibited minimal predicted HBsAg seroreversion risk throughout follow-up (Figure S6). Collectively, both trajectory-based visualization and quantitative mixed-effects modeling indicate a strong longitudinal association between anti-HBs negativity (<10 IU/L) and HBsAg seroreversion.

## 4. Discussion

It is increasingly recognized that on-treatment reductions in HBsAg achieved through direct suppression of viral antigen production do not necessarily translate into restoration of HBV-specific immune response. Following treatment discontinuation and drug washout, HBsAg rebound may occur, highlighting the importance of combining direct-acting antiviral agents with immunomodulators to achieve a durable functional cure [15]. In this context, despite the limitations associated with subcutaneous administration and its relatively unfavorable tolerability profile, PEG-IFNα continues to play a unique role in chronic HBV infection treatment through its combined antiviral activity and immunomodulatory effects [16]. Emerging clinical evidence suggests that PEG-IFNα-based combinations with novel anti-HBV agents can improve long-term off-treatment HBsAg loss rate [17], and that sequential PEG-IFNα treatment following NA or novel antiviral therapies may reduce the risk of post-treatment HBV relapse [18,19]; however, the kinetics of HBsAg seroreversion following PEG-IFNα-based novel-agent combinations and its association with HBV DNA and/or ALT elevation remain poorly characterized. Against this background, we performed a post hoc pooled analysis of two prospective phase 2 trials to evaluate the incidence, timing, magnitude, and clinical correlates of HBsAg seroreversion in participants who achieved HBsAg loss following PEG-IFNα monotherapy or PEG-IFNα combined with the therapeutic vaccine BRII-179 or the siRNA elebsiran. Potential predictors for HBsAg seroreversion were also explored. Several key findings emerged.

A key finding of this analysis is that detectable HBsAg following PEG-IFNα-based therapy should not necessarily be interpreted as loss of treatment benefit. Although HBsAg seroreversion occurred at rates comparable to those reported in other prospective studies [4], all HBsAg seroreversion events remained below 100 IU/mL, with the majority remaining below 10 IU/mL. Moreover, most participants who discontinued NA maintained sustained virologic suppression together with low-level or undetectable HBsAg throughout follow-up, and clinically relevant ALT elevation was rare. More than 90% of participants who discontinued NA achieved “partial cure”, defined as sustained HBsAg < 100 IU/mL with undetectable HBV DNA for at least 24 weeks off therapy [3]. Notably, all participants from BRII-179-002 who experienced HBsAg seroreversion within 24 weeks after NA discontinuation maintained HBsAg < 100 IU/mL through 48 weeks after NA discontinuation, supporting the durability of response despite HBsAg reappearance (Figure 3). Recently, it has been proposed that clinical trials should move beyond viewing functional cure as the sole endpoint and embrace a more individualized assessment of treatment response, recognizing partial cure as a meaningful intermediate treatment outcome on the pathway toward functional cure. This perspective is supported by evidence that partial cure is generally durable [20] and is associated with a low risk of clinical relapse and HCC [2]. Our findings support a more nuanced interpretation of post-treatment outcomes following finite PEG-IFNα-based therapies. Rather than a simple binary classification of functional cure versus relapse, post-treatment outcomes may span a spectrum ranging from sustained HBsAg loss to low-level HBsAg reappearance while maintaining virologic control. In this context, low-level HBsAg seroreversion with persistently suppressed HBV DNA may represent an acceptable and clinically favorable outcome rather than loss of meaningful therapeutic benefit.

Another important finding is the association observed between anti-HBs levels and the durability of HBsAg loss. Among all evaluated factors, anti-HBs level >100 IU/L at EOT was the strongest predictor of sustained HBsAg loss, consistent with findings from previous retrospective real-world studies [10,21]. Longitudinal mixed-effects modeling further demonstrated a time-dependent increase in HBsAg seroreversion risk among participants with negative anti-HBs. The association between anti-HBs positivity and reduced HBsAg seroreversion during follow-up was also consistent with prior observational studies [7,22]. Collectively, these findings support anti-HBs as a potential marker of post-treatment HBsAg loss durability. Importantly, HBsAg seroreversion occurred in a subset of participants despite the presence of anti-HBs. This observation is consistent with the concept that treatment-induced anti-HBs responses may be heterogeneous in functional quality [23,24] and therefore may not, on their own, confer complete protection against HBsAg reappearance. Consequently, anti-HBs levels should not be viewed as a sole basis for clinical decision-making. Rather, they may serve as a surrogate indicator of the robustness and quality of immune responses against HBV achieved at the end of therapy. While higher anti-HBs levels appear to identify individuals more likely to maintain off-treatment benefit after PEG-IFNα-based novel regimens, additional immunological mechanisms are likely required to ensure durable long-term suppression of HBsAg.

Previous studies reported that HBsAg loss occurring later during PEG-IFNα treatment, and consequently a shorter treatment duration after HBsAg loss (<12 weeks before EOT), was associated with an increased risk of HBsAg recurrence [7,8,9,22,25,26,27], whereas the potential benefit of NA consolidation therapy remains controversial [9,19]. The third key finding of our analysis was that a longer duration of PEG-IFNα-based treatment after HBsAg loss (≥12 versus <12 weeks) was associated with a numerically lower risk of HBsAg seroreversion, suggesting that prolonged immune modulation by PEG-IFNα may contribute to HBsAg loss durability. In contrast, no statistically significant association between the duration of NA consolidation and HBsAg seroreversion was observed in either the primary or the common-window sensitivity analyses. However, several factors limit the interpretation of this finding. Follow-up schedules differed according to trial design, NA consolidation duration was not prospectively randomized, and only a small number of HBsAg seroreversion events occurred after NA discontinuation. Consequently, the analysis was likely underpowered to detect modest effects of consolidation duration.

In addition, baseline HBeAg positivity also showed a trend toward being associated with HBsAg seroreversion in Kaplan–Meier analyses. Given the extremely small number of HBeAg-positive participants, this observation should be viewed as a potential signal rather than a definitive predictor. Nevertheless, its consistency with previous reports [27,28] suggested that residual viral replicative activity or underlying immune tolerance at baseline may influence HBsAg loss durability after treatment completion, warranting closer post-treatment monitoring of this population and further evaluation in larger prospective studies.

These findings have several implications for future clinical trial design. First, the modest magnitude and limited clinical consequences of HBsAg seroreversion suggest that “partial cure” merits further evaluation as an endpoint reflecting serologic and virologic durability after treatment discontinuation. Second, anti-HBs warrants further validation as a candidate biomarker for risk-adapted post-treatment monitoring. Third, the observed trend toward a lower risk of HBsAg seroreversion with longer PEG-IFNα-based treatment after HBsAg loss and the lack of apparent association between prolonged NA consolidation and HBsAg seroreversion, together with the absence of significant HBV DNA rebound or ALT flares following NA discontinuation, suggests that it may be clinically feasible and safe to evaluate PEG-IFNα and NA consolidation strategies and determine their optimal duration for achieving and maintaining HBV functional cure in future prospective trials.

This study has several limitations. First, the sample size and number of HBsAg seroreversion events were modest, resulting in a relatively low event-to-covariate ratio in the multivariable Cox proportional hazards model, which may increase the risk of overfitting and unstable effect estimates. Accordingly, model-derived associations should be regarded as exploratory rather than confirmatory. Second, this was a post hoc pooled analysis of two trials that differed in design, baseline HBeAg status and HBsAg levels, treatment regimens, NA consolidation schedules, and follow-up duration. Although common variable definitions and trial-stratified analyses were applied, residual between-trial heterogeneity and confounding cannot be excluded. In particular, the observed association between baseline HBeAg positivity and HBsAg seroreversion should be interpreted with considerable caution because only four participants in ENSURE were HBeAg-positive. Furthermore, follow-up duration was determined by trial-specific discontinuation schedules, and the number of HBsAg seroreversion events after NA discontinuation was small. Consequently, the absence of a statistically significant association between NA consolidation duration and HBsAg seroreversion should not be interpreted as evidence that prolonged NA consolidation provides no clinical benefit. Third, the analysis was restricted to participants who achieved HBsAg loss and therefore may not be generalizable to all individuals receiving PEG-IFNα-based therapy. In addition, most participants were Asian and HBeAg-negative, which may also limit the generalizability of the findings. Fourth, given the persistence of covalently closed circular DNA (cccDNA) in hepatocytes of individuals with chronic HBV infection, the absence of liver histological or intrahepatic virologic assessments represents a limitation of this study. Moreover, HBsAg reappearance after treatment discontinuation could reflect renewed antigen expression from transcriptionally active cccDNA, ongoing HBsAg production from integrated HBV DNA, or both, and the present dataset cannot distinguish among these mechanisms. The lack of observed HBsAg seroreversion or quantifiable HBV DNA (≥ 10 IU/mL) during follow-up does not necessarily exclude ongoing occult intrahepatic viral activity. Therefore, our findings inform the durability of serologic responses rather than sterilizing virologic cure. Finally, multiple subgroup and time-to-event analyses were conducted without adjustment for multiplicity. As a result, nominal *p* values may be susceptible to inflated type I error. Accordingly, all findings should be considered hypothesis-generating and require confirmation in larger, prospective trials with longer off-treatment follow-up.

## 5. Conclusions

HBsAg seroreversion after PEG-IFNα-based therapy, with or without the therapeutic vaccine BRII-179 or the siRNA elebsiran, occurred in a subset of participants but was generally modest in magnitude and infrequently associated with virologic or biochemical relapse. Higher anti-HBs levels at EOT and persistent anti-HBs positivity post-EOT were associated with greater durability of HBsAg loss and warrant prospective evaluation as a potential biomarker to inform risk-adapted off-treatment monitoring. Exploratory analyses showed numerically higher HBsAg seroreversion rates among HBeAg-positive participants and among those with PEG-IFNα treatment duration less than 12 weeks after HBsAg loss, although these observations should be interpreted cautiously given the limited number of events, trial-specific design differences, and lack of statistical significance. In addition, the relationship between prolonged NA consolidation and HBsAg seroreversion remains to be established and requires further investigation in adequately powered prospective studies. Collectively, these hypothesis-generating findings contribute to a more nuanced interpretation of post-treatment serological outcomes, suggest that “partial cure” may merit further evaluation as an intermediate endpoint reflecting post-treatment serologic and virologic durability, and may help inform the design of future trials evaluating strategies to achieve durable HBV functional cure.

## Figures and Tables

**Table 1:** Baseline demographic and disease characteristics for pooled participants.

Characteristics	Overall (N = 55)
Age, years	
Mean (SD)	41.93 (8.22)
Age group, n (%)	
≤45 years	35 (63.6)
>45 years	20 (36.4)
Sex, n (%)	
Female	18 (32.7)
Male	37 (67.3)
Race, n (%)	
Asian	54 (98.2)
White	1 (1.8)
BMI at baseline, kg/m^2^	
Mean (SD)	22.77 (3.32)
BMI group, n (%)	
<24 kg/m^2^	35 (63.6)
≥24 kg/m^2^	20 (36.4)
HBeAg status at baseline, n (%)	
Negative	51 (92.7)
Positive	4 (7.3)
Treatment on top of NA background therapy, n (%)	
PEG-IFNα combination therapy	42 (76.4)
PEG-IFNα monotherapy	13 (23.6)
Anti-HBs at EOT, n (%)	
>100 IU/L	26 (47.3)
>10 and ≤100 IU/L	8 (14.5)
≤10 IU/L	21 (38.2)
ALT at EOT, n (%)	
≤40 U/L	24 (43.6)
>40 U/L	31 (56.4)
PEG-IFNα-based treatment duration after HBsAg loss, weeks	
Median	17.90
Min, Max	0.0, 36.9
PEG-IFNα-based treatment duration after HBsAg loss by group, n (%)	
≥12 weeks	34 (61.8)
<12 weeks	21 (38.2)
NA consolidation duration, weeks	
Median	23.90
Min, Max	10.1, 29.0
NA consolidation duration by group, n (%)	
≥24 weeks	32 (58.2)
<24 weeks	23 (41.8)

PEG-IFNα-based treatment duration after HBsAg loss: Treatment duration of PEG-IFNα with or without BRII-179/elebsiran from HBsAg loss to EOT; NA consolidation period: participants did not discontinue NA = duration from EOT to EOS; participants discontinued NA = duration from EOT to NA discontinuation monitoring period Day 1. Analysis visit window for the 24-week NA consolidation period was defined as within 2 weeks of the target visit. ALT, alanine aminotransferase; anti-HBs, hepatitis B surface antibody; BMI, body mass index; EOS, end of study; EOT, end of treatment; n, number of participants; HBeAg, hepatitis B e antigen; HBsAg, hepatitis B surface antigen; IU, international unit; max, maximum; min, minimum; NA, nucleos(t)ide analogue; PEG-IFNα, pegylated interferon alpha; SD, standard deviation.

**Table 2:** Summary of hepatitis B surface antigen seroreversion rates in overall and subgroup populations.

	HBsAg Seroreversion Rate n/N (%)	95% Confidence Interval
Overall	24/55 (43.6)	(30.3, 57.7)
Age		
≤45 years	19/35 (54.3)	(36.6, 71.2)
>45 years	5/20 (25.0)	(8.7, 49.1)
Sex		
Female	9/18 (50.0)	(26.0, 74.0)
Male	15/37 (40.5)	(24.8, 57.9)
BMI at baseline		
<24 kg/m^2^	16/35 (45.7)	(28.8, 63.4)
≥24 kg/m^2^	8/20 (40.0)	(19.1, 63.9)
HBeAg status at baseline		
Negative	21/51 (41.2)	(27.6, 55.8)
Positive	3/4 (75.0)	(19.4, 99.4)
Treatment		
PEG-IFNα-based combination	18/42 (42.9)	(27.7, 59.0)
PEG-IFNα monotherapy	6/13 (46.2)	(19.2, 74.9)
PEG-IFNα-based treatment duration after HBsAg loss		
≥12 weeks	11/34 (32.4)	(17.4, 50.5)
<12 weeks	13/21 (61.9)	(38.4, 81.9)
Anti-HBs at EOT		
>100 IU/L	6/26 (23.1)	(9.0, 43.6)
≤100 IU/L	18/29 (62.1)	(42.3, 79.3)
ALT at EOT		
≤40 U/L	10/24 (41.7)	(22.1, 63.4)
>40 U/L	14/31 (45.2)	(27.3, 64.0)

Two-sided 95% confidence intervals were calculated using the Clopper–Pearson exact method. ALT, alanine aminotransferase; anti-HBs, hepatitis B surface antibody; BMI, body mass index; CI, confidence interval; EOT, end of treatment; HBeAg, hepatitis B e antigen; HBsAg, hepatitis B surface antigen; IU, international unit; n, number of participants with HBsAg seroreversion; N, number of participants in the subgroup; PEG-IFNα, pegylated interferon alpha.

**Table 3:** Exploratory trial-stratified Cox proportional hazards model for time to hepatitis B surface antigen seroreversion.

Variable	Hazard Ratio	95% Confidence Interval	*p* Value
Anti-HBs level at EOT (>100 versus ≤100 IU/L)	0.30	(0.10, 0.92)	0.036
PEG-IFNα-based treatment duration from HBsAg loss to EOT (≥12 versus <12 weeks)	0.82	(0.32, 2.09)	0.679

Hazard ratios were estimated using a Cox proportional hazards model stratified by parent trial, with STUDYID specified in the STRATA statement. Stratification allowed separate baseline hazard functions for BRII-179-002 and ENSURE while estimating common covariate effects across studies. Anti-HBs, hepatitis B surface antibody; EOT, end of treatment; HBsAg, hepatitis B surface antigen; IU, international unit; PEG-IFNα, pegylated interferon alpha.

## Data Availability

The data presented in this study are available on reasonable request from the corresponding authors. The data are not publicly available because they contain participant-level clinical trial data and are subject to applicable privacy, ethical, regulatory, and contractual restrictions.

## Supplementary Materials

The following supporting information can be downloaded at: https://www.mdpi.com/article/10.3390/vaccines14100868/s1, Supplementary Data: List of Investigators and Institutional Review Boards/Ethics Committees for BRII-179-002 and ENSURE; Supplementary Methods for BRII-179-002 and ENSURE; Table S1: Summary of HBsAg seroreversion rates in participants who discontinued NA; Table S2: Summary of maximum HBsAg levels after seroreversion; Table S3: Cumulative incidence and log-rank test for time to HBsAg seroreversion by baseline HBeAg status; Table S4: Cumulative incidence and log-rank test for time to HBsAg seroreversion by treatment regimen; Table S5: Cumulative incidence and log-rank test for time to HBsAg seroreversion (with BRII-179 exposure vs. without BRII-179 exposure); Table S6: Cumulative incidence and log-rank test for time to HBsAg seroreversion by PEG-IFNα-based treatment duration after HBsAg loss; Table S7: Cumulative incidence and log-rank test for time to HBsAg seroreversion by NA consolidation duration among participants who discontinued NA; Table S8: Cumulative incidence and log-rank test for time to HBsAg seroreversion by NA consolidation duration among participants who discontinued NA—sensitivity analysis in the common 48-week observation window after EOT; Table S9: Cumulative incidence and log-rank test for time to HBsAg seroreversion by anti-HBs level at EOT; Table S10: Association between anti-HBs status and HBsAg seroreversion within 48 weeks after EOT estimated using a logistic mixed-effects model; Figure S1: Trial design of BRII-179-002; Figure S2: Trial design of ENSURE; Figure S3: Participant disposition in BRII-179-002 and ENSURE; Figure S4: Participants with HBsAg seroreversion and HBV DNA ≥ 100 IU/mL; Figure S5: Kaplan–Meier plots of time to HBsAg seroreversion; Figure S6: Model-based predicted probability of HBsAg seroreversion according to anti-HBs status over follow-up. File S1: BRII-179-002 clinical study protocol and amendments; File S2: BRII-179-002 statistical analysis plan and amendments; File S3: ENSURE clinical study protocol and amendments; File S4: ENSURE statistical analysis plan and amendments.
