# Supplementary material for: Profile and Predictors of Hepatitis B Surface Antigen Seroreversion Following PEG-IFNα with or Without Therapeutic Vaccine BRII-179 or siRNA Elebsiran: A Post Hoc Pooled Analysis of Two Randomized Controlled Trials in Patients with Chronic Hepatitis B Virus Infection
Source: Vaccines (Basel). 2026 Oct 1;14(10):868. doi: 10.3390/vaccines14100868 (PMC13709818; doi:10.3390/vaccines14100868)
Supplement: Supplementary file 1 [file vaccines-14-00868-s001.zip › Supplementary Data.pdf]

Profile and predictors of hepatitis B surface antigen seroreversion following PEG-IFNα with or without therapeutic vaccine BRII-179 or siRNA elebsiran: a post hoc pooled analysis of two randomized controlled trials in patients with chronic hepatitis B virus infection

Table of Contents

List of Investigators and Institutional Review Boards/Ethics Committees 2
BRII-179-002 Study 2
ENSURE Study 3
Supplementary Methods 6
BRII-179-002 Study Design 6
Inclusion Criteria 7
Exclusion Criteria 8
ENSURE Study Design 12
Supplementary Tables 21
Table S1. Summary of HBsAg Seroreversion rates in participants discontinued NA 21
Table S2. Summary of Maximum HBsAg Level after Seroreversion 21
Table S3. Cumulative Incidence and Log-rank Test for Time to HBsAg Seroreversion (HBeAg at Baseline Positive versus Negative) 22
Table S4. Cumulative Incidence and Log-rank Test for Time to HBsAg Seroreversion (PEG-IFNα based Combinations versus PEG-IFNα monotherapy) 22
Table S5. Cumulative Incidence and Log-rank Test for Time to HBsAg Rebound (With BRII-179 vs. Without BRII-179) 23
Table S6. Cumulative Incidence and Log-rank Test for Time to HBsAg Seroreversion (PEG-IFNα based treatment duration after HBsAg loss ≥12 versus<12 weeks) 23
Table S7. Cumulative Incidence and Log-rank Test for Time to HBsAg Seroreversion (NA consolidation duration ≥24 versus<24 weeks) in Participants discontinued NA and had HBsAg < LLOQ on the first day of NA Discontinuation 24
Table S8. Cumulative Incidence and Log-rank Test for Time to HBsAg Seroreversion (NA consolidation duration ≥24 versus<24 weeks) in Participants discontinued NA and had HBsAg < LLOQ on the first day of NA Discontinuation – Sensitivity Analysis in the Common Follow Up Window 24
Table S9. Cumulative Incidence and Log-rank Test for Time to HBsAg Seroreversion (Anti-HBs Level at EOT >100 versus ≤100 IU/L) 25
Table S10. Association between anti-HBs status and HBsAg seroreversion within 48 weeks after EOT estimated using a logistic mixed-effects model 26
Supplementary Figures 27
Fig. S1. Trial design of BRII-179-002 27
Fig. S2. Trial design of ENSURE 27
Fig. S3. Participant disposition in BRII-179-002 and ENSURE 28
Fig. S4. Participants with HBsAg seroreversion with HBV DNA ≥ 100 IU/mL. 29
Fig. S5. Kaplan-Meier plots of time to HBsAg seroreversion 30
Fig. S6. Model-based predicted probability of HBsAg seroreversion according to anti-HBs status over follow-up 31

List of Investigators and Institutional Review Boards/Ethics Committees

BRII-179-002 Study

Investigator Name	Name / Address of Ethics Committee	Initial Approval Number* /Date
Zhiliang Gao	Name: Ethics Committee of Third Affiliated Hospital, Sun Yat-Sen University<br>Address: No. 600, Tianhe Road, Tianhe District, Guangzhou	2021-017-01/30 Jun 2021
Jiangping Li	Name: Ethics Committee of Guangzhou Eighth People's Hospital, Guangzhou Medical University<br>Address: 8 Huaying Road, Baiyun District, Guangzhou	202115170/30 Sep 2021
Jun Chen	Name: Ethics Committee of Shenzhen Third People's Hospital<br>Address: No. 29, Buji Bulan Road, Longgang District, Shenzhen	2021-003/09 Oct 2021
Xinyue Chen	Name: Ethics Committee of Beijing Youan Hospital, Capital Medical University<br>Address: No.8, Xitoutiao, You 'an Gate, Fengtai District, Beijing	2021-159 /06 Sep 2021
Libo Yan	Name: Ethics Committee of Clinical Trial, West China Hospital of Sichuan University<br>Address: No.37, Guoxue Lane, Wuhou District, Chengdu City, Sichuan Province	2021-213/06 Sep 2021
Rong Hu	Name: Ethics Committee of Public health clinical center of Chengdu<br>Address: No. 18 Jingjusi Road, Jinjiang District, Chengdu	2021-011-01/16 Nov 2021
Peng Hu	Name: Ethics Committee of the Second Affiliated Hospital of Chongqing Medical University<br>Address: 74 Linjiang Road, Yuzhong District, Chongqing	2021-92-1/24 Nov 2021
Qing Xie	Name: Ruijin Hospital Ethics Committee<br>Address: No. 197, Ruijin 2nd Road, Shanghai, China	2021-144/17 Dec 2021
Jiming Zhang	Name: Ethics Committee of Huashan Hospital, Fudan University<br>Address: 12 Urumqi Middle Road, Shanghai	2021-946/24 Nov 2021
Fangfang Lv	Name: Ethics Committee, Sir Run Run Shaw Hospital, Zhejiang University School of Medicine<br>Address: No. 3 Qingchun East Road, Shangcheng District, Hangzhou	20211109-2/16 Nov 2021

Investigator Name	Name / Address of Ethics Committee	Initial Approval Number* /Date
Yan Huang	Name: Medical Ethics Committee of Xiangya Hospital Central South University<br>Address: 87 Xiangya Road, Changsha, Hunan	202112245/20 Dec 2021
Yingren Zhao	Name: Ethics Committee of The First Affiliated Hospital of Xi'An Jiaotong University<br>Address: 277 Yanta West Road, Xi 'an City	2021-187/09 Oct 2021
Yueyong Zhu	Name: Ethics Committee of The First Affiliated Hospital of Fujian Medical University<br>Address: 20 Cha-Zhong Road, Fuzhou City	2021-056-01/10 Nov 2021
Jiabin Li	Name: Ethics Committee of the First Affiliated Hospital of Anhui Medical University<br>Address: No.218, Jixi Road, Hefei City	2021-14-05/04 Nov 2021
Xiaobo Lu	Name: Ethic Committee of the First Affiliated Hospital of Xinjiang Medical University<br>Address: No. 137, Liyue Shan South Road, High-tech Zone, Urumqi, Xinjiang Uygur Autonomous Region	D211027-01/02 Nov 2021
Jiawei Geng	Name: Ethics Committee of the First People's Hospital of Yunnan Province<br>Address: 157 Jinbi Road, Xishan District, Kunming City, Yunnan Province	2021-022/12 Nov 2021
Qin Ning	Name: Clinical Trial Ethics Committee of Huazhong University of Science and Technology<br>Address: 1095 Jiefang Avenue, Wuhan City, Hubei Province	2022-69/27 Apr 2022

* Approvals for amendment review, resubmission, etc. numbered by "initial approval number-xx" or any other similar format that starts with the initial approval number are not detailed in this table.

ENSURE Study

Investigator Name	Name / Address of EC/IRB	Initial Approval Number* /Date
Martin Weltman	Nepean Blue Mountains Local Health District Ethics Committee (Grouped sites All AU Sites)<br>Nepean Hospital, Derby St, Penrith NSW 2750	2023/ETH00904 /27 Mar 2024
James O'beirne	Nepean Blue Mountains Local Health District Ethics Committee (Grouped sites All AU Sites)<br>Nepean Hospital, Derby St, Penrith NSW 2750	
Mark William Douglas	Nepean Blue Mountains Local Health District Ethics Committee (Grouped sites All AU Sites)<br>Nepean Hospital, Derby St, Penrith NSW 2750	

Investigator Name	Name / Address of EC/IRB	Initial Approval Number* /Date
Jeong Heo	Pusan National University Hospital Institutional Review Board<br><br>3F, Biomedical Research Institution Building, 179, Gudeok-Ro, Seo-Gu, Busan, 49241, Korea	2306-008-138/04 Jul 2023
Dong Joon Kim	Hallym University Chuncheon Sacred Heart Hospital Institutional Review Board<br><br>B1, Suin Building, 84 Sakju-ro, Chuncheon-si, Gangwon-do, Republic of Korea	CHUNCHEON2023-06-001/19 Jun 2023
Yong Han Paik	Samsung Medical Center Institutional Review Board<br>81, Irwon-ro,Gangnam-gu, Seoul,Republic of Korea	SMC2023-06-005/28 Jun 2023
Won Young Tak	Kyungpook National University Hospital Institutional Review Board<br><br>1st Floor, 3-dong, 130, Dongdeok-ro, Jung-gu, Daegu, Republic of Korea, 41944	KNUH2023-05-034/26 Jun 2023
Jooho Lee	CHA University Bundang Medical Center Institutional Review Board<br><br>Clinical Trials Center, 3rd Floor, CHA Bundang Medical Center, 64 Yatap-ro, Bundang-gu, Seongnam-si, Gyeonggi-do, Republic of Korea	CHAMC2023-10-237/08 Nov 2023
Young-Suk Lim	Asan Medical Center Institutional Review Board<br><br>88, Olympic-ro 43-gil, Songpa-gu, Seoul, Republic of Korea	S2023-2321-0001/16 Nov 2023
Man Fung Yuen	Institutional Review Board of The University of Hong Kong/Hospital Authority Hong Kong West Cluster (HKU/HA HKW IRB)<br>Room 901, 9/F, Administration Block, Queen Mary Hospital, 102 Pok Fu Lam Road, Hong Kong	UW 23-342/04 Jul 2023
Grace Lai-hung Wong	Joint Chinese University of Hong Kong-New Territories East Cluster Clinical Research Ethics Committe<br><br>8/F, Lui Che Wii Clinical Sciences Building, Prince of Wales Hospital, Sha Tin, Hong Kong	2023.268-T/10 Jul 2023
Rajneesh Kumar	SingHealth Centralised Institutional Review Board (CIRB) / Singapore Health Services Pte Ltd, 10 Hospital Boulevard, #19-01 SingHealth Tower, S168582	2023/2312 /11 Aug 2023
Rahul Kumar	SingHealth Centralised Institutional Review Board (CIRB) / Singapore Health Services Pte Ltd, 10 Hospital Boulevard, #19-01 SingHealth Tower, S168582	
Chia-yen Dai	Kaohsiung Medical University Chung-Ho Memorial Hospital Institutional Review Board/ No. 100, Ziyou 1st Rd., Sanmin Dist., Kaohsiung City 807, Taiwan	KKMUHIRB-F(II)-20230127/13 Sep 2023
Chun-jen Liu	National Taiwan University Hospital Research Ethics Committee/ 7, Chung - Shan South Road, Taipei, Taiwan 100	202306008MSB/30 Jun 2023

Investigator Name	Name / Address of EC/IRB	Initial Approval Number* /Date
Yi-Hsiang Huang	Taipei Veterans General Hospital Institutional Review Board /No.201, Sec. 2, Shipai Rd., Beitou District, Taipei City, Taiwan 11217	2023-10-001AU/09 Sep 2023
Pisit Tangkijvanich	Institutional Review Board Faculty Of Medicine Chulalongkorn University/ 1873 Rama 4 Road, Patumwan, Bangkok 10330, Thailand	0673/66 /16 Aug 2023
Witsarut Manasirisuk	Center for Ethics in Human Research, Khon Kaen University/ Room 5317, 3rd Floor Wadwichakarn Building, Faculty of Medicine, Khon Kaen University, Mittraphap road, Nai Muang Subdistrict, Muang District, Khon Kaen, 40002 Thailand	IRB00001189/13 Sep 2023
Suparat Khemnark	Institutional Review Board, Bamrasnaradura Infectious Disease Institute/ Bamrasnaradura Infectious Diseases Institute, 3rd Floor, Administrative building 38 Soi. Tiwanon 14, Tiwanon Rd, Talat Khwan Subdistrict, Muang District, Nonthaburi, 11000 Thailand	COA-CREC071/2023 /16 Aug 2023
Apinya Leerapun	Research Ethics Committee, Faculty of Medicine, Chiang Mai University/ 110 Intavaroros Rd., Amphoe Muang, Chiang Mai, Thailand 50200	
Teerha Piratvisuth	Human Research Ethics Unit Faculty of Medicine, Prince Of Songkla University/ 4th floor, Administrative Building, Faculty of Medicine, Prince of Songkla University,15 Kanjanawanit Rd., Hat Yai, Thailand 90110	
Jidong Jia	Bioethics Committee, Beijing Friendship Hospital, Capital Medical University/ No.95 Yong'an Road, Xicheng District, Beijing	JXHL2300112/26 Jul 2023
Peng Hu	The Second Affiliated Hospital of Chongqing Medical University/ No. 74 Linjiang Road, Yuzhong District, Chongqing	2023-125/02 Aug 2023
Bingliang Lin	The Third Affiliated Hospital of Sun Yat-sen University/No. 600 Tianhe Road, Tianhe District, Guangzhou City, Guangdong Province	YW2023-041/30 Jan 2024
Xinyue Chen	Ethics Committee, Beijing You'an Hospital, Capital Medical University/ No.8, West Toutiao, You’anmenwai, Fengtai District, Beijing, China	2023-156 /25 Sep 2023
Junqi Niu	Ethics Committee of the First Hospital of Jilin University/ No.1 Xinmin Street, Changchun City, Jilin Province, China	23Y276-001/27 Sep 2023
Fangfang Lv	Ethics Committee of Sir Run Run Shaw Hospital, Zhejiang University School of Medicine/ No. 3 Qingchun East Road, Hangzhou City, Zhejiang Province, China	2023-1266/25 Aug 2023
Qing Xie	Shanghai Jiao Tong University School of Medicine, Ruijin Hospital Ethics Commitee/ No. 197, Rui Jin 2nd Road, Shanghai, China	2023-102/01 Sep 2023

* Approvals for amendment review, resubmission, etc. numbered by “initial approval number-xx” or any other similar format that starts with the initial approval number are not detailed in this table.

Supplementary Methods

BRII-179-002 Study Design

BRII-179-002 was a multicenter, randomized, double-blind, placebo-controlled, parallel-group Phase 2 study aimed to evaluate the clinical effect of adding BRII-179 to existing PEG-IFNα and nucleos(t)ide analogue (NA) therapy in participants with chronic HBV infection without cirrhosis. This study consisted of 2 parts: Phase 2a and 2b. The Phase 2a part was initiated on 10 Dec 2021 and conducted at 17 centers that randomized 114 participants in China. This part was completed on 12 Jul 2024. The Phase 2b part was not initiated.

Fig. S1 provides a schematic of the study design of Phase 2a part.

Eligible HBeAg negative participants with chronic HBV infection without cirrhosis who received PEG-IFNα for 24 to 28 doses and NA therapy for at least 12 months with HBV DNA< LLOQ for ≥ 6 months were randomized 1:1 to receive BRII 179 40 μg or 0.9% sodium chloride placebo intramuscular (IM) injection every 3 weeks for a total of 7 doses over 18 weeks in addition to the existing PEG-IFNα for 24 weeks and continuous NA therapy. Participants were stratified at randomization by screening HBsAg level (3 categories: ≥0.05 to < 1 IU/mL, ≥1 to < 10 IU/mL, and ≥10 to <100 IU/mL).

The estimated total duration for each participant included screening up to 28 days, 24-week dosing period, and 24-week follow-up period. Participants completed investigational treatment with BRII 179 or placebo at Week 18; PEG-IFNα at Week 24, while NA treatment continued into the follow-up period.

The investigator evaluated whether participants were eligible for discontinuation of NA therapy starting from Week 36 and every 4 weeks until Week 48 based on the following NA discontinuation criteria:

- Alanine aminotransferase (ALT) < 2 × upper limit of normal (ULN) (results from previous visit) AND
- HBV DNA < LLOQ (results from previous visit) AND
- Two consecutive assessments of HBsAg < 0.05 IU/mL (assessed starting from Week 28 laboratory results)

Participants who discontinued their NA therapy were followed in the NA discontinuation monitoring period (NDMP) for an additional 48 weeks from the day of NA discontinuation.

Inclusion Criteria

Participants who met all of the following criteria were eligible for inclusion into the study:

1. A signed and dated written ICF prior to study participation.
2. Age ≥ 18 (or age of legal consent, whichever was older), and ≤ 60 at the time of screening.
3. Documented chronic HBV infection (positive serum HBsAg for ≥ 6 months, negative hepatitis B surface antibody [HBsAb]).
4. Negative HBeAg.
5. HBsAg ≥ 0.05 IU/mL and < 100 IU/mL at screening.
6. HBV DNA < LLOQ at screening (central lab).
7. Must have been receiving first-line NRTI for example, entecavir or tenofovir (e.g. TDF, tenofovir amibufenamide or TAF, etc) therapy for ≥ 12 months (with evidence of HBV DNA<LLOQ for ≥ 6 months, local lab results accepted) and PEG-IFNα for 24 to 28 doses (for participants who were not strictly administered once a week due to objective reasons such as adverse reactions, the number of treatment weeks should not exceed treatment

doses by 4) of the Day 1 visit randomization and could provide the related treatment history.

8. Female participants must have a negative pregnancy test or confirmation of post-menopausal status. Post-menopausal status is defined as 12 months with no menses without alternative medical cause. Women of childbearing potential (WOCBP) must have a negative serum pregnancy test at screening and a negative urine pregnancy test on Day 1, could not be breast feeding, and must be willing to use highly effective methods of contraception 14 days before study intervention administration through 12 weeks after last study intervention administration.
9. Agreed not to donate blood during the duration of the study.
10. Serum ALT and aspartate aminotransferase (AST) ≤ 5 × ULN at screening.

Exclusion Criteria

Participants who met any of following criteria were not eligible for inclusion into the study:

1. Any clinically significant chronic medical condition other than chronic HBV infection that, in the opinion of the Investigator, made the patient unsuitable for participation in the study.
2. Any clinically significant acute condition such as fever (> 38 °C) or acute respiratory illness within 7 days of first study intervention administration.
3. Significant liver fibrosis or cirrhosis as defined by having a FibroScan result of > 9 kilopascal (kPa) at screening, unless the participant had a result of ≤ 9 kPa from a FibroScan evaluation performed within 6 months prior to screening or liver biopsy performed within 1 year prior to screening that confirmed the absence of Metavir F3

fibrosis or F4 cirrhosis. If a participant had both procedures in the specified timeframes prior to screening, the most recent result should have been used to determine eligibility.

4. History or evidence of drug or alcohol abuse as assessed by the Investigator, within 12 months before screening.
5. History of intolerance to IM injection.
6. History of clinically significant chronic liver disease from any cause other than chronic HBV infection.
7. History of hepatic decompensation, including ascites, hepatic encephalopathy and/or esophageal or gastric varices.
8. Treatment by immunosuppressant (including but not limited to corticosteroids at a prednisolone equivalent dose > 20 mg/day) within 30 days before study intervention administration at the Day 1 visit and throughout the duration of study participation.
9. Known history of immunological function impairment, including but not limited to:
   - Autoimmune disease (e.g., multiple sclerosis, type 1 diabetes mellitus, myasthenia gravis, Crohn's disease and other inflammatory bowel diseases, celiac disease, systemic lupus erythematosus, scleroderma, including diffuse systemic form and limited cutaneous form of systemic sclerosis or CREST syndrome, systemic sclerosis, dermatomyositis polymyositis, rheumatoid arthritis, juvenile idiopathic arthritis, autoimmune thyroiditis including Hashimoto thyroiditis, Grave's or Basedow's disease, immune thrombocytopenic purpura, autoimmune hemolytic anemia, autoimmune hepatitis, psoriasis, vitiligo, vasculitis, Guillain Barré syndrome, Addison's disease, Bell's palsy, and alopecia areata); OR

- Primary immunodeficiency disorders (e.g., common variable immune deficiency, defective phagocytic cell function and neutropenia syndromes, and complement deficiency); OR
- Secondary immunodeficiency disorders (e.g., resulting from acquired immunodeficiency syndrome caused by human immunodeficiency virus [HIV] infection, solid organ transplant, and splenectomy).

10. Any HBV vaccine (licensed or experimental) prior to study enrollment and throughout the duration of study participation.
11. Immunization with attenuated vaccines (e.g., Mumps, Measles, Rubella) within 30 days before study intervention administration at the Day 1 visit and throughout the duration of study participation.
12. Had received blood products or immunoglobulin within 90 days before study intervention administration at the Day 1 visit or was likely to require blood products during the study.
13. Participated in another clinical trial with an investigational drug or a biologic within 30 days or within 5 drug half-lives (whichever is longer) before study intervention administration at the Day 1 visit. Participants must agree not to take part in any other study at any time during their participation in this study, inclusive of the follow-up period.
14. Previous or current psychiatric condition that precludes compliance with the protocol. Specifically excluded were persons with psychoses, ongoing risk for suicide, or history of suicide attempt or gesture within the past 5 years.
15. The participant had any history of cancer requiring locoregional or systemic therapy within 5 years of randomization or had current disease. Participants with completely surgically

resected tumors with no evidence of disease for more than 3 years, or a history of low risk basal cell carcinoma were accepted (low risk being defined as 1) location on the trunk of the body, arms, legs, cheeks, forehead, temples, scalp, neck, or chin, 2) < 2 cm in size, 3) nodular or superficial, 4) primary cancer that had not come back after treatment, 5) edge of the cancerous area was clear and smooth, and 6) not located in or around nerves).

16. Any skin abnormality or tattoo that would limit post treatment injection site assessment.
17. Known hypersensitivity or contraindication to BRII-179 or its excipients.
18. Unwilling, or unable in the opinion of the Investigator, to comply with study requirements, including the use of an adequate birth control method.
19. Active infection with HIV, hepatitis C virus or hepatitis Delta virus.
20. Donated more than 500 mL of blood within 90 days before study intervention administration at the Day 1 visit.
21. Renal impairment with estimated glomerular filtration rate (eGFR) ≤ 60 ml/min/1.73 m^2 at screening.
22. Uncontrolled diabetes mellitus as determined by the Investigator.
23. Diagnosis of advanced stage heart failure (New York Heart Association Class III or Class IV) or unstable angina.
24. Any laboratory test abnormality that is considered as clinically significant or may contradict continued treatment with PEG-IFNα by the Investigator.

ENSURE Study Design

ENSURE (or BRII-835-002) was an open-label, phase 2 study (NCT05970289), which was initiated in August 2023 at 26 sites across 7 countries/ regions in Asia-Pacific region and was completed on 23 Jan 2026. Fig. S2 provides a schematic of the trial design.

This study was designed to evaluate the efficacy and safety of elebsiran and PEG-IFNα combination therapy in participants with chronic HBV infection without cirrhosis receiving NA as background therapy. It consists of 2 parts:

A randomized Part I (Cohorts 1-3): Eligible participants were randomized at 1:1:1 to Cohort 1 (48 weekly doses of PEG-IFNα 180 µg administered subcutaneously (SC) every week (QW) starting on Day 1), Cohort 2 (48 doses of PEG-IFNα 180 µg administered SC QW and 13 doses of elebsiran 200 mg administered SC every 4 weeks (Q4W), both starting on Day 1), and Cohort 3 (48 doses of PEG-IFNα 180 µg administered SC QW and 13 doses of elebsiran 100 mg administered SC Q4W, both starting on Day 1), using an Interactive Response Technology (IRT) system. Participants were stratified at randomization by their HBsAg level at screening (> 100 to ≤ 500 IU/mL, > 500 to ≤ 1,500 IU/mL, and > 1,500 to ≤ 3,000 IU/mL).

A single arm Part II (Cohort 4): Eligible participants who had completed 9 doses of elebsiran and BRII-179 approximately 1.5 years ago on average in BRII-179-835-001 study were enrolled to Cohort 4 and received 48 doses of PEG-IFNα 180 µg administered SC QW and 13 doses of elebsiran 100 mg administered SC Q4W, both starting on Day 1.

After completing the 48-week treatment period, all participants from the 4 cohorts continued NA treatment and were followed for an additional 24 weeks until Week 72. At Week 72, participants were assessed for eligibility to discontinue NA therapy. Those who met all the predefined NA discontinuation criteria (HBsAg < LLOQ; HBV DNA < LLOQ; negative HBeAg; and ALT ≤ 2

× ULN) for at least 2 consecutive visits at Week 72 stopped NA and entered a 24-week NDMP. Participants who did not meet the criteria for NA discontinuation continued NA therapy and completed the study at Week 72.

Inclusion Criteria

Participants eligible to be included in the study must meet all the following criteria.

Cohorts 1-3 (BRII-179 naïve):

1. A signed and dated written Informed Consent Form (ICF) prior to study participation.
2. Age 18 (or age of legal consent, whichever is older) to 60 years, both inclusive.
3. Body mass index (BMI) ≥ 18 kg/m^2 and ≤ 32 kg/m^2.
4. Chronic HBV infection as defined by a positive serum HBsAg for ≥ 6 months prior to screening.
5. Must be on NRTI therapy consisting of either entecavir, tenofovir disoproxil, tenofovir amibufenamide, or tenofovir alafenamide and with HBV-DNA < LLOQ for at least 6 months before screening.
6. HBsAg > 100 IU/mL and ≤ 3,000 IU/mL at screening.
7. HBV DNA < LLOQ at screening.
8. Serum ALT and AST ≤ ULN at screening.
9. Female participants must have a negative pregnancy test or confirmation of postmenopausal status. Post-menopausal status is defined as 12 months with no menses without alternative medical cause. Women of child-bearing potential (WOCBP) must have a negative serum pregnancy test at screening and a negative urine pregnancy test on

Day 1, cannot be breast feeding at screening and throughout the study, and must be willing to use highly effective methods of contraception 14 days before study drug administration through 48 weeks after last study drug administration. Female participants must also agree to refrain from egg donation and in vitro fertilization from the time of study intervention administration through 48 weeks after the last dose of study intervention.

10. Male participants with female partners of child-bearing potential must agree to meet one of the following contraception requirements from the time of study drug administration until 48 weeks after last study drug administration: vasectomy with documentation of azoospermia, or male condom use plus partner use of 1 of the contraceptive options listed for contraception for WOCBP. Male participants must also agree not to donate sperm for the 48 weeks following last study drug administration.
11. Agrees to not donate blood during the duration of the study.
12. Willing and able to comply with the study procedures and requirements.

Cohort 4 (BRII-179 treated):

13. A signed and dated written Informed Consent Form (ICF) prior to study participation.
14. Age ≥ 18 years (or age of legal consent, whichever is older).
15. Body mass index (BMI) ≥ 18 kg/m^2 and ≤ 32 kg/m^2.
16. Completed BRII-179-835-001 study and completed BRII-179 treatment.
17. Must be on NRTI therapy consisting of either entecavir, tenofovir disoproxil, tenofovir amibufenamide, or tenofovir alafenamide and with HBV-DNA < LLOQ for at least 6 months before screening.

18. HBsAg > 0.05 IU/mL at screening.

19. HBV DNA < LLOQ at screening.

20. Serum ALT and AST ≤ ULN at screening.

21. Female participants must have a negative pregnancy test or confirmation of postmenopausal status. Post-menopausal status is defined as 12 months with no menses without alternative medical cause. Women of child-bearing potential (WOCBP) must have a negative serum pregnancy test at screening and a negative urine pregnancy test on Day 1, cannot be breast feeding at screening and throughout the study, and must be willing to use highly effective methods of contraception 14 days before study drug administration through 48 weeks after last study drug administration. Female participants must also agree to refrain from egg donation and in vitro fertilization from the time of study intervention administration through 48 weeks after the last dose of study intervention.

22. Male participants with female partners of child-bearing potential must agree to meet 1 of the following contraception requirements from the time of study drug administration until 48 weeks after last study drug administration: vasectomy with documentation of azoospermia, or male condom use plus partner use of 1 of the contraceptive options listed for contraception for WOCBP. Male participants must also agree not to donate sperm for the 48 weeks following last study drug administration.

23. Agrees to not donate blood during the duration of the study.

24. Willing and able to comply with the study procedures and requirements.

Cohort 4 only enrolls participants from BRII-179-835-001 study in APAC region excluding mainland China.

Exclusion Criteria

Participants are excluded from the study if there is evidence of any of the following at screening or prior to randomization, as appropriate:

1. Current or prior treatment with:
    a) Small interfering RNAs or antisense oligonucleotides within 12 months prior to screening
    b) Interferon-based therapy within 12 months prior to screening
    c) Telbivudine within 6 months prior to screening
    d) Myelotoxic drugs such as systemic cytostatic agents (i.e., cancer chemotherapeutic medications) within 30 days before study drug administration at the Day 1 visit
    e) Systemic immunosuppressant (including but not limited to corticosteroids at a prednisolone equivalent dose > 40 mg/day) within 30 days before study drug administration at the Day 1 visit
    f) Granulocyte colony-stimulating factor (G-CSF), granulocyte-macrophage colony-stimulating factor (GM-CSF), recombinant human interleukin-11 (oprelvekin), or erythropoietin (EPO) within 6 months prior to screening
    g) Any HBV therapeutic vaccines prior to screening (**applicable to Cohorts 1-3 only**).

2. Any clinically significant chronic medical condition other than chronic HBV infection that, in the opinion of the investigator makes the participant unsuitable for participation in the study.
3. Any clinically significant acute condition such as fever (>38°C) or acute respiratory illness within 7 days of first study drug administration.
4. Significant liver fibrosis or cirrhosis as defined by having a FibroScan result of > 9 kPa at screening.
5. History or evidence of drug or alcohol abuse as assessed by the Investigator, within the 12 months before screening.
6. History of clinically significant chronic liver disease from any cause other than chronic HBV infection.
7. History of hepatic decompensation, including but not limited to ascites, hepatic encephalopathy and/or esophageal or gastric varices.
8. Diagnosed or suspected hepatocellular carcinoma. Participant with screening alpha-fetoprotein (AFP) concentration ≥200 nanogram per milliliter (ng/mL) should be excluded. If the screening AFP concentration is ≥50 ng/mL and <200 ng/mL, the absence of liver mass must be documented by imaging at screening.
9. History of thyroid disease not adequately controlled on prescribed medications, or clinically significant signs of thyroid dysfunction as determined by the Investigator at screening.
10. Known history of immunological function impairment, including but not limited to:

a) Autoimmune disease (e.g., multiple sclerosis, type 1 diabetes mellitus , myasthenia gravis, Crohn's disease and other inflammatory bowel diseases, celiac disease, systemic lupus erythematosus, scleroderma, including diffuse systemic form and limited cutaneous form of systemic sclerosis or CREST syndrome, systemic sclerosis, dermatomyositis polymyositis, rheumatoid arthritis, juvenile idiopathic arthritis, autoimmune thyroiditis including Hashimoto thyroiditis, Grave's or Basedow's disease, immune thrombocytopenic purpura, autoimmune hemolytic anemia, autoimmune hepatitis, psoriasis, vitiligo, vasculitis, Guillain Barré syndrome, Addison's disease, Bell's palsy, and alopecia areata); OR

b) Primary immunodeficiency disorders (e.g., common variable immune deficiency, defective phagocytic cell function and neutropenia syndromes, and complement deficiency); OR

c) Secondary immunodeficiency disorders (e.g., resulting from acquired immunodeficiency syndrome caused by human immunodeficiency virus (HIV) infection, solid organ transplant, and splenectomy).

11. Current or past history of infection with human immunodeficiency virus (HIV), hepatitis C virus (HCV) or hepatitis Delta virus (HDV).

12. Renal impairment with estimated glomerular filtration rate (eGFR) $\leq$ 60 mL/min/1.73m^2 at screening.

13. Any laboratory test abnormality that is considered as clinically significant or may contradict treatment with PEG-IFNα, including but not limited to below laboratory abnormalities:

a) Hemoglobin < 100 g/L

b) Absolute neutrophil count < 1,500/mm^3 (< 1,000/ mm^3 for African)

c) Platelet count < 90,000/ mm^3

14. Uncontrolled diabetes mellitus as determined by the Investigator.

15. Uncontrolled hypertension as determined by the Investigator.

16. Diagnosis of advanced stage heart failure (New York Heart Association Class III or Class IV or unstable angina).

17. Previous or current psychiatric condition that precludes compliance with the protocol. Specifically excluded are persons with severe psychiatric disorders as determined by the Investigator, including, but not limited to schizophrenia, psychosis, severe depression, bipolar illness, severe anxiety, ongoing risk for suicide, or history of suicide attempt or gesture within the past 5 years.

18. The participant has any history of cancer requiring locoregional or systemic therapy within 5 years of randomization or has current disease. Participants with completely surgically resected tumors with no evidence of disease for more than 3 years, or a history of low risk basal cell carcinoma will be accepted (low risk being defined as 1) location on the trunk of the body, arms, legs, cheeks, forehead, temples, scalp, neck, or chin, 2) < 2 cm in size, 3) nodular or superficial, 4) primary cancer that has not come back after treatment, 5) edge of the cancerous area is clear and smooth, and 6) not located in or around nerves).

19. History of intolerance to SC injection.

20. History of intolerance or hypersensitivity to interferon/pegylated interferon.

21. Known hypersensitivity or contraindication to an siRNA/GalNAc or their excipients.

22. Any skin abnormality or tattoo that would limit post treatment injection site assessment.
23. Participated in another clinical trial with an investigational drug or a biologic within 30 days or within 5 drug half-lives (whichever is longer) before study drug administration at the Day 1 visit. Participants must agree not to take part in any other study at any time during their participation in this study, inclusive of the follow-up period and NRTI Discontinuation Monitoring Period.
24. Unwilling, or unable in the opinion of the Investigator, to comply with study requirements.

Supplementary Tables

Table S1. Summary of HBsAg Seroreversion Rates in Participants Discontinued NA

	Seroreversion Rate n/N (%)	95% Confidence Interval
Participants having HBsAg Seroreversion during NA discontinuation period	11/41 (26.8)	(14.2, 42.9)
NA consolidation period < 24 weeks	5/20 (25.0)	(8.7, 49.1)
NA consolidation period ≥ 24 weeks	6/21 (28.6)	(11.3, 52.2)
Participants having HBsAg Seroreversion within 24 weeks of NA discontinuation period	8/41 (19.5)	(8.8, 34.9)
NA consolidation period < 24 weeks	3/20 (15.0)	(3.2, 37.9)
NA consolidation period ≥ 24 weeks	5/21 (23.8)	(8.2, 47.2)

This table summarized HBsAg seroreversion in participants discontinued NA and had HBsAg < LLOQ on the first day of NA Discontinuation. Analysis visit window for the 24-week NA consolidation period was defined as within 2 weeks of the target visit. LLOQ, lower limit of quantification; n, number of participants with HBsAg seroreversion; N, number of overall participants; NA, nucleos(t)ide analogue; PEG-IFNα, pegylated interferon alpha.

Table S2. Summary of Maximum HBsAg Level after Seroreversion

	0.05 - 1 IU/mL n/N (%)	1 - 10 IU/mL n/N (%)	10 - 100 IU/mL n/N (%)
PEG-IFNα based combination	8/18 (44.4)	7/18 (38.9)	3/18 (16.7)
PEG-IFNα monotherapy	2/6 (33.3)	1/6 (16.7)	3/6 (50.0)
HBsAg seroreversion during NA consolidation	4/13 (30.8)	5/13 (38.5)	4*/13 (30.8)
HBsAg seroreversion after NA consolidation	6/11 (54.5)	3/11 (27.3)	2/11 (18.2)
NA consolidation period <24 weeks	3/5 (60.0)	1/5 (20.0)	1/5 (20.0)
NA consolidation period ≥24 weeks	3/6 (50.0)	2/6 (33.3)	1/6 (16.7)
Total	10/24 (41.7)	8/24 (33.3)	6/24 (25.0)

NA consolidation period: participants did not discontinue NA = duration from end of treatment to end of study; participants discontinued NA = duration from end of treatment to NA discontinuation monitoring period Day 1. Analysis visit window for the 24-week NA consolidation period was defined as within 2 weeks of the target visit.
Two participants who developed HBsAg seroreversion on the first day of NA discontinuation were considered not to have truly fulfilled the criteria for NA discontinuation and were accordingly classified as having HBsAg seroreversion during the NA consolidation phase.
n, number of participants with HBsAg seroreversion; N, number of overall participants; NA, nucleos(t)ide analogue; PEG-IFNα, pegylated interferon alpha.

Table S3. Cumulative Incidence and Log-rank Test for Time to HBsAg Seroreversion (HBeAg at Baseline Positive versus Negative)

	HBeAg Positive (N=4) n (%)	HBeAg Negative (N=51) n (%)
HBsAg Seroreversion Status, n (%)		
Seroreversion	3 (75.0)	21 (41.2)
Censor	1 (25.0)	30 (58.8)
Cumulative HBsAg Seroreversion-free Rate (%), 95% CI		
Week 12 (95% CI)	100.0 [100.0, 100.0]	92.2 [80.4, 97.0]
Week 24 (95% CI)	75.0 [12.8, 96.1]	80.4 [66.6, 88.9]
Week 36 (95% CI)	37.5 [1.1, 80.8]	70.3 [55.7, 80.9]
Week 48 (95% CI)	0.0 [-, -]	66.3 [51.5, 77.5]
Week 60 (95% CI)	0.0 [-, -]	57.1 [40.7, 70.4]
Time to HBsAg Seroreversion (Days)		
Median (95% CI)	245.0 [114.0, -]	505.0 [338.0, -]
Log-rank Test p-value	0.0722	

CI, confidence interval; N, number of participants.

Table S4. Cumulative Incidence and Log-rank Test for Time to HBsAg Seroreversion (PEG-IFNα Based Combinations versus PEG-IFNα Monotherapy)

	PEG-IFNα based combinations (N=42) n (%)	PEG-IFNα monotherapy (N=13) n (%)
HBsAg Seroreversion Status, n (%)		
Seroreversion	18 (42.9)	6 (46.2)
Censor	24 (57.1)	7 (53.8)
Cumulative HBsAg Seroreversion-free Rate (%), 95% CI		
Week 12 (95% CI)	97.6 [84.3, 99.7]	76.9 [44.2, 91.9]
Week 24 (95% CI)	83.3 [68.2, 91.7]	69.2 [37.3, 87.2]
Week 36 (95% CI)	68.2 [51.5, 80.2]	69.2 [37.3, 87.2]
Week 48 (95% CI)	60.6 [43.9, 73.7]	69.2 [37.3, 87.2]
Week 60 (95% CI)	49.6 [30.3, 66.2]	61.5 [30.8, 81.8]
Time to HBsAg Seroreversion (Days)		
Median (95% CI)	383.0 [254.0, -]	505.0 [54.0, -]
Log-rank Test p-value	0.8324	

CI, confidence interval; PEG-IFNα, pegylated interferon alpha.

Table S5. Cumulative Incidence and Log-rank Test for Time to HBsAg Seroreversion (With BRII-179 Exposure vs. Without BRII-179 Exposure)

	With BRII-179 exposure (N=30) n (%)	Without BRII-179 exposure (N=25) n (%)
HBsAg Rebound Status, n (%)		
Rebound	12 (40.0)	12 (48.0)
Censor	18 (60.0)	13 (52.0)
Cumulative HBsAg Rebound-free Rate (%), 95% CI		
Week 12 (95% CI)	96.7 [78.6, 99.5]	88.0 [67.3, 96.0]
Week 24 (95% CI)	80.0 [60.8, 90.5]	80.0 [58.4, 91.1]
Week 36 (95% CI)	66.1 [46.1, 80.1]	71.6 [49.4, 85.3]
Week 48 (95% CI)	62.6 [42.7, 77.3]	63.2 [41.1, 78.9]
Week 60 (95% CI)	56.3 [34.9, 73.1]	51.0 [27.9, 70.1]
Time to HBsAg Rebound (Days)		
Median (95% CI)	- [245.0, -]	505.0 [185.0, -]
Log-rank Test p-value	0.7351	

CI, confidence interval

Note: participants in Part II of ENSURE who have been treated with BRII-179 in the BRII-179-835-001 study was included in "with BRII-179 exposure".

Table S6. Cumulative Incidence and Log-rank Test for Time to HBsAg Seroreversion (PEG-IFNα Based Treatment Duration after HBsAg Loss ≥12 versus<12 Weeks)

	**≥12 weeks (N=34) n (%)**	**<12 weeks (N=21) n (%)**
HBsAg Seroreversion Status, n (%)		
Seroreversion	11 (32.4)	13 (61.9)
Censor	23 (67.6)	8 (38.1)
Cumulative HBsAg Seroreversion-free Rate (%), 95% CI		
Week 12 (95% CI)	100.0 [100.0, 100.0]	81.0 [56.9, 92.4]
Week 24 (95% CI)	85.3 [68.2, 93.6]	71.4 [47.2, 86.0]
Week 36 (95% CI)	72.7 [54.0, 84.8]	61.9 [38.1, 78.8]
Week 48 (95% CI)	69.5 [50.7, 82.3]	52.4 [29.7, 70.9]
Week 60 (95% CI)	66.2 [47.2, 79.7]	41.9 [20.9, 61.7]
Time to HBsAg Seroreversion (Days)		
Median (95% CI)	- [338.0, -]	362.0 [153.0, -]
Log-rank Test p-value	0.1203	

CI, confidence interval; PEG-IFNα, pegylated interferon alpha.

Table S7. Cumulative Incidence and Log-rank Test for Time to HBsAg Seroreversion (NA Consolidation Duration ≥24 versus<24 Weeks) in Participants Discontinued NA and Had HBsAg < LLOQ on the First Day of NA Discontinuation

	≥24 weeks (N=21) n (%)	<24 weeks (N=20) n (%)
HBsAg Seroreversion Status, n (%)		
Seroreversion	6 (28.6)	5 (25.0)
Censor	15 (71.4)	15 (75.0)
Cumulative HBsAg Seroreversion-free Rate (%), 95% CI		
Week 12 (95% CI)	100.0 [100.0, 100.0]	100.0 [100.0, 100.0]
Week 24 (95% CI)	100.0 [100.0, 100.0]	95.0 [69.5, 99.3]
Week 36 (95% CI)	95.2 [70.7, 99.3]	84.4 [59.1, 94.7]
Week 48 (95% CI)	81.0 [56.9, 92.4]	84.4 [59.1, 94.7]
Week 60 (95% CI)	54.0 [9.5, 85.1]	73.9 [48.2, 88.2]
Time to HBsAg Seroreversion (Days)		
Median (95% CI)	505.0 [362.0, -]	- [383.0, -]
Log-rank Test p-value	0.7580	

Analysis visit window for the 24-week NA consolidation period was defined as within 2 weeks of the target visit.
CI, confidence interval; NA, nucleos(t)ide analogue.

Table S8. Cumulative Incidence and Log-rank Test for Time to HBsAg Seroreversion (NA Consolidation Duration ≥24 versus<24 Weeks) in Participants Discontinued NA and Had HBsAg < LLOQ on the First Day of NA Discontinuation – Sensitivity Analysis in the Common 48-Week Observation Window after EOT

	≥24 weeks (N=21) n (%)	<24 weeks (N=20) n (%)
HBsAg Seroreversion Status, n (%)		
Seroreversion	5 (23.8)	4 (20.0)
Censor	16 (76.2)	16 (80.0)
Cumulative HBsAg Seroreversion-free Rate (%), 95% CI		
Week 12 (95% CI)	100.0 [100.0, 100.0]	100.0 [100.0, 100.0]
Week 24 (95% CI)	100.0 [100.0, 100.0]	95.0 [69.5, 99.3]
Week 36 (95% CI)	95.2 [70.7, 99.3]	84.1 [58.3, 94.6]
Week 48 (95% CI)	81.0 [56.9, 92.4]	84.1 [58.3, 94.6]
Time to HBsAg Seroreversion (Days)		
Median (95% CI)	362.0 [-, -]	- [338.0, -]
Log-rank Test p-value	0.6460	

Analysis visit window for the 24-week NA consolidation period was defined as within 2 weeks of the target visit.
CI, confidence interval; NA, nucleos(t)ide analogue.
Sensitivity analysis was conducted using a common 48-week observation window after EOT. Participants without documented HBsAg seroreversion were censored at 48 weeks after EOT or at their last available post-EOT HBsAg assessment if follow-up ended earlier.

Table S9. Cumulative Incidence and Log-rank Test for Time to HBsAg Seroreversion (Anti-HBs Level at EOT >100 versus ≤100 IU/L)

	**Anti-HBs at EOT >100 IU/L (N=26) n (%)**	**Anti-HBs at EOT ≤100 IU/L (N=29) n (%)**
HBsAg Seroreversion Status, n (%)		
Seroreversion	6 (23.1)	18 (62.1)
Censor	20 (76.9)	11 (37.9)
Cumulative HBsAg Seroreversion-free Rate (%), 95% CI		
Week 12 (95% CI)	100.0 [100.0, 100.0]	86.2 [67.3, 94.6]
Week 24 (95% CI)	92.3 [72.6, 98.0]	69.0 [48.8, 82.5]
Week 36 (95% CI)	79.7 [57.9, 91.0]	58.6 [38.8, 74.0]
Week 48 (95% CI)	75.5 [53.4, 88.2]	51.7 [32.5, 67.9]
Week 60 (95% CI)	75.5 [53.4, 88.2]	40.0 [22.1, 57.4]
Time to HBsAg Seroreversion (Days)		
Median (95% CI)	- [-, -]	338.0 [153.0, -]
Log-rank Test p-value	0.0169	

Anti-HBs, hepatitis B surface antibody; CI, confidence interval; EOT, end of treatment; N, number of participants.

Table S10. Association between Anti-HBs Status and HBsAg Seroreversion Within 48 Weeks after EOT Estimated Using a Logistic Mixed-Effects Model

Variable	Estimate (log-odds)	OR (95% CI)	P-value
Anti-HBs < 10 IU/L	5.30	199.76 (2.66->999)	0.016
Follow-up week (per week)	0.10	1.11 (1.02-1.20)	0.020

ORs and 95% CIs were estimated using a logistic mixed-effects model with a participant-specific random intercept to account for repeated longitudinal measurements. Anti-HBs status and follow-up time were included as fixed effects.

Anti-HBs, hepatitis B surface antibody; CI, confidence interval; OR, odds ratio.

Supplementary Figures

Figure S1. Trial design of BRII-179-002

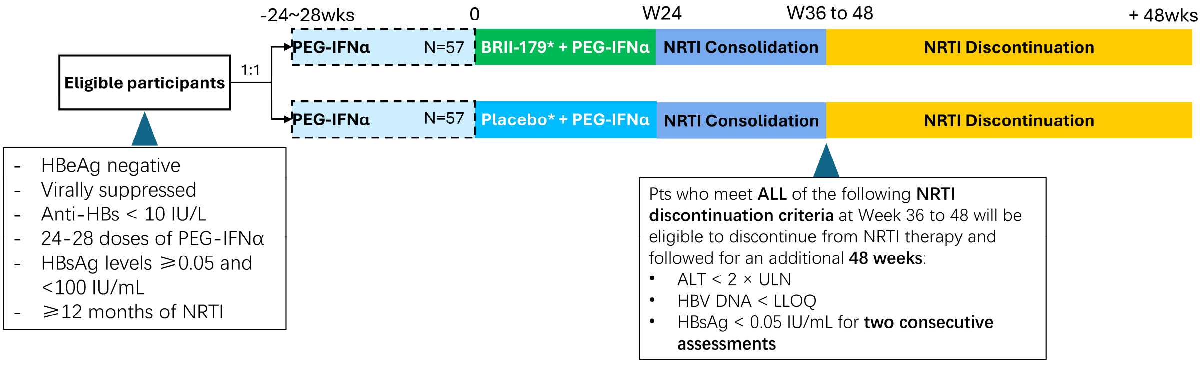

*BRII-179 40 µg or placebo were given every 3 weeks intramuscularly for 7 doses in addition to continued PEG-IFNα-2b 180 µg every week subcutaneously for 24 weeks
ALT, alanine aminotransferase; anti-HBs, hepatitis B surface antibody; LLOQ, lower limit of quantification; N, number of participants; NA, nucleos(t)ide analogue; PEG-IFNα, pegylated interferon alfa; ULN, upper limit of normal; W, Week; wks, weeks.

Figure S2. Trial design of ENSURE

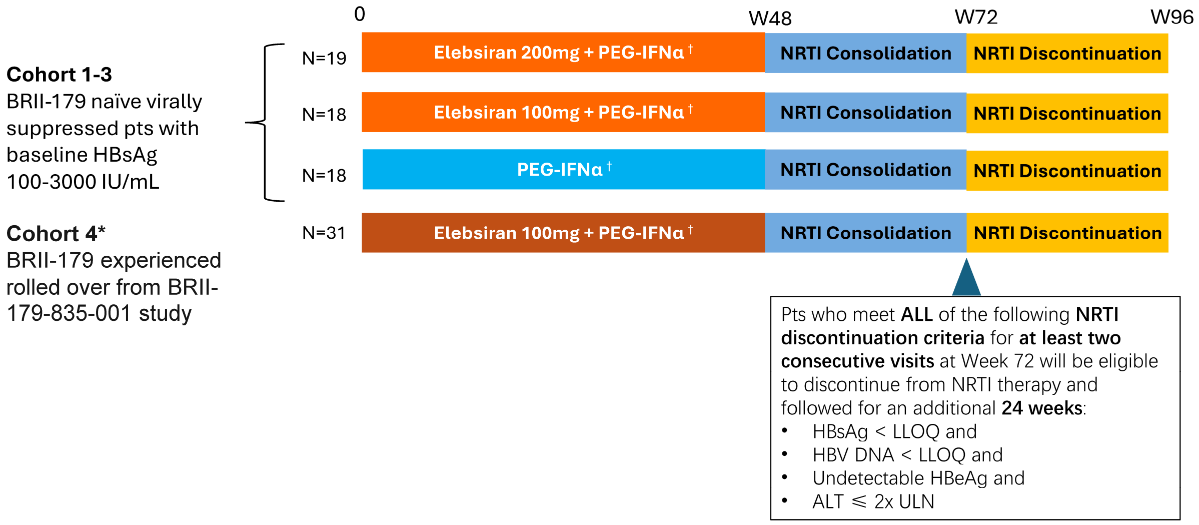

*Participants received elebsiran 100 mg Q4W SC x 9 doses and BRII-179 40 µg Q4W IM x 9 doses, completed treatment ≥ 1 year
† Elebsiran was given Q4W SC for 13 doses; PEG-IFNα-2a 180µg was given QW SC for 48 weeks
ALT, alanine aminotransferase; LLOQ, lower limit of quantification; N, number of participants; NA, nucleos(t)ide analogue; PEG-IFNα, pegylated interferon alfa; Pts, participants; QW, every week; Q4W, every 4 weeks; QW, SC, subcutaneously; ULN, upper limit of normal; W, Week.

Figure S3. Participant disposition in BRII-179-002 and ENSURE

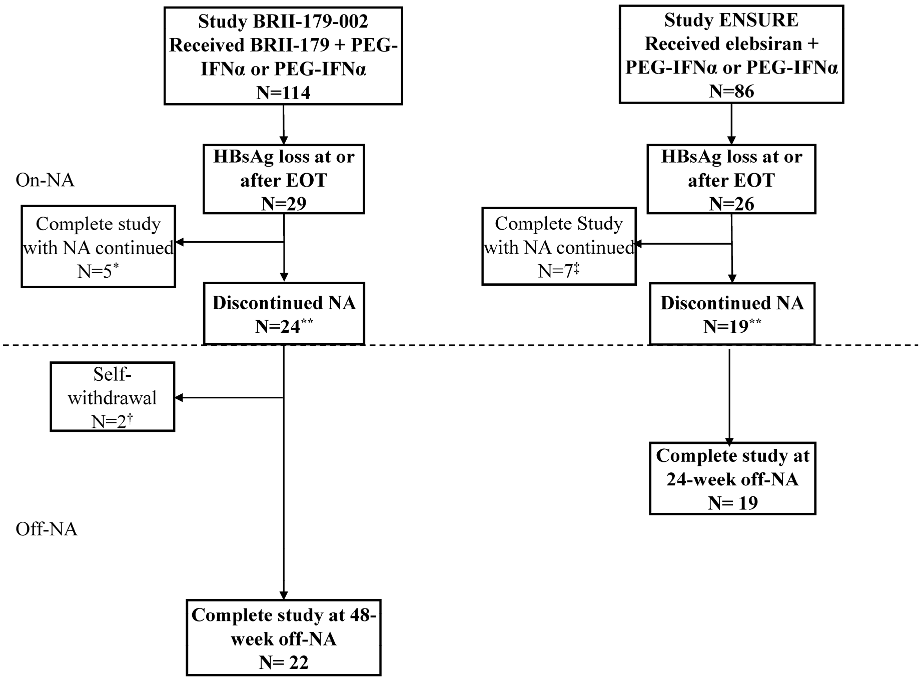

In BRII-179-002, 27 participants had HBsAg < LLOQ at EOT, another 2 participants achieved HBsAg loss at 4 weeks post EOT; In ENSURE, 25 participants had HBsAg < LLOQ at EOT, one participant achieved HBsAg loss at 8 weeks post EOT.

*, all had HBsAg seroreversion and complete study at 24-week post-EOT; ** including one had HBsAg seroreversioned on the first day of NA discontinuation; †, including the one had HBsAg seroreversioned on the first day of NA discontinuation, the other one remained HBsAg loss; ‡, one remained HBeAg positive without HBsAg seroreversion, others had HBsAg seroreversion, they complete study at 24-week post-EOT; #, the two participants had HBsAg seroreversion on the first day of NA discontinuation were excluded from this population. EOT, end of treatment; N, number of participants; NA, nucleos(t)ide analogue; PEG-IFNα, pegylated interferon alpha; wks, weeks.

Figure S4. Participants with HBsAg seroreversion with HBV DNA ≥ 100 IU/mL

A

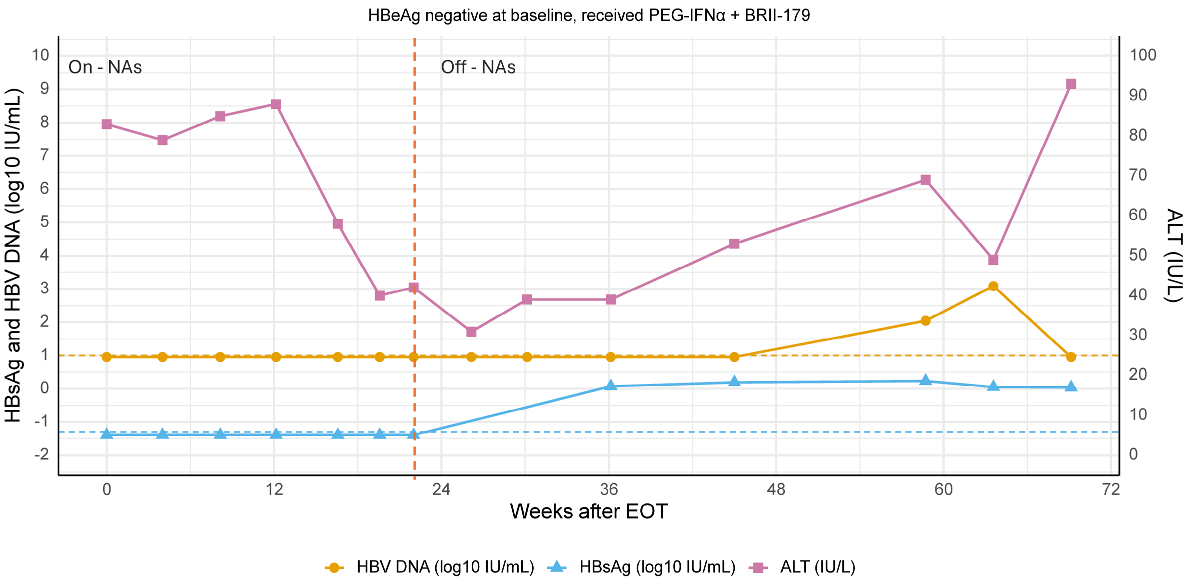

B

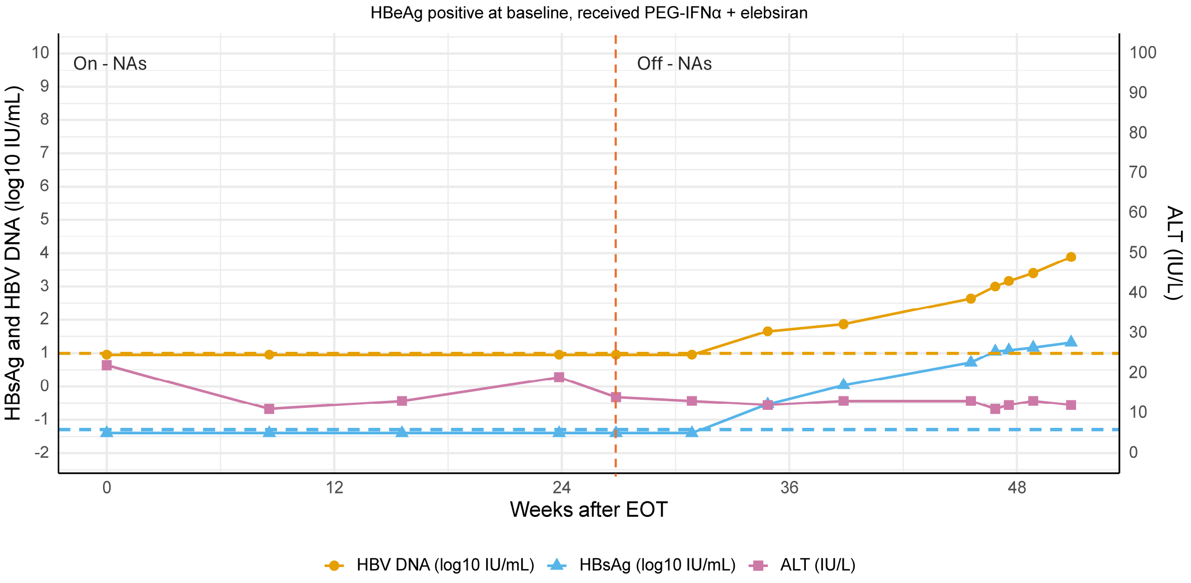

(A) Participant 23 (Fig. 3), HBeAg negative at baseline, received PEG-IFNα plus BRII-179 and underwent a 20-week NA consolidation before NA discontinuation. (B) Participant 16 (Fig. 3), HBeAg positive at baseline, received PEG-IFNα plus elebsiran and underwent a 24-week NA consolidation before NA discontinuation. The participant had maximum seroreversion HBsAg level of 20.7 IU/mL, HBV DNA level of 7710 IU/mL. ALT, alanine aminotransferase; anti-HBs, hepatitis B surface antibody; EOT, end of treatment; PEG-IFNα, pegylated interferon alpha; ULN, upper limit of normal.

Figure S5. Kaplan-Meier plots of time to HBsAg seroreversion

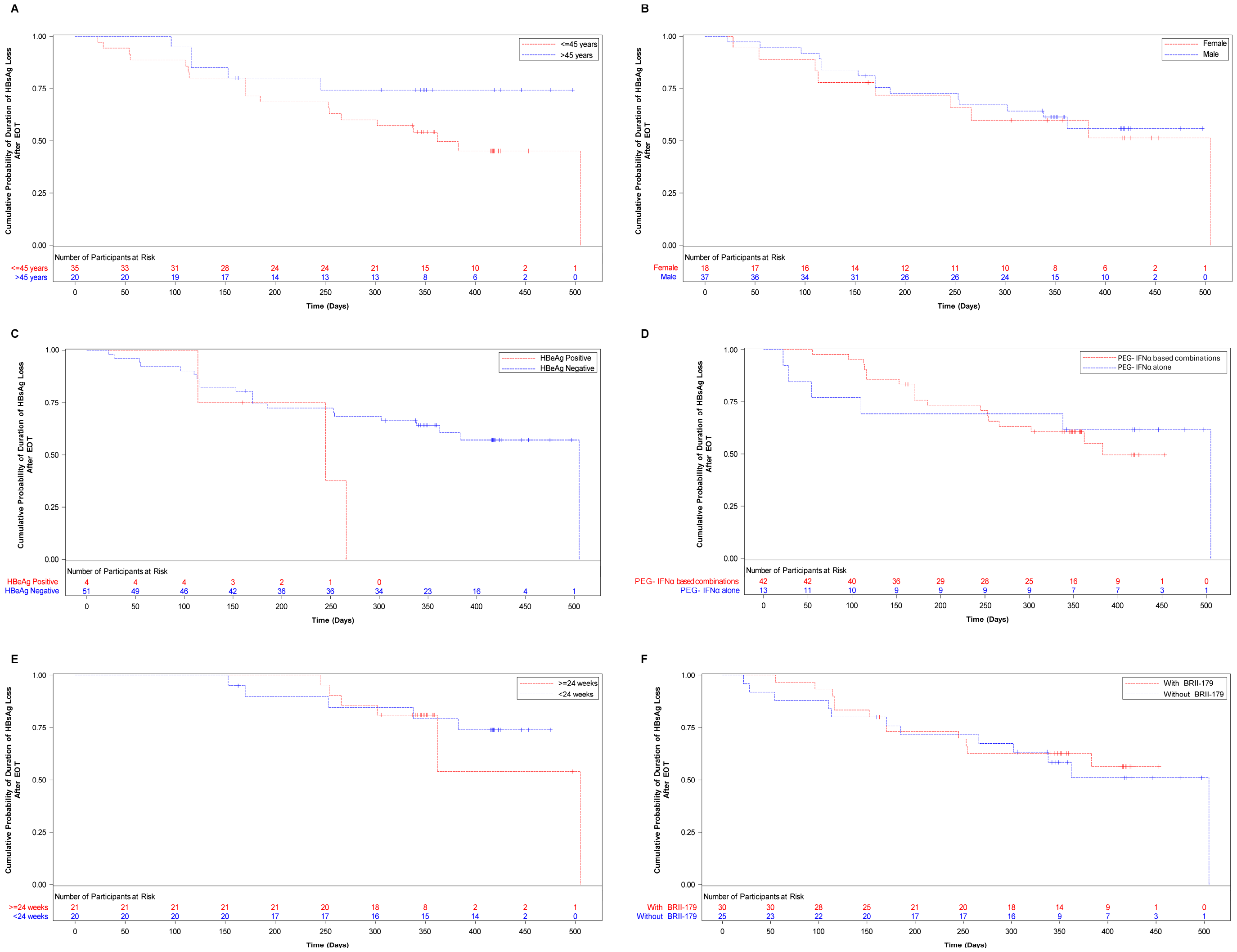

(A) age ≤ 45 versus >45 years at baseline of the parent trial. (B) sex: female versus male. (C) HBeAg status at baseline: positive versus negative. (D) treatment: PEG-IFNα based combination therapies versus PEG-IFNα monotherapy. (E) NA consolidation duration in participants who discontinued NA: ≥24 versus<24 weeks. (F) With BRII-179 versus without BRII-179. Anti-HBs, hepatitis B surface antibody; EOT, end of treatment.

Figure S6. Model-based predicted probability of HBsAg seroreversion according to anti-HBs status over follow-up

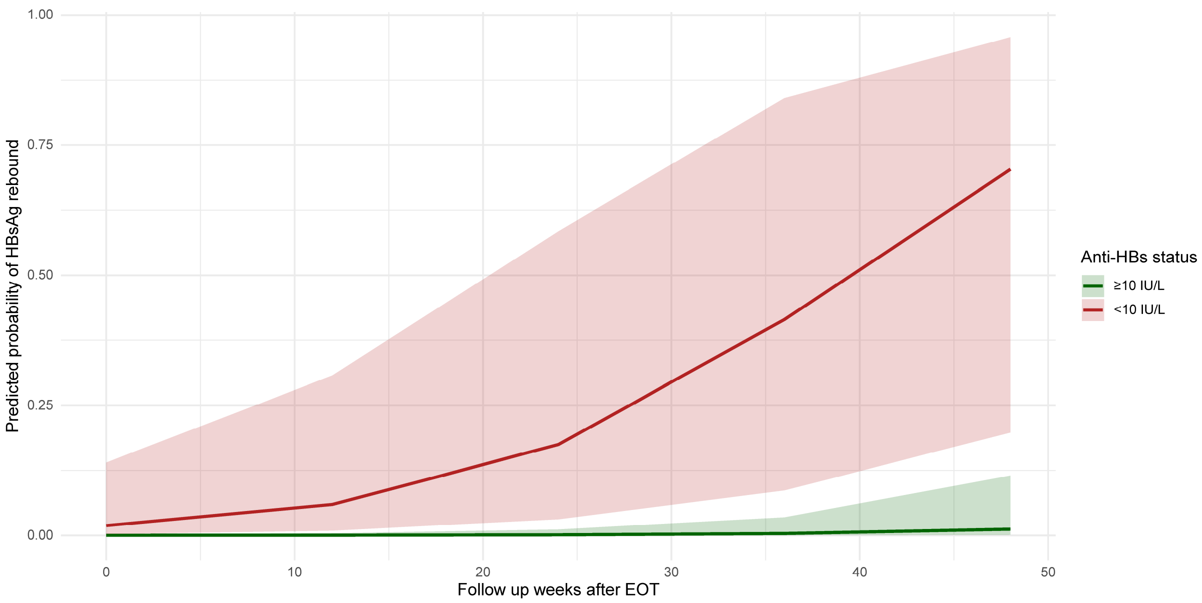

Anti-HBs, hepatitis B surface antibody; EOT, end of treatment
