# Supplementary material for: Profile and Predictors of Hepatitis B Surface Antigen Seroreversion Following PEG-IFNα with or Without Therapeutic Vaccine BRII-179 or siRNA Elebsiran: A Post Hoc Pooled Analysis of Two Randomized Controlled Trials in Patients with Chronic Hepatitis B Virus Infection
Source: Vaccines (Basel). 2026 Oct 1;14(10):868. doi: 10.3390/vaccines14100868 (PMC13709818; doi:10.3390/vaccines14100868)
Supplement: Supplementary file 1 [file vaccines-14-00868-s001.zip › File_S1_BRII-179-002_Clinical_Study_Protocol_and_Amendments.pdf]

Title

Protocol Title: An Adaptive Multicenter, Randomized, Double-Blind, Placebo-Controlled, Parallel-Group Study to Investigate the Efficacy and Safety of BRII-179 (VBI-2601) for the Treatment of Chronic Hepatitis B Virus (HBV) Infection

Protocol Number: BRII-179-002

Compound: BRII-179 (VBI-2601)

Study Phase: Phase 2/ Phase 3

Short Title: Short Title: Study to Investigate Efficacy and Safety of BRII-179 (VBI-2601) for the Treatment of Chronic HBV Infection

Sponsor: Brii Biosciences Limited

Co-Sponsor/ Registration Agent: Brii Biosciences (Beijing) Ltd.

Legal Registered Address: Room 301, 3/F, Building 7,
No. 1 Yongtaizhuang North Road，
Tiandilinfeng（Dongsheng Area）
Haidian District, Beijing 100192,
China

Protocol Version: Version 1.0

Approval Date: 30 April 2021

Sponsor Signatory

[redacted]

Medical Monitor Name and Contact Information will be provided separately.

INVESTIGATOR AGREEMENT PAGE

For protocol number BRII-179-002

I confirm agreement to conduct the study in compliance with the protocol, *International Conference on Harmonisation Good Clinical Practices (ICH GCP)* as well as applicable local regulatory and ethical requirements.

I acknowledge that I am responsible for overall study conduct. I agree to personally conduct or supervise the described clinical study.

I agree to ensure that all associates, colleagues and employees assisting in the conduct of the study are informed about their obligations. Mechanisms are in place to ensure that site staff receive the appropriate information throughout study.

Investigator Name: ____

____ ____________________________

Investigator Signature **Date** (dd/mm/yyyy)

Table of Contents

1. PROTOCOL SUMMARY 8
   1.1. Synopsis 8
   1.2. Phase 2 (N=106) 8
   1.3. Phase 3 (N=480) 9
   1.4. Schema 12
   1.5. Schedule of Activities (SoA) 13
      1.5.1. BRII-179 (VBI-2601) 32-week Dosing Period and 16-week Follow-up Period 13
      1.5.2. NrtI Discontinuation Monitoring Period 15

2. INTRODUCTION 16
   2.1. Background 16
   2.2. Study Rationale 17
   2.3. Benefit/Risk Assessment 18

3. OBJECTIVES AND ENDPOINTS 19
   3.1. Objectives 19
   3.2. Endpoints 19
      3.2.1. Primary Endpoints 19
      3.2.2. Secondary Endpoints 19

4. STUDY DESIGN 21
   4.1. Overall Design 21
      4.1.1. Independent DSMB 21
      4.1.2. NrtI Discontinuation 21
      4.1.3. PEG-IFN-α Therapy During the Dosing Period 22
   4.2. Scientific Rationale for Study Design 24
   4.3. Justification for Dose 25
   4.4. End of Study Definition 25

5. STUDY POPULATION 27
   5.1. Inclusion Criteria 27
   5.2. Exclusion Criteria 28
   5.3. Lifestyle Considerations 30
   5.4. Screen Failures 30

6. STUDY INTERVENTION 31
   6.1. Study Intervention(s) Administered 31
      6.1.1. Formulations 31
      6.1.2. Dosage and Administration of BRII-179 and Placebo 31
   6.2. Preparation/Handling/Storage/Accountability 32
   6.3. Randomization and Blinding 32
   6.4. Concomitant Therapy 33
      6.4.1. Medication Adherence with NrtI and PEG-IFN-α 34
      6.4.2. Permitted Medications 34
      6.4.3. Prohibited Medications 35
   6.5. Safety Follow-up and Management of Reactions to Study Drug 35

6.6. Treatment of Study Intervention Overdose 36
6.7. Intervention after the End of the Study 36

7. DISCONTINUATION OF STUDY INTERVENTION AND SUBJECT DISCONTINUATION/WITHDRAWAL 37
7.1. Discontinuation of Study Intervention 37
7.1.1. Criteria for Discontinuation of Study Intervention 37
7.2. Subject Discontinuation/Withdrawal from the Study 37
7.2.1. Lost to Follow up 38

8. STUDY ASSESSMENTS AND PROCEDURES 39
8.1. Schedule of Assessments 39
8.1.1. Screening 39
8.1.2. Randomization (Day 1) 39
8.1.3. Dosing Period 39
8.1.4. Follow-up Period 40
8.1.5. NrtI Discontinuation Monitoring Period 40
8.1.6. Unscheduled Visits 40
8.1.7. Early Termination Visit 40
8.2. Detailed Description of Study Procedures 41
8.2.1. Medical History 41
8.2.2. Physical Examinations 41
8.2.3. Fibroscan 41
8.2.4. Alcohol Intake Assessment 41
8.2.5. Vital Signs 41
8.2.6. Electrocardiograms 42
8.2.7. Laboratory Assessments 42
8.2.8. Laboratory Events of Clinical Interest 43
8.3. Adverse Events and Serious Adverse Events 43
8.3.1. Time Period and Frequency for Collecting AE and SAE Information 43
8.3.2. Method of Detecting AEs and SAEs 44
8.3.3. Follow-up of AEs and SAEs 44
8.3.4. Regulatory Reporting Requirements for SAEs 44
8.3.5. Special Situations 45
8.4. Pharmacodynamics 46
8.5. Genetics 46
8.6. [Health Economics] OR [Medical Resource Utilization and Health Economics] 46

9. STATISTICAL CONSIDERATIONS 47
9.1. Statistical Hypotheses 47
9.1.1. Phase 2 Period 47
9.1.2. Phase 3 Period 47
9.2. Sample Size Determination 47
9.2.1. Phase 2 Period 47
9.2.2. Phase 3 Period 47
9.3. Data Monitoring Guidelines for an Independent DSMB 48
9.4. Rationale for Early Futility Analysis 49
9.5. Interim Analyses Guidelines 49
9.5.1. Early Assessment of Futility 49

9.5.2. Monitoring Guidelines for the Primary Endpoint in Phase 3 Period49
9.6. Analysis Sets 50
9.7. Statistical Analyses........ 50
9.7.1. General considerations........ 51
9.7.2. Analysis of the Primary Efficacy Endpoint for Phase 2........ 51
9.7.3. Analysis of the Primary Efficacy Endpoint for Phase 3........ 51
9.7.4. Analyses of Secondary Efficacy Endpoints and Subgroups........ 52
9.7.5. Analysis of Safety Outcome........ 52

10. SUPPORTING DOCUMENTATION AND OPERATIONAL CONSIDERATIONS........ 53
10.1. Appendix 1: Regulatory, Ethical, and Study Oversight Considerations........ 53
10.1.1. Regulatory and Ethical Considerations........ 53
10.1.2. Financial Disclosure........ 53
10.1.3. Informed Consent Process........ 53
10.1.4. Data Protection........ 54
10.1.5. Data Quality Assurance........ 54
10.1.6. Source Documents........ 55
10.1.7. Study and Site Start and Closure........ 55
10.1.8. Publication Policy........ 56
10.2. Appendix 2: Clinical Laboratory Tests........ 57
10.3. Appendix 3: Adverse Events: Definitions and Procedures for Recording, Evaluating, Follow-up, and Reporting........ 58
10.3.1. Definition of AE........ 58
10.3.2. Definition of SAE........ 59
10.3.3. Recording and Follow-Up of AE and/or SAE........ 60
10.3.4. Reporting of SAEs........ 62
10.3.5. Reporting of Laboratory Events of Clinical Interest........ 62
10.4. Appendix 4: Contraceptive Requirements and Collection and Reporting of Pregnancy Information........ 63
10.5. Appendix 5: Assessments in Subjects Meeting Laboratory ECI Criteria........ 65

11. REFERENCES........ 67

ABBREVIATIONS

AE	adverse event
ALT	alanine aminotransferase
anti-HBs	antibodies to hepatitis B surface antigen
DNA	deoxyribonucleic acid
DSMB	Data and Safety Monitoring Board
ECG	electrocardiogram
ECI	events of clinical interest
EPO	erythropoietin
ET	early termination
GM-CSF	granulocyte-macrophage colony-stimulating factor
HBeAg	hepatitis B e-antigen
HBsAg	hepatitis B surface antigen
HBV	hepatitis B virus
HCV	hepatitis C virus
HDV	hepatitis D virus
HIV	human immunodeficiency virus
ICF	informed consent form
IFN-α	interferon-alpha
IMP	Investigational medicinal product
IU	international units
kPa	kilopascal
Kg	kilogram
LLOQ	lower limit of quantification
m	meter
µg	microgram
ml	milliliter
NCI-CTCAE	National Cancer Institute Common Terminology Criteria for Adverse Events
NIMP	Non-investigational medicinal product
NrtI	nucleos(t)ide reverse transcriptase inhibitors
ULN	upper limit of normal
PEG-IFN-α	pegylated interferon-alpha
PD	Pharmacodynamic
PK	pharmacokinetic
SoA	schedule of activities
SAE	serious adverse event
WOCBP	women of child-bearing potential

1. PROTOCOL SUMMARY

1.1. Synopsis

Protocol Title: An Adaptive Multicenter, Randomized, Double-Blind, Placebo-Controlled, Parallel-Group Study to Investigate the Efficacy and Safety of BRII-179 (VBI-2601) for the Treatment of Chronic Hepatitis B Virus (HBV) Infection

Rationale:
Multiple therapies with differential mechanisms of actions to treat hepatitis B virus (HBV) is an encouraging strategy to achieve higher rates of hepatitis B surface antigen (HBsAg) loss and ideally HBsAg seroconversion. The present study (BRII-179-002) will evaluate the efficacy and safety of adding a recombinant protein immunotherapeutic (BRII-179 [VBI-2601]) treatment regimen to existing pegylated interferon-alpha (PEG-IFN-α) and nucleos(t)ide reverse transcriptase inhibitor (NrtI) therapy in chronic HBV patients from the Everest Project (NCT04035837). The primary objective of the Everest Project is to achieve functional cure of HBV infection by optimizing treatment strategies of NrtIs and PEG-IFN-α.

Individualized regimens of IFN and NrtI therapies have shown promising efficacy in reducing HBsAg with some patients achieving HBsAg clearance (Ren, 2016; Marcellin, 2016). This result is more pronounced in patients on optimized HBV treatment to low HBsAg levels, which is the rationale of including subjects from the Everest Project in this study. Preclinical and clinical studies have also shown the benefit of low HBsAg levels for breaking tolerance with a therapeutic HBV vaccine (Zhu, 2016; Michler, 2020; Roggendorf, 2019). The recombinant protein immunotherapeutic, BRII-179 (VBI-2601), may break immunological tolerance and restore and/or augment adaptive T cell and antibody responses to the surface antigens. These responses together have the potential to better achieve a functional cure (sustained loss of HBsAg in blood with or without seroconversion to the surface antigen antibody) of chronic HBV infection.

Objectives and Endpoints

This adaptive study design will begin with a phase 2 evaluation of 106 subjects, followed by a transition into a larger phase 3 evaluation which will expand enrollment to include an additional 374 subjects to the study (N=480). The futility assessment performed in Phase 2 to determine graduation to Phase 3 will use the following criterion: BRII-179 is superior (i.e., 1-sided $p < 0.3$) to placebo in achieving HBsAg loss at Week 24.

1.2. Phase 2 (N=106)

Objectives	Endpoints
Primary	
To determine the efficacy of BRII-179 to increase HBsAg loss through Week 24	• Percentage of subjects with HBsAg loss at Week 24
Secondary	

To assess the antiviral activity of BRII-179 (VBI-2601) therapy added to PEG-IFN-α + NrtI	• Change from baseline of HBsAg from Day 1 to Week 24<br>• Percentage of subjects with HBsAg loss at any timepoint
To assess safety of BRII-179 (VBI-2601) therapy	• Percentage of subjects with adverse events (AEs) and serious adverse events (SAEs)<br>• Percentage of subjects with abnormalities in clinical laboratory tests

1.3. Phase 3 (N=480)

Objectives	**Endpoints**
Primary	
To evaluate functional cure of chronic HBV subjects receiving BRII-179 (VBI-2601) therapy added to PEG-IFN-α + NrtI	• Percentage of subjects with sustained HBsAg and HBV deoxyribonucleic acid (DNA) loss (defined as undetectable HBsAg and HBV DNA for at least 24 weeks) after NrtI withdrawal
Secondary	
To assess the antiviral activity of BRII-179 (VBI-2601) therapy added to PEG-IFN-α + NrtI	• Percentage of subjects with undetectable HBV-DNA (HBV DNA < Lower Limit of Quantification (LLOQ)) 24 and 48 weeks after NrtI withdrawal<br>• Percentage of subjects meeting the NrtI withdrawal criteria by end of follow-up period (Week 48)<br>• Percentage of subjects with HBsAg loss at any timepoint<br>• Percentage of subjects with HBsAg seroconversion at any timepoint<br>• Percentage of subjects with antibodies to hepatitis B surface antigen (anti-HBs) response by end of follow-up period (Week 48)<br>• Change from baseline of HBsAg from Day 1 to End of Treatment (Week 32)<br>• Time to achieve HBsAg seroclearance (HBsAg loss) and seroconversion<br>• Percentage of subjects requiring NrtI re-treatment during follow up
To assess safety of BRII-179 (VBI-2601) therapy	• Percentage of subjects with adverse events (AEs) and serious adverse events (SAEs)<br>• Percentage of subjects with abnormalities in clinical laboratory tests

Overall Design

Study BRII-179-002 is a double-blind, randomized, placebo-controlled, parallel-group study of adding BRII-179 (VBI-2601) to existing PEG-IFN-α and NrtI therapy in patients with chronic HBV infection without cirrhosis enrolled in the Everest Project study being conducted in China (NCT04035837).

The Everest Project is a multicenter real-world study being conducted in over 260 hospitals in China. The plan is to recruit 30,000 patients with chronic HBV effectively treated with NrtI therapy for at least one year; who are hepatitis B e-antigen (HBeAg) negative with HBV DNA <100 IU/ml and HBsAg <1500 IU/mL at study entry. As part of the study participation, subjects receive PEG-IFN-α as "add-on" or "switch to" treatment for 48 to 96 weeks. The primary objective of the Everest Project is to achieve functional cure of HBV infection by optimizing treatment strategies of NrtIs and PEG-IFN-α.

Eligible subjects from the Everest Project study, who have received PEG-IFN-α for 24 to 26 weeks and NrtI therapy for at least 18 months, will be randomized 1:1 to receive BRII-179 (VBI-2601) or placebo in addition to the existing PEG-IFN-α and NrtI therapy. Subjects will be stratified at randomization by screening HBsAg level (3 categories: ≥0.05 to < 1 IU/mL, ≥1 to < 10 IU/mL, and ≥10 to <100 IU/mL). Subjects will discontinue PEG-IFN- α at Week 24; subjects will complete investigational treatment with BRII-179 or placebo at Week 32; while NrtI therapy will continue into the Follow-up Period. The BRII-179-002 study design schema can be found in Section 1.4.

Estimated total duration for each subject will include the following: screening up to 14 days, 32-week dosing period, and up to a 16-week follow-up period. Eligible subjects that meet NrtI discontinuation criteria (Section 4.1.2.1) (eligibility assessed at Week 36 to Week 48) may enter a NrtI discontinuation monitoring period for an additional 48 weeks from the day of NrtI discontinuation (Section 1.5.2).

Number of Subjects:

A total of 480 evaluable adult subjects who have received PEG-IFN-α for 24 to 26 weeks and NrtI therapy for at least 18 months from the Everest Project are planned. The initial Phase 2 period will evaluate 106 subjects, followed by a transition into a larger phase 3 evaluation which will expand enrollment to include an additional 374 subjects to the study (N=480).

Intervention Groups and Duration:

Eligible subjects currently receiving PEG-IFN-α and NrtI therapy will receive the study drug BRII-179 (VBI-2601) or matching placebo.

Independent Data and Safety Monitoring Board (DSMB)

An independent DSMB will monitor patient safety and treatment efficacy data for this study. The details for the operation and responsibilities of the DSMB will be defined in a separate DSMB Charter. The Charter will delineate the composition, duties, responsibilities, and procedures of the DSMB, data required at each meeting, as well as the analyses that will be conducted.

Disclosure Statement: This is a phase 2/ phase 3, randomized, multicenter, double-blind, placebo-controlled, parallel-group study to evaluate the efficacy and safety of BRII-179 (VBI-2601) in patients with chronic HBV receiving PEG-IFN-α and NrtI therapy. All references to "Sponsor" contained herein shall refer to Brii Biosciences.

1.4. Schema

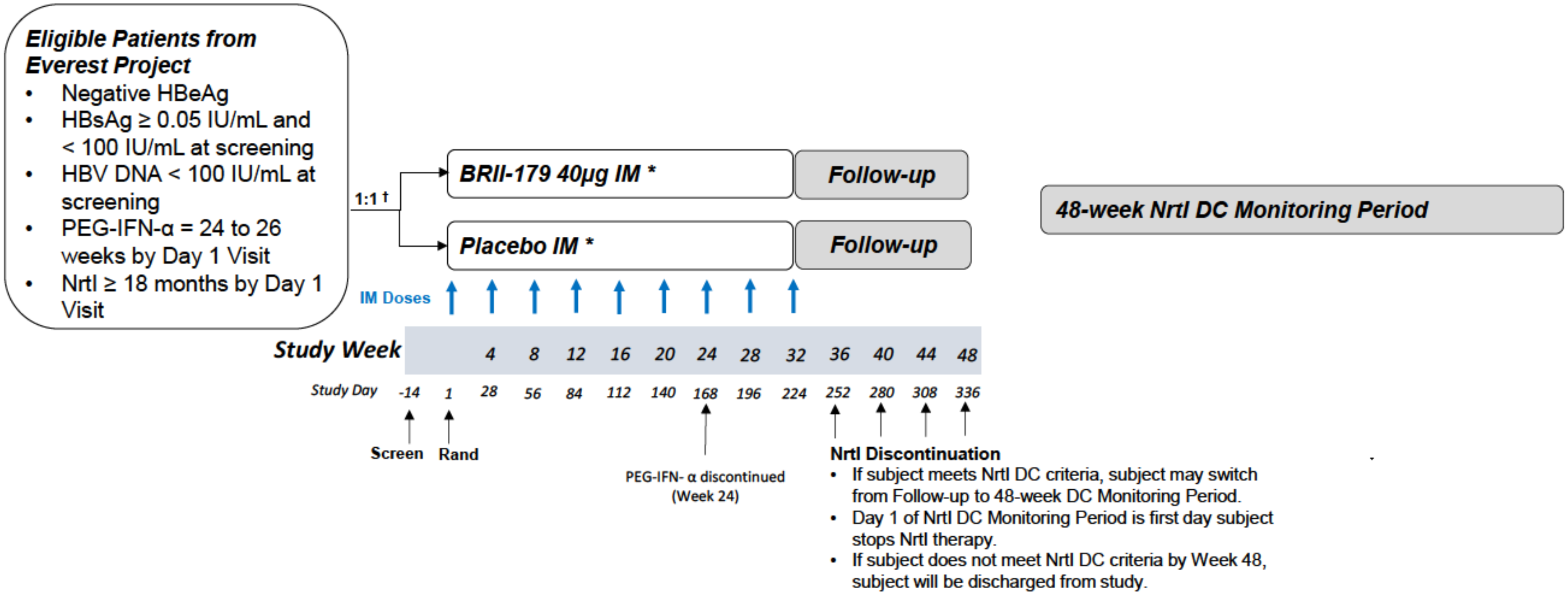

DC = discontinuation; IM = Intramuscular; Rand = Randomization

* Concomitant therapy of PEG-IFN-α and NrtI during dosing period; PEG-IFN- α discontinued at Week 24; NrtI therapy will be continued into the Follow-up Period.

† Subjects stratified by HBsAg level

1.5. Schedule of Activities (SoA)

1.5.1. BRII-179 (VBI-2601) 32-week Dosing Period and 16-week Follow-up Period

Study Stage	Screen	Dosing Period[b]									Follow-up Period			
**Study Visit Week**	Screen	Rand	W4	W8	W12	W16	W20	W24	W28	W32/ET	W36[c]	W40[c]	W44[c]	W48[c]/ET
**Study Visit Day ± Visit Window**	D -14 to -1	D1[a]	D28 ± 3	D56 ± 3	D84 ± 3	D112 ± 3	D140 ± 3	D168 ± 3	D196 ± 3	D224 ± 3	D252 ± 5	D280 ± 5	D308 ± 5	D336 ± 5
Informed consent form (ICF)	X													
Inclusion/exclusion criteria	X	X[d]												
Demography	X													
Medical history including current medical conditions and HBV genotype[e]	X													
Fibroscan	X													
Full physical exam[f]	X				X					X				X
Symptom-directed physical exam		X	X	X		X	X	X	X		X	X	X	
Alcohol intake assessment[g]		X	X	X	X	X	X	X	X	X	X	X	X	X
Vital signs[h]	X	X			X			X		X				
12-lead ECG[i]	X	X								X				
Subject instructed to complete diary		X	X	X	X	X	X	X	X	X	X	X	X	X
Urinalysis	X													
Pregnancy test[j]	X	X	X	X	X	X	X	X	X	X	X	X	X	X
Safety Laboratory Assessments	X	X	X	X	X	X	X	X	X	X	X	X	X	X
Randomization		X												
Study drug administration		X	X	X	X	X	X	X[k]	X	X[l]				
Screening viral serology	X													
Blood sample for HBsAg quantitative	X	X		X		X		X		X	X	X	X	X
Blood sample for anti-HBs quantitative	X	X				X		X		X		X		X
Blood sample for HBV DNA quantitative[m]	X	X				X		X		X	X	X	X	X
Adverse event assessment[n]	←------------------------------------X------------------------------------→													
Concomitant medications review	←------------------------------------X------------------------------------→													
NrtI medication adherence review	←------------------------------------X------------------------------------→													
PEG-IFN-α medication adherence review[o]	←------------------------X------------------------→													

AE = adverse event; anti-HBs = antibodies to hepatitis B surface antigen; HBsAg = hepatitis B surface antigen; ECG = electrocardiogram; ET = early termination; HBV = Hepatitis B Virus; NrtI = nucleoside/nucleotide reverse transcriptase inhibitor; ULN = Upper Limit of Normal

Note: When scheduled at the same timepoints, the assessments of vital signs and 12-lead ECGs must be performed before blood sample collections.

a Assessments performed predose unless otherwise specified.

b If a subject withdraws prematurely from the study prior to their Week 32 visit, ET assessments should be performed within 14 days. Follow Up Visit will be conducted approximately 4 weeks after the last dose of study drug; required only if the participant has ongoing AEs or lab abnormalities at the last on-study visit, and this visit may be conducted by telephone.

c Subjects that meet NrtI discontinuation criteria (Section 4.1.2.1) may discontinue from the Follow-up Period and enter into the NrtI Discontinuation Monitoring Period (Section 1.5.2). Day 1 of the NrtI Discontinuation Monitoring Period is the first day subject stops their NrtI therapy.

d Evaluation of inclusion and exclusion criteria related to pregnancy testing, medical history and concomitant medications prior to dosing.

e A complete medical history will be taken at screening including HBV genotype if available. Any changes should be updated prior to dosing.

f Height and weight will be recorded at screening. Body mass index will be calculated based on the measurements. Only weight will be collected afterwards.

g Subjects' alcohol intake will be recorded while on study.

h Vital signs (blood pressure, pulse rate, respiratory rate, and temperature) should be measured after the subject has rested comfortably for approximately 5 minutes.

i 12-lead safety ECGs should be measured in the supine position after the subject has rested comfortably for 10 minutes.

j Women of childbearing potential are required to have pregnancy tests. Negative pregnancy test must be confirmed prior to study drug administration. Urine pregnancy test results from local laboratory are acceptable (except for the screening visit).

k Subjects will no longer continue PEG-IFN-α after Week 24; NrtI therapy will continue.

l Subjects will complete investigational treatment with BRII-179 or placebo; NrtI therapy will continue into the Follow-up Period

m If a subject experiences alanine aminotransferase (ALT) flare (as defined by ALT > 2x ULN) additional HBV DNA quantitation sample(s) may be collected.

n All AEs related to screening activities must be collected from the time of consent until end of study; any other events reported during the screening period should be reported as medical history. All SAEs must be collected from the time of consent until end of study.

o Subjects will be instructed to bring PEG-IFN-α concomitant medications to the trial site at every visit (including all empty containers), as applicable, beginning with the screening visit. Adherence will be assessed by counting of empty containers and unused medication. See Section 6.4.1.

1.5.2. NrtI Discontinuation Monitoring Period

Study Stage	NrtI Discontinuation Monitoring Period[b,c]						
**Study Visit Week**		W4	W8	W12	W24	W36	W48/ET
**Study Visit Day ± Visit Window**	D1[a]	D28 ± 3	D56 ± 3	D84 ± 3	D168 ± 7	D252 ± 7	D336 ± 7
Safety Laboratory Assessments[d]	X	X	X	X	X	X	X
Symptom-directed physical exam	X	X	X	X	X	X	X
Fibroscan							X
Pregnancy test		X					X
Blood sample for HBsAg quantitative	X			X	X	X	X
Blood sample for anti-HBs quantitative	X			X	X	X	X
Blood sample for HBV DNA quantitative	X	X	X	X	X	X	X
Concomitant medications	←-- X --→						
Adverse event assessment	←-- X --→						
NrtI retreatment surveillance[e]	←-- X --→						
ECI surveillance[f]	←-- X --→						

AE = adverse event; anti-HBs = antibodies to hepatitis B surface antigen; ECI = events of clinical interest; ET = early termination; HBsAg = hepatitis B surface antigen; HBV = Hepatitis B Virus; NrtI = nucleoside/nucleotide; ULN = Upper Limit of Normal

[a] All timepoints are relative to the day of NrtI discontinuation (D1) which is the first day subjects stop their NrtI therapy.

[b] All subjects who discontinue NrtI therapy will be followed through 48 weeks, even if NrtI retreatment occurs. If a subject withdraws prematurely from the study prior to the W48 visit, ET assessments should be performed.

[c] NrtI retreatment guidelines are provided in Section 4.1.2.2.

[d] Safety laboratory assessments are limited to liver function tests.

[e] Investigator should monitor subjects for NrtI retreatment criteria. See Section 4.1.2.2.

[f] Subject will be monitored for clinical laboratory events of clinical interest (ECI) and additional assessments may be performed if indicated. ECI for this study include ALT > 3x ULN, if Day 1 predose ALT ≤ ULN; ALT > 2x baseline, if Day 1 predose ALT > ULN.

2. INTRODUCTION

2.1. Background

Chronic HBV infection remains an important global public health problem that leads to serious sequelae over time, including cirrhosis, liver failure, and hepatocellular carcinoma (Trepo, 2014). An estimated 290 million people are living with chronic HBV infection worldwide, and almost 800,000 people die annually due to associated sequelae (Stanaway, 2016).

Human HBV is a deoxyribonucleic acid (DNA) virus in the Hepadnaviridae family, with 10 HBV genotypes (A to J). The distribution of these genotypes varies geographically, with A and D being more common in Northern Europe, North America, and Western and Central Asia; and B and C being more common in Southeast Asia and China. HBV genotypes may influence disease progression rates and treatment response (Kramvis, 2014).

Chronic HBV infection is characterized by detection of HBV surface antigen (HBsAg) in the blood for > 6 months. The prevalence of chronic HBV infection varies geographically, with a range of < 2% in low to > 8% in high prevalence countries (Schweitzer, 2015). In high prevalence countries, such as those in sub-Saharan Africa and East Asia, transmission occurs predominantly in infants and children by perinatal and horizontal routes. In more industrialized countries, new infections are highest among young adults and transmission occurs predominantly via injection drug use and high-risk sexual behaviors. The risk of developing chronic HBV infection depends on the age at the time of infection. While only ~10% of people infected as adults develop chronic HBV infection, 90% of infants infected perinatally or during the first 6 months of life, and 20% to 60% of children infected between 6 months and 5 years of age remain chronically infected. Twenty-five percent of people who acquire HBV during infancy and childhood will develop primary liver cancer or cirrhosis during adulthood.

Current treatment options for chronic HBV infection are limited. One option is long-term Nucleoside reverse transcriptase inhibitors (NrtI) therapy, for example tenofovir (tenofovir disoproxil fumarate or tenofovir alafenamide) or entecavir (Lok, 2016). However, NrtI therapy does not result in a cure of HBV infection. The viral DNA is not cleared from the infected cell, and viral protein production, in particular the production of HBsAg, typically continues at high levels as measured in blood (Scaglione, 2012). These high levels of HBsAg subviral particles are proposed to mediate HBV-specific immune dysfunction by decoying and exhausting host cellular and humoral adaptive immune responses. Therefore, reduction or clearance of HBsAg from blood is considered central to achieving a functional cure (sustained loss of HBsAg in blood with or without seroconversion to the surface antigen antibody) and is considered an optimal outcome of treatment (Block, 2013). An alternative treatment option is a finite course of Pegylated Interferon α (PEG-IFN-α) therapy which can induce a functional cure, but only in a small percentage of patients (< 10%) (Konerman, 2016). Unfortunately, PEG-IFN-α therapy carries considerable risk of dose-limiting adverse events (AEs) which may be severe and/or serious, and discontinuation rates are significant (Perrillo, 2009).

The failure of NrtI therapy to produce a functional cure and the limitations of PEG-IFN-α therapy highlight the clinical need for effective, well-tolerated HBV therapies of finite duration.

2.2. Study Rationale

BRII-179 (VBI-2601) is a recombinant virus-like particle-based (VLP) based protein immunotherapeutic derived from the 3rd generation HBV preventative vaccine, Sci-B-Vac® consists of all three HBV surface (S) antigen forms: small, middle and large proteins. The large S protein contains the N-terminal preS1 and preS2 domains and the adjacent small S protein. The middle S protein contains preS2 and the adjacent small S protein.

It has been hypothesized that high levels of circulating HBsAg lead to immune tolerance of HBV and contribute to chronic HBV infection. BRII-179 (VBI-2601) utilizes Pre-S1, Pre-S2, and S protein components as the immunogen in a therapeutic application of preS1/S2 containing HBsAg. Additionally, to have an effective T cell response, BRII-179 (VBI-2601) differs from Sci-B-Vac® and most of the recombinant envelope vaccines by using aluminium phosphate as an adjuvant rather than aluminum hydroxide. As a new formulation of higher doses of Sci-B-Vac® with aluminum-phosphate adjuvant, BRII-179 (VBI-2601) shows enhanced Th1 responses against the HBV surface antigens. BRII-179 has been shown to be safe and well-tolerated in chronic HBV (CHB) patients under Nrtl therapy in a phase 1b/2a study (BRII-179-001). Administration of BRII-179 carrying S, Pre-S1 and Pre-S2 regions of HBV envelope is immunogenic at both B and T cell levels in CHB patients.

Multiple therapies with differential mechanisms of actions to treat HBV is an encouraging strategy to achieve higher rates of HBsAg loss and ideally HBsAg seroconversion. The present study (BRII-179-002) will evaluate the efficacy and safety of adding a recombinant protein immunotherapeutic (BRII-179 [VBI-2601]) treatment regimen to existing PEG-IFN-α and NrtI therapy in chronic HBV patients who have participated in the Everest Project (NCT04035837). The primary objective of the Everest Project is to achieve functional cure of HBV infection by optimizing combination and sequential treatment strategies of NrtIs and PEG-IFN-α in patients with chronic HBV infection (negative HBeAg) and low levels of HBsAg (≤ 1500 IU/mL) and HBV DNA (< 100 IU/mL).

Individualized regimens of combination therapy of IFN-α, an innate immunomodulator and antiviral agent and NrtI, a direct acting antiviral agent have shown promising efficacy in reducing HBsAg with some patients achieving HBsAg clearance (Ren, 2016; Marcellin, 2016). This result is more pronounced in patients on optimized HBV treatment to low baseline HBsAg levels, which is the rationale of including subjects from the Everest Project in this study. Preclinical and clinical studies have also shown the benefit of low HBsAg levels for breaking tolerance with a therapeutic HBV vaccine (Zhu, 2016; Michler, 2020; Roggendorf, 2019). BRII-179 (VBI-2601), as an immunomodulator, can break immunological tolerance and restore and/or augment cellular T cell and antibody responses to the HBV surface antigens which in turn result in HBsAg clearance. Therefore, the initial treatment of PEG-IFN-α and NrtI followed by BRII-179 add-on treatment could increase the potential to achieve higher functional cure rate in chronic HBV infected patients.

2.3. Benefit/Risk Assessment

This study will provide information on the efficacy and safety of BRII-179 (VBI-2601) added to PEG-IFN-α and NrtI optimized therapy to achieve functional cure in patients with chronic HBV infection.

BRII-179 (VBI-2601) with or without IFN-α as an adjuvant, administered every 4 weeks for a total of 4 doses, have been evaluated in adult subjects with chronic HBV infection. Favorable safety and tolerability profiles were observed at 40 µg dose level. In addition, the pivotal chronic toxicology studies also support the proposed BRII-179 (VBI-2601) dose regimen of 40 µg every 4 weeks for 9 doses.

The proposed addition of BRII-179 (VBI-2601) to PEG-IFN-α and NrtI therapy may further reduce HBsAg effectively and induce HBV-specific immunity which may lead to immune control of chronic HBV infection. The safety profile of BRII-179 (VBI-2601) is supportive of the overall risk/benefit profile for the proposed dose regimen and treatment duration.

More detailed information about the known and expected benefits and risks and reasonably expected adverse events of BRII-179 (VBI-2601) may be found in the Investigator’s Brochure.

3. OBJECTIVES AND ENDPOINTS

3.1. Objectives

The primary objective is:

- Phase 2: To determine the efficacy of BRII-179 to increase HBsAg loss through 24 Weeks
- Phase 3: To evaluate functional cure of chronic HBV subjects receiving BRII-179 (VBI-2601) therapy added to PEG-IFN-α + NrtI.

The secondary objectives are:

- To assess the antiviral activity of BRII-179 (VBI-2601) therapy added to PEG-IFN-α + NrtI.

- To assess safety of BRII-179 (VBI-2601) therapy

3.2. Endpoints

3.2.1. Primary Endpoints

- Phase 2: Percentage of subjects with HBsAg loss at Week 24

- Phase 3: Percentage of subjects with sustained HBsAg and HBV DNA loss (defined as undetectable HBsAg and HBV DNA for at least 24 weeks) after NrtI withdrawal

3.2.2. Secondary Endpoints

- Phase 2: Change from baseline of HBsAg from Day 1 to Week 24

- Phase 2: Percentage of subjects with HBsAg loss at any timepoint

- Phase 3: Percentage of subjects with undetectable HBV-DNA (HBV DNA < LLOQ) 24 and 48 weeks after NrtI withdrawal

- Phase 3: Percentage of subjects meeting the NrtI withdrawal criteria by end of follow-up period (Week 48)

- Phase 3: Percentage of subjects with HBsAg loss at any timepoint

- Phase 3: Percentage of subjects with HBsAg seroconversion at any timepoint

- Phase 3: Percentage of subjects with anti-HBs response by end of follow-up period (Week 48)

- Phase 3: Change from baseline of HBsAg from Day 1 to End of Treatment (Week 32)

- Phase 3: Time to achieve HBsAg seroclearance (HBsAg loss) and seroconversion
- Phase 3: Percentage of subjects requiring NrtI re-treatment during follow up
- Phase 2 and Phase 3: Percentage of subjects with adverse events (AEs) and serious adverse events (SAEs)
- Phase 2 and Phase 3: Percentage of subjects with abnormalities in clinical laboratory tests

4. STUDY DESIGN

4.1. Overall Design

Study BRII-179-002 is a double-blind, randomized, placebo-controlled, parallel-group study of adding BRII-179 (VBI-2601) to existing PEG IFN-α and NrtI therapy in patients with chronic HBV infection without cirrhosis enrolled in the Everest Project study being conducted in China (NCT04035837).

The Everest Project is a multicenter real-world study being conducted in over 260 hospitals in China. The plan is to recruit 30,000 patients with chronic HBV effectively treated with NrtI therapy for at least one year; who are HBeAg negative with HBV DNA <100 IU/ml and HBsAg <1500 IU/mL at study entry. As part of the study participation, subjects receive PEG-IFN-α as "add-on" or "switch to" treatment for 48 to 96 weeks. The primary objective of the Everest Project is to achieve functional cure of HBV infection by optimizing treatment strategies of NrtIs and PEG-IFN-α.

Eligible subjects from the Everest Project study, who have received PEG-IFN-α for 24 to 26 weeks and NrtI therapy for at least 18 months, will be randomized 1:1 to receive BRII-179 (VBI-2601) or placebo in addition to the existing PEG-IFN-α and NrtI therapy. Subjects will be stratified at randomization by screening HBsAg level (3 categories: ≥0.05 to < 1 IU/mL, ≥1 to < 10 IU/mL, and ≥10 to <100 IU/mL). Subjects will discontinue PEG-IFN- α at Week 24; subjects will complete investigational treatment with BRII-179 or placebo at Week 32; while NrtI therapy will continue into the Follow-up Period. The BRII-179-002 study design schema can be found in Section 1.4.

Estimated total duration for each subject will include the following: screening up to 14 days, 32-week dosing period, and up to a 16-week follow-up period. Eligible subjects that meet NrtI discontinuation criteria (Section 4.1.2.1) (eligibility assessed at Week 36 to Week 48) may enter a NrtI discontinuation monitoring period for an additional 48 weeks from the day of NrtI discontinuation (Section 1.5.2).

4.1.1. Independent DSMB

An independent DSMB will monitor patient safety and treatment efficacy data for this study. The details for the operation and responsibilities of the DSMB will be defined in a separate DSMB Charter. The Charter will delineate the composition, duties, responsibilities, and procedures of the DSMB, data required at each meeting, as well as the analyses that will be conducted.

4.1.2. NrtI Discontinuation

4.1.2.1. NrtI Discontinuation Criteria

The investigator will evaluate whether subjects are eligible for discontinuation of NrtI therapy starting at Week 36 and potentially monthly until Week 48 based on the following NrtI discontinuation criteria:

- Alanine aminotransferase (ALT) < 2x ULN (results from previous visit) AND
- HBV DNA < LLOQ (results from previous visit) AND
- 2 consecutive assessments of HBsAg < 0.05 IU/mL (assessed starting from Week 28 laboratory results)

Subjects who are eligible to discontinue from NrtI therapy may discontinue from the Schedule of Activities in Section 1.5.1 and may instead continue into the NrtI Discontinuation Monitoring Period of the study. These subjects will follow the Schedule of Activities for subjects who have discontinued from the NrtI therapy (Section 1.5.2). Subjects who discontinue from their NrtI therapy will be followed in the NrtI Discontinuation Monitoring Period for an additional 48 weeks from their day of NrtI discontinuation.

4.1.2.2. NrtI Retreatment

It is recommended that the Investigator should consider retreatment with NrtI therapy during the NrtI Discontinuation Monitoring Period if subjects meet the following criteria at the same visit (based on Papatheodoridi, 2020):

- ALT > 15x ULN with positive HBV DNA
- ALT 10-15x ULN with positive HBV DNA: retreatment may be implemented at investigator's discretion
- ALT 5-15x ULN with elevated total bilirubin > 2 mg/dL (predominantly direct) and/or prolongation of prothrombin time (PT) ≥ 3 seconds with positive HBV DNA
- ALT 2-5x ULN and HBV DNA >2000 IU/mL persisting for ≥ 12 consecutive weeks
- ALT > 3x ULN and HBV DNA > 100,000 IU/mL at the same visit

Whether subjects are retreated with NrtI is at the discretion of the Investigator following good medical judgement. Subjects who are retreated with NrtI therapy should continue to be followed per the SoA Section 1.5.2 for the duration of the 48-week NrtI Discontinuation Monitoring Period.

4.1.3. PEG-IFN-α Therapy During the Dosing Period

Subjects entering the Dosing Period of the study will continue their peg-IFN-α treatment. The dose of peg-IFN-α is based on relevant clinical guidelines and information from the package insert which is considered as proper by the investigator. Temporary interruptions of peg-IFN-α administration are discouraged. Subjects are to remain on treatment for the entire duration of the study until completion of Week 24 and the dose of peg-IFN-α should not be changed during the trial unless dose reduction or discontinuation is indicated because of laboratory abnormalities/adverse events (see Section 4.1.3.1 for instructions for dose reduction). Subjects must be counselled regarding the importance of not missing doses of peg-IFN-α.

In the case that a patient does miss a dose(s):

- If the peg-IFN-α injection was late by 1 to 3 days, the next dose should be administered immediately. The subsequent dose should then be administered on the next scheduled injection date.
- If the peg-IFN-α injection was late by 4 to 7 days, patient should wait to receive the next scheduled injection (1 dose is skipped).
- If the peg-IFN-α injection was late by >7 days, treatment can be restarted at any time by the investigator and the next dose should be on scheduled injection dates.

4.1.3.1. Dose Adjustment for adverse drug reactions

When a peg-IFN-α dose modification is required for moderate to severe adverse reactions (clinical and/or laboratory), initial dose reduction should be performed. However, in some cases, further dose reduction may be needed. Following improvement of the adverse reaction, re-escalation of the peg-IFN-α dose may be considered.

Hematology:

Peg-IFN-α Hematological Dose Modification Guidelines

Laboratory Values	Reduce PEG-IFN-α Dose:	Discontinue PEG-IFN-α if
Absolute neutrophil count (ANC)	≥750/mm^3: Maintain dose<br>< 750/mm^3: Reduce dose	< 500/mm^3: Treatment should be suspended until ANC values return to more than 1000/mm^3<br>Re-institute at low dose and monitor ANC
Platelet	≥ 50.000/mm^3: Maintain dose<br>< 50.000/mm^3: Reduce to low dose	<25.000/mm^3

Liver function:

In patients with progressive or persistent ALT increases > 10 x ULN and >2 x baseline values, the dose of peg-IFN-α should be reduced and more frequent monitoring of liver function should be performed. If ALT increases are further progressive despite dose reduction or accompanied by increased bilirubin or evidence of hepatic decompensation, therapy should be immediately discontinued. After peg-IFN-α dose reduction or withholding, therapy can be resumed after ALT flares subside.

Other disorders:

Peg-IFN-α may cause or aggravate neuropsychiatric, autoimmune, endocrine, ophthalmologic, and infectious disorders. Monitor closely and withdraw therapy with persistently severe or worsening signs or symptoms of the above disorders. Please see package insert.

4.2. Scientific Rationale for Study Design

This is an adaptive phase 2/ phase 3, double-blind, randomized, placebo-controlled, parallel-group study of adding BRII-179 (VBI-2601) to existing PEG-IFN-α and NrtI therapy in patients with chronic HBV infection without cirrhosis enrolled from the Everest Project study being conducted in China. The study design will provide an opportunity to evaluate the ability of BRII-179 (VBI-2601) to achieve a clinically meaningful increase in functional cure (sustained loss of HBsAg in blood with or without seroconversion to the surface antigen antibody) in subjects with chronic HBV on optimized PEG IFN-α and NrtI therapy as compared to therapy consisting of optimized PEG IFN-α and NrtI therapy alone.

There is increasing evidence that low levels of quantitative HBsAg may improve the potential for HBsAg loss, and this is most evident in patients who lose HBsAg after stopping long-term NrtI therapy. It has been observed that patients with decreasing HBsAg and a lower HBsAg level < 100 IU/mL at the end of treatment were able to maintain control of HBV after discontinuation of long-term NrtI therapy (Chang, 2015; Yao 2017; Jeng, 2018; Papatheodoridi, 2020). Recent reporting from the Everest Project seems to corroborate this observation from previous studies. In 1427 patients on background NrtI therapy who have completed 24 to 26 weeks of PEG-IFN-α treatment and 516 patients who have completed 48 weeks of PEG-IFN-α treatment, the overall rate of HBsAg loss for patients treated ≥12, ≥24, ≥36 and ≥48 weeks was 13.00%, 16.06%, 20.49% and 23.01%, respectively. Subgroup analysis based on baseline HBsAg stratification (0.05–100, 100–500, 500–1000, 1000–1500 IU/mL) found that the HBsAg loss rate was highest in patients with baseline HBsAg between 0.05 and 100 IU/mL (34.93% for patients treated ≥ 48 weeks) (Ze-Qian, 2020).

Viral rebound may occur shortly after NrtI withdrawal in some cases, which requires close monitoring during study participation in order to control ALT flares. The HBV viral relapse after NrtI withdrawal may induce the host immune response and potentially a long-term immune control against the viral infection. Sustained HBsAg loss and/or virological response were observed in HBeAg-negative patients stopping NrtI therapy in prospective cohort studies (van Bömmel, 2017) as well as in a randomized controlled study (Berg, 2017). This study design should allow for the evaluation of the potential benefit from immune responses resulting from BRII-179 and decreased viral antigen exposure after long-term NrtI cessation.

Surrogate endpoints of HBV functional cure measure biochemical, virological, and histological outcomes. Based on current knowledge, the best surrogate of HBV functional cure is HBsAg loss confirmed on 2 occasions at least 6 months apart without requirement forHBsAg seroconversion plus undetectable HBV DNA (Cornberg, 2020). The incidence of sustained HBsAg loss and undetectable HBV DNA will be used to assess the potential of BRII-179 (VBI-2601) to induce functional cure.

The target population of this study is HBeAg-negative non-cirrhotic patients with chronic HBV infection that has been virologically suppressed (baseline HBsAg ≥ 0.05 IU/mL and

< 100 IU/mL). This represents a patient population who may have a higher likelihood of sustained HBsAg loss.

4.3. Justification for Dose

It is believed that high levels of HBsAg have a negative impact on immune profile and may tolerize or delete HBV specific T cells in Chronic HBV patients (Kondo, 2013; Ferrari, 2015; Lebossé, 2017; Wang, 2013). As demonstrated in previous nonclinical studies (Zhu, 2016; Michler, 2020), initial reductions of HBsAg by therapeutic interventions enabled follow-up HBV vaccine treatments to break tolerance and achieve functional cure in animal HBV models. The BRII-179-001 study, in chronic HBV patients, demonstrated that BRII-179 at 20 or 40 ug with or without IFN-α was safe and well tolerated as well as induced humoral and cellular immune responses in a significant number of subjects under Nrtl background therapy. In the BRII-179-001 clinical study, antibody response to pre-S1 and/or pre-S2 antigens was only seen in cohorts admixed with IFN-α, suggesting addition of IFN-α as co-adjuvant may further enhance the restimulation of immune response to pre-S1 and pre-S2 antigens. This is consistent with the preclinical study results in the AAV-HBV model. When high HBsAg levels were lowered by siRNA treatment, BRII-179 treatment significantly delayed rebound of HBsAg level compared to siRNA treatment alone in the AAV-HBV model. It is thus hypothesized that significant HBsAg reduction by optimized PEG-IFN-α and NrtI therapy in chronic HBV patients will provide an optimal treatment window to maximize the BRII-179 potential in restoring and sustaining immune control of HBV infection, and ultimately lead to functional cure.

High doses of prophylactic HBV vaccines and increasing the number of doses (6-11) have been demonstrated to provide improved and durable immune response in chronic HBV patients (Hoa, 2009; Roggendorf, 2019; Wong, 2018). Therefore, it is postulated that reducing HBsAg level with optimized PEG IFN-α and NrtI therapy followed by the addition of BRII-179 (VBI-2601), e.g., up to 9 monthly injections of 40 µg of BRII-179, in chronic HBV patients could provide desirable HBV specific humoral and T cell responses that may restore and sustain immune control of HBV infection, thereby achieving functional cure. In addition, the pivotal chronic toxicology studies also support the proposed BRII-179 (VBI-2601) dose regimen of 40 µg every 4 weeks for 9 doses. More detailed information may be found in the Investigator's Brochure for BRII-179 (VBI-2601).

In summary, currently available safety, tolerability, pharmacokinetic (PK), pharmacodynamic (PD) and PK/PD results from BRII-179 (VBI-2601), as well as the safety margin assessment from the toxicology studies, support the proposed BRII-179 (VBI-2601) treatment regimen to achieve functional cure in patients with chronic HBV infection.

4.4. End of Study Definition

A subject is considered to have completed the study if he/she has completed all phases of the study including the last scheduled procedure shown in the Schedule of Activities.

The end of the study is defined as the date of the last visit of the last subject in the study or last scheduled procedure shown in the Schedule of Activities for the last subject in the trial.

5. STUDY POPULATION

A total of 480 evaluable adult subjects with virologically suppressed chronic HBV infection without cirrhosis receiving PEG-IFN-α and NrtI therapy are planned for this study.

Prospective approval of protocol deviations to recruitment and enrollment criteria, also known as protocol waivers or exemptions, is not permitted. Adherence to the criteria as specified in the protocol is essential.

5.1. Inclusion Criteria

Everest Project subjects eligible to be included in the study must meet all of the following criteria. All laboratory test requirements for inclusion will be performed by central laboratory unless specified otherwise.

1. A signed and dated written Informed Consent Form (ICF) prior to study participation.
2. Age ≥ 18 (or age of legal consent, whichever is older) , and ≤ 60 at time of screening.
3. Documented chronic HBV infection (positive serum HBsAg for ≥ 6 months).
4. Negative HBeAg
5. HBsAg ≥ 0.05 IU/mL and < 100 IU/mL at screening.
6. HBV DNA < 100 IU/mL at screening
7. Must be receiving NrtI therapy for ≥ 18 months and PEG-IFN-α for 24 to 26 weeks of the Day 1 visit.
8. Female subjects must have a negative pregnancy test or confirmation of post-menopausal status. Post-menopausal status is defined as 12 months with no menses without alternative medical cause. Women of child-bearing potential (WOCBP) must have a negative serum pregnancy test at screening and a negative urine pregnancy test on Day 1, cannot be breast feeding, and must be willing to use highly effective methods of contraception 14 days before study drug administration through 12 weeks after last study drug administration.
9. Agrees to not donate blood during the duration of the study.
10. Serum ALT and AST ≤ 2x ULN at screening. Study screening laboratory tests may be repeated once for values considered to be erroneous with medical monitor approval.

5.2. Exclusion Criteria

Everest Project subjects are excluded from the study if any of the following criteria apply:

1. Any clinically significant chronic medical condition other than chronic HBV infection that, in the opinion of the investigator makes the patient unsuitable for participation in the study.

2. Any clinically significant acute condition such as fever (> 38º C) or acute respiratory illness within 7 days of first study drug administration.

3. Significant liver fibrosis or cirrhosis as defined by having either a FibroScan result of > 9kPa at screening, unless the subject has results from a FibroScan evaluation performed within 6 months prior to screening or liver biopsy performed within 1 year prior to screening that confirm the absence of Metavir F3 fibrosis or F4 cirrhosis. If a subject has had both procedures in the specified timeframes, the most recent result should be used to determine eligibility.

4. History or evidence of drug or alcohol abuse as assessed by the Investigator, within the 12 months before screening.

5. History of intolerance to IM injection.

6. History of clinically significant chronic liver disease from any cause other than chronic HBV infection.

7. History of hepatic decompensation, including ascites, hepatic encephalopathy and/or esophageal or gastric varices.

8. Treatment by immunosuppressant (including but not limited to corticosteroids at a prednisolone equivalent dose > 20 mg/day) within 30 days before study drug administration at the Day 1 visit and throughout the duration of study participation.

9. Known history of immunological function impairment, including but not limited to:

   - Autoimmune disease (e.g., multiple sclerosis, type 1 diabetes mellitus , myasthenia gravis, Crohns disease and other inflammatory bowel diseases, celiac disease, systemic lupus erythematosus, scleroderma, including diffuse systemic form and limited cutaneous form of systemic sclerosis or CREST syndrome, systemic sclerosis, dermatomyositis polymyositis, rheumatoid arthritis, juvenile idiopathic arthritis, autoimmune thyroiditis including Hashimoto thyroiditis, Grave’s or Basedow’s disease, immune thrombocytopenic purpura, autoimmune hemolytic anemia, autoimmune hepatitis, psoriasis, vitiligo, vasculitis, Guillain Barré syndrome, Addison’s disease, Bell’s palsy, and alopecia areata); OR

- Primary immunodeficiency disorders (e.g., common variable immune deficiency, defective phagocytic cell function and neutropenia syndromes, and complement deficiency); OR

- Secondary immunodeficiency disorders (e.g., resulting from acquired immunodeficiency syndrome caused by human immunodeficiency virus (HIV) infection, solid organ transplant, and splenectomy).

10. Any HBV vaccine (licensed or experimental) prior to study enrolment and throughout the duration of study participation.

11. Immunization with attenuated vaccines (e.g., Mumps, Measles, Rubella) within 30 days before study drug administration at the Day 1 visit and throughout the duration of study participation.

12. Has received blood products or immunoglobulin within 90 days before study drug administration at the Day 1 visit or likely to require blood products during the study.

13. Participated in another clinical trial with an investigational drug or a biologic within 30 days or within 5 drug half-lives (whichever is longer) before study drug administration at the Day 1 visit. Subjects must agree not to take part in any other study at any time during their participation in this study, inclusive of the follow-up period.

14. Has received granulocyte macrophage colony stimulating factor (GM CSF) or erythropoietin (EPO) within 30 days before study drug administration at the Day 1 visit or likely to require GM CSF/EPO during the study.

15. Previous or current psychiatric condition that precludes compliance with the protocol. Specifically excluded are persons with psychoses, ongoing risk for suicide, or history of suicide attempt or gesture within the past 5 years.

16. The subject has any history of cancer requiring locoregional or systemic therapy within 5 years of randomization or has current disease. Subjects with completely surgically resected tumors with no evidence of disease for more than 3 years, or a history of low risk basal cell carcinoma will be accepted (low risk being defined as 1) location on the trunk of the body, arms, legs, cheeks, forehead, temples, scalp, neck, or chin, 2) < 2 cm in size, 3) nodular or superficial, 4) primary cancer that has not come back after treatment, 5) edge of the cancerous area is clear and smooth, and 6) not located in or around nerves).

17. Any skin abnormality or tattoo that would limit post treatment injection site assessment.

18. Known hypersensitivity or contraindication to BRII-179 (VBI-2601) or its excipients.

19. Unwilling, or unable in the opinion of the investigator, to comply with study requirements, including the use of an adequate birth control method.

20. Active infection with human immunodeficiency virus (HIV), hepatitis C virus (HCV) or hepatitis Delta virus.

21. Donated more than 500 mL of blood within 90 days before study drug administration.

22. Renal impairment with estimated glomerular filtration rate (eGFR) ≤ 60 ml/min/1.73 m^2 at screening.

23. Uncontrolled diabetes mellitus as determined by the Investigator.

24. Diagnosis of advanced stage heart failure (New York Heart Association Class III or Class IV or unstable angina).

25. Any laboratory test abnormality that is considered as clinically significant or may contradict continued treatment with peg-IFN by the investigator.

5.3. Lifestyle Considerations

1. Alcohol consumption is limited to no more than 1 unit per day (unit: 1 glass of wine [125 mL] = 1 measure of spirits [30 mL] = one-half pint of beer [284 mL]) during the participation of this study.

5.4. Screen Failures

Screen failures are defined as subjects who consent to participate in the clinical study but are not subsequently randomly assigned to study drug. A minimal set of screen failure information is required to ensure transparent reporting of screen failure subjects to meet the Consolidated Standards of Reporting Trials (CONSORT) publishing requirements and to respond to queries from regulatory authorities. Minimal information includes demography, screen failure details, eligibility criteria, and any serious adverse event (SAE).

Individuals who do not meet the criteria for participation in this study (screen failure) may not be rescreened.

6. STUDY INTERVENTION

6.1. Study Intervention(s) Administered

6.1.1. Formulations

6.1.1.1. BRII-179 and Placebo

The following study drugs will be used in this study:

- BRII-179 40µg IM injection
- Placebo IM injection

BRII-179 (VBI-2601) is aluminium phosphate absorbed HBsAg Bulk Drug Substance (HBsAg spherical particles [~30 nm mean diameter] suspended in phosphate-buffered saline) and 8% Sucrose, in addition to 10 mM phosphate buffer.

Placebo will be prepared from commercially available 0.9% Sodium Chloride for Injection which will be provided to the study site. Subjects assigned to placebo will receive a volume of placebo that would be equal to the volume that would be required if that subject had been assigned to IP.

6.1.1.2. Other Study Medications

Brii Biosciences will also be providing commercially available PEG-INF-α.

PEG-INF-α is a colorless, clear liquid formulation of peginterferon α-2b from modified recombinant human interferon α2b expressed in yeast system with polyethylene glycol (40 kD Y type) and excipients of sodium chloride, sodium acetate, mannitol, aspartic acid, water for injection, and sodium hydroxide to adjust pH.

6.1.2. Dosage and Administration of BRII-179 and Placebo

**Arm Name**	**BRII-179 (VBI-2601)**	**Placebo**
**Intervention Name**	BRII-179 (VBI-2601)	Placebo
**Type**	Drug	Placebo
**Dose Formulation**	Solution for Injection	0.9% Sodium Chloride for Injection
**Dose Level(s)**	BRII-179 40 µg	Placebo
**Route of Administration**	BRII-179 in one syringe; administer via IM injection	Placebo in one syringe; administer via IM injection

Arm Name	BRII-179 (VBI-2601)	Placebo
	Refer to the Pharmacy Manual for the details.	Refer to the Pharmacy Manual for the details.
**Use**	Experimental	Experimental
**IMP and NIMP**	IMP	Not applicable
**Sourcing**	Provided centrally by the Sponsor	Provided centrally by the Sponsor
**Packaging and Labeling**	IMP will be labeled as required per country requirement.	Placebo will be prepared and labelled as instructed in the Pharmacy Manual

On dosing days, the pharmacist or designee will prepare the required amount of study drugs into syringe for injection. A qualified clinical investigative site staff member under the supervision of the investigator or designee will administer the study drug. The injection site will be marked and mapped for later observation and should be documented. If a local reaction around the injection site occurs, photographs may be obtained. Please refer to Section 6.5 for further details.

6.2. Preparation/Handling/Storage/Accountability

- Only subjects enrolled in the study may receive study drug and only authorized site staff may supply or administer study drug. All study drug must be stored in a secure, environmentally controlled, and monitored (manual or automated) area in accordance with the labeled storage conditions with access limited to the investigator and authorized site staff.
- The investigator, institution, or the head of the medical institution (where applicable) is responsible for study drug accountability, reconciliation, and record maintenance (i.e., receipt, reconciliation, and final disposition records).
- Further guidance and information for the final disposition of unused study drugs are provided in the Pharmacy Manual.

6.3. Randomization and Blinding

Subjects will be assigned to study treatment in accordance with the randomization schedule. The randomization code will be generated by using a validated computerized system. Subjects will be randomized using an Interactive Web Response System (IWRS). Once a randomization number is assigned to a subject, it cannot be reassigned to any other subject in the study. Study intervention will be dispensed at the study visits

summarized in SoA (Section 1.5.1). Returned study intervention should not be re-dispensed to the subjects.

Study drug will be double-blind. Neither the subject nor the study physician will know which study drug the subject is receiving. The investigator or treating physician may unblind a subject's treatment assignment **only in the case of an emergency** when knowledge of the study treatment is essential for the appropriate clinical management or welfare of the subject. Whenever possible, the investigator must first discuss options with the Medical Monitor or appropriate sponsor study personnel before unblinding the subject's treatment assignment. If this is impractical, the investigator must notify the sponsor as soon as possible, but without revealing the treatment assignment of the unblinded subject, unless that information is important for the safety of subjects currently in the study. The date and reason for the unblinding must be recorded in the appropriate data collection tool.

Sponsor clinical safety and pharmacovigilance staff may unblind the treatment assignment for any subject with an SAE. If the SAE requires that an expedited regulatory report be sent to one or more regulatory agencies, a copy of the report, identifying the subject's treatment assignment, may be sent to clinical investigators in accordance with local regulations and/or sponsor policy.

The primary reason for treatment discontinuation (the event or condition that led to the unblinding) will be recorded in the eCRF. Subject may continue in the follow-up period of the study once the treatment code is unblinded (see Section 7.1 Discontinuation of Study Intervention).

Subjects will receive study drugs directly from the investigator or designee, under medical supervision. The date and time of each dose administered in the clinic will be recorded in the source documents and recorded in the eCRF. The dose of study drug and study subject identification will be confirmed at the time of dosing by a member of the study site staff other than the person administering the study drug.

6.4. Concomitant Therapy

All subjects from the Everest Project are required to receive NrtI therapy for ≥ 18 months and PEG-IFN-α for 24 to 26 weeks prior to the Day 1 visit. Investigators should remind patients the importance of taking NrtI daily unless their NrtI treatment is discontinued.

Any medications that the subject is receiving at the time of enrollment or receives during the study must be recorded along with:

- Reason for use
- Dates of administration including start and end dates
- Dosage information including dose and frequency

Should subjects have a need to initiate treatment with any excluded concomitant medication, the Medical Monitor must be consulted prior to initiation of the new

medication. In instances where an excluded medication is initiated prior to discussion with the Sponsor, the investigator must notify the Sponsor as soon as he/she is aware of the use of the excluded medication. The Medical Monitor should be contacted if there are any questions regarding concomitant or prior therapy.

6.4.1. Medication Adherence with NrtI and PEG-IFN-α

Every attempt should be made to ensure good adherence with NrtI and PEG-IFN-α therapy. Adherence to NrtI and PEG-IFN-α should be assessed at every relevant visit. Adherence of peg-IFN-α will be assessed by counting of empty containers and unused medication. Subjects will be instructed to bring all empty containers of PEG-IFN-α to the trial site at every visit, as applicable, for adherence assessment. Adherence of NrtI will be assessed diary cards for recording that are provided to subjects.

Subjects who have prematurely discontinued NrtI or PEG-IFN- α should be encouraged to continue with study participation.

6.4.2. Permitted Medications

The following medications/treatments are permitted:

- Hormone replacement therapy.
- Oral, injectable, subdermal, intravaginal, or implantable contraceptives, as well as intrauterine device, and intrauterine hormone-releasing system are permitted for contraception.
- Inhaled or topical steroids are allowed.
- Prescription drugs necessary to treat hypertension. Hypertension must be well controlled on a stable dose of medication for > 6 months prior to study drug administration at Day 1 visit.
- Prescription drugs necessary to treat diabetes mellitus. Diabetes mellitus must be controlled on a stable dose of medication for > 6 months prior to study drug administration at Day 1 visit.
- Paracetamol (≤ 2 g/day), aspirin (≤ 3 g/day), or ibuprofen (< 1.2 g/day).
- Antihistamines.
- Prescription inhaler or nebulizer to treat asthma (inhaled bronchodilator or inhaled steroid). Asthma must be well controlled, defined as historical use of a rescue bronchodilator on average no more than twice per week.
- A stable dose and regimen of other medication(s) (prescription or OTC) that the subject is taking regularly (i.e. medications for chronic conditions such as high cholesterol or depression must be unchanged in dose or type for at least 6 months) is permitted.
- Licensed SARS-CoV-2 vaccines, including those approved for emergency use by local authorities, for the prophylaxis of COVID-19 are allowed. Each COVID-19 vaccine administration should be ideally with a minimum interval of 14 days

before or after BRII-179 (VBI-2601) administration. However, COVID-19 vaccines may be administered within a shorter period in situations to avoid barriers or delays to COVID-19 vaccination.

6.4.3. Prohibited Medications

The following medications are excluded while subjects are participating in the study.

Prior to study enrolment and throughout the duration of study participation:

- Any HBV vaccine (licensed or experimental)

Within 30 days before study drug administration at the Day 1 visit and throughout the duration of study participation:

- Immunosuppressant (including but not limited to corticosteroids at a prednisolone equivalent dose > 20 mg/day)
- Immunization with attenuated vaccines (e.g., Mumps, Measles, Rubella), except COVID vaccines
- Any investigational drug or biologic other than the study product(s) used during this study
- Granulocyte-macrophage colony-stimulating factor (GM-CSF) or erythropoietin (EPO)
- Any OTC medicines or herbal remedies that may cause fluctuations of liver test results

Within 90 days before study drug administration at the Day 1 visit and throughout the duration of study participation:

- Blood products or immunoglobin

6.5. Safety Follow-up and Management of Reactions to Study Drug

The following safety observation procedures will be performed immediately following study drug administration and during the follow-up period for all subjects:

- Subjects will remain in the clinic for at least one hour post study drug administration. The observation period will include an assessment for rare adverse reactions.
- Any unusual signs or symptoms reported during the initial one hour of observation will prompt continued close monitoring.
- Based on their condition, subjects may be asked to remain in the clinic for more than one hour after the study drug administration (reason will be recorded in source data).

- All data (including assessment of solicited local and systemic reactions) will be recorded in the source document during and after the post-observation period.

A measurement device template (in mm) for measuring solicited local reactions (erythema [redness] and swelling), and an oral thermometer for measuring daily temperature, will be provided at the first study drug administration visit, study Day 1. Subjects will be advised to record their temperature in the diary card.

Refer to Sections 8.3 and 10.3 for further details on the recording, assessment, and reporting of AEs.

6.6. Treatment of Study Intervention Overdose

An overdose is defined as an accidental or intentional administration of a quantity of a medicinal product given per administration or cumulatively which is above the maximum recommended dose as per protocol which results in clinical signs and symptoms. These should be recorded by the investigator on the AE/SAE pages. In the event of an overdose of study medication, the investigator should use clinical judgement in treating the overdose and contact the study medical monitor.

The Sponsor is not recommending specific treatment guidelines for overdose and toxicity management. The investigator is advised to refer to the relevant document(s) for detailed information pertaining to the study drug being used in this study. Such documents may include, but not be limited to, the IB or equivalent document provided by the Sponsor.

6.7. Intervention after the End of the Study

The Sponsor will not provide post-study treatment. The investigator is responsible for ensuring that consideration has been given to the post-study care of the patient’s medical condition.

7. DISCONTINUATION OF STUDY INTERVENTION AND SUBJECT DISCONTINUATION/WITHDRAWAL

7.1. Discontinuation of Study Intervention

If a subject discontinues study dosing (for example, as a result of meeting criteria for discontinuation of study intervention described in Section 7.1.1), every attempt should be made to keep the subject in the study and continue to perform the required study-related visits and procedures for the remaining duration of study participation in the Dosing Period and Follow-up Period.

Subjects who have discontinued study drug may be reassessed for possible resumption of study drug dosing not to exceed the protocol defined number of doses, dosing interval, or dosing period duration. Subjects who have discontinued study drug will be assessed for NrtI discontinuation as described in Section 4.1.2.1 upon continued study participation into the Follow-up Period.

If continued study participation is not possible or acceptable to the subject or investigator, please refer to Section 7.2 Subject Discontinuation/Withdrawal from the Study.

7.1.1. Criteria for Discontinuation of Study Intervention

A subject who has received at least one dose of BRII-179 (VBI-2601) will have additional doses withheld if any of the following criteria are met:

- Signs or symptoms of severe (NCI-CTCAE Grade 3 or higher) hypersensitivity or anaphylaxis
- Significant abnormalities in liver chemistry, defined as any of the following are met:
    - Serum ALT > 10 × ULN
    - Serum ALT > 5 × ULN, with no change in HBsAg, defined as a < 50% decrease from the baseline predose value
    - Serum ALT or AST > 3 × ULN with a concomitant total bilirubin > 2 × ULN or INR > 1.5
- Any clinical manifestations of hepatic decompensation

The Sponsor may request DSMB evaluation of specific data in the event of liver flare to fully characterize transaminase elevations. The subject may receive remaining doses of study drug only after DSMB approval.

7.2. Subject Discontinuation/Withdrawal from the Study

- A subject may withdraw from the study at any time at his/her own request or may be withdrawn at any time at the discretion of the investigator for safety, behavioral, compliance, or administrative reasons. This is expected to be uncommon.
- At the time of discontinuing from the study, if possible, an Early Termination Visit should be performed (Section 8.1.7) for data to be collected at the time of study

discontinuation and follow-up. The subject will be permanently discontinued from both the study intervention and from the study at that time.

- If the subject withdraws consent for disclosure of future information, the sponsor may retain and continue to use any data collected before such a withdrawal of consent.
- If a subject withdraws from the study, he/she may request destruction of any samples taken and not tested, and the investigator must document this in the site study records.
- Subjects who are withdrawn from the study will NOT be replaced and cannot be rescreened.
- The primary reason for subject withdrawal will be recorded in the eCRF. Primary reasons for withdrawal will be categorized as:
    - Adverse event
    - Protocol deviation
    - Withdrew consent, not due to an AE
    - Lost to follow up
    - Study closed/terminated

7.2.1. Lost to Follow up

A subject will be considered lost to follow-up if he or she repeatedly fails to return for scheduled visits and is unable to be contacted by the study site.

The following actions must be taken if a subject fails to return to the clinic for a required study visit:

- The site must attempt to contact the subject and reschedule the missed visit as soon as possible and counsel the subject on the importance of maintaining the assigned visit schedule and ascertain whether or not the subject wishes to and/or should continue in the study.
- Before a subject is deemed lost to follow up, the investigator or designee must make every effort to regain contact with the subject (where possible, 3 telephone calls and, if necessary, a certified letter to the subject's last known mailing address or local equivalent methods). These contact attempts should be documented in the subject's medical record.
- Should the subject continue to be unreachable, only then will he/she be considered to have withdrawn from the study with a primary reason of "Lost to Follow-up".

Discontinuation of specific sites or of the study as a whole are handled as part of Section 10.1.7, Appendix 1.

8. STUDY ASSESSMENTS AND PROCEDURES

8.1. Schedule of Assessments

Study procedures and their timing are summarized in the SoAs (Section 1.5). Adherence to the study design requirements, including those specified in the SoAs, is essential and required for study conduct. Protocol waivers or exemptions are not allowed. Immediate safety concerns should be discussed with the sponsor immediately upon occurrence or awareness to determine if the subject should continue or discontinue study drug.

8.1.1. Screening

Written informed consent must be obtained prior to initiation of any study procedures. All screening evaluations must be completed and reviewed to confirm that potential subjects meet all eligibility criteria. The investigator will maintain a screening log to record details of all subjects screened and to confirm eligibility or record reasons for screening failure.

Screening will be performed no more than 2 weeks prior to randomization and will include written consent, determination of subject eligibility, collection of demographics and medical history/ current medical conditions as well as a physical examination, vital signs, 12-lead ECG, clinical laboratory tests, and other assessments per the SoA (Section 1.5.1).

Adverse events related to screening activities must be collected from the time of consent until the end of the study; any other events reported during the screening period should be reported as medical history. All SAEs must be collected from the time of consent until end of study.

8.1.2. Randomization (Day 1)

Eligible subjects will be randomized 1:1 to either 40 µg of BRII-179 administrated IM or matching placebo administrated IM. Subjects will receive their first dose of study drug on Day 1, however subject eligibility must be confirmed prior to study drug administration and blood sample collections will be made prior to study drug administration. Other assessments to be conducted on Day 1 are detailed in the SoA (Section 1.5.1).

8.1.3. Dosing Period

All subjects will receive BRII-179 (VBI-2601) or placebo administered IM every 4 weeks for a total of 9 doses. Subjects will discontinue PEG-IFN- α at Week 24; subjects will complete investigational treatment with BRII-179 or placebo at Week 32; while NrtI therapy will continue into the Follow-up Period.

Subjects will return to the investigative site on an outpatient basis for antiviral activity monitoring and safety assessment as specified in the SoA (Section 1.5.1).

Prior to study drug administration, there will be a review of ongoing eligibility to receive the study drug and continue with study participation. Diary cards will be collected and

there will be a review of any local and systemic reactions. Blood samples will be collected prior to each study drug administration.

8.1.4. Follow-up Period

Subjects will return to the investigative site for the specified assessments per the SoA (Section 1.5.1), including but not limited to physical examination, vital signs, clinical laboratory tests, and review of adverse events and concomitant medications.

Subjects will be assessed by the investigator whether they are eligible for discontinuation of NrtI therapy starting at Week 36 and potentially monthly until Week 48. Subjects that have NOT achieved NrtI discontinuation criteria by Week 48 will be discharged from the study.

8.1.5. NrtI Discontinuation Monitoring Period

Eligible subjects who meet NrtI withdrawal criteria will be informed by the investigator to discontinue their NrtI medications on the first day of their scheduled Day 1 visit of the NrtI Discontinuation Monitoring Period. These subjects will follow the NrtI Discontinuation Monitoring Period SoA (Section 1.5.2).

Subjects who discontinue from their NrtI therapy will be followed in the NrtI Discontinuation Monitoring Period for an additional 48 weeks from the day of NrtI discontinuation. Subjects who are retreated with NrtI therapy should continue to be followed per SoA (Section 1.5.2) for the duration of the 48-week NrtI Discontinuation Monitoring Period.

8.1.6. Unscheduled Visits

Subjects may be asked to come for an unscheduled visit for clinical assessment of an adverse event if warranted, as determined by the investigator. The unscheduled visit will be recorded in the eCRF.

8.1.7. Early Termination Visit

In cases where a subject prematurely discontinues from the study, the investigator must make every effort to have the subject return to the clinic within 14 days for an Early Termination (ET) Visit. Assessments for ET visits will include but not limited to physical examination, pregnancy test, clinical laboratory tests, and other assessments per the SoA (Section 1.5.1).

A follow Up Visit will be conducted approximately 4 weeks after the last dose of study drug. This will be required only if the subject has ongoing AEs or lab abnormalities at the last on-study visit. This visit may be conducted by telephone.

8.2. Detailed Description of Study Procedures

8.2.1. Medical History

A complete medical history, including details regarding illnesses and allergies, date(s) of onset, and whether condition(s) is currently ongoing (current medical conditions), medication history, including prior HBV treatment history and HBV genotype history (if available), will be collected on all subjects during screening and should be updated prior to dosing at the Day 1 visit.

8.2.2. Physical Examinations

A complete physical examination will include, at a minimum, general appearance, head, neck, chest/respiratory, heart/cardiovascular, gastrointestinal/liver, extremities, skin, and neurological assessments. Height and weight will also be measured and recorded. Body mass index will be calculated from height and weight.

A symptom-directed physical examination will be performed according to investigator discretion.

8.2.3. Fibroscan

To exclude the presence of cirrhosis, subjects will have a FibroScan evaluation, unless the subject has results from a FibroScan evaluation performed within 6 months prior to screening or liver biopsy performed within 1 year prior to screening that confirm the absence of Metavir F3 fibrosis or F4 cirrhosis. If a subject has had both procedures in the specified timeframes, the most recent result should be used to determine eligibility.

A FibroScan will also be conducted at the Week 48/ ET Visit in the NrtI Discontinuation Monitoring Period.

8.2.4. Alcohol Intake Assessment

Alcohol intake during the study will be recorded.

8.2.5. Vital Signs

Temperature (oral preferred), pulse rate, respiratory rate, and blood pressure will be assessed after the subject has rested comfortably for approximately 5 minutes. Pulse rate and blood pressure measurements will be assessed with a completely automated device. Manual techniques will be used only if an automated device is not available. Blood pressure should be based on 2 separate measurements. When scheduled at the same timepoints, vital signs and 12-lead ECG must be assessed first before physical examinations, blood sample collections, and urine collection.

8.2.6. Electrocardiograms

12-lead ECGs will be recorded and reviewed on-site by the investigator as outlined in SoA (Section 1.5.1).

8.2.7. Laboratory Assessments

8.2.7.1. Screening Viral Serology

The screening viral serology parameters will include chronic HBV infection and active infection with HIV, HCV, and hepatitis Delta virus. Chronic HBV infection is defined as serum HBsAg > 6 months.

8.2.7.2. Clinical Safety Laboratory Assessments

Fasting clinical safety laboratory tests to be performed are described in Section 10.2.

The investigator must review the laboratory report, document this review, and record any clinically significant changes occurring during the study in the AE section of the eCRF. The laboratory reports must be filed with the source documents. Clinically significant abnormal laboratory findings are those which are not associated with the underlying disease, unless judged by the investigator to be more severe than expected for the subject's condition.

In the event of an unexplained clinically relevant abnormal laboratory test occurring after study drug administration, the test should be repeated and followed at investigator discretion until it has returned to normal or baseline, and/or a diagnosis is made to adequately explain the abnormality.

8.2.7.3. Pregnancy Testing

A pregnancy test or confirmation of post-menopausal status must be confirmed for all female subjects. Post-menopausal status is defined as 12 months with no menses without alternative medical cause. Pregnancy tests will be performed for WOCBP only. Serum and urine pregnancy testing will be performed per the SoAs (Section 1.5) and any time pregnancy is suspected. A WOCBP who is known to be pregnant or who does not have a negative pregnancy test at screening is not eligible for study participation. During the study, the results of these pregnancy tests must be known prior to study drug administration. A WOCBP determined to be pregnant while on study will be followed until the pregnancy outcome is known, as described in Section 8.3.5.1.

8.2.7.4. Assessment of Antiviral Activity

Assessment of antiviral activity will include HBsAg, anti-HBs, and HBV DNA.

Details regarding the processing, shipping, and analysis of the samples will be provided in the Laboratory Manual.

8.2.8. Laboratory Events of Clinical Interest

A clinical laboratory event of clinical interest (ECI) is a laboratory value of scientific and medical interest specific to understanding of the investigational product and may require additional follow-up testing, close monitoring, and rapid communication by the investigator to the Sponsor.

During NrtI Discontinuation Monitoring Period, any laboratory events of ECI will be monitored, defined as the following:

- ALT > 3x ULN, if Day 1 predose ALT ≤ ULN
- ALT > 2x baseline, if Day 1 predose ALT > ULN

All laboratory ECIs must be reported to the Sponsor within 24 hours of the investigator's knowledge of the event. See Section 10.3.5. In addition, the assessments outlined in Section 10.5 should be performed as soon as possible and no later than two weeks after the initial ALT value meeting criteria for a laboratory ECI.

The laboratory ECI will also be evaluated by the investigator to determine if the event meets criteria for an AE. If AE criteria are met, all clinical sequelae in relation to these laboratory ECIs will be reported as AEs or SAEs, if applicable.

8.3. Adverse Events and Serious Adverse Events

The investigator and any qualified designees are responsible for detecting, documenting, and recording events that meet the definition of an AE or SAE and remain responsible for following up AEs that are serious, considered related to the study drug or study procedures, or that caused the subject to discontinue the study drug or study.

The definitions and procedures for reporting, recording, evaluating, and follow-up of an AE or SAE can be found in Section 10.3, Appendix 3.

8.3.1. Time Period and Frequency for Collecting AE and SAE Information

All SAEs will be collected from the signing of the informed consent form (ICF) until the last follow-up visit at the time points specified in the SoAs (Section 1.5).

All AE related to screening activities will be collected from the signing the ICF until the last follow-up visit at the time points specified in the SoAs (Section 1.5).

Medical occurrences that begin before the start of study drug but after obtaining informed consent will be recorded as medical history/ current medical conditions and not as an AE.

All SAEs will be recorded and reported to the sponsor or designee immediately and under no circumstance should this exceed 24 hours, as indicated in Section 10.3, Appendix 3. The investigator will submit any updated SAE data to the sponsor within 24 hours of it being available.

Investigators are not obligated to actively seek AEs or SAEs in former study subjects. However, if the investigator learns of any SAE, including a death, at any time after a subject has been discharged from the study, and he/she considers the event to be reasonably related to the study drug or study participation, the investigator must promptly notify the sponsor.

8.3.2. Method of Detecting AEs and SAEs

The method of recording, evaluating, and assessing causality of AEs and SAEs and the procedures for completing and transmitting SAE reports are provided in Section 10.3.

Care will be taken not to introduce bias when detecting AEs and/or SAEs. Open-ended and non-leading verbal questioning of the subject is the preferred method to inquire about AE occurrences.

8.3.3. Follow-up of AEs and SAEs

After the initial AE/SAE report, the investigator is required to proactively follow each subject at subsequent visits/contacts. All SAEs will be followed until resolution, until the condition stabilizes, until the event is otherwise explained, or until the subject is lost to follow-up (as defined in Section 7.2). Further information on follow-up procedures is provided in Section 10.3, Appendix 3.

8.3.4. Regulatory Reporting Requirements for SAEs

- Prompt notification by the investigator to the sponsor of a SAE is essential so that legal obligations and ethical responsibilities towards the safety of subjects and the safety of a study drug under clinical investigation are met.
- The sponsor has a legal responsibility to notify both the local regulatory authority and other regulatory agencies about the safety of a study drug under clinical investigation. The sponsor will comply with country-specific regulatory requirements relating to safety reporting to the regulatory authority, Institutional Review Boards (IRB)/Independent Ethics Committees (IEC), and investigators.
- For all studies except those utilizing medical devices investigator safety reports must be prepared for suspected unexpected serious adverse reactions (SUSAR) according to local regulatory requirements and sponsor policy and forwarded to investigators as necessary.
- An investigator who receives an investigator safety report describing a SAE or other specific safety information (e.g., summary or listing of SAEs) from the sponsor will review and then file it along with the Investigator’s Brochure and will notify the IRB/IEC, if appropriate according to local requirements.

8.3.5. Special Situations

Special situation reports include all reports of medication error, abuse, misuse, overdose, reports of adverse events associated with product complaints, and pregnancy reports regardless of an associated AE.

Medication error is any unintentional error in the prescribing, dispensing, or administration of a medicinal product while in the control of the health care provider, subject, or consumer.

Abuse is defined as persistent or sporadic intentional excessive use of a medicinal product by a subject.

Misuse is defined as any intentional and inappropriate use of a medicinal product that is not in accordance with the protocol instructions or the local prescribing information.

An overdose is defined as an accidental or intentional administration of a quantity of a medicinal product given per administration or cumulatively which is above the maximum recommended dose as per protocol which results in clinical signs and symptoms . In cases of a discrepancy in drug accountability, overdose will be established only when it is clear that the subject has taken the excess dose(s). Overdose cannot be established when the pharmacist cannot account for the discrepancy except in cases in which the investigator has reason to suspect that the additional dose(s) were administered to the subject.

Product complaint is defined as complaints arising from potential deviations in the manufacture, packaging, or distribution of the medicinal product.

8.3.5.1. Pregnancy

- Details of all pregnancies in female subjects will be collected after the start of study drug through 12 weeks after last study drug administration.
- If a pregnancy is reported, the investigator should inform the sponsor within 24 hours of learning of the pregnancy and should follow the procedures outlined in Section 10.4.
- Abnormal pregnancy outcomes (e.g., spontaneous abortion, fetal death, stillbirth, congenital anomalies, ectopic pregnancy) are considered SAEs.

8.3.5.2. Other Special Situations

- All other special situations must be reported to the sponsor within 24 hours of the investigator becoming aware of the situation. These reports must consist of situations that involve study drug and/or protocol-required concomitant medications, but do not apply to non-required concomitant medications.
- Special situations involving non-required concomitant medications do not need to be reported; however, for special situations that result in AEs due to a non-required concomitant medication, the AE should be reported.

- Any inappropriate use *of* concomitant medications prohibited by this protocol should not be reported as "misuse," but may be more appropriately documented as a protocol deviation.
- All clinical sequelae in relation to these special situation reports will be reported as AEs or SAEs at the same time. Details of the symptoms and signs, clinical management, and outcome will be reported, when available.

8.4. Pharmacodynamics

Pharmacodynamic parameters are not evaluated in this study.

8.5. Genetics

Human genetics are not evaluated in this study.

8.6. [Health Economics] OR [Medical Resource Utilization and Health Economics]

Health Economics/Medical Resource Utilization and Health Economics parameters are not evaluated in this study.

9. STATISTICAL CONSIDERATIONS

9.1. Statistical Hypotheses

9.1.1. Phase 2 Period

The primary endpoint for the Phase 2 period is HBsAg loss at Week 24. The null hypothesis is that there is no difference in the percentage of HBsAg loss at Week 24 between BRII-179 + PEG-IFN-α + NrtI and BRII-179 placebo + PEG-IFN- α +NrtI groups.

9.1.2. Phase 3 Period

The primary endpoint for the Phase 3 period is sustained HBsAg and HBV DNA loss (defined as HBsAg and HBV DNA undetectable for at least 24 weeks) after NrtI withdrawal. The null hypothesis is that there is no difference in the percentage of sustained HBsAg and HBV DNA loss between BRII-179+PEG-IFN- α + NrtI and BRII-179 placebo + PEG-IFN- α + NrtI groups.

9.2. Sample Size Determination

9.2.1. Phase 2 Period

The early futility analysis based on an intermediate outcome of efficacy using the HBsAg loss at Week 24, when about 106 subjects complete 24 weeks of BRII-179 or placebo in combination with PEG-IFN- α and NrtI treatment, will be conducted.

A sample size of 106 evaluable subjects provides 95% power to detect a difference of 20% in the percentage of HBsAg loss at Week 24 at the alpha level of 0.3 (1-sided), where 20% is considered as a clinically meaningful increase in HBsAg loss following adding BRII-179 to PEG-IFN- α and NrtI treatment. Everest project data suggest 24 weeks treatment of PEG-IFN- α + NrtI led to 26% of subjects with HBsAg loss (personal communication). In this sample size estimation, power calculation method is normal approximation and test method is Z-test (PASS, Version 15.0).

9.2.2. Phase 3 Period

Currently, there are limited data available regarding sustained HBsAg and HBV DNA loss (defined as HBsAg and HBV DNA undetectable for at least 24 weeks) after 24 weeks treatment of PEG-IFN- α + NrtI. Table 1 displays a range of sample size for sustained HBsAg and HBV DNA loss with PEG-IFN-α + NrtI treatment ranging from 22% to 35% and a minimum of clinically meaningful increase of 15% after adding BRII-179 to PEG-IFN- α + NrtI treatment.

Table 1. Sample Size Calculation for Four Scenarios of Sustained HBsAg Loss

Expected Sustained HBsAg and HBV DNA Loss (%)		Total Sample Size (Per Arm)	Power	Alpha (1-sided)
Placebo + PEG-IFN- α + NrtI	BRII-179 + PEG-IFN-α + NrtI			
22	37	420 (210)	90%	0.025
26	41	452 (226)		
**30**	**45**	**480 (240)**		
35	50	492 (246)		

A total sample size of 480 subjects, including 106 subjects from Phase 2 period, provides 90% power to detect an increase of 15% in sustained HBsAg and HBV DNA loss, using a 1-sided type I error rate of 2.5%, with the following assumptions:

Proportion of sustained HBsAg and HBV DNA loss in PEG-IFN- α + NrtI group is 30%.

A clinically meaningful increase in sustained HBsAg loss is 15% with add-on of BRII-179 treatment.

Non-binding stopping guideline for futility using a moderately aggressive Type II error spending function, implemented using the Lan-DeMets O'Brien-Fleming approximation spending function approach.

Two groups will be compared using Cochran-Mantel-Haenszel test, stratified at randomization by screening HBsAg level (3 categories: ≥0.05 to < 1 IU/mL, ≥1 to < 10 IU/mL, and ≥10 to <100 IU/mL).

Sample size calculation was conducted using Group Sequential Tests for Two Proportions module in PASS (Version 15.0), with a plan of 3 interim looks.

The sample size for Phase 3 period may be re-estimated based on a review of blinded, pooled HBsAg and HBV DNA loss rate at Week 24 after all subjects enrolled in Phase 2 complete 24 weeks follow-up.

9.3. Data Monitoring Guidelines for an Independent DSMB

An independent DSMB will review interim data and use pre-specified guidelines for early evidence of sufficient activity of BRII-179 that justifies continuing enrolment. This assessment will be made by the DSMB when Week 24 HBsAg and HBV DNA data for approximately 106 subjects are available.

If the trial advances to Phase 3, the DSMB will continue to review interim data on a regular basis and use pre-specified guidelines to determine clear evidence of efficacy for the primary outcome, and if so recommend unblinding of the trial results. Conversely, the

DSMB may recommend discontinuation of BRII-179 if futility assessments indicate that there is low probability that BRII-179 will achieve statistical significance for the primary endpoint of sustained HBsAg loss.

9.4. Rationale for Early Futility Analysis

The early futility analysis based on the intermediate outcome of efficacy using the HBsAg loss at Week 24 will be carried out for approximately 106 subjects completing 24 weeks treatment. The exact number may vary depending on the rate of enrolment and the timing of the DSMB meeting. This early futility analysis is based on Week 24 outcomes because too few primary endpoints of sustained HBsAg and HBV DNA loss expected when 106 subjects with only 24 weeks of follow-up are available. The sample size of 106 subjects provides 95% power to detect a difference of 20% in HBsAg loss (BRII-179 - placebo) at the 0.30 (1-sided) level of significance.

The rationale for a 1-sided alpha level of 0.30 and power 0.95 is based on previous work for 2-stage cancer trials, where like the current trial an intermediate outcome was used to assess early activity, and a definitive outcome was used to assess efficacy at the end of the trial. A re-analysis of 4 trials suggested a 1-sided significance level between 0.2 and 0.3 was optimal for making a good decision in the early futility assessment (Barthel, 2009). A subsequent paper focused on the potential for estimation bias in selected and stopped treatments and concluded that its degree was generally small (Choodari-Oskooei, 2013).

9.5. Interim Analyses Guidelines

Stopping guidelines for use by the DSMB are described below. More specific guidance may be specified in the SAP. Stopping boundaries will be derived to allow for 3 interim looks. The DSMB will be asked to recommend early termination or modification only when there is clear and substantial evidence of a treatment difference.

9.5.1. Early Assessment of Futility

When approximately 106 subjects have been enrolled and followed for 24 weeks, a futility assessment will be carried out. The criterion that will be used for the early futility assessment is that if BRII-179 is superior (i.e., 1-sided $p < 0.3$) to placebo in achieving HBsAg loss, then enrollment of the trial will expand to complete the phase 3 trial.

9.5.2. Monitoring Guidelines for the Primary Endpoint in Phase 3 Period

As a guideline, stopping boundaries will be provided to the DSMB to monitor the primary endpoint (sustained HBsAg and HBV DNA loss). For monitoring overwhelming benefit, the Lan-DeMets spending function analogue of the O'Brien-Fleming boundaries will be used. The Lan-DeMets boundary used will be chosen to preserve a 1-sided 0.025 level of significance. For computing the Lan-DeMets boundary, the information fraction at each of 3 interim analyses will be the number of subjects enrolled at the interim analysis, i.e., 120, 240, and 360 subjects completing 48 weeks of follow-up for those who

do not enter NrtI withdrawal follow-up period or 72 weeks of follow-up for those who enter NrtI withdrawal follow-up period, respectively.

Futility analyses will be carried out. The aim of these analyses will be to consider whether BRII-179 should be discontinued due to a low probability of achieving statistical significance for the primary endpoint of sustained HBsAg loss. Conditional power calculations of sustained HBsAg loss will be presented. In the futility analysis, the treatment effect for the future, as yet unobserved follow-up will be assumed as hypothesized in the study design (difference = 15%). Typical futility guidelines recommend stopping a trial when conditional power is below 10%-15%, with the higher value later in follow-up as measured by information time. These analyses will be presented to the DSMB by the unblinded statisticians.

9.6. Analysis Sets

The following analysis sets are defined:

Population	Description
All Subjects Screened	All subjects, for whom a record exists on the clinical database, will be included in All Subjects Screened.<br>It will be used for reporting study disposition, inclusion and exclusion criteria deviations, etc.
All Subjects Randomized	All subjects, who are randomized will be included in All Subjects Randomized. It will be used for reporting demographics, baseline characteristics, medical history, etc.
Full Analysis Set	All subjects, who are randomized and received at least one dose of BRII-179 or placebo, will be included in Full Analysis. It will be used as the primary analysis set of all efficacy endpoints.
Per Protocol Set	All subjects, who are randomized; and not identified with major protocol deviation that substantially affects the treatment effect of study medication; received all planned doses of BRII-179 or placebo; will be included in Per Protocol Set. It will be used as analysis set for key efficacy endpoint analyses. The analysis based on PP analysis set will be considered as sensitivity analysis.
Safety Analysis Set	All subjects randomly assigned to study drug and who take at least 1 dose of study drug will be included in Safety Analysis Set. It will be used for reporting safety parameters.

9.7. Statistical Analyses

This section outlines the general analytic strategy for the study. The statistical analysis plan (SAP) will be finalized prior to Database Lock (DBL) and it will include a more detailed description of the statistical analyses described in this section. This section is a

summary of the planned statistical analyses of the most important endpoints including primary and key secondary endpoints.

9.7.1. General considerations

Unless otherwise stated, the following rule will apply:

Summaries and displays will be presented by treatment group.

For categorical variables, the frequency and percentage will be applied as descriptive statistics.

For continuous variables, the mean, standard variation, median, inter-quartiles, and ranges will be applied as descriptive statistics.

Specific algorithm regarding how missing HBsAg and HBV DNA values will be handled in deriving the primary efficacy endpoints and sustained HBsAg loss will be detailed in the statistical analysis plan.

Unless specified otherwise, missing values will be handled using the Data-As-Observed (DAO) approach, that is, any missing value will be excluded from the analysis, except for the baseline value, which will be replaced with the latest pre-treatment result, if available.

Only scheduled visits will be presented of summaries; unscheduled visits will be listed but will not be summarized.

9.7.2. Analysis of the Primary Efficacy Endpoint for Phase 2

For evaluating graduation criterion (ie, expanding the study to Phase 3 period), the primary efficacy endpoint of the phase 2 trial, percentage of HBsAg loss at Week 24 (from Day 155 to Day 182), will be analysed based on Cochran-Mantel-Haenszel method, stratified at randomization by screening HBsAg level (3 categories). The differences in the percentage of HBsAg loss and associated 95% confidence interval will be presented. If one sided p-value is < 0.3, the trial will continue to Phase 3. Subjects with missing HBsAg value at Week 24 will be considered as not achieving HBsAg loss at Week 24.

9.7.3. Analysis of the Primary Efficacy Endpoint for Phase 3

The primary efficacy endpoint of the phase 3 trial, percentage of sustained HBsAg and HBV DNA loss defined as undetectable HBsAg and HBV DNA for at least 24 weeks after NrtI withdrawal. Subjects who do not meet NrtI withdrawal criteria or are retreated with NrtI after NrtI withdrawal will be classified as not achieving sustained HBsAg and HBV DNA loss. The primary endpoint will be analyzed based on Cochran-Mantel-Haenszel method, stratified at randomization by screening HBsAg level (3 categories: ≥0.05 to < 1 IU/mL, ≥1 to < 10 IU/mL, and ≥10 to <100 IU/mL). The differences in the percentage of sustained HBsAg loss and associated 95% confidence interval will be presented.

9.7.4. Analyses of Secondary Efficacy Endpoints and Subgroups

Percentage of subjects meeting NrtI withdrawal criteria and other secondary endpoints assessed as proportion will be analysed in the same way as for the primary endpoint.

Time to event endpoints will be compared using stratified log-rank test and continuous endpoints will be compared using Wilcoxon ranks sum test.

Additionally, subgroup analysis by age, gender, and HBsAg level at baseline (3 categories: >0.05 to < 1 IU/mL, ≥1 to < 10 IU/mL, and ≥10 to <100 IU/mL) will be conducted with respect to the primary endpoint.

9.7.5. Analysis of Safety Outcome

The Safety Analysis population will be used for clinical review of safety relevant parameters.

All AEs will be coded using the Medical Dictionary for Regulatory Activities (MedDRA). Treatment-Emergent Adverse Events (TEAEs) are defined as any AEs with an onset date of on or after study drug start date and no later than 30 days after permanent discontinuation of study drug. Number and percentage of TEAEs will be summarized by cohort.

Graded laboratory abnormalities will be defined using NCI-CTCAE Version 5.0 grading scale. The incidence of treatment-emergent laboratory abnormalities, defined as values that increase at least 1 toxicity grade from baseline at any postbaseline timepoint up to 30 days after permanent discontinuation of study drug will be summarized by cohort.

10. SUPPORTING DOCUMENTATION AND OPERATIONAL CONSIDERATIONS

10.1. Appendix 1: Regulatory, Ethical, and Study Oversight Considerations

10.1.1. Regulatory and Ethical Considerations

- This study will be conducted in accordance with the protocol and with the following:
  - Consensus ethical principles derived from international guidelines including the Declaration of Helsinki and Council for International Organizations of Medical Sciences (CIOMS) International Ethical Guidelines
  - Applicable ICH Good Clinical Practice (GCP) Guidelines
  - Applicable laws and regulations
- The protocol, protocol amendments, ICF, Investigator Brochure, and other relevant documents (e.g., advertisements) must be submitted to an IRB/IEC by the investigator and reviewed and approved by the IRB/IEC before the study is initiated.
- Any amendments to the protocol will require IRB/IEC approval before implementation of changes made to the study design, except for changes necessary to eliminate an immediate hazard to study subjects.
- The investigator will be responsible for the following:
  - Providing written summaries of the status of the study to the IRB/IEC annually or more frequently in accordance with the requirements, policies, and procedures established by the IRB/IEC
  - Notifying the IRB/IEC of SAEs or other significant safety findings as required by IRB/IEC procedures
  - Providing oversight of the conduct of the study at the site and adherence to requirements of 21 CFR, ICH guidelines, the IRB/IEC, European regulation 536/2014 for clinical studies (if applicable), and all other applicable local regulations

10.1.2. Financial Disclosure

Investigators and sub-investigators will provide the sponsor with sufficient, accurate financial information as requested to allow the sponsor to submit complete and accurate financial certification or disclosure statements to the appropriate regulatory authorities. Investigators are responsible for providing information on financial interests during the course of the study and for 1 year after completion of the study.

10.1.3. Informed Consent Process

- The investigator or his/her representative will explain the nature of the study to the subject or his/her legally authorized representative and answer all questions regarding the study.

- Subjects must be informed that their participation is voluntary. Subjects or their legally authorized representative will be required to sign a statement of informed consent that meets the requirements of 21 CFR 50, local regulations, ICH guidelines, Health Insurance Portability and Accountability Act (HIPAA) requirements, where applicable, and the IRB/IEC or study center.
- The medical record must include a statement that written informed consent was obtained before the subject was enrolled in the study and the date the written consent was obtained. The authorized person obtaining the informed consent must also sign the ICF.
- Subjects must be re-consented to the most current version of the ICF(s) during their participation in the study.
- A copy of the ICF(s) must be provided to the subject or the subject's legally authorized representative.

10.1.4. Data Protection

- Subjects will be assigned a unique identifier by the sponsor. Any subject records or datasets that are transferred to the sponsor will contain the identifier only; subject names or any information which would make the subject identifiable will not be transferred.
- The subject must be informed that his/her personal study-related data will be used by the sponsor in accordance with local data protection law. The level of disclosure must also be explained to the subject who will be required to give consent for their data to be used as described in the informed consent
- The subject must be informed that his/her medical records may be examined by Clinical Quality Assurance auditors or other authorized personnel appointed by the sponsor, by appropriate IRB/IEC members, and by inspectors from regulatory authorities.

10.1.5. Data Quality Assurance

- All relevant subject data relating to the study will be recorded on printed or electronic CRF unless transmitted to the sponsor or designee electronically (e.g., laboratory data). The investigator is responsible for verifying that data entries are accurate and correct by physically or electronically signing the CRF.
- The investigator must maintain accurate documentation (source data) that supports the information entered in the CRF.
- The investigator must permit study-related monitoring, audits, IRB/IEC review, and regulatory agency inspections and provide direct access to source data documents.
- Monitoring details describing strategy (e.g., risk-based initiatives in operations and quality such as Risk Management and Mitigation Strategies and Analytical Risk-Based Monitoring), methods, responsibilities and requirements, including handling

of noncompliance issues and monitoring techniques (central, remote, or on-site monitoring) are provided in the Monitoring Plan.

- The sponsor or designee is responsible for the data management of this study including quality checking of the data.
- The sponsor assumes accountability for actions delegated to other individuals (e.g., Contract Research Organizations).
- Study monitors will perform ongoing source data verification to confirm that data entered into the CRF by authorized site personnel are accurate, complete, and verifiable from source documents; that the safety and rights of subjects are being protected; and that the study is being conducted in accordance with the currently approved protocol and any other study agreements, ICH GCP, and all applicable regulatory requirements.
- Records and documents, including signed ICFs, pertaining to the conduct of this study must be retained by the investigator. No records may be destroyed during the retention period without the written approval of the sponsor. No records may be transferred to another location or party without written notification to the sponsor.

10.1.6. Source Documents

- Source documents provide evidence for the existence of the subject and substantiate the integrity of the data collected. Source documents are filed at the investigator’s site.
- Data reported on the CRF or entered in the eCRF that are transcribed from source documents must be consistent with the source documents or the discrepancies must be explained. The investigator may need to request previous medical records or transfer records, depending on the study. Also, current medical records must be available.
- Source data refer to all information recorded in the original record of certified copy of the clinical study, including clinical findings, observation results and other relevant activity records needed for reconstruction and evaluation of the clinical study.

10.1.7. Study and Site Start and Closure

The study start date is the date on which the clinical study will be open for recruitment of subjects.

The sponsor designee reserves the right to close the study site or terminate the study at any time for any reason at the sole discretion of the sponsor. Study sites will be closed upon study completion. A study site is considered closed when all required documents and study supplies have been collected and a study-site closure visit has been performed.

The investigator may initiate study-site closure at any time, provided there is reasonable cause and sufficient notice is given in advance of the intended termination.

Reasons for the early closure of a study site by the sponsor or investigator may include but are not limited to:

- Failure of the investigator to comply with the protocol, the requirements of the IRB/IEC or local health authorities, the sponsor's procedures, or GCP guidelines
- Inadequate recruitment of subjects by the investigator
- Discontinuation of further study drug development

If the study is prematurely terminated or suspended, the sponsor shall promptly inform the Investigators, the IECs/IRBs, the regulatory authorities, and any contract research organization(s) used in the study of the reason for termination or suspension, as specified by the applicable regulatory requirements. The Investigator shall promptly inform the subject and should assure appropriate subject therapy and/or follow-up.

10.1.8. Publication Policy

The sponsor intends to publish the results of clinical trials regardless of outcome. Results may be published in peer-reviewed journals and/or presented at scientific congresses. The sponsor will comply with the requirements for publication and disclosure of clinical trial information. In accordance with standard editorial and ethical practice, the sponsor will generally support publication of multicenter studies only in their entirety and not as individual site data. Authorship will be determined by mutual agreement based upon International Committee of Medical Journal Editors criteria and guidelines [ICMJE, 2018]. Individuals who do not meet authorship criteria do not warrant named authorship. Nonauthor contributors may be acknowledged in the manuscript when published or recognized in a manner consistent with the target journal policy.

10.2. Appendix 2: Clinical Laboratory Tests

The tests detailed in Table 2 will be performed by the central laboratory. Urine pregnancy test will be performed by the local or central laboratory.

Investigators must document their review of each laboratory safety report.

Additional tests may be performed at any time during the study as determined necessary by the investigator or required by local regulations.

Table 2: Protocol-Required Safety Laboratory Assessments

**Hematology**	
Complete blood count with differential, including red blood cell count, hemoglobin, hematocrit, white blood cell count, count and percentage of neutrophils, lymphocytes, eosinophils, basophils, and monocytes, and platelet count	
**Chemistry**	
Albumin	Estimated glomerular filtration rate (eGFR)
Blood urea nitrogen (BUN)	Gamma glutamyl transferase (GGT)
Calcium	Glucose
Carbon dioxide/bicarbonate	Lactate dehydrogenase (LDH)
Chloride	Potassium
Creatine kinase	Sodium
Creatinine	Uric acid
**Liver Function Tests**	
Alkaline phosphatase (ALP)	Aspartate aminotransferase (AST)
Alanine aminotransferase (ALT)	Bilirubin (total and direct)
**Coagulation Parameters**	
International normalized ratio (INR)	Prothrombin time (PT)
**Urinalysis**	
Glucose	Proteins
Red blood cells (RBCs)	
**Pregnancy Testing**	
Beta-human chorionic gonadotropin (WOCBP only)	Urine pregnancy test
**Thyroid Panel**	
Thyroid stimulating hormone (TSH)[1]	

[1] Thyroid function will be tested every 12 weeks during PEG-IFN-α dosing: Screening, Week 12, and Week 24.

10.3. Appendix 3: Adverse Events: Definitions and Procedures for Recording, Evaluating, Follow-up, and Reporting

10.3.1. Definition of AE

**AE Definition**
• An AE is any untoward medical occurrence in a patient or clinical study subject, temporally associated with the use of study drug, whether or not considered related to the study drug.<br>• NOTE: An AE can therefore be any unfavorable and unintended sign (including an abnormal laboratory finding), symptom, or disease (new or exacerbated) temporally associated with the use of study drug.

**Events Meeting the AE Definition**
• Any abnormal laboratory test results (hematology, clinical chemistry, or urinalysis) or other safety assessments (e.g., ECG, radiological scans, vital signs measurements), including those that worsen from baseline, considered clinically significant in the medical and scientific judgment of the investigator (i.e., not related to progression of underlying disease).<br>• Exacerbation of a chronic or intermittent pre-existing condition including either an increase in frequency and/or intensity of the condition.<br>• New conditions detected or diagnosed after study drug administration even though it may have been present before the start of the study.<br>• Signs, symptoms, or the clinical sequelae of a suspected drug-drug interaction.<br>• Signs, symptoms, or the clinical sequelae of a suspected overdose of either study drug or a concomitant medication. Overdose per se will not be reported as an AE/SAE unless it is an intentional overdose taken with possible suicidal/self-harming intent. Such overdoses should be reported regardless of sequelae.

**Events NOT Meeting the AE Definition**
• Any clinically significant abnormal laboratory findings or other abnormal safety assessments which are associated with the underlying disease, unless judged by the investigator to be more severe than expected for the subject's condition.<br>• The disease/disorder being studied or expected progression, signs, or symptoms of the disease/disorder being studied, unless more severe than expected for the subject's condition.

- Medical or surgical procedure (e.g., endoscopy, appendectomy): the condition that leads to the procedure is the AE.
- Situations in which an untoward medical occurrence did not occur (social and/or convenience admission to a hospital).
- Anticipated day-to-day fluctuations of pre-existing disease(s) or condition(s) present or detected at the start of the study that do not worsen.

10.3.2. Definition of SAE

If an event is not an AE per definition above, then it cannot be an SAE even if serious conditions are met (e.g., hospitalization for signs/symptoms of the disease under study, death due to progression of disease).

**A SAE is defined as any untoward medical occurrence that, at any dose:**
**a. Results in death**
**b. Is life-threatening**<br>The term 'life-threatening' in the definition of 'serious' refers to an event in which the subject was at risk of death at the time of the event. It does not refer to an event, which hypothetically might have caused death, if it were more severe.
**c. Requires inpatient hospitalization or prolongation of existing hospitalization**<br>• In general, hospitalization signifies that the subject has been detained (usually involving at least an overnight stay) at the hospital or emergency ward for observation and/or treatment that would not have been appropriate in the physician's office or outpatient setting. Complications that occur during hospitalization are AEs. If a complication prolongs hospitalization or fulfills any other serious criteria, the event is serious. When in doubt as to whether "hospitalization" occurred or was necessary, the AE should be considered serious.<br>• Hospitalization for elective treatment of a pre-existing condition that did not worsen from baseline is not considered an AE.
**d. Results in persistent disability/incapacity**<br>• The term disability means a substantial disruption of a person's ability to conduct normal life functions.<br>• This definition is not intended to include experiences of relatively minor medical significance such as uncomplicated headache, nausea, vomiting, diarrhea, influenza, and accidental trauma (e.g., sprained ankle) which may interfere with or prevent everyday life functions but do not constitute a substantial disruption.

**e. Is a congenital anomaly/birth defect**
**f. Other situations:**<br>• Medical or scientific judgment should be exercised in deciding whether SAE reporting is appropriate in other situations such as important medical events that may not be immediately life-threatening or result in death or hospitalization but may jeopardize the subject or may require medical or surgical intervention to prevent one of the other outcomes listed in the above definition. These events should usually be considered serious.<br>• Examples of such events include invasive or malignant cancers, intensive treatment in an emergency room or at home for allergic bronchospasm, blood dyscrasias or convulsions that do not result in hospitalization, or development of drug dependency or drug abuse.

10.3.3. Recording and Follow-Up of AE and/or SAE

**AE and SAE Recording**
• When an AE/SAE occurs, it is the responsibility of the investigator to review all documentation (e.g., hospital progress notes, laboratory reports, and diagnostics reports) related to the event.<br>• The investigator will then record all relevant AE/SAE information in the CRF.<br>• It is **not** acceptable for the investigator to send photocopies of the subject's medical records to the Sponsor in lieu of completion of the AE/SAE CRF page.<br>• There may be instances when copies of medical records for certain cases are requested by the Sponsor. In this case, all subject identifiers, with the exception of the subject number, will be redacted on the copies of the medical records before submission to the Sponsor.<br>• The investigator will attempt to establish a diagnosis of the event based on signs, symptoms, and/or other clinical information. Whenever possible, the diagnosis (not the individual signs/symptoms) will be documented as the AE/SAE.
**Assessment of Intensity**
The investigator will make an assessment of intensity for each AE and SAE reported during the study based on the National Cancer Institute Common Terminology Criteria for Adverse Events (NCI-CTCAE) Version 5.0.
**Assessment of Causality**
• The investigator or a qualified assistant investigator is obligated to assess the relationship between study drug and each occurrence of each AE/SAE.<br>• Relationship between AEs and the study drug: "related" should be entered when there is either (a) a *reasonable possibility* that the AE/SAE is associated

with study drug **or** (b) no reasonable alternative explanation can be found. Otherwise, the relationship between AEs and the study drug should be classified as "unrelated".

- A "reasonable possibility" of a relationship conveys that there are facts, evidence, and/or arguments to suggest a causal relationship, rather than a relationship cannot be ruled out.
- The investigator will use clinical judgment to determine the relationship.
- Alternative causes, such as underlying disease(s), concomitant therapy, and other risk factors, as well as the temporal relationship of the event to study drug administration will be considered and investigated.
- The investigator will also consult the Investigator's Brochure (IB) and/or Product Information, for marketed products, in his/her assessment.
- For each AE/SAE, the investigator **must** document in the medical notes that he/she has reviewed the AE/SAE and has provided an assessment of causality.
- There may be situations in which an SAE has occurred and the investigator has minimal information to include in the initial report. However, it is very important that the investigator always make an assessment of causality for every event before the initial transmission of the SAE data.
- The investigator may change his/her opinion of causality in light of follow-up information and send a SAE follow-up report with the updated causality assessment.
- The causality assessment is one of the criteria used when determining regulatory reporting requirements.

Follow-up of AEs and SAEs

- The investigator is obligated to perform or arrange for the conduct of supplemental measurements and/or evaluations as medically indicated or as requested by the Sponsor to elucidate the nature and/or causality of the AE or SAE as fully as possible. This may include additional laboratory tests or investigations, histopathological examinations, or consultation with other health care professionals.
- If a subject dies during participation in the study or during a recognized follow-up period, the investigator will provide the Sponsor with a copy of any post-mortem findings including histopathology.
- New or updated information will be recorded in the originally completed CRF.
- The investigator will submit any updated SAE data to the Sponsor within 24 hours of receipt of the information.

10.3.4. Reporting of SAEs

SAE Reporting to the Sponsor via a Paper SAE Reporting Form
• The investigator must complete a paper SAE report form and send to the Sponsor or its representative within 24 hours of the investigator's knowledge of the event.<br>• Contacts for SAE reporting can be found on the SAE Reporting Form.

10.3.5. Reporting of Laboratory Events of Clinical Interest

All laboratory ECIs must be reported to the Sponsor or its representative within 24 hours of the investigator's knowledge of the event. Contact for laboratory ECIs reporting can be found on the ECI Reporting Form.

The laboratory ECI will also be evaluated by the investigator to determine if the event meets criteria for an AE. If AE criteria are met, all clinical sequelae in relation to these laboratory ECIs will be reported as AEs or SAEs, if applicable.

10.4. Appendix 4: Contraceptive Requirements and Collection and Reporting of Pregnancy Information

Contraceptive Requirements:

WOCBP may be included in this study and include any female subject who has experienced menarche and who is not post-menopausal or permanently sterilized (eg, bilateral tubal occlusion, hysterectomy, or bilateral salpingectomy).

WOCBP must be willing to use highly effective methods of contraception 14 days before dose, throughout study participation, and for 12 weeks after last dose of study drug administration. Highly effective methods of birth control result in a low failure rate (ie, less than 1% per year). Birth control methods which are considered highly effective include:

- Established use of combined (estrogen and progestogen containing) oral, intravaginal, or transdermal hormonal methods of contraception associated with inhibition of ovulation **OR** established use of progestogen-only oral, injectable, or implantable hormonal methods of contraception associated with inhibition of ovulation. It is not currently known whether study drug will impact the effectiveness of hormonal contraceptive methods; therefore, it is recommended to use an additional form of contraception (ie, barrier method) throughout the study and for 12 weeks after last study drug administration.
- Placement of an intrauterine device
- Placement of an intrauterine hormone-releasing system
- Surgical sterilization of male partner (with the appropriate post-vasectomy documentation of the absence of sperm in the ejaculate; for female subjects on the study, the vasectomized male partner should be the sole partner for that subject)
- True sexual abstinence, when in line with the preferred and usual lifestyle of the subject. Periodic abstinence (e.g., calendar, ovulation, symptothermal, post-ovulation methods) and withdrawal are not acceptable methods of contraception. Abstinent subjects have to agree to use 1 of the above-mentioned contraceptive methods, if they start sexual relationships during the study and for up to 12 weeks after last dose of study drug administration, or for as long as the subject is followed on study, whichever is longer.
- Barrier method in combination with hormonal contraceptive, as described above

Post-menopausal status is defined as 12 months with no menses without an alternative medical cause.

Collection of Pregnancy Information:

Female Subjects who become pregnant

- The investigator will collect pregnancy information on any female subject who becomes pregnant while participating in this study. The initial information will be recorded on the appropriate form and submitted to the sponsor within 24 hours of learning of a subject's pregnancy.
- The subject will be followed to determine the outcome of the pregnancy. The investigator will collect follow-up information on the subject and the neonate and the information will be forwarded to the sponsor. Generally, follow-up will not be required for longer than 6 to 8 weeks beyond the estimated delivery date. Any termination of pregnancy will be reported, regardless of fetal status (presence or absence of anomalies) or indication for the procedure.
- While pregnancy itself is not considered to be an AE or SAE, any pregnancy complication or elective termination of a pregnancy for medical reasons will be reported as an AE or SAE.
- A spontaneous abortion (occurring at <22 weeks gestational age) or still birth (occurring at >22 weeks gestational age) is always considered to be an SAE and will be reported as such.
- Any post-study pregnancy related SAE considered reasonably related to the study drug by the investigator will be reported to the sponsor as described in Section 8.3.4. While the investigator is not obligated to actively seek this information in former study subjects, he or she may learn of an SAE through spontaneous reporting.
- Any female subject who becomes pregnant while participating in the study will discontinue study drug.

10.5. Appendix 5: Assessments in Subjects Meeting Laboratory ECI Criteria

Additional assessment will be performed in subjects who experience ALT elevations meeting laboratory ECI criteria (Section 8.2.8) during the NrtI Discontinuation Monitoring Period. Following the occurrence of event of ECI, the following assessments in Table 3 will be performed one time, as soon as possible, and no later than two weeks after the initial ALT value meeting criteria for a laboratory ECI. The full panel of assessments should only be performed once; individual assessments may be repeated per investigator discretion, as needed.

Table 3 Assessments in Subjects Meeting Laboratory ECI Criteria

**Hematology**	
Complete blood count with differential, including red blood cell count, hemoglobin, hematocrit, white blood cell count, count and percentage of neutrophils, lymphocytes, eosinophils, basophils, and monocytes, and platelet count	
**Chemistry**	
Albumin	Estimated glomerular filtration rate (eGFR)
Blood urea nitrogen (BUN)	Gamma glutamyl transferase (GGT)
Calcium	Glucose
Carbon dioxide/bicarbonate	Lactate dehydrogenase (LDH)
Chloride	Potassium
Creatine kinase	Sodium
Creatinine	Uric acid
**Liver Function Tests**	
Alkaline phosphatase (ALP)	Aspartate aminotransferase (AST)
Alanine aminotransferase (ALT)	Bilirubin (total and direct)
**Coagulation Parameters**	
International normalized ratio (INR)	Prothrombin time (PT)
**Extended Hepatic Panel**	
Quantitative HBsAg	Ferritin
Quantitative HBV DNA	Aldolase
Quantitative HBeAg	Blood phosphatidylethanol
HAV antibody IgM	Urinary ethyglucuronide
HCV antibody and HCV RNA	Anti-nuclear antibodies
HDV antibody or RNA	Anti-smooth muscle antibodies
HEV antibody IgM	Anti-LKM1 antibody
HDV antibody or RNA	Anti-mitochondrial antibodies
Epstein-Barr Virus antibodies, IgM and IgG	Anti-SLA antibodies
Cytomegalovirus antibodies, IgM and IgG	
Ceruloplasmin	
**Imaging**	
Abdominal ultrasound, including Doppler flow, if applicable; CT or MRI acceptable in place of ultrasound if clinically indicated	
**Focused Medical History**	

Use of any potentially hepatotoxic concomitant medications, including over the counter medications and herbal remedies	Alcohol consumption and drugs of abuse
Exposure to other potentially hepatotoxic agents including any work-related exposures (eg. solvents, pesticides, etc.)	Exercise history

CT = computed tomography; HAV = hepatitis A virus; HCV = hepatitis C virus; HDV = hepatitis D virus; HEV = hepatitis E virus; IgG = immunoglobulin G antibody; IgM = immunoglobulin M antibody; LKM1 = liver/kidney microsome-1 antibody; MRI = magnetic resonance imaging; PCR = polymerase chain reaction; RNA = ribonucleic acid; SLA = soluble liver antigen.

11. REFERENCES

Barthel FM-S, Parmar MKB, Royston P. How do multi-stage, multi-arm trials compare to the traditional two-arm parallel group design – a reanalysis of 4 trials. Trials 2009; 10:21, doi:.10.1186/1745-6215-10-21.

Berg T, Simon KG, Mauss S, Schott E, Heyne R, Klass DM, Eisenbach C, Welzel TM, Zachoval R, Felten G, Schulze-Zur-Wiesch J, Cornberg M, Op den Brouw ML, Jump B, Reiser H, Gallo L, Warger T, Petersen J; FINITE CHB study investigators. Long-term response after stopping tenofovir disoproxil fumarate in non-cirrhotic HBeAg-negative patients - FINITE study. *Journal of Hepatology*. 2017 Nov;67(5):918-924.

Chang, M. L., Liaw, Y. F., and Hadziyannis, S. J. (2015). Systematic review: cessation of long-term nucleos(t)ide analogue therapy in patients with hepatitis B e antigen-negative chronic hepatitis B. *Alimentary Pharmacology & Therapeutics*, 2015; *42*(3): 243–257.

Choodari-Oskooei B, Parmar MKB, Royston P, Bowden J. Impact of lack-of-benefit stopping rules on treatment effect estimates of two-arm multi-stage (TAMS) trials with time to event outcome. Trials 2013, 14:23, http://www.trialsjournal.com/content/14/1/23.

Cornberg M, Lok AS, Terrault NA, Zoulim F, and the 2019 EASL-AASLD HBV Treatment Endpoints Conference Faculty. Guidance for design and endpoints of clinical trials in chronic hepatitis B – Report from the 2019 EASL-AASLD HBV Treatment Endpoints Conference. *Journal of Hepatology*. 2020; 72(3): 539-337.

Ferrari C. HBV and the immune response. *Liver Int*. 2015; 35 (Suppl. 1): 121–128.

Hoa PT, Huy NT, Thu le T, Nga CN, Nakao K, Eguchi K, Chi NH, Hoang BH, Hirayama K. Randomized controlled study investigating viral suppression and serological response following pre-S1/pre-S2/S vaccine therapy combined with lamivudine treatment in HBeAg-positive patients with chronic hepatitis B. *Antimicrob Agents Chemother*. 2009; 53:5134-40.

Jeng WJ, Chen YC, Chien RN, Sheen IS, Liaw YF. Incidence and predictors of HBsAg seroclearance after cessation of nucleos(t)ide analogue therapy in HBeAg negative chronic hepatitis B. *Hepatology*. 2018; 68(2): 425-434.

Kondo Y, Ninomiya M, Kakazu E, Kimura O, Shimosegawa T. Hepatitis B surface antigen could contribute to the immunopathogenesis of hepatitis B virus infection. *ISRN Gastroenterol*. 2013;2013:935295.

Konerman MA, Lok AS. Interferon Treatment for Hepatitis B. *Clinics in Liver Disease*. 2016; 20(4): 645-665.

Kramvis A. Genotypes and genetic variability of hepatitis B virus. *Intervirology* 2014; 57(3-4): 141-150.

Lebossé F, Testoni B, Fresquet J, Facchetti F, Galmozzi E, Fournier M, Hervieu V, Berthillon P, Berby F, Bordes I, Durantel D, Levrero M, Lampertico P, Zoulim F. Intrahepatic innate immune response pathways are downregulated in untreated chronic hepatitis B. *J Hepatol*. 2017;66(5):897-909.

Lok AS, McMahon BJ, Brown RS, Wong JB, Ahmed AT, Farah W, Almasri J, Alahdab F, Benkhadra K, Mouchli MA, Singh S, Mohamed EA, Abu Dabrh AM, Prokop LJ, Wang Z, Murad MH, Mohammed K. Antiviral therapy for chronic hepatitis B viral infection in adults: A systematic review and meta-analysis. *Hepatology* 2016; 63(1):284-306.

Marcellin P, Ahn SH, Ma X, Caruntu FA, Tak WY, Elkashab M, et al. Combination of tenofovir disoproxil fumarate and peginterferon α-2a increases loss of hepatitis B surface antigen in patients with chronic hepatitis B. Gastroenterology 2016;150(1):134–44.

Michler T, Kosinska AD, Festag J, Bunse T, Su J, Ringelhan M, Imhof H, Grimm D, Steiger K, Mogler C, Heikenwalder M, Michel ML, Guzman CA, Milstein S, Sepp-Lorenzino L, Knolle P, Protzer U. Knockdown of Virus Antigen Expression Increases Therapeutic Vaccine Efficacy in High-Titer Hepatitis B Virus Carrier Mice. Gastroenterology. 2020 May;158(6):1762-1775.

Papatheodoridi M, Hadziyannis E, Berby F, Zachou K, Testoni B, Rigopoulou E, Gatselis NK, Lyberopoulou A, Vlachogiannakos I, Manolakopoulos S, Dalekos GN, Zoulim F, Papatheodoridis GV. Predictors of hepatitis B surface antigen loss, relapse and retreatment after discontinuation of effective oral antiviral therapy in noncirrhotic HBeAg-negative chronic hepatitis B. *J Viral Hepat.* 2020; 27(2): 118-126.

Perrillo, R. "Benefits and risks of IFN therapy for hepatitis B." *Hepatology.* 2009; 49(5): S103-111.

Ren H, Hu P, Chen XY, Gong GZ, Shang J, Zhang WH, et al. Switching to PegIFNα-2a in NUC treated CHB patients(NEW SWITCH Study)： comparison 48 and 96 weeks. Hepatol Int 2016;10(Suppl 1):S43–4.

Roggendorf H, Krawczyk A, Lindemann M, Shouval D, Michler T, Roggendorf M, Gerken G. Induction of functional control in chronic hepatitis B patients with low level HBsAg using a combination of a PreS1/S2/S HBV vaccine (Sci-B-Vac®) and a nucleoside analogue. *J Infect Dis Ther*. 2019; 7:389-393.

Schweitzer A, Horn J, Mikolajczyk RT, Krause G, Ott JJ. Estimations of worldwide prevalence of chronic hepatitis B virus infection: a systematic review of data published between 1965 and 2013. *Lancet* 2015; 386(10003): 1546-1555.

Stanaway JD, Flaxman AD, Naghavi M, Fitzmaurice C, Vos T, Abubakar I, Abu-Raddad LJ, Assadi R, Bhala N, Cowie B, Forouzanfour MH, Groeger J, Hanafiah KM, Jacobsen KH, James SL, MacLachlan J, Malekzadeh R, Martin NK, Mokdad AA, Mokdad AH, Murray CJL, Plass D, Rana S, Rein DB, Richardus JH, Sanabria J, Saylan M, Shahraz S, So S, Vlassov V, Weiderpass E, Wiersma ST, Younis M, Yu C, Zaki M, Cooke GS. The

global burden of viral hepatitis from 1990 to 2013: findings from the Global Burden of Disease Study 2013. *Lancet* 2016; 388(10049): 1081-1088.

Trepo CHL, Chan, Lok A. Hepatitis B virus infection. *Lancet* 2014;384(9959): 2053-2063.

van Bömmel F, Berg T. Stopping long-term treatment with nucleos(t)ide analogues is a favourable option for selected patients with HBeAg-negative chronic hepatitis B. *Liver International*. 2018;38 Suppl 1:90-96.

Wang S, Chen Z, Hu C, Qian F, Cheng Y, Wu M, Shi B, Chen J, Hu Y, Yuan Z. Hepatitis B virus surface antigen selectively inhibits TLR2 ligand-induced IL-12 production in monocytes/macrophages by interfering with JNK activation. *J Immunol*. 2013 May 15;190(10):5142-51.

Wong TCL, Fung JYY, Chok KSH, Cheung TT, Chan ACY, Dai WC, Ng KKC, Chan SC, Lo, CM. Hepatitis B vaccination in patients receiving oral antiviral therapy without hepatitis B immunoglobulin after liver transplant. *Transplant Proc*. 2018; 50:3681-3688.

Yao CC, Hung CH, Hu TH, Lu SN, Wang JH, Lee CM, and Chen CH. Incidence and predictors of HBV relapse after cessation of nucleoside analogues in HBeAg-negative patients with HBsAg ≤ 200 IU/mL. *Scientific Reports*. 2017; 7(1): 1839.

Ze-Qian Wu, Dong-Ying Xie, Lei Fu, Jia Wei, Jia Shang, Qing He, Wen-hua Zhang, Guang-yu Huang, Yanzhong Peng, Ye Gu, Jia-bin Li, Ying Guo, Yu-juan Guan, Jia-wei Geng, Huan-wei Zheng, Wen-jun Gao, Wen-jing Zhao, Ying-jun Tian, Yi-lan Zeng, Ren Chen, Shengwang Gu, Zhi-liang Gao. Functional cure based on pegylated interferon ? in long-term nucleoside analog suppressed HBeAg negative chronic hepatitis B: a multicenter real-world study (Everest project in China), an interim report. *Journal of Hepatology* 2020; 73: S653–S915.

Zhu D, Liu L, Yang D, Fu S, Bian Y, Sun Z, He J, Su L, Zhang L, Peng H, Fu YX. Clearing Persistent Extracellular Antigen of Hepatitis B Virus: An Immunomodulatory Strategy To Reverse Tolerance for an Effective Therapeutic Vaccination. *J Immunol*. 2016; 196(7): 3079-87.

Title

Protocol Title: An Adaptive Multicenter, Randomized, Double-Blind, Placebo-Controlled, Parallel-Group Study to Investigate the Efficacy and Safety of BRII-179 (VBI-2601) for the Treatment of Chronic Hepatitis B Virus (HBV) Infection

Protocol Number: BRII-179-002

Compound: BRII-179 (VBI-2601)

Study Phase: Phase 2/ Phase 3

Short Title: Short Title: Study to Investigate Efficacy and Safety of BRII-179 (VBI-2601) for the Treatment of Chronic HBV Infection

Sponsor: Brii Biosciences Limited

Co-Sponsor/ Registration Agent: Brii Biosciences (Beijing) Ltd.

Legal Registered Address: Room 301, 3/F, Building 7,
No. 1 Yongtaizhuang North Road，
Tiandilinfeng（Dongsheng Area）
Haidian District, Beijing 100192,
China

Protocol Version: Version 2.0

Approval Date: 16 Jun 2021

Sponsor Signatory

[redacted]

Medical Monitor Name and Contact Information will be provided separately.

INVESTIGATOR AGREEMENT PAGE

For protocol number BRII-179-002

I confirm agreement to conduct the study in compliance with the protocol, *International Conference on Harmonisation Good Clinical Practices (ICH GCP)* as well as applicable local regulatory and ethical requirements.

I acknowledge that I am responsible for overall study conduct. I agree to personally conduct or supervise the described clinical study.

I agree to ensure that all associates, colleagues and employees assisting in the conduct of the study are informed about their obligations. Mechanisms are in place to ensure that site staff receive the appropriate information throughout study.

Investigator Name: ____

____ ____________________________

Investigator Signature **Date** (dd/mm/yyyy)

DOCUMENT HISTORY

Document number, date of Issue and summary of changes:

Document	Date of Issue	Major Amendment
1.0	Apr 30th,2021	Primary version
2.0	Jun 16th,2021	Modfied the inclusion/exclision criteria,the criteria for discontinuation of study intervention and added the the safety evaluation for PEG-IFN-α

Protocol Amendment Explanation

This version’s amendment is based on the further discussion with investigators.

The summary of changes are as below table.

Bold text represents the added text and the ~~strikethrough~~ represents the deleted text in the blow amendment summary table.

**Section and Headings**	**Prior version (version 1.0, Apr 30th,2021)**	**Current version (version 2.0, Jun 16th,2021)**	**Rational**
1.5 Schedule of Activities (SoA)<br><br>Note m	If a subject experiences alanine aminotransferase (ALT) flare (as defined by ALT>2*ULN)	If a subject experiences alanine aminotransferase (ALT) flare (as defined by ALT > 2x ULN)	It's not consistent with the revised inclusion criteria, the criteria would be judged by investigator
4.1.3 PEG-IFN-α Therapy During the Dosing Period	Subjects entering the Dosing Period of the study will continue their peg-IFN-α treatment,	Subjects entering the Dosing Period of the study will continue their peg-IFN-α treatment, **investigator should monitor the related adverse events and take necessary medical treatment**	To emphasis the investigator should monitor the peg-IFN-α related adverse events and take necessary medical treatment
4.3.1.1 Dose Adjustment for adverse drug reactions	In patients with progressive or persistent ALT increases > 10 x ULN and >2 x baseline values	In patients with progressive or persistent ALT increases > 10 x ULN ~~and >2 x baseline values~~	To be consistent with inclusion/exclusion criteria, and the resumed therapy should be determined by investigator based on clinical judgement

	After peg-IFN-α dose reduction or withholding, therapy can be resumed after ALT flares subside	After peg-IFN-α dose reduction or withholding, therapy can be resumed after **improvement of liver function by investigator's judgment.**	
5.1 Inclusion Criteria<br><br>5.2 Exclusion Criteria	6.HBV DNA < 100 IU/mL at screening<br><br>10.Serum ALT and AST ≤ 2x ULN at screening.<br><br>8. including but not limited to corticosteroids at a prednisolone equivalent dose > 20 mg/day	6. HBV DNA < LLOQ at screening (central lab)<br><br>10.Serum ALT and AST ≤ **5**x ULN at screening.<br><br>8. including but not limited to corticosteroids at **⩾1 time** prednisolone equivalent dose > 20 mg/day	Optimize the inclusion and exclusion criteria
5.3 Lifestyle Considerations	Alcohol consumption is limited to no more than 1 unit per day (unit: 1 glass of wine [125 mL] = 1 measure of spirits [30 mL] = one-half pint of beer [284 mL]) during the participation of this study	Alcohol consumption is limited to no more than 1 unit per day (unit: wine [about 125 mL] = white spirit [about 30 mL] = beer [about 284 mL]) during the participation of this study.	Increase the accuracy of the description

7.1.1 Criteria for Discontinuation of Study Intervention	Serum ALT > 5 × ULN, with no change in HBsAg, defined as a < 50% decrease from the baseline predose value	~~Serum ALT > 5 × ULN, with no change in HBsAg, defined as a < 50% decrease from the baseline predose value~~	Optimize the criteria for discontinuation of study intervention
8.2.7.2 Clinical Safety Laboratory Assessments	Fasting clinical safety laboratory tests to be performed are described in Section 10.2.	Fasting clinical safety laboratory tests to be performed are described in Section 10.2.<br>**The investigator should conduct additional clinical assessment for the adverse effects of PEG-IFN-α, including but not limited to radiological examination, ophthalmology assessment, immunological marker test, blood sugar and psychological assessment.**	Added the safety evaluation for PEG-IFN- α

Table of Contents

1. PROTOCOL SUMMARY ... 12
1.1. Synopsis ... 12
1.2. Phase 2 (N=106) ... 12
1.3. Phase 3 (N=480) ... 13
1.4. Schema ... 16
1.5. Schedule of Activities (SoA) ... 17
1.5.1. BRII-179 (VBI-2601) 32-week Dosing Period and 16-week Follow-up Period ... 17
1.5.2. NrtI Discontinuation Monitoring Period ... 19

2. INTRODUCTION ... 20
2.1. Background ... 20
2.2. Study Rationale ... 21
2.3. Benefit/Risk Assessment ... 22

3. OBJECTIVES AND ENDPOINTS ... 23
3.1. Objectives ... 23
3.2. Endpoints ... 23
3.2.1. Primary Endpoints ... 23
3.2.2. Secondary Endpoints ... 23

4. STUDY DESIGN ... 25
4.1. Overall Design ... 25
4.1.1. Independent DSMB ... 25
4.1.2. NrtI Discontinuation ... 25
4.1.3. PEG-IFN-α Therapy During the Dosing Period ... 26
4.2. Scientific Rationale for Study Design ... 28
4.3. Justification for Dose ... 29
4.4. End of Study Definition ... 30

5. STUDY POPULATION ... 31
5.1. Inclusion Criteria ... 31
5.2. Exclusion Criteria ... 32
5.3. Lifestyle Considerations ... 34
5.4. Screen Failures ... 34

6. STUDY INTERVENTION ... 35
6.1. Study Intervention(s) Administered ... 35
6.1.1. Formulations ... 35
6.1.2. Dosage and Administration of BRII-179 and Placebo ... 35
6.2. Preparation/Handling/Storage/Accountability ... 36
6.3. Randomization and Blinding ... 36
6.4. Concomitant Therapy ... 37
6.4.1. Medication Adherence with NrtI and PEG-IFN-α ... 38
6.4.2. Permitted Medications ... 38
6.4.3. Prohibited Medications ... 39
6.5. Safety Follow-up and Management of Reactions to Study Drug ... 39

6.6. Treatment of Study Intervention Overdose 40
6.7. Intervention after the End of the Study 40

7. DISCONTINUATION OF STUDY INTERVENTION AND SUBJECT DISCONTINUATION/WITHDRAWAL 41
7.1. Discontinuation of Study Intervention 41
7.1.1. Criteria for Discontinuation of Study Intervention 41
7.2. Subject Discontinuation/Withdrawal from the Study 41
7.2.1. Lost to Follow up 42

8. STUDY ASSESSMENTS AND PROCEDURES 43
8.1. Schedule of Assessments 43
8.1.1. Screening 43
8.1.2. Randomization (Day 1) 43
8.1.3. Dosing Period 43
8.1.4. Follow-up Period 44
8.1.5. NrtI Discontinuation Monitoring Period 44
8.1.6. Unscheduled Visits 44
8.1.7. Early Termination Visit 44
8.2. Detailed Description of Study Procedures 45
8.2.1. Medical History 45
8.2.2. Physical Examinations 45
8.2.3. Fibroscan 45
8.2.4. Alcohol Intake Assessment 45
8.2.5. Vital Signs 45
8.2.6. Electrocardiograms 46
8.2.7. Laboratory Assessments 46
8.2.8. Laboratory Events of Clinical Interest 47
8.3. Adverse Events and Serious Adverse Events 47
8.3.1. Time Period and Frequency for Collecting AE and SAE Information 47
8.3.2. Method of Detecting AEs and SAEs 48
8.3.3. Follow-up of AEs and SAEs 48
8.3.4. Regulatory Reporting Requirements for SAEs 48
8.3.5. Special Situations 49
8.4. Pharmacodynamics 50
8.5. Genetics 50
8.6. [Health Economics] OR [Medical Resource Utilization and Health Economics] 50

9. STATISTICAL CONSIDERATIONS 51
9.1. Statistical Hypotheses 51
9.1.1. Phase 2 Period 51
9.1.2. Phase 3 Period 51
9.2. Sample Size Determination 51
9.2.1. Phase 2 Period 51
9.2.2. Phase 3 Period 51
9.3. Data Monitoring Guidelines for an Independent DSMB 52
9.4. Rationale for Early Futility Analysis 53
9.5. Interim Analyses Guidelines 53
9.5.1. Early Assessment of Futility 53

9.5.2. Monitoring Guidelines for the Primary Endpoint in Phase 3 Period53
9.6. Analysis Sets 54
9.7. Statistical Analyses 54
9.7.1. General considerations 55
9.7.2. Analysis of the Primary Efficacy Endpoint for Phase 2 55
9.7.3. Analysis of the Primary Efficacy Endpoint for Phase 3 55
9.7.4. Analyses of Secondary Efficacy Endpoints and Subgroups 56
9.7.5. Analysis of Safety Outcome 56

10. SUPPORTING DOCUMENTATION AND OPERATIONAL CONSIDERATIONS 57
10.1. Appendix 1: Regulatory, Ethical, and Study Oversight Considerations 57
10.1.1. Regulatory and Ethical Considerations 57
10.1.2. Financial Disclosure 57
10.1.3. Informed Consent Process 57
10.1.4. Data Protection 58
10.1.5. Data Quality Assurance 58
10.1.6. Source Documents 59
10.1.7. Study and Site Start and Closure 59
10.1.8. Publication Policy 60
10.2. Appendix 2: Clinical Laboratory Tests 61
10.3. Appendix 3: Adverse Events: Definitions and Procedures for Recording, Evaluating, Follow-up, and Reporting 62
10.3.1. Definition of AE 62
10.3.2. Definition of SAE 63
10.3.3. Recording and Follow-Up of AE and/or SAE 64
10.3.4. Reporting of SAEs 66
10.3.5. Reporting of Laboratory Events of Clinical Interest 66
10.4. Appendix 4: Contraceptive Requirements and Collection and Reporting of Pregnancy Information 67
10.5. Appendix 5: Assessments in Subjects Meeting Laboratory ECI Criteria 69

11. REFERENCES 71

ABBREVIATIONS

AE	adverse event
ALT	alanine aminotransferase
anti-HBs	antibodies to hepatitis B surface antigen
DNA	deoxyribonucleic acid
DSMB	Data and Safety Monitoring Board
ECG	electrocardiogram
ECI	events of clinical interest
EPO	erythropoietin
ET	early termination
GM-CSF	granulocyte-macrophage colony-stimulating factor
HBeAg	hepatitis B e-antigen
HBsAg	hepatitis B surface antigen
HBV	hepatitis B virus
HCV	hepatitis C virus
HDV	hepatitis D virus
HIV	human immunodeficiency virus
ICF	informed consent form
IFN-α	interferon-alpha
IMP	Investigational medicinal product
IU	international units
kPa	kilopascal
Kg	kilogram
LLOQ	lower limit of quantification
m	meter
µg	microgram
ml	milliliter
NCI-CTCAE	National Cancer Institute Common Terminology Criteria for Adverse Events
NIMP	Non-investigational medicinal product
NrtI	nucleos(t)ide reverse transcriptase inhibitors
ULN	upper limit of normal
PEG-IFN-α	pegylated interferon-alpha
PD	Pharmacodynamic
PK	pharmacokinetic
SoA	schedule of activities
SAE	serious adverse event
WOCBP	women of child-bearing potential

1. PROTOCOL SUMMARY

1.1. Synopsis

Protocol Title: An Adaptive Multicenter, Randomized, Double-Blind, Placebo-Controlled, Parallel-Group Study to Investigate the Efficacy and Safety of BRII-179 (VBI-2601) for the Treatment of Chronic Hepatitis B Virus (HBV) Infection

Rationale:
Multiple therapies with differential mechanisms of actions to treat hepatitis B virus (HBV) is an encouraging strategy to achieve higher rates of hepatitis B surface antigen (HBsAg) loss and ideally HBsAg seroconversion. The present study (BRII-179-002) will evaluate the efficacy and safety of adding a recombinant protein immunotherapeutic (BRII-179 [VBI-2601]) treatment regimen to existing pegylated interferon-alpha (PEG-IFN-α) and nucleos(t)ide reverse transcriptase inhibitor (NrtI) therapy in chronic HBV patients from the Everest Project (NCT04035837). The primary objective of the Everest Project is to achieve functional cure of HBV infection by optimizing treatment strategies of NrtIs and PEG-IFN-α.

Individualized regimens of IFN and NrtI therapies have shown promising efficacy in reducing HBsAg with some patients achieving HBsAg clearance (Ren, 2016; Marcellin, 2016). This result is more pronounced in patients on optimized HBV treatment to low HBsAg levels, which is the rationale of including subjects from the Everest Project in this study. Preclinical and clinical studies have also shown the benefit of low HBsAg levels for breaking tolerance with a therapeutic HBV vaccine (Zhu, 2016; Michler, 2020; Roggendorf, 2019). The recombinant protein immunotherapeutic, BRII-179 (VBI-2601), may break immunological tolerance and restore and/or augment adaptive T cell and antibody responses to the surface antigens. These responses together have the potential to better achieve a functional cure (sustained loss of HBsAg in blood with or without seroconversion to the surface antigen antibody) of chronic HBV infection.

Objectives and Endpoints

This adaptive study design will begin with a phase 2 evaluation of 106 subjects, followed by a transition into a larger phase 3 evaluation which will expand enrollment to include an additional 374 subjects to the study (N=480). The futility assessment performed in Phase 2 to determine graduation to Phase 3 will use the following criterion: BRII-179 is superior (i.e., 1-sided $p < 0.3$) to placebo in achieving HBsAg loss at Week 24.

1.2. Phase 2 (N=106)

Objectives	Endpoints
Primary	
To determine the efficacy of BRII-179 to increase HBsAg loss through Week 24	• Percentage of subjects with HBsAg loss at Week 24
Secondary	

To assess the antiviral activity of BRII-179 (VBI-2601) therapy added to PEG-IFN-α + NrtI	• Change from baseline of HBsAg from Day 1 to Week 24<br>• Percentage of subjects with HBsAg loss at any timepoint
To assess safety of BRII-179 (VBI-2601) therapy	• Percentage of subjects with adverse events (AEs) and serious adverse events (SAEs)<br>• Percentage of subjects with abnormalities in clinical laboratory tests

1.3. Phase 3 (N=480)

Objectives	**Endpoints**
Primary	
To evaluate functional cure of chronic HBV subjects receiving BRII-179 (VBI-2601) therapy added to PEG-IFN-α + NrtI	• Percentage of subjects with sustained HBsAg and HBV deoxyribonucleic acid (DNA) loss (defined as undetectable HBsAg and HBV DNA for at least 24 weeks) after NrtI withdrawal
Secondary	
To assess the antiviral activity of BRII-179 (VBI-2601) therapy added to PEG-IFN-α + NrtI	• Percentage of subjects with undetectable HBV-DNA (HBV DNA < Lower Limit of Quantification (LLOQ)) 24 and 48 weeks after NrtI withdrawal<br>• Percentage of subjects meeting the NrtI withdrawal criteria by end of follow-up period (Week 48)<br>• Percentage of subjects with HBsAg loss at any timepoint<br>• Percentage of subjects with HBsAg seroconversion at any timepoint<br>• Percentage of subjects with antibodies to hepatitis B surface antigen (anti-HBs) response by end of follow-up period (Week 48)<br>• Change from baseline of HBsAg from Day 1 to End of Treatment (Week 32)<br>• Time to achieve HBsAg seroclearance (HBsAg loss) and seroconversion<br>• Percentage of subjects requiring NrtI re-treatment during follow up
To assess safety of BRII-179 (VBI-2601) therapy	• Percentage of subjects with adverse events (AEs) and serious adverse events (SAEs)<br>• Percentage of subjects with abnormalities in clinical laboratory tests

Overall Design

Study BRII-179-002 is a double-blind, randomized, placebo-controlled, parallel-group study of adding BRII-179 (VBI-2601) to existing PEG-IFN-α and NrtI therapy in patients with chronic HBV infection without cirrhosis enrolled in the Everest Project study being conducted in China (NCT04035837).

The Everest Project is a multicenter real-world study being conducted in over 260 hospitals in China. The plan is to recruit 30,000 patients with chronic HBV effectively treated with NrtI therapy for at least one year; who are hepatitis B e-antigen (HBeAg) negative with HBV DNA <100 IU/ml and HBsAg <1500 IU/mL at study entry. As part of the study participation, subjects receive PEG-IFN-α as “add-on” or “switch to” treatment for 48 to 96 weeks. The primary objective of the Everest Project is to achieve functional cure of HBV infection by optimizing treatment strategies of NrtIs and PEG-IFN-α.

Eligible subjects from the Everest Project study, who have received PEG-IFN-α for 24 to 26 weeks and NrtI therapy for at least 18 months, will be randomized 1:1 to receive BRII-179 (VBI-2601) or placebo in addition to the existing PEG-IFN-α and NrtI therapy. Subjects will be stratified at randomization by screening HBsAg level (3 categories: ≥0.05 to < 1 IU/mL, ≥1 to < 10 IU/mL, and ≥10 to <100 IU/mL). Subjects will discontinue PEG-IFN- α at Week 24; subjects will complete investigational treatment with BRII-179 or placebo at Week 32; while NrtI therapy will continue into the Follow-up Period. The BRII-179-002 study design schema can be found in Section 1.4.

Estimated total duration for each subject will include the following: screening up to 14 days, 32-week dosing period, and up to a 16-week follow-up period. Eligible subjects that meet NrtI discontinuation criteria (Section 4.1.2.1) (eligibility assessed at Week 36 to Week 48) may enter a NrtI discontinuation monitoring period for an additional 48 weeks from the day of NrtI discontinuation (Section 1.5.2).

Number of Subjects:

A total of 480 evaluable adult subjects who have received PEG-IFN-α for 24 to 26 weeks and NrtI therapy for at least 18 months from the Everest Project are planned. The initial Phase 2 period will evaluate 106 subjects, followed by a transition into a larger phase 3 evaluation which will expand enrollment to include an additional 374 subjects to the study (N=480).

Intervention Groups and Duration:

Eligible subjects currently receiving PEG-IFN-α and NrtI therapy will receive the study drug BRII-179 (VBI-2601) or matching placebo.

Independent Data and Safety Monitoring Board (DSMB)

An independent DSMB will monitor patient safety and treatment efficacy data for this study. The details for the operation and responsibilities of the DSMB will be defined in a separate DSMB Charter. The Charter will delineate the composition, duties, responsibilities, and procedures of the DSMB, data required at each meeting, as well as the analyses that will be conducted.

Disclosure Statement: This is a phase 2/ phase 3, randomized, multicenter, double-blind, placebo-controlled, parallel-group study to evaluate the efficacy and safety of BRII-179 (VBI-2601) in patients with chronic HBV receiving PEG-IFN-α and NrtI therapy. All references to "Sponsor" contained herein shall refer to Brii Biosciences.

1.4. Schema

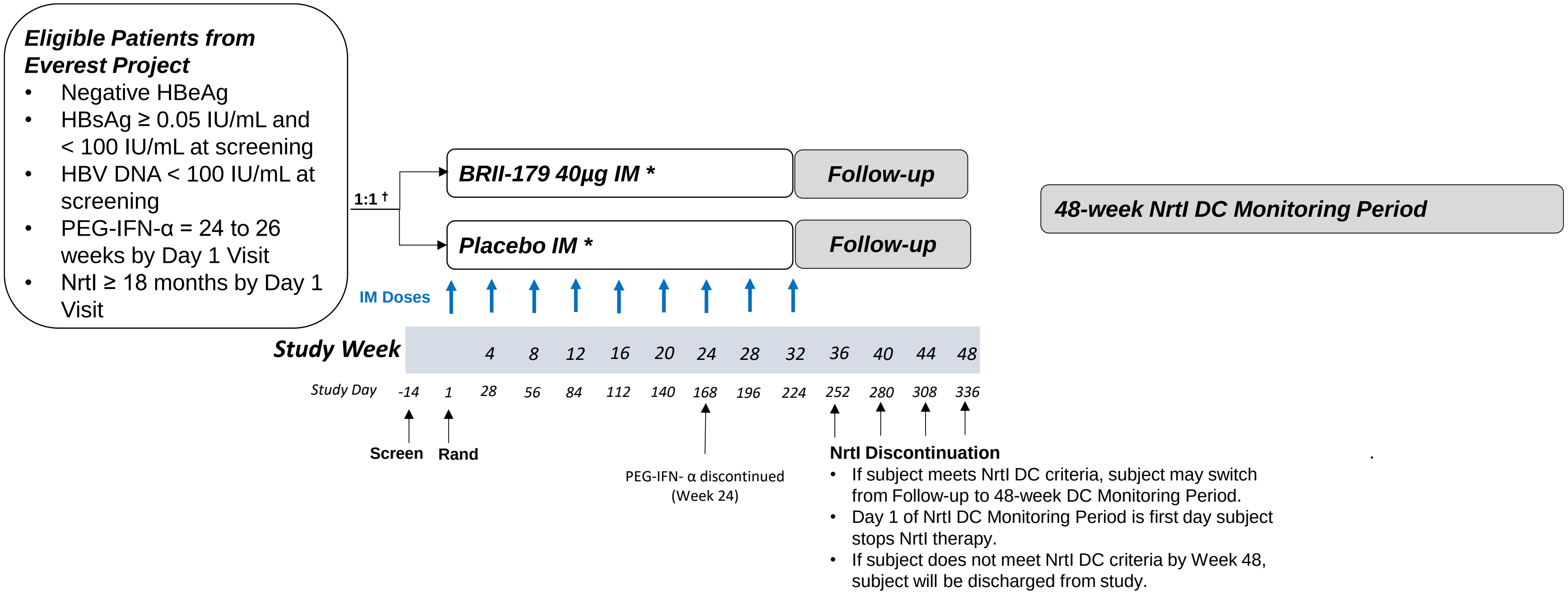

DC = discontinuation; IM = Intramuscular; Rand = Randomization

* Concomitant therapy of PEG-IFN-α and NrtI during dosing period; PEG-IFN- α discontinued at Week 24; NrtI therapy will be continued into the Follow-up Period.

† Subjects stratified by HBsAg level

1.5. Schedule of Activities (SoA)

1.5.1. BRII-179 (VBI-2601) 32-week Dosing Period and 16-week Follow-up Period

Study Stage	Screen	Dosing Period[b]									Follow-up Period			
**Study Visit Week**	Screen	Rand	W4	W8	W12	W16	W20	W24	W28	W32/ET	W36[c]	W40[c]	W44[c]	W48[c]/ET
**Study Visit Day ± Visit Window**	D -14 to -1	D1[a]	D28 ± 3	D56 ± 3	D84 ± 3	D112 ± 3	D140 ± 3	D168 ± 3	D196 ± 3	D224 ± 3	D252 ± 5	D280 ± 5	D308 ± 5	D336 ± 5
Informed consent form (ICF)	X													
Inclusion/exclusion criteria	X	X[d]												
Demography	X													
Medical history including current medical conditions and HBV genotype[e]	X													
Fibroscan	X													
Full physical exam[f]	X				X					X				X
Symptom-directed physical exam		X	X	X		X	X	X	X		X	X	X	
Alcohol intake assessment[g]		X	X	X	X	X	X	X	X	X	X	X	X	X
Vital signs[h]	X	X			X			X		X				
12-lead ECG[i]	X	X								X				
Subject instructed to complete diary		X	X	X	X	X	X	X	X	X	X	X	X	X
Urinalysis	X													
Pregnancy test[j]	X	X	X	X	X	X	X	X	X	X	X	X	X	X
Safety Laboratory Assessments	X	X	X	X	X	X	X	X	X	X	X	X	X	X
Randomization		X												
Study drug administration		X	X	X	X	X	X	X[k]	X	X[l]				
Screening viral serology	X													
Blood sample for HBsAg quantitative	X	X		X		X		X		X	X	X	X	X
Blood sample for anti-HBs quantitative	X	X				X		X		X		X		X
Blood sample for HBV DNA quantitative[m]	X	X				X		X		X	X	X	X	X
Adverse event assessment[n]	←------------------------X------------------------→													
Concomitant medications review	←------------------------X------------------------→													
NrtI medication adherence review	←------------------------X------------------------→													
PEG-IFN-α medication adherence review[o]	←--------------X--------------→													

AE = adverse event; anti-HBs = antibodies to hepatitis B surface antigen; HBsAg = hepatitis B surface antigen; ECG = electrocardiogram; ET = early termination; HBV = Hepatitis B Virus; NrtI = nucleoside/nucleotide reverse transcriptase inhibitor; ULN = Upper Limit of Normal

Note: When scheduled at the same timepoints, the assessments of vital signs and 12-lead ECGs must be performed before blood sample collections.

a Assessments performed predose unless otherwise specified.

[b] If a subject withdraws prematurely from the study prior to their Week 32 visit, ET assessments should be performed within 14 days. Follow Up Visit will be conducted approximately 4 weeks after the last dose of study drug; required only if the participant has ongoing AEs or lab abnormalities at the last on-study visit, and this visit may be conducted by telephone.

[c] Subjects that meet NrtI discontinuation criteria (Section 4.1.2.1) may discontinue from the Follow-up Period and enter into the NrtI Discontinuation Monitoring Period (Section 1.5.2). Day 1 of the NrtI Discontinuation Monitoring Period is the first day subject stops their NrtI therapy.

[d] Evaluation of inclusion and exclusion criteria related to pregnancy testing, medical history and concomitant medications prior to dosing.

[e] A complete medical history will be taken at screening including HBV genotype if available. Any changes should be updated prior to dosing.

[f] Height and weight will be recorded at screening. Body mass index will be calculated based on the measurements. Only weight will be collected afterwards.

[g] Subjects' alcohol intake will be recorded while on study.

[h] Vital signs (blood pressure, pulse rate, respiratory rate, and temperature) should be measured after the subject has rested comfortably for approximately 5 minutes.

[i] 12-lead safety ECGs should be measured in the supine position after the subject has rested comfortably for 10 minutes.

[j] Women of childbearing potential are required to have pregnancy tests. Negative pregnancy test must be confirmed prior to study drug administration. Urine pregnancy test results from local laboratory are acceptable (except for the screening visit).

[k] Subjects will no longer continue PEG-IFN-α after Week 24; NrtI therapy will continue.

[l] Subjects will complete investigational treatment with BRII-179 or placebo; NrtI therapy will continue into the Follow-up Period

[m] If a subject experiences alanine aminotransferase (ALT) flare additional HBV DNA quantitation sample(s) may be collected.

[n] All AEs related to screening activities must be collected from the time of consent until end of study; any other events reported during the screening period should be reported as medical history. All SAEs must be collected from the time of consent until end of study.

[o] Subjects will be instructed to bring PEG-IFN-α concomitant medications to the trial site at every visit (including all empty containers), as applicable, beginning with the screening visit. Adherence will be assessed by counting of empty containers and unused medication. See Section 6.4.1.

1.5.2. NrtI Discontinuation Monitoring Period

**Study Stage**	**NrtI Discontinuation Monitoring Period[b,c]**						
**Study Visit Week**		W4	W8	W12	W24	W36	W48/ET
**Study Visit Day ± Visit Window**	D1[a]	D28 ± 3	D56 ± 3	D84 ± 3	D168 ± 7	D252 ± 7	D336 ± 7
Safety Laboratory Assessments[d]	X	X	X	X	X	X	X
Symptom-directed physical exam	X	X	X	X	X	X	X
Fibroscan							X
Pregnancy test		X					X
Blood sample for HBsAg quantitative	X			X	X	X	X
Blood sample for anti-HBs quantitative	X			X	X	X	X
Blood sample for HBV DNA quantitative	X	X	X	X	X	X	X
Concomitant medications	←-------------------- X --------------------→						
Adverse event assessment	←-------------------- X --------------------→						
NrtI retreatment surveillance[e]	←-------------------- X --------------------→						
ECI surveillance[f]	←-------------------- X --------------------→						

AE = adverse event; anti-HBs = antibodies to hepatitis B surface antigen; ECI = events of clinical interest; ET = early termination; HBsAg = hepatitis B surface antigen; HBV = Hepatitis B Virus; NrtI = nucleoside/nucleotide; ULN = Upper Limit of Normal

[a] All timepoints are relative to the day of NrtI discontinuation (D1) which is the first day subjects stop their NrtI therapy.

[b] All subjects who discontinue NrtI therapy will be followed through 48 weeks, even if NrtI retreatment occurs. If a subject withdraws prematurely from the study prior to the W48 visit, ET assessments should be performed.

[c] NrtI retreatment guidelines are provided in Section 4.1.2.2.

[d] Safety laboratory assessments are limited to liver function tests.

[e] Investigator should monitor subjects for NrtI retreatment criteria. See Section 4.1.2.2.

[f] Subject will be monitored for clinical laboratory events of clinical interest (ECI) and additional assessments may be performed if indicated. ECI for this study include ALT > 3x ULN, if Day 1 predose ALT ≤ ULN; ALT > 2x baseline, if Day 1 predose ALT > ULN.

2. INTRODUCTION

2.1. Background

Chronic HBV infection remains an important global public health problem that leads to serious sequelae over time, including cirrhosis, liver failure, and hepatocellular carcinoma (Trepo, 2014). An estimated 290 million people are living with chronic HBV infection worldwide, and almost 800,000 people die annually due to associated sequelae (Stanaway, 2016).

Human HBV is a deoxyribonucleic acid (DNA) virus in the Hepadnaviridae family, with 10 HBV genotypes (A to J). The distribution of these genotypes varies geographically, with A and D being more common in Northern Europe, North America, and Western and Central Asia; and B and C being more common in Southeast Asia and China. HBV genotypes may influence disease progression rates and treatment response (Kramvis, 2014).

Chronic HBV infection is characterized by detection of HBV surface antigen (HBsAg) in the blood for > 6 months. The prevalence of chronic HBV infection varies geographically, with a range of < 2% in low to > 8% in high prevalence countries (Schweitzer, 2015). In high prevalence countries, such as those in sub-Saharan Africa and East Asia, transmission occurs predominantly in infants and children by perinatal and horizontal routes. In more industrialized countries, new infections are highest among young adults and transmission occurs predominantly via injection drug use and high-risk sexual behaviors. The risk of developing chronic HBV infection depends on the age at the time of infection. While only ~10% of people infected as adults develop chronic HBV infection, 90% of infants infected perinatally or during the first 6 months of life, and 20% to 60% of children infected between 6 months and 5 years of age remain chronically infected. Twenty-five percent of people who acquire HBV during infancy and childhood will develop primary liver cancer or cirrhosis during adulthood.

Current treatment options for chronic HBV infection are limited. One option is long-term Nucleoside reverse transcriptase inhibitors (NrtI) therapy, for example tenofovir (tenofovir disoproxil fumarate or tenofovir alafenamide) or entecavir (Lok, 2016). However, NrtI therapy does not result in a cure of HBV infection. The viral DNA is not cleared from the infected cell, and viral protein production, in particular the production of HBsAg, typically continues at high levels as measured in blood (Scaglione, 2012). These high levels of HBsAg subviral particles are proposed to mediate HBV-specific immune dysfunction by decoying and exhausting host cellular and humoral adaptive immune responses. Therefore, reduction or clearance of HBsAg from blood is considered central to achieving a functional cure (sustained loss of HBsAg in blood with or without seroconversion to the surface antigen antibody) and is considered an optimal outcome of treatment (Block, 2013). An alternative treatment option is a finite course of Pegylated Interferon α (PEG-IFN-α) therapy which can induce a functional cure, but only in a small percentage of patients (< 10%) (Konerman, 2016). Unfortunately, PEG-IFN-α therapy carries considerable risk of dose-limiting adverse events (AEs) which may be severe and/or serious, and discontinuation rates are significant (Perrillo, 2009).

The failure of NrtI therapy to produce a functional cure and the limitations of PEG-IFN-α therapy highlight the clinical need for effective, well-tolerated HBV therapies of finite duration.

2.2. Study Rationale

BRII-179 (VBI-2601) is a recombinant virus-like particle-based (VLP) based protein immunotherapeutic derived from the 3rd generation HBV preventative vaccine, Sci-B-Vac® consists of all three HBV surface (S) antigen forms: small, middle and large proteins. The large S protein contains the N-terminal preS1 and preS2 domains and the adjacent small S protein. The middle S protein contains preS2 and the adjacent small S protein.

It has been hypothesized that high levels of circulating HBsAg lead to immune tolerance of HBV and contribute to chronic HBV infection. BRII-179 (VBI-2601) utilizes Pre-S1, Pre-S2, and S protein components as the immunogen in a therapeutic application of preS1/S2 containing HBsAg. Additionally, to have an effective T cell response, BRII-179 (VBI-2601) differs from Sci-B-Vac® and most of the recombinant envelope vaccines by using aluminium phosphate as an adjuvant rather than aluminum hydroxide. As a new formulation of higher doses of Sci-B-Vac® with aluminum-phosphate adjuvant, BRII-179 (VBI-2601) shows enhanced Th1 responses against the HBV surface antigens. BRII-179 has been shown to be safe and well-tolerated in chronic HBV (CHB) patients under Nrtl therapy in a phase 1b/2a study (BRII-179-001). Administration of BRII-179 carrying S, Pre-S1 and Pre-S2 regions of HBV envelope is immunogenic at both B and T cell levels in CHB patients.

Multiple therapies with differential mechanisms of actions to treat HBV is an encouraging strategy to achieve higher rates of HBsAg loss and ideally HBsAg seroconversion. The present study (BRII-179-002) will evaluate the efficacy and safety of adding a recombinant protein immunotherapeutic (BRII-179 [VBI-2601]) treatment regimen to existing PEG-IFN-α and NrtI therapy in chronic HBV patients who have participated in the Everest Project (NCT04035837). The primary objective of the Everest Project is to achieve functional cure of HBV infection by optimizing combination and sequential treatment strategies of NrtIs and PEG-IFN-α in patients with chronic HBV infection (negative HBeAg) and low levels of HBsAg (≤ 1500 IU/mL) and HBV DNA (< 100 IU/mL).

Individualized regimens of combination therapy of IFN-α, an innate immunomodulator and antiviral agent and NrtI, a direct acting antiviral agent have shown promising efficacy in reducing HBsAg with some patients achieving HBsAg clearance (Ren, 2016; Marcellin, 2016). This result is more pronounced in patients on optimized HBV treatment to low baseline HBsAg levels, which is the rationale of including subjects from the Everest Project in this study. Preclinical and clinical studies have also shown the benefit of low HBsAg levels for breaking tolerance with a therapeutic HBV vaccine (Zhu, 2016; Michler, 2020; Roggendorf, 2019). BRII-179 (VBI-2601), as an immunomodulator, can break immunological tolerance and restore and/or augment cellular T cell and antibody responses to the HBV surface antigens which in turn result in HBsAg clearance. Therefore, the initial treatment of PEG-IFN-α and NrtI followed by BRII-179 add-on treatment could increase the potential to achieve higher functional cure rate in chronic HBV infected patients.

2.3. Benefit/Risk Assessment

This study will provide information on the efficacy and safety of BRII-179 (VBI-2601) added to PEG-IFN-α and NrtI optimized therapy to achieve functional cure in patients with chronic HBV infection.

BRII-179 (VBI-2601) with or without IFN-α as an adjuvant, administered every 4 weeks for a total of 4 doses, have been evaluated in adult subjects with chronic HBV infection. Favorable safety and tolerability profiles were observed at 40 μg dose level. In addition, the pivotal chronic toxicology studies also support the proposed BRII-179 (VBI-2601) dose regimen of 40 μg every 4 weeks for 9 doses.

The proposed addition of BRII-179 (VBI-2601) to PEG-IFN-α and NrtI therapy may further reduce HBsAg effectively and induce HBV-specific immunity which may lead to immune control of chronic HBV infection. The safety profile of BRII-179 (VBI-2601) is supportive of the overall risk/benefit profile for the proposed dose regimen and treatment duration.

More detailed information about the known and expected benefits and risks and reasonably expected adverse events of BRII-179 (VBI-2601) may be found in the Investigator's Brochure.

3. OBJECTIVES AND ENDPOINTS

3.1. Objectives

The primary objective is:

- Phase 2: To determine the efficacy of BRII-179 to increase HBsAg loss through 24 Weeks
- Phase 3: To evaluate functional cure of chronic HBV subjects receiving BRII-179 (VBI-2601) therapy added to PEG-IFN-α + NrtI.

The secondary objectives are:

- To assess the antiviral activity of BRII-179 (VBI-2601) therapy added to PEG-IFN-α + NrtI.

- To assess safety of BRII-179 (VBI-2601) therapy

3.2. Endpoints

3.2.1. Primary Endpoints

- Phase 2: Percentage of subjects with HBsAg loss at Week 24

- Phase 3: Percentage of subjects with sustained HBsAg and HBV DNA loss (defined as undetectable HBsAg and HBV DNA for at least 24 weeks) after NrtI withdrawal

3.2.2. Secondary Endpoints

- Phase 2: Change from baseline of HBsAg from Day 1 to Week 24

- Phase 2: Percentage of subjects with HBsAg loss at any timepoint

- Phase 3: Percentage of subjects with undetectable HBV-DNA (HBV DNA < LLOQ) 24 and 48 weeks after NrtI withdrawal

- Phase 3: Percentage of subjects meeting the NrtI withdrawal criteria by end of follow-up period (Week 48)

- Phase 3: Percentage of subjects with HBsAg loss at any timepoint

- Phase 3: Percentage of subjects with HBsAg seroconversion at any timepoint

- Phase 3: Percentage of subjects with anti-HBs response by end of follow-up period (Week 48)

- Phase 3: Change from baseline of HBsAg from Day 1 to End of Treatment (Week 32)

- Phase 3: Time to achieve HBsAg seroclearance (HBsAg loss) and seroconversion
- Phase 3: Percentage of subjects requiring NrtI re-treatment during follow up
- Phase 2 and Phase 3: Percentage of subjects with adverse events (AEs) and serious adverse events (SAEs)
- Phase 2 and Phase 3: Percentage of subjects with abnormalities in clinical laboratory tests

4. STUDY DESIGN

4.1. Overall Design

Study BRII-179-002 is a double-blind, randomized, placebo-controlled, parallel-group study of adding BRII-179 (VBI-2601) to existing PEG IFN-α and NrtI therapy in patients with chronic HBV infection without cirrhosis enrolled in the Everest Project study being conducted in China (NCT04035837).

The Everest Project is a multicenter real-world study being conducted in over 260 hospitals in China. The plan is to recruit 30,000 patients with chronic HBV effectively treated with NrtI therapy for at least one year; who are HBeAg negative with HBV DNA <100 IU/ml and HBsAg <1500 IU/mL at study entry. As part of the study participation, subjects receive PEG-IFN-α as "add-on" or "switch to" treatment for 48 to 96 weeks. The primary objective of the Everest Project is to achieve functional cure of HBV infection by optimizing treatment strategies of NrtIs and PEG-IFN-α.

Eligible subjects from the Everest Project study, who have received PEG-IFN-α for 24 to 26 weeks and NrtI therapy for at least 18 months, will be randomized 1:1 to receive BRII-179 (VBI-2601) or placebo in addition to the existing PEG-IFN-α and NrtI therapy. Subjects will be stratified at randomization by screening HBsAg level (3 categories: ≥0.05 to < 1 IU/mL, ≥1 to < 10 IU/mL, and ≥10 to <100 IU/mL). Subjects will discontinue PEG-IFN- α at Week 24; subjects will complete investigational treatment with BRII-179 or placebo at Week 32; while NrtI therapy will continue into the Follow-up Period. The BRII-179-002 study design schema can be found in Section 1.4.

Estimated total duration for each subject will include the following: screening up to 14 days, 32-week dosing period, and up to a 16-week follow-up period. Eligible subjects that meet NrtI discontinuation criteria (Section 4.1.2.1) (eligibility assessed at Week 36 to Week 48) may enter a NrtI discontinuation monitoring period for an additional 48 weeks from the day of NrtI discontinuation (Section 1.5.2).

4.1.1. Independent DSMB

An independent DSMB will monitor patient safety and treatment efficacy data for this study. The details for the operation and responsibilities of the DSMB will be defined in a separate DSMB Charter. The Charter will delineate the composition, duties, responsibilities, and procedures of the DSMB, data required at each meeting, as well as the analyses that will be conducted.

4.1.2. NrtI Discontinuation

4.1.2.1. NrtI Discontinuation Criteria

The investigator will evaluate whether subjects are eligible for discontinuation of NrtI therapy starting at Week 36 and potentially monthly until Week 48 based on the following NrtI discontinuation criteria:

- Alanine aminotransferase (ALT) < 2x ULN (results from previous visit) AND
- HBV DNA < LLOQ (results from previous visit) AND
- 2 consecutive assessments of HBsAg < 0.05 IU/mL (assessed starting from Week 28 laboratory results)

Subjects who are eligible to discontinue from NrtI therapy may discontinue from the Schedule of Activities in Section 1.5.1 and may instead continue into the NrtI Discontinuation Monitoring Period of the study. These subjects will follow the Schedule of Activities for subjects who have discontinued from the NrtI therapy (Section 1.5.2). Subjects who discontinue from their NrtI therapy will be followed in the NrtI Discontinuation Monitoring Period for an additional 48 weeks from their day of NrtI discontinuation.

4.1.2.2. NrtI Retreatment

It is recommended that the Investigator should consider retreatment with NrtI therapy during the NrtI Discontinuation Monitoring Period if subjects meet the following criteria at the same visit (based on Papatheodoridi, 2020):

- ALT > 15x ULN with positive HBV DNA
- ALT 10-15x ULN with positive HBV DNA: retreatment may be implemented at investigator's discretion
- ALT 5-15x ULN with elevated total bilirubin > 2 mg/dL (predominantly direct) and/or prolongation of prothrombin time (PT) ≥ 3 seconds with positive HBV DNA
- ALT 2-5x ULN and HBV DNA >2000 IU/mL persisting for ≥ 12 consecutive weeks
- ALT > 3x ULN and HBV DNA > 100,000 IU/mL at the same visit

Whether subjects are retreated with NrtI is at the discretion of the Investigator following good medical judgement. Subjects who are retreated with NrtI therapy should continue to be followed per the SoA Section 1.5.2 for the duration of the 48-week NrtI Discontinuation Monitoring Period.

4.1.3. PEG-IFN-α Therapy During the Dosing Period

Subjects entering the Dosing Period of the study will continue their peg-IFN-α treatment, investigator should monitor the related adverse events and take necessary medical treatment. The dose of peg-IFN-α is based on relevant clinical guidelines and information from the package insert which is considered as proper by the investigator. Temporary interruptions of peg-IFN-α administration are discouraged. Subjects are to remain on treatment for the entire duration of the study until completion of Week 24 and the dose of peg-IFN-α should not be changed during the trial unless dose reduction or discontinuation is indicated because of laboratory abnormalities/adverse events (see Section 4.1.3.1 for instructions for dose reduction). Subjects must be counselled regarding the importance of not missing doses of peg-IFN-α.

In the case that a patient does miss a dose(s):

- If the peg-IFN-α injection was late by 1 to 3 days, the next dose should be administered immediately. The subsequent dose should then be administered on the next scheduled injection date.
- If the peg-IFN-α injection was late by 4 to 7 days, patient should wait to receive the next scheduled injection (1 dose is skipped).
- If the peg-IFN-α injection was late by >7 days, treatment can be restarted at any time by the investigator and the next dose should be on scheduled injection dates.

4.1.3.1. Dose Adjustment for adverse drug reactions

When a peg-IFN-α dose modification is required for moderate to severe adverse reactions (clinical and/or laboratory), initial dose reduction should be performed. However, in some cases, further dose reduction may be needed. Following improvement of the adverse reaction, re-escalation of the peg-IFN-α dose may be considered.

Hematology:

Peg-IFN-α Hematological Dose Modification Guidelines

Laboratory Values	Reduce PEG-IFN-α Dose:	Discontinue PEG-IFN-α if
Absolute neutrophil count (ANC)	≥750/mm^3: Maintain dose<br>< 750/mm^3: Reduce dose	< 500/mm^3: Treatment should be suspended until ANC values return to more than 1000/mm^3<br>Re-institute at low dose and monitor ANC
Platelet	≥ 50.000/mm^3: Maintain dose<br>< 50.000/mm^3: Reduce to low dose	<25.000/mm^3

Liver function:

In patients with progressive or persistent ALT increases > 10 x ULN , the dose of peg-IFN-α should be reduced and more frequent monitoring of liver function should be performed. If ALT increases are further progressive despite dose reduction or accompanied by increased bilirubin or evidence of hepatic decompensation, therapy should be immediately discontinued. After peg-IFN-α dose reduction or withholding, therapy can be resumed after improvement of liver function by investigator's judgment.

Other disorders:

Peg-IFN-α may cause or aggravate neuropsychiatric, autoimmune, endocrine, ophthalmologic, and infectious disorders. Monitor closely and withdraw therapy with persistently severe or worsening signs or symptoms of the above disorders. Please see package insert.

4.2. Scientific Rationale for Study Design

This is an adaptive phase 2/ phase 3, double-blind, randomized, placebo-controlled, parallel-group study of adding BRII-179 (VBI-2601) to existing PEG-IFN-α and NrtI therapy in patients with chronic HBV infection without cirrhosis enrolled from the Everest Project study being conducted in China. The study design will provide an opportunity to evaluate the ability of BRII-179 (VBI-2601) to achieve a clinically meaningful increase in functional cure (sustained loss of HBsAg in blood with or without seroconversion to the surface antigen antibody) in subjects with chronic HBV on optimized PEG IFN-α and NrtI therapy as compared to therapy consisting of optimized PEG IFN-α and NrtI therapy alone.

There is increasing evidence that low levels of quantitative HBsAg may improve the potential for HBsAg loss, and this is most evident in patients who lose HBsAg after stopping long-term NrtI therapy. It has been observed that patients with decreasing HBsAg and a lower HBsAg level < 100 IU/mL at the end of treatment were able to maintain control of HBV after discontinuation of long-term NrtI therapy (Chang, 2015; Yao 2017; Jeng, 2018; Papatheodoridi, 2020). Recent reporting from the Everest Project seems to corroborate this observation from previous studies. In 1427 patients on background NrtI therapy who have completed 24 to 26 weeks of PEG-IFN-α treatment and 516 patients who have completed 48 weeks of PEG-IFN-α treatment, the overall rate of HBsAg loss for patients treated ≥12, ≥24, ≥36 and ≥48 weeks was 13.00%, 16.06%, 20.49% and 23.01%, respectively. Subgroup analysis based on baseline HBsAg stratification (0.05–100, 100–500, 500–1000, 1000–1500 IU/mL) found that the HBsAg loss rate was highest in patients with baseline HBsAg between 0.05 and 100 IU/mL (34.93% for patients treated ≥ 48 weeks) (Ze-Qian, 2020).

Viral rebound may occur shortly after NrtI withdrawal in some cases, which requires close monitoring during study participation in order to control ALT flares. The HBV viral relapse after NrtI withdrawal may induce the host immune response and potentially a long-term immune control against the viral infection. Sustained HBsAg loss and/or virological response were observed in HBeAg-negative patients stopping NrtI therapy in prospective cohort studies (van Bömmel, 2017) as well as in a randomized controlled study (Barthel FM-S, Parmar MKB, Royston P. How do multi-stage, multi-arm trials compare to the traditional two-arm parallel group design – a reanalysis of 4 trials. Trials 2009; 10:21, doi:.10.1186/1745-6215-10-21.

Berg, 2017). This study design should allow for the evaluation of the potential benefit from immune responses resulting from BRII-179 and decreased viral antigen exposure after long-term NrtI cessation.

Surrogate endpoints of HBV functional cure measure biochemical, virological, and histological outcomes. Based on current knowledge, the best surrogate of HBV functional cure is HBsAg loss confirmed on 2 occasions at least 6 months apart without requirement forHBsAg seroconversion plus undetectable HBV DNA (Choodari-Oskooei B, Parmar

MKB, Royston P, Bowden J. Impact of lack-of-benefit stopping rules on treatment effect estimates of two-arm multi-stage (TAMS) trials with time to event outcome. Trials 2013, 14:23, http://www.trialsjournal.com/content/14/1/23.

Cornberg, 2020). The incidence of sustained HBsAg loss and undetectable HBV DNA will be used to assess the potential of BRII-179 (VBI-2601) to induce functional cure.

The target population of this study is HBeAg-negative non-cirrhotic patients with chronic HBV infection that has been virologically suppressed (baseline HBsAg ≥ 0.05 IU/mL and < 100 IU/mL). This represents a patient population who may have a higher likelihood of sustained HBsAg loss.

4.3. Justification for Dose

It is believed that high levels of HBsAg have a negative impact on immune profile and may tolerize or delete HBV specific T cells in Chronic HBV patients (Kondo, 2013; Ferrari, 2015; Lebossé, 2017; Wang, 2013). As demonstrated in previous nonclinical studies (Zhu, 2016; Michler, 2020), initial reductions of HBsAg by therapeutic interventions enabled follow-up HBV vaccine treatments to break tolerance and achieve functional cure in animal HBV models. The BRII-179-001 study, in chronic HBV patients, demonstrated that BRII-179 at 20 or 40 ug with or without IFN-α was safe and well tolerated as well as induced humoral and cellular immune responses in a significant number of subjects under Nrtl background therapy. In the BRII-179-001 clinical study, antibody response to pre-S1 and/or pre-S2 antigens was only seen in cohorts admixed with IFN-α, suggesting addition of IFN-α as co-adjuvant may further enhance the restimulation of immune response to pre-S1 and pre-S2 antigens. This is consistent with the preclinical study results in the AAV-HBV model. When high HBsAg levels were lowered by siRNA treatment, BRII-179 treatment significantly delayed rebound of HBsAg level compared to siRNA treatment alone in the AAV-HBV model. It is thus hypothesized that significant HBsAg reduction by optimized PEG-IFN-α and NrtI therapy in chronic HBV patients will provide an optimal treatment window to maximize the BRII-179 potential in restoring and sustaining immune control of HBV infection, and ultimately lead to functional cure.

High doses of prophylactic HBV vaccines and increasing the number of doses (6-11) have been demonstrated to provide improved and durable immune response in chronic HBV patients (Hoa, 2009; Roggendorf, 2019; Wong, 2018). Therefore, it is postulated that reducing HBsAg level with optimized PEG IFN-α and NrtI therapy followed by the addition of BRII-179 (VBI-2601), e.g., up to 9 monthly injections of 40 μg of BRII-179, in chronic HBV patients could provide desirable HBV specific humoral and T cell responses that may restore and sustain immune control of HBV infection, thereby achieving functional cure. In addition, the pivotal chronic toxicology studies also support the proposed BRII-179 (VBI-2601) dose regimen of 40 μg every 4 weeks for 9 doses. More detailed information may be found in the Investigator’s Brochure for BRII-179 (VBI-2601).

In summary, currently available safety, tolerability, pharmacokinetic (PK), pharmacodynamic (PD) and PK/PD results from BRII-179 (VBI-2601), as well as the safety margin assessment from the toxicology studies, support the proposed BRII-179 (VBI-2601) treatment regimen to achieve functional cure in patients with chronic HBV infection.

4.4. End of Study Definition

A subject is considered to have completed the study if he/she has completed all phases of the study including the last scheduled procedure shown in the Schedule of Activities.

The end of the study is defined as the date of the last visit of the last subject in the study or last scheduled procedure shown in the Schedule of Activities for the last subject in the trial.

5. STUDY POPULATION

A total of 480 evaluable adult subjects with virologically suppressed chronic HBV infection without cirrhosis receiving PEG-IFN-α and NrtI therapy are planned for this study.

Prospective approval of protocol deviations to recruitment and enrollment criteria, also known as protocol waivers or exemptions, is not permitted. Adherence to the criteria as specified in the protocol is essential.

5.1. Inclusion Criteria

Everest Project subjects eligible to be included in the study must meet all of the following criteria. All laboratory test requirements for inclusion will be performed by central laboratory unless specified otherwise.

1. A signed and dated written Informed Consent Form (ICF) prior to study participation.
2. Age ≥ 18 (or age of legal consent, whichever is older) , and ≤ 60 at time of screening.
3. Documented chronic HBV infection (positive serum HBsAg for ≥ 6 months).
4. Negative HBeAg
5. HBsAg ≥ 0.05 IU/mL and < 100 IU/mL at screening.
6. HBV DNA < LLOQ at screening (central lab)
7. Must be receiving NrtI therapy for ≥ 18 months and PEG-IFN-α for 24 to 26 weeks of the Day 1 visit.
8. Female subjects must have a negative pregnancy test or confirmation of post-menopausal status. Post-menopausal status is defined as 12 months with no menses without alternative medical cause. Women of child-bearing potential (WOCBP) must have a negative serum pregnancy test at screening and a negative urine pregnancy test on Day 1, cannot be breast feeding, and must be willing to use highly effective methods of contraception 14 days before study drug administration through 12 weeks after last study drug administration.
9. Agrees to not donate blood during the duration of the study.
10. Serum ALT and AST ≤ 5x ULN at screening. Study screening laboratory tests may be repeated once for values considered to be erroneous with medical monitor approval.

5.2. Exclusion Criteria

Everest Project subjects are excluded from the study if any of the following criteria apply:

1. Any clinically significant chronic medical condition other than chronic HBV infection that, in the opinion of the investigator makes the patient unsuitable for participation in the study.

2. Any clinically significant acute condition such as fever (> 38° C) or acute respiratory illness within 7 days of first study drug administration.

3. Significant liver fibrosis or cirrhosis as defined by having either a FibroScan result of > 9kPa at screening, unless the subject has results from a FibroScan evaluation performed within 6 months prior to screening or liver biopsy performed within 1 year prior to screening that confirm the absence of Metavir F3 fibrosis or F4 cirrhosis. If a subject has had both procedures in the specified timeframes, the most recent result should be used to determine eligibility.

4. History or evidence of drug or alcohol abuse as assessed by the Investigator, within the 12 months before screening.

5. History of intolerance to IM injection.

6. History of clinically significant chronic liver disease from any cause other than chronic HBV infection.

7. History of hepatic decompensation, including ascites, hepatic encephalopathy and/or esophageal or gastric varices.

8. Treatment by immunosuppressant (including but not limited to corticosteroids at ⩾1 time prednisolone equivalent dose > 20 mg/day) within 30 days before study drug administration at the Day 1 visit and throughout the duration of study participation.

9. Known history of immunological function impairment, including but not limited to:

   - Autoimmune disease (e.g., multiple sclerosis, type 1 diabetes mellitus , myasthenia gravis, Crohns disease and other inflammatory bowel diseases, celiac disease, systemic lupus erythematosus, scleroderma, including diffuse systemic form and limited cutaneous form of systemic sclerosis or CREST syndrome, systemic sclerosis, dermatomyositis polymyositis, rheumatoid arthritis, juvenile idiopathic arthritis, autoimmune thyroiditis including Hashimoto thyroiditis, Grave’s or Basedow’s disease, immune thrombocytopenic purpura, autoimmune hemolytic anemia, autoimmune hepatitis, psoriasis, vitiligo, vasculitis, Guillain Barré syndrome, Addison’s disease, Bell’s palsy, and alopecia areata); OR

- Primary immunodeficiency disorders (e.g., common variable immune deficiency, defective phagocytic cell function and neutropenia syndromes, and complement deficiency); OR
- Secondary immunodeficiency disorders (e.g., resulting from acquired immunodeficiency syndrome caused by human immunodeficiency virus (HIV) infection, solid organ transplant, and splenectomy).

10. Any HBV vaccine (licensed or experimental) prior to study enrolment and throughout the duration of study participation.
11. Immunization with attenuated vaccines (e.g., Mumps, Measles, Rubella) within 30 days before study drug administration at the Day 1 visit and throughout the duration of study participation.
12. Has received blood products or immunoglobulin within 90 days before study drug administration at the Day 1 visit or likely to require blood products during the study.
13. Participated in another clinical trial with an investigational drug or a biologic within 30 days or within 5 drug half-lives (whichever is longer) before study drug administration at the Day 1 visit. Subjects must agree not to take part in any other study at any time during their participation in this study, inclusive of the follow-up period.
14. Has received granulocyte macrophage colony stimulating factor (GM CSF) or erythropoietin (EPO) within 30 days before study drug administration at the Day 1 visit or likely to require GM CSF/EPO during the study.
15. Previous or current psychiatric condition that precludes compliance with the protocol. Specifically excluded are persons with psychoses, ongoing risk for suicide, or history of suicide attempt or gesture within the past 5 years.
16. The subject has any history of cancer requiring locoregional or systemic therapy within 5 years of randomization or has current disease. Subjects with completely surgically resected tumors with no evidence of disease for more than 3 years, or a history of low risk basal cell carcinoma will be accepted (low risk being defined as 1) location on the trunk of the body, arms, legs, cheeks, forehead, temples, scalp, neck, or chin, 2) < 2 cm in size, 3) nodular or superficial, 4) primary cancer that has not come back after treatment, 5) edge of the cancerous area is clear and smooth, and 6) not located in or around nerves).
17. Any skin abnormality or tattoo that would limit post treatment injection site assessment.
18. Known hypersensitivity or contraindication to BRII-179 (VBI-2601) or its excipients.
19. Unwilling, or unable in the opinion of the investigator, to comply with study requirements, including the use of an adequate birth control method.

20. Active infection with human immunodeficiency virus (HIV), hepatitis C virus (HCV) or hepatitis Delta virus.

21. Donated more than 500 mL of blood within 90 days before study drug administration.

22. Renal impairment with estimated glomerular filtration rate (eGFR) ≤ 60 ml/min/1.73 m^2 at screening.

23. Uncontrolled diabetes mellitus as determined by the Investigator.

24. Diagnosis of advanced stage heart failure (New York Heart Association Class III or Class IV or unstable angina).

25. Any laboratory test abnormality that is considered as clinically significant or may contradict continued treatment with peg-IFN by the investigator.

5.3. Lifestyle Considerations

1. Alcohol consumption is limited to no more than 1 unit per day (unit: wine [about 125 mL] = white spirit [about 30 mL] = beer [about 284 mL]) during the participation of this study.

5.4. Screen Failures

Screen failures are defined as subjects who consent to participate in the clinical study but are not subsequently randomly assigned to study drug. A minimal set of screen failure information is required to ensure transparent reporting of screen failure subjects to meet the Consolidated Standards of Reporting Trials (CONSORT) publishing requirements and to respond to queries from regulatory authorities. Minimal information includes demography, screen failure details, eligibility criteria, and any serious adverse event (SAE).

Individuals who do not meet the criteria for participation in this study (screen failure) may not be rescreened.

6. STUDY INTERVENTION

6.1. Study Intervention(s) Administered

6.1.1. Formulations

6.1.1.1. BRII-179 and Placebo

The following study drugs will be used in this study:

- BRII-179 40µg IM injection
- Placebo IM injection

BRII-179 (VBI-2601) is aluminium phosphate absorbed HBsAg Bulk Drug Substance (HBsAg spherical particles [~30 nm mean diameter] suspended in phosphate-buffered saline) and 8% Sucrose, in addition to 10 mM phosphate buffer.

Placebo will be prepared from commercially available 0.9% Sodium Chloride for Injection which will be provided to the study site. Subjects assigned to placebo will receive a volume of placebo that would be equal to the volume that would be required if that subject had been assigned to IP.

6.1.1.2. Other Study Medications

Brii Biosciences will also be providing commercially available PEG-INF-α.

PEG-INF-α is a colorless, clear liquid formulation of peginterferon α-2b from modified recombinant human interferon α2b expressed in yeast system with polyethylene glycol (40 kD Y type) and excipients of sodium chloride, sodium acetate, mannitol, aspartic acid, water for injection, and sodium hydroxide to adjust pH.

6.1.2. Dosage and Administration of BRII-179 and Placebo

**Arm Name**	**BRII-179 (VBI-2601)**	**Placebo**
**Intervention Name**	BRII-179 (VBI-2601)	Placebo
**Type**	Drug	Placebo
**Dose Formulation**	Solution for Injection	0.9% Sodium Chloride for Injection
**Dose Level(s)**	BRII-179 40 µg	Placebo
**Route of Administration**	BRII-179 in one syringe; administer via IM injection	Placebo in one syringe; administer via IM injection

Arm Name	BRII-179 (VBI-2601)	Placebo
	Refer to the Pharmacy Manual for the details.	Refer to the Pharmacy Manual for the details.
**Use**	Experimental	Experimental
**IMP and NIMP**	IMP	Not applicable
**Sourcing**	Provided centrally by the Sponsor	Provided centrally by the Sponsor
**Packaging and Labeling**	IMP will be labeled as required per country requirement.	Placebo will be prepared and labelled as instructed in the Pharmacy Manual

On dosing days, the pharmacist or designee will prepare the required amount of study drugs into syringe for injection. A qualified clinical investigative site staff member under the supervision of the investigator or designee will administer the study drug. The injection site will be marked and mapped for later observation and should be documented. If a local reaction around the injection site occurs, photographs may be obtained. Please refer to Section 6.5 for further details.

6.2. Preparation/Handling/Storage/Accountability

- Only subjects enrolled in the study may receive study drug and only authorized site staff may supply or administer study drug. All study drug must be stored in a secure, environmentally controlled, and monitored (manual or automated) area in accordance with the labeled storage conditions with access limited to the investigator and authorized site staff.
- The investigator, institution, or the head of the medical institution (where applicable) is responsible for study drug accountability, reconciliation, and record maintenance (i.e., receipt, reconciliation, and final disposition records).
- Further guidance and information for the final disposition of unused study drugs are provided in the Pharmacy Manual.

6.3. Randomization and Blinding

Subjects will be assigned to study treatment in accordance with the randomization schedule. The randomization code will be generated by using a validated computerized system. Subjects will be randomized using an Interactive Web Response System (IWRS). Once a randomization number is assigned to a subject, it cannot be reassigned to any other subject in the study. Study intervention will be dispensed at the study visits

summarized in SoA (Section 1.5.1). Returned study intervention should not be re-dispensed to the subjects.

Study drug will be double-blind. Neither the subject nor the study physician will know which study drug the subject is receiving. The investigator or treating physician may unblind a subject's treatment assignment **only in the case of an emergency** when knowledge of the study treatment is essential for the appropriate clinical management or welfare of the subject. Whenever possible, the investigator must first discuss options with the Medical Monitor or appropriate sponsor study personnel before unblinding the subject's treatment assignment. If this is impractical, the investigator must notify the sponsor as soon as possible, but without revealing the treatment assignment of the unblinded subject, unless that information is important for the safety of subjects currently in the study. The date and reason for the unblinding must be recorded in the appropriate data collection tool.

Sponsor clinical safety and pharmacovigilance staff may unblind the treatment assignment for any subject with an SAE. If the SAE requires that an expedited regulatory report be sent to one or more regulatory agencies, a copy of the report, identifying the subject's treatment assignment, may be sent to clinical investigators in accordance with local regulations and/or sponsor policy.

The primary reason for treatment discontinuation (the event or condition that led to the unblinding) will be recorded in the eCRF. Subject may continue in the follow-up period of the study once the treatment code is unblinded (see Section 7.1 Discontinuation of Study Intervention).

Subjects will receive study drugs directly from the investigator or designee, under medical supervision. The date and time of each dose administered in the clinic will be recorded in the source documents and recorded in the eCRF. The dose of study drug and study subject identification will be confirmed at the time of dosing by a member of the study site staff other than the person administering the study drug.

6.4. Concomitant Therapy

All subjects from the Everest Project are required to receive NrtI therapy for ≥ 18 months and PEG-IFN-α for 24 to 26 weeks prior to the Day 1 visit. Investigators should remind patients the importance of taking NrtI daily unless their NrtI treatment is discontinued.

Any medications that the subject is receiving at the time of enrollment or receives during the study must be recorded along with:

- Reason for use
- Dates of administration including start and end dates
- Dosage information including dose and frequency

Should subjects have a need to initiate treatment with any excluded concomitant medication, the Medical Monitor must be consulted prior to initiation of the new

medication. In instances where an excluded medication is initiated prior to discussion with the Sponsor, the investigator must notify the Sponsor as soon as he/she is aware of the use of the excluded medication. The Medical Monitor should be contacted if there are any questions regarding concomitant or prior therapy.

6.4.1. Medication Adherence with NrtI and PEG-IFN-α

Every attempt should be made to ensure good adherence with NrtI and PEG-IFN-α therapy. Adherence to NrtI and PEG-IFN-α should be assessed at every relevant visit. Adherence of peg-IFN-α will be assessed by counting of empty containers and unused medication. Subjects will be instructed to bring all empty containers of PEG-IFN-α to the trial site at every visit, as applicable, for adherence assessment. Adherence of NrtI will be assessed diary cards for recording that are provided to subjects.

Subjects who have prematurely discontinued NrtI or PEG-IFN- α should be encouraged to continue with study participation.

6.4.2. Permitted Medications

The following medications/treatments are permitted:

- Hormone replacement therapy.
- Oral, injectable, subdermal, intravaginal, or implantable contraceptives, as well as intrauterine device, and intrauterine hormone-releasing system are permitted for contraception.
- Inhaled or topical steroids are allowed.
- Prescription drugs necessary to treat hypertension. Hypertension must be well controlled on a stable dose of medication for > 6 months prior to study drug administration at Day 1 visit.
- Prescription drugs necessary to treat diabetes mellitus. Diabetes mellitus must be controlled on a stable dose of medication for > 6 months prior to study drug administration at Day 1 visit.
- Paracetamol (≤ 2 g/day), aspirin (≤ 3 g/day), or ibuprofen (< 1.2 g/day).
- Antihistamines.
- Prescription inhaler or nebulizer to treat asthma (inhaled bronchodilator or inhaled steroid). Asthma must be well controlled, defined as historical use of a rescue bronchodilator on average no more than twice per week.
- A stable dose and regimen of other medication(s) (prescription or OTC) that the subject is taking regularly (i.e. medications for chronic conditions such as high cholesterol or depression must be unchanged in dose or type for at least 6 months) is permitted.
- Licensed SARS-CoV-2 vaccines, including those approved for emergency use by local authorities, for the prophylaxis of COVID-19 are allowed. Each COVID-19 vaccine administration should be ideally with a minimum interval of 14 days

before or after BRII-179 (VBI-2601) administration. However, COVID-19 vaccines may be administered within a shorter period in situations to avoid barriers or delays to COVID-19 vaccination.

6.4.3. Prohibited Medications

The following medications are excluded while subjects are participating in the study.

Prior to study enrolment and throughout the duration of study participation:

- Any HBV vaccine (licensed or experimental)

Within 30 days before study drug administration at the Day 1 visit and throughout the duration of study participation:

- Immunosuppressant (including but not limited to corticosteroids at a prednisolone equivalent dose > 20 mg/day)
- Immunization with attenuated vaccines (e.g., Mumps, Measles, Rubella), except COVID vaccines
- Any investigational drug or biologic other than the study product(s) used during this study
- Granulocyte-macrophage colony-stimulating factor (GM-CSF) or erythropoietin (EPO)
- Any Chinese traditional medicine or herbal remedies with hepatotoxicity

Within 90 days before study drug administration at the Day 1 visit and throughout the duration of study participation:

- Blood products or immunoglobin

6.5. Safety Follow-up and Management of Reactions to Study Drug

The following safety observation procedures will be performed immediately following study drug administration and during the follow-up period for all subjects:

- Subjects will remain in the clinic for at least one hour post study drug administration. The observation period will include an assessment for rare adverse reactions.
- Any unusual signs or symptoms reported during the initial one hour of observation will prompt continued close monitoring.
- Based on their condition, subjects may be asked to remain in the clinic for more than one hour after the study drug administration (reason will be recorded in source data).

- All data (including assessment of solicited local and systemic reactions) will be recorded in the source document during and after the post-observation period.

A measurement device template (in mm) for measuring solicited local reactions (erythema [redness] and swelling), and an oral thermometer for measuring daily temperature, will be provided at the first study drug administration visit, study Day 1. Subjects will be advised to record their temperature in the diary card.

Refer to Sections 8.3 and 10.3 for further details on the recording, assessment, and reporting of AEs.

6.6. Treatment of Study Intervention Overdose

An overdose is defined as an accidental or intentional administration of a quantity of a medicinal product given per administration or cumulatively which is above the maximum recommended dose as per protocol which results in clinical signs and symptoms. These should be recorded by the investigator on the AE/SAE pages. In the event of an overdose of study medication, the investigator should use clinical judgement in treating the overdose and contact the study medical monitor.

The Sponsor is not recommending specific treatment guidelines for overdose and toxicity management. The investigator is advised to refer to the relevant document(s) for detailed information pertaining to the study drug being used in this study. Such documents may include, but not be limited to, the IB or equivalent document provided by the Sponsor.

6.7. Intervention after the End of the Study

The Sponsor will not provide post-study treatment. The investigator is responsible for ensuring that consideration has been given to the post-study care of the patient’s medical condition.

7. DISCONTINUATION OF STUDY INTERVENTION AND SUBJECT DISCONTINUATION/WITHDRAWAL

7.1. Discontinuation of Study Intervention

If a subject discontinues study dosing (for example, as a result of meeting criteria for discontinuation of study intervention described in Section 7.1.1), every attempt should be made to keep the subject in the study and continue to perform the required study-related visits and procedures for the remaining duration of study participation in the Dosing Period and Follow-up Period.

Subjects who have discontinued study drug may be reassessed for possible resumption of study drug dosing not to exceed the protocol defined number of doses, dosing interval, or dosing period duration. Subjects who have discontinued study drug will be assessed for NrtI discontinuation as described in Section 4.1.2.1 upon continued study participation into the Follow-up Period.

If continued study participation is not possible or acceptable to the subject or investigator, please refer to Section 7.2 Subject Discontinuation/Withdrawal from the Study.

7.1.1. Criteria for Discontinuation of Study Intervention

A subject who has received at least one dose of BRII-179 (VBI-2601) will have additional doses withheld if any of the following criteria are met:

- Signs or symptoms of severe (NCI-CTCAE Grade 3 or higher) hypersensitivity or anaphylaxis
- Significant abnormalities in liver chemistry, defined as any of the following are met:
  - Serum ALT > 10 × ULN
  - Serum ALT or AST > 3 × ULN with a concomitant total bilirubin > 2 × ULN or INR > 1.5
- Any clinical manifestations of hepatic decompensation

The Sponsor may request DSMB evaluation of specific data in the event of liver flare to fully characterize transaminase elevations. The subject may receive remaining doses of study drug only after DSMB approval.

7.2. Subject Discontinuation/Withdrawal from the Study

- A subject may withdraw from the study at any time at his/her own request or may be withdrawn at any time at the discretion of the investigator for safety, behavioral, compliance, or administrative reasons. This is expected to be uncommon.
- At the time of discontinuing from the study, if possible, an Early Termination Visit should be performed (Section 8.1.7) for data to be collected at the time of study discontinuation and follow-up. The subject will be permanently discontinued from both the study intervention and from the study at that time.

- If the subject withdraws consent for disclosure of future information, the sponsor may retain and continue to use any data collected before such a withdrawal of consent.
- If a subject withdraws from the study, he/she may request destruction of any samples taken and not tested, and the investigator must document this in the site study records.
- Subjects who are withdrawn from the study will NOT be replaced and cannot be rescreened.
- The primary reason for subject withdrawal will be recorded in the eCRF. Primary reasons for withdrawal will be categorized as:
  - Adverse event
  - Protocol deviation
  - Withdrew consent, not due to an AE
  - Lost to follow up
  - Study closed/terminated

7.2.1. Lost to Follow up

A subject will be considered lost to follow-up if he or she repeatedly fails to return for scheduled visits and is unable to be contacted by the study site.

The following actions must be taken if a subject fails to return to the clinic for a required study visit:

- The site must attempt to contact the subject and reschedule the missed visit as soon as possible and counsel the subject on the importance of maintaining the assigned visit schedule and ascertain whether or not the subject wishes to and/or should continue in the study.
- Before a subject is deemed lost to follow up, the investigator or designee must make every effort to regain contact with the subject (where possible, 3 telephone calls and, if necessary, a certified letter to the subject’s last known mailing address or local equivalent methods). These contact attempts should be documented in the subject’s medical record.
- Should the subject continue to be unreachable, only then will he/she be considered to have withdrawn from the study with a primary reason of “Lost to Follow-up”.

Discontinuation of specific sites or of the study as a whole are handled as part of Section 10.1.7, Appendix 1.

8. STUDY ASSESSMENTS AND PROCEDURES

8.1. Schedule of Assessments

Study procedures and their timing are summarized in the SoAs (Section 1.5). Adherence to the study design requirements, including those specified in the SoAs, is essential and required for study conduct. Protocol waivers or exemptions are not allowed. Immediate safety concerns should be discussed with the sponsor immediately upon occurrence or awareness to determine if the subject should continue or discontinue study drug.

8.1.1. Screening

Written informed consent must be obtained prior to initiation of any study procedures. All screening evaluations must be completed and reviewed to confirm that potential subjects meet all eligibility criteria. The investigator will maintain a screening log to record details of all subjects screened and to confirm eligibility or record reasons for screening failure.

Screening will be performed no more than 2 weeks prior to randomization and will include written consent, determination of subject eligibility, collection of demographics and medical history/ current medical conditions as well as a physical examination, vital signs, 12-lead ECG, clinical laboratory tests, and other assessments per the SoA (Section 1.5.1).

Adverse events related to screening activities must be collected from the time of consent until the end of the study; any other events reported during the screening period should be reported as medical history. All SAEs must be collected from the time of consent until end of study.

8.1.2. Randomization (Day 1)

Eligible subjects will be randomized 1:1 to either 40 µg of BRII-179 administrated IM or matching placebo administrated IM. Subjects will receive their first dose of study drug on Day 1, however subject eligibility must be confirmed prior to study drug administration and blood sample collections will be made prior to study drug administration. Other assessments to be conducted on Day 1 are detailed in the SoA (Section 1.5.1).

8.1.3. Dosing Period

All subjects will receive BRII-179 (VBI-2601) or placebo administered IM every 4 weeks for a total of 9 doses. Subjects will discontinue PEG-IFN- α at Week 24; subjects will complete investigational treatment with BRII-179 or placebo at Week 32; while NrtI therapy will continue into the Follow-up Period.

Subjects will return to the investigative site on an outpatient basis for antiviral activity monitoring and safety assessment as specified in the SoA (Section 1.5.1).

Prior to study drug administration, there will be a review of ongoing eligibility to receive the study drug and continue with study participation. Diary cards will be collected and

there will be a review of any local and systemic reactions. Blood samples will be collected prior to each study drug administration.

8.1.4. Follow-up Period

Subjects will return to the investigative site for the specified assessments per the SoA (Section 1.5.1), including but not limited to physical examination, vital signs, clinical laboratory tests, and review of adverse events and concomitant medications.

Subjects will be assessed by the investigator whether they are eligible for discontinuation of NrtI therapy starting at Week 36 and potentially monthly until Week 48. Subjects that have NOT achieved NrtI discontinuation criteria by Week 48 will be discharged from the study.

8.1.5. NrtI Discontinuation Monitoring Period

Eligible subjects who meet NrtI withdrawal criteria will be informed by the investigator to discontinue their NrtI medications on the first day of their scheduled Day 1 visit of the NrtI Discontinuation Monitoring Period. These subjects will follow the NrtI Discontinuation Monitoring Period SoA (Section 1.5.2).

Subjects who discontinue from their NrtI therapy will be followed in the NrtI Discontinuation Monitoring Period for an additional 48 weeks from the day of NrtI discontinuation. Subjects who are retreated with NrtI therapy should continue to be followed per SoA (Section 1.5.2) for the duration of the 48-week NrtI Discontinuation Monitoring Period.

8.1.6. Unscheduled Visits

Subjects may be asked to come for an unscheduled visit for clinical assessment of an adverse event if warranted, as determined by the investigator. The unscheduled visit will be recorded in the eCRF.

8.1.7. Early Termination Visit

In cases where a subject prematurely discontinues from the study, the investigator must make every effort to have the subject return to the clinic within 14 days for an Early Termination (ET) Visit. Assessments for ET visits will include but not limited to physical examination, pregnancy test, clinical laboratory tests, and other assessments per the SoA (Section 1.5.1).

A follow Up Visit will be conducted approximately 4 weeks after the last dose of study drug. This will be required only if the subject has ongoing AEs or lab abnormalities at the last on-study visit. This visit may be conducted by telephone.

8.2. Detailed Description of Study Procedures

8.2.1. Medical History

A complete medical history, including details regarding illnesses and allergies, date(s) of onset, and whether condition(s) is currently ongoing (current medical conditions), medication history, including prior HBV treatment history and HBV genotype history (if available), will be collected on all subjects during screening and should be updated prior to dosing at the Day 1 visit.

8.2.2. Physical Examinations

A complete physical examination will include, at a minimum, general appearance, head, neck, chest/respiratory, heart/cardiovascular, gastrointestinal/liver, extremities, skin, and neurological assessments. Height and weight will also be measured and recorded. Body mass index will be calculated from height and weight.

A symptom-directed physical examination will be performed according to investigator discretion.

8.2.3. Fibroscan

To exclude the presence of cirrhosis, subjects will have a FibroScan evaluation, unless the subject has results from a FibroScan evaluation performed within 6 months prior to screening or liver biopsy performed within 1 year prior to screening that confirm the absence of Metavir F3 fibrosis or F4 cirrhosis. If a subject has had both procedures in the specified timeframes, the most recent result should be used to determine eligibility.

A FibroScan will also be conducted at the Week 48/ ET Visit in the NrtI Discontinuation Monitoring Period.

8.2.4. Alcohol Intake Assessment

Alcohol intake during the study will be recorded.

8.2.5. Vital Signs

Temperature (oral preferred), pulse rate, respiratory rate, and blood pressure will be assessed after the subject has rested comfortably for approximately 5 minutes. Pulse rate and blood pressure measurements will be assessed with a completely automated device. Manual techniques will be used only if an automated device is not available. Blood pressure should be based on 2 separate measurements. When scheduled at the same timepoints, vital signs and 12-lead ECG must be assessed first before physical examinations, blood sample collections, and urine collection.

8.2.6. Electrocardiograms

12-lead ECGs will be recorded and reviewed on-site by the investigator as outlined in SoA (Section 1.5.1).

8.2.7. Laboratory Assessments

8.2.7.1. Screening Viral Serology

The screening viral serology parameters will include chronic HBV infection and active infection with HIV, HCV, and hepatitis Delta virus. Chronic HBV infection is defined as serum HBsAg > 6 months.

8.2.7.2. Clinical Safety Laboratory Assessments

Fasting clinical safety laboratory tests to be performed are described in Section 10.2.

The investigator should conduct additional clinical assessment for the adverse effects of PEG-IFN-α, including but not limited to radiological examination, ophthalmology assessment, immunological marker test, blood sugar and psychological assessment.

The investigator must review the laboratory report, document this review, and record any clinically significant changes occurring during the study in the AE section of the eCRF. The laboratory reports must be filed with the source documents. Clinically significant abnormal laboratory findings are those which are not associated with the underlying disease, unless judged by the investigator to be more severe than expected for the subject's condition.

In the event of an unexplained clinically relevant abnormal laboratory test occurring after study drug administration, the test should be repeated and followed at investigator discretion until it has returned to normal or baseline, and/or a diagnosis is made to adequately explain the abnormality.

8.2.7.3. Pregnancy Testing

A pregnancy test or confirmation of post-menopausal status must be confirmed for all female subjects. Post-menopausal status is defined as 12 months with no menses without alternative medical cause. Pregnancy tests will be performed for WOCBP only. Serum and urine pregnancy testing will be performed per the SoAs (Section 1.5) and any time pregnancy is suspected. A WOCBP who is known to be pregnant or who does not have a negative pregnancy test at screening is not eligible for study participation. During the study, the results of these pregnancy tests must be known prior to study drug administration. A WOCBP determined to be pregnant while on study will be followed until the pregnancy outcome is known, as described in Section 8.3.5.1.

8.2.7.4. Assessment of Antiviral Activity

Assessment of antiviral activity will include HBsAg, anti-HBs, and HBV DNA.

Details regarding the processing, shipping, and analysis of the samples will be provided in the Laboratory Manual.

8.2.8. Laboratory Events of Clinical Interest

A clinical laboratory event of clinical interest (ECI) is a laboratory value of scientific and medical interest specific to understanding of the investigational product and may require additional follow-up testing, close monitoring, and rapid communication by the investigator to the Sponsor.

During NrtI Discontinuation Monitoring Period, any laboratory events of ECI will be monitored, defined as the following:

- ALT > 3x ULN, if Day 1 predose ALT ≤ ULN
- ALT > 2x baseline, if Day 1 predose ALT > ULN

All laboratory ECIs must be reported to the Sponsor within 24 hours of the investigator's knowledge of the event. See Section 10.3.5. In addition, the assessments outlined in Section 10.5 should be performed as soon as possible and no later than two weeks after the initial ALT value meeting criteria for a laboratory ECI.

The laboratory ECI will also be evaluated by the investigator to determine if the event meets criteria for an AE. If AE criteria are met, all clinical sequelae in relation to these laboratory ECIs will be reported as AEs or SAEs, if applicable.

8.3. Adverse Events and Serious Adverse Events

The investigator and any qualified designees are responsible for detecting, documenting, and recording events that meet the definition of an AE or SAE and remain responsible for following up AEs that are serious, considered related to the study drug or study procedures, or that caused the subject to discontinue the study drug or study.

The definitions and procedures for reporting, recording, evaluating, and follow-up of an AE or SAE can be found in Section 10.3, Appendix 3.

8.3.1. Time Period and Frequency for Collecting AE and SAE Information

All SAEs will be collected from the signing of the informed consent form (ICF) until the last follow-up visit at the time points specified in the SoAs (Section 1.5).

All AE related to screening activities will be collected from the signing the ICF until the last follow-up visit at the time points specified in the SoAs (Section 1.5).

Medical occurrences that begin before the start of study drug but after obtaining informed consent will be recorded as medical history/ current medical conditions and not as an AE.

All SAEs will be recorded and reported to the sponsor or designee immediately and under no circumstance should this exceed 24 hours, as indicated in Section 10.3, Appendix 3. The investigator will submit any updated SAE data to the sponsor within 24 hours of it being available.

Investigators are not obligated to actively seek AEs or SAEs in former study subjects. However, if the investigator learns of any SAE, including a death, at any time after a subject has been discharged from the study, and he/she considers the event to be reasonably related to the study drug or study participation, the investigator must promptly notify the sponsor.

8.3.2. Method of Detecting AEs and SAEs

The method of recording, evaluating, and assessing causality of AEs and SAEs and the procedures for completing and transmitting SAE reports are provided in Section 10.3.

Care will be taken not to introduce bias when detecting AEs and/or SAEs. Open-ended and non-leading verbal questioning of the subject is the preferred method to inquire about AE occurrences.

8.3.3. Follow-up of AEs and SAEs

After the initial AE/SAE report, the investigator is required to proactively follow each subject at subsequent visits/contacts. All SAEs will be followed until resolution, until the condition stabilizes, until the event is otherwise explained, or until the subject is lost to follow-up (as defined in Section 7.2). Further information on follow-up procedures is provided in Section 10.3, Appendix 3.

8.3.4. Regulatory Reporting Requirements for SAEs

- Prompt notification by the investigator to the sponsor of a SAE is essential so that legal obligations and ethical responsibilities towards the safety of subjects and the safety of a study drug under clinical investigation are met.
- The sponsor has a legal responsibility to notify both the local regulatory authority and other regulatory agencies about the safety of a study drug under clinical investigation. The sponsor will comply with country-specific regulatory requirements relating to safety reporting to the regulatory authority, Institutional Review Boards (IRB)/Independent Ethics Committees (IEC), and investigators.
- For all studies except those utilizing medical devices investigator safety reports must be prepared for suspected unexpected serious adverse reactions (SUSAR) according to local regulatory requirements and sponsor policy and forwarded to investigators as necessary.
- An investigator who receives an investigator safety report describing a SAE or other specific safety information (e.g., summary or listing of SAEs) from the sponsor will

review and then file it along with the Investigator's Brochure and will notify the IRB/IEC, if appropriate according to local requirements.

8.3.5. Special Situations

Special situation reports include all reports of medication error, abuse, misuse, overdose, reports of adverse events associated with product complaints, and pregnancy reports regardless of an associated AE.

Medication error is any unintentional error in the prescribing, dispensing, or administration of a medicinal product while in the control of the health care provider, subject, or consumer.

Abuse is defined as persistent or sporadic intentional excessive use of a medicinal product by a subject.

Misuse is defined as any intentional and inappropriate use of a medicinal product that is not in accordance with the protocol instructions or the local prescribing information.

An overdose is defined as an accidental or intentional administration of a quantity of a medicinal product given per administration or cumulatively which is above the maximum recommended dose as per protocol which results in clinical signs and symptoms . In cases of a discrepancy in drug accountability, overdose will be established only when it is clear that the subject has taken the excess dose(s). Overdose cannot be established when the pharmacist cannot account for the discrepancy except in cases in which the investigator has reason to suspect that the additional dose(s) were administered to the subject.

Product complaint is defined as complaints arising from potential deviations in the manufacture, packaging, or distribution of the medicinal product.

8.3.5.1. Pregnancy

- Details of all pregnancies in female subjects will be collected after the start of study drug through 12 weeks after last study drug administration.
- If a pregnancy is reported, the investigator should inform the sponsor within 24 hours of learning of the pregnancy and should follow the procedures outlined in Section 10.4.
- Abnormal pregnancy outcomes (e.g., spontaneous abortion, fetal death, stillbirth, congenital anomalies, ectopic pregnancy) are considered SAEs.

8.3.5.2. Other Special Situations

- All other special situations must be reported to the sponsor within 24 hours of the investigator becoming aware of the situation. These reports must consist of situations that involve study drug and/or protocol-required concomitant medications, but do not apply to non-required concomitant medications.

- Special situations involving non-required concomitant medications do not need to be reported; however, for special situations that result in AEs due to a non-required concomitant medication, the AE should be reported.
- Any inappropriate use *of* concomitant medications prohibited by this protocol should not be reported as "misuse," but may be more appropriately documented as a protocol deviation.
- All clinical sequelae in relation to these special situation reports will be reported as AEs or SAEs at the same time. Details of the symptoms and signs, clinical management, and outcome will be reported, when available.

8.4. Pharmacodynamics

Pharmacodynamic parameters are not evaluated in this study.

8.5. Genetics

Human genetics are not evaluated in this study.

8.6. [Health Economics] OR [Medical Resource Utilization and Health Economics]

Health Economics/Medical Resource Utilization and Health Economics parameters are not evaluated in this study.

9. STATISTICAL CONSIDERATIONS

9.1. Statistical Hypotheses

9.1.1. Phase 2 Period

The primary endpoint for the Phase 2 period is HBsAg loss at Week 24. The null hypothesis is that there is no difference in the percentage of HBsAg loss at Week 24 between BRII-179 + PEG-IFN-α + NrtI and BRII-179 placebo + PEG-IFN- α +NrtI groups.

9.1.2. Phase 3 Period

The primary endpoint for the Phase 3 period is sustained HBsAg and HBV DNA loss (defined as HBsAg and HBV DNA undetectable for at least 24 weeks) after NrtI withdrawal. The null hypothesis is that there is no difference in the percentage of sustained HBsAg and HBV DNA loss between BRII-179+PEG-IFN- α + NrtI and BRII-179 placebo + PEG-IFN- α + NrtI groups.

9.2. Sample Size Determination

9.2.1. Phase 2 Period

The early futility analysis based on an intermediate outcome of efficacy using the HBsAg loss at Week 24, when about 106 subjects complete 24 weeks of BRII-179 or placebo in combination with PEG-IFN- α and NrtI treatment, will be conducted.

A sample size of 106 evaluable subjects provides 95% power to detect a difference of 20% in the percentage of HBsAg loss at Week 24 at the alpha level of 0.3 (1-sided), where 20% is considered as a clinically meaningful increase in HBsAg loss following adding BRII-179 to PEG-IFN- α and NrtI treatment. Everest project data suggest 24 weeks treatment of PEG-IFN- α + NrtI led to 26% of subjects with HBsAg loss (personal communication). In this sample size estimation, power calculation method is normal approximation and test method is Z-test (PASS, Version 15.0).

9.2.2. Phase 3 Period

Currently, there are limited data available regarding sustained HBsAg and HBV DNA loss (defined as HBsAg and HBV DNA undetectable for at least 24 weeks) after 24 weeks treatment of PEG-IFN- α + NrtI. Table 1 displays a range of sample size for sustained HBsAg and HBV DNA loss with PEG-IFN-α + NrtI treatment ranging from 22% to 35% and a minimum of clinically meaningful increase of 15% after adding BRII-179 to PEG-IFN- α + NrtI treatment.

Table 1. Sample Size Calculation for Four Scenarios of Sustained HBsAg Loss

Expected Sustained HBsAg and HBV DNA Loss (%)		Total Sample Size (Per Arm)	Power	Alpha (1-sided)
Placebo + PEG-IFN- α + NrtI	BRII-179 + PEG-IFN-α + NrtI			
22	37	420 (210)	90%	0.025
26	41	452 (226)		
**30**	**45**	**480 (240)**		
35	50	492 (246)		

A total sample size of 480 subjects, including 106 subjects from Phase 2 period, provides 90% power to detect an increase of 15% in sustained HBsAg and HBV DNA loss, using a 1-sided type I error rate of 2.5%, with the following assumptions:

Proportion of sustained HBsAg and HBV DNA loss in PEG-IFN- α + NrtI group is 30%.

A clinically meaningful increase in sustained HBsAg loss is 15% with add-on of BRII-179 treatment.

Non-binding stopping guideline for futility using a moderately aggressive Type II error spending function, implemented using the Lan-DeMets O'Brien-Fleming approximation spending function approach.

Two groups will be compared using Cochran-Mantel-Haenszel test, stratified at randomization by screening HBsAg level (3 categories: ≥0.05 to < 1 IU/mL, ≥1 to < 10 IU/mL, and ≥10 to <100 IU/mL).

Sample size calculation was conducted using Group Sequential Tests for Two Proportions module in PASS (Version 15.0), with a plan of 3 interim looks.

The sample size for Phase 3 period may be re-estimated based on a review of blinded, pooled HBsAg and HBV DNA loss rate at Week 24 after all subjects enrolled in Phase 2 complete 24 weeks follow-up.

9.3. Data Monitoring Guidelines for an Independent DSMB

An independent DSMB will review interim data and use pre-specified guidelines for early evidence of sufficient activity of BRII-179 that justifies continuing enrolment. This assessment will be made by the DSMB when Week 24 HBsAg and HBV DNA data for approximately 106 subjects are available.

If the trial advances to Phase 3, the DSMB will continue to review interim data on a regular basis and use pre-specified guidelines to determine clear evidence of efficacy for the primary outcome, and if so recommend unblinding of the trial results. Conversely, the

DSMB may recommend discontinuation of BRII-179 if futility assessments indicate that there is low probability that BRII-179 will achieve statistical significance for the primary endpoint of sustained HBsAg loss.

9.4. Rationale for Early Futility Analysis

The early futility analysis based on the intermediate outcome of efficacy using the HBsAg loss at Week 24 will be carried out for approximately 106 subjects completing 24 weeks treatment. The exact number may vary depending on the rate of enrolment and the timing of the DSMB meeting. This early futility analysis is based on Week 24 outcomes because too few primary endpoints of sustained HBsAg and HBV DNA loss expected when 106 subjects with only 24 weeks of follow-up are available. The sample size of 106 subjects provides 95% power to detect a difference of 20% in HBsAg loss (BRII-179 - placebo) at the 0.30 (1-sided) level of significance.

The rationale for a 1-sided alpha level of 0.30 and power 0.95 is based on previous work for 2-stage cancer trials, where like the current trial an intermediate outcome was used to assess early activity, and a definitive outcome was used to assess efficacy at the end of the trial. A re-analysis of 4 trials suggested a 1-sided significance level between 0.2 and 0.3 was optimal for making a good decision in the early futility assessment (Barthel, 2009). A subsequent paper focused on the potential for estimation bias in selected and stopped treatments and concluded that its degree was generally small (Choodari-Oskooei, 2013).

9.5. Interim Analyses Guidelines

Stopping guidelines for use by the DSMB are described below. More specific guidance may be specified in the SAP. Stopping boundaries will be derived to allow for 3 interim looks. The DSMB will be asked to recommend early termination or modification only when there is clear and substantial evidence of a treatment difference.

9.5.1. Early Assessment of Futility

When approximately 106 subjects have been enrolled and followed for 24 weeks, a futility assessment will be carried out. The criterion that will be used for the early futility assessment is that if BRII-179 is superior (i.e., 1-sided $p < 0.3$) to placebo in achieving HBsAg loss, then enrollment of the trial will expand to complete the phase 3 trial.

9.5.2. Monitoring Guidelines for the Primary Endpoint in Phase 3 Period

As a guideline, stopping boundaries will be provided to the DSMB to monitor the primary endpoint (sustained HBsAg and HBV DNA loss). For monitoring overwhelming benefit, the Lan-DeMets spending function analogue of the O’Brien-Fleming boundaries will be used. The Lan-DeMets boundary used will be chosen to preserve a 1-sided 0.025 level of significance. For computing the Lan-DeMets boundary, the information fraction at each of 3 interim analyses will be the number of subjects enrolled at the interim analysis, i.e., 120, 240, and 360 subjects completing 48 weeks of follow-up for those who

do not enter NrtI withdrawal follow-up period or 72 weeks of follow-up for those who enter NrtI withdrawal follow-up period, respectively.

Futility analyses will be carried out. The aim of these analyses will be to consider whether BRII-179 should be discontinued due to a low probability of achieving statistical significance for the primary endpoint of sustained HBsAg loss. Conditional power calculations of sustained HBsAg loss will be presented. In the futility analysis, the treatment effect for the future, as yet unobserved follow-up will be assumed as hypothesized in the study design (difference = 15%). Typical futility guidelines recommend stopping a trial when conditional power is below 10%-15%, with the higher value later in follow-up as measured by information time. These analyses will be presented to the DSMB by the unblinded statisticians.

9.6. Analysis Sets

The following analysis sets are defined:

Population	Description
All Subjects Screened	All subjects, for whom a record exists on the clinical database, will be included in All Subjects Screened.<br>It will be used for reporting study disposition, inclusion and exclusion criteria deviations, etc.
All Subjects Randomized	All subjects, who are randomized will be included in All Subjects Randomized. It will be used for reporting demographics, baseline characteristics, medical history, etc.
Full Analysis Set	All subjects, who are randomized and received at least one dose of BRII-179 or placebo, will be included in Full Analysis. It will be used as the primary analysis set of all efficacy endpoints.
Per Protocol Set	All subjects, who are randomized; and not identified with major protocol deviation that substantially affects the treatment effect of study medication; received all planned doses of BRII-179 or placebo; will be included in Per Protocol Set. It will be used as analysis set for key efficacy endpoint analyses. The analysis based on PP analysis set will be considered as sensitivity analysis.
Safety Analysis Set	All subjects randomly assigned to study drug and who take at least 1 dose of study drug will be included in Safety Analysis Set. It will be used for reporting safety parameters.

9.7. Statistical Analyses

This section outlines the general analytic strategy for the study. The statistical analysis plan (SAP) will be finalized prior to Database Lock (DBL) and it will include a more detailed description of the statistical analyses described in this section. This section is a

summary of the planned statistical analyses of the most important endpoints including primary and key secondary endpoints.

9.7.1. General considerations

Unless otherwise stated, the following rule will apply:

Summaries and displays will be presented by treatment group.

For categorical variables, the frequency and percentage will be applied as descriptive statistics.

For continuous variables, the mean, standard variation, median, inter-quartiles, and ranges will be applied as descriptive statistics.

Specific algorithm regarding how missing HBsAg and HBV DNA values will be handled in deriving the primary efficacy endpoints and sustained HBsAg loss will be detailed in the statistical analysis plan.

Unless specified otherwise, missing values will be handled using the Data-As-Observed (DAO) approach, that is, any missing value will be excluded from the analysis, except for the baseline value, which will be replaced with the latest pre-treatment result, if available.

Only scheduled visits will be presented of summaries; unscheduled visits will be listed but will not be summarized.

9.7.2. Analysis of the Primary Efficacy Endpoint for Phase 2

For evaluating graduation criterion (ie, expanding the study to Phase 3 period), the primary efficacy endpoint of the phase 2 trial, percentage of HBsAg loss at Week 24 (from Day 155 to Day 182), will be analysed based on Cochran-Mantel-Haenszel method, stratified at randomization by screening HBsAg level (3 categories). The differences in the percentage of HBsAg loss and associated 95% confidence interval will be presented. If one sided p-value is < 0.3, the trial will continue to Phase 3. Subjects with missing HBsAg value at Week 24 will be considered as not achieving HBsAg loss at Week 24.

9.7.3. Analysis of the Primary Efficacy Endpoint for Phase 3

The primary efficacy endpoint of the phase 3 trial, percentage of sustained HBsAg and HBV DNA loss defined as undetectable HBsAg and HBV DNA for at least 24 weeks after NrtI withdrawal. Subjects who do not meet NrtI withdrawal criteria or are retreated with NrtI after NrtI withdrawal will be classified as not achieving sustained HBsAg and HBV DNA loss. The primary endpoint will be analyzed based on Cochran-Mantel-Haenszel method, stratified at randomization by screening HBsAg level (3 categories: ≥0.05 to < 1 IU/mL, ≥1 to < 10 IU/mL, and ≥10 to <100 IU/mL). The differences in the percentage of sustained HBsAg loss and associated 95% confidence interval will be presented.

9.7.4. Analyses of Secondary Efficacy Endpoints and Subgroups

Percentage of subjects meeting NrtI withdrawal criteria and other secondary endpoints assessed as proportion will be analysed in the same way as for the primary endpoint.

Time to event endpoints will be compared using stratified log-rank test and continuous endpoints will be compared using Wilcoxon ranks sum test.

Additionally, subgroup analysis by age, gender, and HBsAg level at baseline (3 categories: >0.05 to < 1 IU/mL, ≥1 to < 10 IU/mL, and ≥10 to <100 IU/mL) will be conducted with respect to the primary endpoint.

9.7.5. Analysis of Safety Outcome

The Safety Analysis population will be used for clinical review of safety relevant parameters.

All AEs will be coded using the Medical Dictionary for Regulatory Activities (MedDRA). Treatment-Emergent Adverse Events (TEAEs) are defined as any AEs with an onset date of on or after study drug start date and no later than 30 days after permanent discontinuation of study drug. Number and percentage of TEAEs will be summarized by cohort.

Graded laboratory abnormalities will be defined using NCI-CTCAE Version 5.0 grading scale. The incidence of treatment-emergent laboratory abnormalities, defined as values that increase at least 1 toxicity grade from baseline at any postbaseline timepoint up to 30 days after permanent discontinuation of study drug will be summarized by cohort.

10. SUPPORTING DOCUMENTATION AND OPERATIONAL CONSIDERATIONS

10.1. Appendix 1: Regulatory, Ethical, and Study Oversight Considerations

10.1.1. Regulatory and Ethical Considerations

- This study will be conducted in accordance with the protocol and with the following:
  - Consensus ethical principles derived from international guidelines including the Declaration of Helsinki and Council for International Organizations of Medical Sciences (CIOMS) International Ethical Guidelines
  - Applicable ICH Good Clinical Practice (GCP) Guidelines
  - Applicable laws and regulations
- The protocol, protocol amendments, ICF, Investigator Brochure, and other relevant documents (e.g., advertisements) must be submitted to an IRB/IEC by the investigator and reviewed and approved by the IRB/IEC before the study is initiated.
- Any amendments to the protocol will require IRB/IEC approval before implementation of changes made to the study design, except for changes necessary to eliminate an immediate hazard to study subjects.
- The investigator will be responsible for the following:
  - Providing written summaries of the status of the study to the IRB/IEC annually or more frequently in accordance with the requirements, policies, and procedures established by the IRB/IEC
  - Notifying the IRB/IEC of SAEs or other significant safety findings as required by IRB/IEC procedures
  - Providing oversight of the conduct of the study at the site and adherence to requirements of 21 CFR, ICH guidelines, the IRB/IEC, European regulation 536/2014 for clinical studies (if applicable), and all other applicable local regulations

10.1.2. Financial Disclosure

Investigators and sub-investigators will provide the sponsor with sufficient, accurate financial information as requested to allow the sponsor to submit complete and accurate financial certification or disclosure statements to the appropriate regulatory authorities. Investigators are responsible for providing information on financial interests during the course of the study and for 1 year after completion of the study.

10.1.3. Informed Consent Process

- The investigator or his/her representative will explain the nature of the study to the subject or his/her legally authorized representative and answer all questions regarding the study.

- Subjects must be informed that their participation is voluntary. Subjects or their legally authorized representative will be required to sign a statement of informed consent that meets the requirements of 21 CFR 50, local regulations, ICH guidelines, Health Insurance Portability and Accountability Act (HIPAA) requirements, where applicable, and the IRB/IEC or study center.
- The medical record must include a statement that written informed consent was obtained before the subject was enrolled in the study and the date the written consent was obtained. The authorized person obtaining the informed consent must also sign the ICF.
- Subjects must be re-consented to the most current version of the ICF(s) during their participation in the study.
- A copy of the ICF(s) must be provided to the subject or the subject's legally authorized representative.

10.1.4. Data Protection

- Subjects will be assigned a unique identifier by the sponsor. Any subject records or datasets that are transferred to the sponsor will contain the identifier only; subject names or any information which would make the subject identifiable will not be transferred.
- The subject must be informed that his/her personal study-related data will be used by the sponsor in accordance with local data protection law. The level of disclosure must also be explained to the subject who will be required to give consent for their data to be used as described in the informed consent
- The subject must be informed that his/her medical records may be examined by Clinical Quality Assurance auditors or other authorized personnel appointed by the sponsor, by appropriate IRB/IEC members, and by inspectors from regulatory authorities.

10.1.5. Data Quality Assurance

- All relevant subject data relating to the study will be recorded on printed or electronic CRF unless transmitted to the sponsor or designee electronically (e.g., laboratory data). The investigator is responsible for verifying that data entries are accurate and correct by physically or electronically signing the CRF.
- The investigator must maintain accurate documentation (source data) that supports the information entered in the CRF.
- The investigator must permit study-related monitoring, audits, IRB/IEC review, and regulatory agency inspections and provide direct access to source data documents.
- Monitoring details describing strategy (e.g., risk-based initiatives in operations and quality such as Risk Management and Mitigation Strategies and Analytical Risk-Based Monitoring), methods, responsibilities and requirements, including handling

of noncompliance issues and monitoring techniques (central, remote, or on-site monitoring) are provided in the Monitoring Plan.

- The sponsor or designee is responsible for the data management of this study including quality checking of the data.
- The sponsor assumes accountability for actions delegated to other individuals (e.g., Contract Research Organizations).
- Study monitors will perform ongoing source data verification to confirm that data entered into the CRF by authorized site personnel are accurate, complete, and verifiable from source documents; that the safety and rights of subjects are being protected; and that the study is being conducted in accordance with the currently approved protocol and any other study agreements, ICH GCP, and all applicable regulatory requirements.
- Records and documents, including signed ICFs, pertaining to the conduct of this study must be retained by the investigator. No records may be destroyed during the retention period without the written approval of the sponsor. No records may be transferred to another location or party without written notification to the sponsor.

10.1.6. Source Documents

- Source documents provide evidence for the existence of the subject and substantiate the integrity of the data collected. Source documents are filed at the investigator's site.
- Data reported on the CRF or entered in the eCRF that are transcribed from source documents must be consistent with the source documents or the discrepancies must be explained. The investigator may need to request previous medical records or transfer records, depending on the study. Also, current medical records must be available.
- Source data refer to all information recorded in the original record of certified copy of the clinical study, including clinical findings, observation results and other relevant activity records needed for reconstruction and evaluation of the clinical study.

10.1.7. Study and Site Start and Closure

The study start date is the date on which the clinical study will be open for recruitment of subjects.

The sponsor designee reserves the right to close the study site or terminate the study at any time for any reason at the sole discretion of the sponsor. Study sites will be closed upon study completion. A study site is considered closed when all required documents and study supplies have been collected and a study-site closure visit has been performed.

The investigator may initiate study-site closure at any time, provided there is reasonable cause and sufficient notice is given in advance of the intended termination.

Reasons for the early closure of a study site by the sponsor or investigator may include but are not limited to:

- Failure of the investigator to comply with the protocol, the requirements of the IRB/IEC or local health authorities, the sponsor's procedures, or GCP guidelines
- Inadequate recruitment of subjects by the investigator
- Discontinuation of further study drug development

If the study is prematurely terminated or suspended, the sponsor shall promptly inform the Investigators, the IECs/IRBs, the regulatory authorities, and any contract research organization(s) used in the study of the reason for termination or suspension, as specified by the applicable regulatory requirements. The Investigator shall promptly inform the subject and should assure appropriate subject therapy and/or follow-up.

10.1.8. Publication Policy

The sponsor intends to publish the results of clinical trials regardless of outcome. Results may be published in peer-reviewed journals and/or presented at scientific congresses. The sponsor will comply with the requirements for publication and disclosure of clinical trial information. In accordance with standard editorial and ethical practice, the sponsor will generally support publication of multicenter studies only in their entirety and not as individual site data. Authorship will be determined by mutual agreement based upon International Committee of Medical Journal Editors criteria and guidelines [ICMJE, 2018]. Individuals who do not meet authorship criteria do not warrant named authorship. Nonauthor contributors may be acknowledged in the manuscript when published or recognized in a manner consistent with the target journal policy.

10.2. Appendix 2: Clinical Laboratory Tests

The tests detailed in Table 2 will be performed by the central laboratory. Urine pregnancy test will be performed by the local or central laboratory.

Investigators must document their review of each laboratory safety report.

Additional tests may be performed at any time during the study as determined necessary by the investigator or required by local regulations.

Table 2: Protocol-Required Safety Laboratory Assessments

**Hematology**	
Complete blood count with differential, including red blood cell count, hemoglobin, hematocrit, white blood cell count, count and percentage of neutrophils, lymphocytes, eosinophils, basophils, and monocytes, and platelet count	
**Chemistry**	
Albumin	Estimated glomerular filtration rate (eGFR)
Blood urea nitrogen (BUN)	Gamma glutamyl transferase (GGT)
Calcium	Glucose
Carbon dioxide/bicarbonate	Lactate dehydrogenase (LDH)
Chloride	Potassium
Creatine kinase	Sodium
Creatinine	Uric acid
**Liver Function Tests**	
Alkaline phosphatase (ALP)	Aspartate aminotransferase (AST)
Alanine aminotransferase (ALT)	Bilirubin (total and direct)
**Coagulation Parameters**	
International normalized ratio (INR)	Prothrombin time (PT)
**Urinalysis**	
Glucose	Proteins
Red blood cells (RBCs)	
**Pregnancy Testing**	
Beta-human chorionic gonadotropin (WOCBP only)	Urine pregnancy test
**Thyroid Panel**	
Thyroid stimulating hormone (TSH)[1]	

[1] Thyroid function will be tested every 12 weeks during PEG-IFN-α dosing: Screening, Week 12, and Week 24.

10.3. Appendix 3: Adverse Events: Definitions and Procedures for Recording, Evaluating, Follow-up, and Reporting

10.3.1. Definition of AE

**AE Definition**
• An AE is any untoward medical occurrence in a patient or clinical study subject, temporally associated with the use of study drug, whether or not considered related to the study drug.<br>• NOTE: An AE can therefore be any unfavorable and unintended sign (including an abnormal laboratory finding), symptom, or disease (new or exacerbated) temporally associated with the use of study drug.

**Events Meeting the AE Definition**
• Any abnormal laboratory test results (hematology, clinical chemistry, or urinalysis) or other safety assessments (e.g., ECG, radiological scans, vital signs measurements), including those that worsen from baseline, considered clinically significant in the medical and scientific judgment of the investigator (i.e., not related to progression of underlying disease).<br>• Exacerbation of a chronic or intermittent pre-existing condition including either an increase in frequency and/or intensity of the condition.<br>• New conditions detected or diagnosed after study drug administration even though it may have been present before the start of the study.<br>• Signs, symptoms, or the clinical sequelae of a suspected drug-drug interaction.<br>• Signs, symptoms, or the clinical sequelae of a suspected overdose of either study drug or a concomitant medication. Overdose per se will not be reported as an AE/SAE unless it is an intentional overdose taken with possible suicidal/self-harming intent. Such overdoses should be reported regardless of sequelae.

**Events NOT Meeting the AE Definition**
• Any clinically significant abnormal laboratory findings or other abnormal safety assessments which are associated with the underlying disease, unless judged by the investigator to be more severe than expected for the subject's condition.<br>• The disease/disorder being studied or expected progression, signs, or symptoms of the disease/disorder being studied, unless more severe than expected for the subject's condition.

• Medical or surgical procedure (e.g., endoscopy, appendectomy): the condition that leads to the procedure is the AE.<br>• Situations in which an untoward medical occurrence did not occur (social and/or convenience admission to a hospital).<br>• Anticipated day-to-day fluctuations of pre-existing disease(s) or condition(s) present or detected at the start of the study that do not worsen.

10.3.2. Definition of SAE

If an event is not an AE per definition above, then it cannot be an SAE even if serious conditions are met (e.g., hospitalization for signs/symptoms of the disease under study, death due to progression of disease).

**A SAE is defined as any untoward medical occurrence that, at any dose:**
**a. Results in death**
**b. Is life-threatening**<br>The term 'life-threatening' in the definition of 'serious' refers to an event in which the subject was at risk of death at the time of the event. It does not refer to an event, which hypothetically might have caused death, if it were more severe.
**c. Requires inpatient hospitalization or prolongation of existing hospitalization**<br>• In general, hospitalization signifies that the subject has been detained (usually involving at least an overnight stay) at the hospital or emergency ward for observation and/or treatment that would not have been appropriate in the physician's office or outpatient setting. Complications that occur during hospitalization are AEs. If a complication prolongs hospitalization or fulfills any other serious criteria, the event is serious. When in doubt as to whether "hospitalization" occurred or was necessary, the AE should be considered serious.<br>• Hospitalization for elective treatment of a pre-existing condition that did not worsen from baseline is not considered an AE.
**d. Results in persistent disability/incapacity**<br>• The term disability means a substantial disruption of a person's ability to conduct normal life functions.<br>• This definition is not intended to include experiences of relatively minor medical significance such as uncomplicated headache, nausea, vomiting, diarrhea, influenza, and accidental trauma (e.g., sprained ankle) which may interfere with or prevent everyday life functions but do not constitute a substantial disruption.

**e. Is a congenital anomaly/birth defect**
**f. Other situations:**<br>• Medical or scientific judgment should be exercised in deciding whether SAE reporting is appropriate in other situations such as important medical events that may not be immediately life-threatening or result in death or hospitalization but may jeopardize the subject or may require medical or surgical intervention to prevent one of the other outcomes listed in the above definition. These events should usually be considered serious.<br>• Examples of such events include invasive or malignant cancers, intensive treatment in an emergency room or at home for allergic bronchospasm, blood dyscrasias or convulsions that do not result in hospitalization, or development of drug dependency or drug abuse.

10.3.3. Recording and Follow-Up of AE and/or SAE

**AE and SAE Recording**
• When an AE/SAE occurs, it is the responsibility of the investigator to review all documentation (e.g., hospital progress notes, laboratory reports, and diagnostics reports) related to the event.<br>• The investigator will then record all relevant AE/SAE information in the CRF.<br>• It is **not** acceptable for the investigator to send photocopies of the subject's medical records to the Sponsor in lieu of completion of the AE/SAE CRF page.<br>• There may be instances when copies of medical records for certain cases are requested by the Sponsor. In this case, all subject identifiers, with the exception of the subject number, will be redacted on the copies of the medical records before submission to the Sponsor.<br>• The investigator will attempt to establish a diagnosis of the event based on signs, symptoms, and/or other clinical information. Whenever possible, the diagnosis (not the individual signs/symptoms) will be documented as the AE/SAE.
**Assessment of Intensity**
The investigator will make an assessment of intensity for each AE and SAE reported during the study based on the National Cancer Institute Common Terminology Criteria for Adverse Events (NCI-CTCAE) Version 5.0.
**Assessment of Causality**
• The investigator or a qualified assistant investigator is obligated to assess the relationship between study drug and each occurrence of each AE/SAE.<br>• Relationship between AEs and the study drug: "related" should be entered when there is either (a) a *reasonable possibility* that the AE/SAE is associated

with study drug **or** (b) no reasonable alternative explanation can be found. Otherwise, the relationship between AEs and the study drug should be classified as "unrelated".

- A "reasonable possibility" of a relationship conveys that there are facts, evidence, and/or arguments to suggest a causal relationship, rather than a relationship cannot be ruled out.
- The investigator will use clinical judgment to determine the relationship.
- Alternative causes, such as underlying disease(s), concomitant therapy, and other risk factors, as well as the temporal relationship of the event to study drug administration will be considered and investigated.
- The investigator will also consult the Investigator's Brochure (IB) and/or Product Information, for marketed products, in his/her assessment.
- For each AE/SAE, the investigator **must** document in the medical notes that he/she has reviewed the AE/SAE and has provided an assessment of causality.
- There may be situations in which an SAE has occurred and the investigator has minimal information to include in the initial report. However, it is very important that the investigator always make an assessment of causality for every event before the initial transmission of the SAE data.
- The investigator may change his/her opinion of causality in light of follow-up information and send a SAE follow-up report with the updated causality assessment.
- The causality assessment is one of the criteria used when determining regulatory reporting requirements.

Follow-up of AEs and SAEs

- The investigator is obligated to perform or arrange for the conduct of supplemental measurements and/or evaluations as medically indicated or as requested by the Sponsor to elucidate the nature and/or causality of the AE or SAE as fully as possible. This may include additional laboratory tests or investigations, histopathological examinations, or consultation with other health care professionals.
- If a subject dies during participation in the study or during a recognized follow-up period, the investigator will provide the Sponsor with a copy of any post-mortem findings including histopathology.
- New or updated information will be recorded in the originally completed CRF.
- The investigator will submit any updated SAE data to the Sponsor within 24 hours of receipt of the information.

10.3.4. Reporting of SAEs

SAE Reporting to the Sponsor via a Paper SAE Reporting Form
• The investigator must complete a paper SAE report form and send to the Sponsor or its representative within 24 hours of the investigator's knowledge of the event.<br>• Contacts for SAE reporting can be found on the SAE Reporting Form.

10.3.5. Reporting of Laboratory Events of Clinical Interest

All laboratory ECIs must be reported to the Sponsor or its representative within 24 hours of the investigator's knowledge of the event. Contact for laboratory ECIs reporting can be found on the ECI Reporting Form.

The laboratory ECI will also be evaluated by the investigator to determine if the event meets criteria for an AE. If AE criteria are met, all clinical sequelae in relation to these laboratory ECIs will be reported as AEs or SAEs, if applicable.

10.4. Appendix 4: Contraceptive Requirements and Collection and Reporting of Pregnancy Information

Contraceptive Requirements:

WOCBP may be included in this study and include any female subject who has experienced menarche and who is not post-menopausal or permanently sterilized (eg, bilateral tubal occlusion, hysterectomy, or bilateral salpingectomy).

WOCBP must be willing to use highly effective methods of contraception 14 days before dose, throughout study participation, and for 12 weeks after last dose of study drug administration. Highly effective methods of birth control result in a low failure rate (ie, less than 1% per year). Birth control methods which are considered highly effective include:

- Established use of combined (estrogen and progestogen containing) oral, intravaginal, or transdermal hormonal methods of contraception associated with inhibition of ovulation **OR** established use of progestogen-only oral, injectable, or implantable hormonal methods of contraception associated with inhibition of ovulation. It is not currently known whether study drug will impact the effectiveness of hormonal contraceptive methods; therefore, it is recommended to use an additional form of contraception (ie, barrier method) throughout the study and for 12 weeks after last study drug administration.
- Placement of an intrauterine device
- Placement of an intrauterine hormone-releasing system
- Surgical sterilization of male partner (with the appropriate post-vasectomy documentation of the absence of sperm in the ejaculate; for female subjects on the study, the vasectomized male partner should be the sole partner for that subject)
- True sexual abstinence, when in line with the preferred and usual lifestyle of the subject. Periodic abstinence (e.g., calendar, ovulation, symptothermal, post-ovulation methods) and withdrawal are not acceptable methods of contraception. Abstinent subjects have to agree to use 1 of the above-mentioned contraceptive methods, if they start sexual relationships during the study and for up to 12 weeks after last dose of study drug administration, or for as long as the subject is followed on study, whichever is longer.
- Barrier method in combination with hormonal contraceptive, as described above

Post-menopausal status is defined as 12 months with no menses without an alternative medical cause.

Collection of Pregnancy Information:

Female Subjects who become pregnant

- The investigator will collect pregnancy information on any female subject who becomes pregnant while participating in this study. The initial information will be recorded on the appropriate form and submitted to the sponsor within 24 hours of learning of a subject's pregnancy.
- The subject will be followed to determine the outcome of the pregnancy. The investigator will collect follow-up information on the subject and the neonate and the information will be forwarded to the sponsor. Generally, follow-up will not be required for longer than 6 to 8 weeks beyond the estimated delivery date. Any termination of pregnancy will be reported, regardless of fetal status (presence or absence of anomalies) or indication for the procedure.
- While pregnancy itself is not considered to be an AE or SAE, any pregnancy complication or elective termination of a pregnancy for medical reasons will be reported as an AE or SAE.
- A spontaneous abortion (occurring at <22 weeks gestational age) or still birth (occurring at >22 weeks gestational age) is always considered to be an SAE and will be reported as such.
- Any post-study pregnancy related SAE considered reasonably related to the study drug by the investigator will be reported to the sponsor as described in Section 8.3.4. While the investigator is not obligated to actively seek this information in former study subjects, he or she may learn of an SAE through spontaneous reporting.
- Any female subject who becomes pregnant while participating in the study will discontinue study drug.

10.5. Appendix 5: Assessments in Subjects Meeting Laboratory ECI Criteria

Additional assessment will be performed in subjects who experience ALT elevations meeting laboratory ECI criteria (Section 8.2.8) during the NrtI Discontinuation Monitoring Period. Following the occurrence of event of ECI, the following assessments in Table 3 will be performed one time, as soon as possible, and no later than two weeks after the initial ALT value meeting criteria for a laboratory ECI. The full panel of assessments should only be performed once; individual assessments may be repeated per investigator discretion, as needed.

Table 3 Assessments in Subjects Meeting Laboratory ECI Criteria

**Hematology**	
Complete blood count with differential, including red blood cell count, hemoglobin, hematocrit, white blood cell count, count and percentage of neutrophils, lymphocytes, eosinophils, basophils, and monocytes, and platelet count	
**Chemistry**	
Albumin	Estimated glomerular filtration rate (eGFR)
Blood urea nitrogen (BUN)	Gamma glutamyl transferase (GGT)
Calcium	Glucose
Carbon dioxide/bicarbonate	Lactate dehydrogenase (LDH)
Chloride	Potassium
Creatine kinase	Sodium
Creatinine	Uric acid
**Liver Function Tests**	
Alkaline phosphatase (ALP)	Aspartate aminotransferase (AST)
Alanine aminotransferase (ALT)	Bilirubin (total and direct)
**Coagulation Parameters**	
International normalized ratio (INR)	Prothrombin time (PT)
**Extended Hepatic Panel**	
Quantitative HBsAg	Ferritin
Quantitative HBV DNA	Aldolase
Quantitative HBeAg	Blood phosphatidylethanol
HAV antibody IgM	Urinary ethyglucuronide
HCV antibody and HCV RNA	Anti-nuclear antibodies
HDV antibody or RNA	Anti-smooth muscle antibodies
HEV antibody IgM	Anti-LKM1 antibody
HDV antibody or RNA	Anti-mitochondrial antibodies
Epstein-Barr Virus antibodies, IgM and IgG	Anti-SLA antibodies
Cytomegalovirus antibodies, IgM and IgG	
Ceruloplasmin	
**Imaging**	
Abdominal ultrasound, including Doppler flow, if applicable; CT or MRI acceptable in place of ultrasound if clinically indicated	
**Focused Medical History**	

Use of any potentially hepatotoxic concomitant medications, including over the counter medications and herbal remedies	Alcohol consumption and drugs of abuse
Exposure to other potentially hepatotoxic agents including any work-related exposures (eg. solvents, pesticides, etc.)	Exercise history

CT = computed tomography; HAV = hepatitis A virus; HCV = hepatitis C virus; HDV = hepatitis D virus; HEV = hepatitis E virus; IgG = immunoglobulin G antibody; IgM = immunoglobulin M antibody; LKM1 = liver/kidney microsome-1 antibody; MRI = magnetic resonance imaging; PCR = polymerase chain reaction; RNA = ribonucleic acid; SLA = soluble liver antigen.

11. REFERENCES

Barthel FM-S, Parmar MKB, Royston P. How do multi-stage, multi-arm trials compare to the traditional two-arm parallel group design – a reanalysis of 4 trials. Trials 2009; 10:21, doi:.10.1186/1745-6215-10-21.

Berg T, Simon KG, Mauss S, Schott E, Heyne R, Klass DM, Eisenbach C, Welzel TM, Zachoval R, Felten G, Schulze-Zur-Wiesch J, Cornberg M, Op den Brouw ML, Jump B, Reiser H, Gallo L, Warger T, Petersen J; FINITE CHB study investigators. Long-term response after stopping tenofovir disoproxil fumarate in non-cirrhotic HBeAg-negative patients - FINITE study. *Journal of Hepatology*. 2017 Nov;67(5):918-924.

Chang, M. L., Liaw, Y. F., and Hadziyannis, S. J. (2015). Systematic review: cessation of long-term nucleos(t)ide analogue therapy in patients with hepatitis B e antigen-negative chronic hepatitis B. *Alimentary Pharmacology & Therapeutics*, 2015; *42*(3): 243–257.

Choodari-Oskooei B, Parmar MKB, Royston P, Bowden J. Impact of lack-of-benefit stopping rules on treatment effect estimates of two-arm multi-stage (TAMS) trials with time to event outcome. Trials 2013, 14:23, http://www.trialsjournal.com/content/14/1/23.

Cornberg M, Lok AS, Terrault NA, Zoulim F, and the 2019 EASL-AASLD HBV Treatment Endpoints Conference Faculty. Guidance for design and endpoints of clinical trials in chronic hepatitis B – Report from the 2019 EASL-AASLD HBV Treatment Endpoints Conference. *Journal of Hepatology*. 2020; 72(3): 539-337.

Ferrari C. HBV and the immune response. *Liver Int*. 2015; 35 (Suppl. 1): 121–128.

Hoa PT, Huy NT, Thu le T, Nga CN, Nakao K, Eguchi K, Chi NH, Hoang BH, Hirayama K. Randomized controlled study investigating viral suppression and serological response following pre-S1/pre-S2/S vaccine therapy combined with lamivudine treatment in HBeAg-positive patients with chronic hepatitis B. *Antimicrob Agents Chemother*. 2009; 53:5134-40.

Jeng WJ, Chen YC, Chien RN, Sheen IS, Liaw YF. Incidence and predictors of HBsAg seroclearance after cessation of nucleos(t)ide analogue therapy in HBeAg negative chronic hepatitis B. *Hepatology*. 2018; 68(2): 425-434.

Kondo Y, Ninomiya M, Kakazu E, Kimura O, Shimosegawa T. Hepatitis B surface antigen could contribute to the immunopathogenesis of hepatitis B virus infection. *ISRN Gastroenterol*. 2013;2013:935295.

Konerman MA, Lok AS. Interferon Treatment for Hepatitis B. *Clinics in Liver Disease*. 2016; 20(4): 645-665.

Kramvis A. Genotypes and genetic variability of hepatitis B virus. *Intervirology* 2014; 57(3-4): 141-150.

Lebossé F, Testoni B, Fresquet J, Facchetti F, Galmozzi E, Fournier M, Hervieu V, Berthillon P, Berby F, Bordes I, Durantel D, Levrero M, Lampertico P, Zoulim F. Intrahepatic innate immune response pathways are downregulated in untreated chronic hepatitis B. *J Hepatol*. 2017;66(5):897-909.

Lok AS, McMahon BJ, Brown RS, Wong JB, Ahmed AT, Farah W, Almasri J, Alahdab F, Benkhadra K, Mouchli MA, Singh S, Mohamed EA, Abu Dabrh AM, Prokop LJ, Wang Z, Murad MH, Mohammed K. Antiviral therapy for chronic hepatitis B viral infection in adults: A systematic review and meta-analysis. *Hepatology* 2016; 63(1):284-306.

Marcellin P, Ahn SH, Ma X, Caruntu FA, Tak WY, Elkashab M, et al. Combination of tenofovir disoproxil fumarate and peginterferon α-2a increases loss of hepatitis B surface antigen in patients with chronic hepatitis B. Gastroenterology 2016;150(1):134–44.

Michler T, Kosinska AD, Festag J, Bunse T, Su J, Ringelhan M, Imhof H, Grimm D, Steiger K, Mogler C, Heikenwalder M, Michel ML, Guzman CA, Milstein S, Sepp-Lorenzino L, Knolle P, Protzer U. Knockdown of Virus Antigen Expression Increases Therapeutic Vaccine Efficacy in High-Titer Hepatitis B Virus Carrier Mice. Gastroenterology. 2020 May;158(6):1762-1775.

Papatheodoridi M, Hadziyannis E, Berby F, Zachou K, Testoni B, Rigopoulou E, Gatselis NK, Lyberopoulou A, Vlachogiannakos I, Manolakopoulos S, Dalekos GN, Zoulim F, Papatheodoridis GV. Predictors of hepatitis B surface antigen loss, relapse and retreatment after discontinuation of effective oral antiviral therapy in noncirrhotic HBeAg-negative chronic hepatitis B. *J Viral Hepat.* 2020; 27(2): 118-126.

Perrillo, R. "Benefits and risks of IFN therapy for hepatitis B." *Hepatology*. 2009; 49(5): S103-111.

Ren H, Hu P, Chen XY, Gong GZ, Shang J, Zhang WH, et al. Switching to PegIFNα-2a in NUC treated CHB patients(NEW SWITCH Study)：comparison 48 and 96 weeks. Hepatol Int 2016;10(Suppl 1):S43–4.

Roggendorf H, Krawczyk A, Lindemann M, Shouval D, Michler T, Roggendorf M, Gerken G. Induction of functional control in chronic hepatitis B patients with low level HBsAg using a combination of a PreS1/S2/S HBV vaccine (Sci-B-Vac®) and a nucleoside analogue. *J Infect Dis Ther*. 2019; 7:389-393.

Schweitzer A, Horn J, Mikolajczyk RT, Krause G, Ott JJ. Estimations of worldwide prevalence of chronic hepatitis B virus infection: a systematic review of data published between 1965 and 2013. *Lancet* 2015; 386(10003): 1546-1555.

Stanaway JD, Flaxman AD, Naghavi M, Fitzmaurice C, Vos T, Abubakar I, Abu-Raddad LJ, Assadi R, Bhala N, Cowie B, Forouzanfour MH, Groeger J, Hanafiah KM, Jacobsen KH, James SL, MacLachlan J, Malekzadeh R, Martin NK, Mokdad AA, Mokdad AH, Murray CJL, Plass D, Rana S, Rein DB, Richardus JH, Sanabria J, Saylan M, Shahraz S, So S, Vlassov V, Weiderpass E, Wiersma ST, Younis M, Yu C, Zaki M, Cooke GS. The

global burden of viral hepatitis from 1990 to 2013: findings from the Global Burden of Disease Study 2013. *Lancet* 2016; 388(10049): 1081-1088.

Trepo CHL, Chan, Lok A. Hepatitis B virus infection. *Lancet* 2014;384(9959): 2053-2063.

van Bömmel F, Berg T. Stopping long-term treatment with nucleos(t)ide analogues is a favourable option for selected patients with HBeAg-negative chronic hepatitis B. *Liver International*. 2018;38 Suppl 1:90-96.

Wang S, Chen Z, Hu C, Qian F, Cheng Y, Wu M, Shi B, Chen J, Hu Y, Yuan Z. Hepatitis B virus surface antigen selectively inhibits TLR2 ligand-induced IL-12 production in monocytes/macrophages by interfering with JNK activation. *J Immunol*. 2013 May 15;190(10):5142-51.

Wong TCL, Fung JYY, Chok KSH, Cheung TT, Chan ACY, Dai WC, Ng KKC, Chan SC, Lo, CM. Hepatitis B vaccination in patients receiving oral antiviral therapy without hepatitis B immunoglobulin after liver transplant. *Transplant Proc*. 2018; 50:3681-3688.

Yao CC, Hung CH, Hu TH, Lu SN, Wang JH, Lee CM, and Chen CH. Incidence and predictors of HBV relapse after cessation of nucleoside analogues in HBeAg-negative patients with HBsAg ≤ 200 IU/mL. *Scientific Reports*. 2017; 7(1): 1839.

Ze-Qian Wu, Dong-Ying Xie, Lei Fu, Jia Wei, Jia Shang, Qing He, Wen-hua Zhang, Guang-yu Huang, Yanzhong Peng, Ye Gu, Jia-bin Li, Ying Guo, Yu-juan Guan, Jia-wei Geng, Huan-wei Zheng, Wen-jun Gao, Wen-jing Zhao, Ying-jun Tian, Yi-lan Zeng, Ren Chen, Shengwang Gu, Zhi-liang Gao. Functional cure based on pegylated interferon ? in long-term nucleoside analog suppressed HBeAg negative chronic hepatitis B: a multicenter real-world study (Everest project in China), an interim report. *Journal of Hepatology* 2020; 73: S653–S915.

Zhu D, Liu L, Yang D, Fu S, Bian Y, Sun Z, He J, Su L, Zhang L, Peng H, Fu YX. Clearing Persistent Extracellular Antigen of Hepatitis B Virus: An Immunomodulatory Strategy To Reverse Tolerance for an Effective Therapeutic Vaccination. *J Immunol*. 2016; 196(7): 3079-87.

Title

Protocol Title: A Multicenter, Randomized, Double-Blind, Placebo-Controlled, Parallel-Group Study to Investigate the Efficacy and Safety of BRII-179 (VBI-2601) for the Treatment of Chronic Hepatitis B Virus (HBV) Infection

Protocol Number: BRII-179-002

Compound: BRII-179 (VBI-2601)

Study Phase: Phase 2

Short Title: Short Title: Study to Investigate Efficacy and Safety of BRII-179 (VBI-2601) for the Treatment of Chronic HBV Infection

Sponsor: Brii Biosciences Limited

Co-Sponsor/ Registration Agent: Brii Biosciences (Beijing) Ltd.

Legal Registered Address: Room 301, 3/F, Building 7,
No. 1 Yongtaizhuang North Road，
Tiandilinfeng (Dongsheng Area)
Haidian District, Beijing 100192,
China

Protocol Version: Version 3.0

Approval Date: 3 August 2021

Sponsor Signatory

[redacted]

Medical Monitor Name and Contact Information will be provided separately.

INVESTIGATOR AGREEMENT PAGE

For protocol number BRII-179-002

I confirm agreement to conduct the study in compliance with the protocol, *International Conference on Harmonisation Good Clinical Practices (ICH GCP)* as well as applicable local regulatory and ethical requirements.

I acknowledge that I am responsible for overall study conduct. I agree to personally conduct or supervise the described clinical study.

I agree to ensure that all associates, colleagues and employees assisting in the conduct of the study are informed about their obligations. Mechanisms are in place to ensure that site staff receive the appropriate information throughout study.

Investigator Name: ____

____ ______________________________

Investigator Signature **Date** (dd/mm/yyyy)

Protocol Revision History

The protocol version No., effective date and major revisions are summarized as follows:

Protocol version No.	Protocol effective date
1.0	30-APR-2021
2.0	16-JUN-2021
3.0	3-AUG-2021

Protocol Modifications Clarification

This amendment was made based on re-discussion with the investigator.

Detailed revisions are provided in the table below.

Original Section and Title	Description of Protocol Changes	Rationale
Title Page, 1.1 Synopsis, 1.2 Phase 2, 1.3 Phase 3, Number of Subjects, Disclosure Statement, 3.1 Objectives, 3.2 Endpoints, 4.2 Scientific Rationale for Study Design, 5 Study Population, 9.1 Statistical Hypotheses, 9.2 Sample Size Determination, 9.3 Data Monitoring Guidelines for an Independent DSMB, 9.4 Rationale for Early Futility Analysis, 9.5.2. Guidelines for Monitoring of Phase 3 Primary Endpoints, 9.6 Statistical Analysis	The original adaptive Phase 2/3 study was modified into a Phase 2 study with two parts: 2a and 2b.	Changed to a more traditional study design
1.1 Synopsis, 1.2 Phase 2, 1.3 Phase 3, Number of Subjects, 5 Study Population, 9.2 Sample Size Determination, Table 1. Sample Size Calculation for Four Scenarios of Sustained HBsAg Loss, 9.3 Data Monitoring Guidelines for an Independent DSMB, 9.4 Rationale for Early Futility Analysis	The sample size was increased from 106 to 120 in Part I (Phase 2a) and 480 in Part II (Phase 2b) (600 in total).<br>The "9.2 Sample Size Determination" and "Table 1. Sample Size Calculation for Four Scenarios of Sustained HBsAg Loss" provide detailed justification and methodology for sample size calculation and suggest that the sample size in Phase 2b may be re-estimated after unblinding for data analysis after all 120 subjects enrolled in Phase 2a complete PEG-IFN-α treatment or withdraw from the study.	Sample size was updated after the study was changed from an adaptive Phase 2/3 study to a Phase 2 study with 2 parts
1.2 Phase 2, 3.2.2 Secondary Endpoints	Added secondary objective "To evaluate functional cure in subjects with chronic HBV infection treated with BRII-179 (VBI-2601) therapy in combination with PEG-IFN-α + NrtI" and its corresponding secondary endpoint "Percentage of subjects with sustained HBsAg loss	Study objectives were updated after the study was changed from an adaptive Phase 2/3 study to a Phase 2 study with 2 parts

	and persistently undetectable HBV DNA (defined as undetectable HBsAg and HBV DNA for at least 24 weeks) after NrtI discontinuation. " Added the same secondary objectives and endpoints as in Phase 2b part.	
1.2 Phase 2a, 1.5 (SoA), 3.2.3 Tertiary Endpoint, 9.6.5 Tertiary Efficacy Endpoint and Subgroup Analyses	Added assessment of serum protein level cytokine/chemokine biomarkers change from baseline from Day 1 of dosing to the end of follow-up, and during the NrtI discontinuation monitoring period	Further exploration of the immunogenicity profile of BRII-179 was added to support the overall risk/benefit profile for the proposed dose regimen and treatment duration and to characterize the susceptible population to the drug.
1.2 Phase 2 Secondary Endpoints, 1.3 Phase 3 Secondary Endpoints, Overall Design, Intervention Groups and Duration, 1.4. Schematic, 1.5. Schedule of Assessments, 3.2.2 Secondary Endpoints, 4.1 Overall Design, 4.3.1 Justification for Dose Selection and Number of Doses, 8.1.3 Dosing Period	The treatment duration and number of doses for BRII-179 were adjusted from every 4 weeks for a total of 9 doses over 32 weeks to every 3 weeks for a total of 7 doses over 18 weeks.	Base on supportive nonclinical and clinical data and communication with CDE.
1.4 Schema, 1.5 Schedule of Assessments,4.1 study design，5.1 Inclusion Criteria	Revised eligible subjects from receiving PEG-IFN-α treatment for 24-26 weeks to 24-28 weeks, screening window was revised from 14 days to 28 days	Increase clinical feasibility of subject recruitment.
2.3 Benefit/Risk Assessment	Supplemented human safety and immunogenicity data, nonclinical toxicology study data, and relevant clinical data for antigens and adjuvant components in BRII-179	To provide additional evidence for the safety and immunogenicity profile of BRII-179 to support the overall risk/benefit profile of the proposed study design.
4.2 Scientific Rationale for Study Design	Supplemented the rationale for patient population selection and modified some inaccurate statements.	Supplemented the rationale for patient population selection.
4.3 Justification for Dose Selection	Supplemented additional clinical and nonclinical evidence.	Supplemented the justification for dose selection.
5 Study Population	Clarified that the subjects planned to be recruited in this study are Chinese subjects.	Only the Chinese population will be observed in this study

5.2 Exclusion Criterion 14<br><br>6.4.3 Prohibited Medications	Removed Granulocyte macrophage colony stimulating factor (GM CSF) or erythropoietin (EPO) related contents	Better alignment with clinical practice
6 Study Intervention, Table 6.1.2 Dosage and Administration of BRII-179 and Placebo	IMP and NIMP definitions were modified and NrtI was changed to be provided by the Sponsor. Of which,<br>"BRII-179, placebo, PEG-INF-α and NrtI are all provided by the Sponsor" was specified in "6.1.1 Drug Product" and the instructions for NrtI were supplemented.<br>"Table 6.1.2 Dosage and Administration of BRII-179 and Placebo" was changed to "Table 6.1.2 Study Intervention". BRII-179, placebo, and PEG-INF-α were identified as IMPs and NrtI as NIMP. Specific information on PEG-INF-α and NrtI was also supplemented.	Sponsor provides NrtI to provide stable backbone treatment and to clarify IMP and NIMP definitions of the four kinds of drugs
6.4 Concomitant Therapy, 6.4.1. Drug Compliance with NrtI and PEG-IFN-α, 6.4.2 Permitted Medications	Added "6.4 Compliance of Study Intervention" to define compliance of study intervention. Supplemented coping strategy for treatment interruption.<br>Supplemented the collection time of concomitant therapy in "6.5 Concomitant Therapy".	Clarified the scope of data collection for concomitant therapy<br>Compliance with study intervention in addition to NrtI and PEG-IFN-α was added
6.6. Treatment of Study Intervention Overdose, 8.3.5 Special Situations	Removed "and results in clinical symptoms or signs."	Clarified that overdose is identified if exceeding the maximum recommended dose specified in the protocol, regardless of clinical symptoms
7.1 Discontinuation of Study Intervention	For subjects who discontinue study dosing, removed the "Dosing period" in "and continue to perform the required study-related visits and procedures for the remaining duration of study participation in the Dosing Period and Follow-up Period".	Patients who discontinue study dosing can enter the follow-up phase directly
7.1.1 Criteria for Discontinuation of Study Intervention	Clarified that subjects who discontinue study intervention will continue to undergo follow-up monitoring in the study.<br>Supplementary Criteria:	Added other conditions that may result in discontinuation of study intervention

	Discontinuation of study intervention requested by the subject or the subject's legal representative.<br>The subject has a medical condition or personal condition that, in the opinion of the investigator and/or the Sponsor, would pose an unnecessary risk to the subject if the investigational intervention is continued.<br>The subject's serum pregnancy test is confirmed to be positive.	
8.1.7 Early Termination Visit,1.5 Schedule of Assessments	Removed "A follow Up Visit will be conducted approximately 4 weeks after the last dose of study drug." This will be required only if the subject has ongoing AEs or lab abnormalities at the last on-study visit. "This visit can be conducted by telephone ", and added specification of early termination visits ET2 and ET3	Clarified the definition of different early termination visits at different stages
9.7.2 Analysis of Phase 2 Primary Efficacy Endpoints	Added coping measures for fail of model fit due to too few subjects in a given stratum.	This description was supplemented considering the small number of subjects enrolled in Phase 2a which may lead to the small number of cases in one stratum
9.6.5. Analysis of Safety Outcomes	The definition of TEAE was updated. Safety analyses were clarified as descriptive statistics and summary descriptions of AEs, laboratory tests, vital signs, physical examinations, and 12-lead electrocardiograms were specified.	TEAEs were identified as AEs occur during or after treatment and AEs that are present pre-dose and worsened post-dose
1.5. Schedule of Assessments	Increased the number of visits, shorten treatment duration, and adjusted the examinations for the respective visits	The total duration of BRII-179 or placebo administration was reduced from 32 to 18 weeks due to the change of dosing from every four weeks to every three weeks, and the respective visit requirements need to be adjusted
Other	Updates to other descriptive text	To make the wording more accurate

Table of Contents

TITLE....1

Protocol Revision History....4

TABLE OF CONTENTS....9

1. PROTOCOL SUMMARY....14

1.1. Synopsis....14

1.2. Phase 2a (N=120)....14

1.3. Phase 2b (N=480)....15

1.4. Schema....20

1.5. Schedule of Activities (SoA)....21

1.5.1. BRII-179 (VBI-2601) 18-week and PEG-IFN-α up to 24-week Dosing Period and 24-week Follow-up Period....22

1.5.2. NrtI Discontinuation Monitoring Period....24

2. INTRODUCTION....25

2.1. Background....25

2.2. Study Rationale....26

2.3. Benefit/Risk Assessment....27

3. OBJECTIVES AND ENDPOINTS....28

3.1. Objectives....28

3.1.1. The Primary Objectives....28

3.1.2. The Secondary objectives....28

3.1.3. The Tertiary Objectives....28

3.2. Endpoints....28

3.2.1. Primary Endpoints....28

3.2.2. Secondary Endpoints....28

3.2.3. Tertiary Endpoints....29

4. STUDY DESIGN....30

4.1. Overall Design....30

4.1.1. Independent DSMB....30

4.1.2. NrtI Discontinuation....30

4.1.3. PEG-IFN-α Therapy During the Dosing Period....31

4.2. Scientific Rationale for Study Design....33

4.3. Dosage Instructions....34

4.3.1. Justification for Dose Selection and Number of Doses 34
4.3.2. Maximum dose/exposure in this study 35
4.4. End of Study Definition 35
5. STUDY POPULATION 36
5.1. Inclusion Criteria 36
5.2. Exclusion Criteria 37
5.3. Lifestyle Considerations 39
5.4. Screening Failures 39
6. STUDY INTERVENTION 40
6.1. Study Intervention(s) Administered 40
6.1.1. Formulations 40
6.1.2. Study Intervention 41
6.2. Preparation/Handling/Storage/Accountability 42
6.3. Randomization and Blinding 42
6.4. Compliance of Study Intervention 43
6.5. Concomitant Therapy 43
6.5.1. Permitted Medications 44
6.5.2. Prohibited Medications 44
6.6. Safety Follow-up and Management of Reactions to Study Drug 45
6.7. Treatment of Study Intervention Overdose 45
6.8. Intervention after the End of the Study 46
7. DISCONTINUATION OF STUDY INTERVENTION AND SUBJECT DISCONTINUATION/WITHDRAWAL 47
7.1. Discontinuation of Study Intervention 47
7.1.1. Criteria for Discontinuation of Study Intervention 47
7.2. Subject Discontinuation/Withdrawal from the Study 48
7.2.1. Lost to Follow up 48
8. STUDY ASSESSMENTS AND PROCEDURES 49
8.1. Schedule of Assessments 49
8.1.1. Screening 49
8.1.2. Randomization (Day 1) 49
8.1.3. Dosing Period 49
8.1.4. Follow-up Period 50
8.1.5. NrtI Discontinuation Monitoring Period 50
8.1.6. Unscheduled Visits 50

8.1.7. Early Termination Visit 50
8.2. Detailed Description of Study Procedures 51
8.2.1. Medical History 51
8.2.2. Physical Examinations 51
8.2.3. Fibroscan 51
8.2.4. Alcohol Intake Assessment 51
8.2.5. Vital Signs 51
8.2.6. Electrocardiograms 52
8.2.7. Laboratory Assessments 52
8.2.8. Laboratory Events of Clinical Interest 53
8.3. Adverse Events and Serious Adverse Events 53
8.3.1. Time Period and Frequency for Collecting AE and SAE Information 53
8.3.2. Method of Detecting AEs and SAEs 54
8.3.3. Follow-up of AEs and SAEs 54
8.3.4. Regulatory Reporting Requirements for SAEs 54
8.3.5. Special Situations 54
8.4. Pharmacodynamics 56
8.5. Genetics 56
8.6. [Health Economics] OR [Medical Resource Utilization and Health Economics] 56
9. STATISTICAL CONSIDERATIONS 57
9.1. Statistical Hypotheses 57
9.1.1. Phase 2a Part 57
9.1.2. Phase 2b Part 57
9.2. Sample Size Determination 57
9.2.1. Phase 2a Part 57
9.2.2. Phase 2b Part 57
9.3. Data Monitoring Guidelines for Independent DSMB 58
9.4. Primary Endpoint and Safety Data Analysis in Phase 2 Part 59
9.4.1. Monitoring Guidelines for the Primary Endpoint in Phase 2b Part 59
9.5. Analysis Sets 59
9.6. Statistical Analyses 60
9.6.1. General considerations 60
9.6.2. Analysis of the Primary Efficacy Endpoint for Phase 2a Part 61
9.6.3. Analysis of the Primary Efficacy Endpoint for Phase 2b Part 61

9.6.4. Analyses of Secondary Efficacy Endpoints and Subgroups....61
9.6.5. Tertiary Efficacy Endpoint and Subgroup Analyses of Phase 2a Part....61
9.6.6. Analysis of Safety Outcome....62
10. SUPPORTING DOCUMENTATION AND OPERATIONAL CONSIDERATIONS....63
10.1. Appendix 1: Regulatory, Ethical, and Study Oversight Considerations....63
10.1.1. Regulatory and Ethical Considerations....63
10.1.2. Financial Disclosure....63
10.1.3. Informed Consent Process....63
10.1.4. Data Protection....64
10.1.5. Data Quality Assurance....64
10.1.6. Source Documents....65
10.1.7. Study and Site Start and Closure....65
10.1.8. Publication Policy....66
10.2. Appendix 2: Clinical Laboratory Tests....67
10.3. Appendix 3: Adverse Events: Definitions and Procedures for Recording, Evaluating, Follow-up, and Reporting....68
10.3.1. Definition of AE....68
10.3.2. Definition of SAE....69
10.3.3. Recording and Follow-Up of AE and/or SAE....70
10.3.4. Reporting of SAEs....71
10.3.5. Reporting of Laboratory Events of Clinical Interest....71
10.3.6. Special Situation Report....71
10.4. Appendix 4: Contraceptive Requirements and Collection and Reporting of Pregnancy Information....72
10.5. Appendix 5: Assessments in Subjects Meeting Laboratory ECI Criteria....74
11. REFERENCES....76

ABBREVIATIONS

AE	adverse event
ALT	alanine aminotransferase
HBsAb	hepatitis B surface antibody
DNA	deoxyribonucleic acid
DSMB	Data and Safety Monitoring Board
ECG	electrocardiogram
ECI	events of clinical interest
EPO	erythropoietin
ET	early termination
ETV	entecavir
GM-CSF	granulocyte-macrophage colony-stimulating factor
HBeAg	hepatitis B e-antigen
HBsAg	hepatitis B surface antigen
HBV	hepatitis B virus
HCV	hepatitis C virus
HDV	hepatitis D virus
HIV	human immunodeficiency virus
ICF	informed consent form
IFN-α	interferon-alpha
IMP	Investigational medicinal product
IU	international units
kPa	kilopascal
LLOQ	lower limit of quantification
NCI-CTCAE	National Cancer Institute Common Terminology Criteria for Adverse Events
NIMP	Non-investigational medicinal product
NrtI	nucleos(t)ide reverse transcriptase inhibitors
ULN	upper limit of normal
PEG-IFN-α	pegylated interferon-alpha
PD	Pharmacodynamic
PK	pharmacokinetic
SoA	schedule of activities
SAE	serious adverse event
TDF	tenofovir disoproxil fumarate
TAF	tenofovir alafenamide
WOCBP	women of child-bearing potential

1. PROTOCOL SUMMARY

1.1. Synopsis

Protocol Title: A Multicenter, Randomized, Double-Blind, Placebo-Controlled, Parallel-Group Study to Investigate the Efficacy and Safety of BRII-179 (VBI-2601) for the Treatment of Chronic Hepatitis B Virus (HBV) Infection

Rationale:
Multiple therapies with differential mechanisms of actions to treat hepatitis B virus (HBV) is an encouraging strategy to achieve higher rates of hepatitis B surface antigen (HBsAg) loss and ideally HBsAg seroconversion. The present study (BRII-179-002) will evaluate the efficacy and safety of adding a recombinant protein immunotherapeutic (BRII-179 [VBI-2601]) treatment regimen to existing pegylated interferon-alpha (PEG-IFN-α) and nucleos(t)ide reverse transcriptase inhibitor (NrtI) therapy in chronic HBV patients from the Everest Project (NCT04035837). The primary objective of the Everest Project is to achieve functional cure of HBV infection by optimizing treatment strategies of NrtIs and PEG-IFN-α.

Individualized regimens of IFN and NrtI therapies have shown promising efficacy in reducing HBsAg with some patients achieving HBsAg clearance (Ren, 2016; Marcellin, 2016). This result is more pronounced in patients on optimized HBV treatment to low HBsAg levels, which is the rationale of including subjects from the Everest Project in this study. Preclinical and clinical studies have also shown the benefit of low HBsAg levels for breaking tolerance with a therapeutic HBV vaccine (Zhu, 2016; Michler, 2020; Roggendorf, 2019). The recombinant protein immunotherapeutic, BRII-179 (VBI-2601), may break immunological tolerance and restore and/or augment adaptive T cell and antibody responses to the surface antigens. These responses together have the potential to better achieve a functional cure (sustained loss of HBsAg in blood with or without seroconversion to the surface antigen antibody) of chronic HBV infection.

Objectives and Endpoints

The study will consist of two parts, Part 2a and 2b, of which 120 subjects are planned to be enrolled in the first part (Phase 2a) and 480 subjects are planned to be enrolled in the second part (Phase 2b).

1.2. Phase 2a (N=120)

Objectives	Endpoints
Primary	
To determine the efficacy of BRII-179 (VBI-2601) to increase HBsAg loss through completion of PEG-IFN-α treatment	• Percentage of subjects with HBsAg loss at completion of PEG-IFN-α treatment
Secondary	

▪ To evaluate functional cure of subjects with chronic HBV infection receiving BRII-179 (VBI-2601) therapy in combination with PEG-IFN-α + NrtI<br>▪ To assess the antiviral activity of BRII-179 (VBI-2601) therapy in combination with PEG-IFN-α + NrtI	• Percentage of subjects with sustained HBsAg loss and persistently undetectable HBV DNA (defined as undetectable HBsAg and HBV DNA for at least 24 weeks) after NrtI discontinuation.<br>• HBsAg change from baseline from Day 1 to completion of PEG-IFN-α treatment<br>• Percentage of subjects with HBsAg loss at any timepoint<br>• Percentage of subjects with undetectable HBV-DNA (HBV DNA < Lower Limit of Quantification (LLOQ)) 24 and 48 weeks after NrtI discontinuation<br>• Percentage of subjects meeting the NrtI discontinuation criteria by end of follow-up period (Week 48)<br>• Percentage of subjects with HBsAg seroconversion at any timepoint<br>• Percentage of subjects with hepatitis B surface antibody (HBsAb) response as of the end of the follow-up period (Week 48)<br>• Time to achieve HBsAg seroclearance and seroconversion<br>• Percentage of subjects requiring NrtI re-treatment during follow up
To assess safety of BRII-179 (VBI-2601) therapy	• Percentage of subjects with adverse events (AEs) and serious adverse events (SAEs)<br>• Percentage of subjects with abnormalities in clinical laboratory tests
Tertiary	
To assess the effect on relevant immune markers in subjects with chronic HBV infection treated with BRII-179 (VBI-2601) in combination with PEG-IFN-α + NrtI	• Serum protein level cytokine/chemokine biomarkers change from baseline during the 48-Weekmonitoring period after NrtI discontinuation

1.3. Phase 2b (N=480)

Objectives	Endpoints
Primary	
To evaluate functional cure of subjects with chronic HBV infection receiving BRII-179 (VBI-2601) therapy in combination with PEG-IFN-α + NrtI	• Percentage of subjects with sustained HBsAg and HBV DNA loss (defined as undetectable HBsAg and HBV DNA for at least 24 weeks) after NrtI discontinuation
Secondary	
To assess the antiviral activity of BRII-179 (VBI-2601) therapy in combination with PEG-IFN-α + NrtI	• Percentage of subjects with undetectable HBV-DNA (HBV DNA < Lower Limit of Quantification (LLOQ)) 24 and 48 weeks after NrtI discontinuation

	• Percentage of subjects meeting the NrtI discontinuation criteria by end of follow-up period (Week 48)<br>• Percentage of subjects with HBsAg loss at any timepoint<br>• Percentage of subjects with HBsAg seroconversion at any timepoint<br>• Percentage of subjects with hepatitis B surface antibody (HBsAb) response by end of follow-up period (Week 48)<br>• HBsAg change from baseline from Day 1 to End of Treatment (up to Week 24)<br>• Time to achieve HBsAg seroclearance and seroconversion<br>• Percentage of subjects requiring NrtI re-treatment during follow up
To assess safety of BRII-179 (VBI-2601) therapy	• Percentage of subjects with adverse events (AEs) and serious adverse events (SAEs)<br>• Percentage of subjects with abnormalities in clinical laboratory tests

Overall Design

Study BRII-179-002 is a double-blind, randomized, placebo-controlled, parallel-group study aims to evaluate the clinical effect of adding BRII-179 (VBI-2601) to existing PEG-IFN-α and NrtI therapy in patients with chronic HBV infection without cirrhosis enrolled in the Everest Project study being conducted in China (NCT04035837).

The Everest Project is a multicenter real-world study being conducted in over 260 hospitals in China. The plan is to recruit 30,000 patients with chronic HBV infection effectively treated with NrtI therapy for at least one year; who are hepatitis B e-antigen (HBeAg) negative with HBV DNA <100 IU/ml and HBsAg <1500 IU/mL at study entry. As part of the study participation, subjects receive PEG-IFN-α as "add-on" or "switch to" treatment for 48 to 96 weeks. The primary objective of the Everest Project is to achieve functional cure of HBV infection by optimizing treatment strategies of NrtIs and PEG-IFN-α.

Eligible subjects from the Everest Project study, who have been receiving PEG-IFN-α for 24 to 28 weeks and NrtI therapy for at least 18 months, will be randomized 1:1 to receive BRII-179 (VBI-2601) or placebo in addition to the existing PEG-IFN-α and NrtI therapy. Subjects will be stratified at randomization by screening HBsAg level (3 categories: ≥0.05 to < 1 IU/mL, ≥1 to < 10 IU/mL, and ≥10 to <100 IU/mL. Subjects will complete investigational treatment with BRII-179 or placebo at Week 18; PEG-IFN-α treatment will last for up to 24 weeks after enrollment (a total of 48 weeks of PEG-IFN-α treatment before and after enrollment); while NrtI treatment will continue into the Follow-up Period. The BRII-179-002 study design schema can be found in Section 1.4.

Estimated total duration for each subject will include the following: screening up to 28 days, dosing period up to 24 weeks (18 weeks BRII-179 or placebo investigational treatment; up to 24 weeks PEG-IFN-α treatment), and up to a 28-week follow-up period. Eligible subjects that meet NrtI discontinuation criteria (Section 4.1.2.1) (eligibility assessed at Week 36 to Week 48) may enter a NrtI discontinuation monitoring period for an additional 48 weeks from the day of NrtI discontinuation (Section 1.5.2).

Number of Subjects:

The study will consist of two parts, Part 2a and 2b, of which 120 subjects are planned to be enrolled in the first part (Phase 2a) and 480 subjects are planned to be enrolled in the second part (Phase 2b).

Intervention Groups and Duration:

Eligible subjects currently receiving PEG-IFN-α and NrtI therapy will receive the study drug BRII-179 (VBI-2601) or matching placebo, every three weeks for a total of 7 doses over an 18-week course.

Both groups will be treated with both PEG-IFN-α and NrtI, with PEG-IFN-α lasted for up to 24 weeks after enrollment (a total of 48 weeks of PEG-IFN-α treatment before and after enrollment), and NrtI through the follow-up period.

Independent Data and Safety Monitoring Board (DSMB)

An independent DSMB will monitor patient safety and treatment efficacy data for this study. The details for the operation and responsibilities of the DSMB will be defined in a separate DSMB

Charter. The Charter will delineate the composition, duties, responsibilities, and procedures of the DSMB, data required at each meeting, as well as the analyses that will be conducted.

Disclosure Statement: This is a phase 2, randomized, multicenter, double-blind, placebo-controlled, parallel-group study to evaluate the efficacy and safety of BRII-179 (VBI-2601) in patients with chronic HBV infection receiving PEG-IFN-α and NrtI therapy. All references to "Sponsor" contained herein shall refer to Brii Biosciences.

1.4. Schema

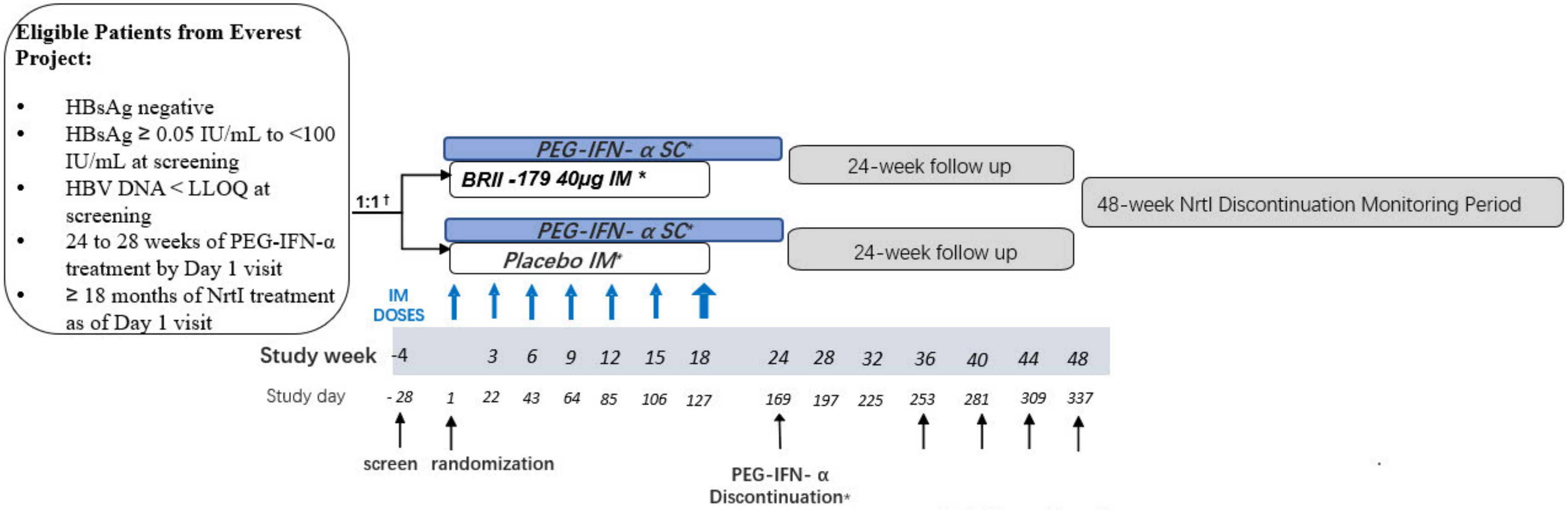

NrtI discontinuation

- Subjects that meet the NrtI discontinuation criteria may switch from the follow-up period to the 48-week NrtI discontinuation monitoring period
- Day 1 of the NrtI Discontinuation Monitoring Period is the first day a subject discontinues NrtI
- Subjects that have not met NrtI discontinuation criteria by Week 48 will be withdrawn from the study

IM: intramuscular ; SC : Subcutaneous Injection

* Each subject will accept a total of 48 weeks PEG-IFN-α treatment; Co-medication of NrtI will continue during the dosing period; NrtI treatment will last until week 48 if the subject could not meet the NrtI discontinuation criteria

† Subjects stratified by HBsAg level

1.5. Schedule of Activities (SoA)

**Study Stage**	**Screening**	**Dosing Period[b]**								**Follow-up Period**					
**Study Visit Week**	Screening	Rand	W3	W6	W9	W12	W15	W18	W20-24 [c] /ET1	W28	W32	W36[d]	W40[d]	W44[d]	W48[d]/ ET2
**Study Visit Day ± Visit Window**	D-28 to-1	D1[a]	D22 ± 3	D43 ± 3	D64 ± 3	D85 ± 3	D106± 3	D127 ± 3	D141-169 ± 3	D197 ± 3	D225 ± 3	D253 ± 5	D281 ± 5	D309 ± 5	D337 ± 5
Informed consent form (ICF)	X														
Inclusion/exclusion criteria	X	X[e]													
Demography	X														
Medical history including current medical conditions and HBV genotype[f]	X														
Fibroscan	X														
Full physical exam[g]	X							X	X						X
Symptom-directed physical exam		X	X	X	X	X	X			X	X	X	X	X	
Alcohol intake assessment[h]		X	X	X	X	X	X	X	X	X	X	X	X	X	X
Vital signs[i]	X	X				X			X						
12-lead ECG[j]	X	X							X						
Subject instructed to complete diary		X	X	X	X	X	X	X	X	X	X	X	X	X	X
Urinalysis	X														
Pregnancy test[k]	X	X	X	X	X	X	X	X	X	X	X	X	X	X	X
Safety Laboratory Assessments[n]	X	X	X	X	X	X	X	X	X	X	X	X	X	X	X
Randomization		X													

Study drug administration		X	X	X	X	X	X	X[l]	X[m]						
Screening viral serology	X														
Blood sample for HbsAg/HBsAb quantitative	X	X		X		X	X	X	X	X	X	X	X	X	X
Blood sample for cytokine/chemokine biomarker quantitation	X	X		X		X		X	X		X		X		X
Blood sample for HBV DNA quantitation[o]	X	X				X			X	X	X	X	X	X	X
Adverse Events assessment[p]	←----------X----------→														
ECI surveillance[q]	←----------X----------→														
Concomitant medications review	←----------X----------→														
NrtI medication adherence review[r]	←----------X----------→														
PEG-IFN-α medication adherence review[r]	←----------X----------→														

1.5.1. BRII-179 (VBI-2601) 18-week and PEG-IFN-α up to 24-week Dosing Period and 24-week Follow-up Period

AE = adverse event; ECI = events of clinical interest; HBsAg = hepatitis B surface antigen; HBsAb = hepatitis B surface antibody; ECG = electrocardiogram; ET1 = early termination 1; ET2 = early termination 2; HBV = Hepatitis B Virus; NrtI = nucleoside/nucleotide reverse transcriptase inhibitor; ULN = Upper Limit of Normal

Note: When scheduled at the same timepoints, the assessments of vital signs and 12-lead ECGs must be performed before blood sample collections.

[a] Assessments performed pre-dose unless otherwise specified.

[b] If a subject withdraws prematurely from the study prior to their Week 20-24 visit, ET1 assessments should be performed within 14 days.

[c] This visit occurs when complete 48 weeks PEG-IFN-α treatment,20-24 weeks after the enrollment.

[d] Subjects that meet NrtI discontinuation criteria (Section 4.1.2.1) may discontinue from the Follow-up Period and enter the NrtI Discontinuation Monitoring Period (Section 1.5.2). Day 1 of the NrtI Discontinuation Monitoring Period is the first day subject stops their NrtI therapy.

[e] Evaluation of inclusion and exclusion criteria related to pregnancy test, medical history and concomitant medications prior to dosing.

[f] A complete medical history will be taken at screening including HBV genotype if available. Any changes should be updated prior to dosing.

[g] Height and weight will be recorded at screening. Body mass index will be calculated based on the measurements. Only weight will be collected afterwards.

[h] Subjects' alcohol intake will be recorded while on study.

[i] Vital signs (blood pressure, pulse rate, respiratory rate, and temperature) should be measured after the subject has rested comfortably for approximately 5 minutes.

[j] 12-lead safety ECGs should be measured in the supine position after the subject has rested comfortably for 10 minutes.

[k] Women of childbearing potential are required to have pregnancy tests. Negative pregnancy test must be confirmed prior to study drug administration. Urine pregnancy test results from local laboratory are acceptable (except for the screening visit).

[l] Subjects will complete investigational treatment with BRII-179 or placebo;

[m] PEG-IFN-α treatment will be continued for up to 24 weeks (PEG-IFN-α for 48 weeks before and after enrollment); NrtI therapy will continue into the Follow-up Period.

[n] Refer to Table 2 Safety Assessments in Appendix 2.

[o] If a subject experiences alanine aminotransferase (ALT) flare, additional HBV DNA quantitation sample(s) may be collected.

[p] All AEs related to screening activities must be collected from the time of consent until end of study; any other events reported during the screening period should be reported as medical history. All SAEs must be collected from the time of consent until end of study.

[q] Subject will be monitored for clinical laboratory events of clinical interest (ECI) and additional assessments may be performed if indicated. ECI for this study include ALT > 3x ULN, if Day 1 pre-dose ALT ≤ ULN; ALT > 2x baseline, if Day 1 pre-dose ALT > ULN.

[r] Subjects will be instructed to bring NrtI and PEG-IFN-α (including all empty containers) to the trial site at every visit, as applicable, beginning with the screening visit. Adherence will be assessed by counting of empty containers and unused medication. See Section 6.4.

1.5.2. NrtI Discontinuation Monitoring Period

**Study Stage**	**NrtI Discontinuation Monitoring Period[b,c]**						
**Study Visit Week**		W4	W8	W12	W24	W36	W48/ET3
**Study Visit Day ± Visit Window**	D1[a]	D28 ± 3	D56 ± 3	D84 ± 3	D168 ± 7	D252 ± 7	D336 ± 7
Safety Laboratory Assessments[d]	X	X	X	X	X	X	X
Symptom-directed physical exam	X	X	X	X	X	X	X
Fibroscan							X
Pregnancy test		X					X
Blood sample for HBsAg/HBsAb quantitation	X			X	X	X	X
Blood sample for cytokine/chemokine biomarker quantitation	X		X		X		X
Blood sample for HBV DNA quantitation	X	X	X	X	X	X	X
Concomitant medications	←------------------------ X ------------------------→						
Adverse event assessment	←------------------------ X ------------------------→						
NrtI retreatment surveillance[e]	←------------------------ X ------------------------→						
ECI surveillance[f]	←------------------------ X ------------------------→						

AE = adverse event; ECI = events of clinical interest; HBsAg = hepatitis B surface antigen; HBsAb = hepatitis B surface antibody; HBV = Hepatitis B Virus; NrtI = nucleoside/nucleotide; ULN = Upper Limit of Normal; ET3 = early termination 3

[a] All timepoints are relative to the day of NrtI discontinuation (D1) which is the first day subjects stop their NrtI therapy.

[b] All subjects who discontinue NrtI therapy will be followed through 48 weeks, even if NrtI retreatment occurs. If a subject withdraws prematurely from the study prior to the W48 visit, ET assessments should be performed.

[c] NrtI retreatment guidelines are provided in Section 4.1.2.2.

[d] Safety laboratory assessments are limited to liver function tests.

[e] Investigator should monitor subjects for NrtI retreatment criteria. See Section 4.1.2.2.

[f] Subject will be monitored for clinical laboratory events of clinical interest (ECI) and additional assessments may be performed if indicated. ECI for this study include ALT > 3x ULN, if Day 1 pre-dose ALT ≤ ULN; ALT > 2x baseline, if Day 1 pre-dose ALT > ULN.

2. INTRODUCTION

2.1. Background

Chronic HBV infection remains an important global public health problem that leads to serious sequelae over time, including cirrhosis, liver failure, and hepatocellular carcinoma (Trepo, 2014). An estimated 290 million people are living with chronic HBV infection worldwide, and almost 800,000 people die annually due to associated sequelae (Stanaway, 2016).

Human HBV is a deoxyribonucleic acid (DNA) virus in the Hepadnaviridae family, with 10 HBV genotypes (A to J). The distribution of these genotypes varies geographically, with A and D being more common in Northern Europe, North America, and Western and Central Asia; and B and C being more common in Southeast Asia and China. HBV genotypes may influence disease progression rates and treatment response (Kramvis, 2014).

Chronic HBV infection is characterized by detection of HBV surface antigen (HBsAg) in the blood for > 6 months. The prevalence of chronic HBV infection varies geographically, with a range of < 2% in low to > 8% in high prevalence countries (Schweitzer, 2015). In high prevalence countries, such as those in sub-Saharan Africa and East Asia, transmission occurs predominantly in infants and children by perinatal and horizontal routes. In more industrialized countries, new infections are highest among young adults and transmission occurs predominantly via injection drug use and high-risk sexual behaviors. The risk of developing chronic HBV infection depends on the age at the time of infection. While only ~10% of people infected as adults develop chronic HBV infection, 90% of infants infected perinatally or during the first 6 months of life, and 20% to 60% of children infected between 6 months and 5 years of age remain chronically infected. Twenty-five percent of people who acquire HBV during infancy and childhood will develop primary liver cancer or cirrhosis during adulthood.

Current treatment options for chronic HBV infection are limited. One option is long-term Nucleoside reverse transcriptase inhibitors (NrtI) therapy, for example tenofovir (tenofovir disoproxil fumarate or tenofovir alafenamide) or entecavir (Lok, 2016). However, NrtI therapy does not result in a cure of HBV infection. The viral DNA is not cleared from the infected cell, and viral protein production, in particular the production of HBsAg, typically continues at high levels as measured in blood (Scaglione, 2012). These high levels of HBsAg subviral particles are proposed to mediate HBV-specific immune dysfunction by decoying and exhausting host cellular and humoral adaptive immune responses. Therefore, reduction or clearance of HBsAg from blood is considered central to achieving a functional cure (sustained loss of HBsAg in blood with or without seroconversion to HBsAb) and is considered an optimal outcome of treatment (Block, 2013). An alternative treatment option is a finite course of Pegylated Interferon α (PEG-IFN-α) therapy which can induce a functional cure, but only in a small percentage of patients (< 10%) (Konerman, 2016). Unfortunately, PEG-IFN-α therapy carries considerable risk of dose-limiting adverse events (AEs) which may be severe and/or serious, and discontinuation rates are significant (Perrillo, 2009).

The failure of NrtI therapy to produce a functional cure and the limitations of PEG-IFN-α therapy highlight the clinical need for effective, well-tolerated HBV therapies of finite duration.

2.2. Study Rationale

BRII-179 (VBI-2601) is a recombinant virus-like particle-based (VLP) based protein immunotherapeutic derived from the 3rd generation HBV prophylaxis vaccine, Sci-B-Vac® consists of all three HBV surface (S) antigen forms: small, middle and large proteins. The large S protein contains the N-terminal preS1 and preS2 domains and the adjacent small S protein. The middle S protein contains preS2 and the adjacent small S protein.

It has been hypothesized that high levels of circulating HBsAg lead to immune tolerance of HBV and contribute to chronic HBV infection. BRII-179 (VBI-2601) utilizes Pre-S1, Pre-S2, and S protein components as the immunogen in a therapeutic application of preS1/S2 containing HBsAg. Additionally, to have an effective T cell response, BRII-179 (VBI-2601) differs from Sci-B-Vac® and most of the recombinant envelope vaccines by using aluminium phosphate as an adjuvant rather than aluminum hydroxide. As a new formulation of higher doses of Sci-B-Vac® with aluminum-phosphate adjuvant, BRII-179 (VBI-2601) shows enhanced Th1 responses against the HBV surface antigens. BRII-179 has been shown to be safe and well-tolerated in chronic HBV (CHB) patients under Nrtl therapy in a phase 1b/2a study (BRII-179-001). Administration of BRII-179 carrying S, Pre-S1 and Pre-S2 regions of HBV envelope is immunogenic at both B and T cell levels in CHB patients.

Multiple therapies with differential mechanisms of actions to treat HBV is an encouraging strategy to achieve higher rates of HBsAg loss and ideally HBsAg seroconversion. The present study (BRII-179-002) will evaluate the efficacy and safety of adding a recombinant protein immunotherapeutic (BRII-179 [VBI-2601]) treatment regimen to existing PEG-IFN-α and NrtI therapy in patients with chronic HBV infection who have participated in the Everest Project (NCT04035837). The primary objective of the Everest Project is to achieve functional cure of HBV infection by optimizing combination and sequential treatment strategies of NrtIs and PEG-IFN-α in patients with chronic HBV infection (negative HBeAg) and low levels of HBsAg (≤ 1500 IU/mL) and HBV DNA (< 100 IU/mL).

Individualized regimens of combination therapy of IFN-α, an innate immunomodulator and antiviral agent and NrtI, a direct acting antiviral agent have shown promising efficacy in reducing HBsAg with some patients achieving HBsAg clearance (Ren, 2016; Marcellin, 2016). This result is more pronounced in patients on optimized HBV treatment to low baseline HBsAg levels, which is the rationale of including subjects from the Everest Project in this study. Preclinical and clinical studies have also shown the benefit of low HBsAg levels for breaking tolerance with a therapeutic HBV vaccine (Zhu, 2016; Michler, 2020; Roggendorf, 2019). BRII-179 (VBI-2601), as an immunomodulator, can break immunological tolerance and restore and/or augment cellular T cell and antibody responses to the HBV surface antigens which in turn result in HBsAg clearance. Therefore, the initial treatment of PEG-IFN-α and NrtI followed by BRII-179 add-on treatment could increase the potential to achieve higher functional cure rate in chronic HBV infected patients.

2.3. Benefit/Risk Assessment

This study will provide information on the efficacy and safety of BRII-179 (VBI-2601) added to PEG-IFN-α and NrtI optimized therapy to achieve functional cure in patients with chronic HBV infection.

There are extensive human safety and immunogenicity data for antigens and adjuvant components used in BRII-179. Sci-B-Vac® with identical antigenic components has been safely used in more than 500,000 people (healthy subjects and CHB patients) worldwide (including the United States, Canada, Europe, Hong Kong of China), including more than 600 subjects who received Sci-B-Vac® with the same aluminum phosphate adjuvant as that in BRII-179 and had a good tolerability profile. In addition, the immunogenicity and safety of Sci-B-Vac® vaccines with aluminum phosphate adjuvant for early clinical studies are comparable to those with aluminum hydroxide adjuvant at a later stage. In the completed acute and chronic toxicology studies, the main toxicity profile of BRII-179 was generally consistent in rabbits and rats (Sci-B-Vac®) at dose levels exceeding 40 times the planned clinical dose of BRII-179, including mild and reversible side effects at intramuscular injection (IM) sites which were generally recoverable. In nonclinical toxicology studies, acute and chronic multiple-dose toxicology studies in rats and New Zealand White rabbits were conducted to support the currently planned number of clinical doses. BRII-179 (VBI-2601) with or without IFN-α as an adjuvant, administered every 4 weeks for a total of 4 doses, have been evaluated in adult subjects with chronic HBV infection. Favorable safety and tolerability profiles were observed at 40 µg dose level. In addition, the 8-month therapeutic vaccine trial (Sci-B-Vac®), which has been administered once a month in the Vietnamese population, has shown good efficacy and good safety data.

Based on the above mentioned available clinical and non-clinical data, the proposed add-on of BRII-179 (VBI-2601) to PEG-IFN-α and NrtI therapy may further reduce HBsAg effectively and induce HBV-specific immunity which may lead to immune control and functional cure of chronic HBV infection. The safety profile of BRII-179 (VBI-2601) is supportive of the overall risk/benefit profile for the proposed dose regimen and treatment duration.

More detailed information about the known and expected benefits and risks and reasonably expected adverse events of BRII-179 (VBI-2601) may be found in the Investigator's Brochure.

3. OBJECTIVES AND ENDPOINTS

3.1. Objectives

3.1.1. The Primary Objectives

- Phase 2a: To evaluate the efficacy of BRII-179 to increase HBsAg loss through completion of PEG-IFN-α treatment
- Phase 2b: To evaluate functional cure of chronic HBV subjects receiving BRII-179 (VBI-2601) therapy in combination with PEG-IFN-α + NrtI.

3.1.2. The Secondary objectives

- To assess the antiviral activity of BRII-179 (VBI-2601) therapy added to PEG-IFN-α + NrtI.

- To assess safety of BRII-179 (VBI-2601) therapy

3.1.3. The Tertiary Objectives

- To assess the effect on relevant immune markers in subjects with chronic HBV infection treated with BRII-179 (VBI-2601) in combination with PEG-IFN-α + NrtI

3.2. Endpoints

3.2.1. Primary Endpoints

- Phase 2a: Percentage of subjects with HBsAg loss at completion of PEG-IFN-α treatment

- Phase 2b: Percentage of subjects with sustained HBsAg and HBV DNA loss (defined as undetectable HBsAg and HBV DNA for at least 24 weeks) after NrtI discontinuation

3.2.2. Secondary Endpoints

Below secondary endpoints apply to both Phase 2a and Phase 2b unless otherwise specified.

- HBsAg change from baseline of from Day 1 to completion of PEG-IFN-α treatment

- Percentage of subjects with HBsAg loss at any timepoint

- Phase 2a only: Percentage of subjects with sustained HBsAg loss and persistently undetectable HBV DNA (defined as undetectable HBsAg and HBV DNA for at least 24 weeks) after NrtI discontinuation

- Percentage of subjects with undetectable HBV-DNA (HBV DNA < LLOQ) 24 and 48 weeks after NrtI discontinuation

- Percentage of subjects meeting the NrtI discontinuation criteria by end of follow-up period (Week 48)
- Percentage of subjects with HBsAg seroconversion at any timepoint
- Percentage of subjects with HBsAb response by end of follow-up period (Week 48)
- HBsAg change from baseline from Day 1 to End of Treatment (up to Week 24)
- Time to achieve HBsAg seroclearance and seroconversion
- Percentage of subjects requiring NrtI re-treatment during follow up
- Percentage of subjects with adverse events (AEs) and serious adverse events (SAEs)
- Percentage of subjects with abnormalities in clinical laboratory tests

3.2.3. Tertiary Endpoints

Phase 2a only: Assessment of serum protein level cytokine/chemokine biomarkers changes from baseline from Day 1 of dosing to the end of follow-up, and during the NrtI discontinuation monitoring period

4. STUDY DESIGN

4.1. Overall Design

Study BRII-179-002 is a double-blind, randomized, placebo-controlled, parallel-group study of adding BRII-179 (VBI-2601) to existing PEG IFN-α and NrtI therapy in patients with chronic HBV infection without cirrhosis enrolled in the Everest Project study being conducted in China (NCT04035837).

The Everest Project is a multicenter real-world study being conducted in over 260 hospitals in China. The plan is to recruit 30,000 patients with chronic HBV effectively treated with NrtI therapy for at least one year; who are HBeAg negative with HBV DNA <100 IU/ml and HBsAg <1500 IU/mL at study entry. As part of the study participation, subjects receive PEG-IFN-α as "add-on" or "switch to" treatment for 48 to 96 weeks. The primary objective of the Everest Project is to achieve functional cure of HBV infection by optimizing treatment strategies of NrtIs and PEG-IFN-α.

Eligible subjects from the Everest Project study, who have received PEG-IFN-α for 24 to 28 weeks and NrtI therapy for at least 18 months, will be randomized 1:1 to receive BRII-179 (VBI-2601) or placebo in addition to the existing PEG-IFN-α and NrtI therapy. Subjects will be stratified at randomization by screening HBsAg level (3 categories: ≥0.05 to < 1 IU/mL, ≥1 to < 10 IU/mL, and ≥10 to <100 IU/mL). Subjects will complete investigational treatment with BRII-179 or placebo at Week 18; PEG-IFN-α treatment will last for up to 24 weeks after enrollment (a total of 48 weeks of PEG-IFN-α treatment before and after enrollment); while NrtI therapy will continue into the Follow-up Period. The BRII-179-002 study design schema can be found in Section 1.4.

Estimated total duration for each subject will include the following: screening up to 28 days, 24-week dosing period (BRII-179 treatment for 18 weeks and PEG-IFN-α treatment for up to 24 weeks), and up to 28-week follow-up period. Eligible subjects that meet NrtI discontinuation criteria (Section 4.1.2.1) (eligibility assessed at Week 36 to Week 48) may enter a NrtI discontinuation monitoring period for an additional 48 weeks from the day of NrtI discontinuation (Section 1.5.2).

4.1.1. Independent DSMB

An independent DSMB will monitor patient safety and treatment efficacy data for this study. The details for the operation and responsibilities of the DSMB will be defined in a separate DSMB Charter. The Charter will delineate the composition, duties, responsibilities, and procedures of the DSMB, data required at each meeting, as well as the analyses that will be conducted.

4.1.2. NrtI Discontinuation

4.1.2.1. NrtI Discontinuation Criteria

The investigator will evaluate whether subjects are eligible for discontinuation of NrtI therapy starting from Week 36 and potentially monthly until Week 48 based on the

following NrtI discontinuation criteria:

- Alanine aminotransferase (ALT) < 2x ULN (results from previous visit) AND
- HBV DNA < LLOQ (results from previous visit) AND
- 2 consecutive assessments of HBsAg < 0.05 IU/mL (assessed starting from Week 28 laboratory results)

Subjects who are eligible to discontinue from NrtI therapy may discontinue from the Schedule of Activities in Section 1.5.1 and may instead continue into the NrtI Discontinuation Monitoring Period of the study. These subjects will follow the Schedule of Activities for subjects who have discontinued from the NrtI therapy (Section 1.5.2). Subjects who discontinue from their NrtI therapy will be followed in the NrtI Discontinuation Monitoring Period for an additional 48 weeks from their day of NrtI discontinuation.

4.1.2.2. NrtI Re-treatment

It is recommended that the Investigator should consider re-treatment with NrtI therapy during the NrtI Discontinuation Monitoring Period if subjects meet the following criteria at the same visit (based on Papatheodoridi, 2020):

- ALT > 15x ULN with positive HBV DNA
- ALT 10-15x ULN with positive HBV DNA: retreatment may be implemented at investigator's discretion
- ALT 5-15x ULN with elevated total bilirubin > 2 mg/dL (predominantly direct) and/or prolongation of prothrombin time (PT) ≥ 3 seconds with positive HBV DNA
- ALT 2-5x ULN and HBV DNA >2000 IU/mL persisting for ≥ 12 consecutive weeks
- ALT > 3x ULN and HBV DNA > 100,000 IU/mL at the same visit

Whether subjects are re-treated with NrtI is at the discretion of the Investigator following good medical judgement. Subjects who are re-treated with NrtI therapy should continue to be followed per the SoA Section 1.5.2 for the duration of the 48-week NrtI Discontinuation Monitoring Period.

4.1.3. PEG-IFN-α Therapy During the Dosing Period

Subjects entering the Dosing Period of the study will continue their peg-IFN-α treatment, the investigator should monitor and medically manage relevant side effects as necessary. The dose of peg-IFN-α is based on relevant clinical guidelines and information from the package insert which is considered as proper by the investigator. Temporary interruptions of peg-IFN-α administration are discouraged. Subjects are to remain on treatment for the entire duration of the study until completion of Week 24 and the dose of peg-IFN-α should not be changed during the trial unless dose reduction or discontinuation is indicated because of laboratory abnormalities/adverse events (see Section 4.1.3.1 for instructions for

dose reduction). Subjects must be counselled regarding the importance of not missing doses of peg-IFN-α.

In the case that a patient does miss a dose(s):

- If the peg-IFN-α injection was late by 1 to 3 days, the next dose should be administered immediately. The subsequent dose should then be administered on the next scheduled injection date.
- If the peg-IFN-α injection was late by 4 to 7 days, patient should wait to receive the next scheduled injection (1 dose is skipped).
- If the peg-IFN-α injection was late by >7 days, treatment can be restarted at any time by the investigator and the next dose should be on scheduled injection dates.

4.1.3.1. Dose Adjustment for adverse drug reactions

If a PEG-IFN-α dose adjustment is required due to moderate to severe adverse reactions (including clinical manifestations and/or laboratory abnormalities), the initial dose reduction should be performed with clinical consideration. However, in some cases, further dose reduction may be needed. As the adverse reactions abate, re-escalation or recovery of the initial dose of PEG-IFN-α may be considered.

Hematology:

Guidelines for Peg-IFN-α Dose Adjustment According to Hematology results

Laboratory Values	PEG-IFN-α Dose Reduction:	Discontinue PEG-IFN-α if
Absolute neutrophil count (ANC)	≥750/mm^3: Maintain dose<br>< 750/mm^3: Reduce dose	< 500/mm^3: Treatment should be suspended until ANC values return to more than 1000/mm^3<br>Re-institute at low dose and monitor ANC
Platelet	≥ 50.000/mm^3: Maintain dose<br>< 50.000/mm^3: Reduce to low dose	<25.000/mm^3

Liver function:

In patients with progressive or persistent ALT increases > 10 x ULN, the dose of peg-IFN-α should be reduced and more frequent monitoring of liver function should be performed. If ALT increases are further progressive despite dose reduction or accompanied by increased bilirubin or evidence of hepatic decompensation, therapy should be immediately discontinued. After dose reduction or interruption of PEG-IFN-α, treatment may be re-instituted at the discretion of the investigator after improvement in liver function.

Other disorders:

Peg-IFN-α may cause or aggravate neuropsychiatric, autoimmune, endocrine, ophthalmologic, and infectious disorders. Monitor closely and withdraw therapy with persistently severe or worsening signs or symptoms of the above disorders. Please see package insert for more information.

4.2. Scientific Rationale for Study Design

This is a phase 2, double-blind, randomized, placebo-controlled, parallel-group study of adding BRII-179 (VBI-2601) to existing PEG-IFN-α and NrtI therapy in patients with chronic HBV infection without cirrhosis enrolled from the Everest Project study being conducted in China. The study design will provide an opportunity to evaluate the ability of BRII-179 (VBI-2601) to achieve a clinically meaningful increase in functional cure (sustained loss of HBsAg in blood with or without seroconversion to HBsAb) in subjects with chronic HBV on optimized PEG IFN-α and NrtI therapy as compared to therapy consisting of optimized PEG IFN-α and NrtI therapy alone.

There is increasing evidence that low levels of quantitative HBsAg may improve the potential for HBsAg loss, and this is most evident in patients who lose HBsAg after stopping long-term NrtI therapy. It has been observed that patients with decreasing HBsAg and a lower HBsAg level < 100 IU/mL at the end of treatment were able to maintain control of HBV after discontinuation of long-term NrtI therapy (Chang, 2015; Yao 2017; Jeng, 2018; Papatheodoridi, 2020). Recent reporting from the Everest Project seems to corroborate this observation from previous studies. In 1427 patients on background NrtI therapy who have completed 24 to 26 weeks of PEG-IFN-α treatment and 516 patients who have completed 48 weeks of PEG-IFN-α treatment, the overall rate of HBsAg loss for patients treated ≥12, ≥24, ≥36 and ≥48 weeks was 13.00%, 16.06%, 20.49% and 23.01%, respectively. Subgroup analysis based on baseline HBsAg stratification (0.05-100, 100-500, 500-1000, 1000-1500 IU/mL) found that the HBsAg loss rate was highest in patients with baseline HBsAg between 0.05 and 100 IU/mL (34.93% for patients treated ≥ 48 weeks) (Ze-Qian, 2020).

Viral rebound may occur shortly after NrtI discontinuation in some cases, which requires close monitoring during study participation in order to control ALT flares. The HBV viral relapse after NrtI discontinuation may induce the host immune response and potentially a long-term immune control against the viral infection. Sustained HBsAg loss and/or virological response were observed in HBeAg-negative patients discontinuate NrtI therapy in prospective cohort studies (van Bömmel) as well as in a randomized controlled study (Barthel FM-S, Parmar MKB, Royston P. How do multi-stage, multi-arm trials compare to the traditional two-arm parallel group design - a reanalysis of 4 trials. Trials 2009; 10:21, doi:.10.1186/1745-6215-10-21.

Berg). This study design was designed to evaluate the potential benefit of decreased viral antigen levels in immune responses to BRII-179 treatment after discontinuation of long-term NrtI therapy.

Surrogate endpoints of HBV functional cure measure biochemical, virological, and histological outcomes. Based on current knowledge, the best surrogate of HBV functional cure is HBsAg loss confirmed on 2 occasions at least 6 months apart with or without HBsAg seroconversion plus HBV DNA below the detection offline (Choodari-Oskooei B, Parmar MKB, Royston P, Bowden J. Impact of lack-of-benefit stopping rules on treatment effect estimates of two-arm multi-stage (TAMS) trials with time to event outcome. Trials 2013, 14:23, http://www.trialsjournal.com/content/14/1/23.

Cornberg, 2020). The incidence of sustained HBsAg loss and undetectable HBV DNA will be used as primary endpoints to assess the potential of BRII-179 (VBI-2601) to induce functional cure.

The target population of this study is HBeAg-negative non-cirrhotic patients with chronic HBV infection that has been virologically suppressed (baseline HBsAg ≥ 0.05 IU/mL and < 100 IU/mL). In the Everest program, we found that some patients had been treated with PEG-IFN-α for 24-28 weeks and NrtI for 18 months and had a preliminary HBsAg decline with HBV DNA below the detection limit, however, could not achieve HBsAg loss upon completion of 48 weeks of PEG-IFN-α treatment. We believe that the immunomodulatory effects of BRII-179 as a therapeutic vaccine can further enhance HBsAg decline and ultimately achieve HBsAg loss, leading to functional cure.

4.3. Dosage Instructions

4.3.1. Justification for Dose Selection and Number of Doses

Sci-B-Vac® has been evaluated previously as a therapeutic vaccine at 20μg and 40 μg dose in multiple clinical studies in chronic HBV patients (Hoa, 2009, Lo, 2007; Roggendorf, 2019). Sci-B-Vac® was well tolerated at these higher doses. Sci-B-Vac® also demonstrated good safety profile when assessed in liver transplant patients at a high dose of 40 μg (Wong, 2018). In a Phase 1b/2a randomized, open-label, controlled study (BRII-179-001) of BRII-179 completed in multiple countries and regions in the Asia-Pacific region including China, 44 subjects with chronic hepatitis B received 40 μg BRII-179 intramuscularly every 4 weeks for a total of 4 doses and it was demonstrated that the drug was safe and well tolerated. Based on these clinical safety data, we selected 40 μg per dose as the dose administered in this study.

In Study BRII-179-001, BRII-179 every 4 weeks for a total of 4 doses elicited HBV-specific T- and B-cell responses in a subset of subjects, however, the response was significantly delayed and with shorter duration compared to that in healthy subjects, and this delayed and low immune response phenomenon coincided with the predicted chronic hepatitis B immunocompromise or tolerance. This study also showed that the intensity of HBV-specific immune responses induced by 4 doses of BRII-179 was not sufficient to achieve a significant HBsAg reduction. In order to achieve better efficacy and considering previously published data of other therapeutic vaccines, we believe that an increase in the number of doses of BRII-179 is needed. The completed preclinical long-term toxicology studies in New Zealand White rabbits with drug administered every 2 weeks for a total of

10 doses over 18 weeks can support clinical studies up to 18 weeks. In addition, dosing every 3 weeks in the AAV-HBV mouse model supported the induction and maintenance of an effective immune response based on nonclinical pharmacodynamic studies. Therefore, we plan to conduct a clinical study with BRII-179 administrated every 3 weeks for a total of 7 doses over 18 weeks to explore the possibility of HBsAg loss.

In summary, currently available safety and tolerability data of BRII-179 (VBI-2601), as well as the safety assessment from the toxicology studies, support the proposed BRII-179 (VBI-2601) treatment regimen to explore functional cure in patients with chronic HBV infection.

4.3.2. Maximum dose/exposure in this study

The maximum dose of BRII-179 is 40 μg every 3 weeks. No other dose adjustment is planned in this study.

4.4. End of Study Definition

A subject is considered to have completed the study if he/she has completed all phases of the study including the last scheduled procedure shown in the Schedule of Activities.

The end of the study is defined as the date of the last visit of the last subject in the study or last scheduled procedure shown in the Schedule of Activities for the last subject in the trial.

5. STUDY POPULATION

A total of 600 Chinese nationality subjects (approximately 120 subjects are planned to be enrolled in Part I (Phase 2a) and approximately 480 subjects are planned to be enrolled in Part II (Phase 2b)) with virologically suppressed chronic HBV infection without cirrhosis receiving PEG-IFN-α and NrtI therapy are planned for this study.

Prospective approval of protocol violations to recruitment and enrollment criteria, also known as protocol waivers or exemptions, is not permitted. Adherence to the criteria as specified in the protocol is essential.

5.1. Inclusion Criteria

Everest Project subjects eligible to be included in the study must meet all of the following criteria. All laboratory test requirements for inclusion will be performed by central laboratory unless specified otherwise.

1. A signed and dated written Informed Consent Form (ICF) prior to study participation.
2. Age ≥ 18 (or age of legal consent, whichever is older), and ≤ 60 at time of screening.
3. Documented chronic HBV infection (positive serum HBsAg for ≥ 6 months, negative HBsAb).
4. Negative HBeAg
5. HBsAg ≥ 0.05 IU/mL and < 100 IU/mL at screening.
6. HBV DNA < 100 IU/mL at screening (central lab).
7. Must be receiving first-line NrtI (ETV or TDF or TAF) therapy for ≥ 18 months and PEG-IFN-α for 24 to 28 weeks of the Day 1 visit and could provide the related treatment history.
8. Female subjects must have a negative pregnancy test or confirmation of post-menopausal status. Post-menopausal status is defined as 12 months with no menses without alternative medical cause. Women of child-bearing potential (WOCBP) must have a negative serum pregnancy test at screening and a negative urine pregnancy test on Day 1, cannot be breast feeding, and must be willing to use highly effective methods of contraception 14 days before study drug administration through 12 weeks after last study drug administration.
9. Agrees to not donate blood during the duration of the study.
10. Serum ALT and AST ≤ 2x ULN at screening. Study screening laboratory tests may be repeated once for values considered to be erroneous with medical monitor approval.

5.2. Exclusion Criteria

Everest Project subjects are excluded from the study if any of the following criteria apply:

1. Any clinically significant chronic medical condition other than chronic HBV infection that, in the opinion of the investigator makes the patient unsuitable for participation in the study.

2. Any clinically significant acute condition such as fever (> 38° C) or acute respiratory illness within 7 days of first study drug administration.

3. Significant liver fibrosis or cirrhosis as defined by having either a FibroScan result of > 9kPa at screening, unless the subject has results from a FibroScan evaluation performed within 6 months prior to screening or liver biopsy performed within 1 year prior to screening that confirm the absence of Metavir F3 fibrosis or F4 cirrhosis. If a subject has had both procedures in the specified timeframes, the most recent result should be used to determine eligibility.

4. History or evidence of drug or alcohol abuse as assessed by the Investigator, within the 12 months before screening.

5. History of intolerance to IM injection.

6. History of clinically significant chronic liver disease from any cause other than chronic HBV infection.

7. History of hepatic decompensation, including ascites, hepatic encephalopathy and/or esophageal or gastric varices.

8. Treatment by immunosuppressant (including but not limited to corticosteroids at a prednisolone equivalent dose > 20 mg/day) within 30 days before study drug administration at the Day 1 visit and throughout the duration of study participation.

9. Known history of immunological function impairment, including but not limited to:

   - Autoimmune disease (e.g., multiple sclerosis, type 1 diabetes mellitus , myasthenia gravis, Crohns disease and other inflammatory bowel diseases, celiac disease, systemic lupus erythematosus, scleroderma, including diffuse systemic form and limited cutaneous form of systemic sclerosis or CREST syndrome, systemic sclerosis, dermatomyositis polymyositis, rheumatoid arthritis, juvenile idiopathic arthritis, autoimmune thyroiditis including Hashimoto thyroiditis, Grave’s or Basedow’s disease, immune thrombocytopenic purpura, autoimmune hemolytic anemia, autoimmune hepatitis, psoriasis, vitiligo, vasculitis, Guillain Barré syndrome, Addison’s disease, Bell’s palsy, and alopecia areata); OR

   - Primary immunodeficiency disorders (e.g., common variable immune deficiency, defective phagocytic cell function and neutropenia syndromes, and complement deficiency); OR

- Secondary immunodeficiency disorders (e.g., resulting from acquired immunodeficiency syndrome caused by human immunodeficiency virus (HIV) infection, solid organ transplant, and splenectomy).

10. Any HBV vaccine (licensed or experimental) prior to study enrollment and throughout the duration of study participation.

11. Immunization with attenuated vaccines (e.g., Mumps, Measles, Rubella) within 30 days before study drug administration at the Day 1 visit and throughout the duration of study participation.

12. Has received blood products or immunoglobulin within 90 days before study drug administration at the Day 1 visit or likely to require blood products during the study.

13. Participated in another clinical trial with an investigational drug or a biologic within 30 days or within 5 drug half-lives (whichever is longer) before study drug administration at the Day 1 visit. Subjects must agree not to take part in any other study at any time during their participation in this study, inclusive of the follow-up period.

14. Previous or current psychiatric condition that precludes compliance with the protocol. Specifically excluded are persons with psychoses, ongoing risk for suicide, or history of suicide attempt or gesture within the past 5 years.

15. The subject has any history of cancer requiring locoregional or systemic therapy within 5 years of randomization or has current disease. Subjects with completely surgically resected tumors with no evidence of disease for more than 3 years, or a history of low risk basal cell carcinoma will be accepted (low risk being defined as 1) location on the trunk of the body, arms, legs, cheeks, forehead, temples, scalp, neck, or chin, 2) < 2 cm in size, 3) nodular or superficial, 4) primary cancer that has not come back after treatment, 5) edge of the cancerous area is clear and smooth, and 6) not located in or around nerves).

16. Any skin abnormality or tattoo that would limit post treatment injection site assessment.

17. Known hypersensitivity or contraindication to BRII-179 (VBI-2601) or its excipients.

18. Unwilling, or unable in the opinion of the investigator, to comply with study requirements, including the use of an adequate birth control method.

19. Active infection with human immunodeficiency virus (HIV), hepatitis C virus (HCV) or hepatitis Delta virus.

20. Donated more than 500 mL of blood within 90 days before study drug administration.

21. Renal impairment with estimated glomerular filtration rate (eGFR) ≤ 60 ml/min/1.73 m^2 at screening.

22. Uncontrolled diabetes mellitus as determined by the Investigator.

23. Diagnosis of advanced stage heart failure (New York Heart Association Class III or Class IV or unstable angina).

24. Any laboratory test abnormality that is considered as clinically significant or may contradict continued treatment with peg-IFN by the investigator.

5.3. Lifestyle Considerations

Alcohol consumption is limited to no more than 1 unit per day (unit: 1 glass of wine [125 mL] = 1 measure of spirits [30 mL] = one-half pint of beer [284 mL]) during the participation of this study.

5.4. Screening Failures

Screening failures are defined as subjects who consent to participate in the clinical study but are not subsequently randomly assigned to study drug. A minimal set of screening failure information is required to ensure transparent reporting of screening failure subjects to meet the Consolidated Standards of Reporting Trials (CONSORT) publishing requirements and to respond to queries from regulatory authorities. Minimal information includes demography, screen failure details, eligibility criteria, and any serious adverse event (SAE).

Individuals who do not meet the criteria for participation in this study (screening failure) may not be rescreened.

6. STUDY INTERVENTION

6.1. Study Intervention(s) Administered

Study intervention is defined as any study intervention(s), marketed product(s), placebo, or medical device(s) intended to be administered to a study participant according to the trial protocol.

Clinical study intervention drugs provided by the Sponsor will be packaged to support enrollment. Clinical study drugs will be affixed with clinical label in accordance with regulatory requirements.

6.1.1. Formulations

The following study drugs will be used and provided in this study:

- BRII-179 40µg IM injection
- Placebo IM injection
- PEG-INF-α Subcutaneous Injection
- NrtI (either entecavir tablets, tenofovir disoproxil tablets or tenofovir alafenamide fumarate tablets) oral administration

BRII-179 (VBI-2601) is aluminium phosphate absorbed HBsAg Bulk Drug Substance (HBsAg spherical particles [~30 nm mean diameter] suspended in phosphate-buffered saline) and 8% Sucrose, in addition to 10 mM phosphate buffer.

Placebo will be prepared from commercially available 0.9% Sodium Chloride for Injection which will be provided to the study site. Subjects assigned to placebo will receive a volume of placebo that would be equal to the volume that would be required if that subject had been assigned to IP.

PEG-INF-α is a colorless, clear liquid formulation of peginterferon α-2b from modified recombinant human interferon α2b expressed in yeast system with polyethylene glycol (40 kD Y type) and excipients of sodium chloride, sodium acetate, mannitol, aspartic acid, water for injection, and sodium hydroxide to adjust pH.

NrtI may be either entecavir tablets, tenofovir disoproxil tablets or tenofovir alafenamide fumarate tablets, and the type of NrtI during treatment should be consistent with that before enrollment (e.g., if entecavir is used 3 months before enrollment, then entecavir should be continued after enrollment). Nrtl should be given orally one tablet daily and should not be discontinued or changed *ad libitum* until discontinuation according to protocol defined criteria or treatment regimen change due to drug resistance.

6.1.2. Study Intervention

**Arm Name**	**Group 1**	**Group 2**	**Group 1 & 2**	**Group 1 & 2**
**Intervention Name**	BRII-179 (VBI-2601)	Placebo	PEG-INF-α	NrtI (entecavir tablets or tenofovir disoproxil fumarate tablets or tenofovir alafenamide fumarate tablets)
**Type**	Drug	Placebo	Drug	Drug
**Dose Formulation**	Solution for Injection	0.9% Sodium Chloride for Injection	Injection	Tablet
**Dose Level(s)**	BRII-179 40 µg	Placebo	180µg	0.5mg/300mg/25mg
**Route of Administration**	BRII-179 in one syringe; administer via IM injection<br><br>Refer to the Pharmacy Manual for the details.	Placebo in one syringe; administer via IM injection<br><br>Refer to the Pharmacy Manual for the details.	1 syringe; Subcutaneous Injection<br><br>Refer to the Pharmacy Manual for the details.	1 tablet, oral<br><br>Refer to the Pharmacy Manual for the details.
**Use**	Experimental	Experimental	Experimental	Experimental
**IMP and NIMP**	IMP	IMP	IMP	NIMP
**Sourcing**	Provided centrally by the Sponsor	Provided centrally by the Sponsor	Provided centrally by the Sponsor	Provided centrally by the Sponsor
**Packaging and Labelling**	IMP will be labelled as required per country requirement.	Placebo will be prepared and labelled as instructed in the Pharmacy Manual	IMP will be labelled as required per country requirement.	NIMP will be labelled as required per country requirement.

On dosing days, the pharmacist or designee will prepare the required amount of study drugs into syringe for injection. A qualified clinical investigative site staff member under the supervision of the investigator or designee will administer the study drug. The injection site will be marked and mapped for later observation and should be documented. If a local reaction around the injection site occurs, photographs may be obtained. Please refer to Section 6.6 for further details.

6.2. Preparation/Handling/Storage/Accountability

- Only subjects enrolled in the study may receive study drug and only authorized site staff may supply or administer study drug. All study drug must be stored in a secure, environmentally controlled, and monitored (manual or automated) area in accordance with the labeled storage conditions with access limited to the investigator and authorized site staff.
- The investigator, institution, or the head of the medical institution (where applicable) is responsible for study drug accountability, reconciliation, and record maintenance (i.e., receipt, reconciliation, and final disposition records).
- Further guidance and information for the final disposition of unused study drugs are provided in the Pharmacy Manual.

6.3. Randomization and Blinding

Subjects will be assigned to study treatment in accordance with the randomization schedule. The randomization code will be generated by using a validated computerized system. Subjects will be randomized using an Interactive Web Response System (IWRS). Once a randomization number is assigned to a subject, it cannot be reassigned to any other subject in the study. Study intervention will be dispensed at the study visits summarized in SoA (Section 1.5.1). Returned study intervention should not be re-dispensed to the subjects.

Study drug will be double-blind. Neither the subject nor the study physician will know which study drug the subject is receiving. The investigator or treating physician may unblind a subject's treatment assignment **only in the case of an emergency** when knowledge of the study treatment is essential for the appropriate clinical management or welfare of the subject. Whenever possible, the investigator must first discuss options with the Medical Monitor or appropriate sponsor study personnel before unblinding the subject's treatment assignment. If this is impractical, the investigator must notify the sponsor as soon as possible, but without revealing the treatment assignment of the unblinded subject, unless that information is important for the safety of subjects currently in the study. The date and reason for the unblinding must be recorded in the appropriate data collection tool.

Sponsor clinical safety and pharmacovigilance staff may unblind the treatment assignment for any subject with an SAE. If the SAE requires that an expedited regulatory report be sent to one or more regulatory agencies, a copy of the report, identifying the subject's treatment assignment, may be sent to clinical investigators in accordance with local regulations and/or sponsor policy.

The primary reason for treatment discontinuation (the event or condition that led to the unblinding) will be recorded in the eCRF. Subject may continue in the follow-up period of the study once the treatment code is unblinded (see Section 7.1 Discontinuation of Study Intervention).

Subjects will receive study drugs directly from the investigator or designee, under medical supervision. The date and time of each dose administered in the clinic will be recorded in the source documents and recorded in the eCRF. The dose of study drug and study subject identification will be confirmed at the time of dosing by a member of the study site staff other than the person administering the study drug.

6.4. Compliance of Study Intervention

In the event of an interruption of study drug treatment ≥ 2 weeks as specified in the protocol, discussion between the investigator and the Sponsor is required, and the decision on subject management should be documented in writing.

Subjects will be instructed to bring NrtI and PEG-IFN-α (including all empty containers) to the trial site at every visit, as applicable, beginning with the screening visit. Adherence will be assessed by counting of empty containers and unused medication.

Every effort should be made to ensure good adherence with NrtI and PEG-IFN-α therapy. Adherence to NrtI and PEG-IFN-α should be assessed at every relevant visit. Adherence of peg-IFN-α will be assessed by counting of empty containers and unused medication. Subjects will be instructed to bring all empty containers of PEG-IFN-α to the trial site at every visit, as applicable, for adherence assessment. Adherence of NrtI will be assessed via review of the information on the diary cards filled in by the subjects.

6.5. Concomitant Therapy

The study will collect all prescription and over-the-counter medications (including traditional Chinese medicine and herbal medicine) that the subject receives from screening and throughout the study.

Any medications that the subject is receiving at the time of enrolment or receives during the study must be recorded along with:

- Reason for use
- Dates of administration including start and end dates
- Dosage information including dose and frequency

Should subjects have a need to initiate treatment with any excluded concomitant medication, the Medical Monitor must be consulted prior to initiation of the new medication. In instances where an excluded medication is initiated prior to discussion with the Sponsor, the investigator must notify the Sponsor as soon as he/she is aware of the use

of the excluded medication. The Medical Monitor should be contacted if there are any questions regarding concomitant or prior therapy.

6.5.1. Permitted Medications

The following medications/treatments are permitted:

- Hormone replacement therapy.
- Oral, injectable, subdermal, intravaginal, or implantable contraceptives, as well as intrauterine device, and intrauterine hormone-releasing system are permitted for contraception.
- Inhaled or topical steroids are allowed.
- Prescription drugs necessary to treat hypertension. Hypertension must be well controlled on a stable dose of medication for > 6 months prior to study drug administration at Day 1 visit.
- Prescription drugs necessary to treat diabetes mellitus. Diabetes mellitus must be controlled on a stable dose of medication for > 6 months prior to study drug administration at Day 1 visit.
- Paracetamol (≤ 2 g/day), aspirin (≤ 3 g/day), or ibuprofen (< 1.2 g/day).
- Antihistamines.
- Prescription inhaler or nebulizer to treat asthma (inhaled bronchodilator or inhaled steroid). Asthma must be well controlled, defined as historical use of a rescue bronchodilator on average no more than twice per week.
- A stable dose and regimen of other medication(s) (prescription or OTC) that the subject is taking regularly (i.e. medications for chronic conditions such as high cholesterol or depression must be unchanged in dose or type for at least 6 months) is permitted.
- Licensed SARS-CoV-2 vaccines, including those authorized for emergency use by local authorities, for the prophylaxis of COVID-19 are allowed. Each COVID-19 vaccine administration should be ideally with a minimum interval of 14 days before or after BRII-179 (VBI-2601) administration. However, COVID-19 vaccines may be administered within a shorter period in situations to avoid barriers or delays to COVID-19 vaccination.

6.5.2. Prohibited Medications

The following medications are excluded while subjects are participating in the study.

Prior to study enrollment and throughout the duration of study participation:

- Any HBV vaccine (licensed or experimental)

Within 30 days before study drug administration at the Day 1 visit and throughout the duration of study participation:

- Immunosuppressant (including but not limited to corticosteroids at a prednisolone equivalent dose > 20 mg/day)
- Immunization with attenuated vaccines (e.g., Mumps, Measles, Rubella), except COVID vaccines
- Any investigational drug or biologic other than the study product(s) used during this study

Any herbal remedies that may cause fluctuations of liver test results

Within 90 days before study drug administration at the Day 1 visit and throughout the duration of study participation:

- Blood products or immunoglobin

6.6. Safety Follow-up and Management of Reactions to Study Drug

The following safety observation procedures will be performed immediately following study drug administration and during the follow-up period for all subjects:

- Subjects will remain in the clinic for at least one hour post study drug administration. The observation period will include an assessment for rare adverse reactions.
- Any unusual signs or symptoms reported during the initial one hour of observation will prompt continued close monitoring.
- Based on their condition, subjects may be asked to remain in the clinic for more than one hour after the study drug administration (reason will be recorded in source data).
- All data (including assessment of solicited local and systemic reactions) will be recorded in the source document during and after the post-observation period.

A measurement device template (in mm) for measuring solicited local reactions (erythema [redness] and swelling), and an oral thermometer for measuring daily temperature, will be provided at the first study drug administration visit, study Day 1. Subjects will be advised to record their temperature in the diary card.

Refer to Sections 8.3 and 10.3 for further details on the recording, assessment, and reporting of AEs.

6.7. Treatment of Study Intervention Overdose

An overdose is defined as an accidental or intentional administration of a quantity of a medicinal product given per administration or cumulatively which is above the maximum recommended dose as per protocol. In the event of an overdose of study medication, the

investigator should use clinical judgement in treating the overdose and contact the study medical monitor.

No specific treatment guidelines for overdose and toxicity management is provided by the Sponsor. The investigator is advised to refer to the relevant document(s) for detailed information pertaining to the study drug being used in this study. Such documents may include, but not be limited to, the IB or equivalent document provided by the Sponsor.

6.8. Intervention after the End of the Study

The Sponsor will not provide post-study treatment. The investigator is responsible for ensuring that consideration has been given to the post-study care of the patient’s medical condition.

7. DISCONTINUATION OF STUDY INTERVENTION AND SUBJECT DISCONTINUATION/WITHDRAWAL

7.1. Discontinuation of Study Intervention

Discontinuation of study intervention does not mean withdrawal from the study.

If a subject discontinues study, every effort should be made to keep the subject in the study. If the subject discontinues both investigational product (BRII-179 or placebo) and PEG-INF-α treatment, the subject should continue to attend the required study visits and procedures for the remaining duration of study participation in Follow-up Period.

Subjects who have discontinued study drug may be reassessed for possible re-institution of study drug dosing not to exceed the protocol defined number of doses, dosing interval, or dosing period duration. Subjects who have discontinued study drug will be assessed for NrtI discontinuation as described in Section 4.1.2.1 upon continued study participation into the Follow-up Period. If NrtI discontinuation criteria are met, 48-week NrtI discontinuation monitoring should be completed according to the protocol.

If continued study participation is not possible or acceptable to the subject or investigator, please refer to Section 7.2 Subject Discontinuation/Withdrawal from the Study.

7.1.1. Criteria for Discontinuation of Study Intervention

Subjects who have received at least one dose of BRII-179 (VBI-2601) will have additional doses withheld but continue to undergo follow-up monitoring in the study if any of the following criterion is met:

- Discontinuation of study intervention requested by the subject or the subject's legal representative.
- Signs or symptoms of severe (NCI-CTCAE Grade 3 or higher) hypersensitivity or anaphylaxis
- Significant abnormalities in liver chemistry, defined as any of the following are met:
  - Serum ALT > 10 × ULN
  - Serum ALT or AST > 3 × ULN with a concomitant total bilirubin > 2 × ULN or INR > 1.5
- Any clinical manifestations of hepatic decompensation

- The Sponsor may request DSMB evaluation of specific data in the event of liver flare to fully characterize transaminase elevations. The subject may receive remaining doses of study drug only after DSMB approval.
- The subject has a medical condition or personal condition that, in the opinion of the investigator and/or the Sponsor, would pose an unnecessary risk to the subject if the investigational intervention is continued.
- The subject's serum pregnancy test is confirmed to be positive.

7.2. Subject Discontinuation/Withdrawal from the Study

- A subject may withdraw from the study at any time at his/her own request or may be withdrawn at any time at the discretion of the investigator for safety, behavioral, compliance, or administrative reasons. This is expected to be uncommon.
- At the time of discontinuing from the study, if possible, an Early Termination Visit should be performed (Section 8.1.7) for data to be collected at the time of study discontinuation and follow-up. The subject will be permanently discontinued from both the study intervention and from the study at that time.
- If the subject withdraws consent for disclosure of future information, the sponsor may retain and continue to use any data collected before such a withdrawal of consent.
- If a subject withdraws from the study, he/she may request destruction of any samples taken and not tested, and the investigator must document this in the site study records.
- Subjects who are withdrawn from the study will NOT be replaced and cannot be rescreened.
- The primary reason for subject withdrawal will be recorded in the eCRF. Primary reasons for withdrawal will be categorized as:
    - Adverse event
    - Protocol deviation
    - Withdrew consent, not due to an AE
    - Lost to follow up
    - Study closed/terminated

7.2.1. Lost to Follow up

A subject will be considered lost to follow-up if he or she repeatedly fails to return for scheduled visits and is unable to be contacted by the study site.

The following actions must be taken if a subject fails to return to the clinic for a required study visit:

- The site must attempt to contact the subject and reschedule the missed visit as soon as possible and counsel the subject on the importance of maintaining the assigned visit schedule and ascertain whether or not the subject wishes to and/or should continue in the study.
- Before a subject is deemed lost to follow up, the investigator or designee must make every effort to regain contact with the subject (where possible, 3 telephone calls and, if necessary, a certified letter to the subject's last known mailing address or local equivalent methods). These contact attempts should be documented in the subject's medical record.
- Should the subject continue to be unreachable, only then will he/she be considered to have withdrawn from the study with a primary reason of "Lost to Follow-up".

Discontinuation of specific sites or of the study as a whole are handled as part of Section 10.1.7, Appendix 1.

8. STUDY ASSESSMENTS AND PROCEDURES

8.1. Schedule of Assessments

Study procedures and their timing are summarized in the SoAs (Section 1.5). Adherence to the study design requirements, including those specified in the SoAs, is essential and required for study conduct. Protocol waivers or exemptions are not allowed. Immediate safety concerns should be discussed with the sponsor immediately upon occurrence or awareness to determine if the subject should continue or discontinue study drug.

8.1.1. Screening

Written informed consent must be obtained prior to initiation of any study procedures. All screening evaluations must be completed and reviewed to confirm that potential subjects meet all eligibility criteria. The investigator will maintain a screening log to record details of all subjects screened and to confirm eligibility or record reasons for screening failure.

Screening will be performed no more than 4 weeks prior to randomization and will include written consent, determination of subject eligibility, collection of demographics and medical history/ current medical conditions as well as a physical examination, vital signs, 12-lead ECG, clinical laboratory tests, and other assessments per the SoA (Section 1.5.1).

Adverse events related to screening activities must be collected from the time of consent until the end of the study; any other events reported during the screening period should be reported as medical history. All SAEs must be collected from the time of consent until end of study.

8.1.2. Randomization (Day 1)

Eligible subjects will be randomized 1:1 to either 40 µg of BRII-179 administrated IM or matching placebo administrated IM. Subjects will receive their first dose of study drug on Day 1, however subject eligibility must be confirmed prior to study drug administration and blood sample collections will be made prior to study drug administration. Other assessments to be conducted on Day 1 are detailed in the SoA (Section 1.5.1).

8.1.3. Dosing Period

All subjects will receive BRII-179 (VBI-2601) or placebo administered IM every 3 weeks for a total of 7 doses. Subjects will receive PEG-IFN-α at the indicated dose level weekly and discontinue investigational treatment with BRII-179 or placebo at Week 18 and discontinue PEG-IFN- α at Week 20-24; while NrtI therapy will continue into the Follow-up Period.

Subjects will return to the study site clinic on an outpatient basis for antiviral activity monitoring and safety assessment as specified in the SoA (Section 1.5.1).

Prior to study drug administration, there will be a review of ongoing eligibility to receive the study drug and continue with study participation. Diary cards will be collected and

there will be a review of any local and systemic reactions. Blood samples will be collected prior to each study drug administration.

8.1.4. Follow-up Period

Subjects will return to the investigative site for the specified assessments per the SoA (Section 1.5.1), including but not limited to physical examination, vital signs, clinical laboratory tests, and review of adverse events and concomitant medications.

Subjects will be assessed by the investigator whether they are eligible for discontinuation of NrtI therapy starting at Week 36 and potentially monthly until Week 48. Subjects that have NOT achieved NrtI discontinuation criteria by Week 48 will be discharged from the study.

8.1.5. NrtI Discontinuation Monitoring Period

Eligible subjects who meet NrtI discontinuation criteria will be informed by the investigator to discontinue their NrtI medications on the first day of their scheduled Day 1 visit of the NrtI Discontinuation Monitoring Period. These subjects will follow the NrtI Discontinuation Monitoring Period SoA (Section 1.5.2).

Subjects who discontinue from their NrtI therapy will be followed in the NrtI Discontinuation Monitoring Period for an additional 48 weeks from the day of NrtI discontinuation. Subjects who are retreated with NrtI therapy should continue to be followed per SoA (Section 1.5.2) for the duration of the 48-week NrtI Discontinuation Monitoring Period.

8.1.6. Unscheduled Visits

Subjects may be asked to come for an unscheduled visit for clinical assessment of an adverse event if warranted, as determined by the investigator. The unscheduled visit will be recorded in the eCRF.

8.1.7. Early Termination Visit

In cases where a subject prematurely discontinues from the study, the investigator must make every effort to have the subject return to the study site within 14 days for an Early Termination (ET) Visit. Assessments for ET visits will include but not limited to physical examination, pregnancy test, clinical laboratory tests, and other assessments per the SoA (Section 1.5.1). A follow-up visit will be conducted approximately 4 weeks after the last dose of study drug.

8.2. Detailed Description of Study Procedures

8.2.1. Medical History

A complete medical history, including details regarding illnesses and allergies, date(s) of onset, and whether condition(s) is currently ongoing (current medical conditions), medication history, including prior HBV treatment history and HBV genotype history (if available), will be collected on all subjects during screening and should be updated prior to dosing at the Day 1 visit.

8.2.2. Physical Examinations

A complete physical examination will include, at a minimum, general appearance, head, neck, chest/respiratory, heart/cardiovascular, gastrointestinal/liver, extremities, skin, and neurological assessments. Height and weight will also be measured and recorded. Body mass index will be calculated from height and weight.

A symptom-directed physical examination will be performed according to investigator discretion.

8.2.3. Fibroscan

To exclude the presence of cirrhosis, subjects will have a FibroScan evaluation, unless the subject has results from a FibroScan evaluation performed within 6 months prior to screening or liver biopsy performed within 1 year prior to screening that confirm the absence of Metavir F3 fibrosis or F4 cirrhosis. If a subject has had both procedures in the specified timeframes, the most recent result should be used to determine eligibility.

A FibroScan will also be conducted at the Week 48/ ET Visit in the NrtI Discontinuation Monitoring Period.

8.2.4. Alcohol Intake Assessment

Alcohol intake during the study will be recorded.

8.2.5. Vital Signs

Temperature (oral preferred), pulse rate, respiratory rate, and blood pressure will be assessed after the subject has rested comfortably for approximately 5 minutes. Pulse rate and blood pressure measurements will be assessed with a completely automated device. Manual techniques will be used only if an automated device is not available. Blood pressure should be based on 2 separate measurements. When scheduled at the same timepoints, vital signs and 12-lead ECG must be assessed first before physical examinations, blood sample collections, and urine collection.

8.2.6. Electrocardiograms

12-lead ECGs will be recorded and reviewed on-site by the investigator as outlined in SoA (Section 1.5.1).

8.2.7. Laboratory Assessments

8.2.7.1. Screening Viral Serology

The screening viral serology parameters will include chronic HBV infection and active infection with HIV, HCV, and hepatitis Delta virus. Chronic HBV infection is defined as serum HBsAg > 6 months.

8.2.7.2. Clinical Safety Laboratory Assessments

Fasting clinical safety laboratory tests to be performed are described in Section 10.2.

The investigator must review the laboratory report, document this review, and record any clinically significant changes occurring during the study in the AE section of the eCRF. The laboratory reports must be filed with the source documents. Clinically significant abnormal laboratory findings are those which are not associated with the underlying disease, unless judged by the investigator to be more severe than expected for the subject's condition.

In the event of an unexplained clinically relevant abnormal laboratory test occurring after study drug administration, the test should be repeated and followed at investigator discretion until it has returned to normal or baseline, and/or a diagnosis is made to adequately explain the abnormality.

8.2.7.3. Pregnancy Test

A pregnancy test or confirmation of post-menopausal status must be confirmed for all female subjects. Post-menopausal status is defined as 12 months with no menses without alternative medical cause. Pregnancy tests will be performed for WOCBP only. Serum and urine pregnancy testing will be performed per the SoAs (Section 1.5) and any time pregnancy is suspected. A WOCBP who is known to be pregnant or who does not have a negative pregnancy test at screening is not eligible for study participation. During the study, the results of these pregnancy tests must be known prior to study drug administration. A WOCBP determined to be pregnant while on study will be followed until the pregnancy outcome is known, as described in Section 8.3.5.1.

8.2.7.4. Assessment of Antiviral Activity

Assessment of antiviral activity will include HBsAg, HBsAb, and HBV DNA.

Details regarding the processing, shipping, and analysis of the samples will be provided in the Laboratory Manual.

8.2.8. Laboratory Events of Clinical Interest

A clinical laboratory event of clinical interest (ECI) is a laboratory value of scientific and medical interest specific to understanding of the investigational product and may require additional follow-up testing, close monitoring, and rapid communication by the investigator to the Sponsor.

During NrtI Discontinuation Monitoring Period, any laboratory events of ECI will be monitored, defined as the following:

- ALT > 3x ULN, if Day 1 pre-dose ALT ≤ ULN
- ALT > 2x baseline, if Day 1 pre-dose ALT > ULN

All laboratory ECIs must be reported to the Sponsor within 24 hours of the investigator's knowledge of the event. See Section 10.3.5. In addition, the assessments outlined in Section 10.5 should be performed as soon as possible and no later than two weeks after the initial ALT value meeting criteria for a laboratory ECI.

The laboratory ECI will also be evaluated by the investigator to determine if the event meets criteria for an AE. If AE criteria are met, all clinical sequelae in relation to these laboratory ECIs will be reported as AEs or SAEs, if applicable.

8.3. Adverse Events and Serious Adverse Events

The investigator and any qualified designees are responsible for detecting, documenting, and recording events that meet the definition of an AE or SAE and remain responsible for following up AEs that are serious, considered related to the study drug or study procedures, or that caused the subject to discontinue the study drug or study.

The definitions and procedures for reporting, recording, evaluating, and follow-up of an AE or SAE can be found in Section 10.3, Appendix 3.

8.3.1. Time Period and Frequency for Collecting AE and SAE Information

All SAEs will be collected from the signing of the informed consent form (ICF) until the last follow-up visit at the time points specified in the SoAs (Section 1.5).

All AE related to screening activities will be collected from the signing the ICF until the last follow-up visit at the time points specified in the SoAs (Section 1.5).

Medical occurrences that begin before the start of study drug but after obtaining informed consent will be recorded as medical history/ current medical conditions and not as an AE.

All SAEs will be recorded and reported to the sponsor or designee immediately and under no circumstance should this exceed 24 hours, as indicated in Section 10.3, Appendix 3. The investigator will submit any updated SAE data to the sponsor within 24 hours of it being available.

Investigators are not obligated to actively seek AEs or SAEs in former study subjects. However, if the investigator learns of any SAE, including a death, at any time after a subject has been discharged from the study, and he/she considers the event to be reasonably related to the study drug or study participation, the investigator must promptly notify the sponsor.

8.3.2. Method of Detecting AEs and SAEs

The method of recording, evaluating, and assessing causality of AEs and SAEs and the procedures for completing and transmitting SAE reports are provided in Section 10.3.

Care will be taken not to introduce bias when detecting AEs and/or SAEs. Open-ended and non-leading verbal questioning of the subject is the preferred method to inquire about AE occurrences.

8.3.3. Follow-up of AEs and SAEs

After the initial AE/SAE report, the investigator is required to proactively follow each subject at subsequent visits/contacts. All SAEs will be followed until resolution, until the condition stabilizes, until the event is otherwise explained, or until the subject is lost to follow-up (as defined in Section 7.2). Further information on follow-up procedures is provided in Section 10.3, Appendix 3.

8.3.4. Regulatory Reporting Requirements for SAEs

- Prompt notification by the investigator to the sponsor of a SAE is essential so that legal obligations and ethical responsibilities towards the safety of subjects and the safety of a study drug under clinical investigation are met.
- The sponsor has a legal responsibility to notify both the local regulatory authority and other regulatory agencies about the safety of a study drug under clinical investigation. The sponsor will comply with country-specific regulatory requirements relating to safety reporting to the regulatory authority, Institutional Review Boards (IRB)/Independent Ethics Committees (IEC), and investigators.
- For all studies except those utilizing medical devices investigator safety reports must be prepared for suspected unexpected serious adverse reactions (SUSAR) according to local regulatory requirements and sponsor policy and forwarded to investigators as necessary.
- An investigator who receives an investigator safety report describing a SAE or other specific safety information (e.g., summary or listing of SAEs) from the sponsor will review and then file it along with the Investigator's Brochure and will notify the IRB/IEC, if appropriate according to local requirements.

8.3.5. Special Situations

Special situation reports include all reports of medication error, abuse, misuse, overdose, reports of adverse events associated with product complaints, and pregnancy reports

(Including pregnancies in female patients and pregnancies in female partners of male patients) regardless of an associated AE.

Medication error is any unintentional error in the prescribing, dispensing, or administration of a medicinal product while in the control of the health care provider, subject, or consumer.

Abuse is defined as persistent or sporadic intentional excessive use of a medicinal product by a subject.

Misuse is defined as any intentional and inappropriate use of a medicinal product that is not in accordance with the protocol instructions or the local prescribing information.

An overdose is defined as an accidental or intentional administration of a quantity of a medicinal product given per administration or cumulatively which is above the maximum recommended dose as per protocol. In cases of a discrepancy in drug accountability, overdose will be established only when it is clear that the subject has taken the excess dose(s). Overdose cannot be established when the pharmacist cannot account for the discrepancy except in cases in which the investigator has reason to suspect that the additional dose(s) were administered to the subject.

Product complaint is defined as complaints arising from potential deviations in the manufacture, packaging, or distribution of the medicinal product.

8.3.5.1. Pregnancy

- Details of all pregnancies in female subjects will be collected after the start of study drug through 12 weeks after last study drug administration.
- If a pregnancy is reported, the investigator should inform the sponsor within 24 hours of learning of the pregnancy and should follow the procedures outlined in Section 10.4.
- Abnormal pregnancy outcomes (e.g., spontaneous abortion, fetal death, stillbirth, congenital anomalies, ectopic pregnancy) are considered SAEs.

8.3.5.2. Other Special Situations

- All other special situations must be reported to the sponsor within 24 hours of the investigator becoming aware of the situation. These reports must consist of situations that involve study drug and/or protocol-required concomitant medications, but do not apply to concomitant medications not required in the protocol.
- Special situations involving non-required concomitant medications do not need to be reported; however, for special situations that result in AEs due to a non-required concomitant medication, the AE should be reported.
- Any inappropriate use of concomitant medications prohibited by this protocol should not be reported as “misuse,” but may be more appropriately documented as a protocol deviation.
- All clinical results in relation to these special situation reports will be reported as AEs or SAEs at the same time. Details of the symptoms and signs, clinical management, and outcome will be reported, when available.

8.4. Pharmacodynamics

Pharmacodynamic parameters are not evaluated in this study.

8.5. Genetics

Human genetics are not evaluated in this study.

8.6. [Health Economics] OR [Medical Resource Utilization and Health Economics]

Health Economics/Medical Resource Utilization and Health Economics parameters are not evaluated in this study.

9. STATISTICAL CONSIDERATIONS

9.1. Statistical Hypotheses

9.1.1. Phase 2a Part

The primary endpoint for the Phase 2a part is percentage of subjects with HBsAg loss at completion of PEG-IFN-α treatment. The null hypothesis is that there is no difference in the percentage of HBsAg loss at completion of PEG-IFN-α treatment between BRII-179 + PEG-IFN-α + NrtI and BRII-179 placebo + PEG-IFN- α +NrtI groups.

9.1.2. Phase 2b Part

The primary endpoint for the Phase 2b part is sustained HBsAg and HBV DNA loss (defined as HBsAg and HBV DNA undetectable for at least 24 weeks) after NrtI discontinuation. The null hypothesis is that there is no difference in the percentage of sustained HBsAg and HBV DNA loss between BRII-179+PEG-IFN- α + NrtI and BRII-179 placebo + PEG-IFN- α + NrtI groups.

9.2. Sample Size Determination

9.2.1. Phase 2a Part

The early futility evaluation based on an intermediate outcome of efficacy using the HBsAg loss at completion of PEG-IFN-α treatment will be conducted when approximately 120 subjects complete 18 weeks BRII-179 or placebo investigational treatment and up to 24 weeks PEG-IFN- α treatment or withdraw the study prior to last scheduled PEG-IFN-α treatment.

Everest project data suggest 25% subjects can achieve HBsAg loss after 24 weeks treatment of PEG-IFN- α + NrtI. The 20% difference in percentage of HBsAg loss is considered to be a clinically meaningful increase in HBsAg clearance with the addition of BRII-179 to PEG-IFN-α and NrtI therapy, based on this point, with the assumption of a 25% of HBsAg loss by placebo in combination with PEG-IFN-α and NrtI treatment, and a 45% of HBsAg loss by BRII-179 in combination with PEG-IFN-α and NrtI treatment, a sample size of 120 evaluable subjects in an one-sided Type I error of $\alpha = 0.3$ can provide a power of more than 95% to reject the null hypothesis. In this sample size estimation, power calculation method is normal approximation and test method is Z-test (SAS9.4).

9.2.2. Phase 2b Part

Currently, there are limited data available regarding sustained HBsAg loss and sustained HBV DNA undetectable (defined as HBsAg and HBV DNA undetectable for at least 24 weeks) after a total of 48 weeks treatment of PEG-IFN- α in planned target population in this study. Table 1 displays a range of sample size for sustained HBsAg and HBV DNA loss with PEG-IFN-α + NrtI treatment ranging from 22% to 35% and a minimum of clinically meaningful increase of 15% after adding BRII-179 to PEG-IFN- α + NrtI treatment.

Table 1. Sample Size Calculation for Four Scenarios of Sustained HBsAg Loss

Expected Sustained HBsAg loss and HBV DNA undetectable (%)		Total Sample Size (Per Arm)	Power	Alpha (1-sided)
Placebo + PEG-IFN- α + NrtI	BRII-179 + PEG-IFN-α + NrtI			
22	37	412 (206)	90%	0.025
26	41	442 (221)		
**30**	**45**	**466 (233)**		
35	50	486 (243)		

Based on the following assumptions, a 15% increase in the percentage of sustained HBsAg loss and HBV DNA undetectable at a one-sided Type I error level of 2.5%. It is assumed 466 subjects enrolled will provide 90% power to reject the null hypothesis, further decided to enrol a total of 480 subjects, which will provide with approximately 91% power to reject the null hypothesis.

Proportion of sustained HBsAg and HBV DNA loss in PEG-IFN- α + NrtI group is 30%.

A clinically meaningful increase in sustained HBsAg loss is 15% with add-on of BRII-179 treatment.

Non-binding stopping guideline for futility using a moderately aggressive Type II error spending function (Gamma=-2), implemented using the Lan-DeMets O'Brien-Fleming approximation spending function approach.

The sample size calculation will be performed using proc seqdesign in SAS9.4 and it is planned to perform 3 interim analysis and 1 final analysis.

Data analyses will be performed when 120 subjects enrolled in Phase 2a part complete the last scheduled PEG-IFN-α treatment or withdraw from the study prior to last scheduled PEG-IFN-α treatment.

The sample size for Phase 2b part may be re-estimated after evaluation of efficacy data from subjects in Phase 2a part.

9.3. Data Monitoring Guidelines for Independent DSMB

An independent DSMB will review the safety and efficacy data of Phase 2a part of the study. Based on the results of the Phase 2a part, the DSMB will build the rationale for the recommendation on whether to proceed to Phase 2b part or not. DSMB will perform data review when approximately 120 subjects enrolled in the Phase 2a part complete the last scheduled PEG-IFN-α treatment and HBsAg and HBV DNA results are available or withdraw from the study prior to last scheduled PEG-IFN-α treatmen.

If the trial proceeds to Phase 2b part, the DSMB will continue to review interim data on a regular basis and use pre-specified guidelines to determine clear evidence of efficacy for the primary outcome, and if so the DSMB will provide recommendation to unblind the trial results. Conversely, the DSMB may provide recommendation to discontinue the BRII-179 treatment if futility assessments indicate that there is low probability to achieve statistical significance for the primary endpoint of sustained HBsAg loss with BRII-179 treatment.

9.4. Primary Endpoint and Safety Data Analysis in Phase 2 Part

An early futility analysis based on HBsAg loss will be performed on approximately 120 subjects who complete last scheduled PEG-IFN-α treatment or withdraw from the trial prior to last scheduled PEG-IFN-α treatment. The sample size of 120 subjects provides above 95% power to reject the null hypothesis at the 0.30 (1-sided) level of significance.

Brii will make the decision on whether to proceed to Phase 2b part or not based on HBsAg loss futility analysis result at completion of last scheduled PEG-IFN-α treatment and safety data analysis, as well as the recommendation provided by the independent DSMB.

9.4.1. Monitoring Guidelines for the Primary Endpoint in Phase 2b Part

As a guideline, stopping boundaries will be provided to the DSMB to monitor the primary endpoint (sustained HBsAg loss and sustained HBV DNA undetectable). For monitoring overwhelming benefit, the Lan-DeMets spending function analogue of the O'Brien-Fleming boundaries will be used. The Lan-DeMets boundary used will be chosen to preserve a 1-sided 0.025 level of significance. For computing the Lan-DeMets boundary, the information fraction at each of 3 interim analyses will be the number of subjects enrolled at the interim analysis, i.e., 120, 240, and 360 subjects who complete 48 weeks of follow-up or withdraw before week 48 (for those who do not enter NrtI withdrawal follow-up period) or complete at least 24 weeks of follow-up or withdraw from the study prior to at least 24 weeks of follow-up (for those who enter NrtI discontinuation follow-up period), respectively.

Futility analyses will be performed. The aim of these analyses will be to consider whether BRII-179 should be discontinued due to a low probability of achieving statistical significance for the primary endpoint of sustained HBsAg loss. Conditional power calculations of sustained HBsAg loss will be presented. Typical futility guidelines recommend stopping a trial when conditional power is below 10%-15%These analyses will be presented to the DSMB by the unblinded statisticians.

9.5. Analysis Sets

The following analysis sets are defined:

Population	Description
All Subjects Screened	All subjects, for whom a record exists on the clinical database, will be included in All Subjects Screened.<br>It will be used for reporting study disposition, inclusion and exclusion criteria deviations, etc.
All Subjects Randomized	All subjects, who are randomized will be included in All Subjects Randomized. It will be used for reporting demographics, baseline characteristics, medical history, etc.
Full Analysis Set	All subjects, who are randomized and received at least one dose of BRII-179 or placebo, will be included in Full Analysis. It will be used as the primary analysis set of all efficacy endpoints.
Per Protocol Set	All subjects, who are randomized; and not identified with major protocol deviation that substantially affects the treatment effect of study medication; received all planned doses of BRII-179 or placebo; will be included in Per Protocol Set. It will be used as analysis set for key efficacy endpoint analyses. The analysis based on PP analysis set will be considered as sensitivity analysis.
Safety Analysis Set	All subjects randomly assigned to study drug and who take at least 1 dose of study drug will be included in Safety Analysis Set. It will be used for reporting safety parameters.

9.6. Statistical Analyses

This section outlines the general analytic strategy for the study. The statistical analysis plan (SAP) will be finalized prior to Database Lock (DBL) and it will include a more detailed description of the statistical analyses described in this section. This section is a summary of the planned statistical analyses of the most important endpoints including primary and key secondary endpoints.

9.6.1. General considerations

Unless otherwise stated, the following rule will apply:

Summaries and displays will be presented by treatment group.

For categorical variables, the frequency and percentage will be applied as descriptive statistics.

For continuous variables, the mean, standard variation, median, inter-quartiles, and ranges will be applied as descriptive statistics.

Specific algorithm regarding how missing HBsAg and HBV DNA values will be handled in deriving the primary efficacy endpoints and sustained HBsAg loss will be detailed in the statistical analysis plan.

Unless specified otherwise, missing values will be handled using the Data-As-Observed (DAO) approach, that is, any missing value will be excluded from the analysis, except for the baseline value, which will be replaced with the latest pre-treatment result, if available.

Only scheduled visits will be presented of summaries; unscheduled visits will be listed but will not be summarized.

9.6.2. Analysis of the Primary Efficacy Endpoint for Phase 2a Part

The primary efficacy endpoint of the phase 2a part, percentage of HBsAg loss at completion of PEG-IFN-α treatment, will be analysed based on Cochran-Mantel-Haenszel method, stratified at randomization by screening HBsAg level (3 categories). The differences in the percentage of HBsAg loss and associated 95% confidence interval will be presented. If too few cases in a stratum result in failure of stratified analysis, it will be considered to remove the stratification factors and a non-stratified analysis or Fisher's exact probability method will be used to compare the percentage of HBsAg loss between the two groups.

9.6.3. Analysis of the Primary Efficacy Endpoint for Phase 2b Part

The primary efficacy endpoint of the phase 2b part, percentage of sustained HBsAg loss and sustained HBV DNA undetectable defined as undetectable HBsAg and HBV DNA for at least 24 weeks after NrtI discontinuation. Subjects who do not meet NrtI withdrawal criteria or are retreated with NrtI after NrtI discontinuation will be classified as not achieving sustained HBsAg loss and HBV DNA undetectable. The primary endpoint will be analyzed based on Cochran-Mantel-Haenszel method, stratified at randomization by screening HBsAg level (3 categories: ≥0.05 to < 1 IU/mL, ≥1 to < 10 IU/mL, and ≥10 to <100 IU/mL). The differences in the percentage of sustained HBsAg loss and sustained HBV DNA undetectable together with associated adjusted 95% confidence interval will be presented.

9.6.4. Analyses of Secondary Efficacy Endpoints and Subgroups

Percentage of subjects meeting NrtI discontinuation criteria and other secondary endpoints assessed as proportion will be analysed in the same way as for the primary endpoint.

Time to event endpoints will be compared using stratified log-rank test and continuous endpoints will be compared using Wilcoxon ranks sum test.

Additionally, subgroup analysis by age, gender, and HBsAg level at baseline (3 categories: ≥0.05 to < 1 IU/mL, ≥1 to < 10 IU/mL, and ≥10 to <100 IU/mL) will be conducted with respect to the primary endpoint.

9.6.5. Tertiary Efficacy Endpoint and Subgroup Analyses of Phase 2a Part

Wilcoxon rank-sum test will be used to compare the difference between treatment groups in the change from baseline in selected cytokine/chemokine biomarkers during treatment and discontinuation monitoring period, and Spearman rank correlation test will be used to test the correlation between these changes and sustained HBsAg loss and HBV DNA undetectable.

9.6.6. Analysis of Safety Outcome

The Safety Analysis population will be used for clinical review of safety relevant parameters.

A treatment-emergent adverse event (TEAE) is any AE that has an onset date that is on or after the day of study drug administration or any AE that has been present before treatment and worsens relative to the pre-treatment state after administration of study drug.

Descriptive statistics will be used for the safety analysis, and all adverse events (AEs) will be summarized and described according to the latest version of the Medical Dictionary for Regulatory Activities (MedDRA) codes. Coded treatment-emergent adverse events, investigational product-related adverse events, serious adverse events, and adverse events leading to subject withdrawal will be summarized and described by System Organ Class (SOC), Preferred Term (PT), and group, and number and percentage of subject with AE will be summarized. Treatment-emergent adverse events, investigational product-related adverse events, serious adverse events, and adverse events leading to subject withdrawal will also be summarized by severity. All adverse events will be tabulated. Graded laboratory abnormalities will be defined using CTCAE Version 5.0 grading scale. Laboratory tests, vital signs, physical examinations, and 12-lead electrocardiograms will be summarized descriptively. For vital signs, laboratory tests (quantitative measures), and 12-lead electrocardiograms (quantitative measures), observed values and their changes from baseline will be summarized descriptively. Subjects with post-treatment abnormalities of clinical significance will be listed for all tests.

10. SUPPORTING DOCUMENTATION AND OPERATIONAL CONSIDERATIONS

10.1. Appendix 1: Regulatory, Ethical, and Study Oversight Considerations

10.1.1. Regulatory and Ethical Considerations

- This study will be conducted in accordance with the protocol and with the following:
  - Consensus ethical principles derived from international guidelines including the Declaration of Helsinki and Council for International Organizations of Medical Sciences (CIOMS) International Ethical Guidelines
  - Applicable ICH Good Clinical Practice (GCP) Guidelines
  - Applicable laws and regulations
- The protocol, protocol amendments, ICF, Investigator Brochure, and other relevant documents (e.g., advertisements) must be submitted to an IRB/IEC by the investigator and reviewed and approved by the IRB/IEC before the study is initiated.
- Any amendments to the protocol will require IRB/IEC approval before implementation of changes made to the study design, except for changes necessary to eliminate an immediate hazard to study subjects.
- The investigator will be responsible for the following:
  - Providing written summaries of the status of the study to the IRB/IEC annually or more frequently in accordance with the requirements, policies, and procedures established by the IRB/IEC
  - Notifying the IRB/IEC of SAEs or other significant safety findings as required by IRB/IEC procedures
  - Providing oversight of the conduct of the study at the site and adherence to requirements of 21 CFR, ICH guidelines, the IRB/IEC, European regulation 536/2014 for clinical studies (if applicable), and all other applicable local regulations

10.1.2. Financial Disclosure

Investigators and sub-investigators will provide the sponsor with sufficient, accurate financial information as requested to allow the sponsor to submit complete and accurate financial certification or disclosure statements to the appropriate regulatory authorities. Investigators are responsible for providing information on financial interests during the course of the study and for 1 year after completion of the study.

10.1.3. Informed Consent Process

- The investigator or his/her representative will explain the nature of the study to the subject or his/her legally authorized representative and answer all questions regarding the study.

- Subjects must be informed that their participation is voluntary. Subjects or their legally authorized representative will be required to sign a statement of informed consent that meets the requirements of 21 CFR 50, local regulations, ICH guidelines, Health Insurance Portability and Accountability Act (HIPAA) requirements, where applicable, and the IRB/IEC or study center.
- The medical record must include a statement that written informed consent was obtained before the subject was enrolled in the study and the date the written consent was obtained. The authorized person obtaining the informed consent must also sign the ICF.
- Subjects must be re-consented to the most current version of the ICF(s) during their participation in the study.
- A copy of the ICF(s) must be provided to the subject or the subject's legally authorized representative.

10.1.4. Data Protection

- Subjects will be assigned a unique identifier by the sponsor. Any subject records or datasets that are transferred to the sponsor will contain the identifier only; subject names or any information which would make the subject identifiable will not be transferred.
- The subject must be informed that his/her personal study-related data will be used by the sponsor in accordance with local data protection law. The level of disclosure must also be explained to the subject who will be required to give consent for their data to be used as described in the informed consent
- The subject must be informed that his/her medical records may be examined by Clinical Quality Assurance auditors or other authorized personnel appointed by the sponsor, by appropriate IRB/IEC members, and by inspectors from regulatory authorities.

10.1.5. Data Quality Assurance

- All relevant subject data relating to the study will be recorded on printed or electronic CRF unless transmitted to the sponsor or designee electronically (e.g., laboratory data). The investigator is responsible for verifying that data entries are accurate and correct by physically or electronically signing the CRF.
- The investigator must maintain accurate documentation (source data) that supports the information entered in the CRF.
- The investigator must permit study-related monitoring, audits, IRB/IEC review, and regulatory agency inspections and provide direct access to source data documents.
- Monitoring details describing strategy (e.g., risk-based initiatives in operations and quality such as Risk Management and Mitigation Strategies and Analytical Risk-

Based Monitoring), methods, responsibilities and requirements, including handling of noncompliance issues and monitoring techniques (central, remote, or on-site monitoring) are provided in the Monitoring Plan.

- The sponsor or designee is responsible for the data management of this study including quality checking of the data.
- The sponsor assumes accountability for actions delegated to other individuals (e.g., Contract Research Organizations).
- Study monitors will perform ongoing source data verification to confirm that data entered into the CRF by authorized site personnel are accurate, complete, and verifiable from source documents; that the safety and rights of subjects are being protected; and that the study is being conducted in accordance with the currently approved protocol and any other study agreements, ICH GCP, and all applicable regulatory requirements.
- Records and documents, including signed ICFs, pertaining to the conduct of this study must be retained by the investigator. No records may be destroyed during the retention period without the written approval of the sponsor. No records may be transferred to another location or party without written notification to the sponsor.

10.1.6. Source Documents

- Source documents provide evidence for the existence of the subject and substantiate the integrity of the data collected. Source documents are filed at the investigator's site.
- Data reported on the CRF or entered in the eCRF that are transcribed from source documents must be consistent with the source documents or the discrepancies must be explained. The investigator may need to request previous medical records or transfer records, depending on the study. Also, current medical records must be available.
- Source data refer to all information recorded in the original record of certified copy of the clinical study, including clinical findings, observation results and other relevant activity records needed for reconstruction and evaluation of the clinical study.

10.1.7. Study and Site Start and Closure

The study start date is the date on which the clinical study will be open for recruitment of subjects.

The sponsor designee reserves the right to close the study site or terminate the study at any time for any reason at the sole discretion of the sponsor. Study sites will be closed upon study completion. A study site is considered closed when all required documents and study supplies have been collected and a study-site closure visit has been performed.

The investigator may initiate study-site closure at any time, provided there is reasonable cause and sufficient notice is given in advance of the intended termination.

Reasons for the early closure of a study site by the sponsor or investigator may include but are not limited to:

- Failure of the investigator to comply with the protocol, the requirements of the IRB/IEC or local health authorities, the sponsor's procedures, or GCP guidelines
- Inadequate recruitment of subjects by the investigator
- Discontinuation of further study drug development

If the study is prematurely terminated or suspended, the sponsor shall promptly inform the Investigators, the IECs/IRBs, the regulatory authorities, and any contract research organization(s) used in the study of the reason for termination or suspension, as specified by the applicable regulatory requirements. The Investigator shall promptly inform the subject and should assure appropriate subject therapy and/or follow-up.

10.1.8. Publication Policy

The sponsor intends to publish the results of clinical trials regardless of outcome. Results may be published in peer-reviewed journals and/or presented at scientific congresses. The sponsor will comply with the requirements for publication and disclosure of clinical trial information. In accordance with standard editorial and ethical practice, the sponsor will generally support publication of multicenter studies only in their entirety and not as individual site data. Authorship will be determined by mutual agreement based upon International Committee of Medical Journal Editors criteria and guidelines [ICMJE, 2018]. Individuals who do not meet authorship criteria do not warrant named authorship. Nonauthor contributors may be acknowledged in the manuscript when published or recognized in a manner consistent with the target journal policy.

10.2. Appendix 2: Clinical Laboratory Tests

The tests detailed in Table 2 will be performed by the central laboratory. Urine pregnancy test will be performed by the local or central laboratory.

Investigators must document their review of each laboratory safety report.

Additional tests may be performed at any time during the study as determined necessary by the investigator or required by local regulations.

Table 2: Protocol-Required Safety Laboratory Assessments

**Hematology**	
Complete blood count with differential, including red blood cell count, hemoglobin, hematocrit, white blood cell count, count and percentage of neutrophils, lymphocytes, eosinophils, basophils, and monocytes, and platelet count	
**Chemistry**	
Albumin	Estimated glomerular filtration rate (eGFR)
Blood urea nitrogen (BUN)	Gamma glutamyl transferase (GGT)
Calcium	Glucose
Carbon dioxide/bicarbonate	Lactate dehydrogenase (LDH)
Chloride	Potassium
Creatine kinase	Sodium
Creatinine	Uric acid
**Liver Function Tests**	
Alkaline phosphatase (ALP)	Aspartate aminotransferase (AST)
Alanine aminotransferase (ALT)	Bilirubin (total and direct)
**Coagulation Parameters**	
International normalized ratio (INR)	Prothrombin time (PT)
**Urinalysis**	
Glucose	Proteins
Red blood cells (RBCs)	
**Pregnancy Testing**	
Beta-human chorionic gonadotropin (WOCBP only)	Urine pregnancy test
**Thyroid Panel**	
Thyroid stimulating hormone (TSH)[1]	

[1] Thyroid function will be tested every 12 weeks during PEG-IFN-α dosing: Screening, Week 12, and Week20- 24.

10.3. Appendix 3: Adverse Events: Definitions and Procedures for Recording, Evaluating, Follow-up, and Reporting

10.3.1. Definition of AE

**AE Definition**
• An AE is any untoward medical occurrence in a patient or clinical study subject, temporally associated with the use of study drug, whether or not considered related to the study drug.<br>• NOTE: An AE can therefore be any unfavorable and unintended sign (including an abnormal laboratory finding), symptom, or disease (new or exacerbated) temporally associated with the use of study drug.

**Events Meeting the AE Definition**
• Any abnormal laboratory test results (hematology, clinical chemistry, or urinalysis) or other safety assessments (e.g., ECG, radiological scans, vital signs measurements), including those that worsen from baseline, considered clinically significant in the medical and scientific judgment of the investigator (i.e., not related to progression of underlying disease).<br>• Exacerbation of a chronic or intermittent pre-existing condition including either an increase in frequency and/or intensity of the condition.<br>• New conditions detected or diagnosed after study drug administration even though it may have been present before the start of the study.<br>• Signs, symptoms, or the clinical sequelae of a suspected drug-drug interaction.<br>• Signs, symptoms, or the clinical sequelae of a suspected overdose of either study drug or a concomitant medication. Overdose per se will not be reported as an AE/SAE unless it is an intentional overdose taken with possible suicidal/self-harming intent. Such overdoses should be reported regardless of sequelae.

**Events NOT Meeting the AE Definition**
• Any clinically significant abnormal laboratory findings or other abnormal safety assessments which are associated with the underlying disease, unless judged by the investigator to be more severe than expected for the subject's condition.<br>• The disease/disorder being studied or expected progression, signs, or symptoms of the disease/disorder being studied, unless more severe than expected for the subject's condition.<br>• Medical or surgical procedure (e.g., endoscopy, appendectomy): the condition that leads to the procedure is the AE.<br>• Situations in which an untoward medical occurrence did not occur (social and/or convenience admission to a hospital).<br>• Anticipated day-to-day fluctuations of pre-existing disease(s) or condition(s) present or detected at the start of the study that do not worsen.

10.3.2. Definition of SAE

If an event is not an AE per definition above, then it cannot be an SAE even if serious conditions are met (e.g., hospitalization for signs/symptoms of the disease under study, death due to progression of disease).

**A SAE is defined as any untoward medical occurrence that, at any dose:**
**a. Results in death**
**b. Is life-threatening**<br>The term 'life-threatening' in the definition of 'serious' refers to an event in which the subject was at risk of death at the time of the event. It does not refer to an event, which hypothetically might have caused death, if it were more severe.
**c. Requires inpatient hospitalization or prolongation of existing hospitalization**<br>• In general, hospitalization signifies that the subject has been detained (usually involving at least an overnight stay) at the hospital or emergency ward for observation and/or treatment that would not have been appropriate in the physician's office or outpatient setting. Complications that occur during hospitalization are AEs. If a complication prolongs hospitalization or fulfills any other serious criteria, the event is serious. When in doubt as to whether "hospitalization" occurred or was necessary, the AE should be considered serious.<br>• Hospitalization for elective treatment of a pre-existing condition that did not worsen from baseline is not considered an AE.
**d. Results in persistent disability/incapacity**<br>• The term disability means a substantial disruption of a person's ability to conduct normal life functions.<br>• This definition is not intended to include experiences of relatively minor medical significance such as uncomplicated headache, nausea, vomiting, diarrhea, influenza, and accidental trauma (e.g., sprained ankle) which may interfere with or prevent everyday life functions but do not constitute a substantial disruption.
**e. Is a congenital anomaly/birth defect**
**f. Other important medical events:**<br>• Medical and scientific judgment should be exercised to determine whether expedited reporting is appropriate in other situations; in general, any important medical event that may not be immediately life-threatening, or result in death, or hospitalization, but for which medical interventions are required to prevent any of the above from happening, is also considered a serious adverse event. For example, intensive treatment in the emergency room or at home for allergic bronchospasm, blood dyscrasias or convulsions that do not result in hospitalisation, or development of drug dependency or drug abuse, etc.

10.3.3. Recording and Follow-Up of AE and/or SAE

AE and SAE Recording
• When an AE/SAE occurs, it is the responsibility of the investigator to review all documentation (e.g., hospital progress notes, laboratory reports, and diagnostics reports) related to the event.<br>• The investigator will then record all relevant AE/SAE information in the CRF.<br>• It is **not** acceptable for the investigator to send photocopies of the subject's medical records to the Sponsor in lieu of completion of the AE/SAE CRF page.<br>• There may be instances when copies of medical records for certain cases are requested by the Sponsor. In this case, all subject identifiers, with the exception of the subject number, will be redacted on the copies of the medical records before submission to the Sponsor.<br>• The investigator will attempt to establish a diagnosis of the event based on signs, symptoms, and/or other clinical information. Whenever possible, the diagnosis (not the individual signs/symptoms) will be documented as the AE/SAE.
**Assessment of Intensity**
The investigator will make an assessment of intensity for each AE and SAE reported during the study based on the National Cancer Institute Common Terminology Criteria for Adverse Events (NCI-CTCAE) Version 5.0.
**Assessment of Causality**
• The investigator or a qualified assistant investigator is obligated to assess the relationship between study drug and each occurrence of each AE/SAE.<br>• Relationship between AEs and the study drug: "related" should be entered when there is either (a) a *reasonable possibility* that the AE/SAE is associated with study drug **or** (b) no reasonable alternative explanation can be found. Otherwise, the relationship between AEs and the study drug should be classified as "unrelated".<br>• A "reasonable possibility" of a relationship conveys that there are facts, evidence, and/or arguments to suggest a causal relationship, rather than a relationship cannot be ruled out.<br>• The investigator will use clinical judgment to determine the relationship.<br>• Alternative causes, such as underlying disease(s), concomitant therapy, and other risk factors, as well as the temporal relationship of the event to study drug administration will be considered and investigated.<br>• The investigator will also consult the Investigator's Brochure (IB) and/or Product Information, for marketed products, in his/her assessment.<br>• For each AE/SAE, the investigator **must** document in the medical notes that he/she has reviewed the AE/SAE and has provided an assessment of causality.<br>• There may be situations in which an SAE has occurred and the investigator has minimal information to include in the initial report. However, it is very important that the investigator always make an assessment of causality for every event before the initial transmission of the SAE data.

- The investigator may change his/her opinion of causality in light of follow-up information and send a SAE follow-up report with the updated causality assessment.
- The causality assessment is one of the criteria used when determining regulatory reporting requirements.

Follow-up of AEs and SAEs
• The investigator is obligated to perform or arrange for the conduct of supplemental measurements and/or evaluations as medically indicated or as requested by the Sponsor to elucidate the nature and/or causality of the AE or SAE as fully as possible. This may include additional laboratory tests or investigations, histopathological examinations, or consultation with other health care professionals.<br>• If a subject dies during participation in the study or during a recognized follow-up period, the investigator will provide the Sponsor with a copy of any post-mortem findings including histopathology.<br>• New or updated information will be recorded in the originally completed CRF.<br>• The investigator will submit any updated SAE data to the Sponsor within 24 hours of receipt of the information.

10.3.4. Reporting of SAEs

SAE Reporting to the Sponsor via a Paper SAE Reporting Form
• The investigator must complete a paper SAE report form and send to the Sponsor or its representative (drugsafety-briibio@dmedglobal.com) within 24 hours of the investigator's knowledge of the event.<br>• Contacts for SAE reporting can be found on the SAE Reporting Form.

10.3.5. Reporting of Laboratory Events of Clinical Interest

All laboratory ECIs must be reported to the Sponsor or its representative within 24 hours of the investigator's knowledge of the event. Contact for laboratory ECIs reporting can be found on the ECI Reporting Form.

The laboratory ECI will also be evaluated by the investigator to determine if the event meets criteria for an AE. If AE criteria are met, all clinical sequelae in relation to these laboratory ECIs will be reported as AEs or SAEs, if applicable.

10.3.6. Special Situation Report

For special situations and AEs due to special situations, the investigator should complete a Special Situation Report Form and send it to the Sponsor or its representative (drugsafety-briibio@dmedglobal.com) within 24 hours of becoming aware.

10.4. Appendix 4: Contraceptive Requirements and Collection and Reporting of Pregnancy Information

Contraceptive Requirements:

WOCBP may be included in this study and include any female subject who has experienced menarche and who is not post-menopausal or permanently sterilized (eg, bilateral tubal occlusion, hysterectomy, or bilateral salpingectomy).

WOCBP must be willing to use highly effective methods of contraception 14 days before dose, throughout study participation, and for 12 weeks after last dose of study drug administration. Highly effective methods of birth control result in a low failure rate (ie, less than 1% per year). Birth control methods which are considered highly effective include:

- Established use of combined (estrogen and progestogen containing) oral, intravaginal, or transdermal hormonal methods of contraception associated with inhibition of ovulation **OR** established use of progestogen-only oral, injectable, or implantable hormonal methods of contraception associated with inhibition of ovulation. It is not currently known whether study drug will impact the effectiveness of hormonal contraceptive methods; therefore, it is recommended to use an additional form of contraception (ie, barrier method) throughout the study and for 12 weeks after last study drug administration.
- Placement of an intrauterine device
- Placement of an intrauterine hormone-releasing system
- Surgical sterilization of male partner (with the appropriate post-vasectomy documentation of the absence of sperm in the ejaculate; for female subjects on the study, the vasectomized male partner should be the sole partner for that subject)
- True sexual abstinence, when in line with the preferred and usual lifestyle of the subject. Periodic abstinence (e.g., calendar, ovulation, symptothermal, post-ovulation methods) and withdrawal are not acceptable methods of contraception. Abstinent subjects have to agree to use 1 of the above-mentioned contraceptive methods, if they start sexual relationships during the study and for up to 12 weeks after last dose of study drug administration, or for as long as the subject is followed on study, whichever is longer.
- Barrier method in combination with hormonal contraceptive, as described above

Post-menopausal status is defined as 12 months with no menses without an alternative medical cause.

Collection of Pregnancy Information:

- The investigator will collect pregnancy information about female subjects who are pregnant or male subject’s female partner who are pregnant during the study. The initial information will be recorded on the appropriate form and submitted to the sponsor within 24 hours of learning of a subject's pregnancy.

- The female subject who are pregnant or male subject’s female partner who are pregnant will be followed to determine the outcome of the pregnancy. The investigator will collect follow-up information on the female subject who are pregnant or male subject’s female partner who are pregnant and the neonate and the information will be forwarded to the sponsor. Generally, follow-up will not be required for longer than 6 to 8 weeks beyond the estimated delivery date. Any termination of pregnancy will be reported, regardless of fetal status (presence or absence of anomalies) or indication for the procedure.
- While pregnancy itself is not considered to be an AE or SAE, any pregnancy complication or elective termination of a pregnancy for medical reasons will be reported as an AE or SAE.
- A spontaneous abortion (occurring at <22 weeks gestational age) or still birth (occurring at >22 weeks gestational age) is always considered to be an SAE and will be reported as such.
- Any post-study pregnancy related SAE considered reasonably related to the study drug by the investigator will be reported to the sponsor as described in Section 8.3.4. While the investigator is not obligated to actively seek this information in former study subjects, he or she may learn of an SAE through spontaneous reporting.
- Any female subject who becomes pregnant while participating in the study will discontinue study drug.

10.5. Appendix 5: Assessments in Subjects Meeting Laboratory ECI Criteria

Additional assessment will be performed in subjects who experience ALT elevations meeting laboratory ECI criteria (Section 8.2.8) during the NrtI Discontinuation Monitoring Period. Following the occurrence of event of ECI, the following assessments in Table 3 will be performed one time, as soon as possible, and no later than two weeks after the initial ALT value meeting criteria for a laboratory ECI. The full panel of assessments should only be performed once; individual assessments may be repeated per investigator discretion, as needed.

Table 3 Assessments in Subjects Meeting Laboratory ECI Criteria

**Hematology**	
Complete blood count with differential, including red blood cell count, hemoglobin, hematocrit, white blood cell count, count and percentage of neutrophils, lymphocytes, eosinophils, basophils, and monocytes, and platelet count	
**Chemistry**	
Albumin	Estimated glomerular filtration rate (eGFR)
Blood urea nitrogen (BUN)	Gamma glutamyl transferase (GGT)
Calcium	Glucose
Carbon dioxide/bicarbonate	Lactate dehydrogenase (LDH)
Chloride	Potassium
Creatine kinase	Sodium
Creatinine	Uric acid
**Liver Function Tests**	
Alkaline phosphatase (ALP)	Aspartate aminotransferase (AST)
Alanine aminotransferase (ALT)	Bilirubin (total and direct)
**Coagulation Parameters**	
International normalized ratio (INR)	Prothrombin time (PT)
**Extended Hepatic Panel**	
Quantitative HBsAg	Ferritin
Quantitative HBV DNA	Blood phosphatidylethanol
Quantitative HBeAg	Urinary ethyglucuronide
HAV antibody IgM	Anti-nuclear antibodies
HCV antibody and HCV RNA	Anti-smooth muscle antibodies
HDV antibody or RNA	Anti-LKM1 antibody
HEV antibody IgM	Anti-mitochondrial antibodies
Epstein-Barr Virus antibodies, IgM and IgG	
Cytomegalovirus antibodies, IgM and IgG	
Ceruloplasmin	
**Imaging**	
Abdominal ultrasound, including Doppler flow, if applicable; CT or MRI acceptable in place of ultrasound if clinically indicated	
**Focused Medical History**	
Use of any potentially hepatotoxic concomitant medications, including over the counter medications and herbal remedies	Alcohol consumption and drugs of abuse

Exposure to other potentially hepatotoxic agents including any work-related exposures (eg. solvents, pesticides, etc.)	Exercise history

CT = computed tomography; HAV = hepatitis A virus; HCV = hepatitis C virus; HDV = hepatitis D virus; HEV = hepatitis E virus; IgG = immunoglobulin G antibody; IgM = immunoglobulin M antibody; LKM1 = liver/kidney microsome-1 antibody; MRI = magnetic resonance imaging; PCR = polymerase chain reaction; RNA = ribonucleic acid; SLA = soluble liver antigen.

11. REFERENCES

Barthel FM-S, Parmar MKB, Royston P. How do multi-stage, multi-arm trials compare to the traditional two-arm parallel group design - a reanalysis of 4 trials. Trials 2009; 10:21, doi:.10.1186/1745-6215-10-21.

Berg T, Simon KG, Mauss S, Schott E, Heyne R, Klass DM, Eisenbach C, Welzel TM, Zachoval R, Felten G, Schulze-Zur-Wiesch J, Cornberg M, Op den Brouw ML, Jump B, Reiser H, Gallo L, Warger T, Petersen J; FINITE CHB study investigators. Long-term response after stopping tenofovir disoproxil fumarate in non-cirrhotic HBeAg-negative patients - FINITE study. *Journal of Hepatology*. 2017 Nov;67(5):918-924.

Chang, M. L., Liaw, Y. F., and Hadziyannis, S. J. (2015). Systematic review: cessation of long-term nucleos(t)ide analogue therapy in patients with hepatitis B e antigen-negative chronic hepatitis B. *Alimentary Pharmacology & Therapeutics*, 2015; *42*(3): 243-257.

Choodari-Oskooei B, Parmar MKB, Royston P, Bowden J. Impact of lack-of-benefit stopping rules on treatment effect estimates of two-arm multi-stage (TAMS) trials with time to event outcome. Trials 2013, 14:23, http://www.trialsjournal.com/content/14/1/23.

Cornberg M, Lok AS, Terrault NA, Zoulim F, and the 2019 EASL-AASLD HBV Treatment Endpoints Conference Faculty. Guidance for design and endpoints of clinical trials in chronic hepatitis B - Report from the 2019 EASL-AASLD HBV Treatment Endpoints Conference. *Journal of Hepatology*. 2020; 72(3): 539-337.

Ferrari C. HBV and the immune response. *Liver Int*. 2015; 35 (Suppl. 1): 121-128.

Hoa PT, Huy NT, Thu le T, Nga CN, Nakao K, Eguchi K, Chi NH, Hoang BH, Hirayama K. Randomized controlled study investigating viral suppression and serological response following pre-S1/pre-S2/S vaccine therapy combined with lamivudine treatment in HBeAg-positive patients with chronic hepatitis B. *Antimicrob Agents Chemother*. 2009; 53:5134-40.

Jeng WJ, Chen YC, Chien RN, Sheen IS, Liaw YF. Incidence and predictors of HBsAg seroclearance after cessation of nucleos(t)ide analogue therapy in HBeAg negative chronic hepatitis B. *Hepatology*. 2018; 68(2): 425-434.

Kondo Y, Ninomiya M, Kakazu E, Kimura O, Shimosegawa T. Hepatitis B surface antigen could contribute to the immunopathogenesis of hepatitis B virus infection. *ISRN Gastroenterol*. 2013;2013:935295.

Konerman MA, Lok AS. Interferon Treatment for Hepatitis B. *Clinics in Liver Disease*. 2016; 20(4): 645-665.

Kramvis A. Genotypes and genetic variability of hepatitis B virus. *Intervirology* 2014; 57(3-4): 141-150.

Lebossé F, Testoni B, Fresquet J, Facchetti F, Galmozzi E, Fournier M, Hervieu V, Berthillon P, Berby F, Bordes I, Durantel D, Levrero M, Lampertico P, Zoulim F. Intrahepatic innate immune response pathways are downregulated in untreated chronic hepatitis B. *J Hepatol*. 2017;66(5):897-909.

Lok AS, McMahon BJ, Brown RS, Wong JB, Ahmed AT, Farah W, Almasri J, Alahdab F, Benkhadra K, Mouchli MA, Singh S, Mohamed EA, Abu Dabrh AM, Prokop LJ, Wang Z, Murad MH, Mohammed K. Antiviral therapy for chronic hepatitis B viral infection in adults: A systematic review and meta-analysis. *Hepatology* 2016; 63(1):284-306.

Marcellin P, Ahn SH, Ma X, Caruntu FA, Tak WY, Elkashab M, et al. Combination of tenofovir disoproxil fumarate and peginterferon α-2a increases loss of hepatitis B surface antigen in patients with chronic hepatitis B. Gastroenterology 2016;150(1):134-44.

Michler T, Kosinska AD, Festag J, Bunse T, Su J, Ringelhan M, Imhof H, Grimm D, Steiger K, Mogler C, Heikenwalder M, Michel ML, Guzman CA, Milstein S, Sepp-Lorenzino L, Knolle P, Protzer U. Knockdown of Virus Antigen Expression Increases Therapeutic Vaccine Efficacy in High-Titer Hepatitis B Virus Carrier Mice. Gastroenterology. 2020 May;158(6):1762-1775.

Papatheodoridi M, Hadziyannis E, Berby F, Zachou K, Testoni B, Rigopoulou E, Gatselis NK, Lyberopoulou A, Vlachogiannakos I, Manolakopoulos S, Dalekos GN, Zoulim F, Papatheodoridis GV. Predictors of hepatitis B surface antigen loss, relapse and retreatment after discontinuation of effective oral antiviral therapy in noncirrhotic HBeAg-negative chronic hepatitis B. *J Viral Hepat.* 2020; 27(2): 118-126.

Perrillo, R. "Benefits and risks of IFN therapy for hepatitis B." *Hepatology*. 2009; 49(5): S103-111.

Ren H, Hu P, Chen XY, Gong GZ, Shang J, Zhang WH, et al. Switching to PegIFNα-2a in NUC treated CHB patients(NEW SWITCH Study)： comparison 48 and 96 weeks. Hepatol Int 2016;10(Suppl 1):S43-4.

Roggendorf H, Krawczyk A, Lindemann M, Shouval D, Michler T, Roggendorf M, Gerken G. Induction of functional control in chronic hepatitis B patients with low level HBsAg using a combination of a PreS1/S2/S HBV vaccine (Sci-B-Vac®) and a nucleoside analogue. *J Infect Dis Ther*. 2019; 7:389-393.

Schweitzer A, Horn J, Mikolajczyk RT, Krause G, Ott JJ. Estimations of worldwide prevalence of chronic hepatitis B virus infection: a systematic review of data published between 1965 and 2013. *Lancet* 2015; 386(10003): 1546-1555.

Stanaway JD, Flaxman AD, Naghavi M, Fitzmaurice C, Vos T, Abubakar I, Abu-Raddad LJ, Assadi R, Bhala N, Cowie B, Forouzanfour MH, Groeger J, Hanafiah KM, Jacobsen KH, James SL, MacLachlan J, Malekzadeh R, Martin NK, Mokdad AA, Mokdad AH, Murray CJL, Plass D, Rana S, Rein DB, Richardus JH, Sanabria J, Saylan M, Shahraz S, So S, Vlassov V, Weiderpass E, Wiersma ST, Younis M, Yu C, Zaki M, Cooke GS. The

global burden of viral hepatitis from 1990 to 2013: findings from the Global Burden of Disease Study 2013. *Lancet* 2016; 388(10049): 1081-1088.

Trepo CHL, Chan, Lok A. Hepatitis B virus infection. *Lancet* 2014;384(9959): 2053-2063.

van Bömmel F, Berg T. Stopping long-term treatment with nucleos(t)ide analogues is a favourable option for selected patients with HBeAg-negative chronic hepatitis B. *Liver International*. 2018;38 Suppl 1:90-96.

Wang S, Chen Z, Hu C, Qian F, Cheng Y, Wu M, Shi B, Chen J, Hu Y, Yuan Z. Hepatitis B virus surface antigen selectively inhibits TLR2 ligand-induced IL-12 production in monocytes/macrophages by interfering with JNK activation. *J Immunol*. 2013 May 15;190(10):5142-51.

Wong TCL, Fung JYY, Chok KSH, Cheung TT, Chan ACY, Dai WC, Ng KKC, Chan SC, Lo, CM. Hepatitis B vaccination in patients receiving oral antiviral therapy without hepatitis B immunoglobulin after liver transplant. *Transplant Proc*. 2018; 50:3681-3688.

Yao CC, Hung CH, Hu TH, Lu SN, Wang JH, Lee CM, and Chen CH. Incidence and predictors of HBV relapse after cessation of nucleoside analogues in HBeAg-negative patients with HBsAg ≤ 200 IU/mL. *Scientific Reports*. 2017; 7(1): 1839.

Ze-Qian Wu, Dong-Ying Xie, Lei Fu, Jia Wei, Jia Shang, Qing He, Wen-hua Zhang, Guang-yu Huang, Yanzhong Peng, Ye Gu, Jia-bin Li, Ying Guo, Yu-juan Guan, Jia-wei Geng, Huan-wei Zheng, Wen-jun Gao, Wen-jing Zhao, Ying-jun Tian, Yi-lan Zeng, Ren Chen, Shengwang Gu, Zhi-liang Gao. Functional cure based on pegylated interferon ? in long-term nucleoside analog suppressed HBeAg negative chronic hepatitis B: a multicenter real-world study (Everest project in China), an interim report. *Journal of Hepatology* 2020; 73: S653-S915.

Zhu D, Liu L, Yang D, Fu S, Bian Y, Sun Z, He J, Su L, Zhang L, Peng H, Fu YX. Clearing Persistent Extracellular Antigen of Hepatitis B Virus: An Immunomodulatory Strategy To Reverse Tolerance for an Effective Therapeutic Vaccination. *J Immunol*. 2016; 196(7): 3079-87.

Title

Protocol Title: A Multicenter, Randomized, Double-Blind, Placebo-Controlled, Parallel-Group Study to Investigate the Efficacy and Safety of BRII-179 (VBI-2601) for the Treatment of Chronic Hepatitis B Virus (HBV) Infection

Protocol Number: BRII-179-002

Compound: BRII-179 (VBI-2601)

Study Phase: Phase 2

Short Title: Short Title: Study to Investigate Efficacy and Safety of BRII-179 (VBI-2601) for the Treatment of Chronic HBV Infection

Sponsor: Brii Biosciences Limited

Co-Sponsor/ Registration Agent: Brii Biosciences (Beijing) Ltd.

Legal Registered Address: Room 301, 3/F, Building 7,
No. 1 Yongtaizhuang North Road,
Tiandilinfeng (Dongsheng Area)
Haidian District, Beijing 100192,
China

Protocol Version: Version 4.0

Approval Date: 28 February 2022

Sponsor Signatory

[redacted]

Medical Monitor Name and Contact Information will be provided separately.

INVESTIGATOR AGREEMENT PAGE

For protocol number BRII-179-002

I confirm agreement to conduct the study in compliance with the protocol, *International Conference on Harmonisation Good Clinical Practices (ICH GCP)* as well as applicable local regulatory and ethical requirements.

I acknowledge that I am responsible for overall study conduct. I agree to personally conduct or supervise the described clinical study.

I agree to ensure that all associates, colleagues and employees assisting in the conduct of the study are informed about their obligations. Mechanisms are in place to ensure that site staff receive the appropriate information throughout study.

Investigator Name: ____

____ ___________________________

Investigator Signature **Date** (dd/mm/yyyy)

Protocol Revision History

The protocol version No., effective date and major revisions are summarized as follows:

Protocol version No.	Protocol effective date
1.0	30-APR-2021
2.0	16-JUN-2021
3.0	3-AUG-2021
4.0	28-FEB-2022

Protocol Modifications Clarification

This amendment was made based on re-discussion with the investigator.

Detailed revisions are provided in the table below.

Original Section and Title	Description of Protocol Changes	Rationale
Section 1.3 Phase 2b, Section 1.5 SoA, Section 3.2.2 Secondary Endpoints, Section 4.1 Overall Design, Section 8.1.3 Dosing Period, Section 9.2 Sample Size Determination and Section 10.2 Appendix 2: Clinical Laboratory Tests	Last visit in Dosing Period is revised from "W20-24/ET1" to "W24/ET1", "Study Visit Day ± Visit Window" of this visit is revised from "D141-169 ± 3" to "D169 ± 3". Accordingly, duration of dosing period is revised from "up to 24 weeks" to "24 weeks", "End of Treatment" is revised from "up to Week 24" to "Week 24", duration of follow-up period is revised from "up to 28 weeks" to "24 weeks", treatment duration of PEG-IFN-α after enrollment is revised from "up to 24 weeks" to "24 weeks" ("a total of 48 weeks of PEG-IFN-α treatment before and after enrollment" is removed)	The time interval between the last dose of BRII-179 / placebo and the primary endpoint analysis (i.e., "completion of PEG-IFN-α treatment") was uniformized for all subjects.
Rationale and Overall Design in Section 1.1 Synopsis, Section 2.2 Study Rationale, Section 4.1 Study design, Section 4.2 Scientific Rationale for Study Design, Section 5.1 Inclusion Criteria, and Section 5.2 Exclusion Criteria	The restriction that subjects will have to be participants of the Everest Project is removed from the eligibility criteria.	To improve subject recruitment by expanding the scope of screening for eligible subjects.
Section 5.1 Inclusion Criteria and Section 4.1 Study Design	Inclusion Criteria 7 is revised from "Must be receiving first-line NrtI (ETV or TDF or TAF) therapy for ≥ 18 months and PEG-IFN-α for 24 to 28 weeks of the Day 1 visit and could provide the related treatment history" to " Must be receiving first-line NrtI (ETV or TDF or TAF) therapy for ≥ 12	The previous requirement of "24-28 weeks" were clarified in consideration of the real clinical practice that some patients are not receiving PEG-IFN-α strictly once a week due to objective reasons. In addition, since subjects are no longer required to be from the

	months (with evidence of HBV DNA<LLOQ for ≥ 6 months, local lab results accepted) and PEG-IFN-α for 24 to 28 doses (for subjects who are not strictly administered once a week due to objective reasons such as adverse reactions, the number of treatment weeks should not exceed treatment doses by 4) of the Day 1 visit and could provide the related treatment history.	Everest Project, with clinical practice taken into consideration, ≥12 months of first-line NrtI therapy and evidence of HBV DNA<LLOQ ≥6 months are sufficient to prove that subjects are in sustained HBV DNA control under long-term NrtI treatment.
Section 1.5 SoA and Section 8.2 Detailed Description of Study Procedures	Abdominal ultrasound was included as part of the assessment at screening and every 6 months during the study (Week 24/ET1 in the Dosing Period; Week 48/ET 2 in the Follow-up Period; Day 1, Week 24, and Week 48/ET 3 of the NrtI Discontinuation Monitoring Period)	Hepatocellular carcinoma surveillance
SoA Section 1.5.1 Note b	"ET1 assessments should be performed within 14 days" is revised into "ET1 assessments should be performed within 14 days after study withdrawal"	To clarify the time window of ET1
SoA Section 1.5.1 Note k and Section 10.2 Appendix 2: Clinical Laboratory Tests	Clarified blood pregnancy test will be conducted at central lab at screening visit, urine pregnancy test and blood pregnancy test (if necessary) will be conducted at local laboratory at other time points,	To clarify the sample collection and testing laboratory for pregnancy test.
SoA Section 1.5.1 Note n	"Additional safety laboratory assessments may be performed at any time during the study as determined necessary by the investigator (local laboratory assessment is acceptable)" is added to the note.	To clarify "Additional tests may be performed at any time during the study as determined necessary by the investigator or required by local regulations" in Section 10.2 Appendix 2: Clinical Laboratory
SoA Section 1.5.1 Note o	"If a subject experiences alanine aminotransferase (ALT) flare, additional HBV DNA quantitation sample(s) may be collected"is revised into"If a subject experiences clinically	To clarify timing of "additional HBV DNA quantitation sample(s) may be collected"

	significant alanine aminotransferase (ALT) flare, additional HBV DNA quantitation sample(s) may be collected as determined necessary by the investigator”	
SoA Section 1.5.1 Note p and Section 8.3.1 Time Period and Frequency for Collecting AE and SAE Information	The time period for adverse events (AEs) collection is clarified as “AEs will be collected from signing the ICF until the last visit”. The distinction between AE and medical history for events occurring between signing ICF and initiation of study drug is defined as “if related to study procedures, will be recorded as AEs; otherwise, will be recorded as medical history.”	To clarify the time frame for AE collection and to clarify the wording regarding categorization of events occurring between signing ICF and initiation of study drug as AEs or medical history.
SoA Section 1.5.1	“Subjects will have a FibroScan evaluation during screening. FibroScan evaluation performed within 6 months prior to screening is also acceptable for eligibility evaluation.” is included as Note s	Clarification on FibroScan results that can be used for eligibility evaluation to align with exclusion criterion 3.
Section 5.1 Inclusion Criteria	“Study screening laboratory tests may be repeated once for values considered to be erroneous with medical monitor approval” in inclusion criterion 10 is revised into “Study screening laboratory tests may be repeated once for values considered to be questionable by investigator with medical monitor approval” and put this sentence in another row.	Formatting correction and to clarify on “considered to be erroneous”.
Section 6.1 Study Interventions(s) Administered	Specify “entecavir tablet in combination with tenofovir disoproxil fumarate tablet or tenofovir alafenamide fumarate tablet may be provided if the investigator deems necessary”	To align with local clinical practice
Section 6.5 Concomitant Therapy	The whole “Permitted Medications” section is removed	Provide clear guidance on concomitant therapy, prohibited medications are listed in Section

		6.5.2, other medications are permitted.
Section 7.1 Discontinuation of Study Intervention	Change “if a subject discontinues study, every effort should be made to keep the subject in the study. If the subject discontinues both investigational product (BRII-179 or placebo) and PEG-INF-α treatment, the subject should continue to attend the required study visits and procedures for the remaining duration of study participation in Follow-up Period” into "If a subject discontinues both investigational product (BRII-179 or placebo) and PEG-IFN-α treatment, the subject will be encouraged to continue to attend the required study visits and procedures for the remaining duration of study participation in Follow-up Period"	Wording simplification and clarification
Section 8.1.3 Dosing Period	Specified solicited local/systemic reaction after study drug administration as follows: any local reactions (including redness, swelling, pain, itchiness and tenderness, etc.) and systemic reactions (tiredness, diarrhea, nausea/vomiting, headache, muscle pain, etc,) related to BRII-179 (VBI-2601) or placebo administration.	Provide more specific description regarding solicited local/systemic reaction monitoring after study drug administration and aligned the description with subject diary card.
Section 8.2.3 FibroScan and Section 5.2 Exclusion Criteria	“Subject with a FibroScan result of ≤ 9 kPa at screening can be considered without significant liver fibrosis and cirrhosis. If screening FirboScan result > 9 kPa, however, the subject has a result of ≤ 9 Kpa from a FibroScan evaluation performed within 6 months prior to screening or liver biopsy performed within 1 year prior to screening that confirm the absence of Metavir F3 fibrosis or F4 cirrhosis,	Clarification

	significant liver fibrosis and cirrhosis can be excluded as well. If a subject has had both procedures in the specified timeframes prior to screening, the most recent result should be used to determine eligibility" is clarified in Section 8.2.3.<br><br>Accordingly, exclusion criterion 3 is revised from "Significant liver fibrosis or cirrhosis as defined by having either a FibroScan result of > 9kPa at screening, unless the subject has results from a FibroScan evaluation performed within 6 months prior to screening or liver biopsy performed within 1 year prior to screening that confirm the absence of Metavir F3 fibrosis or F4 cirrhosis. If a subject has had both procedures in the specified timeframes, the most recent result should be used to determine eligibility" to "Significant liver fibrosis or cirrhosis as defined by having a FibroScan result of > 9 kPa at screening. Unless the subject has a result of ≤9 Kpa from a FibroScan evaluation performed within 6 months prior to screening or liver biopsy performed within 1 year prior to screening that confirm the absence of Metavir F3 fibrosis or F4 cirrhosis. If a subject has had both procedures in the specified timeframes prior to screening, the most recent result should be used to determine eligibility" for clarification.	
Section 8.2.9 Laboratory Events of Clinical Interest and Section 10.5 Appendix 5,	The time period for collecting events of clinical interest (ECI) is revised from "NrtI Discontinuation Monitoring Period" to "Day 1 of dosing to the last visit"	To correct the inconsistency between SOA Section 1.5.1 and text description of the time period for ECI collection.

Section 8.3.5.2 Other Special Situations	The criterion for reporting of other special situations is modified as follows: "All other special situations must be reported to the sponsor within 24 hours of the investigator becoming aware of the situation. These reports apply to study drug only. Special situations involving medications other than study drug do not need to be reported; however, for special situations that result in AEs, the AEs should be reported."	To clarify the difference in reporting rules between special situations related to study drug and related to other medications.
Section 9.6.4 Analyses of Secondary Efficacy Endpoints and Subgroups	Change "at baseline" in "subgroup analysis by age, gender, and HBsAg level at baseline (3 categories: ⩾0.05 to < 1 IU/mL, ⩾1 to < 10 IU/mL, and ⩾10 to <100 IU/mL) will be conducted with respect to the primary endpoint" to "at screening"	To clarify that the primary endpoint will be analysed by HBsAg level at screening.
Section 1.2 Phase 2a	Tertiary endpoint in the table was updated to "Serum protein level cytokine/chemokine biomarkers changes from baseline posting dosing, i.e., from Day 1 of dosing to the end of follow-up (Week 48) and during the NrtI discontinuation monitoring period".	To correct the inconsistency of tertiary endpoint description between Section 1.2 Phase 2a table and Section 3.2.3 Tertiary Endpoints.
Throughout the protocol	Typos and minor translation and formatting errors correction	To improve the overall quality of the protocol.

Table of Contents

TITLE..1
Protocol Revision History..4
TABLE OF CONTENTS 11
1. PROTOCOL SUMMARY..16
1.1. Synopsis 16
1.2. Phase 2a (N=120) 16
1.3. Phase 2b (N=480) 17
1.4. Schema..21
1.5. Schedule of Activities (SoA) 22
1.5.1. BRII-179 (VBI-2601) 18-week and PEG-IFN-α 24-week Dosing Period and 24-week Follow-up Period..22
1.5.2. NrtI Discontinuation Monitoring Period..25
2. INTRODUCTION..26
2.1. Background..26
2.2. Study Rationale 27
2.3. Benefit/Risk Assessment..28
3. OBJECTIVES AND ENDPOINTS..29
3.1. Objectives..29
3.1.1. The Primary Objectives 29
3.1.2. The Secondary objectives..29
3.1.3. The Tertiary Objectives 29
3.2. Endpoints..29
3.2.1. Primary Endpoints 29
3.2.2. Secondary Endpoints..29
3.2.3. Tertiary Endpoints 30
4. STUDY DESIGN..31
4.1. Overall Design..31
4.1.1. Independent DSMB..31
4.1.2. NrtI Discontinuation..31
4.1.3. PEG-IFN-α Therapy During the Dosing Period..32
4.2. Scientific Rationale for Study Design 34
4.3. Dosage Instructions..35

4.3.1. Justification for Dose Selection and Number of Doses 35
4.3.2. Maximum dose/exposure in this study 36
4.4. End of Study Definition 36
5. STUDY POPULATION 37
5.1. Inclusion Criteria 37
5.2. Exclusion Criteria 38
5.3. Lifestyle Considerations 40
5.4. Screening Failures 40
6. STUDY INTERVENTION 41
6.1. Study Intervention(s) Administered 41
6.1.1. Formulations 41
6.1.2. Study Intervention 42
6.2. Preparation/Handling/Storage/Accountability 43
6.3. Randomization and Blinding 43
6.4. Compliance of Study Intervention 44
6.5. Concomitant Therapy 44
6.5.1. Prohibited Medications 45
6.6. Safety Follow-up and Management of Reactions to Study Drug 45
6.7. Treatment of Study Intervention Overdose 46
6.8. Intervention after the End of the Study 46
7. DISCONTINUATION OF STUDY INTERVENTION AND SUBJECT DISCONTINUATION/WITHDRAWAL 47
7.1. Discontinuation of Study Intervention 47
7.1.1. Criteria for Discontinuation of Study Intervention 47
7.2. Subject Discontinuation/Withdrawal from the Study 48
7.2.1. Lost to Follow up 48
8. STUDY ASSESSMENTS AND PROCEDURES 49
8.1. Schedule of Assessments 49
8.1.1. Screening 49
8.1.2. Randomization (Day 1) 49
8.1.3. Dosing Period 49
8.1.4. Follow-up Period 50
8.1.5. NrtI Discontinuation Monitoring Period 50
8.1.6. Unscheduled Visits 50
8.1.7. Early Termination Visit 50

8.2. Detailed Description of Study Procedures 51
8.2.1. Medical History 51
8.2.2. Physical Examinations 51
8.2.3. FibroScan 51
8.2.4. Abdominal Ultrasound 51
8.2.5. Alcohol Intake Assessment 51
8.2.6. Vital Signs 51
8.2.7. Electrocardiograms 52
8.2.8. Laboratory Assessments 52
8.2.9. Laboratory Events of Clinical Interest 53
8.3. Adverse Events and Serious Adverse Events 53
8.3.1. Time Period and Frequency for Collecting AE and SAE Information 53
8.3.2. Method of Detecting AEs and SAEs 54
8.3.3. Follow-up of AEs and SAEs 54
8.3.4. Regulatory Reporting Requirements for SAEs 54
8.3.5. Special Situations 55
8.4. Pharmacodynamics 56
8.5. Genetics 56
8.6. [Health Economics] OR [Medical Resource Utilization and Health Economics] 56
9. STATISTICAL CONSIDERATIONS 57
9.1. Statistical Hypotheses 57
9.1.1. Phase 2a Part 57
9.1.2. Phase 2b Part 57
9.2. Sample Size Determination 57
9.2.1. Phase 2a Part 57
9.2.2. Phase 2b Part 57
9.3. Data Monitoring Guidelines for Independent DSMB 58
9.4. Primary Endpoint and Safety Data Analysis in Phase 2 Part 59
9.4.1. Monitoring Guidelines for the Primary Endpoint in Phase 2b Part 59
9.5. Analysis Sets 59
9.6. Statistical Analyses 60
9.6.1. General considerations 60
9.6.2. Analysis of the Primary Efficacy Endpoint for Phase 2a Part 61
9.6.3. Analysis of the Primary Efficacy Endpoint for Phase 2b Part 61

9.6.4. Analyses of Secondary Efficacy Endpoints and Subgroups....61
9.6.5. Tertiary Efficacy Endpoint and Subgroup Analyses of Phase 2a Part....61
9.6.6. Analysis of Safety Outcome....62
10. SUPPORTING DOCUMENTATION AND OPERATIONAL CONSIDERATIONS....63
10.1. Appendix 1: Regulatory, Ethical, and Study Oversight Considerations....63
10.1.1. Regulatory and Ethical Considerations....63
10.1.2. Financial Disclosure....63
10.1.3. Informed Consent Process....63
10.1.4. Data Protection....64
10.1.5. Data Quality Assurance....64
10.1.6. Source Documents....65
10.1.7. Study and Site Start and Closure....65
10.1.8. Publication Policy....66
10.2. Appendix 2: Clinical Laboratory Tests....67
10.3. Appendix 3: Adverse Events: Definitions and Procedures for Recording, Evaluating, Follow-up, and Reporting....68
10.3.1. Definition of AE....68
10.3.2. Definition of SAE....69
10.3.3. Recording and Follow-Up of AE and/or SAE....70
10.3.4. Reporting of SAEs....71
10.3.5. Reporting of Laboratory Events of Clinical Interest....71
10.3.6. Special Situation Report....71
10.4. Appendix 4: Contraceptive Requirements and Collection and Reporting of Pregnancy Information....72
10.5. Appendix 5: Assessments in Subjects Meeting Laboratory ECI Criteria....74
11. REFERENCES....76

ABBREVIATIONS

AE	adverse event
ALT	alanine aminotransferase
HBsAb	hepatitis B surface antibody
DNA	deoxyribonucleic acid
DSMB	Data and Safety Monitoring Board
ECG	electrocardiogram
ECI	events of clinical interest
ET	early termination
ETV	entecavir
HBeAg	hepatitis B e-antigen
HBsAg	hepatitis B surface antigen
HBV	hepatitis B virus
HCV	hepatitis C virus
HDV	hepatitis D virus
HIV	human immunodeficiency virus
ICF	informed consent form
IFN-α	interferon-alpha
IMP	Investigational medicinal product
IU	international units
kPa	kilopascal
LLOQ	lower limit of quantification
NCI-CTCAE	National Cancer Institute Common Terminology Criteria for Adverse Events
NIMP	Non-investigational medicinal product
NrtI	nucleos(t)ide reverse transcriptase inhibitors
ULN	upper limit of normal
PEG-IFN-α	pegylated interferon-alpha
PD	Pharmacodynamic
SoA	schedule of activities
SAE	serious adverse event
TDF	tenofovir disoproxil fumarate
TAF	tenofovir alafenamide
WOCBP	women of child-bearing potential

1. PROTOCOL SUMMARY

1.1. Synopsis

Protocol Title: A Multicenter, Randomized, Double-Blind, Placebo-Controlled, Parallel-Group Study to Investigate the Efficacy and Safety of BRII-179 (VBI-2601) for the Treatment of Chronic Hepatitis B Virus (HBV) Infection

Rationale:
Multiple therapies with differential mechanisms of actions to treat hepatitis B virus (HBV) is an encouraging strategy to achieve higher rates of hepatitis B surface antigen (HBsAg) loss and ideally HBsAg seroconversion. The present study (BRII-179-002) will evaluate the efficacy and safety of adding a recombinant protein immunotherapeutic (BRII-179 [VBI-2601]) treatment regimen to existing pegylated interferon-alpha (PEG-IFN-α) and nucleos(t)ide reverse transcriptase inhibitor (NrtI) therapy in chronic HBV patients. Most subjects in the current study are anticipated to be recruited from the Everest Project (NCT04035837). The primary objective of the Everest Project is to achieve functional cure of HBV infection by optimizing treatment strategies of NrtIs and PEG-IFN-α.

Individualized regimens of IFN and NrtI therapies have shown promising efficacy in reducing HBsAg with some patients achieving HBsAg clearance (Ren, 2016; Marcellin, 2016). This result is more pronounced in patients on optimized HBV treatment to low HBsAg levels, which is the rationale of including subjects from the Everest Project in this study. Preclinical and clinical studies have also shown the benefit of low HBsAg levels for breaking tolerance with a therapeutic HBV vaccine (Zhu, 2016; Michler, 2020; Roggendorf, 2019). The recombinant protein immunotherapeutic, BRII-179 (VBI-2601), may break immunological tolerance and restore and/or augment adaptive T cell and antibody responses to the surface antigens. These responses together have the potential to better achieve a functional cure (sustained loss of HBsAg in blood with or without seroconversion to the surface antigen antibody) of chronic HBV infection.

Objectives and Endpoints

The study will consist of two parts, Part 2a and 2b, of which 120 subjects are planned to be enrolled in the first part (Phase 2a) and 480 subjects are planned to be enrolled in the second part (Phase 2b).

1.2. Phase 2a (N=120)

Objectives	Endpoints
Primary	
To determine the efficacy of BRII-179 (VBI-2601) to increase HBsAg loss through completion of PEG-IFN-α treatment	• Percentage of subjects with HBsAg loss at completion of PEG-IFN-α treatment
Secondary	

▪ To evaluate functional cure of subjects with chronic HBV infection receiving BRII-179 (VBI-2601) therapy in combination with PEG-IFN-α + NrtI	• Percentage of subjects with sustained HBsAg loss and persistently undetectable HBV DNA (defined as undetectable HBsAg and HBV DNA for at least 24 weeks) after NrtI discontinuation.
▪ To assess the antiviral activity of BRII-179 (VBI-2601) therapy in combination with PEG-IFN-α + NrtI	• HBsAg change from baseline from Day 1 to completion of PEG-IFN-α treatment<br>• Percentage of subjects with HBsAg loss at any timepoint<br>• Percentage of subjects with undetectable HBV-DNA (HBV DNA < Lower Limit of Quantification (LLOQ)) 24 and 48 weeks after NrtI discontinuation<br>• Percentage of subjects meeting the NrtI discontinuation criteria by end of follow-up period (Week 48)<br>• Percentage of subjects with HBsAg seroconversion at any timepoint<br>• Percentage of subjects with hepatitis B surface antibody (HBsAb) response as of the end of the follow-up period (Week 48)<br>• Time to achieve HBsAg seroclearance and seroconversion<br>• Percentage of subjects requiring NrtI re-treatment during follow up
To assess safety of BRII-179 (VBI-2601) therapy	• Percentage of subjects with adverse events (AEs) and serious adverse events (SAEs)<br>• Percentage of subjects with abnormalities in clinical laboratory tests
**Tertiary**	
To assess the effect on relevant immune markers in subjects with chronic HBV infection treated with BRII-179 (VBI-2601) in combination with PEG-IFN-α + NrtI	• Serum protein level cytokine/chemokine biomarkers changes from baseline post dosing, i.e., from Day 1 of dosing to the end of follow-up (Week 48) and during the NrtI discontinuation monitoring period

1.3. Phase 2b (N=480)

Objectives	Endpoints
Primary	
To evaluate functional cure of subjects with chronic HBV infection receiving BRII-179 (VBI-2601) therapy in combination with PEG-IFN-α + NrtI	• Percentage of subjects with sustained HBsAg and HBV DNA loss (defined as undetectable HBsAg and HBV DNA for at least 24 weeks) after NrtI discontinuation

Secondary	
To assess the antiviral activity of BRII-179 (VBI-2601) therapy in combination with PEG-IFN-α + NrtI	• Percentage of subjects with undetectable HBV-DNA (HBV DNA < Lower Limit of Quantification (LLOQ)) 24 and 48 weeks after NrtI discontinuation<br>• Percentage of subjects meeting the NrtI discontinuation criteria by end of follow-up period (Week 48)<br>• Percentage of subjects with HBsAg loss at any timepoint<br>• Percentage of subjects with HBsAg seroconversion at any timepoint<br>• Percentage of subjects with hepatitis B surface antibody (HBsAb) response by end of follow-up period (Week 48)<br>• HBsAg change from baseline from Day 1 to End of Treatment (Week 24)<br>• Time to achieve HBsAg seroclearance and seroconversion<br>• Percentage of subjects requiring NrtI re-treatment during follow up
To assess safety of BRII-179 (VBI-2601) therapy	• Percentage of subjects with adverse events (AEs) and serious adverse events (SAEs)<br>• Percentage of subjects with abnormalities in clinical laboratory tests

Overall Design

Study BRII-179-002 is a double-blind, randomized, placebo-controlled, parallel-group study aims to evaluate the clinical effect of adding BRII-179 (VBI-2601) to existing PEG-IFN-α and NrtI therapy in patients with chronic HBV infection without cirrhosis.

Most subjects in the current study are anticipated to be recruited from the Everest Project (NCT04035837). The Everest Project is a multicenter real-world study being conducted in over 260 hospitals in China. The plan is to recruit 30,000 patients with chronic HBV infection effectively treated with NrtI therapy for at least one year; who are hepatitis B e-antigen (HBeAg) negative with HBV DNA <100 IU/ml and HBsAg <1500 IU/mL at study entry. As part of the study participation, subjects receive PEG-IFN-α as "add-on" or "switch to" treatment for 48 to 96 weeks. The primary objective of the Everest Project is to achieve functional cure of HBV infection by optimizing treatment strategies of NrtIs and PEG-IFN-α.

Eligible subjects who have been receiving PEG-IFN-α for 24 to 28 doses and NrtI therapy for at least 12 months with HBV DNA<LLOQ for ≥ 6 months, will be randomized 1:1 to receive BRII-179 (VBI-2601) or placebo in addition to the existing PEG-IFN-α and NrtI therapy. Subjects will be stratified at randomization by screening HBsAg level (3 categories: ≥0.05 to < 1 IU/mL, ≥1 to < 10 IU/mL, and ≥10 to <100 IU/mL. Subjects will complete investigational treatment with BRII-179 or placebo at Week 18; PEG-IFN-α treatment will last for up to 24 weeks after enrollment; while NrtI treatment will continue into the Follow-up Period. The BRII-179-002 study design schema can be found in Section 1.4.

Estimated total duration for each subject will include the following: screening up to 28 days, dosing period 24 weeks (18 weeks BRII-179 or placebo investigational treatment; 24 weeks PEG-IFN-α treatment), and a 24-week follow-up period. Eligible subjects that meet NrtI discontinuation criteria (Section 4.1.2.1) (eligibility assessed at Week 36 to Week 48) may enter a NrtI discontinuation monitoring period for an additional 48 weeks from the day of NrtI discontinuation (Section 1.5.2).

Number of Subjects:

The study will consist of two parts, Part 2a and 2b, of which 120 subjects are planned to be enrolled in the first part (Phase 2a) and 480 subjects are planned to be enrolled in the second part (Phase 2b).

Intervention Groups and Duration:

Eligible subjects currently receiving PEG-IFN-α and NrtI therapy will receive the study drug BRII-179 (VBI-2601) or placebo, every three weeks for a total of 7 doses over an 18-week course.

Both groups will be treated with both PEG-IFN-α and NrtI, with PEG-IFN-α lasted for 24 weeks after enrollment , and NrtI through the follow-up period.

Independent Data and Safety Monitoring Board (DSMB)

An independent DSMB will monitor subject safety and treatment efficacy data for this study. The details for the operation and responsibilities of the DSMB will be defined in a separate DSMB Charter. The Charter will delineate the composition, duties, responsibilities, and procedures of the DSMB, data required at each meeting, as well as the analyses that will be conducted.

Disclosure Statement: This is a phase 2, randomized, multicenter, double-blind, placebo-controlled, parallel-group study to evaluate the efficacy and safety of BRII-179 (VBI-2601) in patients with chronic HBV infection receiving PEG-IFN-α and NrtI therapy. All references to "Sponsor" contained herein shall refer to Brii Biosciences.

1.4. Schema

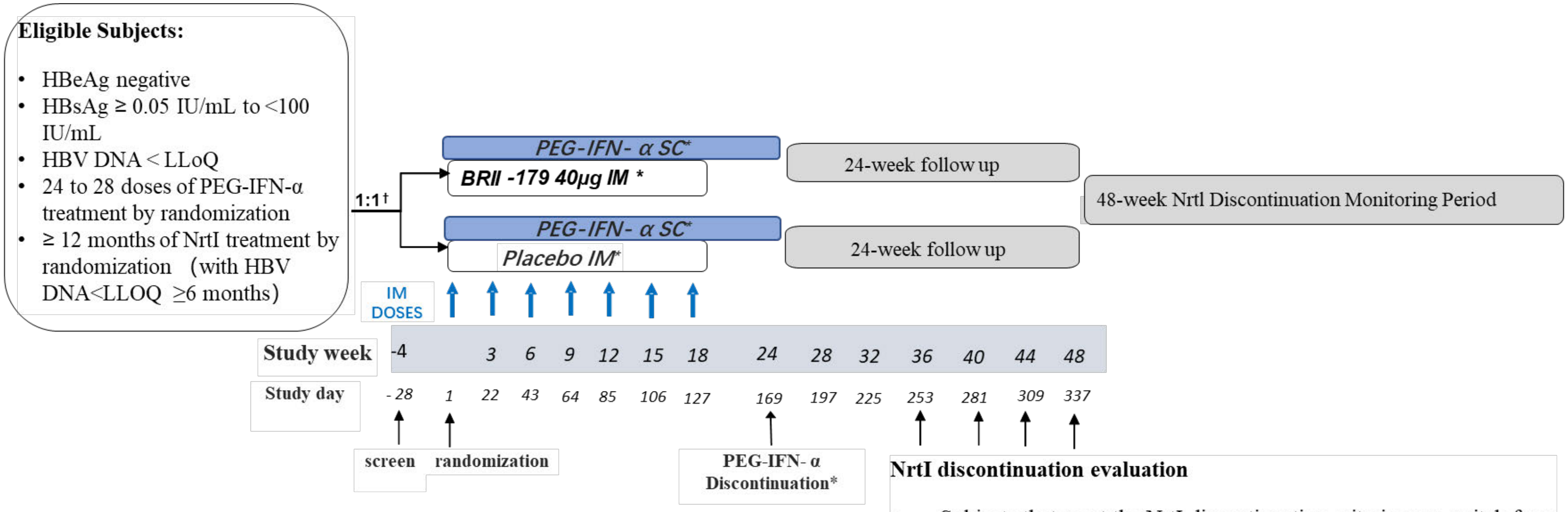

NrtI discontinuation evaluation

- Subjects that meet the NrtI discontinuation criteria may switch from the follow-up period to the 48-week NrtI discontinuation monitoring period
- Day 1 of the NrtI Discontinuation Monitoring Period is the first day a subject discontinues NrtI
- Subjects that have not met NrtI discontinuation criteria by Week 48 will be withdrawn from the study

IM: intramuscular ; SC : Subcutaneous Injection

* Each subject will accept 24 weeks PEG-IFN-α treatment after enrollment; Co-medication of NrtI will continue during the dosing period; NrtI treatment will last until week 48 if the subject could not meet the NrtI discontinuation criteria

† Subjects stratified by HBsAg level

1.5. Schedule of Activities (SoA)

1.5.1. BRII-179 (VBI-2601) 18-week and PEG-IFN-α 24-week Dosing Period and 24-week Follow-up Period

**Study Stage**	**Screening**	**Dosing Period [b]**								**Follow-up Period**					
**Study Visit Week**	Screening	Rand	W3	W6	W9	W12	W15	W18	W24 [c] /ET1	W28	W32	W36[d]	W40[d]	W44[d]	W48[d]/ ET2
**Study Visit Day ± Visit Window**	D-28 to-1	D1[a]	D22 ± 3	D43 ± 3	D64 ± 3	D85 ± 3	D106± 3	D127 ± 3	D169 ± 3	D197 ± 3	D225 ± 3	D253 ± 5	D281 ± 5	D309 ± 5	D337 ± 5
Informed consent form (ICF)	X														
Inclusion/exclusion criteria	X	X[e]													
Demography	X														
Medical history including current medical conditions and HBV genotype [f]	X														
Abdominal Ultrasound	X								X						X
FibroScan	X[s]														
Full physical exam [g]	X							X	X						X
Symptom-directed physical exam		X	X	X	X	X	X			X	X	X	X	X	
Alcohol intake assessment [h]		X	X	X	X	X	X	X	X	X	X	X	X	X	X
Vital signs [i]	X	X				X			X						
12-lead ECG [j]	X	X							X						
Subject instructed to complete diary		X	X	X	X	X	X	X	X	X	X	X	X	X	X
Urinalysis [n]	X														
Pregnancy test [k]	X	X	X	X	X	X	X	X	X	X	X	X	X	X	X
Safety Laboratory Assessments [n]	X	X	X	X	X	X	X	X	X	X	X	X	X	X	X

Randomization		X													
Study drug administration		X	X	X	X	X	X	X[l]	X[m]						
Screening viral serology	X														
Blood sample for HBsAg/HBsAb quantitative	X	X		X		X	X	X	X	X	X	X	X	X	X
Blood sample for cytokine/chemokine biomarker quantitation	X	X		X		X		X	X		X		X		X
Blood sample for HBV DNA quantitation[o]	X	X				X			X	X	X	X	X	X	X
Adverse Events assessment [p]	←---X--→														
ECI surveillance [q]	←---X--→														
Concomitant medications review	←---X--→														
NrtI medication adherence review [r]	←---X--→														
PEG-IFN-α medication adherence review [r]	←---------------------X---------------------→														

AE = adverse event; HBsAg = hepatitis B surface antigen; HBsAb = hepatitis B surface antibody; ECI = events of clinical interest; ECG = electrocardiogram; ET1 = early termination 1; ET2 = early termination 2; HBV = Hepatitis B Virus; NrtI = nucleoside/nucleotide reverse transcriptase inhibitor; ULN = Upper Limit of Normal

Note: When scheduled at the same timepoints, the assessments of vital signs and 12-lead ECGs must be performed before blood sample collections.

[a] Assessments performed pre-dose unless otherwise specified.

[b] If a subject withdraws prematurely from the study prior to their Week 24 visit, ET1 assessments should be performed within 14 days after study withdrawal.

[c] This visit occurs when complete PEG-IFN-α treatment,24 weeks after the enrollment.

[d] Subjects that meet NrtI discontinuation criteria (Section 4.1.2.1) may discontinue from the Follow-up Period and enter the NrtI Discontinuation Monitoring Period (Section 1.5.2). Day 1 of the NrtI Discontinuation Monitoring Period is the first day subject stops their NrtI therapy.

[e] Evaluation of inclusion and exclusion criteria related to pregnancy test, medical history and concomitant medications prior to dosing.

[f] A complete medical history will be taken at screening including HBV genotype if available.

[g] Height and weight will be recorded at screening. Body mass index will be calculated based on the measurements. Only weight will be collected afterwards.

[h] Subjects' alcohol intake will be recorded while on study.

[i] Vital signs (blood pressure, pulse rate, respiratory rate, and temperature) should be measured after the subject has rested comfortably for approximately 5 minutes.
[j] 12-lead safety ECGs should be measured in the supine position after the subject has rested comfortably for 10 minutes.
[k] Women of childbearing potential are required to have pregnancy tests. Negative pregnancy test must be confirmed prior to study drug administration. Blood pregnancy test should be conducted at central lab at screening visit, urine pregnancy test and blood pregnancy test (if necessary) at other time points will be conducted at local laboratory.
[l] Subjects will complete investigational treatment with BRII-179 or placebo;
[m] PEG-IFN-α treatment will be continued for 24 weeks ; NrtI therapy will continue into the Follow-up Period.
[n] Refer to Table 2 Safety Assessments in Appendix 2. Additional safety laboratory assessments may be performed at any time during the study as determined necessary by the investigator (local laboratory assessment is acceptable).
[o] If a subject experiences clinically significant alanine aminotransferase (ALT) flare, additional HBV DNA quantitation sample(s) may be collected as determined necessary by the investigator.
[p] AEs will be collected from signing the ICF until last visit. After informed consent has been obtained but prior to initiation of study drug, untoward medical occurrence, if related to study procedure, will be recorded as AE; otherwise, will be recorded as medical history.
[q] Subject will be monitored for clinical laboratory events of clinical interest (ECI) and additional assessments may be performed if indicated. ECI for this study include ALT > 3x ULN, if Day 1 pre-dose ALT ≤ ULN; ALT > 2x baseline, if Day 1 pre-dose ALT > ULN.
[r] Subjects will be instructed to bring NrtI and PEG-IFN-α (including all empty containers) to the trial site at every visit, as applicable, beginning with the screening visit. Adherence will be assessed by counting of empty containers and unused medication. See Section 6.4.
[s] Subjects will have a FibroScan evaluation during screening. FibroScan evaluation performed within 6 months prior to screening is also acceptable for eligibility evaluation.

1.5.2. NrtI Discontinuation Monitoring Period

**Study Stage**	**NrtI Discontinuation Monitoring Period**[b,c]						
**Study Visit Week**		W4	W8	W12	W24	W36	W48/ET3
**Study Visit Day ± Visit Window**	D1[a]	D28 ± 3	D56 ± 3	D84 ± 3	D168 ± 7	D252 ± 7	D336 ± 7
Safety Laboratory Assessments [d]	X	X	X	X	X	X	X
Symptom-directed physical exam	X	X	X	X	X	X	X
Abdominal Ultrasound	X				X		X
FibroScan							X
Pregnancy test		X					X
Blood sample for HBsAg/HBsAb quantitation	X			X	X	X	X
Blood sample for cytokine/chemokine biomarker quantitation	X		X		X		X
Blood sample for HBV DNA quantitation	X	X	X	X	X	X	X
Concomitant medications	←-------------------X-------------------→						
Adverse event assessment	←-------------------X-------------------→						
NrtI retreatment surveillance [e]	←-------------------X-------------------→						
ECI surveillance [f]	←-------------------X-------------------→						

AE = adverse event; ECI = events of clinical interest; HBsAg = hepatitis B surface antigen; HBsAb = hepatitis B surface antibody; HBV = Hepatitis B Virus; NrtI = nucleoside/nucleotide; ULN = Upper Limit of Normal; ET3 = early termination 3

[a] All timepoints are relative to the day of NrtI discontinuation (D1) which is the first day subjects stop their NrtI therapy.

[b] All subjects who discontinue NrtI therapy will be followed through 48 weeks, even if NrtI retreatment occurs. If a subject withdraws prematurely from the study prior to the W48 visit, ET assessments should be performed.

[c] NrtI retreatment guidelines are provided in Section 4.1.2.2.

[d] Safety laboratory assessments are limited to liver function tests.

[e] Investigator should monitor subjects for NrtI retreatment criteria. See Section 4.1.2.2.

[f] Subject will be monitored for clinical laboratory events of clinical interest (ECI) and additional assessments may be performed if indicated. ECI for this study include ALT > 3x ULN, if Day 1 pre-dose ALT ≤ ULN; ALT > 2x baseline, if Day 1 pre-dose ALT > ULN.

2. INTRODUCTION

2.1. Background

Chronic HBV infection remains an important global public health problem that leads to serious sequelae over time, including cirrhosis, liver failure, and hepatocellular carcinoma (Trepo, 2014). An estimated 290 million people are living with chronic HBV infection worldwide, and almost 800,000 people die annually due to associated sequelae (Stanaway, 2016).

Human HBV is a deoxyribonucleic acid (DNA) virus in the Hepadnaviridae family, with 10 HBV genotypes (A to J). The distribution of these genotypes varies geographically, with A and D being more common in Northern Europe, North America, and Western and Central Asia; and B and C being more common in Southeast Asia and China. HBV genotypes may influence disease progression rates and treatment response (Kramvis, 2014).

Chronic HBV infection is characterized by detection of HBV surface antigen (HBsAg) in the blood for > 6 months. The prevalence of chronic HBV infection varies geographically, with a range of < 2% in low to > 8% in high prevalence countries (Schweitzer, 2015). In high prevalence countries, such as those in sub-Saharan Africa and East Asia, transmission occurs predominantly in infants and children by perinatal and horizontal routes. In more industrialized countries, new infections are highest among young adults and transmission occurs predominantly via injection drug use and high-risk sexual behaviors. The risk of developing chronic HBV infection depends on the age at the time of infection. While only ~10% of people infected as adults develop chronic HBV infection, 90% of infants infected perinatally or during the first 6 months of life, and 20% to 60% of children infected between 6 months and 5 years of age remain chronically infected. Twenty-five percent of people who acquire HBV during infancy and childhood will develop primary liver cancer or cirrhosis during adulthood.

Current treatment options for chronic HBV infection are limited. One option is long-term Nucleoside reverse transcriptase inhibitors (NrtI) therapy, for example tenofovir (tenofovir disoproxil fumarate or tenofovir alafenamide) or entecavir (Lok, 2016). However, NrtI therapy does not result in a cure of HBV infection. The viral DNA is not cleared from the infected cell, and viral protein production, in particular the production of HBsAg, typically continues at high levels as measured in blood (Scaglione, 2012). These high levels of HBsAg subviral particles are proposed to mediate HBV-specific immune dysfunction by decoying and exhausting host cellular and humoral adaptive immune responses. Therefore, reduction or clearance of HBsAg from blood is considered central to achieving a functional cure (sustained loss of HBsAg in blood with or without seroconversion to HBsAb) and is considered an optimal outcome of treatment (Block, 2013). An alternative treatment option is a finite course of Pegylated Interferon α (PEG-IFN-α) therapy which can induce a functional cure, but only in a small percentage of patients (< 10%) (Konerman, 2016). Unfortunately, PEG-IFN-α therapy carries considerable risk of dose-limiting adverse events (AEs) which may be severe and/or serious, and discontinuation rates are significant (Perrillo, 2009).

The failure of NrtI therapy to produce a functional cure and the limitations of PEG-IFN-α therapy highlight the clinical need for effective, well-tolerated HBV therapies of finite duration.

2.2. Study Rationale

BRII-179 (VBI-2601) is a recombinant virus-like particle-based (VLP) based protein immunotherapeutic derived from the 3rd generation HBV prophylaxis vaccine, Sci-B-Vac® consists of all three HBV surface (S) antigen forms: small, middle and large proteins. The large S protein contains the N-terminal preS1 and preS2 domains and the adjacent small S protein. The middle S protein contains preS2 and the adjacent small S protein.

It has been hypothesized that high levels of circulating HBsAg lead to immune tolerance of HBV and contribute to chronic HBV infection. BRII-179 (VBI-2601) utilizes Pre-S1, Pre-S2, and S protein components as the immunogen in a therapeutic application of preS1/S2 containing HBsAg. Additionally, to have an effective T cell response, BRII-179 (VBI-2601) differs from Sci-B-Vac® and most of the recombinant envelope vaccines by using aluminium phosphate as an adjuvant rather than aluminum hydroxide. As a new formulation of higher doses of Sci-B-Vac® with aluminum-phosphate adjuvant, BRII-179 (VBI-2601) shows enhanced Th1 responses against the HBV surface antigens. BRII-179 has been shown to be safe and well-tolerated in chronic HBV (CHB) patients under Nrtl therapy in a phase 1b/2a study (BRII-179-001). Administration of BRII-179 carrying S, Pre-S1 and Pre-S2 regions of HBV envelope is immunogenic at both B and T cell levels in CHB patients.

Multiple therapies with differential mechanisms of actions to treat HBV is an encouraging strategy to achieve higher rates of HBsAg loss and ideally HBsAg seroconversion. The present study (BRII-179-002) will evaluate the efficacy and safety of adding a recombinant protein immunotherapeutic (BRII-179 [VBI-2601]) treatment regimen to existing PEG-IFN-α and NrtI therapy in patients with chronic HBV infection. Most subjects in the current study are anticipated to be recruited from the Everest Project (NCT04035837). The primary objective of the Everest Project is to achieve functional cure of HBV infection by optimizing combination and sequential treatment strategies of NrtIs and PEG-IFN-α in patients with chronic HBV infection (negative HBeAg) and low levels of HBsAg (≤ 1500 IU/mL) and HBV DNA (< 100 IU/mL).

Individualized regimens of combination therapy of IFN-α, an innate immunomodulator and antiviral agent and NrtI, a direct acting antiviral agent have shown promising efficacy in reducing HBsAg with some patients achieving HBsAg clearance (Ren, 2016; Marcellin, 2016). This result is more pronounced in patients on optimized HBV treatment to low baseline HBsAg levels, which is the rationale of including subjects from the Everest Project in this study. Preclinical and clinical studies have also shown the benefit of low HBsAg levels for breaking tolerance with a therapeutic HBV vaccine (Zhu, 2016; Michler, 2020; Roggendorf, 2019). BRII-179 (VBI-2601), as an immunomodulator, can break immunological tolerance and restore and/or augment cellular T cell and antibody responses to the HBV surface antigens which in turn result in HBsAg clearance. Therefore, the initial treatment of PEG-IFN-α and NrtI followed by BRII-179 add-on treatment could increase the potential to achieve higher functional cure rate in chronic HBV infected patients.

2.3. Benefit/Risk Assessment

This study will provide information on the efficacy and safety of BRII-179 (VBI-2601) added to PEG-IFN-α and NrtI optimized therapy to achieve functional cure in patients with chronic HBV infection.

There are extensive human safety and immunogenicity data for antigens and adjuvant components used in BRII-179. Sci-B-Vac® with identical antigenic components has been safely used in more than 500,000 people (healthy subjects and CHB patients) worldwide (including the United States, Canada, Europe, Hong Kong of China), including more than 600 subjects who received Sci-B-Vac® with the same aluminum phosphate adjuvant as that in BRII-179 and had a good tolerability profile. In addition, the immunogenicity and safety of Sci-B-Vac® vaccines with aluminum phosphate adjuvant for early clinical studies are comparable to those with aluminum hydroxide adjuvant at a later stage. In the completed acute and chronic toxicology studies, the main toxicity profile of BRII-179 was generally consistent in rabbits and rats (Sci-B-Vac®) at dose levels exceeding 40 times the planned clinical dose of BRII-179, including mild and reversible side effects at intramuscular injection (IM) sites which were generally recoverable. In nonclinical toxicology studies, acute and chronic multiple-dose toxicology studies in rats and New Zealand White rabbits were conducted to support the currently planned number of clinical doses. BRII-179 (VBI-2601) with or without IFN-α as an adjuvant, administered every 4 weeks for a total of 4 doses, have been evaluated in adult subjects with chronic HBV infection. Favourable safety and tolerability profiles were observed at 40 µg dose level. In addition, the 8-month therapeutic vaccine trial (Sci-B-Vac®), which has been administered once a month in the Vietnamese population, has shown good efficacy and good safety data.

Based on the above mentioned available clinical and non-clinical data, the proposed add-on of BRII-179 (VBI-2601) to PEG-IFN-α and NrtI therapy may further reduce HBsAg effectively and induce HBV-specific immunity which may lead to immune control and functional cure of chronic HBV infection. The safety profile of BRII-179 (VBI-2601) is supportive of the overall risk/benefit profile for the proposed dose regimen and treatment duration.

More detailed information about the known and expected benefits and risks and reasonably expected adverse events of BRII-179 (VBI-2601) may be found in the Investigator’s Brochure.

3. OBJECTIVES AND ENDPOINTS

3.1. Objectives

3.1.1. The Primary Objectives

- Phase 2a: To evaluate the efficacy of BRII-179 to increase HBsAg loss through completion of PEG-IFN-α treatment
- Phase 2b: To evaluate functional cure of chronic HBV subjects receiving BRII-179 (VBI-2601) therapy in combination with PEG-IFN-α + NrtI.

3.1.2. The Secondary objectives

- To assess the antiviral activity of BRII-179 (VBI-2601) therapy added to PEG-IFN-α + NrtI.
- To assess safety of BRII-179 (VBI-2601) therapy

3.1.3. The Tertiary Objectives

- Phase 2a only: To assess the effect on relevant immune markers in subjects with chronic HBV infection treated with BRII-179 (VBI-2601) in combination with PEG-IFN-α + NrtI

3.2. Endpoints

3.2.1. Primary Endpoints

- Phase 2a: Percentage of subjects with HBsAg loss at completion of PEG-IFN-α treatment
- Phase 2b: Percentage of subjects with sustained HBsAg and HBV DNA loss (defined as undetectable HBsAg and HBV DNA for at least 24 weeks) after NrtI discontinuation

3.2.2. Secondary Endpoints

Below secondary endpoints apply to both Phase 2a and Phase 2b unless otherwise specified.

- HBsAg change from baseline of from Day 1 to completion of PEG-IFN-α treatment
- Percentage of subjects with HBsAg loss at any timepoint
- Phase 2a only: Percentage of subjects with sustained HBsAg loss and persistently undetectable HBV DNA (defined as undetectable HBsAg and HBV DNA for at least 24 weeks) after NrtI discontinuation

- Percentage of subjects with undetectable HBV-DNA (HBV DNA < LLOQ) 24 and 48 weeks after NrtI discontinuation
- Percentage of subjects meeting the NrtI discontinuation criteria by end of follow-up period (Week 48)
- Percentage of subjects with HBsAg seroconversion at any timepoint
- Percentage of subjects with HBsAb response by end of follow-up period (Week 48)
- HBsAg change from baseline from Day 1 to End of Treatment (Week 24)
- Time to achieve HBsAg seroclearance and seroconversion
- Percentage of subjects requiring NrtI re-treatment during follow up
- Percentage of subjects with adverse events (AEs) and serious adverse events (SAEs)
- Percentage of subjects with abnormalities in clinical laboratory tests

3.2.3. Tertiary Endpoints

Phase 2a only: Assessment of serum protein level cytokine/chemokine biomarkers changes from baseline post dosing, i.e., from Day 1 of dosing to the end of follow-up (Week 48), and during the NrtI discontinuation monitoring period

4. STUDY DESIGN

4.1. Overall Design

Study BRII-179-002 is a double-blind, randomized, placebo-controlled, parallel-group study of adding BRII-179 (VBI-2601) to existing PEG-IFN-α and NrtI therapy in patients with chronic HBV infection without cirrhosis.

Most subjects of the current study are anticipated to be recruited from the Everest Project (NCT04035837). The Everest Project is a multicenter real-world study being conducted in over 260 hospitals in China. The plan is to recruit 30,000 patients with chronic HBV effectively treated with NrtI therapy for at least one year; who are HBeAg negative with HBV DNA <100 IU/ml and HBsAg <1500 IU/mL at study entry. As part of the study participation, subjects receive PEG-IFN-α as "add-on" or "switch to" treatment for 48 to 96 weeks. The primary objective of the Everest Project is to achieve functional cure of HBV infection by optimizing treatment strategies of NrtIs and PEG-IFN-α.

Eligible subjects who have received PEG-IFN-α for 24 to 28 doses and NrtI therapy for at least 12 months with HBV DNA<LLOQ for ≥ 6 months, will be randomized 1:1 to receive BRII-179 (VBI-2601) or placebo in addition to the existing PEG-IFN-α and NrtI therapy. Subjects will be stratified at randomization by screening HBsAg level (3 categories: ≥0.05 to < 1 IU/mL, ≥1 to < 10 IU/mL, and ≥10 to <100 IU/mL). Subjects will complete investigational treatment with BRII-179 or placebo at Week 18; PEG-IFN-α treatment will last for up to 24 weeks after enrollment (a total of 48 weeks of PEG-IFN-α treatment before and after enrollment); while NrtI therapy will continue into the Follow-up Period. The BRII-179-002 study design schema can be found in Section 1.4.

Estimated total duration for each subject will include the following: screening up to 28 days, 24-week dosing period (BRII-179 treatment for 18 weeks and PEG-IFN-α treatment for 24 weeks), and 24-week follow-up period. Eligible subjects that meet NrtI discontinuation criteria (Section 4.1.2.1) (eligibility assessed at Week 36 to Week 48) may enter a NrtI discontinuation monitoring period for an additional 48 weeks from the day of NrtI discontinuation (Section 1.5.2).

4.1.1. Independent DSMB

An independent DSMB will monitor subject safety and treatment efficacy data for this study. The details for the operation and responsibilities of the DSMB will be defined in a separate DSMB Charter. The Charter will delineate the composition, duties, responsibilities, and procedures of the DSMB, data required at each meeting, as well as the analyses that will be conducted.

4.1.2. NrtI Discontinuation

4.1.2.1. NrtI Discontinuation Criteria

The investigator will evaluate whether subjects are eligible for discontinuation of NrtI therapy starting from Week 36 and every four weeks until Week 48 based on the

following NrtI discontinuation criteria:

- Alanine aminotransferase (ALT) < 2x ULN (results from previous visit) AND
- HBV DNA < LLOQ (results from previous visit) AND
- 2 consecutive assessments of HBsAg < 0.05 IU/mL (assessed starting from Week 28 laboratory results)

Subjects who are eligible to discontinue from NrtI therapy may discontinue from the Schedule of Activities in Section 1.5.1 and may instead continue into the NrtI Discontinuation Monitoring Period of the study. These subjects will follow the Schedule of Activities for subjects who have discontinued from the NrtI therapy (Section 1.5.2). Subjects who discontinue from their NrtI therapy will be followed in the NrtI Discontinuation Monitoring Period for an additional 48 weeks from their day of NrtI discontinuation.

4.1.2.2. NrtI Re-treatment

It is recommended that the Investigator should consider re-treatment with NrtI therapy during the NrtI Discontinuation Monitoring Period if subjects meet the following criteria at the same visit (based on Papatheodoridi, 2020):

- ALT > 15x ULN with positive HBV DNA
- ALT 10-15x ULN with positive HBV DNA: retreatment may be implemented at investigator's discretion
- ALT 5-15x ULN with elevated total bilirubin > 2 mg/dL (predominantly direct) and/or prolongation of prothrombin time (PT) ≥ 3 seconds with positive HBV DNA
- ALT 2-5x ULN and HBV DNA >2000 IU/mL persisting for ≥ 12 consecutive weeks
- ALT > 3x ULN and HBV DNA > 100,000 IU/mL at the same visit

Whether subjects are re-treated with NrtI is at the discretion of the Investigator following good medical judgement. Subjects who are re-treated with NrtI therapy should continue to be followed per the SoA Section 1.5.2 for the duration of the 48-week NrtI Discontinuation Monitoring Period.

4.1.3. PEG-IFN-α Therapy During the Dosing Period

Subjects entering the Dosing Period of the study will continue their PEG-IFN-α treatment, the investigator should monitor and medically manage relevant side effects as necessary. The dose of PEG-IFN-α is based on relevant clinical guidelines and information from the package insert which is considered as proper by the investigator. Temporary interruptions of PEG-IFN-α administration are discouraged. Subjects are to remain on treatment for the entire duration of the study until completion of Week 24 and the dose of PEG-IFN-α should not be changed during the trial unless dose reduction or discontinuation is indicated because of laboratory abnormalities/adverse events (see Section 4.1.3.1 for instructions for

dose reduction). Subjects must be counselled regarding the importance of not missing doses of PEG-IFN-α.

In the case that a subject does miss a dose(s):

- If the PEG-IFN-α injection was late by 1 to 3 days, the next dose should be administered immediately. The subsequent dose should then be administered on the next scheduled injection date.
- If the PEG-IFN-α injection was late by 4 to 7 days, subject should wait to receive the next scheduled injection (1 dose is skipped).
- If the PEG-IFN-α injection was late by >7 days, treatment can be restarted at any time by the investigator and the next dose should be on scheduled injection dates.

4.1.3.1. Dose Adjustment for adverse drug reactions

If a PEG-IFN-α dose adjustment is required due to moderate to severe adverse reactions (including clinical manifestations and/or laboratory abnormalities), the initial dose reduction should be performed with clinical consideration. However, in some cases, further dose reduction may be needed. As the adverse reactions abate, re-escalation or recovery of the initial dose of PEG-IFN-α may be considered.

Hematology:

Guidelines for PEG-IFN-α Dose Adjustment According to Hematology results

Laboratory Values	PEG-IFN-α Dose Reduction	Suspend PEG-IFN-α if
Absolute neutrophil count (ANC)	≥750/mm^3: Maintain dose<br>< 750/mm^3: Reduce dose	< 500/mm^3: Treatment should be suspended until ANC values return to more than 1000/mm^3<br>Re-institute at low dose and monitor ANC
Platelet	≥ 50.000/mm^3: Maintain dose<br>< 50.000/mm^3: Reduce to low dose	<25.000/mm^3

Liver function:

In subjects with progressive or persistent ALT increases > 10 x ULN, the dose of PEG-IFN-α should be reduced and more frequent monitoring of liver function should be performed. If ALT increases are further progressive despite dose reduction or accompanied by increased bilirubin or evidence of hepatic decompensation, therapy should be immediately discontinued. After dose reduction or interruption of PEG-IFN-α, treatment may be re-instituted at the discretion of the investigator after improvement in liver function.

Other disorders:

PEG-IFN-α may cause or aggravate neuropsychiatric, autoimmune, endocrine, ophthalmologic, and infectious disorders. Monitor closely and withdraw therapy with persistently severe or worsening signs or symptoms of the above disorders. Please see package insert for more information.

4.2. Scientific Rationale for Study Design

This is a phase 2, double-blind, randomized, placebo-controlled, parallel-group study of adding BRII-179 (VBI-2601) to existing PEG-IFN-α and NrtI therapy in patients with chronic HBV infection without cirrhosis. The study design will provide an opportunity to evaluate the ability of BRII-179 (VBI-2601) to achieve a clinically meaningful increase in functional cure (sustained loss of HBsAg in blood with or without seroconversion to HBsAb) in subjects with chronic HBV on optimized PEG-IFN-α and NrtI therapy as compared to therapy consisting of optimized PEG-IFN-α and NrtI therapy alone.

There is increasing evidence that low levels of quantitative HBsAg may improve the potential for HBsAg loss, and this is most evident in patients who lose HBsAg after stopping long-term NrtI therapy. It has been observed that patients with decreasing HBsAg and a lower HBsAg level < 100 IU/mL at the end of treatment were able to maintain control of HBV after discontinuation of long-term NrtI therapy (Chang, 2015; Yao 2017; Jeng, 2018; Papatheodoridi, 2020). Recent reporting from the Everest Project seems to corroborate this observation from previous studies. In 1427 patients on background NrtI therapy who have completed 24 to 26 weeks of PEG-IFN-α treatment and 516 patients who have completed 48 weeks of PEG-IFN-α treatment, the overall rate of HBsAg loss for patients treated ≥12, ≥24, ≥36 and ≥48 weeks was 13.00%, 16.06%, 20.49% and 23.01%, respectively. Subgroup analysis based on baseline HBsAg stratification (0.05-100, 100-500, 500-1000, 1000-1500 IU/mL) found that the HBsAg loss rate was highest in patients with baseline HBsAg between 0.05 and 100 IU/mL (34.93% for patients treated ≥ 48 weeks) (Ze-Qian, 2020).

Viral rebound may occur shortly after NrtI discontinuation in some cases, which requires close monitoring during study participation in order to control ALT flares. The HBV viral relapse after NrtI discontinuation may induce the host immune response and potentially a long-term immune control against the viral infection. Sustained HBsAg loss and/or virological response were observed in HBeAg-negative patients discontinuate NrtI therapy in prospective cohort studies (van Bömmel, 2018) as well as in a randomized controlled study (Barthel FM-S, Parmar MKB, Royston P. How do multi-stage, multi-arm trials compare to the traditional two-arm parallel group design - a reanalysis of 4 trials. Trials 2009; 10:21, doi:.10.1186/1745-6215-10-21.

Berg, 2017). This study design was designed to evaluate the potential benefit of decreased viral antigen levels in immune responses to BRII-179 treatment after discontinuation of long-term NrtI therapy.

Surrogate endpoints of HBV functional cure measure biochemical, virological, and histological outcomes. Based on current knowledge, the best surrogate of HBV functional cure is HBsAg loss confirmed on 2 occasions at least 6 months apart with or without HBsAg seroconversion plus HBV DNA below the detection offline (Choodari-Oskooei B, Parmar MKB, Royston P, Bowden J. Impact of lack-of-benefit stopping rules on treatment effect estimates of two-arm multi-stage (TAMS) trials with time to event outcome. Trials 2013, 14:23, http://www.trialsjournal.com/content/14/1/23.

Cornberg, 2020). The incidence of sustained HBsAg loss and undetectable HBV DNA will be used as primary endpoints to assess the potential of BRII-179 (VBI-2601) to induce functional cure.

The target population of this study is HBeAg-negative non-cirrhotic patients with chronic HBV infection that has been virologically suppressed (baseline HBsAg ≥ 0.05 IU/mL and < 100 IU/mL). In the Everest program, we found that some patients had been treated with PEG-IFN-α for 24-28 weeks and NrtI for 18 months and had a preliminary HBsAg decline with HBV DNA below the detection limit, however, could not achieve HBsAg loss upon completion of 48 weeks of PEG-IFN-α treatment. We believe that the immunomodulatory effects of BRII-179 as a therapeutic vaccine can further enhance HBsAg decline and ultimately achieve HBsAg loss, leading to functional cure.

4.3. Dosage Instructions

4.3.1. Justification for Dose Selection and Number of Doses

Sci-B-Vac® has been evaluated previously as a therapeutic vaccine at 20μg and 40 μg dose in multiple clinical studies in chronic HBV patients (Hoa, 2009, Lo, 2007; Roggendorf, 2019). Sci-B-Vac® was well tolerated at these higher doses. Sci-B-Vac® also demonstrated good safety profile when assessed in liver transplant patients at a high dose of 40 μg (Wong, 2018). In a Phase 1b/2a randomized, open-label, controlled study (BRII-179-001) of BRII-179 completed in multiple countries and regions in the Asia-Pacific region including China, 44 subjects with chronic hepatitis B received 40 μg BRII-179 intramuscularly every 4 weeks for a total of 4 doses and it was demonstrated that the drug was safe and well tolerated. Based on these clinical safety data, we selected 40 μg per dose as the dose administered in this study.

In Study BRII-179-001, BRII-179 every 4 weeks for a total of 4 doses elicited HBV-specific T- and B-cell responses in a subset of subjects, however, the response was significantly delayed and with shorter duration compared to that in healthy subjects, and this delayed and low immune response phenomenon coincided with the predicted chronic hepatitis B immunocompromise or tolerance. This study also showed that the intensity of HBV-specific immune responses induced by 4 doses of BRII-179 was not sufficient to achieve a significant HBsAg reduction. In order to achieve better efficacy and considering previously published data of other therapeutic vaccines, we believe that an increase in the number of doses of BRII-179 is needed. The completed preclinical long-term toxicology studies in New Zealand White rabbits with drug administered every 2 weeks for a total of

10 doses over 18 weeks can support clinical studies up to 18 weeks. In addition, dosing every 3 weeks in the AAV-HBV mouse model supported the induction and maintenance of an effective immune response based on nonclinical pharmacodynamic studies. Therefore, we plan to conduct a clinical study with BRII-179 administrated every 3 weeks for a total of 7 doses over 18 weeks to explore the possibility of HBsAg loss.

In summary, currently available safety and tolerability data of BRII-179 (VBI-2601), as well as the safety assessment from the toxicology studies, support the proposed BRII-179 (VBI-2601) treatment regimen to explore functional cure in patients with chronic HBV infection.

4.3.2. Maximum dose/exposure in this study

The maximum dose of BRII-179 is 40 µg every 3 weeks. No other dose adjustment is planned in this study.

4.4. End of Study Definition

A subject is considered to have completed the study if he/she has completed all phases of the study including the last scheduled procedure shown in the Schedule of Activities.

The end of the study is defined as the date of the last visit of the last subject in the study or last scheduled procedure shown in the Schedule of Activities for the last subject in the trial.

5. STUDY POPULATION

A total of 600 Chinese nationality subjects (approximately 120 subjects are planned to be enrolled in Phase 2a part and approximately 480 subjects are planned to be enrolled in Phase 2b part) with virologically suppressed chronic HBV infection without cirrhosis receiving PEG-IFN-α and NrtI therapy are planned for this study.

Prospective approval of protocol violations to recruitment and enrollment criteria, also known as protocol waivers or exemptions, is not permitted. Adherence to the criteria as specified in the protocol is essential.

5.1. Inclusion Criteria

Subjects eligible to be included in the study must meet all of the following criteria. All laboratory test requirements for inclusion will be performed by central laboratory unless specified otherwise.

1. A signed and dated written Informed Consent Form (ICF) prior to study participation.

2. Age ≥ 18 (or age of legal consent, whichever is older), and ≤ 60 at time of screening.

3. Documented chronic HBV infection (positive serum HBsAg for ≥ 6 months, negative HBsAb).

4. Negative HBeAg

5. HBsAg ≥ 0.05 IU/mL and < 100 IU/mL at screening.

6. HBV DNA < LLOQ at screening (central lab).

7. Must be receiving first-line NrtI (ETV or TDF or TAF) therapy for ≥ 12 months (with evidence of HBV DNA<LLOQ for ≥ 6 months, local lab results accepted) and PEG-IFN-α for 24 to 28 doses (for subjects who are not strictly administered once a week due to objective reasons such as adverse reactions, the number of treatment weeks should not exceed treatment doses by 4) of the Day 1 visit and could provide the related treatment history.

8. Female subjects must have a negative pregnancy test or confirmation of post-menopausal status. Post-menopausal status is defined as 12 months with no menses without alternative medical cause. Women of child-bearing potential (WOCBP) must have a negative serum pregnancy test at screening and a negative urine pregnancy test on Day 1, cannot be breast feeding, and must be willing to use highly effective methods of contraception 14 days before study drug administration through 12 weeks after last study drug administration.

9. Agrees to not donate blood during the duration of the study.

10. Serum ALT and AST ≤ 5x ULN at screening.

Study screening laboratory tests may be repeated once for values considered to be questionable by investigator with medical monitor approval.

5.2. Exclusion Criteria

Subjects are excluded from the study if any of the following criteria apply:

1. Any clinically significant chronic medical condition other than chronic HBV infection that, in the opinion of the investigator makes the patient unsuitable for participation in the study.

2. Any clinically significant acute condition such as fever (> 38º C) or acute respiratory illness within 7 days of first study drug administration.

3. Significant liver fibrosis or cirrhosis as defined by having a FibroScan result of > 9 kPa at screening. Unless the subject has a result of ≤9 Kpa from a FibroScan evaluation performed within 6 months prior to screening or liver biopsy performed within 1 year prior to screening that confirm the absence of Metavir F3 fibrosis or F4 cirrhosis. If a subject has had both procedures in the specified timeframes prior to screening, the most recent result should be used to determine eligibility.

4. History or evidence of drug or alcohol abuse as assessed by the Investigator, within the 12 months before screening.

5. History of intolerance to IM injection.

6. History of clinically significant chronic liver disease from any cause other than chronic HBV infection.

7. History of hepatic decompensation, including ascites, hepatic encephalopathy and/or esophageal or gastric varices.

8. Treatment by immunosuppressant (including but not limited to corticosteroids at a prednisolone equivalent dose > 20 mg/day) within 30 days before study drug administration at the Day 1 visit and throughout the duration of study participation.

9. Known history of immunological function impairment, including but not limited to:

   - Autoimmune disease (e.g., multiple sclerosis, type 1 diabetes mellitus , myasthenia gravis, Crohns disease and other inflammatory bowel diseases, celiac disease, systemic lupus erythematosus, scleroderma, including diffuse systemic form and limited cutaneous form of systemic sclerosis or CREST syndrome, systemic sclerosis, dermatomyositis polymyositis, rheumatoid arthritis, juvenile idiopathic arthritis, autoimmune thyroiditis including Hashimoto thyroiditis, Grave's or Basedow's disease, immune thrombocytopenic purpura, autoimmune hemolytic anemia, autoimmune hepatitis, psoriasis, vitiligo, vasculitis, Guillain Barré syndrome, Addison's disease, Bell's palsy, and alopecia areata); OR

- Primary immunodeficiency disorders (e.g., common variable immune deficiency, defective phagocytic cell function and neutropenia syndromes, and complement deficiency); OR

- Secondary immunodeficiency disorders (e.g., resulting from acquired immunodeficiency syndrome caused by human immunodeficiency virus (HIV) infection, solid organ transplant, and splenectomy).

10. Any HBV vaccine (licensed or experimental) prior to study enrollment and throughout the duration of study participation.

11. Immunization with attenuated vaccines (e.g., Mumps, Measles, Rubella) within 30 days before study drug administration at the Day 1 visit and throughout the duration of study participation.

12. Has received blood products or immunoglobulin within 90 days before study drug administration at the Day 1 visit or likely to require blood products during the study.

13. Participated in another clinical trial with an investigational drug or a biologic within 30 days or within 5 drug half-lives (whichever is longer) before study drug administration at the Day 1 visit. Subjects must agree not to take part in any other study at any time during their participation in this study, inclusive of the follow-up period.

14. Previous or current psychiatric condition that precludes compliance with the protocol. Specifically excluded are persons with psychoses, ongoing risk for suicide, or history of suicide attempt or gesture within the past 5 years.

15. The subject has any history of cancer requiring locoregional or systemic therapy within 5 years of randomization or has current disease. Subjects with completely surgically resected tumors with no evidence of disease for more than 3 years, or a history of low risk basal cell carcinoma will be accepted (low risk being defined as 1) location on the trunk of the body, arms, legs, cheeks, forehead, temples, scalp, neck, or chin, 2) < 2 cm in size, 3) nodular or superficial, 4) primary cancer that has not come back after treatment, 5) edge of the cancerous area is clear and smooth, and 6) not located in or around nerves).

16. Any skin abnormality or tattoo that would limit post treatment injection site assessment.

17. Known hypersensitivity or contraindication to BRII-179 (VBI-2601) or its excipients.

18. Unwilling, or unable in the opinion of the investigator, to comply with study requirements, including the use of an adequate birth control method.

19. Active infection with human immunodeficiency virus (HIV), hepatitis C virus (HCV) or hepatitis Delta virus.

20. Donated more than 500 mL of blood within 90 days before study drug administration.

21. Renal impairment with estimated glomerular filtration rate (eGFR) ≤ 60 ml/min/1.73 m^2 at screening.

22. Uncontrolled diabetes mellitus as determined by the Investigator.

23. Diagnosis of advanced stage heart failure (New York Heart Association Class III or Class IV or unstable angina).

24. Any laboratory test abnormality that is considered as clinically significant or may contradict continued treatment with PEG-IFN-α by the investigator.

5.3. Lifestyle Considerations

Alcohol consumption is limited to no more than 1 unit per day (unit: 1 glass of wine [125 mL] = 1 measure of spirits [30 mL] = one-half pint of beer [284 mL]) during the participation of this study.

5.4. Screening Failures

Screening failures are defined as subjects who consent to participate in the clinical study but are not subsequently randomly assigned to study drug. A minimal set of screening failure information is required to ensure transparent reporting of screening failure subjects to meet the Consolidated Standards of Reporting Trials (CONSORT) publishing requirements and to respond to queries from regulatory authorities. Minimal information includes demography, screen failure details, eligibility criteria, and any serious adverse event (SAE).

Individuals who do not meet the criteria for participation in this study (screening failure) may not be rescreened.

6. STUDY INTERVENTION

6.1. Study Intervention(s) Administered

Study intervention is defined as any study intervention(s), marketed product(s), placebo, or medical device(s) intended to be administered to a study participant according to the trial protocol.

Clinical study intervention drugs provided by the Sponsor will be packaged to support enrollment. Clinical study drugs will be affixed with clinical label in accordance with regulatory requirements.

6.1.1. Formulations

The following study drugs will be used and provided in this study:

- BRII-179 40μg IM injection
- Placebo IM injection
- PEG-IFN-α Subcutaneous Injection
- NrtI (either entecavir tablets, tenofovir disoproxil fumarate tablets or tenofovir alafenamide fumarate tablets. Entecavir tablet in combination with tenofovir disoproxil fumarate tablet or tenofovir alafenamide fumarate tablet may be provided if the investigator deems necessary) oral administration

BRII-179 (VBI-2601) is aluminium phosphate absorbed HBsAg Bulk Drug Substance (HBsAg spherical particles [~30 nm mean diameter] suspended in phosphate-buffered saline) and 8% Sucrose, in addition to 10 mM phosphate buffer.

Placebo will be prepared from commercially available 0.9% Sodium Chloride for Injection which will be provided to the study site. Subjects assigned to placebo will receive a volume of placebo that would be equal to the volume that would be required if that subject had been assigned to IP.

PEG-IFN-α is a colorless, clear liquid formulation of peginterferon α-2b from modified recombinant human interferon α2b expressed in yeast system with polyethylene glycol (40 kD Y type) and excipients of sodium chloride, sodium acetate, mannitol, aspartic acid, water for injection, and sodium hydroxide to adjust pH.

NrtI may be either entecavir tablets, tenofovir disoproxil fumarate tablets or tenofovir alafenamide fumarate tablets (entecavir tablet in combination with tenofovir disoproxil fumarate tablet or tenofovir alafenamide fumarate tablet may be provided if the investigator deems necessary), and the type of NrtI during treatment should be consistent with that before enrollment (e.g., if entecavir is used 3 months before enrollment, then entecavir should be continued after enrollment). Nrtl should be given orally one tablet daily and should not be discontinued or changed *ad libitum* until discontinuation according to protocol defined criteria or treatment regimen change due to drug resistance.

6.1.2. Study Intervention

Arm Name	Group 1	Group 2	Group 1 & 2	Group 1 & 2
**Intervention Name**	BRII-179 (VBI-2601)	Placebo	PEG-IFN-α	NrtI (entecavir tablets or tenofovir disoproxil fumarate tablets or tenofovir alafenamide fumarate tablets)
**Type**	Drug	Placebo	Drug	Drug
**Dose Formulation**	Solution for Injection	0.9% Sodium Chloride for Injection	Injection	Tablet
**Dose Level(s)**	BRII-179 40 µg	Placebo	180 µg	0.5mg/300mg/25mg
**Route of Administration**	BRII-179 in one syringe; administer via IM injection<br><br>Refer to the Pharmacy Manual for the details.	Placebo in one syringe; administer via IM injection<br><br>Refer to the Pharmacy Manual for the details.	1 syringe; Subcutaneous Injection<br><br>Refer to the Pharmacy Manual for the details.	1 tablet for each NrtI, oral<br><br>Refer to the Pharmacy Manual for the details.
**Use**	Experimental	Experimental	Experimental	Experimental
**IMP and NIMP**	IMP	IMP	IMP	NIMP
**Sourcing**	Provided centrally by the Sponsor	Provided centrally by the Sponsor	Provided centrally by the Sponsor	Provided centrally by the Sponsor
**Packaging and Labelling**	IMP will be labelled as required per country requirement.	Placebo will be prepared and labelled as instructed in the Pharmacy Manual	IMP will be labelled as required per country requirement.	NIMP will be labelled as required per country requirement.

On dosing days, the pharmacist or designee will prepare the required amount of study drugs into syringe for injection. A qualified clinical investigative site staff member under the supervision of the investigator or designee will administer the study drug. The injection site will be marked and mapped for later observation and should be documented. If a local reaction around the injection site occurs, photographs may be obtained. Please refer to Section 6.6 for further details.

6.2. Preparation/Handling/Storage/Accountability

- Only subjects enrolled in the study may receive study drugs and only authorized site staff may supply or administer study drugs. All study drugs must be stored in a secure, environmentally controlled, and monitored (manual or automated) area in accordance with the labelled storage conditions with access limited to the investigator and authorized site staff.
- The investigator, institution, or the head of the medical institution (where applicable) is responsible for study drug accountability, reconciliation, and record maintenance (i.e., receipt, reconciliation, and final disposition records).
- Further guidance and information for the final disposition of unused study drugs are provided in the Pharmacy Manual.

6.3. Randomization and Blinding

Subjects will be assigned to study treatment in accordance with the randomization schedule. The randomization code will be generated by using a validated computerized system. Subjects will be randomized using an Interactive Web Response System (IWRS). Once a randomization number is assigned to a subject, it cannot be reassigned to any other subject in the study. Study intervention will be dispensed at the study visits summarized in SoA (Section 1.5). Returned study intervention should not be re-dispensed to the subjects.

Study drug will be double-blind. Neither the subject nor the study physician will know which study drug the subject is receiving. The investigator or treating physician may unblind a subject’s treatment assignment **only in the case of an emergency** when knowledge of the study treatment is essential for the appropriate clinical management or welfare of the subject. Whenever possible, the investigator must first discuss options with the Medical Monitor or appropriate sponsor study personnel before unblinding the subject’s treatment assignment. If this is impractical, the investigator must notify the sponsor as soon as possible, but without revealing the treatment assignment of the unblinded subject, unless that information is important for the safety of subjects currently in the study. The date and reason for the unblinding must be recorded in the appropriate data collection tool.

Sponsor clinical safety and pharmacovigilance staff may unblind the treatment assignment for any subject with an SAE. If the SAE requires that an expedited regulatory report be sent to one or more regulatory agencies, a copy of the report, identifying the subject’s treatment assignment, may be sent to clinical investigators in accordance with local regulations and/or sponsor policy.

The primary reason for treatment discontinuation (the event or condition that led to the unblinding) will be recorded in the eCRF. Subject may continue in the follow-up period of the study once the treatment code is unblinded (see Section 7.1 Discontinuation of Study Intervention).

Subjects will receive study drugs directly from the investigator or designee, under medical supervision. The date and time of each dose administered in the clinic will be recorded in the source documents and recorded in the eCRF. The dose of study drug and study subject identification will be confirmed at the time of dosing by a member of the study site staff other than the person administering the study drug.

6.4. Compliance of Study Intervention

In the event of an interruption of study drug treatment ≥ 2 weeks as specified in the protocol, discussion between the investigator and the Sponsor is required, and the decision on subject management should be documented in writing.

Subjects will be instructed to bring NrtI and PEG-IFN-α (including all empty containers) to the trial site at every visit, as applicable, beginning with the screening visit. Adherence will be assessed by counting of empty containers and unused medication.

Every effort should be made to ensure good adherence with NrtI and PEG-IFN-α therapy. Adherence to NrtI and PEG-IFN-α should be assessed at every relevant visit. Adherence of PEG-IFN-α will be assessed by counting of empty containers and unused medication. Subjects will be instructed to bring all empty containers of PEG-IFN-α to the trial site at every visit, as applicable, for adherence assessment. Adherence of NrtI will be assessed via review of the information on the diary cards filled in by the subjects.

6.5. Concomitant Therapy

The study will collect all prescription and over-the-counter medications (including traditional Chinese medicine and herbal medicine) that the subject receives from screening and throughout the study.

Any medications that the subject is receiving at the time of enrolment or receives during the study must be recorded along with:

- Reason for use
- Dates of administration including start and end dates
- Dosage information including dose and frequency

Should subjects have a need to initiate treatment with any excluded concomitant medication, the Medical Monitor must be consulted prior to initiation of the new medication. In instances where an excluded medication is initiated prior to discussion with the Sponsor, the investigator must notify the Sponsor as soon as he/she is aware of the use

of the excluded medication. The Medical Monitor should be contacted if there are any questions regarding concomitant or prior therapy.

6.5.1. Prohibited Medications

The following medications are excluded while subjects are participating in the study.

Prior to study enrollment and throughout the duration of study participation:

- Any HBV vaccine (licensed or experimental)

Within 30 days before study drug administration at the Day 1 visit and throughout the duration of study participation:

- Immunosuppressant (including but not limited to corticosteroids at a prednisolone equivalent dose > 20 mg/day)
- Immunization with attenuated vaccines (e.g., Mumps, Measles, Rubella), except COVID vaccines
- Any investigational drug or biologic other than the study product(s) used during this study
- Any herbal remedies that may cause fluctuations of liver test results

Within 90 days before study drug administration at the Day 1 visit and throughout the duration of study participation:

- Blood products or immunoglobin

6.6. Safety Follow-up and Management of Reactions to Study Drug

The following safety observation procedures will be performed immediately following study drug administration and during the follow-up period for all subjects:

- Subjects will remain in the clinic for at least one hour post study drug administration. The observation period will include an assessment for rare adverse reactions.
- Any unusual signs or symptoms reported during the initial one hour of observation will prompt continued close monitoring.
- Based on their condition, subjects may be asked to remain in the clinic for more than one hour after the study drug administration (reason will be recorded in source data).
- All data (including assessment of solicited local and systemic reactions) will be recorded in the source document during and after the post-observation period.

A measurement device template (in mm) for measuring solicited local reactions (erythema [redness] and swelling), and an oral thermometer for measuring daily temperature, will be provided at the first study drug administration visit, study Day 1. Subjects will be advised to record their temperature in the diary card.

Refer to Sections 8.3 and 10.3 for further details on the recording, assessment, and reporting of AEs.

6.7. Treatment of Study Intervention Overdose

An overdose is defined as an accidental or intentional administration of a quantity of a medicinal product given per administration or cumulatively which is above the maximum recommended dose as per protocol. In the event of an overdose of study medication, the investigator should use clinical judgement in treating the overdose and contact the study medical monitor.

No specific treatment guidelines for overdose and toxicity management are provided by the Sponsor. The investigator is advised to refer to the relevant document(s) for detailed information pertaining to the study drug being used in this study. Such documents may include, but not be limited to, the IB or equivalent document provided by the Sponsor.

6.8. Intervention after the End of the Study

The Sponsor will not provide post-study treatment. The investigator is responsible for ensuring that consideration has been given to the post-study care of the subject’s medical condition.

7. DISCONTINUATION OF STUDY INTERVENTION AND SUBJECT DISCONTINUATION/WITHDRAWAL

7.1. Discontinuation of Study Intervention

Discontinuation of study intervention does not mean withdrawal from the study.

If a subject discontinues both investigational products (BRII-179 or placebo) and PEG-IFN-α treatment, the subject will be encouraged to continue to attend the required study visits and procedures for the remaining duration of study participation in Follow-up Period.

Subjects who have discontinued study drugs may be reassessed for possible re-institution of study drug dosing not to exceed the protocol defined number of doses, dosing interval, or dosing period duration. Subjects who have discontinued study drugs will be assessed for NrtI discontinuation as described in Section 4.1.2.1 upon continued study participation into the Follow-up Period. If NrtI discontinuation criteria are met, 48-week NrtI discontinuation monitoring should be completed according to the protocol.

If continued study participation is not possible or acceptable to the subject or investigator, please refer to Section 7.2 Subject Discontinuation/Withdrawal from the Study.

7.1.1. Criteria for Discontinuation of Study Intervention

Subjects who have received at least one dose of BRII-179 (VBI-2601) will have additional doses withheld but continue to undergo follow-up monitoring in the study if any of the following criterion is met:

- Discontinuation of study intervention requested by the subject or the subject's legal representative.
- Signs or symptoms of severe (NCI-CTCAE Grade 3 or higher) hypersensitivity or anaphylaxis
- Significant abnormalities in liver chemistry, defined as any of the following are met:
  - Serum ALT > 10 × ULN
  - Serum ALT or AST > 3 × ULN with a concomitant total bilirubin > 2 × ULN or INR > 1.5
- Any clinical manifestations of hepatic decompensation

- The Sponsor may request DSMB evaluation of specific data in the event of liver flare to fully characterize transaminase elevations. The subject may receive remaining doses of study drug only after DSMB approval.
- The subject has a medical condition or personal condition that, in the opinion of the investigator and/or the Sponsor, would pose an unnecessary risk to the subject if the investigational intervention is continued.
- The subject's serum pregnancy test is confirmed to be positive.

7.2. Subject Discontinuation/Withdrawal from the Study

- A subject may withdraw from the study at any time at his/her own request or may be withdrawn at any time at the discretion of the investigator for safety, behavioral, compliance, or administrative reasons. This is expected to be uncommon.
- At the time of discontinuing from the study, if possible, an Early Termination Visit should be performed (Section 8.1.7) for data to be collected at the time of study discontinuation and follow-up. The subject will be permanently discontinued from both the study intervention and from the study at that time.
- If the subject withdraws consent for disclosure of future information, the sponsor may retain and continue to use any data collected before such a withdrawal of consent.
- If a subject withdraws from the study, he/she may request destruction of any samples taken and not tested, and the investigator must document this in the site study records.
- Subjects who are withdrawn from the study will NOT be replaced and cannot be rescreened.
- The primary reason for subject withdrawal will be recorded in the eCRF. Primary reasons for withdrawal will be categorized as:
    - Adverse event
    - Protocol deviation
    - Withdrew consent, not due to an AE
    - Lost to follow up
    - Study closed/terminated

7.2.1. Lost to Follow up

A subject will be considered lost to follow-up if he or she repeatedly fails to return for scheduled visits and is unable to be contacted by the study site.

The following actions must be taken if a subject fails to return to the clinic for a required study visit:

- The site must attempt to contact the subject and reschedule the missed visit as soon as possible and counsel the subject on the importance of maintaining the assigned visit schedule and ascertain whether or not the subject wishes to and/or should continue in the study.
- Before a subject is deemed lost to follow up, the investigator or designee must make every effort to regain contact with the subject (where possible, 3 telephone calls and, if necessary, a certified letter to the subject's last known mailing address or local equivalent methods). These contact attempts should be documented in the subject's medical record.
- Should the subject continue to be unreachable, only then will he/she be considered to have withdrawn from the study with a primary reason of "Lost to Follow-up".

Discontinuation of specific sites or of the study as a whole are handled as part of Section 10.1.7, Appendix 1.

8. STUDY ASSESSMENTS AND PROCEDURES

8.1. Schedule of Assessments

Study procedures and their timing are summarized in the SoAs (Section 1.5). Adherence to the study design requirements, including those specified in the SoAs, is essential and required for study conduct. Protocol waivers or exemptions are not allowed. Immediate safety concerns should be discussed with the sponsor immediately upon occurrence or awareness to determine if the subject should continue or discontinue study drug.

8.1.1. Screening

Written informed consent must be obtained prior to initiation of any study procedures. All screening evaluations must be completed and reviewed to confirm that potential subjects meet all eligibility criteria. The investigator will maintain a screening log to record details of all subjects screened and to confirm eligibility or record reasons for screening failure.

Screening will be performed no more than 4 weeks prior to randomization and will include written consent, determination of subject eligibility, collection of demographics and medical history/ current medical conditions as well as a physical examination, vital signs, 12-lead ECG, clinical laboratory tests, and other assessments per the SoA (Section 1.5).

Adverse events will be collected from the time of signing the consent until last visit. After informed consent has been obtained but prior to initiation of study drug, untoward medical occurrence, if related to study procedure, will be recorded as AE; otherwise, will be recorded as medical history. All SAEs must be collected from the time of consent until end of study.

8.1.2. Randomization (Day 1)

Eligible subjects will be randomized 1:1 to either 40 µg of BRII-179 administrated IM or placebo administrated IM. Subjects will receive their first dose of study drug on Day 1, however subject eligibility must be confirmed prior to study drug administration and blood sample collections will be made prior to study drug administration. Other assessments to be conducted on Day 1 are detailed in the SoA (Section 1.5).

8.1.3. Dosing Period

All subjects will receive BRII-179 (VBI-2601) or placebo administered IM every 3 weeks for a total of 7 doses. Subjects will receive PEG-IFN-α at the indicated dose level weekly and discontinue investigational treatment with BRII-179 or placebo at Week 18 and discontinue PEG-IFN- α at Week 24; while NrtI therapy will continue into the Follow-up Period.

Subjects will return to the study site clinic on an outpatient basis for antiviral activity monitoring and safety assessment as specified in the SoA (Section 1.5).

Prior to study drug administration, there will be a review of ongoing eligibility to receive the study drug and continue with study participation. Diary cards will be collected and

there will be a review of any local reaction (including redness, swelling, pain, itchiness and tenderness, etc.) and systemic reaction (tiredness, diarrhea, nausea/vomiting, headache, muscle pain, etc,) related to BRII-179 (VBI-2601) or placebo administration. Blood samples will be collected prior to each study drug administration.

8.1.4. Follow-up Period

Subjects will return to the investigative site for the specified assessments per the SoA (Section 1.5), including but not limited to physical examination, vital signs, clinical laboratory tests, and review of adverse events and concomitant medications.

Subjects will be assessed by the investigator whether they are eligible for discontinuation of NrtI therapy starting at Week 36 and every four weeks until Week 48. Subjects that have NOT achieved NrtI discontinuation criteria by Week 48 will be discharged from the study.

8.1.5. NrtI Discontinuation Monitoring Period

Eligible subjects who meet NrtI discontinuation criteria will be informed by the investigator to discontinue their NrtI medications on the first day of their scheduled Day 1 visit of the NrtI Discontinuation Monitoring Period. These subjects will follow the NrtI Discontinuation Monitoring Period SoA (Section 1.5.2).

Subjects who discontinue from their NrtI therapy will be followed in the NrtI Discontinuation Monitoring Period for an additional 48 weeks from the day of NrtI discontinuation. Subjects who are retreated with NrtI therapy should continue to be followed per SoA (Section 1.5.2) for the duration of the 48-week NrtI Discontinuation Monitoring Period.

8.1.6. Unscheduled Visits

Subjects may be asked to come for an unscheduled visit, for clinical assessment of an adverse event for example, if warranted, as determined by the investigator. The unscheduled visit will be recorded in the eCRF.

8.1.7. Early Termination Visit

In cases where a subject prematurely discontinues from the study, the investigator must make every effort to have the subject return to the study site within 14 days for an Early Termination (ET) Visit. Assessments for ET visits will include but not limited to physical examination, pregnancy test, clinical laboratory tests, and other assessments per the SoA (Section 1.5). A follow-up visit will be conducted approximately 4 weeks after the last dose of study drug.

8.2. Detailed Description of Study Procedures

8.2.1. Medical History

A complete medical history, including details regarding illnesses and allergies, date(s) of onset, and whether condition(s) is currently ongoing (current medical conditions), medication history, including prior HBV treatment history and HBV genotype history (if available), will be collected on all subjects during screening.

8.2.2. Physical Examinations

A complete physical examination will include, at a minimum, general appearance, head, neck, chest/respiratory, heart/cardiovascular, gastrointestinal/liver, extremities, skin, and neurological assessments. Height and weight will also be measured and recorded. Body mass index will be calculated from height and weight.

A symptom-directed physical examination will be performed according to investigator discretion.

8.2.3. FibroScan

To exclude the presence of significant liver fibrosis and cirrhosis, subjects will have a FibroScan evaluation during screening. Subject with a FibroScan result of ≤ 9 kPa at screening can be considered without significant liver fibrosis and cirrhosis. If screening FirboScan result > 9 kPa, however, the subject has a result of ≤ 9 Kpa from a FibroScan evaluation performed within 6 months prior to screening or liver biopsy performed within 1 year prior to screening that confirm the absence of Metavir F3 fibrosis or F4 cirrhosis, significant liver fibrosis and cirrhosis can be excluded as well. If a subject has had both procedures in the specified timeframes prior to screening, the most recent result should be used to determine eligibility.

A FibroScan will also be conducted at the Week 48/ ET3 Visit in the NrtI Discontinuation Monitoring Period.

8.2.4. Abdominal Ultrasound

Abdominal Ultrasound examinations will be performed as outlined in SoA (Section 1.5).

8.2.5. Alcohol Intake Assessment

Alcohol intake during the study will be recorded.

8.2.6. Vital Signs

Temperature (oral preferred), pulse rate, respiratory rate, and blood pressure will be assessed after the subject has rested comfortably for approximately 5 minutes. Pulse rate and blood pressure measurements will be assessed with a completely automated device. Manual techniques will be used only if an automated device is not available. Blood pressure

should be based on 2 separate measurements. When scheduled at the same timepoints, vital signs and 12-lead ECG must be assessed first before physical examinations, blood sample collections, and urine collection.

8.2.7. Electrocardiograms

12-lead ECGs will be recorded and reviewed on-site by the investigator as outlined in SoA (Section 1.5).

8.2.8. Laboratory Assessments

8.2.8.1. Screening Viral Serology

The screening viral serology parameters will include chronic HBV infection and active infection with HIV, HCV, and hepatitis Delta virus. Chronic HBV infection is defined as serum HBsAg ≥ 6 months.

8.2.8.2. Clinical Safety Laboratory Assessments

Fasting clinical safety laboratory tests to be performed are described in Section 10.2.

The investigator must review the laboratory report, document this review, and record any clinically significant changes occurring during the study in the AE section of the eCRF. The laboratory reports must be filed with the source documents. Clinically significant abnormal laboratory findings are those which are not associated with the underlying disease, unless judged by the investigator to be more severe than expected for the subject's condition.

In the event of an unexplained clinically relevant abnormal laboratory test occurring after study drug administration, the test should be repeated and followed at investigator discretion until it has returned to normal or baseline, and/or a diagnosis is made to adequately explain the abnormality.

8.2.8.3. Pregnancy Test

A pregnancy test or confirmation of post-menopausal status must be confirmed for all female subjects. Post-menopausal status is defined as 12 months with no menses without alternative medical cause. Pregnancy tests will be performed for WOCBP only. Serum and urine pregnancy testing will be performed per the SoAs (Section 1.5) and any time pregnancy is suspected. A WOCBP who is known to be pregnant or who does not have a negative pregnancy test at screening is not eligible for study participation. During the study, the results of these pregnancy tests must be known prior to study drug administration. A WOCBP determined to be pregnant while on study will be followed until the pregnancy outcome is known, as described in Section 8.3.5.1.

8.2.8.4. Assessment of Antiviral Activity

Assessment of antiviral activity will include HBsAg, HBsAb, and HBV DNA.

Details regarding the processing, shipping, and analysis of the samples will be provided in the Laboratory Manual.

8.2.9. Laboratory Events of Clinical Interest

A clinical laboratory event of clinical interest (ECI) is a laboratory value of scientific and medical interest specific to understanding of the investigational product and may require additional follow-up testing, close monitoring, and rapid communication by the investigator to the Sponsor.

From Day 1 of dosing to the last visit, any laboratory events of ECI will be monitored, defined as the following:

- ALT > 3x ULN, if Day 1 pre-dose ALT ≤ ULN
- ALT > 2x baseline, if Day 1 pre-dose ALT > ULN

All laboratory ECIs must be reported to the Sponsor within 24 hours of the investigator's knowledge of the event. See Section 10.3.5. In addition, the assessments outlined in Section 10.5 should be performed as soon as possible and no later than two weeks after the initial ALT value meeting criteria for a laboratory ECI.

The laboratory ECI will also be evaluated by the investigator to determine if the event meets criteria for an AE. If AE criteria are met, all clinical sequelae in relation to these laboratory ECIs will be reported as AEs or SAEs, if applicable.

8.3. Adverse Events and Serious Adverse Events

The investigator and any qualified designees are responsible for detecting, documenting, and recording events that meet the definition of an AE or SAE and remain responsible for following up AEs that are serious, considered related to the study drug or study procedures, or that caused the subject to discontinue the study drug or study.

The definitions and procedures for reporting, recording, evaluating, and follow-up of an AE or SAE can be found in Section 10.3, Appendix 3.

8.3.1. Time Period and Frequency for Collecting AE and SAE Information

All SAEs will be collected from the signing of the informed consent form (ICF) until the last visit at the time points specified in the SoAs (Section 1.5).

All AEs will be collected from the signing the ICF until the last visit at the time points specified in the SoAs (Section 1.5).

After informed consent has been obtained but prior to initiation of study drug, untoward medical occurrence, if related to study procedure, should be recorded as AE; otherwise, should be recorded as medical history.

All SAEs will be recorded and reported to the sponsor or designee immediately and under no circumstance should this exceed 24 hours, as indicated in Section 10.3, Appendix 3. The investigator will submit any updated SAE data to the sponsor within 24 hours of it being available.

Investigators are not obligated to actively seek AEs or SAEs after study participation. However, if the investigator learns of any SAE, including a death, at any time after a subject has been discharged from the study, and he/she considers the event to be reasonably related to the study drug or study participation, the investigator must promptly notify the sponsor.

8.3.2. Method of Detecting AEs and SAEs

The method of recording, evaluating, and assessing causality of AEs and SAEs and the procedures for completing and transmitting SAE reports are provided in Section 10.3.

Care will be taken not to introduce bias when detecting AEs and/or SAEs. Open-ended and non-leading verbal questioning of the subject is the preferred method to inquire about AE occurrences.

8.3.3. Follow-up of AEs and SAEs

After the initial AE/SAE report, the investigator is required to proactively follow each subject at subsequent visits/contacts. All SAEs will be followed until resolution, until the condition stabilizes, until the event is otherwise explained, or until the subject is lost to follow-up (as defined in Section 7.2). Further information on follow-up procedures is provided in Section 10.3, Appendix 3.

8.3.4. Regulatory Reporting Requirements for SAEs

- Prompt notification by the investigator to the sponsor of a SAE is essential so that legal obligations and ethical responsibilities towards the safety of subjects and the safety of a study drug under clinical investigation are met.
- The sponsor has a legal responsibility to notify both the local regulatory authority and other regulatory agencies about the safety of a study drug under clinical investigation. The sponsor will comply with country-specific regulatory requirements relating to safety reporting to the regulatory authority, Institutional Review Boards (IRB)/Independent Ethics Committees (IEC), and investigators.
- For all studies except those utilizing medical devices investigator safety reports must be prepared for suspected unexpected serious adverse reactions (SUSAR) according to local regulatory requirements and sponsor policy and forwarded to investigators as necessary.
- An investigator who receives an investigator safety report describing a SAE or other specific safety information (e.g., summary or listing of SAEs) from the sponsor will review and then file it along with the Investigator's Brochure and will notify the IRB/IEC, if appropriate according to local requirements.

8.3.5. Special Situations

Special situation reports include all reports of medication error, abuse, misuse, overdose, reports of adverse events associated with product complaints, and pregnancy reports (Including pregnancies in female subjects and pregnancies in female partners of male subjects) regardless of an associated AE.

Medication error is any unintentional error in the prescribing, dispensing, or administration of a medicinal product while in the control of the health care provider, subject, or consumer.

Abuse is defined as persistent or sporadic intentional excessive use of the medicinal product by a subject.

Misuse is defined as any intentional and inappropriate use of a medicinal product that is not in accordance with the protocol instructions or the local prescribing information.

An overdose is defined as an accidental or intentional administration of a quantity of a medicinal product given per administration or cumulatively which is above the maximum recommended dose as per protocol. In cases of a discrepancy in drug accountability, overdose will be established only when it is clear that the subject has taken the excess dose(s). Overdose cannot be established when the pharmacist cannot account for the discrepancy except in cases in which the investigator has reason to suspect that the additional dose(s) were administered to the subject.

Product complaint is defined as complaints arising from potential deviations in the manufacture, packaging, or distribution of the medicinal product.

8.3.5.1. Pregnancy

- Details of all pregnancies in female subjects will be collected after the start of study drug through 12 weeks after last study drug administration.
- If a pregnancy is reported, the investigator should inform the sponsor within 24 hours of learning of the pregnancy and should follow the procedures outlined in Section 10.4.
- Abnormal pregnancy outcomes (e.g., spontaneous abortion, fetal death, stillbirth, congenital anomalies, ectopic pregnancy) are considered SAEs.

8.3.5.2. Other Special Situations

- All other special situations must be reported to the sponsor within 24 hours of the investigator becoming aware of the situation. These reports apply to study drug only.
- Special situations involving medications other than study drug do not need to be reported; however, for special situations that result in AEs due to a medication other than study drug, the AE should be reported.
- Any inappropriate use of concomitant medications prohibited by this protocol should not be reported as “misuse,” but may be more appropriately documented as a protocol deviation.

- All clinical results in relation to these special situation reports will be reported as AEs or SAEs at the same time. Details of the symptoms and signs, clinical management, and outcome will be reported, when available.

8.4. Pharmacodynamics

Pharmacodynamic parameters are not evaluated in this study.

8.5. Genetics

Human genetics are not evaluated in this study.

8.6. [Health Economics] OR [Medical Resource Utilization and Health Economics]

Health Economics/Medical Resource Utilization and Health Economics parameters are not evaluated in this study.

9. STATISTICAL CONSIDERATIONS

9.1. Statistical Hypotheses

9.1.1. Phase 2a Part

The primary endpoint for the Phase 2a part is percentage of subjects with HBsAg loss at completion of 24-week PEG-IFN-α treatment. The null hypothesis is that there is no difference in the percentage of HBsAg loss at completion of 24-week PEG-IFN-α treatment between BRII-179 + PEG-IFN-α + NrtI and BRII-179 placebo + PEG-IFN- α +NrtI groups.

9.1.2. Phase 2b Part

The primary endpoint for the Phase 2b part is sustained HBsAg and HBV DNA loss (defined as HBsAg and HBV DNA undetectable for at least 24 weeks) after NrtI discontinuation. The null hypothesis is that there is no difference in the percentage of sustained HBsAg and HBV DNA loss between BRII-179+PEG-IFN- α + NrtI and BRII-179 placebo + PEG-IFN- α + NrtI groups.

9.2. Sample Size Determination

9.2.1. Phase 2a Part

The early futility evaluation based on an intermediate outcome of efficacy using the HBsAg loss at completion of 24-week PEG-IFN-α treatment will be conducted when approximately 120 subjects complete 18 weeks BRII-179 or placebo investigational treatment and 24 weeks PEG-IFN- α treatment or withdraw the study prior to last scheduled PEG-IFN-α treatment.

Everest project data suggest 25% subjects can achieve HBsAg loss after 24 weeks treatment of PEG-IFN- α + NrtI. The 20% difference in percentage of HBsAg loss is considered to be a clinically meaningful increase in HBsAg clearance with the addition of BRII-179 to PEG-IFN-α and NrtI therapy, based on this point, with the assumption of a 25% of HBsAg loss by placebo in combination with PEG-IFN-α and NrtI treatment, and a 45% of HBsAg loss by BRII-179 in combination with PEG-IFN-α and NrtI treatment, a sample size of 120 evaluable subjects in an one-sided Type I error of $\alpha = 0.3$ can provide a power of more than 95% to reject the null hypothesis. In this sample size estimation, power calculation method is normal approximation and test method is Z-test (SAS9.4).

9.2.2. Phase 2b Part

Currently, there are limited data available regarding sustained HBsAg loss and sustained HBV DNA undetectable (defined as HBsAg and HBV DNA undetectable for at least 24 weeks) after a total of 48 weeks treatment of PEG-IFN- α in planned target population in this study. Table 1 displays a range of sample size for sustained HBsAg and HBV DNA loss with PEG-IFN-α + NrtI treatment ranging from 22% to 35% and a minimum of clinically meaningful increase of 15% after adding BRII-179 to PEG-IFN- α + NrtI treatment.

Table 1. Sample Size Calculation for Four Scenarios of Sustained HBsAg Loss

Expected Sustained HBsAg loss and HBV DNA undetectable (%)		Total Sample Size (Per Arm)	Power	Alpha (1-sided)
Placebo + PEG-IFN- α + NrtI	BRII-179 + PEG-IFN-α + NrtI			
22	37	412 (206)	90%	0.025
26	41	442 (221)		
**30**	**45**	**466 (233)**		
35	50	486 (243)		

Based on the following assumptions, a 15% increase in the percentage of sustained HBsAg loss and HBV DNA undetectable at a one-sided Type I error level of 2.5%. It is assumed 466 subjects enrolled will provide 90% power to reject the null hypothesis, further decided to enrol a total of 480 subjects, which will provide with approximately 91% power to reject the null hypothesis.

Proportion of sustained HBsAg and HBV DNA loss in PEG-IFN- α + NrtI group is 30%.

A clinically meaningful increase in sustained HBsAg loss is 15% with add-on of BRII-179 treatment.

Non-binding stopping guideline for futility using a moderately aggressive Type II error spending function (Gamma=-2), implemented using the Lan-DeMets O'Brien-Fleming approximation spending function approach.

The sample size calculation will be performed using proc seqdesign in SAS9.4 and it is planned to perform 3 interim analysis and 1 final analysis.

Data analyses will be performed when 120 subjects enrolled in Phase 2a part complete the last scheduled PEG-IFN-α treatment or withdraw from the study prior to last scheduled PEG-IFN-α treatment.

The sample size for Phase 2b part may be re-estimated after evaluation of efficacy data from subjects in Phase 2a part.

9.3. Data Monitoring Guidelines for Independent DSMB

An independent DSMB will review the safety and efficacy data of Phase 2a part of the study. Based on the results of the Phase 2a part, the DSMB will build the rationale for the recommendation on whether to proceed to Phase 2b part or not. DSMB will perform data review when approximately 120 subjects enrolled in the Phase 2a part complete the last scheduled PEG-IFN-α treatment and HBsAg and HBV DNA results are available or withdraw from the study prior to last scheduled PEG-IFN-α treatment.

If the trial proceeds to Phase 2b part, the DSMB will continue to review interim data on a regular basis and use pre-specified guidelines to determine clear evidence of efficacy for the primary outcome, and if so the DSMB will provide recommendation to unblind the trial results. Conversely, the DSMB may provide recommendation to discontinue the BRII-179 treatment if futility assessments indicate that there is low probability to achieve statistical significance for the primary endpoint of sustained HBsAg loss with BRII-179 treatment.

9.4. Primary Endpoint and Safety Data Analysis in Phase 2 Part

An early futility analysis based on HBsAg loss will be performed on approximately 120 subjects who complete last scheduled PEG-IFN-α treatment or withdraw from the trial prior to last scheduled PEG-IFN-α treatment. The sample size of 120 subjects provides above 95% power to reject the null hypothesis at the 0.30 (1-sided) level of significance.

Brii will make the decision on whether to proceed to Phase 2b part or not based on HBsAg loss futility analysis result at completion of last scheduled PEG-IFN-α treatment and safety data analysis, as well as the recommendation provided by the independent DSMB.

9.4.1. Monitoring Guidelines for the Primary Endpoint in Phase 2b Part

As a guideline, stopping boundaries will be provided to the DSMB to monitor the primary endpoint (sustained HBsAg loss and sustained HBV DNA undetectable). For monitoring overwhelming benefit, the Lan-DeMets spending function analogue of the O'Brien-Fleming boundaries will be used. The Lan-DeMets boundary used will be chosen to preserve a 1-sided 0.025 level of significance. For computing the Lan-DeMets boundary, the information fraction at each of 3 interim analyses will be the number of subjects enrolled at the interim analysis, i.e., 120, 240, and 360 subjects who complete 48 weeks of follow-up or withdraw before week 48 (for those who do not enter NrtI withdrawal follow-up period) or complete at least 24 weeks of follow-up or withdraw from the study prior to at least 24 weeks of follow-up after NrtI discontinuation (for those who enter NrtI discontinuation follow-up period), respectively.

Futility analyses will be performed. The aim of these analyses will be to consider whether BRII-179 should be discontinued due to a low probability of achieving statistical significance for the primary endpoint of sustained HBsAg loss. Conditional power calculations of sustained HBsAg loss will be presented. Typical futility guidelines recommend stopping a trial when conditional power is below 10%-15%These analyses will be presented to the DSMB by the unblinded statisticians.

9.5. Analysis Sets

The following analysis sets are defined:

Population	Description
All Subjects Screened	All subjects, for whom a record exists on the clinical database, will be included in All Subjects Screened.<br>It will be used for reporting study disposition, inclusion and exclusion criteria deviations, etc.
All Subjects Randomized	All subjects, who are randomized will be included in All Subjects Randomized. It will be used for reporting demographics, baseline characteristics, medical history, etc.
Full Analysis Set	All subjects, who are randomized and received at least one dose of BRII-179 or placebo, will be included in Full Analysis Set. It will be used as the primary analysis set of all efficacy endpoints.
Per Protocol Set	All subjects, who are randomized; and not identified with major protocol deviation that substantially affects the treatment effect of study medication; received all planned doses of BRII-179 or placebo; will be included in Per Protocol Set. It will be used as analysis set for key efficacy endpoint analyses. The analysis based on Per Protocol analysis set will be considered as sensitivity analysis.
Safety Analysis Set	All subjects randomly assigned to study drug and who take at least 1 dose of study drug will be included in Safety Analysis Set. It will be used for reporting safety parameters.

9.6. Statistical Analyses

This section outlines the general analytic strategy for the study. The statistical analysis plan (SAP) will be finalized prior to Database Lock (DBL) and it will include a more detailed description of the statistical analyses described in this section. This section is a summary of the planned statistical analyses of the most important endpoints including primary and key secondary endpoints.

9.6.1. General considerations

Unless otherwise stated, the following rule will apply:

Summaries and displays will be presented by treatment group.

For categorical variables, the frequency and percentage will be applied as descriptive statistics.

For continuous variables, the mean, standard variation, median, inter-quartiles, and ranges will be applied as descriptive statistics.

Specific algorithm regarding how missing HBsAg and HBV DNA values will be handled in deriving the primary efficacy endpoints and sustained HBsAg loss will be detailed in the statistical analysis plan.

Unless specified otherwise, missing values will be handled using the Data-As-Observed (DAO) approach, that is, any missing value will be excluded from the analysis, except for the baseline value, which will be replaced with the latest pre-treatment result, if available.

Only scheduled visits will be presented of summaries; unscheduled visits will be listed but will not be summarized.

9.6.2. Analysis of the Primary Efficacy Endpoint for Phase 2a Part

The primary efficacy endpoint of the phase 2a part, percentage of HBsAg loss at completion of PEG-IFN-α treatment, will be analysed based on Cochran-Mantel-Haenszel method, stratified at randomization by screening HBsAg level (3 categories). The differences in the percentage of HBsAg loss and associated 95% confidence interval will be presented. If too few cases in a stratum result in failure of stratified analysis, it will be considered to remove the stratification factors and a non-stratified analysis or Fisher's exact probability method will be used to compare the percentage of HBsAg loss between the two groups.

9.6.3. Analysis of the Primary Efficacy Endpoint for Phase 2b Part

The primary efficacy endpoint of the phase 2b part, percentage of sustained HBsAg loss and sustained HBV DNA undetectable defined as undetectable HBsAg and HBV DNA for at least 24 weeks after NrtI discontinuation. Subjects who do not meet NrtI withdrawal criteria or are retreated with NrtI after NrtI discontinuation will be classified as not achieving sustained HBsAg loss and HBV DNA undetectable. The primary endpoint will be analyzed based on Cochran-Mantel-Haenszel method, stratified at randomization by screening HBsAg level (3 categories: ≥0.05 to < 1 IU/mL, ≥1 to < 10 IU/mL, and ≥10 to <100 IU/mL). The differences in the percentage of sustained HBsAg loss and sustained HBV DNA undetectable together with associated adjusted 95% confidence interval will be presented.

9.6.4. Analyses of Secondary Efficacy Endpoints and Subgroups

Percentage of subjects meeting NrtI discontinuation criteria and other secondary endpoints assessed as proportion will be analysed in the same way as for the primary endpoint.

Time to event endpoints will be compared using stratified log-rank test and continuous endpoints will be compared using Wilcoxon ranks sum test.

Additionally, subgroup analysis by age, gender, and HBsAg level at screening (3 categories: ≥0.05 to < 1 IU/mL, ≥1 to < 10 IU/mL, and ≥10 to <100 IU/mL) will be conducted with respect to the primary endpoint.

9.6.5. Tertiary Efficacy Endpoint and Subgroup Analyses of Phase 2a Part

Wilcoxon rank-sum test will be used to compare the difference between treatment groups in the change from baseline in selected cytokine/chemokine biomarkers during treatment and discontinuation monitoring period, and Spearman rank correlation test will be used to test the correlation between these changes and sustained HBsAg loss and HBV DNA undetectable.

9.6.6. Analysis of Safety Outcome

The Safety Analysis population will be used for clinical review of safety relevant parameters.

A treatment-emergent adverse event (TEAE) is any AE that has an onset date that is on or after the day of study drug administration or any AE that has been present before treatment and worsens relative to the pre-treatment state after administration of study drug.

Descriptive statistics will be used for the safety analysis, and all adverse events (AEs) will be summarized and described according to the latest version of the Medical Dictionary for Regulatory Activities (MedDRA) codes. Coded treatment-emergent adverse events, investigational product-related adverse events, serious adverse events, and adverse events leading to subject withdrawal will be summarized and described by System Organ Class (SOC), Preferred Term (PT), and group, and number and percentage of subject with AE will be summarized. Treatment-emergent adverse events, investigational product-related adverse events, serious adverse events, and adverse events leading to subject withdrawal will also be summarized by severity. All adverse events will be tabulated. Graded laboratory abnormalities will be defined using CTCAE Version 5.0 grading scale. Laboratory tests, vital signs, physical examinations, and 12-lead electrocardiograms will be summarized descriptively. For vital signs, laboratory tests (quantitative measures), and 12-lead electrocardiograms (quantitative measures), observed values and their changes from baseline will be summarized descriptively. Subjects with post-treatment abnormalities of clinical significance will be listed for all tests.

10. SUPPORTING DOCUMENTATION AND OPERATIONAL CONSIDERATIONS

10.1. Appendix 1: Regulatory, Ethical, and Study Oversight Considerations

10.1.1. Regulatory and Ethical Considerations

- This study will be conducted in accordance with the protocol and with the following:
    - Consensus ethical principles derived from international guidelines including the Declaration of Helsinki and Council for International Organizations of Medical Sciences (CIOMS) International Ethical Guidelines
    - Applicable ICH Good Clinical Practice (GCP) Guidelines
    - Applicable laws and regulations
- The protocol, protocol amendments, ICF, Investigator Brochure, and other relevant documents (e.g., advertisements) must be submitted to an IRB/IEC by the investigator and reviewed and approved by the IRB/IEC before the study is initiated.
- Any amendments to the protocol will require IRB/IEC approval before implementation of changes made to the study design, except for changes necessary to eliminate an immediate hazard to study subjects.
- The investigator will be responsible for the following:
    - Providing written summaries of the status of the study to the IRB/IEC annually or more frequently in accordance with the requirements, policies, and procedures established by the IRB/IEC
    - Notifying the IRB/IEC of SAEs or other significant safety findings as required by IRB/IEC procedures
    - Providing oversight of the conduct of the study at the site and adherence to requirements of 21 CFR, ICH guidelines, the IRB/IEC, European regulation 536/2014 for clinical studies (if applicable), and all other applicable local regulations

10.1.2. Financial Disclosure

Investigators and sub-investigators will provide the sponsor with sufficient, accurate financial information as requested to allow the sponsor to submit complete and accurate financial certification or disclosure statements to the appropriate regulatory authorities. Investigators are responsible for providing information on financial interests during the course of the study and for 1 year after completion of the study.

10.1.3. Informed Consent Process

- The investigator or his/her representative will explain the nature of the study to the subject or his/her legally authorized representative and answer all questions regarding the study.

- Subjects must be informed that their participation is voluntary. Subjects or their legally authorized representative will be required to sign a statement of informed consent that meets the requirements of 21 CFR 50, local regulations, ICH guidelines, Health Insurance Portability and Accountability Act (HIPAA) requirements, where applicable, and the IRB/IEC or study center.
- The medical record must include a statement that written informed consent was obtained before the subject was enrolled in the study and the date the written consent was obtained. The authorized person obtaining the informed consent must also sign the ICF.
- Subjects must be re-consented to the most current version of the ICF(s) during their participation in the study.
- A copy of the ICF(s) must be provided to the subject or the subject's legally authorized representative.

10.1.4. Data Protection

- Subjects will be assigned a unique identifier by the sponsor. Any subject records or datasets that are transferred to the sponsor will contain the identifier only; subject names or any information which would make the subject identifiable will not be transferred.
- The subject must be informed that his/her personal study-related data will be used by the sponsor in accordance with local data protection law. The level of disclosure must also be explained to the subject who will be required to give consent for their data to be used as described in the informed consent
- The subject must be informed that his/her medical records may be examined by Clinical Quality Assurance auditors or other authorized personnel appointed by the sponsor, by appropriate IRB/IEC members, and by inspectors from regulatory authorities.

10.1.5. Data Quality Assurance

- All relevant subject data relating to the study will be recorded on printed or electronic CRF unless transmitted to the sponsor or designee electronically (e.g., laboratory data). The investigator is responsible for verifying that data entries are accurate and correct by physically or electronically signing the CRF.
- The investigator must maintain accurate documentation (source data) that supports the information entered in the CRF.
- The investigator must permit study-related monitoring, audits, IRB/IEC review, and regulatory agency inspections and provide direct access to source data documents.
- Monitoring details describing strategy (e.g., risk-based initiatives in operations and quality such as Risk Management and Mitigation Strategies and Analytical Risk-

Based Monitoring), methods, responsibilities and requirements, including handling of noncompliance issues and monitoring techniques (central, remote, or on-site monitoring) are provided in the Monitoring Plan.

- The sponsor or designee is responsible for the data management of this study including quality checking of the data.
- The sponsor assumes accountability for actions delegated to other individuals (e.g., Contract Research Organizations).
- Study monitors will perform ongoing source data verification to confirm that data entered into the CRF by authorized site personnel are accurate, complete, and verifiable from source documents; that the safety and rights of subjects are being protected; and that the study is being conducted in accordance with the currently approved protocol and any other study agreements, ICH GCP, and all applicable regulatory requirements.
- Records and documents, including signed ICFs, pertaining to the conduct of this study must be retained by the investigator. No records may be destroyed during the retention period without the written approval of the sponsor. No records may be transferred to another location or party without written notification to the sponsor.

10.1.6. Source Documents

- Source documents provide evidence for the existence of the subject and substantiate the integrity of the data collected. Source documents are filed at the investigator's site.
- Data reported on the CRF or entered in the eCRF that are transcribed from source documents must be consistent with the source documents or the discrepancies must be explained. The investigator may need to request previous medical records or transfer records, depending on the study. Also, current medical records must be available.
- Source data refer to all information recorded in the original record of certified copy of the clinical study, including clinical findings, observation results and other relevant activity records needed for reconstruction and evaluation of the clinical study.

10.1.7. Study and Site Start and Closure

The study start date is the date on which the clinical study will be open for recruitment of subjects.

The sponsor designee reserves the right to close the study site or terminate the study at any time for any reason at the sole discretion of the sponsor. Study sites will be closed upon study completion. A study site is considered closed when all required documents and study supplies have been collected and a study-site closure visit has been performed.

The investigator may initiate study-site closure at any time, provided there is reasonable cause and sufficient notice is given in advance of the intended termination.

Reasons for the early closure of a study site by the sponsor or investigator may include but are not limited to:

- Failure of the investigator to comply with the protocol, the requirements of the IRB/IEC or local health authorities, the sponsor's procedures, or GCP guidelines
- Inadequate recruitment of subjects by the investigator
- Discontinuation of further study drug development

If the study is prematurely terminated or suspended, the sponsor shall promptly inform the Investigators, the IECs/IRBs, the regulatory authorities, and any contract research organization(s) used in the study of the reason for termination or suspension, as specified by the applicable regulatory requirements. The Investigator shall promptly inform the subject and should assure appropriate subject therapy and/or follow-up.

10.1.8. Publication Policy

The sponsor intends to publish the results of clinical trials regardless of outcome. Results may be published in peer-reviewed journals and/or presented at scientific congresses. The sponsor will comply with the requirements for publication and disclosure of clinical trial information. In accordance with standard editorial and ethical practice, the sponsor will generally support publication of multicenter studies only in their entirety and not as individual site data. Authorship will be determined by mutual agreement based upon International Committee of Medical Journal Editors criteria and guidelines [ICMJE, 2018]. Individuals who do not meet authorship criteria do not warrant named authorship. Nonauthor contributors may be acknowledged in the manuscript when published or recognized in a manner consistent with the target journal policy.

10.2. Appendix 2: Clinical Laboratory Tests

The tests detailed in Table 2 will be performed by the central laboratory. Urine pregnancy test will be performed by the central laboratory.

Investigators must document their review of each laboratory safety report.

Additional tests may be performed at any time during the study as determined necessary by the investigator or required by local regulations.

Table 2: Protocol-Required Safety Laboratory Assessments

**Hematology**	
Complete blood count with differential, including red blood cell count, hemoglobin, hematocrit, white blood cell count, count and percentage of neutrophils, lymphocytes, eosinophils, basophils, and monocytes, and platelet count	
**Chemistry**	
Albumin	Estimated glomerular filtration rate (eGFR)
Blood urea nitrogen (BUN)	Gamma glutamyl transferase (GGT)
Calcium	Glucose
Carbon dioxide/bicarbonate	Lactate dehydrogenase (LDH)
Chloride	Potassium
Creatine kinase	Sodium
Creatinine	Uric acid
**Liver Function Tests**	
Alkaline phosphatase (ALP)	Aspartate aminotransferase (AST)
Alanine aminotransferase (ALT)	Bilirubin (total and direct)
**Coagulation Parameters**	
International normalized ratio (INR)	Prothrombin time (PT)
**Urinalysis**	
Glucose	Proteins
Red blood cells (RBCs)	
**Pregnancy Testing**	
Beta-human chorionic gonadotropin (WOCBP only)	Urine pregnancy test
**Thyroid Panel**	
Thyroid stimulating hormone (TSH)[1]	

[1] Thyroid function will be tested every 12 weeks during PEG-IFN-α dosing: Screening, Week 12, and Week 24.

10.3. Appendix 3: Adverse Events: Definitions and Procedures for Recording, Evaluating, Follow-up, and Reporting

10.3.1. Definition of AE

AE Definition
• An AE is any untoward medical occurrence in a patient or clinical study subject, temporally associated with the use of study drug, whether or not considered related to the study drug.<br>• NOTE: An AE can therefore be any unfavorable and unintended sign (including an abnormal laboratory finding), symptom, or disease (new or exacerbated) temporally associated with the use of study drug.

Events Meeting the AE Definition
• Any abnormal laboratory test results (hematology, clinical chemistry, or urinalysis) or other safety assessments (e.g., ECG, radiological scans, vital signs measurements), including those that worsen from baseline, considered clinically significant in the medical and scientific judgment of the investigator (i.e., not related to progression of underlying disease).<br>• Exacerbation of a chronic or intermittent pre-existing condition including either an increase in frequency and/or intensity of the condition.<br>• New conditions detected or diagnosed after study drug administration even though it may have been present before the start of the study.<br>• Signs, symptoms, or the clinical sequelae of a suspected drug-drug interaction.<br>• Signs, symptoms, or the clinical sequelae of a suspected overdose of either study drug or a concomitant medication. Overdose per se will not be reported as an AE/SAE unless it is an intentional overdose taken with possible suicidal/self-harming intent. Such overdoses should be reported regardless of sequelae.

Events NOT Meeting the AE Definition
• Any clinically significant abnormal laboratory findings or other abnormal safety assessments which are associated with the underlying disease, unless judged by the investigator to be more severe than expected for the subject's condition.<br>• The disease/disorder being studied or expected progression, signs, or symptoms of the disease/disorder being studied, unless more severe than expected for the subject's condition.<br>• Medical or surgical procedure (e.g., endoscopy, appendectomy): the condition that leads to the procedure is the AE.<br>• Situations in which an untoward medical occurrence did not occur (social and/or convenience admission to a hospital).<br>• Anticipated day-to-day fluctuations of pre-existing disease(s) or condition(s) present or detected at the start of the study that do not worsen.

10.3.2. Definition of SAE

If an event is not an AE per definition above, then it cannot be an SAE even if serious conditions are met (e.g., hospitalization for signs/symptoms of the disease under study, death due to progression of disease).

**A SAE is defined as any untoward medical occurrence that, at any dose:**
**a. Results in death**
**b. Is life-threatening**<br>The term 'life-threatening' in the definition of 'serious' refers to an event in which the subject was at risk of death at the time of the event. It does not refer to an event, which hypothetically might have caused death, if it were more severe.
**c. Requires inpatient hospitalization or prolongation of existing hospitalization**<br>• In general, hospitalization signifies that the subject has been detained (usually involving at least an overnight stay) at the hospital or emergency ward for observation and/or treatment that would not have been appropriate in the physician's office or outpatient setting. Complications that occur during hospitalization are AEs. If a complication prolongs hospitalization or fulfills any other serious criteria, the event is serious. When in doubt as to whether "hospitalization" occurred or was necessary, the AE should be considered serious.<br>• Hospitalization for elective treatment of a pre-existing condition that did not worsen from baseline is not considered an AE.
**d. Results in persistent disability/incapacity**<br>• The term disability means a substantial disruption of a person's ability to conduct normal life functions.<br>• This definition is not intended to include experiences of relatively minor medical significance such as uncomplicated headache, nausea, vomiting, diarrhea, influenza, and accidental trauma (e.g., sprained ankle) which may interfere with or prevent everyday life functions but do not constitute a substantial disruption.
**e. Is a congenital anomaly/birth defect**
**f. Other important medical events:**<br>• Medical and scientific judgment should be exercised to determine whether expedited reporting is appropriate in other situations; in general, any important medical event that may not be immediately life-threatening, or result in death, or hospitalization, but for which medical interventions are required to prevent any of the above from happening, is also considered a serious adverse event. For example, intensive treatment in the emergency room or at home for allergic bronchospasm, blood dyscrasias or convulsions that do not result in hospitalisation, or development of drug dependency or drug abuse, etc.

10.3.3. Recording and Follow-Up of AE and/or SAE

**AE and SAE Recording**
• When an AE/SAE occurs, it is the responsibility of the investigator to review all documentation (e.g., hospital progress notes, laboratory reports, and diagnostics reports) related to the event.<br>• The investigator will then record all relevant AE/SAE information in the CRF.<br>• It is **not** acceptable for the investigator to send photocopies of the subject's medical records to the Sponsor in lieu of completion of the AE/SAE CRF page.<br>• There may be instances when copies of medical records for certain cases are requested by the Sponsor. In this case, all subject identifiers, with the exception of the subject number, will be redacted on the copies of the medical records before submission to the Sponsor.<br>• The investigator will attempt to establish a diagnosis of the event based on signs, symptoms, and/or other clinical information. Whenever possible, the diagnosis (not the individual signs/symptoms) will be documented as the AE/SAE.
**Assessment of Intensity**
The investigator will make an assessment of intensity for each AE and SAE reported during the study based on the National Cancer Institute Common Terminology Criteria for Adverse Events (NCI-CTCAE) Version 5.0.
**Assessment of Causality**
• The investigator or a qualified assistant investigator is obligated to assess the relationship between study drug and each occurrence of each AE/SAE.<br>• Relationship between AEs and the study drug: "related" should be entered when there is either (a) a *reasonable possibility* that the AE/SAE is associated with study drug **or** (b) no reasonable alternative explanation can be found. Otherwise, the relationship between AEs and the study drug should be classified as "unrelated".<br>• A "reasonable possibility" of a relationship conveys that there are facts, evidence, and/or arguments to suggest a causal relationship, rather than a relationship cannot be ruled out.<br>• The investigator will use clinical judgment to determine the relationship.<br>• Alternative causes, such as underlying disease(s), concomitant therapy, and other risk factors, as well as the temporal relationship of the event to study drug administration will be considered and investigated.<br>• The investigator will also consult the Investigator's Brochure (IB) and/or Product Information, for marketed products, in his/her assessment.<br>• For each AE/SAE, the investigator **must** document in the medical notes that he/she has reviewed the AE/SAE and has provided an assessment of causality.<br>• There may be situations in which an SAE has occurred and the investigator has minimal information to include in the initial report. However, it is very important that the investigator always make an assessment of causality for every event before the initial transmission of the SAE data.

• The investigator may change his/her opinion of causality in light of follow-up information and send a SAE follow-up report with the updated causality assessment.<br>• The causality assessment is one of the criteria used when determining regulatory reporting requirements.

**Follow-up of AEs and SAEs**
• The investigator is obligated to perform or arrange for the conduct of supplemental measurements and/or evaluations as medically indicated or as requested by the Sponsor to elucidate the nature and/or causality of the AE or SAE as fully as possible. This may include additional laboratory tests or investigations, histopathological examinations, or consultation with other health care professionals.<br>• If a subject dies during participation in the study or during a recognized follow-up period, the investigator will provide the Sponsor with a copy of any post-mortem findings including histopathology.<br>• New or updated information will be recorded in the originally completed CRF.<br>• The investigator will submit any updated SAE data to the Sponsor within 24 hours of receipt of the information.

10.3.4. Reporting of SAEs

**SAE Reporting to the Sponsor via a Paper SAE Reporting Form**
• The investigator must complete a paper SAE report form and send to the Sponsor or its representative (drugsafety-briibio@dmedglobal.com) within 24 hours of the investigator's knowledge of the event.<br>• Contacts for SAE reporting can be found on the SAE Reporting Form.

10.3.5. Reporting of Laboratory Events of Clinical Interest

All laboratory ECIs must be reported to the Sponsor or its representative within 24 hours of the investigator's knowledge of the event. Contact for laboratory ECIs reporting can be found on the ECI Reporting Form.

The laboratory ECI will also be evaluated by the investigator to determine if the event meets criteria for an AE. If AE criteria are met, all clinical sequelae in relation to these laboratory ECIs will be reported as AEs or SAEs, if applicable.

10.3.6. Special Situation Report

For special situations and AEs due to special situations, the investigator should complete a Special Situation Report Form and send it to the Sponsor or its representative (drugsafety-briibio@dmedglobal.com) within 24 hours of becoming aware.

10.4. Appendix 4: Contraceptive Requirements and Collection and Reporting of Pregnancy Information

Contraceptive Requirements:

WOCBP may be included in this study and include any female subject who has experienced menarche and who is not post-menopausal or permanently sterilized (eg, bilateral tubal occlusion, hysterectomy, or bilateral salpingectomy).

WOCBP must be willing to use highly effective methods of contraception 14 days before dose, throughout study participation, and for 12 weeks after last dose of study drug administration. Highly effective methods of birth control result in a low failure rate (ie, less than 1% per year). Birth control methods which are considered highly effective include:

- Established use of combined (estrogen and progestogen containing) oral, intravaginal, or transdermal hormonal methods of contraception associated with inhibition of ovulation **OR** established use of progestogen-only oral, injectable, or implantable hormonal methods of contraception associated with inhibition of ovulation. It is not currently known whether study drug will impact the effectiveness of hormonal contraceptive methods; therefore, it is recommended to use an additional form of contraception (ie, barrier method) throughout the study and for 12 weeks after last study drug administration.
- Placement of an intrauterine device
- Placement of an intrauterine hormone-releasing system
- Surgical sterilization of male partner (with the appropriate post-vasectomy documentation of the absence of sperm in the ejaculate; for female subjects on the study, the vasectomized male partner should be the sole partner for that subject)
- True sexual abstinence, when in line with the preferred and usual lifestyle of the subject. Periodic abstinence (e.g., calendar, ovulation, symptothermal, post-ovulation methods) and withdrawal are not acceptable methods of contraception. Abstinent subjects have to agree to use 1 of the above-mentioned contraceptive methods, if they start sexual relationships during the study and for up to 12 weeks after last dose of study drug administration, or for as long as the subject is followed on study, whichever is longer.
- Barrier method in combination with hormonal contraceptive, as described above

Post-menopausal status is defined as 12 months with no menses without an alternative medical cause.

Collection of Pregnancy Information:

- The investigator will collect pregnancy information about female subjects who are pregnant or male subject’s female partner who are pregnant during the study. The initial information will be recorded on the appropriate form and submitted to the sponsor within 24 hours of learning of a subject's pregnancy.

- The female subject who are pregnant or male subject's female partner who are pregnant will be followed to determine the outcome of the pregnancy. The investigator will collect follow-up information on the female subject who are pregnant or male subject's female partner who are pregnant and the neonate and the information will be forwarded to the sponsor. Generally, follow-up will not be required for longer than 6 to 8 weeks beyond the estimated delivery date. Any termination of pregnancy will be reported, regardless of fetal status (presence or absence of anomalies) or indication for the procedure.
- While pregnancy itself is not considered to be an AE or SAE, any pregnancy complication or elective termination of a pregnancy for medical reasons will be reported as an AE or SAE.
- A spontaneous abortion (occurring at <22 weeks gestational age) or still birth (occurring at >22 weeks gestational age) is always considered to be an SAE and will be reported as such.
- Any post-study pregnancy related SAE considered reasonably related to the study drug by the investigator will be reported to the sponsor as described in Section 8.3.4. While the investigator is not obligated to actively seek this information in former study subjects, he or she may learn of an SAE through spontaneous reporting.
- Any female subject who becomes pregnant while participating in the study will discontinue study drug.

10.5. Appendix 5: Assessments in Subjects Meeting Laboratory ECI Criteria

Additional assessment will be performed in subjects who experience ALT elevations meeting laboratory ECI criteria (Section 8.2.9) from Day 1 of dosing to the last follow-up visit. Following the occurrence of event of ECI, the following assessments in Table 3 will be performed one time, as soon as possible, and no later than two weeks after the initial ALT value meeting criteria for a laboratory ECI. The full panel of assessments should only be performed once; individual assessments may be repeated per investigator discretion, as needed.

Table 3 Assessments in Subjects Meeting Laboratory ECI Criteria

**Hematology**	
Complete blood count with differential, including red blood cell count, hemoglobin, hematocrit, white blood cell count, count and percentage of neutrophils, lymphocytes, eosinophils, basophils, and monocytes, and platelet count	
**Chemistry**	
Albumin	Estimated glomerular filtration rate (eGFR)
Blood urea nitrogen (BUN)	Gamma glutamyl transferase (GGT)
Calcium	Glucose
Carbon dioxide/bicarbonate	Lactate dehydrogenase (LDH)
Chloride	Potassium
Creatine kinase	Sodium
Creatinine	Uric acid
**Liver Function Tests**	
Alkaline phosphatase (ALP)	Aspartate aminotransferase (AST)
Alanine aminotransferase (ALT)	Bilirubin (total and direct)
**Coagulation Parameters**	
International normalized ratio (INR)	Prothrombin time (PT)
**Extended Hepatic Panel**	
Quantitative HBsAg	Ferritin
Quantitative HBV DNA	Blood phosphatidylethanol
Quantitative HBeAg	Urinary ethyglucuronide
HAV antibody IgM	Anti-nuclear antibodies
HCV antibody and HCV RNA	Anti-smooth muscle antibodies
HDV antibody or RNA	Anti-LKM1 antibody
HEV antibody IgM	Anti-mitochondrial antibodies
Epstein-Barr Virus antibodies, IgM and IgG	
Cytomegalovirus antibodies, IgM and IgG	
Ceruloplasmin	
**Imaging**	
Abdominal ultrasound, including Doppler flow, if applicable; CT or MRI acceptable in place of ultrasound if clinically indicated	
**Focused Medical History**	
Use of any potentially hepatotoxic concomitant medications, including over the counter medications and herbal remedies	Alcohol consumption and drugs of abuse

Exposure to other potentially hepatotoxic agents including any work-related exposures (eg. solvents, pesticides, etc.)	Exercise history

CT = computed tomography; HAV = hepatitis A virus; HCV = hepatitis C virus; HDV = hepatitis D virus; HEV = hepatitis E virus; IgG = immunoglobulin G antibody; IgM = immunoglobulin M antibody; LKM1 = liver/kidney microsome-1 antibody; MRI = magnetic resonance imaging; PCR = polymerase chain reaction; RNA = ribonucleic acid; SLA = soluble liver antigen.

11. REFERENCES

Barthel FM-S, Parmar MKB, Royston P. How do multi-stage, multi-arm trials compare to the traditional two-arm parallel group design - a reanalysis of 4 trials. Trials 2009; 10:21, doi:.10.1186/1745-6215-10-21.

Berg T, Simon KG, Mauss S, Schott E, Heyne R, Klass DM, Eisenbach C, Welzel TM, Zachoval R, Felten G, Schulze-Zur-Wiesch J, Cornberg M, Op den Brouw ML, Jump B, Reiser H, Gallo L, Warger T, Petersen J; FINITE CHB study investigators. Long-term response after stopping tenofovir disoproxil fumarate in non-cirrhotic HBeAg-negative patients - FINITE study. *Journal of Hepatology*. 2017 Nov;67(5):918-924.

Chang, M. L., Liaw, Y. F., and Hadziyannis, S. J. (2015). Systematic review: cessation of long-term nucleos(t)ide analogue therapy in patients with hepatitis B e antigen-negative chronic hepatitis B. *Alimentary Pharmacology & Therapeutics*, 2015; *42*(3): 243-257.

Choodari-Oskooei B, Parmar MKB, Royston P, Bowden J. Impact of lack-of-benefit stopping rules on treatment effect estimates of two-arm multi-stage (TAMS) trials with time to event outcome. Trials 2013, 14:23, http://www.trialsjournal.com/content/14/1/23.

Cornberg M, Lok AS, Terrault NA, Zoulim F, and the 2019 EASL-AASLD HBV Treatment Endpoints Conference Faculty. Guidance for design and endpoints of clinical trials in chronic hepatitis B - Report from the 2019 EASL-AASLD HBV Treatment Endpoints Conference. *Journal of Hepatology*. 2020; 72(3): 539-337.

Ferrari C. HBV and the immune response. *Liver Int*. 2015; 35 (Suppl. 1): 121-128.

Hoa PT, Huy NT, Thu le T, Nga CN, Nakao K, Eguchi K, Chi NH, Hoang BH, Hirayama K. Randomized controlled study investigating viral suppression and serological response following pre-S1/pre-S2/S vaccine therapy combined with lamivudine treatment in HBeAg-positive patients with chronic hepatitis B. *Antimicrob Agents Chemother*. 2009; 53:5134-40.

Jeng WJ, Chen YC, Chien RN, Sheen IS, Liaw YF. Incidence and predictors of HBsAg seroclearance after cessation of nucleos(t)ide analogue therapy in HBeAg negative chronic hepatitis B. *Hepatology*. 2018; 68(2): 425-434.

Kondo Y, Ninomiya M, Kakazu E, Kimura O, Shimosegawa T. Hepatitis B surface antigen could contribute to the immunopathogenesis of hepatitis B virus infection. *ISRN Gastroenterol*. 2013;2013:935295.

Konerman MA, Lok AS. Interferon Treatment for Hepatitis B. *Clinics in Liver Disease*. 2016; 20(4): 645-665.

Kramvis A. Genotypes and genetic variability of hepatitis B virus. *Intervirology* 2014; 57(3-4): 141-150.

Lebossé F, Testoni B, Fresquet J, Facchetti F, Galmozzi E, Fournier M, Hervieu V, Berthillon P, Berby F, Bordes I, Durantel D, Levrero M, Lampertico P, Zoulim F. Intrahepatic innate immune response pathways are downregulated in untreated chronic hepatitis B. *J Hepatol*. 2017;66(5):897-909.

Lok AS, McMahon BJ, Brown RS, Wong JB, Ahmed AT, Farah W, Almasri J, Alahdab F, Benkhadra K, Mouchli MA, Singh S, Mohamed EA, Abu Dabrh AM, Prokop LJ, Wang Z, Murad MH, Mohammed K. Antiviral therapy for chronic hepatitis B viral infection in adults: A systematic review and meta-analysis. *Hepatology* 2016; 63(1):284-306.

Marcellin P, Ahn SH, Ma X, Caruntu FA, Tak WY, Elkashab M, et al. Combination of tenofovir disoproxil fumarate and peginterferon α-2a increases loss of hepatitis B surface antigen in patients with chronic hepatitis B. Gastroenterology 2016;150(1):134-44.

Michler T, Kosinska AD, Festag J, Bunse T, Su J, Ringelhan M, Imhof H, Grimm D, Steiger K, Mogler C, Heikenwalder M, Michel ML, Guzman CA, Milstein S, Sepp-Lorenzino L, Knolle P, Protzer U. Knockdown of Virus Antigen Expression Increases Therapeutic Vaccine Efficacy in High-Titer Hepatitis B Virus Carrier Mice. Gastroenterology. 2020 May;158(6):1762-1775.

Papatheodoridi M, Hadziyannis E, Berby F, Zachou K, Testoni B, Rigopoulou E, Gatselis NK, Lyberopoulou A, Vlachogiannakos I, Manolakopoulos S, Dalekos GN, Zoulim F, Papatheodoridis GV. Predictors of hepatitis B surface antigen loss, relapse and retreatment after discontinuation of effective oral antiviral therapy in noncirrhotic HBeAg-negative chronic hepatitis B. *J Viral Hepat.* 2020; 27(2): 118-126.

Perrillo, R. "Benefits and risks of IFN therapy for hepatitis B." *Hepatology.* 2009; 49(5): S103-111.

Ren H, Hu P, Chen XY, Gong GZ, Shang J, Zhang WH, et al. Switching to PegIFNα-2a in NUC treated CHB patients(NEW SWITCH Study)： comparison 48 and 96 weeks. Hepatol Int 2016;10(Suppl 1):S43-4.

Roggendorf H, Krawczyk A, Lindemann M, Shouval D, Michler T, Roggendorf M, Gerken G. Induction of functional control in chronic hepatitis B patients with low level HBsAg using a combination of a PreS1/S2/S HBV vaccine (Sci-B-Vac®) and a nucleoside analogue. *J Infect Dis Ther*. 2019; 7:389-393.

Schweitzer A, Horn J, Mikolajczyk RT, Krause G, Ott JJ. Estimations of worldwide prevalence of chronic hepatitis B virus infection: a systematic review of data published between 1965 and 2013. *Lancet* 2015; 386(10003): 1546-1555.

Stanaway JD, Flaxman AD, Naghavi M, Fitzmaurice C, Vos T, Abubakar I, Abu-Raddad LJ, Assadi R, Bhala N, Cowie B, Forouzanfour MH, Groeger J, Hanafiah KM, Jacobsen KH, James SL, MacLachlan J, Malekzadeh R, Martin NK, Mokdad AA, Mokdad AH, Murray CJL, Plass D, Rana S, Rein DB, Richardus JH, Sanabria J, Saylan M, Shahraz S, So S, Vlassov V, Weiderpass E, Wiersma ST, Younis M, Yu C, Zaki M, Cooke GS. The

global burden of viral hepatitis from 1990 to 2013: findings from the Global Burden of Disease Study 2013. *Lancet* 2016; 388(10049): 1081-1088.

Trepo CHL, Chan, Lok A. Hepatitis B virus infection. *Lancet* 2014;384(9959): 2053-2063.

van Bömmel F, Berg T. Stopping long-term treatment with nucleos(t)ide analogues is a favourable option for selected patients with HBeAg-negative chronic hepatitis B. *Liver International*. 2018;38 Suppl 1:90-96.

Wang S, Chen Z, Hu C, Qian F, Cheng Y, Wu M, Shi B, Chen J, Hu Y, Yuan Z. Hepatitis B virus surface antigen selectively inhibits TLR2 ligand-induced IL-12 production in monocytes/macrophages by interfering with JNK activation. *J Immunol*. 2013 May 15;190(10):5142-51.

Wong TCL, Fung JYY, Chok KSH, Cheung TT, Chan ACY, Dai WC, Ng KKC, Chan SC, Lo, CM. Hepatitis B vaccination in patients receiving oral antiviral therapy without hepatitis B immunoglobulin after liver transplant. *Transplant Proc*. 2018; 50:3681-3688.

Yao CC, Hung CH, Hu TH, Lu SN, Wang JH, Lee CM, and Chen CH. Incidence and predictors of HBV relapse after cessation of nucleoside analogues in HBeAg-negative patients with HBsAg ≤ 200 IU/mL. *Scientific Reports*. 2017; 7(1): 1839.

Ze-Qian Wu, Dong-Ying Xie, Lei Fu, Jia Wei, Jia Shang, Qing He, Wen-hua Zhang, Guang-yu Huang, Yanzhong Peng, Ye Gu, Jia-bin Li, Ying Guo, Yu-juan Guan, Jia-wei Geng, Huan-wei Zheng, Wen-jun Gao, Wen-jing Zhao, Ying-jun Tian, Yi-lan Zeng, Ren Chen, Shengwang Gu, Zhi-liang Gao. Functional cure based on pegylated interferon ? in long-term nucleoside analog suppressed HBeAg negative chronic hepatitis B: a multicenter real-world study (Everest project in China), an interim report. *Journal of Hepatology* 2020; 73: S653-S915.

Zhu D, Liu L, Yang D, Fu S, Bian Y, Sun Z, He J, Su L, Zhang L, Peng H, Fu YX. Clearing Persistent Extracellular Antigen of Hepatitis B Virus: An Immunomodulatory Strategy To Reverse Tolerance for an Effective Therapeutic Vaccination. *J Immunol*. 2016; 196(7): 3079-87.

Title

Protocol Title: A Multicenter, Randomized, Double-Blind, Placebo-Controlled, Parallel-Group Study to Investigate the Efficacy and Safety of BRII-179 (VBI-2601) for the Treatment of Chronic Hepatitis B Virus (HBV) Infection

Protocol Number: BRII-179-002

Compound: BRII-179 (VBI-2601)

Study Phase: Phase 2

Short Title: Study to Investigate Efficacy and Safety of BRII-179 (VBI-2601) for the Treatment of Chronic HBV Infection

Sponsor: Brii Biosciences Limited

Co-Sponsor/ Registration Agent: Brii Biosciences (Beijing) Ltd.

Legal Registered Address: Room 301, 3/F, Building 7,
No. 1 Yongtaizhuang North Road，
Tiandilinfeng (Dongsheng Area)
Haidian District, Beijing 100192,
China

Protocol Version: Version 5.0

Approval Date: 9 December 2022

The English translation is for reference only, in case of any inconsistency the Chinese version shall prevail

Sponsor Signatory

[redacted]

Medical Monitor Name and Contact Information will be provided separately.

INVESTIGATOR AGREEMENT PAGE

For protocol number BRII-179-002

I confirm agreement to conduct the study in compliance with the protocol, *International Conference on Harmonisation Good Clinical Practices (ICH GCP)* as well as applicable local regulatory and ethical requirements.

I acknowledge that I am responsible for overall study conduct. I agree to personally conduct or supervise the described clinical study.

I agree to ensure that all associates, colleagues and employees assisting in the conduct of the study are informed about their obligations. Mechanisms are in place to ensure that site staff receive the appropriate information throughout study.

Investigator Name: ____

____ ________________________

Investigator Signature **Date** (dd/mm/yyyy)

The English translation is for reference only, in case of any inconsistency the Chinese version shall prevail

Protocol Amendment Summary of Changes

This amendment was made based on re-discussion with the investigator.

Detailed revisions of the current protocol Version 5.0 are provided in the table below (Previous protocol amendments summary of changes see Section 10.6 Protocol Amendment History).

Original Section and Title	Description of Protocol Changes	Rationale
Section 1.2 Phase 2a, Section 3.2 Endpoints,<br><br>Section 9.2.1 Phase 2a Part, Section 9.4 Primary Endpoint and Safety Data Analysis in Phase 2a Part	"(Week 24)" is added after "completion of PEG-IFN-α treatment" in the description of primary and secondary endpoint for timing clarification<br><br>The timing of the early futility evaluation of primary endpoint is changed from complete "24 weeks PEG-IFN-α treatment or withdraw the study prior to last scheduled PEG-IFN-α treatment" to complete "24 weeks PEG-IFN-α treatment or withdraw the study/*discontinue study drugs* prior to last scheduled PEG-IFN-α treatment"	To clarify the timing of primary analysis and to include the scenario of subjects discontinued study drug(s) but do not withdraw the study
Section 1.2 Phase 2a, Section 3.2.3 Tertiary Endpoints<br><br>Section 8.2.8 Laboratory Assessments<br><br>Section 9.6.5 Tertiary Efficacy Endpoint and Subgroup Analyses of Phase 2a Part	The sample type of "protein level cytokine/chemokine biomarkers" in tertiary endpoint is revised from serum to plasma<br><br>Section 8.2.8.5 is added to provide clarification on cytokine/chemokine biomarkers collection and assessment.<br><br>The description of statistical analysis method of the tertiary endpoint is revised from "Wicoxon rank sum test will be used to compare the difference between treatment groups in the change from baseline in selected cytokine/chemokine biomarkers during treatment and discontinuation monitoring period" to "When applicable, selected data relating to the biomarker analysis of	To clarify the cytokine/chemokine biomarkers collection and assessment<br><br>Revised the statistical analysis method because it is more appropriate to use descriptive statistics instead of inference statistics in the cytokine/chemokine biomarkers results assessment

	cytokine/chemokines, will be summarized with descriptive statistics"	
Section 1.5.1 SoA<br>Section 8.1.2 Randomization (Day 1)	Section 1.5.1 SoA note a. "*Assessments* performed pre-dose unless otherwise specified" is revised to "Performed pre-dose unless otherwise specified"<br>Specify that blood sample collected prior to study drug administration will be "sent to central laboratory for analysis. Such central laboratory results will be available in a few days, hence the evaluation of these lab results cannot be performed on Day 1" in Section 8.1.2	To clarify that the result evaluation of blood samples collected on Day 1 visit will not be performed on Day 1 visit
Section 1.5.1 SoA<br>Section 4.1.3 PEG-IFN-α Therapy During the Dosing Period	Additional safety laboratory assessments "not limited to safety assessments listed in Appendix 2" is added to Section 1.5.1 note n<br>The following instructions for safety monitoring of subjects in PEG-IFN-α treatment were added to Section 4.1.3 "During safety monitoring of PEG-IFN-α-related adverse reactions in the study, investigators should pay close attention to changes in all safety laboratory tests results, including Hematology tests results (see Section 10.2). Relevant laboratory tests (not limited to protocol-specified safety laboratory tests) should be performed and followed in a timely manner at local laboratories if necessary (in case of adverse events or significant laboratory abnormalities). For subjects whose laboratory measures are close to, but not yet meet, the criteria for dose reduction (see Section 4.1.3.1 and the package insert), investigators are advised to increase the frequency of monitoring of relevant laboratory measures in local laboratories. For subjects who meet the criteria for PEG-IFN-α dose adjustment,	Enhance safety monitoring

	investigators should increase the frequency of monitoring of relevant laboratory measures, and if it is still early until next scheduled study visit, the subjects should be advised to have the relevant laboratory measures re-tested at local laboratory 1 to 2 weeks later.”	
Section 5.1 Inclusion Criteria<br>Section 6.1.1 Formulations	Inclusion criterion 7: “Must be receiving first-line NrtI (ETV or TDF or TAF) therapy for ⩾ 12 months” of the Day 1 visit is revised to “Must be receiving first-line NrtI (for example, entecavir (ETV) or tenofovir (eg. TDF, Tenofovir Amibufenamide or TAF, etc.) therapy for ⩾ 12 months.” of the Day 1 visit randomization<br>“If the subject is taking Tenofovir Amibufenamide Tablets before enrollment, investigator can choose either tenofovir disoproxil fumarate tablets or tenofovir alafenamide fumarate tablets provided by sponsor for antiviral treatment according to the specific conditions and willingness of the subject.” is added to the description of NrtI.in Section 6.1.1 Formulations	To include “Tenofovir Amibufenamide” which was recently approved in China for chronic hepatitis B treatment as one of NrtIs allowed by the current protocol.<br>And to provide clarification on NrtIs selection in this study for subjects taking Tenofovir Amibufenamide prior to study participation.
Section 5.2 Exclusion Criteria	Exclusion criterion 20: “Donated more than 500 mL of blood within 90 days before study drug administration.” is revised into “Donated more than 500 mL of blood within 90 days before study drug administration *at the Day 1 visit*”	“at the Day 1 visit” is added to clarify “before study drug administration”
Section 6.6 Safety Follow-up and Management of Reactions to Study Drug	“A measurement device template (in mm) for measuring solicited local reactions (erythema [redness] and swelling), and *an oral* thermometer for measuring daily temperature, will be provided at the first study drug administration visit, study Day 1” is revised into “A measurement device	Both oral and mercury thermometers can be used to measure daily temperature from clinical practice perspective

	template (in mm) for measuring solicited local reactions (erythema [redness] and swelling), and a thermometer for measuring daily temperature, will be provided at the first study drug administration visit, study Day 1"	
Section 9.2.1 Phase 2a Part	"a sample size of 120 evaluable subjects in an one-sided Type I error of α = 0.3 can provide a power of more than 95% to reject the null hypothesis" is revised to "and one-sided Type I error α = 0.3, The sample size in range of 105 to 120 can provide power of more than 95% to reject the null hypothesis (Table 1). In this study the planned sample size will be approximately 120"<br>Table 1 was added titled with 'The power calculated based on different sample size'	During the study, the sample size was re-calculated to adjust statistical power from 96.5% to 95.1% without changing other assumptions, i.e., one-sided Type I error α was 0.3, and HBsAg loss at Week 24 was 45% and 25% for BRII-179 group and placebo group, respectively. Sample size in range of 105 to 120 is rational and acceptable (Table 1 can clearly present all of power are more than 95% when sample sizes are greater than or equal to 105)
Section 9.5 Analysis Sets	The "Serologically Evaluable Full Analysis Set" was added, this analysis set will include all subjects who were randomized and received at least one dose of BRII-179 or placebo, and with HBsAg ≥ 0.05 IU/mL at baseline. The analysis based on Serologically Evaluable Full Analysis Set will be considered as sensitivity analysis.	Considering the potential discrepancy of HBsAg stratification between screening and baseline, i.e., HBsAg ⩾ 0.05 IU/mL at screening but < 0.05 IU/mL at baseline, and the primary endpoint is HBsAg Loss (HBsAg < 0.05 IU/mL) at Week 24， this analysis set can provide primary endpoint analysis in which all of subjects with HBsAg ⩾ 0.05 IU/mL will be included, i.e., subjects with HBsAg < 0.05 IU/mL will be excluded.

Section 9.6 Statistical Analyses	"number of subjects" is added as descriptive statistics in Section 9.6.1 General Considerations<br><br>"12-lead electrocardiograms (quantitative measures)" is removed from descriptive summary in Section 9.6.6 Analysis of Safety Outcome	Description correction
Section 10 Supporting documentation and operational considerations	Add a new Section 10.6 Appendix 6: Protocol Amendment History. Move protocol version 4.0 protocol amendment summary of changes to this section, and supplement previous protocol version 2.0 and 3.0 protocol amendment summary of changes	To complete protocol structure
Throughout the protocol	Typos, minor translation, references, and formatting errors correction	To improve the overall quality of the protocol

Table of Contents

TITLE.....1
Protocol Amendment Summary of Changes.....4
TABLE OF CONTENTS.....9
1. PROTOCOL SUMMARY.....14
1.1. Synopsis.....14
1.2. Phase 2a (N=120).....14
1.3. Phase 2b (N=480).....15
1.4. Schema.....19
1.5. Schedule of Activities (SoA).....20
1.5.1. BRII-179 (VBI-2601) 18-week and PEG-IFN-α 24-week Dosing Period and 24-week Follow-up Period.....20
1.5.2. NrtI Discontinuation Monitoring Period.....23
2. INTRODUCTION.....24
2.1. Background.....24
2.2. Study Rationale.....25
2.3. Benefit/Risk Assessment.....26
3. OBJECTIVES AND ENDPOINTS.....27
3.1. Objectives.....27
3.1.1. The Primary Objectives.....27
3.1.2. The Secondary objectives.....27
3.1.3. The Tertiary Objectives.....27
3.2. Endpoints.....27
3.2.1. Primary Endpoints.....27
3.2.2. Secondary Endpoints.....27
3.2.3. Tertiary Endpoints.....28
4. STUDY DESIGN.....29
4.1. Overall Design.....29
4.1.1. Independent DSMB.....29
4.1.2. NrtI Discontinuation.....29
4.1.3. PEG-IFN-α Therapy During the Dosing Period.....30
4.2. Scientific Rationale for Study Design.....32
4.3. Dosage Instructions.....33

The English translation is for reference only, in case of any inconsistency the Chinese version shall prevail

4.3.1. Justification for Dose Selection and Number of Doses 33
4.3.2. Maximum dose/exposure in this study 34
4.4. End of Study Definition 34
5. STUDY POPULATION 35
5.1. Inclusion Criteria 35
5.2. Exclusion Criteria 36
5.3. Lifestyle Considerations 38
5.4. Screening Failures 38
6. STUDY INTERVENTION 39
6.1. Study Intervention(s) Administered 39
6.1.1. Formulations 39
6.1.2. Study Intervention 40
6.2. Preparation/Handling/Storage/Accountability 41
6.3. Randomization and Blinding 41
6.4. Compliance of Study Intervention 42
6.5. Concomitant Therapy 42
6.5.1. Prohibited Medications 43
6.6. Safety Follow-up and Management of Reactions to Study Drug 43
6.7. Treatment of Study Intervention Overdose 44
6.8. Intervention after the End of the Study 44
7. DISCONTINUATION OF STUDY INTERVENTION AND SUBJECT DISCONTINUATION/WITHDRAWAL 45
7.1. Discontinuation of Study Intervention 45
7.1.1. Criteria for Discontinuation of Study Intervention 45
7.2. Subject Discontinuation/Withdrawal from the Study 46
7.2.1. Lost to Follow up 46
8. STUDY ASSESSMENTS AND PROCEDURES 48
8.1. Schedule of Assessments 48
8.1.1. Screening 48
8.1.2. Randomization (Day 1) 48
8.1.3. Dosing Period 48
8.1.4. Follow-up Period 49
8.1.5. NrtI Discontinuation Monitoring Period 49
8.1.6. Unscheduled Visits 49
8.1.7. Early Termination Visit 49

8.2. Detailed Description of Study Procedures 50
8.2.1. Medical History 50
8.2.2. Physical Examinations 50
8.2.3. FibroScan 50
8.2.4. Abdominal Ultrasound 50
8.2.5. Alcohol Intake Assessment 50
8.2.6. Vital Signs 50
8.2.7. Electrocardiograms 51
8.2.8. Laboratory Assessments 51
8.2.9. Laboratory Events of Clinical Interest 52
8.3. Adverse Events and Serious Adverse Events 52
8.3.1. Time Period and Frequency for Collecting AE and SAE Information 53
8.3.2. Method of Detecting AEs and SAEs 53
8.3.3. Follow-up of AEs and SAEs 53
8.3.4. Regulatory Reporting Requirements for SAEs 53
8.3.5. Special Situations 54
8.4. Pharmacodynamics 55
8.5. Genetics 55
8.6. [Health Economics] OR [Medical Resource Utilization and Health Economics] 55
9. STATISTICAL CONSIDERATIONS 56
9.1. Statistical Hypotheses 56
9.1.1. Phase 2a Part 56
9.1.2. Phase 2b Part 56
9.2. Sample Size Determination 56
9.2.1. Phase 2a Part 56
9.2.2. Phase 2b Part 57
9.3. Data Monitoring Guidelines for Independent DSMB 58
9.4. Primary Endpoint and Safety Data Analysis in Phase 2 Part 58
9.4.1. Monitoring Guidelines for the Primary Endpoint in Phase 2b Part 58
9.5. Analysis Sets 60
9.6. Statistical Analyses 60
9.6.1. General considerations 60
9.6.2. Analysis of the Primary Efficacy Endpoint for Phase 2a Part 61
9.6.3. Analysis of the Primary Efficacy Endpoint for Phase 2b Part 61

The English translation is for reference only, in case of any inconsistency the Chinese version shall prevail

9.6.4. Analyses of Secondary Efficacy Endpoints and Subgroups....61
9.6.5. Tertiary Efficacy Endpoint and Subgroup Analyses of Phase 2a Part....62
9.6.6. Analysis of Safety Outcome....62
10. SUPPORTING DOCUMENTATION AND OPERATIONAL CONSIDERATIONS....62
10.1. Appendix 1: Regulatory, Ethical, and Study Oversight Considerations....62
10.1.1. Regulatory and Ethical Considerations....62
10.1.2. Financial Disclosure....63
10.1.3. Informed Consent Process....63
10.1.4. Data Protection....64
10.1.5. Data Quality Assurance....64
10.1.6. Source Documents....65
10.1.7. Study and Site Start and Closure....65
10.1.8. Publication Policy....66
10.2. Appendix 2: Clinical Laboratory Tests....67
10.3. Appendix 3: Adverse Events: Definitions and Procedures for Recording, Evaluating, Follow-up, and Reporting....68
10.3.1. Definition of AE....68
10.3.2. Definition of SAE....69
10.3.3. Recording and Follow-Up of AE and/or SAE....70
10.3.4. Reporting of SAEs....71
10.3.5. Reporting of Laboratory Events of Clinical Interest....71
10.3.6. Special Situation Report....72
10.4. Appendix 4: Contraceptive Requirements and Collection and Reporting of Pregnancy Information....73
10.5. Appendix 5: Assessments in Subjects Meeting Laboratory ECI Criteria....75
10.6. Appendix 6: Protocol Amendment History....77
11. REFERENCES....90

The English translation is for reference only, in case of any inconsistency the Chinese version shall prevail

ABBREVIATIONS

AE	adverse event
ALT	alanine aminotransferase
HBsAb	hepatitis B surface antibody
DNA	deoxyribonucleic acid
DSMB	Data and Safety Monitoring Board
ECG	electrocardiogram
ECI	events of clinical interest
ET	early termination
ETV	entecavir
HBeAg	hepatitis B e-antigen
HBsAg	hepatitis B surface antigen
HBV	hepatitis B virus
HCV	hepatitis C virus
HDV	hepatitis D virus
HIV	human immunodeficiency virus
ICF	informed consent form
IFN-α	interferon-alpha
IMP	Investigational medicinal product
IU	international units
kPa	kilopascal
LLOQ	lower limit of quantification
NCI-CTCAE	National Cancer Institute Common Terminology Criteria for Adverse Events
NIMP	Non-investigational medicinal product
NrtI	nucleos(t)ide reverse transcriptase inhibitors
ULN	upper limit of normal
PEG-IFN-α	pegylated interferon-alpha
PD	Pharmacodynamic
SoA	schedule of activities
SAE	serious adverse event
TDF	tenofovir disoproxil fumarate
TAF	tenofovir alafenamide
WOCBP	women of child-bearing potential

The English translation is for reference only, in case of any inconsistency the Chinese version shall prevail

1. PROTOCOL SUMMARY

1.1. Synopsis

Protocol Title: A Multicenter, Randomized, Double-Blind, Placebo-Controlled, Parallel-Group Study to Investigate the Efficacy and Safety of BRII-179 (VBI-2601) for the Treatment of Chronic Hepatitis B Virus (HBV) Infection

Rationale:
Multiple therapies with differential mechanisms of actions to treat hepatitis B virus (HBV) is an encouraging strategy to achieve higher rates of hepatitis B surface antigen (HBsAg) loss and ideally HBsAg seroconversion. The present study (BRII-179-002) will evaluate the efficacy and safety of adding a recombinant protein immunotherapeutic (BRII-179 [VBI-2601]) treatment regimen to existing pegylated interferon-alpha (PEG-IFN-α) and nucleos(t)ide reverse transcriptase inhibitor (NrtI) therapy in chronic HBV patients. Most subjects in the current study are anticipated to be recruited from the Everest Project (NCT04035837). The primary objective of the Everest Project is to achieve functional cure of HBV infection by optimizing treatment strategies of NrtIs and PEG-IFN-α.

Individualized regimens of PEG-IFN-α and NrtI therapies have shown promising efficacy in reducing HBsAg with some patients achieving HBsAg clearance (Ren, 2016; Marcellin, 2016). This result is more pronounced in patients on optimized HBV treatment to low HBsAg levels, which is the rationale of including subjects from the Everest Project in this study. Preclinical and clinical studies have also shown the benefit of low HBsAg levels for breaking tolerance with a therapeutic HBV vaccine (Zhu, 2016; Michler, 2020; Roggendorf, 2019). The recombinant protein immunotherapeutic, BRII-179 (VBI-2601), may break immunological tolerance and restore and/or augment adaptive T cell and antibody responses to the surface antigens. These responses together have the potential to better achieve a functional cure (sustained loss of HBsAg in blood with or without seroconversion to the surface antigen antibody) of chronic HBV infection.

Objectives and Endpoints

The study will consist of two parts, Part 2a and 2b, of which approximately 120 subjects are planned to be enrolled in the first part (Phase 2a) and 480 subjects are planned to be enrolled in the second part (Phase 2b).

1.2. Phase 2a (N=120)

Objectives	Endpoints
Primary	
To determine the efficacy of BRII-179 (VBI-2601) to increase HBsAg loss through completion of PEG-IFN-α treatment	• Percentage of subjects with HBsAg loss at completion of PEG-IFN-α treatment (Week 24)
Secondary	

▪ To evaluate functional cure of subjects with chronic HBV infection receiving BRII-179 (VBI-2601) therapy in combination with PEG-IFN-α + NrtI	• Percentage of subjects with sustained HBsAg loss and persistently undetectable HBV DNA (defined as undetectable HBsAg and HBV DNA for at least 24 weeks) after NrtI discontinuation.
▪ To assess the antiviral activity of BRII-179 (VBI-2601) therapy in combination with PEG-IFN-α + NrtI	• HBsAg change from baseline from Day 1 to completion of PEG-IFN-α treatment (Week 24)<br>• Percentage of subjects with HBsAg loss at any timepoint<br>• Percentage of subjects with undetectable HBV-DNA (HBV DNA < Lower Limit of Quantification (LLOQ)) 24 and 48 weeks after NrtI discontinuation<br>• Percentage of subjects meeting the NrtI discontinuation criteria by end of follow-up period (Week 48)<br>• Percentage of subjects with HBsAg seroconversion at any timepoint<br>• Percentage of subjects with hepatitis B surface antibody (HBsAb) response as of the end of the follow-up period (Week 48)<br>• Time to achieve HBsAg seroclearance and seroconversion<br>• Percentage of subjects requiring NrtI re-treatment during follow up
To assess safety of BRII-179 (VBI-2601) therapy	• Percentage of subjects with adverse events (AEs) and serious adverse events (SAEs)<br>• Percentage of subjects with abnormalities in clinical laboratory tests
Tertiary	
To assess the effect on relevant immune markers in subjects with chronic HBV infection treated with BRII-179 (VBI-2601) in combination with PEG-IFN-α + NrtI	• Plasma protein level cytokine/chemokine biomarkers changes from baseline post dosing, i.e., from Day 1 of dosing to the end of follow-up (Week 48) and during the NrtI Discontinuation Monitoring Period

1.3. Phase 2b (N=480)

Objectives	Endpoints
Primary	
To evaluate functional cure of subjects with chronic HBV infection receiving BRII-179 (VBI-2601) therapy in combination with PEG-IFN-α + NrtI	• Percentage of subjects with sustained HBsAg and HBV DNA loss (defined as undetectable HBsAg and HBV DNA for at least 24 weeks) after NrtI discontinuation
Secondary	

To assess the antiviral activity of BRII-179 (VBI-2601) therapy in combination with PEG-IFN-α + NrtI	• Percentage of subjects with undetectable HBV-DNA (HBV DNA < Lower Limit of Quantification (LLOQ)) 24 and 48 weeks after NrtI discontinuation<br>• Percentage of subjects meeting the NrtI discontinuation criteria by end of follow-up period (Week 48)<br>• Percentage of subjects with HBsAg loss at any timepoint<br>• Percentage of subjects with HBsAg seroconversion at any timepoint<br>• Percentage of subjects with hepatitis B surface antibody (HBsAb) response by end of follow-up period (Week 48)<br>• HBsAg change from baseline from Day 1 to End of Treatment (Week 24)<br>• Time to achieve HBsAg seroclearance and seroconversion<br>• Percentage of subjects requiring NrtI re-treatment during follow up
To assess safety of BRII-179 (VBI-2601) therapy	• Percentage of subjects with adverse events (AEs) and serious adverse events (SAEs)<br>• Percentage of subjects with abnormalities in clinical laboratory tests

The English translation is for reference only, in case of any inconsistency the Chinese version shall prevail

Overall Design

Study BRII-179-002 is a double-blind, randomized, placebo-controlled, parallel-group study aims to evaluate the clinical effect of adding BRII-179 (VBI-2601) to existing PEG-IFN-α and NrtI therapy in patients with chronic HBV infection without cirrhosis.

Most subjects in the current study are anticipated to be recruited from the Everest Project (NCT04035837). The Everest Project is a multicenter real-world study being conducted in over 260 hospitals in China. The plan is to recruit 30,000 patients with chronic HBV infection effectively treated with NrtI therapy for at least one year, who are hepatitis B e-antigen (HBeAg) negative with HBV DNA <100 IU/ml and HBsAg <1500 IU/mL at study entry. As part of the study participation, subjects receive PEG-IFN-α as "add-on" or "switch to" treatment for 48 to 96 weeks. The primary objective of the Everest Project is to achieve functional cure of HBV infection by optimizing treatment strategies of NrtIs and PEG-IFN-α.

Eligible subjects who have been receiving PEG-IFN-α for 24 to 28 doses and NrtI therapy for at least 12 months with HBV DNA<LLOQ for ≥ 6 months, will be randomized 1:1 to receive BRII-179 (VBI-2601) or placebo in addition to the existing PEG-IFN-α and NrtI therapy. Subjects will be stratified at randomization by screening HBsAg level (3 categories: ≥0.05 to < 1 IU/mL, ≥1 to < 10 IU/mL, and ≥10 to <100 IU/mL). Subjects will complete investigational treatment with BRII-179 or placebo at Week 18; PEG-IFN-α treatment will last for up to 24 weeks after enrollment, while NrtI treatment will continue into the Follow-up Period. The BRII-179-002 study design schema can be found in Section 1.4.

Estimated total duration for each subject will include the following: screening up to 28 days, dosing period 24 weeks (18 weeks BRII-179 or placebo investigational treatment; 24 weeks PEG-IFN-α treatment), and a 24-week follow-up period. Eligible subjects that meet NrtI discontinuation criteria (Section 4.1.2.1) (eligibility assessed at Week 36 to Week 48) may enter a NrtI discontinuation monitoring period for an additional 48 weeks from the day of NrtI discontinuation (Section 1.5.2).

Number of Subjects:

The study will consist of two parts, Part 2a and 2b, of which approximately 120 subjects are planned to be enrolled in the first part (Phase 2a) and 480 subjects are planned to be enrolled in the second part (Phase 2b).

Intervention Groups and Duration:

Eligible subjects currently receiving PEG-IFN-α and NrtI therapy will receive the study drug BRII-179 (VBI-2601) or placebo, every three weeks for a total of 7 doses over an 18-week course.

Both groups will be treated with both PEG-IFN-α and NrtI, with PEG-IFN-α lasted for 24 weeks after enrollment, and NrtI through the follow-up period.

Independent Data and Safety Monitoring Board (DSMB)

An independent DSMB will monitor subject safety and treatment efficacy data for this study. The details for the operation and responsibilities of the DSMB will be defined in a separate DSMB Charter. The Charter will delineate the composition, duties, responsibilities, and procedures of the DSMB, data required at each meeting, as well as the analyses that will be conducted.

Disclosure Statement: This is a phase 2, randomized, multicenter, double-blind, placebo-controlled, parallel-group study to evaluate the efficacy and safety of BRII-179 (VBI-2601) in patients with chronic HBV infection receiving PEG-IFN-α and NrtI therapy. All references to "Sponsor" contained herein shall refer to Brii Biosciences.

1.4. Schema

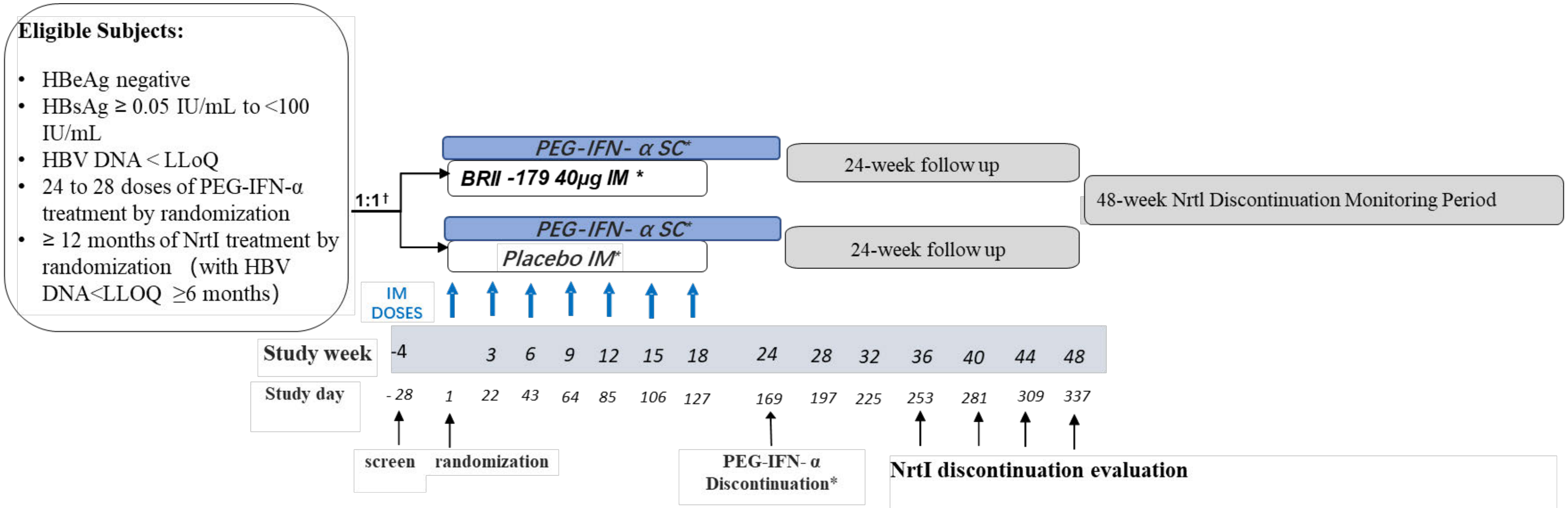

NrtI discontinuation evaluation

- Subjects that meet the NrtI discontinuation criteria may switch from the follow-up period to the 48-week NrtI discontinuation monitoring period
- Day 1 of the NrtI Discontinuation Monitoring Period is the first day a subject discontinues NrtI
- Subjects that have not met NrtI discontinuation criteria by Week 48 will be withdrawn from the study

IM: intramuscular ; SC : Subcutaneous Injection

* Each subject will accept 24 weeks PEG-IFN-α treatment after enrollment; Co-medication of NrtI will continue during the dosing period; NrtI treatment will last until week 48 if the subject could not meet the NrtI discontinuation criteria

† Subjects stratified by HBsAg level

1.5. Schedule of Activities (SoA)

1.5.1. BRII-179 (VBI-2601) 18-week and PEG-IFN-α 24-week Dosing Period and 24-week Follow-up Period

**Study Stage**	**Screening**	**Dosing Period [b]**								**Follow-up Period**					
**Study Visit Week**	Screening	Rand	W3	W6	W9	W12	W15	W18	W24 [c] /ET1	W28	W32	W36[d]	W40[d]	W44[d]	W48[d]/ ET2
**Study Visit Day ± Visit Window**	D-28 to-1	D1[a]	D22 ± 3	D43 ± 3	D64 ± 3	D85 ± 3	D106± 3	D127 ± 3	D169 ± 3	D197 ± 3	D225 ± 3	D253 ± 5	D281 ± 5	D309 ± 5	D337 ± 5
Informed consent form (ICF)	X														
Inclusion/exclusion criteria	X	X[e]													
Demography	X														
Medical history including current medical conditions and HBV genotype [f]	X														
Abdominal Ultrasound	X								X						X
FibroScan	X[s]														
Full physical exam [g]	X							X	X						X
Symptom-directed physical exam		X	X	X	X	X	X			X	X	X	X	X	
Alcohol intake assessment [h]		X	X	X	X	X	X	X	X	X	X	X	X	X	X
Vital signs [i]	X	X				X			X						
12-lead ECG [j]	X	X							X						
Subject instructed to complete diary		X	X	X	X	X	X	X	X	X	X	X	X	X	X
Urinalysis [n]	X														
Pregnancy test [k]	X	X	X	X	X	X	X	X	X	X	X	X	X	X	X

Safety Laboratory Assessments [n]	X	X	X	X	X	X	X	X	X	X	X	X	X	X	X
Randomization		X													
Study drug administration		X	X	X	X	X	X	X[l]	X[m]						
Screening viral serology	X														
Blood sample for HBsAg/HBsAb quantitative	X	X		X		X	X	X	X	X	X	X	X	X	X
Blood sample for cytokine/chemokine biomarker quantitation	X	X		X		X		X	X		X		X		X
Blood sample for HBV DNA quantitation[o]	X	X				X			X	X	X	X	X	X	X
Adverse Events assessment [p]	←-------------------------------------X-------------------------------------→														
ECI surveillance [q]	←-------------------------------------X-------------------------------------→														
Concomitant medications review	←-------------------------------------X-------------------------------------→														
NrtI medication adherence review [r]	←-------------------------------------X-------------------------------------→														
PEG-IFN-α medication adherence review [r]	←--------------------X--------------------→														

AE = adverse event; HBsAg = hepatitis B surface antigen; HBsAb = hepatitis B surface antibody; ECI = events of clinical interest; ECG = electrocardiogram; ET1 = early termination 1; ET2 = early termination 2; HBV = Hepatitis B Virus; NrtI = nucleoside/nucleotide reverse transcriptase inhibitor; ULN = Upper Limit of Normal

Note: When scheduled at the same timepoints, the assessments of vital signs and 12-lead ECGs must be performed before blood sample collections.

[a] Performed pre-dose unless otherwise specified.

[b] If a subject withdraws prematurely from the study prior to their Week 24 visit, ET1 assessments should be performed within 14 days after study withdrawal.

[c] This visit occurs when complete PEG-IFN-α treatment,24 weeks after the enrollment.

[d] Subjects that meet NrtI discontinuation criteria (Section 4.1.2.1) may discontinue from the Follow-up Period and enter the NrtI Discontinuation Monitoring Period (Section 1.5.2). Day 1 of the NrtI Discontinuation Monitoring Period is the first day subject stops their NrtI therapy.

[e] Evaluation of inclusion and exclusion criteria related to pregnancy test, medical history and concomitant medications prior to dosing.

[f] A complete medical history will be taken at screening including HBV genotype if available.

[g] Height and weight will be recorded at screening. Body mass index will be calculated based on the measurements. Only weight will be collected afterwards.

[h] Subjects' alcohol intake will be recorded while on study.

[i] Vital signs (blood pressure, pulse rate, respiratory rate, and temperature) should be measured after the subject has rested comfortably for approximately 5 minutes.

[j] 12-lead safety ECGs should be measured in the supine position after the subject has rested comfortably for 10 minutes.

[k] Women of childbearing potential are required to have pregnancy tests. Negative pregnancy test must be confirmed prior to study drug administration. Blood pregnancy test should be conducted at central lab at screening visit, urine pregnancy test and blood pregnancy test (if necessary) at other time points will be conducted at local laboratory.

[l] Subjects will complete investigational treatment with BRII-179 or placebo.

[m] PEG-IFN-α treatment will be continued for 24 weeks; NrtI therapy will continue into the Follow-up Period.

[n] Refer to Table 2 Safety Assessments in Appendix 2. Additional safety laboratory assessments may be performed at any time during the study as determined necessary by the investigator (not limited to safety assessments listed in Appendix 2, local laboratory assessment is acceptable).

[o] If a subject experiences clinically significant alanine aminotransferase (ALT) flare, additional HBV DNA quantitation sample(s) may be collected as determined necessary by the investigator.

[p] AEs will be collected from signing the ICF until last visit. After informed consent has been obtained but prior to initiation of study drug, untoward medical occurrence, if related to study procedure, will be recorded as AE; otherwise, will be recorded as medical history.

[q] Subject will be monitored for clinical laboratory events of clinical interest (ECI) and additional assessments may be performed if indicated. ECI for this study include ALT > 3x ULN, if Day 1 pre-dose ALT ≤ ULN; ALT > 2x baseline, if Day 1 pre-dose ALT > ULN.

[r] Subjects will be instructed to bring NrtI and PEG-IFN-α (including all empty containers) to the trial site at every visit, as applicable, beginning with the screening visit. Adherence will be assessed by counting of empty containers and unused medication. See Section 6.4.

[s] Subjects will have a FibroScan evaluation during screening. FibroScan evaluation performed within 6 months prior to screening is also acceptable for eligibility evaluation.

1.5.2. NrtI Discontinuation Monitoring Period

Study Stage	NrtI Discontinuation Monitoring Period[b,c]						
**Study Visit Week**		W4	W8	W12	W24	W36	W48/ET3
**Study Visit Day ± Visit Window**	D1[a]	D29 ± 3	D57 ± 3	D85 ± 3	D169 ± 7	D253 ± 7	D337 ± 7
Safety Laboratory Assessments [d]	X	X	X	X	X	X	X
Symptom-directed physical exam	X	X	X	X	X	X	X
Abdominal Ultrasound	X				X		X
FibroScan							X
Pregnancy test		X					X
Blood sample for HBsAg/HBsAb quantitation	X			X	X	X	X
Blood sample for cytokine/chemokine biomarker quantitation	X		X		X		X
Blood sample for HBV DNA quantitation	X	X	X	X	X	X	X
Concomitant medications	←------------------------------------X------------------------------------→						
Adverse event assessment	←------------------------------------X------------------------------------→						
NrtI retreatment surveillance [e]	←------------------------------------X------------------------------------→						
ECI surveillance [f]	←------------------------------------X------------------------------------→						

AE = adverse event; ECI = events of clinical interest; HBsAg = hepatitis B surface antigen; HBsAb = hepatitis B surface antibody; HBV = Hepatitis B Virus; NrtI = nucleoside/nucleotide; ULN = Upper Limit of Normal; ET3 = early termination 3

[a] All timepoints are relative to the day of NrtI discontinuation (D1) which is the first day subjects stop their NrtI therapy.

[b] All subjects who discontinue NrtI therapy will be followed through 48 weeks, even if NrtI retreatment occurs. If a subject withdraws prematurely from the study prior to the W48 visit, ET3 assessments should be performed.

[c] NrtI retreatment guidelines are provided in Section 4.1.2.2.

[d] Safety laboratory assessments are limited to liver function tests.

[e] Investigator should monitor subjects for NrtI retreatment criteria. See Section 4.1.2.2.

[f] Subject will be monitored for clinical laboratory events of clinical interest (ECI) and additional assessments may be performed if indicated. ECI for this study include ALT > 3x ULN, if Day 1 pre-dose ALT ≤ ULN; ALT > 2x baseline, if Day 1 pre-dose ALT > ULN.

2. INTRODUCTION

2.1. Background

Chronic HBV infection remains an important global public health problem that leads to serious sequelae over time, including cirrhosis, liver failure, and hepatocellular carcinoma (Trepo, 2014). An estimated 290 million people are living with chronic HBV infection worldwide, and almost 800,000 people die annually due to associated sequelae (Stanaway, 2016).

Human HBV is a deoxyribonucleic acid (DNA) virus in the Hepadnaviridae family, with 10 HBV genotypes (A to J). The distribution of these genotypes varies geographically, with A and D being more common in Northern Europe, North America, and Western and Central Asia; and B and C being more common in Southeast Asia and China. HBV genotypes may influence disease progression rates and treatment response (Kramvis, 2014).

Chronic HBV infection is characterized by detection of HBV surface antigen (HBsAg) in the blood for > 6 months. The prevalence of chronic HBV infection varies geographically, with a range of < 2% in low to > 8% in high prevalence countries (Schweitzer, 2015). In high prevalence countries, such as those in sub-Saharan Africa and East Asia, transmission occurs predominantly in infants and children by perinatal and horizontal routes. In more industrialized countries, new infections are highest among young adults and transmission occurs predominantly via injection drug use and high-risk sexual behaviors. The risk of developing chronic HBV infection depends on the age at the time of infection. While only ~10% of people infected as adults develop chronic HBV infection, 90% of infants infected perinatally or during the first 6 months of life, and 20% to 60% of children infected between 6 months and 5 years of age remain chronically infected. Twenty-five percent of people who acquire HBV during infancy and childhood will develop primary liver cancer or cirrhosis during adulthood.

Current treatment options for chronic HBV infection are limited. One option is long-term Nucleoside reverse transcriptase inhibitors (NrtI) therapy, for example tenofovir (tenofovir disoproxil fumarate or tenofovir alafenamide) or entecavir (Lok, 2016). However, NrtI therapy does not result in a cure of HBV infection. The viral DNA is not cleared from the infected cell, and viral protein production, in particular the production of HBsAg, typically continues at high levels as measured in blood (Scaglione, 2012). These high levels of HBsAg subviral particles are proposed to mediate HBV-specific immune dysfunction by decoying and exhausting host cellular and humoral adaptive immune responses. Therefore, reduction or clearance of HBsAg from blood is considered central to achieving a functional cure (sustained loss of HBsAg in blood with or without seroconversion to HBsAb) and is considered an optimal outcome of treatment (Block , 2013). An alternative treatment option is a finite course of Pegylated Interferon α (PEG-IFN-α) therapy which can induce a functional cure, but only in a small percentage of patients (< 10%) (Konerman, 2016). Unfortunately, PEG-IFN-α therapy carries considerable risk of dose-limiting adverse events (AEs) which may be severe and/or serious, and discontinuation rates are significant (Perrillo, 2009).

The English translation is for reference only, in case of any inconsistency the Chinese version shall prevail

The failure of NrtI therapy to produce a functional cure and the limitations of PEG-IFN-α therapy highlight the clinical need for effective, well-tolerated HBV therapies of finite duration.

2.2. Study Rationale

BRII-179 (VBI-2601) is a recombinant virus-like particle-based (VLP) based protein immunotherapeutic derived from the 3rd generation HBV prophylaxis vaccine, Sci-B-Vac® consists of all three HBV surface (S) antigen forms: small, middle and large proteins. The large S protein contains the N-terminal preS1 and preS2 domains and the adjacent small S protein. The middle S protein contains preS2 and the adjacent small S protein.

It has been hypothesized that high levels of circulating HBsAg lead to immune tolerance of HBV and contribute to chronic HBV infection. BRII-179 (VBI-2601) utilizes Pre-S1, Pre-S2, and S protein components as the immunogen in a therapeutic application of preS1/S2 containing HBsAg. Additionally, to have an effective T cell response, BRII-179 (VBI-2601) differs from Sci-B-Vac® and most of the recombinant envelope vaccines by using aluminium phosphate as an adjuvant rather than aluminum hydroxide. As a new formulation of higher doses of Sci-B-Vac® with aluminum-phosphate adjuvant, BRII-179 (VBI-2601) shows enhanced Th1 responses against the HBV surface antigens. BRII-179 has been shown to be safe and well-tolerated in chronic HBV (CHB) patients under Nrtl therapy in a phase 1b/2a study (BRII-179-001). Administration of BRII-179 carrying S, Pre-S1 and Pre-S2 regions of HBV envelope is immunogenic at both B and T cell levels in CHB patients.

Multiple therapies with differential mechanisms of actions to treat HBV is an encouraging strategy to achieve higher rates of HBsAg loss and ideally HBsAg seroconversion. The present study (BRII-179-002) will evaluate the efficacy and safety of adding a recombinant protein immunotherapeutic (BRII-179 [VBI-2601]) treatment regimen to existing PEG-IFN-α and NrtI therapy in patients with chronic HBV infection. Most subjects in the current study are anticipated to be recruited from the Everest Project (NCT04035837). The primary objective of the Everest Project is to achieve functional cure of HBV infection by optimizing combination and sequential treatment strategies of NrtIs and PEG-IFN-α in patients with chronic HBV infection (negative HBeAg) and low levels of HBsAg (≤ 1500 IU/mL) and HBV DNA (< 100 IU/mL).

Individualized regimens of combination therapy of IFN-α, an innate immunomodulator and antiviral agent and NrtI, a direct acting antiviral agent have shown promising efficacy in reducing HBsAg with some patients achieving HBsAg clearance (Ren, 2016; Marcellin, 2016). This result is more pronounced in patients on optimized HBV treatment to low baseline HBsAg levels, which is the rationale of including subjects from the Everest Project in this study. Preclinical and clinical studies have also shown the benefit of low HBsAg levels for breaking tolerance with a therapeutic HBV vaccine (Zhu, 2016; Michler, 2020; Roggendorf, 2019). BRII-179 (VBI-2601), as an immunomodulator, can break immunological tolerance and restore and/or augment cellular T cell and antibody responses to the HBV surface antigens which in turn result in HBsAg clearance. Therefore, the initial treatment of PEG-IFN-α and NrtI followed by BRII-179 add-on treatment could increase the potential to achieve higher functional cure rate in chronic HBV infected patients.

2.3. Benefit/Risk Assessment

This study will provide information on the efficacy and safety of BRII-179 (VBI-2601) added to PEG-IFN-α and NrtI optimized therapy to achieve functional cure in patients with chronic HBV infection.

There are extensive human safety and immunogenicity data for antigens and adjuvant components used in BRII-179. Sci-B-Vac® with identical antigenic components has been safely used in more than 500,000 people (healthy subjects and CHB patients) worldwide (including the United States, Canada, Europe, Hong Kong of China), including more than 600 subjects who received Sci-B-Vac® with the same aluminum phosphate adjuvant as that in BRII-179 and had a good tolerability profile. In addition, the immunogenicity and safety of Sci-B-Vac® vaccines with aluminum phosphate adjuvant for early clinical studies are comparable to those with aluminum hydroxide adjuvant at a later stage. In the completed acute and chronic toxicology studies, the main toxicity profile of BRII-179 was generally consistent in rabbits and rats (Sci-B-Vac®) at dose levels exceeding 40 times the planned clinical dose of BRII-179, including mild and reversible side effects at intramuscular injection (IM) sites which were generally recoverable. In nonclinical toxicology studies, acute and chronic multiple-dose toxicology studies in rats and New Zealand White rabbits were conducted to support the currently planned number of clinical doses. BRII-179 (VBI-2601) with or without IFN-α as an adjuvant, administered every 4 weeks for a total of 4 doses, have been evaluated in adult subjects with chronic HBV infection. Favourable safety and tolerability profiles were observed at 40 µg dose level. In addition, the 8-month therapeutic vaccine trial (Sci-B-Vac®), which has been administered once a month in the Vietnamese population, has shown good efficacy and good safety data.

Based on the above mentioned available clinical and non-clinical data, the proposed add-on of BRII-179 (VBI-2601) to PEG-IFN-α and NrtI therapy may further reduce HBsAg effectively and induce HBV-specific immunity which may lead to immune control and functional cure of chronic HBV infection. The safety profile of BRII-179 (VBI-2601) is supportive of the overall risk/benefit profile for the proposed dose regimen and treatment duration.

More detailed information about the known and expected benefits and risks and reasonably expected adverse events of BRII-179 (VBI-2601) may be found in the Investigator's Brochure.

The English translation is for reference only, in case of any inconsistency the Chinese version shall prevail

3. OBJECTIVES AND ENDPOINTS

3.1. Objectives

3.1.1. The Primary Objectives

- Phase 2a: To evaluate the efficacy of BRII-179 to increase HBsAg loss through completion of PEG-IFN-α treatment
- Phase 2b: To evaluate functional cure of chronic HBV subjects receiving BRII-179 (VBI-2601) therapy in combination with PEG-IFN-α + NrtI

3.1.2. The Secondary objectives

- To assess the antiviral activity of BRII-179 (VBI-2601) therapy added to PEG-IFN-α + NrtI

- To assess safety of BRII-179 (VBI-2601) therapy

3.1.3. The Tertiary Objectives

- Phase 2a only: To assess the effect on relevant immune markers in subjects with chronic HBV infection treated with BRII-179 (VBI-2601) in combination with PEG-IFN-α + NrtI

3.2. Endpoints

3.2.1. Primary Endpoints

- Phase 2a: Percentage of subjects with HBsAg loss at completion of PEG-IFN-α treatment (Week 24)

- Phase 2b: Percentage of subjects with sustained HBsAg and HBV DNA loss (defined as undetectable HBsAg and HBV DNA for at least 24 weeks) after NrtI discontinuation

3.2.2. Secondary Endpoints

Below secondary endpoints apply to both Phase 2a and Phase 2b unless otherwise specified.

- HBsAg change from baseline of from Day 1 to completion of PEG-IFN-α treatment (Week 24)

- Percentage of subjects with HBsAg loss at any timepoint

- Phase 2a only: Percentage of subjects with sustained HBsAg loss and persistently undetectable HBV DNA (defined as undetectable HBsAg and HBV DNA for at least 24 weeks) after NrtI discontinuation

- Percentage of subjects with undetectable HBV-DNA (HBV DNA < LLOQ) 24 and 48 weeks after NrtI discontinuation
- Percentage of subjects meeting the NrtI discontinuation criteria by end of follow-up period (Week 48)
- Percentage of subjects with HBsAg seroconversion at any timepoint
- Percentage of subjects with HBsAb response by end of follow-up period (Week 48)
- HBsAg change from baseline from Day 1 to End of Treatment (Week 24)
- Time to achieve HBsAg seroclearance and seroconversion
- Percentage of subjects requiring NrtI re-treatment during follow up
- Percentage of subjects with adverse events (AEs) and serious adverse events (SAEs)
- Percentage of subjects with abnormalities in clinical laboratory tests

3.2.3. Tertiary Endpoints

Phase 2a only: Assessment of plasma protein level cytokine/chemokine biomarkers changes from baseline post dosing, i.e., from Day 1 of dosing to the end of follow-up (Week 48), and during the NrtI Discontinuation Monitoring Period

4. STUDY DESIGN

4.1. Overall Design

Study BRII-179-002 is a double-blind, randomized, placebo-controlled, parallel-group study of adding BRII-179 (VBI-2601) to existing PEG-IFN-α and NrtI therapy in patients with chronic HBV infection without cirrhosis.

Most subjects of the current study are anticipated to be recruited from the Everest Project (NCT04035837). The Everest Project is a multicenter real-world study being conducted in over 260 hospitals in China. The plan is to recruit 30,000 patients with chronic HBV effectively treated with NrtI therapy for at least one year who are HBeAg negative with HBV DNA <100 IU/ml and HBsAg <1500 IU/mL at study entry. As part of the study participation, subjects receive PEG-IFN-α as "add-on" or "switch to" treatment for 48 to 96 weeks. The primary objective of the Everest Project is to achieve functional cure of HBV infection by optimizing treatment strategies of NrtIs and PEG-IFN-α.

Eligible subjects who have received PEG-IFN-α for 24 to 28 doses and NrtI therapy for at least 12 months with HBV DNA<LLOQ for ≥ 6 months, will be randomized 1:1 to receive BRII-179 (VBI-2601) or placebo in addition to the existing PEG-IFN-α and NrtI therapy. Subjects will be stratified at randomization by screening HBsAg level (3 categories: ≥0.05 to < 1 IU/mL, ≥1 to < 10 IU/mL, and ≥10 to <100 IU/mL). Subjects will complete investigational treatment with BRII-179 or placebo at Week 18, PEG-IFN-α treatment will last for up to 24 weeks after enrollment, while NrtI therapy will continue into the Follow-up Period. The BRII-179-002 study design schema can be found in Section 1.4.

Estimated total duration for each subject will include the following: screening up to 28 days, 24-week dosing period (BRII-179 treatment for 18 weeks and PEG-IFN-α treatment for 24 weeks), and 24-week follow-up period. Eligible subjects that meet NrtI discontinuation criteria (Section 4.1.2.1) (eligibility assessed at Week 36 to Week 48) may enter a NrtI discontinuation monitoring period for an additional 48 weeks from the day of NrtI discontinuation (Section 1.5.2).

4.1.1. Independent DSMB

An independent DSMB will monitor subject safety and treatment efficacy data for this study. The details for the operation and responsibilities of the DSMB will be defined in a separate DSMB Charter. The Charter will delineate the composition, duties, responsibilities, and procedures of the DSMB, data required at each meeting, as well as the analyses that will be conducted.

4.1.2. NrtI Discontinuation

4.1.2.1. NrtI Discontinuation Criteria

The investigator will evaluate whether subjects are eligible for discontinuation of NrtI therapy starting from Week 36 and every four weeks until Week 48 based on the

following NrtI discontinuation criteria:

- Alanine aminotransferase (ALT) < 2x ULN (results from previous visit) AND
- HBV DNA < LLOQ (results from previous visit) AND
- 2 consecutive assessments of HBsAg < 0.05 IU/mL (assessed starting from Week 28 laboratory results)

Subjects who are eligible to discontinue from NrtI therapy may discontinue from the Schedule of Activities in Section 1.5.1 and may instead continue into the NrtI Discontinuation Monitoring Period of the study. These subjects will follow the Schedule of Activities for subjects who have discontinued from the NrtI therapy (Section 1.5.2). Subjects who discontinue from their NrtI therapy will be followed in the NrtI Discontinuation Monitoring Period for an additional 48 weeks from their day of NrtI discontinuation.

4.1.2.2. NrtI Re-treatment

It is recommended that the Investigator should consider re-treatment with NrtI therapy during the NrtI Discontinuation Monitoring Period if subjects meet the following criteria at the same visit (based on Papatheodoridi, 2020):

- ALT > 15x ULN with positive HBV DNA
- ALT 10-15x ULN with positive HBV DNA: retreatment may be implemented at investigator's discretion
- ALT 5-15x ULN with elevated total bilirubin > 2 mg/dL (predominantly direct) and/or prolongation of prothrombin time (PT) ≥ 3 seconds with positive HBV DNA
- ALT 2-5x ULN and HBV DNA >2,000 IU/mL persisting for ≥ 12 consecutive weeks
- ALT > 3x ULN and HBV DNA > 100,000 IU/mL at the same visit

Whether subjects are re-treated with NrtI is at the discretion of the Investigator following good medical judgement. Subjects who are re-treated with NrtI therapy should continue to be followed per the SoA Section 1.5.2 for the duration of the 48-week NrtI Discontinuation Monitoring Period.

4.1.3. PEG-IFN-α Therapy During the Dosing Period

Subjects entering the Dosing Period of the study will continue their PEG-IFN-α treatment, the investigator should monitor and medically manage relevant side effects as necessary. The dose of PEG-IFN-α is based on relevant clinical guidelines and information from the package insert which is considered as proper by the investigator. Temporary interruptions of PEG-IFN-α administration are discouraged. Subjects are to remain on treatment for the entire duration of the study until completion of Week 24 and the dose of PEG-IFN-α should not be changed during the trial unless dose reduction or discontinuation is indicated because of laboratory abnormalities/adverse events (see Section 4.1.3.1 for instructions for

dose reduction). Subjects must be counselled regarding the importance of not missing doses of PEG-IFN-α.

In the case that a subject does miss a dose(s):

- If the PEG-IFN-α injection was late by 1 to 3 days, the next dose should be administered immediately. The subsequent dose should then be administered on the next scheduled injection date.
- If the PEG-IFN-α injection was late by 4 to 7 days, subject should wait to receive the next scheduled injection (1 dose is skipped).
- If the PEG-IFN-α injection was late by >7 days, treatment can be restarted at any time by the investigator and the next dose should be on scheduled injection dates.

During safety monitoring of PEG-IFN-α-related adverse reactions in the study, investigators should pay close attention to changes in all safety laboratory tests results, including Hematology tests results (see Section 10.2). Relevant laboratory tests (not limited to protocol-specified safety laboratory tests) should be performed and followed in a timely manner at local laboratories if necessary (in case of adverse events or significant laboratory abnormalities). For subjects whose laboratory measures are close to, but not yet meet, the criteria for dose reduction (see Section 4.1.3.1 and the package insert), investigators are advised to increase the frequency of monitoring of relevant laboratory measures in local laboratories. For subjects who meet the criteria for PEG-IFN-α dose adjustment, investigators should increase the frequency of monitoring of relevant laboratory measures, and if it is still early until next scheduled study visit, the subjects should be advised to have the relevant laboratory measures re-tested at local laboratory 1 to 2 weeks later.

4.1.3.1. Dose Adjustment for adverse drug reactions

If a PEG-IFN-α dose adjustment is required due to moderate to severe adverse reactions (including clinical manifestations and/or laboratory abnormalities), the initial dose reduction should be performed with clinical consideration. However, in some cases, further dose reduction may be needed. As the adverse reactions abate, re-escalation or recovery of the initial dose of PEG-IFN-α may be considered.

For detail information on the dosage adjustment of PEG-IFN-α, please refer to the product label.

Hematology:

Guidelines for PEG-IFN-α Dose Adjustment According to Hematology results

Laboratory Values	PEG-IFN-α Dose Reduction	Suspend PEG-IFN-α if
Absolute neutrophil count (ANC)	≥750/mm^3: Maintain dose<br>< 750/mm^3: Reduce dose	< 500/mm^3: Treatment should be suspended until ANC values return to more than 1,000/mm^3

		Re-institute at low dose and monitor ANC
Platelet	≥ 50,000/mm³: Maintain dose<br>< 50,000/mm³: Reduce to low dose	<25,000/mm³

Liver function:

In subjects with progressive or persistent ALT increases > 10 x ULN, the dose of PEG-IFN-α should be reduced and more frequent monitoring of liver function should be performed. If ALT increases are further progressive despite dose reduction or accompanied by increased bilirubin or evidence of hepatic decompensation, therapy should be immediately discontinued. After dose reduction or interruption of PEG-IFN-α, treatment may be re-instituted at the discretion of the investigator after improvement in liver function.

Other disorders:

PEG-IFN-α may cause or aggravate neuropsychiatric, autoimmune, endocrine, ophthalmologic, and infectious disorders. Monitor closely and withdraw therapy with persistently severe or worsening signs or symptoms of the above disorders. Please see package insert for more information.

4.2. Scientific Rationale for Study Design

This is a phase 2, double-blind, randomized, placebo-controlled, parallel-group study of adding BRII-179 (VBI-2601) to existing PEG-IFN-α and NrtI therapy in patients with chronic HBV infection without cirrhosis. The study design will provide an opportunity to evaluate the ability of BRII-179 (VBI-2601) to achieve a clinically meaningful increase in functional cure (sustained loss of HBsAg in blood with or without seroconversion to HBsAb) in subjects with chronic HBV on optimized PEG-IFN-α and NrtI therapy as compared to therapy consisting of optimized PEG-IFN-α and NrtI therapy alone.

There is increasing evidence that low levels of quantitative HBsAg may improve the potential for HBsAg loss, and this is most evident in patients who lose HBsAg after stopping long-term NrtI therapy. It has been observed that patients with decreasing HBsAg and a lower HBsAg level < 100 IU/mL at the end of treatment were able to maintain control of HBV after discontinuation of long-term NrtI therapy (Chang, 2015; Yao 2017; Jeng, 2018; Papatheodoridi, 2020). Recent reporting from the Everest Project seems to corroborate this observation from previous studies. In 1427 patients on background NrtI therapy who have completed 24 to 26 weeks of PEG-IFN-α treatment and 516 patients who have completed 48 weeks of PEG-IFN-α treatment, the overall rate of HBsAg loss for patients treated ≥12, ≥24, ≥36 and ≥48 weeks was 13.00%, 16.06%, 20.49% and 23.01%, respectively. Subgroup analysis based on baseline HBsAg stratification (0.05-100, 100-500, 500-1000, 1000-1500 IU/mL) found that the HBsAg loss rate was highest in patients

with baseline HBsAg between 0.05 and 100 IU/mL (34.93% for patients treated ≥ 48 weeks) (Ze-Qian, 2020).

Viral rebound may occur shortly after NrtI discontinuation in some cases, which requires close monitoring during study participation in order to control ALT flares. The HBV viral relapse after NrtI discontinuation may induce the host immune response and potentially a long-term immune control against the viral infection. Sustained HBsAg loss and/or virological response were observed in HBeAg-negative patients discontinuate NrtI therapy in prospective cohort studies (van Bömmel, 2018) as well as in a randomized controlled study (Berg, 2017). This study design was designed to evaluate the potential benefit of decreased viral antigen levels in immune responses to BRII-179 treatment after discontinuation of long-term NrtI therapy.

Surrogate endpoints of HBV functional cure measure biochemical, virological, and histological outcomes. Based on current knowledge, the best surrogate of HBV functional cure is HBsAg loss confirmed on 2 occasions at least 6 months apart with or without HBsAg seroconversion plus HBV DNA below the detection offline (Cornberg, 2020). The incidence of sustained HBsAg loss and undetectable HBV DNA will be used as primary endpoints to assess the potential of BRII-179 (VBI-2601) to induce functional cure.

The target population of this study is HBeAg-negative non-cirrhotic patients with chronic HBV infection that has been virologically suppressed (baseline HBsAg ≥ 0.05 IU/mL and < 100 IU/mL). In the Everest program, we found that some patients had been treated with PEG-IFN-α for 24-28 weeks and NrtI for 18 months and had a preliminary HBsAg decline with HBV DNA below the detection limit, however, could not achieve HBsAg loss upon completion of 48 weeks of PEG-IFN-α treatment. We believe that the immunomodulatory effects of BRII-179 as a therapeutic vaccine can further enhance HBsAg decline and ultimately achieve HBsAg loss, leading to functional cure.

4.3. Dosage Instructions

4.3.1. Justification for Dose Selection and Number of Doses

Sci-B-Vac® has been evaluated previously as a therapeutic vaccine at 20μg and 40 μg dose in multiple clinical studies in chronic HBV infected patients (Hoa, 2009, Lo, 2007; Roggendorf, 2019). Sci-B-Vac® was well tolerated at these higher doses. Sci-B-Vac® also demonstrated good safety profile when assessed in liver transplant patients at a high dose of 40 μg (Wong, 2018). In a Phase 1b/2a randomized, open-label, controlled study (BRII-179-001) of BRII-179 completed in multiple countries and regions in the Asia-Pacific region including China, 44 subjects with chronic hepatitis B received up to 40 μg BRII-179 intramuscularly every 4 weeks for a total of 4 doses and it was demonstrated that the drug was safe and well tolerated. Based on these clinical safety data, we selected 40 μg per dose as the dose administered in this study.

In Study BRII-179-001, BRII-179 every 4 weeks for a total of 4 doses elicited HBV-specific T- and B-cell responses in a subset of subjects, however, the response was

The English translation is for reference only, in case of any inconsistency the Chinese version shall prevail

significantly delayed and with shorter duration compared to that in healthy subjects, and this delayed and low immune response phenomenon coincided with the predicted chronic hepatitis B immunocompromise or tolerance. This study also showed that the intensity of HBV-specific immune responses induced by 4 doses of BRII-179 was not sufficient to achieve a significant HBsAg reduction. In order to achieve better efficacy and considering previously published data of other therapeutic vaccines, we believe that an increase in the number of doses of BRII-179 is needed. The completed preclinical long-term toxicology studies in New Zealand White rabbits with drug administered every 2 weeks for a total of 10 doses over 18 weeks can support clinical studies up to 18 weeks. In addition, dosing every 3 weeks in the AAV-HBV mouse model supported the induction and maintenance of an effective immune response based on nonclinical pharmacodynamic studies. Therefore, we plan to conduct a clinical study with BRII-179 administrated every 3 weeks for a total of 7 doses over 18 weeks to explore the possibility of HBsAg loss.

In summary, currently available safety and tolerability data of BRII-179 (VBI-2601), as well as the safety assessment from the toxicology studies, support the proposed BRII-179 (VBI-2601) treatment regimen to explore functional cure in patients with chronic HBV infection.

4.3.2. Maximum dose/exposure in this study

The maximum dose of BRII-179 is 40 μg every 3 weeks. No other dose adjustment is planned in this study.

4.4. End of Study Definition

A subject is considered to have completed the study if he/she has completed all phases of the study including the last scheduled procedure shown in the Schedule of Activities.

The end of the study is defined as the date of the last visit of the last subject in the study or last scheduled procedure shown in the Schedule of Activities for the last subject in the trial.

5. STUDY POPULATION

A total of 600 Chinese nationality subjects (approximately 120 subjects are planned to be enrolled in Phase 2a part and approximately 480 subjects are planned to be enrolled in Phase 2b part) with virologically suppressed chronic HBV infection without cirrhosis receiving PEG-IFN-α and NrtI therapy are planned for this study.

Prospective approval of protocol violations to recruitment and enrollment criteria, also known as protocol waivers or exemptions, is not permitted. Adherence to the criteria as specified in the protocol is essential.

5.1. Inclusion Criteria

Subjects eligible to be included in the study must meet all of the following criteria. All laboratory test requirements for inclusion will be performed by central laboratory unless specified otherwise.

1. A signed and dated written Informed Consent Form (ICF) prior to study participation.

2. Age ≥ 18 (or age of legal consent, whichever is older), and ≤ 60 at time of screening.

3. Documented chronic HBV infection (positive serum HBsAg for ≥ 6 months, negative HBsAb).

4. Negative HBeAg

5. HBsAg ≥ 0.05 IU/mL and < 100 IU/mL at screening.

6. HBV DNA < LLOQ at screening (central lab).

7. Must be receiving first-line NrtI for example, entecavir (ETV) or tenofovir (eg. TDF, Tenofovir Amibufenamide or TAF, etc.) therapy for ≥ 12 months (with evidence of HBV DNA<LLOQ for ≥ 6 months, local lab results accepted) and PEG-IFN-α for 24 to 28 doses (for subjects who are not strictly administered once a week due to objective reasons such as adverse reactions, the number of treatment weeks should not exceed treatment doses by 4) of the Day 1 visit randomization and could provide the related treatment history.

8. Female subjects must have a negative pregnancy test or confirmation of post-menopausal status. Post-menopausal status is defined as 12 months with no menses without alternative medical cause. Women of child-bearing potential (WOCBP) must have a negative serum pregnancy test at screening and a negative urine pregnancy test on Day 1, cannot be breast feeding, and must be willing to use highly effective methods of contraception 14 days before study drug administration through 12 weeks after last study drug administration.

9. Agrees to not donate blood during the duration of the study.

The English translation is for reference only, in case of any inconsistency the Chinese version shall prevail

10. Serum ALT and AST ≤ 5x ULN at screening.

Study screening laboratory tests may be repeated once for values considered to be questionable by investigator with medical monitor approval.

5.2. Exclusion Criteria

Subjects are excluded from the study if any of the following criteria apply:

1. Any clinically significant chronic medical condition other than chronic HBV infection that, in the opinion of the investigator makes the patient unsuitable for participation in the study.

2. Any clinically significant acute condition such as fever (> 38º C) or acute respiratory illness within 7 days of first study drug administration.

3. Significant liver fibrosis or cirrhosis as defined by having a FibroScan result of > 9 kPa at screening. Unless the subject has a result of ≤9 Kpa from a FibroScan evaluation performed within 6 months prior to screening or liver biopsy performed within 1 year prior to screening that confirm the absence of Metavir F3 fibrosis or F4 cirrhosis. If a subject has had both procedures in the specified timeframes prior to screening, the most recent result should be used to determine eligibility.

4. History or evidence of drug or alcohol abuse as assessed by the Investigator, within the 12 months before screening.

5. History of intolerance to IM injection.

6. History of clinically significant chronic liver disease from any cause other than chronic HBV infection.

7. History of hepatic decompensation, including ascites, hepatic encephalopathy and/or esophageal or gastric varices.

8. Treatment by immunosuppressant (including but not limited to corticosteroids at a prednisolone equivalent dose > 20 mg/day) within 30 days before study drug administration at the Day 1 visit and throughout the duration of study participation.

9. Known history of immunological function impairment, including but not limited to:

   - Autoimmune disease (e.g., multiple sclerosis, type 1 diabetes mellitus , myasthenia gravis, Crohns disease and other inflammatory bowel diseases, celiac disease, systemic lupus erythematosus, scleroderma, including diffuse systemic form and limited cutaneous form of systemic sclerosis or CREST syndrome, systemic sclerosis, dermatomyositis polymyositis, rheumatoid arthritis, juvenile idiopathic arthritis, autoimmune thyroiditis including Hashimoto thyroiditis, Grave’s or Basedow’s disease, immune thrombocytopenic purpura, autoimmune hemolytic

anemia, autoimmune hepatitis, psoriasis, vitiligo, vasculitis, Guillain Barré syndrome, Addison's disease, Bell's palsy, and alopecia areata); OR

- Primary immunodeficiency disorders (e.g., common variable immune deficiency, defective phagocytic cell function and neutropenia syndromes, and complement deficiency); OR

- Secondary immunodeficiency disorders (e.g., resulting from acquired immunodeficiency syndrome caused by human immunodeficiency virus (HIV) infection, solid organ transplant, and splenectomy).

10. Any HBV vaccine (licensed or experimental) prior to study enrollment and throughout the duration of study participation.

11. Immunization with attenuated vaccines (e.g., Mumps, Measles, Rubella) within 30 days before study drug administration at the Day 1 visit and throughout the duration of study participation.

12. Has received blood products or immunoglobulin within 90 days before study drug administration at the Day 1 visit or likely to require blood products during the study.

13. Participated in another clinical trial with an investigational drug or a biologic within 30 days or within 5 drug half-lives (whichever is longer) before study drug administration at the Day 1 visit. Subjects must agree not to take part in any other study at any time during their participation in this study, inclusive of the follow-up period.

14. Previous or current psychiatric condition that precludes compliance with the protocol. Specifically excluded are persons with psychoses, ongoing risk for suicide, or history of suicide attempt or gesture within the past 5 years.

15. The subject has any history of cancer requiring locoregional or systemic therapy within 5 years of randomization or has current disease. Subjects with completely surgically resected tumors with no evidence of disease for more than 3 years, or a history of low risk basal cell carcinoma will be accepted (low risk being defined as 1) location on the trunk of the body, arms, legs, cheeks, forehead, temples, scalp, neck, or chin, 2) < 2 cm in size, 3) nodular or superficial, 4) primary cancer that has not come back after treatment, 5) edge of the cancerous area is clear and smooth, and 6) not located in or around nerves).

16. Any skin abnormality or tattoo that would limit post treatment injection site assessment.

17. Known hypersensitivity or contraindication to BRII-179 (VBI-2601) or its excipients.

18. Unwilling, or unable in the opinion of the investigator, to comply with study requirements, including the use of an adequate birth control method.

The English translation is for reference only, in case of any inconsistency the Chinese version shall prevail

19. Active infection with human immunodeficiency virus (HIV), hepatitis C virus (HCV) or hepatitis Delta virus.

20. Donated more than 500 mL of blood within 90 days before study drug administration at the Day 1 visit.

21. Renal impairment with estimated glomerular filtration rate (eGFR) ≤ 60 ml/min/1.73 m^2 at screening.

22. Uncontrolled diabetes mellitus as determined by the Investigator.

23. Diagnosis of advanced stage heart failure (New York Heart Association Class III or Class IV) or unstable angina.

24. Any laboratory test abnormality that is considered as clinically significant or may contradict continued treatment with PEG-IFN-α by the investigator.

5.3. Lifestyle Considerations

Alcohol consumption is limited to no more than 1 unit per day (unit: 1 glass of wine [125 mL] = 1 measure of spirits [30 mL] = one-half pint of beer [284 mL]) during the participation of this study.

5.4. Screening Failures

Screening failures are defined as subjects who consent to participate in the clinical study but are not subsequently randomly assigned to study drug. A minimal set of screening failure information is required to ensure transparent reporting of screening failure subjects to meet the Consolidated Standards of Reporting Trials (CONSORT) publishing requirements and to respond to queries from regulatory authorities. Minimal information includes demography, screen failure details, eligibility criteria, and any serious adverse event (SAE).

Individuals who do not meet the criteria for participation in this study (screening failure) may not be rescreened.

6. STUDY INTERVENTION

6.1. Study Intervention(s) Administered

Study intervention is defined as any study intervention(s), marketed product(s), placebo, or medical device(s) intended to be administered to a study participant according to the trial protocol.

Clinical study intervention drugs provided by the Sponsor will be packaged to support enrollment. Clinical study drugs will be affixed with clinical label in accordance with regulatory requirements.

6.1.1. Formulations

The following study drugs will be used and provided in this study:

- BRII-179 40µg IM injection
- Placebo IM injection
- PEG-IFN-α Subcutaneous Injection
- NrtI (either entecavir tablets, tenofovir disoproxil fumarate tablets or tenofovir alafenamide fumarate tablets. Entecavir tablet in combination with tenofovir disoproxil fumarate tablet or tenofovir alafenamide fumarate tablet may be provided if the investigator deems necessary) oral administration

BRII-179 (VBI-2601) is aluminium phosphate absorbed HBsAg Bulk Drug Substance (HBsAg spherical particles [~30 nm mean diameter] suspended in phosphate-buffered saline) and 8% Sucrose, in addition to 10 mM phosphate buffer.

Placebo will be prepared from commercially available 0.9% Sodium Chloride for Injection which will be provided to the study site. Subjects assigned to placebo will receive a volume of placebo that would be equal to the volume that would be required if that subject had been assigned to IP.

PEG-IFN-α is a colorless, clear liquid formulation of peginterferon α-2b from modified recombinant human interferon α2b expressed in yeast system with polyethylene glycol (40 kD Y type) and excipients of sodium chloride, sodium acetate, mannitol, aspartic acid, water for injection, and sodium hydroxide to adjust pH.

NrtI may be either entecavir tablets, tenofovir disoproxil fumarate tablets or tenofovir alafenamide fumarate tablets (entecavir tablet in combination with tenofovir disoproxil fumarate tablet or tenofovir alafenamide fumarate tablet may be provided if the investigator deems necessary), and the type of NrtI during treatment should be consistent with that before enrollment (e.g., if entecavir is used 3 months before enrollment, then entecavir should be continued after enrollment. If the subject is taking Tenofovir Amibufenamide Tablets before enrollment, investigator can choose either tenofovir disoproxil fumarate tablets or tenofovir alafenamide fumarate tablets provided by sponsor for antiviral treatment according to the specific conditions and willingness of the subject.). Nrtl should

be given orally one tablet daily and should not be discontinued or changed *ad libitum* until discontinuation according to protocol defined criteria or treatment regimen change due to drug resistance.

6.1.2. Study Intervention

**Arm Name**	**Group 1**	**Group 2**	**Group 1 & 2**	**Group 1 & 2**
**Intervention Name**	BRII-179 (VBI-2601)	Placebo	PEG-IFN-α	NrtI (entecavir tablets or tenofovir disoproxil fumarate tablets or tenofovir alafenamide fumarate tablets)
**Type**	Drug	Placebo	Drug	Drug
**Dose Formulation**	Solution for Injection	0.9% Sodium Chloride for Injection	Injection	Tablet
**Dose Level(s)**	BRII-179 40 µg	Placebo	180 µg	0.5mg/300mg/25mg
**Route of Administration**	BRII-179 in one syringe; administer via IM injection<br><br>Refer to the Pharmacy Manual for the details.	Placebo in one syringe; administer via IM injection<br><br>Refer to the Pharmacy Manual for the details.	1 syringe; Subcutaneous Injection<br><br>Refer to the Pharmacy Manual for the details.	1 tablet for each NrtI, oral<br><br>Refer to the Pharmacy Manual for the details.
**Use**	Experimental	Experimental	Experimental	Experimental
**IMP and NIMP**	IMP	IMP	IMP	NIMP
**Sourcing**	Provided centrally by the Sponsor	Provided centrally by the Sponsor	Provided centrally by the Sponsor	Provided centrally by the Sponsor
**Packaging and Labelling**	IMP will be labelled as required per	Placebo will be prepared and labelled as instructed in the	IMP will be labelled as required per	NIMP will be labelled as required

Arm Name	Group 1	Group 2	Group 1 & 2	Group 1 & 2
	country requirement.	Pharmacy Manual	country requirement.	per country requirement.

On dosing days, the pharmacist or designee will prepare the required amount of study drugs into syringe for injection. A qualified clinical investigative site staff member under the supervision of the investigator or designee will administer the study drug. The injection site will be marked and mapped for later observation and should be documented. If a local reaction around the injection site occurs, photographs may be obtained. Please refer to Section 6.6 for further details.

6.2. Preparation/Handling/Storage/Accountability

- Only subjects enrolled in the study may receive study drugs and only authorized site staff may supply or administer study drugs. All study drugs must be stored in a secure, environmentally controlled, and monitored (manual or automated) area in accordance with the labelled storage conditions with access limited to the investigator and authorized site staff.
- The investigator, institution, or the head of the medical institution (where applicable) is responsible for study drug accountability, reconciliation, and record maintenance (i.e., receipt, reconciliation, and final disposition records).
- Further guidance and information for the final disposition of unused study drugs are provided in the Pharmacy Manual.

6.3. Randomization and Blinding

Subjects will be assigned to study treatment in accordance with the randomization schedule. The randomization code will be generated by using a validated computerized system. Subjects will be randomized using an Interactive Web Response System (IWRS). Once a randomization number is assigned to a subject, it cannot be reassigned to any other subject in the study. Study intervention will be dispensed at the study visits summarized in SoA (Section 1.5.1). Returned study intervention should not be re-dispensed to the subjects.

Study drug will be double-blind. Neither the subject nor the study physician will know which study drug the subject is receiving. The investigator or treating physician may unblind a subject's treatment assignment **only in the case of an emergency** when knowledge of the study treatment is essential for the appropriate clinical management or welfare of the subject. Whenever possible, the investigator must first discuss options with the Medical Monitor or appropriate sponsor study personnel before unblinding the subject's treatment assignment. If this is impractical, the investigator must notify the sponsor as soon as possible, but without revealing the treatment assignment of the unblinded subject, unless that information is important for the safety of subjects currently

in the study. The date and reason for the unblinding must be recorded in the appropriate data collection tool.

Sponsor clinical safety and pharmacovigilance staff may unblind the treatment assignment for any subject with an SAE. If the SAE requires that an expedited regulatory report be sent to one or more regulatory agencies, a copy of the report, identifying the subject's treatment assignment, may be sent to clinical investigators in accordance with local regulations and/or sponsor policy.

The primary reason for treatment discontinuation (the event or condition that led to the unblinding) will be recorded in the eCRF. Subject may continue in the follow-up period of the study once the treatment code is unblinded (see Section 7.1 Discontinuation of Study Intervention).

Subjects will receive study drugs directly from the investigator or designee, under medical supervision. The date and time of each dose administered in the clinic will be recorded in the source documents and recorded in the eCRF. The dose of study drug and study subject identification will be confirmed at the time of dosing by a member of the study site staff other than the person administering the study drug.

6.4. Compliance of Study Intervention

In the event of an interruption of study drug treatment ≥ 2 weeks as specified in the protocol, discussion between the investigator and the Sponsor is required, and the decision on subject management should be documented in writing.

Subjects will be instructed to bring NrtI and PEG-IFN-α (including all empty containers) to the trial site at every visit, as applicable, beginning with the screening visit. Adherence will be assessed by counting of empty containers and unused medication.

Every effort should be made to ensure good adherence with NrtI and PEG-IFN-α therapy. Adherence to NrtI and PEG-IFN-α should be assessed at every relevant visit. Adherence of PEG-IFN-α will be assessed by counting of empty containers and unused medication. Subjects will be instructed to bring all empty containers of PEG-IFN-α to the trial site at every visit, as applicable, for adherence assessment. Adherence of NrtI will be assessed via review of the information on the diary cards filled in by the subjects.

6.5. Concomitant Therapy

The study will collect all prescription and over-the-counter medications (including traditional Chinese medicine and herbal medicine) that the subject receives from screening and throughout the study.

Any medications that the subject is receiving at the time of enrolment or receives during the study must be recorded along with:

- Reason for use
- Dates of administration including start and end dates

The English translation is for reference only, in case of any inconsistency the Chinese version shall prevail

- Dosage information including dose and frequency

Should subjects have a need to initiate treatment with any excluded concomitant medication, the Medical Monitor must be consulted prior to initiation of the new medication. In instances where an excluded medication is initiated prior to discussion with the Sponsor, the investigator must notify the Sponsor as soon as he/she is aware of the use of the excluded medication. The Medical Monitor should be contacted if there are any questions regarding concomitant or prior therapy.

6.5.1. Prohibited Medications

The following medications are excluded while subjects are participating in the study.

Prior to study enrollment and throughout the duration of study participation:

- Any HBV vaccine (licensed or experimental)

Within 30 days before study drug administration at the Day 1 visit and throughout the duration of study participation:

- Immunosuppressant (including but not limited to corticosteroids at a prednisolone equivalent dose > 20 mg/day)
- Immunization with attenuated vaccines (e.g., Mumps, Measles, Rubella), except COVID vaccines
- Any investigational drug or biological product other than the study product(s) used during this study
- Any herbal remedies that may cause fluctuations of liver test results

Within 90 days before study drug administration at the Day 1 visit and throughout the duration of study participation:

- Blood products or immunoglobin

6.6. Safety Follow-up and Management of Reactions to Study Drug

The following safety observation procedures will be performed immediately following study drug administration and during the follow-up period for all subjects:

- Subjects will remain in the clinic for at least one hour post study drug administration. The observation period will include an assessment for rare adverse reactions.
- Any unusual signs or symptoms reported during the initial one hour of observation will prompt continued close monitoring.

- Based on their condition, subjects may be asked to remain in the clinic for more than one hour after the study drug administration (reason will be recorded in source data).
- All data (including assessment of solicited local and systemic reactions) will be recorded in the source document during and after the post-observation period.

A measurement device template (in mm) for measuring solicited local reactions (erythema [redness] and swelling), and a thermometer for measuring daily temperature, will be provided at the first study drug administration visit, study Day 1. Subjects will be advised to record their temperature in the diary card.

Refer to Sections 8.3 and 10.3 for further details on the recording, assessment, and reporting of AEs.

6.7. Treatment of Study Intervention Overdose

An overdose is defined as an accidental or intentional administration of a quantity of a medicinal product given per administration or cumulatively which is above the maximum recommended dose as per protocol. In the event of an overdose of study medication, the investigator should use clinical judgement in treating the overdose and contact the study medical monitor.

No specific treatment guidelines for overdose and toxicity management are provided by the Sponsor. The investigator is advised to refer to the relevant document(s) for detailed information pertaining to the study drug being used in this study. Such documents may include, but not be limited to, the IB or equivalent document provided by the Sponsor.

6.8. Intervention after the End of the Study

The Sponsor will not provide post-study treatment. The investigator is responsible for ensuring that consideration has been given to the post-study care of the subject's medical condition.

7. DISCONTINUATION OF STUDY INTERVENTION AND SUBJECT DISCONTINUATION/WITHDRAWAL

7.1. Discontinuation of Study Intervention

Discontinuation of study intervention does not mean withdrawal from the study.

If a subject discontinues both investigational products (BRII-179 or placebo) and PEG-IFN-α treatment, the subject will be encouraged to continue to attend the required study visits and procedures for the remaining duration of study participation in Follow-up Period.

Subjects who have discontinued study drugs may be reassessed for possible re-institution of study drug dosing not to exceed the protocol defined number of doses, dosing interval, or dosing period duration. Subjects who have discontinued study drugs will be assessed for NrtI discontinuation as described in Section 4.1.2.1 upon continued study participation into the Follow-up Period. If NrtI discontinuation criteria are met, 48-week NrtI discontinuation monitoring should be completed according to the protocol.

If continued study participation is not possible or acceptable to the subject or investigator, please refer to Section 7.2 Subject Discontinuation/Withdrawal from the Study.

7.1.1. Criteria for Discontinuation of Study Intervention

Subjects who have received at least one dose of BRII-179 (VBI-2601) will have additional doses withheld but continue to undergo follow-up monitoring in the study if any of the following criterion is met:

- Discontinuation of study intervention requested by the subject or the subject's legal representative.
- Signs or symptoms of severe (NCI-CTCAE Grade 3 or higher) hypersensitivity or anaphylaxis
- Significant abnormalities in liver chemistry, defined as any of the following are met:
  - Serum ALT > 10 × ULN
  - Serum ALT or AST > 3 × ULN with a concomitant total bilirubin > 2 × ULN or INR > 1.5
- Any clinical manifestations of hepatic decompensation

The Sponsor may request DSMB evaluation of specific data in the event of liver flare to fully characterize transaminase elevations. The subject may receive remaining doses of study drug only after DSMB approval.

- The subject has a medical condition or personal condition that, in the opinion of the investigator and/or the Sponsor, would pose an unnecessary risk to the subject if the investigational intervention is continued.

- The subject's serum pregnancy test is confirmed to be positive.

7.2. Subject Discontinuation/Withdrawal from the Study

- A subject may withdraw from the study at any time at his/her own request or may be withdrawn at any time at the discretion of the investigator for safety, behavioral, compliance, or administrative reasons. This is expected to be uncommon.
- At the time of discontinuing from the study, if possible, an Early Termination Visit should be performed (Section 8.1.7) for data to be collected at the time of study discontinuation and follow-up. The subject will be permanently discontinued from both the study intervention and from the study at that time.
- If the subject withdraws consent for disclosure of future information, the sponsor may retain and continue to use any data collected before such a withdrawal of consent.
- If a subject withdraws from the study, he/she may request destruction of any samples taken and not tested, and the investigator must document this in the site study records.
- Subjects who are withdrawn from the study will NOT be replaced and cannot be rescreened.
- The primary reason for subject withdrawal will be recorded in the eCRF. Primary reasons for withdrawal will be categorized as:
    - Adverse event
    - Protocol deviation
    - Withdrew consent, not due to an AE
    - Lost to follow up
    - Study closed/terminated

7.2.1. Lost to Follow up

A subject will be considered lost to follow-up if he or she repeatedly fails to return for scheduled visits and is unable to be contacted by the study site.

The following actions must be taken if a subject fails to return to the clinic for a required study visit:

- The site must attempt to contact the subject and reschedule the missed visit as soon as possible and counsel the subject on the importance of maintaining the assigned visit schedule and ascertain whether or not the subject wishes to and/or should continue in the study.
- Before a subject is deemed lost to follow up, the investigator or designee must make every effort to regain contact with the subject (where possible, 3 telephone calls and, if necessary, a certified letter to the subject’s last known mailing address or local equivalent methods). These contact attempts should be documented in the subject’s medical record.
- Should the subject continue to be unreachable, only then will he/she be considered to have withdrawn from the study with a primary reason of “Lost to Follow-up”.

Discontinuation of specific sites or of the study as a whole are handled as part of Section 10.1.7, Appendix 1.

8. STUDY ASSESSMENTS AND PROCEDURES

8.1. Schedule of Assessments

Study procedures and their timing are summarized in the SoAs (Section 1.5). Adherence to the study design requirements, including those specified in the SoAs, is essential and required for study conduct. Protocol waivers or exemptions are not allowed. Immediate safety concerns should be discussed with the sponsor immediately upon occurrence or awareness to determine if the subject should continue or discontinue study drug.

8.1.1. Screening

Written informed consent must be obtained prior to initiation of any study procedures. All screening evaluations must be completed and reviewed to confirm that potential subjects meet all eligibility criteria. The investigator will maintain a screening log to record details of all subjects screened and to confirm eligibility or record reasons for screening failure.

Screening will be performed no more than 4 weeks prior to randomization and will include written consent, determination of subject eligibility, collection of demographics and medical history/ current medical conditions as well as a physical examination, vital signs, 12-lead ECG, clinical laboratory tests, and other assessments per the SoA (Section 1.5).

Adverse events will be collected from the time of signing the consent until last visit. After informed consent has been obtained but prior to initiation of study drug, untoward medical occurrence, if related to study procedure, will be recorded as AE; otherwise, will be recorded as medical history. All SAEs must be collected from the time of consent until end of study.

8.1.2. Randomization (Day 1)

Eligible subjects will be randomized 1:1 to either 40 µg of BRII-179 administrated IM or placebo administrated IM. Subjects will receive their first dose of study drug on Day 1, however subject eligibility must be confirmed prior to study drug administration and blood sample collections will be made prior to study drug administration and sent to central laboratory for analysis. Such central laboratory results will be available in a few days, hence the evaluation of these lab results cannot be performed on Day 1. Other assessments to be conducted on Day 1 are detailed in the SoA (Section 1.5.1).

8.1.3. Dosing Period

All subjects will receive BRII-179 (VBI-2601) or placebo administered IM every 3 weeks for a total of 7 doses. Subjects will receive PEG-IFN-α at the indicated dose level weekly and discontinue investigational treatment with BRII-179 or placebo at Week 18 and discontinue PEG-IFN- α at Week 24; while NrtI therapy will continue into the Follow-up Period.

Subjects will return to the study site clinic on an outpatient basis for antiviral activity monitoring and safety assessment as specified in the SoA (Section 1.5.1).

Prior to study drug administration, there will be a review of ongoing eligibility to receive the study drug and continue with study participation. Diary cards will be collected and there will be a review of any local reaction (including redness, swelling, pain, itchiness and tenderness, etc.) and systemic reaction (tiredness, diarrhea, nausea/vomiting, headache, muscle pain, etc,) related to BRII-179 (VBI-2601) or placebo administration. Blood samples will be collected prior to each study drug administration.

8.1.4. Follow-up Period

Subjects will return to the investigative site for the specified assessments per the SoA (Section 1.5.1), including but not limited to physical examination, vital signs, clinical laboratory tests, and review of adverse events and concomitant medications.

Subjects will be assessed by the investigator whether they are eligible for discontinuation of NrtI therapy starting at Week 36 and every four weeks until Week 48. Subjects that have NOT achieved NrtI discontinuation criteria by Week 48 will be discharged from the study.

8.1.5. NrtI Discontinuation Monitoring Period

Eligible subjects who meet NrtI discontinuation criteria will be informed by the investigator to discontinue their NrtI medications on the first day of their scheduled Day 1 visit of the NrtI Discontinuation Monitoring Period. These subjects will follow the NrtI Discontinuation Monitoring Period SoA (Section 1.5.2).

Subjects who discontinue from their NrtI therapy will be followed in the NrtI Discontinuation Monitoring Period for an additional 48 weeks from the day of NrtI discontinuation. Subjects who are retreated with NrtI therapy should continue to be followed per SoA (Section 1.5.2) for the duration of the 48-week NrtI Discontinuation Monitoring Period.

8.1.6. Unscheduled Visits

Subjects may be asked to come for an unscheduled visit, for clinical assessment of an adverse event for example, if warranted, as determined by the investigator. The unscheduled visit will be recorded in the eCRF.

8.1.7. Early Termination Visit

In cases where a subject prematurely discontinues from the study, the investigator must make every effort to have the subject return to the study site within 14 days for an Early Termination (ET) Visit. Assessments for ET visits will include but not limited to physical examination, pregnancy test, clinical laboratory tests, and other assessments per the SoA (Section 1.5).

The English translation is for reference only, in case of any inconsistency the Chinese version shall prevail

8.2. Detailed Description of Study Procedures

8.2.1. Medical History

A complete medical history, including details regarding illnesses and allergies, date(s) of onset, and whether condition(s) is currently ongoing (current medical conditions), medication history, including prior HBV treatment history and HBV genotype history (if available), will be collected on all subjects during screening.

8.2.2. Physical Examinations

A complete physical examination will include, at a minimum, general appearance, head, neck, chest/respiratory, heart/cardiovascular, gastrointestinal/liver, extremities, skin, and neurological assessments. Height and weight will also be measured and recorded. Body mass index will be calculated from height and weight.

A symptom-directed physical examination will be performed according to investigator discretion.

8.2.3. FibroScan

To exclude the presence of significant liver fibrosis and cirrhosis, subjects will have a FibroScan evaluation during screening. Subject with a FibroScan result of ≤ 9 kPa at screening can be considered without significant liver fibrosis and cirrhosis. If screening FirboScan result > 9 kPa, however, the subject has a result of ≤ 9 Kpa from a FibroScan evaluation performed within 6 months prior to screening or liver biopsy performed within 1 year prior to screening that confirm the absence of Metavir F3 fibrosis or F4 cirrhosis, significant liver fibrosis and cirrhosis can be excluded as well. If a subject has had both procedures in the specified timeframes prior to screening, the most recent result should be used to determine eligibility.

A FibroScan will also be conducted at the Week 48/ ET3 Visit in the NrtI Discontinuation Monitoring Period.

8.2.4. Abdominal Ultrasound

Abdominal Ultrasound examinations will be performed as outlined in SoA (Section 1.5).

8.2.5. Alcohol Intake Assessment

Alcohol intake during the study will be recorded.

8.2.6. Vital Signs

Temperature (oral preferred), pulse rate, respiratory rate, and blood pressure will be assessed after the subject has rested comfortably for approximately 5 minutes. Pulse rate and blood pressure measurements will be assessed with a completely automated device. Manual techniques will be used only if an automated device is not available. Blood pressure

should be based on 2 separate measurements. When scheduled at the same timepoints, vital signs and 12-lead ECG must be assessed first before physical examinations, blood sample collections, and urine collection.

8.2.7. Electrocardiograms

12-lead ECGs will be recorded and reviewed on-site by the investigator as outlined in SoA (Section 1.5).

8.2.8. Laboratory Assessments

8.2.8.1. Screening Viral Serology

The screening viral serology parameters will include chronic HBV infection and active infection with HIV, HCV, and hepatitis Delta virus. Chronic HBV infection is defined as serum HBsAg ≥ 6 months.

8.2.8.2. Clinical Safety Laboratory Assessments

Fasting clinical safety laboratory tests to be performed are described in Section 10.2.

The investigator can conduct additional clinical assessment for the adverse reactions of PEG-IFN-α, including but not limited to radiological examination, ophthalmology assessment, immunological marker test, blood glucose and psychological assessment. These assessments can be performed at local hospital.

The investigator must review the laboratory report, document this review, and record any clinically significant changes occurring during the study in the AE section of the eCRF. The laboratory reports must be filed with the source documents. Clinically significant abnormal laboratory findings are those which are not associated with the underlying disease, unless judged by the investigator to be more severe than expected for the subject's condition.

In the event of an unexplained clinically relevant abnormal laboratory test occurring after study drug administration, the test should be repeated and followed at investigator discretion until it has returned to normal or baseline, and/or a diagnosis is made to adequately explain the abnormality.

8.2.8.3. Pregnancy Test

A pregnancy test or confirmation of post-menopausal status must be confirmed for all female subjects. Post-menopausal status is defined as 12 months with no menses without alternative medical cause. Pregnancy tests will be performed for WOCBP only. Serum and urine pregnancy testing will be performed per the SoAs (Section 1.5) and any time pregnancy is suspected. A WOCBP who is known to be pregnant or who does not have a negative pregnancy test at screening is not eligible for study participation. During the study, the results of these pregnancy tests must be known prior to study drug administration. A

The English translation is for reference only, in case of any inconsistency the Chinese version shall prevail

WOCBP determined to be pregnant while on study will be followed until the pregnancy outcome is known, as described in Section 8.3.5.1.

8.2.8.4. Assessment of Antiviral Activity

Assessment of antiviral activity will include HBsAg, HBsAb, and HBV DNA.

Details regarding the processing, shipping, and analysis of the samples will be provided in the Laboratory Manual.

8.2.8.5. Cytokine/chemokine Biomarkers Assessment

Blood samples for potential cytokine/chemokine biomarkers assessment will be collected per the SoAs (Section 1.5). See the Laboratory Manual for instructions on sample processing and storage.

8.2.9. Laboratory Events of Clinical Interest

A clinical laboratory event of clinical interest (ECI) is a laboratory value of scientific and medical interest specific to understanding of the investigational product and may require additional follow-up testing, close monitoring, and rapid communication by the investigator to the Sponsor.

From Day 1 of dosing to the last visit, any laboratory events of ECI will be monitored, defined as the following:

- ALT > 3x ULN, if Day 1 pre-dose ALT ≤ ULN
- ALT > 2x baseline, if Day 1 pre-dose ALT > ULN

All laboratory ECIs must be reported to the Sponsor within 24 hours of the investigator's knowledge of the event. See Section 10.3.5. In addition, the assessments outlined in Section 10.5 should be performed as soon as possible and no later than two weeks after the initial ALT value meeting criteria for a laboratory ECI.

The laboratory ECI will also be evaluated by the investigator to determine if the event meets criteria for an AE. If AE criteria are met, all clinical sequelae in relation to these laboratory ECIs will be reported as AEs or SAEs, if applicable.

8.3. Adverse Events and Serious Adverse Events

The investigator and any qualified designees are responsible for detecting, documenting, and recording events that meet the definition of an AE or SAE and remain responsible for following up AEs that are serious, considered related to the study drug or study procedures, or that caused the subject to discontinue the study drug or study.

The definitions and procedures for reporting, recording, evaluating, and follow-up of an AE or SAE can be found in Section 10.3, Appendix 3.

8.3.1. Time Period and Frequency for Collecting AE and SAE Information

All SAEs will be collected from the signing of the informed consent form (ICF) until the last visit at the time points specified in the SoAs (Section 1.5).

All AEs will be collected from the signing the ICF until the last visit at the time points specified in the SoAs (Section 1.5).

After informed consent has been obtained but prior to initiation of study drug, untoward medical occurrence, if related to study procedure, should be recorded as AE; otherwise, should be recorded as medical history.

All SAEs will be recorded and reported to the sponsor or designee immediately and under no circumstance should this exceed 24 hours, as indicated in Section 10.3, Appendix 3. The investigator will submit any updated SAE data to the sponsor within 24 hours of it being available.

Investigators are not obligated to actively seek AEs or SAEs after study participation. However, if the investigator learns of any SAE, including a death, at any time after a subject has been discharged from the study, and he/she considers the event to be reasonably related to the study drug or study participation, the investigator must promptly notify the sponsor.

8.3.2. Method of Detecting AEs and SAEs

The method of recording, evaluating, and assessing causality of AEs and SAEs and the procedures for completing and transmitting SAE reports are provided in Section 10.3.

Care will be taken not to introduce bias when detecting AEs and/or SAEs. Open-ended and non-leading verbal questioning of the subject is the preferred method to inquire about AE occurrences.

8.3.3. Follow-up of AEs and SAEs

After the initial AE/SAE report, the investigator is required to proactively follow each subject at subsequent visits/contacts. All SAEs will be followed until resolution, until the condition stabilizes, until the event is otherwise explained, or until the subject is lost to follow-up (as defined in Section 7.2). Further information on follow-up procedures is provided in Section 10.3, Appendix 3.

8.3.4. Regulatory Reporting Requirements for SAEs

- Prompt notification by the investigator to the sponsor of a SAE is essential so that legal obligations and ethical responsibilities towards the safety of subjects and the safety of a study drug under clinical investigation are met.
- The sponsor has a legal responsibility to notify both the local regulatory authority and other regulatory agencies about the safety of a study drug under clinical investigation.

The English translation is for reference only, in case of any inconsistency the Chinese version shall prevail

The sponsor will comply with country-specific regulatory requirements relating to safety reporting to the regulatory authority, Institutional Review Boards (IRB)/Independent Ethics Committees (IEC), and investigators.

- For all studies except those utilizing medical devices investigator safety reports must be prepared for suspected unexpected serious adverse reactions (SUSAR) according to local regulatory requirements and sponsor policy and forwarded to investigators as necessary.
- An investigator who receives an investigator safety report describing a SAE or other specific safety information (e.g., summary or listing of SAEs) from the sponsor will review and then file it along with the Investigator's Brochure and will notify the IRB/IEC, if appropriate according to local requirements.

8.3.5. Special Situations

Special situation reports include all reports of medication error, abuse, misuse, overdose, reports of adverse events associated with product complaints, and pregnancy reports (Including pregnancies in female subjects and pregnancies in female partners of male subjects) regardless of an associated AE.

Medication error is any unintentional error in the prescribing, dispensing, or administration of a medicinal product while in the control of the health care provider, subject, or consumer.

Abuse is defined as persistent or sporadic intentional excessive use of the medicinal product by a subject.

Misuse is defined as any intentional and inappropriate use of a medicinal product that is not in accordance with the protocol instructions or the local prescribing information.

An overdose is defined as an accidental or intentional administration of a quantity of a medicinal product given per administration or cumulatively which is above the maximum recommended dose as per protocol. In cases of a discrepancy in drug accountability, overdose will be established only when it is clear that the subject has taken the excess dose(s). Overdose cannot be established when the pharmacist cannot account for the discrepancy except in cases in which the investigator has reason to suspect that the additional dose(s) were administered to the subject.

Product complaint is defined as complaints arising from potential deviations in the manufacture, packaging, or distribution of the medicinal product.

8.3.5.1. Pregnancy

- Details of all pregnancies in female subjects will be collected after the start of study drug through 12 weeks after last study drug administration.
- If a pregnancy is reported, the investigator should inform the sponsor within 24 hours of learning of the pregnancy and should follow the procedures outlined in Section 10.4.

- Abnormal pregnancy outcomes (e.g., spontaneous abortion, fetal death, stillbirth, congenital anomalies, ectopic pregnancy) are considered SAEs.

8.3.5.2. Other Special Situations

- All other special situations must be reported to the sponsor within 24 hours of the investigator becoming aware of the situation. These reports apply to study drug only.
- Special situations involving medications other than study drug do not need to be reported; however, for special situations that result in AEs due to a medication other than study drug, the AE should be reported.
- Any inappropriate use of concomitant medications prohibited by this protocol should not be reported as "misuse," but may be more appropriately documented as a protocol deviation.
- All clinical results in relation to these special situation reports will be reported as AEs or SAEs at the same time. Details of the symptoms and signs, clinical management, and outcome will be reported, when available.

8.4. Pharmacodynamics

Pharmacodynamic parameters are not evaluated in this study.

8.5. Genetics

Human genetics are not evaluated in this study.

8.6. [Health Economics] OR [Medical Resource Utilization and Health Economics]

Health Economics/Medical Resource Utilization and Health Economics parameters are not evaluated in this study.

9. STATISTICAL CONSIDERATIONS

9.1. Statistical Hypotheses

9.1.1. Phase 2a Part

The primary endpoint for the Phase 2a part is percentage of subjects with HBsAg loss at completion of 24-week PEG-IFN-α treatment. The null hypothesis is that there is no difference in the percentage of HBsAg loss at completion of 24-week PEG-IFN-α treatment between BRII-179 + PEG-IFN-α + NrtI and BRII-179 placebo + PEG-IFN- α +NrtI groups.

9.1.2. Phase 2b Part

The primary endpoint for the Phase 2b part is sustained HBsAg and HBV DNA loss (defined as HBsAg and HBV DNA undetectable for at least 24 weeks) after NrtI discontinuation. The null hypothesis is that there is no difference in the percentage of sustained HBsAg and HBV DNA loss between BRII-179+PEG-IFN- α + NrtI and BRII-179 placebo + PEG-IFN- α + NrtI groups.

9.2. Sample Size Determination

9.2.1. Phase 2a Part

The early futility evaluation based on an intermediate outcome of efficacy using the HBsAg loss at completion of 24-week PEG-IFN-α treatment will be conducted when approximately 120 subjects complete 18 weeks BRII-179 or placebo investigational treatment and 24 weeks PEG-IFN-α treatment or withdraw the study/discontinue study drugs prior to last scheduled PEG-IFN-α treatment. prior to last scheduled PEG-IFN-α treatment.

Everest project data suggest 25% subjects can achieve HBsAg loss after 24 weeks treatment of PEG-IFN-α + NrtI. The 20% difference in percentage of HBsAg loss is considered to be a clinically meaningful increase in HBsAg clearance with the addition of BRII-179 to PEG-IFN-α and NrtI therapy, based on this point, with the assumption of a 25% of HBsAg loss by placebo in combination with PEG-IFN-α and NrtI treatment, a 45% of HBsAg loss by BRII-179 in combination with PEG-IFN-α and NrtI treatment, and one-sided Type I error $\alpha = 0.3$. The sample size in range of 105 to 120 can provide power of more than 95% to reject the null hypothesis (Table 1). In this study the planned sample size will be approximately 120. In this sample size estimation, power calculation method is normal approximation and test method is Z-test (SAS 9.4).

The English translation is for reference only, in case of any inconsistency the Chinese version shall prevail

Table 1. The power calculated based on different sample size

HBsAg loss		Total Sample Size	Power	Alpha (1-sided)
Placebo + PEG-IFN-α + NrtI	BRII-179 + PEG-IFN-α + NrtI			
25%	45%	105	95.1%	0.3
		110	95.7%	
		115	96.0%	
		120	96.5%	

9.2.2. Phase 2b Part

Currently, there are limited data available regarding sustained HBsAg loss and sustained HBV DNA undetectable (defined as HBsAg and HBV DNA undetectable for at least 24 weeks) after a total of 48 weeks treatment of PEG-IFN- α in planned target population in this study. Table 2 displays a range of sample size for sustained HBsAg and HBV DNA loss with PEG-IFN-α + NrtI treatment ranging from 22% to 35% and a minimum of clinically meaningful increase of 15% after adding BRII-179 to PEG-IFN- α + NrtI treatment.

Table 2. Sample Size Calculation for Four Scenarios of Sustained HBsAg Loss

Expected Sustained HBsAg loss and HBV DNA undetectable (%)		Total Sample Size (Per Arm)	Power	Alpha (1-sided)
Placebo + PEG-IFN- α + NrtI	BRII-179 + PEG-IFN-α + NrtI			
22	37	412 (206)	90%	0.025
26	41	442 (221)		
**30**	**45**	**466 (233)**		
35	50	486 (243)		

Based on the following assumptions, a 15% increase in the percentage of sustained HBsAg loss and HBV DNA undetectable at a one-sided Type I error level of 2.5%. It is assumed 466 subjects enrolled will provide 90% power to reject the null hypothesis, further decided to enrol a total of 480 subjects, which will provide with approximately 91% power to reject the null hypothesis.

Proportion of sustained HBsAg and HBV DNA loss in PEG-IFN-α + NrtI group is 30%.

A clinically meaningful increase in sustained HBsAg loss is 15% with add-on of BRII-179 treatment.

Non-binding stopping guideline for futility using a moderately aggressive Type II error spending function (Gamma=-2), implemented using the Lan-DeMets O'Brien-Fleming approximation spending function approach.

The sample size calculation will be performed using proc seqdesign in SAS9.4 and it is planned to perform 3 interim analysis and 1 final analysis.

Data analyses will be performed when approximately 120 subjects enrolled in Phase 2a part complete the last scheduled PEG-IFN-α treatment or withdraw from the study/discontinue study drugs prior to last scheduled PEG-IFN-α treatment.

The sample size for Phase 2b part may be re-estimated after evaluation of efficacy data from subjects in Phase 2a part.

9.3. Data Monitoring Guidelines for Independent DSMB

An independent DSMB will review the safety and efficacy data of Phase 2a part of the study. Based on the results of the Phase 2a part, the DSMB will build the rationale for the recommendation on whether to proceed to Phase 2b part or not. DSMB will perform data review when approximately 120 subjects enrolled in the Phase 2a part complete the last scheduled PEG-IFN-α treatment and HBsAg and HBV DNA results are available or withdraw from the study prior to last scheduled PEG-IFN-α treatment.

If the trial proceeds to Phase 2b part, the DSMB will continue to review interim data on a regular basis and use pre-specified guidelines to determine clear evidence of efficacy for the primary outcome, and if so the DSMB will provide recommendation to unblind the trial results. Conversely, the DSMB may provide recommendation to discontinue the BRII-179 treatment if futility assessments indicate that there is low probability to achieve statistical significance for the primary endpoint of sustained HBsAg loss with BRII-179 treatment.

9.4. Primary Endpoint and Safety Data Analysis in Phase 2 Part

An early futility analysis based on HBsAg loss will be performed on approximately 120 subjects who complete last scheduled PEG-IFN-α treatment or withdraw from the trial/discontinue study drug prior to last scheduled PEG-IFN-α treatment. The sample size of 120 subjects can provide above 95% power to reject the null hypothesis at the 0.30 (1-sided) level of significance.

Brii will make the decision on whether to proceed to Phase 2b part or not based on HBsAg loss futility analysis result at completion of last scheduled PEG-IFN-α treatment and safety data analysis, as well as the recommendation provided by the independent DSMB.

9.4.1. Monitoring Guidelines for the Primary Endpoint in Phase 2b Part

As a guideline, stopping boundaries will be provided to the DSMB to monitor the primary endpoint (sustained HBsAg loss and sustained HBV DNA undetectable). For monitoring overwhelming benefit, the Lan-DeMets spending function analogue of the O'Brien-

Fleming boundaries will be used. The Lan-DeMets boundary used will be chosen to preserve a 1-sided 0.025 level of significance. For computing the Lan-DeMets boundary, the information fraction at each of 3 interim analyses will be the number of subjects enrolled at the interim analysis, i.e., 120, 240, and 360 subjects who complete 48 weeks of follow-up or withdraw before week 48 (for those who do not enter NrtI withdrawal follow-up period) or complete at least 24 weeks of follow-up or withdraw from the study prior to at least 24 weeks of follow-up after NrtI discontinuation (for those who enter NrtI discontinuation follow-up period), respectively.

Futility analyses will be performed. The aim of these analyses will be to consider whether BRII-179 should be discontinued due to a low probability of achieving statistical significance for the primary endpoint of sustained HBsAg loss. Conditional power calculations of sustained HBsAg loss will be presented. Typical futility guidelines recommend stopping a trial when conditional power is below 10%-15%These analyses will be presented to the DSMB by the unblinded statisticians.

9.5. Analysis Sets

The following analysis sets are defined:

Population	Description
All Subjects Screened	All subjects, for whom a record exists on the clinical database, will be included in All Subjects Screened.<br>It will be used for reporting study disposition, inclusion and exclusion criteria deviations, etc.
All Subjects Randomized	All subjects, who are randomized will be included in All Subjects Randomized. It will be used for reporting demographics, baseline characteristics, medical history, etc.
Full Analysis Set	All subjects, who are randomized and received at least one dose of BRII-179 or placebo, will be included in Full Analysis Set. It will be used as the primary analysis set of all efficacy endpoints.
Serologically Evaluable Full Analysis Set	The Serologically Evaluable Full Analysis Set for HBsAg Loss will include all subjects who were randomized and received at least one dose of BRII-179 or placebo, and with HBsAg ⩾ 0.05 IU/mL at baseline. The analysis based on Serologically Evaluable Full Analysis Set will be considered as sensitivity analysis.
Per Protocol Set	All subjects, who are randomized; and not identified with major protocol deviation that substantially affects the treatment effect of study medication; received all planned doses of BRII-179 or placebo; will be included in Per Protocol Set. It will be used as analysis set for key efficacy endpoint analyses. The analysis based on Per Protocol analysis set will be considered as sensitivity analysis.
Safety Analysis Set	All subjects randomly assigned to study drug and who take at least 1 dose of study drug will be included in Safety Analysis Set. It will be used for reporting safety parameters.

9.6. Statistical Analyses

This section outlines the general analytic strategy for the study. The statistical analysis plan (SAP) will be finalized prior to Database Lock (DBL) and it will include a more detailed description of the statistical analyses described in this section. This section is a summary of the planned statistical analyses of the most important endpoints including primary and key secondary endpoints.

9.6.1. General considerations

Unless otherwise stated, the following rule will apply:

Summaries and displays will be presented by treatment group.

For categorical variables, the frequency and percentage will be applied as descriptive statistics.

For continuous variables, number of subjects, the mean, standard deviation, median, inter-quartiles, and ranges will be applied as descriptive statistics.

Specific algorithm regarding how missing HBsAg and HBV DNA values will be handled in deriving the primary efficacy endpoints and sustained HBsAg loss will be detailed in the statistical analysis plan.

Unless specified otherwise, missing values will be handled using the Data-As-Observed (DAO) approach, that is, any missing value will be excluded from the analysis, except for the baseline value, which will be replaced with the latest pre-treatment result, if available.

Only scheduled visits will be presented of summaries; unscheduled visits will be listed but will not be summarized.

9.6.2. Analysis of the Primary Efficacy Endpoint for Phase 2a Part

The primary efficacy endpoint of the phase 2a part, percentage of HBsAg loss at completion of PEG-IFN-α treatment, will be analysed based on Cochran-Mantel-Haenszel method, stratified at randomization by screening HBsAg level (3 categories). The differences in the percentage of HBsAg loss and associated 95% confidence interval will be presented. If too few cases in a stratum result in failure of stratified analysis, it will be considered to remove the stratification factors and a non-stratified analysis or Fisher's exact probability method will be used to compare the percentage of HBsAg loss between the two groups.

9.6.3. Analysis of the Primary Efficacy Endpoint for Phase 2b Part

The primary efficacy endpoint of the phase 2b part, percentage of sustained HBsAg loss and sustained HBV DNA undetectable defined as undetectable HBsAg and HBV DNA for at least 24 weeks after NrtI discontinuation. Subjects who do not meet NrtI withdrawal criteria or are retreated with NrtI after NrtI discontinuation will be classified as not achieving sustained HBsAg loss and HBV DNA undetectable. The primary endpoint will be analyzed based on Cochran-Mantel-Haenszel method, stratified at randomization by screening HBsAg level (3 categories: ≥0.05 to < 1 IU/mL, ≥1 to < 10 IU/mL, and ≥10 to <100 IU/mL). The differences in the percentage of sustained HBsAg loss and sustained HBV DNA undetectable together with associated adjusted 95% confidence interval will be presented.

9.6.4. Analyses of Secondary Efficacy Endpoints and Subgroups

Percentage of subjects meeting NrtI discontinuation criteria and other secondary endpoints assessed as proportion will be analysed in the same way as for the primary endpoint.

Time to event endpoints will be compared using stratified log-rank test and continuous endpoints will be compared using Wilcoxon ranks sum test.

Additionally, subgroup analysis by age, gender, and HBsAg level at screening (3 categories: ≥0.05 to < 1 IU/mL, ≥1 to < 10 IU/mL, and ≥10 to <100 IU/mL) will be conducted with respect to the primary endpoint.

The English translation is for reference only, in case of any inconsistency the Chinese version shall prevail

9.6.5. Tertiary Efficacy Endpoint and Subgroup Analyses of Phase 2a Part

When applicable, selected data relating to the biomarker analysis of cytokine/chemokines will be summarized with descriptive statistics

9.6.6. Analysis of Safety Outcome

The Safety Analysis population will be used for clinical review of safety relevant parameters.

A treatment-emergent adverse event (TEAE) is any AE that has an onset date that is on or after the day of study drug administration or any AE that has been present before treatment and worsens relative to the pre-treatment state after administration of study drug.

Descriptive statistics will be used for the safety analysis, and all adverse events (AEs) will be summarized and described according to the latest version of the Medical Dictionary for Regulatory Activities (MedDRA) codes. Coded treatment-emergent adverse events, investigational product-related adverse events, serious adverse events, and adverse events leading to subject withdrawal will be summarized and described by System Organ Class (SOC), Preferred Term (PT), and group, and number and percentage of subject with AE will be summarized. Treatment-emergent adverse events, investigational product-related adverse events, serious adverse events, and adverse events leading to subject withdrawal will also be summarized by severity. All adverse events will be tabulated. Graded laboratory abnormalities will be defined using CTCAE Version 5.0 grading scale. Laboratory tests, vital signs, physical examinations, and 12-lead electrocardiograms will be summarized descriptively. For vital signs and laboratory tests (quantitative measures), observed values and their changes from baseline will be summarized descriptively. Subjects with post-treatment abnormalities of clinical significance will be listed for all tests.

10. SUPPORTING DOCUMENTATION AND OPERATIONAL CONSIDERATIONS

10.1. Appendix 1: Regulatory, Ethical, and Study Oversight Considerations

10.1.1. Regulatory and Ethical Considerations

- This study will be conducted in accordance with the protocol and with the following:
    - Consensus ethical principles derived from international guidelines including the Declaration of Helsinki and Council for International Organizations of Medical Sciences (CIOMS) International Ethical Guidelines
    - Applicable ICH Good Clinical Practice (GCP) Guidelines
    - Applicable laws and regulations

The English translation is for reference only, in case of any inconsistency the Chinese version shall prevail

- The protocol, protocol amendments, ICF, Investigator Brochure, and other relevant documents (e.g., advertisements) must be submitted to an IRB/IEC by the investigator and reviewed and approved by the IRB/IEC before the study is initiated.
- Any amendments to the protocol will require IRB/IEC approval before implementation of changes made to the study design, except for changes necessary to eliminate an immediate hazard to study subjects.
- The investigator will be responsible for the following:
    - Providing written summaries of the status of the study to the IRB/IEC annually or more frequently in accordance with the requirements, policies, and procedures established by the IRB/IEC
    - Notifying the IRB/IEC of SAEs or other significant safety findings as required by IRB/IEC procedures
    - Providing oversight of the conduct of the study at the site and adherence to requirements of 21 CFR, ICH guidelines, the IRB/IEC, European regulation 536/2014 for clinical studies (if applicable), and all other applicable local regulations

10.1.2. Financial Disclosure

Investigators and sub-investigators will provide the sponsor with sufficient, accurate financial information as requested to allow the sponsor to submit complete and accurate financial certification or disclosure statements to the appropriate regulatory authorities. Investigators are responsible for providing information on financial interests during the course of the study and for 1 year after completion of the study.

10.1.3. Informed Consent Process

- The investigator or his/her representative will explain the nature of the study to the subject or his/her legally authorized representative and answer all questions regarding the study.
- Subjects must be informed that their participation is voluntary. Subjects or their legally authorized representative will be required to sign a statement of informed consent that meets the requirements of 21 CFR 50, local regulations, ICH guidelines, Health Insurance Portability and Accountability Act (HIPAA) requirements, where applicable, and the IRB/IEC or study center.
- The medical record must include a statement that written informed consent was obtained before the subject was enrolled in the study and the date the written consent was obtained. The authorized person obtaining the informed consent must also sign the ICF.
- Subjects must be re-consented to the most current version of the ICF(s) during their participation in the study.

- A copy of the ICF(s) must be provided to the subject or the subject's legally authorized representative.

10.1.4. Data Protection

- Subjects will be assigned a unique identifier by the sponsor. Any subject records or datasets that are transferred to the sponsor will contain the identifier only; subject names or any information which would make the subject identifiable will not be transferred.
- The subject must be informed that his/her personal study-related data will be used by the sponsor in accordance with local data protection law. The level of disclosure must also be explained to the subject who will be required to give consent for their data to be used as described in the informed consent
- The subject must be informed that his/her medical records may be examined by Clinical Quality Assurance auditors or other authorized personnel appointed by the sponsor, by appropriate IRB/IEC members, and by inspectors from regulatory authorities.

10.1.5. Data Quality Assurance

- All relevant subject data relating to the study will be recorded on printed or electronic CRF unless transmitted to the sponsor or designee electronically (e.g., laboratory data). The investigator is responsible for verifying that data entries are accurate and correct by physically or electronically signing the CRF.
- The investigator must maintain accurate documentation (source data) that supports the information entered in the CRF.
- The investigator must permit study-related monitoring, audits, IRB/IEC review, and regulatory agency inspections and provide direct access to source data documents.
- Monitoring details describing strategy (e.g., risk-based initiatives in operations and quality such as Risk Management and Mitigation Strategies and Analytical Risk-Based Monitoring), methods, responsibilities and requirements, including handling of noncompliance issues and monitoring techniques (central, remote, or on-site monitoring) are provided in the Monitoring Plan.
- The sponsor or designee is responsible for the data management of this study including quality checking of the data.
- The sponsor assumes accountability for actions delegated to other individuals (e.g., Contract Research Organizations).
- Study monitors will perform ongoing source data verification to confirm that data entered into the CRF by authorized site personnel are accurate, complete, and verifiable from source documents; that the safety and rights of subjects are being protected; and that the study is being conducted in accordance with the currently

approved protocol and any other study agreements, ICH GCP, and all applicable regulatory requirements.

- Records and documents, including signed ICFs, pertaining to the conduct of this study must be retained by the investigator. No records may be destroyed during the retention period without the written approval of the sponsor. No records may be transferred to another location or party without written notification to the sponsor.

10.1.6. Source Documents

- Source documents provide evidence for the existence of the subject and substantiate the integrity of the data collected. Source documents are filed at the investigator's site.
- Data reported on the CRF or entered in the eCRF that are transcribed from source documents must be consistent with the source documents or the discrepancies must be explained. The investigator may need to request previous medical records or transfer records, depending on the study. Also, current medical records must be available.
- Source data refer to all information recorded in the original record of certified copy of the clinical study, including clinical findings, observation results and other relevant activity records needed for reconstruction and evaluation of the clinical study.

10.1.7. Study and Site Start and Closure

The study start date is the date on which the clinical study will be open for recruitment of subjects.

The sponsor designee reserves the right to close the study site or terminate the study at any time for any reason at the sole discretion of the sponsor. Study sites will be closed upon study completion. A study site is considered closed when all required documents and study supplies have been collected and a study-site closure visit has been performed.

The investigator may initiate study-site closure at any time, provided there is reasonable cause and sufficient notice is given in advance of the intended termination.

Reasons for the early closure of a study site by the sponsor or investigator may include but are not limited to:

- Failure of the investigator to comply with the protocol, the requirements of the IRB/IEC or local health authorities, the sponsor's procedures, or GCP guidelines
- Inadequate recruitment of subjects by the investigator
- Discontinuation of further study drug development

If the study is prematurely terminated or suspended, the sponsor shall promptly inform the Investigators, the IECs/IRBs, the regulatory authorities, and any contract research organization(s) used in the study of the reason for termination or suspension, as specified

by the applicable regulatory requirements. The Investigator shall promptly inform the subject and should assure appropriate subject therapy and/or follow-up.

10.1.8. Publication Policy

The sponsor intends to publish the results of clinical trials regardless of outcome. Results may be published in peer-reviewed journals and/or presented at scientific congresses. The sponsor will comply with the requirements for publication and disclosure of clinical trial information. In accordance with standard editorial and ethical practice, the sponsor will generally support publication of multicenter studies only in their entirety and not as individual site data. Authorship will be determined by mutual agreement based upon International Committee of Medical Journal Editors criteria and guidelines [ICMJE, 2018]. Individuals who do not meet authorship criteria do not warrant named authorship. Nonauthor contributors may be acknowledged in the manuscript when published or recognized in a manner consistent with the target journal policy.

10.2. Appendix 2: Clinical Laboratory Tests

The tests detailed in Table 3 will be performed by the central laboratory. Urine pregnancy test will be performed by the local laboratory.

Investigators must document their review of each laboratory safety report.

Additional tests may be performed at any time during the study as determined necessary by the investigator or required by local regulations.

Table 3: Protocol-Required Safety Laboratory Assessments

**Hematology**	
Complete blood count with differential, including red blood cell count, hemoglobin, hematocrit, white blood cell count, count and percentage of neutrophils, lymphocytes, eosinophils, basophils, and monocytes, and platelet count	
**Chemistry**	
Albumin	Estimated glomerular filtration rate (eGFR)
Blood urea nitrogen (BUN)	Gamma glutamyl transferase (GGT)
Calcium	Glucose
Carbon dioxide/bicarbonate	Lactate dehydrogenase (LDH)
Chloride	Potassium
Creatine kinase	Sodium
Creatinine	Uric acid
**Liver Function Tests**	
Alkaline phosphatase (ALP)	Aspartate aminotransferase (AST)
Alanine aminotransferase (ALT)	Bilirubin (total and direct)
**Coagulation Parameters**	
International normalized ratio (INR)	Prothrombin time (PT)
**Urinalysis**	
Glucose	Proteins
Red blood cells (RBCs)	
**Pregnancy Testing**	
Beta-human chorionic gonadotropin (WOCBP only)	Urine pregnancy test
**Thyroid Panel**	
Thyroid stimulating hormone (TSH)[1]	

[1] Thyroid function will be tested every 12 weeks during PEG-IFN-α dosing: Screening, Week 12, and Week 24.

10.3. Appendix 3: Adverse Events: Definitions and Procedures for Recording, Evaluating, Follow-up, and Reporting

10.3.1. Definition of AE

**AE Definition**
• An AE is any untoward medical occurrence in a patient or clinical study subject, temporally associated with the use of study drug, whether or not considered related to the study drug.<br>• NOTE: An AE can therefore be any unfavorable and unintended sign (including an abnormal laboratory finding), symptom, or disease (new or exacerbated) temporally associated with the use of study drug.

**Events <u>Meeting</u> the AE Definition**
• Any abnormal laboratory test results (hematology, clinical chemistry, or urinalysis) or other safety assessments (e.g., ECG, radiological scans, vital signs measurements), including those that worsen from baseline, considered clinically significant in the medical and scientific judgment of the investigator (i.e., not related to progression of underlying disease).<br>• Exacerbation of a chronic or intermittent pre-existing condition including either an increase in frequency and/or intensity of the condition.<br>• New conditions detected or diagnosed after study drug administration even though it may have been present before the start of the study.<br>• Signs, symptoms, or the clinical sequelae of a suspected drug-drug interaction.<br>• Signs, symptoms, or the clinical sequelae of a suspected overdose of either study drug or a concomitant medication. Overdose per se will not be reported as an AE/SAE unless it is an intentional overdose taken with possible suicidal/self-harming intent. Such overdoses should be reported regardless of sequelae.

**Events <u>NOT</u> Meeting the AE Definition**
• Any clinically significant abnormal laboratory findings or other abnormal safety assessments which are associated with the underlying disease, unless judged by the investigator to be more severe than expected for the subject's condition.<br>• The disease/disorder being studied or expected progression, signs, or symptoms of the disease/disorder being studied, unless more severe than expected for the subject's condition.<br>• Medical or surgical procedure (e.g., endoscopy, appendectomy): the condition that leads to the procedure is the AE.<br>• Situations in which an untoward medical occurrence did not occur (social and/or convenience admission to a hospital).<br>• Anticipated day-to-day fluctuations of pre-existing disease(s) or condition(s) present or detected at the start of the study that do not worsen.

10.3.2. Definition of SAE

If an event is not an AE per definition above, then it cannot be an SAE even if serious conditions are met (e.g., hospitalization for signs/symptoms of the disease under study, death due to progression of disease).

**A SAE is defined as any untoward medical occurrence that, at any dose:**
**a. Results in death**
**b. Is life-threatening**<br>The term 'life-threatening' in the definition of 'serious' refers to an event in which the subject was at risk of death at the time of the event. It does not refer to an event, which hypothetically might have caused death, if it were more severe.
**c. Requires inpatient hospitalization or prolongation of existing hospitalization**<br>• In general, hospitalization signifies that the subject has been detained (usually involving at least an overnight stay) at the hospital or emergency ward for observation and/or treatment that would not have been appropriate in the physician's office or outpatient setting. Complications that occur during hospitalization are AEs. If a complication prolongs hospitalization or fulfills any other serious criteria, the event is serious. When in doubt as to whether "hospitalization" occurred or was necessary, the AE should be considered serious.<br>• Hospitalization for elective treatment of a pre-existing condition that did not worsen from baseline is not considered an AE.
**d. Results in persistent disability/incapacity**<br>• The term disability means a substantial disruption of a person's ability to conduct normal life functions.<br>• This definition is not intended to include experiences of relatively minor medical significance such as uncomplicated headache, nausea, vomiting, diarrhea, influenza, and accidental trauma (e.g., sprained ankle) which may interfere with or prevent everyday life functions but do not constitute a substantial disruption.
**e. Is a congenital anomaly/birth defect**
**f. Other important medical events:**<br>• Medical and scientific judgment should be exercised to determine whether expedited reporting is appropriate in other situations; in general, any important medical event that may not be immediately life-threatening, or result in death, or hospitalization, but for which medical interventions are required to prevent any of the above from happening, is also considered a serious adverse event. For example, intensive treatment in the emergency room or at home for allergic bronchospasm, blood dyscrasias or convulsions that do not result in hospitalisation, or development of drug dependency or drug abuse, etc.

10.3.3. Recording and Follow-Up of AE and/or SAE

AE and SAE Recording
• When an AE/SAE occurs, it is the responsibility of the investigator to review all documentation (e.g., hospital progress notes, laboratory reports, and diagnostics reports) related to the event.<br>• The investigator will then record all relevant AE/SAE information in the CRF.<br>• It is **not** acceptable for the investigator to send photocopies of the subject's medical records to the Sponsor in lieu of completion of the AE/SAE CRF page.<br>• There may be instances when copies of medical records for certain cases are requested by the Sponsor. In this case, all subject identifiers, with the exception of the subject number, will be redacted on the copies of the medical records before submission to the Sponsor.<br>• The investigator will attempt to establish a diagnosis of the event based on signs, symptoms, and/or other clinical information. Whenever possible, the diagnosis (not the individual signs/symptoms) will be documented as the AE/SAE.
**Assessment of Intensity**
The investigator will make an assessment of intensity for each AE and SAE reported during the study based on the National Cancer Institute Common Terminology Criteria for Adverse Events (NCI-CTCAE) Version 5.0.
**Assessment of Causality**
• The investigator or a qualified assistant investigator is obligated to assess the relationship between study drug and each occurrence of each AE/SAE.<br>• Relationship between AEs and the study drug: "related" should be entered when there is either (a) a *reasonable possibility* that the AE/SAE is associated with study drug **or** (b) no reasonable alternative explanation can be found. Otherwise, the relationship between AEs and the study drug should be classified as "unrelated".<br>• A "reasonable possibility" of a relationship conveys that there are facts, evidence, and/or arguments to suggest a causal relationship, rather than a relationship cannot be ruled out.<br>• The investigator will use clinical judgment to determine the relationship.<br>• Alternative causes, such as underlying disease(s), concomitant therapy, and other risk factors, as well as the temporal relationship of the event to study drug administration will be considered and investigated.<br>• The investigator will also consult the Investigator's Brochure (IB) and/or Product Information, for marketed products, in his/her assessment.<br>• For each AE/SAE, the investigator **must** document in the medical notes that he/she has reviewed the AE/SAE and has provided an assessment of causality.<br>• There may be situations in which an SAE has occurred and the investigator has minimal information to include in the initial report. However, it is very important that the investigator always make an assessment of causality for every event before the initial transmission of the SAE data.

The English translation is for reference only, in case of any inconsistency the Chinese version shall prevail

• The investigator may change his/her opinion of causality in light of follow-up information and send a SAE follow-up report with the updated causality assessment.<br>• The causality assessment is one of the criteria used when determining regulatory reporting requirements.

**Follow-up of AEs and SAEs**
• The investigator is obligated to perform or arrange for the conduct of supplemental measurements and/or evaluations as medically indicated or as requested by the Sponsor to elucidate the nature and/or causality of the AE or SAE as fully as possible. This may include additional laboratory tests or investigations, histopathological examinations, or consultation with other health care professionals.<br>• If a subject dies during participation in the study or during a recognized follow-up period, the investigator will provide the Sponsor with a copy of any post-mortem findings including histopathology.<br>• New or updated information will be recorded in the originally completed CRF.<br>• The investigator will submit any updated SAE data to the Sponsor within 24 hours of receipt of the information.

10.3.4. Reporting of SAEs

**SAE Reporting to the Sponsor via a Paper SAE Reporting Form**
• The investigator must complete a paper SAE report form and send to the Sponsor or its representative (drugsafety-briibio@dmedglobal.com) within 24 hours of the investigator's knowledge of the event.<br>• Contacts for SAE reporting can be found on the SAE Reporting Form.

10.3.5. Reporting of Laboratory Events of Clinical Interest

All laboratory ECIs must be reported to the Sponsor or its representative within 24 hours of the investigator's knowledge of the event. Contact for laboratory ECIs reporting can be found on the ECI Reporting Form.

The laboratory ECI will also be evaluated by the investigator to determine if the event meets criteria for an AE. If AE criteria are met, all clinical sequelae in relation to these laboratory ECIs will be reported as AEs or SAEs, if applicable.

10.3.6. Special Situation Report

For special situations and AEs due to special situations, the investigator should complete a Special Situation Report Form and send it to the Sponsor or its representative (drugsafety-briibio@dmedglobal.com) within 24 hours of becoming aware.

The English translation is for reference only, in case of any inconsistency the Chinese version shall prevail

10.4. Appendix 4: Contraceptive Requirements and Collection and Reporting of Pregnancy Information

Contraceptive Requirements:

WOCBP may be included in this study and include any female subject who has experienced menarche and who is not post-menopausal or permanently sterilized (eg, bilateral tubal occlusion, hysterectomy, or bilateral salpingectomy).

WOCBP must be willing to use highly effective methods of contraception 14 days before dose, throughout study participation, and for 12 weeks after last dose of study drug administration. Highly effective methods of birth control result in a low failure rate (ie, less than 1% per year). Birth control methods which are considered highly effective include:

- Established use of combined (estrogen and progestogen containing) oral, intravaginal, or transdermal hormonal methods of contraception associated with inhibition of ovulation **OR** established use of progestogen-only oral, injectable, or implantable hormonal methods of contraception associated with inhibition of ovulation. It is not currently known whether study drug will impact the effectiveness of hormonal contraceptive methods; therefore, it is recommended to use an additional form of contraception (ie, barrier method) throughout the study and for 12 weeks after last study drug administration.
- Placement of an intrauterine device
- Placement of an intrauterine hormone-releasing system
- Surgical sterilization of male partner (with the appropriate post-vasectomy documentation of the absence of sperm in the ejaculate; for female subjects on the study, the vasectomized male partner should be the sole partner for that subject)
- True sexual abstinence, when in line with the preferred and usual lifestyle of the subject. Periodic abstinence (e.g., calendar, ovulation, symptothermal, post-ovulation methods) and withdrawal are not acceptable methods of contraception. Abstinent subjects have to agree to use 1 of the above-mentioned contraceptive methods, if they start sexual relationships during the study and for up to 12 weeks after last dose of study drug administration, or for as long as the subject is followed on study, whichever is longer.
- Barrier method in combination with hormonal contraceptive, as described above

Post-menopausal status is defined as 12 months with no menses without an alternative medical cause.

Collection of Pregnancy Information:

- The investigator will collect pregnancy information about female subjects who are pregnant or male subject's female partner who are pregnant during the study. The

initial information will be recorded on the appropriate form and submitted to the sponsor within 24 hours of learning of a subject's pregnancy.

- The female subject who are pregnant or male subject's female partner who are pregnant will be followed to determine the outcome of the pregnancy. The investigator will collect follow-up information on the female subject who are pregnant or male subject's female partner who are pregnant and the neonate and the information will be forwarded to the sponsor. Generally, follow-up will not be required for longer than 6 to 8 weeks beyond the estimated delivery date. Any termination of pregnancy will be reported, regardless of fetal status (presence or absence of anomalies) or indication for the procedure.
- While pregnancy itself is not considered to be an AE or SAE, any pregnancy complication or elective termination of a pregnancy for medical reasons will be reported as an AE or SAE.
- A spontaneous abortion (occurring at <22 weeks gestational age) or still birth (occurring at >22 weeks gestational age) is always considered to be an SAE and will be reported as such.
- Any post-study pregnancy related SAE considered reasonably related to the study drug by the investigator will be reported to the sponsor as described in Section 8.3.4. While the investigator is not obligated to actively seek this information in former study subjects, he or she may learn of an SAE through spontaneous reporting.
- Any female subject who becomes pregnant while participating in the study will discontinue study drug.

10.5. Appendix 5: Assessments in Subjects Meeting Laboratory ECI Criteria

Additional assessment will be performed in subjects who experience ALT elevations meeting laboratory ECI criteria (Section 8.2.9) from Day 1 of dosing to the last follow-up visit. Following the occurrence of event of ECI, the following assessments in Table 4 will be performed one time, as soon as possible, and no later than two weeks after the initial ALT value meeting criteria for a laboratory ECI. The full panel of assessments should only be performed once; individual assessments may be repeated per investigator discretion, as needed.

Table 4 Assessments in Subjects Meeting Laboratory ECI Criteria

**Hematology**	
Complete blood count with differential, including red blood cell count, hemoglobin, hematocrit, white blood cell count, count and percentage of neutrophils, lymphocytes, eosinophils, basophils, and monocytes, and platelet count	
**Chemistry**	
Albumin	Estimated glomerular filtration rate (eGFR)
Blood urea nitrogen (BUN)	Gamma glutamyl transferase (GGT)
Calcium	Glucose
Carbon dioxide/bicarbonate	Lactate dehydrogenase (LDH)
Chloride	Potassium
Creatine kinase	Sodium
Creatinine	Uric acid
**Liver Function Tests**	
Alkaline phosphatase (ALP)	Aspartate aminotransferase (AST)
Alanine aminotransferase (ALT)	Bilirubin (total and direct)
**Coagulation Parameters**	
International normalized ratio (INR)	Prothrombin time (PT)
**Extended Hepatic Panel**	
Quantitative HBsAg	Ferritin
Quantitative HBV DNA	Blood phosphatidylethanol
Quantitative HBeAg	Urinary ethyglucuronide
HAV antibody IgM	Anti-nuclear antibodies
HCV antibody and HCV RNA	Anti-smooth muscle antibodies
HDV antibody or RNA	Anti-LKM1 antibody
HEV antibody IgM	Anti-mitochondrial antibodies
Epstein-Barr Virus antibodies, IgM and IgG	
Cytomegalovirus antibodies, IgM and IgG	
Ceruloplasmin	
**Imaging**	
Abdominal ultrasound, including Doppler flow, if applicable; CT or MRI acceptable in place of ultrasound if clinically indicated	
**Focused Medical History**	

The English translation is for reference only, in case of any inconsistency the Chinese version shall prevail

Use of any potentially hepatotoxic concomitant medications, including over the counter medications and herbal remedies	Alcohol consumption and drugs of abuse
Exposure to other potentially hepatotoxic agents including any work-related exposures (eg. solvents, pesticides, etc.)	Exercise history

CT = computed tomography; HAV = hepatitis A virus; HCV = hepatitis C virus; HDV = hepatitis D virus; HEV = hepatitis E virus; IgG = immunoglobulin G antibody; IgM = immunoglobulin M antibody; LKM1 = liver/kidney microsome-1 antibody; MRI = magnetic resonance imaging; PCR = polymerase chain reaction; RNA = ribonucleic acid; SLA = soluble liver antigen.

The English translation is for reference only, in case of any inconsistency the Chinese version shall prevail

10.6. Appendix 6: Protocol Amendment History

The protocol version number., effective date and major revisions are summarized as follows (Protocol amendment summary of changes for the current protocol Version 5.0 is located before Table of Contents):

Protocol version No.	Protocol effective date
1.0	30-APR-2021
2.0	16-JUN-2021
3.0	3-AUG-2021
4.0	28-FEB-2022
5.0	9-DEC-2022

Protocol Version 2.0 (16-JUN-2021) protocol amendment summary of changes:

Section and Headings	Prior version (version 1.0, Apr 30th, 2021)	Current version (version 2.0, Jun 16th, 2021)	Rational
1.5 Schedule of Activities (SoA)<br><br>Note m	If a subject experiences alanine aminotransferase (ALT) flare (as defined by ALT>2*ULN)	If a subject experiences alanine aminotransferase (ALT) flare (as defined by ALT > 2x ULN)	It's not consistent with the revised inclusion criteria, the criteria would be judged by investigator
4.1.3 PEG-IFN-α Therapy During the Dosing Period	Subjects entering the Dosing Period of the study will continue their peg-IFN-α treatment,	Subjects entering the Dosing Period of the study will continue their peg- IFN-α treatment, **investigator should monitor the related adverse events and take necessary medical treatment**	To emphasis the investigator should monitor the peg-IFN-α related adverse events and take necessary medical treatment

4.3.1.1 Dose Adjustment for adverse drug reactions	In patients with progressive or persistent ALT increases > 10 x ULN and >2 x baseline values<br><br>After peg-IFN-α dose reduction or withholding, therapy can be resumed after ALT flares subside	In patients with progressive or persistent ALT increases > 10 x ULN ~~and >2 x baseline values~~<br><br>After peg-IFN-α dose reduction or withholding, therapy can be resumed after **improvement of liver function by investigator's judgment.**	To be consistent with inclusion/exclusion criteria, and the resumed therapy should be determined by investigator based on clinical judgement
5.1 Inclusion Criteria<br><br><br><br><br>5.2 Exclusion Criteria	6.HBV DNA < 100 IU/mL at screening<br><br>10.Serum ALT and AST ≤ 2x ULN at screening.<br><br>8. including but not limited to corticosteroids at a prednisolone equivalent dose > 20 mg/day	6. HBV DNA < LLOQ at screening (central lab)<br><br>10.Serum ALT and AST ≤ 5x ULN at screening.<br><br>8. including but not limited to corticosteroids at ≥1 time prednisolone equivalent dose > 20 mg/day	Optimize the inclusion and exclusion criteria
5.3 Lifestyle Considerations	Alcohol consumption is limited to no more than 1 unit per day (unit: 1 glass of wine [125 mL] = 1 measure of spirits [30 mL] = one-half pint of beer [284 mL]) during the participation of this study	Alcohol consumption is limited to no more than 1 unit per day (unit: wine [about 125 mL] = white spirit [about 30 mL] = beer [about 284 mL]) during the participation of this study.	Increase the accuracy of the description
7.1.1 Criteria for Discontinuation of Study Intervention	Serum ALT > 5 × ULN, with no change in HBsAg, defined as a < 50% decrease from the baseline predose value	~~Serum ALT > 5 × ULN, with no change in HBsAg, defined as a < 50% decrease from the baseline predose value~~	Optimize the criteria for discontinuation of study intervention

8.2.7.2 Clinical Safety Laboratory Assessments	Fasting clinical safety laboratory tests to be performed are described in Section 10.2.	Fasting clinical safety laboratory tests to be performed are described in Section 10.2.<br><br>**The investigator should conduct additional clinical assessment for the adverse effects of PEG- IFN-α, including but not limited to radiological examination, ophthalmology assessment, immunological marker test, blood sugar and psychological assessment.**	Added the safety evaluation for PEG-IFN-α

Protocol Version 3.0 (3-AUG-2021) protocol amendment summary of changes:

**Original Section and Title**	**Description of Protocol Changes**	**Rationale**
Title Page, 1.1 Synopsis, 1.2 Phase 2, 1.3 Phase 3, Number of Subjects, Disclosure Statement, 3.1 Objectives, 3.2 Endpoints, 4.2 Scientific Rationale for Study Design, 5 Study Population, 9.1 Statistical Hypotheses, 9.2 Sample Size Determination, 9.3 Data Monitoring Guidelines for an Independent DSMB, 9.4 Rationale for Early Futility Analysis, 9.5.2. Guidelines for Monitoring of Phase 3 Primary Endpoints, 9.6 Statistical Analysis	The original adaptive Phase 2/3 study was modified into a Phase 2 study with two parts: 2a and 2b.	Changed to a more traditional study design
1.1 Synopsis, 1.2 Phase 2, 1.3 Phase 3, Number of Subjects, 5 Study Population, 9.2 Sample Size Determination, Table 1. Sample Size Calculation for Four Scenarios of Sustained HBsAg Loss, 9.3 Data	The sample size was increased from 106 to 120 in Part I (Phase 2a) and 480 in Part II (Phase 2b) (600 in total).<br><br>The "9.2 Sample Size Determination" and "Table 1. Sample Size Calculation for Four Scenarios of Sustained HBsAg	Sample size was updated after the study was changed from an adaptive Phase 2/3 study to a Phase 2 study with 2 parts

Monitoring Guidelines for an Independent DSMB, 9.4 Rationale for Early Futility Analysis	Loss" provide detailed justification and methodology for sample size calculation and suggest that the sample size in Phase 2b may be re-estimated after unblinding for data analysis after all 120 subjects enrolled in Phase 2a complete PEG-IFN-α treatment or withdraw from the study.	
1.2 Phase 2, 3.2.2 Secondary Endpoints	Added secondary objective "To evaluate functional cure in subjects with chronic HBV infection treated with BRII-179 (VBI-2601) therapy in combination with PEG-IFN-α + NrtI" and its corresponding secondary endpoint "Percentage of subjects with sustained HBsAg loss and persistently undetectable HBV DNA (defined as undetectable HBsAg and HBV DNA for at least 24 weeks) after NrtI discontinuation. " Added the same secondary objectives and endpoints as in Phase 2b part.	Study objectives were updated after the study was changed from an adaptive Phase 2/3 study to a Phase 2 study with 2 parts
1.2 Phase 2a, 1.5 (SoA), 3.2.3 Tertiary Endpoint, 9.6.5 Tertiary Efficacy Endpoint and Subgroup Analyses	Added assessment of serum protein level cytokine/chemokine biomarkers change from baseline from Day 1 of dosing to the end of follow-up, and during the NrtI discontinuation monitoring period	Further exploration of the immunogenicity profile of BRII-179 was added to support the overall risk/benefit profile for the proposed dose regimen and treatment duration and to characterize the susceptible population to the drug.
1.2 Phase 2 Secondary Endpoints, 1.3 Phase 3 Secondary Endpoints, Overall Design, Intervention Groups and Duration, 1.4. Schematic, 1.5. Schedule of Assessments, 3.2.2 Secondary Endpoints, 4.1 Overall Design, 4.3.1 Justification for Dose Selection and Number of Doses, 8.1.3 Dosing Period	The treatment duration and number of doses for BRII-179 were adjusted from every 4 weeks for a total of 9 doses over 32 weeks to every 3 weeks for a total of 7 doses over 18 weeks.	Base on supportive nonclinical and clinical data and communication with CDE.

1.4 Schema, 1.5 Schedule of Assessments,4.1 study design ， 5.1 Inclusion Criteria	Revised eligible subjects from receiving PEG-IFN-α treatment for 24-26 weeks to 24-28 weeks, screening window was revised from 14 days to 28 days	Increase clinical feasibility of subject recruitment.
2.3 Benefit/Risk Assessment	Supplemented human safety and immunogenicity data, nonclinical toxicology study data, and relevant clinical data for antigens and adjuvant components in BRII-179	To provide additional evidence for the safety and immunogenicity profile of BRII-179 to support the overall risk/benefit profile of the proposed study design.
4.2 Scientific Rationale for Study Design	Supplemented the rationale for patient population selection and modified some inaccurate statements.	Supplemented the rationale for patient population selection.
4.3 Justification for Dose Selection	Supplemented additional clinical and nonclinical evidence.	Supplemented the justification for dose selection.
5 Study Population	Clarified that the subjects planned to be recruited in this study are Chinese subjects.	Only the Chinese population will be observed in this study
5.2 Exclusion Criterion 14<br><br>6.4.3 Prohibited Medications	Removed Granulocyte macrophage colony stimulating factor (GM CSF) or erythropoietin (EPO) related contents	Better alignment with clinical practice
6 Study Intervention, Table 6.1.2 Dosage and Administration of BRII-179 and Placebo	IMP and NIMP definitions were modified and NrtI was changed to be provided by the Sponsor. Of which,<br>"BRII-179, placebo, PEG-INF-α and NrtI are all provided by the Sponsor" was specified in "6.1.1 Drug Product" and the instructions for NrtI were supplemented.<br>"Table 6.1.2 Dosage and Administration of BRII-179 and Placebo" was changed to "Table 6.1.2 Study Intervention". BRII-179, placebo, and PEG-INF-α were identified as IMPs and NrtI as NIMP. Specific information on PEG-INF-α and NrtI was also supplemented.	Sponsor provides NrtI to provide stable backbone treatment and to clarify IMP and NIMP definitions of the four kinds of drugs

6.4 Concomitant Therapy, 6.4.1. Drug Compliance with NrtI and PEG-IFN-α, 6.4.2 Permitted Medications	Added "6.4 Compliance of Study Intervention" to define compliance of study intervention. Supplemented coping strategy for treatment interruption.<br>Supplemented the collection time of concomitant therapy in "6.5 Concomitant Therapy".	Clarified the scope of data collection for concomitant therapy<br>Compliance with study intervention in addition to NrtI and PEG-IFN-α was added
6.6. Treatment of Study Intervention Overdose, 8.3.5 Special Situations	Removed "and results in clinical symptoms or signs."	Clarified that overdose is identified if exceeding the maximum recommended dose specified in the protocol, regardless of clinical symptoms
7.1 Discontinuation of Study Intervention	For subjects who discontinue study dosing, removed the "Dosing period" in "and continue to perform the required study-related visits and procedures for the remaining duration of study participation in the Dosing Period and Follow-up Period".	Patients who discontinue study dosing can enter the follow-up phase directly
7.1.1 Criteria for Discontinuation of Study Intervention	Clarified that subjects who discontinue study intervention will continue to undergo follow-up monitoring in the study.<br>Supplementary Criteria:<br>Discontinuation of study intervention requested by the subject or the subject's legal representative.<br>The subject has a medical condition or personal condition that, in the opinion of the investigator and/or the Sponsor, would pose an unnecessary risk to the subject if the investigational intervention is continued.<br>The subject's serum pregnancy test is confirmed to be positive.	Added other conditions that may result in discontinuation of study intervention
8.1.7 Early Termination Visit,1.5 Schedule of Assessments	Removed "A follow Up Visit will be conducted approximately 4 weeks after the last dose of study drug." This will be required only if the subject has ongoing AEs or lab abnormalities at the last on-study visit. "This visit can be	Clarified the definition of different early termination visits at different stages

	conducted by telephone ", and added specification of early termination visits ET2 and ET3	
9.7.2 Analysis of Phase 2 Primary Efficacy Endpoints	Added coping measures for fail of model fit due to too few subjects in a given stratum.	This description was supplemented considering the small number of subjects enrolled in Phase 2a which may lead to the small number of cases in one stratum
9.6.5. Analysis of Safety Outcomes	The definition of TEAE was updated. Safety analyses were clarified as descriptive statistics and summary descriptions of AEs, laboratory tests, vital signs, physical examinations, and 12-lead electrocardiograms were specified.	TEAEs were identified as AEs occur during or after treatment and AEs that are present pre-dose and worsened post-dose
1.5. Schedule of Assessments	Increased the number of visits, shorten treatment duration, and adjusted the examinations for the respective visits	The total duration of BRII-179 or placebo administration was reduced from 32 to 18 weeks due to the change of dosing from every four weeks to every three weeks, and the respective visit requirements need to be adjusted
Other	Updates to other descriptive text	To make the wording more accurate

Protocol Version 4.0 (28-FEB-2022) protocol amendment summary of changes:

**Original Section and Title**	**Description of Protocol Changes**	**Rationale**
Section 1.3 Phase 2b, Section 1.5 SoA, Section 3.2.2 Secondary Endpoints, Section 4.1 Overall Design, Section 8.1.3 Dosing Period, Section 9.2 Sample Size Determination and Section 10.2 Appendix 2: Clinical Laboratory Tests	Last visit in Dosing Period is revised from “W20-24/ET1” to “W24/ET1”, “Study Visit Day ± Visit Window” of this visit is revised from “D141-169 ± 3” to “D169 ± 3”. Accordingly, duration of dosing period is revised from “up to 24 weeks” to “24 weeks”, “End of Treatment” is revised from “up to Week 24” to “Week 24”, duration of follow-up period is revised from “up to 28 weeks” to “24 weeks”, treatment duration of PEG-IFN-α after	The time interval between the last dose of BRII-179 / placebo and the primary endpoint analysis (i.e., “completion of PEG-IFN-α treatment”) was uniformized for all subjects.

	enrollment is revised from “up to 24 weeks” to “24 weeks” (“a total of 48 weeks of PEG-IFN-α treatment before and after enrollment” is removed)	
Rationale and Overall Design in Section 1.1 Synopsis, Section 2.2 Study Rationale, Section 4.1 Study design, Section 4.2 Scientific Rationale for Study Design, Section 5.1 Inclusion Criteria, and Section 5.2 Exclusion Criteria	The restriction that subjects will have to be participants of the Everest Project is removed from the eligibility criteria.	To improve subject recruitment by expanding the scope of screening for eligible subjects.
Section 5.1 Inclusion Criteria and Section 4.1 Study Design	Inclusion Criteria 7 is revised from “Must be receiving first-line NrtI (ETV or TDF or TAF) therapy for ≥ 18 months and PEG-IFN-α for 24 to 28 weeks of the Day 1 visit and could provide the related treatment history” to “ Must be receiving first-line NrtI (ETV or TDF or TAF) therapy for ≥ 12 months (with evidence of HBV DNA<LLOQ for ≥ 6 months, local lab results accepted) and PEG-IFN-α for 24 to 28 doses (for subjects who are not strictly administered once a week due to objective reasons such as adverse reactions, the number of treatment weeks should not exceed treatment doses by 4) of the Day 1 visit and could provide the related treatment history.	The previous requirement of "24-28 weeks" were clarified in consideration of the real clinical practice that some patients are not receiving PEG-IFN-α strictly once a week due to objective reasons. In addition, since subjects are no longer required to be from the Everest Project, with clinical practice taken into consideration, ≥12 months of first-line NrtI therapy and evidence of HBV DNA<LLOQ ≥6 months are sufficient to prove that subjects are in sustained HBV DNA control under long-term NrtI treatment.
Section 1.5 SoA and Section 8.2 Detailed Description of Study Procedures	Abdominal ultrasound was included as part of the assessment at screening and every 6 months during the study (Week 24/ET1 in the Dosing Period; Week 48/ET 2 in the Follow-up Period; Day 1, Week 24, and Week 48/ET 3 of the	Hepatocellular carcinoma surveillance

The English translation is for reference only, in case of any inconsistency the Chinese version shall prevail

	NrtI Discontinuation Monitoring Period)	
SoA Section 1.5.1 Note b	"ET1 assessments should be performed within 14 days" is revised into "ET1 assessments should be performed within 14 days after study withdrawal"	To clarify the time window of ET1
SoA Section 1.5.1 Note k and Section 10.2 Appendix 2: Clinical Laboratory Tests	Clarified blood pregnancy test will be conducted at central lab at screening visit, urine pregnancy test and blood pregnancy test (if necessary) will be conducted at local laboratory at other time points,	To clarify the sample collection and testing laboratory for pregnancy test.
SoA Section 1.5.1 Note n	"Additional safety laboratory assessments may be performed at any time during the study as determined necessary by the investigator (local laboratory assessment is acceptable)" is added to the note.	To clarify "Additional tests may be performed at any time during the study as determined necessary by the investigator or required by local regulations" in Section 10.2 Appendix 2: Clinical Laboratory
SoA Section 1.5.1 Note o	"If a subject experiences alanine aminotransferase (ALT) flare, additional HBV DNA quantitation sample(s) may be collected"is revised into"If a subject experiences clinically significant alanine aminotransferase (ALT) flare, additional HBV DNA quantitation sample(s) may be collected as determined necessary by the investigator"	To clarify timing of "additional HBV DNA quantitation sample(s) may be collected"
SoA Section 1.5.1 Note p and Section 8.3.1 Time Period and Frequency for Collecting AE and SAE Information	The time period for adverse events (AEs) collection is clarified as "AEs will be collected from signing the ICF until the last visit". The distinction between AE and medical history for events occurring between signing ICF and initiation of study drug is defined as "if related to study procedures, will be recorded as AEs; otherwise, will be recorded as medical history."	To clarify the time frame for AE collection and to clarify the wording regarding categorization of events occurring between signing ICF and initiation of study drug as AEs or medical history.

SoA Section 1.5.1	"Subjects will have a FibroScan evaluation during screening. FibroScan evaluation performed within 6 months prior to screening is also acceptable for eligibility evaluation." is included as Note s	Clarification on FibroScan results that can be used for eligibility evaluation to align with exclusion criterion 3.
Section 5.1 Inclusion Criteria	"Study screening laboratory tests may be repeated once for values considered to be erroneous with medical monitor approval" in inclusion criterion 10 is revised into "Study screening laboratory tests may be repeated once for values considered to be questionable by investigator with medical monitor approval" and put this sentence in another row.	Formatting correction and to clarify on "considered to be erroneous".
Section 6.1 Study Interventions(s) Administered	Specify "entecavir tablet in combination with tenofovir disoproxil fumarate tablet or tenofovir alafenamide fumarate tablet may be provided if the investigator deems necessary"	To align with local clinical practice
Section 6.5 Concomitant Therapy	The whole "Permitted Medications" section is removed	Provide clear guidance on concomitant therapy, prohibited medications are listed in Section 6.5.2, other medications are permitted.
Section 7.1 Discontinuation of Study Intervention	Change "if a subject discontinues study, every effort should be made to keep the subject in the study. If the subject discontinues both investigational product (BRII-179 or placebo) and PEG-INF-α treatment, the subject should continue to attend the required study visits and procedures for the remaining duration of study participation in Follow-up Period" into "If a subject discontinues both investigational product (BRII-179 or	Wording simplification and clarification

The English translation is for reference only, in case of any inconsistency the Chinese version shall prevail

	placebo) and PEG-IFN-α treatment, the subject will be encouraged to continue to attend the required study visits and procedures for the remaining duration of study participation in Follow-up Period"	
Section 8.1.3 Dosing Period	Specified solicited local/systemic reaction after study drug administration as follows: any local reactions (including redness, swelling, pain, itchiness and tenderness, etc.) and systemic reactions (tiredness, diarrhea, nausea/vomiting, headache, muscle pain, etc,) related to BRII-179 (VBI-2601) or placebo administration.	Provide more specific description regarding solicited local/systemic reaction monitoring after study drug administration and aligned the description with subject diary card.
Section 8.2.3 FibroScan and Section 5.2 Exclusion Criteria	"Subject with a FibroScan result of ≤ 9 kPa at screening can be considered without significant liver fibrosis and cirrhosis. If screening FirboScan result > 9 kPa, however, the subject has a result of ≤ 9 Kpa from a FibroScan evaluation performed within 6 months prior to screening or liver biopsy performed within 1 year prior to screening that confirm the absence of Metavir F3 fibrosis or F4 cirrhosis, significant liver fibrosis and cirrhosis can be excluded as well. If a subject has had both procedures in the specified timeframes prior to screening, the most recent result should be used to determine eligibility" is clarified in Section 8.2.3.<br><br>Accordingly, exclusion criterion 3 is revised from "Significant liver fibrosis or cirrhosis as defined by having either a FibroScan result of > 9kPa at screening, unless the subject has results from a FibroScan evaluation performed within 6 months prior to screening or	Clarification

	liver biopsy performed within 1 year prior to screening that confirm the absence of Metavir F3 fibrosis or F4 cirrhosis. If a subject has had both procedures in the specified timeframes, the most recent result should be used to determine eligibility" to "Significant liver fibrosis or cirrhosis as defined by having a FibroScan result of > 9 kPa at screening. Unless the subject has a result of ≤9 Kpa from a FibroScan evaluation performed within 6 months prior to screening or liver biopsy performed within 1 year prior to screening that confirm the absence of Metavir F3 fibrosis or F4 cirrhosis. If a subject has had both procedures in the specified timeframes prior to screening, the most recent result should be used to determine eligibility" for clarification.	
Section 8.2.9 Laboratory Events of Clinical Interest and Section 10.5 Appendix 5,	The time period for collecting events of clinical interest (ECI) is revised from "NrtI Discontinuation Monitoring Period" to "Day 1 of dosing to the last visit"	To correct the inconsistency between SOA Section 1.5.1 and text description of the time period for ECI collection.
Section 8.3.5.2 Other Special Situations	The criterion for reporting of other special situations is modified as follows: "All other special situations must be reported to the sponsor within 24 hours of the investigator becoming aware of the situation. These reports apply to study drug only. Special situations involving medications other than study drug do not need to be reported; however, for special situations that result in AEs, the AEs should be reported."	To clarify the difference in reporting rules between special situations related to study drug and related to other medications.
Section 9.6.4 Analyses of Secondary Efficacy	Change "at baseline" in "subgroup analysis by age, gender, and HBsAg level at baseline (3 categories: ⩾0.05 to < 1 IU/mL, ⩾1 to < 10 IU/mL, and ⩾10 to <100 IU/mL) will be	To clarify that the primary endpoint will be analysed by HBsAg level at screening.

Endpoints and Subgroups	conducted with respect to the primary endpoint" to "at screening"	
Section 1.2 Phase 2a	Tertiary endpoint in the table was updated to "Serum protein level cytokine/chemokine biomarkers changes from baseline posting dosing, i.e., from Day 1 of dosing to the end of follow-up (Week 48) and during the NrtI discontinuation monitoring period".	To correct the inconsistency of tertiary endpoint description between Section 1.2 Phase 2a table and Section 3.2.3 Tertiary Endpoints.
Throughout the protocol	Typos and minor translation and formatting errors correction	To improve the overall quality of the protocol.

11. REFERENCES

Berg T, Simon KG, Mauss S, Schott E, Heyne R, Klass DM, Eisenbach C, Welzel TM, Zachoval R, Felten G, Schulze-Zur-Wiesch J, Cornberg M, Op den Brouw ML, Jump B, Reiser H, Gallo L, Warger T, Petersen J; FINITE CHB study investigators. Long-term response after stopping tenofovir disoproxil fumarate in non-cirrhotic HBeAg-negative patients - FINITE study. *Journal of Hepatology*. 2017 Nov;67(5):918-924.

Block TM, Gish R, Guo H, Mehta A, Cuconati A, Thomas London W, Guo JT. Chronic hepatitis B: What should be the goal for new therapies? Antiviral Res 2013; 98(1):27-34.

Chang, M. L., Liaw, Y. F., and Hadziyannis, S. J. (2015). Systematic review: cessation of long-term nucleos(t)ide analogue therapy in patients with hepatitis B e antigen-negative chronic hepatitis B. *Alimentary Pharmacology & Therapeutics*, 2015; *42*(3): 243-257.

Cornberg M, Lok AS, Terrault NA, Zoulim F, and the 2019 EASL-AASLD HBV Treatment Endpoints Conference Faculty. Guidance for design and endpoints of clinical trials in chronic hepatitis B - Report from the 2019 EASL-AASLD HBV Treatment Endpoints Conference. *Journal of Hepatology*. 2020; 72(3): 539-337.

Ferrari C. HBV and the immune response. *Liver Int*. 2015; 35 (Suppl. 1): 121-128.

Hoa PT, Huy NT, Thu le T, Nga CN, Nakao K, Eguchi K, Chi NH, Hoang BH, Hirayama K. Randomized controlled study investigating viral suppression and serological response following pre-S1/pre-S2/S vaccine therapy combined with lamivudine treatment in HBeAg-positive patients with chronic hepatitis B. *Antimicrob Agents Chemother*. 2009; 53:5134-40.

Jeng WJ, Chen YC, Chien RN, Sheen IS, Liaw YF. Incidence and predictors of HBsAg seroclearance after cessation of nucleos(t)ide analogue therapy in HBeAg negative chronic hepatitis B. *Hepatology*. 2018; 68(2): 425-434.

Kondo Y, Ninomiya M, Kakazu E, Kimura O, Shimosegawa T. Hepatitis B surface antigen could contribute to the immunopathogenesis of hepatitis B virus infection. *ISRN Gastroenterol*. 2013;2013:935295.

Konerman MA, Lok AS. Interferon Treatment for Hepatitis B. *Clinics in Liver Disease*. 2016; 20(4): 645-665.

Kramvis A. Genotypes and genetic variability of hepatitis B virus. *Intervirology* 2014; 57(3-4): 141-150.

Lebossé F, Testoni B, Fresquet J, Facchetti F, Galmozzi E, Fournier M, Hervieu V, Berthillon P, Berby F, Bordes I, Durantel D, Levrero M, Lampertico P, Zoulim F. Intrahepatic innate immune response pathways are downregulated in untreated chronic hepatitis B. *J Hepatol*. 2017;66(5):897-909.

Lo CM, Lau GK, Chan SC, Fan ST, Wong J. Efficacy of a Pre-S containing vaccine in patients receiving lamivudine prophylaxis after liver transplantation for chronic hepatitis B. Am J Transplant. 2007;7(2):434-439.

Lok AS, McMahon BJ, Brown RS, Wong JB, Ahmed AT, Farah W, Almasri J, Alahdab F, Benkhadra K, Mouchli MA, Singh S, Mohamed EA, Abu Dabrh AM, Prokop LJ, Wang Z, Murad MH, Mohammed K. Antiviral therapy for chronic hepatitis B viral infection in adults: A systematic review and meta-analysis. *Hepatology* 2016; 63(1):284-306.

Marcellin P, Ahn SH, Ma X, Caruntu FA, Tak WY, Elkashab M, et al. Combination of tenofovir disoproxil fumarate and peginterferon α-2a increases loss of hepatitis B surface antigen in patients with chronic hepatitis B. Gastroenterology 2016;150(1):134-44.

Michler T, Kosinska AD, Festag J, Bunse T, Su J, Ringelhan M, Imhof H, Grimm D, Steiger K, Mogler C, Heikenwalder M, Michel ML, Guzman CA, Milstein S, Sepp-Lorenzino L, Knolle P, Protzer U. Knockdown of Virus Antigen Expression Increases Therapeutic Vaccine Efficacy in High-Titer Hepatitis B Virus Carrier Mice. Gastroenterology. 2020 May;158(6):1762-1775.

Papatheodoridi M, Hadziyannis E, Berby F, Zachou K, Testoni B, Rigopoulou E, Gatselis NK, Lyberopoulou A, Vlachogiannakos I, Manolakopoulos S, Dalekos GN, Zoulim F, Papatheodoridis GV. Predictors of hepatitis B surface antigen loss, relapse and retreatment after discontinuation of effective oral antiviral therapy in noncirrhotic HBeAg-negative chronic hepatitis B. *J Viral Hepat.* 2020; 27(2): 118-126.

Perrillo, R. "Benefits and risks of IFN therapy for hepatitis B." *Hepatology.* 2009; 49(5): S103-111.

Ren H, Hu P, Chen XY, Gong GZ, Shang J, Zhang WH, et al. Switching to PegIFNα-2a in NUC treated CHB patients (NEW SWITCH Study)： comparison 48 and 96 weeks. Hepatol Int 2016;10(Suppl 1):S43-4.

Roggendorf H, Krawczyk A, Lindemann M, Shouval D, Michler T, Roggendorf M, Gerken G. Induction of functional control in chronic hepatitis B patients with low level HBsAg using a combination of a PreS1/S2/S HBV vaccine (Sci-B-Vac®) and a nucleoside analogue. *J Infect Dis Ther*. 2019; 7:389-393.

Scaglione SJ, Lok AS. Effectiveness of hepatitis B treatment in clinical practice. Gastroenterology 2012; 142:1360-1368.

Schweitzer A, Horn J, Mikolajczyk RT, Krause G, Ott JJ. Estimations of worldwide prevalence of chronic hepatitis B virus infection: a systematic review of data published between 1965 and 2013. *Lancet* 2015; 386(10003): 1546-1555.

Stanaway JD, Flaxman AD, Naghavi M, Fitzmaurice C, Vos T, Abubakar I, Abu-Raddad LJ, Assadi R, Bhala N, Cowie B, Forouzanfour MH, Groeger J, Hanafiah KM, Jacobsen KH, James SL, MacLachlan J, Malekzadeh R, Martin NK, Mokdad AA, Mokdad AH,

Murray CJL, Plass D, Rana S, Rein DB, Richardus JH, Sanabria J, Saylan M, Shahraz S, So S, Vlassov V, Weiderpass E, Wiersma ST, Younis M, Yu C, Zaki M, Cooke GS. The global burden of viral hepatitis from 1990 to 2013: findings from the Global Burden of Disease Study 2013. *Lancet* 2016; 388(10049): 1081-1088.

Trepo CHL, Chan, Lok A. Hepatitis B virus infection. *Lancet* 2014;384(9959): 2053-2063.

van Bömmel F, Berg T. Stopping long-term treatment with nucleos(t)ide analogues is a favourable option for selected patients with HBeAg-negative chronic hepatitis B. *Liver International*. 2018;38 Suppl 1:90-96.

Wang S, Chen Z, Hu C, Qian F, Cheng Y, Wu M, Shi B, Chen J, Hu Y, Yuan Z. Hepatitis B virus surface antigen selectively inhibits TLR2 ligand-induced IL-12 production in monocytes/macrophages by interfering with JNK activation. *J Immunol*. 2013 May 15;190(10):5142-51.

Wong TCL, Fung JYY, Chok KSH, Cheung TT, Chan ACY, Dai WC, Ng KKC, Chan SC, Lo, CM. Hepatitis B vaccination in patients receiving oral antiviral therapy without hepatitis B immunoglobulin after liver transplant. *Transplant Proc*. 2018; 50:3681-3688.

Yao CC, Hung CH, Hu TH, Lu SN, Wang JH, Lee CM, and Chen CH. Incidence and predictors of HBV relapse after cessation of nucleoside analogues in HBeAg-negative patients with HBsAg ≤ 200 IU/mL. *Scientific Reports*. 2017; 7(1): 1839.

Ze-Qian Wu, Dong-Ying Xie, Lei Fu, Jia Wei, Jia Shang, Qing He, Wen-hua Zhang, Guang-yu Huang, Yanzhong Peng, Ye Gu, Jia-bin Li, Ying Guo, Yu-juan Guan, Jia-wei Geng, Huan-wei Zheng, Wen-jun Gao, Wen-jing Zhao, Ying-jun Tian, Yi-lan Zeng, Ren Chen, Shengwang Gu, Zhi-liang Gao. Functional cure based on pegylated interferon? in long-term nucleoside analog suppressed HBeAg negative chronic hepatitis B: a multicenter real-world study (Everest project in China), an interim report. *Journal of Hepatology* 2020; 73: S653-S915.

Zhu D, Liu L, Yang D, Fu S, Bian Y, Sun Z, He J, Su L, Zhang L, Peng H, Fu YX. Clearing Persistent Extracellular Antigen of Hepatitis B Virus: An Immunomodulatory Strategy To Reverse Tolerance for an Effective Therapeutic Vaccination. *J Immunol*. 2016; 196(7): 3079-87.
