# Supplementary material for: Profile and Predictors of Hepatitis B Surface Antigen Seroreversion Following PEG-IFNα with or Without Therapeutic Vaccine BRII-179 or siRNA Elebsiran: A Post Hoc Pooled Analysis of Two Randomized Controlled Trials in Patients with Chronic Hepatitis B Virus Infection
Source: Vaccines (Basel). 2026 Oct 1;14(10):868. doi: 10.3390/vaccines14100868 (PMC13709818; doi:10.3390/vaccines14100868)
Supplement: Supplementary file 1 [file vaccines-14-00868-s001.zip › File_S2_BRII-179-002_Statistical_Analysis_Plan_and_Amendments.pdf]

STATISTICAL ANALYSIS PLAN

**Study Title:**	A Multicenter, Randomized, Double-Blind, Placebo-Controlled, Parallel-Group Study to Investigate the Efficacy and Safety of BRII-179 (VBI-2601) for the Treatment of Chronic Hepatitis B Virus (HBV) Infection
**Name of Test Drug:**	BRII-179 (VBI-2601)
**Study Number:**	BRII-179-002
**Protocol Version (Date):**	5.0 (09 December 2022)
**Analysis Type:**	Phase 2a Analysis
**Analysis Plan Version:**	1.0
**Analysis Plan Date:**	21 JUNE 2023
**Analysis Plan Author(s):**	[redacted]

CONFIDENTIAL AND PROPRIETARY INFORMATION

SIGNATURE PAGE

Reason for Signature	Name	Signature	Date (DDMMMYYYY)
Author	[redacted]		
Therapeutic Area Head (or delegate)			
Biometrics Head (or delegate)			

TABLE OF CONTENTS

TABLE OF CONTENTS 3
LIST OF ABBREVIATIONS 5
1. INTRODUCTION 7
1.1. Study Objectives 7
1.2. Study Design 7
1.3. Sample Size and Power 9
1.3.1 Phase 2a Part 9
2. TYPE OF PLANNED ANALYSIS 11
2.1. Week 24 Analysis 11
2.2. Final Analysis 11
3. GENERAL CONSIDERATIONS FOR DATA ANALYSES 12
3.1. Key Definitions 13
3.2. Analysis Sets 14
3.2.1. All Subjects Screened 15
3.2.2. All Subjects Randomized 15
3.2.3. Full Analysis Set 15
3.2.4. Per Protocol Set 15
3.2.5. Safety Analysis Set 15
3.2.6. Serologically Evaluable Full Analysis Set 16
3.2.7. Biomarker Analysis Set 16
3.3. Subject Grouping 16
3.4. Strata and Covariates 16
3.5. Examination of Subject Subgroups 16
3.6. Multiple Comparisons 17
3.7. Missing Data and Outliers 17
3.7.1. Missing Data 17
3.7.2. Outliers 17
3.8. Data Handling Conventions and Transformations 17
3.9. Analysis Visit Windows 18
3.9.1. Selection of Data in the Event of Multiple Records in an Analysis Visit Window 21
4. SUBJECT DISPOSITION 23
4.1. Subject Enrollment and Disposition 23
4.1.1. Duration of Exposure to Study Drug 24
4.1.2. Adherence to Study Drug 24
4.2. Protocol Deviations 25
5. BASELINE CHARACTERISTICS 26
5.1. Demographics and Baseline Characteristics 26
5.2. Medical History 26
6. EFFICACY ANALYSES 28
6.1. Primary Efficacy Endpoint 28
6.1.1. Estimand 28
6.1.2. Analysis methods for the primary endpoint 30

6.2. Secondary Efficacy Endpoints 31
6.2.1. Analysis methods for the secondary endpoints 31
6.3. Tertiary Efficacy Endpoints 32
6.3.1. Analysis methods of the tertiary efficacy endpoints....33

7. SAFETY ANALYSES....34

7.1. Adverse Events and Deaths....34
7.1.1. Adverse Event Dictionary 34
7.1.2. Adverse Event Severity....34
7.1.3. Relationship of Adverse Events to Study Drug....34
7.1.4. Serious Adverse Events....34
7.1.5. Treatment-Emergent Adverse Events....34
7.1.5.1. Definition of Treatment-Emergent Adverse Events 34
7.1.5.2. Incomplete Dates 34
7.1.6. Summaries of Adverse Events and Deaths....35
7.2. Laboratory Evaluations 37
7.2.1. Summaries of Numeric Laboratory Results 37
7.2.2. Graded Laboratory Values 38
7.2.2.1. Treatment-Emergent Laboratory Abnormalities....38
7.2.2.2. Summaries of Laboratory Abnormalities....38
7.2.3. Liver-related Laboratory Evaluations....38
7.3. Body Weight, Height, BMI and Vital Signs....39
7.4. Prior and Concomitant Medications....39
7.4.1. Prior Medications 40
7.4.2. Concomitant Medications....40
7.5. Electrocardiogram Results 41
7.6. Other Safety Measures 41
7.7. Changes From Protocol-Specified Safety Analyses....41

8. EXPLORATORY ANALYSES....42

8.1. Comparing of FibroScan result .... **Error! Bookmark not defined.**
8.2. Probability of Success Estimated by Bayesian Model based on HBsAg Loss at Week 24....42
8.2.1. Statistical methods....42
8.2.1.1. Bayesian Modeling 42
8.2.1.2. Model specification: 43

9. SOFTWARE 45

10. SAP REVISION....46

11. APPENDICES 47

LIST OF ABBREVIATIONS

AE	adverse event
ALT	alanine aminotransferase
AST	aspartate aminotransferase
ATC	Anatomical Therapeutic Chemical
BMI	body mass index
CI	confidence interval
CS	Clinically Significant
CSR	clinical study report
CTCAE	Common Toxicity Criteria for Adverse Events
DSMB	Data and Safety Monitoring Board
ECG	Electrocardiogram
eCRF	electronic case report form
FAS	Full Analysis Set
HBsAb	Hepatitis B surface antibody
HBsAg	Hepatitis B surface antigen
HBV	Hepatitis B virus
HLT	high-level term
ICH	International Conference on Harmonization (of Technical Requirements for Registration of Pharmaceuticals for Human Use)
IWRS	Interactive voice or web response system
LLOQ	Lower limit of quantitation
MedDRA	Medical Dictionary for Regulatory Activities
NCS	Not Clinically Significant
NrtI	Nucleos(t)ide reverse transcriptase inhibitor
PEG-IFN-α	Pegylated interferon-alpha
PPS	per protocol set
PT	preferred term
Q1, Q3	first quartile, third quartile
SAE	serious adverse event
SAP	statistical analysis plan
SD	standard deviation
SI (units)	international system of units
SOC	system organ class
TE	Treatment-emergent
TEAE	treatment-emergent adverse event
TFLs	tables, figures, and listings
ULN	upper limit of normal

WHO	World Health Organization

1. INTRODUCTION

BRII-179 (VBI-2601) is a protein-based immunotherapeutic intended for the treatment of individuals chronically infected with hepatitis B virus (HBV).

Study BRII-179-002 is a multicenter, double-blind, randomized, placebo-controlled, parallel-group phase 2 study aims to evaluate the clinical effect of adding BRII-179 (VBI-2601) to existing pegylated interferon-alpha (PEG-IFN-α) and nucleos(t)ide reverse transcriptase inhibitor (NrtI) therapy in Chinese patients with chronic HBV infection without cirrhosis. It will consist of two parts, Part 2a and 2b, of which 120 subjects are planned to be enrolled in the first part (Phase 2a) and 480 subjects are planned to be enrolled in the second part (Phase 2b).

This statistical analysis plan (SAP) describes the statistical analysis methods and data presentations to be used in tables, figures, and listings (TFLs) of the analysis of phase 2a part. This SAP is based on the study protocol version 5.0 dated 09 December 2022 and the electronic case report form (eCRF version 2.2 dated 24 February 2023). The SAP will be finalized before database freeze for Week 24 Analysis which primary endpoint analysis will be included. Any changes made after the finalization of the SAP will be documented in the CSR.

1.1. Study Objectives

The primary objective of Study BRII-179-002 phase 2a part is:

- To evaluate the efficacy of BRII-179 to increase HBsAg loss through completion of PEG-IFN-α treatment

The secondary objectives of Study BRII-179-002 phase 2a part are as follows:

- To evaluate functional cure of subjects with chronic HBV infection receiving BRII-179 (VBI-2601) therapy in combination with PEG-IFN-α + NrtI
- To assess the antiviral activity of BRII-179 (VBI-2601) therapy in combination with PEG-IFN-α + NrtI
- To assess safety of BRII-179 (VBI-2601) therapy

The tertiary objective of Study BRII-179-002 phase 2a part is:

- To assess the effect on relevant immune markers in subjects with chronic HBV infection treated with BRII-179 (VBI-2601) in combination with PEG- IFN-α + NrtI

1.2. Study Design

Study BRII-179-002 is a double-blind, randomized, placebo-controlled, parallel-group study aims to evaluate the efficacy and safety of adding BRII-179 (VBI-2601) to existing PEG-IFN-α and NrtI therapy in Chinese patients with chronic HBV infection without cirrhosis.

A large part of subjects in the current study are anticipated to be recruited from the Everest Project (NCT04035837). The Everest Project is a multicenter real-world study being conducted in over 260 hospitals in China. The plan is to recruit 30,000 patients with chronic HBV infection effectively treated with NrtI therapy for at least one year; who are hepatitis B e-antigen (HBeAg) negative with HBV DNA <100 IU/ml and HBsAg <1500 IU/mL at study entry. As part of the study participation, subjects receive PEG-IFN-α as "add-on" or "switch to" treatment for 48 to 96 weeks. The primary objective of the Everest Project is to achieve functional cure of HBV infection by optimizing treatment strategies of NrtIs and PEG-IFN-α.

Eligible subjects who have been receiving PEG-IFN-α for 24 to 28 doses and first-line NrtI therapy for at least 12 months with HBV DNA< lower limit of quantification (LLOQ) for ≥ 6 months and HBsAg ≥ 0.05 IU/mL and < 100 IU/mL at screening, will be randomized 1:1 to receive BRII-179 (VBI-2601) or 0.9% Sodium Chloride placebo in addition to the existing PEG-IFN-α and NrtI therapy. Subjects will be stratified at randomization by screening HBsAg level (3 categories: ≥0.05 to < 1 IU/mL, ≥1 to < 10 IU/mL, and ≥10 to <100 IU/mL).

In Dosing Period, subjects will have investigational treatment with BRII-179 or placebo intramuscular injection every three weeks for a total of seven doses over an 18-week course. Subjects in both BRII-179 group and placebo group will be treated with PEG-IFN-α for another 24 weeks after enrollment; while NrtI treatment will continue into the Follow-up Period for all subjects.

Estimated total duration for each subject will include the following: screening up to 28 days, dosing period 24 weeks (18 weeks BRII-179 or placebo investigational treatment; 24 weeks PEG-IFN-α treatment), and a 24-week follow-up period. Eligible subjects that meet NrtI discontinuation criteria(eligibility assessed at Week 36 to Week 48) may enter a NrtI discontinuation monitoring period for an additional 48 weeks from the day of NrtI discontinuation. See Figure 1 for the BRII-179-002 study design schema.

An independent DSMB will monitor subject safety and treatment efficacy data for this study. The details for the operation and responsibilities of the DSMB will be defined in a separate DSMB Charter. The Charter will delineate the composition, duties, responsibilities, and procedures of the DSMB, data required at each meeting, as well as the analyses that will be conducted.

Figure 1 Schema for BRII-179-002 study design

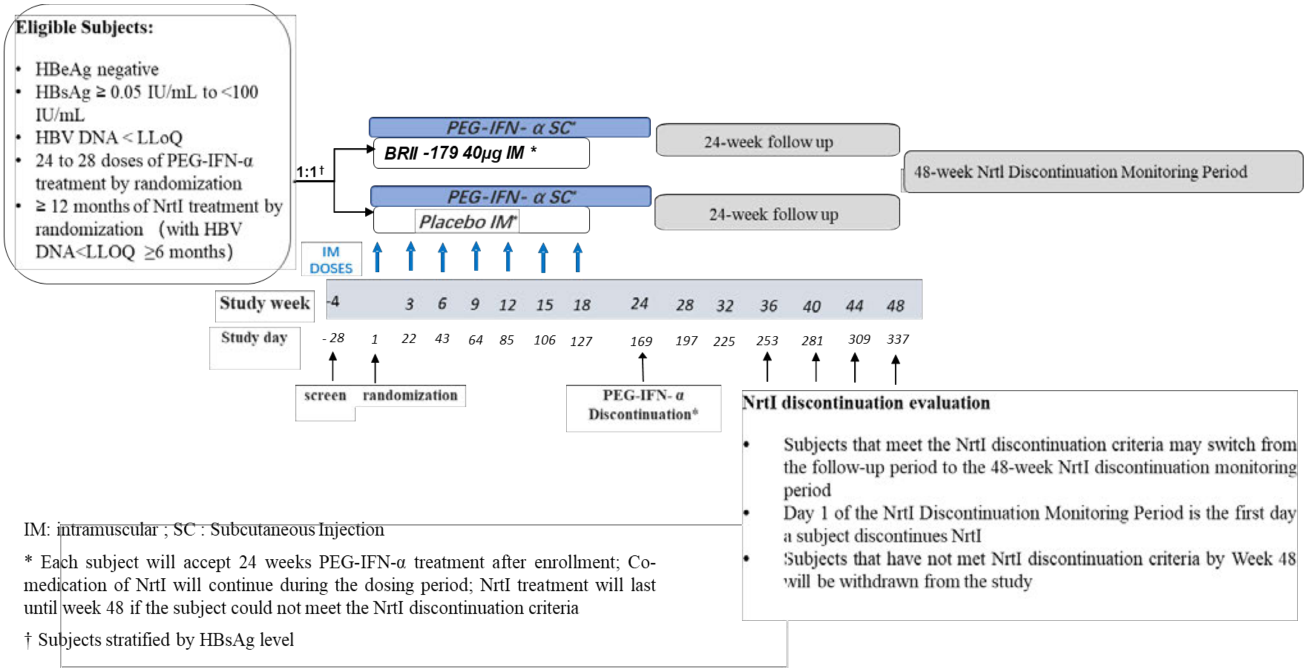

1.3. Sample Size and Power

1.3.1 Phase 2a Part

The early futility evaluation based on an intermediate outcome of efficacy using the HBsAg loss at completion of 24-week PEG-IFN-α treatment will be conducted when approximately 120 subjects complete 18 weeks BRII-179 or placebo investigational treatment and 24 weeks PEG-IFN-α treatment or withdraw the study prior to last scheduled PEG-IFN-α treatment.

The Everest project data suggest 25% subjects can achieve HBsAg loss after 24 weeks treatment of PEG-IFN-α + NrtI. The 20% difference in percentage of HBsAg loss is considered to be a clinically meaningful increase in HBsAg clearance with the addition of BRII-179 to PEG-IFN-α and NrtI therapy, based on this point, with the assumption of a 25% of HBsAg loss by placebo in combination with PEG-IFN-α and NrtI treatment, a 45% of HBsAg loss by BRII-179 in combination with PEG-IFN-α and NrtI treatment, and one-sided Type I error α 0.3, The sample size in range of 105 to 120 can provide power of more than 95% to reject the null hypothesis (Table 1). In this sample size estimation, power calculation method is normal approximation and test method is Z-test (SAS 9.4).

Table 1. The power calculated based on different sample size

HBsAg loss		Total Sample Size	Power	Alpha (1-sided)
Placebo + PEG-IFN-α + NrtI	BRII-179 + PEG-IFN-α + NrtI			
25%	45%	105	95.1%	0.3
		110	95.7%	
		115	96.0%	
		120	96.5%	

2. TYPE OF PLANNED ANALYSIS

2.1. Week 24 Analysis

Primary endpoint analysis timepoint is defined as approximately 120 subjects in total who have (1) completed 18 weeks BRII-179 or placebo investigational treatment and 24 weeks PEG-IFN-α treatment, and (2) Week 24 visit HBsAg results are available; or withdraw from the study/discontinue study drug prior to last scheduled PEG-IFN-α treatment. An early futility analysis based on HBsAg loss will be performed on above timepoint. The sample size of 120 subjects provides above 95% power to reject the null hypothesis at the 0.30 (one-sided) level of significance.

The primary endpoint and key secondary efficacy and safety endpoints by Week 24 will be analyzed, an independent DSMB will review the output andwill build the rationale for the recommendation on whether to proceed to Phase 2b part or not.

Brii will make the decision on whether to proceed to Phase 2b part or not based on HBsAg loss futility analysis result at completion of last scheduled PEG-IFN-α treatment and safety data analysis, as well as the recommendation provided by the independent DSMB.

The DSMB's role and responsibilities and the scope of analysis to be provided to the DSMB are provided in a mutually agreed upon charter that defines the DSMB membership, meeting logistics and meeting frequency.

2.2. Final Analysis

The final analysis will be conducted after all enrolled subjects have completed Phase 2a of the study or prematurely terminated from the study.

3. GENERAL CONSIDERATIONS FOR DATA ANALYSES

All data collected on the electronic case report form (eCRF) will be presented in the data listings and will be listed by subject identification number (in ascending order), age, gender, race and screening body weight and visit, where applicable. All summaries will be presented by treatment groups.

When reporting descriptive statistics, the following rules will apply:

- Continuous variables: Descriptive statistics will include the number of non-missing values (n), arithmetic mean, standard deviation (SD), median, first quartile (Q1), third quartile (Q3), minimum (Min) and maximum (Max) values.

  The minimum and maximum values will be displayed to the same decimal precision as the source data; the Q1, Q3, arithmetic mean and median values will be displayed to one more decimal than the source data; SD value will be displayed to two more decimal than the source data for the specific variable.

  The appropriate precision for derived variables will be determined based on the precision of the data on which the derivations are based, and statistics will be presented in accordance with the abovementioned rules.

  n will be an integer. If no subjects have data at a given time point, then only n=0 will be presented.

  All data will be presented to a maximum of 3 decimal places.

- Categorical variables: Descriptive statistics will include counts and percentages per category. The denominator in all percentage calculations will be the number of subjects in the relevant analysis set with non-missing data, unless specifically stated otherwise. Percentages will be displayed to one decimal place.

  Percentage change from baseline values will be calculated and displayed to 1 decimal place in the listings. In the summaries, the mean and median percentage change from baseline values will be presented to 2 decimal places, the minimum and maximum values will be presented to 1 decimal place and the SD value will be presented to 3 decimal places.

  For categories where all subjects fulfill certain criteria, the percentage value will be displayed as 100. For categories where zero subjects fulfill certain criteria, there will be no percentage displayed. All other percentage displays will use 1 decimal place. Data listings will contain all reported and derived data.

Unless otherwise stated, the following methods will be applied:

- Repeat/unscheduled assessments: Values collected at unscheduled study visits/time points will be allocated to scheduled visit/time points according to the algorithms of analysis visit window together with values collected at scheduled visits/time points will be presented in summary tables. All collected data will be included in the data listings.

- Assessment windows: All assessments will be included in the data listings and no visit windows will be applied to assessments that were performed outside of the protocol specified procedure windows.
- Result display convention: Results will be center aligned in all summary tables and listings. Subject identifiers visit and parameter labels may be left-aligned if required.
- Date and time display conventions: The following display conventions will be applied in all outputs where dates and/or times are displayed:
    - Date only: YYYY-MM-DD
    - Date and time: YYYY-MM-DD HH:MM

If only partial information is available, unknown components of the date or time will be presented as ‘NK’ (not known), i.e., ‘2016-NK-NK’. Times will be reported in military time.

3.1. Key Definitions

The following definitions will be used:

- Baseline: The baseline value is defined as the last available valid (quantifiable continuous or categorical value), non-missing observation for each subject prior to first BRII-179/placebo administration unless otherwise specified. Repeat and unscheduled assessments will be included in the derivation of the baseline values.
- Change from Baseline: The change from baseline value is defined as the difference between the result collected/derived at a post-baseline visit/time point and the baseline value.

  The change from baseline value at each post-baseline visit/time point will be calculated for all continuous parameters using the following formula:

  Change from Baseline Value = Result at Visit/Time Point – Baseline Value

  The change from baseline value will only be calculated if the specific post-baseline visit/time point result and the baseline value for the parameter are both available and will be treated as missing otherwise.
- Fold Change from Baseline: The fold change from baseline value is defined as the ratio of the result collected/derived at a post-baseline visit/time point divided by the baseline value.

  The change from baseline value at each post-baseline visit/time point will be calculated for all continuous parameters using the following formula:

  Fold Change from Baseline Value = Result at Visit/Time Point / Baseline Value

  The fold change from baseline value will only be calculated if the specific post-baseline visit/time point result and the baseline value (not 0) for the parameter are both available and will be treated as missing otherwise.

- Study day: The study day of an event is defined as the relative day of the event starting with the date of the first BRII-179/placebo administration (reference date) as Day 1 (there will be no Day 0).

  The study day of events occurring before the first study drug administration will be calculated as:

  Study Day = (Date of Event - Date of First BRII-179/placebo Administration)

  For events occurring on or after Day 1, study day will be calculated as:

  Study Day = (Date of Event - Date of First BRII-179/placebo Administration) + 1

  Study days will only be calculated for events with complete dates and will be undefined for events that are 'Ongoing' at the end of the study.

- Relative days compared to an alternative reference point will be calculated similarly, but the alternative starting reference start date will be used instead of the date of the first study drug dosing.
- Completion of Week 18 BRII-179/Placebo: Subjects whose BRII-179/Placebo last to Week 18 or later but by Week 24.
- Completion of Week 24 PEG-IFN-α treatment:Subjects whose PEG-IFN-α last to Week 24.
- HBsAg loss/HBsAg seroclearance: HBsAg changing from positive (>=0.05 IU/mL) at baseline to negative (<LLOQ 0.05 IU/mL) at any postbaseline visit.
- Hepatitis B surface antibody (HBsAb) response: at any post-baseline visit with either (i) post-baseline anti-HBs ≥ 2 IU/L if anti-HBs ＜2 IU/L at baseline or (ii) post-baseline anti-HBs ≥ 5 times the baseline anti-HBs if baseline anti-HBs was ≥ 2 IU/L.
- HBsAg seroconversion: HBsAg changing from positive (>=0.05 IU/mL) at baseline to negative (<LLOQ 0.05 IU/mL) AND HBsAb changing from negative (< 10.0 IU/L) at baseline to positive (≥ 10.0 IU/L) at any postbaseline visit.
- Sustained HBsAg loss: quantitative HBsAg < LLOQ 0.05 IU/mL at scheduled and unscheduled visits for ≥ 24 weeks after NrtI discontinuation.
- Persistently undetectable HBV DNA: HBV DNA < LLOQ 10 IU/mL at scheduled and unscheduled visits for ≥ 24 weeks after NrtI discontinuation.
- NrtI discontinuation criteria: a) Alanine aminotransferase (ALT) < 2x ULN (results from previous visit); AND b) HBV DNA < LLOQ 10 IU/mL (results from previous visit); AND c) 2 consecutive assessments of HBsAg < LLOQ 0.05 IU/mL (assessed starting from Week 28 laboratory results).
- NrtI re-treatment: Subjects re-treated with NrtI in the judgment of investigator during NrtI discontinuation monitoring period.

3.2. Analysis Sets

Analysis sets define the subjects to be included in an analysis. Analysis sets and their definitions are provided in this section. The analysis set will be identified and included as a subtitle of each table, figure, and listing.

For each analysis set, the number and percentage of subjects eligible for inclusion.

A listing of reasons for exclusion from analysis sets will be provided by subject.

3.2.1. All Subjects Screened

All subjects, for whom a record exists on the clinical database, will be included in All Subjects Screened. It will be used for reporting study disposition, inclusion and exclusion criteria deviations, etc.

3.2.2. All Subjects Randomized

All subjects, who are randomized will be included in All Subjects Randomized. It will be used for reporting demographics, baseline characteristics, medical history, etc.

3.2.3. Full Analysis Set

All subjects, who are randomized and received at least one dose of BRII-179 or placebo, will be included in Full Analysis Set (FAS). It will be used as the primary analysis set of all efficacy endpoints.

3.2.4. Per Protocol Set

The Per Protocol Set (PPS) will include the subset of the FAS who have:

- HBsAg $\geq$ 0.05 IU/mL at baseline.
- Had not identified with important protocol deviation that significantly impacts data integrity and efficacy analysis (e.g. Week 24 visit was not conducted, Week 24 visit out-of-window >14 days, total dose number of BRII-179/placebo <6 , total dose number of PEG-IFN-α dose <20, prohibited medication administration that will significantly impact efficacy endpoint analysis, etc.).

The PPS will be used as analysis set for efficacy endpoint analyses. The analysis based on PPS will be considered as sensitivity analysis.

3.2.5. Safety Analysis Set

All subjects who are randomized and who take at least 1 dose of study drug will be included in Safety Analysis Set. It will be used for reporting safety parameters. The Safety Analysis Set will be based on actual treatment received if different from the treatment to which they were randomized. The actual treatment received will differ from the randomized treatment only when

their actual treatment differs from randomized treatment for the entire treatment duration. All safety analyses will be based on the Safety Analysis Set.

3.2.6. Serologically Evaluable Full Analysis Set

The Serologically Evaluable FAS for HBsAg Loss will include all subjects who were randomized and received at least one dose of BRII-179 or placebo, and with HBsAg ≥ 0.05 IU/mL at baseline.

3.2.7. Biomarker Analysis Set

When applicable, selected subjects will be included in the Biomarker Analysis Set and their selected data relating to the cytokine/chemokines will be analysed.

3.3. Subject Grouping

For analyses based on the All Subjects Randomized, Full Analysis Set, Per Protocol Set and Serologically Evaluable Full Analysis Set subjects will be grouped according to the treatment groups to which they were randomized. For analyses based on Safety Analysis Set, subjects will be grouped according to the actual treatment received.

3.4. Strata and Covariates

Subjects will be randomly assigned to either BRII-179 (VBI-2601) or placebo in addition to the existing PEG-IFN-α and NrtI therapy via the interactive voice or web response system (IWRS) in a 1:1 ratio using a stratified randomization schedule. Stratification will be based on the following variables:

- HBsAg level at screening (3 categories: ≥0.05 to < 1 IU/mL, ≥1 to < 10 IU/mL, and ≥10 to <100 IU/mL)

If there are discrepancies in stratification factor values between the IWRS and the clinical database, the values recorded in the clinical database will be used for analyses.

No covariate adjustments will be made.

3.5. Examination of Subject Subgroups

Subgrouping of subjects based on randomization stratification factors may be explored for subgroup analyses.

The primary efficacy endpoint will be examined using the following subgroups:

- Age: (a) 18 - 44 Years, (b) 45 - 60 Years

- Gender: (a) Male and (b) Female
- HBsAg level at screening: (a) ≥ 0.05 to < 1 IU/mL, (b) ≥ 1 to < 10 IU/mL, and (c) ≥ 10 to < 100 IU/mL

3.6. Multiple Comparisons

Not Applicable.

3.7. Missing Data and Outliers

3.7.1. Missing Data

In general, missing data will not be imputed unless methods for handling missing data are specified. Exceptions are presented in this document.

For missing last dosing date of study drug, imputation rules are described in Section 4.1.1.1, the handling of missing or incomplete dates for AE onset is described in Section 7.1.5.2, and for prior and concomitant medications in Section 7.4.

For primary and key secondary endpoints analysis, imputation rules are described in Section 6.1.2 and 6.2.1.

3.7.2. Outliers

Outliers will be identified during the data management and data analysis process, but no sensitivity analyses will be conducted. All data will be included in the data analysis.

3.8. Data Handling Conventions and Transformations

The following conventions will be used for the imputation of date of birth when it is partially missing or not collected:

- If only month and year of birth is collected, then “15” will be imputed as the day of birth
- If only year of birth is collected, then “01 July” will be imputed as the day and month of birth
- If year of birth is missing, then date of birth will not be imputed.

If age at Day 1 is not available for a subject, then age derived based on date of birth and the Day 1 visit date will be used instead. If an enrolled subject was not dosed with any study drug, the randomization date will be used instead of the Day 1 visit date. For screen failures, the date the first informed consent was signed will be used for the age derivation. Age required for longitudinal and temporal calculations and analyses (e.g age at date of AE) will be based on age derived from date of birth and the date of the measurement or event, unless otherwise specified.

Data that are continuous in nature but are less than the lower limit of quantitation (LLOQ) or above the upper LLOQ will be imputed as follows:

- A value that is 1 unit less than the LLOQ will be used to calculate descriptive statistics if the datum is reported in the form of "< x" (where x is considered the LLOQ). For example, if the values are reported as < 50 and < 5.0, values of 49 and 4.9, respectively, will be used to calculate summary statistics. An exception to this rule is any value reported as < 1 or < 0.1, etc. For values reported as < 1 or < 0.1, a value of 0.9 or 0.09, respectively, will be used to calculate summary statistics.

- A value that is 1 unit above the LLOQ will be used to calculate descriptive statistics if the datum is reported in the form of "> x" (where x is considered the LLOQ). Values with decimal points will follow the same logic as above.

- The LLOQ will be used to calculate descriptive statistics if the datum is reported in the form of "≤ x" or "≥ x" (where x is considered the LLOQ).

3.9. Analysis Visit Windows

Subject visits might not occur on protocol-specified days. Therefore, for the purpose of analysis, observations will be assigned to analysis windows. The analysis visit windows for the parameters at different periods are listed in Table 3-1 - Table 3-6.

- Table 3-1 Analysis Visit Windows for HBsAg/HBsAb Quantitation

Study Period	Analysis Visit	Nominal Study Day	Lower Limit	Upper Limit
Dosing Period	Baseline	1		1
	Week 6	43	2	64
	Week 12	85	65	95
	Week 15	106	96	116
	Week 18	127	117	148
	Week 24	169	149	183
Follow-up Period	Week 28	197	184	211
	Week 32	225	212	239
	Week 36	253	240	267
	Week 40	281	268	295
	Week 44	309	296	323
	Week 48	337	324	
NrtI Discontinuation Monitoring Period	Day 1	1	1	1
	Week 12	85	2	127
	Week 24	169	128	211
	Week 36	253	212	295
	Week 48	337	296	

- Table 3-2 Analysis Visit Windows for HBV DNA Quantitation

Study Period	Analysis Visit	Nominal Study Day	Lower Limit	Upper Limit
Dosing Period	Baseline	1		1
	Week 12	85	2	127
	Week 24	169	128	183
Follow-up Period	Week 28	197	184	211
	Week 32	225	212	239
	Week 36	253	240	267
	Week 40	281	268	295
	Week 44	309	296	323
	Week 48	337	324	
NrtI Discontinuation Monitoring Period	Day 1	1	1	1
	Week 4	29	2	43
	Week 8	57	44	71
	Week 12	85	72	127
	Week 24	169	128	211
	Week 36	253	212	295
	Week 48	337	296	

- Table 3-3 Analysis Visit Windows for Cytokine/Chemokine Biomarker Quantitation

Study Period	Analysis Visit	Nominal Study Day	Lower Limit	Upper Limit
Dosing Period	Baseline	1		1
	Week 6	43	2	64
	Week 12	85	65	106
	Week 18	127	107	148
	Week 24	169	149	183
Follow-up Period	Week 28	197	184	211
	Week 32	225	212	253
	Week 40	281	254	309
	Week 48	337	310	
NrtI Discontinuation Monitoring Period	Day 1	1	1	1
	Week 8	57	2	113
	Week 24	169	114	253
	Week 48	337	254	

This is not applicable to Week 24 Analysis.

- Table 3-4 Analysis Visit Windows for Safety Laboratory Assessments

Study Period	Analysis Visit	Nominal Study Day	Lower Limit	Upper Limit
Dosing Period	Baseline	1		1
	Week 3	22	2	32
	Week 6	43	33	53
	Week 9	64	54	74
	Week 12	85	75	95
	Week 15	106	96	116
	Week 18	127	117	148
	Week 24	169	149	183
Follow-up Period	Week 28	197	184	211
	Week 32	225	212	239
	Week 36	253	240	267
	Week 40	281	268	295
	Week 44	309	296	323
	Week 48	337	324	
NrtI Discontinuation Monitoring Period	Day 1	1	1	1
	Week 4	29	2	43
	Week 8	57	44	71
	Week 12	85	72	127
	Week 24	169	128	211
	Week 36	253	212	295
	Week 48	337	296	

- Table 3-5 Analysis Visit Windows for Vital Signs and Weight

Vital Signs

Study Period	Analysis Visit	Nominal Study Day	Lower Limit	Upper Limit
Dosing Period	Baseline	1		1
	Week 12	85	2	126
	Week 24	169	127	

Weight

Study Period	Analysis Visit	Nominal Study Day	Lower Limit	Upper Limit
Dosing Period	Baseline	1		1
	Week 18	127	2	148
	Week 24	169	149	253
Follow-up Period	Week 48	337	254	

- Table 3-6 Analysis Visit Windows for 12-lead ECG

Study Period	Analysis Visit	Nominal Study Day	Lower Limit	Upper Limit
Dosing Period	Baseline	1		1
	Week 24	169	2	

3.9.1. Selection of Data in the Event of Multiple Records in an Analysis Visit Window

Depending on the statistical analysis method, single values may be required for each analysis window. For example, change from baseline by visit usually requires a single value, whereas a time-to-event analysis would not require 1 value per analysis window.

If multiple valid, nonmissing measurements exist in an analysis window, records will be chosen based on the following rules if a single value is needed:

- For baseline, the last nonmissing value on or prior to the first dosing date of study drug will be selected, unless specified differently. If there are multiple records with the same time or no time recorded on the same day, the baseline value will be the average of the measurements for continuous data, or the measurement with the lowest severity (e.g., normal will be selected over abnormal for safety electrocardiogram findings) for categorical data, and the most conservative value will be selected for efficacy data (e.g., negative will be selected over positive for HBsAg, whereas positive will be selected over negative for anti-HBs).

- For postbaseline values:

    - Categorical variables:

— The most conservative value (e.g., abnormal will be selected over normal for safety ECG) within the analysis window will be selected, except for HBV serology (HBsAg, anti-HBs), for which the most favorable value (i.e., <LLOQ will be selected over positive for HBsAg, whereas positive will be selected over negative for anti-HBs) will be selected.

    - Continuous variables:

— If there are more than 1 value within an analysis window, the value collected closet to the nominal day will be selected.

— If there are 2 records that are equidistant from the nominal day, the later record will be selected.

— If there is more than 1 record on the selected day, the latest record will be selected; if these measurements were recorded at the same time or no time recorded, the average will

be taken for continuous data and the worse severity will be taken for categorical data, unless otherwise specified.

4. SUBJECT DISPOSITION

4.1. Subject Enrollment and Disposition

A summary of subject enrollment will be provided by treatment groups for each investigator and site name within a country using All Randomized Analysis Set. The summary will present the number and percentage of subjects enrolled. For each column, the denominator for the percentage calculation will be the total number of subjects analyzed for that column.

If there are discrepancies in the value used for stratification assignment between the IWRS and the clinical database, the value collected in the clinical database will be used for the summary. A listing of subjects with discrepancies in the value used for stratification assignment between the IWRS and the clinical database at the time of data finalization will be provided.

A summary of subject disposition will be provided by treatment groups. This summary will present the number of subjects in each of the categories listed below:

- All subjects screened
- Screen failure
- All subjects randomized
- All subjects treated with BRII-179/placebo
- All subjects completed Week 18 BRII-179/placebo
- All subjects completed Week 24 PEG-IFN-α
- All subjects completed Week 18 BRII-179/placebo and Week 24 PEG-IFN-α
- Premature discontinuation of BRII-179/placebo and reasons
- Premature discontinuation of PEG-IFN-α and reasons
- Completed study
- Did not complete the study with reasons for premature discontinuation of study

For the status of study drug and study completion and reasons for premature discontinuation, the number and percentage of subjects in each category will be provided. The denominator for the percentage calculation will be the total number of subjects in the All Subjects Randomized corresponding to that column. In addition, a flowchart will be provided to depict the disposition.

The following by-subject listings will be provided by subject identification (ID) number (in ascending order), age, gender, race and screening body weight to support the above summary tables:

- Reasons for premature study drug or study discontinuation
- Reasons for screen failure (will be provided by screening subject ID number in ascending order)

4.1.1. Duration of Exposure to Study Drug

Duration of Treatment is calculated as (Date of Last Dose - Date of First Dose) + 1 for the completers and calculated as (Discontinued Date-Date of First Dose +1) for subjects with prematurely discontinuation, regardless of any temporary interruptions in study drug administration, and will be expressed in weeks using up to 1 decimal place (e.g., 4.5 weeks). If the last study drug dosing date is missing, the latest date among the clinical visit date, laboratory sample collection date, full physical examination, alcohol intake assessment, diary complete, pregnancy test date that occurred during the dosing-treatment period will be used.

Duration of Treatment and Total number of injections for BRII-179 (VBI-2601), placebo, PEG-IFN-α will be summarized, respectively using descriptive statistics (n, mean, STD, Median, Q1, Q3, Min and Max) for the Safety Analysis Set.

No formal statistical testing is planned.

A by-subject listing of study drug administration and drug accountability will be provided separately by subject ID number, age gender, race, screening body weight and visit in chronological order.

4.1.2. Adherence to Study Drug

The level of prescribed adherence to the study drug regimen will be determined by the total doses of study drug taken relative to the total doses of study drug specified by the protocol.

- The level of prescribed adherence of BRII-179 / placebo and PEG-IFN-α will be expressed as a percentage using the following formula:

$$\text{Prescribed Adherence (\%)} = \left(\frac{\text{Total Doses of Study Drug}}{\text{Total Doses of Study Drug Specified by Protocol}}\right) \times 100$$

Descriptive statistics for the level of prescribed adherence with the number and percentage of subjects belonging to adherence categories (< 70%, ≥ 70 to ≤ 80%, > 80%) will be provided by treatment group for the Safety Analysis Set.

The number and percentage of subjects in each dosing frequency will be provided.

No formal statistical testing is planned.

4.2. Protocol Deviations

Protocol deviations occurring after subjects entered the study are documented during routine monitoring and will be provided by a by-subject listing. Important protocol deviations are departures from the protocol that impact subject safety, efficacy assessment or data integrity. The number and percentage of subjects with important protocol deviations, with 1, 2, 3 or more important protocol deviations, and the total number of important protocol deviations and leading to exclusion from Per Protocol Set by deviation reason (e.g., nonadherence to study drug, violation of select inclusion/exclusion criteria) will be summarized by treatment groups for the All Subjects Randomized.

5. BASELINE CHARACTERISTICS

5.1. Demographics and Baseline Characteristics

Subject demographic variables (i.e., age, gender, childbearing potential for female, race, and ethnicity) and baseline characteristics will be summarized by treatment groups and overall using descriptive statistics for continuous variables and using number and percentage of subjects for categorical variables. The summary of demographic data and baseline characteristics will be provided for the All Subjects Randomized. No formal statistical testing is planned.

Baseline characteristics include:

- Body weight (kg)
- Height (cm)
- Body mass index (BMI; kg/m^2) as continuous variable
- Screening HBsAg level as categories: ≥ 0.05 to < 1 IU/mL, ≥1 to < 10 IU/mL, and ≥10 to <100 IU/mL)
- HBsAg quantitative (IU/mL) and Log10 transformed HBsAg quantitative at baseline as continuous variable
- Alanine aminotransferase (ALT) and Aspartate aminotransferase (AST) as a continuous variable and as categories based on central laboratory normal range (≤ upper limit of normal [ULN], > ULN – 2 × ULN, > 2 × ULN – 5 × ULN)
- FibroScan result (kPa) for subjects have available measurement result at screening / within 6 months prior to screening as continuous variable
- Prior PEG-IFN-α type (PEG-IFN-α 2a and PEG-IFN-α 2b) as categories
- HBV Genotype as categories

A by-subject demographics and baseline characteristics listing, including the informed consent date, will be provided by subject ID number (in ascending order), age, gender, race and screening body weight.

5.2. Medical History

Medical history will be collected at screening.

Medical history will be coded using the Medical Dictionary for Regulatory Activities® (MedDRA) Version 26.0 or higher and will be summarized by system organ class (SOC) and preferred term (PT) for each treatment groups and overall by the number and percentage of subjects using the Safety Analysis Set.

No formal statistical testing is planned.

Listing of general medical history data will be provided.

6. EFFICACY ANALYSES

Efficacy endpoints analyses will be based on Full Analysis Set, Serologically Evaluable Full Analysis Set and Per Protocol Set.

The Week 24 Analysis will be performed when approximately 120 subjects enrolled in the Phase 2a part complete the last scheduled PEG-IFN-α treatment and HBsAg results are available or withdraw from the study/discontinue study drug prior to last scheduled PEG-IFN-α treatment.

6.1. Primary Efficacy Endpoint

Definition of the Primary Efficacy Endpoint:

The primary efficacy endpoint of phase 2a part is percentage of subjects with HBsAg loss (< LLOQ 0.05 IU/mL) at Week 24 of completion of PEG-IFN-α treatment, it will be analysed based on Cochran-Mantel-Haenszel method, stratified at randomization by screening HBsAg level (3 categories: ⩾ 0.05 to < 1 IU/mL, ⩾1 to < 10 IU/mL, and ⩾10 to <100 IU/mL). The differences in the percentage of HBsAg loss and associated 95% confidence interval will be presented. If too few cases in a stratum result in failure of stratified analysis, it will be considered to remove the stratification factors and a non-stratified analysis or Fisher's exact probability method will be used to compare the percentage of HBsAg loss between the two groups.

Null hypothesis: there is no difference in percentage of subjects with HBsAg loss at Week 24 between treatment groups.

H_0: P_{trt} - P_{pbo} = 0

Alternative hypothesis: there is a difference in percentage of subjects with HBsAg loss at Week 24 between treatment groups.

H_1: P_{trt} - P_{pbo} ≠ 0

P_{trt} : indicates the percentage of subjects with HBsAg loss at Week 24 in BRII-179 (VBI-2601).

P_{plo} : indicates the percentage of subjects with HBsAg loss at Week 24 in placebo.

6.1.1. Estimand

The estimand for the primary objective is the difference in percentage of participants with HBsAg loss at Week 24 between BRII-179 + PEG-IFN-α and Placebo + PEG-IFN-α group.

Treatment policy strategies will be employed in this trial, i.e., the occurrence of the intercurrent event is considered irrelevant in defining the treatment effect of interest: the value for the variable of interest is used regardless of whether or not the intercurrent event occurs.

The estimand summary information are listed in the below table

Target Population	Analysis set (analysis population)
The target population are defined according to the inclusion/exclusion criteria in the protocol.	Full Analysis Set and Serologically Evaluable Full Analysis Set
Treatment	
BRII-179 + PEG-IFN-α and<br>Placebo+ PEG-IFN-α	
Endpoint	
Percentage of participants with HBsAg loss at week 24	
Handling of intercurrent events	
Treatment policy strategy and composite strategy will be used. Further details are provided after the table.	
Population-level summary measure	Analysis approach
Differences in percentage of participants with HBsAg loss at Week 24 between BRII-179 and Placebo group.	Cochran-Mantel-Haenszel method, stratified at randomization by screening HBsAg level (3 categories) will be used to compare the percentage of HBsAg loss between BRII-179 and Placebo group, Fisher exact probability method will be used instead if too few cases in a stratum resulted in failure of stratified analysis.

Intercurrent events and handling strategies:

**Intercurrent events**	**Primary treatment strategy**	**Remark for the primary strategy**
Pause treatment<br>(intermittent treatment)	Treatment policy	Comply with clinical practice
Discontinue from treatment	Treatment policy	Comply with clinical

prematurely but remain in the trial		practice
Change background NrtI treatment	Treatment policy	Comply with clinical practice
Discontinue from the trial prematurely due to any reasons at IMP dosing period	Composite variable, patient will be treated as treatment failure	Subjects will be regarded as treatment failure no matter any reasons, such as drug tolerance, etc.

6.1.2. Analysis methods for the primary endpoint

Cochran-Mantel-Haenszel method will be used to compare the percentage of subjects with HBsAg loss between two treatment groups at a two-sided alpha level of 0.6 with stratification at randomization by screening HBsAg level (3 categories). Proportion point estimate and the two-sided 95% exact confidence interval will be calculated based on the weighted average of difference for each randomisation stratum using the normal approximation of binomial proportions. Fisher exact probability method will be used instead if too few cases in a stratum resulted in failure of stratified analysis.

The point estimates and the 2-sided 95% exact CIs based on the Clopper-Pearson method will also be provided for the percentages in each treatment group.

Missing data for the endpoint will be imputed as treatment failure.

The SAS programming specification is listed in appendix 1.

The estimand together with the strategies of treatment policy and composite strategy to handle intercurrent events will serve as primary endpoints analysis.

Subgroup analysis stratified by HBsAg level (3 categories) at screening will be conducted with respect to the percentage of subjects with HBsAg loss.

A Forest plot will graphically present the point estimates and the CIs on the percentage difference (BRII-179 + PEG-IFN-α – Placebo + PEG-IFN-α) by Age, Sex and HBsAg level at screening and overall for each treatment group.

The percentage of subjects with HBsAg loss at Week 24 between two treatment groups will be compared using Full Analysis Set, Serologically Evaluable Full Analysis Set and Per Protocol Set.

6.2. Secondary Efficacy Endpoints

- Percentage of subjects with sustained HBsAg loss and persistently undetectable HBV DNA (defined as undetectable HBsAg and HBV DNA for at least 24 weeks) after NrtI discontinuation
- HBsAg change from baseline from Day 1 to completion of PEG-IFN-α treatment
- Percentage of subjects with HBsAg loss at any timepoint
- Percentage of subjects with undetectable HBV-DNA (HBV DNA < Lower Limit of Quantification (LLOQ)) 24 and 48 weeks after NrtI discontinuation
- Percentage of subjects meeting the NrtI discontinuation criteria by end of follow-up period (Week 48)
- Percentage of subjects with HBsAg seroconversion at any timepoint
- Percentage of subjects with hepatitis B surface antibody (HBsAb) response as of the end of the follow-up period (Week 48)
- Time to achieve HBsAg seroclearance and seroconversion
- Percentage of subjects requiring NrtI re-treatment during follow up

6.2.1. Analysis methods for the secondary endpoints

Percentage of subjects with HBsAg loss and in 3 categories (≥ 0.05 to < 1 IU/mL, ≥1 to < 10 IU/mL, and ≥10 to <100 IU/mL) will be presented for each treatment group by visit. Percentage of subjects with HBsAg seroconversion and percentage of subjects with hepatitis B surface antibody (HBsAb) response will be presented for each treatment group by visit.

Percentage of subjects with HBsAg seroconversion at Week 24, percentage of subjects with hepatitis B surface antibody (HBsAb) response at Week 24, percentage of subjects with sustained HBsAg loss and persistently undetectable HBV DNA after NrtI Discontinuation and percentage of subjects meeting NrtI discontinuation criteria by the end of follow-up period will be analysed with Cochran-Mantel-Haenszel method in the same way as for the primary endpoint. Missing data for these endpoints will be imputed as treatment failure.

Percentage of subjects with HBsAg loss at any timepoint and percentage of subjects requiring NrtI re-treatment during follow up will be summaried only.

Percentage of below items and in 3 HBsAg categories at screening (≥ 0.05 to < 1 IU/mL, ≥1 to < 10 IU/mL, and ≥10 to <100 IU/mL) will also be summarized between treatment groups by visit:

- HBsAg (IU/mL): <0.05, >=0.05 - <1, >=1 - <10, >=10 - <100 and >=100;
- HBsAb (IU/L): <2, >=2 - <10, >=10 - <100 and >=100.

Time to achieve HBsAg seroclearance and seroconversion will be compared using stratified log-rank test, Kaplan-Meier estimates (product-limit estimates) will be presented by treatment groups together with the median time of achieving HBsAg seroclearance and seroconversion, respectively.

Kaplan-Meier plots will be created for treatment groups for the these endpoints. Censoring rules are listed in the below table

	Data of event/censoring	Censoring
Subjects who don't achieve the HBsAg seroclearance	Time from Day 1 to last HBsAg, Anti-HBs sample collection date, respectively.<br><br>HBsAg seroclearance:<br>Subjects with negative HBsAg at baseline or without any postbaseline HBsAg measurement will be censored at Day 1.<br><br>HBsAg seroconversion:<br>The subjects meeting i) Subjects with negative HBsAg or positive HBsAb at baseline; or ii) HBsAg or HBsAb is missing at the same postbaseline measurement date throughout the study will be censored at Day 1.	Y
Subjects who don't achieve the HBsAg seroconversion		
Subjects who achieve the HBsAg seroclearance	Time from Day 1 to date of achieving the target.	N
Subjects who achieve the HBsAg seroconversion		

Plots of the mean (SD) of absolute values and changes from baseline in $\log_{10}$ HBsAg quantitative over time by visit will be presented by screening HBsAg level (3 categories) and overall for each treatment group. Continuous endpoint $\log_{10}$ HBsAg change from baseline at Week 24 will be compared using Wilcoxon ranks sum test by adjusting HBsAg level at screening, Missing data at Week 24 will be imputed as the maximum value at the same visit in the same treatment group.

The SAS programming specification is listed in appendix 1.

By-subject listings will be provided by subject ID number, age, gender, race and screening body weight in chronological order.

6.3. Tertiary Efficacy Endpoints

Assessment of plasma protein level cytokine/chemokine biomarkers changes from baseline post dosing, i.e., from Day 1 of dosing to the end of follow-up (Week 48), and during the NrtI Discontinuation Monitoring Period.

6.3.1. Analysis methods of the tertiary efficacy endpoints

When applicable, the biomarkers such as IFN-γ, TNF-α, IL-10, IL-21, IL-12p70, CXCL10 (IP10), CXCL11 (I-TAC), CCL2 (MCP-1) CCL4 (MIP-1β) and CD40L will be summarized by treatment groups, overall and visit using Biomarker Analysis Set.

6.4. Changes From Protocol-Specified Efficacy Analyses

Estimated probability of success using Bayesian modeling is added to support decision making and optimal selection of options in recommendation letter.

7. SAFETY ANALYSES

7.1. Adverse Events and Deaths

7.1.1. Adverse Event Dictionary

Adverse events (AEs) will be coded using the MedDRA Version 26.0 or higher. System organ class (SOC), high-level group term (HLGT), high-level term (HLT), preferred term (PT), and lower-level term (LLT) will be provided in the AE dataset.

7.1.2. Adverse Event Severity

Adverse events are graded by the investigator as Grade 1, 2, 3, 4, or 5 according to CTCAE Version 5.0 grading scale specified in the protocol. The severity grade of events for which the investigator did not record severity will be categorized as "missing" for tabular summaries and data listings. The missing category will be listed last in summary presentation.

7.1.3. Relationship of Adverse Events to Study Drug

Related AEs are those for which the investigator selected "Related" on the AE CRF to the question of "Relationship to Brii-179/Placebo" and "Relationship to PEG-IFN-α. Relatedness will always default to the investigator's choice, not that of the medical monitor. Events for which the investigator did not record relationship to study drug will be considered related to study drug for summary purposes. However, by-subject data listings will show the relationship as missing.

7.1.4. Serious Adverse Events

Serious adverse events (SAEs) will be identified and captured as SAEs if the AEs met the definitions of SAEs that were specified in the study protocol. SAEs captured and stored in the clinical database will be reconciled with the SAE database from the vender before data lock.

7.1.5. Treatment-Emergent Adverse Events

7.1.5.1. Definition of Treatment-Emergent Adverse Events

Treatment-emergent adverse events (TEAEs) are defined as 1 or both of the following:

- Any AE that has an onset date that is on or after the day of study drug administration
- Any AE that has been present before treatment and worsens relative to the pre-treatment state after administration of study drug

7.1.5.2. Incomplete Dates

If the onset date of the AE is incomplete and the AE stop date is not prior to the first dosing date of study drug, then the month and year (or year alone if month is not recorded) of onset determine whether an AE is treatment emergent. The event is considered treatment emergent if the AE onset is the same as or after the month and year (or year) of the first dosing date of study drug.

An AE with completely missing onset and stop dates, or with the onset date missing and a stop date later than the first dosing date of study drug, will be considered to be treatment emergent.

In addition, an AE with the onset date missing and incomplete stop date with the same or later month and year (or year alone if month is not recorded) as the first dosing date of study drug will be considered treatment emergent.

7.1.6. Summaries of Adverse Events and Deaths

Treatment-emergent AEs will be summarized based on the Safety Analysis Set.

A brief, high-level summary of the number of percentage of subjects who experienced at least 1 TEAE in the categories described below will be provided by treatment group. All deaths observed in the study will also be included in this summary.

The number and percentage of subjects who experienced at least 1 TEAE will be provided and summarized by SOC and PT by treatment and overall. For the AE categories described below, summaries will be provided by SOC, PT and treatment group:

- All TEAEs summarized by SOC and PT
- TEAEs with Grade 1, 2, 3, 4 and 5 in overall summary
- TEAEs with Grade 3 or higher
- All TEAEs occurring in >= 5% of subjects
- All TEAEs occurring in >= 10% of subjects
- BRII-179/Placebo related TEAEs
- PEG-IFN-α related TEAEs
- BRII-179/Placebo and PEG-IFN-α related TEAEs
- BRII-179/Placebo related TEAEs of Grade 3 or higher
- PEG-IFN-α related TEAEs of Grade 3 or higher
- BRII-179/Placebo and PEG-IFN-α related TEAEs of Grade 3 or higher

- TEAEs by maximum severity
- Serious TEAEs
- BRII-179/Placebo related serious TEAEs
- PEG-IFN-α related serious TEAEs
- BRII-179/Placebo and PEG-IFN-α related serious TEAEs
- TEAEs leading to premature discontinuation of BRII-179/Placebo
- TEAEs leading to premature discontinuation of PEG-IFN-α
- TEAEs leading to premature discontinuation of study
- Serious TEAEs leading to death (i.e., outcome of death)
- TE (Treatment-emergent) Solicited systemic reactions
- TE Solicited local reactions (injection site reactions)

Multiple events will be counted only once per subject in each summary. Adverse events will be summarized and listed first in descending order of SOC (and HLT within each SOC if applicable) based on total frequency and then by PT in descending order of total frequency within each SOC. For summaries by severity, the most severe severity will be used for those AEs that occurred more than once in a given subject during the study.

In addition to the above summary tables, all TEAEs, serious TEAEs, treatment-related TEAEs, and treatment-related serious TEAEs will be summarized by PT only in descending order of total frequency.

The counts of TEAEs with grade >= 3 will be compared between BRII-179/placebo and PEG-IFN-α under the assumption of zero-inflated Poisson distribution or zero-inflated negative binominal distribution, the final model selection will be based on the assessing results of goodness of fit (Akaike Information Criterion (AIC) value (the smaller the better) will be used to evaluate relative quality of the regression models, and p-value (>0.05)).

In addition, data listings will be provided for the following:

- All AEs, indicating whether the event is treatment emergent
- All SAEs
- All Deaths

- All SAEs leading to death (i.e., outcome of death)
- All AEs leading to premature discontinuation of BRII-179/placebo
- All AEs leading to premature discontinuation of PEG-IFN-α
- All AEs leading to premature discontinuation of study
- Solicited Local Reaction/Injection Site Reaction

7.2. Laboratory Evaluations

Laboratory data collected during the study will be analyzed and summarized using both quantitative and qualitative methods. Summaries of laboratory data will be provided for the Safety Analysis Set and will include data throughout the trial. If a subject has repeated laboratory values for a given time point, the value from the last evaluation will be used.

Subjects who have permanently discontinued study drug, or have available data at the time of the database snapshot for an analysis will be included in laboratory data analysis. The analysis will be based on values in standard units. When values are below the LLOQ, they will be listed as such, and the closest imputed value will be used for the purpose of calculating summary statistics. Hemolized test results will not be included in the analysis, but they will be listed in by-subject laboratory listings.

A by-subject listing for laboratory test results will be provided by subject ID number, age, gender, race, screening body weight and visit in chronological order for hematology, chemistry including liver function tests, coagulation parameters, urinalysis and thyroid panel separately. Values falling outside of the relevant reference range and/or having a severity grade of 1 or higher on the CTCAE 5.0 severity grading scale will be flagged in the data listings, as appropriate. Unscheduled laboratory test results will be also listed.

No formal statistical testing is planned.

7.2.1. Summaries of Numeric Laboratory Results

Descriptive statistics will be provided by treatment for each laboratory test specified in the study protocol as follows:

- Baseline values
- Values at each postbaseline visit
- Change from baseline at each postbaseline visit

A baseline laboratory value will be defined as the last measurement obtained on or prior to the date/time of first dose of study drug. Change from baseline to a postbaseline visit will be defined as the visit value minus the baseline value.

7.2.2. Graded Laboratory Values

The CTCAE Version 5.0 will be used to assign toxicity grades (1 to 5) to laboratory results for analysis. For laboratory tests with criteria for both increased and decreased levels, analyses for each direction (i.e., increased, decreased) will be presented separately.

7.2.2.1. Treatment-Emergent Laboratory Abnormalities

Treatment-emergent laboratory abnormalities are defined as values that increase at least 1 toxicity grade from baseline at any postbaseline visit. If the relevant baseline laboratory value is missing, any abnormality of at least Grade 1 observed within the time frame specified above will be considered treatment emergent.

7.2.2.2. Summaries of Laboratory Abnormalities

Laboratory data that are categorical will be summarized using the number and percentage of subjects in the study with the given response at baseline and each scheduled postbaseline visit.

The following summaries (number and percentage of subjects) for treatment-emergent laboratory abnormalities will be provided by lab test and treatment; subjects will be categorized according to the most severe postbaseline abnormality grade for a given lab test:

- Graded laboratory abnormalities

For all summaries of laboratory abnormalities, the denominator is the number of subjects with nonmissing postbaseline values.

7.2.3. Liver-related Laboratory Evaluations

Liver-related abnormalities after initial study drug dosing will be examined from Day 1 of dosing to the last visit and summarized using the number and percentage of subjects who were reported to have the following laboratory test values for postbaseline measurements:

- Aspartate aminotransferase (AST): (a) > 3 times of the upper limit of reference range (ULN); (b) > 5 x ULN; (c) > 10 x ULN; (d) > 20 x ULN
- Alanine aminotransferase (ALT): (a) > 3 x ULN; (b) > 5 x ULN; (c) > 10 x ULN; (d) > 20 x ULN
- AST or ALT: (a) > 3 x ULN; (b) > 5 x ULN; (c) > 10 x ULN; (d) > 20 x ULN

- Total bilirubin: > 2 x ULN
- Alkaline phosphatase (ALP) > 1.5 x ULN
- AST or ALT > 3 x ULN and total bilirubin: (a) > 1.5 x ULN; (b) > 2 x ULN

The summary will include data from all postbaseline visits. For individual laboratory tests, subjects will be counted once based on the most severe postbaseline values. For both the composite endpoint of AST or ALT and total bilirubin, subjects will be counted once when the criteria are met at the same postbaseline visit date. The denominator is the number of subjects in the Safety Analysis Set who have nonmissing postbaseline values of all relevant tests at the same postbaseline visit date. A listing of subjects who met at least 1 of the above criteria will be provided.

Events of Clinical Interest (ECI) will be summarized and listings will be provided accordingly, defined as the following:

- ALT > 3x ULN, if Day 1 pre-dose ALT ≤ ULN
- ALT > 2x baseline, if Day 1 pre-dose ALT > ULN

7.3. Body Weight, Height, BMI and Vital Signs

The actual values of vital sign parameters including temperature, pulse rate, respiratory rate, and systolic and diastolic blood pressure. Descriptive statistics will be provided by treatment group for vital signs and body weight as follows:

- Baseline value
- Values at each postbaseline visit
- Change from baseline at each postbaseline visit

A baseline value will be defined as the last available value collected on or prior to the date/time of first dose of study drug. Change from baseline to a postbaseline visit will be defined as the postbaseline value minus the baseline value. Vital signs measured at unscheduled visits will be included for the baseline value selection.

No formal statistical testing is planned.

A by-subject listing of vital signs, body weight, height and BMI will be provided by subject ID number, age, gender, race, screening body weight and visit in chronological order.

7.4. Prior and Concomitant Medications

Medications collected at screening and during the study will be coded using the World Health Organization (WHO) Drug dictionary version B3 September 1, 2023 or later (except for prior PEG-IFN-α treatment, prior NrtI therapy and NrtI therapy in the study).

7.4.1. Prior Medications

Prior medications are defined as any medication before a subject took the first dose of study drug.

Prior medications will be summarized by Anatomical Therapeutic Chemical (ATC) drug class Level 2 and preferred name using the number and percentage of subjects for each treatment and overall. A subject reporting the same medication more than once will be counted only once within each ATC drug class when calculating the number and percentage of subjects who received that medication. Medications may appear under multiple ATC drug classes. The summary will be ordered descending by ATC medical class in overall frequency and then by preferred term in order of descending overall frequency within each ATC medical class. For drugs with the same frequency, sorting will be done alphabetically.

For the purposes of analysis, any medication with a start date prior to the first dosing date of study drug will be included in the prior medication summary regardless of when the stop date is. If a partial start date is entered the medication will be considered prior unless the month and year (if day is missing) or year (if day and month are missing) of the start date are after the first dosing date. Medications with a completely missing start date will be included in the prior medication summary, unless otherwise specified.

Summaries will be based on the Safety Analysis Set. No formal statistical testing is planned.

A summary of prior medications will be provided.

7.4.2. Concomitant Medications

Concomitant medications are defined as medications taken after a subject took the first study drug. Use of concomitant medications will be summarized by Anatomical Therapeutic Chemical (ATC) drug class Level 2 and preferred name using the number and percentage of subjects for each treatment. A subject reporting the same medication more than once within each ATC drug class will be counted only once when calculating the number and percentage of subjects who received that medication. Medications may appear under multiple ATC drug classes. The summary will be ordered descending by ATC medical class in overall frequency and then by preferred term in descending overall frequency within each ATC medical class. For drugs with the same frequency, sorting will be done alphabetically.

For the purposes of analysis, any medications with a start date prior to or on the first dosing date of study drug and continued to be taken after the first dosing date, or started after the first dosing date but prior to or on the last dosing date of study drug will be considered concomitant medications. Medications started and stopped on the same day as the first dosing date or the last

dosing date of study drug will also be considered concomitant. Medications with a stop date prior to the date of first dosing date of study drug or a start date after the last dosing date of study drug will be excluded from the concomitant medication summary. If a partial stop date is entered, any medication with the month and year (if day is missing) or year (if day and month are missing) prior to the date of first study drug administration will be excluded from the concomitant medication summary. If a partial start date is entered, any medication with the month and year (if day is missing) or year (if day and month are missing) after the study drug stop date will be excluded from the concomitant medication summary. Medications with completely missing start and stop dates will be included in the concomitant medication summary, unless otherwise specified. Summaries will be based on the Safety Analysis Set. No formal statistical testing is planned.

A summary of concomitant medications will be provided.

All prior and concomitant medications (other than per-protocol study drugs) will be provided in a by-subject listing sorted by subject ID number, age, gender, race, screening body weight and administration date in chronological order.

7.5. Electrocardiogram Results

Electrocardiogram (ECG) will be summarized by treatment groups and visit.

The summary of overall interpretation findings table will present counts and percentages for the reported results at baseline and each post-baseline visit. Result categories will be ordered as ‘Normal’, ‘Abnormal - Not Clinically Significant (NCS)’ and ‘Abnormal - Clinically Significant (CS)’.

The worst post baseline value will be the worst non-missing value after baseline and during the reference period. Scheduled visits, unscheduled visits, and repeat measurements will be included to derive the worst post baseline values (Abnormal CS is the worst, Abnormal NCS is next, then normal).

Additionally, shift tables from baseline in categories as ‘Normal’, ‘Abnormal - Not Clinically Significant (NCS)’, ‘Abnormal - Clinically Significant (CS)’ and ‘Missing’, will be summarized by counts and percentages.

A by-subject listing for ECG assessment results will be provided by subject ID number, age, gender, race, screening body weight and visit in chronological order.

7.6. Other Safety Measures

By-subject listings of physical examination and pregnancy testing result will be provided by subject ID number, gender, age, race, screening body weight and visit in chronological order.

7.7. Changes From Protocol-Specified Safety Analyses

8. EXPLORATORY ANALYSES

8.1. Probability of Success Estimated by Bayesian Model based on HBsAg Loss at Week 24

Probability of success will be estimated using Bayesian modeling based on percentage difference of HBsAg loss at Week 24 between two treatment groups to support DSMB review of the Week 24 efficacy data and recommendation on advance to Phase 2b part or not, as well as the Brii decision making.

8.1.1. Statistical methods

8.1.1.1. Bayesian Modeling

The analysis will consider percentage difference of binary endpoint HBsAg loss at Week 24 between two treatment groups as an outcome, the level of evidence required for decision making how to advance to next stage is expressed in terms of Bayesian probability statements based on posterior probability distributions:

Probability (BRII-179 + PEG-IFN-α is better than Placebo + PEG-IFN-α by at least D) is greater than certain threshold X where D and X are defined as below:

> D is how much higher percentage for this outcome which are predefined as 0.1, 0.15 and 0.2.
>
> X is threshold value.

Take D is 0.2 and X is 0.6 as an example, the Probalility can be interpreted as:

The choice of 0.6 for this probability indicates that there is a 3 to 2 odds of BRII-179 + PEG-IFN-α being better than Placebo + PEG-IFN-α by the desired amount 20%.

Initially non-informative prior distributions for paramerters will be used. Samples from the posterior distributions for all parameters will be obtained using a Markov Chain Monte Carlo (MCMC) sampling technique with 20,000 iterations following a burn-in of size 2000.

Diagnostic tests will be performed to assess convergence and autocorrelation. Alternative programming conventions (thinning, parameterization, number of chains, seed, size of burn-in) may be explored if, using initial programming code, satisfactory mixing is not achieved with computer memory limitations.

The mean for posterior distributions will be presented.

This model will not adjust for additional covariates or variables used in the stratification of randomization as the goal is to describe the differences in outcomes for those receiving BRII-179 + PEG-IFN-α versus Placebo + PEG-IFN-α.

8.1.1.2. Model specification:

The percentage of subjects reaching HBsAg loss at Week 24 will be compared between treatment groups using a Bayesian logistic regression model. The model specification will be:

$$r_{\mathrm{ij}}|p_{\mathrm{ij}} \sim Binary(p_{\mathrm{ij}})$$

$$logit(p_{\mathrm{ij}}) = \beta_0 + \beta_1 x_{ij}$$

Where *i* indexes subjects in group *j* (*j*=1 denotes the placebo group and *j*=2 denotes the treatment group), x_{ij}=0 if *j*=1 and x_{ij}=1 if *j*=2, p_{ij} is the probability of HBsAg loss at Week 24, r_{ij} is 1 if the subjects reached HBsAg loss at Week 24 and zero otherwise, β_0 is the log odds of reaching HBsAg loss at Week 24 in the placebo group, and β_1 is the log odds ratio between the treatment group and placebo group.
The following independent non-informative priors will be used:

$$\beta_0, \beta_1 \sim N(0, 100^2).$$

To obtain samples from the posterior distribution for the difference in the percentage of subjects reaching HBsAg loss at Week 24 in the treatment group (p_2) and the placebo group (p_1), we will transform the samples from the posterior distributions for β_0, β_1:

$$p_1 = \frac{\exp(\beta_0)}{1 + \exp(\beta_0)}$$

$$p_2 = \frac{\exp(\beta_0 + \beta_1)}{1 + \exp(\beta_0 + \beta_1)}$$

$$p_2 - p_1 = \frac{\exp(\beta_0 + \beta_1)}{1 + \exp(\beta_0 + \beta_1)} - \frac{\exp(\beta_0)}{1 + \exp(\beta_0)}$$

Features of the posterior distribution of the difference ($p_2 - p_1$) will be estimated using descriptive statistics of the MCMC samples. Based on the estimated posterior distribution for the difference ($p_2 - p_1$), we will estimate the probability that the difference is greater than D. The probability that the difference in the percentage reaching HBsAg loss at Week 24 between treatment groups is greater than

$$P(p_2 - p_1) > D$$

will be calculated as the number of samples where the difference is greater than D divided by the total number of samples. If this probability is greater than certain threshold, this will be considered as achieving the desired level of evidence for decision making.

The SAS programming specification is listed in appendix 1.

9. SOFTWARE

SAS® Software Version 9.4. SAS Institute Inc., Cary, NC, USA.

10. SAP REVISION

Revision Date (DD MMM YYYY)	Section	Summary of Revision	Reason for Revision
Draft version 0.1<br>09NOV2022			The initial version.

11. APPENDICES

Appendix 1. Programming Specifications

1) Treatment difference in percentages adjusted by stratification factor at screening:

```
ods output
commonrelrisks = _commonrelrisks
crosstabfreqs = _crosstabfreqs
cmh = _cmh
commonpdiff = _commonpdiff
;
proc freq;
tables stratafactor * trt * response/cmh commonriskdiff; * p-value from general association;
run;
```

The percentage difference and the corresponding 95% CIs will be calculated using the stratum-adjusted Mantel-Haenszel (MH) method {Koch 1989}, stratified by HBsAg status at screening, as follows:

$P_1 - P_2 \pm Z_{(1-\alpha/2)} \times SE(P_1 - P_2)$,
where
$(P_1 - P_2) = \frac{\sum w_h d_h}{\sum w_h}$, is the stratum-adjusted MH percentage difference, where
$d_h = P_{1h} - P_{2h}$ is the percentage difference of Groups 1 and 2 in stratum h (h = 1 and 2).
$w_h = \frac{n_{1h} n_{2h}}{n_{1h} + n_{2h}}$, is the weight based on the harmonic mean of sample sizes for stratum h, where n_{1h} and n_{2h} are the sample sizes of Groups 1 and 2 in stratum h.
$SE(P_1 - P_2) = \sqrt{\frac{\sum w_h^2 \left[\frac{p_{1h}^*(1-p_{1h}^*)}{n_{1h}-1} + \frac{p_{2h}^*(1-p_{2h}^*)}{n_{2h}-1}\right]}{(\sum w_h)^2}}$, where $p_{1h}^* = \frac{m_{1h}+0.5}{n_{1h}+1}$ and $p_{2h}^* = \frac{m_{2h}+0.5}{n_{2h}+1}$, where m_{1h} and m_{2h} are the numbers of subjects with HBsAg loss at Week 24 in Groups 1 and 2 in stratum h.

$\alpha = 0.05$ for this study.

$Z_{(1-\alpha/2)} = Z_{0.975} = 1.96$ is the 97.5^{th} percentile of the normal distribution.

If the computed lower confidence bound is less than -1, the lower bound is defined as -1. If the computed upper confidence bound is greater than 1, then the upper bound is defined as 1.

Reference:

Koch GG, Carr GJ, Amara IA, Stokes ME, Uryniak TJ. Categorical Data Analysis. Chapter 13 in Berry, D.A. (ed.). Statistical Methodology in the Pharmaceutical Sciences. New York: Marcel Dekker, Inc., 1989:pp. 414-21.

2) Treatment difference in percentages not adjusted by stratification factor at screening:

```
ods output
crosstabfreqs = _crosstabfreqs
chisq= _chisq
fishersexact= _fishersexact
pdiffcls = _pdiffcls
;
proc freq;
tables trt * response/riskdiff(cl = (exact)) ;
exact fisher riskdiff(method = score) ;
run;
```

The 95% CI for percentage estimate for each treatment is calculated based on the Clopper-Pearson exact method.

```
proc freq;
by trt;
tables event/ binomial;
exact binomial;
run;
```

3) Wilcoxon Rank sum test adjusted by stratification factor at screening:

```
ods output
crosstabfreqs = _crosstabfreqs
cmh = _cmh
;
proc freq;
table stratafactor * trt * response/cmh2 scores = modridit;
run;
```

4) Log-rank test for time to event between treatment groups by stratification factor at screening:

```
ods output
productlimitestimates = _productlimitestimates
homtests = _homtests
quartiles = _quartiles
means = _means
```

```
homstats = _homstats
;
proc lifetest;
strata stratafactor;
time days*censor(1);
test trt;
run;
```

The binary indicator variable (CENSOR) with a value of 0 indicates the time to the event of interest is complete or 1 indicates the time to the event is censored. DAYS is a time to event variable.

5) Zero-inflated Poisson or zero-inflated negative binomial model for comparison of Grade >= 3 TEAEs counts between two treatment groups:

```
%* Section: ZIP model. ;
ods output
modelfit=fit_zip
lsmeans = lsmns_zip
parameterestimates = paramesti_zip
diffs = dif_zip
;
proc genmod data = ae_all_final;
  class trt01an ;
  model cnt_event_grd3 = trt01an /dist=zip link=log offset=log_follow_up_dur;
  zeromodel /link = logit;
  lsmeans trt01an / /*bylevel e*/ diff pdiff cl exp /*or ilink*/ ;
   output out=zip predicted=pred pzero=pzero;
run;
ods output close ;

%* Section: ZINB Model ;

ods output
modelfit=fit_zinb
lsmeans = lsmns_zinb
parameterestimates = paramesti_zinb
diffs = dif_zinb
;
proc genmod data = ae_all_final;
  class trt01an ;
  model cnt_event_grd3 = trt01an /dist=zinb link=log offset=log_follow_up_dur;
  zeromodel /link = logit;
```

```
  lsmeans trt01an / /*bylevel e*/ diff pdiff cl exp /*or ilink*/ ;
   output out=zinb predicted=pred pzero=pzero;
run;
ods output close ;
```

6) Bayesian model:

```
ods graphics on;
proc mcmc data=trials dic mchistory=brief nbi=2000 nmc=20000 stats=all plots(smooth)
monitor=(prob1 prob2 prob3)
    outpost =post stats(alpha=(0.05) percentage=(2.5 25 50 75 97.5)) seed=20220802 ;
    ods output postsummaries = postsummaries_res ;
    ods output postintervals = postintervals_res ;

    parms (beta0 beta1) 0 ;
    prior beta: ~normal(0, var=10000);

    mu = beta0 + beta1*trtpn ;
    p = logistic(mu);

    model resp ~ binary(p);

        begincnst;
                px1 = 0.10;
                px2 = 0.15;
                px3 = 0.20;
        endcnst;

        beginprior;
            pc = logistic(beta0);
            pt = logistic(beta0 + beta1);
            p_dif = pt - pc ;

                prob1 = (p_dif > px1);
                prob2 = (p_dif > px2);
                prob3 = (p_dif > px3);
        endprior;
run;
ods graphics off;
```

STATISTICAL ANALYSIS PLAN

**Study Title:**	A Multicenter, Randomized, Double-Blind, Placebo-Controlled, Parallel-Group Study to Investigate the Efficacy and Safety of BRII-179 (VBI-2601) for the Treatment of Chronic Hepatitis B Virus (HBV) Infection
**Name of Test Drug:**	BRII-179 (VBI-2601)
**Study Number:**	BRII-179-002
**Protocol Version (Date):**	5.0 (09 December 2022)
**Analysis Type:**	Phase 2a Analysis
**Analysis Plan Version:**	2.0
**Analysis Plan Date:**	30 NOVEMBER 2023
**Analysis Plan Author(s):**	[redacted]

CONFIDENTIAL AND PROPRIETARY INFORMATION

SIGNATURE PAGE

Reason for Signature	Name	Signature	Date (DDMMMYYYY)
Author	[redacted]		
Therapeutic Area Head (or delegate)			
Biometrics Head (or delegate)			

TABLE OF CONTENTS

TABLE OF CONTENTS ... 3
LIST OF ABBREVIATIONS ... 6
1. INTRODUCTION ... 8
1.1. Study Objectives ... 8
1.2. Study Design ... 9
1.3. Sample Size and Power ... 10
1.3.1 Phase 2a Part ... 10
2. TYPE OF PLANNED ANALYSIS ... 12
2.1. Week 24 Analysis ... 12
2.2. Week 36 Analysis ... 12
2.3. Week 48 Analysis ... 12
2.4. NDMP Week 24 Analysis ... 13
2.5. Final Analysis ... 13
3. GENERAL CONSIDERATIONS FOR DATA ANALYSES ... 14
3.1. Key Definitions ... 15
3.2. Analysis Sets ... 17
3.2.1. All Subjects Screened ... 17
3.2.2. All Subjects Randomized ... 17
3.2.3. Full Analysis Set ... 17
3.2.4. Per Protocol Set ... 17
3.2.5. Safety Analysis Set ... 18
3.2.6. Serologically Evaluable Full Analysis Set ... 18
3.2.7. Biomarker Analysis Set ... 18
3.3. Subject Grouping ... 18
3.4. Strata and Covariates ... 18
3.5. Examination of Subject Subgroups ... 19
3.6. Multiple Comparisons ... 19
3.7. Missing Data and Outliers ... 19
3.7.1. Missing Data ... 19
3.7.2. Outliers ... 20
3.8. Data Handling Conventions and Transformations ... 20
3.9. Analysis Visit Windows ... 20
3.9.1. Selection of Data in the Event of Multiple Records in an Analysis Visit Window ... 23
4. SUBJECT DISPOSITION ... 25
4.1. Subject Enrollment and Disposition ... 25
4.1.1. Duration of Exposure to Study Drug ... 26
4.1.2. Adherence to Study Drug ... 26
4.2. Protocol Deviations ... 27
5. BASELINE CHARACTERISTICS ... 28
5.1. Demographics and Baseline Characteristics ... 28
5.2. Medical History ... 28
6. EFFICACY ANALYSES ... 30

6.1. Primary Efficacy Endpoint 30
6.1.1. Estimand 30
6.1.2. Analysis methods for the primary endpoint 32
6.2. Secondary Efficacy Endpoints 33
6.2.1. Analysis methods for the secondary endpoints 33
6.3. Tertiary Efficacy Endpoints 35
6.3.1. Analysis methods of the tertiary efficacy endpoints 35
6.4. Changes From Protocol-Specified Efficacy Analyses 35

7. SAFETY ANALYSES 36

7.1. Adverse Events and Deaths 36
7.1.1. Adverse Event Dictionary 36
7.1.2. Adverse Event Severity 36
7.1.3. Relationship of Adverse Events to Study Drug 36
7.1.4. Serious Adverse Events 36
7.1.5. Treatment-Emergent Adverse Events 36
7.1.5.1. Definition of Treatment-Emergent Adverse Events 36
7.1.5.2. Incomplete Dates 36
7.1.6. Summaries of Adverse Events and Deaths 37
7.2. Laboratory Evaluations 39
7.2.1. Summaries of Numeric Laboratory Results 39
7.2.2. Graded Laboratory Values 40
7.2.2.1. Treatment-Emergent Laboratory Abnormalities 40
7.2.2.2. Summaries of Laboratory Abnormalities 40
7.2.3. Liver-related Laboratory Evaluations 40
7.3. Body Weight, Height, BMI and Vital Signs 41
7.4. Prior and Concomitant Medications 42
7.4.1. Prior Medications 42
7.4.2. Concomitant Medications 42
7.5. Electrocardiogram Results 43
7.6. Other Safety Measures 43

8. EXPLORATORY ANALYSES 44

8.1. Probability of Success Estimated by Bayesian Model based on HBsAg Loss at Week 24 44
8.1.1. Statistical methods 44
8.1.1.1. Bayesian Modeling 44
8.1.1.2. Model specification 45
8.2. Week 36 Analysis, Week 48 Analysis, NDMP Week 24 Analysis and Final Analysis 46
8.2.1. Summary of HBsAg Loss, HBsAg Seroconversion and HBsAb Levels at different timepoint and any Timepoint up to different timepoint 46
8.2.2. Summary of HBsAg Loss in Subjects Achieved HBsAb Response and with Different HBsAb Levels 47
8.2.3. Summary of Any HBsAg Rebound after Week 24 in Subjects Achieved HBsAg Loss at Week 24 47
8.2.4. Summary of HBsAb Levels in Subjects with Different HBsAg Loss Status at Any Postbaseline Timepoint 47
8.2.5. Summary of HBsAg and HBsAb Levels Change from Baseline at Any Postbaseline Timepoint 48
8.2.6. The Impact of Treatment, Maximum Postbaseline HBsAb Level or HBsAb Response and HBsAg Level at Screening on HBsAg Loss 48
8.2.7. Sensitivity Analysis of Modified Sustained HBsAg Loss and Persistently Undetectable HBV DNA after NrtI Discontinuation 48

9. SOFTWARE 49
10. SAP REVISION 50
11. APPENDICES 52

LIST OF ABBREVIATIONS

AE	adverse event
ALT	alanine aminotransferase
AST	aspartate aminotransferase
ATC	Anatomical Therapeutic Chemical
BMI	body mass index
CI	confidence interval
CS	Clinically Significant
CSR	clinical study report
CTCAE	Common Toxicity Criteria for Adverse Events
DSMB	Data and Safety Monitoring Board
ECG	Electrocardiogram
eCRF	electronic case report form
FAS	Full Analysis Set
HBsAb	Hepatitis B surface antibody
HBsAg	Hepatitis B surface antigen
HBV	Hepatitis B virus
HLT	high-level term
ICH	International Conference on Harmonization (of Technical Requirements for Registration of Pharmaceuticals for Human Use)
IWRS	Interactive voice or web response system
LLOQ	Lower limit of quantitation
LOCF	Last observation carried forward
MedDRA	Medical Dictionary for Regulatory Activities
NCS	Not Clinically Significant
NrtI	Nucleos(t)ide reverse transcriptase inhibitor
PEG-IFNα	Pegylated interferon-alpha
PPS	per protocol set
PT	preferred term
Q1, Q3	first quartile, third quartile
SAE	serious adverse event
SAP	statistical analysis plan
SD	standard deviation
SI (units)	international system of units
SOC	system organ class
TE	Treatment-emergent
TEAE	treatment-emergent adverse event
TFLs	tables, figures, and listings

ULN	upper limit of normal
WHO	World Health Organization

1. INTRODUCTION

BRII-179 (VBI-2601) is a protein-based immunotherapeutic intended for the treatment of individuals chronically infected with hepatitis B virus (HBV).

Study BRII-179-002 is a multicenter, double-blind, randomized, placebo-controlled, parallel-group phase 2 study aims to evaluate the clinical effect of adding BRII-179 (VBI-2601) to existing pegylated interferon-alpha (PEG-IFNα) and nucleos(t)ide reverse transcriptase inhibitor (NrtI) therapy in Chinese patients with chronic HBV infection without cirrhosis. It will consist of two parts, Part 2a and 2b, of which 120 subjects are planned to be enrolled in the first part (Phase 2a) and 480 subjects are planned to be enrolled in the second part (Phase 2b).

This statistical analysis plan (SAP) describes the statistical analysis methods and data presentations to be used in tables, figures, and listings (TFLs) of the analysis of phase 2a part. This SAP is based on the study protocol version 5.0 dated 09 December 2022 and the electronic case report form (eCRF version 2.2 dated 24 February 2023). The SAP will be finalized before database freeze for Week 24 Analysis which primary endpoint analysis will be included. Any changes made after the finalization of the SAP will be documented in the CSR.

1.1. Study Objectives

The primary objective of Study BRII-179-002 phase 2a part is:

- To evaluate the efficacy of BRII-179 to increase HBsAg loss through completion of PEG-IFNα treatment

The secondary objectives of Study BRII-179-002 phase 2a part are as follows:

- To evaluate functional cure of subjects with chronic HBV infection receiving BRII-179 (VBI-2601) therapy in combination with PEG-IFNα + NrtI
- To assess the antiviral activity of BRII-179 (VBI-2601) therapy in combination with PEG-IFNα + NrtI
- To assess safety of BRII-179 (VBI-2601) therapy

The tertiary objective of Study BRII-179-002 phase 2a part is:

- To assess the effect on relevant immune markers in subjects with chronic HBV infection treated with BRII-179 (VBI-2601) in combination with PEG- IFNα + NrtI

1.2. Study Design

Study BRII-179-002 is a double-blind, randomized, placebo-controlled, parallel-group study aims to evaluate the efficacy and safety of adding BRII-179 (VBI-2601) to existing PEG-IFNα and NrtI therapy in Chinese patients with chronic HBV infection without cirrhosis.

A large part of subjects in the current study are anticipated to be recruited from the Everest Project (NCT04035837). The Everest Project is a multicenter real-world study being conducted in over 260 hospitals in China. The plan is to recruit 30,000 patients with chronic HBV infection effectively treated with NrtI therapy for at least one year; who are hepatitis B e-antigen (HBeAg) negative with HBV DNA <100 IU/ml and HBsAg <1500 IU/mL at study entry. As part of the study participation, subjects receive PEG-IFNα as “add-on” or “switch to” treatment for 48 to 96 weeks. The primary objective of the Everest Project is to achieve functional cure of HBV infection by optimizing treatment strategies of NrtIs and PEG-IFNα.

Eligible subjects who have been receiving PEG-IFNα for 24 to 28 doses and first-line NrtI therapy for at least 12 months with HBV DNA < lower limit of quantification (LLOQ) for ≥ 6 months and HBsAg ≥ 0.05 IU/mL and < 100 IU/mL at screening, will be randomized 1:1 to receive BRII-179 (VBI-2601) or 0.9% Sodium Chloride placebo in addition to the existing PEG-IFNα and NrtI therapy. Subjects will be stratified at randomization by screening HBsAg level (3 categories: ≥0.05 to < 1 IU/mL, ≥1 to < 10 IU/mL, and ≥10 to <100 IU/mL).

In Dosing Period, subjects will have investigational treatment with BRII-179 or placebo intramuscular injection every three weeks for a total of seven doses over an 18-week course. Subjects in both BRII-179 group and placebo group will be treated with PEG-IFNα for another 24 weeks after enrollment; while NrtI treatment will continue into the Follow-up Period for all subjects.

Estimated total duration for each subject will include the following: screening up to 28 days, dosing period 24 weeks (18 weeks BRII-179 or placebo investigational treatment; 24 weeks PEG-IFNα treatment), and a 24-week follow-up period. Eligible subjects that meet NrtI discontinuation criteria (eligibility assessed at Week 36 to Week 48) may enter a NrtI discontinuation monitoring period for an additional 48 weeks from the day of NrtI discontinuation. See Figure 1 for the BRII-179-002 study design schema.

An independent DSMB will monitor subject safety and treatment efficacy data for this study. The details for the operation and responsibilities of the DSMB will be defined in a separate DSMB Charter. The Charter will delineate the composition, duties, responsibilities, and procedures of the DSMB, data required at each meeting, as well as the analyses that will be conducted.

Figure 1 Schema for BRII-179-002 study design

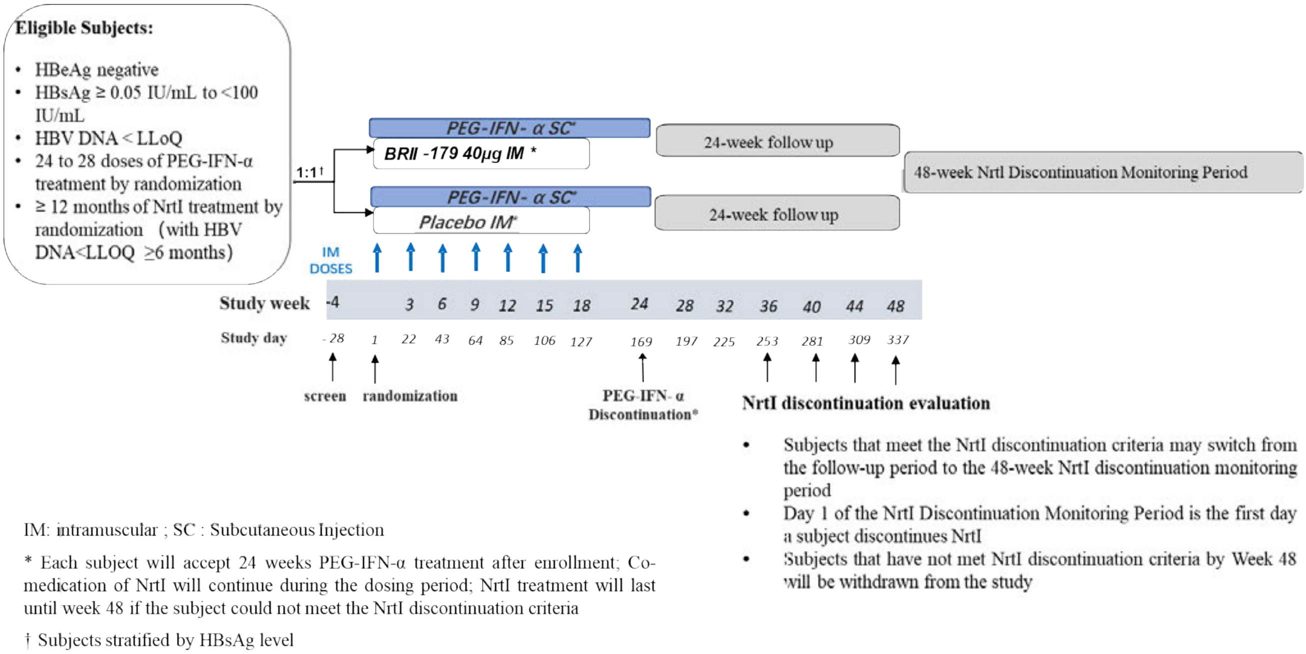

1.3. Sample Size and Power

1.3.1 Phase 2a Part

The early futility evaluation based on an intermediate outcome of efficacy using the HBsAg loss at completion of 24-week PEG-IFNα treatment will be conducted when approximately 120 subjects complete 18 weeks BRII-179 or placebo investigational treatment and 24 weeks PEG-IFNα treatment or withdraw the study prior to last scheduled PEG-IFNα treatment.

The Everest project data suggest 25% subjects can achieve HBsAg loss after 24 weeks treatment of PEG-IFNα + NrtI. The 20% difference in percentage of HBsAg loss is considered to be a clinically meaningful increase in HBsAg clearance with the addition of BRII-179 to PEG-IFNα and NrtI therapy, based on this point, with the assumption of a 25% of HBsAg loss by placebo in combination with PEG-IFNα and NrtI treatment, a 45% of HBsAg loss by BRII-179 in combination with PEG-IFNα and NrtI treatment, and one-sided Type I error α 0.3, The sample size in range of 105 to 120 can provide power of more than 95% to reject the null hypothesis (Table 1). In this sample size estimation, power calculation method is normal approximation and test method is Z-test (SAS 9.4).

Table 1. The power calculated based on different sample size

HBsAg loss		Total Sample Size	Power	Alpha (1-sided)
Placebo + PEG-IFNα + NrtI	BRII-179 + PEG-IFNα + NrtI			
25%	45%	105	95.1%	0.3
		110	95.7%	
		115	96.0%	
		120	96.5%	

2. TYPE OF PLANNED ANALYSIS

2.1. Week 24 Analysis

Primary endpoint analysis timepoint is defined as approximately 120 subjects in total who have (1) completed 18 weeks BRII-179 or placebo investigational treatment and 24 weeks PEG-IFNα treatment, and (2) Week 24 visit HBsAg results are available; or withdraw from the study/discontinue study drug prior to last scheduled PEG-IFNα treatment. An early futility analysis based on HBsAg loss will be performed on above timepoint. The sample size of 120 subjects provides above 95% power to reject the null hypothesis at the 0.30 (one-sided) level of significance.

The primary endpoint and key secondary efficacy and safety endpoints by Week 24 will be analyzed, an independent DSMB will review the outputs and will build the rationale for the recommendation on whether to proceed to Phase 2b part or not.

Brii will make the decision on whether to proceed to Phase 2b part or not based on HBsAg loss futility analysis result at completion of last scheduled PEG-IFNα treatment and safety data analysis, as well as the recommendation provided by the independent DSMB.

The DSMB's role and responsibilities and the scope of analysis to be provided to the DSMB are provided in a mutually agreed upon charter that defines the DSMB membership, meeting logistics and meeting frequency.

2.2. Week 36 Analysis

To further evaluate the 3-month off-treatment HBsAg loss and HBsAb response, the key secondary efficacy and safety endpoints by Study Week 36 will be analysed.

A treatment group-level unblinding of data as of the cut-off date when last subject completed the Week 36 visit/NrtI discontinuation monitoring period Day 1 visit or prematurely discontinued from the study will be provided by the independent unblinded team for this Week 36 analysis.
The summarized efficacy and safety results by treatment group from highly selected tables and figures as of the data cut-off date will be provided to limited Brii personnel. Investigators, subjects and study team will remain blinded to the individual-level data until study Week 48.

2.3. Week 48 Analysis

To further evaluate key secondary efficacy and safety endpoints 24 weeks after completion of PEG-IFN-α treatment (Week 24), individual level unblinding will be conducted after all subjects completed study Week 48 or prematurely discontinued from the study. In this analysis sponsor study team will be unblinded, but investigators and subjects maintained blinded as before.

2.4. NDMP Week 24 Analysis

To evaluate key secondary efficacy and safety endpoints 24 weeks after NRTI discontinuation, NDMP Week 24 Analysis will be conducted after all subjects who entered NDMP completed NDMP Week 24 visit or prematurely discontinued from the study.

2.5. Final Analysis

The final analysis will be conducted after all enrolled subjects have completed Phase 2a of the study or prematurely discontinued from the study.

3. GENERAL CONSIDERATIONS FOR DATA ANALYSES

All data collected on the electronic case report form (eCRF) will be presented in the data listings and will be listed by subject identification number (in ascending order), age, gender, race and screening body weight and visit, where applicable. All summaries will be presented by treatment groups.

When reporting descriptive statistics, the following rules will apply:

- Continuous variables: Descriptive statistics will include the number of non-missing values (n), arithmetic mean, standard deviation (SD), median, first quartile (Q1), third quartile (Q3), minimum (Min) and maximum (Max) values.

  The minimum and maximum values will be displayed to the same decimal precision as the source data; the Q1, Q3, arithmetic mean and median values will be displayed to one more decimal than the source data; SD value will be displayed to two more decimal than the source data for the specific variable.

  The appropriate precision for derived variables will be determined based on the precision of the data on which the derivations are based, and statistics will be presented in accordance with the abovementioned rules.

  n will be an integer. If no subjects have data at a given time point, then only n=0 will be presented.

  All data will be presented to a maximum of 3 decimal places.

- Categorical variables: Descriptive statistics will include counts and percentages per category. The denominator in all percentage calculations will be the number of subjects in the relevant analysis set with non-missing data, unless specifically stated otherwise. Percentages will be displayed to one decimal place.

  Percentage change from baseline values will be calculated and displayed to 1 decimal place in the listings. In the summaries, the mean and median percentage change from baseline values will be presented to 2 decimal places, the minimum and maximum values will be presented to 1 decimal place and the SD value will be presented to 3 decimal places.

  For categories where all subjects fulfill certain criteria, the percentage value will be displayed as 100. For categories where zero subjects fulfill certain criteria, there will be no percentage displayed. All other percentage displays will use 1 decimal place. Data listings will contain all reported and derived data.

Unless otherwise stated, the following methods will be applied:

- Repeat/unscheduled assessments: Values collected at unscheduled study visits/time points will be allocated to scheduled visit/time points according to the algorithms of analysis visit window together with values collected at scheduled visits/time points will be presented in summary tables. All collected data will be included in the data listings.

- Assessment windows: All assessments will be included in the data listings and no visit windows will be applied to assessments that were performed outside of the protocol specified procedure windows.
- Result display convention: Results will be center aligned in all summary tables and listings. Subject identifiers visit and parameter labels may be left-aligned if required.
- Date and time display conventions: The following display conventions will be applied in all outputs where dates and/or times are displayed:
  - Date only: YYYY-MM-DD
  - Date and time: YYYY-MM-DD HH:MM

If only partial information is available, unknown components of the date or time will be presented as ‘NK’ (not known), i.e., ‘2016-NK-NK’. Times will be reported in military time.

3.1. Key Definitions

The following definitions will be used:

- Baseline: The baseline value is defined as the last available valid (quantifiable continuous or categorical value), non-missing observation for each subject prior to first BRII-179/placebo administration unless otherwise specified. Repeat and unscheduled assessments will be included in the derivation of the baseline values.
- Change from Baseline: The change from baseline value is defined as the difference between the result collected/derived at a post-baseline visit/time point and the baseline value.

  The change from baseline value at each post-baseline visit/time point will be calculated for all continuous parameters using the following formula:

  Change from Baseline Value = Result at Visit/Time Point – Baseline Value

  The change from baseline value will only be calculated if the specific post-baseline visit/time point result and the baseline value for the parameter are both available and will be treated as missing otherwise.
- Fold Change from Baseline: The fold change from baseline value is defined as the ratio of the result collected/derived at a post-baseline visit/time point divided by the baseline value.

  The change from baseline value at each post-baseline visit/time point will be calculated for all continuous parameters using the following formula:

  Fold Change from Baseline Value = Result at Visit/Time Point / Baseline Value

  The fold change from baseline value will only be calculated if the specific post-baseline visit/time point result and the baseline value (not 0) for the parameter are both available and will be treated as missing otherwise.

- Study day: The study day of an event is defined as the relative day of the event starting with the date of the first BRII-179/placebo administration (reference date) as Day 1 (there will be no Day 0).

  The study day of events occurring before the first study drug administration will be calculated as:

  Study Day = (Date of Event - Date of First BRII-179/placebo Administration)

  For events occurring on or after Day 1, study day will be calculated as:

  Study Day = (Date of Event - Date of First BRII-179/placebo Administration) + 1

  Study days will only be calculated for events with complete dates and will be undefined for events that are 'Ongoing' at the end of the study.

  Relative days compared to an alternative reference point will be calculated similarly, but the alternative starting reference start date will be used instead of the date of the first study drug dosing.

- Completion of Week 18 BRII-179/Placebo: Subjects whose BRII-179/Placebo last to Week 18 or later but by Week 24.
- Completion of Week 24 PEG-IFNα treatment: Subjects whose PEG-IFNα last to Week 24.
- HBsAg loss/HBsAg seroclearance: HBsAg changing from positive (>=0.05 IU/mL) at baseline to negative (<LLOQ 0.05 IU/mL) at any postbaseline visit.
- Hepatitis B surface antibody (HBsAb) response: at any post-baseline visit with either (i) post-baseline anti-HBs ≥ 2 IU/L if anti-HBs <2 IU/L at baseline or (ii) post-baseline anti-HBs ≥ 5 times the baseline anti-HBs if baseline anti-HBs was ≥ 2 IU/L.
- HBsAg seroconversion: HBsAg changing from positive (>=0.05 IU/mL) at baseline to negative (<LLOQ 0.05 IU/mL) AND HBsAb changing from negative (< 10.0 IU/L) at baseline to positive (≥ 10.0 IU/L) at any postbaseline visit.
- HBsAg Rebound: HBsAg change from negative (<LLOQ 0.05 IU/mL) to positive (>=0.05 IU/mL) at any postbaseline visit.
- HBsAg Blip: HBsAg change from negative (<LLOQ 0.05 IU/mL) to positive (>=0.05 IU/mL) at single visit (scheduled and unscheduled), then back to negative (<LLOQ 0.05 IU/mL) at next visit (scheduled and unscheduled) after NrtI discontinuation.
- Sustained HBsAg loss: quantitative HBsAg < LLOQ 0.05 IU/mL at scheduled and unscheduled visits for ≥ 24 weeks after NrtI discontinuation.
- HBV DNA Blip: HBV DNA change from < LLOQ 10 IU/mL to >= 10 IU/mL at single visit (scheduled and unscheduled), then back to < LLOQ 10 IU/mL at next visit (scheduled and unscheduled) after NrtI discontinuation.
- Persistently undetectable HBV DNA: HBV DNA < LLOQ 10 IU/mL at scheduled and unscheduled visits for ≥ 24 weeks after NrtI discontinuation.

- Functional cure: Sustained HBsAg loss and persistently undetectable HBV DNA (HBV DNA < LLOQ 10 IU/mL) maintained for 24 weeks from NDMP Day 1 to NDMP Week 24.
- Modified functional cure: Sustained HBsAg loss and persistently undetectable HBV DNA (HBV DNA < LLOQ 10 IU/mL) maintained for 24 weeks from NDMP Day 1 to NDMP Week 24 with only single visit HBsAg blip and/or HBV DNA blip permitted.
- NrtI discontinuation criteria: a) Alanine aminotransferase (ALT) < 2x ULN (results from previous visit); AND b) HBV DNA < LLOQ 10 IU/mL (results from previous visit); AND c) 2 consecutive assessments of HBsAg < LLOQ 0.05 IU/mL (assessed starting from Week 28 laboratory results).
- NrtI re-treatment: Subjects re-treated with NrtI in the judgment of investigator during NrtI discontinuation monitoring period.

3.2. Analysis Sets

Analysis sets define the subjects to be included in an analysis. Analysis sets and their definitions are provided in this section. The analysis set will be identified and included as a subtitle of each table, figure, and listing.

For each analysis set, the number and percentage of subjects eligible for inclusion.

A listing of reasons for exclusion from analysis sets will be provided by subject.

3.2.1. All Subjects Screened

All subjects, for whom a record exists on the clinical database, will be included in All Subjects Screened. It will be used for reporting study disposition, inclusion and exclusion criteria deviations, etc.

3.2.2. All Subjects Randomized

All subjects, who are randomized will be included in All Subjects Randomized. It will be used for reporting demographics, baseline characteristics, medical history, etc.

3.2.3. Full Analysis Set

All subjects, who are randomized and received at least one dose of BRII-179 or placebo, will be included in Full Analysis Set (FAS). It will be used as the primary analysis set of all efficacy endpoints.

3.2.4. Per Protocol Set

The Per Protocol Set (PPS) will include the subset of the FAS who have:

- a. HBsAg ≥ 0.05 IU/mL at baseline.

- b. Total dose number of BRII-179/placebo >=6.
- c. Have HBsAg data in the analysis visit window (Week 24, 36, 48, NDMP Week 24 [for subjects entered NDMP] and NDMP Week 48 [for subjects entered NDMP]) for related analysis such as Week 24, 36, 48, NDMP Week 24 and Final Analysis, respectively.
- d. Had not identified with important protocol deviation that significantly impacts data integrity and efficacy analysis.

Note: for subjects who did not enter NDMP, if they met criteria a, b and d, they will be included into the PPS for NDMP Week 24 analysis and Final Analysis.

The PPS will be used as analysis set for efficacy endpoint analyses. The analysis based on PPS will be considered as sensitivity analysis.

3.2.5. Safety Analysis Set

All subjects who are randomized and who take at least 1 dose of study drug will be included in Safety Analysis Set. It will be used for reporting safety parameters. The Safety Analysis Set will be based on actual treatment received if different from the treatment to which they were randomized. The actual treatment received will differ from the randomized treatment only when their actual treatment differs from randomized treatment for the entire treatment duration. All safety analyses will be based on the Safety Analysis Set.

3.2.6. Serologically Evaluable Full Analysis Set

The Serologically Evaluable FAS for HBsAg Loss will include all subjects who were randomized and received at least one dose of BRII-179 or placebo, and with HBsAg ≥ 0.05 IU/mL at baseline.

3.2.7. Biomarker Analysis Set

When applicable, selected subjects will be included in the Biomarker Analysis Set and their selected data relating to the cytokine/chemokines will be analysed.

3.3. Subject Grouping

For analyses based on the All Subjects Randomized, Full Analysis Set, Per Protocol Set and Serologically Evaluable Full Analysis Set subjects will be grouped according to the treatment groups to which they were randomized. For analyses based on Safety Analysis Set, subjects will be grouped according to the actual treatment received.

3.4. Strata and Covariates

Subjects will be randomly assigned to either BRII-179 (VBI-2601) or placebo in addition to the existing PEG-IFNα and NrtI therapy via the interactive voice or web response system (IWRS) in a 1:1 ratio using a stratified randomization schedule. Stratification will be based on the following variables:

- HBsAg level at screening (3 categories: ≥0.05 to < 1 IU/mL, ≥1 to < 10 IU/mL, and ≥10 to <100 IU/mL)

If there are discrepancies in stratification factor values between the IWRS and the clinical database, the values recorded in the clinical database will be used for analyses.

No covariate adjustments will be made.

3.5. Examination of Subject Subgroups

Subgrouping of subjects based on randomization stratification factors may be explored for subgroup analyses.

The primary efficacy endpoint will be examined using the following subgroups:

- Age: (a) 18 - 44 Years, (b) 45 - 60 Years
- Gender: (a) Male and (b) Female
- HBsAg level at screening: (a) ≥ 0.05 to < 1 IU/mL, (b) ≥ 1 to < 10 IU/mL, and (c) ≥ 10 to < 100 IU/mL

3.6. Multiple Comparisons

Not Applicable for this study Phase 2a part.

3.7. Missing Data and Outliers

3.7.1. Missing Data

In general, missing data will not be imputed unless methods for handling missing data are specified. Exceptions are presented in this document.

For missing last dosing date of study drug, imputation rules are described in Section 4.1.1.1, the handling of missing or incomplete dates for AE onset is described in Section 7.1.5.2, and for prior and concomitant medications in Section 0.

For primary and key secondary endpoints analysis, imputation rules are described in Section 6.1.2 and 6.2.1.

3.7.2. Outliers

Outliers will be identified during the data management and data analysis process, but no sensitivity analyses will be conducted. All data will be included in the data analysis.

3.8. Data Handling Conventions and Transformations

The following conventions will be used for the imputation of date of birth when it is partially missing or not collected:

- If only month and year of birth is collected, then “15” will be imputed as the day of birth
- If only year of birth is collected, then “01 July” will be imputed as the day and month of birth
- If year of birth is missing, then date of birth will not be imputed.

If age at Day 1 is not available for a subject, then age derived based on date of birth and the Day 1 visit date will be used instead. If an enrolled subject was not dosed with any study drug, the randomization date will be used instead of the Day 1 visit date. For screen failures, the date the first informed consent was signed will be used for the age derivation. Age required for longitudinal and temporal calculations and analyses (e.g age at date of AE) will be based on age derived from date of birth and the date of the measurement or event, unless otherwise specified.

Data that are continuous in nature but are less than the lower limit of quantitation (LLOQ) or above the upper LLOQ will be imputed as follows:

- A value that is 1 unit less than the LLOQ will be used to calculate descriptive statistics if the datum is reported in the form of “< x” (where x is considered the LLOQ). For example, if the values are reported as < 50 and < 5.0, values of 49 and 4.9, respectively, will be used to calculate summary statistics. An exception to this rule is any value reported as < 1 or < 0.1, etc. For values reported as < 1 or < 0.1, a value of 0.9 or 0.09, respectively, will be used to calculate summary statistics.
- A value that is 1 unit above the LLOQ will be used to calculate descriptive statistics if the datum is reported in the form of “> x” (where x is considered the LLOQ). Values with decimal points will follow the same logic as above.
- The LLOQ will be used to calculate descriptive statistics if the datum is reported in the form of “≤ x” or “≥ x” (where x is considered the LLOQ).

3.9. Analysis Visit Windows

Subject visits might not occur on protocol-specified days. Therefore, for the purpose of analysis, observations will be assigned to analysis windows. The analysis visit windows for the parameters at different periods are listed in Table 3-1 - Table 3-7.

- Table 3-1 Analysis Visit Windows for HBsAg/HBsAb Quantitation

Study Period	Analysis Visit	Nominal Study Day	Lower Limit	Upper Limit
Dosing Period	Baseline	1		1
	Week 6	43	2	64
	Week 12	85	65	95
	Week 15	106	96	116
	Week 18	127	117	148
	Week 24	169	149	183
Follow-up Period	Week 28	197	184	211
	Week 32	225	212	239
	Week 36	253	240	267
	Week 40	281	268	295
	Week 44	309	296	323
	Week 48	337	324	351
NrtI Discontinuation Monitoring Period	Day 1	1	1	1
	Week 12	85	2	127
	Week 24	169	128	211
	Week 36	253	212	295
	Week 48	337	296	351

- Table 3-2 Analysis Visit Windows for HBV DNA Quantitation

Study Period	Analysis Visit	Nominal Study Day	Lower Limit	Upper Limit
Dosing Period	Baseline	1		1
	Week 12	85	2	127
	Week 24	169	128	183
Follow-up Period	Week 28	197	184	211
	Week 32	225	212	239
	Week 36	253	240	267
	Week 40	281	268	295
	Week 44	309	296	323
	Week 48	337	324	351
NrtI Discontinuation Monitoring Period	Day 1	1	1	1
	Week 4	29	2	43
	Week 8	57	44	71
	Week 12	85	72	127
	Week 24	169	128	211
	Week 36	253	212	295
	Week 48	337	296	351

- Table 3-3 Analysis Visit Windows for Cytokine/Chemokine Biomarker Quantitation

Study Period	Analysis Visit	Nominal Study Day	Lower Limit	Upper Limit
Dosing Period	Baseline	1		1
	Week 6	43	2	64
	Week 12	85	65	106
	Week 18	127	107	148
	Week 24	169	149	183
Follow-up Period	Week 28	197	184	211
	Week 32	225	212	253
	Week 40	281	254	309
	Week 48	337	310	351
NrtI Discontinuation Monitoring Period	Day 1	1	1	1
	Week 8	57	2	113
	Week 24	169	114	253
	Week 48	337	254	351

This is not applicable to Week 24 Analysis.

- Table 3-4 Analysis Visit Windows for Safety Laboratory Assessments

Study Period	Analysis Visit	Nominal Study Day	Lower Limit	Upper Limit
Dosing Period	Baseline	1		1
	Week 3	22	2	32
	Week 6	43	33	53
	Week 9	64	54	74
	Week 12	85	75	95
	Week 15	106	96	116
	Week 18	127	117	148
	Week 24	169	149	183
Follow-up Period	Week 28	197	184	211
	Week 32	225	212	239
	Week 36	253	240	267
	Week 40	281	268	295
	Week 44	309	296	323
	Week 48	337	324	351
NrtI Discontinuation Monitoring Period	Day 1	1	1	1
	Week 4	29	2	43
	Week 8	57	44	71
	Week 12	85	72	127
	Week 24	169	128	211
	Week 36	253	212	295
	Week 48	337	296	351

- Table 3-5 Analysis Visit Windows for Vital Signs and Weight

Vital Signs

Study Period	Analysis Visit	Nominal Study Day	Lower Limit	Upper Limit
Dosing Period	Baseline	1		1
	Week 12	85	2	126
	Week 24	169	127	183

Weight

Study Period	Analysis Visit	Nominal Study Day	Lower Limit	Upper Limit
Dosing Period	Baseline	1		1
	Week 18	127	2	148
	Week 24	169	149	253
Follow-up Period	Week 48	337	254	351

- Table 3-6 Analysis Visit Windows for 12-lead ECG

Study Period	Analysis Visit	Nominal Study Day	Lower Limit	Upper Limit
Dosing Period	Baseline	1		1
	Week 24	169	2	183

- Table 3-7 Analysis Visit Window for Follow Up Week 48 for Subjects Entered NDMP

Analysis Visit	Nominal Study Day	Lower Limit	Upper Limit	Study Week When Entered NDMP (NDMP Day 1)	Study Week for Week 48 Analysis Visit Window Mapping (NDMP Week 12)
Week 48	337	323	379	Week 36	Week 48
				Week 40	Week 52
				Week 44	Week 56

3.9.1. Selection of Data in the Event of Multiple Records in an Analysis Visit Window

Depending on the statistical analysis method, single values may be required for each analysis window. For example, change from baseline by visit usually requires a single value, whereas a time-to-event analysis would not require 1 value per analysis window.

If multiple valid, nonmissing measurements exist in an analysis window, records will be chosen based on the following rules if a single value is needed:

- For baseline, the last nonmissing value on or prior to the first dosing date of study drug will be selected, unless specified differently. If there are multiple records with the same time or no time recorded on the same day, the baseline value will be the average of the measurements for continuous data, or the measurement with the lowest severity (e.g., normal will be selected over abnormal for safety electrocardiogram findings) for categorical data, and the most conservative value will be selected for efficacy data (e.g., negative will be selected over positive for HBsAg, whereas positive will be selected over negative for anti-HBs).

- For postbaseline values:

    - Categorical variables:

— The most conservative value (e.g., abnormal will be selected over normal for safety ECG) within the analysis window will be selected, except for HBV serology (HBsAg, anti-HBs), for which the most favorable value (i.e., <LLOQ will be selected over positive for HBsAg, whereas positive will be selected over negative for anti-HBs) will be selected.

    - Continuous variables:

— If there are more than 1 value within an analysis window, the value collected closet to the nominal day will be selected.

— If there are 2 records that are equidistant from the nominal day, the later record will be selected.

— If there is more than 1 record on the selected day, the latest record will be selected; if these measurements were recorded at the same time or no time recorded, the average will be taken for continuous data and the worse severity will be taken for categorical data, unless otherwise specified.

4. SUBJECT DISPOSITION

4.1. Subject Enrollment and Disposition

A summary of subject enrollment will be provided by treatment groups for each investigator and site name within a country using All Randomized Analysis Set. The summary will present the number and percentage of subjects enrolled. For each column, the denominator for the percentage calculation will be the total number of subjects analyzed for that column.

If there are discrepancies in the value used for stratification assignment between the IWRS and the clinical database, the value collected in the clinical database will be used for the summary. A listing of subjects with discrepancies in the value used for stratification assignment between the IWRS and the clinical database at the time of data finalization will be provided.

A summary of subject disposition will be provided by treatment groups. This summary will present the number of subjects in each of the categories listed below:

- All subjects screened
- Screen failure
- All subjects randomized
- All subjects treated with BRII-179/placebo
- All subjects completed Week 18 BRII-179/placebo
- All subjects completed Week 24 PEG-IFNα
- All subjects completed Week 18 BRII-179/placebo and Week 24 PEG-IFNα
- Premature discontinuation of BRII-179/placebo and reasons
- Premature discontinuation of PEG-IFNα and reasons
- Completed study
- Did not complete the study with reasons for premature discontinuation of study

For the status of study drug and study completion and reasons for premature discontinuation, the number and percentage of subjects in each category will be provided. The denominator for the percentage calculation will be the total number of subjects in the All Subjects Randomized corresponding to that column. In addition, a flowchart will be provided to depict the disposition.

The following by-subject listings will be provided by subject identification (ID) number (in ascending order), age, gender, race and screening body weight to support the above summary tables:

- Reasons for premature study drug or study discontinuation
- Reasons for screen failure (will be provided by screening subject ID number in ascending order)

4.1.1. Duration of Exposure to Study Drug

Duration of Treatment is calculated as (Date of Last Dose - Date of First Dose) + 1 for the completers and calculated as (Discontinued Date-Date of First Dose +1) for subjects with prematurely discontinuation, regardless of any temporary interruptions in study drug administration, and will be expressed in weeks using up to 1 decimal place (e.g., 4.5 weeks). If the last study drug dosing date is missing, the latest date among the clinical visit date, laboratory sample collection date, full physical examination, alcohol intake assessment, diary complete, pregnancy test date that occurred during the dosing-treatment period will be used.

Duration of Treatment and Total number of injections for BRII-179 (VBI-2601), placebo, PEG-IFNα will be summarized, respectively using descriptive statistics (n, mean, SD, Median, Q1, Q3, Min and Max) for the Safety Analysis Set.

No formal statistical testing is planned.

A by-subject listing of study drug administration and drug accountability will be provided separately by subject ID number, age gender, race, screening body weight and visit in chronological order.

4.1.2. Adherence to Study Drug

The level of prescribed adherence to the study drug regimen will be determined by the total doses of study drug taken relative to the total doses of study drug specified by the protocol.

- The level of prescribed adherence of BRII-179 / placebo and PEG-IFNα will be expressed as a percentage using the following formula:

$$\text{Prescribed Adherence (\%)} = \left(\frac{\text{Total Doses of Study Drug}}{\text{Total Doses of Study Drug Specified by Protocol}}\right) \times 100$$

Descriptive statistics for the level of prescribed adherence with the number and percentage of subjects belonging to adherence categories (< 70%, ≥ 70 to ≤ 80%, > 80%) will be provided by treatment group for the Safety Analysis Set.

The number and percentage of subjects in each dosing frequency will be provided.

No formal statistical testing is planned.

4.2. Protocol Deviations

Protocol deviations occurring after subjects entered the study are documented during routine monitoring and will be provided by a by-subject listing. Important protocol deviations are departures from the protocol that impact subject safety, efficacy assessment or data integrity. The number and percentage of subjects with important protocol deviations, with 1, 2, 3 or more important protocol deviations, and the total number of important protocol deviations and leading to exclusion from Per Protocol Set by deviation reason (e.g., nonadherence to study drug, violation of select inclusion/exclusion criteria) will be summarized by treatment groups for the All Subjects Randomized.

5. BASELINE CHARACTERISTICS

5.1. Demographics and Baseline Characteristics

Subject demographic variables (i.e., age, gender, childbearing potential for female, race, and ethnicity) and baseline characteristics will be summarized by treatment groups and overall using descriptive statistics for continuous variables and using number and percentage of subjects for categorical variables. The summary of demographic data and baseline characteristics will be provided for the All Subjects Randomized. No formal statistical testing is planned.

Baseline characteristics include:

- Body weight (kg)
- Height (cm)
- Body mass index (BMI; kg/m^2) as continuous variable
- Screening HBsAg level as categories: ≥ 0.05 to < 1 IU/mL, ≥1 to < 10 IU/mL, and ≥10 to <100 IU/mL)
- HBsAg quantitative (IU/mL) and Log10 transformed HBsAg quantitative at baseline as continuous variable
- Alanine aminotransferase (ALT) and Aspartate aminotransferase (AST) as a continuous variable and as categories based on central laboratory normal range (≤ upper limit of normal [ULN], > ULN – 2 × ULN, > 2 × ULN – 5 × ULN)
- FibroScan result (kPa) for subjects have available measurement result at screening / within 6 months prior to screening as continuous variable
- Prior PEG-IFNα type (PEG-IFNα 2a and PEG-IFNα 2b) as categories
- HBV Genotype as categories

A by-subject demographics and baseline characteristics listing, including the informed consent date, will be provided by subject ID number (in ascending order), age, gender, race and screening body weight.

5.2. Medical History

Medical history will be collected at screening.

Medical history will be coded using the Medical Dictionary for Regulatory Activities® (MedDRA) Version 26.0 or higher and will be summarized by system organ class (SOC) and preferred term (PT) for each treatment groups and overall by the number and percentage of subjects using the Safety Analysis Set.

No formal statistical testing is planned.

Listing of general medical history data will be provided.

6. EFFICACY ANALYSES

Efficacy endpoints analyses will be based on Full Analysis Set, Serologically Evaluable Full Analysis Set and Per Protocol Set.

The Week 24 Analysis will be performed when approximately 120 subjects enrolled in the Phase 2a part complete the last scheduled PEG-IFNα treatment and HBsAg results are available or withdraw from the study/discontinue study drug prior to last scheduled PEG-IFNα treatment.

6.1. Primary Efficacy Endpoint

Definition of the Primary Efficacy Endpoint:

The primary efficacy endpoint of phase 2a part is percentage of subjects with HBsAg loss (< LLOQ 0.05 IU/mL) at Week 24 of completion of PEG-IFNα treatment, it will be analysed based on Cochran-Mantel-Haenszel method, stratified at randomization by screening HBsAg level (3 categories: ⩾ 0.05 to < 1 IU/mL, ⩾1 to < 10 IU/mL, and ⩾10 to <100 IU/mL). The differences in the percentage of HBsAg loss and associated 95% confidence interval will be presented. If too few cases in a stratum result in failure of stratified analysis, it will be considered to remove the stratification factors and a non-stratified analysis or Fisher's exact probability method will be used to compare the percentage of HBsAg loss between the two groups.

Null hypothesis: there is no difference in percentage of subjects with HBsAg loss at Week 24 between treatment groups.

H_0: $P_{trt} - P_{pbo} = 0$

Alternative hypothesis: there is a difference in percentage of subjects with HBsAg loss at Week 24 between treatment groups.

H_1: $P_{trt} - P_{pbo} \neq 0$

P_{trt} : indicates the percentage of subjects with HBsAg loss at Week 24 in BRII-179 (VBI-2601).

P_{pbo} : indicates the percentage of subjects with HBsAg loss at Week 24 in placebo.

6.1.1. Estimand

The estimand for the primary objective is the difference in percentage of participants with HBsAg loss at Week 24 between BRII-179 + PEG-IFNα and Placebo + PEG-IFNα group.

Treatment policy strategies will be employed in this trial, i.e., the occurrence of the intercurrent event is considered irrelevant in defining the treatment effect of interest: the value for the variable of interest is used regardless of whether or not the intercurrent event occurs.

The estimand summary information are listed in the below table

Target Population	Analysis set (analysis population)
The target population are defined according to the inclusion/exclusion criteria in the protocol.	Full Analysis Set and Serologically Evaluable Full Analysis Set
Treatment	
BRII-179 + PEG-IFNα and<br>Placebo+ PEG-IFNα	
Endpoint	
Percentage of participants with HBsAg loss at week 24	
Handling of intercurrent events	
Treatment policy strategy and composite strategy will be used. Further details are provided after the table.	
Population-level summary measure	Analysis approach
Differences in percentage of participants with HBsAg loss at Week 24 between BRII-179 and Placebo group.	Cochran-Mantel-Haenszel method, stratified at randomization by screening HBsAg level (3 categories) will be used to compare the percentage of HBsAg loss between BRII-179 and Placebo group, Fisher exact probability method will be used instead if too few cases in a stratum resulted in failure of stratified analysis.

Intercurrent events and handling strategies:

**Intercurrent events**	**Primary treatment strategy**	**Remark for the primary strategy**
Pause treatment (intermittent treatment)	Treatment policy	Comply with clinical practice

Discontinue from treatment prematurely but remain in the trial	Treatment policy	Comply with clinical practice
Change background NrtI treatment	Treatment policy	Comply with clinical practice
Discontinue from the trial prematurely due to any reasons at IMP dosing period	Composite variable, subject will be treated as treatment failure	Subjects will be regarded as treatment failure no matter any reasons, such as drug tolerance, etc.

6.1.2. Analysis methods for the primary endpoint

Cochran-Mantel-Haenszel method will be used to compare the percentage of subjects with HBsAg loss between two treatment groups at a two-sided alpha level of 0.6 with stratification at randomization by screening HBsAg level (3 categories). Proportion point estimate and the two-sided 95% exact confidence interval will be calculated based on the weighted average of difference for each randomisation stratum using the normal approximation of binomial proportions. Fisher exact probability method will be used instead if too few cases in a stratum resulted in failure of stratified analysis.

The point estimates and the 2-sided 95% exact CIs based on the Clopper-Pearson method will also be provided for the percentages in each treatment group.

Missing data for the endpoint will be imputed as treatment failure.

The SAS programming specification is listed in appendix 1.

The estimand together with the strategies of treatment policy and composite strategy to handle intercurrent events will serve as primary endpoints analysis.

Subgroup analysis stratified by HBsAg level (3 categories) at screening will be conducted with respect to the percentage of subjects with HBsAg loss.

A Forest plot will graphically present the point estimates and the CIs on the percentage difference (BRII-179 + PEG-IFNα – Placebo + PEG-IFNα) by Age, Gender and HBsAg level at screening and overall for each treatment group.

The percentage of subjects with HBsAg loss at Week 24 between two treatment groups will be compared using Full Analysis Set, Serologically Evaluable Full Analysis Set and Per Protocol Set.

6.2. Secondary Efficacy Endpoints

- Percentage of subjects with sustained HBsAg loss and persistently undetectable HBV DNA (defined as undetectable HBsAg and HBV DNA for at least 24 weeks) after NrtI discontinuation
- HBsAg change from baseline from Day 1 to completion of PEG-IFNα treatment
- Percentage of subjects with HBsAg loss at any timepoint
- Percentage of subjects with undetectable HBV-DNA (HBV DNA < Lower Limit of Quantification (LLOQ)) 24 and 48 weeks after NrtI discontinuation
- Percentage of subjects meeting the NrtI discontinuation criteria by end of follow-up period (Week 48)
- Percentage of subjects with HBsAg seroconversion at any timepoint
- Percentage of subjects with hepatitis B surface antibody (HBsAb) response as of the end of the follow-up period (Week 48)
- Time to achieve HBsAg seroclearance and seroconversion
- Percentage of subjects requiring NrtI re-treatment during follow up

6.2.1. Analysis methods for the secondary endpoints

Percentage of subjects with HBsAg loss and in 3 categories (≥ 0.05 to < 1 IU/mL, ≥1 to < 10 IU/mL, and ≥10 to <100 IU/mL) will be presented for each treatment group by visit. Percentage of subjects with HBsAg seroconversion and percentage of subjects with hepatitis B surface antibody (HBsAb) response will be presented for each treatment group by visit.

Percentage of subjects with HBsAg seroconversion at Week 24, percentage of subjects with hepatitis B surface antibody (HBsAb) response at Week 24, percentage of subjects with sustained HBsAg loss and persistently undetectable HBV DNA after NrtI Discontinuation by NDMP Week 24 (functional cure) and percentage of subjects meeting NrtI discontinuation criteria by the end of follow-up period will be analysed with Cochran-Mantel-Haenszel method in the same way as for the primary endpoint. Missing data for these endpoints will be imputed as treatment failure. Additionally, the subject will be treated as treatment failure when this subject is re-treated with NrtI therapy before any of end date of sustained HBsAg loss or persistently undetectable HBV DNA after NrtI discontinuation per composite variable strategy.

Percentage of subjects with sustained HBsAg loss and persistently undetectable HBV DNA after NrtI Discontinuationby NDMP Week 24, 36 and 48will be summarized by treatment groups. Meanwhile, percentage of subjects with undetectable HBV-DNA (HBV DNA < LLOQ 10 IU/mL) at NDMP Week 12, 24, 36 and 48 after NrtI discontinuation will be summarized by treatment groups as well.

Percentage of subjects with HBsAg loss at any timepoint and percentage of subjects requiring NrtI re-treatment during follow up will be summarized only.

Percentage of below items and in 3 HBsAg categories at screening (≥ 0.05 to < 1 IU/mL, ≥1 to < 10 IU/mL, and ≥10 to <100 IU/mL) will also be summarized between treatment groups by visit:

- HBsAg (IU/mL): <0.05, >=0.05 - <1, >=1 - <10, >=10 - <100 and >=100;
- HBsAb (IU/L): <2, >=2 - <10, >=10 - <100 and >=100.

Time to achieve HBsAg seroclearance and seroconversion will be compared using stratified log-rank test, Kaplan-Meier estimates (product-limit estimates) will be presented by treatment groups together with the median time of achieving HBsAg seroclearance and seroconversion, respectively.

Kaplan-Meier plots will be created for treatment groups for the these endpoints. Censoring rules are listed in the below table

	Data of event/censoring	Censoring
Subjects who don't achieve the HBsAg seroclearance	Time from Day 1 to last HBsAg, Anti-HBs sample collection date, respectively.<br><br>HBsAg seroclearance:<br>Subjects with negative HBsAg at baseline or without any postbaseline HBsAg measurement will be censored at Day 1.<br><br>HBsAg seroconversion:<br>The subjects meeting i) Subjects with negative HBsAg or positive HBsAb at baseline; or ii) HBsAg or HBsAb is missing at the same postbaseline measurement date throughout the study will be censored at Day 1.	Y
Subjects who don't achieve the HBsAg seroconversion		
Subjects who achieve the HBsAg seroclearance	Time from Day 1 to date of achieving the target.	N
Subjects who achieve the HBsAg seroconversion		

Plots of the mean (SD) of absolute values and changes from baseline in log_{10} HBsAg quantitative over time by visit will be presented by screening HBsAg level (3 categories) and overall for each treatment group. Continuous endpoint log_{10} HBsAg change from baseline at Week 24 will be compared using Wilcoxon ranks sum test by adjusting HBsAg level at screening, Missing data at Week 24 will be imputed as the maximum value at the same visit in the same treatment group.

The SAS programming specification is listed in appendix 1.

By-subject listings will be provided by subject ID number, age, gender, race and screening body weight in chronological order.

6.3. Tertiary Efficacy Endpoints

Assessment of plasma protein level cytokine/chemokine biomarkers changes from baseline post dosing, i.e., from Day 1 of dosing to the end of follow-up (Week 48), and during the NrtI Discontinuation Monitoring Period.

6.3.1. Analysis methods of the tertiary efficacy endpoints

When applicable, the biomarkers such as IFN-γ, TNF-α, IL-10, IL-21, IL-12p70, CXCL10 (IP10), CXCL11 (I-TAC), CCL2 (MCP-1) CCL4 (MIP-1β) and CD40L will be summarized by treatment groups, overall and visit using Biomarker Analysis Set.

6.4. Changes From Protocol-Specified Efficacy Analyses

Estimated probability of success using Bayesian modeling is added to support decision making and optimal selection of options in recommendation letter.

Some exploratory analyses in Week 36 Analysis, Week 48 Analysis, NDMP Week 24 Analysis and Final Analysis are added to support drug development decision making.

7. SAFETY ANALYSES

7.1. Adverse Events and Deaths

7.1.1. Adverse Event Dictionary

Adverse events (AEs) will be coded using the MedDRA Version 26.0 or higher. System organ class (SOC), high-level group term (HLGT), high-level term (HLT), preferred term (PT), and lower-level term (LLT) will be provided in the AE dataset.

7.1.2. Adverse Event Severity

Adverse events are graded by the investigator as Grade 1, 2, 3, 4, or 5 according to CTCAE Version 5.0 grading scale specified in the protocol. The severity grade of events for which the investigator did not record severity will be categorized as "missing" for tabular summaries and data listings. The missing category will be listed last in summary presentation.

7.1.3. Relationship of Adverse Events to Study Drug

Related AEs are those for which the investigator selected "Related" on the AE CRF to the question of "Relationship to Brii-179/Placebo" and "Relationship to PEG-IFNα. Relatedness will always default to the investigator's choice, not that of the medical monitor. Events for which the investigator did not record relationship to study drug will be considered related to study drug for summary purposes. However, by-subject data listings will show the relationship as missing.

7.1.4. Serious Adverse Events

Serious adverse events (SAEs) will be identified and captured as SAEs if the AEs met the definitions of SAEs that were specified in the study protocol. SAEs captured and stored in the clinical database will be reconciled with the SAE database from the vender before data lock.

7.1.5. Treatment-Emergent Adverse Events

7.1.5.1. Definition of Treatment-Emergent Adverse Events

Treatment-emergent adverse events (TEAEs) are defined as 1 or both of the following:

- Any AE that has an onset date that is on or after the day of study drug administration
- Any AE that has been present before treatment and worsens relative to the pre-treatment state after administration of study drug

7.1.5.2. Incomplete Dates

If the onset date of the AE is incomplete and the AE stop date is not prior to the first dosing date of study drug, then the month and year (or year alone if month is not recorded) of onset determine whether an AE is treatment emergent. The event is considered treatment emergent if the AE onset is the same as or after the month and year (or year) of the first dosing date of study drug.

An AE with completely missing onset and stop dates, or with the onset date missing and a stop date later than the first dosing date of study drug, will be considered to be treatment emergent.

In addition, an AE with the onset date missing and incomplete stop date with the same or later month and year (or year alone if month is not recorded) as the first dosing date of study drug will be considered treatment emergent.

7.1.6. Summaries of Adverse Events and Deaths

Treatment-emergent AEs will be summarized based on the Safety Analysis Set.

A brief, high-level summary of the number of percentage of subjects who experienced at least 1 TEAE in the categories described below will be provided by treatment group. All deaths observed in the study will also be included in this summary.

The number and percentage of subjects who experienced at least 1 TEAE will be provided and summarized by SOC and PT by treatment and overall. For the AE categories described below, summaries will be provided by SOC, PT and treatment group:

- All TEAEs summarized by SOC and PT
- TEAEs with Grade 1, 2, 3, 4 and 5 in overall summary
- TEAEs with Grade 3 or higher
- All TEAEs occurring in >= 5% of subjects
- All TEAEs occurring in >= 10% of subjects
- BRII-179/Placebo related TEAEs
- PEG-IFNα related TEAEs
- BRII-179/Placebo and PEG-IFNα related TEAEs
- BRII-179/Placebo related TEAEs of Grade 3 or higher
- PEG-IFNα related TEAEs of Grade 3 or higher
- BRII-179/Placebo and PEG-IFNα related TEAEs of Grade 3 or higher

- TEAEs by maximum severity
- Serious TEAEs
- BRII-179/Placebo related serious TEAEs
- PEG-IFNα related serious TEAEs
- BRII-179/Placebo and PEG-IFNα related serious TEAEs
- TEAEs leading to premature discontinuation of BRII-179/Placebo
- TEAEs leading to premature discontinuation of PEG-IFNα
- TEAEs leading to premature discontinuation of study
- Serious TEAEs leading to death (i.e., outcome of death)
- TE (Treatment-emergent) Solicited systemic reactions
- TE Solicited local reactions (injection site reactions)

Multiple events will be counted only once per subject in each summary. Adverse events will be summarized and listed first in descending order of SOC (and HLT within each SOC if applicable) based on total frequency and then by PT in descending order of total frequency within each SOC. For summaries by severity, the most severe severity will be used for those AEs that occurred more than once in a given subject during the study.

In addition to the above summary tables, all TEAEs, serious TEAEs, treatment-related TEAEs, and treatment-related serious TEAEs will be summarized by PT only in descending order of total frequency.

The counts of TEAEs with grade >= 3 will be compared between BRII-179/placebo and PEG-IFNα under the assumption of zero-inflated Poisson distribution or zero-inflated negative binominal distribution, the final model selection will be based on the assessing results of goodness of fit (Akaike Information Criterion (AIC) value (the smaller the better) will be used to evaluate relative quality of the regression models, and p-value (>0.05)).

The characteristics about BRII-179/placebo related solicited local reaction / injection site reaction and solicited systemic reaction will be summarized (the duration, whether to lead to study drugs or study discontinuation, any concomitant medicine used, severity, serious or not, etc.).

In addition, data listings will be provided for the following:

- All AEs, indicating whether the event is treatment emergent

- All SAEs
- All Deaths
- All SAEs leading to death (i.e., outcome of death)
- All AEs leading to premature discontinuation of BRII-179/placebo
- All AEs leading to premature discontinuation of PEG-IFNα
- All AEs leading to premature discontinuation of study
- Solicited Local Reaction/Injection Site Reaction

7.2. Laboratory Evaluations

Laboratory data collected during the study will be analyzed and summarized using both quantitative and qualitative methods. Summaries of laboratory data will be provided for the Safety Analysis Set and will include data throughout the trial. If a subject has repeated laboratory values for a given time point, the value from the last evaluation will be used.

Subjects who have permanently discontinued study drug, or have available data at the time of the database snapshot for an analysis will be included in laboratory data analysis. The analysis will be based on values in standard units. When values are below the LLOQ, they will be listed as such, and the closest imputed value will be used for the purpose of calculating summary statistics. Hemolized test results will not be included in the analysis, but they will be listed in by-subject laboratory listings.

A by-subject listing for laboratory test results will be provided by subject ID number, age, gender, race, screening body weight and visit in chronological order for hematology, chemistry including liver function tests, coagulation parameters, urinalysis and thyroid panel separately. Values falling outside of the relevant reference range and/or having a severity grade of 1 or higher on the CTCAE 5.0 severity grading scale will be flagged in the data listings, as appropriate. Unscheduled laboratory test results will be also listed.

No formal statistical testing is planned.

7.2.1. Summaries of Numeric Laboratory Results

Descriptive statistics will be provided by treatment for each laboratory test specified in the study protocol as follows:

- Baseline values
- Values at each postbaseline visit

- Change from baseline at each postbaseline visit

A baseline laboratory value will be defined as the last measurement obtained on or prior to the date/time of first dose of study drug. Change from baseline to a postbaseline visit will be defined as the visit value minus the baseline value.

7.2.2. Graded Laboratory Values

The CTCAE Version 5.0 will be used to assign toxicity grades (1 to 5) to laboratory results for analysis. For laboratory tests with criteria for both increased and decreased levels, analyses for each direction (i.e., increased, decreased) will be presented separately.

7.2.2.1. Treatment-Emergent Laboratory Abnormalities

Treatment-emergent laboratory abnormalities are defined as values that increase at least 1 toxicity grade from baseline at any postbaseline visit. If the relevant baseline laboratory value is missing, any abnormality of at least Grade 1 observed within the time frame specified above will be considered treatment emergent.

7.2.2.2. Summaries of Laboratory Abnormalities

Laboratory data that are categorical will be summarized using the number and percentage of subjects in the study with the given response at baseline and each scheduled postbaseline visit.

The following summaries (number and percentage of subjects) for treatment-emergent laboratory abnormalities will be provided by lab test and treatment; subjects will be categorized according to the most severe postbaseline abnormality grade for a given lab test:

- Graded laboratory abnormalities

For all summaries of laboratory abnormalities, the denominator is the number of subjects with nonmissing postbaseline values.

7.2.3. Liver-related Laboratory Evaluations

Liver-related abnormalities after initial study drug dosing will be examined from Day 1 of dosing to the last visit and summarized using the number and percentage of subjects who were reported to have the following laboratory test values for postbaseline measurements:

- Aspartate aminotransferase (AST): (a) > 3 times of the upper limit of reference range (ULN); (b) > 5 x ULN; (c) > 10 x ULN; (d) > 20 x ULN

- Alanine aminotransferase (ALT): (a) > 3 x ULN; (b) > 5 x ULN; (c) > 10 x ULN; (d) > 20 x ULN

- AST or ALT: (a) > 3 x ULN; (b) > 5 x ULN; (c) > 10 x ULN; (d) > 20 x ULN
- Total bilirubin: > 2 x ULN
- Alkaline phosphatase (ALP) > 1.5 x ULN
- AST or ALT > 3 x ULN and total bilirubin: (a) > 1.5 x ULN; (b) > 2 x ULN

The summary will include data from all postbaseline visits. For individual laboratory tests, subjects will be counted once based on the most severe postbaseline values. For both the composite endpoint of AST or ALT and total bilirubin, subjects will be counted once when the criteria are met at the same postbaseline visit date. The denominator is the number of subjects in the Safety Analysis Set who have nonmissing postbaseline values of all relevant tests at the same postbaseline visit date. A listing of subjects who met at least 1 of the above criteria will be provided.

Events of Clinical Interest (ECI) will be summarized and listings will be provided accordingly, defined as the following:

- ALT > 3x ULN, if Day 1 pre-dose ALT ≤ ULN
- ALT > 2x baseline, if Day 1 pre-dose ALT > ULN

7.3. Body Weight, Height, BMI and Vital Signs

The actual values of vital sign parameters including temperature, pulse rate, respiratory rate, and systolic and diastolic blood pressure. Descriptive statistics will be provided by treatment group for vital signs and body weight as follows:

- Baseline value
- Values at each postbaseline visit
- Change from baseline at each postbaseline visit

A baseline value will be defined as the last available value collected on or prior to the date/time of first dose of study drug. Change from baseline to a postbaseline visit will be defined as the postbaseline value minus the baseline value. Vital signs measured at unscheduled visits will be included for the baseline value selection.

No formal statistical testing is planned.

A by-subject listing of vital signs, body weight, height and BMI will be provided by subject ID number, age, gender, race, screening body weight and visit in chronological order.

7.4. Prior and Concomitant Medications

Medications collected at screening and during the study will be coded using the World Health Organization (WHO) Drug dictionary version B3 September 1, 2023 or later (except for prior PEG-IFN-ɑ treatment, prior NrtI therapy and NrtI therapy in the study).

7.4.1. Prior Medications

Prior medications are defined as any medication before a subject took the first dose of study drug.

Prior medications will be summarized by Anatomical Therapeutic Chemical (ATC) drug class Level 2 and preferred name using the number and percentage of subjects for each treatment and overall. A subject reporting the same medication more than once will be counted only once within each ATC drug class when calculating the number and percentage of subjects who received that medication. Medications may appear under multiple ATC drug classes. The summary will be ordered descending by ATC medical class in overall frequency and then by preferred term in order of descending overall frequency within each ATC medical class. For drugs with the same frequency, sorting will be done alphabetically.

For the purposes of analysis, any medication with a start date prior to the first dosing date of study drug will be included in the prior medication summary regardless of when the stop date is. If a partial start date is entered the medication will be considered prior unless the month and year (if day is missing) or year (if day and month are missing) of the start date are after the first dosing date. Medications with a completely missing start date will be included in the prior medication summary, unless otherwise specified.

Summaries will be based on the Safety Analysis Set. No formal statistical testing is planned.

A summary of prior medications will be provided.

7.4.2. Concomitant Medications

Concomitant medications are defined as medications taken after a subject took the first study drug. Use of concomitant medications will be summarized by Anatomical Therapeutic Chemical (ATC) drug class Level 2 and preferred name using the number and percentage of subjects for each treatment. A subject reporting the same medication more than once within each ATC drug class will be counted only once when calculating the number and percentage of subjects who received that medication. Medications may appear under multiple ATC drug classes. The summary will be ordered descending by ATC medical class in overall frequency and then by preferred term in descending overall frequency within each ATC medical class. For drugs with the same frequency, sorting will be done alphabetically.

For the purposes of analysis, any medications with a start date prior to or on the first dosing date of study drug and continued to be taken after the first dosing date, or started after the first dosing

date but prior to or on the last dosing date of study drug will be considered concomitant medications. Medications started and stopped on the same day as the first dosing date or the last dosing date of study drug will also be considered concomitant. Medications with a stop date prior to the date of first dosing date of study drug or a start date after the last dosing date of study drug will be excluded from the concomitant medication summary. If a partial stop date is entered, any medication with the month and year (if day is missing) or year (if day and month are missing) prior to the date of first study drug administration will be excluded from the concomitant medication summary. If a partial start date is entered, any medication with the month and year (if day is missing) or year (if day and month are missing) after the study drug stop date will be excluded from the concomitant medication summary. Medications with completely missing start and stop dates will be included in the concomitant medication summary, unless otherwise specified. Summaries will be based on the Safety Analysis Set. No formal statistical testing is planned.

A summary of concomitant medications will be provided.

All prior and concomitant medications (other than per-protocol study drugs) will be provided in a by-subject listing sorted by subject ID number, age, gender, race, screening body weight and administration date in chronological order.

7.5. Electrocardiogram Results

Electrocardiogram (ECG) will be summarized by treatment groups and visit.

The summary of overall interpretation findings table will present counts and percentages for the reported results at baseline and each post-baseline visit. Result categories will be ordered as 'Normal', 'Abnormal - Not Clinically Significant (NCS)' and 'Abnormal - Clinically Significant (CS)'.

The worst post baseline value will be the worst non-missing value after baseline and during the reference period. Scheduled visits, unscheduled visits, and repeat measurements will be included to derive the worst post baseline values (Abnormal CS is the worst, Abnormal NCS is next, then normal).

Additionally, shift tables from baseline in categories as 'Normal', 'Abnormal - Not Clinically Significant (NCS)', 'Abnormal - Clinically Significant (CS)' and 'Missing', will be summarized by counts and percentages.

A by-subject listing for ECG assessment results will be provided by subject ID number, age, gender, race, screening body weight and visit in chronological order.

7.6. Other Safety Measures

By-subject listings of physical examination and pregnancy testing result will be provided by subject ID number, gender, age, race, screening body weight and visit in chronological order.

8. EXPLORATORY ANALYSES

8.1. Probability of Success Estimated by Bayesian Model based on HBsAg Loss at Week 24

Probability of success will be estimated using Bayesian modeling based on percentage difference of HBsAg loss at Week 24 between two treatment groups to support DSMB review of the Week 24 efficacy data and recommendation on advance to Phase 2b part or not, as well as the Brii decision making.

8.1.1. Statistical methods

8.1.1.1. Bayesian Modeling

The analysis will consider percentage difference of binary endpoint HBsAg loss at Week 24 between two treatment groups as an outcome, the level of evidence required for decision making how to advance to next stage is expressed in terms of Bayesian probability statements based on posterior probability distributions:

Probability (BRII-179 + PEG-IFNα is better than Placebo + PEG-IFNα by at least D) is greater than certain threshold X where D and X are defined as below:

D is how much higher percentage for this outcome which are predefined as 0.1, 0.15 and 0.2.

X is threshold value.

Take D is 0.2 and X is 0.6 as an example, the Probalility can be interpreted as:

The choice of 0.6 for this probability indicates that there is a 3 to 2 odds of BRII-179 + PEG-IFNα being better than Placebo + PEG-IFNα by the desired amount 20%.

Initially non-informative prior distributions for paramerters will be used. Samples from the posterior distributions for all parameters will be obtained using a Markov Chain Monte Carlo (MCMC) sampling technique with 20,000 iterations following a burn-in of size 2000.

Diagnostic tests will be performed to assess convergence and autocorrelation. Alternative programming conventions (thinning, parameterization, number of chains, seed, size of burn-in) may be explored if, using initial programming code, satisfactory mixing is not achieved with computer memory limitations.

The mean for posterior distributions will be presented.

This model will not adjust for additional covariates or variables used in the stratification of randomization as the goal is to describe the differences in outcomes for those receiving BRII-179 + PEG-IFNα versus Placebo + PEG-IFNα.

8.1.1.2. Model specification:

The percentage of subjects reaching HBsAg loss at Week 24 will be compared between treatment groups using a Bayesian logistic regression model. The model specification will be:

$$r_{ij}|p_{ij} \sim Binary(p_{ij})$$

$$logit(p_{ij}) = \beta_0 + \beta_1 x_{ij}$$

Where *i* indexes subjects in group *j* (*j*=1 denotes the placebo group and *j*=2 denotes the treatment group), x_{ij}=0 if *j*=1 and x_{ij}=1 if *j*=2, p_{ij} is the probability of HBsAg loss at Week 24, r_{ij} is 1 if the subjects reached HBsAg loss at Week 24 and zero otherwise, β_0 is the log odds of reaching HBsAg loss at Week 24 in the placebo group, and β_1 is the log odds ratio between the treatment group and placebo group.
The following independent non-informative priors will be used:

$$\beta_0, \beta_1 \sim N(0, 100^2).$$

To obtain samples from the posterior distribution for the difference in the percentage of subjects reaching HBsAg loss at Week 24 in the treatment group (p_2) and the placebo group (p_1), we will transform the samples from the posterior distributions for β_0, β_1:

$$p_1 = \frac{\exp(\beta_0)}{1 + \exp(\beta_0)}$$

$$p_2 = \frac{\exp(\beta_0 + \beta_1)}{1 + \exp(\beta_0 + \beta_1)}$$

$$p_2 - p_1 = \frac{\exp(\beta_0 + \beta_1)}{1 + \exp(\beta_0 + \beta_1)} - \frac{\exp(\beta_0)}{1 + \exp(\beta_0)}$$

Features of the posterior distribution of the difference ($p_2 - p_1$) will be estimated using descriptive statistics of the MCMC samples. Based on the estimated posterior distribution for the difference ($p_2 - p_1$), we will estimate the probability that the difference is greater than D. The probability that the difference in the percentage reaching HBsAg loss at Week 24 between treatment groups is greater than

$$P(p_2 - p_1) > D$$

will be calculated as the number of samples where the difference is greater than D divided by the total number of samples. If this probability is greater than certain threshold, this will be considered as achieving the desired level of evidence for decision making.

The SAS programming specification is listed in appendix 1.

8.2. Week 36 Analysis, Week 48 Analysis, NDMP Week 24 Analysis and Final Analysis

Missing HBsAg data at Week 36, NDMP Week 24 and NDMP Week 48 will be imputed as failure (not HBsAg loss), but for Week 48, the imputation rule will be applied as follows:

1) For subjects entered NDMP (Refer to Table 3-7 for analysis visit window):
   a. If NDMP Day 1 visit at Study Week 36, Week 40 or Week 44, the HBsAg result at analysis visit window NDMP Week 12 will be used as imputation result.
   b. If NDMP Week 12 HBsAg data is still missing, Week 48 HBsAg loss will be imputed as failure.

2) For subjects not entered NDMP:
   a. Missing data will be imputed as failure.

Missing HBsAb data at Week 36, NDMP Week 24 and NDMP Week 48 will be imputed using last observation carried forward (LOCF) method, but for Week 48, the imputation rule will be applied as follows:

1) For subjects entered NDMP (Refer to Table 3-7 for analysis visit window):
   a. If NDMP Day 1 visit at Study Week 36, Week 40 or Week 44, the HBsAb result at analysis visit window NDMP Week 12 will be used as imputation result.
   b. If NDMP Week 12 HBsAb data is still missing, Week 48 HBsAb will be imputed using last observation carried forward (LOCF) method.

2) For subjects not entered NDMP:
   a. Missing data will be imputed using last observation carried forward (LOCF) method.

Full Analysis Set and Per Protocol Set will be used for this summary.

8.2.1. Summary of HBsAg Loss, HBsAg Seroconversion and HBsAb Levels at different timepoint and any Timepoint up to different timepoint

Percentage of HBsAg loss, HBsAg Seroconversion, achieved HBsAb response and HBsAb levels at different timepoint (eg., Week 24, Week 36, Week 48, etc.), and the maximum postbaseline HBsAb levels at any timepoint up to different timepoint (eg., Week 24, Week 36, Week 48, etc.) will be summarized by treatment groups.

- HBsAb levels (IU/L): 1) <2, >=2 - <10, >=10 - <100 and >=100; 2) >=2, >=10, >=100 and >=500.

Based on NDMP entering status, spaghetti plot of subject serum HBsAb level, bar chart of HBsAb categories at different timepoint and maximum postbaseline HBsAb categories by Week 48 by treatment group will be also provided.

8.2.2. Summary of HBsAg Loss in Subjects Achieved HBsAb Response and with Different HBsAb Levels

Percentage of HBsAg loss in subjects achieved HBsAb response and with different HBsAb levels at different timepoint (eg., Week 24, Week 36, Week 48, etc.) and the maximum postbaseline HBsAb levels up to different timepoint (eg., Week 24, Week 36, Week 48, etc.) will be summarized by treatment groups.

- HBsAb levels (IU/L): 1) <2, >=2 - <10, >=10 - <100 and >=100; 2) >=2, >=10, >=100 and >=500.

8.2.3. Summary of Any HBsAg Rebound after Week 24 in Subjects Achieved HBsAg Loss at Week 24

Any HBsAg rebound after Week 24 in different periods, i.e., Week 24 - Week 48 and NrtI Discontinuation Monitoring Period will be summarized by treatment groups.

The proportion of HBsAg rebound in subjects with different HBsAb levels will be summarized by treatment groups:

- HBsAb levels at Week 24 to HBsAg rebound occurred in different period after Week 24
- The maximum postbaseline HBsAb levels up to Week 24 to HBsAg rebound occurred in different period after Week 24
- HBsAb Levels at NDMP Day 1 to HBsAg Rebound Occurred during NDMP

HBsAb levels (IU/L): 1) <2, >=2 - <10, >=10 - <100 and >=100; 2) >=2, >=10, >=100 and >=500.

8.2.4. Summary of HBsAb Levels in Subjects with Different HBsAg Loss Status at Any Postbaseline Timepoint

- The proportion of subjects with or without HBsAg loss at any postbaseline timepoint who achieved different maximum postbaseline HBsAb levels will be summarized by treatment groups.

- Maximum postbaseline HBsAb levels (IU/L): 1) <2, >=2 - <10, >=10 - <100 and >=100; 2) >=2, >=10, >=100 and >=500.

8.2.5. Summary of HBsAg and HBsAb Levels Change from Baseline at Any Postbaseline Timepoint

The mean (SD) of absolute values and changes from baseline in $\log_{10}$ HBsAg and $\log_{10}$ HBsAb, and the median (Q1, Q3) of absolute values and changes from baseline in HBsAb over time by visit will be presented by screening HBsAg level (3 categories) and overall for each treatment group.

8.2.6. The Impact of Treatment, Maximum Postbaseline HBsAb Level or HBsAb Response and HBsAg Level at Screening on HBsAg Loss

To explore the impact of factors such as treatment group, maximum postbaseline HBsAb level or HBsAb response on outcome 'HBsAg loss at certain visit', univariate and multivariate logistic regression model will be used by considering HBsAg level at screening as a covariate.

All variables will be entered and carried to the multivariate logistic regression model using a complete model. P-value < 0.05 will be considered statistically significant. The goodness of fit will be assessed for this model.

The odds ratio and 95% confidence interval will be provided.

It will be concluded that the contrast category is superior to reference category if the lower bound of the 2-sided 95% CI of the odds ratio (compared/reference) is more than 1.

The impact of HBsAb on sustained HBsAg seroclearance will be plotted as well.

8.2.7. Sensitivity Analysis of Modified Sustained HBsAg Loss and Persistently Undetectable HBV DNA after NrtI Discontinuation

In NDMP Week 24 Analysis and Final Analysis, percentage of subjects with sustained HBsAg loss and persistently undetectable HBV DNA after NrtI Discontinuation by NDMP Week 24 (modified functional cure), and modified sustained HBsAg loss and persistently undetectable HBV DNA after NrtI Discontinuation by NDMP Week 36 and Week 48 will be summarized by treatment groups.

Percentage of subjects meeting modified functional cure will be analysed with Cochran-Mantel-Haenszel method in the same way as for the primary endpoint.

9. SOFTWARE

SAS® Software Version 9.4. SAS Institute Inc., Cary, NC, USA.

10. SAP REVISION

Revision Date (DD MMM YYYY)	Section	Summary of Revision	Reason for Revision
Draft version 0.1<br>09NOV2022			The initial version.
Version 1.0<br>21JUN2023			The 1st version
Version 1.1<br>07AUG2023	2.2 Week 36 Analysis<br>2.3 Week 48 Analysis	The description about Week 36 Analysis and Week 48 Analysis were added.	1) Week 36 Analysis is required to support corporate annual data publication plan;<br>2) Week 48 Analysis is required based on the option B in recommendation letter of Week 24 DSMB meeting.
	3.1 Key Definitions	The definition about HBsAg Rebound was added.	It will be required by Week 36 Analysis.
	3.2.4 Per Protocol Set	1) Add another criteria: total dose number of BRII-179/placebo >= 6.<br>2) Remove the detailed criteria description about IPD definition for Week 24 Analysis.	1) Total dose number of BRII-179/placebo >= 6 can be regarded as one of study compliance criterias.<br>2) PPS definition can be applied to Week 36 and Final Analysis by removing this detailed description.
	3.9 Analysis Visit Window	Upper limit for Week 48 was added by nominal study day + 14.	Fourteen days was considered per protocol 8.1.7 Early Termination Visit.
	6.2.1 Analysis methods for the secondary endpoints	Add handing strategy for subjects with re-treatment after NrtI discontinuation by using composite variable strategy.	Composite variable strategy is reasonable for clinical practice regarding subjects with re-treatment after NrtI discontinuation.
	8.2 Week 36 Analysis	Add this new analysis	Week 36 analysis is to evaluate the sustained HBsAg loss and impact of anti-HBs level on HBsAg loss sustainability to support corporate annual data publication plan on the primary endpoint and 12 weeks off-treatment efficacy and safety result, and the potential health authority communication on the development plan of BRII-179.
Version 2.0<br>07AUG2023	3.1 Key Definitions	Add HBsAg blip, HBV DNA blip, functional cure and modified functional cure.	In NDMP Week 24 Analysis, the percentage of functional cure and modified functional cure will be calculated.

	8.2 Week 36 Analysis, Week 48 Analysis, NDMP Week 24 Analysis and Final Analysis	Add new imputation rule for analysis visit window Week 48 for HBsAg and HBsAb by considering sujects entered NDMP.	Due to lack of analysis visit window Week 48 for subjects entered NDMP at Week 36, 40 and 44, the corresponding study week 48 will be mapped from NDMP related visit windows.
	8.2.5. Summary of HBsAg and HBsAb Levels Change from Baseline at Any Postbaseline Timepoint	Add the summary about the change from baseline in log10 HBsAg, log10 HBsAb and HBsAb over time by visit for each treatment group.	As external experts suggested that the average of 12 and 24 weeks HBsAb level after EOT should be provided to observe the difference of HBsAb level between EOT and 12 and 24 weeks post EOT.
	8.2.7. Sensitivity Analysis of Modified Sustained HBsAg Loss and Persistently Undetectable HBV DNA after NrtI Discontinuation	Add the summary about the percentage of modified sustained HBsAg loss and persistently undetectable HBV DNA after NrtI discontinuation at each NDMP visit. Meanwhile, the comparison of modified functional cure between two treatment groups was added as well.	As a sensitivity analysis, the HBsAg and/or HBV DNA blip will be permitted in this summary.

11. APPENDICES

Appendix 1. Programming Specifications

1) Treatment difference in percentages adjusted by stratification factor at screening:

```
ods output
commonrelrisks = _commonrelrisks
crosstabfreqs = _crosstabfreqs
cmh = _cmh
commonpdiff = _commonpdiff
;
proc freq;
tables stratafactor * trt * response/cmh commonriskdiff; * p-value from general association;
run;
```

The percentage difference and the corresponding 95% CIs will be calculated using the stratum-adjusted Mantel-Haenszel (MH) method {Koch 1989}, stratified by HBsAg status at screening, as follows:

$P_1 - P_2 \pm Z_{(1-\alpha/2)} \times SE(P_1 - P_2)$,

where

$(P_1 - P_2) = \frac{\sum w_h d_h}{\sum w_h}$, is the stratum-adjusted MH percentage difference, where

$d_h = P_{1h} - P_{2h}$ is the percentage difference of Groups 1 and 2 in stratum h (h = 1 and 2).

$w_h = \frac{n_{1h} n_{2h}}{n_{1h}+n_{2h}}$, is the weight based on the harmonic mean of sample sizes for stratum h, where n_{1h} and n_{2h} are the sample sizes of Groups 1 and 2 in stratum h.

$SE(P_1 - P_2) = \frac{\sqrt{\sum w_h^2 [\frac{p^*_{1h}(1-p^*_{1h})}{n_{1h}-1} + \frac{p^*_{2h}(1-p^*_{2h})}{n_{2h}-1}]}}{(\sum w_h)^2}$, where $p^*_{1h} = \frac{m_{1h}+0.5}{n_{1h}+1}$ and $p^*_{2h} = \frac{m_{2h}+0.5}{n_{2h}+1}$, where m_{1h} and m_{2h} are the numbers of subjects with HBsAg loss at Week 24 in Groups 1 and 2 in stratum h.

$\alpha = 0.05$ for this study.

$Z_{(1-\alpha/2)} = Z_{0.975} = 1.96$ is the 97.5^{th} percentile of the normal distribution.

If the computed lower confidence bound is less than -1, the lower bound is defined as -1. If the computed upper confidence bound is greater than 1, then the upper bound is defined as 1.

Reference:

Koch GG, Carr GJ, Amara IA, Stokes ME, Uryniak TJ. Categorical Data Analysis. Chapter 13 in Berry, D.A. (ed.). Statistical Methodology in the Pharmaceutical Sciences. New York: Marcel Dekker, Inc., 1989:pp. 414-21.

2) Treatment difference in percentages not adjusted by stratification factor at screening:

```
ods output
crosstabfreqs = _crosstabfreqs
chisq= _chisq
fishersexact= _fishersexact
pdiffcls = _pdiffcls
;
proc freq;
tables trt * response/riskdiff(cl = (exact)) ;
exact fisher riskdiff(method = score) ;
run;
```

The 95% CI for percentage estimate for each treatment is calculated based on the Clopper-Pearson exact method.

```
proc freq;
by trt;
tables event/ binomial;
exact binomial;
run;
```

3) Wilcoxon Rank sum test adjusted by stratification factor at screening:

```
ods output
crosstabfreqs = _crosstabfreqs
cmh = _cmh
;
proc freq;
table stratafactor * trt * response/cmh2 scores = modridit;
run;
```

4) Log-rank test for time to event between treatment groups by stratification factor at screening:

```
ods output
productlimitestimates = _productlimitestimates
homtests = _homtests
quartiles = _quartiles
means = _means
```

```
homstats = _homstats
;
proc lifetest;
strata stratafactor;
time days*censor(1);
test trt;
run;
```

The binary indicator variable (CENSOR) with a value of 0 indicates the time to the event of interest is complete or 1 indicates the time to the event is censored. DAYS is a time to event variable.

5) Zero-inflated Poisson or zero-inflated negative binomial model for comparison of Grade >= 3 TEAEs counts between two treatment groups:

```
%* Section: ZIP model. ;
ods output
modelfit=fit_zip
lsmeans = lsmns_zip
parameterestimates = paramesti_zip
diffs = dif_zip
;
proc genmod data = ae_all_final;
  class trt01an ;
  model cnt_event_grd3 = trt01an /dist=zip link=log offset=log_follow_up_dur;
  zeromodel /link = logit;
  lsmeans trt01an / /*bylevel e*/ diff pdiff cl exp /*or ilink*/ ;
   output out=zip predicted=pred pzero=pzero;
run;
ods output close ;

%* Section: ZINB Model ;

ods output
modelfit=fit_zinb
lsmeans = lsmns_zinb
parameterestimates = paramesti_zinb
diffs = dif_zinb
;
proc genmod data = ae_all_final;
  class trt01an ;
  model cnt_event_grd3 = trt01an /dist=zinb link=log offset=log_follow_up_dur;
  zeromodel /link = logit;
```

```
 lsmeans trt01an / /*bylevel e*/ diff pdiff cl exp /*or ilink*/ ;
  output out=zinb predicted=pred pzero=pzero;
run;
ods output close ;
```

6) Bayesian model:

```
ods graphics on;
proc mcmc data=trials dic mchistory=brief nbi=2000 nmc=20000 stats=all plots(smooth)
monitor=(prob1 prob2 prob3)
    outpost =post stats(alpha=(0.05) percentage=(2.5 25 50 75 97.5)) seed=20220802 ;
    ods output postsummaries = postsummaries_res ;
    ods output postintervals = postintervals_res ;

    parms (beta0 beta1) 0 ;
    prior beta: ~normal(0, var=10000);

    mu = beta0 + beta1*trtpn ;
    p = logistic(mu);

    model resp ~ binary(p);

        begincnst;
                px1 = 0.10;
                px2 = 0.15;
                px3 = 0.20;
        endcnst;

        beginprior;
            pc = logistic(beta0);
            pt = logistic(beta0 + beta1);
            p_dif = pt - pc ;

                prob1 = (p_dif > px1);
                prob2 = (p_dif > px2);
                prob3 = (p_dif > px3);
        endprior;
run;
ods graphics off;
```

BRII-179-002_SAP_for_Phase_2a_Analysis_Final Version_2.0_20231130

Final Audit Report 2023-11-30

Created:	2023-11-30
By:	Chunming Li (chunming.li@briibio.com)
Status:	Signed
Transaction ID:	CBJCHBCAABAAbml4BY28UBewOZfrkDmCyR0LNU-cnAOa

"BRII-179-002_SAP_for_Phase_2a_Analysis_Final Version_2.0_20231130" History

Document created by Chunming Li (chunming.li@briibio.com)
2023-11-30 - 9:00:33 AM GMT

Chunming Li (chunming.li@briibio.com) authenticated with Adobe Acrobat Sign.
Challenge: The user opened the agreement.
2023-11-30 - 9:02:46 AM GMT

Chunming Li (chunming.li@briibio.com) authenticated with Adobe Acrobat Sign.
Challenge: The user clicked on the signature field: 'Signature 1'.
2023-11-30 - 9:03:28 AM GMT

Document e-signed by Chunming Li (chunming.li@briibio.com)
Signing reason: I am the author/submitter of this document.
Signature Date: 2023-11-30 - 9:03:42 AM GMT - Time Source: server

Document emailed to Weihong Liu (weihong.liu@briibio.com) for signature
2023-11-30 - 9:03:44 AM GMT

Email viewed by Weihong Liu (weihong.liu@briibio.com)
2023-11-30 - 9:04:23 AM GMT

Weihong Liu (weihong.liu@briibio.com) authenticated with Adobe Acrobat Sign.
Challenge: The user opened the agreement.
2023-11-30 - 9:05:17 AM GMT

Weihong Liu (weihong.liu@briibio.com) authenticated with Adobe Acrobat Sign.
Challenge: The user clicked on the signature field: 'Signature 2'.
2023-11-30 - 9:08:50 AM GMT

Document e-signed by Weihong Liu (weihong.liu@briibio.com)

Signing reason: I reviewed / approved this document.

Signature Date: 2023-11-30 - 9:09:03 AM GMT - Time Source: server

Document emailed to David Margolis (david.margolis@briibio.com) for signature

2023-11-30 - 9:09:04 AM GMT

Email viewed by David Margolis (david.margolis@briibio.com)

2023-11-30 - 1:18:04 PM GMT

David Margolis (david.margolis@briibio.com) authenticated with Adobe Acrobat Sign.

Challenge: The user opened the agreement.

2023-11-30 - 1:19:45 PM GMT

David Margolis (david.margolis@briibio.com) authenticated with Adobe Acrobat Sign.

Challenge: The user clicked on the signature field: 'Signature 3'.

2023-11-30 - 1:20:50 PM GMT

Document e-signed by David Margolis (david.margolis@briibio.com)

Signing reason: I reviewed / approved this document.

Signature Date: 2023-11-30 - 1:21:06 PM GMT - Time Source: server

Agreement completed.

2023-11-30 - 1:21:06 PM GMT
