# Supplementary material for: Profile and Predictors of Hepatitis B Surface Antigen Seroreversion Following PEG-IFNα with or Without Therapeutic Vaccine BRII-179 or siRNA Elebsiran: A Post Hoc Pooled Analysis of Two Randomized Controlled Trials in Patients with Chronic Hepatitis B Virus Infection
Source: Vaccines (Basel). 2026 Oct 1;14(10):868. doi: 10.3390/vaccines14100868 (PMC13709818; doi:10.3390/vaccines14100868)
Supplement: Supplementary file 1 [file vaccines-14-00868-s001.zip › File_S3_ENSURE_Clinical_Study_Protocol_and_Amendments.pdf]

TITLE

Protocol Title: A Phase 2 Multicenter, Randomized, Open-label Study to Investigate the Efficacy and Safety of BRII-835 (VIR-2218) and Pegylated Interferon Alpha (PEG-IFNα) Combination Therapy for the Treatment of Chronic Hepatitis B Virus (HBV) Infection

Protocol Number: BRII-835-002

Compound: BRII-835 (VIR-2218)

Study Phase: 2

Short Title: Study to Investigate the Efficacy and Safety of BRII-835 and PEG-IFNα Combination Therapy Treating Chronic HBV Infection

Sponsor Name: Brii Biosciences Limited

Co-Sponsor/Registration Agent: Brii Biosciences (Beijing) Co. Limited

Legal Registered Address: Room 301, 3/F, Building 7, No. 1 Yongtaizhuang North Road, Tiandilinfeng (Dongsheng Area), Haidian District, Beijing 100192, China

Protocol Version: 1.0

Version Date: 31 March 2023

Information contained in this document is proprietary to Brii Biosciences Limited. The information is provided to you in confidence which is requested under an agreed upon and signed Confidentiality and Disclosure Agreement. Do not give this document or any copy of it or reveal any proprietary information contained in it to any third party (other than those in your organization who are assisting you in this work and are bound by the Confidentiality and Disclosure Agreement) without the prior written permission of an authorized representative of Brii Biosciences Limited.

SPONSOR SIGNATURE PAGE

Sponsor's Authorized Officer:

[redacted]

INVESTIGATOR SIGNATURE PAGE

For protocol number BRII-835-002

I confirm agreement to conduct the study in compliance with the protocol, *International Conference on Harmonisation Good Clinical Practices (ICH GCP)* as well as applicable local regulatory and ethical requirements.

I acknowledge that I am responsible for overall study conduct. I agree to personally conduct or supervise the described clinical study.

I agree to ensure that all associates, colleagues and employees assisting in the conduct of the study are informed about their obligations. Mechanisms are in place to ensure that site staff receive the appropriate information throughout study.

Investigator Name (Printed): ____

______________________________	______________________
**Investigator Signature**	**Date (dd/mm/yyyy)**

TABLE OF CONTENTS

TITLE....... 1
SPONSOR SIGNATURE PAGE 2
INVESTIGATOR SIGNATURE PAGE 3
TABLE OF CONTENTS 4
LIST OF TABLES 8
LIST OF FIGURES 8
ABBREVIATIONS 9
1. PROTOCOL SUMMARY 12
1.1. Synopsis 12
1.2. Schema 17
1.3. Schedule of Assessment (SoA) 18
1.3.1. Dosing Period and Follow-up Period 18
1.3.2. NRTI Discontinuation Monitoring Period 22
2. INTRODUCTION 24
2.1. Background 24
2.2. BRII-835 24
2.2.1. Summary of Nonclinical Experience 25
2.2.2. Summary of Clinical Experience 25
2.3. PEG-IFNα 27
2.4. Rationale for BRII-835 and PEG-IFNα Combination Therapy for the Treatment of Chronic HBV Infection 27
2.5. Benefit/Risk Assessment 28
3. STUDY OBJECTIVES AND ENDPOINTS 29
3.1. Study Objectives 29
3.1.1. Primary Objectives 29
3.1.2. Secondary Objectives 29
[redacted] 29
3.2. Study Endpoints 29
3.2.1. Primary Endpoints 29

3.2.2. Secondary Endpoints 29
[redacted] [redacted] 30
4. STUDY DESIGN 31
4.1. Overall Design 31
4.1.1. Planned Cohorts and Dosing 31
4.1.2. NRTI Discontinuation 32
4.1.3. Safety Review Committee 33
4.2. Scientific Rationale for Study Design 33
4.3. Justification of Dose 34
4.4. End of Study Definition 36
5. STUDY POPULATION 37
5.1. Inclusion Criteria 37
5.2. Exclusion Criteria 38
5.3. Screening Failures 40
5.4. Lifestyle Considerations 40
6. TREATMENT OF PARTICIPANTS 41
6.1. Study Drugs 41
6.1.1. General Information of Study Drugs 41
6.1.2. Study Drug Dose and Administration 41
6.1.3. Study Drug Materials and Management 42
6.2. Randomization and Blinding 44
6.3. Dose Adjustment Criteria 44
6.3.1. Safety Criteria for PEG-IFNα Dose Adjustment 44
6.4. Background Treatment 45
6.5. Concomitant Medications 46
6.5.1. Prohibited Medications 46
6.6. Treatment Compliance 46
6.6.1. PEG-IFNα Treatment Compliance 46
6.6.2. NRTI Treatment Compliance 47
7. DISCONTINUATION OF STUDY INTERVENTION AND PARTICIPANT DISCONTINUATION/WITHDRAWAL 48
7.1. Discontinuation of Study Intervention 48

7.2. Participant Discontinuation/Withdrawal from the Study 49
7.3. Lost to Follow up 49
8. STUDY ASSESSMENT AND PROCEDURES 51
8.1. Schedule of Assessments 51
8.1.1. Screening 51
8.1.2. Randomization (Day 1) 51
8.1.3. Dosing Period 52
8.1.4. End of Treatment Visit 52
8.1.5. Follow-up Period 52
8.1.6. NRTI Discontinuation Monitoring Period 53
8.1.7. Unscheduled Visits 53
8.1.8. End of Study Visit 53
8.2. Efficacy Assessments 53
8.2.1. Antiviral Activities 53
8.3. Safety Assessments 53
8.3.1. Physical Examination 53
8.3.2. Vital Signs 54
8.3.3. Alcohol Intake Assessment 54
8.3.4. Liver Stiffness 54
8.3.5. Ultrasound 54
8.3.6. Laboratory Assessments 54
8.3.7. Laboratory Events of Clinical Interest 55
8.4. Adverse Events and Serious Adverse Events 55
8.4.1. Definition of Adverse Events 56
8.4.2. Assessment of Adverse Events 57
8.4.3. Time period and frequency for collecting AEs and SAEs 58
8.4.4. Recording of AEs and SAEs 59
8.4.5. Follow-up of AEs and SAEs 59
8.4.6. Reporting of SAEs 60
8.4.7. Regulatory Reporting Requirements for SAEs 60
8.4.8. Special Situation Reports 60
8.5. Pharmacokinetic (PK) Assessments 62

8.6. Assessment of Anti-Drug Antibody (ADA) to BRII-835 62
[redacted] [redacted] 62
9. STATISTICS 64
9.1. Statistical Hypotheses 64
9.2. Estimands 64
9.3. Sample size determination 65
9.4. Analysis Sets 67
9.5. Statistical Methods 68
9.6. Statistical Analyses 68
9.6.1. Efficacy Analysis 68
9.6.2. Safety Analysis 71
9.6.3. Pharmacokinetic Analysis 72
9.6.4. Baseline and Other Summaries and Analyses 72
9.7. Interim Analysis 72
9.8. Final Analysis 73
10. REFERENCES 74
11. APPENDICES 78
11.1. Appendix 1: Regulatory, Ethical, and Study Oversight Considerations 78
11.1.1. Regulatory and Ethical Considerations 78
11.1.2. Finance Disclosure 78
11.1.3. Informed Consent Process 79
11.1.4. Data Protection 79
11.1.5. Data Quality Assurance 79
11.1.6. Source Documents 80
11.1.7. Study and Site Start and Closure 81
11.1.8. Publication Policy 81
11.2. Appendix 2: Clinical Safety Laboratory Tests 82
11.3. Appendix 3 Assessments in Participants Meeting Laboratory ECI Criteria 84
11.4. Appendix 4: Contraceptive Requirements and Collection and Reporting of Pregnancy Information 86

LIST OF TABLES

Table 1 Study Drug Dosage and Administration 41
Table 2 PEG-IFNα Dose Modification Guidelines 44
Table 3 CTCAE (V5.0) General Guideline of Severity of AEs 58
Table 4 Pharmacokinetics Assessment Timepoints 62
Table 5 95% Credible Interval of Response Rate by Sample Size 65
Table 6 Operational Characteristics by Sample Size 66
Table 7 Analysis Sets 67
Table 8 Protocol-Required Safety Laboratory Assessments 82
Table 9 Assessments in Participants Meeting Laboratory ECI Criteria 84
Table 10 Optional Assessments in Participants Meeting Laboratory ECI Criteria 84

LIST OF FIGURES

Figure 1: Study Design 17

ABBREVIATIONS

ADA	Anti-drug antibody
AE	Adverse event
AFP	Alpha-fetoprotein
ALP	Alkaline phosphatase
ALT	Alanine aminotransferase
ANA	Anti-nuclear antibodies
ANC	Absolute neutrophil count
BMI	Body Mass Index
cccDNA	Covalently closed circular DNA
CHB	Chronic hepatitis B
CIOMS	Council for International Organizations of Medical Sciences
CMO	Contracted Manufacturing Organization
CRF	Case report form
DNA	Deoxyribonucleic acid
ECG	Electrocardiogram
ECI	Event of clinical interest
eGFR	Estimated glomerular filtration rate
EPO	Erythropoietin
FAS	Full Analysis Set
GalNAc	N-acetyl galactosamine
GCP	Good Clinical Practice
G-CSF	Granulocyte colony-stimulating factor
GGT	Gamma glutamyl transferase
GM-CSF	Granulocyte macrophage colony stimulating factor
GMP	Good Manufacturing Practice
HBeAg	Hepatitis B e-antigen
HBsAg	Hepatitis B surface antigen
HBV	Hepatitis B virus
HCV	Hepatitis C virus
HDV	Hepatitis D virus
HIV	Human immunodeficiency virus

ICF	Informed consent form
ICH	International Conference on Harmonization
ICMJE	International Committee of Medical Journal Editors
IEC	Independent Ethics Committee
IFN-α	Interferon-alpha
IMP	Investigational Medication Products
INR	International normalized ratio
IRB	Institutional Review Board
IRT	Interactive Response Technology
ISR	Injection site reaction
LLOQ	Lower limit of quantification
MedDRA	Medical Dictionary for Regulatory Activities
mITT	Modified Intent-To-Treat
NCI-CTCAE	National Cancer Institute Common Terminology Criteria for Adverse Events
NRTI	Nucleos(t)ide reverse transcriptase inhibitor
OTC	Over-the-counter
PBMC	Peripheral blood mononuclear cell
PD	Pharmacodynamic
PEG-IFNα	Pegylated interferon alpha
PK	Pharmacokinetic
PP	Per-Protocol
PT	Preferred term
RBC	Red blood cells
RNAi	RNA interference
SAE	Serious adverse event
SAP	Statistical analysis plan
SC	Subcutaneous
SD	Standard deviation
siRNA	Small interfering RNA
SoA	Schedule of assessments
SOC	System organ category
SOP	Standard operating procedure

SRC	Safety Review Committee
SUSAR	Suspected unexpected serious adverse reaction
TSH	Thyroid stimulating hormone
TEAE	Treatment emergent adverse event
ULN	Upper limit of normal
WBC	White blood cells
WOCBP	Women of child-bearing potential

1. PROTOCOL SUMMARY

1.1. Synopsis

**Name of Sponsor/Company:** Brii Biosciences Limited		
**Name of Investigational Products:** BRII-835 (VIR-2218), Pegylated Interferon Alpha (PEG-IFNα)		
**Protocol Number:** BRII-835-002	**Phase:** 2	**Country:** Asia and Pacific (APAC) region
**Title of Study:** A Phase 2 Multicenter, Randomized, Open-label Study to Investigate the Efficacy and Safety of BRII-835 (VIR-2218) and Pegylated Interferon Alpha (PEG-IFNα) Combination Therapy for the Treatment of Chronic Hepatitis B Virus (HBV) Infection		
**Study Investigational Site(s):** Multiple sites in APAC region		
**Study Objectives:**<br>**Primary:**<br>• To evaluate the efficacy and safety of BRII-835 and PEG-IFNα combination therapy in participants with chronic HBV infection<br>**Secondary:**<br>• To assess the antiviral activity of BRII-835 and PEG-IFNα combination therapy<br>• To assess BRII-835 pharmacokinetic (PK) exposure and evaluate the anti-drug antibody (ADA) to BRII-835<br>[redacted]<br>**Study Endpoints:**<br>The primary endpoints for this study are to evaluate follows:<br>• Proportion of participants with HBV surface antigen (HBsAg) loss at end of treatment<br>• Proportion of participants with HBsAg loss at 24 weeks post end of treatment<br>• Proportion of participants with treatment-emergent adverse events (TEAEs)<br>• Proportion of participants with serious adverse events (SAEs)<br>The secondary endpoints for this study are to evaluate follows:<br>• Proportion of participants with HBsAg loss at Week 48<br>• Proportion of participants achieving functional cure (defined as sustained undetectable HBsAg and sustained suppression of HBV DNA [<lower limit of quantification (LLOQ)] ≥ 24 weeks after discontinuation of all treatment, including NRTI)<br>• Proportion of participants achieving HBsAg loss and HBV DNA < LLOQ at 24 weeks after discontinuation of all treatment, including NRTI		

- Proportion of participants meeting NRTI discontinuation criteria at 24 weeks post end of treatment
- Absolute serum HBsAg and change from baseline over time through 24 weeks post end of treatment
- Nadir serum HBsAg and maximum reduction of serum HBsAg from baseline throughout 48-week treatment
- Time to achieve first serum HBsAg loss through Week 72
- Proportion of participants with Hepatitis B e antigen (HBeAg) loss and/or HBeAg seroconversion (baseline HBeAg-positive participants) at any timepoint
- Proportion of participants with HBsAg seroconversion at any timepoint
- Plasma concentrations of BRII-835 (VIR-2218) and its metabolite at selected timepoints
- The occurrence of ADA to BRII-835 and associated titer, if positive

[redacted]

Study Design:

This study is designed to evaluate the efficacy and safety of BRII-835 and PEG-IFNα combination therapy in participants with chronic HBV infection without cirrhosis receiving NRTI therapy as background therapy.

A total of up to 75 participants with HBeAg negative or HBeAg positive chronic HBV infection will be randomized at 1:1:1 to the following three cohorts:

- Cohort 1: participants will receive 48 doses of PEG-IFNα, each administered every week starting on Day 1.
- Cohort 2: participants will receive 48 doses of PEG-IFNα, each administered every week starting on Day 1 and 13 doses of 200 mg BRII-835, each administered every 4 weeks starting on Day 1.
- Cohort 3: participants will receive 48 doses of PEG-IFNα, each administered every week starting on Day 1 and 13 doses of 100 mg BRII-835, each administered every 4 weeks starting on Day 1.

Participants who meet all of the following NRTI discontinuation criteria for at least two consecutive visits at Week 72 will be eligible to discontinue from NRTI therapy:

- HBsAg < LLOQ and
- HBV DNA < LLOQ and
- Undetectable HBeAg and
- ALT ≤ 2x ULN

Investigator should confirm participants' eligibility as early as possible within 2 weeks post Week 72 visit. Participants who discontinue NRTI will enter the NRTI Discontinuation Monitoring Period of the study within 1 week of the investigator confirming eligibility. Participants will be followed in the NRTI Discontinuation Monitoring Period for an additional 24 weeks.

Participants who do not meet the criteria for NRTI discontinuation will continue NRTI therapy and will end the study at Week 72.

An SRC for this study will monitor participant safety and consider study-specific data as well as relevant background information about the study drugs and target population under study to inform on study conduct. The SRC charter outlining the member composition and operating procedures will be developed before the study starts.

Number of Participants: Up to 75 participants are planned to be enrolled in the study.

Randomization and Stratification:

Eligible participants will be randomized 1:1:1 to one of the three cohorts and stratified by HBsAg level at screening (> 100 to ≤ 500 IU/mL, > 500 to ≤ 1,500 IU/mL, and > 1,500 to ≤ 3,000 IU/ml). Maximum number of participants allowed in each stratum of each cohort will be capped as six in the Interactive Response Technology (IRT) initial system set up for the first enrolled 45 participants and the limitation may be adjusted based on enrollment status.

Main Criteria for Inclusion:

Participants aged 18 to 60 years with chronic HBV infection without cirrhosis, effectively treated with NRTI therapy for at least 6 months, with HBV DNA < LLOQ and HBsAg > 100 IU/mL and ≤3,000 IU/ml at screening.

Investigational Product, Dosage and Mode of Administration:

BRII-835 will be supplied as a sterile solution for subcutaneous (SC) injection at a free acid concentration of 200 mg/mL.

PEG-IFNα will be supplied as a sterile, preservative-free solution for SC injection.

Cohort 1: PEG-IFNα 180 mcg administered by SC injection every week

Cohort 2: PEG-IFNα 180 mcg administered by SC injection every week and 200 mg BRII-835 administered by SC injection every four weeks

Cohort 3: PEG-IFNα 180 mcg administered by SC injection every week and 100 mg BRII-835 administered by SC injection every four weeks

Duration of the Study:

Estimated total duration for each participant will include the following: screening up to 4 weeks, dosing period 48 weeks, and a 24-week follow-up period after the last dose of the study drug. Eligible participants that meet NRTI discontinuation criteria (Section 4.1.2.1) may enter a NRTI discontinuation monitoring period for an additional 24 weeks from the day of NRTI discontinuation.

Sample Size Determination:

Up to 75 participants will be randomly assigned in 1:1:1 ratio to each of the three cohorts. A Bayesian framework was used to calculate the sample size. It is assumed that the number of responders follows a Binomial distribution, with a weakly informative prior (Beta (0.5, 0.5)) for the true response rate.

Primary Efficacy Analysis:

The primary endpoints are 1) the Proportion of participants with HBsAg loss at end of treatment and 2) Proportion of participants with HBsAg loss at 24 weeks post end of treatment.

A Bayesian hierarchical model (Yuen, 2022; Jones, 2011) as primary analysis will be used to estimate the posterior probability of response rate for each cohort by incorporating the baseline HBsAg level stratification factor. The point estimate of the primary outcome and 95% equal-tailed credible interval will be drawn from the model.

In addition, combine some two adjacent baseline stratification levels to reduce HBsAg stratification factors from three levels to two levels (HBsAg >100 IU/mL and HBsAg ≤1,500 IU/mL versus HBsAg >1,500 IU/mL and HBsAg ≤3,000IU/mL; HBsAg >100 IU/mL and HBsAg ≤500 IU/mL versus HBsAg >500 IU/mL and HBsAg ≤3,000 IU/mL) to perform the stratified Bayesian hierarchical analysis if model is converged using the similar approach as the primary analysis.

The primary efficacy analysis will be performed within Modified Intent-To-Treat (mITT) analysis set and a supplemental analysis based on Per-Protocol (PP) analysis set with a similar analysis will be presented.

Safety Analysis:

The Safety Population is defined as all participants who receive injection of any study medication. Participants will be classified and summarized by treatment received. Safety will be assessed using AEs, SAE, vital signs, clinical laboratory tests with clinician assessment, and concomitant medication data. The incidence of TEAEs will be summarized by Medical Dictionary for Regulatory Activities (MedDRA) System Organ Class (SOC) and preferred term (PT), summaries will be provided by severity and by causality (related, not related) to study drug.

Pharmacokinetic Analysis:

The Pharmacokinetic (PK) population is defined as all participants who received BRII-835, have at least one evaluable plasma BRII-835 concentration, and have no protocol deviation thought to impact on the PK data analysis. Plasma concentrations of BRII-835 and its metabolite will be listed for each participant and descriptively summarized, including n, mean, standard deviation (SD), median, minimum, maximum, geometric mean, and geometric coefficient of variation (CV), by treatment cohort and timepoints using PK population.

The Occurrence of ADA to BRII-835 and Associated Titer, if Positive.

Descriptive statistics for the endpoint will be performed based on ADA analysis set.

Interim Analysis and Summaries

Three interim analyses are planned. The first two interim analyses will be conducted when approximately 45 enrolled participants complete the 48-week treatment and last available participant of all enrolled participants completes the 48-week treatment, respectively. If the pace of enrollment is robust, and the primary endpoint proportion of participants with HBsAg loss at Week 48 is expected to occur less or equal to two months between the two interim analyses, the two analyses may be collapsed into one analysis.

The third interim analysis will be conducted when last available participant completes follow-up 24 weeks post end of treatment.

**Final Analysis**
The final analysis will be performed when last available participant completes the last scheduled visit according to the SoA.<br>Further details will be provided in the Statistical Analysis Plan.

1.2. Schema

Figure 1: Study Design

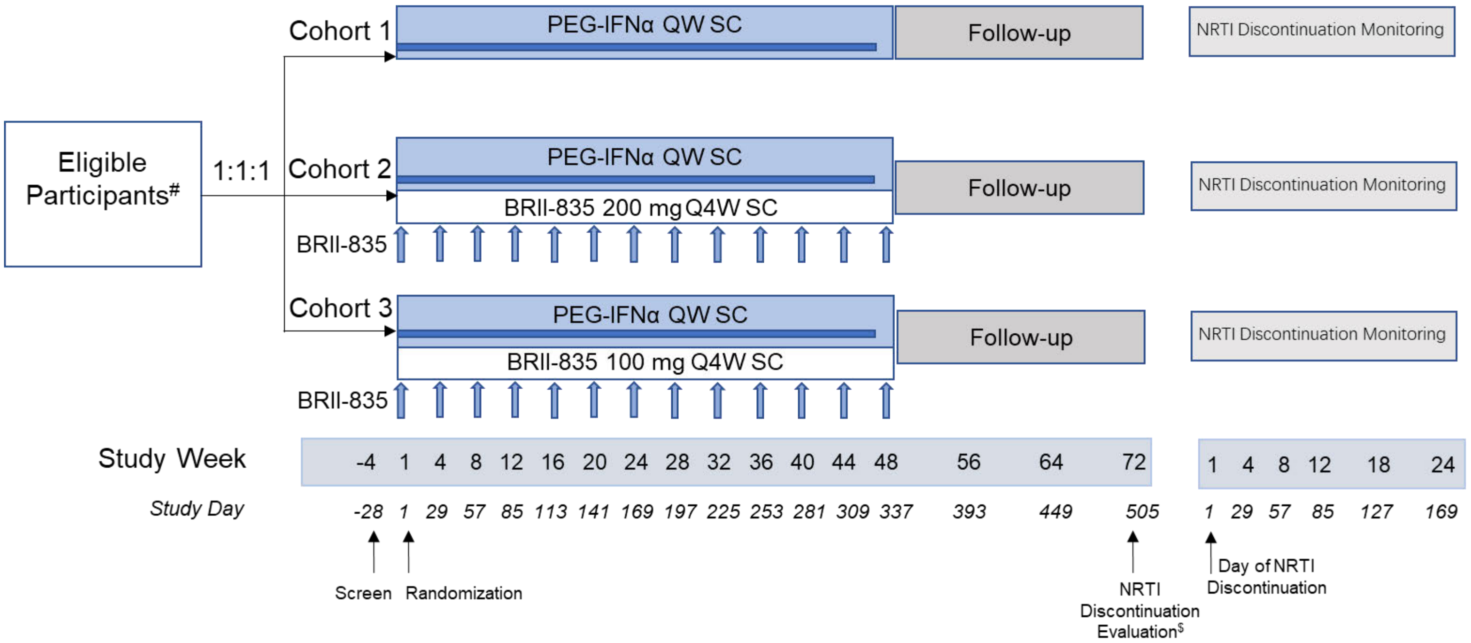

SC: subcutaneous injection

Participants will be stratified by HBsAg levels at screening.
$ Participants meeting the NRTI discontinuation criteria may enter into the NRTI Discontinuation Monitoring Period for an additional 24 weeks. Participants that do not meet the NRTI discontinuation criteria will continue NRTI therapy and end from the study

1.3. Schedule of Assessment (SoA)

1.3.1. Dosing Period and Follow-up Period

**Study Stage**	**Screen**	**Dosing Period [a, b]**														**Follow-up Period**		
**Study Visit Week**		Rand	W2	W4	W8	W12	W16	W20	W24	W28	W32	W36	W40	W44	W48 /EOT	W56	W64	W72/ EOS[c]
**Study Visit Day ± Visit Window**	D -28 to -1	D1	D15 ± 3	D29 ± 3	D57 ± 3	D85 ± 3	D113 ± 3	D141 ± 3	D169 ± 3	D197 ± 3	D225 ± 3	D253 ± 3	D281 ± 3	D309 ± 3	D337 ± 3	D393 ± 5	D449 ± 5	D505 ± 7
Informed consent form (ICF)	X																	
Inclusion/exclusion criteria	X	X																
Demography	X																	
Medical history including HBV genotype [d]	X	X																
Full physical exam [e]	X					X			X						X			X
Symptom-directed physical exam		X	X	X	X		X	X		X	X	X	X	X		X	X	
Abdominal Ultrasound	X								X						X			X
FibroScan	X								X						X			X
Alcohol intake assessment	X	X	X	X	X	X	X	X	X	X	X	X	X	X	X	X	X	X
Vital signs [f]	X	X													X			X
12-lead ECG [g]	X																	
Urinalysis	X	X				X			X			X			X			X

Screening serology panel [h]	X																	
Pregnancy test [i]	X	X	X	X	X	X	X	X	X	X	X	X	X	X	X			X
AFP	X								X						X			X
TSH, T3, T4, ANA	X					X			X			X			X			
Hematology, Chemistry, and Coagulation assessments [j]	X	X	X	X	X	X	X	X	X	X	X	X	X	X	X	X	X	X
Randomization[k]		X																
BRII-835 administration [l]		X		X	X	X	X	X	X	X	X	X	X	X	X			
PEG-IFNα administration [m]		←- X -→																
Blood sample for BRII-835 PK analysis [n]		X									X				X			
Blood sample for ADA to BRII-835		X									X				X			
Blood sample for quantitative HBsAg	X	X		X	X	X	X	X	X	X	X	X	X	X	X	X	X	X
Blood sample for quantitative anti-HBs	X	X					X		X		X		X		X	X	X	X
Blood sample for quantitative HBV DNA [o]	X	X				X			X			X			X	X		X
Blood sample for qualitative HBeAg and anti-HBe		X[u]					X[u]				X[u]				X[u]			X[u]
[redacted]		[redacted]		[redacted]					[redacted]	[redacted]					[redacted]			[redacted]
[redacted]		[redacted]				[redacted]			[redacted]						[redacted]			[redacted]

[redacted]		[redacted]			
AE assessment [r]	←------------------------------------ X ------------------------------------→				
Concomitant medications review	←------------------------------------ X ------------------------------------→				
PEG-IFNα medication adherence review [s]		←----------------------------- X -----------------------------→			
NRTI medication adherence review [s]	←------------------------------------ X ------------------------------------→				
ECI surveillance [t]		←------------------------------------ X ------------------------------------→			

AE = adverse event; AFP = alpha- fetoprotein; ANA = anti-nuclear antibodies; anti-HBs = hepatitis B surface antibody; ECG = electrocardiogram; ECI = events of clinical interest; EOT=end of treatment; EOS= end of study; HBeAg = hepatitis B e-antigen; anti-HBe = hepatitis B e-antibody; HBsAg = hepatitis B surface antigen; HBV = Hepatitis B Virus; NRTI = nucleos(t)ide reverse transcriptase inhibitor; ULN = upper limit of normal

[a] Assessments will be performed pre-dose unless otherwise specified.

[b] If a participant permanently discontinues treatment prior to Week 48, EOT assessments should be performed within 14 days post the last dose of study drugs (see Section 7.1).

[c] End of Study (EOS) visit must be conducted within 14 days when a participant prematurely discontinues from the study, unless the participant withdraws consent for study participation or is lost to follow-up.

[d] A medical history for at least 24 months prior to screening will be taken, including HBV genotype if available.

[e] Height and weight will be recorded at screening. Body mass index (BMI) will be calculated based on the measurements. Only weight will be collected afterwards.

[f] Vital signs (blood pressure, pulse rate, respiratory rate, and temperature) should be measured after the participant has rested comfortably for approximately 10 minutes.

[g] 12-lead ECG should be measured in the supine position after the participant has rested comfortably for approximately10 minutes.

[h] Screening serology panel includes anti-HCV, anti-HDV, and anti-HIV.

[i] Women of childbearing potential are required to have pregnancy tests. Negative pregnancy test must be confirmed prior to study drug administration. Blood pregnancy test should be conducted at central lab at screening visit, urine pregnancy test and blood pregnancy test (if necessary) at other time points will be conducted at local laboratory.

[j] Additional safety laboratory assessments may be performed at any time during the study as determined necessary by the investigator (not limited to safety assessments listed in protocol, local laboratory assessment is acceptable).

[k] Eligible participants will be randomized 1:1:1 to Cohort 1, 2, or 3 within 48 hours prior to study drug administration on Day 1.

[l] BRII-835 will be given to those who randomized to Cohorts 2/3, at the 2 dose levels. Please refer to Section 6.1.3.4.

[m] PEG-IFNα will be given to all participants at 180 mcg every week for 48 doses. The participants will be instructed to self-inject PEG-IFNα on the same day of each week and dose modification may be considered given certain medical condition. Please refer to Section 6.1.3.4.

[n] BRII-835 PK sample collection is referred to Section 8.5. Predose sampling should be conducted ≤ 2 hours prior to dosing. Postdose sampling should be conducted ≥ 2 hours and ≤ 12 hours after dosing.

[o] If a participant experiences alanine aminotransferase (ALT) > 3x ULN or HBV DNA ≥ 200 IU/mL, additional HBV DNA quantitation sample(s) will be collected at the next scheduled visit.

[redacted]

[q] [redacted]

[r] AEs will be collected from signing the ICF until last visit. After informed consent has been obtained but prior to initiation of study drug, untoward medical occurrence, if related to study procedure, will be recorded as AE; otherwise, will be recorded as medical history.

[s] Participants will be instructed to bring NRTI and PEG-IFNα (including all empty containers) to the trial site at every visit, as applicable, beginning with the screening visit. Adherence will be assessed by counting of empty containers and unused medication. Please refer to Section 6.6 for details.

[t] Participants will be monitored for clinical laboratory events of clinical interest (ECI) and additional assessments may be performed if indicated. ECI for this study include ALT > 5x ULN (Section 8.3.7).

[u] Samples for qualitative HBeAg assessment will be collected in participants with positive HBeAg at Day 1. Additional samples for qualitative HBeAg may be collected when the participant experiences HBV DNA ≥ 200 IU/mL. Samples for anti-HBe will be collected in all participants at all required timepoints.

[redacted]

1.3.2. NRTI Discontinuation Monitoring Period

Study Stage		NRTI Discontinuation Monitoring Period [b]				
**Study Visit Week**		W4	W8	W12	W18	W24/ EOS[c]
**Study Visit Day ± Visit Window**	D1[a]	D29 ± 3	D57 ± 3	D85± 3	D127± 3	D169 ± 7
Full physical exam						X
Symptom-directed physical exam	X	X	X	X	X	
Abdominal ultrasound						X
Fibroscan						X
Alcohol intake assessment	X	X	X	X	X	X
Vital signs[d]						X
Urinalysis						X
AFP						X
Hematology, Chemistry, and Coagulation assessments[e]	X	X	X	X	X	X
Blood sample for quantitative HBsAg	X	X	X	X	X	X
Blood sample for quantitative anti-HBs				X		X
Blood sample for quantitative HBV DNA[f]	X	X	X	X	X	X
[redacted]						[redacted]
[redacted]						[redacted]
HBV sequencing	←------ X [j] ------→					

Concomitant medications	←------------------------------X------------------------------→
Adverse event assessment	←------------------------------X------------------------------→
ECI surveillance [i]	←------------------------------X------------------------------→

AFP = alpha- fetoprotein; ECI = events of clinical interest; EOS= end of study; HBeAg = hepatitis e-antigen; HBsAg = hepatitis B surface antigen; HBV = Hepatitis B Virus; NRTI = nucleoside/nucleotide; AFP = alpha-fetoprotein; anti-HBe = hepatitis B e-antibody; anti-HBs = hepatitis B surface antibody.

[a] Participants that meet the NRTI discontinuation criteria will enter the NRTI discontinuation monitoring period within 1 week of the investigator confirming eligibility. All timepoints are relative to the day of NRTI discontinuation (D1) which is the first day the NRTI withdrawal eligible participant discontinuing their NRTI therapy.

[b] All participants who discontinue NRTI therapy will be followed through Week 24, even if NRTI retreatment occurs. Investigator should monitor participants for NRTI retreatment criteria. Participant experiencing HBV DNA increase ≥ 2 log_{10} IU/mL within a 2-week period should return to the site for unscheduled visits to measure HBV DNA levels and liver function tests as soon as possible and continue returning to site weekly until HBV DNA levels stabilize (details refer to Section 4.1.2.2).

[c] If a participant withdraws prematurely from the study prior to the W24 visit, EOS assessments should be performed.

[d] Vital signs (blood pressure, pulse rate, respiratory rate, and temperature) should be measured after the participant has rested comfortably for approximately 10 minutes.

[e] Additional safety laboratory assessments may be performed at any time during the study as determined necessary by the investigator (not limited to safety assessments listed in protocol, local laboratory assessment is acceptable).

[f] If a participant experiences HBV DNA > 200 IU/mL, additional HBV DNA sample(s) should be collected within 2 weeks.

[redacted]

[i] Participants will be monitored for clinical laboratory events of clinical interest (ECI) and additional assessments may be performed if indicated. ECI for this study include ALT > 5x ULN (See Section 8.3.7).

[j] Blood samples may be collected for HBV sequencing as determined necessary by the investigator.

2. INTRODUCTION

2.1. Background

Chronic hepatitis B virus (HBV) infection remains an important global public health problem, especially in China, that leads to serious sequelae over time, including cirrhosis, liver failure, and hepatocellular carcinoma (Trepo, 2014). An estimated 296 million people are living with chronic HBV infection worldwide, and around 820,000 people die annually due to associated sequelae (Stanaway, 2016; WHO, 2021). In China, the disease burden remains large with more than 80 million chronic HBV infections and a death rate of 11.4 death per 100,000, which is among the highest rate of the world (Polaris Observatory Collaborators, 2020; GBD 2019 Hepatitis B Collaborators, 2022).

Current treatment options for chronic HBV infection are limited. One option is long-term nucleos(t)ide reverse transcriptase inhibitor (NRTI) therapy, for example tenofovir (tenofovir disoproxil fumarate or tenofovir alafenamide) or entecavir (Lok, 2016). NRTI therapy suppresses HBV replication and viremia but does not eliminate the covalently closed circular DNA (cccDNA) or integrated DNA. Therefore, transcription and translation of viral proteins, in particular the production of HBsAg, typically continue at high levels as measured in blood (Scaglione, 2012). An alternative treatment option is a finite course of Pegylated Interferon α (PEG-IFNα) therapy which can induce a functional cure, but only in a small percentage of patients (< 10%) (Konerman, 2016). The failure of NRTI therapy to produce a functional cure and the limitations of low response rate of PEG-IFNα therapy highlight the clinical need for effective, well-tolerated HBV therapies of finite duration.

Several investigational agents are currently under development for the treatment of chronic HBV infection. Early-phase clinical studies have shown that neither direct antiviral agents nor immune modulators are likely to achieve a significant high functional cure rate when used alone. The growing consensus in the field is that combination therapies with multiple mechanism of actions involving inhibition of viral replication, reduction of viral antigen burden and stimulation of anti-viral immune response will likely be needed for success (Zoulim, 2015; Revill, 2019; Fanning, 2019; Lim, 2023). Persistent high antigen load is thought to be a major factor driving the exhaustion of antiviral adaptive immunity. New antiviral agents that can substantially reduce HBV antigen production could be rationally combined with direct immunotherapies in the hope of achieving a synergistic effect.

2.2. BRII-835

BRII-835 is a novel synthetic ribonucleic acid interference (RNAi) therapeutic that is being developed for the treatment of chronic HBV infection by Vir Biotechnology, Inc (Vir) in partnership with Alnylam Pharmaceuticals (Alnylam) globally and in partnership with Brii Biosciences for development in Greater China. BRII-835 is designed to target HBV transcripts containing a region in the HBx gene that is common to all HBV transcripts and highly conserved among HBV genotypes. It is conjugated to an *N*-acetyl galactosamine (GalNAc) ligand to facilitate the delivery to hepatocytes via the asialoglycoprotein receptor (ASGPR).

The use of siRNA offers a novel strategy for the treatment of chronic HBV infection. The siRNA antisense strand binds to the complementary target mRNA; the duplexes load to the RNA-induced silencing complex that exploit the endogenous RNA-interference pathway to enable sequence-specific RNA cleavage and degradation. siRNA can have multiple antiviral effects, including degradation of the pgRNA, thus inhibiting viral replication, and degradation of all viral messenger RNA (mRNA) transcripts. It is postulated that high levels of circulating HBV surface antigens have negative impact on immune profile and may tolerize or deplete HBV specific T cells in chronic hepatitis B (CHB) patients (Kondo, 2013; Lebosse, 2017; Ferrari, 2015; Wang, 2013). This was supported by the previous findings that reductions of HBsAg by therapeutic interventions such as siRNA or anti-HBs antibody enabled HBV vaccines to break immune tolerance and achieve functional cure in the recombinant AAV/HBV infection murine models (Zhu, 2016; Micheler, 2020). These studies provide scientific basis to treat chronic HBV patients for functional cure with a combination of immunomodulators such as PEG-IFNα, and an agent such as BRII-835 (VIR-2218), an siRNA targeting HBV transcript to lower viral antigens including HBsAg.

BRII-835 has demonstrated potent, pan-genotypic, dose-dependent pharmacologic activity in all in vitro and in vivo models tested, resulting in significant and sustained HBsAg reduction. The available clinical data showed that BRII-835 was generally well-tolerated with good safety profile and induced significant reduction of HBsAg in CHB patients (Lim, 2023). Therefore, BRII-835 may act as a critical component in combination therapies to reduce viral antigen burden and facilitate the recovery of anti-viral immunity which can be further boosted by immunotherapy.

2.2.1. Summary of Nonclinical Experience

The pharmacology, pharmacokinetics and toxicology of BRII-835 has been evaluated *in vitro* and in multiple species. The summary of nonclinical pharmacology, pharmacokinetic (toxicokinetic) and toxicology data supports the safe use of BRII-835 for the treatment of patients chronically infected with HBV. Refer to the Investigator's Brochure for BRII-835 (VIR-2218) nonclinical information.

2.2.2. Summary of Clinical Experience

BRII-835 has been and is being evaluated in several clinical trials in healthy adult participants and CHB patients, which include one completed study VIR-2218-1005 in China, and 4 ongoing studies: VIR-2218-1001 (VIR-2218 alone or in combination with PEG-IFNα), VIR-2218-1006 (VIR-2218, VIR-3434, and/or PEG-IFNα), VIR-2218-1007 (also known as BRII-179-835-001) and VIR-2218-1008 sponsored by Gilead Sciences, Inc. (also known as GS-US-465-4439).

VIR-2218-1001 is an ongoing Phase 1/2 study evaluating single and multiple ascending doses of BRII-835 as a single agent (Parts A to C) or single agent/coadministered with PEG-IFNα (Part D/F). Part A is single ascending dose phase in healthy adult participants to evaluate the safety/tolerability and the PK of BRII-835. Part B and C are multiple ascending dose phase in noncirrhotic adult participants with chronic HBV infection on NRTI therapy to evaluate the safety, tolerability, and PK of multiple doses of BRII-835. Part D-F are Phase 2 open-label study to evaluate the safety and tolerability of BRII-835 alone or in combination with PEG-IFNα

(sequential or concurrent initiation) in noncirrhotic adult participants with chronic HBV infection on NRTI therapy.

The safety data in Part A-C showed that BRII-835 alone was general well-tolerated with good safety profile in healthy adults and CHB patients. The majority of AEs were Grade 1 or 2 in severity and considered not related to study drug. No clinically significant laboratory abnormalities were observed. The available safety data in Part D/F showed that a higher proportion of participants who received BRII-835+PEG-IFNα had AEs compared with single-agent BRII-835 (85.5% vs 60.0%). The majority of AEs were Grade 1 or 2 in severity and were considered not related to BRII-835; a higher proportion of AEs were considered related to PEG-IFNα compared to those related to BRII-835 and were consistent with the known adverse reactions of PEG-IFNα.

The antiviral activity data in Parts B/C showed that greater reductions in mean serum $\log_{10}$ HBsAg were observed in participants administered BRII-835 compared with placebo and the reductions in serum $\log_{10}$ HBsAg were dose-dependent, where higher BRII-835 doses were associated with greater reductions. Half of participants administered BRII-835 achieved an absolute HBsAg level < 100 IU/mL by Week 16. The available antiviral data in Parts D-F showed that greater reductions in mean serum $\log_{10}$ HBsAg were observed at nadir in participants who had concurrent treatment of BRII-835+PEG-IFNα for ≥ 24 weeks compared with BRII-835 alone, concurrent initiation of BRII-835+PEG-IFNα for 12 weeks, or 12 weeks of BRII-835+PEG-IFNα after 12 weeks of BRII-835 lead-in. More than two-thirds of participants (67%) achieved an absolute HBsAg level < 100 IU/mL at Week 12 and most (89%) achieved an absolute HBsAg level < 100 IU/mL at Week 24. It is noticeable that concurrent initiation of BRII-835 and PEG-IFNα combination achieved the greatest reductions in HBsAg through Week 48; and longer duration of concurrent BRII-835 with PEG-IFNα resulted in lower HBsAg levels, and 30.8% (4/13) achieved HBsAg loss with anti-HBs seroconversion (with anti-HBs >10 mIU/mL) at Week 48 (Yuen, 2022).

The available PK data in Part A − C showed that BRII-835 was rapidly absorbed after SC injection and has a short plasma $t_{1/2}$ of 2 to 5 hours. The PK of BRII-835 in participants with chronic HBV infection was similar to that in healthy participants. Following two doses of BRII-835 administered 4 weeks apart in participants with chronic HBV infection, no accumulation of BRII-835 in plasma was evident. Overall, PK and PK/PD relationships in participants with chronic HBV infection support the further development of BRII-835 for the treatment of HBV.

Study VIR-2218-1005 is a Phase 2 double-blind, randomized, placebo-controlled study consisting of 2 parts which were conducted in China. The study includes adult participants with HBeAg-negative (Part One) or HBeAg-positive (Part Two) chronic HBV infection without cirrhosis who are on NRTI therapy. Study drug (BRII-835 [VIR-2218] or placebo) is administered via SC injection. A total of 16 participants received BRII-835 across Part One and Part Two of this completed study. BRII-835 was generally well tolerated as 2 monthly doses of up to 100 mg in participants with noncirrhotic chronic HBV infection. Greater reductions in mean serum HBsAg were observed for participants administered BRII-835 compared with placebo among both HBeAg-negative and HBeAg-positive participants.

Study VIR-2218-1007 (Study BRII-179-835-001) is an ongoing Phase 2 open-label, randomized parallel-group study in adult participants with chronic HBV infection without cirrhosis who are on NRTI therapy. BRII-835 is administered alone or coadministered with therapeutic vaccine BRII-179 or BRII-179 with IFNα as co-adjuvant. Preliminary safety and efficacy data through Week 40 showed that among 50 participants randomized and dosed, a total of 46 participants have completed all assigned treatments. Either alone or in combination with therapeutic vaccine BRII-179 was generally well tolerated with similar HBsAg reductions from baseline across all dose regimens (Yuen, 2023). Mean changes in $\log_{10}$ HBsAg from baseline at Week 40 were -1.68 [SD: 0.37] IU/mL for BRII-835 alone regimen, and -1.75 [SD: 0.60] and -1.77 [SD: 0.72] IU/mL for BRII-179-containing regimens, respectively. More than 40% of participants receiving combination regimens had anti-HBs antibodies > 100 IU/L. This study is still ongoing.

2.3. PEG-IFNα

Interferon α (IFNα) is the only licensed drug for hepatitis B with immunomodulatory as well as viral inhibitory properties (PEGASYS, 2017; Rijckborst, 2010). It acts by inducing interferon stimulated genes (ISGs) at various points of the HBV lifecycle through different pathways that cause an increase in breakdown in viral RNAs and protection against viral injury (Ivashkiv, 2014). IFNα also stimulates cell-mediated immune responses which target infected hepatocytes leading to a decrease in cells that harbor the intrahepatic HBV cccDNA molecules responsible for persistence of HBV infection. Additionally it may reshape the immune landscape to induce adaptive immunity by coordinating various immune cells, including NK cells, macrophages, DCs and T cells (Gill, 2016; Micco, 2013).

Pegylation of IFNα (PEG-IFNα) allowing weekly injection is currently recommended as the first-line therapy for compensated CHB patients and the recommended dose and duration of PEG-IFNα therapy for both HBeAg-positive and HBeAg negative CHB is 180 mcg per week for 48 weeks. PEG-IFNα is associated with several side-effects including influenza-like symptoms, neutropenia, thrombocytopenia, depression, and less commonly exacerbation or unmasking of autoimmune illnesses as well as hepatitis flares, however, these flares have also been associated with HBeAg and HBsAg clearance. The main advantages of PEG-IFNα in CHB treatment are finite treatment duration, the absence of resistance, and the potential for immune-mediated control of HBV infection with an opportunity to obtain a sustained virological response off-treatment, especially a chance of HBsAg seroclearance in a subset of CHB patients (Asian-Pacific clinical practice guidelines on the management of hepatitis B, 2016). However, functional cure after PEG-IFNα monotherapy is only confined in a small percentage of CHB patients (< 10%) (Konerman, 2016).

2.4. Rationale for BRII-835 and PEG-IFNα Combination Therapy for the Treatment of Chronic HBV Infection

HBV proteins including HBsAg, HBeAg, HBx and polymerase have been demonstrated to block multiple interferon response pathways and inhibit interferon-stimulated gene (ISG) induction in vitro, hence suppressing the direct anti-viral effects of PEG-IFNα (Luangsay, 2015; Xu, 2016; Tsunematsu, 2017; Liu, 2015; Wang, 2010). In addition, the anti-HBV immunity stimulated by PEG-IFNα may be decoyed and exhausted by excessive amount of HBsAg-containing subviral

particles, impairing the effects of PEG-IFNα (Tout, 2020). Therefore, lowering tolerogens may be important for enhancing the immune response induced by PEG-IFNα therapy in CHB patients, increasing the rate of HBsAg seroclearance.

BRII-835, siRNA inhibiting HBV transcription can directly reduce HBV antigen burden and may restore immune function that in turn, enhances the immune modulation effect induced by PEG-IFNα therapy, together increasing the rate of the HBsAg seroclearance. The recent study evaluating the combination of BRII-835 (VIR-2218) and PEG-IFNα in CHB patients under NRTI background therapy demonstrated a proof of principle that concurrent initiation of BRII-835 and PEG-IFNα therapy (48 weeks) resulted in enhanced responses in its HBsAg reductions, and notable rates of HBsAg seroclearance with anti-HBs seroconversion by the end of treatment (30.8% ,4/13) (Yuen, 2022). These preliminary data strongly suggest that the siRNA can unlock the potential of immunomodulator PEG-IFNα, together in combination may increase the rate of functional cure for chronic HBV infection.

2.5. Benefit/Risk Assessment

This study will provide information on the safety, efficacy, and dose regimens of BRII-835 and PEG-IFNα combination therapy, which has the potential to functionally cure chronic HBV infection.

The available data showed that BRII-835 with or without PEG-IFNα was generally well-tolerated in participants with chronic HBV infection. Additionally, reductions in HBsAg have been observed across participants treated with BRII-835. Longer duration of combination treatment of siRNA and PEG-IFNα showed a trend of improved functional cure rate compared to historical PEG-IFNα monotherapy data, suggesting a potential additive effect in increasing HBsAg loss in selected CHB population.

The potential risks with PEG-IFNα and BRII-835 have been considered in the inclusion and exclusion criteria. Only participants who meet all of the inclusion criteria and none of the exclusion criteria (as specified in the protocol) will be allowed to participate in this study. Safety will be closely monitored by the investigator throughout the study. Several safety measures have been proposed to minimize potential risks to participants, including PEG-IFNα dose modification, treatment discontinuation and withdrawal criteria, etc. The potential risks with BRII-835 include injection site reactions (ISRs), immunogenicity and liver function test abnormalities. No ISRs or liver transaminase elevation SAEs have been reported and all nonserious ISRs to date have resolved without sequelae. Specific indicators of abnormal liver function will continue to be included as part of stopping rules in clinical studies of BRII-835, and the sponsor will continue to exclude participants who may be intolerant to SC injections or who are at high risk of hepatotoxicity. In addition, an SRC will be established to monitor safety data.

Taken together, the safety and antiviral activity data support a favorable benefit-risk profile for the evaluation of 100 mg or 200 mg BRII-835 and PEG-IFNα combination therapy for the treatment of chronic HBV infection.

3. STUDY OBJECTIVES AND ENDPOINTS

3.1. Study Objectives

3.1.1. Primary Objectives

- To evaluate the efficacy and safety of BRII-835 and PEG-IFNα combination therapy in participants with chronic HBV infection

3.1.2. Secondary Objectives

- To assess the antiviral activity of BRII-835 and PEG-IFNα combination therapy
- To assess BRII-835 PK exposure and evaluate anti-drug antibody (ADA) to BRII-835

3.1.3. [redacted]

[redacted]

3.2. Study Endpoints

3.2.1. Primary Endpoints

The primary endpoints for this study are to evaluate follows:

- Proportion of participants with HBsAg loss at end of treatment
- Proportion of participants with HBsAg loss at 24 weeks post end of treatment
- Proportion of participants with treatment-emergent adverse events (TEAEs)
- Proportion of participants with serious adverse events (SAEs)

3.2.2. Secondary Endpoints

The secondary endpoints for this study are to evaluate follows:

- Proportion of participants with HBsAg loss at Week 48
- Proportion of participants achieving functional cure (defined as sustained undetectable HBsAg and sustained suppression of HBV DNA [<LLOQ] ≥ 24 weeks after discontinuation of all treatment, including NRTI)
- Proportion of participants achieving HBsAg loss and HBV DNA<LLOQ at 24 weeks after discontinuation of all treatments, including NRTI
- Proportion of participants meeting NRTI discontinuation criteria at 24 weeks post end of treatment

- Absolute serum HBsAg and change from baseline over time through 24 weeks post end of treatment
- Nadir serum HBsAg and maximum reduction of serum HBsAg from baseline throughout 48-week treatment
- Time to achieve first serum HBsAg loss through Week 72
- Proportion of participants with Hepatitis B e antigen (HBeAg) loss and HBeAg seroconversion (baseline HBeAg-positive participants) at any timepoint
- Proportion of participants with HBsAg seroconversion at any timepoint
- Plasma concentrations of BRII-835 and its metabolite at selected timepoints
- The occurrence of ADA to BRII-835 and associated titer, if positive.

3.2.3. [redacted]

[redacted]

- [redacted]
- [redacted]
- [redacted]

4. STUDY DESIGN

4.1. Overall Design

This is a Phase 2 open-label, randomized study of BRII-835 and PEG-IFNα combination therapy in adult participants with chronic HBV infection without cirrhosis receiving NRTI therapy as background therapy. This study is designed to evaluate the efficacy and safety of BRII-835 and PEG-IFNα combination therapy. Two different dose levels of BRII-835 in combination with PEG-IFNα will be evaluated.

Participants with HBeAg negative or HBeAg positive chronic HBV infection are eligible to participate in this study. Eligible participants will be stratified by their serum HBsAg level at screening and randomized 1:1:1 to Cohort 1, 2, or 3. Up to 75 participants are planned. The total duration of study participation for each participant will include screening period (up to 4 weeks), dosing period (48 weeks), follow-up period (24 weeks), and NRTI discontinuation monitoring period (24 weeks when applicable).

All participants will be assessed following the SoA (Section 1.3). The study design schema can be found in Section 1.2.

4.1.1. Planned Cohorts and Dosing

Eligible participants will be stratified by their serum HBsAg level at screening (>100 IU/mL and ≤ 500 IU/mL, > 500 and ≤ 1,500 IU/mL, or > 1,500 and ≤ 3,000 IU/mL) and randomized to receive the study intervention per the following dose regimens. Participants enrolled will remain outpatient during study participation.

- Cohort 1: Each participant will receive 48 doses of PEG-IFNα 180 mcg administered SC every week starting on Day 1
- Cohort 2: Each participant will receive 48 doses of PEG-IFNα 180 mcg administered SC every week and 13 doses of BRII-835 200 mg administered SC every 4 weeks, both starting on Day 1
- Cohort 3: Each participant will receive 48 doses of PEG-IFNα 180 mcg administered SC every week and 13 doses of BRII-835 100 mg administered SC every 4 weeks, both starting on Day 1

All participants will be followed for the safety, tolerability, and antiviral activity after completion of study intervention. The assessments and procedures performed at each visit are further described in the SoA (Section 1.3).

Participants will be assessed by the investigator for their eligibility to discontinue NRTI therapy at Week 72. Participants who meet the NRTI discontinuation criteria may enter the NRTI discontinuation monitoring period for an additional 24 weeks. Participants who do not meet the NRTI discontinuation criteria will continue NRTI therapy and will end from the study.

4.1.2. NRTI Discontinuation

4.1.2.1. NRTI Discontinuation Criteria

Upon participants completing Week 72 visits, the investigator will evaluate whether participants are eligible for discontinuation of NRTI therapy based on the following NRTI discontinuation criteria:

- HBsAg < LLOQ AND
- HBV DNA < LLOQ AND
- Undetectable HBeAg AND
- ALT ≤ 2x ULN

Participants who meet all of the above criteria for at least two consecutive visits at Week 72 are eligible to discontinue from NRTI therapy. Investigator should confirm participants' eligibility as early as possible within 2 weeks post Week 72 visit. Participants who discontinue NRTI will enter the NRTI Discontinuation Monitoring Period of the study within 1 week of the investigator confirming eligibility. Participants will not take their NRTI medications on the first day of the NRTI Discontinuation Monitoring Period and will be followed for an additional 24 weeks per the SoA (Section 1.3).

4.1.2.2. NRTI Re-treatment

It is recommended that the Investigator should consider re-treatment with NRTI therapy during the NRTI Discontinuation Monitoring Period if the participant meets any of the following criteria:

- ALT > 10x ULN with HBV DNA >LLOQ at the same visit
- ALT 5-10x ULN with HBV DNA >LLOQ persisting for ≥ 4 consecutive weeks
- ALT > ULN and HBV DNA > 2,000 IU/mL persisting for ≥ 4 consecutive weeks
- ALT > ULN with total bilirubin > 2x ULN and/or prolongation of prothrombin time (PT) > 2 seconds, and HBV DNA > LLOQ at the same visit
- HBV DNA > 100,000 IU/mL at any visit
- Confirmed HBeAg seroreversion (i.e., HBeAg positive after being HBeAg negative at NRTI discontinuation)
- Any other clinically significant event(s) warranting initiation of NRTI therapy in the opinion of the Investigator

Whether participants should be re-treated with NRTI is at the discretion of the Investigator following good medical judgement. Participants who are re-treated with NRTI therapy should continue to be followed per the SoA (Section 1.3.2) for the duration of the 24-week NRTI Discontinuation Monitoring Period.

Participants with HBV DNA increase ≥ 2 $\log_{10}$ IU/mL within a 2-week period should:

- Return to the site for an unscheduled visit to measure HBV DNA levels and liver function tests as soon as possible after the investigator being aware of the HBV DNA increase
- Continue returning to site weekly until HBV DNA levels stabilize (do not increase ≥ 1 log_{10} IU/mL over a 4-week period or start decreasing for at least 2 consecutive visits) after which they will continue follow-up per the SOA for the NRTI Discontinuation Monitoring Period
- If any unscheduled visits needed as outlined above coincide with an existing visit per the SoA, the scheduled assessments should be conducted

4.1.3. Safety Review Committee

A Safety Review Committee (SRC) for this study will monitor participant safety and consider study-specific data as well as relevant background information about the study drugs and target population under study to inform on study conduct. The primary purpose of the SRC is to protect the safety of participants in this study.

Ad hoc SRC meetings will take place as needed, i.e., for a significant safety event such as criteria for study intervention discontinuation being reached. Decisions to suspend dosing or discontinue individual participants from study intervention will be made according to the predetermined criteria (Section 7). Participants may resume study intervention only following SRC approval. Additionally, the SRC may recommend discontinuation of or changes to the study to the Sponsor. The SRC charter outlining the member composition and operating procedures will be developed before the study initiation.

4.2. Scientific Rationale for Study Design

This Phase 2, open-label, randomized study will evaluate the efficacy and safety of BRII-835 and PEG-IFNα combination therapy. This study design will provide an opportunity to explore the ability of two different dose levels of BRII-835 to achieve a clinical meaningful increase in the rate of HBsAg loss and functional cure when in combination with PEG-IFNα.

The principle of mechanism of the combination therapy of BRII-835 and PEG-IFNα leading to an enhanced HBsAg loss in CHB patients was demonstrated recently in a small cohort of patients in a Phase 2 study (VIR-2218-1001). The concurrent initiation of BRII-835 and PEG-IFNα 48 weeks treatment resulted in the greatest reduction in HBsAg levels, and notable end of therapy HBsAg loss with anti-HBs seroconversion (30.8%, 4/13) compared to BRII-835 alone, or historical PEG-IFNα alone as well as PEG-IFNα following a BRII-835 lead-in (Yuen, 2022). This finding provided the basis to select the concurrent initiation of BRII-835 and PEG-IFNα combination therapy in this study.

The target population of this study is HBeAg-negative or HBeAg-positive non-cirrhotic patients with chronic HBV infection that has been virologically suppressed with NRTI therapy. This represents a patient population with acceptable heterogeneity who has been evaluated in BRII-835 single drug studies (VIR-2218-1001 study and VIR-2218-1005 study) and notable HBsAg reduction has been observed. The previous data of PEG-IFNα in combination with NRTI showed that HBsAg loss and functional cure rate could be higher in patients with baseline HBsAg

<3,000 IU/ml or 1,500 IU/ml (Hu, 2018; Gan, 2022). Based on the scientific rationale that siRNA reduction in HBsAg by BRII-835 may potentiate effect of PEG-IFNα, the combination of BRII-835 and PEG-IFNα are anticipated to increase the likelihood of HBsAg loss and functional cure rate compared to PEG-IFNα in patients with baseline HBsAg 100-3,000 IU/ml. The preliminary end of therapy data in VIR-2218-1001 study also showed higher HBsAg loss rate (40%) could be achieved compared to historical PEG-IFNα data in patients with lower baseline HBsAg (<1,500 IU/mL) that received 48 weeks concurrent initiation of BRII-835 and PEG-IFNα combination therapy (Yuen, 2022).

Viral rebound may occur shortly after NRTI withdrawal in some cases, which requires close monitoring during study participation in order to control ALT flares. To safely evaluate the durability of response (i.e., sustained HBV DNA suppression and HBsAg loss) post study drug treatment, this study design allows additional 24 weeks NRTI treatment before evaluating the criteria for stopping NRTI therapy.

Surrogate endpoints of HBV functional cure measure biochemical, virological, and histological outcomes. Based on current knowledge, the best surrogate of HBV functional cure is HBsAg loss confirmed on 2 occasions at least 6 months apart with or without HBsAg seroconversion plus HBV DNA below the limit of detection (Cornberg, 2020).

4.3. Justification of Dose

Three parallel cohorts are planned for this study to evaluate 180 mcg weekly PEG-IFNα treatment alone for 48 weeks and 180 mcg weekly PEG-IFNα treatment in combination with two dose levels of BRII-835 at 100 mg or 200 mg administered every 4 weeks for 13 doses, respectively.

The current recommended PEG-IFNα dosage regimen for the treatment of chronic HBV infection is 180 mcg per week for a duration of 48 weeks (Terrault, 2016); this regimen will be used for PEG-IFNα dosing in this study.

The selection of BRII-835 dose levels is based on the safety/tolerability, PK, and antiviral activity data collected from a completed Phase 2 study VIR-2218-1005, an ongoing Phase 1/2 study VIR-2218-1001, and an ongoing Phase 2 study BRII-835-179-001 (also known as VIR-2218-1007).

The ongoing Phase 1/2 Study VIR-2218-1001 Parts D-F are evaluating the safety, tolerability, and antiviral activity of BRII-835 alone or in combination with PEG-IFNα (sequential or concurrent initiation) in noncirrhotic adult participants with chronic HBV infection on NRTI therapy. Patients received six doses of 200 mg BRII-835 alone achieved HBsAg reduction from baseline with a maximum mean decrease of -1.9 log10 IU/mL. To date, available data showed that BRII-835 and PEG-IFNα combination therapy achieved deeper reduction in HBsAg levels with continuous HBsAg declines observed through the combination treatment period. In a small cohort of patients (N=13), 48 weeks of concurrent 200 mg BRII-835 administered every four weeks for 13 doses and PEG-IFNα administered weekly for 48 weeks resulted in greatest HBsAg reductions compared to BRII-835 alone or PEG-IFNα following a BRII-835 lead-in, and achieved the highest rates of HBsAg seroclearance with anti-HBs seroconversion by the end of

treatment (30.8%, 4 of 13 participants) (Yuen, 2022). Safety data demonstrated that the BRII-835 and PEG-IFNα combination was generally well tolerated and exhibited safety profiles supportive of continued clinical development. The majority of TEAEs were Grade 1 or 2 in severity and most TEAEs reported are consistent with known effect of PEG-IFNα.

Study VIR-2218-1005 evaluated the safety/tolerability, PK, and efficacy of BRII-835 alone in both HBeAg-negative and HBeAg-positive Chinese participants with chronic HBV infection. BRII-835 was administered as two monthly subcutaneous (SC) doses of up to 100 mg. Results showed that two doses of BRII-835 up to 100 mg led to substantial reductions in HBsAg in both HBeAg-negative and HBeAg-positive participants. The reductions in HBsAg levels were more pronounced at the 100 mg dose level, with the mean maximum HBsAg reductions from baseline of -1.3 log10 IU/mL up to Week 16. The levels of serum HBsAg remained below baseline levels for up to Week 48, although a gradual rebound was observed. No participants had serum HBsAg loss or anti-HBs seroconversion at any timepoint. BRII-835 was generally well tolerated with a good safety profile; no apparent relationship between incidence of AE or treatment-emergent laboratory abnormalities and BRII-835 dose level or HBeAg status was observed.

In the ongoing Phase 2 study BRII-179-835-001 in patients with chronic HBV infection and with baseline HBsAg levels between 150 IU/mL and 3,000 IU/mL, 100 mg BRII-835 was administered every 4 weeks for 9 doses alone or in combination with BRII-179 (a therapeutic vaccine) with or without IFNα as co-adjuvant. Preliminary efficacy data showed that all cohorts achieved HBsAg reductions in serum HBsAg levels from baseline at the end of treatment with a mean decrease of -1.7 to -1.8 $\log_{10}$ IU/mL. Either BRII-835 alone or in combination with BRII-179 was generally well tolerated (Yuen, 2023).

Thus, based on the similar antiviral activity, especially observed in Study BRII-179-835-001 with 100 mg BRII-835 and Study VIR-2218-1001 with 200 mg BRII-835, as well as the good safety/tolerability profiles of BRII-835 either administered alone, in combination with BRII-179 with or without IFNα as co-adjuvant, or in combination with PEG-IFNα, the dose levels of BRII-835 at 100 mg or 200 mg administered every 4 weeks for a longer treatment duration (i.e., 13 doses) is deemed to be appropriate for further evaluation of the efficacy and safety when in combination with PEG-IFNα in this study.

The PK data to date showed that BRII-835 was rapidly absorbed after SC administration and had a short plasma $t_{1/2}$ of 2 to 5 hours. Plasma exposures for both BRII-835 and its metabolite AS(N-1)3'VIR-2218 increased in a dose dependent manner. Participants with chronic HBV infection had similar PK profiles as healthy participants. No accumulation of BRII-835 or AS(N-1)3'VIR-2218 in plasma was evident after multiple dosing.

In summary, the safety, PK and efficacy profiles support exploration of the proposed combination treatment regimens of BRII-835 at 100 mg and 200 mg every 4 weeks for 13 doses and PEG-IFNα at 180 mcg per week for 48 weeks. This study will provide information that enables determining the optimal combination of BRII-835 and PEG-IFNα, which could achieve significant and durable HBsAg reduction, have good safety/tolerability profiles, as well as minimize the economic burden on patients.

4.4. End of Study Definition

A participant is considered to have completed the study if he/she has completed all visits of the study including the last scheduled procedure shown in the SoA.

The end of the study is defined as the date of the last visit of the last participant in the study or last scheduled procedure shown in the SoA for the last participant in the trial.

5. STUDY POPULATION

5.1. Inclusion Criteria

Participants eligible to be included in the study must meet all of the following criteria. All laboratory test requirements for inclusion will be performed by central laboratory unless specified otherwise.

1. A signed and dated written Informed Consent Form (ICF) prior to study participation.
2. Age 18 (or age of legal consent, whichever is older) to 60 years, both inclusive.
3. Body mass index (BMI) ≥ 18 kg/m^2 and ≤ 32 kg/m^2.
4. Chronic HBV infection as defined by a positive serum HBsAg for ≥ 6 months prior to screening.
5. Must be on NRTI therapy consisting of either entecavir, tenofovir disoproxil, tenofovir amibufenamide, or tenofovir alafenamide and with HBV-DNA < LLOQ for at least 6 months before screening (local laboratory results accepted).
6. HBsAg > 100 IU/mL and ≤ 3,000 IU/mL at screening.
7. HBV DNA < LLOQ at screening.
8. Serum ALT and AST ≤ ULN at screening.
9. Female participants must have a negative pregnancy test or confirmation of postmenopausal status. Post-menopausal status is defined as 12 months with no menses without alternative medical cause. Women of child-bearing potential (WOCBP) must have a negative serum pregnancy test at screening and a negative urine pregnancy test on Day 1, cannot be breast feeding at screening and throughout the study, and must be willing to use highly effective methods of contraception 14 days before study drug administration through 48 weeks after last study drug administration (Section 11.4, Appendix 4). Female participants must also agree to refrain from egg donation and in vitro fertilization from the time of study intervention administration through 48 weeks after the last dose of study intervention.
10. Male participants with female partners of child-bearing potential must agree to meet 1 of the following contraception requirements from the time of study drug administration until 48 weeks after last study drug administration: vasectomy with documentation of azoospermia, or male condom use plus partner use of 1 of the contraceptive options listed for contraception for WOCBP. Male participants must also agree not to donate sperm for the 48 weeks following last study drug administration.
11. Agrees to not donate blood during the duration of the study.
12. Willing and able to comply with the study procedures and requirements.

Study screening laboratory tests may be repeated once for values considered to be questionable by investigator with medical monitor approval.

5.2. Exclusion Criteria

Participants will be excluded from the study if there is evidence of any of the following at screening or prior to randomization, as appropriate:

1. Current or prior treatment with:
   a) Small interfering RNAs or antisense oligonucleotides within 12 months prior to screening
   b) Interferon-based therapy within 12 months prior to screening
   c) Telbivudine within 6 months prior to screening
   d) Myelotoxic drugs such as systemic cytostatic agents (i.e., cancer chemotherapeutic medications) within 30 days before study drug administration at the Day 1 visit
   e) Systemic immunosuppressant (including but not limited to corticosteroids at a prednisolone equivalent dose > 40 mg/day) within 30 days before study drug administration at the Day 1 visit
   f) Granulocyte colony-stimulating factor (G-CSF), granulocyte-macrophage colony-stimulating factor (GM-CSF), recombinant human interleukin-11 (oprelvekin), or erythropoietin (EPO) within 6 months prior to screening
2. Any clinically significant chronic medical condition other than chronic HBV infection that, in the opinion of the investigator makes the participant unsuitable for participation in the study.
3. Any clinically significant acute condition such as fever (>38°C) or acute respiratory illness within 7 days of first study drug administration.
4. Significant liver fibrosis or cirrhosis as defined by having a FibroScan result of > 9 kPa at screening.
5. History or evidence of drug or alcohol abuse (80 g of alcohol/day for men and 40 g of alcohol/day for women) as assessed by the Investigator, within the 12 months before screening.
6. History of clinically significant chronic liver disease from any cause other than chronic HBV infection.
7. History of hepatic decompensation, including but not limited to ascites, hepatic encephalopathy and/or esophageal or gastric varices.
8. Diagnosed or suspected hepatocellular carcinoma. Participant with screening alpha-fetoprotein (AFP) concentration ≥200 nanogram per milliliter (ng/mL) should be excluded. If the screening AFP concentration is ≥50 ng/mL and <200 ng/mL, the absence of liver mass must be documented by imaging at screening.

9. History of thyroid disease not adequately controlled on prescribed medications, or clinically significant signs of thyroid dysfunction as determined by the Investigator at screening.
10. Known history of immunological function impairment, including but not limited to:
    a) Autoimmune disease (e.g., multiple sclerosis, type 1 diabetes mellitus , myasthenia gravis, Crohn's disease and other inflammatory bowel diseases, celiac disease, systemic lupus erythematosus, scleroderma, including diffuse systemic form and limited cutaneous form of systemic sclerosis or CREST syndrome, systemic sclerosis, dermatomyositis polymyositis, rheumatoid arthritis, juvenile idiopathic arthritis, autoimmune thyroiditis including Hashimoto thyroiditis, Grave's or Basedow's disease, immune thrombocytopenic purpura, autoimmune hemolytic anemia, autoimmune hepatitis, psoriasis, vitiligo, vasculitis, Guillain Barré syndrome, Addison's disease, Bell's palsy, and alopecia areata); OR
    b) Primary immunodeficiency disorders (e.g., common variable immune deficiency, defective phagocytic cell function and neutropenia syndromes, and complement deficiency); OR
    c) Secondary immunodeficiency disorders (e.g., resulting from acquired immunodeficiency syndrome caused by human immunodeficiency virus (HIV) infection, solid organ transplant, and splenectomy).
11. Current or past history of infection with human immunodeficiency virus (HIV), hepatitis C virus (HCV) or hepatitis Delta virus (HDV).
12. Renal impairment with estimated glomerular filtration rate (eGFR) ≤ 60 ml/min/1.73m^2 at screening.
13. Any laboratory test abnormality that is considered as clinically significant or may contradict treatment with PEG-IFNα, including but not limited to below laboratory abnormalities:
    a) Hemoglobin < 100 g/L
    b) Absolute neutrophil count < 1,500/mm^3 (< 1000/ mm^3 for African)
    c) Platelet count < 90,000/ mm^3
14. Uncontrolled diabetes mellitus as determined by the Investigator.
15. Uncontrolled hypertension as determined by the Investigator.
16. Diagnosis of advanced stage heart failure (New York Heart Association Class III or Class IV or unstable angina).
17. Previous or current psychiatric condition that precludes compliance with the protocol. Specifically excluded are persons with severe psychiatric disorders as determined by the Investigator, including, but not limited to schizophrenia, psychosis, severe depression, bipolar illness, severe anxiety, ongoing risk for suicide, or history of suicide attempt or gesture within the past 5 years.

18. The participant has any history of cancer requiring locoregional or systemic therapy within 5 years of randomization or has current disease. Participants with completely surgically resected tumors with no evidence of disease for more than 3 years, or a history of low risk basal cell carcinoma will be accepted (low risk being defined as 1) location on the trunk of the body, arms, legs, cheeks, forehead, temples, scalp, neck, or chin, 2) < 2 cm in size, 3) nodular or superficial, 4) primary cancer that has not come back after treatment, 5) edge of the cancerous area is clear and smooth, and 6) not located in or around nerves).
19. History of intolerance to SC injection.
20. History of intolerance or hypersensitivity to interferon/pegylated interferon.
21. Known hypersensitivity or contraindication to an siRNA/GalNAc or their excipients.
22. Any skin abnormality or tattoo that would limit post treatment injection site assessment.
23. Participated in another clinical trial with an investigational drug or a biologic within 30 days or within 5 drug half-lives (whichever is longer) before study drug administration at the Day 1 visit. Participants must agree not to take part in any other study at any time during their participation in this study, inclusive of the follow-up period and NRTI Discontinuation Monitoring Period.
24. Unwilling, or unable in the opinion of the Investigator, to comply with study requirements.

5.3. Screening Failures

Screening failures are defined as participants who consent to participate in the clinical study but are not subsequently randomly assigned to study drug. A minimal set of screening failure information is required to ensure transparent reporting of screening failure participants to meet the Consolidated Standards of Reporting Trials (CONSORT) publishing requirements and to respond to queries from regulatory authorities. Minimal information includes demography, screen failure details, eligibility criteria, and any SAE.

Individuals who do not meet the criteria for participation in this study (screening failure) should not be rescreened.

5.4. Lifestyle Considerations

Alcohol consumption is limited to no more than 1 unit per day (unit: 1 glass of wine [125 mL] = 1 measure of spirits [30 mL] = one-half pint of beer [284 mL]) during the participation of this study.

Participants will abstain from strenuous exercise from the time of screening throughout the study participation.

6. TREATMENT OF PARTICIPANTS

6.1. Study Drugs

6.1.1. General Information of Study Drugs

BRII-835 (VIR-2218) is a synthetic, chemically modified small interfering RNA (siRNA) targeting HBV RNA with a covalently attached triantennary N-acetyl-galactosamine (GalNAc) ligand that allows for specific uptake by hepatocytes. BRII-835 will be supplied as a clear, colorless to pale yellow, sterile solution for SC injection at a free acid concentration of 200 mg/mL.

PEG-IFNα is a covalent conjugate of recombinant alfa-2a interferon (approximate molecular weight [MW] 20,000 daltons) with a single branched bis-monomethoxy polyethylene glycol (PEG) chain (approximate MW 40,000 daltons). The PEG moiety is linked at a single site to the interferon alfa moiety via a stable amide bond to lysine. Peginterferon alfa-2a has an approximate molecular weight of 60,000 daltons. Interferon alfa-2a is produced using recombinant DNA technology in which a cloned human leukocyte interferon gene is inserted into and expressed in *Escherichia coli*. PEG-IFNα is a sterile, preservative-free, colorless to slightly yellowish solution available as an injection and is administered subcutaneously. PEG-IFNα (Pegasys, brand name Pegasys®) will be supplied as prefilled syringes containing 180 mcg/ 0.5 mL or 135 mcg/ 0.5 mL peginterferon alfa-2a.

Pegasys 180 micrograms solution for injection and Pegasys 135 micrograms solution for injection in pre-filled syringe each contains 0.5 ml solution with excipients of 180 micrograms (for 180 mcg/ 0.5 mL) and 135 micrograms (for 135 mcg/ 0.5 mL) peginterferon alfa-2a also contains sodium chloride, polysorbate 80, benzyl alcohol, sodium acetate, acetic acid and water for injections.

6.1.2. Study Drug Dose and Administration

Table 1 Study Drug Dosage and Administration

Cohort Name	Cohorts 1, 2, and 3	Cohort 2	Cohort 3
**Intervention Name**	PEG-IFNα (Pegasys, brand name Pegasys®)	BRII-835 (VIR-2218)	BRII-835 (VIR-2218)
**Dose Level**	180 mcg every week	200 mg every 4 weeks	100 mg every 4 weeks
**Dose Formulation**	Solution for Injection	Solution for Injection	Solution for Injection
**Unit Dose**	180 mcg/ 0.5 mL<br>135 mcg/ 0.5 mL	200 mg/mL	200 mg/mL

**Pack Volume**	0.5 mL/ pre-filled syringe	0.5 mL/ vial	0.5 mL/ vial
**Route of Administration**	SC injection	SC injection	SC injection

6.1.3. Study Drug Materials and Management

6.1.3.1. Study Drug Packaging and Labeling

PEG-IFNα (Pegasys®) and BRII-835 will be centrally supplied by Sponsor as Investigational Medication Products (IMPs).

Single panel label with one local language and/or booklet label with multiple languages will be applied to the inner pack and outer pack of IMPs per country regulation and requirements.

IMPs packaging and labeling and activities will be conducted in a contracted 3rd party Contracted Manufacturing Organization (CMO) fully compliant with Good Manufacturing Practice (GMP).

6.1.3.2. Study Drug Storage

BRII-835 should be stored upright and refrigerated (2°C - 8°C) in a secure, temperature controlled, and locked environment with restricted access.

PEG-IFNα (Pegasys®) should be stored in a refrigerator (2°C - 8°C). Do not freeze or shake. Keep the pre-filled syringes in the outer carton in order to protect from light.

6.1.3.3. Study Drug Preparation

Participant dose of BRII-835 preparation should follow the site sterilization dose preparation Standard Operating Procedure (SOP) or procedures described in the Pharmacy Manual at site by designated site staff.

Appropriate amount of PEG-IFNα (Pegasys®) will be dispensed to participant during each visit in dosing period.

Participants should be trained by designated site staff about how to store Pegasys® at home, strictly follow the instructed administration dose and follow the procedures addressed in the effective pharmacy manual to prepare for the injection at home.

6.1.3.4. Study Drug Administration

Each successfully randomized participant will receive an appropriate amount of PEG-IFNα (Pegasys®) in each visit in dosing period for taking home administration. Keep this medicine out of the sight and reach of children.

All participants should be trained in proper self-injection technique on Day 1. Pegasys® should be inspected visually for particulate matter and discoloration before administration, and not used if particulate matter is visible or product is discolored. Prefilled syringes with particulate matter or discoloration should be returned to the pharmacist. Participants will receive the first dose of

PEG-IFNα on Day 1 in the clinic and the following doses once weekly at home. Participants will be instructed to inject PEG-IFNα on the same day each week (e.g., each Monday).

Participants enrolled in Cohort 2 or Cohort 3 will receive BRII-835 at the assigned dose levels once every four weeks in the clinic. On dosing days, the pharmacist or designee will prepare the required amount of study drug into syringe for injection. A qualified clinical investigative site staff member under the supervision of the investigator or designee will administer the study drug. The injection site will be marked and mapped for later observation and should be documented. If a local reaction around the injection site occurs, photographs may be obtained. Participants will remain in the clinic for at least one hour post study drug administration. The observation period will include an assessment for rare adverse reactions.

Refer to the Pharmacy Manual for detailed instructions of study drug preparation and administration.

6.1.3.5. Study Drug Accountability

An IRT will be used to manage the IMPs supply. The initial site supply will be triggered by site initiation register in IRT.

Please follow IRT user specification to proceed any IMPs transaction at site.

The IMPs handling activities at site (e.g. IMPs shipment receiving, storage, temperature monitoring, storage temperature excursion case reporting, unused IMPs return, destruction of unused or expired IMPs) are addressed in pharmacy manual. Follow the effective pharmacy manual for details.

The investigator, institution, or the head of the medical institution (where applicable) is responsible for study intervention accountability, reconciliation, and record maintenance (i.e., receipt, reconciliation, and final disposition records).

Refer to the Pharmacy Manual for the details.

6.1.3.6. Study Drug Handling and Disposal

Unused IMPs could be destroyed at site in accordance with site SOP and local requirements or be returned back to a 3rd party for central destruction with written sponsor approval.

Any waste material should be disposed in accordance with local requirements and medical hazard disposal law.

The participants will be instructed by investigator or designated site staff to put the used Pegasys® syringe(s) back to the original outer pack(s) after injection and return them back to the site at the next visit. Participant should follow the instruction not to throw away any medicines via wastewater or household waste to help to protect the environment.

The unused PEG-IFNα should be stored in a refrigerator (2°C - 8°C) at home, no need to return back to site at the next visit. Investigator or designated site staff should check the availability of any unused PEG-IFNα with participant before requesting the new PEG-IFNα assignment via IRT.

Refer to the Pharmacy Manual for the details.

6.2. Randomization and Blinding

Up to 75 participants will be randomly assigned in 1:1:1 ratio to each of the three cohorts using an IRT system. The randomization code will be generated by using a validated computerized system. Before the study is initiated, the log in information and directions for the IRT system will be provided to each site. The site will contact the IRT system prior to the start of study drug administration for each participant. Once a randomization number is assigned to a participant, it cannot be reassigned to any other participant in the study. Study intervention will be dispensed at the study visits summarized in SoA. Returned study intervention should not be re-dispensed to the participants.

Participants will be stratified at randomization by screening HBsAg level (>100 IU/mL and ≤500 IU/mL, >500 IU/mL and ≤1,500 IU/mL, >1,500 IU/mL and ≤3,000 IU/mL). Maximum number of participants allowed in each stratum of each cohort will be capped as six in the IRT initial system set up for the first 45 enrolled participants and the limitation may be adjusted based on enrollment status.

As this is an open-label study, blinding procedures are not applicable.

6.3. Dose Adjustment Criteria

The dose of BRII-835 will not be adjusted once randomized to Cohort 2 or Cohort 3.

The dose of PEG-IFNα is based on relevant clinical guidelines and information from the package insert which is considered as proper by the investigator. The dose of PEG-IFNα should not be changed during the study unless dose modification or discontinuation is necessary as indicated by laboratory abnormalities/adverse events.

6.3.1. Safety Criteria for PEG-IFNα Dose Adjustment

If severe adverse reactions or laboratory abnormalities develop during PEG-IFNα therapy, the dose should be modified until the adverse reactions abate. If intolerance persists after dose adjustment, further PEG-IFNα dosing should be suspended. Table 2 provides guidance for dose modifications and discontinuation of PEG-IFNα based on laboratory abnormalities.

Table 2 PEG-IFNα Dose Modification Guidelines

Laboratory Parameters	PEG-IFNα Dose Reduction	PEG-IFNα Dose Interruption/Discontinuation
Absolute neutrophil count (ANC)	≥500, < 750 cells/mm^3: Reduce to 135 mcg once weekly	< 500 cells/mm^3: Treatment should be suspended until ANC values return to more than 1,000 cells/mm^3. Re-institute at 90 mcg and monitor ANC.

Platelet count	≥25,000, < 50,000 cells/mm^3: Reduce to 90 mcg once weekly	<25,000 cells/mm^3: Discontinue treatment
Alanine transaminase<br><br>(ALT)	≥5, < 10 × ULN: more frequent monitoring of liver function should be performed and consideration should be given to either reducing the dosage to 135 mcg or temporarily suspending treatment. After dosage reduction or withholding, therapy can be resumed after ALT flares subside (see Section 11.3).	≥ 10 × ULN: Discontinue treatment

During PEG-IFNα treatment, participants should be monitored for severe adverse reactions, including but not limited to neuropsychiatric, autoimmune, endocrine, ophthalmologic, and infectious disorders. Monitor closely and withdraw therapy with persistently severe or worsening signs or symptoms of the above disorders.

For detail information on dosage adjustment, please refer to the package insert of PEG-IFNα.

During safety monitoring of PEG-IFNα-related adverse reactions in the study, investigators should pay close attention to changes in all safety laboratory tests results (see Section 8.3.6.1). Relevant laboratory tests (not limited to protocol-specified safety laboratory tests) should be performed and followed in a timely manner if necessary (in case of adverse events or significant laboratory abnormalities). For participants whose laboratory measures are close to, but not yet met, the criteria for dose adjustment, investigators are advised to increase the frequency of monitoring of relevant laboratory measures. For participants who meet the criteria for PEG-IFNα dose adjustment, investigators should increase the frequency of monitoring of relevant laboratory measures, and if it is still early until next scheduled study visit, the participants should be advised to have the relevant laboratory measures re-tested 1 to 2 weeks later. Retesting may be conducted at local laboratories under urgent circumstances.

6.4. Background Treatment

All participants are required to be on NRTI therapy for at least 6 months at the screening visit and continued throughout the study. Participants must remain on the same NRTI at screening to the end of their study participation unless medically indicated otherwise, i.e., development of resistance.

Allowed NRTI therapy must consist of the following:

- Entecavir
- Tenofovir disoproxil
- Tenofovir amubufenamide

- Tenofovir alafenamide

6.5. Concomitant Medications

Medications specifically prohibited in the exclusion criteria are not allowed during the ongoing study. If there is a clinical indication for any medication specifically prohibited, discontinuation from study intervention may be required. The investigator should discuss any questions regarding this with the Sponsor medical monitor. The final decision on any supportive therapy or vaccination rests with the investigator and/or the participant's primary physician. However, the decision to continue the participant on study intervention requires the mutual agreement of the investigator, the Sponsor, and the participant.

6.5.1. Prohibited Medications

The following medications are excluded throughout the duration of study participation.

Within 30 days before study drug administration at the Day 1 visit and throughout the duration of study participation:

- Myelotoxic drugs such as systemic cytostatic agents (i.e., cancer chemotherapeutic medications).
- Systemic immunosuppressant (short term treatment of corticosteroids ≤40 mg/day and ≤ 2 weeks is allowed per the specialist's discretion as medically indicated)
- Any investigational drugs/biological products other than the study product(s) used during this study
- Any hepatotoxic agents including OTC medicines and herbal remedies that may cause fluctuations of liver test results

Within 90 days before study drug administration at the Day 1 visit and throughout the duration of study participation:

- Blood products or immunoglobin

Within 6 months before screening and throughout the duration of study participation:

- Telbivudine

6.6. Treatment Compliance

6.6.1. PEG-IFNα Treatment Compliance

The dose of PEG-IFNα is based on relevant clinical guidelines and information from the package insert which is considered as proper by the investigator. Temporary interruptions of PEG-IFNα administration are discouraged. Participants are to remain on treatment for the entire duration of the study until completion of 48-week treatment and the dose of PEG-IFNα should not be changed during the trial unless dose reduction or discontinuation is necessary as indicated by laboratory abnormalities / adverse events (see Section 6.3 for instructions for dose adjustment). Participants must be counselled regarding the importance of not missing doses of PEG-IFNα.

Participants should be instructed to inject PEG-IFNα on the same day each week (e.g., each Monday). In case that the participant missed any dose(s) at the planned day(s):

- Inject PEG-IFNα immediately if the subsequent dose to be administered on the next scheduled day will be at least 4 days after this day; OR
- Skip the current dose if the subsequent dose to be administered on the next scheduled day will be no more than 3 days after this day. Efforts should be made to administer the subsequent dose on the scheduled day

Participants will be instructed to bring all used syringes of PEG-IFNα without disposing the remaining medication to the trial site at every visit, as applicable, for adherence assessment. Adherence of PEG-IFNα will be assessed by counting of used syringes and remaining medication at every relevant visit. Every effort should be made to ensure good adherence with PEG-IFNα therapy.

6.6.2. NRTI Treatment Compliance

Participants will be instructed to bring all empty containers of NRTI including unused medications to the trial site at every visit, as applicable, for adherence assessment. Adherence of NRTI will be assessed by counting of empty containers and unused medications at every relevant visit. Every effort should be made to ensure good adherence with NRTI therapy.

7. DISCONTINUATION OF STUDY INTERVENTION AND PARTICIPANT DISCONTINUATION/WITHDRAWAL

7.1. Discontinuation of Study Intervention

A participant who has received at least one dose of BRII-835 or PEG-IFNα will hold the additional doses of either study drug if any of the following criterion is met:

- Discontinuation of study drug requested by the participant or the participant's legal representative.
- Signs or symptoms of severe National Cancer Institute Common Terminology Criteria for Adverse Events (NCI-CTCAE) (Grade 3 or higher) hypersensitivity or anaphylaxis
- Significant abnormalities in liver chemistry, defined as any of the following are met:
  - Serum ALT > 10 × ULN
  - Serum ALT or AST > 3 × ULN with a concomitant total bilirubin > 2 × ULN or INR > 1.5
  - Serum ALT ≥ 3× ULN with newly onset of any severe symptoms of suspected hepatic decompensation, including but not limited to fatigue, nausea, vomiting, and/or right upper abdominal pain
- Any clinical manifestations of hepatic decompensation
- Any clinical manifestations of severe depression
- The participant has a medical condition or personal condition that, in the opinion of the investigator and/or the Sponsor, would pose an unnecessary risk to the participant if the investigational intervention is continued.
- The participant's serum pregnancy test is confirmed to be positive.

The Sponsor may request SRC evaluation of specific data in the event of liver flare to fully characterize transaminase elevations. The participant may be resumed to receive remaining doses of study drug only following SRC approval.

In addition, PEG-IFNα treatment should be discontinued if a participant meets any of the following criteria:

- Significant abnormalities in hematology, defined as any of the following criteria are met:
  - Absolute neutrophil count < 500 cells/mm^3
  - Platelet count < 25,000 cells/mm^3
- Severe known side effects, including but not limited to development of worsening of neuropsychiatric, endocrine, ophthalmologic, and autoimmune disorders that, in the opinion of the investigator and/or the Sponsor, would pose unnecessary risk to the participant if the investigational intervention is continued

If a participant permanently discontinues one of the study drugs, this will result in early discontinuation of study treatment for this participant. The participant will not receive the remaining doses of the other study drug and will receive the End of Treatment (EOT) assessments within 14 days post the last dose of study drugs and will be followed per the SoA of the Follow-up Period.

See the SoA (Section 1.3) for data to be collected at the time of discontinuation of study drug and follow-up and for any further evaluations that need to be completed.

7.2. Participant Discontinuation/Withdrawal from the Study

- A participant may withdraw from the study at any time at his/her own request, or may be withdrawn at any time at the discretion of the Investigator for safety, behavioral, compliance, or administrative reasons. This is expected to be uncommon.
- At the time of discontinuing from the study, if possible, an early discontinuation visit will be conducted, as shown in the SoA. See SoA for data to be collected at the time of study discontinuation and follow-up and for any further evaluations that need to be completed.
- The participant will be permanently discontinued from both the study intervention and the study at that time.
- If the participant withdraws consent for disclosure of future information, the sponsor may retain and continue to use any data collected before such a withdrawal of consent.
- If a participant withdraws from the study, he/she may request destruction of any samples taken and not tested, and the investigator must document this in the site study records.
- Participants who are withdrawn from the study will NOT be replaced and cannot be rescreened.
- The primary reason for participant withdrawal will be documented in the source document and in the CRF. Primary reasons for withdrawal will be categorized as:
    - Adverse event
    - Protocol violation
    - Withdrawal by participants
    - Lost to follow up
    - Investigator’s decision
    - Death
    - Study closed/terminated

7.3. Lost to Follow up

A participant will be considered lost to follow-up if he or she repeatedly fails to return for scheduled visits and is unable to be contacted by the study site.

The following actions must be taken if a participant fails to return to the clinic for a required study visit:

- The site must attempt to contact the participant and reschedule the missed visit as soon as possible and counsel the participant on the importance of maintaining the assigned visit schedule and ascertain whether or not the participant wishes to and/or should continue in the study.
- Before a participant is deemed lost to follow up, the investigator or designee must make every effort to regain contact with the participant (where possible, 3 telephone calls and, if necessary, a certified letter to the participant's last known mailing address or local equivalent methods). These contact attempts should be documented in the participant's medical record.
- Should the participant continue to be unreachable, he/she will be considered to have withdrawn from the study.

Discontinuation of specific sites or of the study as a whole are handled as part of Section 11.1.7 (Appendix 1).

8. STUDY ASSESSMENT AND PROCEDURES

8.1. Schedule of Assessments

Study procedures and their timing are summarized in the SoA (Section 1.3). Adherence to the study design requirements, including those specified in the SoA, is essential and required for study conduct. Protocol waivers or exemptions are not allowed. Immediate safety concerns should be discussed with the sponsor immediately upon occurrence or awareness to determine if the participant should continue or discontinue study intervention.

8.1.1. Screening

Written informed consent must be obtained prior to initiation of any study procedures. All screening evaluations must be completed and reviewed to confirm that potential participants meet all eligibility criteria. The Investigator will maintain a screening log to record details of all participants screened and to confirm eligibility or record reasons for screening failure.

Screening will be performed no more than 4 weeks prior to the first dose for each cohort and will include written consent, determination of participant eligibility, collection of demographics and medical history as well as a physical examination (including vital signs measurement), 12-lead ECG, clinical laboratory tests, and other assessments per the SoA.

A medical history for at least 24 months prior to study screening, including details regarding illnesses and allergies, date(s) of onset, and whether condition(s) is currently ongoing (current medical conditions), medication history, including prior HBV treatment history and HBV genotype history (if available), will be collected on all participants during screening and should be updated prior to randomization. Efforts should be made to collect the prior HBV treatment history as complete as possible since the initial diagnosis of chronic HBV infection.

The screening viral serology parameters will include HIV antibody, HCV antibody, and HDV antibody to exclude current or past history of infection.

Adverse events related to protocol-mandated procedures during screening period must be collected from the time of consent onwards; any other events occurring during the screening period should be reported as medical history. All SAEs must be collected from the time of consent onwards.

8.1.2. Randomization (Day 1)

Participant eligibility must be confirmed prior to randomization and study drug administration on Day 1. Eligible participants will be randomized 1:1:1 to Cohort 1, 2, or 3 within 48 hours prior to study drug administration at the Day 1 visit. Eligible participants will receive their first dose of BRII-835 and will be assessed on physical examination (including vital sign measurement), clinical laboratory tests, and other assessments per the SoA. Participants will have the blood sample collection prior to study drug administration.

8.1.3. Dosing Period

Participants will return to the investigative site on an outpatient basis for safety, tolerability, and antiviral activity monitoring at the specified timepoints per the SoA.

All participants will receive 48 doses of PEG-IFNα administered SC every week from Day 1. Participants will be trained in proper self-injection technique and will receive the first dose of PEG-IFNα on Day 1 in the clinic. The following doses will be administered once weekly at home. Participants will be instructed to inject PEG-IFNα on the same day each week (e.g., each Monday). Participants must be counselled regarding the importance of not missing doses or overdosing of PEG-IFNα to the instructed dosage.

Participants enrolled in Cohort 2 and Cohort 3 will receive BRII-835 administered SC at the assigned dose levels every 4 weeks, i.e., at Day 1, Week 4, Week 8, Week 12, Week 16, Week 20, Week 24, Week 28, Week 32, Week 36, Week 40, Week 44 and Week 48 in the clinic.

At each visit during the Dosing Period, there will be a review of ongoing eligibility to receive the study drugs and continue with study participation. There will also be a blood sample collection prior to each study drug administration (when applicable). Participants enrolled in Cohort 2 and Cohort 3 will remain in the clinic at least one hour after each BRII-835 administration to be observed for rare adverse reactions. The injection site will be marked and mapped for later observation and should be documented. If a local reaction around the injection site occurs, photographs may be obtained.

If a participant permanently discontinues treatment prior to treatment completion, the EOT assessments should be performed within 14 days after treatment discontinuation per the SoA.

8.1.4. End of Treatment Visit

Participants may discontinue study drugs before the planned end of treatment (Week 48) or may withdraw from the study before the end of treatment (Week 48). In those cases, efforts should be made to conduct the EOT visit within 14 days after last dose of all study drugs. EOT assessments should be performed per the SoA.

8.1.5. Follow-up Period

Participants will return to the investigative site as outpatient for the specified in person assessments per the SoA, including but not limited to physical examination (including vital signs measurement), clinical laboratory tests (including safety, virological, and serological parameters), and review of adverse events and concomitant medications.

Participants will be assessed by the Investigator whether they are eligible for discontinuation of NRTI therapy upon completion of their Week 72 visits. Eligible participants to discontinue NRTI therapy may enter into NRTI Discontinuation Monitoring Period for an additional 24 weeks per the SoA.

Participants who have NOT achieved NRTI discontinuation criteria (Section 4.1.2.1) by Week 72 will end the study and NRTI treatment should be continued.

8.1.6. NRTI Discontinuation Monitoring Period

Eligible participants who meet NRTI discontinuation criteria will be informed by the investigator to discontinue their NRTI medications when all related lab test results be available. Participants will not take their NRTI medications on the first day of NRTI Discontinuation Monitoring Period. Participants will be instructed not to stop their NRTI medications early until the scheduled Day 1 of NRTI Discontinuation Monitoring Period.

Participants who discontinue from their NRTI therapy will be followed in the NRTI Discontinuation Monitoring Period for an additional 24 weeks from the day of NRTI discontinuation per the SoA (Section 1.3.2). Participants who are retreated with NRTI therapy should continue to be followed per SoA for the duration of the 24-week NRTI Discontinuation Monitoring Period.

8.1.7. Unscheduled Visits

Participants may be asked to come for an unscheduled visit for clinical assessment of an AE if warranted, as determined by the investigator. The unscheduled visit will be documented in source documents and recorded in the CRF.

8.1.8. End of Study Visit

In cases where a participant prematurely discontinues from the study, the investigator must make every effort to have the participant return to the clinic within 14 days for an End of Study (EOS) Visit, unless the participant withdraws consent for study participation or is lost to follow-up. Assessments for EOS visits will include but not limited to physical examination, pregnancy test (for female participants), clinical laboratory tests, and other assessments per the SoA.

8.2. Efficacy Assessments

8.2.1. Antiviral Activities

Assessment of antiviral activity will include quantitative HBV DNA, quantitative HBsAg, quantitative anti-HBs, qualitative HBeAg, and quanlitative anti-HBe.

Details regarding the processing, shipping, and analysis of the samples will be provided in the Laboratory Manual.

8.3. Safety Assessments

8.3.1. Physical Examination

A complete physical examination will include, at a minimum, general appearance, head, neck, chest/respiratory, heart/cardiovascular, gastrointestinal/liver, extremities, skin, and neurological assessments. Height and weight will also be measured and recorded. BMI will be calculated from height and weight.

A symptom-directed physical examination will be performed according to investigator discretion.

8.3.2. Vital Signs

Temperature (oral preferred), pulse rate, respiratory rate, and blood pressure will be assessed after the participant has rested comfortably for approximately 10 minutes. Pulse rate and blood pressure measurements will be assessed with a completely automated device. Manual techniques will be used only if an automated device is not available. When scheduled at the same timepoints, vital signs and 12-lead ECG are recommended to be assessed prior to physical examinations, blood sample collections, and urine collection.

8.3.3. Alcohol Intake Assessment

Alcohol intake during the study will be recorded and reviewed.

8.3.4. Liver Stiffness

Liver stiffness will be measured by FibroScan examinations as outlined in SoA.

Screening FibroScan result will be used to exclude the presence of significant liver fibrosis and cirrhosis. Participant with a FibroScan result of ≤ 9 kPa at screening can be considered without significant liver fibrosis and cirrhosis.

8.3.5. Ultrasound

Abdominal Ultrasound examinations will be performed and reviewed on-site by the Investigator at screening and every 6 months during the study for HCC surveillance as outlined in SoA.

8.3.6. Laboratory Assessments

8.3.6.1. Clinical Safety Laboratory Assessments

Clinical safety laboratory tests to be performed are outlined in SoA and described in details in Section 11.2 Appendix 2.

The investigator must review the laboratory report, document this review, and record any clinically significant changes occurring during the study in the AE section of the CRF. The laboratory reports must be filed with the source documents. Clinically significant abnormal laboratory findings are those which are not associated with the underlying disease, unless judged by the investigator to be more severe than expected for the participant's condition.

In the event of an unexplained clinically relevant abnormal laboratory test occurring after study drug administration, the test should be repeated and followed at investigator discretion until it has returned to normal or baseline, and/or a diagnosis is made to adequately explain the abnormality.

8.3.6.2. Additional Clinical Assessment for The Adverse Reactions of PEG-IFNα

The investigator can conduct additional clinical assessments for the adverse reactions of PEG-IFNα, including but not limited to radiological examination, ophthalmology assessment, immunological markers, blood glucose, and psychological assessment. These assessments can be performed at local laboratory.

8.3.6.3. Pregnancy Testing

A pregnancy test or confirmation of post-menopausal status must be confirmed for all female participants. Post-menopausal status is defined as 12 months with no menses without alternative medical cause. An elevated FSH level in the post-menopausal range should be used to confirm a post-menopausal state in women not using hormonal contraception or hormone replacement therapy. In the absence of 12 months of amenorrhea, confirmation with more than one FSH measurement is required. Pregnancy tests will be performed for WOCBP only. Serum and urine pregnancy testing will be performed per the SoA and any time pregnancy is suspected. A WOCBP who is known to be pregnant or who does not have a negative pregnancy test at screening is not eligible for study participation. During the study, negative results of these pregnancy tests must be confirmed prior to study drug administration. A WOCBP determined to be pregnant while on study will be followed until the pregnancy outcome is known.

8.3.7. Laboratory Events of Clinical Interest

A clinical laboratory event of clinical interest (ECI) is a laboratory value of scientific and medical interest specific to understanding of the investigational product and may require additional follow-up testing, close monitoring, and rapid communication by the Investigator to the Sponsor.

Any laboratory ECIs will be monitored, defined as ALT ≥ 5x ULN, throughout this study.

All laboratory ECIs must be timely reviewed by the investigator and the assessment outlined in Section 11.3 should be performed as soon as possible and no later than one week after the initial ALT value meeting criteria for a laboratory ECI. Once an ECI subsides, i.e., the ALT level returns to ≤ ULN, a new ECI will be triggered if ALT increases and meets the criteria defined above.

The laboratory ECI will also be evaluated by the investigator to determine if the event meets criteria for an AE. If AE criteria are met, all clinical sequelae in relation to these laboratory ECIs will be reported as AEs or SAEs, if applicable.

8.4. Adverse Events and Serious Adverse Events

AEs will be reported by the participant.

The investigator and any qualified designees are responsible for detecting, documenting, and recording events that meet the definition of an AE or SAE and remain responsible for following

up AEs that are serious, considered related to the study intervention or study procedures, or that caused the participant to discontinue the study intervention or study.

8.4.1. Definition of Adverse Events

8.4.1.1. Adverse Event (AE)

An adverse event is any untoward medical occurrence in a clinical study participant administered a medicinal product, which does not necessarily have a causal relationship with the treatment. An AE can therefore be any unfavorable and/or unintended sign, symptom, or disease temporally associated with the use of a medicinal product, whether or not considered related to the medicinal product. AEs may also include pre- or post-treatment complications that occur as a result of protocol specified procedures, lack of efficacy, overdose, drug abuse/misuse reports, or occupational exposure. Pre-existing conditions (recorded as medical history), which change in nature or severity should also be considered AEs.

An AE does not include the following:

- Medical or surgical procedures such as surgery, endoscopy, tooth extraction, and transfusion. The condition that leads to the procedure may be an adverse event and must be reported.
- Pre-existing diseases, conditions, or laboratory abnormalities present or detected before the screening visit that do not worsen
- Situations where an untoward medical occurrence has not occurred (eg, hospitalization for elective surgery, social and/or convenience admissions)
- Overdose of study drug without clinical sequelae
- Any medical condition or clinically significant laboratory abnormality with an onset date before the consent form is signed and not related to a protocol-associated procedure is not an AE. It is considered to be pre-existing and should be documented as medical history
- Any clinically significant abnormal laboratory findings or other abnormal safety assessments which are associated with the underlying disease, unless judged by the investigator to be more severe than expected for the participant’s condition

The criteria for considering whether an abnormal clinical laboratory finding or other abnormal assessments (eg, electrocardiogram) should be reported as an adverse event:

- Test result is associated with accompanying symptoms
- Test result requires medical/surgical intervention
- Test result leads to dose changing of study drug
- Test result is considered to be an adverse event by the investigator

If the laboratory abnormality is part of a clinical syndrome, record the syndrome or diagnosis (eg, anemia), not the laboratory result (ie, decreased hemoglobin).

8.4.1.2. Serious Adverse Event (SAE)

A serious adverse event as defined by ICH is any untoward medical occurrence that at any dose:

- Results in death
- Is life-threatening: An AE is life threatening if the participant was at immediate risk of death from the event as it occurred; ie, it does not include a reaction that if it had occurred in a more serious form might have caused death. For example, drug induced hepatitis that resolved without evidence of hepatic failure would not be considered life threatening even though drug induced hepatitis can be fatal.
- Requires inpatient hospitalization or prolongation of existing hospitalization: AEs requiring hospitalization should be considered SAEs. Hospitalization for elective surgery or routine clinical procedures that are not the result of AE (eg, elective surgery for a pre-existing condition that has not worsened) need not be considered AEs or SAEs. If anything untoward is reported during the procedure, that occurrence must be reported as an AE, either 'serious' or 'non-serious' according to the usual criteria. In general, hospitalization signifies that the participant has been detained (usually involving at least an overnight stay) at the hospital or emergency ward for observation and/or treatment that would not have been appropriate in the physician's office or outpatient setting. When in doubt as to whether 'hospitalization' occurred or was necessary, the AE should be considered serious.
- Results in persistent or significant disability/incapacity: An AE is incapacitating or disabling if the experience results in a substantial and/or permanent disruption of the participant's ability to carry out normal life functions.
- Is a congenital anomaly/birth defect in the offspring of a participant who received study drug(s).
- Other medically significant events: Important medical events that may not result in death, be life-threatening, or require hospitalization, may be considered an SAE when, based upon appropriate medical judgment, they may jeopardize the participant and may require medical or surgical intervention to prevent one of the outcomes listed in the above definition. Examples of such events are:
    - ➢ Intensive treatment in an emergency room or at home for allergic bronchospasm
    - ➢ Blood dyscrasias or convulsions that do not result in inpatient hospitalization
    - ➢ Development of drug dependency or drug abuse

8.4.2. Assessment of Adverse Events

8.4.2.1. Assessment of Causality

An Investigator or a qualified sub-investigator must make the determination of relationship to the study drug for each AE (Related or Not Related). The Investigator should decide whether, in his or her medical judgment, there is a reasonable possibility that the event may have been caused by

the study drug. A "reasonable possibility" of a relationship conveys that there are facts, evidence, and/or arguments to suggest a causal relationship, rather than a relationship cannot be ruled out. "Related" should be entered when there is either a reasonable possibility that the AE/SAE is associated with study intervention or no reasonable alternative explanation can be found. Otherwise, the relationship between AEs and the study intervention should be classified as "Unrelated".

8.4.2.2. Assessment of Severity

The investigator will make an assessment of intensity for each AE reported during the study based on the NCI-CTCAE Version 5.0. The CTCAE displays Grade 1 through 5 with unique clinical descriptions of severity for each AE based on this general guideline (Table 3).

Table 3 CTCAE (V5.0) General Guideline of Severity of AEs

Severity	Definition
Grade 1	Mild; asymptomatic or mild symptoms; clinical or diagnostic observations only; intervention not indicated.
Grade 2	Moderate; minimal, local or noninvasive intervention indicated; limiting age-appropriate instrumental ADL. Instrumental ADL refer to preparing meals, shopping for groceries or clothes, using the telephone, managing money, etc.
Grade 3	Severe or medically significant but not immediately life-threatening; hospitalization or prolongation of hospitalization indicated; disabling; limiting self-care ADL. Self-care ADL refer to bathing, dressing and undressing, feeding self, using the toilet, taking medications, and not bedridden.
Grade 4	Life-threatening consequences; urgent intervention indicated.
Grade 5	Death related to AE.

ADL = activities of daily living
A semi-colon indicates 'or' within the description of the grade
Source: NCI 2017

8.4.3. Time Period and Frequency for Collecting AEs and SAEs

AEs

After informed consent, but prior to initiation of study intervention, the following type of events should be reported: AEs related to protocol-mandated procedures. Other medical occurrences that begin before the start of study intervention but after obtaining informed consent, that are deemed not related to the screening activities, will be recorded as Medical History. Following initiation of study intervention, collect all AEs, regardless of cause or relationship to study drug, until the participant has completed the study.

SAEs

All SAEs, regardless of cause or relationship to study drug, that occur after the participant first consent to participate in the study (ie, signing the informed consent) and throughout the duration of the study, until the participant has completed the study, must be reported as instructed. This also includes any SAEs resulting from protocol-mandated procedures performed after informed consent is signed.

Investigators are not obligated to actively seek SAEs after the participant completed the study. However, if the investigator learns of any SAEs that occur after study participation has concluded and the event is deemed relevant to the use of study drug, he/she should promptly document and report the event to the Sponsor.

8.4.4. Recording of AEs and SAEs

When an AE/SAE occurs, it is the responsibility of the investigator to review all documentation (e.g., hospital progress notes, laboratory reports, and diagnostics reports) related to the event.

The investigator will then record all relevant AE/SAE information in the CRF and additional SAE form.

It is not encouraged to directly send photocopies of the participant's medical records to the Sponsor in lieu of completion of the AE/SAE form.

There may be instances when copies of medical records for certain cases are requested by the Sponsor. In this case, all participant identifiers, with the exception of the participant number, will be redacted on the copies of the medical records before submission to the Sponsor.

The investigator will attempt to establish a diagnosis of the event based on signs, symptoms, and/or other clinical information. Whenever possible, the diagnosis (not the individual signs/symptoms) will be documented as the AE/SAE.

8.4.5. Follow-up of AEs and SAEs

During the course of the study all AEs/SAEs should be proactively followed up for each participant. All SAEs should be followed until the event is resolved, stabilized, otherwise explained, or the participant is lost to follow-up. In addition, the investigator will make every attempt to follow all nonserious AEs for outcome. This may imply that observations will continue beyond the last planned visit per protocol.

The investigator is obligated to perform or arrange for the conduct of supplemental measurements and/or evaluations as medically indicated or as requested by the Sponsor to elucidate the nature and/or causality of the AE or SAE as fully as possible. This may include additional laboratory tests or investigations, histopathological examinations, or consultation with other health care professionals.

For fatal or life-threatening events, copies of hospital case reports, discharge summaries, autopsy reports, and other documents should be submitted by email when requested and applicable.

The investigator will submit any updated SAE data to the Sponsor within 24 hours of receipt of the information.

Additional information may be requested to ensure the timely completion of accurate safety reports.

8.4.6. Reporting of SAEs

All SAEs must be reported by sending to the Sponsor or designee (Novotech Safety) via secure e-mail connection within 24 hours of the first awareness of the event. The Investigator must complete, sign and date the SAE form, verify the accuracy of the information recorded on the SAE form with the corresponding source documents, and send a copy to sponsor or its designee.

Contacts for SAE reporting: Novotech Safety at email: safety@novotech-cro.com

8.4.7. Regulatory Reporting Requirements for SAEs

Prompt notification by the investigator to the sponsor of an SAE is essential so that legal obligations and ethical responsibilities towards the safety of participants and the safety of a study intervention under clinical investigation are met.

The sponsor has a legal responsibility to notify both the local regulatory authority and other regulatory agencies about the safety of a study intervention under clinical investigation. The sponsor will comply with country-specific regulatory requirements relating to safety reporting to the regulatory authority, Institutional Review Boards (IRB)/Independent Ethics Committees (IEC), and investigators.

Safety reports must be prepared for suspected unexpected serious adverse reactions (SUSAR) according to local regulatory requirements and sponsor policy and forwarded to investigators as necessary.

An investigator who receives an investigator safety report describing an SAE or other specific safety information from the sponsor will review and then file it along with the Investigator's Brochure and will notify the IRB/IEC, if appropriate according to local requirements.

8.4.8. Special Situation Reports

8.4.8.1. Definition of Special Situations

Special situation reports include all reports of medication error, abuse, misuse, overdose, reports of adverse events associated with product complaints, and pregnancy reports regardless of an associated AE.

Medication error is any unintentional error in the prescribing, dispensing, or administration of a medicinal product while in the control of the health care provider, participant, or consumer.

Abuse is defined as persistent or sporadic intentional excessive use of a medicinal product by a subject.

Misuse is defined as any intentional and inappropriate use of a medicinal product that is not in accordance with the protocol instructions or the local prescribing information.

An overdose is defined as an accidental or intentional administration of a quantity of a medicinal product given per administration or cumulatively which is above the maximum recommended dose as per protocol (as it applies to the daily dose of the participant in question). In cases of a discrepancy in drug accountability, overdose will be established only when it is clear that the subject has taken the excess dose(s). Overdose cannot be established when the pharmacist cannot account for the discrepancy except in cases in which the investigator has reason to suspect that the additional dose(s) were administered to the participant.

Product complaint is defined as complaints arising from potential deviations in the manufacture, packaging, or distribution of the medicinal product.

Pregnancy is defined as the any pregnancy event of a female participant entered in a study as well as pregnancy occurring in a female partner of a male participant entered in a study with a medicinal product. If a participant becomes pregnant during the course of the study, study drug should be discontinued immediately.

8.4.8.2. Instruction for reporting special situations

8.4.8.2.1. Pregnancy reporting

If a female participant becomes pregnant after the start of study drug administration through 48 weeks after the last study drug administration, the participant will be instructed to report this to the investigator. The investigator must report the pregnancy to the Sponsor or designee (safety@novotech-cro.com) within 24 hours of being notified of the pregnancy. The participant should receive any necessary counseling regarding the risk of continuing the pregnancy and the possible effects on the fetus.

The pregnancy should be followed by the investigator to determine the outcome of the pregnancy. The investigator will collect follow-up information on the mother and infant and will notify the Sponsor within 24 hours of obtaining updated information regarding the pregnancy. At the completion of the pregnancy, the investigator will document the outcome of the pregnancy. Abnormal pregnancy outcomes (e.g., spontaneous abortion, fetal death, stillbirth, congenital anomalies, ectopic pregnancy) are considered SAEs. Elective abortions without complications should not be handled as AEs. Every effort will be done to follow the infant for up to 6-8 weeks after birth.

If the partner of a male participant becomes pregnant after the start of study drug administration through 48 weeks after the last study drug administration, the participant will be instructed to report this to the investigator. The partner will be asked to provide consent to be followed until the outcome of the pregnancy is known and for up to 6-8 weeks after birth (where permitted). The investigator must report the pregnancy to the Sponsor or designee (safety@novotech-cro.com) within 24 hours of being notified of the pregnancy. The participant and the partner should receive any necessary counseling regarding the risk of continuing the pregnancy and the possible effects of the fetus. After obtaining consent, the investigator must report available follow-up information on the course and outcome of the pregnancy within 24 hours of receipt of the information.

8.4.8.2.2. Reporting other special situations

All other special situations must be reported to the Sponsor or designee (safety@novotech-cro.com) within 24 hours of the investigator becoming aware of the situation. These reports must consist of situations that involve study drug.

Special situations involving medications other than study drug do not need to be reported; however, for special situations that result in AEs due to a medication other than study drug, the AE should be reported.

Any inappropriate use of concomitant medications prohibited by this protocol should not be reported as “misuse” but may be more appropriately documented as a protocol deviation.

All clinical sequelae in relation to these special situation reports will be reported as AEs or SAEs at the same time. Details of the symptoms and signs, clinical management, and outcome will be reported, when available.

8.5. Pharmacokinetic (PK) Assessments

Blood samples will be collected for measurement of plasma concentrations of BRII-835 and its metabolite as specified in the SoA. Timepoints for the collection of BRII-835 PK samples are provided in Table 4. Instructions for the collection and handling of blood samples will be provided in the Laboratory Manual. The actual date and time (24-hour clock time) of each sample will be recorded.

Table 4 Pharmacokinetics Assessment Timepoints

Study Day/Week	Protocol Time		
	Predose[a]	Dose	Postdose[b]
Day 1	X		X
Day 225/ Week 32	X		X
Day 337/ Week 48	X		X

[a] At ≤ 2 hours prior to dosing
[b] At ≥ 2 hours and ≤ 12 hours after dosing

8.6. Assessment of Anti-Drug Antibody (ADA) to BRII-835

Blood samples will be collected for analyses of ADA to BRII-835 as specified in the SoA. See the Laboratory Manual for instructions on sample processing and storage.

8.7. [redacted]

[redacted]

[redacted]

[redacted]

- [redacted]
- [redacted]

[redacted]

[redacted]

[redacted]

9. STATISTICS

Detailed methodology for summary and statistical analyses of the data collected in this study is outlined here and further detailed in the statistical analysis plan (SAP), which will be maintained by the sponsor. The SAP may modify what is outlined in the protocol where appropriate; however, any major modifications of the primary endpoint definitions or their analyses will be reflected in a protocol amendment.

9.1. Statistical Hypotheses

An estimation approach with no hypothesis testing is used to analyze the primary outcomes. A Bayesian framework will be used to calculate the point estimate of the primary outcomes and 95% credible interval.

9.2. Estimands

Primary Estimand 1

The primary estimand 1 of the study is a while on treatment estimand, participants prematurely discontinue treatment will be treated as intercurrent event, which estimates the on-treatment effect.

- Population: Participants with CHB as defined by the inclusion and exclusion criteria to reflect the targeted participant population;
- Variable: HBsAg loss at end of treatment;
- Treatment: Cohort 1, 2, and 3. Estimation of the within-group response rate;
- Intercurrent event: The intercurrent event is prematurely discontinue treatment;
- Population-level summary: Proportion of participants who achieve HBsAg loss at end of treatment for each cohort

Primary Estimand 1 is the primary estimand for the primary endpoint proportion of participants with HBV surface antigen (HBsAg) loss at end of treatment.

Primary Estimand 2

Primary Estimand 2 is a composite estimand, participants withdraw from the trial prematurely will be treated as non-responders, which estimates the effect of randomized treatment accounting for treatment adherence and response.

- Population: Participants with CHB as defined by the inclusion and exclusion criteria to reflect the targeted participant population;
- Variable: Responder is defined as participant achieves HBsAg loss at 24 weeks post end of treatment; for participants who drop out early for any reason the outcome will be defined as ‘non-responsive’ after that point;
- Treatment: Cohort 1, 2, and 3. Estimation of the within-group response rate;

- Intercurrent event: The intercurrent event is captured through the variable definition (i.e., withdrawal from the trial prematurely)
- Population-level summary: Proportion of participants who achieve response for each cohort

Primary Estimand 2 (composite estimand) is the primary estimand for the primary endpoint (Proportion of participants with HBsAg loss at 24 weeks post end of treatment) and key secondary efficacy endpoints (Proportion of participants with HBV surface antigen [HBsAg] loss at Week 48; Proportion of participants achieving sustained undetectable HBsAg and sustained suppression of HBV DNA<LLOQ ≥ 24 weeks after discontinuation of all treatment, including NRT; Proportion of participants achieving HBsAg loss and HBV DNA < LLOQ 24 weeks after discontinuation of all treatment, including NRTI).

Secondary Estimand(s)

Hypothetical Estimand (Estimand 3)

The secondary estimand of this study is a hypothetical estimand, which estimates the effect had all participants maintain their randomized treatment.

- Population: Participants with CHB as defined by the inclusion and exclusion criteria to reflect the targeted participant population;
- Variable: Change from baseline in HBsAg at Week 48 and Week 72;
- Treatment: Cohort 1, 2, and 3;
- Intercurrent event: All data collected will be utilized; Discontinuation of, interruption in, and non-adherence to treatment will be handled assuming they had not happened.
- Population-level summary: Difference in least-square means between each BRII-835 dose cohort and PEG-IFNα monotherapy cohort.

Change from baseline to each specific post baseline scheduled time points in a continuous outcome measure such as HBsAg will be based on hypothetical estimand.

9.3. Sample size determination

Up to 75 participants will be randomly assigned in 1:1:1 ratio to each of the three cohorts. A Bayesian framework will be used to calculate the sample size. It is assumed that the number of responders follows a Binomial distribution, with a weakly informative prior (Beta (0.5, 0.5)) for the true response rate. The precision for a range of response rates with 95% credible intervals are shown in Table 5:

Table 5 95% Credible Interval of Response Rate by Sample Size

Sample size per cohort	Number of responders	Response rate (%)	95% Credible interval* (%)

15	4	27	8-50
	5	33	13-57
	6	40	18-63
	7	47	25-71
	8	53	29-76
	9	60	36-82
25	6	24	10-41
	7	28	13-46
	8	32	16-51
	9	36	19-55
	10	40	22-58
	11	44	26-63
	12	48	30-67
	13	52	32-70

* 95% highest posterior density interval.

For the 15 participants in each cohort the lower bounds of 95% credible interval exclude the response rate of 10% if observed response rate is greater than 27% and exclude the response rate of 20% if observed response rate is greater than 40% and exclude the response rate of 30% if observed response rate is greater than 53%.

For the 25 participants in each cohort the lower bounds of 95% credible interval exclude the response rate of 10% if observed response rate is greater than 24% and exclude the response rate of 20% if observed response rate is greater than 36% and exclude the response rate of 30% if observed response rate is greater than 48%.The operational characteristics are based on posterior confidence that the true rate exceeds a threshold of interest, as shown in Table 6. For instance, when 4 out of 15 participants or 6 out of 25 participants are responders, the probability (true response rate>15%) is larger than 85%.

Table 6 Operational Characteristics by Sample Size

Sample size per cohort	Number of responders	Probability (True response rate)>15%	Probability (True response rate)>20%	Probability (True response rate)>25%	Probability (True response rate)>30%	Probability (True response rate)>35%
15	1	18.4%	8.5%	3.7%	1.4%	0.5%
	2	45.7%	27.2%	14.4%	7%	3.2%

	3	72.8%	52.6%	34.1%	20.1%	10.9%
	4	89.7%	75.6%	57.9%	40.6%	25.7%
	5	96.8%	90.4%	78.3%	62.7%	45.7%
	6	99.2%	96.6%	90.7%	80.4%	66.6%
	7	99.9%	99.2%	96.9%	92%	83.4%
25	2	16.2%	5.4%	1.6%	0.4%	0.1%
	3	35.7%	16.1%	6%	1.9%	0.5%
	4	58.2%	32.1%	14.9%	5.8%	1.9%
	5	77.4%	52.1%	29.4%	13.8%	5.5%
	6	89.1%	70.5%	46.9%	26.3%	12.1%
	7	95.9%	84.8%	65.5%	42.7%	23.6%
	8	98.5%	92.5%	79.4%	59.8%	38.3%
	9	99.6%	97.4%	89.6%	75%	55.2%
	10	99.9%	99%%	95.2%	86.1%	70.3%
	11	100%	99.7%	98.2%	93.3%	82.6%

9.4. Analysis Sets

Data for all participants will be assessed to determine if participants meet the criteria for inclusion in each analysis population prior to releasing the database and classifications will be documented per standard operating procedures.

Table 7 Analysis Sets

Population	Description
Screened Analysis Set	All participants who signed the ICF
Full Analysis Set (FAS)	All participants randomly assigned to study intervention
Safety Analysis Set (SAS)	All participants randomly assigned to study intervention and who take at least 1 dose of study drug. Participants will be analyzed according to the study intervention they received
Modified Intent-To-Treat (mITT)	All participants randomly assigned to study intervention, who take at least 1 dose of study drug, with at least 1 postbaseline visit through

	Week 48. Participants will be analyzed according to the study intervention to which they were randomized.
Per-Protocol (PP)	All participants in the mITT set without important protocol deviations considered to impact the interpretation of the primary efficacy endpoints. Protocol deviations will be reviewed to generate the list of participants with significant deviations to be excluded from the PP analysis set. The PP exclusion criteria will be finalized prior to releasing the database.
Pharmacokinetic Analysis Set (PAS)	All participants who received BRII-835, had a at least 1 evaluable PK concentration, and had no protocol deviations thought to impact on the PK data analysis.
ADA Analysis Set	All participants who received BRII-835 and had at least 1 non-missing ADA result from the ADA assay after first dose of the study drug. Participants will be analyzed according to the treatment actually received.

9.5. Statistical Methods

For continuous variables, descriptive statistics will include the following information: the number of participants reflected in the calculation (n), mean, standard deviation, first quartile (Q1), median, third quartile (Q3), minimum, and maximum. For categorical or ordinal data, frequencies and percentages will be displayed for each category.

Primary efficacy analysis will be Bayesian framework.

Statistical analysis will be performed using Statistical Analysis Software Version 9.4 or higher.

9.6. Statistical Analyses

9.6.1. Efficacy Analysis

9.6.1.1. Primary Efficacy analysis:

- The primary endpoints are 1) Proportion of participants with HBsAg loss at end of treatment 2) Proportion of participants with HBsAg loss at 24 weeks post end of treatment.

A Bayesian hierarchical model (Yuen, 2022; Jones, 2011) as primary analysis will be used to estimate the posterior probability of response rate for each cohort by incorporating the baseline HBsAg level stratification factor. The point estimate of the primary outcome and 95% equal-tailed credible interval will be drawn from the model.

Model for each group:

Number of responders $r_g \sim$ Binomial (n_g, p_g), g=1,2,3

$\theta_g = \text{logit}(p_g) = \log(p_g/(1-p_g)) = \gamma_0 + \gamma_1 I_{\{B+\}1} + \gamma_2 I_{\{B+\}2} + \psi_g$, g=1,2,3

where γ_0, γ_1, γ_2, ψ_g are all parameters, Thus,

$\theta_1 = \gamma_0 + \psi_1$

$\theta_2 = \gamma_0 + \gamma_1 + \psi_2$

$\theta_3 = \gamma_0 + \gamma_2 + \psi_3$

Priors:

$\gamma_k \sim$ Normal(0, 10^6), k=0,1,2

$\psi_g \sim$ Normal(0, ω^2)

$\omega \sim$ Half-normal(1)

Where r_g indicates the number of responders among n_g participants, p_g as the response rate r_g/n_g, θ_g indicates the log odds of treatment response $\log(p_g/(1-p_g))$, index g=1,2,3 refer to the stratum numbers, γ_k represent fixed effects of baseline HBsAg stratification factor, and ψ_g denotes a random effect in stratum g.

Stratum	$I_{\{B+\}1}$	$I_{\{B+\}2}$
HBsAg>100 IU/mL and HBsAg≤500 IU/mL	0	0
HBsAg>500 IU/mL and HBsAg≤1,500 IU/mL	1	0
HBsAg>1,500 IU/mL and HBsAg≤3,000 IU/mL	0	1

$I_{\{B+\}1}$ and $I_{\{B+\}2}$ are the dummy variables of $I_{\{B+\}}$

For each cohort, the posterior distribution of response rate $P(p_g|\text{data})$, g=1,2,3 was derived for each stratum using the model specified above.

The posterior distribution of the group-level response rate will be derived using a mixture of the posterior distributions of response rate for each analysis stratum in that group. The weights are proportional to the sample size of each stratum.

For some models with low number of events (0–1 event), the stratified Bayesian hierarchical model does not converge. In such cases a reduced model that does not consider baseline stratification will be fitted. The model estimates the point estimate and 95% highest posterior density credible interval of proportion of participants achieving the primary endpoint assuming the number of responders followed a binomial distribution:

Number of responders r ~ Binomial (n, p)

Where p is the response rate, with a non-informative (Jeffrey's) prior: p ~Beta (0.5, 0.5).

The posterior distribution for p is: P (p|y, N) ~ Beta (y + 0.5, N – y + 0.5).

In addition, combine some two adjacent baseline stratification levels to reduce HBsAg stratification factors from three levels to two levels (HBsAg >100 IU/mL and HBsAg ≤1,500 IU/mL versus HBsAg >1,500 IU/mL and HBsAg ≤3,000 IU/mL; HBsAg >100 IU/mL and HBsAg ≤500 IU/mL versus HBsAg >500 IU/mL and HBsAg ≤3,000 IU/mL) to perform the

stratified Bayesian hierarchical analysis if model is converged using the similar approach as the primary analysis.

Samples from the posterior distribution of the response rates in each cohort will be used to obtain posterior probabilities of interest (e.g., Probability [Difference of proportion of achieving primary endpoints between cohorts > threshold Data]); threshold such as 5%, 10%, 15%, 20% or any other percentiles of interest may be explored.

The primary efficacy analysis will be performed within mITT analysis set and a supplemental analysis based on PP analysis set with the similar analysis will be presented.

9.6.1.2. Secondary Endpoints Analyses

- Proportion of participants achieving functional cure (defined as sustained undetectable HBsAg and sustained suppression of HBV DNA <LLOQ ≥24 weeks after discontinuation of all treatment, including NRTI)
- Proportion of participants achieving HBsAg loss and HBV DNA <LLOQ 24 weeks after discontinuation of all treatment, including NRTI
- Proportion of participants meeting NRTI discontinuation criteria at 24 weeks post end of treatment

The analyses for the three endpoints are similar to the primary analysis of co-primary endpoint Proportion of participants with HBsAg loss at 24 weeks post end of treatment and will be based on mITT analysis set.

Absolute serum HBsAg and change from baseline over time through 24 weeks post end of treatment

Numerical value of HBsAg and change from baseline will be summarized by cohort and by visit. Change from baseline in HBsAg to all scheduled visits in log base 10 scale will be statistically analyzed using a Mixed Model for Repeated Measures model. The model will include log base 10 transformed baseline as a covariate, treatment, visit, treatment by visit as fixed effects, and within-participant errors will be modeled with an unstructured, heterogeneous autoregressive (1), or compound symmetry variance matrix in that order if a model does not converge. Estimates of least square mean (LSM) values and the LSM differences between each BRII-835 dose cohort and PEG-IFNα monotherapy cohort, and their respective 95% confidence interval and p-values will be calculated. The analyses will be done using mITT analysis set with hypothetical estimand. If the model is not converged only descriptive statistics will be presented.

In the first interim analysis, absolute serum HBsAg and change from baseline over time through Week 48 will be performed.

Nadir serum HBsAg and maximum reduction of serum HBsAg from baseline

Nadir serum HBsAg and maximum reduction of serum HBsAg from baseline will be summarized by cohort based on mITT analysis set.

Time to achieve first time of serum HBsAg loss through Week 72

Time to achieve first time of serum HBsAg loss will be analyzed by taking account of losses to follow-up using the stratified log-rank test with randomization strata as a stratification factor based on mITT analysis set, and tabular summaries of the Kaplan Meier (KM) curves providing the median, quartiles, and range will be provided for each cohort, and the KM curves will be presented graphically. In addition, a Cox proportional hazard regression model where the estimate of the hazard ratio for treatment (each BRII-835 dose cohort vs PEG-IFNα monotherapy cohort) will be performed, its 95% CI, and p value will be provided if the model is converged.

In the first interim analysis, time to achieve first time of serum HBsAg loss throughout Week 48 will be performed.

Proportion of participants with Hepatitis B e antigen (HBeAg) loss and/or HBeAg seroconversion (baseline HBeAg-positive participants)

Descriptive statistics by cohort based on mITT analysis set will be performed for this endpoint.

Proportion of participants with HBsAg seroconversion at any timepoint

Descriptive statistics by cohort based on mITT analysis set will be performed for this endpoint.

9.6.1.3. [redacted]

- [redacted]
- [redacted]
- [redacted]

[redacted]

9.6.1.4. Multiplicity

No multiplicity will be adjusted.

9.6.2. Safety Analysis

Safety analysis will be assessed using AEs, SAE, vital signs, clinical laboratory tests with clinician assessment, and concomitant medication data. Number and percentage of participants with TEAEs will be summarized by Medical Dictionary for Regulatory Activities (MedDRA)

System Organ Class (SOC) and preferred term (PT), and summaries will be provided by severity and by causality (related, not related) to study drug. Continuous safety data (including absolute and change from baseline values) will be summarized by descriptive statistics, including n, mean, standard deviation (SD), median, Q1, Q3 and minimum and maximum values. Categorical endpoints will be summarized by frequency and percentage.

9.6.3. Pharmacokinetic Analysis

Plasma concentrations of BRII-835 and its metabolite will be listed for each participant and descriptively summarized, including n, mean, standard deviation (SD), median, minimum, maximum, geometric mean, and geometric coefficient of variation (CV), by treatment cohort and timepoints using PK analysis set.

9.6.4. Baseline and Other Summaries and Analyses

The demographic characteristics will be summarized by cohort based on FAS. This will include age, gender, race, baseline weight and baseline BMI. All baseline disease characteristics will be summarized by cohort within the FAS.

9.6.4.1. Study Conduct and Participant Disposition

Participant evaluation groups will be presented for all screened participants, and participant disposition will be summarized within the FAS. The number of participants screened and randomized will be presented. The number of participants treated, completed and discontinued, as well as the number of participants in each analysis set will be summarized by Cohort. For participants who did not complete the study, the reasons for withdrawal from the study will be presented. In addition, the number of participants who were excluded from the PP analysis set will be summarized by reasons for the exclusion.

9.6.4.2. Study Treatment Exposure

Duration of treatment will be summarized based on SAS. The duration of treatment will be calculated as follows:

Duration of treatment = Date of last dose of study drug - date of first dose of study drug +1.

9.6.4.3. Prior and Concomitant Medications

The frequency of prior and concomitant medications will be summarized by cohort based on the WHO-drug coding dictionary based on SAS.

9.6.4.4. The occurrence of ADA to BRII-835 and associated titer (if positive)

Descriptive statistics for the endpoint will be performed based on ADA analysis set.

9.7. Interim Analysis

- **Interim analysis and summaries**

Three interim analyses are planned. The first two interim analyses will be conducted when approximately 45 enrolled participants complete the 48-week treatment and last available participant of all enrolled participants complete the 48-week treatment, respectively. If the pace of enrollment is robust, and the primary endpoint proportion of participants with HBsAg loss at Week 48 is expected to occur less or equal to two months between the two interim analyses, the two analyses may be collapsed into one analysis. Endpoints with measurement time after the 48-week treatment except for safety relevant data will not be analyzed in the first two interim analyses.

The third interim analysis will be conducted when last available participant completes follow-up 24 weeks post end of treatment.

9.8. Final Analysis

The final analysis will be performed when last available participant completes the last scheduled visit according to the SoA.

Further details will be provided in the SAP.

10. REFERENCES

Asian-Pacific clinical practice guidelines on the management of hepatitis B: a 2015 update. Hepatol Int. 2016 Jan;10(1):1-98.

Bourlière M, et al. Effect on HBs antigen clearance of addition of pegylated interferon alfa-2a to nucleos(t)ide analogue therapy versus nucleos(t)ide analogue therapy alone in patients with HBe antigen-negative chronic hepatitis B and sustained undetectable plasma hepatitis B virus DNA: a randomised, controlled, open-label trial. Lancet Gastroenterol Hepatol. 2017 Mar;2(3):177-188.

Cornberg M, Lok AS, Terrault NA, Zoulim F, and the 2019 EASL-AASLD HBV Treatment Endpoints Conference Faculty. Guidance for design and endpoints of clinical trials in chronic hepatitis B – Report from the 2019 EASL-AASLD HBV Treatment Endpoints Conference. Journal of Hepatology. 2020; 72(3): 539-337.

Fanning GC, Zoulim F, Hou J, Bertoletti A. Therapeutic strategies for hepatitis B virus infection: towards a cure.Nat Rev Drug Discov. 2019 Nov;18(11):827-844.

Ferrari C. HBV and the immune response. Liver Int. 2015; 35 (Suppl. 1): 121–128.

Gan W. Functional cure based on pegylated interferon α-2b therapy in nucleoside analog-suppressed HBeAg negative chronic hepatitis B: A multicenter real-world study (Everest Project in China) – 4 years data update. AASLD 2022, oral presentation.

GBD 2019 Hepatitis B Collaborators. Global, regional, and national burden of hepatitis B, 1990-2019: a systematic analysis for the Global Burden of Disease Study 2019. Lancet Gastroenterol Hepatol. 2022;7(9):796-829.

Gill U, Peppa D, Micco L, Singh H, Carey I, et al. Interferon alpha induces sustained changes in NK cell responsiveness to hepatitis B viral load suppression in vivo. PLoS Pathog. 2016; Aug 3;12(8):e1005788.

Hu P, et al. HBsAg Loss with Peg-interferon Alfa-2a in Hepatitis B Patients with Partial Response to Nucleos(t)ide Analog: New Switch Study. J Clin Transl Hepatol 2018 Mar 28;6(1):25-34.

International Committee of Medical Journals Editors (ICMJE). Recommendations for the Conduct, Reporting, Editing and Publication of Scholarly Work in Medical Journals. http://www.icmje.org. Updated May 2022. Accessed December 3, 2022.

Ivashkiv LB, Donlin LT. Nat Rev Immunol. 2014 Jan;14(1):36-49.

Janssen HL, et al. Pegylated interferon alfa-2b alone or in combination with lamivudine for HBeAg-positive chronic hepatitis B: a randomized trial. Lancet 2005;365:123–129.

Jones HE, Ohlssen DI, Neuenschwander B, Racine A, Branson M. Bayesian models for subgroup analysis in clinical trials. Clin Trials 2011;8:129–143.

Kondo Y, Ninomiya M, Kakazu E, Kimura O, Shimosegawa T. Hepatitis B surface antigen could contribute to the immunopathogenesis of hepatitis B virus infection. ISRN Gastroenterol. 2013;2013:935295.

Konerman MA, Lok AS. Interferon Treatment for Hepatitis B. Clinics in Liver Disease. 2016; 20(4): 645-665.

Lau GK, et al. Peginterferon Alfa-2a, lamivudine, and the combination for HBeAg-positive chronic hepatitis B. N Engl J Med 2005;352:2682–2695.

Lebossé F, Testoni B, Fresquet J, Facchetti F, Galmozzi E, Fournier M, Hervieu V, Berthillon P, Berby F, Bordes I, Durantel D, Levrero M, Lampertico P, Zoulim F. Intrahepatic innate immune response pathways are downregulated in untreated chronic hepatitis B. J Hepatol. 2017;66(5):897-909.

Liaw YF, Kao JH, Piratvisuth T, et al. Asian-Pacific consensus statement on the management of chronic hepatitis B: a 2012 update. Hepatol Int 2012;6:531-61.

Lim SG, Baumert TF, Boni C, Gane E, Levrero M, et al.The scientific basis of combination therapy for chronic hepatitis B functional cure. Nat Rev Gastroenterol Hepatol. 2023; Jan 11. doi: 10.1038/s41575-022-00724-5.

Liu, S., Peng, N., Xie, J., Hao, Q., Zhang, M., Zhang, Y., et al. Human hepatitis B virus surface and e antigens inhibit major vault protein signaling in interferon induction pathways. J. Hepatol. 2015; 62, 1015–1023.

Lok AS, McMahon BJ, Brown RS, Wong JB, Ahmed AT, Farah W, Almasri J, Alahdab F, Benkhadra K, Mouchli MA, Singh S, Mohamed EA, Abu Dabrh AM, Prokop LJ, Wang Z, Murad MH, Mohammed K. Antiviral therapy for chronic hepatitis B viral infection in adults: A systematic review and meta-analysis. Hepatology 2016; 63(1):284-306.

Luangsay, S., Gruffaz, M., Isorce, N., Testoni, B., Michelet, M., Faure-Dupuy, S., et al. Early inhibition of hepatocyte innate responses by hepatitis B virus. J. Hepatol. 2015; 63, 1314–1322.

Marcellin P, et al. Peginterferon alfa-2aalone, lamivudine alone, and the two in combination in patients with HBeAg-negative chronic hepatitis B. N Engl J Med 2004;351:1206–1217.

Micco L, Peppa D, Loggi E, Schurich A, Jefferson L, Cursaro C, et al. Differential boosting of innate and adaptive antiviral responses during pegylated-interferon-alpha therapy of chronic hepatitis B. J Hepatol. 2013;58(2):225-33.

Michler, T., A. D. Kosinska, J. Festag, T. Bunse, J. Su, M. Ringelhan, H. Imhof, D. Grimm, K. Steiger, C. Mogler, M. Heikenwalder, M. L. Michel, C. A. Guzman, S. Milstein, L. Sepp-Lorenzino, P. Knolle and U. Protzer (2020). "Knockdown of Virus Antigen Expression Increases Therapeutic Vaccine Efficacy in High-Titer Hepatitis B Virus Carrier Mic. e." Gastroenterology 158(6): 1762-1775 e1769.

Ning Q, Han M, Sun Y, Jiang J, Tan D, Hou J, Tang H, Sheng J, Zhao M. Switching from entecavir to PegIFN alfa-2a in patients with HBeAg-positive chronic hepatitis B: a

randomised open-label trial (OSST trial). Randomized Controlled Trial J Hepatol. 2014 Oct;61(4):777-84.

PEGASYS® (peginterferon alfa-2a) [US package insert]. South San Francisco, CA: Genentech, Inc.; 2017.

Polaris Observatory Collaborators. HBV progress towards coverage targets. https: //cdafound.org/polaris-countries-dashboard.

Revill PA, Penicaud C, Brechot C, et al. Meeting the Challenge of Eliminating Chronic Hepatitis B Infection. Genes (Basel). 2019;10(4).

Rijckborst V, Janssen HL. The role of interferon in hepatitis B therapy. Curr Hepat Rep. 2010;9(4):231-238.

Scaglione SJ, Lok AS. Effectiveness of hepatitis B treatment in clinical practice. Gastroenterology 2012; 142:1360-1368.

Stanaway JD, Flaxman AD, Naghavi M, Fitzmaurice C, Vos T, Abubakar I, Abu-Raddad LJ, Assadi R, Bhala N, Cowie B, Forouzanfour MH, Groeger J, Hanafiah KM, Jacobsen KH, James SL, MacLachlan J, Malekzadeh R, Martin NK, Mokdad AA, Mokdad AH, Murray CJL, Plass D, Rana S, Rein DB, Richardus JH, Sanabria J, Saylan M, Shahraz S, So S, Vlassov V, Weiderpass E, Wiersma ST, Younis M, Yu C, Zaki M, Cooke GS. The global burden of viral hepatitis from 1990 to 2013: findings from the Global Burden of Disease Study 2013. Lancet 2016; 388(10049): 1081-1088.

Terrault NA, Bzowej NH, Chang KM, et al. AASLD guidelines for treatment of chronic hepatitis B. Hepatology 2016;63:261-83.

Tout I, Loureiro D, Mansouri A, Soumelis V, Boyer N, et al. Hepatitis B surface antigen seroclearance: Immune mechanisms, clinical impact, importance for drug development. J Hepatol.2020 Aug;73(2):409-422.

Trepo CHL, Chan, Lok A. Hepatitis B virus infection. Lancet 2014;384(9959): 2053-2063.

Tsunematsu, S., Suda, G., Yamasaki, K., Kimura, M., Izumi, T., Umemura, M., et al. Hepatitis B virus X protein impairs alpha-interferon signaling via upregulation of suppressor of cytokine signaling 3 and protein phosphatase 2A. J. Med. Virol. 2017; 89, 267–275.

Wang, H., and Ryu, W. S. Hepatitis B virus polymerase blocks pattern recognition receptor signaling via interaction with DDX3: implications for immune evasion. PLoS Pathog. 2010; 6:e1000986.

Wang S, Chen Z, Hu C, Qian F, Cheng Y, Wu M, Shi B, Chen J, Hu Y, Yuan Z. Hepatitis B virus surface antigen selectively inhibits TLR2 ligand-induced IL-12 production in monocytes/macrophages by interfering with JNK activation. J Immunol. 2013 May 15;190(10):5142-51.

WHO. Hepatitis B. 2021. https://www.who.int/news-room/fact-sheets/detail/hepatitis-b.

Wong GLH, Gane E, Lok ASF. How to achieve functional cure of HBV: Stopping NUCs, adding interferon or new drug development? J Hepatol. 2022 Jun;76(6):1249-1262.

Xu, F., Song, H., Li, N., and Tan, G. HBsAg blocks TYPE I IFN induced up-regulation of A3G through inhibition of STAT3. Biochem. Biophys. Res. Commun. 2016; 473, 219–223.

Yuen M-F, Lim S-G, Plesniak R, et al. Efficacy and safety of bepirovirsen in chronic hepatitis B infection. N Engl J Med. DOI: 10.1056/NEJMoa2210027.

Yuen MF, Lim YS, Cloutier D, et al., Preliminary 48-week safety and efficacy data for VIR-2218 alone and in combination with pegylated interferon alfa in participants with chronic HBV infection. AASLD, The Liver Meeting 4-8 November 2022.

Yuen MF, Wong GL, Douglas M, et al. Preliminary safety and efficacy of the combination therapy of BRII-835 (VIR-2218) and BRII-179 (VBI-2601) treating chronic HBV injection. APASL Annual Meeting 15-19 February 2023.

Zhu D, Liu L, Yang D, Fu S, Bian Y, Sun Z, He J, Su L, Zhang L, Peng H, Fu YX. J Immunol.2016 Apr 1;196(7):3079-87.

Zoulim F, Durantel D. Antiviral therapies and prospects for a cure of chronic hepatitis B. Cold Spring Harb Perspect Med. 2015;5(4).

11. APPENDICES

11.1. Appendix 1: Regulatory, Ethical, and Study Oversight Considerations

11.1.1. Regulatory and Ethical Considerations

- This study will be conducted in accordance with the protocol and with the following:
  - Consensus ethical principles derived from international guidelines including the Declaration of Helsinki and Council for International Organizations of Medical Sciences (CIOMS) International Ethical Guidelines
  - Applicable ICH Good Clinical Practice (GCP) Guidelines
  - Applicable laws and regulations
- The protocol, protocol amendments, ICF, Investigator's Brochure, and other relevant documents (e.g., advertisements) must be submitted to an IRB/IEC by the investigator and reviewed and approved by the IRB/IEC before the study is initiated.
- Any amendments to the protocol will require IRB/IEC approval before implementation of changes made to the study design, except for changes necessary to eliminate an immediate hazard to study participants.
- The investigator will be responsible for the following:
  - Providing written summaries of the status of the study to the IRB/IEC annually or more frequently in accordance with the requirements, policies, and procedures established by the IRB/IEC
  - Notifying the IRB/IEC of SAEs or other significant safety findings as required by IRB/IEC procedures
  - Providing oversight of the conduct of the study at the site and adherence to requirements of 21 CFR, ICH guidelines, the IRB/IEC, European regulation 536/2014 for clinical studies (if applicable), and all other applicable local regulations

11.1.2. Finance Disclosure

Investigators and sub-investigators will provide the sponsor with sufficient, accurate financial information as requested to allow the sponsor to submit complete and accurate financial certification or disclosure statements to the appropriate regulatory authorities. Investigators are responsible for providing information on financial interests during the course of the study and for 1 year after completion of the study.

11.1.3. Informed Consent Process

- The investigator or his/her representative will explain the nature of the study to the participant or his/her legally authorized representative and answer all questions regarding the study.
- Participants must be informed that their participation is voluntary. Participants or their legally authorized representative will be required to sign a statement of informed consent that meets the requirements of 21 CFR 50, local regulations, ICH guidelines, Health Insurance Portability and Accountability Act (HIPAA) requirements, where applicable, and the IRB/IEC or study center.
- The medical record must include a statement that written informed consent was obtained before the participant was enrolled in the study and the date the written consent was obtained. The authorized person obtaining the informed consent must also sign the ICF.
- Participants must be re-consented to the most current version of the ICF(s) during their participation in the study.
- A copy of the ICF(s) must be provided to the participant or the participant's legally authorized representative.

11.1.4. Data Protection

- Participants will be assigned a unique identifier by the sponsor. Any participant records or datasets that are transferred to the sponsor will contain the identifier only; participant names or any information which would make the participant identifiable will not be transferred.
- The participant must be informed that his/her personal study-related data will be used by the sponsor in accordance with local data protection law. The level of disclosure must also be explained to the participant who will be required to give consent for their data to be used as described in the informed consent.
- The participant must be informed that his/her medical records may be examined by Clinical Quality Assurance auditors or other authorized personnel appointed by the sponsor, by appropriate IRB/IEC members, and by inspectors from regulatory authorities.

11.1.5. Data Quality Assurance

- All relevant participant data relating to the study will be documented in source documents and appropriately recorded on CRF unless transmitted to the sponsor or designee electronically (e.g., laboratory data). The investigator is responsible for verifying that data entries are accurate and correct by physically or electronically signing the CRF.

- The investigator must maintain accurate documentation (source data) that supports the information entered in the CRF.
- The investigator must permit study-related monitoring, audits, IRB/IEC review, and regulatory agency inspections and provide direct access to source data documents.
- Monitoring details describing strategy (e.g., risk-based initiatives in operations and quality such as Risk Management and Mitigation Strategies and Analytical Risk-Based Monitoring), methods, responsibilities and requirements, including handling of noncompliance issues and monitoring techniques (central, remote, or on-site monitoring) are provided in the Monitoring Plan.
- The sponsor or designee is responsible for the data management of this study including quality checking of the data.
- The sponsor assumes accountability for actions delegated to other individuals (e.g., Contract Research Organizations).
- Study monitors will perform ongoing source data verification to confirm that data entered into the CRF by authorized site personnel are accurate, complete, and verifiable from source documents; that the safety and rights of participants are being protected; and that the study is being conducted in accordance with the currently approved protocol and any other study agreements, ICH GCP, and all applicable regulatory requirements.
- Records and documents, including signed ICFs, pertaining to the conduct of this study must be retained by the investigator. No records may be destroyed during the retention period without the written approval of the sponsor. No records may be transferred to another location or party without written notification to the sponsor.

11.1.6. Source Documents

- Source documents provide evidence for the existence of the participant and substantiate the integrity of the data collected. Source documents are filed at the investigator’s site.
- Data reported on the CRF or entered in the CRF that are transcribed from source documents must be consistent with the source documents or the discrepancies must be explained. The investigator may need to request previous medical records or transfer records, depending on the study. Also, current medical records must be available.
- Source data refer to all information recorded in the original record of certified copy of the clinical study, including clinical findings, observation results and other relevant activity records needed for reconstruction and evaluation of the clinical study.

11.1.7. Study and Site Start and Closure

The study start date is the date on which the clinical study will be open for recruitment of participants.

The sponsor designee reserves the right to close the study site or terminate the study at any time for any reason at the sole discretion of the sponsor. Study sites will be closed upon study completion. A study site is considered closed when all required documents and study supplies have been collected and a study-site closure visit has been performed.

The investigator may initiate study-site closure at any time, provided there is reasonable cause and sufficient notice is given in advance of the intended termination.

Reasons for the early closure of a study site by the sponsor or investigator may include but are not limited to:

- Failure of the investigator to comply with the protocol, the requirements of the IRB/IEC or local health authorities, the sponsor's procedures, or GCP guidelines
- Inadequate recruitment of participants by the investigator
- Discontinuation of further study intervention development

If the study is prematurely terminated or suspended, the sponsor shall promptly inform the Investigators, the IECs/IRBs, the regulatory authorities, and any contract research organization(s) used in the study of the reason for termination or suspension, as specified by the applicable regulatory requirements. The Investigator shall promptly inform the participant and should assure appropriate therapy and/or follow-up.

11.1.8. Publication Policy

The sponsor intends to publish the results of clinical trials regardless of outcome. Results may be published in peer-reviewed journals and/or presented at scientific congresses. The sponsor will comply with the requirements for publication and disclosure of clinical trial information. In accordance with standard editorial and ethical practice, the sponsor will generally support publication of multi-center studies only in their entirety and not as individual site data. Authorship will be determined by mutual agreement based upon International Committee of Medical Journal Editors criteria and guidelines (ICMJE, 2022). Individuals who do not meet authorship criteria do not warrant named authorship. Nonauthor contributors may be acknowledged in the manuscript when published or recognized in a manner consistent with the target journal policy.

11.2. Appendix 2: Clinical Safety Laboratory Tests

The tests detailed in Table 8 will be performed by the central laboratory.

The laboratory reports must be filed with the source documents. Investigators must review the laboratory report, document their review of each laboratory safety report, and record any clinically significant changes occurring during the study in the medical history section or AE section of the CRF. Clinically significant abnormal laboratory findings are those which are not associated with the underlying disease, unless judged by the investigator to be more severe than expected for the participant's condition.

Additional tests may be performed at any time during the study as determined necessary by the investigator or required by local regulations.

Table 8 Protocol-Required Safety Laboratory Assessments

**Hematology**	
Complete blood count with differential, including red blood cell count, hemoglobin, hematocrit, white blood cell count, count and percentage of neutrophils, lymphocytes, eosinophils, basophils, and monocytes, and platelet count	
**Chemistry**	
Albumin	Glucose
Calcium	Sodium
Potassium	Chloride
Creatine kinase	Carbon dioxide/bicarbonate
Blood urea nitrogen (BUN)	Estimated glomerular filtration rate (eGFR)
Creatinine	Uric acid
Alanine aminotransferase (ALT)	Aspartate aminotransferase (AST)
Gamma glutamyl transferase (GGT)	Lactate dehydrogenase (LDH)
Alkaline phosphatase (ALP)	Bilirubin (total and direct)
**Coagulation Parameters**	
International normalized ratio (INR)	Prothrombin time (PT)
**Urinalysis**	
Glucose	Proteins

Red blood cells (RBCs)	
**Pregnancy Testing** (WOCBP only) [1]	
Beta-human chorionic gonadotropin	Urine pregnancy test
**Thyroid Panel** [2]	
Thyroid stimulating hormone (TSH)	T3
T4	
**Anti-nuclear antibodies (ANA)** [2]	
**AFP** [3]	

[1] Blood Beta-human chorionic gonadotropin should be conducted at central lab at screening visit, Urine pregnancy test and blood pregnancy test (if necessary) at other time points will be performed by the local laboratory.

[2] Thyroid panel and ANA will be tested every 12 weeks during PEG-IFNα dosing: Screening, Week 12, Week 24, Week 36 and Week 48.

3 AFP will be tested every 6 months during the study: Screening, Week 24, Week 48, Week 72 and NRTI Discontinuation Monitoring Period Week 24

11.3. Appendix 3 Assessments in Participants Meeting Laboratory ECI Criteria

Additional assessment will be performed in participants who experience ALT elevations meeting laboratory ECI criteria (Section 8.3.7) from Day 1 of dosing to the last follow-up visit. Following the occurrence of event of ECI, the following assessments in Table 9 will be performed, as soon as possible, and no later than one week after the initial ALT value meeting criteria for a laboratory ECI.

The optional assessments in Table 10 may be conducted per investigator discretion as needed. Extended hepatic panel will be tested at central laboratory. Imaging will be performed and reviewed at the investigational site(s) by certified site staff.

Table 9 Assessments in Participants Meeting Laboratory ECI Criteria

**Hematology**	
Complete blood count with differential, including red blood cell count, hemoglobin, hematocrit, white blood cell count, count and percentage of neutrophils, lymphocytes, eosinophils, basophils, and monocytes, and platelet count	
**Chemistry**	
Albumin	Glucose
Calcium	Sodium
Potassium	Chloride
Creatine kinase	Carbon dioxide/bicarbonate
Blood urea nitrogen (BUN)	Estimated glomerular filtration rate (eGFR)
Creatinine	Uric acid
Alanine aminotransferase (ALT)	Aspartate aminotransferase (AST)
Gamma glutamyl transferase (GGT)	Lactate dehydrogenase (LDH)
Alkaline phosphatase (ALP)	Bilirubin (total and direct)
**Coagulation Parameters**	
International normalized ratio (INR)	Prothrombin time (PT)
**Focused Medical History**	
Use of any potentially hepatotoxic concomitant medications, including over the counter medications and herbal remedies	Alcohol consumption and drugs of abuse
Exposure to other potentially hepatotoxic agents including any work-related exposures (eg. solvents, pesticides, etc.)	Exercise history

Table 10 Optional Assessments in Participants Meeting Laboratory ECI Criteria

**Extended Hepatic Panel**	
Quantitative HBsAg	Ferritin
Quantitative HBV DNA	Blood phosphatidylethanol
Qualitative HBeAg	Urinary ethyglucuronide

HAV antibody IgM	Anti-nuclear antibodies
HCV antibody and HCV RNA	Anti-smooth muscle antibodies
HDV antibody or RNA	Anti-LKM1 antibody
HEV antibody IgM	Anti-mitochondrial antibodies
Epstein-Barr Virus antibodies, IgM and IgG	Cytomegalovirus antibodies, IgM and IgG
Ceruloplasmin	
**Imaging**	
Abdominal ultrasound, including Doppler flow, if applicable; CT or MRI acceptable in place of ultrasound if clinically indicated	

CT = computed tomography; HAV = hepatitis A virus; HCV = hepatitis C virus; HDV = hepatitis D virus; HEV = hepatitis E virus; IgG = immunoglobulin G antibody; IgM = immunoglobulin M antibody; LKM1 = liver/kidney microsome-1 antibody; MRI = magnetic resonance imaging; RNA = ribonucleic acid; SLA = soluble liver antigen.

11.4. Appendix 4: Contraceptive Requirements and Collection and Reporting of Pregnancy Information

Contraceptive Requirements:

WOCBP may be included in this study and include any female participant who has experienced menarche and who is not post-menopausal or permanently sterilized (eg, bilateral tubal occlusion, hysterectomy, bilateral oophorectomy, or bilateral salpingectomy).

WOCBP must be willing to use highly effective methods of contraception 14 days before dose, throughout study participation, and for 48 weeks after last dose of study drug administration. Highly effective methods of birth control result in a low failure rate (ie, less than 1% per year). Birth control methods which are considered highly effective include:

- Established use of combined (estrogen and progestogen containing) oral, intravaginal, or transdermal hormonal methods of contraception associated with inhibition of ovulation **OR** established use of progestogen-only oral, injectable, or implantable hormonal methods of contraception associated with inhibition of ovulation. It is not currently known whether study intervention will impact the effectiveness of hormonal contraceptive methods; therefore, it is recommended to use an additional form of contraception (ie, barrier method) throughout the study and for 48 weeks after last study drug administration.
- Placement of an intrauterine device
- Placement of an intrauterine hormone-releasing system
- Surgical sterilization of male partner (with the appropriate post-vasectomy documentation of the absence of sperm in the ejaculate; for female participants on the study, the vasectomized male partner should be the sole partner for that participant)
- True sexual abstinence from heterosexual contact, when in line with the preferred and usual lifestyle of the participant. Periodic abstinence (e.g., calendar, ovulation, symptothermal, postovulation methods) and withdrawal are not acceptable methods of contraception. Abstinent participants have to agree to use 1 of the above-mentioned contraceptive methods, if they start sexual heterosexual relationships during the study and for up to 48 weeks after last dose of study drug administration
- Barrier method in combination with hormonal contraceptive, as described above

Post-menopausal status is defined as 12 months with no menses without an alternative medical cause. A high FSH level in the post-menopausal range should be used to confirm a post-menopausal state in women not using hormonal contraception or hormone replacement therapy (HRT). In the absence of 12 months of amenorrhea, confirmation with more than one FSH measurement is required.

Females on HRT and whose menopausal status is in doubt must discontinue HRT to allow confirmation of postmenopausal status before study enrollment.

Female participants must also refrain from egg donation and in vitro fertilization from the time of study treatment administration until 48 weeks after study drug administration.

Male participants with female partners of child-bearing potential must agree to meet 1 of the following contraception requirements from the time of study treatment administration until the last follow-up visit.

- Vasectomy with documentation of azoospermia
- Male condom plus partner use of 1 of the contraceptive options listed above for contraception for WOCBP (hormonal contraceptive, intrauterine device)

Male participants must also agree not to donate sperm for the 48 weeks following last study drug administration.

TITLE

Protocol Title: A Phase 2 Multicenter, Randomized, Open-label Study to Investigate the Efficacy and Safety of BRII-835 (VIR-2218) and Pegylated Interferon Alpha (PEG-IFNα) Combination Therapy for the Treatment of Chronic Hepatitis B Virus (HBV) Infection

Protocol Number: BRII-835-002

Compound: BRII-835 (VIR-2218)

Study Phase: 2

Short Title: Study to Investigate the Efficacy and Safety of BRII-835 and PEG-IFNα Combination Therapy Treating Chronic HBV Infection

Sponsor Name: Brii Biosciences Limited

Co-Sponsor/Registration Agent: Brii Biosciences (Beijing) Co. Limited

Legal Registered Address: Room 301, 3/F, Building 7, No. 1 Yongtaizhuang North Road, Tiandilinfeng (Dongsheng Area), Haidian District, Beijing 100192, China

Protocol Version: 2.0

Version Date: 31 August 2023

Information contained in this document is proprietary to Brii Biosciences Limited. The information is provided to you in confidence which is requested under an agreed upon and signed Confidentiality and Disclosure Agreement. Do not give this document or any copy of it or reveal any proprietary information contained in it to any third party (other than those in your organization who are assisting you in this work and are bound by the Confidentiality and Disclosure Agreement) without the prior written permission of an authorized representative of Brii Biosciences Limited.

SPONSOR SIGNATURE PAGE

Sponsor's Authorized Officer:

[redacted]

INVESTIGATOR SIGNATURE PAGE

For protocol number BRII-835-002

I confirm agreement to conduct the study in compliance with the protocol, *International Conference on Harmonisation Good Clinical Practices (ICH GCP)* as well as applicable local regulatory and ethical requirements.

I acknowledge that I am responsible for overall study conduct. I agree to personally conduct or supervise the described clinical study.

I agree to ensure that all associates, colleagues and employees assisting in the conduct of the study are informed about their obligations. Mechanisms are in place to ensure that site staff receive the appropriate information throughout study.

Investigator Name (Printed): ____

____ ______________________

Investigator Signature **Date (dd/mm/yyyy)**

PROTOCOL AMENDMENT SUMMARY OF CHANGES

DOCUMENT HISTORY	
**Document**	**Date**
Amendment 1 (Version 2.0)	31 August 2023
Original Protocol (Version 1.0)	31 March 2023

Amendment 1 (Version 2.0) 31 August 2023

Overall Rationale for the Amendment

The emerging clinical data showed that high anti-HBs titers in participants with chronic HBV infection at the end of treatment (EOT) of BRII-835+PEG-IFNα combination therapy may predict the sustainability of HBsAg seroclearance at 24 weeks post EOT (Yuen, 2023). The presence of HBsAb-secreting specific B cells in peripheral blood of chronic hepatitis B (CHB) patients may also be an important immunological indicator to predict interferon treatment efficacy (Geng, 2023), suggesting the potential value of humoral response for functional cure. BRII-179, a recombinant virus-like particle (VLP)-based protein immunotherapeutic, is safe and well-tolerated in phase 1b/2a BRII-179-001 and phase 2 BRII-179-835-001 studies, in which robust anti-HBs responses were induced in a proportion of patients receiving BRII-179 or BRII-179 in combination with BRII-835, an siRNA (Ma, 2021; Yuen, 2023). It is hypothesized that patients who responded to BRII-179, especially those with higher anti-HBs titers, may have a less dysfunctional immunological profile to virus and be able to respond better to curative therapies, e.g., BRII-835+PEG-IFNα. This amendment would allow eligible participants who received BRII-179 from BRII-179-835-001 to roll over to this study and evaluate the efficacy of BRII-835+PEG-IFNα combination.

Summary of Changes

Section # and Name	Description of Change	Brief Rationale
Section 1.2 Schema	Added study schema for Cohort 4 (BRII-179-835-001 extension cohort)	Provided information for the eligible participants from BRII-179-835-001 study to roll over to the new cohort
Section 1.3.1 SoA, Dosing and Follow-up Period	Added study procedures for Cohort 4;<br><br>Clarified study visit names during Follow-up Period;<br><br>Clarified PK/ADA [redacted] sample collections;	Provided information for the eligible participants from BRII-179-835-001 study to roll over to the new cohort<br><br>Provided clarifications to study visit schedules and sample collections

Section # and Name	Description of Change	Brief Rationale
	Clarified requirements for completion of dosing card, urine pregnancy test, and NRTI adherence review.	
Section 1.3.2 SoA, NRTI Discontinuation Monitoring Period	Added HBeAg monitoring during NRTI discontinuation monitoring period	Added more stringent safety monitoring measures after NRTI discontinuation
Section 2.2.2 Summary of Clinical Experience	Updated latest clinical data for BRII-835	Provided updates on the latest clinical experience of BRII-835
Section 2.4 Rationale for BRII-835 and PEG-IFNα Combination Therapy for the Treatment of Chronic HBV Infection	Updated the rationale of the new cohort based on the latest clinical data from the ongoing studies	Provided the scientific background and rationale adding the new cohort
Section 4.1 Overall Design	Added description of the design and dosing regimen for Cohort 4	Provided information for the eligible participants from BRII-179-835-001 study to roll over to the new cohort
Section 4.2 Scientific Rationale for Study Design<br><br>Section 4.3 Justification of Dose	Provided the scientific rationale for subjects from BRII-179-835-001 to roll over to Cohort 4	Provided the scientific background and rationale adding the new cohort, and dose justification for the new cohort
Section 5.1 Inclusion Criteria and 5.2	Added inclusion criteria for Cohort 4; clarified the exclusion criteria for Cohorts 1-3;	Provided information for the eligible participants from BRII-179-835-001 study to roll over to the new cohort

Section # and Name	Description of Change	Brief Rationale
Exclusion Criteria	Clarified alcohol abuse exclusion criterion.	
Section 6.1 Study Drugs<br>Section 6.2 Randomization and Blinding<br>Section 6.3 Dose Adjustment Criteria	Incorporated changes corresponding to Cohort 4	Provided information for the eligible participants from BRII-179-835-001 study to roll over to the new cohort
Section 6.6.2 NRTI Treatment Compliance	Clarified NRTI adherence requirement	Provided clarifications to study procedures
Section 8.1 Schedule of Assessments	Updated and clarified the study procedures corresponding to Cohort 4	Provided information for the eligible participants from BRII-179-835-001 study to roll over to the new cohort
Section 8.5 Pharmacokinetic (PK) Assessments	Clarified PK sample collection timepoints	Provided clarifications to study procedures
Section 9.2 Estimand<br>Section 9.3 Sample Size Determination<br>Section 9.4 Analysis Sets<br>Section 9.6 Statistical Analysis<br>Section 9.7 Interim Analysis	Clarified the estimands and analysis sets corresponding to Cohort 4<br>Clarified the statistical analysis in corresponding to Cohort 4	Provided clarifications to the analysis plan including Cohort 4 from statistical perspective

Section # and Name	Description of Change	Brief Rationale
Section 9.8 Final Analysis		
Section 11.2 Appendix 2 Clinical Safety Laboratory Tests	Clarified requirements for urine pregnancy test	Provided clarifications to study procedures
Section 11.3 Appendix 3 Assessments in Participants Meeting Laboratory ECI Criteria	Clarified two optional assessments would not be conducted in mainland China	Provided clarifications to study procedures

TABLE OF CONTENTS

TITLE 1
SPONSOR SIGNATURE PAGE 2
INVESTIGATOR SIGNATURE PAGE 3
PROTOCOL AMENDMENT SUMMARY OF CHANGES 4
TABLE OF CONTENTS 8
LIST OF TABLES 12
LIST OF FIGURES 12
ABBREVIATIONS 13
1. PROTOCOL SUMMARY 16
1.1. Synopsis 16
1.2. Schema 21
1.3. Schedule of Assessment (SoA) 22
1.3.1. Dosing Period and Follow-up Period 22
1.3.2. NRTI Discontinuation Monitoring Period 25
2. INTRODUCTION 27
2.1. Background 27
2.2. BRII-835 27
2.2.1. Summary of Nonclinical Experience 28
2.2.2. Summary of Clinical Experience 28
2.3. PEG-IFNα 30
2.4. Rationale for BRII-835 and PEG-IFNα Combination Therapy for the Treatment of Chronic HBV Infection 31
2.5. Benefit/Risk Assessment 31
3. STUDY OBJECTIVES AND ENDPOINTS 33
3.1. Study Objectives 33
3.1.1. Primary Objectives 33
3.1.2. Secondary Objectives 33
[redacted] 33
3.2. Study Endpoints 33
3.2.1. Primary Endpoints 33

3.2.2. Secondary Endpoints 33
[redacted] 34
4. STUDY DESIGN 35
4.1. Overall Design 35
4.1.1. Planned Cohorts and Dosing 35
4.1.2. NRTI Discontinuation 36
4.1.3. Safety Review Committee 37
4.2. Scientific Rationale for Study Design 37
4.3. Justification of Dose 39
4.4. End of Study Definition 41
5. STUDY POPULATION 42
5.1. Inclusion Criteria 42
5.2. Exclusion Criteria 44
5.3. Screening Failures 46
5.4. Lifestyle Considerations 46
6. TREATMENT OF PARTICIPANTS 47
6.1. Study Drugs 47
6.1.1. General Information of Study Drugs 47
6.1.2. Study Drug Dose and Administration 47
6.1.3. Study Drug Materials and Management 48
6.2. Randomization and Blinding 50
6.3. Dose Adjustment Criteria 50
6.3.1. Safety Criteria for PEG-IFNα Dose Adjustment 50
6.4. Background Treatment 52
6.5. Concomitant Medications 52
6.5.1. Prohibited Medications 52
6.6. Treatment Compliance 53
6.6.1. PEG-IFNα Treatment Compliance 53
6.6.2. NRTI Treatment Compliance 53
7. DISCONTINUATION OF STUDY INTERVENTION AND PARTICIPANT DISCONTINUATION/WITHDRAWAL 54
7.1. Discontinuation of Study Intervention 54

7.2. Participant Discontinuation/Withdrawal from the Study 55
7.3. Lost to Follow up 56
8. STUDY ASSESSMENT AND PROCEDURES 57
8.1. Schedule of Assessments 57
8.1.1. Screening 57
8.1.2. Day 1 57
8.1.3. Dosing Period 58
8.1.4. End of Treatment Visit 58
8.1.5. Follow-up Period 58
8.1.6. NRTI Discontinuation Monitoring Period 59
8.1.7. Unscheduled Visits 59
8.1.8. End of Study Visit 59
8.2. Efficacy Assessments 59
8.2.1. Antiviral Activities 59
8.3. Safety Assessments 60
8.3.1. Physical Examination 60
8.3.2. Vital Signs 60
8.3.3. Alcohol Intake Assessment 60
8.3.4. Liver Stiffness 60
8.3.5. Ultrasound 60
8.3.6. Laboratory Assessments 60
8.3.7. Laboratory Events of Clinical Interest 61
8.4. Adverse Events and Serious Adverse Events 62
8.4.1. Definition of Adverse Events 62
8.4.2. Assessment of Adverse Events 64
8.4.3. Time Period and Frequency for Collecting AEs and SAEs 64
8.4.4. Recording of AEs and SAEs 65
8.4.5. Follow-up of AEs and SAEs 65
8.4.6. Reporting of SAEs 66
8.4.7. Regulatory Reporting Requirements for SAEs 66
8.4.8. Special Situation Reports 66
8.5. Pharmacokinetic (PK) Assessments 68

8.6. Assessment of Anti-Drug Antibodies (ADA) to BRII-835 69
[redacted] [redacted] 69
9. STATISTICS 70
9.1. Statistical Hypotheses 70
9.2. Estimands 70
9.3. Sample Size Determination 71
9.4. Analysis Sets 73
9.5. Statistical Methods 75
9.6. Statistical Analyses 75
9.6.1. Efficacy Analysis 75
9.6.2. Safety Analysis 78
9.6.3. Pharmacokinetic Analysis 78
9.6.4. Baseline and Other Summaries and Analyses 79
9.7. Interim Analysis 79
9.8. Final Analysis 80
10. REFERENCES 81
11. APPENDICES 85
11.1. Appendix 1: Regulatory, Ethical, and Study Oversight Considerations 85
11.1.1. Regulatory and Ethical Considerations 85
11.1.2. Finance Disclosure 85
11.1.3. Informed Consent Process 86
11.1.4. Data Protection 86
11.1.5. Data Quality Assurance 86
11.1.6. Source Documents 87
11.1.7. Study and Site Start and Closure 88
11.1.8. Publication Policy 88
11.2. Appendix 2: Clinical Safety Laboratory Tests 89
11.3. Appendix 3 Assessments in Participants Meeting Laboratory ECI Criteria 91
11.4. Appendix 4: Contraceptive Requirements and Collection and Reporting of Pregnancy Information 93
11.5. Appendix 5: Protocol Amendment History 95

LIST OF TABLES

Table 1 Study Drug Dosage and Administration....47
Table 2 PEG-IFNα Dose Modification Guidelines....51
Table 3 CTCAE (V5.0) General Guideline of Severity of AEs....64
Table 4 Pharmacokinetics Assessment Timepoints....68
Table 5 95% Credible Interval of Response Rate by Sample Size for Cohorts 1-3....72
Table 6 Operational Characteristics by Sample Size....73
Table 7 Analysis Sets....74
Table 8 Protocol-Required Safety Laboratory Assessments....89
Table 9 Assessments in Participants Meeting Laboratory ECI Criteria....91
Table 10 Optional Assessments in Participants Meeting Laboratory ECI Criteria....92

LIST OF FIGURES

Figure 1: Study Design....21

ABBREVIATIONS

ADA	Anti-drug antibodies
AE	Adverse event
AFP	Alpha-fetoprotein
ALP	Alkaline phosphatase
ALT	Alanine aminotransferase
ANA	Anti-nuclear antibodies
ANC	Absolute neutrophil count
BMI	Body Mass Index
cccDNA	Covalently closed circular DNA
CHB	Chronic hepatitis B
CIOMS	Council for International Organizations of Medical Sciences
CMO	Contracted Manufacturing Organization
CRF	Case report form
DNA	Deoxyribonucleic acid
ECG	Electrocardiogram
ECI	Event of clinical interest
EOT	End of treatment
EOS	End of study
eGFR	Estimated glomerular filtration rate
EPO	Erythropoietin
FAS	Full Analysis Set
GalNAc	N-acetyl galactosamine
GCP	Good Clinical Practice
G-CSF	Granulocyte colony-stimulating factor
GGT	Gamma glutamyl transferase
GM-CSF	Granulocyte macrophage colony stimulating factor
GMP	Good Manufacturing Practice
HBeAg	Hepatitis B e-antigen
HBsAb	Hepatitis B surface antibodies
HBsAg	Hepatitis B surface antigen
HBV	Hepatitis B virus

HCV	Hepatitis C virus
HDV	Hepatitis D virus
HIV	Human immunodeficiency virus
ICF	Informed consent form
ICH	International Conference on Harmonization
ICMJE	International Committee of Medical Journal Editors
IEC	Independent Ethics Committee
IFN-α	Interferon-alpha
IMP	Investigational Medication Products
INR	International normalized ratio
IRB	Institutional Review Board
IRT	Interactive Response Technology
ISR	Injection site reaction
LLOQ	Lower limit of quantification
MedDRA	Medical Dictionary for Regulatory Activities
mITT	Modified Intent-To-Treat
NCI-CTCAE	National Cancer Institute Common Terminology Criteria for Adverse Events
NRTI	Nucleos(t)ide reverse transcriptase inhibitor
OTC	Over-the-counter
PBMC	Peripheral blood mononuclear cell
PD	Pharmacodynamic
PEG-IFNα	Pegylated interferon alpha
PK	Pharmacokinetic
PP	Per-Protocol
PT	Preferred term
RBC	Red blood cells
RNAi	RNA interference
SAE	Serious adverse event
SAP	Statistical analysis plan
SC	Subcutaneous
SD	Standard deviation
siRNA	Small interfering RNA

SoA	Schedule of assessments
SOC	System organ category
SOP	Standard operating procedure
SRC	Safety Review Committee
SUSAR	Suspected unexpected serious adverse reaction
TSH	Thyroid stimulating hormone
TEAE	Treatment emergent adverse event
ULN	Upper limit of normal
VLP	Virus-like particle
WBC	White blood cells
WOCBP	Women of child-bearing potential

1. PROTOCOL SUMMARY

1.1. Synopsis

Name of Sponsor/Company: Brii Biosciences Limited

Name of Investigational Products: BRII-835 (VIR-2218), Pegylated Interferon Alpha (PEG-IFNα)

**Protocol Number:** BRII-835-002	**Phase:** 2	**Country:** Asia and Pacific (APAC) region

Title of Study: A Phase 2 Multicenter, Randomized, Open-label Study to Investigate the Efficacy and Safety of BRII-835 (VIR-2218) and Pegylated Interferon Alpha (PEG-IFNα) Combination Therapy for the Treatment of Chronic Hepatitis B Virus (HBV) Infection

Study Investigational Site(s): Multiple sites in APAC region

Study Objectives:

Primary:

- To evaluate the efficacy and safety of BRII-835 and PEG-IFNα combination therapy in participants with chronic HBV infection

Secondary:

- To assess the antiviral activity of BRII-835 and PEG-IFNα combination therapy
- To assess BRII-835 pharmacokinetic (PK) exposure and evaluate the anti-drug antibodies (ADA) to BRII-835

[redacted]

[redacted]

[redacted]

Study Endpoints:

The primary endpoints for this study are to evaluate:

- Proportion of participants with HBV surface antigen (HBsAg) loss at end of treatment
- Proportion of participants with HBsAg loss at 24 weeks post end of treatment
- Proportion of participants with treatment-emergent adverse events (TEAEs)
- Proportion of participants with serious adverse events (SAEs)

The secondary endpoints for this study are to evaluate:

- Proportion of participants with HBsAg loss at Week 48
- Proportion of participants achieving functional cure (defined as sustained undetectable HBsAg and sustained suppression of HBV DNA [<lower limit of quantification (LLOQ)] ≥ 24 weeks after discontinuation of all treatment, including NRTI)

- Proportion of participants achieving HBsAg loss and HBV DNA < LLOQ at 24 weeks after discontinuation of all treatment, including NRTI
- Proportion of participants meeting NRTI discontinuation criteria at 24 weeks post end of treatment
- Absolute serum HBsAg and change from baseline over time through 24 weeks post end of treatment
- Nadir serum HBsAg and maximum reduction of serum HBsAg from baseline throughout 48-week treatment
- Time to achieve first serum HBsAg loss through Week 72
- Proportion of participants with Hepatitis B e antigen (HBeAg) loss and/or HBeAg seroconversion (baseline HBeAg-positive participants) at any timepoint
- Proportion of participants with HBsAg seroconversion at any timepoint
- Plasma concentrations of BRII-835 and its metabolite at selected timepoints
- The occurrence of ADA to BRII-835 and associated titer, if positive

[redacted]

- [redacted]
- [redacted]
- [redacted]

Study Design:

This study is designed to evaluate the efficacy and safety of BRII-835 and PEG-IFNα combination therapy in participants with chronic HBV infection without cirrhosis receiving NRTI therapy as background therapy.

Cohorts 1-3 (BRII-179 naïve):

A total of up to 75 participants with HBeAg negative or HBeAg positive chronic HBV infection will be randomized at 1:1:1 to the following three cohorts:

- Cohort 1: participants will receive 48 doses of PEG-IFNα, each administered every week starting on Day 1.
- Cohort 2: participants will receive 48 doses of PEG-IFNα, each administered every week starting on Day 1 and 13 doses of 200 mg BRII-835, each administered every 4 weeks starting on Day 1.
- Cohort 3: participants will receive 48 doses of PEG-IFNα, each administered every week starting on Day 1 and 13 doses of 100 mg BRII-835, each administered every 4 weeks starting on Day 1.

<u>**Cohort 4 (BRII-179 treated):**</u>

Participants who completed BRII-179-835-001 study in APAC region (did not include mainland China), previously treated with BRII-179 and did not achieve HBsAg seroclearance, will be eligible to roll over to Cohort 4. Up to 45 participants will be enrolled in the following cohort:

- Cohort 4: participants will receive 48 doses of PEG-IFNα, each administered every week starting on Day 1 and 13 doses of 100 mg BRII-835, each administered every 4 weeks starting on Day 1.

NRTI discontinuation:

Participants who meet all of the following NRTI discontinuation criteria for at least two consecutive visits at Week 72 will be eligible to discontinue from NRTI therapy:

- HBsAg < LLOQ and
- HBV DNA < LLOQ and
- Undetectable HBeAg and
- ALT ≤ 2x ULN

Investigator should confirm participants' eligibility as early as possible within 2 weeks post Week 72 visit. Participants who discontinue NRTI will enter the NRTI Discontinuation Monitoring Period of the study within 1 week of the investigator confirming eligibility. Participants will be followed in the NRTI Discontinuation Monitoring Period for an additional 24 weeks.

Participants who do not meet the criteria for NRTI discontinuation will continue NRTI therapy and will end the study at Week 72.

An SRC for this study will monitor participant safety and consider study-specific data as well as relevant background information about the study drugs and target population under study to inform on study conduct. The SRC charter outlining the member composition and operating procedures will be developed before the study starts.

Number of Participants: Up to 75 participants are planned to be enrolled in Cohorts 1-3, and up to 45 participants will be enrolled in Cohort 4 (BRII-179-835-001 extension cohort).

Randomization and Stratification:

Eligible participants for Cohorts 1-3 will be randomized 1:1:1 to one of the three cohorts and stratified by HBsAg level at screening (> 100 to ≤ 500 IU/mL, > 500 to ≤ 1,500 IU/mL, and > 1,500 to ≤ 3,000 IU/ml). Maximum number of participants allowed in each stratum of each cohort will be capped as six in the Interactive Response Technology (IRT) initial system set up for the first enrolled 45 participants and the limitation may be adjusted based on enrollment status.

Randomization does not apply to Cohort 4, the BRII-179-835-001 extension cohort.

Main Criteria for Inclusion:

Cohorts 1-3 (BRII-179 naïve):

Participants aged 18 to 60 years with chronic HBV infection without cirrhosis, effectively treated with NRTI therapy for at least 6 months, with HBV DNA < LLOQ and HBsAg > 100 IU/mL and ≤ 3,000 IU/mL at screening.

Cohort 4 (BRII-179 treated):

Participants who completed BRII-179 treatment, did not achieve HBsAg seroclearance in BRII-179-835-001 study, are continuing NRTI therapy and have HBV DNA < LLOQ at screening.

Investigational Product, Dosage and Mode of Administration:

BRII-835 will be supplied as a sterile solution for subcutaneous (SC) injection at a free acid concentration of 200 mg/mL.<br>PEG-IFNα will be supplied as a sterile, preservative-free solution for SC injection.<br>Cohort 1: PEG-IFNα 180 mcg administered by SC injection every week<br>Cohort 2: PEG-IFNα 180 mcg administered by SC injection every week and 200 mg BRII-835 administered by SC injection every four weeks<br>Cohort 3 and Cohort 4: PEG-IFNα 180 mcg administered by SC injection every week and 100 mg BRII-835 administered by SC injection every four weeks
**Duration of the Study:**<br>Estimated total duration for each participant will include the following: screening up to 4 weeks, dosing period 48 weeks, and a 24-week follow-up period after the last dose of the study drug. Eligible participants that meet NRTI discontinuation criteria (Section 4.1.2.1) may enter a NRTI discontinuation monitoring period for an additional 24 weeks from the day of NRTI discontinuation.
**Sample Size Determination:**<br>Up to 75 participants will be randomly assigned in 1:1:1 ratio among Cohorts 1-3. A Bayesian framework was used to calculate the sample size. It is assumed that the number of responders follows a Binomial distribution, with a weakly informative prior (Beta (0.5, 0.5)) for the true response rate. Up to 45 participants will be enrolled in Cohort 4.<br>**Primary Efficacy Analysis:**<br>The primary endpoints are 1) the Proportion of participants with HBsAg loss at end of treatment and 2) Proportion of participants with HBsAg loss at 24 weeks post end of treatment.<br>A Bayesian hierarchical model (Yuen, 2022; Jones, 2011) as primary analysis will be used to estimate the posterior probability of response rate for each cohort of Cohorts 1-3 by incorporating the baseline HBsAg level stratification factor. The point estimate of the primary outcome and 95% equal-tailed credible interval will be drawn from the model.<br>In addition, for Cohorts 1-3 combine some two adjacent baseline stratification levels to reduce HBsAg stratification factors from three levels to two levels (HBsAg >100 IU/mL and HBsAg ≤1,500 IU/mL versus HBsAg >1,500 IU/mL and HBsAg ≤3,000IU/mL; HBsAg >100 IU/mL and HBsAg ≤500 IU/mL versus HBsAg >500 IU/mL and HBsAg ≤3,000 IU/mL) to perform the stratified Bayesian hierarchical analysis if model is converged using the similar approach as the primary analysis.<br>For Cohort 4 descriptive statistics will be reported, and comparisons with other cohorts may be conducted.<br>The primary efficacy analysis will be performed within Modified Intent-To-Treat (mITT) analysis set and a supplemental analysis based on Per-Protocol (PP) analysis set with a similar analysis will be presented.<br>**Safety Analysis:**<br>The Safety Population is defined as all participants who receive injection of any study medication. Participants will be classified and summarized by treatment received. Safety will be assessed using AEs, SAE, vital signs, clinical laboratory tests with clinician assessment, and concomitant medication data. The incidence of TEAEs will be summarized by Medical Dictionary for Regulatory Activities

(MedDRA) System Organ Class (SOC) and preferred term (PT), summaries will be provided by severity and by causality (related, not related) to study drug.

Pharmacokinetic Analysis:

The Pharmacokinetic (PK) population is defined as all participants who received BRII-835, have at least one evaluable plasma BRII-835 concentration, and have no protocol deviation thought to impact on the PK data analysis. Plasma concentrations of BRII-835 and its metabolite will be listed for each participant and descriptively summarized, including n, mean, standard deviation (SD), median, minimum, maximum, geometric mean, and geometric coefficient of variation (CV), by treatment cohort and timepoints using PK population.

The Occurrence of ADA to BRII-835 and Associated Titer, if Positive.

Descriptive statistics for the endpoint will be performed based on ADA analysis set.

Interim Analysis and Summaries

Three interim analyses are planned. The first two interim analyses will be conducted when approximately 45 enrolled participants of Cohorts 1-3 complete the 48-week treatment and last available participant of all enrolled participants (Cohorts 1-4) completes the 48-week treatment, respectively. If the pace of enrollment is robust, and the primary endpoint proportion of participants with HBsAg loss at Week 48 is expected to occur less or equal to two months between the two interim analyses, the two analyses may be collapsed into one analysis.

The third interim analysis will be conducted when last available participant of Cohorts 1-3 completes follow-up 24 weeks post end of treatment.

Final Analysis

The final analysis will be performed when last available participant of Cohorts 1-4 completes the last scheduled visit according to the SoA.

Further details will be provided in the Statistical Analysis Plan.

1.2. Schema

Figure 1: Study Design

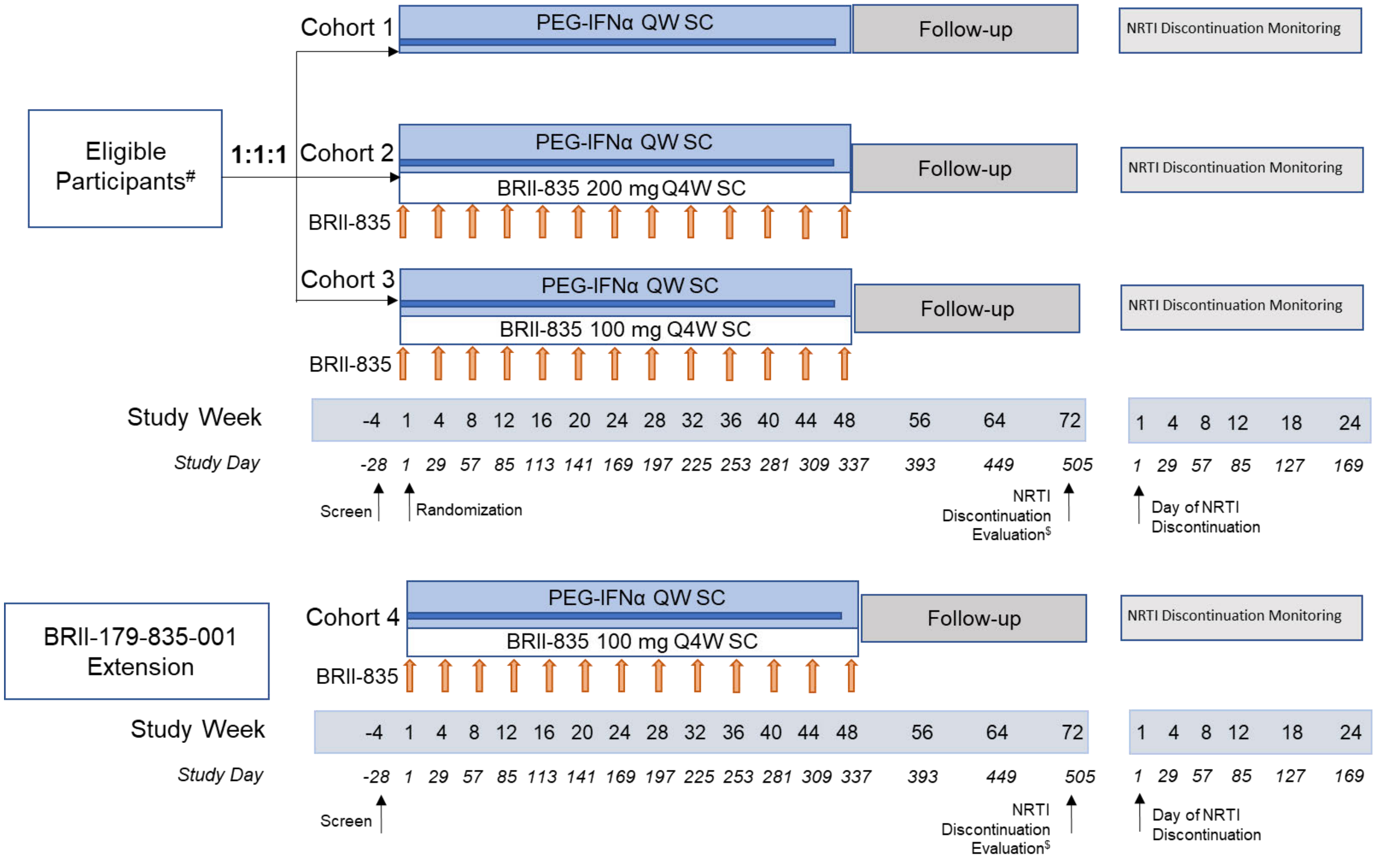

QW: every week; Q4W: every 4 weeks; SC: subcutaneous injection
Participants enrolled in Cohorts 1-3 will be stratified by HBsAg levels at screening
$ Participants meeting the NRTI discontinuation criteria may enter into the NRTI Discontinuation Monitoring Period for an additional 24 weeks. Participants that do not meet the NRTI discontinuation criteria will continue NRTI therapy and end from the study

1.3. Schedule of Assessment (SoA)

1.3.1. Dosing Period and Follow-up Period

Study Stage	Screen	Dosing Period [a, b]														Follow-up Period		
**Study Visit Week**			W2	W4	W8	W12	W16	W20	W24	W28	W32	W36	W40	W44	W48 /EOT	W56 (FU1)	W64 (FU2)	W72 (FU3)/ EOS[c]
**Study Visit Day ± Visit Window**	D -28 to -1	D1	D15 ± 3	D29 ± 3	D57 ± 3	D85 ± 3	D113 ± 3	D141 ± 3	D169 ± 3	D197 ± 3	D225 ± 3	D253 ± 3	D281 ± 3	D309 ± 3	D337 ± 3	D393 ± 5	D449 ± 5	D505 ± 7
Informed consent form (ICF)	X																	
Inclusion/exclusion criteria	X	X																
Demography	X																	
Medical history including HBV genotype [d]	X	X																
Full physical exam [e]	X					X			X						X			X
Symptom-directed physical exam		X	X	X	X		X	X		X	X	X	X	X		X	X	
Abdominal ultrasound	X								X						X			X
FibroScan	X								X						X			X
Alcohol intake assessment	X	X	X	X	X	X	X	X	X	X	X	X	X	X	X	X	X	X
Participant instructed to complete dosing card		X	X	X	X	X	X	X	X	X	X	X	X	X				
Vital signs [f]	X	X													X			X
12-lead ECG [g]	X																	
Urinalysis	X	X				X			X			X			X			X
Screening serology panel [h]	X																	
Pregnancy test [i]	X	X	X	X	X	X	X	X	X	X	X	X	X	X	X			X
AFP	X								X						X			X
TSH, T3, T4, ANA	X					X			X			X			X			
Hematology, Chemistry, and Coagulation assessments [j]	X	X	X	X	X	X	X	X	X	X	X	X	X	X	X	X	X	X
Randomization[k]		X																
BRII-835 administration [l]		X		X	X	X	X	X	X	X	X	X	X	X	X			
PEG-IFNα administration [m]		←- - - - - - - - - - - - - - X - - - - - - - - - - - - - -→																

Blood sample for BRII-835 PK analysis [n]		X									X				X			
Blood sample for ADA to BRII-835[n]		X									X				X			
Blood sample for quantitative HBsAg	X	X		X	X	X	X	X	X	X	X	X	X	X	X	X	X	X
Blood sample for quantitative anti-HBs	X	X					X		X		X		X		X	X	X	X
Blood sample for quantitative HBV DNA [o]	X	X				X			X			X			X	X		X
Blood sample for qualitative HBeAg and anti-HBe		X[u]					X[u]				X[u]				X[u]			X[u]
[redacted]		[redacted]		[redacted]					[redacted]						[redacted]			[redacted]
[redacted]		[redacted]				[redacted]			[redacted]						[redacted]			[redacted]
[redacted]		[redacted]																
AE assessment [r]	←-----------X-----------→																	
Concomitant medications review	←-----------X-----------→																	
PEG-IFNα medication adherence review [s]		←-----------X-----------→																
NRTI medication adherence review [s]	←-----------X-----------→																	
ECI surveillance [t]		←-----------X-----------→																

AE = adverse event; AFP = alpha- fetoprotein; ANA = anti-nuclear antibodies; anti-HBs = hepatitis B surface antibody; ECG = electrocardiogram; ECI = events of clinical interest; EOT=end of treatment; EOS= end of study; HBeAg = hepatitis B e-antigen; anti-HBe = hepatitis B e-antibody; HBsAg = hepatitis B surface antigen; HBV = Hepatitis B Virus; NRTI = nucleos(t)ide reverse transcriptase inhibitor; ULN = upper limit of normal

a Assessments will be performed pre-dose unless otherwise specified.

b If a participant permanently discontinues treatment prior to Week 48, EOT assessments should be performed within 14 days post the last dose of study drugs (see Section 7.1) and continue the visit in follow-up period (see Section 8.1.5).

c End of Study (EOS) visit must be conducted within 14 days when a participant prematurely discontinues from the study, unless the participant withdraws consent for study participation or is lost to follow-up.

d A medical history for at least 24 months prior to screening will be taken, including HBV genotype if available.

e Height and weight will be recorded at screening. Body mass index (BMI) will be calculated based on the measurements. Only weight will be collected afterwards.

f Vital signs (blood pressure, pulse rate, respiratory rate, and temperature) should be measured after the participant has rested comfortably for approximately 10 minutes.

g 12-lead ECG should be measured in the supine position after the participant has rested comfortably for approximately10 minutes.

h Screening serology panel includes anti-HCV, anti-HDV, and anti-HIV.

i Women of childbearing potential are required to have pregnancy tests. Negative pregnancy test must be confirmed prior to study drug administration. Blood pregnancy test should be conducted at central lab at screening visit, and urine pregnancy test will be conducted after screening. Urine and/or blood pregnancy tests may be conducted at local laboratory if necessary.

j Additional safety laboratory assessments may be performed at any time during the study as determined necessary by the investigator (not limited to safety assessments listed in protocol, local laboratory assessment is acceptable).

k Eligible participants of Cohorts 1-3 will be randomized 1:1:1 to each of the three cohorts within 48 hours prior to study drug administration on Day 1. Randomization does not apply to participants enrolled in Cohort 4.

l BRII-835 will be given to those who are assigned to Cohort 2 at 200 mg every 4 weeks, and to those who are assigned to Cohorts 3 or 4 at 100 mg every 4 weeks. Please refer to Section 6.1.3.4.

m PEG-IFNα will be given to all participants at 180 mcg every week for 48 doses. The participants will be instructed to self-inject PEG-IFNα on the same day of each week and dose modification may be considered given certain medical condition. Please refer to Section 6.1.3.4.

n BRII-835 PK and ADA samples will be collected in participants assigned to Cohorts 2 and 3 (referred to Section 8.5). Predose PK sampling should be conducted ≤ 2 hours prior to dosing. Postdose PK sampling should be conducted between ≥ 2 hours and ≤ 12 hours after dosing.

o If a participant experiences alanine aminotransferase (ALT) > 3x ULN or HBV DNA ≥ 200 IU/mL, additional HBV DNA quantitation sample(s) will be collected at the next scheduled visit.

[redacted]

r AEs will be collected from signing the ICF until last visit. After informed consent has been obtained but prior to initiation of study drug, untoward medical occurrence, if related to study procedure, will be recorded as AE; otherwise, will be recorded as medical history.

s Participants will be instructed to bring all used syringes of PEG-IFNα without disposing the remaining medication to the trial site at every visit, as applicable. Adherence of NRTI can be assessed by counting of empty containers and unused medication. Please refer to Section 6.6 for details.

t Participants will be monitored for clinical laboratory events of clinical interest (ECI) and additional assessments may be performed if indicated. ECI for this study include ALT > 5x ULN (Section 8.3.7).

u Samples for qualitative HBeAg assessment will be collected in participants with positive HBeAg at Day 1. Additional samples for qualitative HBeAg may be collected when the participant experiences HBV DNA ≥ 200 IU/mL. Samples for anti-HBe will be collected in all participants at all required timepoints.

[redacted]

1.3.2. NRTI Discontinuation Monitoring Period

Study Stage		NRTI Discontinuation Monitoring Period [b]				
**Study Visit Week**		W4	W8	W12	W18	W24/ EOS[c]
**Study Visit Day ± Visit Window**	D1[a]	D29 ± 3	D57 ± 3	D85± 3	D127± 3	D169 ± 7
Full physical exam						X
Symptom-directed physical exam	X	X	X	X	X	
Abdominal ultrasound						X
Fibroscan						X
Alcohol intake assessment	X	X	X	X	X	X
Vital signs[d]						X
Urinalysis						X
AFP						X
Hematology, Chemistry, and Coagulation assessments[e]	X	X	X	X	X	X
Blood sample for quantitative HBsAg	X	X	X	X	X	X
Blood sample for quantitative anti-HBs				X		X
Blood sample for quantitative HBV DNA[f]	X	X	X	X	X	X
Blood sample for qualitative HBeAg		X	X	X	X	X
[redacted]						[redacted]
[redacted]						[redacted]
HBV sequencing	←------------------ X [j] ------------------→					
Concomitant medications	←------------------ X ------------------→					
Adverse event assessment	←------------------ X ------------------→					
ECI surveillance [i]	←------------------ X ------------------→					

AFP = alpha- fetoprotein; ECI = events of clinical interest; EOS= end of study; HBeAg = hepatitis e-antigen; HBsAg = hepatitis B surface antigen; HBV = Hepatitis B Virus; NRTI = nucleoside/nucleotide; AFP = alpha-fetoprotein; anti-HBe = hepatitis B e-antibody; anti-HBs = hepatitis B surface antibody.

[a] Participants that meet the NRTI discontinuation criteria will enter the NRTI discontinuation monitoring period within 1 week of the investigator confirming eligibility. All timepoints are relative to the day of NRTI discontinuation (D1) which is the first day the NRTI withdrawal eligible participant discontinuing their NRTI therapy.

[b] All participants who discontinue NRTI therapy will be followed through Week 24, even if NRTI retreatment occurs. Investigator should monitor participants for NRTI retreatment criteria. Participant experiencing HBV DNA increase $\geq 2 \log_{10}$ IU/mL within a 2-week period should return to the site for unscheduled visits to measure HBV DNA levels and liver function tests as soon as possible and continue returning to site weekly until HBV DNA levels stabilize (details refer to Section 4.1.2.2).

[c] If a participant withdraws prematurely from the study prior to the W24 visit, EOS assessments should be performed.

[d] Vital signs (blood pressure, pulse rate, respiratory rate, and temperature) should be measured after the participant has rested comfortably for approximately 10 minutes.
[e] Additional safety laboratory assessments may be performed at any time during the study as determined necessary by the investigator (not limited to safety assessments listed in protocol, local laboratory assessment is acceptable).
[f] If a participant experiences HBV DNA > 200 IU/mL, additional HBV DNA sample(s) should be collected within 2 weeks.
[redacted]
[i] Participants will be monitored for clinical laboratory events of clinical interest (ECI) and additional assessments may be performed if indicated. ECI for this study include ALT > 5x ULN (See Section 8.3.7).
[j] Blood samples may be collected for HBV sequencing as determined necessary by the investigator.

2. INTRODUCTION

2.1. Background

Chronic hepatitis B virus (HBV) infection remains an important global public health problem, especially in China, that leads to serious sequelae over time, including cirrhosis, liver failure, and hepatocellular carcinoma (Trepo, 2014). An estimated 296 million people are living with chronic HBV infection worldwide, and around 820,000 people die annually due to associated sequelae (Stanaway, 2016; WHO, 2021). In China, the disease burden remains large with more than 80 million chronic HBV infections and a death rate of 11.4 death per 100,000, which is among the highest rate of the world (Polaris Observatory Collaborators, 2020; GBD 2019 Hepatitis B Collaborators, 2022).

Current treatment options for chronic HBV infection are limited. One option is long-term nucleos(t)ide reverse transcriptase inhibitor (NRTI) therapy, for example tenofovir (tenofovir disoproxil fumarate or tenofovir alafenamide) or entecavir (Lok, 2016). NRTI therapy suppresses HBV replication and viremia but does not eliminate the covalently closed circular DNA (cccDNA) or integrated DNA. Therefore, transcription and translation of viral proteins, in particular the production of HBsAg, typically continue at high levels as measured in blood (Scaglione, 2012). An alternative treatment option is a finite course of Pegylated Interferon α (PEG-IFNα) therapy which can induce a functional cure, but only in a small percentage of patients (< 10%) (Konerman, 2016). The failure of NRTI therapy to produce a functional cure and the limitations of low response rate of PEG-IFNα therapy highlight the clinical need for effective, well-tolerated HBV therapies of finite duration.

Several investigational agents are currently under development for the treatment of chronic HBV infection. Early-phase clinical studies have shown that neither direct antiviral agents nor immune modulators are likely to achieve a significant high functional cure rate when used alone. The growing consensus in the field is that combination therapies with multiple mechanism of actions involving inhibition of viral replication, reduction of viral antigen burden and stimulation of anti-viral immune response will likely be needed for success (Zoulim, 2015; Revill, 2019; Fanning, 2019; Lim, 2023). Persistent high antigen load is thought to be a major factor driving the exhaustion of antiviral adaptive immunity. New antiviral agents that can substantially reduce HBV antigen production could be rationally combined with direct immunotherapies in the hope of achieving a synergistic effect.

2.2. BRII-835

BRII-835 is a novel synthetic ribonucleic acid interference (RNAi) therapeutic that is being developed for the treatment of chronic HBV infection by Vir Biotechnology, Inc (Vir) in partnership with Alnylam Pharmaceuticals (Alnylam) globally and in partnership with Brii Biosciences for development in Greater China. BRII-835 is designed to target HBV transcripts containing a region in the HBx gene that is common to all HBV transcripts and highly conserved

among HBV genotypes. It is conjugated to an *N*-acetyl galactosamine (GalNAc) ligand to facilitate the delivery to hepatocytes via the asialoglycoprotein receptor (ASGPR).

The use of siRNA offers a novel strategy for the treatment of chronic HBV infection. The siRNA antisense strand binds to the complementary target mRNA; the duplexes load to the RNA-induced silencing complex that exploit the endogenous RNA-interference pathway to enable sequence-specific RNA cleavage and degradation. siRNA can have multiple antiviral effects, including degradation of the pgRNA, thus inhibiting viral replication, and degradation of all viral messenger RNA (mRNA) transcripts. It is postulated that high levels of circulating HBV surface antigens have negative impact on immune profile and may tolerize or deplete HBV specific T cells in chronic hepatitis B (CHB) patients (Kondo, 2013; Lebosse, 2017; Ferrari, 2015; Wang, 2013). This was supported by the previous findings that reductions of HBsAg by therapeutic interventions such as siRNA or anti-HBs antibody enabled HBV vaccines to break immune tolerance and achieve functional cure in the recombinant AAV/HBV infection murine models (Zhu, 2016; Micheler, 2020). These studies provide scientific basis to treat chronic HBV patients for functional cure with a combination of immunomodulators such as PEG-IFNα, and an agent such as BRII-835 (VIR-2218), an siRNA targeting HBV transcript to lower viral antigens including HBsAg.

BRII-835 has demonstrated potent, pan-genotypic, dose-dependent pharmacologic activity in all in vitro and in vivo models tested, resulting in significant and sustained HBsAg reduction. The available clinical data showed that BRII-835 was generally well-tolerated with good safety profile and induced significant reduction of HBsAg in CHB patients (Lim, 2023). Therefore, BRII-835 may act as a critical component in combination therapies to reduce viral antigen burden and facilitate the recovery of anti-viral immunity which can be further boosted by immunotherapy.

2.2.1. Summary of Nonclinical Experience

The pharmacology, pharmacokinetics and toxicology of BRII-835 has been evaluated *in vitro* and in multiple species. The summary of nonclinical pharmacology, pharmacokinetic (toxicokinetic) and toxicology data supports the safe use of BRII-835 for the treatment of patients chronically infected with HBV. Refer to the Investigator's Brochure for BRII-835 (VIR-2218) nonclinical information.

2.2.2. Summary of Clinical Experience

BRII-835 has been and is being evaluated in several clinical trials in healthy adult participants and CHB patients, which include one completed study VIR-2218-1005 in China, and 4 ongoing studies: VIR-2218-1001 (VIR-2218 alone or in combination with PEG-IFNα), VIR-2218-1006 (VIR-2218, VIR-3434, and/or PEG-IFNα), VIR-2218-1007 (also known as BRII-179-835-001) and VIR-2218-1008 sponsored by Gilead Sciences, Inc. (also known as GS-US-465-4439).

VIR-2218-1001 is an ongoing Phase 1/2 study evaluating single and multiple ascending doses of BRII-835 as a single agent (Parts A to C) or single agent/coadministered with PEG-IFNα (Part D/F). Part A is single ascending dose phase in healthy adult participants to evaluate the safety/tolerability and the PK of BRII-835. Part B and C are multiple ascending dose phase in

noncirrhotic adult participants with chronic HBV infection on NRTI therapy to evaluate the safety, tolerability, and PK of multiple doses of BRII-835. Part D-F are Phase 2 open-label study to evaluate the safety and tolerability of BRII-835 alone or in combination with PEG-IFNα (sequential or concurrent initiation) in noncirrhotic adult participants with chronic HBV infection on NRTI therapy.

The safety data in Part A-C showed that BRII-835 alone was general well-tolerated with good safety profile in healthy adults and CHB patients. The majority of AEs were Grade 1 or 2 in severity and considered not related to study drug. No clinically significant laboratory abnormalities were observed. The available safety data in Part D/F showed that a higher proportion of participants who received BRII-835+PEG-IFNα had AEs compared with single-agent BRII-835 (85.5% vs 60.0%). The majority of AEs were Grade 1 or 2 in severity and were considered not related to BRII-835; a higher proportion of AEs were considered related to PEG-IFNα compared to those related to BRII-835 and were consistent with the known adverse reactions of PEG-IFNα.

The antiviral activity data In Parts B/C showed that greater reductions in mean serum $\log_{10}$ HBsAg were observed in participants administered BRII-835 compared with placebo and the reductions in serum $\log_{10}$ HBsAg were dose-dependent, where higher BRII-835 doses were associated with greater reductions. Half of participants administered BRII-835 achieved an absolute HBsAg level < 100 IU/mL by Week 16. The available antiviral data in Parts D-F showed that greater reductions in mean serum $\log_{10}$ HBsAg were observed at nadir in participants who had concurrent treatment of BRII-835+PEG-IFNα for ≥ 24 weeks compared with BRII-835 alone, concurrent initiation of BRII-835+PEG-IFNα for 12 weeks, or 12 weeks of BRII-835+PEG-IFNα after 12 weeks of BRII-835 lead-in. More than two-thirds of participants (67%) achieved an absolute HBsAg level < 100 IU/mL at Week 12 and most (89%) achieved an absolute HBsAg level < 100 IU/mL at Week 24. It is noticeable that concurrent initiation of BRII-835 and PEG-IFNα combination achieved the greatest reductions in HBsAg through Week 48; and longer duration of concurrent BRII-835 with PEG-IFNα resulted in lower HBsAg levels, and 30.8% (4/13) achieved HBsAg loss with anti-HBs seroconversion (with anti-HBs >10 mIU/mL) at Week 48 (Yuen, 2022). More recently, the emerging data in Parts D-F of 24 weeks post end of treatment (EOT) revealed a strong association between anti-HBs titers at end of treatment and the durability of HBsAg seroclearance. Among participants who had HBsAg seroclearance by EOT, all participants (4/4) with anti-HBs levels > 500 mIU/mL at EOT had sustained HBsAg seroclearance at 24 weeks post EOT; while all participants (3/3) with anti-HBs < 100 mIU/mL at EOT experienced HBsAg rebound (Yuen, 2023).

The available PK data in Part A−C showed that BRII-835 was rapidly absorbed after SC injection and has a short plasma $t_{1/2}$ of 2 to 5 hours. The PK of BRII-835 in participants with chronic HBV infection was similar to that in healthy participants. Following two doses of BRII-835 administered 4 weeks apart in participants with chronic HBV infection, no accumulation of BRII-835 in plasma was evident. Overall, PK and PK/PD relationships in participants with chronic HBV infection support the further development of BRII-835 for the treatment of HBV.

Study VIR-2218-1005 is a Phase 2 double-blind, randomized, placebo-controlled study consisting of 2 parts which were conducted in China. The study includes adult participants with

HBeAg-negative (Part One) or HBeAg-positive (Part Two) chronic HBV infection without cirrhosis who are on NRTI therapy. Study drug (BRII-835 [VIR-2218] or placebo) is administered via SC injection. A total of 16 participants received BRII-835 across Part One and Part Two of this completed study. BRII-835 was generally well tolerated as 2 monthly doses of up to 100 mg in participants with noncirrhotic chronic HBV infection. Greater reductions in mean serum HBsAg were observed for participants administered BRII-835 compared with placebo among both HBeAg-negative and HBeAg-positive participants.

Study BRII-179-835-001 (VIR-2218-1007) is an ongoing Phase 2 open-label, randomized parallel-group study in adult participants with chronic HBV infection without cirrhosis who are on NRTI therapy. This study is conducted in APAC countries and regions, including Australia, New Zealand, Singapore, Thailand, Hong Kong, South Korea, and Taiwan. BRII-835 is administered alone or coadministered with therapeutic vaccine BRII-179 or BRII-179 with IFNα as co-adjuvant. Preliminary safety and efficacy data through Week 40 showed that among 50 participants randomized and dosed, a total of 46 participants have completed all assigned treatments. Either alone or in combination with therapeutic vaccine BRII-179 was generally well tolerated with similar HBsAg reductions from baseline across all dose regimens (Yuen, 2023). Mean changes in log_{10} HBsAg from baseline at Week 40 were -1.68 [SD: 0.37] IU/mL for BRII-835 alone regimen, and -1.75 [SD: 0.60] and -1.77 [SD: 0.72] IU/mL for BRII-179-containing regimens, respectively. More than 40% of participants receiving combination regimens had anti-HBs antibodies > 100 IU/L. As of the end of July 2023, all participants have completed study participation. More than 65% of the participants completed the last dose of BRII-835 treatment for at least 12 months.

2.3. PEG-IFNα

Interferon α (IFNα) is the only licensed drug for hepatitis B with immunomodulatory as well as viral inhibitory properties (PEGASYS, 2017; Rijckborst, 2010). It acts by inducing interferon stimulated genes (ISGs) at various points of the HBV lifecycle through different pathways that cause an increase in breakdown in viral RNAs and protection against viral injury (Ivashkiv, 2014). IFNα also stimulates cell-mediated immune responses which target infected hepatocytes leading to a decrease in cells that harbor the intrahepatic HBV cccDNA molecules responsible for persistence of HBV infection. Additionally it may reshape the immune landscape to induce adaptive immunity by coordinating various immune cells, including NK cells, macrophages, DCs and T cells (Gill, 2016; Micco, 2013).

Pegylation of IFNα (PEG-IFNα) allowing weekly injection is currently recommended as the first-line therapy for compensated CHB patients and the recommended dose and duration of PEG-IFNα therapy for both HBeAg-positive and HBeAg negative CHB is 180 mcg per week for 48 weeks. PEG-IFNα is associated with several side-effects including influenza-like symptoms, neutropenia, thrombocytopenia, depression, and less commonly exacerbation or unmasking of autoimmune illnesses as well as hepatitis flares, however, these flares have also been associated with HBeAg and HBsAg clearance. The main advantages of PEG-IFNα in CHB treatment are finite treatment duration, the absence of resistance, and the potential for immune-mediated control of HBV infection with an opportunity to obtain a sustained virological response off-

treatment, especially a chance of HBsAg seroclearance in a subset of CHB patients (Asian-Pacific clinical practice guidelines on the management of hepatitis B, 2016). However, functional cure after PEG-IFNα monotherapy is only confined in a small percentage of CHB patients (< 10%) (Konerman, 2016).

2.4. Rationale for BRII-835 and PEG-IFNα Combination Therapy for the Treatment of Chronic HBV Infection

HBV proteins including HBsAg, HBeAg, HBx and polymerase have been demonstrated to block multiple interferon response pathways and inhibit interferon-stimulated gene (ISG) induction in vitro, hence suppressing the direct anti-viral effects of PEG-IFNα (Luangsay, 2015; Xu, 2016; Tsunematsu, 2017; Liu, 2015; Wang, 2010). In addition, the anti-HBV immunity stimulated by PEG-IFNα may be decoyed and exhausted by excessive amount of HBsAg-containing subviral particles, impairing the effects of PEG-IFNα (Tout, 2020). Therefore, lowering tolerogens may be important for enhancing the immune response induced by PEG-IFNα therapy in CHB patients, increasing the rate of HBsAg seroclearance.

BRII-835, siRNA inhibiting HBV transcription can directly reduce HBV antigen burden and may restore immune function that in turn, enhances the immune modulation effect induced by PEG-IFNα therapy, together increasing the rate of the HBsAg seroclearance. The recent study evaluating the combination of BRII-835 (VIR-2218) and PEG-IFNα in CHB patients under NRTI background therapy demonstrated a proof of principle that concurrent initiation of BRII-835 and PEG-IFNα therapy (48 weeks) resulted in enhanced responses in its HBsAg reductions, and notable rates of HBsAg seroclearance with anti-HBs seroconversion by EOT (30.8%, 4/13) (Yuen, 2022). The 24-week follow-up data further demonstrated the correlation between durability of HBsAg seroclearance post treatment and titer of anti-HBs antibody (>500 mIU/mL) at EOT (Yuen, 2023). These preliminary data strongly suggest that the siRNA can unlock the potential of immunomodulator PEG-IFNα, together in combination may increase the rate of functional cure for chronic HBV infection. It also suggested the potential of anti-HBs response as an on-treatment biomarker of off-treatment sustained response.

2.5. Benefit/Risk Assessment

This study will provide information on the safety, efficacy, and dose regimens of BRII-835 and PEG-IFNα combination therapy, which has the potential to functionally cure chronic HBV infection.

The available data showed that BRII-835 with or without PEG-IFNα was generally well-tolerated in participants with chronic HBV infection. Additionally, reductions in HBsAg have been observed across participants treated with BRII-835. Longer duration of combination treatment of siRNA and PEG-IFNα showed a trend of improved functional cure rate compared to historical PEG-IFNα monotherapy data, suggesting a potential additive effect in increasing HBsAg loss in selected CHB population.

The potential risks with PEG-IFNα and BRII-835 have been considered in the inclusion and exclusion criteria. Only participants who meet all of the inclusion criteria and none of the

exclusion criteria (as specified in the protocol) will be allowed to participate in this study. Safety will be closely monitored by the investigator throughout the study. Several safety measures have been proposed to minimize potential risks to participants, including PEG-IFNα dose modification, treatment discontinuation and withdrawal criteria, etc. The potential risks with BRII-835 include injection site reactions (ISRs), immunogenicity and liver function test abnormalities. No ISRs or liver transaminase elevation SAEs have been reported and all nonserious ISRs to date have resolved without sequelae. Specific indicators of abnormal liver function will continue to be included as part of stopping rules in clinical studies of BRII-835, and the sponsor will continue to exclude participants who may be intolerant to SC injections or who are at high risk of hepatotoxicity. In addition, an SRC will be established to monitor safety data.

Taken together, the safety and antiviral activity data support a favorable benefit-risk profile for the evaluation of 100 mg or 200 mg BRII-835 and PEG-IFNα combination therapy for the treatment of chronic HBV infection.

3. STUDY OBJECTIVES AND ENDPOINTS

3.1. Study Objectives

3.1.1. Primary Objectives

- To evaluate the efficacy and safety of BRII-835 and PEG-IFNα combination therapy in participants with chronic HBV infection

3.1.2. Secondary Objectives

- To assess the antiviral activity of BRII-835 and PEG-IFNα combination therapy
- To assess BRII-835 PK exposure and evaluate anti-drug antibodies (ADA) to BRII-835

3.1.3. [redacted]

3.2. Study Endpoints

3.2.1. Primary Endpoints

The primary endpoints for this study are to evaluate:

- Proportion of participants with HBsAg loss at end of treatment
- Proportion of participants with HBsAg loss at 24 weeks post end of treatment
- Proportion of participants with treatment-emergent adverse events (TEAEs)
- Proportion of participants with serious adverse events (SAEs)

3.2.2. Secondary Endpoints

The secondary endpoints for this study are to evaluate:

- Proportion of participants with HBsAg loss at Week 48
- Proportion of participants achieving functional cure (defined as sustained undetectable HBsAg and sustained suppression of HBV DNA [<LLOQ] ≥ 24 weeks after discontinuation of all treatment, including NRTI)
- Proportion of participants achieving HBsAg loss and HBV DNA<LLOQ at 24 weeks after discontinuation of all treatments, including NRTI
- Proportion of participants meeting NRTI discontinuation criteria at 24 weeks post end of treatment

- Absolute serum HBsAg and change from baseline over time through 24 weeks post end of treatment
- Nadir serum HBsAg and maximum reduction of serum HBsAg from baseline throughout 48-week treatment
- Time to achieve first serum HBsAg loss through Week 72
- Proportion of participants with Hepatitis B e antigen (HBeAg) loss and HBeAg seroconversion (baseline HBeAg-positive participants) at any timepoint
- Proportion of participants with HBsAg seroconversion at any timepoint
- Plasma concentrations of BRII-835 and its metabolite at selected timepoints
- The occurrence of ADA to BRII-835 and associated titer, if positive

3.2.3. [redacted]

[redacted]

[redacted]

[redacted]

[redacted]

4. STUDY DESIGN

4.1. Overall Design

This is a Phase 2 open-label, randomized study of BRII-835 and PEG-IFNα combination therapy in adult participants with chronic HBV infection without cirrhosis receiving NRTI therapy as background therapy. This study is designed to evaluate the efficacy and safety of BRII-835 and PEG-IFNα combination therapy. Two different dose levels of BRII-835 in combination with PEG-IFNα will be evaluated.

Participants with HBeAg negative or HBeAg positive chronic HBV infection are eligible to participate in Cohorts 1-3 of this study. Eligible participants will be stratified by their serum HBsAg level at screening and randomized 1:1:1 to each of the three cohorts. Up to 75 participants are planned for Cohorts 1-3.

In addition, this study will evaluate the efficacy of BRII-835 and PEG-IFNα combination in participants previously treated with BRII-179, a therapeutic recombinant HBV vaccine. Eligible participants who completed BRII-179-835-001 study will roll over to Cohort 4. Up to 45 participants will be enrolled.

All participants will remain outpatient during study participation and will be followed for the safety, tolerability, and antiviral activity after completion of study intervention. The assessments and procedures performed at each visit are further described in the SoA (Section 1.3).

Participants will be assessed by the investigator for their eligibility to discontinue NRTI therapy at Week 72. Participants who meet the NRTI discontinuation criteria may enter the NRTI discontinuation monitoring period for an additional 24 weeks. Participants who do not meet the NRTI discontinuation criteria will continue NRTI therapy and will complete the study.

The total duration of study participation for each participant will include screening period (up to 4 weeks), dosing period (48 weeks), follow-up period (24 weeks), and NRTI discontinuation monitoring period (24 weeks when applicable). The study design schema can be found in Section 1.2.

4.1.1. Planned Cohorts and Dosing

Cohorts 1-3 (BRII-179 naïve):

Eligible participants of Cohorts 1-3 will be stratified by their serum HBsAg level at screening (>100 IU/mL and ≤ 500 IU/mL, > 500 and ≤ 1,500 IU/mL, or > 1,500 and ≤ 3,000 IU/mL) and randomized to receive the study intervention per the following dose regimens.

- Cohort 1: Each participant will receive 48 doses of PEG-IFNα 180 mcg administered SC every week starting on Day 1
- Cohort 2: Each participant will receive 48 doses of PEG-IFNα 180 mcg administered SC every week and 13 doses of BRII-835 200 mg administered SC every 4 weeks, both starting on Day 1

- Cohort 3: Each participant will receive 48 doses of PEG-IFNα 180 mcg administered SC every week and 13 doses of BRII-835 100 mg administered SC every 4 weeks, both starting on Day 1

Cohort 4 (BRII-179 treated):

Eligible participants who completed BRII-179-835-001 study will be assigned to Cohort 4 and receive the following study intervention.

- Cohort 4: Each participant will receive 48 doses of PEG-IFNα 180 mcg administered SC every week and 13 doses of BRII-835 100 mg administered SC every 4 weeks, both starting on Day 1

4.1.2. NRTI Discontinuation

4.1.2.1. NRTI Discontinuation Criteria

Upon participants completing Week 72 visits, the investigator will evaluate whether participants are eligible for discontinuation of NRTI therapy based on the following NRTI discontinuation criteria:

- HBsAg < LLOQ AND
- HBV DNA < LLOQ AND
- Undetectable HBeAg AND
- ALT ≤ 2x ULN

Participants who meet all of the above criteria for at least two consecutive visits at Week 72 are eligible to discontinue from NRTI therapy. Investigator should confirm participants' eligibility as early as possible within 2 weeks post Week 72 visit. Participants who discontinue NRTI will enter the NRTI Discontinuation Monitoring Period of the study within 1 week of the investigator confirming eligibility. Participants will not take their NRTI medications on the first day of the NRTI Discontinuation Monitoring Period and will be followed for an additional 24 weeks per the SoA (Section 1.3).

4.1.2.2. NRTI Re-treatment

It is recommended that the Investigator should consider re-treatment with NRTI therapy during the NRTI Discontinuation Monitoring Period if the participant meets any of the following criteria:

- ALT > 10x ULN with HBV DNA ＞LLOQ at the same visit
- ALT 5-10x ULN with HBV DNA ＞LLOQ persisting for ≥ 4 consecutive weeks
- ALT > ULN and HBV DNA > 2,000 IU/mL persisting for ≥ 4 consecutive weeks
- ALT > ULN with total bilirubin > 2x ULN and/or prolongation of prothrombin time (PT) > 2 seconds, and HBV DNA > LLOQ at the same visit

- HBV DNA > 100,000 IU/mL at any visit
- Confirmed HBeAg seroreversion (i.e., HBeAg positive after being HBeAg negative at NRTI discontinuation)
- Any other clinically significant event(s) warranting initiation of NRTI therapy in the opinion of the Investigator

Whether participants should be re-treated with NRTI is at the discretion of the Investigator following good medical judgement. Participants who are re-treated with NRTI therapy should continue to be followed per the SoA (Section 1.3.2) for the duration of the 24-week NRTI Discontinuation Monitoring Period.

Participants with HBV DNA increase ≥ 2 log_{10} IU/mL within a 2-week period should:

- Return to the site for an unscheduled visit to measure HBV DNA levels and liver function tests as soon as possible after the investigator being aware of the HBV DNA increase
- Continue returning to site weekly until HBV DNA levels stabilize (do not increase ≥ 1 log_{10} IU/mL over a 4-week period or start decreasing for at least 2 consecutive visits) after which they will continue follow-up per the SOA for the NRTI Discontinuation Monitoring Period
- If any unscheduled visits needed as outlined above coincide with an existing visit per the SoA, the scheduled assessments should be conducted

4.1.3. Safety Review Committee

A Safety Review Committee (SRC) for this study will monitor participant safety and consider study-specific data as well as relevant background information about the study drugs and target population under study to inform on study conduct. The primary purpose of the SRC is to protect the safety of participants in this study.

Ad hoc SRC meetings will take place as needed, i.e., for a significant safety event such as criteria for study intervention discontinuation being reached. Decisions to suspend dosing or discontinue individual participants from study intervention will be made according to the predetermined criteria (Section 7). Participants may resume study intervention only following SRC approval. Additionally, the SRC may recommend discontinuation of or changes to the study to the Sponsor. The SRC charter outlining the member composition and operating procedures will be developed before the study initiation.

4.2. Scientific Rationale for Study Design

This Phase 2, open-label, randomized study will evaluate the efficacy and safety of BRII-835 and PEG-IFNα combination therapy. This study design will provide an opportunity to explore the ability of two different dose levels of BRII-835 to achieve a clinical meaningful increase in the rate of HBsAg loss and functional cure when in combination with PEG-IFNα.

The principle of mechanism of the combination therapy of BRII-835 and PEG-IFNα leading to an enhanced HBsAg loss in CHB patients was demonstrated recently in a small cohort of patients in a Phase 2 study (VIR-2218-1001). The concurrent initiation of BRII-835 and PEG-

IFNα 48 weeks treatment resulted in the greatest reduction in HBsAg levels, and notable end of therapy HBsAg loss with anti-HBs seroconversion (30.8%, 4/13) compared to BRII-835 alone, or historical PEG-IFNα alone as well as PEG-IFNα following a BRII-835 lead-in (Yuen, 2022). This finding provided the basis to select the concurrent initiation of BRII-835 and PEG-IFNα combination therapy in this study.

The target population of this study is HBeAg-negative or HBeAg-positive non-cirrhotic patients with chronic HBV infection that has been virologically suppressed with NRTI therapy. This represents a patient population with acceptable heterogeneity who has been evaluated in BRII-835 single drug studies (VIR-2218-1001 study and VIR-2218-1005 study) and notable HBsAg reduction has been observed. The previous data of PEG-IFNα in combination with NRTI showed that HBsAg loss and functional cure rate could be higher in patients with baseline HBsAg <3,000 IU/ml or 1,500 IU/ml (Hu, 2018; Gan, 2022). Based on the scientific rationale that siRNA reduction in HBsAg by BRII-835 may potentiate effect of PEG-IFNα, the combination of BRII-835 and PEG-IFNα are anticipated to increase the likelihood of HBsAg loss and functional cure rate compared to PEG-IFNα in patients with baseline HBsAg 100-3,000 IU/ml. The preliminary end of therapy data in VIR-2218-1001 study also showed higher HBsAg loss rate (40%) could be achieved compared to historical PEG-IFNα data in patients with lower baseline HBsAg (<1,500 IU/mL) that received 48 weeks concurrent initiation of BRII-835 and PEG-IFNα combination therapy (Yuen, 2022). Sustainable HBsAg loss 24 weeks post EOT was observed in participants receiving 48-week BRII-835 and PEG-IFNα combination therapy regardless of their baseline HBsAg levels or HBeAg status (Yuen, 2023). Viral rebound may occur shortly after NRTI withdrawal in some cases, which requires close monitoring during study participation in order to control ALT flares. To safely evaluate the durability of response (i.e., sustained HBV DNA suppression and HBsAg loss) post study drug treatment, this study design allows additional 24 weeks NRTI treatment before evaluating the criteria for stopping NRTI therapy.

Surrogate endpoints of HBV functional cure measure biochemical, virological, and histological outcomes. Based on current knowledge, the best surrogate of HBV functional cure is HBsAg loss confirmed on 2 occasions at least 6 months apart with or without HBsAg seroconversion plus HBV DNA below the limit of detection (Cornberg, 2020).

Rationale for Cohort 4:

The most recent emerging data demonstrated that anti-HBs response were associated with the sustainability of HBsAg seroclearance post EOT in CHB patients receiving BRII-835+PEG-IFNα combination therapy. In this study, all 7/13 subjects achieved HBsAg seroclearance after 48-week combination therapy have anti-HBs seroconversion, and all of them (4/4) with anti-HBs titers > 500 mIU/mL at EOT had sustained HBsAg seroclearance at 24 weeks post EOT; while participants (3/3) with anti-HBs titers < 100 mIU/mL at EOT experienced HBsAg rebound, suggesting high anti-HBs titers at EOT are correlated with the persistence of seroclearance (Yuen, 2023). Additionally, CHB patients with anti-HBs-producing B cells detected in PBMC at baseline may be an important immunological indicator to predict IFN treatment efficacy, showing a higher chance to reach functional cure to PEG-IFNα treatment (Geng, 2023). These findings suggested that the potential of potent humoral immune response to HBsAg in CHB patients for achieving and maintaining sustained functional cure.

BRII-179, a recombinant virus-like particle (VLP)-based protein immunotherapeutic consisting of three HBV surface antigens (PreS1, PreS2, and S), has demonstrated to be safe/well-tolerated and induce HBV-specific humoral and cellular immune responses in a proportion of CHB patients in phase 2 studies. In phase 1b/2a BRII-179-001 study, anti-HBs responses were induced in >30% patients receiving four doses of BRII-179 admixed with or without IFN-α as co-adjuvant (Ma, 2021). A more robust anti-HBs responses were observed in participants when received nine doses of BRII-835 + BRII-179 with or without IFN-α co-adjuvant, demonstrating a positive anti-HBs antibody response in greater than 50% participants with ≥ 40% participants had anti-HBs titers > 100 mIU/ml (Yuen, 2023).

Therefore, it is hypothesized that CHB patients previously showing activated immune responses, especially those with enhanced anti-HBs response in BRII-179-835-001 study, may have a less dysfunctional immunological profile to viral antigens and be able to respond better to the curative immunotherapy therapy such as BRII-835+PEG-IFNα.

4.3. Justification of Dose

Cohorts 1-4 are planned for this study to evaluate 180 mcg weekly PEG-IFNα treatment alone for 48 weeks and 180 mcg weekly PEG-IFNα treatment in combination with two dose levels of BRII-835 at 100 mg or 200 mg administered every 4 weeks for 13 doses, respectively.

The current recommended PEG-IFNα dosage regimen for the treatment of chronic HBV infection is 180 mcg per week for a duration of 48 weeks (Terrault, 2016); this regimen will be used for PEG-IFNα dosing in this study.

The selection of BRII-835 dose levels is based on the safety/tolerability, PK, and antiviral activity data collected from a completed Phase 2 study VIR-2218-1005, an ongoing Phase 1/2 study VIR-2218-1001, and an ongoing Phase 2 study BRII-835-179-001 (also known as VIR-2218-1007).

The ongoing Phase 1/2 Study VIR-2218-1001 Parts D-F are evaluating the safety, tolerability, and antiviral activity of BRII-835 alone or in combination with PEG-IFNα (sequential or concurrent initiation) in noncirrhotic adult participants with chronic HBV infection on NRTI therapy. Patients received six doses of 200 mg BRII-835 alone achieved HBsAg reduction from baseline with a maximum mean decrease of -1.9 log10 IU/mL. To date, available data showed that BRII-835 and PEG-IFNα combination therapy achieved deeper reduction in HBsAg levels with continuous HBsAg declines observed through the combination treatment period. In a small cohort of patients (N=13), 48 weeks of concurrent 200 mg BRII-835 administered every four weeks for 13 doses and PEG-IFNα administered weekly for 48 weeks resulted in greatest HBsAg reductions compared to BRII-835 alone or PEG-IFNα following a BRII-835 lead-in, and achieved the highest rates of HBsAg seroclearance with anti-HBs seroconversion by the end of treatment (30.8%, 4 of 13 participants) (Yuen, 2022). Safety data demonstrated that the BRII-835 and PEG-IFNα combination was generally well tolerated and exhibited safety profiles supportive of continued clinical development. The majority of TEAEs were Grade 1 or 2 in severity and most TEAEs reported are consistent with known effect of PEG-IFNα.

Study VIR-2218-1005 evaluated the safety/tolerability, PK, and efficacy of BRII-835 alone in both HBeAg-negative and HBeAg-positive Chinese participants with chronic HBV infection. BRII-835 was administered as two monthly subcutaneous (SC) doses of up to 100 mg. Results showed that two doses of BRII-835 up to 100 mg led to substantial reductions in HBsAg in both HBeAg-negative and HBeAg-positive participants. The reductions in HBsAg levels were more pronounced at the 100 mg dose level, with the mean maximum HBsAg reductions from baseline of -1.3 log10 IU/mL up to Week 16. The levels of serum HBsAg remained below baseline levels for up to Week 48, although a gradual rebound was observed. No participants had serum HBsAg loss or anti-HBs seroconversion at any timepoint. BRII-835 was generally well tolerated with a good safety profile; no apparent relationship between incidence of AE or treatment-emergent laboratory abnormalities and BRII-835 dose level or HBeAg status was observed.

In the ongoing Phase 2 study BRII-179-835-001 in patients with chronic HBV infection and with baseline HBsAg levels between 150 IU/mL and 3,000 IU/mL, 100 mg BRII-835 was administered every 4 weeks for 9 doses alone or in combination with BRII-179 (a therapeutic vaccine) with or without IFNα as co-adjuvant. Preliminary efficacy data showed that all cohorts achieved HBsAg reductions in serum HBsAg levels from baseline at the end of treatment with a mean decrease of -1.7 to -1.8 $\log_{10}$ IU/mL. Either BRII-835 alone or in combination with BRII-179 was generally well tolerated (Yuen, 2023).

Thus, based on the similar antiviral activity, especially observed in Study BRII-179-835-001 with 100 mg BRII-835 and Study VIR-2218-1001 with 200 mg BRII-835, as well as the good safety/tolerability profiles of BRII-835 either administered alone, in combination with BRII-179 with or without IFNα as co-adjuvant, or in combination with PEG-IFNα, the dose levels of BRII-835 at 100 mg or 200 mg administered every 4 weeks for a longer treatment duration (i.e., 13 doses) is deemed to be appropriate for further evaluation of the efficacy and safety when in combination with PEG-IFNα in this study.

The PK data to date showed that BRII-835 was rapidly absorbed after SC administration and had a short plasma $t_{1/2}$ of 2 to 5 hours. Plasma exposures for both BRII-835 and its metabolite AS(N-1)3'VIR-2218 increased in a dose dependent manner. Participants with chronic HBV infection had similar PK profiles as healthy participants. No accumulation of BRII-835 or AS(N-1)3'VIR-2218 in plasma was evident after multiple dosing.

All subjects receiving 100 mg of BRII-835 achieved significant HBsAg reductions and the dose has been well tolerated. Therefore, in Cohort 4, 100 mg BRII-835 will continue to be the dose to be evaluated.

In summary, the safety, PK and efficacy profiles support exploration of the proposed combination treatment regimens of BRII-835 at 100 mg and 200 mg every 4 weeks for 13 doses and PEG-IFNα at 180 mcg per week for 48 weeks. This study will provide information that enables determining the optimal combination of BRII-835 and PEG-IFNα, which could achieve significant and durable HBsAg reduction, have good safety/tolerability profiles, as well as minimize the economic burden on patients.

4.4. End of Study Definition

A participant is considered to have completed the study if he/she has completed all visits of the study including the last scheduled procedure shown in the SoA.

The end of the study is defined as the date of the last visit of the last participant in the study or last scheduled procedure shown in the SoA for the last participant in the trial.

5. STUDY POPULATION

5.1. Inclusion Criteria

Participants eligible to be included in the study must meet all of the following criteria. All laboratory test requirements for inclusion will be performed by central laboratory unless specified otherwise.

Cohorts 1-3 (BRII-179 naïve):

1. A signed and dated written Informed Consent Form (ICF) prior to study participation.
2. Age 18 (or age of legal consent, whichever is older) to 60 years, both inclusive.
3. Body mass index (BMI) $\geq$ 18 kg/m^2 and $\leq$ 32 kg/m^2.
4. Chronic HBV infection as defined by a positive serum HBsAg for ≥ 6 months prior to screening.
5. Must be on NRTI therapy consisting of either entecavir, tenofovir disoproxil, tenofovir amibufenamide, or tenofovir alafenamide and with HBV-DNA < LLOQ for at least 6 months before screening (local laboratory results accepted).
6. HBsAg > 100 IU/mL and ≤ 3,000 IU/mL at screening.
7. HBV DNA < LLOQ at screening.
8. Serum ALT and AST ≤ ULN at screening.
9. Female participants must have a negative pregnancy test or confirmation of postmenopausal status. Post-menopausal status is defined as 12 months with no menses without alternative medical cause. Women of child-bearing potential (WOCBP) must have a negative serum pregnancy test at screening and a negative urine pregnancy test on Day 1, cannot be breast feeding at screening and throughout the study, and must be willing to use highly effective methods of contraception 14 days before study drug administration through 48 weeks after last study drug administration (Section 11.4, Appendix 4). Female participants must also agree to refrain from egg donation and in vitro fertilization from the time of study intervention administration through 48 weeks after the last dose of study intervention.
10. Male participants with female partners of child-bearing potential must agree to meet 1 of the following contraception requirements from the time of study drug administration until 48 weeks after last study drug administration: vasectomy with documentation of azoospermia, or male condom use plus partner use of 1 of the contraceptive options listed for contraception for WOCBP. Male participants must also agree not to donate sperm for the 48 weeks following last study drug administration.
11. Agrees to not donate blood during the duration of the study.

12. Willing and able to comply with the study procedures and requirements.

Cohort 4 (BRII-179 treated):

1. A signed and dated written Informed Consent Form (ICF) prior to study participation.
2. Age ≥ 18 years (or age of legal consent, whichever is older).
3. Body mass index (BMI) ≥ 18 kg/m^2 and ≤ 32 kg/m^2.
4. Completed BRII-179-835-001 study and completed BRII-179 treatment.
5. Must be on NRTI therapy consisting of either entecavir, tenofovir disoproxil, tenofovir amibufenamide, or tenofovir alafenamide and with HBV-DNA < LLOQ for at least 6 months before screening (local laboratory results accepted).
6. HBsAg > 0.05 IU/mL at screening.
7. HBV DNA < LLOQ at screening.
8. Serum ALT and AST ≤ ULN at screening.
9. Female participants must have a negative pregnancy test or confirmation of postmenopausal status. Post-menopausal status is defined as 12 months with no menses without alternative medical cause. Women of child-bearing potential (WOCBP) must have a negative serum pregnancy test at screening and a negative urine pregnancy test on Day 1, cannot be breast feeding at screening and throughout the study, and must be willing to use highly effective methods of contraception 14 days before study drug administration through 48 weeks after last study drug administration (Section 11.4, Appendix 4). Female participants must also agree to refrain from egg donation and in vitro fertilization from the time of study intervention administration through 48 weeks after the last dose of study intervention.
10. Male participants with female partners of child-bearing potential must agree to meet 1 of the following contraception requirements from the time of study drug administration until 48 weeks after last study drug administration: vasectomy with documentation of azoospermia, or male condom use plus partner use of 1 of the contraceptive options listed for contraception for WOCBP. Male participants must also agree not to donate sperm for the 48 weeks following last study drug administration.
11. Agrees to not donate blood during the duration of the study.
12. Willing and able to comply with the study procedures and requirements.

Study screening laboratory tests may be repeated once for values considered to be questionable by investigator with medical monitor approval.

Cohort 4 will only enroll participants from BRII-179-835-001 study in APAC region excluding mainland China.

5.2. Exclusion Criteria

Participants will be excluded from the study if there is evidence of any of the following at screening or prior to randomization, as appropriate:

1. Current or prior treatment with:
    a) Small interfering RNAs or antisense oligonucleotides within 12 months prior to screening
    b) Interferon-based therapy within 12 months prior to screening
    c) Telbivudine within 6 months prior to screening
    d) Myelotoxic drugs such as systemic cytostatic agents (i.e., cancer chemotherapeutic medications) within 30 days before study drug administration at the Day 1 visit
    e) Systemic immunosuppressant (including but not limited to corticosteroids at a prednisolone equivalent dose > 40 mg/day) within 30 days before study drug administration at the Day 1 visit
    f) Granulocyte colony-stimulating factor (G-CSF), granulocyte-macrophage colony-stimulating factor (GM-CSF), recombinant human interleukin-11 (oprelvekin), or erythropoietin (EPO) within 6 months prior to screening
    g) Any HBV therapeutic vaccines prior to screening (**applicable to Cohorts 1-3 only**).
2. Any clinically significant chronic medical condition other than chronic HBV infection that, in the opinion of the investigator makes the participant unsuitable for participation in the study.
3. Any clinically significant acute condition such as fever (>38°C) or acute respiratory illness within 7 days of first study drug administration.
4. Significant liver fibrosis or cirrhosis as defined by having a FibroScan result of > 9 kPa at screening.
5. History or evidence of drug or alcohol abuse as assessed by the Investigator, within the 12 months before screening.
6. History of clinically significant chronic liver disease from any cause other than chronic HBV infection.
7. History of hepatic decompensation, including but not limited to ascites, hepatic encephalopathy and/or esophageal or gastric varices.
8. Diagnosed or suspected hepatocellular carcinoma. Participant with screening alpha-fetoprotein (AFP) concentration ≥200 nanogram per milliliter (ng/mL) should be excluded. If the screening AFP concentration is ≥50 ng/mL and <200 ng/mL, the absence of liver mass must be documented by imaging at screening.
9. History of thyroid disease not adequately controlled on prescribed medications, or clinically significant signs of thyroid dysfunction as determined by the Investigator at screening.

10. Known history of immunological function impairment, including but not limited to:
    a) Autoimmune disease (e.g., multiple sclerosis, type 1 diabetes mellitus , myasthenia gravis, Crohn's disease and other inflammatory bowel diseases, celiac disease, systemic lupus erythematosus, scleroderma, including diffuse systemic form and limited cutaneous form of systemic sclerosis or CREST syndrome, systemic sclerosis, dermatomyositis polymyositis, rheumatoid arthritis, juvenile idiopathic arthritis, autoimmune thyroiditis including Hashimoto thyroiditis, Grave's or Basedow's disease, immune thrombocytopenic purpura, autoimmune hemolytic anemia, autoimmune hepatitis, psoriasis, vitiligo, vasculitis, Guillain Barré syndrome, Addison's disease, Bell's palsy, and alopecia areata); OR
    b) Primary immunodeficiency disorders (e.g., common variable immune deficiency, defective phagocytic cell function and neutropenia syndromes, and complement deficiency); OR
    c) Secondary immunodeficiency disorders (e.g., resulting from acquired immunodeficiency syndrome caused by human immunodeficiency virus (HIV) infection, solid organ transplant, and splenectomy).
11. Current or past history of infection with human immunodeficiency virus (HIV), hepatitis C virus (HCV) or hepatitis Delta virus (HDV).
12. Renal impairment with estimated glomerular filtration rate (eGFR) $\leq$ 60 ml/min/1.73m^2 at screening.
13. Any laboratory test abnormality that is considered as clinically significant or may contradict treatment with PEG-IFNα, including but not limited to below laboratory abnormalities:
    a) Hemoglobin < 100 g/L
    b) Absolute neutrophil count < 1,500/mm^3 (< 1000/ mm^3 for African)
    c) Platelet count < 90,000/ mm^3
14. Uncontrolled diabetes mellitus as determined by the Investigator.
15. Uncontrolled hypertension as determined by the Investigator.
16. Diagnosis of advanced stage heart failure (New York Heart Association Class III or Class IV or unstable angina).
17. Previous or current psychiatric condition that precludes compliance with the protocol. Specifically excluded are persons with severe psychiatric disorders as determined by the Investigator, including, but not limited to schizophrenia, psychosis, severe depression, bipolar illness, severe anxiety, ongoing risk for suicide, or history of suicide attempt or gesture within the past 5 years.
18. The participant has any history of cancer requiring locoregional or systemic therapy within 5 years of randomization or has current disease. Participants with completely surgically resected tumors with no evidence of disease for more than 3 years, or a history of low risk basal cell carcinoma will be accepted (low risk being defined as 1)

location on the trunk of the body, arms, legs, cheeks, forehead, temples, scalp, neck, or chin, 2) < 2 cm in size, 3) nodular or superficial, 4) primary cancer that has not come back after treatment, 5) edge of the cancerous area is clear and smooth, and 6) not located in or around nerves).

19. History of intolerance to SC injection.
20. History of intolerance or hypersensitivity to interferon/pegylated interferon.
21. Known hypersensitivity or contraindication to an siRNA/GalNAc or their excipients.
22. Any skin abnormality or tattoo that would limit post treatment injection site assessment.
23. Participated in another clinical trial with an investigational drug or a biologic within 30 days or within 5 drug half-lives (whichever is longer) before study drug administration at the Day 1 visit. Participants must agree not to take part in any other study at any time during their participation in this study, inclusive of the follow-up period and NRTI Discontinuation Monitoring Period.
24. Unwilling, or unable in the opinion of the Investigator, to comply with study requirements.

5.3. Screening Failures

Screening failures are defined as participants who consent to participate in the clinical study but are not subsequently randomly assigned to study drug. A minimal set of screening failure information is required to ensure transparent reporting of screening failure participants to meet the Consolidated Standards of Reporting Trials (CONSORT) publishing requirements and to respond to queries from regulatory authorities. Minimal information includes demography, screen failure details, eligibility criteria, and any SAE.

Individuals who do not meet the criteria for participation in this study (screening failure) should not be rescreened.

5.4. Lifestyle Considerations

Alcohol consumption is limited to no more than 1 unit per day (unit: 1 glass of wine [125 mL] = 1 measure of spirits [30 mL] = one-half pint of beer [284 mL]) during the participation of this study.

Participants will abstain from strenuous exercise from the time of screening throughout the study participation.

6. TREATMENT OF PARTICIPANTS

6.1. Study Drugs

6.1.1. General Information of Study Drugs

BRII-835 (VIR-2218) is a synthetic, chemically modified small interfering RNA (siRNA) targeting HBV RNA with a covalently attached triantennary N-acetyl-galactosamine (GalNAc) ligand that allows for specific uptake by hepatocytes. BRII-835 will be supplied as a clear, colorless to pale yellow, sterile solution for SC injection at a free acid concentration of 200 mg/mL.

PEG-IFNα is a covalent conjugate of recombinant alfa-2a interferon (approximate molecular weight [MW] 20,000 daltons) with a single branched bis-monomethoxy polyethylene glycol (PEG) chain (approximate MW 40,000 daltons). The PEG moiety is linked at a single site to the interferon alfa moiety via a stable amide bond to lysine. Peginterferon alfa-2a has an approximate molecular weight of 60,000 daltons. Interferon alfa-2a is produced using recombinant DNA technology in which a cloned human leukocyte interferon gene is inserted into and expressed in *Escherichia coli*. PEG-IFNα is a sterile, preservative-free, colorless to slightly yellowish solution available as an injection and is administered subcutaneously. PEG-IFNα (Pegasys, brand name Pegasys®) will be supplied as prefilled syringes containing 180 mcg/ 0.5 mL or 135 mcg/ 0.5 mL peginterferon alfa-2a.

Pegasys 180 micrograms solution for injection and Pegasys 135 micrograms solution for injection in pre-filled syringe each contains 0.5 ml solution with excipients of 180 micrograms (for 180 mcg/ 0.5 mL) and 135 micrograms (for 135 mcg/ 0.5 mL) peginterferon alfa-2a also contains sodium chloride, polysorbate 80, benzyl alcohol, sodium acetate, acetic acid and water for injections.

6.1.2. Study Drug Dose and Administration

Table 1 Study Drug Dosage and Administration

Cohort Name	Cohorts 1, 2, 3, and 4	Cohort 2	Cohort 3 and 4
**Intervention Name**	PEG-IFNα (Pegasys, brand name Pegasys®)	BRII-835 (VIR-2218)	BRII-835 (VIR-2218)
**Dose Level**	180 mcg every week	200 mg every 4 weeks	100 mg every 4 weeks
**Dose Formulation**	Solution for Injection	Solution for Injection	Solution for Injection
**Unit Dose**	180 mcg/ 0.5 mL<br>135 mcg/ 0.5 mL	200 mg/mL	200 mg/mL

**Pack Volume**	0.5 mL/ pre-filled syringe	0.5 mL/ vial	0.5 mL/ vial
**Route of Administration**	SC injection	SC injection	SC injection

6.1.3. Study Drug Materials and Management

6.1.3.1. Study Drug Packaging and Labeling

PEG-IFNα (Pegasys®) and BRII-835 will be centrally supplied by Sponsor as Investigational Medication Products (IMPs).

Single panel label with one local language and/or booklet label with multiple languages will be applied to the inner pack and outer pack of IMPs per country regulation and requirements.

IMPs packaging and labeling and activities will be conducted in a contracted 3rd party Contracted Manufacturing Organization (CMO) fully compliant with Good Manufacturing Practice (GMP).

6.1.3.2. Study Drug Storage

BRII-835 should be stored upright and refrigerated (2°C – 8°C) in a secure, temperature controlled, and locked environment with restricted access.

PEG-IFNα (Pegasys®) should be stored in a refrigerator (2°C - 8°C). Do not freeze or shake. Keep the pre-filled syringes in the outer carton in order to protect from light.

6.1.3.3. Study Drug Preparation

Participant dose of BRII-835 preparation should follow the site sterilization dose preparation Standard Operating Procedure (SOP) or procedures described in the Pharmacy Manual at site by designated site staff.

Appropriate amount of PEG-IFNα (Pegasys®) will be dispensed to participant during each visit in dosing period.

Participants should be trained by designated site staff about how to store Pegasys® at home, strictly follow the instructed administration dose and follow the procedures addressed in the effective pharmacy manual to prepare for the injection at home.

6.1.3.4. Study Drug Administration

Each successfully randomized participant will receive an appropriate amount of PEG-IFNα (Pegasys®) in each visit in dosing period for taking home administration. Keep this medicine out of the sight and reach of children.

All participants should be trained in proper self-injection technique on Day 1. Pegasys® should be inspected visually for particulate matter and discoloration before administration, and not used if particulate matter is visible or product is discolored. Prefilled syringes with particulate matter or discoloration should be returned to the pharmacist. Participants will receive the first dose of

PEG-IFNα on Day 1 in the clinic and the following doses once weekly at home. Participants will be instructed to inject PEG-IFNα on the same day each week (e.g., each Monday).

Participants enrolled in Cohort 2, 3, or 4 will receive BRII-835 at the assigned dose levels once every four weeks in the clinic. On dosing days, the pharmacist or designee will prepare the required amount of study drug into syringe for injection. A qualified clinical investigative site staff member under the supervision of the investigator or designee will administer the study drug. The injection site will be marked and mapped for later observation and should be documented. If a local reaction around the injection site occurs, photographs may be obtained. Participants will remain in the clinic for at least one hour post study drug administration. The observation period will include an assessment for rare adverse reactions.

Refer to the Pharmacy Manual for detailed instructions of study drug preparation and administration.

6.1.3.5. Study Drug Accountability

An IRT will be used to manage the IMPs supply. The initial site supply will be triggered either by site initiation or activation registration in IRT, or will be requested by clinical research organization (CRO) or site personnel manually (details refer to the Pharmacy Manual).

Please follow IRT user specification to proceed any IMPs transaction at site.

The IMPs handling activities at site (e.g. IMPs shipment receiving, storage, temperature monitoring, storage temperature excursion case reporting, unused IMPs return, destruction of unused or expired IMPs) are addressed in pharmacy manual. Follow the effective pharmacy manual for details.

The investigator, institution, or the head of the medical institution (where applicable) is responsible for study intervention accountability, reconciliation, and record maintenance (i.e., receipt, reconciliation, and final disposition records).

Refer to the Pharmacy Manual for the details.

6.1.3.6. Study Drug Handling and Disposal

Unused IMPs could be destroyed at site in accordance with site SOP and local requirements or be returned back to a 3rd party for central destruction with written sponsor approval.

Any waste material should be disposed in accordance with local requirements and medical hazard disposal law.

The participants will be instructed by investigator or designated site staff to put the used Pegasys® syringe(s) back to the original outer pack(s) after injection and return them back to the site at the next visit. Participant should follow the instruction not to throw away any medicines via wastewater or household waste to help to protect the environment.

The unused PEG-IFNα should be stored in a refrigerator (2°C - 8°C) at home, no need to return back to site at the next visit. Investigator or designated site staff should check the availability of

any unused PEG-IFNα with participant before requesting the new PEG-IFNα assignment via IRT.

Refer to the Pharmacy Manual for the details.

6.2. Randomization and Blinding

Up to 75 participants will be randomly assigned in 1:1:1 ratio among Cohorts 1-3 using an IRT system. The randomization code will be generated by using a validated computerized system. Before the study is initiated, the log in information and directions for the IRT system will be provided to each site. The site will contact the IRT system prior to the start of study drug administration for each participant. Once a randomization number is assigned to a participant, it cannot be reassigned to any other participant in the study. Study intervention will be dispensed at the study visits summarized in SoA. Returned study intervention should not be re-dispensed to the participants.

Participants will be stratified at randomization by screening HBsAg level (>100 IU/mL and ≤500 IU/mL, >500 IU/mL and ≤1,500 IU/mL, >1,500 IU/mL and ≤3,000 IU/mL). Maximum number of participants allowed in each stratum of each cohort will be capped as six in the IRT initial system set up for the first 45 enrolled participants and the limitation may be adjusted based on enrollment status.

Randomization does not apply to Cohort 4 (BRII-179-835-001 extension cohort).

As this is an open-label study, blinding procedures are not applicable.

6.3. Dose Adjustment Criteria

The dose of BRII-835 will not be adjusted once assigned to Cohort 2, Cohort 3, or Cohort 4.

The dose of PEG-IFNα is based on relevant clinical guidelines and information from the package insert which is considered as proper by the investigator. The dose of PEG-IFNα should not be changed during the study unless dose modification or discontinuation is necessary as indicated by laboratory abnormalities/adverse events.

6.3.1. Safety Criteria for PEG-IFNα Dose Adjustment

If severe adverse reactions or laboratory abnormalities develop during PEG-IFNα therapy, the dose should be modified until the adverse reactions abate. If intolerance persists after dose adjustment, further PEG-IFNα dosing should be suspended. Table 2 provides guidance for dose modifications and discontinuation of PEG-IFNα based on laboratory abnormalities.

Table 2 PEG-IFNα Dose Modification Guidelines

Laboratory Parameters	PEG-IFNα Dose Reduction	PEG-IFNα Dose Interruption/Discontinuation
Absolute neutrophil count (ANC)	≥500, < 750 cells/mm^3: Reduce to 135 mcg once weekly	< 500 cells/mm^3: Treatment should be suspended until ANC values return to more than 1,000 cells/mm^3. Re-institute at 90 mcg and monitor ANC.
Platelet count	≥25,000, < 50,000 cells/mm^3: Reduce to 90 mcg once weekly	<25,000 cells/mm^3: Discontinue treatment
Alanine transaminase (ALT)	≥5, < 10 × ULN: more frequent monitoring of liver function should be performed and consideration should be given to either reducing the dosage to 135 mcg or temporarily suspending treatment. After dosage reduction or withholding, therapy can be resumed after ALT flares subside (see Section 11.3).	≥ 10 × ULN: Discontinue treatment

During PEG-IFNα treatment, participants should be monitored for severe adverse reactions, including but not limited to neuropsychiatric, autoimmune, endocrine, ophthalmologic, and infectious disorders. Monitor closely and withdraw therapy with persistently severe or worsening signs or symptoms of the above disorders.

For detail information on dosage adjustment, please refer to the package insert of PEG-IFNα.

During safety monitoring of PEG-IFNα-related adverse reactions in the study, investigators should pay close attention to changes in all safety laboratory tests results (see Section 8.3.6.1). Relevant laboratory tests (not limited to protocol-specified safety laboratory tests) should be performed and followed in a timely manner if necessary (in case of adverse events or significant laboratory abnormalities). For participants whose laboratory measures are close to, but not yet met, the criteria for dose adjustment, investigators are advised to increase the frequency of monitoring of relevant laboratory measures. For participants who meet the criteria for PEG-IFNα dose adjustment, investigators should increase the frequency of monitoring of relevant laboratory measures, and if it is still early until next scheduled study visit, the participants should be advised to have the relevant laboratory measures re-tested 1 to 2 weeks later. Retesting may be conducted at local laboratories under urgent circumstances.

6.4. Background Treatment

All participants are required to be on NRTI therapy for at least 6 months at the screening visit and continued throughout the study. Participants must remain on the same NRTI at screening to the end of their study participation unless medically indicated otherwise, i.e., development of resistance.

Allowed NRTI therapy must consist of the following:

- Entecavir
- Tenofovir disoproxil
- Tenofovir amubufenamide
- Tenofovir alafenamide

6.5. Concomitant Medications

Medications specifically prohibited in the exclusion criteria are not allowed during the ongoing study. If there is a clinical indication for any medication specifically prohibited, discontinuation from study intervention may be required. The investigator should discuss any questions regarding this with the Sponsor medical monitor. The final decision on any supportive therapy or vaccination rests with the investigator and/or the participant's primary physician. However, the decision to continue the participant on study intervention requires the mutual agreement of the investigator, the Sponsor, and the participant.

6.5.1. Prohibited Medications

The following medications are excluded throughout the duration of study participation.

Within 30 days before study drug administration at the Day 1 visit and throughout the duration of study participation:

- Myelotoxic drugs such as systemic cytostatic agents (i.e., cancer chemotherapeutic medications).
- Systemic immunosuppressant (short term treatment of corticosteroids ≤40 mg/day and ≤ 2 weeks is allowed per the specialist's discretion as medically indicated)
- Any investigational drugs/biological products other than the study product(s) used during this study
- Any hepatotoxic agents including OTC medicines and herbal remedies that may cause fluctuations of liver test results

Within 90 days before study drug administration at the Day 1 visit and throughout the duration of study participation:

- Blood products or immunoglobin

Within 6 months before screening and throughout the duration of study participation:

- Telbivudine

6.6. Treatment Compliance

6.6.1. PEG-IFNα Treatment Compliance

The dose of PEG-IFNα is based on relevant clinical guidelines and information from the package insert which is considered as proper by the investigator. Temporary interruptions of PEG-IFNα administration are discouraged. Participants are to remain on treatment for the entire duration of the study until completion of 48-week treatment and the dose of PEG-IFNα should not be changed during the trial unless dose reduction or discontinuation is necessary as indicated by laboratory abnormalities / adverse events (see Section 6.3 for instructions for dose adjustment). Participants must be counselled regarding the importance of not missing doses of PEG-IFNα.

Participants should be instructed to inject PEG-IFNα on the same day each week (e.g., each Monday). In case that the participant missed any dose(s) at the planned day(s):

- Inject PEG-IFNα immediately if the subsequent dose to be administered on the next scheduled day will be at least 4 days after this day; OR
- Skip the current dose if the subsequent dose to be administered on the next scheduled day will be no more than 3 days after this day. Efforts should be made to administer the subsequent dose on the scheduled day

Participants will be instructed to bring all used syringes of PEG-IFNα without disposing the remaining medication to the trial site at every visit, as applicable, for adherence assessment. Adherence of PEG-IFNα will be assessed by counting of used syringes and remaining medication at every relevant visit. Every effort should be made to ensure good adherence with PEG-IFNα therapy.

6.6.2. NRTI Treatment Compliance

Every effort should be made to ensure good adherence with NRTI therapy. Adherence of NRTI can be assessed by counting of empty containers and unused medications at every relevant visit, when applicable.

7. DISCONTINUATION OF STUDY INTERVENTION AND PARTICIPANT DISCONTINUATION/WITHDRAWAL

7.1. Discontinuation of Study Intervention

A participant who has received at least one dose of BRII-835 or PEG-IFNα will hold the additional doses of either study drug if any of the following criterion is met:

- Discontinuation of study drug requested by the participant or the participant's legal representative
- Signs or symptoms of severe National Cancer Institute Common Terminology Criteria for Adverse Events (NCI-CTCAE) (Grade 3 or higher) hypersensitivity or anaphylaxis
- Significant abnormalities in liver chemistry, defined as any of the following are met:
    - Serum ALT > 10 × ULN
    - Serum ALT or AST > 3 × ULN with a concomitant total bilirubin > 2 × ULN or INR > 1.5
    - Serum ALT ≥ 3× ULN with newly onset of any severe symptoms of suspected hepatic decompensation, including but not limited to fatigue, nausea, vomiting, and/or right upper abdominal pain
- Any clinical manifestations of hepatic decompensation
- Any clinical manifestations of severe depression
- The participant has a medical condition or personal condition that, in the opinion of the investigator and/or the Sponsor, would pose an unnecessary risk to the participant if the investigational intervention is continued.
- The participant's serum pregnancy test is confirmed to be positive.

The Sponsor may request SRC evaluation of specific data in the event of liver flare to fully characterize transaminase elevations. The participant may be resumed to receive remaining doses of study drug only following SRC approval.

In addition, PEG-IFNα treatment should be discontinued if a participant meets any of the following criteria:

- Significant abnormalities in hematology, defined as any of the following criteria are met:
    - Absolute neutrophil count < 500 cells/mm^3
    - Platelet count < 25,000 cells/mm^3
- Severe known side effects, including but not limited to development of worsening of neuropsychiatric, endocrine, ophthalmologic, and autoimmune disorders that, in the

opinion of the investigator and/or the Sponsor, would pose unnecessary risk to the participant if the investigational intervention is continued

If a participant permanently discontinues one of the study drugs, this will result in early discontinuation of study treatment for this participant. The participant will not receive the remaining doses of the other study drug and will receive EOT assessments within 14 days post the last dose of study drugs and will be followed per the SoA of the Follow-up Period.

See the SoA (Section 1.3) for data to be collected at the time of discontinuation of study drug and follow-up and for any further evaluations that need to be completed.

7.2. Participant Discontinuation/Withdrawal from the Study

- A participant may withdraw from the study at any time at his/her own request, or may be withdrawn at any time at the discretion of the Investigator for safety, behavioral, compliance, or administrative reasons. This is expected to be uncommon.
- At the time of discontinuing from the study, if possible, an early discontinuation visit will be conducted, as shown in the SoA. See SoA for data to be collected at the time of study discontinuation and follow-up and for any further evaluations that need to be completed.
- The participant will be permanently discontinued from both the study intervention and the study at that time.
- If the participant withdraws consent for disclosure of future information, the sponsor may retain and continue to use any data collected before such a withdrawal of consent.
- If a participant withdraws from the study, he/she may request destruction of any samples taken and not tested, and the investigator must document this in the site study records.
- Participants who are withdrawn from the study will NOT be replaced and cannot be rescreened.
- The primary reason for participant withdrawal will be documented in the source document and in the CRF. Primary reasons for withdrawal will be categorized as:
    - Adverse event
    - Protocol violation
    - Withdrawal by participants
    - Lost to follow up
    - Investigator’s decision
    - Death
    - Study closed/terminated

7.3. Lost to Follow up

A participant will be considered lost to follow-up if he or she repeatedly fails to return for scheduled visits and is unable to be contacted by the study site.

The following actions must be taken if a participant fails to return to the clinic for a required study visit:

- The site must attempt to contact the participant and reschedule the missed visit as soon as possible and counsel the participant on the importance of maintaining the assigned visit schedule and ascertain whether or not the participant wishes to and/or should continue in the study.
- Before a participant is deemed lost to follow up, the investigator or designee must make every effort to regain contact with the participant (where possible, 3 telephone calls and, if necessary, a certified letter to the participant's last known mailing address or local equivalent methods). These contact attempts should be documented in the participant's medical record.
- Should the participant continue to be unreachable, he/she will be considered to have withdrawn from the study.

Discontinuation of specific sites or of the study as a whole are handled as part of Section 11.1.7 (Appendix 1).

8. STUDY ASSESSMENT AND PROCEDURES

8.1. Schedule of Assessments

Study procedures and their timing are summarized in the SoA (Section 1.3). Adherence to the study design requirements, including those specified in the SoA, is essential and required for study conduct. Protocol waivers or exemptions are not allowed. Immediate safety concerns should be discussed with the sponsor immediately upon occurrence or awareness to determine if the participant should continue or discontinue study intervention.

8.1.1. Screening

Written informed consent must be obtained prior to initiation of any study procedures. All screening evaluations must be completed and reviewed to confirm that potential participants meet all eligibility criteria. The Investigator will maintain a screening log to record details of all participants screened and to confirm eligibility or record reasons for screening failure.

Screening will be performed no more than 4 weeks prior to the first dose for each cohort and will include written consent, determination of participant eligibility, collection of demographics and medical history as well as a physical examination (including vital signs measurement), 12-lead ECG, clinical laboratory tests, and other assessments per the SoA.

A medical history for at least 24 months prior to study screening, including details regarding illnesses and allergies, date(s) of onset, and whether condition(s) is currently ongoing (current medical conditions), medication history, including prior HBV treatment history and HBV genotype history (if available), will be collected on all participants during screening and should be updated prior to randomization. Efforts should be made to collect the prior HBV treatment history as complete as possible since the initial diagnosis of chronic HBV infection. History of participation of the previous study will be collected in participants rolled over from BRII-179-835-001.

The screening viral serology parameters will include HIV antibody, HCV antibody, and HDV antibody to exclude current or past history of infection.

Adverse events related to protocol-mandated procedures during screening period must be collected from the time of consent onwards; any other events occurring during the screening period should be reported as medical history. All SAEs must be collected from the time of consent onwards.

8.1.2. Day 1

Participant eligibility must be confirmed prior to enrollment and study drug administration on Day 1. Eligible participants of Cohorts 1-3 will be randomized 1:1:1 to each of the three cohorts within 48 hours prior to study drug administration at the Day 1 visit. Eligible participants of the previous study BRII-179-835-001 will be assigned to Cohort 4. Eligible participants will receive their first dose of study drug(s) and will be instructed of proper self-injection techniques for further PEG-IFNα administration at home and completion of dosing card. Participants will be

assessed on physical examination (including vital sign measurement), clinical laboratory tests, and other assessments per the SoA. Participants will have the blood sample collection prior to study drug administration unless otherwise specified.

8.1.3. Dosing Period

Participants will return to the investigative site on an outpatient basis for safety, tolerability, and antiviral activity monitoring at the specified timepoints per the SoA.

All participants will receive 48 doses of PEG-IFNα administered SC every week from Day 1. Participants will be trained in proper self-injection technique and will receive the first dose of PEG-IFNα on Day 1 in the clinic. The following doses will be administered once weekly at home. Participants will be instructed to inject PEG-IFNα on the same day each week (e.g., each Monday). Participants must be counselled regarding the importance of not missing doses or overdosing of PEG-IFNα to the instructed dosage.

Participants enrolled in Cohort 2, Cohort 3, and Cohort 4 will receive BRII-835 administered SC at the assigned dose levels every 4 weeks, i.e., at Day 1, Week 4, Week 8, Week 12, Week 16, Week 20, Week 24, Week 28, Week 32, Week 36, Week 40, Week 44 and Week 48 in the clinic.

At each visit during the Dosing Period, there will be a review of ongoing eligibility to receive the study drugs and continue with study participation. There will also be a blood sample collection prior to each study drug administration (when applicable). Participants enrolled in Cohort 2, Cohort 3, and Cohort 4 will remain in the clinic at least one hour after each BRII-835 administration to be observed for rare adverse reactions. The injection site will be marked and mapped for later observation and should be documented. If a local reaction around the injection site occurs, photographs may be obtained.

If a participant permanently discontinues treatment prior to treatment completion, the EOT assessments should be performed within 14 days after treatment discontinuation per the SoA.

8.1.4. End of Treatment Visit

Participants may discontinue study drugs before the planned end of treatment (Week 48) or may withdraw from the study before the end of treatment (Week 48). In those cases, efforts should be made to conduct the EOT visit within 14 days after last dose of all study drugs. EOT assessments should be performed per the SoA.

8.1.5. Follow-up Period

Participants will return to the investigative site as outpatient for the specified in person assessments per the SoA, including but not limited to physical examination (including vital signs measurement), clinical laboratory tests (including safety, virological, and serological parameters), and review of adverse events and concomitant medications.

Participants will be assessed by the Investigator whether they are eligible for discontinuation of NRTI therapy upon completion of their Week 72 visits. Eligible participants to discontinue NRTI

therapy may enter into NRTI Discontinuation Monitoring Period for an additional 24 weeks per the SoA.

Participants who have NOT achieved NRTI discontinuation criteria (Section 4.1.2.1) by Week 72 will end the study and NRTI treatment should be continued.

If a participant discontinues study treatment before the planned EOT (Week 48) and does not withdraw from study participation, he/she will return to the investigational site every 8 weeks upon EOT for the specified assessments (safety follow-up) per the SoA.

8.1.6. NRTI Discontinuation Monitoring Period

Eligible participants who meet NRTI discontinuation criteria will be informed by the investigator to discontinue their NRTI medications when all related lab test results be available. Participants will not take their NRTI medications on the first day of NRTI Discontinuation Monitoring Period. Participants will be instructed not to stop their NRTI medications early until the scheduled Day 1 of NRTI Discontinuation Monitoring Period.

Participants who discontinue from their NRTI therapy will be followed in the NRTI Discontinuation Monitoring Period for an additional 24 weeks from the day of NRTI discontinuation per the SoA (Section 1.3.2). Participants who are retreated with NRTI therapy should continue to be followed per SoA for the duration of the 24-week NRTI Discontinuation Monitoring Period.

8.1.7. Unscheduled Visits

Participants may be asked to come for an unscheduled visit for clinical assessment of an AE if warranted, as determined by the investigator. The unscheduled visit will be documented in source documents and recorded in the CRF.

8.1.8. End of Study Visit

In cases where a participant prematurely discontinues from the study, the investigator must make every effort to have the participant return to the clinic within 14 days for an End of Study (EOS) Visit, unless the participant withdraws consent for study participation or is lost to follow-up. Assessments for EOS visits will include but not limited to physical examination, pregnancy test (for female participants), clinical laboratory tests, and other assessments per the SoA.

8.2. Efficacy Assessments

8.2.1. Antiviral Activities

Assessment of antiviral activity will include quantitative HBV DNA, quantitative HBsAg, quantitative anti-HBs, qualitative HBeAg, and qualitative anti-HBe.

Details regarding the processing, shipping, and analysis of the samples will be provided in the Laboratory Manual.

8.3. Safety Assessments

8.3.1. Physical Examination

A complete physical examination will include, at a minimum, general appearance, head, neck, chest/respiratory, heart/cardiovascular, gastrointestinal/liver, extremities, skin, and neurological assessments. Height and weight will also be measured and recorded. BMI will be calculated from height and weight.

A symptom-directed physical examination will be performed according to investigator discretion.

8.3.2. Vital Signs

Temperature (oral preferred), pulse rate, respiratory rate, and blood pressure will be assessed after the participant has rested comfortably for approximately 10 minutes. Pulse rate and blood pressure measurements will be assessed with a completely automated device. Manual techniques will be used only if an automated device is not available. When scheduled at the same timepoints, vital signs and 12-lead ECG are recommended to be assessed prior to physical examinations, blood sample collections, and urine collection.

8.3.3. Alcohol Intake Assessment

Alcohol intake during the study will be recorded and reviewed.

8.3.4. Liver Stiffness

Liver stiffness will be measured by FibroScan examinations as outlined in SoA.

Screening FibroScan result will be used to exclude the presence of significant liver fibrosis and cirrhosis. Participant with a FibroScan result of ≤ 9 kPa at screening can be considered without significant liver fibrosis and cirrhosis.

8.3.5. Ultrasound

Abdominal Ultrasound examinations will be performed and reviewed on-site by the Investigator at screening and every 6 months during the study for hepatocellular carcinoma surveillance as outlined in SoA.

8.3.6. Laboratory Assessments

8.3.6.1. Clinical Safety Laboratory Assessments

Clinical safety laboratory tests to be performed are outlined in SoA and described in details in Section 11.2 Appendix 2.

The investigator must review the laboratory report, document this review, and record any clinically significant changes occurring during the study in the AE section of the CRF. The laboratory reports must be filed with the source documents. Clinically significant abnormal

laboratory findings are those which are not associated with the underlying disease, unless judged by the investigator to be more severe than expected for the participant's condition.

In the event of an unexplained clinically relevant abnormal laboratory test occurring after study drug administration, the test should be repeated and followed at investigator discretion until it has returned to normal or baseline, and/or a diagnosis is made to adequately explain the abnormality.

8.3.6.2. Additional Clinical Assessment for The Adverse Reactions of PEG-IFNα

The investigator can conduct additional clinical assessments for the adverse reactions of PEG-IFNα, including but not limited to radiological examination, ophthalmology assessment, immunological markers, blood glucose, and psychological assessment. These assessments can be performed at local laboratory.

8.3.6.3. Pregnancy Testing

A pregnancy test or confirmation of post-menopausal status must be confirmed for all female participants. Post-menopausal status is defined as 12 months with no menses without alternative medical cause. An elevated FSH level in the post-menopausal range should be used to confirm a post-menopausal state in women not using hormonal contraception or hormone replacement therapy. In the absence of 12 months of amenorrhea, confirmation with more than one FSH measurement is required. Pregnancy tests will be performed for WOCBP only. Serum and urine pregnancy testing will be performed per the SoA and any time pregnancy is suspected. A WOCBP who is known to be pregnant or who does not have a negative pregnancy test at screening is not eligible for study participation. During the study, negative results of these pregnancy tests must be confirmed prior to study drug administration. A WOCBP determined to be pregnant while on study will be followed until the pregnancy outcome is known.

8.3.7. Laboratory Events of Clinical Interest

A clinical laboratory event of clinical interest (ECI) is a laboratory value of scientific and medical interest specific to understanding of the investigational product and may require additional follow-up testing, close monitoring, and rapid communication by the Investigator to the Sponsor.

Any laboratory ECIs will be monitored, defined as ALT ≥ 5x ULN, throughout this study.

All laboratory ECIs must be timely reviewed by the investigator and the assessment outlined in Section 11.3 should be performed as soon as possible and no later than one week after the initial ALT value meeting criteria for a laboratory ECI. Once an ECI subsides, i.e., the ALT level returns to ≤ ULN, a new ECI will be triggered if ALT increases and meets the criteria defined above.

The laboratory ECI will also be evaluated by the investigator to determine if the event meets criteria for an AE. If AE criteria are met, all clinical sequelae in relation to these laboratory ECIs will be reported as AEs or SAEs, if applicable.

8.4. Adverse Events and Serious Adverse Events

AEs will be reported by the participant.

The investigator and any qualified designees are responsible for detecting, documenting, and recording events that meet the definition of an AE or SAE and remain responsible for following up AEs that are serious, considered related to the study intervention or study procedures, or that caused the participant to discontinue the study intervention or study.

8.4.1. Definition of Adverse Events

8.4.1.1. Adverse Event (AE)

An adverse event is any untoward medical occurrence in a clinical study participant administered a medicinal product, which does not necessarily have a causal relationship with the treatment. An AE can therefore be any unfavorable and/or unintended sign, symptom, or disease temporally associated with the use of a medicinal product, whether or not considered related to the medicinal product. AEs may also include pre- or post-treatment complications that occur as a result of protocol specified procedures, lack of efficacy, overdose, drug abuse/misuse reports, or occupational exposure. Pre-existing conditions (recorded as medical history), which change in nature or severity should also be considered AEs.

An AE does not include the following:

- Medical or surgical procedures such as surgery, endoscopy, tooth extraction, and transfusion. The condition that leads to the procedure may be an adverse event and must be reported.
- Pre-existing diseases, conditions, or laboratory abnormalities present or detected before the screening visit that do not worsen
- Situations where an untoward medical occurrence has not occurred (eg, hospitalization for elective surgery, social and/or convenience admissions)
- Overdose of study drug without clinical sequelae
- Any medical condition or clinically significant laboratory abnormality with an onset date before the consent form is signed and not related to a protocol-associated procedure is not an AE. It is considered to be pre-existing and should be documented as medical history
- Any clinically significant abnormal laboratory findings or other abnormal safety assessments which are associated with the underlying disease, unless judged by the investigator to be more severe than expected for the participant’s condition

The criteria for considering whether an abnormal clinical laboratory finding or other abnormal assessments (eg, electrocardiogram) should be reported as an adverse event:

- Test result is associated with accompanying symptoms
- Test result requires medical/surgical intervention
- Test result leads to dose changing of study drug

- Test result is considered to be an adverse event by the investigator

If the laboratory abnormality is part of a clinical syndrome, record the syndrome or diagnosis (eg, anemia), not the laboratory result (ie, decreased hemoglobin).

8.4.1.2. Serious Adverse Event (SAE)

A serious adverse event as defined by ICH is any untoward medical occurrence that at any dose:

- Results in death
- Is life-threatening: An AE is life threatening if the participant was at immediate risk of death from the event as it occurred; ie, it does not include a reaction that if it had occurred in a more serious form might have caused death. For example, drug induced hepatitis that resolved without evidence of hepatic failure would not be considered life threatening even though drug induced hepatitis can be fatal.
- Requires inpatient hospitalization or prolongation of existing hospitalization: AEs requiring hospitalization should be considered SAEs. Hospitalization for elective surgery or routine clinical procedures that are not the result of AE (eg, elective surgery for a pre-existing condition that has not worsened) need not be considered AEs or SAEs. If anything untoward is reported during the procedure, that occurrence must be reported as an AE, either ‘serious’ or ‘non-serious’ according to the usual criteria. In general, hospitalization signifies that the participant has been detained (usually involving at least an overnight stay) at the hospital or emergency ward for observation and/or treatment that would not have been appropriate in the physician’s office or outpatient setting. When in doubt as to whether ‘hospitalization’ occurred or was necessary, the AE should be considered serious.
- Results in persistent or significant disability/incapacity: An AE is incapacitating or disabling if the experience results in a substantial and/or permanent disruption of the participant’s ability to carry out normal life functions.
- Is a congenital anomaly/birth defect in the offspring of a participant who received study drug(s).
- Other medically significant events: Important medical events that may not result in death, be life-threatening, or require hospitalization, may be considered an SAE when, based upon appropriate medical judgment, they may jeopardize the participant and may require medical or surgical intervention to prevent one of the outcomes listed in the above definition. Examples of such events are:
    - Intensive treatment in an emergency room or at home for allergic bronchospasm
    - Blood dyscrasias or convulsions that do not result in inpatient hospitalization
    - Development of drug dependency or drug abuse

8.4.2. Assessment of Adverse Events

8.4.2.1. Assessment of Causality

An Investigator or a qualified sub-investigator must make the determination of relationship to the study drug for each AE (Related or Not Related). The Investigator should decide whether, in his or her medical judgment, there is a reasonable possibility that the event may have been caused by the study drug. A "reasonable possibility" of a relationship conveys that there are facts, evidence, and/or arguments to suggest a causal relationship, rather than a relationship cannot be ruled out. "Related" should be entered when there is either a reasonable possibility that the AE/SAE is associated with study intervention or no reasonable alternative explanation can be found. Otherwise, the relationship between AEs and the study intervention should be classified as "Unrelated".

8.4.2.2. Assessment of Severity

The investigator will make an assessment of intensity for each AE reported during the study based on the NCI-CTCAE Version 5.0. The CTCAE displays Grade 1 through 5 with unique clinical descriptions of severity for each AE based on this general guideline (Table 3).

Table 3 CTCAE (V5.0) General Guideline of Severity of AEs

Severity	Definition
Grade 1	Mild; asymptomatic or mild symptoms; clinical or diagnostic observations only; intervention not indicated.
Grade 2	Moderate; minimal, local or noninvasive intervention indicated; limiting age-appropriate instrumental ADL. Instrumental ADL refer to preparing meals, shopping for groceries or clothes, using the telephone, managing money, etc.
Grade 3	Severe or medically significant but not immediately life-threatening; hospitalization or prolongation of hospitalization indicated; disabling; limiting self-care ADL. Self-care ADL refer to bathing, dressing and undressing, feeding self, using the toilet, taking medications, and not bedridden.
Grade 4	Life-threatening consequences; urgent intervention indicated.
Grade 5	Death related to AE.

ADL = activities of daily living
A semi-colon indicates 'or' within the description of the grade.
Source: NCI 2017

8.4.3. Time Period and Frequency for Collecting AEs and SAEs

AEs

After informed consent, but prior to initiation of study intervention, the following type of events should be reported: AEs related to protocol-mandated procedures. Other medical occurrences that begin before the start of study intervention but after obtaining informed consent, that are

deemed not related to the screening activities, will be recorded as Medical History. Following initiation of study intervention, collect all AEs, regardless of cause or relationship to study drug, until the participant has completed the study.

SAEs

All SAEs, regardless of cause or relationship to study drug, that occur after the participant first consent to participate in the study (ie, signing the informed consent) and throughout the duration of the study, until the participant has completed the study, must be reported as instructed. This also includes any SAEs resulting from protocol-mandated procedures performed after informed consent is signed.

Investigators are not obligated to actively seek SAEs after the participant completed the study. However, if the investigator learns of any SAEs that occur after study participation has concluded and the event is deemed relevant to the use of study drug, he/she should promptly document and report the event to the Sponsor.

8.4.4. Recording of AEs and SAEs

When an AE/SAE occurs, it is the responsibility of the investigator to review all documentation (e.g., hospital progress notes, laboratory reports, and diagnostics reports) related to the event.

The investigator will then record all relevant AE/SAE information in the CRF and additional SAE form.

It is not encouraged to directly send photocopies of the participant's medical records to the Sponsor in lieu of completion of the AE/SAE form.

There may be instances when copies of medical records for certain cases are requested by the Sponsor. In this case, all participant identifiers, with the exception of the participant number, will be redacted on the copies of the medical records before submission to the Sponsor.

The investigator will attempt to establish a diagnosis of the event based on signs, symptoms, and/or other clinical information. Whenever possible, the diagnosis (not the individual signs/symptoms) will be documented as the AE/SAE.

8.4.5. Follow-up of AEs and SAEs

During the course of the study all AEs/SAEs should be proactively followed up for each participant. All SAEs should be followed until the event is resolved, stabilized, otherwise explained, or the participant is lost to follow-up. In addition, the investigator will make every attempt to follow all nonserious AEs for outcome. This may imply that observations will continue beyond the last planned visit per protocol.

The investigator is obligated to perform or arrange for the conduct of supplemental measurements and/or evaluations as medically indicated or as requested by the Sponsor to elucidate the nature and/or causality of the AE or SAE as fully as possible. This may include additional laboratory tests or investigations, histopathological examinations, or consultation with other health care professionals.

For fatal or life-threatening events, copies of hospital case reports, discharge summaries, autopsy reports, and other documents should be submitted by email when requested and applicable.

The investigator will submit any updated SAE data to the Sponsor within 24 hours of receipt of the information.

Additional information may be requested to ensure the timely completion of accurate safety reports.

8.4.6. Reporting of SAEs

All SAEs must be reported by sending to the Sponsor or designee (Novotech Safety) via secure e-mail connection within 24 hours of the first awareness of the event. The Investigator must complete, sign and date the SAE form, verify the accuracy of the information recorded on the SAE form with the corresponding source documents, and send a copy to sponsor or its designee.

Contacts for SAE reporting: Novotech Safety at email: safety@novotech-cro.com

8.4.7. Regulatory Reporting Requirements for SAEs

Prompt notification by the investigator to the sponsor of an SAE is essential so that legal obligations and ethical responsibilities towards the safety of participants and the safety of a study intervention under clinical investigation are met.

The sponsor has a legal responsibility to notify both the local regulatory authority and other regulatory agencies about the safety of a study intervention under clinical investigation. The sponsor will comply with country-specific regulatory requirements relating to safety reporting to the regulatory authority, Institutional Review Boards (IRB)/Independent Ethics Committees (IEC), and investigators.

Safety reports must be prepared for suspected unexpected serious adverse reactions (SUSAR) according to local regulatory requirements and sponsor policy and forwarded to investigators as necessary.

An investigator who receives an investigator safety report describing an SAE or other specific safety information from the sponsor will review and then file it along with the Investigator's Brochure and will notify the IRB/IEC, if appropriate according to local requirements.

8.4.8. Special Situation Reports

8.4.8.1. Definition of Special Situations

Special situation reports include all reports of medication error, abuse, misuse, overdose, reports of adverse events associated with product complaints, and pregnancy reports regardless of an associated AE.

Medication error is any unintentional error in the prescribing, dispensing, or administration of a medicinal product while in the control of the health care provider, participant, or consumer.

Abuse is defined as persistent or sporadic intentional excessive use of a medicinal product by a subject.

Misuse is defined as any intentional and inappropriate use of a medicinal product that is not in accordance with the protocol instructions or the local prescribing information.

An overdose is defined as an accidental or intentional administration of a quantity of a medicinal product given per administration or cumulatively which is above the maximum recommended dose as per protocol (as it applies to the daily dose of the participant in question). In cases of a discrepancy in drug accountability, overdose will be established only when it is clear that the subject has taken the excess dose(s). Overdose cannot be established when the pharmacist cannot account for the discrepancy except in cases in which the investigator has reason to suspect that the additional dose(s) were administered to the participant.

Product complaint is defined as complaints arising from potential deviations in the manufacture, packaging, or distribution of the medicinal product.

Pregnancy is defined as the any pregnancy event of a female participant entered in a study as well as pregnancy occurring in a female partner of a male participant entered in a study with a medicinal product. If a participant becomes pregnant during the course of the study, study drug should be discontinued immediately.

8.4.8.2. Instruction for reporting special situations

8.4.8.2.1. Pregnancy reporting

If a female participant becomes pregnant after the start of study drug administration through 48 weeks after the last study drug administration, the participant will be instructed to report this to the investigator. The investigator must report the pregnancy to the Sponsor or designee (safety@novotech-cro.com) within 24 hours of being notified of the pregnancy. The participant should receive any necessary counseling regarding the risk of continuing the pregnancy and the possible effects on the fetus.

The pregnancy should be followed by the investigator to determine the outcome of the pregnancy. The investigator will collect follow-up information on the mother and infant and will notify the Sponsor within 24 hours of obtaining updated information regarding the pregnancy. At the completion of the pregnancy, the investigator will document the outcome of the pregnancy. Abnormal pregnancy outcomes (e.g., spontaneous abortion, fetal death, stillbirth, congenital anomalies, ectopic pregnancy) are considered SAEs. Elective abortions without complications should not be handled as AEs. Every effort will be done to follow the infant for up to 6-8 weeks after birth.

If the partner of a male participant becomes pregnant after the start of study drug administration through 48 weeks after the last study drug administration, the participant will be instructed to report this to the investigator. The partner will be asked to provide consent to be followed until the outcome of the pregnancy is known and for up to 6-8 weeks after birth (where permitted). The investigator must report the pregnancy to the Sponsor or designee (safety@novotech-cro.com)

within 24 hours of being notified of the pregnancy. The participant and the partner should receive any necessary counseling regarding the risk of continuing the pregnancy and the possible effects of the fetus. After obtaining consent, the investigator must report available follow-up information on the course and outcome of the pregnancy within 24 hours of receipt of the information.

8.4.8.2.2. Reporting other special situations

All other special situations must be reported to the Sponsor or designee (safety@novotech-cro.com) within 24 hours of the investigator becoming aware of the situation. These reports must consist of situations that involve study drug.

Special situations involving medications other than study drug do not need to be reported; however, for special situations that result in AEs due to a medication other than study drug, the AE should be reported.

Any inappropriate use of concomitant medications prohibited by this protocol should not be reported as “misuse” but may be more appropriately documented as a protocol deviation.

All clinical sequelae in relation to these special situation reports will be reported as AEs or SAEs at the same time. Details of the symptoms and signs, clinical management, and outcome will be reported, when available.

8.5. Pharmacokinetic (PK) Assessments

Blood samples will be collected for measurement of plasma concentrations of BRII-835 and its metabolite as specified in the SoA. Timepoints for the collection of BRII-835 PK samples are provided in Table 4. Instructions for the collection and handling of blood samples will be provided in the Laboratory Manual. The actual date and time (24-hour clock time) of each sample will be recorded.

Table 4 Pharmacokinetics Assessment Timepoints

Study Day/Week	Protocol Time		
	Predose[a]	Dose	Postdose[b]
Day 1	X		X
Day 225/ Week 32	X		X
Day 337/ Week 48	X		X

[a] At ≤ 2 hours prior to dosing
[b] Between ≥ 2 hours and ≤ 12 hours after dosing

8.6. Assessment of Anti-Drug Antibodies (ADA) to BRII-835

Blood samples will be collected for analyses of ADA to BRII-835 as specified in the SoA. See the Laboratory Manual for instructions on sample processing and storage.

8.7. [redacted]

[redacted]

[redacted]

[redacted]

- [redacted]
- [redacted]

[redacted]

[redacted]

[redacted]

9. STATISTICS

Detailed methodology for summary and statistical analyses of the data collected in this study is outlined here and further detailed in the statistical analysis plan (SAP), which will be maintained by the sponsor. The SAP may modify what is outlined in the protocol where appropriate; however, any major modifications of the primary endpoint definitions or their analyses will be reflected in a protocol amendment.

9.1. Statistical Hypotheses

An estimation approach with no hypothesis testing is used to analyze the primary outcomes. A Bayesian framework will be used to calculate the point estimate of the primary outcomes and 95% credible interval.

9.2. Estimands

Primary Estimand 1

The primary estimand 1 of the study is a while on treatment estimand, participants prematurely discontinue treatment will be treated as intercurrent event, which estimates the on-treatment effect.

- Population: Participants with CHB as defined by the inclusion and exclusion criteria to reflect the targeted participant population
- Variable: HBsAg loss at end of treatment
- Treatment: Cohort 1, 2, 3 and 4. Estimation of the within-group response rate
- Intercurrent event: The intercurrent event is prematurely discontinue treatment
- Population-level summary: Proportion of participants who achieve HBsAg loss at end of treatment for each cohort

Primary Estimand 1 is the primary estimand for the primary endpoint proportion of participants with HBV surface antigen (HBsAg) loss at end of treatment.

Primary Estimand 2

Primary Estimand 2 is a composite estimand, participants withdraw from the trial prematurely will be treated as non-responders, which estimates the effect of randomized treatment accounting for treatment adherence and response.

- Population: Participants with CHB as defined by the inclusion and exclusion criteria to reflect the targeted participant population
- Variable: Responder is defined as participant achieves HBsAg loss at 24 weeks post end of treatment; for participants who drop out early for any reason the outcome will be defined as 'non-responsive' after that point
- Treatment: Cohort 1, 2, 3 and 4. Estimation of the within-group response rate

- Intercurrent event: The intercurrent event is captured through the variable definition (i.e., withdrawal from the trial prematurely)
- Population-level summary: Proportion of participants who achieve response for each cohort

Primary Estimand 2 (composite estimand) is the primary estimand for the primary endpoint (Proportion of participants with HBsAg loss at 24 weeks post end of treatment) and key secondary efficacy endpoints (Proportion of participants with HBV surface antigen [HBsAg] loss at Week 48; Proportion of participants achieving sustained undetectable HBsAg and sustained suppression of HBV DNA<LLOQ $\geq$ 24 weeks after discontinuation of all treatment, including NRT; Proportion of participants achieving HBsAg loss and HBV DNA < LLOQ 24 weeks after discontinuation of all treatment, including NRTI).

Secondary Estimand(s)

Hypothetical Estimand (Estimand 3)

The secondary estimand of this study is a hypothetical estimand, which estimates the effect had all participants maintain their randomized treatment.

- Population: Participants with CHB as defined by the inclusion and exclusion criteria to reflect the targeted participant population
- Variable: Change from baseline in HBsAg at Week 48 and Week 72
- Treatment: Cohort 1, 2, 3 and 4
- Intercurrent event: All data collected will be utilized; Discontinuation of, interruption in, and non-adherence to treatment will be handled assuming they had not happened
- Population-level summary: Difference in least-square means between each BRII-835 dose cohort and PEG-IFNα monotherapy cohort

Change from baseline to each specific post baseline scheduled time points in a continuous outcome measure such as HBsAg will be based on hypothetical estimand.

9.3. Sample Size Determination

Up to 75 participants will be randomly assigned in 1:1:1 ratio to each of Cohorts 1-3. A Bayesian framework will be used to calculate the sample size. It is assumed that the number of responders follows a Binomial distribution, with a weakly informative prior (Beta (0.5, 0.5)) for the true response rate. The precision for a range of response rates with 95% credible intervals are shown in Table 5:

Up to 45 participants will be enrolled in Cohort 4. No formal sample size is calculated due to the exploratory nature of Cohort 4.

Table 5 95% Credible Interval of Response Rate by Sample Size for Cohorts 1-3

Sample size per cohort	Number of responders	Response rate (%)	95% Credible interval* (%)
15	4	27	8-50
	5	33	13-57
	6	40	18-63
	7	47	25-71
	8	53	29-76
	9	60	36-82
25	6	24	10-41
	7	28	13-46
	8	32	16-51
	9	36	19-55
	10	40	22-58
	11	44	26-63
	12	48	30-67
	13	52	32-70

* 95% highest posterior density interval.

For the 15 participants in each cohort the lower bounds of 95% credible interval exclude the response rate of 10% if observed response rate is greater than 27% and exclude the response rate of 20% if observed response rate is greater than 40% and exclude the response rate of 30% if observed response rate is greater than 53%.

For the 25 participants in each cohort the lower bounds of 95% credible interval exclude the response rate of 10% if observed response rate is greater than 24% and exclude the response rate of 20% if observed response rate is greater than 36% and exclude the response rate of 30% if observed response rate is greater than 48%.The operational characteristics are based on posterior confidence that the true rate exceeds a threshold of interest, as shown in Table 6. For instance, when 4 out of 15 participants or 6 out of 25 participants are responders, the probability (true response rate>15%) is larger than 85%.

Table 6 Operational Characteristics by Sample Size

Sample size per cohort	Number of responders	Probability (True response rate)>15%	Probability (True response rate)>20%	Probability (True response rate)>25%	Probability (True response rate)>30%	Probability (True response rate)>35%
15	1	18.4%	8.5%	3.7%	1.4%	0.5%
	2	45.7%	27.2%	14.4%	7%	3.2%
	3	72.8%	52.6%	34.1%	20.1%	10.9%
	4	89.7%	75.6%	57.9%	40.6%	25.7%
	5	96.8%	90.4%	78.3%	62.7%	45.7%
	6	99.2%	96.6%	90.7%	80.4%	66.6%
	7	99.9%	99.2%	96.9%	92%	83.4%
25	2	16.2%	5.4%	1.6%	0.4%	0.1%
	3	35.7%	16.1%	6%	1.9%	0.5%
	4	58.2%	32.1%	14.9%	5.8%	1.9%
	5	77.4%	52.1%	29.4%	13.8%	5.5%
	6	89.1%	70.5%	46.9%	26.3%	12.1%
	7	95.9%	84.8%	65.5%	42.7%	23.6%
	8	98.5%	92.5%	79.4%	59.8%	38.3%
	9	99.6%	97.4%	89.6%	75%	55.2%
	10	99.9%	99%%	95.2%	86.1%	70.3%
	11	100%	99.7%	98.2%	93.3%	82.6%

9.4. Analysis Sets

Data for all participants will be assessed to determine if participants meet the criteria for inclusion in each analysis population prior to releasing the database and classifications will be documented per standard operating procedures.

Table 7 Analysis Sets

Population	Description
Screened Analysis Set	All participants who signed the ICF
Full Analysis Set (FAS)	All participants assigned to study intervention
Safety Analysis Set (SAS)	All participants assigned to study intervention and who take at least 1 dose of study drug. Participants will be analyzed according to the study intervention they received
Modified Intent-To-Treat (mITT)	All participants assigned to study intervention, who take at least 1 dose of study drug, with at least 1 postbaseline visit through Week 48. Participants will be analyzed according to the study intervention to which they were randomized for Cohorts 1- 3, and for Cohort 4 participants will be analyzed according to the study intervention specified in the protocol.
Per-Protocol (PP)	All participants in the mITT set without important protocol deviations considered to impact the interpretation of the primary efficacy endpoints. Protocol deviations will be reviewed to generate the list of participants with significant deviations to be excluded from the PP analysis set. The PP exclusion criteria will be finalized prior to releasing the database.
Pharmacokinetic Analysis Set (PAS)	All participants who received BRII-835, had a at least 1 evaluable PK concentration, and had no protocol deviations thought to impact on the PK data analysis.
ADA Analysis Set	All participants who received BRII-835 and had at least 1 non-missing ADA result from the ADA assay after first dose of the study drug. Participants will be analyzed according to the treatment actually received.
[redacted]	[redacted]

9.5. Statistical Methods

For continuous variables, descriptive statistics will include the following information: the number of participants reflected in the calculation (n), mean, standard deviation, first quartile (Q1), median, third quartile (Q3), minimum, and maximum. For categorical or ordinal data, frequencies and percentages will be displayed for each category.

Primary efficacy analysis will be Bayesian framework.

Statistical analysis will be performed using Statistical Analysis Software Version 9.4 or higher.

9.6. Statistical Analyses

9.6.1. Efficacy Analysis

9.6.1.1. Primary Efficacy Analysis:

- The primary endpoints are 1) Proportion of participants with HBsAg loss at end of treatment 2) Proportion of participants with HBsAg loss at 24 weeks post end of treatment.

A Bayesian hierarchical model (Yuen, 2022; Jones, 2011) as primary analysis will be used to estimate the posterior probability of response rate for each cohort of Cohorts 1-3 by incorporating the baseline HBsAg level stratification factor. The point estimate of the primary outcome and 95% equal-tailed credible interval will be drawn from the model.

Model for each group:
Number of responders $r_g \sim$ Binomial (n_g, p_g), g=1,2,3
$\theta_g = \text{logit}(p_g) = \log(p_g/(1-p_g)) = \gamma_0 + \gamma_1 I_{\{B+\}1} + \gamma_2 I_{\{B+\}2+} \psi_g$, g=1,2,3
where γ_0, γ_1, γ_2, ψ_g are all parameters, Thus,
$\theta_1 = \gamma_0 + \psi_1$
$\theta_2 = \gamma_0 + \gamma_1 + \psi_2$
$\theta_3 = \gamma_0 + \gamma_2 + \psi_3$
Priors:
$\gamma_k \sim$ Normal$(0, 10^6)$, k=0,1,2
$\psi_g \sim$ Normal$(0, \omega^2)$
$\omega \sim$ Half-normal(1)
Where r_g indicates the number of responders among n_g participants, p_g as the response rate r_g/n_g, θ_g indicates the log odds of treatment response $\log(p_g/(1-p_g))$, index g=1,2,3 refer to the stratum numbers,
γ_k represent fixed effects of baseline HBsAg stratification factor, and ψ_g denotes a random effect in stratum g.

Stratum	$I_{\{B+\}1}$	$I_{\{B+\}2}$
HBsAg>100 IU/mL and HBsAg≤500 IU/mL	0	0
HBsAg>500 IU/mL and HBsAg≤1,500 IU/mL	1	0
HBsAg>1,500 IU/mL and HBsAg≤3,000 IU/mL	0	1

$I_{\{B+\}1}$ and $I_{\{B+\}2}$ are the dummy variables of $I_{\{B+\}}$.

For each cohort of Cohorts 1-3, the posterior distribution of response rate $P(p_g|data)$, g=1,2,3 was derived for each stratum using the model specified above.

The posterior distribution of the group-level response rate will be derived using a mixture of the posterior distributions of response rate for each analysis stratum in that group. The weights are proportional to the sample size of each stratum.

For some models with low number of events (0–1 event), the stratified Bayesian hierarchical model does not converge. In such cases a reduced model that does not consider baseline stratification will be fitted. The model estimates the point estimate and 95% highest posterior density credible interval of proportion of participants achieving the primary endpoint assuming the number of responders followed a binomial distribution:

Number of responders r ~ Binomial (n, p)

Where p is the response rate, with a non-informative (Jeffrey's) prior: p ~Beta (0.5, 0.5).

The posterior distribution for p is: P (p|y, N) ~ Beta (y + 0.5, N – y + 0.5).

The same reduced model will be used to estimate the posterior distribution of Cohort 4 as no stratification factor is planned in this Cohort.

In addition, for Cohorts 1-3 combine some two adjacent baseline stratification levels to reduce HBsAg stratification factors from three levels to two levels (HBsAg >100 IU/mL and HBsAg ≤1,500 IU/mL versus HBsAg >1,500 IU/mL and HBsAg ≤3,000 IU/mL; HBsAg >100 IU/mL and HBsAg ≤500 IU/mL versus HBsAg >500 IU/mL and HBsAg ≤3,000 IU/mL) to perform the stratified Bayesian hierarchical analysis if model is converged using the similar approach as the primary analysis.

Samples from the posterior distribution of the response rates in each cohort of Cohorts 1-3 will be used to obtain posterior probabilities of interest (e.g., Probability [Difference of proportion of achieving primary endpoints between cohorts > threshold Data]); threshold such as 5%, 10%, 15%, 20% or any other percentiles of interest may be explored.

The primary efficacy analysis will be performed within mITT analysis set and a supplemental analysis based on PP analysis set with the similar analysis will be presented.

9.6.1.2. Secondary Endpoints Analyses

- Proportion of participants with HBsAg loss at Week 48

- Proportion of participants achieving functional cure (defined as sustained undetectable HBsAg and sustained suppression of HBV DNA <LLOQ ≥24 weeks after discontinuation of all treatment, including NRTI)
- Proportion of participants achieving HBsAg loss and HBV DNA <LLOQ 24 weeks after discontinuation of all treatment, including NRTI
- Proportion of participants meeting NRTI discontinuation criteria at 24 weeks post end of treatment

The analyses for the four endpoints are similar to the primary analysis of co-primary endpoint Proportion of participants with HBsAg loss at 24 weeks post end of treatment and will be based on mITT analysis set.

Absolute serum HBsAg and change from baseline over time through 24 weeks post end of treatment

Numerical value of HBsAg and change from baseline will be summarized by cohort and by visit.

For Cohorts 1-3 change from baseline in HBsAg to all scheduled visits in log base 10 scale will be statistically analyzed using a Mixed Model for Repeated Measures model. The model will include log base 10 transformed baseline as a covariate, treatment, visit, treatment by visit as fixed effects, and within-participant errors will be modeled with an unstructured, heterogeneous autoregressive (1), or compound symmetry variance matrix in that order if a model does not converge. Estimates of least square mean (LSM) values and the LSM differences between each BRII-835 dose cohort and PEG-IFNα monotherapy cohort, and their respective 95% confidence interval and p-values will be calculated. The analyses will be done using mITT analysis set with hypothetical estimand. If the model is not converged only descriptive statistics will be presented.

In the first interim analysis, absolute serum HBsAg and change from baseline over time through Week 48 will be performed.

Nadir serum HBsAg and maximum reduction of serum HBsAg from baseline

Nadir serum HBsAg and maximum reduction of serum HBsAg from baseline will be summarized by cohort based on mITT analysis set.

Time to achieve first time of serum HBsAg loss through Week 72

Time to achieve first time of serum HBsAg loss will be analyzed by taking account of losses to follow-up using the stratified log-rank test with randomization strata as a stratification factor based on mITT analysis set, and tabular summaries of the Kaplan Meier curves providing the median, quartiles, and range will be provided for each cohort, and the KM curves will be presented graphically. In addition, for Cohorts 1-3 a Cox proportional hazard regression model where the estimate of the hazard ratio for treatment (each BRII-835 dose cohort vs PEG-IFNα monotherapy cohort) will be performed, its 95% CI, and p value will be provided if the model is converged.

In the first interim analysis, time to achieve first time of serum HBsAg loss throughout Week 48 will be performed.

Proportion of participants with Hepatitis B e antigen (HBeAg) loss and/or HBeAg seroconversion (baseline HBeAg-positive participants)

Descriptive statistics by cohort based on mITT analysis set will be performed for this endpoint.

Proportion of participants with HBsAg seroconversion at any timepoint

Descriptive statistics by cohort based on mITT analysis set will be performed for this endpoint.

9.6.1.3. [redacted]

- [redacted]
- [redacted]
- [redacted]

[redacted]

9.6.1.4. Multiplicity

No multiplicity will be adjusted.

9.6.2. Safety Analysis

Safety analysis will be assessed using AEs, SAE, vital signs, clinical laboratory tests with clinician assessment, and concomitant medication data. Number and percentage of participants with TEAEs will be summarized by Medical Dictionary for Regulatory Activities (MedDRA) System Organ Class (SOC) and preferred term (PT), and summaries will be provided by severity and by causality (related, not related) to study drug. Continuous safety data (including absolute and change from baseline values) will be summarized by descriptive statistics, including n, mean, standard deviation (SD), median, Q1, Q3 and minimum and maximum values. Categorical endpoints will be summarized by frequency and percentage.

9.6.3. Pharmacokinetic Analysis

Plasma concentrations of BRII-835 and its metabolite will be listed for each participant and descriptively summarized, including n, mean, standard deviation (SD), median, minimum, maximum, geometric mean, and geometric coefficient of variation (CV), by treatment cohort and timepoints using PK analysis set.

9.6.4. Baseline and Other Summaries and Analyses

The demographic characteristics will be summarized by cohort based on FAS. This will include age, gender, race, baseline weight and baseline BMI. All baseline disease characteristics will be summarized by cohort within the FAS.

9.6.4.1. Study Conduct and Participant Disposition

Participant evaluation groups will be presented for all screened participants, and participant disposition will be summarized within the FAS. The number of participants screened and randomized will be presented. The number of participants treated, completed and discontinued, as well as the number of participants in each analysis set will be summarized by Cohort. For participants who did not complete the study, the reasons for withdrawal from the study will be presented. In addition, the number of participants who were excluded from the PP analysis set will be summarized by reasons for the exclusion.

9.6.4.2. Study Treatment Exposure

Duration of treatment will be summarized based on SAS. The duration of treatment will be calculated as follows:

Duration of treatment = Date of last dose of study drug – date of first dose of study drug +1.

9.6.4.3. Prior and Concomitant Medications

The frequency of prior and concomitant medications will be summarized by cohort based on the WHO-drug coding dictionary based on SAS.

9.6.4.4. The occurrence of ADA to BRII-835 and associated titer (if positive)

Descriptive statistics for the endpoint will be performed based on ADA analysis set.

9.7. Interim Analysis

- **Interim analysis and summaries**

Three interim analyses are planned. The first two interim analyses will be conducted when approximately 45 enrolled participants of Cohorts 1-3 complete the 48-week treatment and last available participant of all enrolled participants (Cohorts 1-4) complete the 48-week treatment, respectively. If the pace of enrollment is robust, and the primary endpoint proportion of participants with HBsAg loss at Week 48 is expected to occur less or equal to two months between the two interim analyses, the two analyses may be collapsed into one analysis. Endpoints with measurement time after the 48-week treatment except for safety relevant data will not be analyzed in the first two interim analyses.

The third interim analysis will be conducted when last available participant of Cohorts 1-3 completes follow-up 24 weeks post end of treatment.

9.8. Final Analysis

The final analysis will be performed when last available participant of Cohorts 1-4 completes the last scheduled visit according to the SoA.

Further details will be provided in the SAP.

10. REFERENCES

Asian-Pacific clinical practice guidelines on the management of hepatitis B: a 2015 update. Hepatol Int. 2016 Jan;10(1):1-98.

Bourlière M, et al. Effect on HBs antigen clearance of addition of pegylated interferon alfa-2a to nucleos(t)ide analogue therapy versus nucleos(t)ide analogue therapy alone in patients with Hbe antigen-negative chronic hepatitis B and sustained undetectable plasma hepatitis B virus DNA: a 81olaris81ed, controlled, open-label trial. Lancet Gastroenterol Hepatol. 2017 Mar;2(3):177-188.

Cornberg M, Lok AS, Terrault NA, Zoulim F, and the 2019 EASL-AASLD HBV Treatment Endpoints Conference Faculty. Guidance for design and endpoints of clinical trials in chronic hepatitis B – Report from the 2019 EASL-AASLD HBV Treatment Endpoints Conference. Journal of Hepatology. 2020; 72(3): 539-337.

Fanning GC, Zoulim F, Hou J, Bertoletti A. Therapeutic strategies for hepatitis B virus infection: towards a cure.Nat Rev Drug Discov. 2019 Nov;18(11):827-844.

Ferrari C. HBV and the immune response. Liver Int. 2015; 35 (Suppl. 1): 121–128.

Gan W. Functional cure based on pegylated interferon α-2b therapy in nucleoside analog-suppressed HBeAg negative chronic hepatitis B: A multicenter real-world study (Everest Project in China) – 4 years data update. AASLD 2022, oral presentation.

GBD 2019 Hepatitis B Collaborators. Global, regional, and national burden of hepatitis B, 1990-2019: a systematic analysis for the Global Burden of Disease Study 2019. Lancet Gastroenterol Hepatol. 2022;7(9):796-829.

Geng Y, Wan Y, Yin S, et al. Dynamic monitoring of HBsAg-specific B cells in CHB patients and its clinical significance in predicting interferon efficacy. EASL Congress 2023, Poster presentation WED-131-YI.

Gill U, Peppa D, Micco L, Singh H, Carey I, et al. Interferon alpha induces sustained changes in NK cell responsiveness to hepatitis B viral load suppression in vivo. PloS Pathog. 2016; Aug 3;12(8):e1005788.

Hu P, et al. HBsAg Loss with Peg-interferon Alfa-2a in Hepatitis B Patients with Partial Response to Nucleos(t)ide Analog: New Switch Study. J Clin Transl Hepatol 2018 Mar 28;6(1):25-34.

International Committee of Medical Journals Editors (ICMJE). Recommendations for the Conduct, Reporting, Editing and Publication of Scholarly Work in Medical Journals. http://www.icmje.org. Updated May 2022. Accessed December 3, 2022.

Ivashkiv LB, Donlin LT. Nat Rev Immunol. 2014 Jan;14(1):36-49.

Janssen HL, et al. Pegylated interferon alfa-2b alone or in combination with lamivudine for HBeAg-positive chronic hepatitis B: a randomized trial. Lancet 2005;365:123–129.

Jones HE, Ohlssen DI, Neuenschwander B, Racine A, Branson M. Bayesian models for subgroup analysis in clinical trials. Clin Trials 2011;8:129–143.

Kondo Y, Ninomiya M, Kakazu E, Kimura O, Shimosegawa T. Hepatitis B surface antigen could contribute to the immunopathogenesis of hepatitis B virus infection. ISRN Gastroenterol. 2013;2013:935295.

Konerman MA, Lok AS. Interferon Treatment for Hepatitis B. Clinics in Liver Disease. 2016; 20(4): 645-665.

Lau GK, et al. Peginterferon Alfa-2a, lamivudine, and the combination for HBeAg-positive chronic hepatitis B. N Engl J Med 2005;352:2682–2695.

Lebossé F, Testoni B, Fresquet J, Facchetti F, Galmozzi E, Fournier M, Hervieu V, Berthillon P, Berby F, Bordes I, Durantel D, Levrero M, Lampertico P, Zoulim F. Intrahepatic innate immune response pathways are downregulated in untreated chronic hepatitis B. J Hepatol. 2017;66(5):897-909.

Liaw YF, Kao JH, Piratvisuth T, et al. Asian-Pacific consensus statement on the management of chronic hepatitis B: a 2012 update. Hepatol Int 2012;6:531-61.

Lim SG, Baumert TF, Boni C, Gane E, Levrero M, et al.The scientific basis of combination therapy for chronic hepatitis B functional cure. Nat Rev Gastroenterol Hepatol. 2023; Jan 11. doi: 10.1038/s41575-022-00724-5.

Liu, S., Peng, N., Xie, J., Hao, Q., Zhang, M., Zhang, Y., et al. Human hepatitis B virus surface and e antigens inhibit major vault protein signaling in interferon induction pathways. J. Hepatol. 2015; 62, 1015–1023.

Lok AS, McMahon BJ, Brown RS, Wong JB, Ahmed AT, Farah W, Almasri J, Alahdab F, Benkhadra K, Mouchli MA, Singh S, Mohamed EA, Abu Dabrh AM, Prokop LJ, Wang Z, Murad MH, Mohammed K. Antiviral therapy for chronic hepatitis B viral infection in adults: A systematic review and meta-analysis. Hepatology 2016 ; 63(1) :284-306.

Luangsay, S., Gruffaz, M., Isorce, N., Testoni, B., Michelet, M., Faure-Dupuy, S., et al. Early inhibition of hepatocyte innate responses by hepatitis B virus. J. Hepatol. 2015; 63, 1314–1322.

Ma H, Lim TH, Leerapun A, et al. Therapeutic vaccine BRII-179 restores HBV-specific immune responses in patients with chronic HBV in a phas Ib/IIa study. JHEP Rep. 2021;3(6):100361.

Marcellin P, et al. Peginterferon alfa-2aalone, lamivudine alone, and the two in combination in patients with HBeAg-negative chronic hepatitis B. N Engl J Med 2004;351:1206–1217.

Micco L, Peppa D, Loggi E, Schurich A, Jefferson L, Cursaro C, et al. Differential boosting of innate and adaptive antiviral responses during pegylated-interferon-alpha therapy of chronic hepatitis B. J Hepatol. 2013;58(2):225-33.

Michler, T., A. D. Kosinska, J. Festag, T. Bunse, J. Su, M. Ringelhan, H. Imhof, D. Grimm, K. Steiger, C. Mogler, M. Heikenwalder, M. L. Michel, C. A. Guzman, S. Milstein, L. Sepp-Lorenzino, P. Knolle and U. Protzer (2020). "Knockdown of Virus Antigen Expression Increases Therapeutic Vaccine Efficacy in High-Titer Hepatitis B Virus Carrier Mic. E." Gastroenterology 158(6): 1762-1775 e1769.

Ning Q, Han M, Sun Y, Jiang J, Tan D, Hou J, Tang H, Sheng J, Zhao M. Switching from entecavir to PegIFN alfa-2a in patients with HBeAg-positive chronic hepatitis B: a 83olaris83ed open-label trial (OSST trial). Randomized Controlled Trial J Hepatol. 2014 Oct;61(4):777-84.

PEGASYS® (peginterferon alfa-2a) [US package insert]. South San Francisco, CA: Genentech, Inc.; 2017.

Polaris Observatory Collaborators. HBV progress towards coverage targets. https: //cdafound.org/83olaris-countries-dashboard.

Revill PA, Penicaud C, Brechot C, et al. Meeting the Challenge of Eliminating Chronic Hepatitis B Infection. Genes (Basel). 2019;10(4).

Rijckborst V, Janssen HL. The role of interferon in hepatitis B therapy. Curr Hepat Rep. 2010;9(4):231-238.

Scaglione SJ, Lok AS. Effectiveness of hepatitis B treatment in clinical practice. Gastroenterology 2012; 142:1360-1368.

Stanaway JD, Flaxman AD, Naghavi M, Fitzmaurice C, Vos T, Abubakar I, Abu-Raddad LJ, Assadi R, Bhala N, Cowie B, Forouzanfour MH, Groeger J, Hanafiah KM, Jacobsen KH, James SL, MacLachlan J, Malekzadeh R, Martin NK, Mokdad AA, Mokdad AH, Murray CJL, Plass D, Rana S, Rein DB, Richardus JH, Sanabria J, Saylan M, Shahraz S, So S, Vlassov V, Weiderpass E, Wiersma ST, Younis M, Yu C, Zaki M, Cooke GS. The global burden of viral hepatitis from 1990 to 2013: findings from the Global Burden of Disease Study 2013. Lancet 2016; 388(10049): 1081-1088.

Terrault NA, Bzowej NH, Chang KM, et al. AASLD guidelines for treatment of chronic hepatitis B. Hepatology 2016;63:261-83.

Tout I, Loureiro D, Mansouri A, Soumelis V, Boyer N, et al. Hepatitis B surface antigen seroclearance: Immune mechanisms, clinical impact, importance for drug development. J Hepatol.2020 Aug;73(2):409-422.

Trepo CHL, Chan, Lok A. Hepatitis B virus infection. Lancet 2014;384(9959): 2053-2063.

Tsunematsu, S., Suda, G., Yamasaki, K., Kimura, M., Izumi, T., Umemura, M., et al. Hepatitis B virus X protein impairs alpha-interferon signaling via upregulation of suppressor of cytokine signaling 3 and protein phosphatase 2A. J. Med. Virol. 2017; 89, 267–275.

Wang, H., and Ryu, W. S. Hepatitis B virus polymerase blocks pattern recognition receptor signaling via interaction with DDX3: implications for immune evasion. PloS Pathog. 2010; 6:e1000986.

Wang S, Chen Z, Hu C, Qian F, Cheng Y, Wu M, Shi B, Chen J, Hu Y, Yuan Z. Hepatitis B virus surface antigen selectively inhibits TLR2 ligand-induced IL-12 production in monocytes/macrophages by interfering with JNK activation. J Immunol. 2013 May 15;190(10):5142-51.

WHO. Hepatitis B. 2021. https://www.who.int/news-room/fact-sheets/detail/hepatitis-b.

Wong GLH, Gane E, Lok ASF. How to achieve functional cure of HBV: Stopping NUCs, adding interferon or new drug development? J Hepatol. 2022 Jun;76(6):1249-1262.

Xu, F., Song, H., Li, N., and Tan, G. HBsAg blocks TYPE I IFN induced up-regulation of A3G through inhibition of STAT3. Biochem. Biophys. Res. Commun. 2016; 473, 219–223.

Yuen M-F, Lim S-G, Plesniak R, et al. Efficacy and safety of bepirovirsen in chronic hepatitis B infection. N Engl J Med. DOI: 10.1056/NEJMoa2210027.

Yuen MF, Lim YS, Cloutier D, et al., Preliminary 48-week safety and efficacy data for VIR-2218 alone and in combination with pegylated interferon alfa in participants with chronic HBV infection. AASLD, The Liver Meeting 4-8 November 2022.

Yuen MF, Wong GL, Douglas M, et al. Preliminary safety and efficacy of the combination therapy of BRII-835 (VIR-2218) and BRII-179 (VBI-2601) treating chronic HBV injection. APASL Annual Meeting 15-19 February 2023.

Yuen MF, Lim YS, Yoon KT, et al. Safety and efficacy of VIR-2218 with or without pegylated interferon alfa in virally-suppressed participants with chronic hepatitis B virus infection: post-treatment follow up. EASL Congress 21-24 June 2023.

Zhu D, Liu L, Yang D, Fu S, Bian Y, Sun Z, He J, Su L, Zhang L, Peng H, Fu YX. J Immunol.2016 Apr 1;196(7):3079-87.

Zoulim F, Durantel D. Antiviral therapies and prospects for a cure of chronic hepatitis B. Cold Spring Harb Perspect Med. 2015;5(4).

11. APPENDICES

11.1. Appendix 1: Regulatory, Ethical, and Study Oversight Considerations

11.1.1. Regulatory and Ethical Considerations

- This study will be conducted in accordance with the protocol and with the following:
  - Consensus ethical principles derived from international guidelines including the Declaration of Helsinki and Council for International Organizations of Medical Sciences (CIOMS) International Ethical Guidelines
  - Applicable ICH Good Clinical Practice (GCP) Guidelines
  - Applicable laws and regulations
- The protocol, protocol amendments, ICF, Investigator's Brochure, and other relevant documents (e.g., advertisements) must be submitted to an IRB/IEC by the investigator and reviewed and approved by the IRB/IEC before the study is initiated.
- Any amendments to the protocol will require IRB/IEC approval before implementation of changes made to the study design, except for changes necessary to eliminate an immediate hazard to study participants.
- The investigator will be responsible for the following:
  - Providing written summaries of the status of the study to the IRB/IEC annually or more frequently in accordance with the requirements, policies, and procedures established by the IRB/IEC
  - Notifying the IRB/IEC of SAEs or other significant safety findings as required by IRB/IEC procedures
  - Providing oversight of the conduct of the study at the site and adherence to requirements of 21 CFR, ICH guidelines, the IRB/IEC, European regulation 536/2014 for clinical studies (if applicable), and all other applicable local regulations

11.1.2. Finance Disclosure

Investigators and sub-investigators will provide the sponsor with sufficient, accurate financial information as requested to allow the sponsor to submit complete and accurate financial certification or disclosure statements to the appropriate regulatory authorities. Investigators are responsible for providing information on financial interests during the course of the study and for 1 year after completion of the study.

11.1.3. Informed Consent Process

- The investigator or his/her representative will explain the nature of the study to the participant or his/her legally authorized representative and answer all questions regarding the study.
- Participants must be informed that their participation is voluntary. Participants or their legally authorized representative will be required to sign a statement of informed consent that meets the requirements of 21 CFR 50, local regulations, ICH guidelines, Health Insurance Portability and Accountability Act (HIPAA) requirements, where applicable, and the IRB/IEC or study center.
- The medical record must include a statement that written informed consent was obtained before the participant was enrolled in the study and the date the written consent was obtained. The authorized person obtaining the informed consent must also sign the ICF.
- Participants must be re-consented to the most current version of the ICF(s) during their participation in the study.
- A copy of the ICF(s) must be provided to the participant or the participant's legally authorized representative.

11.1.4. Data Protection

- Participants will be assigned a unique identifier by the sponsor. Any participant records or datasets that are transferred to the sponsor will contain the identifier only; participant names or any information which would make the participant identifiable will not be transferred.
- The participant must be informed that his/her personal study-related data will be used by the sponsor in accordance with local data protection law. The level of disclosure must also be explained to the participant who will be required to give consent for their data to be used as described in the informed consent.
- The participant must be informed that his/her medical records may be examined by Clinical Quality Assurance auditors or other authorized personnel appointed by the sponsor, by appropriate IRB/IEC members, and by inspectors from regulatory authorities.

11.1.5. Data Quality Assurance

- All relevant participant data relating to the study will be documented in source documents and appropriately recorded on CRF unless transmitted to the sponsor or designee electronically (e.g., laboratory data). The investigator is responsible for verifying that data entries are accurate and correct by physically or electronically signing the CRF.

- The investigator must maintain accurate documentation (source data) that supports the information entered in the CRF.
- The investigator must permit study-related monitoring, audits, IRB/IEC review, and regulatory agency inspections and provide direct access to source data documents.
- Monitoring details describing strategy (e.g., risk-based initiatives in operations and quality such as Risk Management and Mitigation Strategies and Analytical Risk-Based Monitoring), methods, responsibilities and requirements, including handling of noncompliance issues and monitoring techniques (central, remote, or on-site monitoring) are provided in the Monitoring Plan.
- The sponsor or designee is responsible for the data management of this study including quality checking of the data.
- The sponsor assumes accountability for actions delegated to other individuals (e.g., Contract Research Organizations).
- Study monitors will perform ongoing source data verification to confirm that data entered into the CRF by authorized site personnel are accurate, complete, and verifiable from source documents; that the safety and rights of participants are being protected; and that the study is being conducted in accordance with the currently approved protocol and any other study agreements, ICH GCP, and all applicable regulatory requirements.
- Records and documents, including signed ICFs, pertaining to the conduct of this study must be retained by the investigator. No records may be destroyed during the retention period without the written approval of the sponsor. No records may be transferred to another location or party without written notification to the sponsor.

11.1.6. Source Documents

- Source documents provide evidence for the existence of the participant and substantiate the integrity of the data collected. Source documents are filed at the investigator's site.
- Data reported on the CRF or entered in the CRF that are transcribed from source documents must be consistent with the source documents or the discrepancies must be explained. The investigator may need to request previous medical records or transfer records, depending on the study. Also, current medical records must be available.
- Source data refer to all information recorded in the original record of certified copy of the clinical study, including clinical findings, observation results and other relevant activity records needed for reconstruction and evaluation of the clinical study.

11.1.7. Study and Site Start and Closure

The study start date is the date on which the clinical study will be open for recruitment of participants.

The sponsor designee reserves the right to close the study site or terminate the study at any time for any reason at the sole discretion of the sponsor. Study sites will be closed upon study completion. A study site is considered closed when all required documents and study supplies have been collected and a study-site closure visit has been performed.

The investigator may initiate study-site closure at any time, provided there is reasonable cause and sufficient notice is given in advance of the intended termination.

Reasons for the early closure of a study site by the sponsor or investigator may include but are not limited to:

- Failure of the investigator to comply with the protocol, the requirements of the IRB/IEC or local health authorities, the sponsor's procedures, or GCP guidelines
- Inadequate recruitment of participants by the investigator
- Discontinuation of further study intervention development

If the study is prematurely terminated or suspended, the sponsor shall promptly inform the Investigators, the IECs/IRBs, the regulatory authorities, and any contract research organization(s) used in the study of the reason for termination or suspension, as specified by the applicable regulatory requirements. The Investigator shall promptly inform the participant and should assure appropriate therapy and/or follow-up.

11.1.8. Publication Policy

The sponsor intends to publish the results of clinical trials regardless of outcome. Results may be published in peer-reviewed journals and/or presented at scientific congresses. The sponsor will comply with the requirements for publication and disclosure of clinical trial information. In accordance with standard editorial and ethical practice, the sponsor will generally support publication of multi-center studies only in their entirety and not as individual site data. Authorship will be determined by mutual agreement based upon International Committee of Medical Journal Editors criteria and guidelines (ICMJE, 2022). Individuals who do not meet authorship criteria do not warrant named authorship. Nonauthor contributors may be acknowledged in the manuscript when published or recognized in a manner consistent with the target journal policy.

11.2. Appendix 2: Clinical Safety Laboratory Tests

The tests detailed in Table 8 will be performed by the central laboratory.

The laboratory reports must be filed with the source documents. Investigators must review the laboratory report, document their review of each laboratory safety report, and record any clinically significant changes occurring during the study in the medical history section or AE section of the CRF. Clinically significant abnormal laboratory findings are those which are not associated with the underlying disease, unless judged by the investigator to be more severe than expected for the participant's condition.

Additional tests may be performed at any time during the study as determined necessary by the investigator or required by local regulations.

Table 8 Protocol-Required Safety Laboratory Assessments

**Hematology**	
Complete blood count with differential, including red blood cell count, hemoglobin, hematocrit, white blood cell count, count and percentage of neutrophils, lymphocytes, eosinophils, basophils, and monocytes, and platelet count	
**Chemistry**	
Albumin	Glucose
Calcium	Sodium
Potassium	Chloride
Creatine kinase	Carbon dioxide/bicarbonate
Blood urea nitrogen (BUN)	Estimated glomerular filtration rate (eGFR)
Creatinine	Uric acid
Alanine aminotransferase (ALT)	Aspartate aminotransferase (AST)
Gamma glutamyl transferase (GGT)	Lactate dehydrogenase (LDH)
Alkaline phosphatase (ALP)	Bilirubin (total and direct)
**Coagulation Parameters**	
International normalized ratio (INR)	Prothrombin time (PT)
**Urinalysis**	
Glucose	Proteins
Red blood cells (RBCs)	
**Pregnancy Testing** (WOCBP only) [1]	
Beta-human chorionic gonadotropin	Urine pregnancy test
**Thyroid Panel** [2]	
Thyroid stimulating hormone (TSH)	T3
T4	
**Anti-nuclear antibodies (ANA)** [2]	
**AFP** [3]	

[1] Blood beta-human chorionic gonadotropin should be conducted at central lab at screening visit, and urine pregnancy test will be conducted after screening. Urine and/or blood pregnancy tests may be conducted at local laboratory if necessary.

[2] Thyroid panel and ANA will be tested every 12 weeks during PEG-IFNα dosing: Screening, Week 12, Week 24, Week 36 and Week 48.

3 AFP will be tested every 6 months during the study: Screening, Week 24, Week 48, Week 72 and NRTI Discontinuation Monitoring Period Week 24.

11.3. Appendix 3 Assessments in Participants Meeting Laboratory ECI Criteria

Additional assessment will be performed in participants who experience ALT elevations meeting laboratory ECI criteria (Section 8.3.7) from Day 1 of dosing to the last follow-up visit. Following the occurrence of event of ECI, the following assessments in Table 9 will be performed, as soon as possible, and no later than one week after the initial ALT value meeting criteria for a laboratory ECI.

The optional assessments in Table 10 may be conducted per investigator discretion as needed. Extended hepatic panel will be tested at central laboratory. Imaging will be performed and reviewed at the investigational site(s) by certified site staff.

Table 9 Assessments in Participants Meeting Laboratory ECI Criteria

**Hematology**	
Complete blood count with differential, including red blood cell count, hemoglobin, hematocrit, white blood cell count, count and percentage of neutrophils, lymphocytes, eosinophils, basophils, and monocytes, and platelet count	
**Chemistry**	
Albumin	Glucose
Calcium	Sodium
Potassium	Chloride
Creatine kinase	Carbon dioxide/bicarbonate
Blood urea nitrogen (BUN)	Estimated glomerular filtration rate (eGFR)
Creatinine	Uric acid
Alanine aminotransferase (ALT)	Aspartate aminotransferase (AST)
Gamma glutamyl transferase (GGT)	Lactate dehydrogenase (LDH)
Alkaline phosphatase (ALP)	Bilirubin (total and direct)
**Coagulation Parameters**	
International normalized ratio (INR)	Prothrombin time (PT)
**Focused Medical History**	
Use of any potentially hepatotoxic concomitant medications, including over the counter medications and herbal remedies	Alcohol consumption and drugs of abuse
Exposure to other potentially hepatotoxic agents including any work-related exposures (eg. Solvents, pesticides, etc.)	Exercise history

Table 10 Optional Assessments in Participants Meeting Laboratory ECI Criteria

**Extended Hepatic Panel**	
Quantitative HBsAg	Ferritin
Quantitative HBV DNA	Blood phosphatidylethanol*
Qualitative HBeAg	Urinary ethyglucuronide*
HAV antibody IgM	Anti-nuclear antibodies
HCV antibody and HCV RNA	Anti-smooth muscle antibodies
HDV antibody or RNA	Anti-LKM1 antibody
HEV antibody IgM	Anti-mitochondrial antibodies
Epstein-Barr Virus antibodies, IgM and IgG	Cytomegalovirus antibodies, IgM and IgG
Ceruloplasmin	
**Imaging**	
Abdominal ultrasound, including Doppler flow, if applicable; CT or MRI acceptable in place of ultrasound if clinically indicated	

CT = computed tomography; HAV = hepatitis A virus; HCV = hepatitis C virus; HDV = hepatitis D virus; HEV = hepatitis E virus; IgG = immunoglobulin G antibody; IgM = immunoglobulin M antibody; LKM1 = liver/kidney microsome-1 antibody; MRI = magnetic resonance imaging; RNA = ribonucleic acid; SLA = soluble liver antigen.
* Not applicable to mainland China due to the unavailability of assays

11.4. Appendix 4: Contraceptive Requirements and Collection and Reporting of Pregnancy Information

Contraceptive Requirements:

WOCBP may be included in this study and include any female participant who has experienced menarche and who is not post-menopausal or permanently sterilized (eg, bilateral tubal occlusion, hysterectomy, bilateral oophorectomy, or bilateral salpingectomy).

WOCBP must be willing to use highly effective methods of contraception 14 days before dose, throughout study participation, and for 48 weeks after last dose of study drug administration. Highly effective methods of birth control result in a low failure rate (ie, less than 1% per year). Birth control methods which are considered highly effective include:

- Established use of combined (estrogen and progestogen containing) oral, intravaginal, or transdermal hormonal methods of contraception associated with inhibition of ovulation **OR** established use of progestogen-only oral, injectable, or implantable hormonal methods of contraception associated with inhibition of ovulation. It is not currently known whether study intervention will impact the effectiveness of hormonal contraceptive methods; therefore, it is recommended to use an additional form of contraception (ie, barrier method) throughout the study and for 48 weeks after last study drug administration.
- Placement of an intrauterine device
- Placement of an intrauterine hormone-releasing system
- Surgical sterilization of male partner (with the appropriate post-vasectomy documentation of the absence of sperm in the ejaculate; for female participants on the study, the vasectomized male partner should be the sole partner for that participant)
- True sexual abstinence from heterosexual contact, when in line with the preferred and usual lifestyle of the participant. Periodic abstinence (e.g., calendar, ovulation, symptothermal, postovulation methods) and withdrawal are not acceptable methods of contraception. Abstinent participants have to agree to use 1 of the above-mentioned contraceptive methods, if they start sexual heterosexual relationships during the study and for up to 48 weeks after last dose of study drug administration
- Barrier method in combination with hormonal contraceptive, as described above

Post-menopausal status is defined as 12 months with no menses without an alternative medical cause. A high FSH level in the post-menopausal range should be used to confirm a post-menopausal state in women not using hormonal contraception or hormone replacement therapy (HRT). In the absence of 12 months of amenorrhea, confirmation with more than one FSH measurement is required.

Females on HRT and whose menopausal status is in doubt must discontinue HRT to allow confirmation of postmenopausal status before study enrollment.

Female participants must also refrain from egg donation and in vitro fertilization from the time of study treatment administration until 48 weeks after study drug administration.

Male participants with female partners of child-bearing potential must agree to meet 1 of the following contraception requirements from the time of study treatment administration until the last follow-up visit.

- Vasectomy with documentation of azoospermia
- Male condom plus partner use of 1 of the contraceptive options listed above for contraception for WOCBP (hormonal contraceptive, intrauterine device)

Male participants must also agree not to donate sperm for the 48 weeks following last study drug administration.

11.5. Appendix 5: Protocol Amendment History

The Protocol Amendment Summary of Changes for the current amendment is located directly before the Table of Contents (TOC).

Amendment 1 (Version 2.0) 31 August 2023

Overall Rationale

The emerging clinical data showed that high anti-HBs titers in participants with chronic HBV infection at the end of treatment (EOT) of BRII-835+PEG-IFNα combination therapy may predict the sustainability of HBsAg seroclearance at 24 weeks post EOT (Yuen, 2023). The presence of HBsAb-secreting specific B cells in peripheral blood of CHB patients may also be an important immunological indicator to predict IFN treatment efficacy (Geng, 2023), suggesting the potential value of humoral response for functional cure. BRII-179, a recombinant virus-like particle (VLP)-based protein immunotherapeutic, is safe and well-tolerated in phase 1b/2a BRII-179-001 and phase 2 BRII-179-835-001 studies, in which robust anti-HBs responses were induced in a proportion of patients receiving BRII-179 or BRII-179 in combination with BRII-835, an siRNA (Ma, 2021; Yuen, 2023). It is hypothesized that patients who responded to BRII-179, especially those with higher anti-HBs titers, may have a less dysfunctional immunological profile to virus and be able to respond better to curative therapies, e.g., BRII-835+PEG-IFNα. This amendment would allow eligible participants who received BRII-179 from BRII-179-835-001 to roll over to this study and evaluate the efficacy of BRII-835+PEG-IFNα combination.

Method of Amendment

The specific areas where changes were made to the protocol are listed below. Wording that has been deleted will be indicated with a strikethrough (ex. ~~Word~~) and new wording will be indicated in Bold type (ex. **Word**).

Amendment Details

Section 1.1 Synopsis

Study Endpoints:
The primary endpoints for this study are to evaluate ~~follows~~: *continued with the original text*
The secondary endpoints for this study are to evaluate ~~follows~~: *continued with the original text*

[redacted]

Study Design:
Cohorts 1-3 (BRII-179 naïve):
A total of up to 75 participants with HBeAg negative or HBeAg positive chronic HBV infection will be randomized at 1:1:1 to the following three cohorts… *continued with the original text*
Cohort 4 (BRII-179 treated):

Participants who completed BRII-179-835-001 study in APAC region (did not include mainland China), previously treated with BRII-179 and did not achieve HBsAg seroclearance, will be eligible to roll over to Cohort 4. Up to 45 participants will be enrolled in the following cohort:

- **Cohort 4: participants will receive 48 doses of PEG-IFNα, each administered every week starting on Day 1 and 13 doses of 100 mg BRII-835, each administered every 4 weeks starting on Day 1.**

NRTI discontinuation:
Participants who meet all of the following NRTI discontinuation criteria for at least two consecutive visits at Week 72 will be eligible to discontinue from NRTI therapy… *continued with the original text*

Number of Participants:
Up to 75 participants are planned to be enrolled in ~~the study~~ **Cohorts 1-3, and up to 45 participants will be enrolled in Cohort 4 (BRII-179-835-001 extension cohort)**.

Randomization and Stratification:
Eligible participants **for Cohorts 1-3** will be randomized 1:1:1 to one of the three cohorts and stratified by HBsAg level at screening.
Randomization does not apply to Cohort 4, the BRII-179-835-001 extension cohort.

Main Criteria for Inclusion:
Cohorts 1-3 (BRII-179 naïve):
Participants aged 18 to 60 years with chronic HBV infection without cirrhosis, effectively treated with NRTI therapy for at least 6 months, with HBV DNA < LLOQ and HBsAg > 100 IU/mL and ≤ 3,000 IU/mL at screening.
Cohort 4 (BRII-179 treated):
Participants who completed BRII-179 treatment, did not achieve HBsAg seroclearance in BRII-179-835-001 study, are continuing NRTI therapy and have HBV DNA < LLOQ at screening.

Investigational Product, Dosage and Mode of Administration:
Cohort 3 and **Cohort 4**: PEG-IFNα 180 mcg administered by SC injection every week and 100 mg BRII-835 administered by SC injection every four weeks

Sample Size Determination:
Up to 75 participants will be randomly assigned in 1:1:1 ration ~~to each of the three cohorts~~ **among Cohorts 1-3**. **Up to 45 participants will be enrolled in Cohort 4**.

Primary Efficacy Analysis:
In addition, **for Cohorts 1-3** combine some two adjacent baseline stratification levels to reduce HBsAg stratification factors from three levels to two levels to perform the stratified Bayesian hierarchical analysis if model is converged using the similar approach as the primary analysis.

For Cohort 4 descriptive statistics will be reported, and comparisons with other cohorts may be conducted.

Interim Analysis and Summaries:
Three interim analyses are planned. The first two interim analyses will be conducted when approximately 45 enrolled participants **of Cohorts 1-3** complete the 48-week treatment and last available participant of all enrolled participants **(Cohorts 1-4)** completes the 48-week treatment, respectively.
The third interim analysis will be conducted when last available participant **of Cohorts 1-3** completes follow-up 24-weeks post end of treatment.

Final Analysis:
The final analysis will be performed when last available participant **of Cohorts 1-4** completes the last scheduled visit according to the SoA.

Section 1.2 Schema

Updated Figure 1 to reflect the study scheme including a new Cohort 4

Section 1.3.1 Schedule of Assessment (SoA) Dosing Period and Follow-up Period

Study Visit Week	~~Rand~~	W56 **(FU1)**	W64 **(FU2)**	W72 **(FU3)**/EOS
Study Visit Day ± Visit Window	D1	D393 ± 5	D449 ± 5	D505 ± 7

Study Stage	Dosing Period			
Study Visit Week		W2	*… each dosing visit*	W44
Study Visit Day ± Visit Window	D1	D15 ± 3	*… each dosing visit*	D309 ± 3
**Participant instructed to complete dosing card**	**X**	**X**	**X**	**X**

Footnote b: If a participant permanently discontinues treatment prior to Week 48, EOT assessments should be performed within 14 days post the last dose of study drugs (see Section 7.1) **and continue the visit in follow-up period (see Section 8.1.5)**.

Footnote i: Blood pregnancy test should be conducted at central lab at screening visit, ~~urine pregnancy test and blood pregnancy test (if necessary) at other time points will be conducted at local laboratory~~ **and urine pregnancy test will be conducted after screening. Urine and/or blood pregnancy tests may be conducted at local laboratory if necessary**.

Footnote k: Eligible participants **of Cohorts 1-3** will be randomized 1:1:1 to ~~Cohorts 1, 2, or 3~~ **each of the three cohorts** within 48 hours prior to study drug administration on Day 1. **Randomization dose not apply to participants enrolled in Cohort 4**.

Footnote l: BRII-835 will be given to those who ~~randomized to Cohorts 2/3 at the 2 dose levels~~ **are assigned to Cohort 2 at 200 mg every 4 weeks, and to those who are assigned to Cohorts 3 or 4 at 100 mg every 4 weeks**. Please refer to Section 6.1.3.4.

Footnote n: BRII-835 PK **and ADA** samples **will be collected in participants assigned to Cohorts 2 and 3** (referred to Section 8.5). Predose **PK** sampling should be conducted ≤ 2 hours prior to dosing. Postdose **PK** sampling should be conducted **between** ≥ 2 hours and ≤ 12 hours after dosing.

[redacted]

[redacted]

Footnote s: Participants will be instructed to bring ~~NRTI and~~ **all used syringes of** PEG-IFNα **without disposing the remaining medication** ~~(including all empty containers)~~ to the trial site at every visit, as applicable~~, beginning with the screening visit~~. Adherence **of NRTI can** ~~will~~ be assessed by counting of empty containers and unused medication.

<u>Section 1.3.2 Schedule of Assessment (SoA) NRTI Discontinuation Monitoring Period</u>

Study Stage	NRTI Discontinuation Monitoring Period			
Study Visit Week		W4	*… each visit*	W24/EOS
Study Visit Day ± Visit Window	D1	D29 ± 3	*… each visit*	D169 ± 7
**Blood sample for qualitative HBeAg**		**X**	**X**	**X**

[redacted]

<u>Section 2.2.2 Summary of Clinical Experience</u>

The available antiviral data in Parts D-F showed that greater reductions in mean serum $\log_{10}$ HBsAg were observed at nadir in participants who had concurrent treatment of BRII-835+PEG-IFNα for ≥ 24 weeks compared with BRII-835 alone, concurrent initiation of BRII-835+PEG-IFNα for 12 weeks, or 12 weeks of BRII-835+PEG-IFNα after 12 weeks of BRII-835 lead-in. More than two-thirds of participants (67%) achieved an absolute HBsAg level < 100 IU/mL at Week 12 and most (89%) achieved an absolute HBsAg level < 100 IU/mL at Week 24. It is

noticeable that concurrent initiation of BRII-835 and PEG-IFNα combination achieved the greatest reductions in HBsAg through Week 48; and longer duration of concurrent BRII-835 with PEG-IFNα resulted in lower HBsAg levels, and 30.8% (4/13) achieved HBsAg loss with anti-HBs seroconversion (with anti-HBs >10 mIU/mL) at Week 48 (Yuen, 2022). **More recently, the emerging data in Parts D-F of 24 weeks post end of treatment (EOT) revealed a strong association between anti-HBs titers at end of treatment and the durability of HBsAg seroclearance. Among participants who had HBsAg seroclearance by EOT, all participants (4/4) with anti-HBs levels > 500 mIU/mL at EOT had sustained HBsAg seroclearance at 24 weeks post EOT; while all participants (3/3) with anti-HBs < 100 mIU/mL at EOT experienced HBsAg rebound (Yuen, 2023).**

Study BRII-179-835-001 (VIR-2218-1007) is an ongoing Phase 2 open-label, randomized parallel-group study in adult participants with chronic HBV infection without cirrhosis who are on NRTI therapy. **This study is conducted in APAC countries and regions, including Australia, New Zealand, Singapore, Thailand, Hong Kong, South Korea, and Taiwan**.

As of the end of July 2023, all participants have completed study participation. More than 65% of the participants completed the last dose of BRII-835 treatment for at least 12 months.

Section 2.4 Rationale for BRII-835 and PEG-IFNα Combination Therapy for the Treatment of Chronic HBV Infection

The recent study evaluating the combination of BRII-835 (VIR-2218) and PEG-IFNα in CHB patients under NRTI background therapy demonstrated a proof of principle that concurrent initiation of BRII-835 and PEG-IFNα therapy (48 weeks) resulted in enhanced responses in its HBsAg reductions, and notable rates of HBsAg seroclearance with anti-HBs seroconversion by EOT (30.8% ,4/13) (Yuen, 2022). **The 24-week follow-up data further demonstrated the correlation between durability of HBsAg seroclearance post treatment and titer of anti-HBs antibody (>500 mIU/mL) at EOT (Yuen, 2023).** These preliminary data strongly suggest that the siRNA can unlock the potential of immunomodulator PEG-IFNα, together in combination may increase the rate of functional cure for chronic HBV infection. **It also suggested the potential of anti-HBs response as an on-treatment biomarker of off-treatment sustained response**.

Section 3.2.1 Primary Endpoints

The primary endpoints for this study are to evaluate ~~follows~~: *continued with the original text*

Section 3.2.2 Secondary Endpoints

The secondary endpoints for this study are to evaluate ~~follows~~: *continued with the original text*

[redacted]

[redacted]

Section 4.1 Overall Design

Participants with HBeAg negative or HBeAg positive chronic HBV infection are eligible to participate in **Cohorts 1-3 of** this study. Eligible participants will be stratified by their serum HBsAg level at screening and randomized 1:1:1 to ~~Cohorts 1, 2, or 3~~ **each of the three cohorts**. Up to 75 participants are planned **for Cohorts 1-3.**

In addition, this study will evaluate the efficacy of BRII-835 and PEG-IFNα combination in participants previously treated with BRII-179, a therapeutic recombinant HBV vaccine. Eligible participants who completed BRII-179-835-001 study will roll over to Cohort 4. Up to 45 participants will be enrolled.

All participants will remain outpatient during study participation and will be followed for the safety, tolerability, and antiviral activity after completion of study intervention. The assessments and procedures performed at each visit are further described in the SoA (Section 1.3).

Participants will be assessed by the investigator for their eligibility to discontinue NRTI therapy at Week 72. Participants who meet the NRTI discontinuation criteria may enter the NRTI discontinuation monitoring period for an additional 24 weeks. Participants who do not meet the NRTI discontinuation criteria will continue NRTI therapy and will end from the study.

The total duration of study participation for each participant will include screening period (up to 4 weeks), dosing period (48 weeks), follow-up period (24 weeks), and NRTI discontinuation monitoring period (24 weeks when applicable). **The study design schema can be found in Section 1.2**.

Section 4.1.1 Planned Cohorts and Dosing

Cohorts 1-3 (BRII-179 naïve):

Eligible participants **of Cohorts 1-3** will be stratified by their serum HBsAg level at screening (>100 IU/mL and ≤ 500 IU/mL, > 500 and ≤ 1,500 IU/mL, or > 1,500 and ≤ 3,000 IU/mL) and randomized to receive the study intervention per the following dose regimens. ~~Participants enrolled will remain outpatient during study participation.~~

- Cohort 1: Each participant will receive 48 doses of PEG-IFNα 180 mcg administered SC every week starting on Day 1
- Cohort 2: Each participant will receive 48 doses of PEG-IFNα 180 mcg administered SC every week and 13 doses of BRII-835 200 mg administered SC every 4 weeks, both starting on Day 1
- Cohort 3: Each participant will receive 48 doses of PEG-IFNα 180 mcg administered SC every week and 13 doses of BRII-835 100 mg administered SC every 4 weeks, both starting on Day 1

Cohort 4 (BRII-179 treated):

Eligible participants who completed BRII-179-835-001 study will be assigned to Cohort 4 and receive the following study intervention.

- **Cohort 4: Each participant will receive 48 doses of PEG-IFNα 180 mcg administered SC every week and 13 doses of BRII-835 100 mg administered SC every 4 weeks, both starting on Day 1**

Section 4.2 Scientific Rationale for Study Design

The preliminary end of therapy data in VIR-2218-1001 study also showed higher HBsAg loss rate (40%) could be achieved compared to historical PEG-IFNα data in patients with lower baseline HBsAg (<1,500 IU/mL) that received 48 weeks concurrent initiation of BRII-835 and PEG-IFNα combination therapy (Yuen, 2022). **Sustainable HBsAg loss 24 weeks post EOT was observed in participants receiving 48-week BRII-835 and PEG-IFNα combination therapy regardless of their baseline HBsAg levels or HBeAg status (Yuen, 2023).**

Rationale for Cohort 4:

The most recent emerging data demonstrated that anti-HBs response were associated with the sustainability of HBsAg seroclearance post EOT in CHB patients receiving BRII-835+PEG-IFNα combination therapy. In this study, all 7/13 subjects achieved HBsAg seroclearance after 48-week combination therapy have anti-HBs Ab seroconversion, and all of them (4/4) with anti-HBs titers > 500 mIU/mL at EOT had sustained HBsAg seroclearance at 24 weeks post EOT; while participants (3/3) with anti-HBs titers < 100 mIU/mL at EOT experienced HBsAg rebound, suggesting high anti-HBs titers at EOT are correlated with the persistence of seroclearance (Yuen, 2023). Additionally, CHB patients with anti-HBs-producing B cells detected in PBMC at baseline may be an important immunological indicator to predict IFN treatment efficacy, showing a higher chance to reach functional cure to PEG-IFNα treatment (Geng, 2023). These findings suggested that the potential of potent humoral immune response to HBsAg in CHB patients for achieving and maintaining sustained functional cure.

BRII-179, a recombinant virus-like particle (VLP)-based protein immunotherapeutic consisting of three HBV surface antigens (PreS1, PreS2, and S), has demonstrated to be safe/well-tolerated and induce HBV-specific humoral and cellular immune responses in a proportion of CHB patients in phase 2 studies. In phase 1b/2a BRII-179-001 study, anti-HBs responses were induced in >30% patients receiving four doses of BRII-179 admixed with or without IFN-α as co-adjuvant (Ma, 2021). A more robust anti-HBs responses were observed in participants when received nine doses of BRII-835 + BRII-179 with or without IFN-α co-adjuvant, demonstrating a positive anti-HBs antibody response in greater than 50% participants with ≥ 40% participants had anti-HBs titers > 100 mIU/ml (Yuen, 2023).

Therefore, it is hypothesized that CHB patients previously showing activated immune responses, especially those with enhanced anti-HBs response in BRII-179-835-001 study,

may have a less dysfunctional immunological profile to viral antigens and be able to respond better to the curative immunotherapy therapy such as BRII-835+PEG-IFNα.

Section 4.3 Justification of Dose

~~Three parallel cohorts~~ **Cohorts 1-4** are planned for this study to evaluate 180 mcg weekly PEG-IFNα treatment alone for 48 weeks and 180 mcg weekly PEG-IFNα treatment in combination with two dose levels of BRII-835 at 100 mg or 200 mg administered every 4 weeks for 13 doses, respectively.

All subjects receiving 100 mg of BRII-835 achieved significant HBsAg reductions and the dose has been well tolerated. Therefore, in Cohort 4, 100 mg BRII-835 will continue to be the dose to be evaluated.

Section 5.1 Inclusion Criteria

Cohorts 1-3 (BRII-179 naïve): *continued with the original text*

Cohort 4 (BRII-179 treated):

1. **A signed and dated written Informed Consent Form (ICF) prior to study participation.**
2. **Age ≥ 18 years (or age of legal consent, whichever is older).**
3. **Body mass index (BMI) ≥ 18 kg/m^2 and ≤ 32 kg/m^2.**
4. **Completed BRII-179-835-001 study and completed BRII-179 treatment.**
5. **Must be on NRTI therapy consisting of either entecavir, tenofovir disoproxil, tenofovir amibufenamide, or tenofovir alafenamide and with HBV-DNA < LLOQ for at least 6 months before screening (local laboratory results accepted).**
6. **HBsAg > 0.05 IU/mL at screening.**
7. **HBV DNA < LLOQ at screening.**
8. **Serum ALT and AST ≤ ULN at screening.**
9. **Female participants must have a negative pregnancy test or confirmation of postmenopausal status. Post-menopausal status is defined as 12 months with no menses without alternative medical cause. Women of child-bearing potential (WOCBP) must have a negative serum pregnancy test at screening and a negative urine pregnancy test on Day 1, cannot be breast feeding at screening and throughout the study, and must be willing to use highly effective methods of contraception 14 days before study drug administration through 48 weeks after last study drug administration (Section 11.4, Appendix 4). Female participants must also agree to refrain from egg donation and in vitro fertilization from the time of study**

intervention administration through 48 weeks after the last dose of study intervention.

10. **Male participants with female partners of child-bearing potential must agree to meet 1 of the following contraception requirements from the time of study drug administration until 48 weeks after last study drug administration: vasectomy with documentation of azoospermia, or male condom use plus partner use of 1 of the contraceptive options listed for contraception for WOCBP. Male participants must also agree not to donate sperm for the 48 weeks following last study drug administration.**
11. **Agrees to not donate blood during the duration of the study.**
12. **Willing and able to comply with the study procedures and requirements.**

Study screening laboratory tests may be repeated once for values considered to be questionable by investigator with medical monitor approval.

Cohort 4 will only enroll participants from BRII-179-835-001 study in APAC region excluding mainland China.

Section 5.2 Exclusion Criteria

1. Current or prior treatment with:
   a) Small interfering RNAs or antisense oligonucleotides within 12 months prior to screening
   b) Interferon-based therapy within 12 months prior to screening
   c) Telbivudine within 6 months prior to screening
   d) Myelotoxic drugs such as systemic cytostatic agents (i.e., cancer chemotherapeutic medications) within 30 days before study drug administration at the Day 1 visit
   e) Systemic immunosuppressant (including but not limited to corticosteroids at a prednisolone equivalent dose > 40 mg/day) within 30 days before study drug administration at the Day 1 visit
   f) Granulocyte colony-stimulating factor (G-CSF), granulocyte-macrophage colony-stimulating factor (GM-CSF), recombinant human interleukin-11 (oprelvekin), or erythropoietin (EPO) within 6 months prior to screening
   g) Any HBV therapeutic vaccines prior to screening (applicable to Cohorts 1-3 only)

5. History or evidence of drug or alcohol abuse ~~(80 g of alcohol/day for men and 40 g of alcohol/day for women)~~ as assessed by the Investigator, within the 12 months before screening.

Section 6.1.2 Study Drug Dose and Administration

Cohort Name	Cohorts 1, 2, ~~and~~ 3**, and 4**	Cohort 2	Cohort 3 **and 4**
Intervention Name	PEG-IFNα (Pegasys, brand name Pegasys®)	BRII-835 (VIR-2218)	BRII-835 (VIR-2218)
Dose Level	180 mcg every week	200 mg every 4 weeks	100 mg every 4 weeks
Dose Formulation	Solution for Injection	Solution for Injection	Solution for Injection
Unit Dose	180 mcg/ 0.5 mL<br>135 mcg/ 0.5 mL	200 mg/mL	200 mg/mL
Pack Volume	0.5 mL/ pre-filled syringe	0.5 mL/ vial	0.5 mL/ vial
Route of Administration	SC injection	SC injection	SC injection

Section 6.1.3.4 Study Drug Administration

Participants enrolled in Cohort 2 ~~or Cohort 3~~ **3, or 4** will receive BRII-835 at the assigned dose levels once every four weeks in the clinic.

Section 6.1.3.5 Study Drug Accountability

An IRT will be used to manage the IMPs supply. The initial site supply will be triggered **either** by site initiation ~~register~~ **or activation registration** in IRT**, or will be requested by CRO or site personnel manually (details refer to the Pharmacy Manual).**

Section 6.2 Randomization and Blinding

Up to 75 participants will be randomly assigned in 1:1:1 ratio ~~to each of the three cohorts~~ **among Cohorts 1-3** using an IRT system.

Randomization does not apply to Cohort 4 (BRII-179-835-001 extension cohort).

Section 6.3 Dose Adjustment Criteria

The dose of BRII-835 will not be adjusted once **assigned** ~~randomized~~ to Cohort 2 ~~or~~ Cohort 3, **or Cohort 4**.

Section 6.6.2 NRTI Treatment Compliance

Every effort should be made to ensure good adherence with NRTI therapy. ~~Participants will be instructed to bring all empty containers of NRTI including unused medications to the trial site at every visit, as applicable, for adherence assessment.~~ Adherence of NRTI **can** ~~will~~ be assessed by counting of empty containers and unused medications at every relevant visit**, when applicable**. ~~Every effort should be made to ensure good adherence with NRTI therapy.~~

Section 8.1.1 Screening

Efforts should be made to collect the prior HBV treatment history as complete as possible since the initial diagnosis of chronic HBV infection. **History of participation of the previous study will be collected in participants rolled over from BRII-179-835-001.**

Section 8.1.2 ~~Randomization (~~Day 1~~)~~

Participant eligibility must be confirmed prior to ~~randomization~~ **enrollment** and study drug administration on Day 1. Eligible participants **of Cohorts 1-3** will be randomized 1:1:1 to ~~Cohorts 1, 2, or 3~~ **each of the three cohorts** within 48 hours prior to study drug administration at the Day 1 visit. **Eligible participants of the previous study BRII-179-835-001 will be assigned to Cohort 4.** Eligible participants will receive their first dose of ~~BRII-835~~ **study drug(s)** and will be **instructed of proper self-injection techniques for further PEG-IFNα administration at home and completion of dosing card. Participants will be** assessed on physical examination (including vital sign measurement), clinical laboratory tests, and other assessments per the SoA. Participants will have the blood sample collection prior to study drug administration unless **otherwise specified**.

Section 8.1.3 Dosing Period

Participants enrolled in Cohort 2, ~~and~~ Cohort 3**, and Cohort 4** will receive BRII-835 administered SC at the assigned dose levels every 4 weeks, i.e., at Day 1, Week 4, Week 8, Week 12, Week 16, Week 20, Week 24, Week 28, Week 32, Week 36, Week 40, Week 44 and Week 48 in the clinic.

Participants enrolled in Cohort 2, ~~and~~ Cohort 3**, and Cohort 4** will remain in the clinic at least one hour after each BRII-835 administration to be observed for rare adverse reactions.

Section 8.1.5 Follow-up Period

If a participant discontinues study treatment before the planned EOT(Week 48) and does not withdraw from study participation, he/she will return to the investigational site every 8 weeks upon EOT for the specified assessments (safety follow-up) per the SOA.

Section 8.2.1 Antiviral Activities

Assessment of antiviral activity will include quantitative HBV DNA, quantitative HBsAg, quantitative anti-HBs, qualitative HBeAg, and ~~quanlitative~~ **qualitative** anti-HBe.

Section 8.5 Pharmacokinetic (PK) Assessments

Table 4 footnote b: ~~At~~ **Between** ≥ 2 hours and ≤ 12 hours after dosing

Section 9.2 Estimand

Primary Estimand 1

- Treatment: Cohort 1, 2, 3, **and 4**. Estimation of the within-group response rate;

Primary Estimand 2

- Treatment: Cohort 1, 2, 3 **and 4**. Estimantion of the within-group response rate;

Secondary Estimand(s)

- Treatment: Cohort 1, 2, 3 **and 4**;

Section 9.3 Sample Size Determination

Up to 75 participants will be randomly assigned in 1:1:1 ration to each of ~~the three cohorts~~ **Cohorts 1-3**.

Up to 45 participants will be enrolled in Cohort 4.

No formal sample size is calculated due to the exploratory nature of Cohort 4.

Table 5 95% Credible Interval of Response Rate by Sample Size **for Cohorts 1-3**

Section 9.4 Analysis Sets

Table 7 Analysis Sets

Population	Description
Screened Analysis Set	All participants who signed the ICF
Full Analysis Set (FAS)	All participants ~~randomly~~ assigned to study intervention
Safety Analysis Set (SAS)	All participants ~~randomly~~ assigned to study intervention and who take at least 1 dose of study drug. Participants will be analyzed according to the study intervention they received
Modified Intent-To-Treat (mITT)	All participants ~~randomly~~ assigned to study intervention, who take at least 1 dose of study drug, with at least 1 postbaseline visit through Week 48. Participants will be analyzed according to the study intervention to which they were randomized **for Cohorts 1- 3, and**

	**for Cohort 4 participants will be analyzed according to the study intervention specified in the protocol.**
Per-Protocol (PP)	All participants in the mITT set without important protocol deviations considered to impact the interpretation of the primary efficacy endpoints. Protocol deviations will be reviewed to generate the list of participants with significant deviations to be excluded from the PP analysis set. The PP exclusion criteria will be finalized prior to releasing the database.
Pharmacokinetic Analysis Set (PAS)	All participants who received BRII-835, had a at least 1 evaluable PK concentration, and had no protocol deviations thought to impact on the PK data analysis.
ADA Analysis Set	All participants who received BRII-835 and had at least 1 non-missing ADA result from the ADA assay after first dose of the study drug. Participants will be analyzed according to the treatment actually received.
[redacted]	[redacted]

Section 9.6.1.1 Primary Efficacy Analysis

A Bayesian hierarchical model (Yuen, 2022; Jones, 2011) as primary analysis will be used to estimate the posterior probability of response rate for each cohort **of Cohorts 1-3** by incorporating the baseline HBsAg level stratification factor.

For each cohort of Cohorts 1-3, the posterior distribution of response rate $P(p_g|data)$, g=1,2,3 was derived for each stratum using the model specified above.

The same reduced model will be used to estimate the posterior distribution of Cohort 4 as no stratification factor is planned in this Cohort.

In addition, **for Cohorts 1-3** combine some two adjacent baseline stratification levels to reduce HBsAg stratification factors from three levels to two levels (HBsAg >100 IU/mL and HBsAg ≤1,500 IU/mL versus HBsAg >1,500 IU/mL and HBsAg ≤3,000 IU/mL; HBsAg >100 IU/mL and HBsAg ≤500 IU/mL versus HBsAg >500 IU/mL and HBsAg ≤3,000 IU/mL) to perform the stratified Bayesian hierarchical analysis if model is converged using the similar approach as the primary analysis.

Section 9.6.1.2 Secondary Endpoints Analysis

- **Proportion of participants with HBsAg loss at Week 48**

The analyses for the ~~three~~ **four** endpoints are similar to the primary analysis of co-primary endpoint Proportion of participants with HBsAg loss at 24 weeks post end of treatment and will be based on mITT analysis set.

For Cohorts 1-3 change from baseline in HBsAg to all scheduled visits in log base 10 scale will be statistically analyzed using a Mixed Model for Repeated Measures model.

In addition, **for Cohorts 1-3** a Cox proportional hazard regression model where the estimate of the hazard ratio for treatment (each BRII-835 dose cohort vs PEG-IFNα monotherapy cohort) will be performed, its 95% CI, and p value will be provided if the model is converged.

Section 9.7 Interim Analysis

Three interim analyses are planned. The first two interim analyses will be conducted when approximately 45 enrolled participants **of Cohorts 1-3** complete the 48-week treatment and last available participant of all enrolled participants **(Cohorts 1-4)** complete the 48-week treatment, respectively.

The third interim analysis will be conducted when last available participant **of Cohorts 1-3** completes follow-up 24 weeks post end of treatment.

Section 9.8 Final Analysis

The final analysis will be performed when last available participant **of Cohorts 1-4** completes the last scheduled visit according to the SoA.

Section 11.2 Appendix 2 Clinical Safety Laboratory Tests

Table 8 footnote 1: Blood beta-human chorionic gonadotropin should be conducted at central lab at screening visit, ~~urine pregnancy test and blood pregnancy test (if necessary) at other time points will be conducted at local laboratory~~ **and urine pregnancy test will be conducted after screening. Urine and/or blood pregnancy tests may be conducted at local laboratory if necessary**.

Section 11.3 Appendix 3 Assessments in Participants Meeting Laboratory ECI Criteria

Table 10 Optional Assessments in Participants Meeting Laboratory ECI Criteria

Blood phosphatidylethanol*	Urinary ethyglucuronide*

*** Not applicable to China mainland due to the unavailability of assays**
