# Supplementary material for: Profile and Predictors of Hepatitis B Surface Antigen Seroreversion Following PEG-IFNα with or Without Therapeutic Vaccine BRII-179 or siRNA Elebsiran: A Post Hoc Pooled Analysis of Two Randomized Controlled Trials in Patients with Chronic Hepatitis B Virus Infection
Source: Vaccines (Basel). 2026 Oct 1;14(10):868. doi: 10.3390/vaccines14100868 (PMC13709818; doi:10.3390/vaccines14100868)
Supplement: Supplementary file 1 [file vaccines-14-00868-s001.zip › File_S4_ENSURE_Statistical_Analysis_Plan_and_Amendments.pdf]

STATISTICAL ANALYSIS PLAN

**Study Title:**	A Phase 2 Multicenter, Randomized, Open-label Study to Investigate the Efficacy and Safety of BRII-835 (VIR-2218) and Pegylated Interferon Alpha (PEG-IFNα) Combination Therapy for the Treatment of Chronic Hepatitis B Virus (HBV) Infection
**Name of Test Drug:**	BRII-835 (VIR-2218); Pegylated Interferon Alpha (PEG-IFNα)
**Study Number:**	BRII-835-002
**Protocol Version (Date):**	2.0 (31 August 2023)
**Analysis Type:**	Phase 2
**Analysis Plan Version:**	1.0
**Analysis Plan Date:**	
**Analysis Plan Author(s):**	[redacted]

CONFIDENTIAL AND PROPRIETARY INFORMATION

SIGNATURE PAGE

Reason for Signature	Name	Signature	Date (DDMMMYYYY)
Author	[redacted]		
Therapeutic Area Head (or delegate)			
Biometrics Head (or delegate)			

TABLE OF CONTENTS

TABLE OF CONTENTS 3
LIST OF ABBREVIATIONS 5
1. INTRODUCTION 7
1.1. Study Objectives 7
1.2. Study Design 7
1.3. Sample Size and Power 10
2. TYPE OF PLANNED ANALYSIS 13
2.1. Interim Analyses 13
2.1.1. Dose Escalation Analysis 13
2.2. Final Analysis 13
3. GENERAL CONSIDERATIONS FOR DATA ANALYSES 14
3.1. Key Definitions 15
3.2. Analysis Sets 16
3.2.1. Screened Analysis Set 17
3.2.2. Full Analysis Set (FAS) 17
3.2.3. Safety Analysis Set (SAS) 17
3.2.4. Modified Intent-To-Treat Analysis Set (mITT) 17
3.2.5. Per Protocol Set (PP) 17
3.2.6. Pharmacokinetic Analysis Set (PAS) 18
3.2.7. ADA Analysis Set 18
3.2.8. PBMC Analysis Set 18
3.3. Subject Grouping 18
3.4. Strata and Covariates 18
3.5. Subgroup analysis 18
3.6. Multiple Comparisons 19
3.7. Missing Data and Outliers 19
3.7.1. Missing Data 19
3.7.2. Outliers 19
3.8. Data Handling Conventions and Transformations 19
3.9. Analysis Visit Windows 21
3.9.1. Selection of Data in the Event of Multiple Records in an Analysis Visit Window 25
4. SUBJECT DISPOSITION 26
4.1. Subject Enrollment and Disposition 26
4.1.1. Duration of Exposure to Study Drug 27
4.1.2. Adherence to Study Drug 27
4.2. Protocol Deviations 28
5. BASELINE CHARACTERISTICS 29
5.1. Demographics and Baseline Characteristics 29
5.2. Other Baseline Characteristics 29
5.3. Medical History 29
6. EFFICACY ANALYSES 30
6.1. Primary Efficacy Endpoint 30

6.1.1. Definition of the Primary Efficacy Endpoint: 30
6.1.2. Estimand 30
6.1.3. Analysis methods for the primary endpoints 32
6.2. Secondary Efficacy Endpoints 34
6.2.1. Analysis Methods for Secondary Efficacy Endpoint 34
6.2.2. Exploratory Endpoints: 35
7. SAFETY ANALYSES 36
7.1. Adverse Events and Deaths 36
7.1.1. Adverse Event Dictionary 36
7.1.2. Adverse Event Severity 36
7.1.3. Relationship of Adverse Events to Study Drug 36
7.1.4. Serious Adverse Events 36
7.1.5. Treatment-Emergent Adverse Events 36
7.1.5.1. Definition of Treatment-Emergent Adverse Events 36
7.1.5.2. Incomplete Dates 36
7.1.6. Summaries of Adverse Events and Deaths 37
7.1.6.1. Summaries of AE Incidence by Severity 37
7.2. Laboratory Evaluations 39
7.2.1. Summaries of Numeric Laboratory Results 39
7.2.2. Graded Laboratory Values 39
7.2.2.1. Treatment-Emergent Laboratory Abnormalities 40
7.2.2.2. Summaries of Laboratory Abnormalities 40
7.2.3. Liver-related Laboratory Evaluations 40
7.3. Vital Signs 41
7.4. Prior and Concomitant Medications 41
7.4.1. Prior Medications 41
7.4.2. Concomitant Medications 42
7.5. Electrocardiogram Results 43
7.6. Other Safety Measures 43
8. ADA 44
9. CHANGES FROM PROTOCOL-SPECIFIED EFFICACY ANALYSES 45
9.1. Changes From Protocol-Specified Efficacy Analyses 45
9.2. Changes From Protocol-Specified Safety Analyses 45
10. PHARMACOKINETIC EVALUATION/ANALYSIS 46
11. SOFTWARE 47
12. SAP REVISION 48

LIST OF ABBREVIATIONS

ADA	anti-drug antibody
AE	adverse event
ALT	alanine aminotransferase
AST	aspartate aminotransferase
BLQ	below the limit of quantitation
BMI	body mass index
CFR	Code of Federal Regulations
CI	confidence interval
CK	creatine kinase
CRF	case report form
CSR	clinical study report
CTCAE	Common Toxicity Criteria for Adverse Events
ECG	Electrocardiogram
FAS	Full Analysis Set
Hb	Hemoglobin
HBV	hepatitis B virus
HLT	high-level term
ICH	International Conference on Harmonization (of Technical Requirements for Registration of Pharmaceuticals for Human Use)
ITT	intent to treat
LLT	lower-level term
LOQ	limit of quantitation
MedDRA	Medical Dictionary for Regulatory Activities
mITT	modified intent to treat
NrtI	nucleos(t)ide reverse transcriptase inhibitors
PBMC	peripheral blood mononuclear cell
PK	pharmacokinetic
PP	per protocol
PT	preferred term
Q1, Q3	first quartile, third quartile
SAP	statistical analysis plan
SC	subcutaneously
SD	standard deviation
SI (units)	international system of units
SOC	system organ class
SRC	safety review committee
TEAE	treatment-emergent adverse event

TFLs	tables, figures, and listings
ULN	upper limit of normal
WHO	World Health Organization

1. INTRODUCTION

This statistical analysis plan (SAP) describes the statistical analysis methods and data presentations to be used in tables, figures, and listings (TFLs) in the clinical study report (CSR) for Study BRII-835-002. This SAP is based on the study protocol version 2.0 dated 31 Aug 2023 and the electronic case report form (eCRF). The SAP will be finalized before database finalization. Any changes made after the finalization of the SAP will be documented in the CSR.

This statistical analysis plan (SAP) describes the statistical analysis methods and data presentations to be used in tables, figures, and listings (TFLs) of three interim analyses for Study BRII-835-002. The first two interim analyses will be conducted when approximately 45 enrolled participants of Cohorts 1-3 complete the 48-week treatment and last available participant of all enrolled participants (Cohorts 1-4) completes the 48-week treatment, respectively. If the pace of enrollment is robust, and the primary endpoint proportion of participants with HBsAg loss at Week 48 is expected to occur less or equal to two months between the two interim analyses, the two analyses may be collapsed into one analysis.

1.1. Study Objectives

The primary objective of this study is as follows:

- To evaluate the efficacy and safety of BRII-835 and PEG-IFNα combination therapy in participants with chronic HBV infection

The secondary objectives of this study are as follows:

- To assess the antiviral activity of BRII-835 and PEG-IFNα combination therapy
- To assess BRII-835 pharmacokinetic (PK) exposure and evaluate the anti-drug antibodies (ADA) to BRII-835

The exploratory objectives of this study are as follows

- To evaluate potential biomarkers for host immune responses to therapy
- To evaluate other viral markers of HBV infection

1.2. Study Design

This is a Phase 2 open-label, randomized study of BRII-835 and PEG-IFNα combination therapy in adult participants with chronic HBV infection without cirrhosis receiving NRTI therapy as background therapy. This study is designed to evaluate the efficacy and safety of BRII-835 and PEG-IFNα combination therapy. Two different dose levels of BRII-835 in combination with PEG-IFNα will be evaluated.

Participants with HBeAg negative or HBeAg positive chronic HBV infection are eligible to participate in Cohorts 1-3 of this study. Eligible participants will be stratified by their serum HBsAg level at screening and randomized 1:1:1 to each of the three cohorts. Up to 75 participants are planned for Cohorts 1-3.

In addition, this study will evaluate the efficacy of BRII-835 and PEG-IFNα combination in participants previously treated with BRII-179, a therapeutic recombinant HBV vaccine. Eligible participants who completed BRII-179-835-001 study will roll over to Cohort 4. Up to 45 participants will be enrolled.

All participants will remain outpatient during study participation and will be followed for the safety, tolerability, and antiviral activity after completion of study intervention. The assessments and procedures performed at each visit are further described in the SoA (Section 1.3) of the protocol.

Participants will be assessed by the investigator for their eligibility to discontinue NRTI therapy at Week 72. Participants who meet the NRTI discontinuation criteria may enter the NRTI discontinuation monitoring period for an additional 24 weeks. Participants who do not meet the NRTI discontinuation criteria will continue NRTI therapy and will complete the study.

The total duration of study participation for each participant will include screening period (up to 4 weeks), dosing period (48 weeks), follow-up period (24 weeks), and NRTI discontinuation monitoring period (24 weeks when applicable). The study design schema can be found in Section 1.2 in the protocol.

Planned Cohorts and Dosing

Cohorts 1-3 (BRII-179 naïve):

Eligible participants of Cohorts 1-3 will be stratified by their serum HBsAg level at screening (>100 IU/mL and ≤ 500 IU/mL, > 500 and ≤ 1,500 IU/mL, or > 1,500 and ≤ 3,000 IU/mL) and randomized to receive the study intervention per the following dose regimens.

- Cohort 1: Each participant will receive 48 doses of PEG-IFNα 180 mcg administered SC every week starting on Day 1
- Cohort 2: Each participant will receive 48 doses of PEG-IFNα 180 mcg administered SC every week and 13 doses of BRII-835 200 mg administered SC every 4 weeks, both starting on Day 1
- Cohort 3: Each participant will receive 48 doses of PEG-IFNα 180 mcg administered SC every week and 13 doses of BRII-835 100 mg administered SC every 4 weeks, both starting on Day 1

Cohort 4 (BRII-179 treated):

Eligible participants who completed BRII-179-835-001 study will be assigned to Cohort 4 and receive the following study intervention.

Cohort 4: Each participant will receive 48 doses of PEG-IFNα 180 mcg administered SC every week and 13 doses of BRII-835 100 mg administered SC every 4 weeks, both starting on Day 1

Safety Review Committee: A Safety Review Committee (SRC) for this study will monitor participant safety and consider study-specific data as well as relevant background information about the study drugs and target population under study to inform on study conduct. The primary purpose of the SRC is to protect the safety of participants in this study.

Ad hoc SRC meetings will take place as needed, i.e., for a significant safety event such as criteria for study intervention discontinuation being reached. Decisions to suspend dosing or discontinue individual participants from study intervention will be made according to the predetermined criteria (Section 7 in the protocol). Participants may resume study intervention only following SRC approval. Additionally, the SRC may recommend discontinuation of or changes to the study to the Sponsor. The SRC charter outlining the member composition and operating procedures will be developed before the study initiation.

NRTI Discontinuation

NRTI Discontinuation Criteria

Upon participants completing Week 72 visits, the investigator will evaluate whether participants are eligible for discontinuation of NRTI therapy based on the following NRTI discontinuation criteria:

- HBsAg < LLOQ AND
- HBV DNA < LLOQ AND
- Undetectable HBeAg AND
- ALT ≤ 2x ULN

Participants who meet all of the above criteria for at least two consecutive visits at Week 72 are eligible to discontinue from NRTI therapy. Investigator should confirm participants' eligibility as early as possible within 2 weeks post Week 72 visit. Participants who discontinue NRTI will enter the NRTI Discontinuation Monitoring Period of the study within 1 week of the investigator confirming eligibility. Participants will not take their NRTI medications on the first day of the NRTI Discontinuation Monitoring Period and will be followed for an additional 24 weeks per the SoA (Section 1.3) in the protocol.

NRTI Re-treatment

It is recommended that the Investigator should consider re-treatment with NRTI therapy during the NRTI Discontinuation Monitoring Period if the participant meets any of the following criteria:

- ALT > 10x ULN with HBV DNA ＞LLOQ at the same visit
- ALT 5-10x ULN with HBV DNA ＞LLOQ persisting for ≥ 4 consecutive weeks
- ALT > ULN and HBV DNA > 2,000 IU/mL persisting for ≥ 4 consecutive weeks

- ALT > ULN with total bilirubin > 2x ULN and/or prolongation of prothrombin time (PT) > 2 seconds, and HBV DNA > LLOQ at the same visit
- HBV DNA > 100,000 IU/mL at any visit
- Confirmed HBeAg seroreversion (i.e., HBeAg positive after being HBeAg negative at NRTI discontinuation)
- Any other clinically significant event(s) warranting initiation of NRTI therapy in the opinion of the Investigator

Whether participants should be re-treated with NRTI is at the discretion of the Investigator following good medical judgement. Participants who are re-treated with NRTI therapy should continue to be followed per the SoA (Section 1.3.2) in the protocol for the duration of the 24-week NRTI Discontinuation Monitoring Period.

Participants with HBV DNA increase ≥ 2 $\log_{10}$ IU/mL within a 2-week period should:

- Return to the site for an unscheduled visit to measure HBV DNA levels and liver function tests as soon as possible after the investigator being aware of the HBV DNA increase
- Continue returning to site weekly until HBV DNA levels stabilize (do not increase ≥ 1 $\log_{10}$ IU/mL over a 4-week period or start decreasing for at least 2 consecutive visits) after which they will continue follow-up per the SOA for the NRTI Discontinuation Monitoring Period
- If any unscheduled visits needed as outlined above coincide with an existing visit per the SoA in the protocol, the scheduled assessments should be conducted

1.3. Sample Size and Power

Up to 75 participants will be randomly assigned in 1:1:1 ratio to each of Cohorts 1-3. A Bayesian framework will be used to calculate the sample size. It is assumed that the number of responders follows a Binomial distribution, with a weakly informative prior (Beta (0.5, 0.5)) for the true response rate. The precision for a range of response rates with 95% credible intervals are shown in Table 1:

Up to 45 participants will be enrolled in Cohort 4. No formal sample size is calculated due to the exploratory nature of Cohort 4.

Table 1 95% Credible Interval of Response Rate by Sample Size for Cohorts 1-3

Sample size per cohort	Number of responders	Response rate (%)	95% Credible interval* (%)
15	4	27	8-50
	5	33	13-57

	6	40	18-63
	7	47	25-71
	8	53	29-76
	9	60	36-82
25	6	24	10-41
	7	28	13-46
	8	32	16-51
	9	36	19-55
	10	40	22-58
	11	44	26-63
	12	48	30-67
	13	52	32-70

* 95% highest posterior density interval.

For the 15 participants in each cohort the lower bounds of 95% credible interval exclude the response rate of 10% if observed response rate is greater than 27% and exclude the response rate of 20% if observed response rate is greater than 40% and exclude the response rate of 30% if observed response rate is greater than 53%.

For the 25 participants in each cohort the lower bounds of 95% credible interval exclude the response rate of 10% if observed response rate is greater than 24% and exclude the response rate of 20% if observed response rate is greater than 36% and exclude the response rate of 30% if observed response rate is greater than 48%.The operational characteristics are based on posterior confidence that the true rate exceeds a threshold of interest, as shown in Table . For instance, when 4 out of 15 participants or 6 out of 25 participants are responders, the probability (true response rate>15%) is larger than 85%.

Table 2 Operational Characteristics by Sample Size

**Sample size per cohort**	**Number of responders**	**Probability (True response rate)>15%**	**Probability (True response rate)>20%**	**Probability (True response rate)>25%**	**Probability (True response rate)>30%**	**Probability (True response rate)>35%**
15	1	18.4%	8.5%	3.7%	1.4%	0.5%
	2	45.7%	27.2%	14.4%	7%	3.2%
	3	72.8%	52.6%	34.1%	20.1%	10.9%

	4	89.7%	75.6%	57.9%	40.6%	25.7%
	5	96.8%	90.4%	78.3%	62.7%	45.7%
	6	99.2%	96.6%	90.7%	80.4%	66.6%
	7	99.9%	99.2%	96.9%	92%	83.4%
25	2	16.2%	5.4%	1.6%	0.4%	0.1%
	3	35.7%	16.1%	6%	1.9%	0.5%
	4	58.2%	32.1%	14.9%	5.8%	1.9%
	5	77.4%	52.1%	29.4%	13.8%	5.5%
	6	89.1%	70.5%	46.9%	26.3%	12.1%
	7	95.9%	84.8%	65.5%	42.7%	23.6%
	8	98.5%	92.5%	79.4%	59.8%	38.3%
	9	99.6%	97.4%	89.6%	75%	55.2%
	10	99.9%	99%%	95.2%	86.1%	70.3%
	11	100%	99.7%	98.2%	93.3%	82.6%

2. TYPE OF PLANNED ANALYSIS

2.1. Interim Analyses

Prior to the final analysis, three interim analyses will be conducted. The first two interim analyses will be conducted when approximately 45 enrolled participants of Cohorts 1-3 complete the 48-week treatment and last available participant of all enrolled participants (Cohorts 1-4) complete the 48-week treatment, respectively. If the pace of enrollment is robust, and the proportion of participants with HBsAg loss at Week 48 is expected to occur less or equal to two months between the two interim analyses, the two analyses may be collapsed into one analysis. Endpoints with measurement time after the 48-week treatment except for safety relevant data will not be analyzed in the first two interim analyses.

The third interim analysis will be conducted when last available participant of Cohorts 1-3 completes follow-up 24 weeks post end of treatment.

The sponsor has decided to stop participant enrollment in December 2023, with a total of 55 participants enrolled in Cohorts 1-3. Consequently, the study team has agreed that the first interim analysis will be conducted once these 55 participants complete the 48-week treatment. During this interim analysis, only descriptive statistics will be performed, and no per-protocol (PP) analysis will be conducted.

2.1.1. Dose Escalation Analysis

Not Applicable.

2.2. Final Analysis

The final analysis will be performed when last available participant of Cohorts 1-4 completes the last scheduled visit according to the SoA.

3. GENERAL CONSIDERATIONS FOR DATA ANALYSES

All data collected on the electronic case report form (eCRF) will be presented in the data listings and will be listed by subject identification number (in ascending order), sex, age at screening, screening body weight and visit, where applicable. All summaries will be presented by Cohort.

When reporting descriptive statistics, the following rules will be applied, except for PK concentration data:

- Continuous variables: Descriptive statistics will include the number of non-missing values (n), arithmetic mean, standard deviation (SD), median, first quartile (Q1), third quartile (Q3), minimum and maximum values.

  The minimum and maximum values will be displayed to the same decimal precision as the source data; the Q1, Q3, arithmetic mean and median values will be displayed to one more decimal than the source data; SD value will be displayed to two more decimal than the source data for the specific variable.

  The appropriate precision for derived variables will be determined based on the precision of the data on which the derivations are based, and statistics will be presented in accordance with the abovementioned rules.

  n will be an integer. If no subjects have data at a given time point, then only n=0 will be presented.

  All data will be presented to a maximum of 3 decimal places.

- Categorical variables: Descriptive statistics will include counts and percentages per category. The denominator in all percentage calculations will be the number of subjects in the relevant analysis set with non-missing data, unless specifically stated otherwise. Percentages will be displayed to one decimal place.

  Percentage change from baseline values will be calculated and displayed to 1 decimal place in the listings. In the summaries, the mean and median percentage change from baseline values will be presented to 2 decimal places, the minimum and maximum values will be presented to 1 decimal place and the SD value will be presented to 3 decimal places.

  For categories where all subjects fulfill certain criteria, the percentage value will be displayed as 100. For categories where zero subjects fulfill certain criteria, there will be no percentage displayed. All other percentage displays will use 1 decimal place. Data listings will contain all reported and derived data.

Unless otherwise stated, the following methods will be applied:

- Repeat/unscheduled assessments: Values collected at unscheduled study visits/time points will be allocated to scheduled visit/time points according to the algorithms of analysis visit window together with values collected at scheduled visits/time points and be presented in summary tables. All collected data will be included in the data listings.

- Assessment windows: All assessments will be included in the data listings and no visit windows will be applied to exclude assessments that were performed outside of the protocol specified procedure windows.
- Result display convention: Results will be center aligned in all summary tables and listings. Subject identifiers, visits, and parameter labels may be left-aligned if required.
- Date and time display conventions: The following display conventions will be applied in all outputs where dates and/or times are displayed:
  - Date only: YYYY-MM-DD
  - Date and time: YYYY-MM-DD HH:MM

If only partial information is available, unknown components of the date or time will be presented as 'NK' (not known), i.e., '2016-NK-NK'. Time will be reported in military time.

3.1. Key Definitions

The following definitions will be used:

- Baseline: The baseline value is defined as the last available, valid (quantifiable continuous or categorical value), and non-missing observation for each subject prior to first study drug administration unless otherwise specified. Repeat and unscheduled assessments will be included in the derivation of the baseline values
- Change from Baseline: The change from baseline value is defined as the difference between the result collected/derived at a post-baseline visit/time point and the baseline value.

  The change from baseline value at each post-baseline visit/time point will be calculated for all continuous parameters using the following formula:

  Change from Baseline Value = Result at Visit/Time Point – Baseline Value

  The change from baseline value will only be calculated if the specific post-baseline visit/time point result and the baseline value for the parameter are both available; otherwise, will be treated as missing.
- Study day: The study day of an event is defined as the relative day of the event starting with the date of the first study drug administration (reference date) as Day 1 (there will be no Day 0).

  The study day of events occurring before the first study drug administration will be calculated as:

  Study Day = (Date of Event - Date of First Study Drug Administration)

  For events occurring on or after Day 1, study day will be calculated as:

  Study Day = (Date of Event - Date of First Study Drug Administration) + 1

Study days will only be calculated for events with complete dates and will be undefined for events that are ‘Ongoing’ at the end of the study.

- Relative days compared to an alternative reference point will be calculated similarly, but the alternative starting reference start date will be used instead of the date of the first study drug dosing.
- HBsAg loss: HBsAg changing from positive at baseline to <LLOQ at any post-baseline visit.
- Anti-HBs seroconversion: Anti-HBs changing from negative (<10 IU/L) at baseline to positive (>=10 IU/L) at any post-baseline visit.
- Anti-HBs response: at any post-baseline visit with either (i) post-baseline anti-HBs ≥ 2 IU/L if anti-HBs ＜2 IU/L at baseline or (ii) post-baseline anti-HBs ≥ 5 times the baseline anti-HBs if baseline anti-HBs was ≥ 2 IU/L.
- HBsAg seroconversion: HBsAg loss plus anti-HBs positivity (>=10 IU/L) at any post-baseline visit
- Sustained HBsAg loss: quantitative HBsAg < LLOQ at all visits (scheduled and unscheduled) for ≥ 24 weeks.
- Anti-HBe seroconversion: Anti-HBe changing from negative or missing at baseline to positive at any post-baseline visit.
- HBeAg loss (HBeAg$^+$ subjects only): HBeAg changing from positive at baseline to negative at any post-baseline visit.
- HBeAg Seroconversion (HBeAg+ subjects only): HBeAg loss plus anti-HBe positivity at any post-baseline visit.
- Functional cure: sustained undetectable HBsAg (HBsAg < LLOQ) and sustained suppression of HBV DNA < LLOQ ≥ 24 weeks including all visits (scheduled and unscheduled) after discontinuation of all treatment, including NRTI.
- Modified Functional cure: HBsAg <LLOQ and HBV DNA<LLOQ at each analysis window in the NRTI discontinuation monitoring period. Any observation of HBsAg ^{3} LLOQ or HBV DNA ^{3} LLOQ must be confirmed at a consecutive visit (including unscheduled visits) for the participants to be classed as having lost their sustainability, Participants who have a value of HBsAg ^{3} LLOQ or HBV DNA ^{3}LLOQ at their last visit, which cannot be confirmed due to no further follow up, will be treated as ‘treatment failure’.

The LLOQ for HBsAg is 0.05 IU/mL, and 10 IU/mL for HBV DNA.

3.2. Analysis Sets

Analysis sets define the subjects to be included in an analysis. Analysis sets and their definitions are provided in this section. The analysis set will be identified and included as a subtitle of each table, figure, and listing.

For each analysis set, the number and percentage of subjects eligible for inclusion will be provided.

A listing of exclusion from analysis sets will be provided by subject.

3.2.1. Screened Analysis Set

The Screened Analysis Set will consist of all subjects who sign the informed consent form. The listing of eligibility criteria will be based on this population.

3.2.2. Full Analysis Set (FAS)

All participants assigned to study intervention will be included in Full Analysis Set.

3.2.3. Safety Analysis Set (SAS)

All participants assigned to study intervention and who take at least 1 dose of study drug. Participants will be analyzed according to the study intervention they received. Participants will be analyzed by Cohort, subtotal for Cohort 2-4 and overall.

All safety analyses will be based on Safety Analysis Set.

3.2.4. Modified Intent-To-Treat Analysis Set (mITT)

All participants assigned to study intervention, who take at least 1 dose of study drug, with at least 1 postbaseline visit through Week 48. Participants will be analyzed according to the study intervention to which they were randomized for Cohorts 1- 3, and for Cohort 4 participants will be analyzed according to the study intervention specified in the protocol.

3.2.5. Per Protocol Set (PP)

The Per Protocol Set will include the subset of mITT who fulfil:

- HBsAg ≥ 0.05 IU/mL at baseline
- Total number of dose compliance ≥ 80% and ≤ 120% for BRII-835 and PEG-IFNα
- Have HBsAg data in the analysis visit window for the primary efficacy endpoints analysis
- Had not identified with important (major) protocol deviation that significantly impacts the interpretation of the primary efficacy endpoints

A Per Protocol review meeting will be held to go through the important protocol deviations to identify the PP population prior to database freeze prior to the second and onwards interim analyses and database lock for the final analysis.

PP Set will be used as analysis population of antiviral activity and immunological markers**.**

3.2.6. Pharmacokinetic Analysis Set (PAS)

All participants who received BRII-835, had a at least 1 evaluable PK concentration, and had no protocol deviations thought to impact on the PK data analysis.

3.2.7. ADA Analysis Set

All participants who received BRII-835 and had at least 1 non-missing ADA result from the ADA assay after first dose of the study drug. Participants will be analyzed by Cohort, subtotal for Cohort 2-4 and overall.

3.2.8. PBMC Analysis Set

Participants in Cohort 1, 3, and 4 evaluable for immune profiling will be included in the PBMC Analysis Set. Here "evaluable" refers to whose whole blood is collected and qualified for immune cell and cytokine/chemokine profiling. The analysis for the PBMC data will be based on this set.

3.3. Subject Grouping

For analyses based on the FAS, mITT, SAS and PP Set, subjects will be grouped according to the Cohort.

3.4. Strata and Covariates

Up to 75 participants will be randomly assigned in 1:1:1 ratio among Cohorts 1-3 using an IRT system. Stratification will be based on the following variables:

Screening HBsAg level (>100 IU/mL and ≤500 IU/mL, >500 IU/mL and ≤1,500 IU/mL, >1,500 IU/mL and ≤3,000 IU/mL).

If there are discrepancies in stratification factor values between the IWRS and the clinical database, the values recorded in the clinical database will be used for analyses.

For Cohort 4 no randomization is performed.

3.5. Subgroup analysis

- In addition, for Cohorts 1-3 combine some two adjacent baseline stratification levels to reduce HBsAg stratification factors from three levels to two levels (HBsAg >100 IU/mL and

HBsAg ≤1,500 IU/mL versus HBsAg >1,500 IU/mL and HBsAg ≤3,000 IU/mL; HBsAg >100 IU/mL and HBsAg ≤500 IU/mL versus HBsAg >500 IU/mL and HBsAg ≤3,000 IU/mL) to perform the stratified Bayesian hierarchical analysis if model is converged using the similar approach as the primary analysis.

- Further stratify Cohort 4 into two subgroups (Anti-HBs responder/ Anti-HBs non-responder or HBsAg>=10 IU/L/HBsAg<10 IU/L) based on anti-HBs titer by Week 24 and anti-HBs titer by Week 44 in BRII-179-835-001 study, descriptive statistics for antiviral activity and immunological markers will be presented.

3.6. Multiple Comparisons

NA

3.7. Missing Data and Outliers

3.7.1. Missing Data

In general, missing data will not be imputed unless methods for handling missing data are specified. Exceptions are presented in this document.

For missing last dosing date of study drug, imputation rules are described in Section 4.1.1, the handling of missing or incomplete dates for AE onset is described in Section 7.1.5.2, and for prior HBV and concomitant medications in Section 7.4.

3.7.2. Outliers

Outliers will be identified during the data management and data analysis process, but no sensitivity analyses will be conducted. All data will be included in the data analysis.

3.8. Data Handling Conventions and Transformations

The following conventions will be used for the imputation of date of birth when it is partially missing or not collected:

- If only month and year of birth is collected, then “15” will be imputed as the day of birth
- If only year of birth is collected, then “01 July” will be imputed as the day and month of birth
- If year of birth is missing, then date of birth will not be imputed.

If age at Day 1 is not available for a subject, then age derived based on date of birth and the Day 1 visit date will be used instead. If an enrolled subject was not dosed with any study drug, the

randomization date will be used instead of the Day 1 visit date. For screen failures, the date the first informed consent was signed will be used for the age derivation. Age required for longitudinal and temporal calculations and analyses (e.g age at date of AE) will be based on age derived from date of birth and the date of the measurement or event, unless otherwise specified.

Non-PK data that are continuous in nature but are less than the lower limit of quantitation (LOQ) or above the upper LOQ will be imputed as follows:

- A value that is 1 unit less than the LOQ will be used to calculate descriptive statistics if the datum is reported in the form of "< x" (where x is considered the LOQ). For example, if the values are reported as < 50 and < 5.0, values of 49 and 4.9, respectively, will be used to calculate summary statistics. An exception to this rule is any value reported as < 1 or < 0.1, etc. For values reported as < 1 or < 0.1, a value of 0.9 or 0.09, respectively, will be used to calculate summary statistics.
- A value that is 1 unit above the LOQ will be used to calculate descriptive statistics if the datum is reported in the form of "> x" (where x is considered the LOQ). Values with decimal points will follow the same logic as above.
- The LOQ will be used to calculate descriptive statistics if the datum is reported in the form of "≤ x" or "≥ x" (where x is considered the LOQ).

Plasma concentrations that are below the limit of quantitation (BLQ) will be presented as "BLQ" in the concentration data listing. BLQ concentrations will be treated as 0 at predose time points, and as missing at postdose time points for determination of summary and order statistics. Geometric mean and geometric CV% will not be calculated if the concentrations is BLQ or missing at predose.

The following conventions will be used for the presentation of summary and order statistics:

- If at least 1 subject has a concentration value of BLQ for the time point, the minimum value will be displayed as "BLQ."
- If more than 25% of the subjects have concentration values of BLQ for a given time point, the minimum value will be displayed as "BLQ."
- If more than 50% of the subjects have concentration values of BLQ for a given time point, the minimum and median values will be displayed as "BLQ."
- If more than 75% of the subjects have concentration values of BLQ for a given time point, the minimum and median values will be displayed as "BLQ."
- If all subjects have concentration values of BLQ for a given time point, all order statistics (minimum, median, and maximum) and summary statistics will be displayed as "BLQ."

3.9. Analysis Visit Windows

Subject visits might not occur on protocol-specified days. Therefore, for the purpose of analysis, observations will be assigned to analysis windows. The analysis visit windows for the parameters at different periods are listed in Table 3.1 – Table 3.6

- Prior to NrtI Discontinuation Monitoring Period

Table 3.1 Analysis Visit Windows for HBsAg Prior to NrtI Discontinuation Monitoring Period

Analysis Visit	Nominal Study Day	Lower Limit	Upper Limit
Baseline	1	None	1
Week 4	29	2	43
Week 8	57	44	71
Week 12	85	72	99
Week 16	113	100	127
Week 20	141	128	155
Week 24	169	156	183
Week 28	197	184	211
Week 32	225	212	239
Week 36	253	240	267
Week 40	281	268	295
Week 44	309	296	323
Week 48	337	324	365
Week 56	393	366	421
Week 64	449	422	477

Week 72	505	478	533

One of the primary endpoints is the proportion of participants with HBsAg loss at weeks post actual end of treatment, a participant discontinues study treatment before the planned EOT (Week 48) and does not withdraw from study participation, he/she will return to the investigational site every 8 weeks upon EOT for the specified assessments and the corresponding analysis windows for HBsAg is as follows

Analysis Visit	Reference day	Lower Limit	Upper Limit
EoT	1	None	1
FU Week 8	57	2	85
FU Week 16	113	86	141
FU Week 24	169	142	197

Table 3.2 Analysis Visit Windows for Anti-HBs Prior to NrtI Discontinuation Monitoring Period

Analysis Visit	Nominal Study Day	Lower Limit	Upper Limit
Baseline	1	None	1
Week 16	113	2	141
Week 24	169	142	197
Week 32	225	198	253
Week 40	281	254	309
Week 48	337	310	365
Week 56	393	366	421
Week 64	449	422	477
Week 72	505	478	533

Table 3.3 Analysis Visit Windows for HBV DNA Prior to NrtI Discontinuation Monitoring Period

Analysis Visit	Nominal Study Day	Lower Limit	Upper Limit
Baseline	1	None	1
Week 12	85	2	127
Week 24	169	128	211
Week 36	253	212	295
Week 48	337	296	365
Week 56	393	366	449
Week 72	505	450	533

Table 3.4 Analysis Visit Windows for HBeAg , Anti-HBe Prior to NrtI Discontinuation Monitoring Period

Analysis Visit	Nominal Study Day	Lower Limit	Upper Limit
Baseline	1	None	1
Week 16	113	2	169
Week 32	225	170	281
Week 48	337	282	421
Week 72	505	422	533

Tabel 3.5 Analysis Visit Windows for Hematology, Chemistry and Coagulation Assessments Prior to NrtI Discontinuation Monitoring Period

Analysis Visit	Nominal Study Day	Lower Limit	Upper Limit
Baseline	1	None	1

Week 2	15	2	22
Week 4	29	23	43
Week 8	57	44	71
Week 12	85	72	99
Week 16	113	100	127
Week 20	141	128	155
Week 24	169	156	183
Week 28	197	184	211
Week 32	225	212	239
Week 36	253	240	267
Week 40	281	268	295
Week 44	309	296	323
Week 48	337	324	365
Week 56	393	366	421
Week 64	449	422	477
Week 72	505	478	533

- NrtI Discontinuation Monitoring Period

Table 3.6 Analysis Visit Windows for HBsAg, HBV DNA, HBeAg, Hematology, Chemistry and Coagulation during NrtI Discontinuation Monitoring Period

Analysis Visit	NDMP Day	Lower Limit	Upper Limit
NDMP Baseline	1	None	1
NDMP Week 4	29	2	43

NDMP Week 8	57	44	71
NDMP Week 12	85	72	106
NDMP Week 18	127	107	148
NDMP Week 24	169	149	190

*NDMP: NrtI Discontinuation Monitoring Period ; NDMP baseline is not applicable for HBeAg according to SOA in the protocol

3.9.1. Selection of Data in the Event of Multiple Records in an Analysis Visit Window

Depending on the statistical analysis method, single values may be required for each analysis window. For example, change from baseline by visit usually requires a single value, whereas a time-to-event analysis would not require 1 value per analysis window.

If multiple valid, nonmissing measurements exist in an analysis window, records will be chosen based on the following rules if a single value is needed:

- For baseline, the last nonmissing value on or prior to the first dosing date of study drug will be selected, unless specified differently. If there are multiple records with the same time or no time recorded on the same day, the baseline value will be the average of the measurements for continuous data, or the measurement with the lowest severity (e.g., normal will be selected over abnormal for safety electrocardiogram findings) for categorical data, and the most conservative value will be selected for efficacy data (e.g., negative will be selected over positive for HBeAg and HBsAg, whereas positive will be selected over negative for Anti-HBe and Anti-HBs).

- For post-baseline values:

  — The most conservative value for safety lab parameters (e.g., abnormal will be selected over normal) within the analysis window will be selected

  — If there are more than 1 value within an analysis window, the value collected closet to the nominal day will be selected.

  — If there are 2 records that are equidistant from the nominal day, the later record will be selected.

  — If there is more than 1 record on the selected day, the latest record will be selected; if these measurements were recorded at the same time or no time recorded, the average will be taken for continuous data.

4. SUBJECT DISPOSITION

4.1. Subject Enrollment and Disposition

A summary of subject enrollment will be provided by Cohort for each country/region, investigator and site name and presented in Full Analysis Set. The summary will present the number and percentage of subjects enrolled. For each column, the denominator for the percentage calculation will be the total number of subjects analyzed for that column.

A similar enrollment table will be provided by randomization stratum. The denominator for the percentage of subjects in the stratum will be the total number of enrolled subjects. If there are discrepancies in the value used for stratification assignment between the IWRS and the clinical database, the value collected in the clinical database will be used. A listing of subjects with discrepancies in the value used for stratification assignment between the IWRS and the clinical database at the time of data finalization will be provided.

The randomization schedule used for the study will be provided as an appendix in the CSR.

- A summary of subject disposition will be provided by Cohort. This summary will present the number of subjects screened, subjects who meet all eligibility criteria but not randomized, , , and the number of subjects in each of the categories listed below:
- All screened subject
- Screen Failure
  - Subjects not met eligibility criteria
  - Subjects met eligibility but not enrolled
- Subject enrolled
- All treated subject
- Completed study drug administration
- Did not complete study drug administration with reasons for premature discontinuation of study drug
- Completed study
- Did not complete the study with reasons for premature discontinuation of study

For the status of study drug and study completion and reasons for premature discontinuation, the number and percentage of subjects in each category will be provided. The denominator for the percentage calculation will be the total number of subjects in the Full Analysis Set corresponding to that column. In addition, a flowchart will be provided to depict the disposition.

The following by-subject listings will be provided by subject identification (ID) number (in ascending order), age, sex and screening body weight to support the above summary tables:

- Reasons for premature study drug or study discontinuation
- Reasons for screen failure (will be provided by screened subject ID number in ascending order)

4.1.1. Duration of Exposure to Study Drug

Duration of treatment is calculated as (Date of Last Dose - Date of First Dose + 1) for the completers and calculated as (Discontinued Date-Date of First Dose +1) for subjects with prematurely discontinuation, regardless of any temporary interruptions in study drug administration, and will be expressed in weeks using up to 1 decimal place (e.g., 4.5 weeks). If the last study drug dosing date is missing, the latest date among the clinical visit date, laboratory sample collection date, and vital signs assessment date that occurred during the dosing-treatment period will be used.

Duration of Treatment and Total number of injections for BRII-835 and PEG-IFNα will be summarized, respectively using descriptive statistics (n, mean, STD, Median, Q1, Q3, Min, Max) for Safety Analysis Set.

In addition Total number of injections will be summarized by counts and percentages for Safety Analysis Set.

No formal statistical testing is planned.

A by-subject listing of study drug administration and drug accountability will be provided separately by subject ID number, sex, age, race, body weight at Screening, and visit in chronological order.

4.1.2. Adherence to Study Drug

The level of prescribed adherence to the study drug regimen will be determined by the total doses of study drug taken relative to the total doses of study drug specified by the protocol.

The level of prescribed adherence of BRII-835 and PEG-IFNα will be expressed as a percentage using the following formula:

$$\text{Prescribed Adherence (\%)} = \left(\frac{\text{Total Doses of Study Drug}}{\text{Total Doses of Study Drug Specified by Protocol}}\right) \text{ x } 100$$

Descriptive statistics for the level of prescribed adherence with the number and percentage of subjects belonging to adherence categories (e.g., *{< 80%, ≥ 80 to ≤ 90%, > 90%}*) will be provided by treatment group for the Safety Analysis Set.

No formal statistical testing is planned.

Number of Dose administration will be summarized by Cohort, Cohort 2-4 and overall based on Safety Analysis Set.

4.2. Protocol Deviations

Protocol deviations occurring after subjects entered the study are documented during routine monitoring.The number and percentage of subjects with important protocol deviations, and the total number of important protocol deviations by deviation reason (e.g., nonadherence to study drug, violation of select inclusion/exclusion criteria) will be summarized by Cohort for the Full Analysis Set. In addition a by-subject listing subjects with all protocol deviation will be provided.

5. BASELINE CHARACTERISTICS

5.1. Demographics and Baseline Characteristics

Subject demographic variables (i.e., age, sex, race, and ethnicity) and baseline characteristics (body weight [in kg], height [in cm], body mass index [BMI; in kg/m^2]) will be summarized by Cohort, subtotal for Cohort 1-3 and overall using descriptive statistics for continuous variables and using number and percentage of subjects for categorical variables. The summary of demographic data will be provided for the Full Analysis Set.

A by-subject demographic listing, including the informed consent date, will be provided by subject ID number (in ascending order), sex, age, race, and body weight at Screening.

5.2. Other Baseline Characteristics

Other baseline characteristics include HBsAg, Log10 transformed HBsAg, Anti-HBs, HBeAg at Screening and HBV genotype. These baseline characteristics will be summarized by Cohort, subtotal for Cohort 1-3 and overall using descriptive statistics for continuous variables and using number and percentage of subjects for categorical variables. The summary of these baseline characteristics will be provided for the Full Analysis Set. No formal statistical testing is planned.

A by-subject listing of other baseline characteristics will be provided by subject ID number (in ascending order), sex, age, race, and body weight at Screening.

5.3. Medical History

Medical history will be collected at screening.

Medical history will be summarized by Cohort, subtotal for Cohort 2-4 and overall by the number and percentage of subjects using the Safety Analysis Set. No formal statistical testing is planned.

Listing of General medical history data willbe provided.

6. EFFICACY ANALYSES

Efficacy endpoints analyses will be based on mITT and PP will serve as supplementary analysis.

6.1. Primary Efficacy Endpoint

6.1.1. Definition of the Primary Efficacy Endpoint:

The primary efficacy endpoints includes

1) Proportion of participants with HBV surface antigen (HBsAg) loss at end of treatment

2) Proportion of participants with HBsAg loss at 24 weeks post end of treatment

An estimation approach with no hypothesis testing is used to analyze the primary endpoints. A Bayesian hierarchy logistics regression will be used to calculate the point estimate and 95% credible interval of the primary endpoints

6.1.2. Estimand

Estimand for the primary endpoints:

- ➢ Proportion of participants with HBV surface antigen (HBsAg) loss at end of treatment
- ➢ Proportion of participants with HBsAg loss at 24 weeks post end of treatment
  - Population: Participants with chronic HBV infection as defined by the inclusion and exclusion criteria to reflect the targeted participant population.
  - Variable:
    - HBsAg loss at end of treatment.
    - HBsAg loss at 24 weeks post end of treatment
  - Treatment: Cohort 1, 2, 3 and 4.
  - Intercurrent events: Use of rescue medication, discontinuation of/interruption of/non-adherence to study intervention(s) The use of rescue medication, interruption of, and non-adherence to study intervention(s) will be ignored (treatment policy), and for the primary endpoint HBsAg loss at end of treatment , discontinuation of treatment will be handled using while-on-treatment strategy.
  - Population-level summary: Percentage of participants who achieve HBsAg loss at end of treatment and 24 weeks post end of treatment, respectively.

The last available HBsAg will be used for the analysis of primary endpoint HBsAg loss at end of treatment, if no any HBsAg value collected the missing value will be handled as 'Non-responder'

Estimand for the secondary endpoints:

- Population: Participants with chronic HBV infection as defined by the inclusion and exclusion criteria to reflect the targeted participant population.
- Variable:

  Continuous variables:

  – Absolute serum HBsAg and change from baseline over time through 24 weeks post end of treatment

  – Nadir serum HBsAg and maximum reduction of serum HBsAg from baseline throughout 48-week treatment

  Categorical variables:

  – HBsAg loss at Week 48

  – Functional cure (defined as sustained undetectable HBsAg and sustained suppression of HBV DNA [<lower limit of quantification (LLOQ)] ≥ 24 weeks after discontinuation of all treatment, including NRTI)

  – HBsAg loss and HBV DNA < LLOQ at 24 weeks after discontinuation of all treatment, including NRTI

  – Participants meeting NRTI discontinuation criteria at 24 weeks post end of treatment

  – Participants with Hepatitis B e antigen (HBeAg) loss and/or HBeAg seroconversion (baseline HBeAg-positive participants) at any timepoint

  – Proportion of participants with HBsAg seroconversion at any timepoint

  Time to event variable:

  – Time to achieve first serum HBsAg loss through Week 72
- Treatment: Cohort 1, 2, 3 and 4.
- Intercurrent events: Data collected will be utilized irrespective of discontinuation of, interruption of, and non-adherence to study intervention(s) or initiating rescue medication (treatment policy). However, the use of rescue medication will be treated as "treatment failure" (composite policy) for HBV DNA suppression related variables.
- Population-level summary: For continuous variables, descriptive statistics such as n, mean, standard deviation, median, interquartile, and ranges will be applied. For

categorical variables, percentage of each cohort will be presented. Hazards ratio between the cohort 1-3, and descriptive statistics for cohort 4 will be presented for time to event variables.

Missing value will be censored for time to event variable and will be handled as 'Non-responder' for categorical variables, for the analysis of the endpoint HBsAg loss at Week 48 if the HBsAg value at Week 48 analysis visit window is missing however the HBsAg are loss at both Week 44 and Week 52, the HBsAg value at Weeek 48 will be imputed as HBsAg loss.

6.1.3. Analysis methods for the primary endpoints

The primary endpoints are 1) Proportion of participants with HBsAg loss at end of treatment 2) Proportion of participants with HBsAg loss at 24 weeks post end of treatment.

A Bayesian hierarchical model (Yuen, 2022; Jones, 2011) as primary analysis will be used to estimate the posterior probability of response rate for each cohort of Cohorts 1-3 by incorporating the baseline HBsAg level stratification factor. The point estimate of the primary outcome and 95% equal-tailed credible interval will be drawn from the model.

Model for each group:

Number of responders $r_g \sim$ Binomial (n_g, p_g), g=1,2,3

$\theta_g = \text{logit}(p_g) = \log(p_g/(1-p_g)) = \gamma_0 + \gamma_1 I_{\{B+\}1} + \gamma_2 I_{\{B+\}2} + \psi_g$, g=1,2,3

where γ_0, γ_1, γ_2, ψ_g are all parameters, Thus,

$\theta_1 = \gamma_0 + \psi_1$

$\theta_2 = \gamma_0 + \gamma_1 + \psi_2$

$\theta_3 = \gamma_0 + \gamma_2 + \psi_3$

Priors:

$\gamma_k \sim \text{Normal}(0, 10^6)$, k=0,1,2

$\psi_g \sim \text{Normal}(0, \omega^2)$

$\omega \sim \text{Half-normal}(1)$

Where r_g indicates the number of responders among n_g participants, p_g as the response rate r_g/n_g, θ_g indicates the log odds of treatment response $\log(p_g/(1-p_g))$, index g=1,2,3 refer to the stratum numbers,

γ_k represent fixed effects of baseline HBsAg stratification factor, and ψ_g denotes a random effect in stratum g.

Stratum	$I_{\{B+\}1}$	$I_{\{B+\}2}$
HBsAg>100 IU/mL and HBsAg≤500 IU/mL	0	0
HBsAg>500 IU/mL and HBsAg≤1,500 IU/mL	1	0

HBsAg>1,500 IU/mL and HBsAg≤3,000 IU/mL	0	1

$I_{\{B+\}1}$ and $I_{\{B+\}2}$ are the dummy variables of $I_{\{B+\}}$.

For each cohort of Cohorts 1-3, the posterior distribution of response rate P(p_g|data), g=1,2,3 was derived for each stratum using the model specified above.
The posterior distribution of the group-level response rate will be derived using a mixture of the posterior distributions of response rate for each analysis stratum in that group. The weights are proportional to the sample size of each stratum.

Samples from the posterior distributions for all parameters will be obtained using a Markov Chain Monte Carlo (MCMC) sampling technique with 20,000 iterations following a burn-in of size 2000.

Diagnostic tests will be performed to assess convergence and autocorrelation. Alternative programming conventions (blocking, thinning, parameterization, number of chains, seeds, size of burn-in, initial values for each chain) may be explored if, using initial programming code, satisfactory mixing is not achieved within computer memory limitations.

For some models with low number of events (0–1 event), the stratified Bayesian hierarchical model does not converge. In such cases a reduced model that does not consider baseline stratification will be fitted. The model estimates the point estimate and 95% highest posterior density credible interval of proportion of participants achieving the primary endpoint assuming the number of responders followed a binomial distribution:

Number of responders r ~ Binomial (n, p)

Where p is the response rate, with a non-informative (Jeffrey's) prior: p ~Beta (0.5, 0.5).

The posterior distribution for p is: P (p|y, N) ~ Beta (y + 0.5, N – y + 0.5).

The same reduced model will be used to estimate the posterior distribution of Cohort 4 as no stratification factor is planned in this Cohort.

In addition, for Cohorts 1-3 combine some two adjacent baseline stratification levels to reduce HBsAg stratification factors from three levels to two levels (HBsAg >100 IU/mL and HBsAg ≤1,500 IU/mL versus HBsAg >1,500 IU/mL and HBsAg ≤3,000 IU/mL; HBsAg >100 IU/mL and HBsAg ≤500 IU/mL versus HBsAg >500 IU/mL and HBsAg ≤3,000 IU/mL) to perform the stratified Bayesian hierarchical analysis if model is converged using the similar approach as the primary analysis.

Samples from the posterior distribution of the response rates in each cohort of Cohorts 1-3 will be used to obtain posterior probabilities of interest (e.g., Probability [Difference of proportion of achieving primary endpoints between cohorts > threshold Data]); threshold such as 5%, 10%, 15%, 20% or any other percentiles of interest may be explored.

The primary efficacy analysis will be performed within mITT analysis set and a supplemental analysis based on PP analysis set with the similar analysis will be presented.

6.2. Secondary Efficacy Endpoints

6.2.1. Analysis Methods for Secondary Efficacy Endpoint

- Proportion of participants with HBsAg loss at Week 48
- Proportion of participants achieving functional cure (defined as sustained undetectable HBsAg and sustained suppression of HBV DNA <LLOQ ≥24 weeks after discontinuation of all treatment, including NRTI)
- Proportion of participants achieving HBsAg loss and HBV DNA <LLOQ 24 weeks after discontinuation of all treatment, including NRTI
- Proportion of participants meeting NRTI discontinuation criteria at 24 weeks post end of treatment

A Bayesian hirearcy model similar to primary analysis will be used to analyse the four endpoints, the analysis will be based on mITT and PP will serve as supplementary analysis.

Absolute serum HBsAg and change from baseline over time through 24 weeks post end of treatment

Numerical value of HBsAg and change from baseline will be summarized by cohort and by visit. The available data for each visit will be presented.

For Cohorts 1-3 change from baseline in HBsAg to all scheduled visits in log base 10 scale will be statistically analyzed using a Mixed Model for Repeated Measures model. The model will include log base 10 transformed baseline as a covariate, cohort, visit, cohort by visit as fixed effects, and within-participant errors will be modeled with an unstructured, heterogeneous autoregressive (1), or compound symmetry variance matrix in that order if a model does not converge. Estimates of least square mean (LSM) values and the LSM differences between each BRII-835 dose cohort and PEG-IFNα monotherapy cohort, and their respective 95% confidence interval and p-values will be calculated. The analyses will be done using mITT analysis set with hypothetical estimand. If the model is not converged only descriptive statistics will be presented.

In the first interim analysis, absolute serum HBsAg and change from baseline over time through Week 48 will be performed.

Nadir serum HBsAg and maximum reduction of serum HBsAg from baseline

Nadir serum HBsAg and maximum reduction of serum HBsAg from baseline will be summarized by cohort based on mITT analysis set.

Time to achieve first time of serum HBsAg loss through Week 72

Time to achieve first time of serum HBsAg loss will be analyzed by taking account of losses to follow-up using the stratified log-rank test with randomization strata as a stratification factor

based on mITT analysis set, and tabular summaries of the Kaplan Meier curves providing the median, quartiles, and range will be provided for each cohort, and the KM curves will be presented graphically. In addition, for Cohorts 1-3 a Cox proportional hazard regression model where the estimate of the hazard ratio for treatment (each BRII-835 dose cohort vs PEG-IFNα monotherapy cohort) will be performed, its 95% CI, and p value will be provided if the model is converged.

	Data of event/censoring	Censoring
Patients who lost to follow up or patients who withdraw from the trial	Time from randomization to last lab test date	Y
Patients who don't achieve the HBsAg loss	Time from randomization to last HBsAg sample collection date	Y
Patients who achieve the HBsAg loss	Time from randomization to date of achieving first time HBsAg loss	N

6.2.2. Exploratory Endpoints:

- Evaluation of potential biomarkers for host responses to treatment and/or infection including but not limited to innate and HBV-specific immunological responses
- Detection of other HBV viral markers including but not limited to serum HBcrAg and HBV RNA
- Proportion of virologic and clinical relapse after NRTI withdrawal. Virologic relapse is defined as HBV DNA > 2,000 IU/mL measured at 2 consecutive study visits; clinical relapse is defined as virologic relapse AND ALT > 2x ULN

No statistical analysis will be performed for the exploratory endpoints but summary of descriptive statistics will be conducted.

The proportion of subjects who have ever had Virologic relapse and Clinical relapse after NRTI withdrawal will be summarized by Cohort, subtotal for Cohort 1-3, overall and visit.

For HBV RNA result of "TND" or a numerical result smaller than the LLOQ (ie, 857 copies/mL) will be imputed as 856 copies/mL.

For HBcrAg result of "BQL" or a numerical result smaller than the LLOQ (ie, 3.0 log10 U/mL) will be imputed as 2.9 log10 U/mL.

.

7. SAFETY ANALYSES

7.1. Adverse Events and Deaths

7.1.1. Adverse Event Dictionary

Clinical and laboratory adverse events (AEs) will be coded using the current version of MedDRA. System organ class (SOC), high-level group term (HLGT), high-level term (HLT), preferred term (PT), and lower-level term (LLT) will be provided in the AE dataset.

7.1.2. Adverse Event Severity

Adverse events are graded by the investigator as Grade 1, 2, 3, 4, or 5 according to toxicity criteria specified in the protocol. The severity grade of events for which the investigator did not record severity will be categorized as "missing" for tabular summaries and data listings. The missing category will be listed last in summary presentation.

7.1.3. Relationship of Adverse Events to Study Drug

Related AEs are those for which the investigator selected "Related" on the AE CRF to the question of "Related to Study Treatment." Relatedness will always default to the investigator's choice, not that of the medical monitor. Events for which the investigator did not record relationship to study drug will be considered related to study drug for summary purposes. However, by-subject data listings will show the relationship as missing.

7.1.4. Serious Adverse Events

Serious adverse events (SAEs) will be identified and captured as SAEs if the AEs met the definitions of SAEs that were specified in the study protocol. SAEs captured and stored in the clinical database will be reconciled with the SAE database from the vender Drug Safety before data finalization.

7.1.5. Treatment-Emergent Adverse Events

7.1.5.1. Definition of Treatment-Emergent Adverse Events

Treatment-emergent adverse events (TEAEs) are defined as 1 or both of the following:

- Any AEs with an onset date on or after the study drug start date
- Any existing AEs worsen after treatment

7.1.5.2. Incomplete Dates

If the onset date of the AE is incomplete and the AE stop date is not prior to the first dosing date of study drug, then the month and year (or year alone if month is not recorded) of onset determine whether an AE is treatment emergent. The event is considered treatment emergent if the AE onset is the same as or after the month and year (or year) of the first dosing date of study drug.

An AE with completely missing onset and stop dates, or with the onset date missing and a stop date later than the first dosing date of study drug, will be considered to be treatment emergent.

In addition, an AE with the onset date missing and incomplete stop date with the same or later month and year (or year alone if month is not recorded) as the first dosing date of study drug will be considered treatment emergent.

7.1.6. Summaries of Adverse Events and Deaths

Treatment-emergent AEs will be summarized by Cohort, subtotal for Cohort 2-4 and overall based on Safety Analysis Set.

7.1.6.1. Summaries of AE Incidence by Severity

A brief, high-level summary of the number of percentage of subjects who experienced at least 1 TEAE in the categories described below will be provided by treatment. All deaths observed in the study will also be included in this summary.

The number and percentage of subjects who experienced at least 1 TEAE will be provided and summarized by SOC and PT by Cohort, subtotal for Cohort 2-4 and overall.

For the AE categories described below, summaries will be provided by SOC, PT and treatment:

- TEAEs by maximum severity
- TEAEs with Grade 3 or higher
- treatment-related TEAEs
- BRII-835 treatment-related AEs
- PEG-IFNα treatment-related AEs
- Both BRII-835 and PEG-IFNα treatment-related AEs
- treatment-related TEAEs of Grade 3 or higher
- BRII-835 treatment-related TEAEs of Grade 3 or higher
- BRII-835+PEG-IFNαtreatment-related TEAEs of Grade 3 or higher

- PEG-IFNαtreatment-related TEAEs of Grade 3 or higher
- treatment-related TEAEs by maximum severity
- TE SAEs
- treatment-related TESAEs
- BRII-835 treatment-related SAEs
- BRII-835+PEG-IFNα treatment-related SAEs
- Treatment-related TESAEs
- Injection site reaction
- TEAEs leading to premature discontinuation of BRII-835
- TEAEs leading to premature discontinuation of BRII-835+PEG-IFNα
- TEAEs leading to premature discontinuation of PEG-IFNα
- TEAEs leading to premature discontinuation of study
- TE SAEs leading to death (i.e., outcome of death)

Multiple events will be counted only once per subject in each summary. Adverse events will be summarized and listed first in descending order of total frequency of SOC and then by PT in descending order of total frequency within each SOC. For summaries by severity, the most severe severity will be used for those AEs that occurred more than once in a given subject during the study.

In addition to the above summary tables, all TEAEs, TE SAEs, TE treatment-related AEs, and TE treatment-related SAEs will be summarized by PT only in descending order of total frequency.

In addition, data listings will be provided for the following:

- All AEs, indicating whether the event is treatment emergent
- All SAEs
- All Deaths
- All SAEs leading to death (i.e., outcome of death)

- All AEs leading to premature discontinuation of study drug
- All AEs leading to premature discontinuation of study

7.2. Laboratory Evaluations

Laboratory data collected during the study will be analyzed and summarized using both quantitative and qualitative methods. Summaries of laboratory data will be provided by Cohort, subtotal for Cohort 2-4 and overall based on Safety Analysis Set and will include data throughout the trial.

For subjects who have permanently discontinued study drug, or all available data at the time of the database snapshot for subjects who were ongoing at the time of an interim analysis. The analysis will be based on values in standard units. When values are below the LOQ, they will be listed as such, and the closest imputed value will be used for the purpose of calculating summary statistics. Hemolized test results will not be included in the analysis, but they will be listed in by-subject laboratory listings.

A by-subject listing for laboratory test results will be provided by subject ID number, age, sex, race, body weight at Screening and visit in chronological order for hematology, serum chemistry, and urinalysis separately. Values falling outside of the relevant reference range and/or having a severity of Grade 1 or higher based on NCI-CTCAE Version 5.0 grading scale will be flagged in the data listings, as appropriate.

No formal statistical testing is planned.

7.2.1. Summaries of Numeric Laboratory Results

Descriptive statistics will be provided by treatment for each laboratory test specified in the study protocol as follows:

- Baseline values
- Values at each post-baseline visit
- Change from baseline at each post-baseline visit

A baseline laboratory value will be defined as the last measurement obtained on or prior to the date/time of first dose of study drug. Change from baseline to a post-baseline visit will be defined as the visit value minus the baseline value.

7.2.2. Graded Laboratory Values

The NCI-CTCAE Version 5.0 will be used to assign toxicity grades (1 to 5) to laboratory results for analysis. For laboratory tests with criteria for both increased and decreased levels, analyses for each direction (i.e., increased, decreased) will be presented separately.

7.2.2.1. Treatment-Emergent Laboratory Abnormalities

Treatment-emergent laboratory abnormalities are defined as values that increase at least 1 toxicity grade from baseline at any post-baseline visit. If the relevant baseline laboratory value is missing, any abnormality of at least Grade 1 observed within the time frame specified above will be considered treatment-emergent laboratory abnormalities.

7.2.2.2. Summaries of Laboratory Abnormalities

Laboratory data that are categorical will be summarized using the number and percentage of subjects in the study with the given response at baseline and each scheduled post-baseline visit.

The following summaries (number and percentage of subjects) for treatment-emergent laboratory abnormalities will be provided by lab test and treatment; subjects will be categorized according to the most severe post-baseline abnormality grade for a given lab test:

- Graded laboratory abnormalities

For all summaries of laboratory abnormalities, the denominator is the number of subjects with non-missing post-baseline values.

7.2.3. Liver-related Laboratory Evaluations

Liver-related abnormalities after initial study drug dosing will be examined and summarized using the number and percentage of subjects who were reported to have the following laboratory test values for postbaseline measurements:

- Aspartate aminotransferase (AST): (a) > 3 times of the upper limit of reference range (ULN); (b) > 5 x ULN; (c) > 10 x ULN; (d) > 20 x ULN
- Alanine aminotransferase (ALT): (a) > 3 x ULN; (b) > 5 x ULN; (c) > 10 x ULN; (d) > 20 x ULN
- AST or ALT: (a) > 3 x ULN; (b) > 5 x ULN; (c) > 10 x ULN; (d) > 20 x ULN
- Total bilirubin: > 2 x ULN
- Alkaline phosphatase (ALP) > 1.5 x ULN
- AST or ALT > 3 x ULN and total bilirubin: (a) > 1.5 x ULN; (b) > 2 x ULN

The summary will include data from all post-baseline visits. For individual laboratory tests, subjects will be counted once based on the most severe post-baseline values. For both the composite endpoint of AST or ALT and total bilirubin, subjects will be counted once when the criteria are met at the same post-baseline visit date. The denominator is the number of subjects in the Safety Analysis Set who have non-missing post-baseline values of all relevant tests at the same post-baseline visit date. A listing of subjects who met at least 1 of the above criteria will be provided.

7.3. Vital Signs

Descriptive statistics will be provided by Cohort for vital signs as follows:

- Baseline value
- Values at each postbaseline visit
- Change from baseline at each postbaseline visit

A baseline value will be defined as the last available value collected on or prior to the date/time of first dose of study drug. Change from baseline to a postbaseline visit will be defined as the postbaseline value minus the baseline value. Vital signs measured at unscheduled visits will be included for the baseline value selection.

No formal statistical testing is planned.

A by-subject listing of vital signs will be provided by subject ID number, sex, age, race, screening body weight and visit in chronological order.

7.4. Prior and Concomitant Medications

Medications collected at screening and during the study will be coded using the current version of the World Health Organization (WHO) Drug dictionary.

7.4.1. Prior Medications

Prior HBV medications are defined as any medications taken before a subject took the first study drug.

Prior medications will be summarized by Anatomical Therapeutic Chemical (ATC) drug class Level 3 and preferred name using the number and percentage of subjects for each treatment and overall. A subject reporting the same medication more than once will be counted only once within each ATC drug class when calculating the number and percentage of subjects who received that medication. Medications may appear under multiple ATC drug classes. The summary will be ordered alphabetically by ATC medical class and then by preferred term in

order of descending overall frequency within each ATC medical class. For drugs with the same frequency, sorting will be done alphabetically.

For the purposes of analysis, any medication with a start date prior to the first dosing date of study drug will be included in the prior medication summary regardless of when the stop date is. If a partial start date is entered the medication will be considered prior unless the month and year (if day is missing) or year (if day and month are missing) of the start date are after the first dosing date. Medications with a completely missing start date will be included in the prior medication summary, unless otherwise specified.

Summaries will be based on the Safety Analysis Set. No formal statistical testing is planned.

7.4.2. Concomitant Medications

Concomitant medications are defined as medications taken while a subject took study drug. Use of concomitant medications will be summarized by Anatomical Therapeutic Chemical (ATC) drug class Level 3 and preferred name using the number and percentage of subjects for each treatment. A subject reporting the same medication more than once within each ATC drug class will be counted only once when calculating the number and percentage of subjects who received that medication. Medications may appear under multiple ATC drug classes. The summary will be ordered alphabetically by ATC medical class and then by preferred term in descending overall frequency within each ATC medical class. For drugs with the same frequency, sorting will be done alphabetically.

For the purposes of analysis, any medications with a start date prior to or on the first dosing date of study drug and continued to be taken after the first dosing date, or started after the first dosing date but prior to or on the last dosing date of study drug will be considered concomitant medications. Medications started and stopped on the same day as the first dosing date or the last dosing date of study drug will also be considered concomitant. Medications with a stop date prior to the date of first dosing date of study drug or a start date after the last dosing date of study drug will be excluded from the concomitant medication summary. If a partial stop date is entered, any medication with the month and year (if day is missing) or year (if day and month are missing) prior to the date of first study drug administration will be excluded from the concomitant medication summary. If a partial start date is entered, any medication with the month and year (if day is missing) or year (if day and month are missing) after the study drug stop date will be excluded from the concomitant medication summary. Medications with completely missing start and stop dates will be included in the concomitant medication summary, unless otherwise specified. Summaries will be based on the Safety Analysis Set. No formal statistical testing is planned.

All prior and concomitant medications (other than per-protocol study drugs) will be provided in a by-subject listing sorted by subject ID number, age, sex, race, screening body weight and administration date in chronological order.

7.5. Electrocardiogram Results

A by-subject listing for ECG assessment results will be provided by subject ID number, sex, age, race, screening body weight.

7.6. Other Safety Measures

No additional safety measures are specified in the protocol.

8. ADA

Desriptive statistics based on ADA Analysis Set will be performed for ADA.

9. CHANGES FROM PROTOCOL-SPECIFIED EFFICACY ANALYSES

9.1. Changes From Protocol-Specified Efficacy Analyses

NA

9.2. Changes From Protocol-Specified Safety Analyses

There are no deviations from the protocol-specified safety analyses.

10. PHARMACOKINETIC EVALUATION/ANALYSIS

Individual subject concentration data for BRII-835 and its metabolite A-312327 will be listed and summarized using descriptive statistics based on PK Analysis Set. Summary statistics (numbers of subjects, arithmetic mean, SD, coefficient of variation [CV], median, minimum, maximum, geometric mean and Geometric CV) will be presented by time point and Cohort.

No PK parameters will be reported in this study since only limited (sparse) PK samples were collected. The concentration data from this study will be pooled with data from other studies for population PK analysis, which will be reported separately.

11. SOFTWARE

SAS® Software Version 9.4. SAS Institute Inc., Cary, NC, USA.

12. SAP REVISION

Revision Date (DD MMM YYYY)	Section	Summary of Revision	Reason for Revision

835-002 SAP

Final Audit Report 2024-11-08

Created:	2024-11-05
By:	Weihong Liu (weihong.liu@briibio.com)
Status:	Signed
Transaction ID:	CBJCHBCAABAAkMb7Sst9uVIQ2kQ_JiHc3q-mLN75GZ70

"835-002 SAP" History

Document created by Weihong Liu (weihong.liu@briibio.com)
2024-11-05 - 8:44:17 AM GMT

Document emailed to Weihong Liu (weihong.liu@briibio.com) for signature
2024-11-05 - 8:45:19 AM GMT

Document emailed to Xiaofei Chen (xiaofei.chen@briibio.com) for signature
2024-11-05 - 8:45:20 AM GMT

Document emailed to David Margolis (david.margolis@briibio.com) for signature
2024-11-05 - 8:45:20 AM GMT

Weihong Liu (weihong.liu@briibio.com) authenticated with Adobe Acrobat Sign.
Challenge: The user opened the agreement.
2024-11-05 - 8:45:48 AM GMT

Weihong Liu (weihong.liu@briibio.com) authenticated with Adobe Acrobat Sign.
Challenge: The user clicked on the signature field: 'Signature 1'.
2024-11-05 - 8:46:14 AM GMT

Document e-signed by Weihong Liu (weihong.liu@briibio.com)
Signing reason: I am the author/submitter of this document.
Signature Date: 2024-11-05 - 8:46:31 AM GMT - Time Source: server

Email viewed by David Margolis (david.margolis@briibio.com)
2024-11-05 - 1:41:38 PM GMT

David Margolis (david.margolis@briibio.com) authenticated with Adobe Acrobat Sign.
Challenge: The user opened the agreement.
2024-11-05 - 1:42:06 PM GMT

David Margolis (david.margolis@briibio.com) authenticated with Adobe Acrobat Sign.

Challenge: The user clicked on the signature field: 'Signature 3'.

2024-11-05 - 1:42:37 PM GMT

Document e-signed by David Margolis (david.margolis@briibio.com)

Signing reason: I reviewed / approved this document.

Signature Date: 2024-11-05 - 1:42:46 PM GMT - Time Source: server

Email viewed by Xiaofei Chen (xiaofei.chen@briibio.com)

2024-11-08 - 7:34:15 AM GMT

Xiaofei Chen (xiaofei.chen@briibio.com) authenticated with Adobe Acrobat Sign.

Challenge: The user opened the agreement.

2024-11-08 - 7:36:38 AM GMT

Xiaofei Chen (xiaofei.chen@briibio.com) authenticated with Adobe Acrobat Sign.

Challenge: The user clicked on the signature field: 'Signature 2'.

2024-11-08 - 7:38:37 AM GMT

Document e-signed by Xiaofei Chen (xiaofei.chen@briibio.com)

Signing reason: I reviewed / approved this document.

Signature Date: 2024-11-08 - 7:39:28 AM GMT - Time Source: server

Agreement completed.

2024-11-08 - 7:39:28 AM GMT

STATISTICAL ANALYSIS PLAN

**Study Title:**	A Phase 2 Multicenter, Randomized, Open-label Study to Investigate the Efficacy and Safety of BRII-835 (VIR-2218) and Pegylated Interferon Alpha (PEG-IFNα) Combination Therapy for the Treatment of Chronic Hepatitis B Virus (HBV) Infection
**Name of Test Drug:**	BRII-835 (VIR-2218); Pegylated Interferon Alpha (PEG-IFNα)
**Study Number:**	BRII-835-002
**Protocol Version (Date):**	2.0 (31 August 2023)
**Analysis Type:**	Phase 2
**Analysis Plan Version:**	2.0
**Analysis Plan Date:**	20Feb2025
**Analysis Plan Author(s):**	[redacted]

CONFIDENTIAL AND PROPRIETARY INFORMATION

SIGNATURE PAGE

Reason for Signature	Name	Signature	Date (DDMMMYYYY)
Author	[redacted]		
Therapeutic Area Head (or delegate)			
Chief Medical Officer (or delegate)			

TABLE OF CONTENTS

TABLE OF CONTENTS 3
LIST OF ABBREVIATIONS 5
1. INTRODUCTION 7
1.1. Study Objectives 7
1.2. Study Design 7
1.3. Sample Size and Power 10
2. TYPE OF PLANNED ANALYSIS 13
2.1. Interim Analyses 13
2.1.1. Dose Escalation Analysis 13
2.2. Final Analysis 13
3. GENERAL CONSIDERATIONS FOR DATA ANALYSES 15
3.1. Key Definitions 16
3.2. Analysis Sets 17
3.2.1. Screened Analysis Set 18
3.2.2. Full Analysis Set (FAS) 18
3.2.3. Safety Analysis Set (SAS) 18
3.2.4. Modified Intent-To-Treat Analysis Set (mITT) 18
3.2.5. Per Protocol Set (PP) 18
3.2.6. Pharmacokinetic Analysis Set (PAS) 19
3.2.7. ADA Analysis Set 19
3.2.8. PBMC Analysis Set 19
3.3. Subject Grouping 19
3.4. Strata and Covariates 19
3.5. Subgroup analysis 19
3.6. Multiple Comparisons 20
3.7. Missing Data and Outliers 20
3.7.1. Missing Data 20
3.7.2. Outliers 20
3.8. Data Handling Conventions and Transformations 20
3.9. Analysis Visit Windows 22
3.9.1. Selection of Data in the Event of Multiple Records in an Analysis Visit Window 26
4. SUBJECT DISPOSITION 28
4.1. Subject Enrollment and Disposition 28
4.1.1. Duration of Exposure to Study Drug 29
4.1.2. Adherence to Study Drug 29
4.2. Protocol Deviations 30
5. BASELINE CHARACTERISTICS 31
5.1. Demographics and Baseline Characteristics 31
5.2. Other Baseline Characteristics 31
5.3. Medical History 31
6. EFFICACY ANALYSES 32
6.1. Primary Efficacy Endpoint 32

6.1.1. Definition of the Primary Efficacy Endpoint: 32
6.1.2. Estimand 32
6.1.3. Analysis methods for the primary endpoints 33
6.2. Secondary Efficacy Endpoints 35
6.2.1. Estimand for the secondary endpoints: 35
6.2.2. Analysis Methods for Secondary Efficacy Endpoint 36
6.2.3. Exploratory Endpoints: 37
7. SAFETY ANALYSES 39
7.1. Adverse Events and Deaths 39
7.1.1. Adverse Event Dictionary 39
7.1.2. Adverse Event Severity 39
7.1.3. Relationship of Adverse Events to Study Drug 39
7.1.4. Serious Adverse Events 39
7.1.5. Treatment-Emergent Adverse Events 39
7.1.5.1. Definition of Treatment-Emergent Adverse Events 39
7.1.5.2. Incomplete Dates 39
7.1.6. Summaries of Adverse Events and Deaths 40
7.1.6.1. Summaries of AE Incidence by Severity 40
7.2. Laboratory Evaluations 42
7.2.1. Summaries of Numeric Laboratory Results 42
7.2.2. Graded Laboratory Values 42
7.2.2.1. Treatment-Emergent Laboratory Abnormalities 43
7.2.2.2. Summaries of Laboratory Abnormalities 43
7.2.3. Liver-related Laboratory Evaluations 43
7.3. Vital Signs 44
7.4. Prior and Concomitant Medications 44
7.4.1. Prior Medications 44
7.4.2. Concomitant Medications 45
7.5. Electrocardiogram Results 45
7.6. Other Safety Measures 46
8. ADA 47
9. PHARMACOKINETIC EVALUATION/ANALYSIS 48
10. CHANGES FROM PROTOCOL-SPECIFIED EFFICACY ANALYSES 49
10.1. Changes From Protocol-Specified Efficacy Analyses 49
10.2. Changes From Protocol-Specified Safety Analyses 49
10.3. Changes From Protocol Interim Analyses 49
10.4. Changes From Protocol Sample Size 49
11. SOFTWARE 50
12. SAP REVISION 51

LIST OF ABBREVIATIONS

ADA	anti-drug antibody
AE	adverse event
ALT	alanine aminotransferase
AST	aspartate aminotransferase
BLQ	below the limit of quantitation
BMI	body mass index
CFR	Code of Federal Regulations
CI	confidence interval
CK	creatine kinase
CRF	case report form
CSR	clinical study report
CTCAE	Common Toxicity Criteria for Adverse Events
ECG	Electrocardiogram
FAS	Full Analysis Set
Hb	Hemoglobin
HBV	hepatitis B virus
HLT	high-level term
ICH	International Conference on Harmonization (of Technical Requirements for Registration of Pharmaceuticals for Human Use)
ITT	intent to treat
LLT	lower-level term
LOQ	limit of quantitation
MedDRA	Medical Dictionary for Regulatory Activities
mITT	modified intent to treat
NrtI	nucleos(t)ide reverse transcriptase inhibitors
PBMC	peripheral blood mononuclear cell
PK	pharmacokinetic
PP	per protocol
PT	preferred term
Q1, Q3	first quartile, third quartile
SAP	statistical analysis plan
SC	subcutaneously
SD	standard deviation
SI (units)	international system of units
SOC	system organ class
SRC	safety review committee
TEAE	treatment-emergent adverse event

TFLs	tables, figures, and listings
ULN	upper limit of normal
WHO	World Health Organization

1. INTRODUCTION

This statistical analysis plan (SAP) describes the statistical analysis methods and data presentations to be used in tables, figures, and listings (TFLs) in the clinical study report (CSR) for Study BRII-835-002. This SAP is based on the study protocol version 2.0 dated 31 Aug 2023 and the electronic case report form (eCRF). The SAP will be finalized before database finalization. Any changes made after the finalization of the SAP will be documented in the CSR.

This statistical analysis plan (SAP) describes the statistical analysis methods and data presentations to be used in tables, figures, and listings (TFLs) of three interim analyses for Study BRII-835-002. The first two interim analyses will be conducted when approximately 45 enrolled participants of Cohorts 1-3 complete the 48-week treatment and last available participant of all enrolled participants (Cohorts 1-4) completes the 48-week treatment, respectively. If the pace of enrollment is robust, and the primary endpoint proportion of participants with HBsAg loss at Week 48 is expected to occur less or equal to two months between the two interim analyses, the two analyses may be collapsed into one analysis.

1.1. Study Objectives

The primary objective of this study is as follows:

- To evaluate the efficacy and safety of BRII-835 and PEG-IFNα combination therapy in participants with chronic HBV infection

The secondary objectives of this study are as follows:

- To assess the antiviral activity of BRII-835 and PEG-IFNα combination therapy
- To assess BRII-835 pharmacokinetic (PK) exposure and evaluate the anti-drug antibodies (ADA) to BRII-835

The exploratory objectives of this study are as follows

- To evaluate potential biomarkers for host immune responses to therapy
- To evaluate other viral markers of HBV infection

1.2. Study Design

This is a Phase 2 open-label, randomized study of BRII-835 and PEG-IFNα combination therapy in adult participants with chronic HBV infection without cirrhosis receiving NRTI therapy as background therapy. This study is designed to evaluate the efficacy and safety of BRII-835 and PEG-IFNα combination therapy. Two different dose levels of BRII-835 in combination with PEG-IFNα will be evaluated.

Participants with HBeAg negative or HBeAg positive chronic HBV infection are eligible to participate in Cohorts 1-3 of this study. Eligible participants will be stratified by their serum HBsAg level at screening and randomized 1:1:1 to each of the three cohorts. Up to 75 participants are planned for Cohorts 1-3.

In addition, this study will evaluate the efficacy of BRII-835 and PEG-IFNα combination in participants previously treated with BRII-179, a therapeutic recombinant HBV vaccine. Eligible participants who completed BRII-179-835-001 study will roll over to Cohort 4. Up to 45 participants will be enrolled.

All participants will remain outpatient during study participation and will be followed for the safety, tolerability, and antiviral activity after completion of study intervention. The assessments and procedures performed at each visit are further described in the SoA (Section 1.3) of the protocol.

Participants will be assessed by the investigator for their eligibility to discontinue NRTI therapy at Week 72. Participants who meet the NRTI discontinuation criteria may enter the NRTI discontinuation monitoring period for an additional 24 weeks. Participants who do not meet the NRTI discontinuation criteria will continue NRTI therapy and will complete the study.

The total duration of study participation for each participant will include screening period (up to 4 weeks), dosing period (48 weeks), follow-up period (24 weeks), and NRTI discontinuation monitoring period (24 weeks when applicable). The study design schema can be found in Section 1.2 in the protocol.

Planned Cohorts and Dosing

Cohorts 1-3 (BRII-179 naïve):

Eligible participants of Cohorts 1-3 will be stratified by their serum HBsAg level at screening (>100 IU/mL and ≤ 500 IU/mL, > 500 and ≤ 1,500 IU/mL, or > 1,500 and ≤ 3,000 IU/mL) and randomized to receive the study intervention per the following dose regimens.

- Cohort 1: Each participant will receive 48 doses of PEG-IFNα 180 mcg administered SC every week starting on Day 1
- Cohort 2: Each participant will receive 48 doses of PEG-IFNα 180 mcg administered SC every week and 13 doses of BRII-835 200 mg administered SC every 4 weeks, both starting on Day 1
- Cohort 3: Each participant will receive 48 doses of PEG-IFNα 180 mcg administered SC every week and 13 doses of BRII-835 100 mg administered SC every 4 weeks, both starting on Day 1

Cohort 4 (BRII-179 treated):

Eligible participants who completed BRII-179-835-001 study will be assigned to Cohort 4 and receive the following study intervention.

Cohort 4: Each participant will receive 48 doses of PEG-IFNα 180 mcg administered SC every week and 13 doses of BRII-835 100 mg administered SC every 4 weeks, both starting on Day 1

Safety Review Committee: A Safety Review Committee (SRC) for this study will monitor participant safety and consider study-specific data as well as relevant background information about the study drugs and target population under study to inform on study conduct. The primary purpose of the SRC is to protect the safety of participants in this study.

Ad hoc SRC meetings will take place as needed, i.e., for a significant safety event such as criteria for study intervention discontinuation being reached. Decisions to suspend dosing or discontinue individual participants from study intervention will be made according to the predetermined criteria (Section 7 in the protocol). Participants may resume study intervention only following SRC approval. Additionally, the SRC may recommend discontinuation of or changes to the study to the Sponsor. The SRC charter outlining the member composition and operating procedures will be developed before the study initiation.

NRTI Discontinuation

NRTI Discontinuation Criteria

Upon participants completing Week 72 visits, the investigator will evaluate whether participants are eligible for discontinuation of NRTI therapy based on the following NRTI discontinuation criteria:

- HBsAg < LLOQ AND
- HBV DNA < LLOQ AND
- Undetectable HBeAg AND
- ALT ≤ 2x ULN

Participants who meet all of the above criteria for at least two consecutive visits at Week 72 are eligible to discontinue from NRTI therapy. Investigator should confirm participants' eligibility as early as possible within 2 weeks post Week 72 visit. Participants who discontinue NRTI will enter the NRTI Discontinuation Monitoring Period of the study within 1 week of the investigator confirming eligibility. Participants will not take their NRTI medications on the first day of the NRTI Discontinuation Monitoring Period and will be followed for an additional 24 weeks per the SoA (Section 1.3) in the protocol.

NRTI Re-treatment

It is recommended that the Investigator should consider re-treatment with NRTI therapy during the NRTI Discontinuation Monitoring Period if the participant meets any of the following criteria:

- ALT > 10x ULN with HBV DNA ＞LLOQ at the same visit
- ALT 5-10x ULN with HBV DNA ＞LLOQ persisting for ≥ 4 consecutive weeks
- ALT > ULN and HBV DNA > 2,000 IU/mL persisting for ≥ 4 consecutive weeks

- ALT > ULN with total bilirubin > 2x ULN and/or prolongation of prothrombin time (PT) > 2 seconds, and HBV DNA > LLOQ at the same visit
- HBV DNA > 100,000 IU/mL at any visit
- Confirmed HBeAg seroreversion (i.e., HBeAg positive after being HBeAg negative at NRTI discontinuation)
- Any other clinically significant event(s) warranting initiation of NRTI therapy in the opinion of the Investigator

Whether participants should be re-treated with NRTI is at the discretion of the Investigator following good medical judgement. Participants who are re-treated with NRTI therapy should continue to be followed per the SoA (Section 1.3.2) in the protocol for the duration of the 24-week NRTI Discontinuation Monitoring Period.

Participants with HBV DNA increase ≥ 2 $\log_{10}$ IU/mL within a 2-week period should:

- Return to the site for an unscheduled visit to measure HBV DNA levels and liver function tests as soon as possible after the investigator being aware of the HBV DNA increase
- Continue returning to site weekly until HBV DNA levels stabilize (do not increase ≥ 1 $\log_{10}$ IU/mL over a 4-week period or start decreasing for at least 2 consecutive visits) after which they will continue follow-up per the SOA for the NRTI Discontinuation Monitoring Period
- If any unscheduled visits needed as outlined above coincide with an existing visit per the SoA in the protocol, the scheduled assessments should be conducted

1.3. Sample Size and Power

Up to 75 participants will be randomly assigned in 1:1:1 ratio to each of Cohorts 1-3. A Bayesian framework will be used to calculate the sample size. It is assumed that the number of responders follows a Binomial distribution, with a weakly informative prior (Beta (0.5, 0.5)) for the true response rate. The precision for a range of response rates with 95% credible intervals are shown in Table 1:

Up to 45 participants will be enrolled in Cohort 4. No formal sample size is calculated due to the exploratory nature of Cohort 4.

Table 1 95% Credible Interval of Response Rate by Sample Size for Cohorts 1-3

Sample size per cohort	Number of responders	Response rate (%)	95% Credible interval* (%)
15	4	27	8-50
	5	33	13-57

	6	40	18-63
	7	47	25-71
	8	53	29-76
	9	60	36-82
25	6	24	10-41
	7	28	13-46
	8	32	16-51
	9	36	19-55
	10	40	22-58
	11	44	26-63
	12	48	30-67
	13	52	32-70

* 95% highest posterior density interval.

For the 15 participants in each cohort the lower bounds of 95% credible interval exclude the response rate of 10% if observed response rate is greater than 27% and exclude the response rate of 20% if observed response rate is greater than 40% and exclude the response rate of 30% if observed response rate is greater than 53%.

For the 25 participants in each cohort the lower bounds of 95% credible interval exclude the response rate of 10% if observed response rate is greater than 24% and exclude the response rate of 20% if observed response rate is greater than 36% and exclude the response rate of 30% if observed response rate is greater than 48%.The operational characteristics are based on posterior confidence that the true rate exceeds a threshold of interest, as shown in Table . For instance, when 4 out of 15 participants or 6 out of 25 participants are responders, the probability (true response rate>15%) is larger than 85%.

Table 2 Operational Characteristics by Sample Size

**Sample size per cohort**	**Number of responders**	**Probability (True response rate)>15%**	**Probability (True response rate)>20%**	**Probability (True response rate)>25%**	**Probability (True response rate)>30%**	**Probability (True response rate)>35%**
15	1	18.4%	8.5%	3.7%	1.4%	0.5%
	2	45.7%	27.2%	14.4%	7%	3.2%
	3	72.8%	52.6%	34.1%	20.1%	10.9%

	4	89.7%	75.6%	57.9%	40.6%	25.7%
	5	96.8%	90.4%	78.3%	62.7%	45.7%
	6	99.2%	96.6%	90.7%	80.4%	66.6%
	7	99.9%	99.2%	96.9%	92%	83.4%
25	2	16.2%	5.4%	1.6%	0.4%	0.1%
	3	35.7%	16.1%	6%	1.9%	0.5%
	4	58.2%	32.1%	14.9%	5.8%	1.9%
	5	77.4%	52.1%	29.4%	13.8%	5.5%
	6	89.1%	70.5%	46.9%	26.3%	12.1%
	7	95.9%	84.8%	65.5%	42.7%	23.6%
	8	98.5%	92.5%	79.4%	59.8%	38.3%
	9	99.6%	97.4%	89.6%	75%	55.2%
	10	99.9%	99%%	95.2%	86.1%	70.3%
	11	100%	99.7%	98.2%	93.3%	82.6%

2. TYPE OF PLANNED ANALYSIS

2.1. Interim Analyses

Prior to the final analysis, three interim analyses will be conducted.

The first two interim analyses will be conducted when approximately 45 enrolled participants of Cohorts 1-3 complete the 48-week treatment and last available participant of all enrolled participants (Cohorts 1-4) complete the 48-week treatment, respectively. If the pace of enrollment is robust, and the proportion of participants with HBsAg loss at Week 48 is expected to occur less or equal to two months between the two interim analyses, the two analyses may be collapsed into one analysis. Endpoints with measurement time after the 48-week treatment except for safety relevant data will not be analyzed in the first two interim analyses.

The sponsor had decided to stop participant enrollment in December 2023, with a total of 55 participants enrolled in Cohorts 1-3. Consequently, the study team had agreed that the first interim analysis conducted when 55 participants completed the 48-week treatment. During this interim analysis, only descriptive statistics had been performed, and no per-protocol (PP) analysis was conducted.

The second interim analysis will still at the timepoint when the last participant of Cohorts 1-4 complete the 48 week treatment.

The third interim analysis was originally planned to be conducted when last available participant of Cohorts 1-3 completes follow-up 24 weeks post end of treatment. The sponsor decided to conduct the third interim analysis when last available participant of Cohorts 1-4 completes follow-up 24 weeks post end of treatment (see Section 10.3).

Sponsor further decided analysis based on per-protocol population will not be conducted at second interim analysis. Starting from second interim analysis in addition to descriptive statistics all available statistical analysis will be performed. The second interim analysis will include all efficacy endpoints except for PK and ADA of BRII-835 throughout Week 48, and the endpoints with measurement time after the 48-week treatment except for safety relevant data will not be analyzed in the second interim analyses. The third interim analysis will be also include the efficacy endpoints of the corresponding time points.

PK and ADA analysis will be performed at the third interim analysis as they should be available by then. Exploratory endpoints including viral and immune biomarkers will not be conducted until the whole study completes.

2.1.1. Dose Escalation Analysis

Not Applicable.

2.2. Final Analysis

The final analysis will be performed when last available participant of Cohorts 1-4 completes the last scheduled visit according to the SoA.

3. GENERAL CONSIDERATIONS FOR DATA ANALYSES

All data collected on the electronic case report form (eCRF) will be presented in the data listings and will be listed by subject identification number (in ascending order), sex, age at screening, screening body weight and visit, where applicable. All summaries will be presented by Cohort.

When reporting descriptive statistics, the following rules will be applied, except for PK concentration data:

- Continuous variables: Descriptive statistics will include the number of non-missing values (n), arithmetic mean, standard deviation (SD), median, first quartile (Q1), third quartile (Q3), minimum and maximum values.

  The minimum and maximum values will be displayed to the same decimal precision as the source data; the Q1, Q3, arithmetic mean and median values will be displayed to one more decimal than the source data; SD value will be displayed to two more decimal than the source data for the specific variable.

  The appropriate precision for derived variables will be determined based on the precision of the data on which the derivations are based, and statistics will be presented in accordance with the abovementioned rules.

  n will be an integer. If no subjects have data at a given time point, then only n=0 will be presented.

  All data will be presented to a maximum of 3 decimal places.

- Categorical variables: Descriptive statistics will include counts and percentages per category. The denominator in all percentage calculations will be the number of subjects in the relevant analysis set with non-missing data, unless specifically stated otherwise. Percentages will be displayed to one decimal place.

  Percentage change from baseline values will be calculated and displayed to 1 decimal place in the listings. In the summaries, the mean and median percentage change from baseline values will be presented to 2 decimal places, the minimum and maximum values will be presented to 1 decimal place and the SD value will be presented to 3 decimal places.

  For categories where all subjects fulfill certain criteria, the percentage value will be displayed as 100. For categories where zero subjects fulfill certain criteria, there will be no percentage displayed. All other percentage displays will use 1 decimal place. Data listings will contain all reported and derived data.

Unless otherwise stated, the following methods will be applied:

- Repeat/unscheduled assessments: Values collected at unscheduled study visits/time points will be allocated to scheduled visit/time points according to the algorithms of analysis visit window together with values collected at scheduled visits/time points and be presented in summary tables. All collected data will be included in the data listings.

- Assessment windows: All assessments will be included in the data listings and no visit windows will be applied to exclude assessments that were performed outside of the protocol specified procedure windows.
- Result display convention: Results will be center aligned in all summary tables and listings. Subject identifiers, visits, and parameter labels may be left-aligned if required.
- Date and time display conventions: The following display conventions will be applied in all outputs where dates and/or times are displayed:
  - Date only: YYYY-MM-DD
  - Date and time: YYYY-MM-DD HH:MM

If only partial information is available, unknown components of the date or time will be presented as 'NK' (not known), i.e., '2016-NK-NK'. Time will be reported in military time.

3.1. Key Definitions

The following definitions will be used:

- Baseline: The baseline value is defined as the last available, valid (quantifiable continuous or categorical value), and non-missing observation for each subject prior to first study drug administration unless otherwise specified. Repeat and unscheduled assessments will be included in the derivation of the baseline values
- Change from Baseline: The change from baseline value is defined as the difference between the result collected/derived at a post-baseline visit/time point and the baseline value.

  The change from baseline value at each post-baseline visit/time point will be calculated for all continuous parameters using the following formula:

  Change from Baseline Value = Result at Visit/Time Point – Baseline Value

  The change from baseline value will only be calculated if the specific post-baseline visit/time point result and the baseline value for the parameter are both available; otherwise, will be treated as missing.
- Study day: The study day of an event is defined as the relative day of the event starting with the date of the first study drug administration (reference date) as Day 1 (there will be no Day 0).

  The study day of events occurring before the first study drug administration will be calculated as:

  Study Day = (Date of Event - Date of First Study Drug Administration)

  For events occurring on or after Day 1, study day will be calculated as:

  Study Day = (Date of Event - Date of First Study Drug Administration) + 1

Study days will only be calculated for events with complete dates and will be undefined for events that are 'Ongoing' at the end of the study.

- Relative days compared to an alternative reference point will be calculated similarly, but the alternative starting reference start date will be used instead of the date of the first study drug dosing.
- HBsAg loss: HBsAg changing from positive at baseline to <LLOQ at any post-baseline visit.
- Anti-HBs seroconversion: Anti-HBs changing from negative (<10 IU/L) at baseline to positive (>=10 IU/L) at any post-baseline visit.
- HBsAg seroconversion: HBsAg loss plus anti-HBs positivity (>=10 IU/L) at any post-baseline visit
- Sustained HBsAg loss: quantitative HBsAg < LLOQ at all visits (scheduled and unscheduled) for ≥ 24 weeks (168 days). Sustained days = (last date of HBsAg <LLOQ - first date of HBsAg <LLOQ) +1, during which the values at all visits < LLOQ.
- Anti-HBe seroconversion: Anti-HBe changing from negative or missing at baseline to positive at any post-baseline visit.
- HBeAg loss ($HBeAg^+$ subjects only): HBeAg changing from positive at baseline to negative at any post-baseline visit.
- HBeAg seroconversion (HBeAg+ subjects only): HBeAg loss plus anti-HBe positivity at any post-baseline visit.
- Functional cure: sustained undetectable HBsAg (HBsAg < LLOQ) and sustained suppression of HBV DNA < LLOQ ≥ 24 weeks including all visits (scheduled and unscheduled) after discontinuation of all treatment, including NRTI.
- Modified Functional cure: HBsAg <LLOQ and HBV DNA<LLOQ at each analysis window in the NRTI discontinuation monitoring period. Any observation of HBsAg>= LLOQ or HBV DNA >= LLOQ must be confirmed at a consecutive visit (including unscheduled visits) for the participants to be classed as having lost their sustainability, Participants who have a value of HBsAg >= LLOQ or HBV DNA >=LLOQ at their last visit, which cannot be confirmed due to no further follow up, will be treated as 'treatment failure'.

The LLOQ for HBsAg is 0.05 IU/mL, and 10 IU/mL for HBV DNA.

3.2. Analysis Sets

Analysis sets define the subjects to be included in an analysis. Analysis sets and their definitions are provided in this section. The analysis set will be identified and included as a subtitle of each table, figure, and listing.

For each analysis set, the number and percentage of subjects eligible for inclusion will be provided.

A listing of exclusion from analysis sets will be provided by subject.

3.2.1. Screened Analysis Set

The Screened Analysis Set will consist of all subjects who sign the informed consent form. The listing of eligibility criteria will be based on this population.

3.2.2. Full Analysis Set (FAS)

All participants assigned to study intervention will be included in Full Analysis Set.

3.2.3. Safety Analysis Set (SAS)

All participants assigned to study intervention and who take at least 1 dose of study drug. Participants will be analyzed according to the study intervention they received. Participants will be analyzed by Cohort, subtotal for Cohort 2-4 and overall.

All safety analyses will be based on Safety Analysis Set.

3.2.4. Modified Intent-To-Treat Analysis Set (mITT)

All participants assigned to study intervention, who take at least 1 dose of study drug, with at least 1 postbaseline visit through Week 48. Participants will be analyzed according to the study intervention to which they were randomized for Cohorts 1- 3, and for Cohort 4 participants will be analyzed according to the study intervention specified in the protocol.

3.2.5. Per Protocol Set (PP)

The Per Protocol Set will include the subset of mITT who fulfil:

- HBsAg ≥ 0.05 IU/mL at baseline
- Total number of dose compliance ≥ 80% and ≤ 120% for BRII-835 and PEG-IFNα
- Have HBsAg data in the analysis visit window for the primary efficacy endpoints analysis
- Had not identified with important (major) protocol deviation that significantly impacts the interpretation of the primary efficacy endpoints

A Per Protocol review meeting will be held to go through the important protocol deviations to identify the PP population prior to the database lock for the final analysis.

PP Set will be used as analysis population of antiviral activity and immunological markers.

3.2.6. Pharmacokinetic Analysis Set (PAS)

All participants who received BRII-835, had a at least 1 evaluable PK concentration, and had no protocol deviations thought to impact on the PK data analysis.

3.2.7. ADA Analysis Set

All participants who received BRII-835 and had at least 1 non-missing ADA result from the ADA assay after first dose of the study drug. Participants will be analyzed by Cohort, subtotal for Cohort 2-4 and overall.

3.2.8. PBMC Analysis Set

Participants in Cohort 1, 2, 3, and 4 evaluable for immune profiling will be included in the PBMC Analysis Set. Here "evaluable" refers to whose whole blood is collected and qualified for immune cell and cytokine/chemokine profiling. The analysis for the PBMC data will be based on this set.

3.3. Subject Grouping

For analyses based on the FAS, mITT, SAS and PP Set, subjects will be grouped according to the Cohort.

3.4. Strata and Covariates

Up to 75 participants will be randomly assigned in 1:1:1 ratio among Cohorts 1-3 using an IRT system. Stratification will be based on the following variables:

Screening HBsAg level (>100 IU/mL and ≤500 IU/mL, >500 IU/mL and ≤1,500 IU/mL, >1,500 IU/mL and ≤3,000 IU/mL).

If there are discrepancies in stratification factor values between the IWRS and the clinical database, the values recorded in the clinical database will be used for analyses.

For Cohort 4 no randomization is performed.

3.5. Subgroup analysis

- In addition, for Cohorts 1-3 combine some two adjacent screening stratification levels (i.e. randomization strata) to reduce HBsAg stratification factors from three levels to two levels (HBsAg >100 IU/mL and HBsAg ≤1,500 IU/mL versus HBsAg >1,500 IU/mL and HBsAg ≤3,000 IU/mL; HBsAg >100 IU/mL and HBsAg ≤500 IU/mL versus HBsAg >500 IU/mL and HBsAg ≤3,000 IU/mL) to perform the stratified Bayesian hierarchical analysis if model is converged using the similar approach as the primary analysis.

- Further stratify Cohort 4 into two subgroups analysis listed below:

- Subgroup analysis 1 will be conducted between responder and non-responder by Week 24 in study BRII-179-835-001. Week 24 responder is defined as peak anti-HBs titer ≥ 2 IU/L by Week 24 for participants with baseline anti-HBs < 2 IU/L OR peak anti-HBs titer ≥ 10 IU/L by Week 24 for participants with baseline anti-HBs ≥ 2 IU/L and < 10IU/L in BRII-179-835-001 study.

- Subgroup analysis 2 will be conducted between responder and non-responder by Week 44 in study BRII-179-835-001. Week 44 responder is defined as peak anti-HBs titer ≥ = 10 IU/L by Week 44 in BRII-179-835-001 study.

The descriptive statistics for antiviral activity and immunological markers will be presented. The unstratified bayesian analysis will be performed by subgroup of cohort 4, respectively with similar method taken as cohort 4.

- HBsAg change over time will be summarized by cohort 1- 4 based on baseline HBsAg level categories (HBsAg >100 IU/mL and HBsAg ≤500 IU/mL, HBsAg >500 IU/mL and HBsAg ≤1500 IU/mL, HBsAg >1500 IU/mL and HBsAg ≤3000 IU/mL, HBsAg≤1500 IU/mL). The subgroup of HBsAg <=1000 IU/mL, that had conducted in the first interim analysis, will not be required, but may be conducted depending on the exploratory needs.

3.6. Multiple Comparisons

NA

3.7. Missing Data and Outliers

3.7.1. Missing Data

In general, missing data will not be imputed unless methods for handling missing data are specified. Exceptions are presented in this document.

For missing last dosing date of study drug, imputation rules are described in Section 4.1.1, the handling of missing or incomplete dates for AE onset is described in Section 7.1.5.2, and for prior HBV and concomitant medications in Section 7.4.

3.7.2. Outliers

Outliers will be identified during the data management and data analysis process, but no sensitivity analyses will be conducted. All data will be included in the data analysis.

3.8. Data Handling Conventions and Transformations

The following conventions will be used for the imputation of date of birth when it is partially missing or not collected:

- If only month and year of birth is collected, then "15" will be imputed as the day of birth
- If only year of birth is collected, then "01 July" will be imputed as the day and month of birth
- If year of birth is missing, then date of birth will not be imputed.

If age at Day 1 is not available for a subject, then age derived based on date of birth and the Day 1 visit date will be used instead. If an enrolled subject was not dosed with any study drug, the randomization date will be used instead of the Day 1 visit date. For screen failures, the date the first informed consent was signed will be used for the age derivation. Age required for longitudinal and temporal calculations and analyses (e.g age at date of AE) will be based on age derived from date of birth and the date of the measurement or event, unless otherwise specified.

Non-PK data that are continuous in nature but are less than the lower limit of quantitation (LOQ) or above the upper LOQ will be imputed as follows:

- A value that is 1 unit less than the LOQ will be used to calculate descriptive statistics if the datum is reported in the form of "< x" (where x is considered the LOQ). For example, if the values are reported as < 50 and < 5.0, values of 49 and 4.9, respectively, will be used to calculate summary statistics. An exception to this rule is any value reported as < 1 or < 0.1, etc. For values reported as < 1 or < 0.1, a value of 0.9 or 0.09, respectively, will be used to calculate summary statistics.
- A value that is 1 unit above the LOQ will be used to calculate descriptive statistics if the datum is reported in the form of "> x" (where x is considered the LOQ). Values with decimal points will follow the same logic as above.
- The LOQ will be used to calculate descriptive statistics if the datum is reported in the form of "≤ x" or "≥ x" (where x is considered the LOQ).

Plasma concentrations that are below the limit of quantitation (BLQ) will be presented as "BLQ" in the concentration data listing. BLQ concentrations will be treated as 0 at predose time points, and as missing at postdose time points for determination of summary and order statistics. Geometric mean and geometric CV% will not be calculated if the concentrations is BLQ or missing at predose.

The following conventions will be used for the presentation of summary and order statistics:

- If at least 1 subject has a concentration value of BLQ for the time point, the minimum value will be displayed as "BLQ."

- If more than 25% of the subjects have concentration values of BLQ for a given time point, the minimum value will be displayed as "BLQ."
- If more than 50% of the subjects have concentration values of BLQ for a given time point, the minimum and median values will be displayed as "BLQ."
- If more than 75% of the subjects have concentration values of BLQ for a given time point, the minimum and median values will be displayed as "BLQ."
- If all subjects have concentration values of BLQ for a given time point, all order statistics (minimum, median, and maximum) and summary statistics will be displayed as "BLQ."

3.9. Analysis Visit Windows

Subject visits might not occur on protocol-specified days. Therefore, for the purpose of analysis, observations will be assigned to analysis windows. The analysis visit windows for the parameters at different periods are listed in Table 3.1 – Table 3.6

- Prior to NrtI Discontinuation Monitoring Period

Table 3.1 Analysis Visit Windows for HBsAg Prior to NRTI Discontinuation Monitoring Period

Analysis Visit	Nominal Study Day	Lower Limit	Upper Limit
Baseline	1	None	1
Week 4	29	2	43
Week 8	57	44	71
Week 12	85	72	99
Week 16	113	100	127
Week 20	141	128	155
Week 24	169	156	183
Week 28	197	184	211
Week 32	225	212	239

Week 36	253	240	267
Week 40	281	268	295
Week 44	309	296	323
Week 48	337	324	365
Week 56	393	366	421
Week 64	449	422	477
Week 72	505	478	533

One of the primary endpoints is the proportion of participants with HBsAg loss at weeks post actual end of treatment, a participant discontinues study treatment before the planned EOT (Week 48) and does not withdraw from study participation, he/she will return to the investigational site every 8 weeks upon EOT for the specified assessments and the corresponding analysis windows for HBsAg is as follows

Analysis Visit	Reference day	Lower Limit	Upper Limit
EoT	1	None	1
FU Week 8	57	2	85
FU Week 16	113	86	141
FU Week 24	169	142	197

Table 3.2 Analysis Visit Windows for Anti-HBs Prior to NRTI Discontinuation Monitoring Period

Analysis Visit	Nominal Study Day	Lower Limit	Upper Limit
Baseline	1	None	1
Week 16	113	2	141
Week 24	169	142	197

Week 32	225	198	253
Week 40	281	254	309
Week 48	337	310	365
Week 56	393	366	421
Week 64	449	422	477
Week 72	505	478	533

Table 3.3 Analysis Visit Windows for HBV DNA Prior to NRTI Discontinuation Monitoring Period

Analysis Visit	Nominal Study Day	Lower Limit	Upper Limit
Baseline	1	None	1
Week 12	85	2	127
Week 24	169	128	211
Week 36	253	212	295
Week 48	337	296	365
Week 56	393	366	449
Week 72	505	450	533

Table 3.4 Analysis Visit Windows for HBeAg , Anti-HBe Prior to NrtI Discontinuation Monitoring Period

Analysis Visit	Nominal Study Day	Lower Limit	Upper Limit
Baseline	1	None	1
Week 16	113	2	169

Week 32	225	170	281
Week 48	337	282	421
Week 72	505	422	533

Tabel 3.5 Analysis Visit Windows for Hematology, Chemistry and Coagulation Assessments Prior to NrtI Discontinuation Monitoring Period

Analysis Visit	Nominal Study Day	Lower Limit	Upper Limit
Baseline	1	None	1
Week 2	15	2	22
Week 4	29	23	43
Week 8	57	44	71
Week 12	85	72	99
Week 16	113	100	127
Week 20	141	128	155
Week 24	169	156	183
Week 28	197	184	211
Week 32	225	212	239
Week 36	253	240	267
Week 40	281	268	295
Week 44	309	296	323
Week 48	337	324	365
Week 56	393	366	421
Week 64	449	422	477

Week 72	505	478	533

- NRTI Discontinuation Monitoring Period

Table 3.6 Analysis Visit Windows for HBsAg, HBV DNA, HBeAg, Hematology, Chemistry and Coagulation during NRTII Discontinuation Monitoring Period

Analysis Visit	NDMP Day	Lower Limit	Upper Limit
NDMP Baseline	1	None	1
NDMP Week 4	29	2	43
NDMP Week 8	57	44	71
NDMP Week 12	85	72	106
NDMP Week 18	127	107	148
NDMP Week 24	169	149	190

*NDMP: NRTI Discontinuation Monitoring Period; NDMP baseline is not applicable for HBeAg according to SOA in the protocol

3.9.1. Selection of Data in the Event of Multiple Records in an Analysis Visit Window

Depending on the statistical analysis method, single values may be required for each analysis window. For example, change from baseline by visit usually requires a single value, whereas a time-to-event analysis would not require 1 value per analysis window.

If multiple valid, nonmissing measurements exist in an analysis window, records will be chosen based on the following rules if a single value is needed:

- For baseline, the last nonmissing value on or prior to the first dosing date of study drug will be selected, unless specified differently. If there are multiple records with the same time or no time recorded on the same day, the baseline value will be the average of the measurements for continuous data, or the measurement with the lowest severity (e.g., normal will be selected over abnormal for safety electrocardiogram findings) for categorical data, and the most conservative value will be selected for efficacy data (e.g., negative will be selected over positive for HBeAg and HBsAg, whereas positive will be selected over negative for Anti-HBe and Anti-HBs).

- For post-baseline values:

- — The most conservative value for safety lab parameters (e.g., abnormal will be selected over normal) within the analysis window will be selected
- — If there are more than 1 value within an analysis window, the value collected closet to the nominal day will be selected.
- — If there are 2 records that are equidistant from the nominal day, the later record will be selected.

- If there is more than 1 record on the selected day, the latest record will be selected; if these measurements were recorded at the same time or no time recorded, the average will be taken for continuous data.

4. SUBJECT DISPOSITION

4.1. Subject Enrollment and Disposition

A summary of subject enrollment will be provided by Cohort for each country/region, investigator and site name and presented in Full Analysis Set. The summary will present the number and percentage of subjects enrolled. For each column, the denominator for the percentage calculation will be the total number of subjects analyzed for that column.

A similar enrollment table will be provided by randomization stratum. The denominator for the percentage of subjects in the stratum will be the total number of enrolled subjects. If there are discrepancies in the value used for stratification assignment between the IWRS and the clinical database, the value collected in the clinical database will be used. A listing of subjects with discrepancies in the value used for stratification assignment between the IWRS and the clinical database at the time of data finalization will be provided.

The randomization schedule used for the study will be provided as an appendix in the CSR.

- A summary of subject disposition will be provided by Cohort. This summary will present the number of subjects screened, subjects who meet all eligibility criteria but not randomized, and the number of subjects in each of the categories listed below:
- All screened subject
- Screen Failure
  - Subjects not met eligibility criteria
  - Subjects met eligibility but not enrolled
- Subject enrolled
- All treated subject
- Completed study drug administration
- Did not complete study drug administration with reasons for premature discontinuation of study drug
- Completed study
- Did not complete the study with reasons for premature discontinuation of study

For the status of study drug and study completion and reasons for premature discontinuation, the number and percentage of subjects in each category will be provided. The denominator for the percentage calculation will be the total number of subjects in the Full Analysis Set corresponding to that column. In addition, a flowchart will be provided to depict the disposition.

The following by-subject listings will be provided by subject identification (ID) number (in ascending order), age, sex and screening body weight to support the above summary tables:

- Reasons for premature study drug or study discontinuation
- Reasons for screen failure (will be provided by screened subject ID number in ascending order)

4.1.1. Duration of Exposure to Study Drug

Duration of treatment is calculated as (Date of Last Dose - Date of First Dose + 1) for the completers and calculated as (Discontinued Date-Date of First Dose +1) for subjects with prematurely discontinuation, regardless of any temporary interruptions in study drug administration, and will be expressed in weeks using up to 1 decimal place (e.g., 4.5 weeks). If the last study drug dosing date is missing, the latest date among the clinical visit date, laboratory sample collection date, and vital signs assessment date that occurred during the dosing-treatment period will be used.

Duration of Treatment and Total number of injections for BRII-835 and PEG-IFNα will be summarized, respectively using descriptive statistics (n, mean, STD, Median, Q1, Q3, Min, Max) for Safety Analysis Set.

In addition, Total number of injections will be summarized by counts and percentages for Safety Analysis Set.

No formal statistical testing is planned.

A by-subject listing of study drug administration and drug accountability will be provided separately by subject ID number, sex, age, race, body weight at Screening, and visit in chronological order.

4.1.2. Adherence to Study Drug

The level of prescribed adherence to the study drug regimen will be determined by the total doses of study drug taken relative to the total doses of study drug specified by the protocol.

The level of prescribed adherence of BRII-835 and PEG-IFNα will be expressed as a percentage using the following formula:

$$\text{Prescribed Adherence (\%)} = \left(\frac{\text{Total Doses of Study Drug}}{\text{Total Doses of Study Drug Specified by Protocol}}\right) \text{ x } 100$$

Descriptive statistics for the level of prescribed adherence with the number and percentage of subjects belonging to adherence categories (e.g., *{< 80%, ≥ 80 to ≤ 90%, > 90%}*) will be provided by treatment group for the Safety Analysis Set.

No formal statistical testing is planned.

Number of Dose administration will be summarized by Cohort, Cohort 2-4 and overall based on Safety Analysis Set.

4.2. Protocol Deviations

Protocol deviations occurring after subjects entered the study are documented during routine monitoring. The number and percentage of subjects with important protocol deviations, and the total number of important protocol deviations by deviation reason (e.g., nonadherence to study drug, violation of select inclusion/exclusion criteria) will be summarized by Cohort for the Full Analysis Set. In addition, a by-subject listing subjects with all participant level protocol deviation will be provided.

5. BASELINE CHARACTERISTICS

5.1. Demographics and Baseline Characteristics

Subject demographic variables (i.e., age, sex, race, and ethnicity) and baseline characteristics (body weight [in kg], height [in cm], body mass index [BMI; in kg/m^2]) will be summarized by Cohort, subtotal for Cohort 1-3 and overall using descriptive statistics for continuous variables and using number and percentage of subjects for categorical variables. The summary of demographic data will be provided for the Full Analysis Set.

A by-subject demographic listing, including the informed consent date, will be provided by subject ID number (in ascending order), sex, age, race, and body weight at Screening.

5.2. Other Baseline Characteristics

Other baseline characteristics include HBsAg, Log10 transformed HBsAg, Anti-HBs, HBeAg at Screening and HBV genotype. These baseline characteristics will be summarized by Cohort, subtotal for Cohort 1-3 and overall using descriptive statistics for continuous variables and using number and percentage of subjects for categorical variables. The summary of these baseline characteristics will be provided for the Full Analysis Set. No formal statistical testing is planned.

A by-subject listing of other baseline characteristics will be provided by subject ID number (in ascending order), sex, age, race, and body weight at Screening.

5.3. Medical History

Medical history will be collected at screening.

Medical history will be summarized by Cohort, subtotal for Cohort 2-4 and overall by the number and percentage of subjects using the Safety Analysis Set. No formal statistical testing is planned.

Listing of general medical history data will be provided.

6. EFFICACY ANALYSES

Efficacy endpoints analyses will be based on mITT and PP will serve as supplementary analysis.

6.1. Primary Efficacy Endpoint

6.1.1. Definition of the Primary Efficacy Endpoint:

The primary efficacy endpoints includes

1) Proportion of participants with HBV surface antigen (HBsAg) loss at end of treatment
2) Proportion of participants with HBsAg loss at 24 weeks post end of treatment

An estimation approach with no hypothesis testing is used to analyze the primary endpoints. A Bayesian hierarchy logistics regression will be used to calculate the point estimate and 95% credible interval of the primary endpoints for cohort 1-3, for cohort 4 a unstratified Bayesian estimation will be conducted.

6.1.2. Estimand

Estimand for the primary endpoints:

- ➢ Proportion of participants with HBV surface antigen (HBsAg) loss at end of treatment
- ➢ Proportion of participants with HBsAg loss at 24 weeks post end of treatment

  - Population: Participants with chronic HBV infection as defined by the inclusion and exclusion criteria to reflect the targeted participant population.
  - Variable:
    - HBsAg loss at end of treatment.
    - HBsAg loss at 24 weeks post end of treatment
  - Treatment: Cohort 1, 2, 3 and 4.
  - Intercurrent events: Use of rescue medication, discontinuation of/interruption of/non-adherence to study intervention(s). The use of rescue medication, interruption of, and non-adherence to study intervention(s), will be ignored (treatment policy); The discontinuation of study intervention(s) will be handled using while-on-treatment strategy for variable 1 and will be ignored (treatment policy) for variable 2.

- Population-level summary: Percentage of participants who achieve HBsAg loss at end of treatment and 24 weeks post end of treatment, respectively.

The last available HBsAg will be used for the analysis of primary endpoint HBsAg loss at end of treatment, if no any HBsAg value collected, the missing value will be handled as 'Non-responder'

6.1.3. Analysis methods for the primary endpoints

The primary endpoints are 1) Proportion of participants with HBsAg loss at end of treatment 2) Proportion of participants with HBsAg loss at 24 weeks post end of treatment.

A Bayesian hierarchical model (Yuen, 2022; Jones, 2011) as primary analysis will be used to estimate the posterior probability of response rate for each cohort of Cohorts 1-3 by incorporating the screening HBsAg level stratification factor (i.e. randomization strata). The point estimate of the primary outcome and 95% equal-tailed credible interval will be drawn from the model.

Model for each group:
Number of responders $r_g \sim$ Binomial (n_g, p_g), g=1,2,3
θ_g =logit(p_g)=log(p_g/(1-p_g))= $\gamma_0 + \gamma_1 I_{\{B+\}1} + \gamma_2 I_{\{B+\}2+} \psi_g$, g=1,2,3
where γ_0, γ_1, γ_2, ψ_g are all parameters, Thus,
$\theta_1 = \gamma_0 + \psi_1$
$\theta_2 = \gamma_0 + \gamma_1 + \psi_2$
$\theta_3 = \gamma_0 + \gamma_2 + \psi_3$
Priors:
$\gamma_k \sim$ Normal(0, 10^6), k=0,1,2
$\psi_g \sim$ Normal(0, ω^2)
$\omega \sim$ Half-normal(1)
Where r_g indicates the number of responders among n_g participants, p_g as the response rate r_g/n_g, θ_g indicates the log odds of treatment response log(p_g/(1-p_g)), index g=1,2,3 refer to the stratum numbers,
γ_k represent fixed effects of screening HBsAg stratification factor, and ψ_g denotes a random effect in stratum g.

Stratum	$I_{\{B+\}1}$	$I_{\{B+\}2}$
HBsAg>100 IU/mL and HBsAg≤500 IU/mL	0	0
HBsAg>500 IU/mL and HBsAg≤1,500 IU/mL	1	0
HBsAg>1,500 IU/mL and HBsAg≤3,000 IU/mL	0	1

$I_{\{B+\}1}$ and $I_{\{B+\}2}$ are the dummy variables of $I_{\{B+\}}$.

For each cohort of Cohorts 1-3, the posterior distribution of response rate $P(p_g|data)$, g=1,2,3 was derived for each stratum using the model specified above.
The posterior distribution of the group-level response rate will be derived using a mixture of the posterior distributions of response rate for each analysis stratum in that group. The weights are proportional to the sample size of each stratum.

Samples from the posterior distributions for all parameters will be obtained using a Markov Chain Monte Carlo (MCMC) sampling technique with 20,000 iterations following a burn-in of size 2000.

Diagnostic tests will be performed to assess convergence and autocorrelation. Alternative programming conventions (blocking, thinning, parameterization, number of chains, seeds, size of burn-in, initial values for each chain) may be explored if, using initial programming code, satisfactory mixing is not achieved within computer memory limitations.

For some models with low number of events (0–1 event), the stratified Bayesian hierarchical model does not converge. In such cases a reduced model that does not consider screening stratification will be fitted. The model estimates the point estimate and 95% highest posterior density credible interval of proportion of participants achieving the primary endpoint assuming the number of responders followed a binomial distribution:

Number of responders r ~ Binomial (n, p)

Where p is the response rate, with a non-informative (Jeffrey's) prior: p ~Beta (0.5, 0.5).

The posterior distribution for p is: P (p|y, N) ~ Beta (y + 0.5, N – y + 0.5).

The same reduced model will be used to estimate the posterior distribution of Cohort 4 as no stratification factor is planned in this Cohort.

In addition, for Cohorts 1-3 combine some two adjacent screening stratification levels (i.e. randomization strata) to reduce HBsAg stratification factors from three levels to two levels (HBsAg >100 IU/mL and HBsAg ≤1,500 IU/mL versus HBsAg >1,500 IU/mL and HBsAg ≤3,000 IU/mL; H BsAg >100 IU/mL and HBsAg ≤500 IU/mL versus HBsAg >500 IU/mL and HBsAg ≤3,000 IU/mL) to perform the stratified Bayesian hierarchical analysis if model is converged using the similar approach as the primary analysis.

Samples from the posterior distribution of the response rates in each cohort of Cohorts 1-3 will be used to obtain posterior probabilities of interest (e.g., Probability [Difference of proportion of achieving primary endpoints between cohorts > threshold Data]); threshold such as 5%, 10%, 15%, 20% or any other percentiles of interest may be explored.

The primary efficacy analysis will be performed within mITT analysis set and a supplemental analysis based on PP analysis set with the similar analysis will be presented.

6.2. Secondary Efficacy Endpoints

6.2.1. Estimand for the secondary endpoints:

- Population: Participants with chronic HBV infection as defined by the inclusion and exclusion criteria to reflect the targeted participant population.
- Variable:

  Continuous variables:

  – Absolute serum HBsAg and change from baseline over time through 24 weeks post end of treatment

  – Nadir serum HBsAg and maximum reduction of serum HBsAg from baseline throughout 48-week treatment

  Categorical variables:

  – HBsAg loss at Week 48

  – Functional cure (defined as sustained undetectable HBsAg and sustained suppression of HBV DNA [<lower limit of quantification (LLOQ)] ≥ 24 weeks after discontinuation of all treatment, including NRTI)

  – HBsAg loss and HBV DNA < LLOQ at 24 weeks after discontinuation of all treatment, including NRTI

  – Participants meeting NRTI discontinuation criteria at 24 weeks post end of treatment

  – Participants with Hepatitis B e antigen (HBeAg) loss and/or HBeAg seroconversion (baseline HBeAg-positive participants) at any timepoint

  – Proportion of participants with HBsAg seroconversion at any timepoint

  Time to event variable:

  – Time to achieve first serum HBsAg loss through Week 72

- Treatment: Cohort 1, 2, 3 and 4.
- Intercurrent events: Data collected will be utilized irrespective of discontinuation of, interruption of, and non-adherence to study intervention(s) or initiating rescue medication (treatment policy). However, the use of rescue medication will be treated as “treatment failure” (composite policy) for HBV DNA suppression related variables.
- Population-level summary: For continuous variables, descriptive statistics such as n, mean, standard deviation, median, interquartile, and ranges will be applied. For categorical variables, percentage of each cohort will be presented. Hazards ratio

between the cohort 1-3, and descriptive statistics for cohort 4 will be presented for time to event variables.

Missing value will be censored for time to event variable and will be handled as 'Non-responder' for categorical variables, for the analysis of the endpoint HBsAg loss at Week 48 if the HBsAg value at Week 48 analysis visit window is missing however the HBsAg are loss at both Week 44 and Week 52, the HBsAg value at Weeek 48 will be imputed as HBsAg loss.

6.2.2. Analysis Methods for Secondary Efficacy Endpoint

- **Proportion of participants with HBsAg loss at Week 48**
- **Proportion of participants achieving functional cure (defined as sustained undetectable HBsAg and sustained suppression of HBV DNA <LLOQ ⩾24 weeks after discontinuation of all treatment, including NRTI)**
- **Proportion of participants achieving HBsAg loss and HBV DNA <LLOQ 24 weeks after discontinuation of all treatment, including NRTI**
- **Proportion of participants meeting NRTI discontinuation criteria at 24 weeks post end of treatment**

A Bayesian hierarchical model similar to primary analysis will be used to analyse the four endpoints, the analysis will be based on mITT and PP will serve as supplementary analysis.

- **Absolute serum HBsAg and change from baseline over time through 24 weeks post end of treatment**

Numerical value of HBsAg and change from baseline will be summarized by cohort and by visit. The available data for each visit will be presented.

For Cohorts 1-3 change from baseline in HBsAg to all scheduled visits in log base 10 scale will be statistically analyzed using a Mixed Model for Repeated Measures model. The model will include log base 10 transformed baseline as a covariate, cohort, visit, cohort by visit as fixed effects, and within-participant errors will be modeled with an unstructured, heterogeneous autoregressive (1), or compound symmetry variance matrix in that order if a model does not converge. Estimates of least square mean (LSM) values and the LSM differences between each BRII-835 dose cohort and PEG-IFNα monotherapy cohort, and their respective 95% confidence interval and p-values will be calculated. The analyses will be done using mITT analysis set with hypothetical estimand. If the model is not converged only descriptive statistics will be presented.

In the first interim analysis, absolute serum HBsAg and change from baseline over time through Week 48 will be performed.

- **Nadir serum HBsAg and maximum reduction of serum HBsAg from baseline**

Nadir serum HBsAg and maximum reduction of serum HBsAg from baseline will be summarized by cohort based on mITT analysis set.

- **Time to achieve first time of serum HBsAg loss through Week 72**

Time to achieve first time of serum HBsAg loss (Days) defined as the duration from the first study drug administration to the time to achieve first time of serum HBsAg loss before or on Week 72.
Duration (Days)= event/censoring date – the first study drug administration date + 1.

Time to achieve first time of serum HBsAg loss will be analyzed by taking account of losses to follow-up using the stratified log-rank test with randomization strata as a stratification factor based on mITT analysis set, and tabular summaries of the Kaplan Meier curves providing the median and quartiles -will be provided for each cohort, and the KM curves will be presented graphically. In addition, for Cohorts 1-3 a Cox proportional hazard regression model where the estimate of the hazard ratio for treatment (, pairwise comparison between three cohorts) will be performed with randomization strata as fixed effect, its 95% CI, and p value will be provided if the model is converged.

Cohort 4 will be further analyzed by subgroups specified in section 3.5, Time to achieve first time of serum HBsAg loss will be analyzed by taking account of losses to follow-up using the log-rank test based on mITT analysis set, and tabular summaries of the Kaplan Meier curves providing the median, quartiles, and range will be provided for each subgroup, and the KM curves will be presented graphically. In addition, for cohort 4 a cox proportional hazard regression model where the estimate of the hazard ratio for subgroup (responder vs non-responder by Week 24 and Week 44 respectively) will be performed its 95% CI, and p value will be provided if the model is converged.

	Data of event/censoring	Censoring
Participants who lost to follow up or participants who withdrew from the trial	Time from randomization tolast lab collection date	Y
Participants who did’t achieve the HBsAg loss	Time from randomization to last HBsAg sample collection date	Y
Participants who achieved the HBsAg loss	Time from randomization to date of achieving first time HBsAg loss	N

6.2.3. Exploratory Endpoints:

- Evaluation of potential biomarkers for host responses to treatment and/or infection including but not limited to innate and HBV-specific immunological responses

- Detection of other HBV viral markers including but not limited to serum HBcrAg and HBV RNA
- Proportion of virologic and clinical relapse after NRTI withdrawal. Virologic relapse is defined as HBV DNA > 2,000 IU/mL measured at 2 consecutive study visits; clinical relapse is defined as virologic relapse AND ALT > 2x ULN

No statistical analysis will be performed for the exploratory endpoints but summary of descriptive statistics will be conducted.

The proportion of subjects who have ever had Virologic relapse and Clinical relapse after NRTI withdrawal will be summarized by Cohort, subtotal for Cohort 1-3, overall and visit.

For HBV RNA result of "TND" or a numerical result smaller than the LLOQ (ie, 857 copies/mL) will be imputed as 856 copies/mL.

For HBcrAg result of "BQL" or a numerical result smaller than the LLOQ (ie, 3.0 log10 U/mL) will be imputed as 2.9 log10 U/mL.

.

7. SAFETY ANALYSES

7.1. Adverse Events and Deaths

7.1.1. Adverse Event Dictionary

Clinical and laboratory adverse events (AEs) will be coded using the current version of MedDRA. System organ class (SOC), high-level group term (HLGT), high-level term (HLT), preferred term (PT), and lower-level term (LLT) will be provided in the AE dataset.

7.1.2. Adverse Event Severity

Adverse events are graded by the investigator as Grade 1, 2, 3, 4, or 5 according to toxicity criteria specified in the protocol. The severity grade of events for which the investigator did not record severity will be categorized as "missing" for tabular summaries and data listings. The missing category will be listed last in summary presentation.

7.1.3. Relationship of Adverse Events to Study Drug

Related AEs are those for which the investigator selected "Related" on the AE CRF to the question of "Related to Study Treatment." Relatedness will always default to the investigator's choice, not that of the medical monitor. Events for which the investigator did not record relationship to study drug will be considered related to study drug for summary purposes. However, by-subject data listings will show the relationship as missing.

7.1.4. Serious Adverse Events

Serious adverse events (SAEs) will be identified and captured as SAEs if the AEs met the definitions of SAEs that were specified in the study protocol. SAEs captured and stored in the clinical database will be reconciled with the SAE database from the vender Drug Safety before data finalization.

7.1.5. Treatment-Emergent Adverse Events

7.1.5.1. Definition of Treatment-Emergent Adverse Events

Treatment-emergent adverse events (TEAEs) are defined as 1 or both of the following:

- Any AEs with an onset date on or after the study drug start date
- Any existing AEs worsen after treatment

7.1.5.2. Incomplete Dates

If the onset date of the AE is incomplete and the AE stop date is not prior to the first dosing date of study drug, then the month and year (or year alone if month is not recorded) of onset determine whether an AE is treatment emergent. The event is considered treatment emergent if the AE onset is the same as or after the month and year (or year) of the first dosing date of study drug.

An AE with completely missing onset and stop dates, or with the onset date missing and a stop date later than the first dosing date of study drug, will be considered to be treatment emergent.

In addition, an AE with the onset date missing and incomplete stop date with the same or later month and year (or year alone if month is not recorded) as the first dosing date of study drug will be considered treatment emergent.

7.1.6. Summaries of Adverse Events and Deaths

Treatment-emergent AEs will be summarized by Cohort, subtotal for Cohort 2-4 and overall based on Safety Analysis Set.

7.1.6.1. Summaries of AE Incidence by Severity

A brief, high-level summary of the number of percentage of subjects who experienced at least 1 TEAE in the categories described below will be provided by treatment. All deaths observed in the study will also be included in this summary.

The number and percentage of subjects who experienced at least 1 TEAE will be provided and summarized by SOC and PT by Cohort, subtotal for Cohort 2-4 and overall.

For the AE categories described below, summaries will be provided by SOC, PT and treatment:

- TEAEs by maximum severity
- TEAEs with Grade 3 or higher
- treatment-related TEAEs
- BRII-835 treatment-related AEs
- PEG-IFNα treatment-related AEs
- Both BRII-835 and PEG-IFNα treatment-related AEs
- treatment-related TEAEs of Grade 3 or higher
- BRII-835 treatment-related TEAEs of Grade 3 or higher
- BRII-835+PEG-IFNαtreatment-related TEAEs of Grade 3 or higher

- PEG-IFNαtreatment-related TEAEs of Grade 3 or higher
- treatment-related TEAEs by maximum severity
- TE SAEs
- treatment-related TESAEs
- BRII-835 treatment-related SAEs
- BRII-835+PEG-IFNα treatment-related SAEs
- Injection site reaction
- TEAEs leading to premature discontinuation of BRII-835
- TEAEs leading to premature discontinuation of BRII-835+PEG-IFNα
- TEAEs leading to premature discontinuation of PEG-IFNα
- TEAEs leading to premature discontinuation of study
- TE SAEs leading to death (i.e., outcome of death)

Multiple events will be counted only once per subject in each summary. Adverse events will be summarized and listed first in descending order of total frequency of SOC and then by PT in descending order of total frequency within each SOC. For summaries by severity, the most severe severity will be used for those AEs that occurred more than once in a given subject during the study.

In addition to the above summary tables, all TEAEs, TE SAEs, TE treatment-related AEs, and TE treatment-related SAEs will be summarized by PT only in descending order of total frequency.

In addition, data listings will be provided for the following:

- All AEs, indicating whether the event is treatment emergent
- All SAEs
- All Deaths
- All SAEs leading to death (i.e., outcome of death)
- All AEs leading to premature discontinuation of study drug

- All AEs leading to premature discontinuation of study

7.2. Laboratory Evaluations

Laboratory data collected during the study will be analyzed and summarized using both quantitative and qualitative methods. Summaries of laboratory data will be provided by Cohort, subtotal for Cohort 2-4 and overall based on Safety Analysis Set and will include data throughout the trial.

For subjects who have permanently discontinued study drug, or all available data at the time of the database snapshot for subjects who were ongoing at the time of an interim analysis. The analysis will be based on values in standard units. When values are below the LOQ, they will be listed as such, and the closest imputed value will be used for the purpose of calculating summary statistics. Hemolized test results will not be included in the analysis, but they will be listed in by-subject laboratory listings.

A by-subject listing for laboratory test results will be provided by subject ID number, age, sex, race, body weight at Screening and visit in chronological order for hematology, serum chemistry, and urinalysis separately. Values falling outside of the relevant reference range and/or having a severity of Grade 1 or higher based on NCI-CTCAE Version 5.0 grading scale will be flagged in the data listings, as appropriate.

No formal statistical testing is planned.

7.2.1. Summaries of Numeric Laboratory Results

Descriptive statistics will be provided by treatment for each laboratory test specified in the study protocol as follows:

- Baseline values
- Values at each post-baseline visit
- Change from baseline at each post-baseline visit

A baseline laboratory value will be defined as the last measurement obtained on or prior to the date/time of first dose of study drug. Change from baseline to a post-baseline visit will be defined as the visit value minus the baseline value.

7.2.2. Graded Laboratory Values

The NCI-CTCAE Version 5.0 will be used to assign toxicity grades (1 to 5) to laboratory results for analysis. For laboratory tests with criteria for both increased and decreased levels, analyses for each direction (i.e., increased, decreased) will be presented separately.

7.2.2.1. Treatment-Emergent Laboratory Abnormalities

Treatment-emergent laboratory abnormalities are defined as values that increase at least 1 toxicity grade from baseline at any post-baseline visit. If the relevant baseline laboratory value is missing, any abnormality of at least Grade 1 observed within the time frame specified above will be considered treatment-emergent laboratory abnormalities.

7.2.2.2. Summaries of Laboratory Abnormalities

Laboratory data that are categorical will be summarized using the number and percentage of subjects in the study with the given response at baseline and each scheduled post-baseline visit.

The following summaries (number and percentage of subjects) for treatment-emergent laboratory abnormalities will be provided by lab test and treatment; subjects will be categorized according to the most severe post-baseline abnormality grade for a given lab test:

- Graded laboratory abnormalities

For all summaries of laboratory abnormalities, the denominator is the number of subjects with non-missing post-baseline values.

7.2.3. Liver-related Laboratory Evaluations

Liver-related abnormalities after initial study drug dosing will be examined and summarized using the number and percentage of subjects who were reported to have the following laboratory test values for postbaseline measurements:

- Aspartate aminotransferase (AST): (a) > 3 times of the upper limit of reference range (ULN); (b) > 5 x ULN; (c) > 10 x ULN; (d) > 20 x ULN
- Alanine aminotransferase (ALT): (a) > 3 x ULN; (b) > 5 x ULN; (c) > 10 x ULN; (d) > 20 x ULN
- AST or ALT: (a) > 3 x ULN; (b) > 5 x ULN; (c) > 10 x ULN; (d) > 20 x ULN
- Total bilirubin: > 2 x ULN
- Alkaline phosphatase (ALP) > 1.5 x ULN
- AST or ALT > 3 x ULN and total bilirubin: (a) > 1.5 x ULN; (b) > 2 x ULN

The summary will include data from all post-baseline visits. For individual laboratory tests, subjects will be counted once based on the most severe post-baseline values. For both the composite endpoint of AST or ALT and total bilirubin, subjects will be counted once when the criteria are met at the same post-baseline visit date. The denominator is the number of subjects in

the Safety Analysis Set who have non-missing post-baseline values of all relevant tests at the same post-baseline visit date. A listing of subjects who met at least 1 of the above criteria will be provided.

7.3. Vital Signs

Descriptive statistics will be provided by Cohort for vital signs as follows:

- Baseline value
- Values at each postbaseline visit
- Change from baseline at each postbaseline visit

A baseline value will be defined as the last available value collected on or prior to the date/time of first dose of study drug. Change from baseline to a postbaseline visit will be defined as the postbaseline value minus the baseline value. Vital signs measured at unscheduled visits will be included for the baseline value selection.

No formal statistical testing is planned.

A by-subject listing of vital signs will be provided by subject ID number, sex, age, race, screening body weight and visit in chronological order.

7.4. Prior and Concomitant Medications

Medications collected at screening and during the study will be coded using the current version of the World Health Organization (WHO) Drug dictionary.

7.4.1. Prior Medications

Prior HBV medications are defined as any medications taken before a subject took the first study drug.

Prior medications will be summarized by Anatomical Therapeutic Chemical (ATC) drug class Level 3 and preferred name using the number and percentage of subjects for each treatment and overall. A subject reporting the same medication more than once will be counted only once within each ATC drug class when calculating the number and percentage of subjects who received that medication. Medications may appear under multiple ATC drug classes. The summary will be ordered alphabetically by ATC medical class and then by preferred term in order of descending overall frequency within each ATC medical class. For drugs with the same frequency, sorting will be done alphabetically.

For the purposes of analysis, any medication with a start date prior to the first dosing date of study drug will be included in the prior medication summary regardless of when the stop date is.

If a partial start date is entered the medication will be considered prior unless the month and year (if day is missing) or year (if day and month are missing) of the start date are after the first dosing date. Medications with a completely missing start date will be included in the prior medication summary, unless otherwise specified.

Summaries will be based on the Safety Analysis Set. No formal statistical testing is planned.

7.4.2. Concomitant Medications

Concomitant medications are defined as medications taken while a subject took study drug. Use of concomitant medications will be summarized by Anatomical Therapeutic Chemical (ATC) drug class Level 3 and preferred name using the number and percentage of subjects for each treatment. A subject reporting the same medication more than once within each ATC drug class will be counted only once when calculating the number and percentage of subjects who received that medication. Medications may appear under multiple ATC drug classes. The summary will be ordered alphabetically by ATC medical class and then by preferred term in descending overall frequency within each ATC medical class. For drugs with the same frequency, sorting will be done alphabetically.

For the purposes of analysis, any medications with a start date prior to or on the first dosing date of study drug and continued to be taken after the first dosing date, or started after the first dosing date but prior to or on the last dosing date of study drug will be considered concomitant medications. Medications started and stopped on the same day as the first dosing date or the last dosing date of study drug will also be considered concomitant. Medications with a stop date prior to the date of first dosing date of study drug or a start date after the last dosing date of study drug will be excluded from the concomitant medication summary. If a partial stop date is entered, any medication with the month and year (if day is missing) or year (if day and month are missing) prior to the date of first study drug administration will be excluded from the concomitant medication summary. If a partial start date is entered, any medication with the month and year (if day is missing) or year (if day and month are missing) after the study drug stop date will be excluded from the concomitant medication summary. Medications with completely missing start and stop dates will be included in the concomitant medication summary, unless otherwise specified. Summaries will be based on the Safety Analysis Set. No formal statistical testing is planned.

All prior and concomitant medications (other than per-protocol study drugs) will be provided in a by-subject listing sorted by subject ID number, age, sex, race, screening body weight and administration date in chronological order.

7.5. Electrocardiogram Results

A by-subject listing for ECG assessment results will be provided by subject ID number, sex, age, race, screening body weight.

7.6. Other Safety Measures

No additional safety measures are specified in the protocol.

8. ADA

Desriptive statistics based on ADA Analysis Set will be performed for ADA.

9. PHARMACOKINETIC EVALUATION/ANALYSIS

Individual subject concentration data for BRII-835 and its metabolite A-312327 will be listed and summarized using descriptive statistics based on PK Analysis Set. Summary statistics (numbers of subjects, arithmetic mean, SD, coefficient of variation [CV], median, minimum, maximum, geometric mean and Geometric CV) will be presented by time point and Cohort.

No PK parameters will be reported in this study since only limited (sparse) PK samples were collected. The concentration data from this study will be pooled with data from other studies for population PK analysis, which will be reported separately.

10. CHANGES FROM PROTOCOL-SPECIFIED ANALYSES

10.1. Changes From Protocol-Specified Efficacy Analyses

There are no deviations from the protocol-specified efficacy analyses. SAP combined the primary estimand 1 and estimand 2 of protocol into one primary estimand, intercurrent events are redefined for this combined primary estimand as in SAP section 6.1.2. For the secondary estimand(s), SAP emphasized that the use of rescue medication will be treated as "treatment failure" (composite policy) for HBV DNA suppression related variables

10.2. Changes From Protocol-Specified Safety Analyses

There are no deviations from the protocol-specified safety analyses.

10.3. Changes From Protocol Interim Analyses

The first interim analyses originally would be conducted when approximately 45 enrolled participants of Cohorts 1-3 complete the 48-week treatment in protocol, actually conducted when 55 participants of Cohorts 1-3 finished the 48-week treatment.

Considering that cohort 4 may also have been completed 24 weeks follow-up post EOT when the third interim analysis is conducting, the third analysis will contain cohort 4 and be conducted when last available participant of Cohorts 1-4 completes follow-up 24 weeks post end of treatment.

10.4. Changes From Protocol Sample Size

Up to 75 participants originally will be enrolled for Cohorts 1-3 and 45 participants enrolled for Cohort 4 in protocol. Actually, 55 participants enrolled in Cohorts 1-3 and 31 participants in Cohort 4. The second, subsequent interim analysis and final analysis will be conducted based on the above actually enrolled participants.

11. SOFTWARE

SAS® Software Version 9.4. SAS Institute Inc., Cary, NC, USA.

12. SAP REVISION

Revision Version/ Date (DD MMM YYYY)	Section	Summary of Revision	Reason for Revision
v2.0/20Feb2025	2.1.Interim Analyses	• Cohorts 4 will be conducted in the third interim analysis;<br>• Describes the interim analysis content with the corresponding timepoints.	Cohorts 4 data follow-up 24 weeks post end of treatment will be available when the third interim analysis
	3.5.Subgroup analysis	• Change the Subgroup definition for Cohort 4: responder vs non-responder by Week 44 in BRII-179-835-001 study.<br>• HBsAg change over time will be summarized by cohort 1- 4 based on baseline HBsAg level categories, excluded HBsAg <=1000 IU/mL, that was done in the first interim analysis, not required in subsequent analyses, depending on the exploratory needs.	Additional subgroup exploratory analysis based on medical considerations
	6.1.Primary Efficacy Endpoint	• Bayesian hierarchy logistics regression will be used for cohort 1-3, For cohort 4 a unstratified Bayesian estimation will be conducted.	Hierarchy Bayesian only applied to cohort 1-3 due to stratified randomization, and cohort 4 will be used unstratified Bayesian model.
	6.2.Secondary Efficacy Endpoints	• Time to achieve first time of serum HBsAg loss through Week 72: Cohort 4 will stratified by updated subgroup definition in section 3.5	Updated subgroup definition based on medical considerations
	10. Changes From Protocol-Analyses	• Add the changes from the protocol-specified analyses	Descrbe the key changes from the protocol-specified analyses

STATISTICAL ANALYSIS PLAN

**Study Title:**	A Phase 2 Multicenter, Randomized, Open-label Study to Investigate the Efficacy and Safety of BRII-835 (VIR-2218) and Pegylated Interferon Alpha (PEG-IFNα) Combination Therapy for the Treatment of Chronic Hepatitis B Virus (HBV) Infection
**Name of Test Drug:**	BRII-835 (VIR-2218); Pegylated Interferon Alpha (PEG-IFNα)
**Study Number:**	BRII-835-002
**Protocol Version (Date):**	2.0 (31 August 2023)
**Analysis Type:**	Phase 2
**Analysis Plan Version:**	3.0
**Analysis Plan Date:**	31 Jul 2025
**Analysis Plan Author(s):**	[redacted]

CONFIDENTIAL AND PROPRIETARY INFORMATION

SIGNATURE PAGE

Reason for Signature	Name	Signature	Date (DDMMMYYYY)
Author	[redacted]	[redacted]	
Therapeutic Area Head (or delegate)	[redacted]		
Chief Medical Officer (or delegate)	[redacted]		

TABLE OF CONTENTS

STATISTICAL ANALYSIS PLAN 1
TABLE OF CONTENTS 3
LIST OF ABBREVIATIONS 5
1. INTRODUCTION 7
1.1. Study Objectives 7
1.2. Study Design 7
1.2.1. Planned Cohorts and Dosing 8
1.2.2. NRTI Discontinuation 9
1.3. Sample Size and Power 10
2. TYPE OF PLANNED ANALYSIS 13
2.1. Interim Analyses 13
2.1.1. Dose Escalation Analysis 13
2.2. Final Analysis 13
3. GENERAL CONSIDERATIONS FOR DATA ANALYSES 15
3.1. Key Definitions 16
3.2. Analysis Sets 17
3.2.1. Screened Analysis Set 18
3.2.2. Full Analysis Set (FAS) 18
3.2.3. Safety Analysis Set (SAS) 18
3.2.4. Modified Intent-To-Treat Analysis Set (mITT) 18
3.2.5. Modified Efficacy Analysis Set 18
3.2.6. Per Protocol Set (PP) 18
3.2.7. Pharmacokinetic Analysis Set (PAS) 19
3.2.8. ADA Analysis Set 19
[redacted] 19
3.3. Subject Grouping 19
3.4. Strata and Covariates 19
3.5. Sensitivity and Subgroup Analysis 19
3.6. Multiple Comparisons 20
3.7. Missing Data and Outliers 20
3.7.1. Missing Data 20
3.7.2. Outliers 20
3.8. Data Handling Conventions and Transformations 20
3.9. Analysis Visit Windows 22
3.9.1. Selection of Data in the Event of Multiple Records in an Analysis Visit Window 26
4. SUBJECT DISPOSITION 28
4.1. Subject Enrollment and Disposition 28
4.1.1. Duration of Exposure to Study Drug 29
4.1.2. Adherence to Study Drug 29
4.2. Protocol Deviations 30
5. BASELINE CHARACTERISTICS 31

5.1. Demographics and Baseline Characteristics 31
5.2. Other Baseline Characteristics 31
5.3. Medical History 31
6. EFFICACY ANALYSES 32
6.1. Primary Efficacy Endpoint 32
6.1.1. Definition of the Primary Efficacy Endpoint: 32
6.1.2. Estimand 32
6.1.3. Analysis methods for the primary endpoints 33
6.2. Secondary Efficacy Endpoints 35
6.2.1. Estimand for the secondary endpoints: 35
6.2.2. Analysis Methods for Secondary Efficacy Endpoint 36
[redacted] [redacted]: 37
7. SAFETY ANALYSES 39
7.1. Adverse Events and Deaths 39
7.1.1. Adverse Event Dictionary 39
7.1.2. Adverse Event Severity 39
7.1.3. Relationship of Adverse Events to Study Drug 39
7.1.4. Serious Adverse Events 39
7.1.5. Treatment-Emergent Adverse Events 39
7.1.6. Summaries of Adverse Events and Deaths 40
7.2. Laboratory Evaluations 42
7.2.1. Summaries of Numeric Laboratory Results 42
7.2.2. Graded Laboratory Values 42
7.2.3. Liver-related Laboratory Evaluations 43
7.3. Vital Signs 44
7.4. Prior and Concomitant Medications 44
7.4.1. Prior Medications 44
7.4.2. Concomitant Medications 45
7.5. Electrocardiogram Results 45
7.6. Other Safety Measures 45
8. ADA 46
9. PHARMACOKINETIC EVALUATION/ANALYSIS 47
10. CHANGES FROM PROTOCOL-SPECIFIED ANALYSES 48
10.1. Changes From Protocol-Specified Efficacy Analyses 48
10.2. Changes From Protocol-Specified Safety Analyses 48
10.3. Changes From Protocol-Interim Analyses 48
10.4. Changes From Protocol-Sample Size 48
11. SOFTWARE 49
12. SAP REVISION 50

LIST OF ABBREVIATIONS

ADA	anti-drug antibody
AE	adverse event
ALT	alanine aminotransferase
AST	aspartate aminotransferase
BLQ	below the limit of quantitation
BMI	body mass index
CFR	Code of Federal Regulations
CI	confidence interval
CK	creatine kinase
CRF	case report form
CSR	clinical study report
CTCAE	Common Toxicity Criteria for Adverse Events
ECG	Electrocardiogram
FAS	Full Analysis Set
Hb	Hemoglobin
HBV	hepatitis B virus
HLT	high-level term
ICH	International Conference on Harmonization (of Technical Requirements for Registration of Pharmaceuticals for Human Use)
ITT	intent to treat
LLT	lower-level term
LOQ	limit of quantitation
MedDRA	Medical Dictionary for Regulatory Activities
mITT	modified intent to treat
NrtI	nucleos(t)ide reverse transcriptase inhibitors
PBMC	peripheral blood mononuclear cell
PK	pharmacokinetic
PP	per protocol
PT	preferred term
Q1, Q3	first quartile, third quartile
SAP	statistical analysis plan
SC	subcutaneously
SD	standard deviation
SI (units)	international system of units
SOC	system organ class
SRC	safety review committee
TEAE	treatment-emergent adverse event
TFLs	tables, figures, and listings

ULN	upper limit of normal
WHO	World Health Organization

1. INTRODUCTION

This statistical analysis plan (SAP) describes the statistical analysis methods and data presentations to be used in tables, figures, and listings (TFLs) in the clinical study report (CSR) for Study BRII-835-002. This SAP is based on the study protocol version 2.0 dated 31 Aug 2023 and the electronic case report form (eCRF). The SAP will be finalized before database finalization. Any changes made after the finalization of the SAP will be documented in the CSR.

This statistical analysis plan (SAP) describes the statistical analysis methods and data presentations to be used in tables, figures, and listings (TFLs) of three interim analyses for Study BRII-835-002. The first two interim analyses will be conducted when approximately 45 enrolled participants of Cohorts 1-3 complete the 48-week treatment and last available participant of all enrolled participants (Cohorts 1-4) completes the 48-week treatment, respectively. If the pace of enrollment is robust, and the primary endpoint proportion of participants with HBsAg loss at Week 48 is expected to occur less or equal to two months between the two interim analyses, the two analyses may be collapsed into one analysis.

1.1. Study Objectives

The primary objective of this study is as follows:

- To evaluate the efficacy and safety of BRII-835 and PEG-IFNα combination therapy in participants with chronic HBV infection

The secondary objectives of this study are as follows:

- To assess the antiviral activity of BRII-835 and PEG-IFNα combination therapy
- To assess BRII-835 pharmacokinetic (PK) exposure and evaluate the anti-drug antibodies (ADA) to BRII-835

[redacted]

[redacted]

1.2. Study Design

This is a Phase 2 open-label, randomized study of BRII-835 and PEG-IFNα combination therapy in adult participants with chronic HBV infection without cirrhosis receiving NRTI therapy as background therapy. This study is designed to evaluate the efficacy and safety of BRII-835 and PEG-IFNα combination therapy. Two different dose levels of BRII-835 in combination with PEG-IFNα will be evaluated.

Participants with HBeAg negative or HBeAg positive chronic HBV infection are eligible to participate in Cohorts 1-3 of this study. Eligible participants will be stratified by their serum HBsAg level at screening and randomized 1:1:1 to each of the three cohorts. Up to 75 participants are planned for Cohorts 1-3.

In addition, this study will evaluate the efficacy of BRII-835 and PEG-IFNα combination in participants previously treated with BRII-179, a therapeutic recombinant HBV vaccine. Eligible participants who completed BRII-179-835-001 study will roll over to Cohort 4. Up to 45 participants will be enrolled.

All participants will remain outpatient during study participation and will be followed for the safety, tolerability, and antiviral activity after completion of study intervention. The assessments and procedures performed at each visit are further described in the SoA (Section 1.3) of the protocol.

Participants will be assessed by the investigator for their eligibility to discontinue NRTI therapy at Week 72. Participants who meet the NRTI discontinuation criteria may enter the NRTI discontinuation monitoring period for an additional 24 weeks. Participants who do not meet the NRTI discontinuation criteria will continue NRTI therapy and will complete the study.

The total duration of study participation for each participant will include screening period (up to 4 weeks), dosing period (48 weeks), follow-up period (24 weeks), and NRTI discontinuation monitoring period (24 weeks when applicable). The study design schema can be found in Section 1.2 in the protocol.

1.2.1. Planned Cohorts and Dosing

Cohorts 1-3 (BRII-179 naïve):

Eligible participants of Cohorts 1-3 will be stratified by their serum HBsAg level at screening (>100 IU/mL and ≤ 500 IU/mL, > 500 and ≤ 1,500 IU/mL, or > 1,500 and ≤ 3,000 IU/mL) and randomized to receive the study intervention per the following dose regimens.

- Cohort 1: Each participant will receive 48 doses of PEG-IFNα 180 mcg administered SC every week starting on Day 1
- Cohort 2: Each participant will receive 48 doses of PEG-IFNα 180 mcg administered SC every week and 13 doses of BRII-835 200 mg administered SC every 4 weeks, both starting on Day 1
- Cohort 3: Each participant will receive 48 doses of PEG-IFNα 180 mcg administered SC every week and 13 doses of BRII-835 100 mg administered SC every 4 weeks, both starting on Day 1

Cohort 4 (BRII-179 treated):

Eligible participants who completed BRII-179-835-001 study will be assigned to Cohort 4 and receive the following study intervention.

Cohort 4: Each participant will receive 48 doses of PEG-IFNα 180 mcg administered SC every week and 13 doses of BRII-835 100 mg administered SC every 4 weeks, both starting on Day 1

Safety Review Committee: A Safety Review Committee (SRC) for this study will monitor participant safety and consider study-specific data as well as relevant background information about the study drugs and target population under study to inform on study conduct. The primary purpose of the SRC is to protect the safety of participants in this study.

Ad hoc SRC meetings will take place as needed, i.e., for a significant safety event such as criteria for study intervention discontinuation being reached. Decisions to suspend dosing or discontinue individual participants from study intervention will be made according to the predetermined criteria (Section 7 in the protocol). Participants may resume study intervention only following SRC approval. Additionally, the SRC may recommend discontinuation of or changes to the study to the Sponsor. The SRC charter outlining the member composition and operating procedures will be developed before the study initiation.

1.2.2. NRTI Discontinuation

NRTI Discontinuation Criteria

Upon participants completing Week 72 visits, the investigator will evaluate whether participants are eligible for discontinuation of NRTI therapy based on the following NRTI discontinuation criteria:

- HBsAg < LLOQ AND
- HBV DNA < LLOQ AND
- Undetectable HBeAg AND
- ALT ≤ 2x ULN

Participants who meet all of the above criteria for at least two consecutive visits at Week 72 are eligible to discontinue from NRTI therapy. Investigator should confirm participants' eligibility as early as possible within 2 weeks post Week 72 visit. Participants who discontinue NRTI will enter the NRTI Discontinuation Monitoring Period of the study within 1 week of the investigator confirming eligibility. Participants will not take their NRTI medications on the first day of the NRTI Discontinuation Monitoring Period and will be followed for an additional 24 weeks per the SoA (Section 1.3) in the protocol.

NRTI Re-treatment

It is recommended that the Investigator should consider re-treatment with NRTI therapy during the NRTI Discontinuation Monitoring Period if the participant meets any of the following criteria:

- ALT > 10x ULN with HBV DNA ＞LLOQ at the same visit
- ALT 5-10x ULN with HBV DNA ＞LLOQ persisting for ≥ 4 consecutive weeks
- ALT > ULN and HBV DNA > 2,000 IU/mL persisting for ≥ 4 consecutive weeks

- ALT > ULN with total bilirubin > 2x ULN and/or prolongation of prothrombin time (PT) > 2 seconds, and HBV DNA > LLOQ at the same visit
- HBV DNA > 100,000 IU/mL at any visit
- Confirmed HBeAg seroreversion (i.e., HBeAg positive after being HBeAg negative at NRTI discontinuation)
- Any other clinically significant event(s) warranting initiation of NRTI therapy in the opinion of the Investigator

Whether participants should be re-treated with NRTI is at the discretion of the Investigator following good medical judgement. Participants who are re-treated with NRTI therapy should continue to be followed per the SoA (Section 1.3.2) in the protocol for the duration of the 24-week NRTI Discontinuation Monitoring Period.

Participants with HBV DNA increase ≥ 2 $\log_{10}$ IU/mL within a 2-week period should:

- Return to the site for an unscheduled visit to measure HBV DNA levels and liver function tests as soon as possible after the investigator being aware of the HBV DNA increase
- Continue returning to site weekly until HBV DNA levels stabilize (do not increase ≥ 1 $\log_{10}$ IU/mL over a 4-week period or start decreasing for at least 2 consecutive visits) after which they will continue follow-up per the SOA for the NRTI Discontinuation Monitoring Period
- If any unscheduled visits needed as outlined above coincide with an existing visit per the SoA in the protocol, the scheduled assessments should be conducted

1.3. Sample Size and Power

Up to 75 participants will be randomly assigned in 1:1:1 ratio to each of Cohorts 1-3. A Bayesian framework will be used to calculate the sample size. It is assumed that the number of responders follows a Binomial distribution, with a weakly informative prior (Beta (0.5, 0.5)) for the true response rate. The precision for a range of response rates with 95% credible intervals are shown in Table 1:

Up to 45 participants will be enrolled in Cohort 4. No formal sample size is calculated due to the exploratory nature of Cohort 4.

Table 1 95% Credible Interval of Response Rate by Sample Size for Cohorts 1-3

Sample size per cohort	Number of responders	Response rate (%)	95% Credible interval* (%)
15	4	27	8-50
	5	33	13-57
	6	40	18-63

	7	47	25-71
	8	53	29-76
	9	60	36-82
25	6	24	10-41
	7	28	13-46
	8	32	16-51
	9	36	19-55
	10	40	22-58
	11	44	26-63
	12	48	30-67
	13	52	32-70

* 95% highest posterior density interval.

For the 15 participants in each cohort the lower bounds of 95% credible interval exclude the response rate of 10% if observed response rate is greater than 27% and exclude the response rate of 20% if observed response rate is greater than 40% and exclude the response rate of 30% if observed response rate is greater than 53%.

For the 25 participants in each cohort the lower bounds of 95% credible interval exclude the response rate of 10% if observed response rate is greater than 24% and exclude the response rate of 20% if observed response rate is greater than 36% and exclude the response rate of 30% if observed response rate is greater than 48%.The operational characteristics are based on posterior confidence that the true rate exceeds a threshold of interest, as shown in Table . For instance, when 4 out of 15 participants or 6 out of 25 participants are responders, the probability (true response rate>15%) is larger than 85%.

Table 2 Operational Characteristics by Sample Size

**Sample size per cohort**	**Number of responders**	**Probability (True response rate)>15%**	**Probability (True response rate)>20%**	**Probability (True response rate)>25%**	**Probability (True response rate)>30%**	**Probability (True response rate)>35%**
15	1	18.4%	8.5%	3.7%	1.4%	0.5%
	2	45.7%	27.2%	14.4%	7%	3.2%
	3	72.8%	52.6%	34.1%	20.1%	10.9%
	4	89.7%	75.6%	57.9%	40.6%	25.7%

	5	96.8%	90.4%	78.3%	62.7%	45.7%
	6	99.2%	96.6%	90.7%	80.4%	66.6%
	7	99.9%	99.2%	96.9%	92%	83.4%
25	2	16.2%	5.4%	1.6%	0.4%	0.1%
	3	35.7%	16.1%	6%	1.9%	0.5%
	4	58.2%	32.1%	14.9%	5.8%	1.9%
	5	77.4%	52.1%	29.4%	13.8%	5.5%
	6	89.1%	70.5%	46.9%	26.3%	12.1%
	7	95.9%	84.8%	65.5%	42.7%	23.6%
	8	98.5%	92.5%	79.4%	59.8%	38.3%
	9	99.6%	97.4%	89.6%	75%	55.2%
	10	99.9%	99%%	95.2%	86.1%	70.3%
	11	100%	99.7%	98.2%	93.3%	82.6%

2. TYPE OF PLANNED ANALYSIS

2.1. Interim Analyses

Prior to the final analysis, three interim analyses will be conducted.

The first two interim analyses will be conducted when approximately 45 enrolled participants of Cohorts 1-3 complete the 48-week treatment and last available participant of all enrolled participants (Cohorts 1-4) complete the 48-week treatment, respectively. If the pace of enrollment is robust, and the proportion of participants with HBsAg loss at Week 48 is expected to occur less or equal to two months between the two interim analyses, the two analyses may be collapsed into one analysis. Endpoints with measurement time after the 48-week treatment except for safety relevant data will not be analyzed in the first two interim analyses.

The sponsor had decided to stop participant enrollment in December 2023, with a total of 55 participants enrolled in Cohorts 1-3. Consequently, the study team had agreed that the first interim analysis conducted when 55 participants completed the 48-week treatment. During this interim analysis, only descriptive statistics had been performed, and no per-protocol (PP) analysis was conducted.

The second interim analysis will still at the timepoint when the last participant of Cohorts 1-4 complete the 48 week treatment.

The third interim analysis was originally planned to be conducted when last available participant of Cohorts 1-3 completes follow-up 24 weeks post end of treatment. The sponsor decided to conduct the third interim analysis when last available participant of Cohorts 1-4 completes follow-up 24 weeks post end of treatment (see Section 10.3).

Sponsor further decided analysis based on per-protocol population will not be conducted at second interim analysis. Starting from second interim analysis in addition to descriptive statistics all available statistical analysis will be performed. The second interim analysis will include all efficacy endpoints except for PK and ADA of BRII-835 throughout Week 48, and the endpoints with measurement time after the 48-week treatment except for safety relevant data will not be analyzed in the second interim analyses. The third interim analysis will also include the efficacy endpoints of the corresponding time points except for PK and ADA.

PK and ADA analysis will be performed at the final analysis. [redacted]

2.1.1. Dose Escalation Analysis

Not Applicable.

2.2. Final Analysis

The final analysis will be performed when last available participant of Cohorts 1-4 completes the last scheduled visit according to the SoA.

3. GENERAL CONSIDERATIONS FOR DATA ANALYSES

All data collected on the electronic case report form (eCRF) will be presented in the data listings and will be listed by subject identification number (in ascending order), sex, age at screening, screening body weight and visit, where applicable. All summaries will be presented by Cohort.

When reporting descriptive statistics, the following rules will be applied, except for PK concentration data:

- Continuous variables: Descriptive statistics will include the number of non-missing values (n), arithmetic mean, standard deviation (SD), median, first quartile (Q1), third quartile (Q3), minimum and maximum values.

  The minimum and maximum values will be displayed to the same decimal precision as the source data; the Q1, Q3, arithmetic mean and median values will be displayed to one more decimal than the source data; SD value will be displayed to two more decimal than the source data for the specific variable.

  The appropriate precision for derived variables will be determined based on the precision of the data on which the derivations are based, and statistics will be presented in accordance with the abovementioned rules.

  n will be an integer. If no subjects have data at a given time point, then only n=0 will be presented.

  All data will be presented to a maximum of 2 decimal places.

- Categorical variables: Descriptive statistics will include counts and percentages per category. The denominator in all percentage calculations will be the number of subjects in the relevant analysis set with non-missing data, unless specifically stated otherwise. Percentages will be displayed to one decimal place.

  Percentage change from baseline values will be calculated and displayed to 1 decimal place in the listings. In the summaries, the mean and median percentage change from baseline values will be presented to 2 decimal places, the minimum and maximum values will be presented to 1 decimal place and the SD value will be presented to 3 decimal places.

  For categories where all subjects fulfill certain criteria, the percentage value will be displayed as 100. For categories where zero subjects fulfill certain criteria, there will be no percentage displayed. All other percentage displays will use 1 decimal place. Data listings will contain all reported and derived data.

Unless otherwise stated, the following methods will be applied:

- Repeat/unscheduled assessments: Values collected at unscheduled study visits/time points will be allocated to scheduled visit/time points according to the algorithms of analysis visit window together with values collected at scheduled visits/time points and be presented in summary tables. All collected data will be included in the data listings.

- Assessment windows: All assessments will be included in the data listings and no visit windows will be applied to exclude assessments that were performed outside of the protocol specified procedure windows.
- Result display convention: Results will be center aligned in all summary tables and listings. Subject identifiers, visits, and parameter labels may be left-aligned if required.
- Date and time display conventions: The following display conventions will be applied in all outputs where dates and/or times are displayed:
  - Date only: YYYY-MM-DD
  - Date and time: YYYY-MM-DD HH:MM

If only partial information is available, unknown components of the date or time will be presented as 'NK' (not known), i.e., '2016-NK-NK'. Time will be reported in military time.

3.1. Key Definitions

The following definitions will be used:

- Baseline: The baseline value is defined as the last available, valid (quantifiable continuous or categorical value), and non-missing observation for each subject prior to first study drug administration unless otherwise specified. Repeat and unscheduled assessments will be included in the derivation of the baseline values
- Change from Baseline: The change from baseline value is defined as the difference between the result collected/derived at a post-baseline visit/time point and the baseline value.

  The change from baseline value at each post-baseline visit/time point will be calculated for all continuous parameters using the following formula:

  Change from Baseline Value = Result at Visit/Time Point – Baseline Value

  The change from baseline value will only be calculated if the specific post-baseline visit/time point result and the baseline value for the parameter are both available; otherwise, will be treated as missing.
- Study day: The study day of an event is defined as the relative day of the event starting with the date of the first study drug administration (reference date) as Day 1 (there will be no Day 0).

  The study day of events occurring before the first study drug administration will be calculated as:

  Study Day = (Date of Event - Date of First Study Drug Administration)

  For events occurring on or after Day 1, study day will be calculated as:

  Study Day = (Date of Event - Date of First Study Drug Administration) + 1

Study days will only be calculated for events with complete dates and will be undefined for events that are 'Ongoing' at the end of the study.

- Relative days compared to an alternative reference point will be calculated similarly, but the alternative starting reference start date will be used instead of the date of the first study drug dosing.
- HBsAg loss: HBsAg changing from positive at baseline to <LLOQ at any post-baseline visit.
- Anti-HBs seroconversion: Anti-HBs changing from negative (<10 IU/L) at baseline to positive (>=10 IU/L) at any post-baseline visit.
- HBsAg seroconversion: HBsAg loss plus anti-HBs positivity (>=10 IU/L) at any post-baseline visit
- Sustained HBsAg loss: quantitative HBsAg < LLOQ at all visits (scheduled and unscheduled) for ≥ 24 weeks (≥ 161 days, i.e. 168 days (24 weeks) minus 7 days (Visit window)). Sustained days = (last date of HBsAg <LLOQ - first date of HBsAg <LLOQ) +1, during which the values at all visits < LLOQ.
- Anti-HBe seroconversion: Anti-HBe changing from negative or missing at baseline to positive at any post-baseline visit.
- HBeAg loss ($HBeAg^{+}$ subjects only): HBeAg changing from positive at baseline to negative at any post-baseline visit.
- HBeAg seroconversion (HBeAg+ subjects only): HBeAg loss plus anti-HBe positivity at any post-baseline visit.
- Functional cure: sustained undetectable HBsAg (HBsAg < LLOQ) and sustained suppression of HBV DNA < LLOQ ≥ 24 weeks including all visits (scheduled and unscheduled) after discontinuation of all treatment, including NRTI.
- Modified Functional cure: HBsAg <LLOQ and HBV DNA<LLOQ at each analysis window in the NRTI discontinuation monitoring period. Any observation of HBsAg>= LLOQ or HBV DNA >= LLOQ must be confirmed at a consecutive visit (including unscheduled visits) for the participants to be classed as having lost their sustainability, Participants who have a value of HBsAg >= LLOQ or HBV DNA >=LLOQ at their last visit, which cannot be confirmed due to no further follow up, will be treated as 'treatment failure'.

The LLOQ for HBsAg is 0.05 IU/mL, and 10 IU/mL for HBV DNA.

3.2. Analysis Sets

Analysis sets define the subjects to be included in an analysis. Analysis sets and their definitions are provided in this section. The analysis set will be identified and included as a subtitle of each table, figure, and listing.

For each analysis set, the number and percentage of subjects eligible for inclusion will be provided.

A listing of exclusion from analysis sets will be provided by subject.

3.2.1. Screened Analysis Set

The Screened Analysis Set will consist of all subjects who sign the informed consent form. The listing of eligibility criteria will be based on this population.

3.2.2. Full Analysis Set (FAS)

All participants assigned to study intervention will be included in Full Analysis Set.

3.2.3. Safety Analysis Set (SAS)

All participants assigned to study intervention and who take at least 1 dose of study drug. Participants will be analyzed according to the study intervention they received. Participants will be analyzed by Cohort, subtotal for Cohort 2-4 and overall.

All safety analyses will be based on Safety Analysis Set.

3.2.4. Modified Intent-To-Treat Analysis Set (mITT)

All participants assigned to study intervention, who take at least 1 dose of study drug, with at least 1 post-baseline visit through Week 48. Participants will be analyzed according to the study intervention to which they were randomized for Cohorts 1- 3, and for Cohort 4 participants will be analyzed according to the study intervention specified in the protocol.

3.2.5. Modified Efficacy Analysis Set

Participants will be analyzed as well according to modified efficacy analysis set as supplementary analysis. The modified efficacy analysis set is defined as all participants who have baseline HBsAg>100 IU/mL and take at least 1 dose of study drug, with at least 1 post-baseline visit through Week 48.

3.2.6. Per Protocol Set (PP)

The Per Protocol Set will include the subset of mITT who fulfil:

- HBsAg ≥ 0.05 IU/mL at baseline
- Total number of dose compliance ≥ 80% and ≤ 120% for BRII-835 and PEG-IFNα
- Have HBsAg data in the analysis visit window (week 48/week 72) for the primary efficacy endpoints analysis

- Had not identified with important (major) protocol deviation that significantly impacts the interpretation of the primary efficacy endpoints

A Per Protocol review meeting will be held to go through the important protocol deviations to identify the PP population prior to the database lock for the final analysis.

PP Set will be used as analysis population of antiviral activity [redacted]

3.2.7. Pharmacokinetic Analysis Set (PAS)

All participants who received BRII-835, had a at least 1 evaluable PK concentration, and had no protocol deviations thought to impact on the PK data analysis.

3.2.8. ADA Analysis Set

All participants who received BRII-835 and had at least 1 non-missing ADA result from the ADA assay after first dose of the study drug. Participants will be analyzed by Cohort, subtotal for Cohort 2-4 and overall.

[redacted]

[redacted]

3.3. Subject Grouping

For analyses based on the FAS, mITT, SAS and PP Set, subjects will be grouped according to the Cohort.

3.4. Strata and Covariates

Up to 75 participants will be randomly assigned in 1:1:1 ratio among Cohorts 1-3 using an IRT system. Stratification will be based on the following variables:

Screening HBsAg level (>100 IU/mL and ≤500 IU/mL, >500 IU/mL and ≤1,500 IU/mL, >1,500 IU/mL and ≤3,000 IU/mL).

If there are discrepancies in stratification factor values between the IWRS and the clinical database, the values recorded in the clinical database will be used for analyses.

For Cohort 4 no randomization is performed.

3.5. Sensitivity and Subgroup Analysis

In addition, for Cohorts 1-3 combine some two adjacent screening stratification levels (i.e. randomization strata) to reduce HBsAg stratification factors from three levels to two levels

(HBsAg >100 IU/mL and HBsAg ≤1,500 IU/mL versus HBsAg >1,500 IU/mL and HBsAg ≤3,000 IU/mL; HBsAg >100 IU/mL and HBsAg ≤500 IU/mL versus HBsAg >500 IU/mL and HBsAg ≤3,000 IU/mL) to perform the stratified Bayesian hierarchical analysis as sensitivity analyses if model is converged using the similar approach as the primary analysis.

Further stratify Cohort 4 into subgroup analysis listed below:

- Subgroup analysis will be conducted between responder and non-responder by Week 44 in study BRII-179-835-001. Week 44 responder is defined as peak anti-HBs titer ≥ 10 IU/L by Week 44 in BRII-179-835-001 study.

The descriptive statistics for antiviral activity [redacted] will be presented. The unstratified bayesian analysis will be performed by subgroup of cohort 4, respectively with similar method taken as cohort 4.

HBsAg change over time will be summarized by cohort 1- 4 based on baseline HBsAg level categories (HBsAg >100 IU/mL and HBsAg ≤500 IU/mL, HBsAg >500 IU/mL and HBsAg ≤1500 IU/mL, HBsAg >1500 IU/mL and HBsAg ≤3000 IU/mL, HBsAg≤1500 IU/mL). The subgroup of HBsAg <=1000 IU/mL, that had conducted in the first interim analysis, will not be required, but may be conducted depending on the exploratory needs.

3.6. Multiple Comparisons

NA

3.7. Missing Data and Outliers

3.7.1. Missing Data

In general, missing data will not be imputed unless methods for handling missing data are specified. Exceptions are presented in this document.

For missing last dosing date of study drug, imputation rules are described in Section 4.1.1, the handling of missing or incomplete dates for AE onset is described in Section 7.1.5.2, and for prior HBV and concomitant medications in Section 7.4.

3.7.2. Outliers

Outliers will be identified during the data management and data analysis process, but no sensitivity analyses will be conducted. All data will be included in the data analysis.

3.8. Data Handling Conventions and Transformations

The following conventions will be used for the imputation of date of birth when it is partially missing or not collected:

- If only month and year of birth is collected, then "15" will be imputed as the day of birth
- If only year of birth is collected, then "01 July" will be imputed as the day and month of birth
- If year of birth is missing, then date of birth will not be imputed.

If age at Day 1 is not available for a subject, then age derived based on date of birth and the Day 1 visit date will be used instead. If an enrolled subject was not dosed with any study drug, the randomization date will be used instead of the Day 1 visit date. For screen failures, the date the first informed consent was signed will be used for the age derivation. Age required for longitudinal and temporal calculations and analyses (e.g age at date of AE) will be based on age derived from date of birth and the date of the measurement or event, unless otherwise specified.

Non-PK data that are continuous in nature but are less than the lower limit of quantitation (LOQ) or above the upper LOQ will be imputed as follows:

- A value that is 1 unit less than the LOQ will be used to calculate descriptive statistics if the datum is reported in the form of "< x" (where x is considered the LOQ). For example, if the values are reported as < 50 and < 5.0, values of 49 and 4.9, respectively, will be used to calculate summary statistics. An exception to this rule is any value reported as < 1 or < 0.1, etc. For values reported as < 1 or < 0.1, a value of 0.9 or 0.09, respectively, will be used to calculate summary statistics.
- A value that is 1 unit above the LOQ will be used to calculate descriptive statistics if the datum is reported in the form of "> x" (where x is considered the LOQ). Values with decimal points will follow the same logic as above.
- The LOQ will be used to calculate descriptive statistics if the datum is reported in the form of "≤ x" or "≥ x" (where x is considered the LOQ).

Plasma concentrations that are below the limit of quantitation (BLQ) will be presented as "BLQ" in the concentration data listing. BLQ concentrations will be treated as 0 at predose time points, and as missing at postdose time points for determination of summary and order statistics. Geometric mean and geometric CV% will not be calculated if the concentrations is BLQ or missing at predose.

The following conventions will be used for the presentation of summary and order statistics:

- If at least 1 subject has a concentration value of BLQ for the time point, the minimum value will be displayed as "BLQ."
- If more than 25% of the subjects have concentration values of BLQ for a given time point, the minimum value will be displayed as "BLQ."

- If more than 50% of the subjects have concentration values of BLQ for a given time point, the minimum and median values will be displayed as "BLQ."
- If more than 75% of the subjects have concentration values of BLQ for a given time point, the minimum and median values will be displayed as "BLQ."
- If all subjects have concentration values of BLQ for a given time point, all order statistics (minimum, median, and maximum) and summary statistics will be displayed as "BLQ."

3.9. Analysis Visit Windows

Subject visits might not occur on protocol-specified days. Therefore, for the purpose of analysis, observations will be assigned to analysis windows. The analysis visit windows for the parameters at different periods are listed in Table 3.1 – Table 3.6

- Prior to NrtI Discontinuation Monitoring Period

Table 3.1 Analysis Visit Windows for HBsAg Prior to NRTI Discontinuation Monitoring Period

Analysis Visit	Nominal Study Day	Lower Limit	Upper Limit
Baseline	1	None	1
Week 4	29	2	43
Week 8	57	44	71
Week 12	85	72	99
Week 16	113	100	127
Week 20	141	128	155
Week 24	169	156	183
Week 28	197	184	211
Week 32	225	212	239
Week 36	253	240	267
Week 40	281	268	295

Week 44	309	296	323
Week 48	337	324	365
Week 56	393	366	421
Week 64	449	422	477
Week 72	505	478	533

One of the primary endpoints is the proportion of participants with HBsAg loss at weeks post actual end of treatment, a participant discontinues study treatment before the planned EOT (Week 48) and does not withdraw from study participation, he/she will return to the investigational site every 8 weeks upon EOT for the specified assessments and the corresponding analysis windows for HBsAg is as follows

Table 3.2 Analysis Visit Windows of HBsAg at 24 weeks post end of study treatment for participants discontinue study treatment

Analysis Visit	Reference day	Lower Limit	Upper Limit
EoT	1	None	1
FU Week 8	57	2	85
FU Week 16	113	86	141
FU Week 24	169	142	197

Table 3.3 Analysis Visit Windows for Anti-HBs Prior to NRTI Discontinuation Monitoring Period

Analysis Visit	Nominal Study Day	Lower Limit	Upper Limit
Baseline	1	None	1
Week 16	113	2	141
Week 24	169	142	197
Week 32	225	198	253

Week 40	281	254	309
Week 48	337	310	365
Week 56	393	366	421
Week 64	449	422	477
Week 72	505	478	533

Table 3.4 Analysis Visit Windows for HBV DNA Prior to NRTI Discontinuation Monitoring Period

Analysis Visit	Nominal Study Day	Lower Limit	Upper Limit
Baseline	1	None	1
Week 12	85	2	127
Week 24	169	128	211
Week 36	253	212	295
Week 48	337	296	365
Week 56	393	366	449
Week 72	505	450	533

Table 3.5 Analysis Visit Windows for HBeAg , Anti-HBe Prior to NrtI Discontinuation Monitoring Period

Analysis Visit	Nominal Study Day	Lower Limit	Upper Limit
Baseline	1	None	1
Week 16	113	2	169
Week 32	225	170	281
Week 48	337	282	421

Week 72	505	422	533

Tabel 3.6 Analysis Visit Windows for Hematology, Chemistry and Coagulation Assessments Prior to NrtI Discontinuation Monitoring Period

Analysis Visit	Nominal Study Day	Lower Limit	Upper Limit
Baseline	1	None	1
Week 2	15	2	22
Week 4	29	23	43
Week 8	57	44	71
Week 12	85	72	99
Week 16	113	100	127
Week 20	141	128	155
Week 24	169	156	183
Week 28	197	184	211
Week 32	225	212	239
Week 36	253	240	267
Week 40	281	268	295
Week 44	309	296	323
Week 48	337	324	365
Week 56	393	366	421
Week 64	449	422	477
Week 72	505	478	533

- NRTI Discontinuation Monitoring Period

Table 3.7 Analysis Visit Windows for HBsAg, HBV DNA, HBeAg, Hematology, Chemistry and Coagulation during NRTI Discontinuation Monitoring Period

Analysis Visit	NDMP Day	Lower Limit	Upper Limit
NDMP Baseline	1	None	1
NDMP Week 4	29	2	43
NDMP Week 8	57	44	71
NDMP Week 12	85	72	106
NDMP Week 18	127	107	148
NDMP Week 24	169	149	190

*NDMP: NRTI Discontinuation Monitoring Period; NDMP baseline is not applicable for HBeAg according to SOA in the protocol

3.9.1. Selection of Data in the Event of Multiple Records in an Analysis Visit Window

Depending on the statistical analysis method, single values may be required for each analysis window. For example, change from baseline by visit usually requires a single value, whereas a time-to-event analysis would not require 1 value per analysis window.

If multiple valid, nonmissing measurements exist in an analysis window, records will be chosen based on the following rules if a single value is needed:

- For baseline, the last nonmissing value on or prior to the first dosing date of study drug will be selected, unless specified differently. If there are multiple records with the same time or no time recorded on the same day, the baseline value will be the average of the measurements for continuous data, or the measurement with the lowest severity (e.g., normal will be selected over abnormal for safety electrocardiogram findings) for categorical data, and the most conservative value will be selected for efficacy data (e.g., negative will be selected over positive for HBeAg and HBsAg, whereas positive will be selected over negative for Anti-HBe and Anti-HBs).

- For post-baseline values:

  — The most conservative value for safety lab parameters (e.g., abnormal will be selected over normal) within the analysis window will be selected

  - If there are more than 1 value within an analysis window, the value collected closet to the nominal day will be selected.
  - If there are 2 records that are equidistant from the nominal day, the later record will be selected.
- If there is more than 1 record on the selected day, the latest record will be selected; if these measurements were recorded at the same time or no time recorded, the average will be taken for continuous data.

4. SUBJECT DISPOSITION

4.1. Subject Enrollment and Disposition

A summary of subject enrollment will be provided by Cohort for each country/region, investigator and site name and presented in Full Analysis Set. The summary will present the number and percentage of subjects enrolled. For each column, the denominator for the percentage calculation will be the total number of subjects analyzed for that column.

A similar enrollment table will be provided by randomization stratum. The denominator for the percentage of subjects in the stratum will be the total number of enrolled subjects. If there are discrepancies in the value used for stratification assignment between the IWRS and the clinical database, the value collected in the clinical database will be used. A listing of subjects with discrepancies in the value used for stratification assignment between the IWRS and the clinical database at the time of data finalization will be provided.

The randomization schedule used for the study will be provided as an appendix in the CSR.

- A summary of subject disposition will be provided by Cohort. This summary will present the number of subjects screened, subjects who meet all eligibility criteria but not randomized, and the number of subjects in each of the categories listed below:
- All screened subject
- Screen Failure
  - Subjects not met eligibility criteria
  - Subjects met eligibility but not enrolled
- Subject enrolled
- All treated subject
- Completed study drug administration
- Did not complete study drug administration with reasons for premature discontinuation of study drug
- Completed study
- Did not complete the study with reasons for premature discontinuation of study

For the status of study drug and study completion and reasons for premature discontinuation, the number and percentage of subjects in each category will be provided. The denominator for the percentage calculation will be the total number of subjects in the Full Analysis Set corresponding to that column. In addition, a flowchart will be provided to depict the disposition.

The following by-subject listings will be provided by subject identification (ID) number (in ascending order), age, sex and screening body weight to support the above summary tables:

- Reasons for premature study drug or study discontinuation
- Reasons for screen failure (will be provided by screened subject ID number in ascending order)

4.1.1. Duration of Exposure to Study Drug

Duration of treatment is calculated as (Date of Last Dose - Date of First Dose + 1) for the completers and calculated as (Discontinued Date-Date of First Dose +1) for subjects with prematurely discontinuation, regardless of any temporary interruptions in study drug administration, and will be expressed in weeks using up to 1 decimal place (e.g., 4.5 weeks). If the last study drug dosing date is missing, the latest date among the clinical visit date, laboratory sample collection date, and vital signs assessment date that occurred during the dosing-treatment period will be used.

Duration of Treatment and Total number of injections for BRII-835 and PEG-IFNα will be summarized, respectively using descriptive statistics (n, mean, STD, Median, Q1, Q3, Min, Max) for Safety Analysis Set.

In addition, Total number of injections will be summarized by counts and percentages for Safety Analysis Set.

No formal statistical testing is planned.

A by-subject listing of study drug administration and drug accountability will be provided separately by subject ID number, sex, age, race, body weight at Screening, and visit in chronological order.

4.1.2. Adherence to Study Drug

The level of prescribed adherence to the study drug regimen will be determined by the total doses of study drug taken relative to the total doses of study drug specified by the protocol.

The level of prescribed adherence of BRII-835 and PEG-IFNα will be expressed as a percentage using the following formula:

$$\text{Prescribed Adherence (\%)} = \left(\frac{\text{Total Doses of Study Drug}}{\text{Total Doses of Study Drug Specified by Protocol}}\right) \text{ x } 100$$

Descriptive statistics for the level of prescribed adherence with the number and percentage of subjects belonging to adherence categories (e.g., *{< 80%, ≥ 80 to ⩽ 90%, > 90%}*) will be provided by treatment group for the Safety Analysis Set.

No formal statistical testing is planned.

Number of Dose administration will be summarized by Cohort, Cohort 2-4 and overall based on Safety Analysis Set.

4.2. Protocol Deviations

Protocol deviations occurring after subjects entered the study are documented during routine monitoring. The number and percentage of subjects with important protocol deviations, and the total number of important protocol deviations by deviation reason (e.g., nonadherence to study drug, violation of select inclusion/exclusion criteria) will be summarized by Cohort for the Full Analysis Set. In addition, a by-subject listing subjects with all participant level protocol deviation will be provided.

5. BASELINE CHARACTERISTICS

5.1. Demographics and Baseline Characteristics

Subject demographic variables (i.e., age, sex, race, and ethnicity) and baseline characteristics (body weight [in kg], height [in cm], body mass index [BMI; in kg/m^2]) will be summarized by Cohort, subtotal for Cohort 1-3 and overall using descriptive statistics for continuous variables and using number and percentage of subjects for categorical variables. The summary of demographic data will be provided for the Full Analysis Set.

A by-subject demographic listing, including the informed consent date, will be provided by subject ID number (in ascending order), sex, age, race, and body weight at Screening.

5.2. Other Baseline Characteristics

Other baseline characteristics include HBsAg, Log10 transformed HBsAg, Anti-HBs, HBeAg at Screening and HBV genotype. These baseline characteristics will be summarized by Cohort, subtotal for Cohort 1-3 and overall using descriptive statistics for continuous variables and using number and percentage of subjects for categorical variables. The summary of these baseline characteristics will be provided for the Full Analysis Set. No formal statistical testing is planned.

A by-subject listing of other baseline characteristics will be provided by subject ID number (in ascending order), sex, age, race, and body weight at Screening.

5.3. Medical History

Medical history will be collected at screening.

Medical history will be summarized by Cohort, subtotal for Cohort 2-4 and overall by the number and percentage of subjects using the Safety Analysis Set. No formal statistical testing is planned.

Listing of general medical history data will be provided.

6. EFFICACY ANALYSES

Efficacy endpoints analyses will be based on mITT. PP will serve as supplementary analysis only for the primary efficacy endpoints.

6.1. Primary Efficacy Endpoint

6.1.1. Definition of the Primary Efficacy Endpoint:

The primary efficacy endpoints includes

1) Proportion of participants with HBV surface antigen (HBsAg) loss at end of treatment
2) Proportion of participants with HBsAg loss at 24 weeks post end of treatment

An estimation approach with no hypothesis testing is used to analyze the primary endpoints. A Bayesian hierarchy logistics regression will be used to calculate the point estimate and 95% credible interval of the primary endpoints for cohort 1-3, for cohort 4 a unstratified Bayesian estimation will be conducted.

6.1.2. Estimand

Estimand for the primary endpoints:

- Proportion of participants with HBV surface antigen (HBsAg) loss at end of treatment
- Proportion of participants with HBsAg loss at 24 weeks post end of treatment

  - Population: Participants with chronic HBV infection as defined by the inclusion and exclusion criteria to reflect the targeted participant population.
  - Variable:
    - HBsAg loss at end of treatment.
    - HBsAg loss at 24 weeks post end of treatment
  - Treatment: Cohort 1, 2, 3 and 4.
  - Intercurrent events: Use of rescue medication, discontinuation of/interruption of/non-adherence to study intervention(s).

    The use of rescue medication, interruption of, and non-adherence to study intervention(s), will be ignored (treatment policy);

The discontinuation of study intervention(s) will be handled using while-on-treatment strategy for variable 1 and will be ignored (treatment policy) for variable 2.

- Population-level summary: Percentage of participants who achieve HBsAg loss at end of treatment and 24 weeks post end of treatment, respectively.

The last available HBsAg will be used for the analysis of primary endpoint HBsAg loss at end of treatment, if no any HBsAg value collected, the missing value will be handled as 'Non-responder'

6.1.3. Analysis methods for the primary endpoints

The primary endpoints are 1) Proportion of participants with HBsAg loss at end of treatment 2) Proportion of participants with HBsAg loss at 24 weeks post end of treatment.

A Bayesian hierarchical model (Yuen, 2022; Jones, 2011) as primary analysis will be used to estimate the posterior probability of response rate for each cohort of Cohorts 1-3 by incorporating the screening HBsAg level stratification factor (i.e. randomization strata). The point estimate of the primary outcome and 95% equal-tailed credible interval will be drawn from the model.

Model for each group:
Number of responders r_g ~ Binomial (n_g, p_g), g=1,2,3
θ_g =logit(p_g)=log(p_g/(1-p_g))= $\gamma_0+\gamma_1 I_{\{B+\}1}+\gamma_2 I_{\{B+\}2}+\psi_g$, g=1,2,3
where γ_0, γ_1, γ_2, ψ_g are all parameters, Thus,
$\theta_1=\gamma_0+\psi_1$
$\theta_2=\gamma_0+\gamma_1+\psi_2$
$\theta_3=\gamma_0+\gamma_2+\psi_3$
Priors:
γ_k ~ Normal(0, 10^6), k=0,1,2
ψ_g ~ Normal(0, ω^2)
ω ~ Half-normal(1)
Where r_g indicates the number of responders among n_g participants, p_g as the response rate r_g/n_g, θ_g indicates the log odds of treatment response log(p_g/(1-p_g)), index g=1,2,3 refer to the stratum numbers,
γ_k represent fixed effects of screening HBsAg stratification factor, and ψ_g denotes a random effect in stratum g.

Stratum	$I_{\{B+\}1}$	$I_{\{B+\}2}$
HBsAg>100 IU/mL and HBsAg≤500 IU/mL	0	0
HBsAg>500 IU/mL and HBsAg≤1,500 IU/mL	1	0
HBsAg>1,500 IU/mL and HBsAg≤3,000 IU/mL	0	1

$I_{\{B+\}1}$ and $I_{\{B+\}2}$ are the dummy variables of $I_{\{B+\}}$.

For each cohort of Cohorts 1-3, the posterior distribution of response rate $P(p_g|data)$, g=1,2,3 was derived for each stratum using the model specified above.
The posterior distribution of the group-level response rate will be derived using a mixture of the posterior distributions of response rate for each analysis stratum in that group. The weights are proportional to the sample size of each stratum.

Samples from the posterior distributions for all parameters will be obtained using a Markov Chain Monte Carlo (MCMC) sampling technique with 20,000 iterations following a burn-in of size 2000.

Diagnostic tests will be performed to assess convergence and autocorrelation. Alternative programming conventions (blocking, thinning, parameterization, number of chains, seeds, size of burn-in, initial values for each chain) may be explored if, using initial programming code, satisfactory mixing is not achieved within computer memory limitations.

For some models with low number of events (0–1 event), the stratified Bayesian hierarchical model does not converge. In such cases a reduced model that does not consider screening stratification will be fitted. The model estimates the point estimate and 95% highest posterior density credible interval of proportion of participants achieving the primary endpoint assuming the number of responders followed a binomial distribution:

Number of responders $r \sim Binomial(n, p)$

Where p is the response rate, with a non-informative (Jeffrey's) prior: $p \sim Beta(0.5, 0.5)$.

The posterior distribution for p is: $P(p|y, N) \sim Beta(y + 0.5, N - y + 0.5)$.

The same reduced model will be used to estimate the posterior distribution of Cohort 4 as no stratification factor is planned in this Cohort.

In addition, for Cohorts 1-3 combine some two adjacent screening stratification levels (i.e. randomization strata) to reduce HBsAg stratification factors from three levels to two levels (HBsAg >100 IU/mL and HBsAg ≤1,500 IU/mL versus HBsAg >1,500 IU/mL and HBsAg ≤3,000 IU/mL; H BsAg >100 IU/mL and HBsAg ≤500 IU/mL versus HBsAg >500 IU/mL and HBsAg ≤3,000 IU/mL) to perform the stratified Bayesian hierarchical analysis if model is converged using the similar approach as the primary analysis.

Samples from the posterior distribution of the response rates in each cohort of Cohorts 1-3 will be used to obtain posterior probabilities of interest (e.g., Probability [Difference of proportion of achieving primary endpoints between cohorts > threshold Data]); threshold such as 5%, 10%, 15%, 20% or any other percentiles of interest may be explored.

The primary efficacy analysis will be performed within mITT analysis set and a supplemental analysis based on PP analysis set with the similar analysis will be presented.

6.2. Secondary Efficacy Endpoints

6.2.1. Estimand for the secondary endpoints:

- Population: Participants with chronic HBV infection as defined by the inclusion and exclusion criteria to reflect the targeted participant population.
- Variable:

  Continuous variables:

  – Absolute serum HBsAg and change from baseline over time through 24 weeks post end of treatment

  – Nadir serum HBsAg and maximum reduction of serum HBsAg from baseline throughout 48-week treatment

  Categorical variables:

  – HBsAg loss at Week 48

  – Functional cure (defined as sustained undetectable HBsAg and sustained suppression of HBV DNA [<lower limit of quantification (LLOQ)] ≥ 24 weeks after discontinuation of all treatment, including NRTI)

  – HBsAg loss and HBV DNA < LLOQ at 24 weeks after discontinuation of all treatment, including NRTI

  – Participants meeting NRTI discontinuation criteria at 24 weeks post end of treatment

  – Participants with Hepatitis B e antigen (HBeAg) loss and/or HBeAg seroconversion (baseline HBeAg-positive participants) at any timepoint

  – Proportion of participants with HBsAg seroconversion at any timepoint

  Time to event variable:

  – Time to achieve first serum HBsAg loss through Week 72
- Treatment: Cohort 1, 2, 3 and 4.
- Intercurrent events: Data collected will be utilized irrespective of discontinuation of, interruption of, and non-adherence to study intervention(s) or initiating rescue medication (treatment policy). However, the use of rescue medication will be treated as “treatment failure” (composite policy) for HBV DNA suppression related variables.
- Population-level summary: For continuous variables, descriptive statistics such as n, mean, standard deviation, median, interquartile, and ranges will be applied. For categorical variables, percentage of each cohort will be presented. Hazards ratio between the cohort 1-3, and descriptive statistics for cohort 4 will be presented for time to event variables.

Missing value will be censored for time to event variable and will be handled as 'Non-responder' for categorical variables, for the analysis of the endpoint HBsAg loss at Week 48 if the HBsAg value at Week 48 analysis visit window is missing however the HBsAg are loss at both Week 44 and Week 52, the HBsAg value at Weeek 48 will be imputed as HBsAg loss.

6.2.2. Analysis Methods for Secondary Efficacy Endpoint

- Proportion of participants with HBsAg loss at Week 48
- Proportion of participants achieving functional cure (defined as sustained undetectable HBsAg and sustained suppression of HBV DNA <LLOQ ⩾24 weeks after discontinuation of all treatment, including NRTI)
- Proportion of participants achieving HBsAg loss and HBV DNA <LLOQ 24 weeks after discontinuation of all treatment, including NRTI
- Proportion of participants meeting NRTI discontinuation criteria at 24 weeks post end of treatment

A Bayesian hierarchical model similar to primary analysis will be used to analyse the four endpoints above.

- Absolute serum HBsAg and change from baseline over time through 24 weeks post end of treatment

Numerical value of HBsAg and change from baseline will be summarized by cohort and by visit. The available data for each visit will be presented.

For Cohorts 1-3 change from baseline in HBsAg to all scheduled visits in log base 10 scale will be statistically analyzed using a Mixed Model for Repeated Measures model. The model will include log base 10 transformed baseline as a covariate, cohort, visit, cohort by visit as fixed effects, and within-participant errors will be modeled with an unstructured, heterogeneous autoregressive (1), or compound symmetry variance matrix in that order if a model does not converge. Estimates of least square mean (LSM) values and the LSM differences between each BRII-835 dose cohort and PEG-IFNα monotherapy cohort, and their respective 95% confidence interval and p-values will be calculated. The analyses will be done using mITT analysis set with hypothetical estimand. If the model is not converged only descriptive statistics will be presented.

In the first interim analysis, absolute serum HBsAg and change from baseline over time through Week 48 will be performed.

- Nadir serum HBsAg and maximum reduction of serum HBsAg from baseline

Nadir serum HBsAg and maximum reduction of serum HBsAg from baseline will be summarized by cohort based on mITT analysis set.

- Time to achieve first time of serum HBsAg loss through Week 72

Time to achieve first time of serum HBsAg loss (Days) defined as the duration from the first study drug administration to the time to achieve first time of serum HBsAg loss before or on Week 72.
Duration (Days)= event/censoring date – the first study drug administration date + 1.

Time to achieve first time of serum HBsAg loss will be analyzed by taking account of losses to follow-up using the stratified log-rank test with randomization strata as a stratification factor based on mITT analysis set, and tabular summaries of the Kaplan Meier curves providing the median and quartiles -will be provided for each cohort, and the KM curves will be presented graphically. In addition, for Cohorts 1-3 a Cox proportional hazard regression model where the estimate of the hazard ratio for treatment (, pairwise comparison between three cohorts) will be performed with randomization strata as fixed effect, its 95% CI, and p value will be provided if the model is converged.

Cohort 4 will be further analyzed by subgroups specified in section 3.5, Time to achieve first time of serum HBsAg loss will be analyzed by taking account of losses to follow-up using the log-rank test based on mITT analysis set, and tabular summaries of the Kaplan Meier curves providing the median, quartiles, and range will be provided for each subgroup, and the KM curves will be presented graphically. In addition, for cohort 4 a cox proportional hazard regression model where the estimate of the hazard ratio for subgroup (responder vs non-responder by Week 24 and Week 44 respectively) will be performed its 95% CI, and p value will be provided if the model is converged.

	Data of event/censoring	Censoring
Participants who lost to follow up or participants who withdrew from the trial	Time from randomization tolast lab collection date	Y
Participants who did't achieve the HBsAg loss	Time from randomization to last HBsAg sample collection date	Y
Participants who achieved the HBsAg loss	Time from randomization to date of achieving first time HBsAg loss	N

6.2.3. [redacted]

- [redacted]
- [redacted]

- [redacted]

[redacted]

HBsAb (anti-HBs) levels in participants achieved HBsAg loss at end of treatment and Week 24 post end of treatment will be summarized by Cohort.

[redacted]

.

7. SAFETY ANALYSES

7.1. Adverse Events and Deaths

7.1.1. Adverse Event Dictionary

Clinical and laboratory adverse events (AEs) will be coded using the current version of MedDRA. System organ class (SOC), high-level group term (HLGT), high-level term (HLT), preferred term (PT), and lower-level term (LLT) will be provided in the AE dataset.

7.1.2. Adverse Event Severity

Adverse events are graded by the investigator as Grade 1, 2, 3, 4, or 5 according to toxicity criteria specified in the protocol. The severity grade of events for which the investigator did not record severity will be categorized as "missing" for tabular summaries and data listings. The missing category will be listed last in summary presentation.

7.1.3. Relationship of Adverse Events to Study Drug

Related AEs are those for which the investigator selected "Related" on the AE CRF to the question of "Related to Study Treatment." Relatedness will always default to the investigator's choice, not that of the medical monitor. Events for which the investigator did not record relationship to study drug will be considered related to study drug for summary purposes. However, by-subject data listings will show the relationship as missing.

7.1.4. Serious Adverse Events

Serious adverse events (SAEs) will be identified and captured as SAEs if the AEs met the definitions of SAEs that were specified in the study protocol. SAEs captured and stored in the clinical database will be reconciled with the SAE database from the vender Drug Safety before data finalization.

7.1.5. Treatment-Emergent Adverse Events

7.1.5.1. Definition of Treatment-Emergent Adverse Events

Treatment-emergent adverse events (TEAEs) are defined as 1 or both of the following:

- Any AEs with an onset date on or after the study drug start date
- Any existing AEs worsen after treatment

7.1.5.2. Incomplete Dates

If the onset date of the AE is incomplete and the AE stop date is not prior to the first dosing date of study drug, then the month and year (or year alone if month is not recorded) of onset determine whether an AE is treatment emergent. The event is considered treatment emergent if the AE onset is the same as or after the month and year (or year) of the first dosing date of study drug.

An AE with completely missing onset and stop dates, or with the onset date missing and a stop date later than the first dosing date of study drug, will be considered to be treatment emergent.

In addition, an AE with the onset date missing and incomplete stop date with the same or later month and year (or year alone if month is not recorded) as the first dosing date of study drug will be considered treatment emergent.

7.1.6. Summaries of Adverse Events and Deaths

Treatment-emergent AEs will be summarized by Cohort, subtotal for Cohort 2-4 and overall based on Safety Analysis Set.

7.1.6.1. Summaries of AE Incidence by Severity

A brief, high-level summary of the number of percentage of subjects who experienced at least 1 TEAE in the categories described below will be provided by treatment. All deaths observed in the study will also be included in this summary.

The number and percentage of subjects who experienced at least 1 TEAE will be provided and summarized by SOC and PT by Cohort, subtotal for Cohort 2-4 and overall.

For the AE categories described below, summaries will be provided by SOC, PT and treatment:

- TEAEs by maximum severity
- TEAEs with Grade 3 or higher
- treatment-related TEAEs
- BRII-835 treatment-related AEs
- PEG-IFNα treatment-related AEs
- Both BRII-835 and PEG-IFNα treatment-related AEs
- treatment-related TEAEs of Grade 3 or higher
- BRII-835 treatment-related TEAEs of Grade 3 or higher
- BRII-835+PEG-IFNαtreatment-related TEAEs of Grade 3 or higher

- PEG-IFNαtreatment-related TEAEs of Grade 3 or higher
- treatment-related TEAEs by maximum severity
- TE SAEs
- treatment-related TESAEs
- BRII-835 treatment-related SAEs
- BRII-835+PEG-IFNα treatment-related SAEs
- Injection site reaction
- TEAEs leading to premature discontinuation of BRII-835
- TEAEs leading to premature discontinuation of BRII-835+PEG-IFNα
- TEAEs leading to premature discontinuation of PEG-IFNα
- TEAEs leading to premature discontinuation of study
- TE SAEs leading to death (i.e., outcome of death)

Multiple events will be counted only once per subject in each summary. Adverse events will be summarized and listed first in descending order of total frequency of SOC and then by PT in descending order of total frequency within each SOC. For summaries by severity, the most severe severity will be used for those AEs that occurred more than once in a given subject during the study.

In addition to the above summary tables, all TEAEs, TE SAEs, TE treatment-related AEs, and TE treatment-related SAEs will be summarized by PT only in descending order of total frequency.

In addition, data listings will be provided for the following:

- All AEs, indicating whether the event is treatment emergent
- All SAEs
- All Deaths
- All SAEs leading to death (i.e., outcome of death)
- All AEs leading to premature discontinuation of study drug

- All AEs leading to premature discontinuation of study

7.2. Laboratory Evaluations

Laboratory data collected during the study will be analyzed and summarized using both quantitative and qualitative methods. Summaries of laboratory data will be provided by Cohort, subtotal for Cohort 2-4 and overall based on Safety Analysis Set and will include data throughout the trial.

For subjects who have permanently discontinued study drug, or all available data at the time of the database snapshot for subjects who were ongoing at the time of an interim analysis. The analysis will be based on values in standard units. When values are below the LOQ, they will be listed as such, and the closest imputed value will be used for the purpose of calculating summary statistics. Hemolized test results will not be included in the analysis, but they will be listed in by-subject laboratory listings.

A by-subject listing for laboratory test results will be provided by subject ID number, age, sex, race, body weight at Screening and visit in chronological order for hematology, serum chemistry, and urinalysis separately. Values falling outside of the relevant reference range and/or having a severity of Grade 1 or higher based on NCI-CTCAE Version 5.0 grading scale will be flagged in the data listings, as appropriate.

No formal statistical testing is planned.

7.2.1. Summaries of Numeric Laboratory Results

Descriptive statistics will be provided by treatment for each laboratory test specified in the study protocol as follows:

- Baseline values
- Values at each post-baseline visit
- Change from baseline at each post-baseline visit

A baseline laboratory value will be defined as the last measurement obtained on or prior to the date/time of first dose of study drug. Change from baseline to a post-baseline visit will be defined as the visit value minus the baseline value.

7.2.2. Graded Laboratory Values

The NCI-CTCAE Version 5.0 will be used to assign toxicity grades (1 to 5) to laboratory results for analysis. For laboratory tests with criteria for both increased and decreased levels, analyses for each direction (i.e., increased, decreased) will be presented separately.

7.2.2.1. Treatment-Emergent Laboratory Abnormalities

Treatment-emergent laboratory abnormalities are defined as values that increase at least 1 toxicity grade from baseline at any post-baseline visit. If the relevant baseline laboratory value is missing, any abnormality of at least Grade 1 observed within the time frame specified above will be considered treatment-emergent laboratory abnormalities.

7.2.2.2. Summaries of Laboratory Abnormalities

Laboratory data that are categorical will be summarized using the number and percentage of subjects in the study with the given response at baseline and each scheduled post-baseline visit.

The following summaries (number and percentage of subjects) for treatment-emergent laboratory abnormalities will be provided by lab test and treatment; subjects will be categorized according to the most severe post-baseline abnormality grade for a given lab test:

- Graded laboratory abnormalities

For all summaries of laboratory abnormalities, the denominator is the number of subjects with non-missing post-baseline values.

7.2.3. Liver-related Laboratory Evaluations

Liver-related abnormalities after initial study drug dosing will be examined and summarized using the number and percentage of subjects who were reported to have the following laboratory test values for post-baseline measurements:

- Aspartate aminotransferase (AST): (a) > 3 times of the upper limit of reference range (ULN); (b) > 5 x ULN; (c) > 10 x ULN; (d) > 20 x ULN
- Alanine aminotransferase (ALT): (a) > 3 x ULN; (b) > 5 x ULN; (c) > 10 x ULN; (d) > 20 x ULN
- AST or ALT: (a) > 3 x ULN; (b) > 5 x ULN; (c) > 10 x ULN; (d) > 20 x ULN
- Total bilirubin: > 2 x ULN
- Alkaline phosphatase (ALP) > 1.5 x ULN
- AST or ALT > 3 x ULN and total bilirubin: (a) > 1.5 x ULN; (b) > 2 x ULN

The summary will include data from all post-baseline visits. For individual laboratory tests, subjects will be counted once based on the most severe post-baseline values. For both the composite endpoint of AST or ALT and total bilirubin, subjects will be counted once when the criteria are met at the same post-baseline visit date. The denominator is the number of subjects in the Safety Analysis Set who have non-missing post-baseline values of all relevant tests at the

same post-baseline visit date. A listing of subjects who met at least 1 of the above criteria will be provided.

7.3. Vital Signs

Descriptive statistics will be provided by Cohort for vital signs as follows:

- Baseline value
- Values at each post-baseline visit
- Change from baseline at each post-baseline visit

A baseline value will be defined as the last available value collected on or prior to the date/time of first dose of study drug. Change from baseline to a post-baseline visit will be defined as the post-baseline value minus the baseline value. Vital signs measured at unscheduled visits will be included for the baseline value selection.

No formal statistical testing is planned.

A by-subject listing of vital signs will be provided by subject ID number, sex, age, race, screening body weight and visit in chronological order.

7.4. Prior and Concomitant Medications

Medications collected at screening and during the study will be coded using the current version of the World Health Organization (WHO) Drug dictionary.

7.4.1. Prior Medications

Prior HBV medications are defined as any medications taken before a subject took the first study drug.

Prior medications will be summarized by Anatomical Therapeutic Chemical (ATC) drug class Level 3 and preferred name using the number and percentage of subjects for each treatment and overall. A subject reporting the same medication more than once will be counted only once within each ATC drug class when calculating the number and percentage of subjects who received that medication. Medications may appear under multiple ATC drug classes. The summary will be ordered alphabetically by ATC medical class and then by preferred term in order of descending overall frequency within each ATC medical class. For drugs with the same frequency, sorting will be done alphabetically.

For the purposes of analysis, any medication with a start date prior to the first dosing date of study drug will be included in the prior medication summary regardless of when the stop date is. If a partial start date is entered the medication will be considered prior unless the month and year (if day is missing) or year (if day and month are missing) of the start date are after the first dosing

date. Medications with a completely missing start date will be included in the prior medication summary, unless otherwise specified.

Summaries will be based on the Safety Analysis Set. No formal statistical testing is planned.

7.4.2. Concomitant Medications

Concomitant medications are defined as medications taken while a subject took study drug. Use of concomitant medications will be summarized by Anatomical Therapeutic Chemical (ATC) drug class Level 3 and preferred name using the number and percentage of subjects for each treatment. A subject reporting the same medication more than once within each ATC drug class will be counted only once when calculating the number and percentage of subjects who received that medication. Medications may appear under multiple ATC drug classes. The summary will be ordered alphabetically by ATC medical class and then by preferred term in descending overall frequency within each ATC medical class. For drugs with the same frequency, sorting will be done alphabetically.

For the purposes of analysis, any medications with a start date prior to or on the first dosing date of study drug and continued to be taken after the first dosing date, or started after the first dosing date but prior to or on the last dosing date of study drug will be considered concomitant medications. Medications started and stopped on the same day as the first dosing date or the last dosing date of study drug will also be considered concomitant. Medications with a stop date prior to the date of first dosing date of study drug or a start date after the last dosing date of study drug will be excluded from the concomitant medication summary. If a partial stop date is entered, any medication with the month and year (if day is missing) or year (if day and month are missing) prior to the date of first study drug administration will be excluded from the concomitant medication summary. If a partial start date is entered, any medication with the month and year (if day is missing) or year (if day and month are missing) after the study drug stop date will be excluded from the concomitant medication summary. Medications with completely missing start and stop dates will be included in the concomitant medication summary, unless otherwise specified. Summaries will be based on the Safety Analysis Set. No formal statistical testing is planned.

All prior and concomitant medications (other than per-protocol study drugs) will be provided in a by-subject listing sorted by subject ID number, age, sex, race, screening body weight and administration date in chronological order.

7.5. Electrocardiogram Results

A by-subject listing for ECG assessment results will be provided by subject ID number, sex, age, race, screening body weight.

7.6. Other Safety Measures

No additional safety measures are specified in the protocol.

8. ADA

Desriptive statistics based on ADA Analysis Set will be performed for ADA.

9. PHARMACOKINETIC EVALUATION/ANALYSIS

Individual subject concentration data for BRII-835 and its metabolite A-312327 will be listed and summarized using descriptive statistics based on PK Analysis Set. Summary statistics (numbers of subjects, number of subjects with concentration BLQ, arithmetic mean, SD, coefficient of variation [CV], median, minimum, maximum, geometric mean and Geometric CV) will be presented by time point and Cohort. For summary statistics, BLQ values will be treated as zero at predose (including nominal timepoint of D1,D225, D337) and missing for postdose (including nominal timepoint of D1,D225, D337).

No PK parameters will be reported in this study since only limited (sparse) PK samples were collected. The concentration data from this study will be pooled with data from other studies for population PK analysis, which will be reported separately.

10. CHANGES FROM PROTOCOL-SPECIFIED ANALYSES

10.1. Changes From Protocol-Specified Efficacy Analyses

There are no deviations from the protocol-specified efficacy analyses. SAP combined the primary estimand 1 and estimand 2 of protocol into one primary estimand, intercurrent events are redefined for this combined primary estimand as in SAP section 6.1.2. For the secondary estimand(s), SAP emphasized that the use of rescue medication will be treated as "treatment failure" (composite policy) for HBV DNA suppression related variables

10.2. Changes From Protocol-Specified Safety Analyses

There are no deviations from the protocol-specified safety analyses.

10.3. Changes From Protocol-Interim Analyses

The first interim analyses originally would be conducted when approximately 45 enrolled participants of Cohorts 1-3 complete the 48-week treatment in protocol, actually conducted when 55 participants of Cohorts 1-3 finished the 48-week treatment.

Considering that cohort 4 may also have been completed 24 weeks follow-up post EOT when the previous planned third interim analysis is conducting, thereby, the revised third interim analysis will contain cohort 4 and be conducted when last available participant of Cohorts 1-4 completes follow-up 24 weeks post end of treatment.

10.4. Changes From Protocol-Sample Size

Up to 75 participants originally will be enrolled for Cohorts 1-3 and 45 participants enrolled for Cohort 4 in protocol. Actually, 55 participants enrolled in Cohorts 1-3 and 31 participants in Cohort 4. The second, subsequent interim analysis and final analysis will be conducted based on the above actually enrolled participants.

11. SOFTWARE

SAS® Software Version 9.4. SAS Institute Inc., Cary, NC, USA.

12. SAP REVISION

Revision Version/ Date (DD MMM YYYY)	Section	Summary of Revision	Reason for Revision
v2.0/20Feb2025	2.1.Interim Analyses	• Cohorts 4 will be conducted in the third interim analysis;<br>• Describes the interim analysis content with the corresponding timepoints.	Cohorts 4 data follow-up 24 weeks post end of treatment will be available when the third interim analysis.
	3.5.Subgroup analysis	• Change the Subgroup definition for Cohort 4: responder vs non-responder by Week 44 in BRII-179-835-001 study;<br>• HBsAg change over time will be summarized by cohort 1- 4 based on baseline HBsAg level categories, excluded HBsAg <=1000 IU/mL, that was done in the first interim analysis, not required in subsequent analyses, depending on the exploratory needs.	Additional subgroup exploratory analysis based on medical considerations.
	6.1.Primary Efficacy Endpoint	• Bayesian hierarchy logistics regression will be used for cohort 1-3, For cohort 4 a unstratified Bayesian estimation will be conducted.	Hierarchy Bayesian only applied to cohort 1-3 due to stratified randomization, and cohort 4 will be used unstratified Bayesian model.
	6.2.Secondary Efficacy Endpoints	• Time to achieve first time of serum HBsAg loss through Week 72: Cohort 4	Updated subgroup definition based on medical considerations.

		will stratified by updated subgroup definition in section 3.5	
	10. Changes From Protocol-Analyses	• Add the changes from the protocol-specified analyses.	Descrbe the key changes from the protocol-specified analyses.
v3.0/MMJul2025	2.1.Interim Analyses	• The third interim analysis will be also include the efficacy endpoints of the corresponding time points except for PK and ADA.	PK and ADA analysis will be performed at the final interim analysis.
	3.GENERAL CONSIDERATIONS FOR DATA ANALYSES	• All data will be presented to a maximum of 2 decimal places.	Two decimal places can meet the analysis requirements.
	3.1.Key Definitions	• Sustained HBsAg loss: quantitative HBsAg < LLOQ at all visits (scheduled and unscheduled) for ≥ 24 weeks (≥ 161 days, i.e. 168 days (24 weeks) minus 7 days (Visit window)).	Considering the deduction of a 7-day window period, the sustained duration of ≥24 weeks is defined as being ≥161 days (24 weeks -7 days).
	3.2 Analysis Sets	• Added modified efficacy analysis set for cohort 4	As supplementary analysis of cohort 4 to exclude patients having baseline HBsAg<=100 IU/mL
	3.5 Sensitivity and Subgroup Analysis	• Change this section from subgroup analysis to sensitivity and subgroup analysis<br>• Remove subgroup analysis by week 24 response in study BRII-179-835-001	The section also covered the sensitivity analysis.<br>Subgroup analysis by week 24 response in study BRII-179-835-001 is not needed based on medical judgement after IA 2 analysis.
	3.9.Analysis Visit Windows	• Add the title of table 3.2	The participants of prematurely discontinue treatment refers to this table.

	6. EFFICACY ANALYSES	• Per Protocol Set (PP) will serve as supplementary analysis only for the primary efficacy endpoints.<br>• Add HBaAb in exploratory analysis.	Unless otherwise specified, PP analysis is not required for secondary efficacy endpoints.<br>Detect HBaAb level at the end of treatment and week 24 post end of treatment
	9. PHARMACOKINETIC EVALUATION/ANALYSIS	• Individual subject concentration data for BRII-835<br>• For summary statistics, BLQ values will be treated as zero at predose (including nominal timepoint of D1,D225, D337) and missing for postdose (including nominal timepoint of D1,D225, D337).	The substance of BRII-835 is ALN-HBV02 AS.<br>Specify the data processing method of BLQ.

APPENDIX

Updates to Section 12 SAP Revision

To enhance readability, SAP revision history below was updated post SAP v3.0 signoff for content clarification, wording improvement, and correction of typos and grammatic errors. There were no changes to any established analyses in SAP v3.0.

Revision Version/ Date (DD MMM YYYY)	Section	Summary of Revision	Reason for Revision
v2.0/20Feb2025	2.1 Interim Analyses	• Cohorts 4 will be added in the third interim analysis;<br>• Describes the interim analysis content with the corresponding timepoints.	Cohorts 4 follow-up data at 24 weeks post end of treatment will be available when the third interim analysis.
	3.5 Subgroup analysis	• Provided clear definition for Cohort 4 subgroup analysis (responder vs non-responder by anti-HBs response by Week 24 and Week 44 in BRII-179-835-001);<br>• HBsAg change over time will be summarized by cohort 1- 4 based on baseline HBsAg level categories, with a cutoff by HBsAg 1000 IU/mL excluded from the analysis.	Updated subgroup exploratory analysis based on medical considerations.
	6.1 Primary Efficacy Endpoint	• Bayesian hierarchy logistics regression will be used for cohort 1-3, For cohort 4 a unstratified Bayesian estimation will be conducted.	Bayesian hierarchy model is only applied to cohort 1-3 due to stratified randomization, and unstratified Bayesian model will be used for cohort 4.

Revision Version/ Date (DD MMM YYYY)	Section	Summary of Revision	Reason for Revision
	6.2 Secondary Efficacy Endpoints	• Time to achieve first time of serum HBsAg loss through Week 72: Cohort 4 analysis will be stratified by updated subgroup definition in section 3.5	Updated subgroup definition based on medical considerations.
	10. Changes From Protocol-Analyses	• Add the changes from the protocol-specified analyses.	Describe the key changes from the protocol-specified analyses.
v3.0/31Jul2025	2.1 Interim Analyses	• The third interim analysis will include the efficacy endpoints of the corresponding time points except for PK and ADA.	PK and ADA analysis will be performed at the final analysis.
	3.GENERAL CONSIDERATIONS FOR DATA ANALYSES	• All data will be presented to a maximum of 2 decimal places.	Two decimal places can meet the analysis requirements.
	3.1 Key Definitions	• Sustained HBsAg loss: quantitative HBsAg < LLOQ at all visits (scheduled and unscheduled) for ≥ 24 weeks (≥ 161 days, i.e. 168 days (24 weeks) minus 7 days (Visit window)).	Considering the deduction of a 7-day window period, the sustained duration of ≥24 weeks is defined as being ≥161 days (24 weeks -7 days).
	3.2 Analysis Sets	• Added modified efficacy analysis set for cohort 4	As supplementary analysis of cohort 4 to exclude patients having baseline HBsAg<=100 IU/mL
	3.5 Sensitivity and Subgroup Analysis	• Change this section from subgroup analysis to sensitivity and subgroup analysis	The section also covered the sensitivity analysis.<br>Subgroup analysis by week 24 response in

Revision Version/ Date (DD MMM YYYY)	Section	Summary of Revision	Reason for Revision
		• Remove subgroup analysis by week 24 response in study BRII-179-835-001	study BRII-179-835-001 is not needed based on medical judgement after IA 2 analysis.
	3.9. Analysis Visit Windows	• Add the title of table 3.2	Refer to this table for analysis visit windows of the participants of prematurely discontinue treatment.
	6. EFFICACY ANALYSES	• Per Protocol Set (PP) will serve as supplementary analysis only for the primary efficacy endpoints.<br>• Add HBsAb Summary analysis in exploratory analysis section.	Unless otherwise specified, PP analysis is not required for secondary efficacy endpoints.<br>Describe HBsAb level at the end of treatment and 24 weeks post end of treatment
	9. PHARMACOKINETIC EVALUATION/ANALYSIS	• For summary statistics, BLQ values will be treated as zero at predose (including nominal timepoint of D1,D225, D337) and missing for postdose (including nominal timepoint of D1,D225, D337).	Specify the data processing method of BLQ.
Revision Version/ Date (DD MMM YYYY)	Section	Summary of Revision	Reason for Revision
v2.0/20Feb2025	2.1 Interim Analyses	• Cohorts 4 will be added in the third interim analysis;<br>• Describes the interim analysis content with the corresponding timepoints.	Cohorts 4 follow-up data at 24 weeks post end of treatment will be available when the third interim analysis.
	3.5 Subgroup analysis	• Provided clear definition for Cohort 4 subgroup	Updated subgroup exploratory analysis based on medical

Revision Version/ Date (DD MMM YYYY)	Section	Summary of Revision	Reason for Revision
		analysis (responder vs non-responder by anti-HBs response by Week 24 and Week 44 in BRII-179-835-001);<br>• HBsAg change over time will be summarized by cohort 1- 4 based on baseline HBsAg level categories, with a cutoff by HBsAg 1000 IU/mL excluded from the analysis.	considerations.
	6.1 Primary Efficacy Endpoint	• Bayesian hierarchy logistics regression will be used for cohort 1-3, For cohort 4 a unstratified Bayesian estimation will be conducted.	Bayesian hierarchy model is only applied to cohort 1-3 due to stratified randomization, and unstratified Bayesian model will be used for cohort 4.
	6.2 Secondary Efficacy Endpoints	• Time to achieve first time of serum HBsAg loss through Week 72: Cohort 4 analysis will be stratified by updated subgroup definition in section 3.5	Updated subgroup definition based on medical considerations.
	10. Changes From Protocol-Analyses	• Add the changes from the protocol-specified analyses.	Describe the key changes from the protocol-specified analyses.
v3.0/31Jul2025	2.1 Interim Analyses	• The third interim analysis will include the efficacy endpoints of the corresponding time points except	PK and ADA analysis will be performed at the final analysis.

Revision Version/ Date (DD MMM YYYY)	Section	Summary of Revision	Reason for Revision
		for PK and ADA.	
	3.GENERAL CONSIDERATIONS FOR DATA ANALYSES	• All data will be presented to a maximum of 2 decimal places.	Two decimal places can meet the analysis requirements.
	3.1 Key Definitions	• Sustained HBsAg loss: quantitative HBsAg < LLOQ at all visits (scheduled and unscheduled) for ≥ 24 weeks (≥ 161 days, i.e. 168 days (24 weeks) minus 7 days (Visit window)).	Considering the deduction of a 7-day window period, the sustained duration of ≥24 weeks is defined as being ≥161 days (24 weeks -7 days).
	3.2 Analysis Sets	• Added modified efficacy analysis set for cohort 4	As supplementary analysis of cohort 4 to exclude patients having baseline HBsAg<=100 IU/mL
	3.5 Sensitivity and Subgroup Analysis	• Change this section from subgroup analysis to sensitivity and subgroup analysis<br>• Remove subgroup analysis by week 24 response in study BRII-179-835-001	The section also covered the sensitivity analysis.<br>Subgroup analysis by week 24 response in study BRII-179-835-001 is not needed based on medical judgement after IA 2 analysis.
	3.9. Analysis Visit Windows	• Add the title of table 3.2	Refer to this table for analysis visit windows of the participants of prematurely discontinue treatment.
	6. EFFICACY ANALYSES	• Per Protocol Set (PP) will serve as supplementary analysis only for the primary efficacy endpoints.<br>• Add HBsAb Summary analysis in exploratory	Unless otherwise specified, PP analysis is not required for secondary efficacy endpoints.<br>Describe HBsAb level at the end of treatment and 24 weeks post end of

Revision Version/ Date (DD MMM YYYY)	Section	Summary of Revision	Reason for Revision
		analysis section.	treatment
	9. PHARMACOKINETIC EVALUATION/ANALYSIS	• For summary statistics, BLQ values will be treated as zero at predose (including nominal timepoint of D1,D225, D337) and missing for postdose (including nominal timepoint of D1,D225, D337).	Specify the data processing method of BLQ.

Brii Biosciences
Breakthrough innovation & insight

STATISTICAL ANALYSIS PLAN

**Study Title:**	A Phase 2 Multicenter, Randomized, Open-label Study to Investigate the Efficacy and Safety of BRII-835 (VIR-2218) and Pegylated Interferon Alpha (PEG-IFNα) Combination Therapy for the Treatment of Chronic Hepatitis B Virus (HBV) Infection
**Name of Test Drug:**	BRII-835 (VIR-2218); Pegylated Interferon Alpha (PEG-IFNα)
**Study Number:**	BRII-835-002
**Protocol Version (Date):**	2.0 (31 Aug 2023)
**Analysis Type:**	Phase 2
**Analysis Plan Version:**	4.0
**Analysis Plan Date:**	28 Jan 2026
**Analysis Plan Author(s):**	[redacted]

CONFIDENTIAL AND PROPRIETARY INFORMATION

SIGNATURE PAGE

Reason for Signature	Name	Signature	Date (DDMMMYYYY)
Author	[redacted]		
Therapeutic Area Head (or delegate)			
Chief Medical Officer (or delegate)			

TABLE OF CONTENTS

STATISTICAL ANALYSIS PLAN 1
TABLE OF CONTENTS 3
LIST OF ABBREVIATIONS 5
1. INTRODUCTION 7
1.1. Study Objectives 7
1.2. Study Design 7
1.2.1. Planned Cohorts and Dosing 8
1.2.2. NRTI Discontinuation 9
1.3. Sample Size and Power 10
2. TYPE OF PLANNED ANALYSIS 13
2.1. Interim Analyses 13
2.1.1. Dose Escalation Analysis 13
2.2. Final Analysis 13
3. GENERAL CONSIDERATIONS FOR DATA ANALYSES 14
3.1. Key Definitions 15
3.2. Analysis Sets 16
3.2.1. Screened Analysis Set 17
3.2.2. Full Analysis Set (FAS) 17
3.2.3. Safety Analysis Set (SAS) 17
3.2.4. Modified Intent-To-Treat Analysis Set (mITT) 17
3.2.5. Modified Efficacy Analysis Set 17
3.2.6. Per Protocol Set (PP) 17
3.2.7. Pharmacokinetic Analysis Set (PAS) 18
3.2.8. ADA Analysis Set 18
3.2.9. PBMC Analysis Set 18
3.3. Subject Grouping 18
3.4. Strata and Covariates 18
3.5. Sensitivity and Subgroup Analysis 18
3.6. Multiple Comparisons 19
3.7. Missing Data and Outliers 19
3.7.1. Missing Data 19
3.7.2. Outliers 19
3.8. Data Handling Conventions and Transformations 19
3.9. Analysis Visit Windows 21
3.9.1. Selection of Data in the Event of Multiple Records in an Analysis Visit Window 25
4. SUBJECT DISPOSITION 27
4.1. Subject Enrollment and Disposition 27
4.1.1. Duration of Exposure to Study Drug 28
4.1.2. Adherence to Study Drug 28
4.2. Protocol Deviations 29
5. BASELINE CHARACTERISTICS 30

5.1. Demographics and Baseline Characteristics 30
5.2. Other Baseline Characteristics 30
5.3. Medical History 30
6. EFFICACY ANALYSES 31
6.1. Primary Efficacy Endpoint 31
6.1.1. Definition of the Primary Efficacy Endpoint: 31
6.1.2. Estimand 31
6.1.3. Analysis methods for the primary endpoints 32
6.2. Secondary Efficacy Endpoints 34
6.2.1. Estimand for the secondary endpoints: 34
6.2.2. Analysis Methods for Secondary Efficacy Endpoint 35
6.2.3. Exploratory Endpoints: 36
7. SAFETY ANALYSES 38
7.1. Adverse Events and Deaths 38
7.1.1. Adverse Event Dictionary 38
7.1.2. Adverse Event Severity 38
7.1.3. Relationship of Adverse Events to Study Drug 38
7.1.4. Serious Adverse Events 38
7.1.5. Treatment-Emergent Adverse Events 38
7.1.6. Summaries of Adverse Events and Deaths 39
7.2. Laboratory Evaluations 41
7.2.1. Summaries of Numeric Laboratory Results 41
7.2.2. Graded Laboratory Values 41
7.2.3. Liver-related Laboratory Evaluations 42
7.3. Vital Signs 43
7.4. Prior and Concomitant Medications 43
7.4.1. Prior Medications 43
7.4.2. Concomitant Medications 44
7.5. Electrocardiogram Results 44
7.6. Other Safety Measures 44
8. ADA 45
9. PHARMACOKINETIC EVALUATION/ANALYSIS 46
10. CHANGES FROM PROTOCOL-SPECIFIED ANALYSES 47
10.1. Changes From Protocol-Specified Efficacy Analyses 47
10.2. Changes From Protocol-Specified Safety Analyses 47
10.3. Changes From Protocol-Interim Analyses 47
10.4. Changes From Protocol-Sample Size 47
11. SOFTWARE 48
12. SAP REVISION 49

LIST OF ABBREVIATIONS

ADA	anti-drug antibody
AE	adverse event
ALT	alanine aminotransferase
AST	aspartate aminotransferase
BLQ	below the limit of quantitation
BMI	body mass index
CFR	Code of Federal Regulations
CI	confidence interval
CK	creatine kinase
CRF	case report form
CSR	clinical study report
CTCAE	Common Toxicity Criteria for Adverse Events
ECG	Electrocardiogram
FAS	Full Analysis Set
Hb	Hemoglobin
HBV	hepatitis B virus
HLT	high-level term
ICH	International Conference on Harmonization (of Technical Requirements for Registration of Pharmaceuticals for Human Use)
ITT	intent to treat
LLT	lower-level term
LOQ	limit of quantitation
MedDRA	Medical Dictionary for Regulatory Activities
mITT	modified intent to treat
NrtI	nucleos(t)ide reverse transcriptase inhibitors
PBMC	peripheral blood mononuclear cell
PK	pharmacokinetic
PP	per protocol
PT	preferred term
Q1, Q3	first quartile, third quartile
SAP	statistical analysis plan
SC	subcutaneously
SD	standard deviation
SI (units)	international system of units
SOC	system organ class
SRC	safety review committee
TEAE	treatment-emergent adverse event
TFLs	tables, figures, and listings

ULN	upper limit of normal
WHO	World Health Organization

1. INTRODUCTION

This statistical analysis plan (SAP) describes the statistical analysis methods and data presentations to be used in tables, figures, and listings (TFLs) in the clinical study report (CSR) for Study BRII-835-002. This SAP is based on the study protocol version 2.0 dated 31 Aug 2023 and the electronic case report form (eCRF). The SAP will be finalized before database finalization. Any changes made after the finalization of the SAP will be documented in the CSR.

This statistical analysis plan (SAP) describes the statistical analysis methods and data presentations to be used in tables, figures, and listings (TFLs) of three interim analyses and final analysis for Study BRII-835-002. The first two interim analyses will be conducted when approximately 45 enrolled participants of Cohorts 1-3 complete the 48-week treatment and last available participant of all enrolled participants (Cohorts 1-4) completes the 48-week treatment, respectively. If the pace of enrollment is robust, and the primary endpoint proportion of participants with HBsAg loss at Week 48 is expected to occur less or equal to two months between the two interim analyses, the two analyses may be collapsed into one analysis. The final analysis will be performed when last available participant of Cohorts 1-4 completes the last scheduled visit according to the SoA.

1.1. Study Objectives

The primary objective of this study is as follows:

- To evaluate the efficacy and safety of BRII-835 and PEG-IFNα combination therapy in participants with chronic HBV infection

The secondary objectives of this study are as follows:

- To assess the antiviral activity of BRII-835 and PEG-IFNα combination therapy
- To assess BRII-835 pharmacokinetic (PK) exposure and evaluate the anti-drug antibodies (ADA) to BRII-835

The exploratory objectives of this study are as follows

- To evaluate potential biomarkers for host immune responses to therapy
- To evaluate other viral markers of HBV infection

1.2. Study Design

This is a Phase 2 open-label, randomized study of BRII-835 and PEG-IFNα combination therapy in adult participants with chronic HBV infection without cirrhosis receiving NRTI therapy as background therapy. This study is designed to evaluate the efficacy and safety of BRII-835 and

PEG-IFNα combination therapy. Two different dose levels of BRII-835 in combination with PEG-IFNα will be evaluated.

Participants with HBeAg negative or HBeAg positive chronic HBV infection are eligible to participate in Cohorts 1-3 of this study. Eligible participants will be stratified by their serum HBsAg level at screening and randomized 1:1:1 to each of the three cohorts. Up to 75 participants are planned for Cohorts 1-3.

In addition, this study will evaluate the efficacy of BRII-835 and PEG-IFNα combination in participants previously treated with BRII-179, a therapeutic recombinant HBV vaccine. Eligible participants who completed BRII-179-835-001 study will roll over to Cohort 4. Up to 45 participants will be enrolled.

All participants will remain outpatient during study participation and will be followed for the safety, tolerability, and antiviral activity after completion of study intervention. The assessments and procedures performed at each visit are further described in the SoA (Section 1.3) of the protocol.

Participants will be assessed by the investigator for their eligibility to discontinue NRTI therapy at Week 72. Participants who meet the NRTI discontinuation criteria may enter the NRTI discontinuation monitoring period for an additional 24 weeks. Participants who do not meet the NRTI discontinuation criteria will continue NRTI therapy and will complete the study.

The total duration of study participation for each participant will include screening period (up to 4 weeks), dosing period (48 weeks), follow-up period (24 weeks), and NRTI discontinuation monitoring period (24 weeks when applicable). The study design schema can be found in Section 1.2 in the protocol.

1.2.1. Planned Cohorts and Dosing

Cohorts 1-3 (BRII-179 naïve):

Eligible participants of Cohorts 1-3 will be stratified by their serum HBsAg level at screening (>100 IU/mL and ≤ 500 IU/mL, > 500 and ≤ 1,500 IU/mL, or > 1,500 and ≤ 3,000 IU/mL) and randomized to receive the study intervention per the following dose regimens.

- Cohort 1: Each participant will receive 48 doses of PEG-IFNα 180 mcg administered SC every week starting on Day 1
- Cohort 2: Each participant will receive 48 doses of PEG-IFNα 180 mcg administered SC every week and 13 doses of BRII-835 200 mg administered SC every 4 weeks, both starting on Day 1
- Cohort 3: Each participant will receive 48 doses of PEG-IFNα 180 mcg administered SC every week and 13 doses of BRII-835 100 mg administered SC every 4 weeks, both starting on Day 1

Cohort 4 (BRII-179 treated):

Eligible participants who completed BRII-179-835-001 study will be assigned to Cohort 4 and receive the following study intervention.

Cohort 4: Each participant will receive 48 doses of PEG-IFNα 180 mcg administered SC every week and 13 doses of BRII-835 100 mg administered SC every 4 weeks, both starting on Day 1

Safety Review Committee: A Safety Review Committee (SRC) for this study will monitor participant safety and consider study-specific data as well as relevant background information about the study drugs and target population under study to inform on study conduct. The primary purpose of the SRC is to protect the safety of participants in this study.

Ad hoc SRC meetings will take place as needed, i.e., for a significant safety event such as criteria for study intervention discontinuation being reached. Decisions to suspend dosing or discontinue individual participants from study intervention will be made according to the predetermined criteria (Section 7 in the protocol). Participants may resume study intervention only following SRC approval. Additionally, the SRC may recommend discontinuation of or changes to the study to the Sponsor. The SRC charter outlining the member composition and operating procedures will be developed before the study initiation.

1.2.2. NRTI Discontinuation

NRTI Discontinuation Criteria

Upon participants completing Week 72 visits, the investigator will evaluate whether participants are eligible for discontinuation of NRTI therapy based on the following NRTI discontinuation criteria:

- HBsAg < LLOQ AND
- HBV DNA < LLOQ AND
- Undetectable HBeAg AND
- ALT ≤ 2x ULN

Participants who meet all of the above criteria for at least two consecutive visits at Week 72 are eligible to discontinue from NRTI therapy. Investigator should confirm participants' eligibility as early as possible within 2 weeks post Week 72 visit. Participants who discontinue NRTI will enter the NRTI Discontinuation Monitoring Period of the study within 1 week of the investigator confirming eligibility. Participants will not take their NRTI medications on the first day of the NRTI Discontinuation Monitoring Period and will be followed for an additional 24 weeks per the SoA (Section 1.3) in the protocol.

NRTI Re-treatment

It is recommended that the Investigator should consider re-treatment with NRTI therapy during the NRTI Discontinuation Monitoring Period if the participant meets any of the following criteria:

- ALT > 10x ULN with HBV DNA ＞LLOQ at the same visit
- ALT 5-10x ULN with HBV DNA ＞LLOQ persisting for ≥ 4 consecutive weeks

- ALT > ULN and HBV DNA > 2,000 IU/mL persisting for ≥ 4 consecutive weeks
- ALT > ULN with total bilirubin > 2x ULN and/or prolongation of prothrombin time (PT) > 2 seconds, and HBV DNA > LLOQ at the same visit
- HBV DNA > 100,000 IU/mL at any visit
- Confirmed HBeAg seroreversion (i.e., HBeAg positive after being HBeAg negative at NRTI discontinuation)
- Any other clinically significant event(s) warranting initiation of NRTI therapy in the opinion of the Investigator

Whether participants should be re-treated with NRTI is at the discretion of the Investigator following good medical judgement. Participants who are re-treated with NRTI therapy should continue to be followed per the SoA (Section 1.3.2) in the protocol for the duration of the 24-week NRTI Discontinuation Monitoring Period.

Participants with HBV DNA increase ≥ 2 log_{10} IU/mL within a 2-week period should:

- Return to the site for an unscheduled visit to measure HBV DNA levels and liver function tests as soon as possible after the investigator being aware of the HBV DNA increase
- Continue returning to site weekly until HBV DNA levels stabilize (do not increase ≥ 1 log_{10} IU/mL over a 4-week period or start decreasing for at least 2 consecutive visits) after which they will continue follow-up per the SOA for the NRTI Discontinuation Monitoring Period
- If any unscheduled visits needed as outlined above coincide with an existing visit per the SoA in the protocol, the scheduled assessments should be conducted

1.3. Sample Size and Power

Up to 75 participants will be randomly assigned in 1:1:1 ratio to each of Cohorts 1-3. A Bayesian framework will be used to calculate the sample size. It is assumed that the number of responders follows a Binomial distribution, with a weakly informative prior (Beta (0.5, 0.5)) for the true response rate. The precision for a range of response rates with 95% credible intervals are shown in Table 1:

Up to 45 participants will be enrolled in Cohort 4. No formal sample size is calculated due to the exploratory nature of Cohort 4.

Table 1 95% Credible Interval of Response Rate by Sample Size for Cohorts 1-3

Sample size per cohort	Number of responders	Response rate (%)	95% Credible interval* (%)
15	4	27	8-50
	5	33	13-57

	6	40	18-63
	7	47	25-71
	8	53	29-76
	9	60	36-82
25	6	24	10-41
	7	28	13-46
	8	32	16-51
	9	36	19-55
	10	40	22-58
	11	44	26-63
	12	48	30-67
	13	52	32-70

* 95% highest posterior density interval.

For the 15 participants in each cohort the lower bounds of 95% credible interval exclude the response rate of 10% if observed response rate is greater than 27% and exclude the response rate of 20% if observed response rate is greater than 40% and exclude the response rate of 30% if observed response rate is greater than 53%.

For the 25 participants in each cohort the lower bounds of 95% credible interval exclude the response rate of 10% if observed response rate is greater than 24% and exclude the response rate of 20% if observed response rate is greater than 36% and exclude the response rate of 30% if observed response rate is greater than 48%.The operational characteristics are based on posterior confidence that the true rate exceeds a threshold of interest, as shown in Table . For instance, when 4 out of 15 participants or 6 out of 25 participants are responders, the probability (true response rate>15%) is larger than 85%.

Table 2 Operational Characteristics by Sample Size

**Sample size per cohort**	**Number of responders**	**Probability (True response rate)>15%**	**Probability (True response rate)>20%**	**Probability (True response rate)>25%**	**Probability (True response rate)>30%**	**Probability (True response rate)>35%**
15	1	18.4%	8.5%	3.7%	1.4%	0.5%
	2	45.7%	27.2%	14.4%	7%	3.2%
	3	72.8%	52.6%	34.1%	20.1%	10.9%

	4	89.7%	75.6%	57.9%	40.6%	25.7%
	5	96.8%	90.4%	78.3%	62.7%	45.7%
	6	99.2%	96.6%	90.7%	80.4%	66.6%
	7	99.9%	99.2%	96.9%	92%	83.4%
25	2	16.2%	5.4%	1.6%	0.4%	0.1%
	3	35.7%	16.1%	6%	1.9%	0.5%
	4	58.2%	32.1%	14.9%	5.8%	1.9%
	5	77.4%	52.1%	29.4%	13.8%	5.5%
	6	89.1%	70.5%	46.9%	26.3%	12.1%
	7	95.9%	84.8%	65.5%	42.7%	23.6%
	8	98.5%	92.5%	79.4%	59.8%	38.3%
	9	99.6%	97.4%	89.6%	75%	55.2%
	10	99.9%	99%%	95.2%	86.1%	70.3%
	11	100%	99.7%	98.2%	93.3%	82.6%

2. TYPE OF PLANNED ANALYSIS

2.1. Interim Analyses

Prior to the final analysis, three interim analyses will be conducted.

The first two interim analyses will be conducted when approximately 45 enrolled participants of Cohorts 1-3 complete the 48-week treatment and last available participant of all enrolled participants (Cohorts 1-4) complete the 48-week treatment, respectively. If the pace of enrollment is robust, and the proportion of participants with HBsAg loss at Week 48 is expected to occur less or equal to two months between the two interim analyses, the two analyses may be collapsed into one analysis. Endpoints with measurement time after the 48-week treatment except for safety relevant data will not be analyzed in the first two interim analyses.

The sponsor had decided to stop participant enrollment in December 2023, with a total of 55 participants enrolled in Cohorts 1-3. Consequently, the study team had agreed that the first interim analysis conducted when 55 participants completed the 48-week treatment. During this interim analysis, only descriptive statistics had been performed, and no per-protocol (PP) analysis was conducted.

The second interim analysis will still be at the timepoint when the last participant of Cohorts 1-4 completes the week 48 treatment.

The third interim analysis was originally planned to be conducted when last available participant of Cohorts 1-3 completes follow-up 24 weeks post end of treatment. The sponsor decided to conduct the third interim analysis when last available participant of Cohorts 1-4 completes follow-up 24 weeks post end of treatment (see Section 10.3).

Sponsor further decided analysis based on per-protocol population will not be conducted at second interim analysis. Starting from second interim analysis in addition to descriptive statistics all available statistical analysis will be performed. The second interim analysis will include all efficacy endpoints except for PK and ADA of BRII-835 throughout Week 48, and the endpoints with measurement time after the 48-week treatment except for safety relevant data will not be analyzed in the second interim analyses. The third interim analysis will also include the efficacy endpoints of the corresponding time points except for PK and ADA.

2.1.1. Dose Escalation Analysis

Not Applicable.

2.2. Final Analysis

The final analysis will be performed when last available participant of Cohorts 1-4 completes the last scheduled visit according to the SoA.

3. GENERAL CONSIDERATIONS FOR DATA ANALYSES

All data collected on the electronic case report form (eCRF) will be presented in the data listings and will be listed by subject identification number (in ascending order), sex, age at screening, screening body weight and visit, where applicable. All summaries will be presented by Cohort.

When reporting descriptive statistics, the following rules will be applied, except for PK concentration data:

- Continuous variables: Descriptive statistics will include the number of non-missing values (n), arithmetic mean, standard deviation (SD), median, first quartile (Q1), third quartile (Q3), minimum and maximum values.

  The minimum and maximum values will be displayed to the same decimal precision as the source data; the Q1, Q3, arithmetic mean and median values will be displayed to one more decimal than the source data; SD value will be displayed to two more decimal than the source data for the specific variable.

  The appropriate precision for derived variables will be determined based on the precision of the data on which the derivations are based, and statistics will be presented in accordance with the abovementioned rules.

  n will be an integer. If no subjects have data at a given time point, then only n=0 will be presented.

  All data will be presented to a maximum of 2 decimal places.

- Categorical variables: Descriptive statistics will include counts and percentages per category. The denominator in all percentage calculations will be the number of subjects in the relevant analysis set with non-missing data, unless specifically stated otherwise. Percentages will be displayed to one decimal place.

  Percentage change from baseline values will be calculated and displayed to 1 decimal place in the listings. In the summaries, the mean and median percentage change from baseline values will be presented to 2 decimal places, the minimum and maximum values will be presented to 1 decimal place and the SD value will be presented to 3 decimal places.

  For categories where all subjects fulfill certain criteria, the percentage value will be displayed as 100. For categories where zero subjects fulfill certain criteria, there will be no percentage displayed. All other percentage displays will use 1 decimal place. Data listings will contain all reported and derived data.

Unless otherwise stated, the following methods will be applied:

- Repeat/unscheduled assessments: Values collected at unscheduled study visits/time points will be allocated to scheduled visit/time points according to the algorithms of analysis visit window together with values collected at scheduled visits/time points and be presented in summary tables. All collected data will be included in the data listings.

- Assessment windows: All assessments will be included in the data listings and no visit windows will be applied to exclude assessments that were performed outside of the protocol specified procedure windows.
- Result display convention: Results will be center aligned in all summary tables and listings. Subject identifiers, visits, and parameter labels may be left-aligned if required.
- Date and time display conventions: The following display conventions will be applied in all outputs where dates and/or times are displayed:
    - Date only: YYYY-MM-DD
    - Date and time: YYYY-MM-DD HH:MM

If only partial information is available, unknown components of the date or time will be presented as ‘NK’ (not known), i.e., ‘2016-NK-NK’. Time will be reported in military time.

3.1. Key Definitions

The following definitions will be used:

- Baseline: The baseline value is defined as the last available, valid (quantifiable continuous or categorical value), and non-missing observation for each subject prior to first study drug administration unless otherwise specified. Repeat and unscheduled assessments will be included in the derivation of the baseline values
- Change from Baseline: The change from baseline value is defined as the difference between the result collected/derived at a post-baseline visit/time point and the baseline value.

  The change from baseline value at each post-baseline visit/time point will be calculated for all continuous parameters using the following formula:

  Change from Baseline Value = Result at Visit/Time Point – Baseline Value

  The change from baseline value will only be calculated if the specific post-baseline visit/time point result and the baseline value for the parameter are both available; otherwise, will be treated as missing.
- Study day: The study day of an event is defined as the relative day of the event starting with the date of the first study drug administration (reference date) as Day 1 (there will be no Day 0).

  The study day of events occurring before the first study drug administration will be calculated as:

  Study Day = (Date of Event - Date of First Study Drug Administration)

  For events occurring on or after Day 1, study day will be calculated as:

  Study Day = (Date of Event - Date of First Study Drug Administration) + 1

Study days will only be calculated for events with complete dates and will be undefined for events that are 'Ongoing' at the end of the study.

- Relative days compared to an alternative reference point will be calculated similarly, but the alternative starting reference start date will be used instead of the date of the first study drug dosing.
- HBsAg loss: HBsAg changing from positive at baseline to <LLOQ at any post-baseline visit.
- Anti-HBs seroconversion: Anti-HBs changing from negative (<10 IU/L) at baseline to positive (>=10 IU/L) at any post-baseline visit.
- HBsAg seroconversion: HBsAg loss plus anti-HBs positivity (>=10 IU/L) at any post-baseline visit
- Sustained HBsAg loss: quantitative HBsAg < LLOQ at all visits (scheduled and unscheduled) for ≥ 24 weeks (≥ 161 days, i.e. 168 days (24 weeks) minus 7 days (Visit window)). Sustained days = (last date of HBsAg <LLOQ - first date of HBsAg <LLOQ) +1, during which the values at all visits < LLOQ.
- Anti-HBe seroconversion: Anti-HBe changing from negative or missing at baseline to positive at any post-baseline visit.
- HBeAg loss (HBeAg+ subjects only): HBeAg changing from positive at baseline to negative at any post-baseline visit.
- HBeAg seroconversion (HBeAg+ subjects only): HBeAg loss plus anti-HBe positivity at any post-baseline visit.
- Functional cure: sustained undetectable HBsAg (HBsAg < LLOQ) and sustained suppression of HBV DNA < LLOQ ≥ 24 weeks including all available visits after discontinuation of all treatment, including NRTI. Sustained duration as (Last date of HBsAg and HBV DNA < LLOQ) – (First date of HBsAg and HBV DNA < LLOQ after discontinuation of all treatment) +1 ⩾ 161 days, during which the values of HBsAg and HBV DNA at all available visits < LLOQ. At least 24 weeks is defined as 161 days (23 weeks) due to visit window (±1 week).

The LLOQ for HBsAg is 0.05 IU/mL, and 10 IU/mL for HBV DNA.

3.2. Analysis Sets

Analysis sets define the subjects to be included in an analysis. Analysis sets and their definitions are provided in this section. The analysis set will be identified and included as a subtitle of each table, figure, and listing.

For each analysis set, the number and percentage of subjects eligible for inclusion will be provided.

A listing of exclusion from analysis sets will be provided by subject.

3.2.1. Screened Analysis Set

The Screened Analysis Set will consist of all subjects who sign the informed consent form. The listing of eligibility criteria will be based on this population.

3.2.2. Full Analysis Set (FAS)

All participants assigned to study intervention will be included in Full Analysis Set.

3.2.3. Safety Analysis Set (SAS)

All participants assigned to study intervention and who take at least 1 dose of study drug. Participants will be analyzed according to the study intervention they received. Participants will be analyzed by Cohort, subtotal for Cohort 2-4 and overall.

All safety analyses will be based on Safety Analysis Set.

3.2.4. Modified Intent-To-Treat Analysis Set (mITT)

All participants assigned to study intervention, who take at least 1 dose of study drug, with at least 1 post-baseline visit through Week 48. Participants will be analyzed according to the study intervention to which they were randomized for Cohorts 1- 3, and for Cohort 4 participants will be analyzed according to the study intervention specified in the protocol.

3.2.5. Modified Efficacy Analysis Set

Participants will be analyzed as well according to modified efficacy analysis set as supplementary analysis. The modified efficacy analysis set is defined as all participants who have baseline HBsAg>100 IU/mL and take at least 1 dose of study drug, with at least 1 post-baseline visit through Week 48.

3.2.6. Per Protocol Set (PP)

The Per Protocol Set will include the subset of mITT who fulfil all of the following:

- HBsAg ≥ 0.05 IU/mL at baseline
- Total number of dose compliance ≥ 80% and ≤ 120% for BRII-835 and PEG-IFNα
- Have HBsAg data in the analysis visit window (week 48/week 72) for the primary efficacy endpoints analysis
- Not identified with important (major) protocol deviation that significantly impacts the interpretation of the primary efficacy endpoints

A Per Protocol review meeting will be held to go through the important protocol deviations to identify the PP population prior to the database lock for the final analysis.

3.2.7. Pharmacokinetic Analysis Set (PAS)

All participants who received BRII-835, had at least 1 evaluable PK concentration, and had no protocol deviations thought to impact on the PK data analysis.

3.2.8. ADA Analysis Set

All participants who received BRII-835 and had at least 1 non-missing ADA result from the ADA assay after first dose of the study drug. Participants will be analyzed by cohort (cohort 2 and cohort 3) and overall.

3.2.9. PBMC Analysis Set

Participants who had evaluable data for immune profiling will be included in the PBMC Analysis Set. Here "evaluable" refers to whose whole blood is collected and qualified for immune cell and cytokine/chemokine profiling. The analysis for the PBMC data will be based on this set.

3.3. Subject Grouping

For analyses based on the FAS, mITT, SAS and PP Set, subjects will be grouped according to the Cohort.

3.4. Strata and Covariates

Up to 75 participants will be randomly assigned in 1:1:1 ratio among Cohorts 1-3 using an IRT system. Stratification will be based on the following variables:

Screening HBsAg level (>100 IU/mL and ≤500 IU/mL, >500 IU/mL and ≤1,500 IU/mL, >1,500 IU/mL and ≤3,000 IU/mL).

If there are discrepancies in stratification factor values between the IWRS and the clinical database, the values recorded in the clinical database will be used for analyses.

For Cohort 4 no randomization is performed.

3.5. Sensitivity and Subgroup Analysis

In addition, for Cohorts 1-3 combine some two adjacent screening stratification levels (i.e. randomization strata) to reduce HBsAg stratification factors from three levels to two levels (HBsAg >100 IU/mL and HBsAg ≤1,500 IU/mL versus HBsAg >1,500 IU/mL and HBsAg ≤3,000 IU/mL; HBsAg >100 IU/mL and HBsAg ≤500 IU/mL versus HBsAg >500 IU/mL and HBsAg ≤3,000 IU/mL) to perform the stratified Bayesian hierarchical analysis as sensitivity analyses if model is converged using the similar approach as the primary analysis.

Further stratify Cohort 4 into subgroup analysis listed below:

- Subgroup analysis will be conducted between responder and non-responder by Week 44 in study BRII-179-835-001. Week 44 responder is defined as peak anti-HBs titer ≥ 10 IU/L by Week 44 in BRII-179-835-001 study.

The descriptive statistics for antiviral activity and immunological markers will be presented. The unstratified Bayesian analysis will be performed by subgroup of Cohort 4, respectively with similar method taken as Cohort 4.

HBsAg change over time will be summarized by Cohort 1- 4 based on baseline HBsAg level categories (HBsAg >100 IU/mL and HBsAg ≤500 IU/mL, HBsAg >500 IU/mL and HBsAg ≤1500 IU/mL, HBsAg >1500 IU/mL and HBsAg ≤3000 IU/mL, HBsAg≤1500 IU/mL). The subgroup of HBsAg <=1000 IU/mL, that had conducted in the first interim analysis, will not be required, but may be conducted depending on the exploratory needs.

3.6. Multiple Comparisons

NA

3.7. Missing Data and Outliers

3.7.1. Missing Data

In general, missing data will not be imputed unless methods for handling missing data are specified. Exceptions are presented in this document.

For missing last dosing date of study drug, imputation rules are described in Section 4.1.1, the handling of missing or incomplete dates for AE onset is described in Section 7.1.5.2, and for prior HBV and concomitant medications in Section 7.4.

3.7.2. Outliers

Outliers will be identified during the data management and data analysis process, but no sensitivity analyses will be conducted. All data will be included in the data analysis.

3.8. Data Handling Conventions and Transformations

The following conventions will be used for the imputation of date of birth when it is partially missing or not collected:

- If only month and year of birth is collected, then "15" will be imputed as the day of birth
- If only year of birth is collected, then "01 July" will be imputed as the day and month of birth

- If year of birth is missing, then date of birth will not be imputed.

If age at Day 1 is not available for a subject, then age derived based on date of birth and the Day 1 visit date will be used instead. If an enrolled subject was not dosed with any study drug, the randomization date will be used instead of the Day 1 visit date. For screen failures, the date the first informed consent was signed will be used for the age derivation. Age required for longitudinal and temporal calculations and analyses (e.g age at date of AE) will be based on age derived from date of birth and the date of the measurement or event, unless otherwise specified.

Non-PK data that are continuous in nature but are less than the lower limit of quantitation (LOQ) or above the upper LOQ will be imputed as follows:

- A value that is 1 unit less than the LOQ will be used to calculate descriptive statistics if the datum is reported in the form of "< x" (where x is considered the LOQ). For example, if the values are reported as < 50 and < 5.0, values of 49 and 4.9, respectively, will be used to calculate summary statistics. An exception to this rule is any value reported as < 1 or < 0.1, etc. For values reported as < 1 or < 0.1, a value of 0.9 or 0.09, respectively, will be used to calculate summary statistics.

- A value that is 1 unit above the LOQ will be used to calculate descriptive statistics if the datum is reported in the form of "> x" (where x is considered the LOQ). Values with decimal points will follow the same logic as above.

- The LOQ will be used to calculate descriptive statistics if the datum is reported in the form of "≤ x" or "≥ x" (where x is considered the LOQ).

Plasma concentrations that are below the limit of quantitation (BLQ) will be presented as "BLQ" in the concentration data listing. BLQ concentrations will be treated as 0 at predose time points, and as missing at postdose time points for determination of summary and order statistics. Geometric mean and geometric CV% will not be calculated if the concentrations is BLQ or missing at predose.

The following conventions will be used for the presentation of summary and order statistics:

- If at least 1 subject has a concentration value of BLQ for the time point, the minimum value will be displayed as "BLQ."

- If more than 25% of the subjects have concentration values of BLQ for a given time point, the minimum value will be displayed as "BLQ."

- If more than 50% of the subjects have concentration values of BLQ for a given time point, the minimum and median values will be displayed as "BLQ."

- If more than 75% of the subjects have concentration values of BLQ for a given time point, the minimum and median values will be displayed as "BLQ."

- If all subjects have concentration values of BLQ for a given time point, all order statistics (minimum, median, and maximum) and summary statistics will be displayed as "BLQ."

3.9. Analysis Visit Windows

Subject visits might not occur on protocol-specified days. Therefore, for the purpose of analysis, observations will be assigned to analysis windows. The analysis visit windows for the parameters at different periods are listed in Table 3.1 – Table 3.6

- Prior to NrtI Discontinuation Monitoring Period

Table 3.1 Analysis Visit Windows for HBsAg Prior to NRTI Discontinuation Monitoring Period

Analysis Visit	Nominal Study Day	Lower Limit	Upper Limit
Baseline	1	None	1
Week 4	29	2	43
Week 8	57	44	71
Week 12	85	72	99
Week 16	113	100	127
Week 20	141	128	155
Week 24	169	156	183
Week 28	197	184	211
Week 32	225	212	239
Week 36	253	240	267
Week 40	281	268	295
Week 44	309	296	323
Week 48	337	324	365
Week 56	393	366	421

Week 64	449	422	477
Week 72	505	478	533

One of the primary endpoints is the proportion of participants with HBsAg loss at weeks post actual end of treatment, a participant discontinues study treatment before the planned EOT (Week 48) and does not withdraw from study participation, he/she will return to the investigational site every 8 weeks upon EOT for the specified assessments and the corresponding analysis windows for HBsAg is as follows

Table 3.2 Analysis Visit Windows of HBsAg at 24 weeks post end of study treatment for participants discontinue study treatment

Analysis Visit	Reference day	Lower Limit	Upper Limit
EoT	1	None	1
FU Week 8	57	2	85
FU Week 16	113	86	141
FU Week 24	169	142	197

Table 3.3 Analysis Visit Windows for Anti-HBs Prior to NRTI Discontinuation Monitoring Period

Analysis Visit	Nominal Study Day	Lower Limit	Upper Limit
Baseline	1	None	1
Week 16	113	2	141
Week 24	169	142	197
Week 32	225	198	253
Week 40	281	254	309
Week 48	337	310	365
Week 56	393	366	421

Week 64	449	422	477
Week 72	505	478	533

Table 3.4 Analysis Visit Windows for HBV DNA Prior to NRTI Discontinuation Monitoring Period

Analysis Visit	Nominal Study Day	Lower Limit	Upper Limit
Baseline	1	None	1
Week 12	85	2	127
Week 24	169	128	211
Week 36	253	212	295
Week 48	337	296	365
Week 56	393	366	449
Week 72	505	450	533

Table 3.5 Analysis Visit Windows for HBeAg , Anti-HBe Prior to NrtI Discontinuation Monitoring Period

Analysis Visit	Nominal Study Day	Lower Limit	Upper Limit
Baseline	1	None	1
Week 16	113	2	169
Week 32	225	170	281
Week 48	337	282	421
Week 72	505	422	533

Tabel 3.6 Analysis Visit Windows for Hematology, Chemistry and Coagulation Assessments Prior to NrtI Discontinuation Monitoring Period

Analysis Visit	Nominal Study Day	Lower Limit	Upper Limit
Baseline	1	None	1
Week 2	15	2	22
Week 4	29	23	43
Week 8	57	44	71
Week 12	85	72	99
Week 16	113	100	127
Week 20	141	128	155
Week 24	169	156	183
Week 28	197	184	211
Week 32	225	212	239
Week 36	253	240	267
Week 40	281	268	295
Week 44	309	296	323
Week 48	337	324	365
Week 56	393	366	421
Week 64	449	422	477
Week 72	505	478	533

- NRTI Discontinuation Monitoring Period

Table 3.7 Analysis Visit Windows for HBsAg, HBV DNA, HBeAg, Hematology, Chemistry and Coagulation during NRTI Discontinuation Monitoring Period

Analysis Visit	NDMP Day	Lower Limit	Upper Limit

NDMP Baseline	1	None	1
NDMP Week 4	29	2	43
NDMP Week 8	57	44	71
NDMP Week 12	85	72	106
NDMP Week 18	127	107	148
NDMP Week 24	169	149	190

*NDMP: NRTI Discontinuation Monitoring Period; NDMP baseline is not applicable for HBeAg according to SOA in the protocol

3.9.1. Selection of Data in the Event of Multiple Records in an Analysis Visit Window

Depending on the statistical analysis method, single values may be required for each analysis window. For example, change from baseline by visit usually requires a single value, whereas a time-to-event analysis would not require 1 value per analysis window.

If multiple valid, nonmissing measurements exist in an analysis window, records will be chosen based on the following rules if a single value is needed:

- For baseline, the last nonmissing value on or prior to the first dosing date of study drug will be selected, unless specified differently. If there are multiple records with the same time or no time recorded on the same day, the baseline value will be the average of the measurements for continuous data, or the measurement with the lowest severity (e.g., normal will be selected over abnormal for safety electrocardiogram findings) for categorical data, and the most conservative value will be selected for efficacy data (e.g., negative will be selected over positive for HBeAg and HBsAg, whereas positive will be selected over negative for Anti-HBe and Anti-HBs).
- For post-baseline values:
  - The most conservative value for safety lab parameters (e.g., abnormal will be selected over normal) within the analysis window will be selected
  - If there are more than 1 value within an analysis window, the value collected closet to the nominal day will be selected.
  - If there are 2 records that are equidistant from the nominal day, the later record will be selected.

- If there is more than 1 record on the selected day, the latest record will be selected; if these measurements were recorded at the same time or no time recorded, the average will be taken for continuous data.

4. SUBJECT DISPOSITION

4.1. Subject Enrollment and Disposition

A summary of subject enrollment will be provided by Cohort for each country/region, investigator and site name and presented in Full Analysis Set. The summary will present the number and percentage of subjects enrolled. For each column, the denominator for the percentage calculation will be the total number of subjects analyzed for that column.

A similar enrollment table will be provided by randomization stratum. The denominator for the percentage of subjects in the stratum will be the total number of enrolled subjects. If there are discrepancies in the value used for stratification assignment between the IWRS and the clinical database, the value collected in the clinical database will be used. A listing of subjects with discrepancies in the value used for stratification assignment between the IWRS and the clinical database at the time of data finalization will be provided.

The randomization schedule used for the study will be provided as an appendix in the CSR.

- A summary of subject disposition will be provided by Cohort. This summary will present the number of subjects screened, subjects who meet all eligibility criteria but not randomized, and the number of subjects in each of the categories listed below:
- All screened subject
- Screen Failure
  - Subjects not met eligibility criteria
  - Subjects met eligibility but not enrolled
- Subject enrolled
- All treated subject
- Completed study drug administration
- Did not complete study drug administration with reasons for premature discontinuation of study drug
- Completed study
- Did not complete the study with reasons for premature discontinuation of study

For the status of study drug and study completion and reasons for premature discontinuation, the number and percentage of subjects in each category will be provided. The denominator for the percentage calculation will be the total number of subjects in the Full Analysis Set corresponding to that column. In addition, a flowchart will be provided to depict the disposition.

The following by-subject listings will be provided by subject identification (ID) number (in ascending order), age, sex and screening body weight to support the above summary tables:

- Reasons for premature study drug or study discontinuation
- Reasons for screen failure (will be provided by screened subject ID number in ascending order)

4.1.1. Duration of Exposure to Study Drug

Duration of treatment is calculated as (Date of Last Dose - Date of First Dose + 1) for the completers and calculated as (Discontinued Date-Date of First Dose +1) for subjects with prematurely discontinuation, regardless of any temporary interruptions in study drug administration, and will be expressed in weeks using up to 1 decimal place (e.g., 4.5 weeks). If the last study drug dosing date is missing, the latest date among the clinical visit date, laboratory sample collection date, and vital signs assessment date that occurred during the dosing-treatment period will be used.

Duration of Treatment and Total number of injections for BRII-835 and PEG-IFNα will be summarized, respectively using descriptive statistics (n, mean, STD, Median, Q1, Q3, Min, Max) for Safety Analysis Set.

In addition, Total number of injections will be summarized by counts and percentages for Safety Analysis Set.

No formal statistical testing is planned.

A by-subject listing of study drug administration and drug accountability will be provided separately by subject ID number, sex, age, race, body weight at Screening, and visit in chronological order.

4.1.2. Adherence to Study Drug

The level of prescribed adherence to the study drug regimen will be determined by the total doses of study drug taken relative to the total doses of study drug specified by the protocol.

The level of prescribed adherence of BRII-835 and PEG-IFNα will be expressed as a percentage using the following formula:

$$\text{Prescribed Adherence (\%)} = \left(\frac{\text{Total Doses of Study Drug}}{\text{Total Doses of Study Drug Specified by Protocol}}\right) \text{x } 100$$

Descriptive statistics for the level of prescribed adherence with the number and percentage of subjects belonging to adherence categories (e.g., *{< 80%, ≥ 80 to ≤ 90%, > 90%}*) will be provided by treatment group for the Safety Analysis Set.

No formal statistical testing is planned.

Number of Dose administration will be summarized by Cohort, Cohort 2-4 and overall based on Safety Analysis Set.

4.2. Protocol Deviations

Protocol deviations occurring after subjects entered the study are documented during routine monitoring. The number and percentage of subjects with important protocol deviations, and the total number of important protocol deviations by deviation reason (e.g., nonadherence to study drug, violation of select inclusion/exclusion criteria) will be summarized by Cohort for the Full Analysis Set. In addition, a by-subject listing subjects with all participant level protocol deviation will be provided.

5. BASELINE CHARACTERISTICS

5.1. Demographics and Baseline Characteristics

Subject demographic variables (i.e., age, sex, race, and ethnicity) and baseline characteristics (body weight [in kg], height [in cm], body mass index [BMI; in kg/m^2]) will be summarized by Cohort, subtotal for Cohort 1-3 and overall using descriptive statistics for continuous variables and using number and percentage of subjects for categorical variables. The summary of demographic data will be provided for the Full Analysis Set.

A by-subject demographic listing, including the informed consent date, will be provided by subject ID number (in ascending order), sex, age, race, and body weight at Screening.

5.2. Other Baseline Characteristics

Other baseline characteristics include HBsAg, Log10 transformed HBsAg, Anti-HBs, HBeAg at Screening and HBV genotype, etc. These baseline characteristics will be summarized by Cohort, subtotal for Cohort 1-3 and overall using descriptive statistics for continuous variables and using number and percentage of subjects for categorical variables. The summary of these baseline characteristics will be provided for the Full Analysis Set. No formal statistical testing is planned.

A by-subject listing of other baseline characteristics will be provided by subject ID number (in ascending order), sex, age, race, and body weight at Screening.

5.3. Medical History

Medical history will be collected at screening.

Medical history will be summarized by Cohort, subtotal for Cohort 2-4 and overall by the number and percentage of subjects using the Safety Analysis Set. No formal statistical testing is planned.

Listing of general medical history data will be provided.

6. EFFICACY ANALYSES

Efficacy endpoints analyses will be based on mITT. PP will serve as supplementary analysis only for the primary efficacy endpoints.

6.1. Primary Efficacy Endpoint

6.1.1. Definition of the Primary Efficacy Endpoint:

The primary efficacy endpoints include:

1) Proportion of participants with HBV surface antigen (HBsAg) loss at end of treatment
2) Proportion of participants with HBsAg loss at 24 weeks post end of treatment

An estimation approach with no hypothesis testing is used to analyze the primary endpoints. A Bayesian hierarchy logistics regression will be used to calculate the point estimate and 95% credible interval of the primary endpoints for Cohort 1-3, for Cohort 4 a unstratified Bayesian estimation will be conducted.

6.1.2. Estimand

Estimand for the primary endpoints:

- ➢ Proportion of participants with HBV surface antigen (HBsAg) loss at end of treatment
- ➢ Proportion of participants with HBsAg loss at 24 weeks post end of treatment

  - Population: Participants with chronic HBV infection as defined by the inclusion and exclusion criteria to reflect the targeted participant population.
  - Variable:
    - HBsAg loss at end of treatment.
    - HBsAg loss at 24 weeks post end of treatment
  - Treatment: Cohort 1, 2, 3 and 4.
  - Intercurrent events: Use of rescue medication, discontinuation of/interruption of/non-adherence to study intervention(s).

    The use of rescue medication, interruption of, and non-adherence to study intervention(s), will be ignored (treatment policy).

The discontinuation of study intervention(s) will be handled using while-on-treatment strategy for variable 1 and will be ignored (treatment policy) for variable 2.

- Population-level summary: Percentage of participants who achieve HBsAg loss at end of treatment and 24 weeks post end of treatment, respectively.

The last available HBsAg will be used for the analysis of primary endpoint HBsAg loss at end of treatment, if no any HBsAg value collected, the missing value will be handled as 'Non-responder'.

6.1.3. Analysis methods for the primary endpoints

The primary endpoints are 1) Proportion of participants with HBsAg loss at end of treatment 2) Proportion of participants with HBsAg loss at 24 weeks post end of treatment.

A Bayesian hierarchical model (Yuen, 2022; Jones, 2011) as primary analysis will be used to estimate the posterior probability of response rate for each cohort of Cohorts 1-3 by incorporating the screening HBsAg level stratification factor (i.e. randomization strata). The point estimate of the primary outcome and 95% equal-tailed credible interval will be drawn from the model.

Model for each group:
Number of responders r_g ~ Binomial (n_g, p_g), g=1,2,3
$\theta_g = \text{logit}(p_g) = \log(p_g/(1-p_g)) = \gamma_0 + \gamma_1 I_{\{B+\}1} + \gamma_2 I_{\{B+\}2} + \psi_g$, g=1,2,3
where γ_0, γ_1, γ_2, ψ_g are all parameters, Thus,
$\theta_1 = \gamma_0 + \psi_1$
$\theta_2 = \gamma_0 + \gamma_1 + \psi_2$
$\theta_3 = \gamma_0 + \gamma_2 + \psi_3$
Priors:
$\gamma_k \sim \text{Normal}(0, 10^6)$, k=0,1,2
$\psi_g \sim \text{Normal}(0, \omega^2)$
$\omega \sim \text{Half-normal}(1)$
Where r_g indicates the number of responders among n_g participants, p_g as the response rate r_g/n_g, θ_g indicates the log odds of treatment response $\log(p_g/(1-p_g))$, index g=1,2,3 refer to the stratum numbers,
γ_k represent fixed effects of screening HBsAg stratification factor, and ψ_g denotes a random effect in stratum g.

Stratum	$I_{\{B+\}1}$	$I_{\{B+\}2}$
HBsAg>100 IU/mL and HBsAg≤500 IU/mL	0	0
HBsAg>500 IU/mL and HBsAg≤1,500 IU/mL	1	0
HBsAg>1,500 IU/mL and HBsAg≤3,000 IU/mL	0	1

$I_{\{B+\}1}$ and $I_{\{B+\}2}$ are the dummy variables of $I_{\{B+\}}$.

For each cohort of Cohorts 1-3, the posterior distribution of response rate $P(p_g|data)$, g=1,2,3 was derived for each stratum using the model specified above.
The posterior distribution of the group-level response rate will be derived using a mixture of the posterior distributions of response rate for each analysis stratum in that group. The weights are proportional to the sample size of each stratum.

Samples from the posterior distributions for all parameters will be obtained using a Markov Chain Monte Carlo (MCMC) sampling technique with 20,000 iterations following a burn-in of size 2000.

Diagnostic tests will be performed to assess convergence and autocorrelation. Alternative programming conventions (blocking, thinning, parameterization, number of chains, seeds, size of burn-in, initial values for each chain) may be explored if, using initial programming code, satisfactory mixing is not achieved within computer memory limitations.

For some models with low number of events (0–1 event), the stratified Bayesian hierarchical model does not converge. In such cases a reduced model that does not consider screening stratification will be fitted. The model estimates the point estimate and 95% highest posterior density credible interval of proportion of participants achieving the primary endpoint assuming the number of responders followed a binomial distribution:

Number of responders $r \sim \text{Binomial}(n, p)$

Where p is the response rate, with a non-informative (Jeffrey's) prior: $p \sim \text{Beta}(0.5, 0.5)$.

The posterior distribution for p is: $P(p|y, N) \sim \text{Beta}(y + 0.5, N - y + 0.5)$.

The same reduced model will be used to estimate the posterior distribution of Cohort 4 as no stratification factor is planned in this Cohort.

In addition, for Cohorts 1-3 combine some two adjacent screening stratification levels (i.e. randomization strata) to reduce HBsAg stratification factors from three levels to two levels (HBsAg >100 IU/mL and HBsAg ≤1,500 IU/mL versus HBsAg >1,500 IU/mL and HBsAg ≤3,000 IU/mL; H BsAg >100 IU/mL and HBsAg ≤500 IU/mL versus HBsAg >500 IU/mL and HBsAg ≤3,000 IU/mL) to perform the stratified Bayesian hierarchical analysis if model is converged using the similar approach as the primary analysis.

Samples from the posterior distribution of the response rates in each cohort of Cohorts 1-3 will be used to obtain posterior probabilities of interest (e.g., Probability [Difference of proportion of achieving primary endpoints between cohorts > threshold Data]); threshold such as 5%, 10%, 15%, 20% or any other percentiles of interest may be explored.

The primary efficacy analysis will be performed within mITT analysis set and a supplemental analysis based on PP analysis set with the similar analysis will be presented.

6.2. Secondary Efficacy Endpoints

6.2.1. Estimand for the secondary endpoints:

- Population: Participants with chronic HBV infection as defined by the inclusion and exclusion criteria to reflect the targeted participant population.
- Variable:

  Continuous variables:

  - Absolute serum HBsAg and change from baseline over time through 24 weeks post end of treatment
  - Nadir serum HBsAg and maximum reduction of serum HBsAg from baseline throughout 48-week treatment

  Categorical variables:

  - HBsAg loss at Week 48
  - Functional cure (defined as sustained undetectable HBsAg and sustained suppression of HBV DNA [<lower limit of quantification (LLOQ)] ≥ 24 weeks after discontinuation of all treatment, including NRTI)
  - HBsAg loss and HBV DNA < LLOQ at 24 weeks after discontinuation of all treatment, including NRTI
  - Participants meeting NRTI discontinuation criteria at 24 weeks post end of treatment
  - Participants with Hepatitis B e antigen (HBeAg) loss and/or HBeAg seroconversion (baseline HBeAg-positive participants) at any timepoint
  - Proportion of participants with HBsAg seroconversion at any timepoint

  Time to event variable:

  - Time to achieve first serum HBsAg loss through Week 72
- Treatment: Cohort 1, 2, 3 and 4.
- Intercurrent events: Data collected will be utilized irrespective of discontinuation of, interruption of, and non-adherence to study intervention(s) or initiating rescue medication (treatment policy). However, the use of rescue medication will be treated as “treatment failure” (composite policy) for HBV DNA suppression related variables.
- Population-level summary: For continuous variables, descriptive statistics such as n, mean, standard deviation, median, interquartile, and ranges will be applied. For categorical variables, percentage of each cohort will be presented. Hazards ratio between the cohort 1-3, and descriptive statistics for cohort 4 will be presented for time to event variables.

Missing value will be censored for time to event variable and will be handled as 'Non-responder' for categorical variables, for the analysis of the endpoint HBsAg loss at Week 48 if the HBsAg value at Week 48 analysis visit window is missing however the HBsAg are loss at both Week 44 and Week 52, the HBsAg value at Week 48 will be imputed as HBsAg loss.

6.2.2. Analysis Methods for Secondary Efficacy Endpoint

- Proportion of participants with HBsAg loss at Week 48
- Proportion of participants achieving functional cure (defined as sustained undetectable HBsAg and sustained suppression of HBV DNA <LLOQ ⩾24 weeks after discontinuation of all treatment, including NRTI) Proportion of participants achieving HBsAg loss and HBV DNA <LLOQ at 24 weeks after discontinuation of all treatment, including NRTI
- Proportion of participants meeting NRTI discontinuation criteria at 24 weeks post end of treatment

A Bayesian hierarchical model which is similar to primary analysis will be used to analyze the four endpoints above.

- Absolute serum HBsAg and change from baseline over time through 24 weeks post end of treatment

Numerical value of HBsAg and change from baseline will be summarized by cohort and by visit. The available data for each visit will be presented.

For Cohorts 1-3 change from baseline in HBsAg to all scheduled visits in log base 10 scale will be statistically analyzed using a Mixed Model for Repeated Measures model. The model will include log base 10 transformed baseline as a covariate, cohort, visit, cohort by visit as fixed effects, and within-participant errors will be modeled with an unstructured, heterogeneous autoregressive (1), or compound symmetry variance matrix in that order if a model does not converge. Estimates of least square mean (LSM) values and the LSM differences between each BRII-835 dose cohort and PEG-IFNα monotherapy cohort, and their respective 95% confidence interval and p-values will be calculated. The analyses will be done using mITT analysis set with hypothetical estimand. If the model is not converged only descriptive statistics will be presented.

In the first interim analysis, absolute serum HBsAg and change from baseline over time through Week 48 will be performed.

- Nadir serum HBsAg and maximum reduction of serum HBsAg from baseline

Nadir serum HBsAg and maximum reduction of serum HBsAg from baseline will be summarized by cohort based on mITT analysis set.

- Time to achieve first time of serum HBsAg loss through Week 72

Time to achieve first time of serum HBsAg loss (Days) defined as the duration from the first study drug administration to the time to achieve first time of serum HBsAg loss before or on Week 72.
Duration (Days)= event/censoring date – the first study drug administration date + 1.

Time to achieve first time of serum HBsAg loss will be analyzed by taking account of losses to follow-up using the stratified log-rank test with randomization strata as a stratification factor based on mITT analysis set, and tabular summaries of the Kaplan Meier curves providing the median and quartiles -will be provided for each cohort, and the KM curves will be presented graphically. In addition, for Cohorts 1-3 a Cox proportional hazard regression model where the estimate of the hazard ratio for treatment (pairwise comparison between three cohorts) will be performed with randomization strata as fixed effect, its 95% CI, and p value will be provided if the model is converged.

Cohort 4 will be further analyzed by subgroups specified in Section 3.5, Time to achieve first time of serum HBsAg loss will be analyzed by taking account of losses to follow-up using the log-rank test based on mITT analysis set, and tabular summaries of the Kaplan Meier curves providing the median, quartiles, and range will be provided for each subgroup, and the KM curves will be presented graphically. In addition, for cohort 4 a cox proportional hazard regression model where the estimate of the hazard ratio for subgroup (responder vs non-responder by Week 24 and Week 44 respectively) will be performed its 95% CI, and p value will be provided if the model is converged.

	Data of event/censoring	Censoring
Participants who lost to follow up or withdrew from the trial	Time from the first study drug administration to last HBsAg sample collection date	Y
Participants who didn't achieve the HBsAg loss	Time from the first study drug administration to last HBsAg sample collection date	Y
Participants who achieved the HBsAg loss	Time from the first study drug administration to date of achieving first time HBsAg loss	N

6.2.3. Exploratory Endpoints:

- Evaluation of potential biomarkers for host responses to treatment and/or infection including but not limited to innate and HBV-specific immunological responses
- Detection of other HBV viral markers including but not limited to serum HBcrAg and HBV RNA

- Proportion of virologic and clinical relapse after NRTI withdrawal. Virologic relapse is defined as HBV DNA > 2,000 IU/mL measured at 2 consecutive study visits; clinical relapse is defined as virologic relapse AND ALT > 2x ULN

No statistical analysis will be performed for the exploratory endpoints but summary of descriptive statistics will be conducted.

The proportion of subjects who have ever had Virologic relapse and Clinical relapse after NRTI withdrawal will be summarized by Cohort, subtotal for Cohort 1-3, overall and visit.

For participants who achieved HBsAg loss at end of treatment, HBsAb (anti-HBs) levels at end of treatment and 24 weeks post end of treatment will be summarized by Cohort separately.

7. SAFETY ANALYSES

7.1. Adverse Events and Deaths

7.1.1. Adverse Event Dictionary

Clinical and laboratory adverse events (AEs) will be coded using the current version of MedDRA. System organ class (SOC), high-level group term (HLGT), high-level term (HLT), preferred term (PT), and lower-level term (LLT) will be provided in the AE dataset.

7.1.2. Adverse Event Severity

Adverse events are graded by the investigator as Grade 1, 2, 3, 4, or 5 according to toxicity criteria specified in the protocol. The severity grade of events for which the investigator did not record severity will be categorized as “missing” for tabular summaries and data listings. The missing category will be listed last in summary presentation.

7.1.3. Relationship of Adverse Events to Study Drug

Related AEs are those for which the investigator selected “Related” on the AE CRF to the question of “Related to Study Treatment.” Relatedness will always default to the investigator’s choice, not that of the medical monitor. Events for which the investigator did not record relationship to study drug will be considered related to study drug for summary purposes. However, by-subject data listings will show the relationship as missing.

7.1.4. Serious Adverse Events

Serious adverse events (SAEs) will be identified and captured as SAEs if the AEs met the definitions of SAEs that were specified in the study protocol. SAEs captured and stored in the clinical database will be reconciled with the SAE database from the vender Drug Safety before data finalization.

7.1.5. Treatment-Emergent Adverse Events

7.1.5.1. Definition of Treatment-Emergent Adverse Events

Treatment-emergent adverse events (TEAEs) are defined as 1 or both of the following:

- Any AEs with an onset date on or after the study drug start date
- Any existing AEs worsen after treatment

7.1.5.2. Incomplete Dates

If the onset date of the AE is incomplete and the AE stop date is not prior to the first dosing date of study drug, then the month and year (or year alone if month is not recorded) of onset determine whether an AE is treatment emergent. The event is considered treatment emergent if the AE onset is the same as or after the month and year (or year) of the first dosing date of study drug.

An AE with completely missing onset and stop dates, or with the onset date missing and a stop date later than the first dosing date of study drug, will be considered to be treatment emergent.

In addition, an AE with the onset date missing and incomplete stop date with the same or later month and year (or year alone if month is not recorded) as the first dosing date of study drug will be considered treatment emergent.

7.1.6. Summaries of Adverse Events and Deaths

Treatment-emergent AEs will be summarized by Cohort, subtotal for Cohort 2-4 and overall based on Safety Analysis Set.

7.1.6.1. Summaries of AE Incidence by Severity

A brief, high-level summary of the number of percentage of subjects who experienced at least 1 TEAE in the categories described below will be provided by treatment. All deaths observed in the study will also be included in this summary.

The number and percentage of subjects who experienced at least 1 TEAE will be provided and summarized by SOC and PT by Cohort, subtotal for Cohort 2-4 and overall.

For the AE categories described below, summaries will be provided by SOC, PT and treatment:

- TEAEs by maximum severity
- TEAEs with Grade 3 or higher
- treatment-related TEAEs
- BRII-835 treatment-related AEs
- PEG-IFNα treatment-related AEs
- Both BRII-835 and PEG-IFNα treatment-related AEs
- treatment-related TEAEs of Grade 3 or higher
- BRII-835 treatment-related TEAEs of Grade 3 or higher
- BRII-835+PEG-IFNαtreatment-related TEAEs of Grade 3 or higher

- PEG-IFNαtreatment-related TEAEs of Grade 3 or higher
- treatment-related TEAEs by maximum severity
- TE SAEs
- treatment-related TESAEs
- BRII-835 treatment-related SAEs
- BRII-835+PEG-IFNα treatment-related SAEs
- Injection site reaction
- TEAEs leading to premature discontinuation of BRII-835
- TEAEs leading to premature discontinuation of BRII-835+PEG-IFNα
- TEAEs leading to premature discontinuation of PEG-IFNα
- TEAEs leading to premature discontinuation of study
- TE SAEs leading to death (i.e., outcome of death)

Multiple events will be counted only once per subject in each summary. Adverse events will be summarized and listed first in descending order of total frequency of SOC and then by PT in descending order of total frequency within each SOC. For summaries by severity, the most severe severity will be used for those AEs that occurred more than once in a given subject during the study.

In addition to the above summary tables, all TEAEs, TE SAEs, TE treatment-related AEs, and TE treatment-related SAEs will be summarized by PT only in descending order of total frequency.

In addition, data listings will be provided for the following:

- All AEs, indicating whether the event is treatment emergent
- All SAEs
- All Deaths
- All SAEs leading to death (i.e., outcome of death)
- All AEs leading to premature discontinuation of study drug

- All AEs leading to premature discontinuation of study

7.2. Laboratory Evaluations

Laboratory data collected during the study will be analyzed and summarized using both quantitative and qualitative methods. Summaries of laboratory data will be provided by Cohort, subtotal for Cohort 2-4 and overall based on Safety Analysis Set and will include data throughout the trial.

For subjects who have permanently discontinued study drug, or all available data at the time of the database snapshot for subjects who were ongoing at the time of an interim analysis. The analysis will be based on values in standard units. When values are below the LOQ, they will be listed as such, and the closest imputed value will be used for the purpose of calculating summary statistics. Hemolized test results will not be included in the analysis, but they will be listed in by-subject laboratory listings.

A by-subject listing for laboratory test results will be provided by subject ID number, age, sex, race, body weight at Screening and visit in chronological order for hematology, serum chemistry, and urinalysis separately. Values falling outside of the relevant reference range and/or having a severity of Grade 1 or higher based on NCI-CTCAE Version 5.0 grading scale will be flagged in the data listings, as appropriate.

No formal statistical testing is planned.

7.2.1. Summaries of Numeric Laboratory Results

Descriptive statistics will be provided by treatment for each laboratory test specified in the study protocol as follows:

- Baseline values
- Values at each post-baseline visit
- Change from baseline at each post-baseline visit

A baseline laboratory value will be defined as the last measurement obtained on or prior to the date/time of first dose of study drug. Change from baseline to a post-baseline visit will be defined as the visit value minus the baseline value.

7.2.2. Graded Laboratory Values

The NCI-CTCAE Version 5.0 will be used to assign toxicity grades (1 to 5) to laboratory results for analysis. For laboratory tests with criteria for both increased and decreased levels, analyses for each direction (i.e., increased, decreased) will be presented separately.

7.2.2.1. Treatment-Emergent Laboratory Abnormalities

Treatment-emergent laboratory abnormalities are defined as values that increase at least 1 toxicity grade from baseline at any post-baseline visit. If the relevant baseline laboratory value is missing, any abnormality of at least Grade 1 observed within the time frame specified above will be considered treatment-emergent laboratory abnormalities.

7.2.2.2. Summaries of Laboratory Abnormalities

Laboratory data that are categorical will be summarized using the number and percentage of subjects in the study with the given response at baseline and each scheduled post-baseline visit.

The following summaries (number and percentage of subjects) for treatment-emergent laboratory abnormalities will be provided by lab test and treatment; subjects will be categorized according to the most severe post-baseline abnormality grade for a given lab test:

- Graded laboratory abnormalities

For all summaries of laboratory abnormalities, the denominator is the number of subjects with non-missing post-baseline values.

7.2.3. Liver-related Laboratory Evaluations

Liver-related abnormalities after initial study drug dosing will be examined and summarized using the number and percentage of subjects who were reported to have the following laboratory test values for post-baseline measurements:

- Aspartate aminotransferase (AST): (a) > 3 times of the upper limit of reference range (ULN); (b) > 5 x ULN; (c) > 10 x ULN; (d) > 20 x ULN
- Alanine aminotransferase (ALT): (a) > 3 x ULN; (b) > 5 x ULN; (c) > 10 x ULN; (d) > 20 x ULN
- AST or ALT: (a) > 3 x ULN; (b) > 5 x ULN; (c) > 10 x ULN; (d) > 20 x ULN
- Total bilirubin: > 2 x ULN
- Alkaline phosphatase (ALP) > 1.5 x ULN
- AST or ALT > 3 x ULN and total bilirubin: (a) > 1.5 x ULN; (b) > 2 x ULN

The summary will include data from all post-baseline visits. For individual laboratory tests, subjects will be counted once based on the most severe post-baseline values. For both the composite endpoint of AST or ALT and total bilirubin, subjects will be counted once when the criteria are met at the same post-baseline visit date. The denominator is the number of subjects in the Safety Analysis Set who have non-missing post-baseline values of all relevant tests at the

same post-baseline visit date. A listing of subjects who met at least 1 of the above criteria will be provided.

7.3. Vital Signs

Descriptive statistics will be provided by Cohort for vital signs as follows:

- Baseline value
- Values at each post-baseline visit
- Change from baseline at each post-baseline visit

A baseline value will be defined as the last available value collected on or prior to the date/time of first dose of study drug. Change from baseline to a post-baseline visit will be defined as the post-baseline value minus the baseline value. Vital signs measured at unscheduled visits will be included for the baseline value selection.

No formal statistical testing is planned.

A by-subject listing of vital signs will be provided by subject ID number, sex, age, race, screening body weight and visit in chronological order.

7.4. Prior and Concomitant Medications

Medications collected at screening and during the study will be coded using the current version of the World Health Organization (WHO) Drug dictionary.

7.4.1. Prior Medications

Prior HBV medications are defined as any medications taken before a subject took the first study drug.

Prior medications will be summarized by Anatomical Therapeutic Chemical (ATC) drug class Level 3 and preferred name using the number and percentage of subjects for each treatment and overall. A subject reporting the same medication more than once will be counted only once within each ATC drug class when calculating the number and percentage of subjects who received that medication. Medications may appear under multiple ATC drug classes. The summary will be ordered alphabetically by ATC medical class and then by preferred term in order of descending overall frequency within each ATC medical class. For drugs with the same frequency, sorting will be done alphabetically.

For the purposes of analysis, any medication with a start date prior to the first dosing date of study drug will be included in the prior medication summary regardless of when the stop date is. If a partial start date is entered the medication will be considered prior unless the month and year (if day is missing) or year (if day and month are missing) of the start date are after the first dosing

date. Medications with a completely missing start date will be included in the prior medication summary, unless otherwise specified.

Summaries will be based on the Safety Analysis Set. No formal statistical testing is planned.

7.4.2. Concomitant Medications

Concomitant medications are defined as medications taken while a subject took study drug. Use of concomitant medications will be summarized by Anatomical Therapeutic Chemical (ATC) drug class Level 3 and preferred name using the number and percentage of subjects for each treatment. A subject reporting the same medication more than once within each ATC drug class will be counted only once when calculating the number and percentage of subjects who received that medication. Medications may appear under multiple ATC drug classes. The summary will be ordered alphabetically by ATC medical class and then by preferred term in descending overall frequency within each ATC medical class. For drugs with the same frequency, sorting will be done alphabetically.

For the purposes of analysis, any medications with a start date prior to or on the first dosing date of study drug and continued to be taken after the first dosing date, or started after the first dosing date but prior to or on the last dosing date of study drug will be considered concomitant medications. Medications started and stopped on the same day as the first dosing date or the last dosing date of study drug will also be considered concomitant. Medications with a stop date prior to the date of first dosing date of study drug or a start date after the last dosing date of study drug will be excluded from the concomitant medication summary. If a partial stop date is entered, any medication with the month and year (if day is missing) or year (if day and month are missing) prior to the date of first study drug administration will be excluded from the concomitant medication summary. If a partial start date is entered, any medication with the month and year (if day is missing) or year (if day and month are missing) after the study drug stop date will be excluded from the concomitant medication summary. Medications with completely missing start and stop dates will be included in the concomitant medication summary, unless otherwise specified. Summaries will be based on the Safety Analysis Set. No formal statistical testing is planned.

All prior and concomitant medications (other than per-protocol study drugs) will be provided in a by-subject listing sorted by subject ID number, age, sex, race, screening body weight and administration date in chronological order.

7.5. Electrocardiogram Results

A by-subject listing for ECG assessment results will be provided by subject ID number, sex, age, race, screening body weight.

7.6. Other Safety Measures

No additional safety measures are specified in the protocol.

8. ADA

Descriptive statistics based on ADA Analysis Set will be performed for ADA.

9. PHARMACOKINETIC EVALUATION/ANALYSIS

Individual subject concentration data for BRII-835 and its metabolite A-312327 will be listed and summarized using descriptive statistics based on PK Analysis Set. Summary statistics (numbers of subjects, number of subjects with concentration BLQ, arithmetic mean, SD, coefficient of variation [CV], median, minimum, maximum, geometric mean and Geometric CV) will be presented by time point and Cohort. For summary statistics, BLQ values will be treated as zero at predose (including nominal timepoint of D1, D225, D337) and missing for postdose (including nominal timepoint of D1, D225, D337).

No PK parameters will be reported in this study since only limited (sparse) PK samples were collected. The concentration data from this study will be pooled with data from other studies for population PK analysis, which will be reported separately.

10. CHANGES FROM PROTOCOL-SPECIFIED ANALYSES

10.1. Changes From Protocol-Specified Efficacy Analyses

There are no deviations from the protocol-specified efficacy analyses. SAP combined the primary estimand 1 and estimand 2 of protocol into one primary estimand, intercurrent events are redefined for this combined primary estimand as in SAP section 6.1.2. For the secondary estimand(s), SAP emphasized that the use of rescue medication will be treated as "treatment failure" (composite policy) for HBV DNA suppression related variables.

10.2. Changes From Protocol-Specified Safety Analyses

There are no deviations from the protocol-specified safety analyses.

10.3. Changes From Protocol-Interim Analyses

The first interim analyses originally would be conducted when approximately 45 enrolled participants of Cohorts 1-3 complete the 48-week treatment in protocol, actually conducted when 55 participants of Cohorts 1-3 finished the 48-week treatment.

Considering that Cohort 4 may also have completed 24 weeks follow-up post EOT when the previous planned third interim analysis is being conducted, thereby, the revised third interim analysis will contain cohort 4 and be conducted when last available participant of Cohorts 1-4 completes follow-up 24 weeks post end of treatment.

10.4. Changes From Protocol-Sample Size

Up to 75 participants originally will be enrolled for Cohorts 1-3 and up to 45 participants enrolled for Cohort 4 in protocol. Actually, 55 participants enrolled in Cohorts 1-3 and 31 participants in Cohort 4. The second, subsequent interim analysis and final analysis will be conducted based on the above actually enrolled participants.

11. SOFTWARE

SAS® Software Version 9.4. SAS Institute Inc., Cary, NC, USA.

12. SAP REVISION

Revision Version/ Date (DD MMM YYYY)	Section	Summary of Revision	Reason for Revision
v2.0/20Feb2025	2.1 Interim Analyses	• Cohorts 4 will be added in the third interim analysis.<br>• Describes the interim analysis content with the corresponding timepoints.	Cohorts 4 follow-up data at 24 weeks post end of treatment will be available when the third interim analysis.
	3.5 Subgroup analysis	• Provided clear definition for Cohort 4 subgroup analysis (responder vs non-responder by anti-HBs response by Week 24 and Week 44 in BRII-179-835-001);<br>• HBsAg change over time will be summarized by cohort 1- 4 based on baseline HBsAg level categories, with a cutoff by HBsAg 1000 IU/mL excluded from the analysis.	Updated subgroup exploratory analysis based on medical considerations.
	6.1 Primary Efficacy Endpoint	• Bayesian hierarchy logistics regression will be used for cohort 1-3, For cohort 4 a unstratified Bayesian estimation will be conducted.	Bayesian hierarchy model is only applied to cohort 1-3 due to stratified randomization, and unstratified Bayesian model will be used for cohort 4.
	6.2 Secondary Efficacy Endpoints	• Time to achieve first time of serum HBsAg loss through Week 72: Cohort 4 analysis will be stratified by updated subgroup definition in section 3.5	Updated subgroup definition based on medical considerations.
	10. Changes From Protocol-Analyses	• Add the changes from the protocol-specified analyses.	Describe the key changes from the protocol-specified analyses.
v3.0/31Jul2025	2.1 Interim Analyses	• The third interim analysis will include the efficacy endpoints of the corresponding time points except for PK and ADA.	PK and ADA analysis will be performed at the final analysis.

	3.GENERAL CONSIDERATIONS FOR DATA ANALYSES	• All data will be presented to a maximum of 2 decimal places.	Two decimal places can meet the analysis requirements.
	3.1 Key Definitions	• Sustained HBsAg loss: quantitative HBsAg < LLOQ at all visits (scheduled and unscheduled) for ≥ 24 weeks (≥ 161 days, i.e. 168 days (24 weeks) minus 7 days (Visit window)).	Considering the deduction of a 7-day window period, the sustained duration of ≥24 weeks is defined as being ≥161 days (24 weeks -7 days).
	3.2 Analysis Sets	• Added modified efficacy analysis set for cohort 4	As supplementary analysis of cohort 4 to exclude patients having baseline HBsAg<=100 IU/mL
	3.5 Sensitivity and Subgroup Analysis	• Change this section from subgroup analysis to sensitivity and subgroup analysis<br>• Remove subgroup analysis by week 24 response in study BRII-179-835-001	The section also covered the sensitivity analysis.<br>Subgroup analysis by week 24 response in study BRII-179-835-001 is not needed based on medical judgement after IA 2 analysis.
	3.9. Analysis Visit Windows	• Add the title of table 3.2	Refer to this table for analysis visit windows of the participants of prematurely discontinue treatment.
	6. EFFICACY ANALYSES	• Per Protocol Set (PP) will serve as supplementary analysis only for the primary efficacy endpoints.<br>• Add HBsAb Summary analysis in exploratory analysis section.	Unless otherwise specified, PP analysis is not required for secondary efficacy endpoints.<br>Describe HBsAb level at the end of treatment and 24 weeks post end of treatment
	9. PHARMACOKINETIC EVALUATION/ANALYSIS	• For summary statistics, BLQ values will be treated as zero at predose (including nominal timepoint of D1, D225, D337) and missing for postdose (including nominal timepoint of D1, D225, D337).	Specify the data processing method of BLQ.

V4.0/28Jan2026	3.1. Key Definitions	• Clarify more details of definition of functional cure to facilitate further analysis.<br>• Remove definition of modified functional cure	The relevant analysis based on modified functional cure is not needed based on medical judgement
	3.2 Analysis Sets	• Clarify the definitions of ADA Analysis Set and PBMC Analysis Set	Wording changes to avoid confusion.
	6.2.2. Analysis Methods for Secondary Efficacy Endpoint	• Clarify the censor/event rules of time to event analysis	Wording changes to avoid confusion.
	6.2.3. Exploratory Endpoints	• Revise the description about anti-HBs summary analysis for the participants who achieved HBsAg loss at end of treatment<br>• Remove the description about HBV RNA and HBcrAg results	Wording changes to avoid confusion.<br><br>HBV RNA and HBcrAg are not conducted in the study.
